# *De novo* genome assembly and annotation of rice sheath rot fungus *Sarocladium oryzae* reveals genes involved in Helvolic acid and Cerulenin biosynthesis pathways

**DOI:** 10.1186/s12864-016-2599-0

**Published:** 2016-03-31

**Authors:** Shailaja Hittalmani, H. B. Mahesh, Channappa Mahadevaiah, Mothukapalli Krishnareddy Prasannakumar

**Affiliations:** Marker Assisted Selection Laboratory, Department of Genetics and Plant Breeding, University of Agricultural Sciences, Bengaluru, 560065 India; Department of Plant Pathology, University of Agricultural Sciences, Bengaluru, 560065 India

**Keywords:** Rice, Sheath rot, *Sarocladium*, Genome, Helvolic acid, Cerulenin

## Abstract

**Background:**

Sheath rot disease caused by *Sarocladium oryzae* is an emerging threat for rice cultivation at global level. However, limited information with respect to genomic resources and pathogenesis is a major setback to develop disease management strategies. Considering this fact, we sequenced the whole genome of highly virulent *Sarocladium oryzae* field isolate, Saro-13 with 82x sequence depth.

**Results:**

The genome size of *S. oryzae* was 32.78 Mb with contig N50 18.07 Kb and 10526 protein coding genes. The functional annotation of protein coding genes revealed that *S. oryzae* genome has evolved with many expanded gene families of major super family, proteinases, zinc finger proteins, sugar transporters, dehydrogenases/reductases, cytochrome P450, WD domain G-beta repeat and FAD-binding proteins. Gene orthology analysis showed that around 79.80 % of *S. oryzae* genes were orthologous to other Ascomycetes fungi. The polyketide synthase dehydratase, ATP-binding cassette (ABC) transporters, amine oxidases, and aldehyde dehydrogenase family proteins were duplicated in larger proportion specifying the adaptive gene duplications to varying environmental conditions. Thirty-nine secondary metabolite gene clusters encoded for polyketide synthases, nonribosomal peptide synthase, and terpene cyclases. Protein homology based analysis indicated that nine putative candidate genes were found to be involved in helvolic acid biosynthesis pathway. The genes were arranged in cluster and structural organization of gene cluster was similar to helvolic acid biosynthesis cluster in *Metarhizium anisophilae*. Around 9.37 % of *S. oryzae* genes were identified as pathogenicity genes, which are experimentally proven in other phytopathogenic fungi and enlisted in pathogen-host interaction database. In addition, we also report 13212 simple sequences repeats (SSRs) which can be deployed in pathogen identification and population dynamic studies in near future.

**Conclusions:**

Large set of pathogenicity determinants and putative genes involved in helvolic acid and cerulenin biosynthesis will have broader implications with respect to *Sarocladium* disease biology. This is the first genome sequencing report globally and the genomic resources developed from this study will have wider impact worldwide to understand Rice-*Sarocladium* interaction.

**Electronic supplementary material:**

The online version of this article (doi:10.1186/s12864-016-2599-0) contains supplementary material, which is available to authorized users.

## Background

*Sarocladium oryzae* [(Sawada) W. Gams & D. Hawksw] is an Ascomycetes fungus causing sheath rot disease in rice. It has recently emerged as a major threat for rice production in rice growing ecosystems in the world. In addition to rice, this fungus infects other important cereal food crops such as maize, sorghum, pearl millet, finger millet, and foxtail millet [1]. The commonly occurring weedy species in rice fields also acts as collateral hosts and source of natural inoculum in endemic areas [2].

*S. oryzae* produces white, sparsely branched and septate mycelium. Conidiophores are branched once or twice with 3–4 phialades in a whorl. The conidium is a aseptate, hyaline, cylindrical in shape and located on tip of phialades [3]. The conidium germinates and invades rice through the stomata and wounds caused by insects. Later mycelium grows intercellularly within vascular and mesophyll tissues [4]. The pathogen infects the uppermost leaf sheath enclosing young panicle and lesion length may range from 1 to 5 cm and lesion may enlarge to whole flag leaf sheath in severe cases. The necrotic lesions on flag leaf retards translocation of nutrients from foliage to panicle leading to complete suppression of panicle exertion. This results in production of partially filled chaffy grains, and yield loss ranging from of 3 to 85 % [5, 6]. Despite the considerable loss caused by this fungus, the life cycle and infection biology has been meagerly studied. Sheath rot symptom is also induced by application of Cerulenin which was demonstrated by developing Cerulenin negative mutants, which did not produce rot symptoms [7]. Also virulent strains of the fungi known to secrete proteinases at significantly higher levels compared to less virulent strains indicating the possible roles of fungal proteinases in plant pathogenicity.

The genomic resources for *S. oryzae* in public databases (NCBI) are limited to internal transcribed spacer (ITS) region sequences of ribosomal DNA and our previous QTL mapping study [8]. Due to lack of information on genes involved in pathogenicity/virulence, host–pathogen interactions and microsatellites markers, rice-*Sarocladium* pathosystem has not been studied well at global level. Considering these facts, we sequenced whole genome of highly virulent isolate of *S. oryzae* (Saro-13) from major rice growing region of South India. This is the first report of *de novo* genome assembly and annotation of *S. oryzae.* We carried out detailed analyses of gene families, secondary metabolite gene clusters, pathogenicity related genes, transposable repeat elements, phylogenetic relationship with other fungi and microsatellites. In addition, we analysed putative genes involved in helvolic acid and cerulenin biosynthesis pathways, which are very important in *Sarocladium* disease biology. The genomic resources generated from this study can be translated into designing better disease management strategies to mitigate sheath rot disease epidemics globally and widen the understanding of rice-*Sarocladium* pathosystem.

## Methods

### Isolation of fungus and confirmation

Diseased flag leaf sheath sampled over 25 locations in major rice growing regions of Karnataka state, India was used for isolation of fungus. Diseased sheath was surface sterilized using 0.05 % mercuric chloride solution followed by three times washing with sterile water. Sterilized diseased sheath pieces were incubated at room temperature for 4–5 days and germinating spores were transferred to potato dextrose agar (PDA) medium. Based on morphological features of conidiophores, phialades and conidiospores [3], the fungus was identified as *S. oryzae*. The virulence test of *S. oryzae* was carried out by standard mycelial inoculation [9] and detached tiller assay. The virulent field isolate Saro-13 isolated from Shrirangapatna (12.401035° N, 76.695754° E), Mandya District, a major rice growing region under cauvery command area was selected for whole genome sequencing. The fungus was characterized for internal transcriber region using ITS-4 and ITS-5 markers [10] to confirm the fungus as *S. oryzae*.

### DNA isolation, Illumina library preparation and sequencing

The virulent strain of *S. oryzae* (Saro-13) was grown on potato dextrose broth (PDB) medium for three days. The mycelium was grinded using liquid nitrogen and genomic DNA was isolated using nucleo-pore gDNA fungal and bacterial mini kit (Genaxy, Catalogue# NP-7006D). The DNA quality and quantity was assessed by Nanodrop and Qubit (Applied Biosystems), respectively. The genomic DNA was sheared to generate fragments of approximately 400–600 bp in Covaris microtube with the E220 system (Covaris, Inc., Woburn, MA, USA). The fragment size distribution was checked using Agilent Bioanalyzer (Agilent Technologies, Santa Clara, CA) with high sensitivity DNA Kit (Agilent Technologies). The fragmented DNA was cleaned up using HighPrep beads (MagBio Genomics Inc, Gaithersburg, Maryland). The Illumina paired-end library was prepared as per manufacturers instruction using NEXTflex DNA sequencing Kit (Catalogue # 5140–02, Bioo Scientific). The paired end library was sequenced using Illumina NextSeq 500 in Genotypic Technologies, Bengaluru and the length of read sequence was 151 nts from both the ends of the fragment.

#### Preprocessing of raw sequence reads

The low quality bases with Phred score less than Q30 (accuracy less than 99.99 % of the base called) and adapter sequence contamination in raw sequence reads of Illumina was discarded using FASTX-Toolkit (http://hannonlab.cshl.edu/fastx_toolkit/index.html).

### Genome assembly and functional annotation

*De novo* assembly of *S. oryzae* was performed using SPAdes assembler [11]. SPAdes assembler corrected sequencing errors in reads and performed scaffolding to output *de novo* assembled scaffolds. The assembled scaffolds were screened for sequences of mitochondrial genome contaminants. The gene prediction was performed using Augustus 3.0.3 (−−species = fusarium_graminearum --strand = both --genemodel = complete) [12, 13]. Functional annotation of genes was done by searching homology with Ascomycetes protein sequences of SwissProt (http://www.uniprot.org) using BLASTP with an e-value threshold of 1^e-10^. The annotation of protein domain structures was performed using InterProScan5 software [14]. The gene ontology (GO) terms were assigned by KAAS server [15].

### Analysis of orthologous gene families in Ascomycetes fungi

Gene families were inferred using orthoMCL (with default parameters like percentMatchCutoff = 50 and evalueExponentCutoff = −5) [16] by comparing proteins of *S. oryzae* with other Ascomycetes fungi like *Magnaporthe oryzae* (strain 70–15, http://www.broadinstitute.org/), *Fusarium graminearum (*strain PH-1, http://www.broadinstitute.org/), *Acremonium chrysogenum (*strain ATCC 11550, NCBI Accession number JPKY01000001)*,* and *Fusarium oxysporum (*strain 4287*,*http://www.broadinstitute.org). The groups having at least one copy from each genome were considered as core orthologous groups (COGs).

### Phylogenetic relationship

Based on orthoMCL clustering, 100 single copy ortholog gene groups from five fungal species were selected randomly and aligned separately using MUSCLE [17] version 3.8.31 with default parameters. Poorly aligned regions were removed by Gblocks [18] and all hundred Multiple Sequence Alignments (MSA) were concatenated. Then, 1000 bootstrap replicates were performed using SEQBOOT program in Phylip package version 3.696 [19]. The maximum-likelihood tree was constructed by PhyML [20] V3.1 (−−datatype aa --model WAG --bootstrap 1000) with 1000 bootstrap replicates to infer phylogenetic relationship of *S. oryzae* to other Ascomycetes fungi (*M. oryzae*, *F. graminearum*, *A. chrysogenum,* and *F. oxysporum).* The consensus tree was drawn using FigTree V1.4.2 (http://tree.bio.ed.ac.uk/software/figtree/). The phylogenetic analysis results are deposited in TreeBASE (http://purl.org/phylo/treebase/phylows/study/TB2:S19046) and Dryad Digital Repository (doi:10.5061/dryad.674p4).

#### Pathogenicity genes in *S*. *oryzae*

The fungal pathogenicity genes were retrieved from the Pathogen-Host Interaction (PHI) database [21] (http://www.phi-base.org) and BLASTP was performed against *S. oryzae* proteome. Protein alignments with more than 40 % identity and 70 % query coverage were considered as putative pathogenicity genes in *S. oryzae*.

#### Secretome analysis

Signal peptides and cleavage sites of *S. oryzae* proteins were predicted using SignalP version 4.1 (http://www.cbs.dtu.dk/services/SignalP/) and all proteins with signal peptides were analysed for presence of transmembrane (TM) domains using web servers like Phobius (http://phobius.sbc.su.se) and TMHMM version 2.0 (http://www.cbs.dtu.dk/services/TMHMM/). Subsequently, mitochondrial and chloroplast targeting signal containing *S. oryzae* proteins were removed based on prediction by TargetP 1.1 (http://www.cbs.dtu.dk/services/TargetP/). Finally, proteins containing a potential GPI (glycosyl phosphatidyl inositol)-anchor signal identified by PredGPI (http://gpcr.biocomp.unibo.it/predgpi/) web server were discarded.

#### Analysis of carbohydrate-active (CAZy) enzymes in *S. oryzae* proteome

The *S. oryzae* proteins from secretome analysis were subjected for CAZy annotation using CAT [22] and dbCAN [23] servers, which are based on the CAZy (Carbohydrate-Active Enzyme) database classification [24]. The results from both were combined when the e-value less than 10^−05^ and classified as per type of reaction catalyzed like Glycoside Hydrolases (GHs), Glycosyl Transferases (GTs), Polysaccharide Lyases (PLs), Carbohydrate Esterases (CEs), Carbohydrate-Binding Modules (CBMs), and Auxillary Activities (AAs) as described in CAZY database (http://www.cazy.org/Welcome-to-the-Carbohydrate-Active.html).

#### Secondary metabolite gene cluster analysis

The scaffold sequences of *S. oryzae* were analysed for secondary metabolites gene clusters using antiSMASH [25].

#### Cytochrome P450 family and transcription factors (TFs) analyses

The cytochrome P450 gene family classification in *S. oryzae* was done using Fungal Cytochrome P450 Database (FCPD; http://p450.riceblast.snu.ac.kr/). The proteins encoding for TFs were classified based on Fungal Transcription Factors Database (FTFD; http://ftfd.snu.ac.kr/).

#### Pathway analysis of helvolic acid and Cerulenin biosynthesis

We retrieved amino acid sequences of putative genes involved in helvolic acid biosynthesis from *Aspergillus* genome database (AspDB; http://www.aspgd.org) and protein homology search was carried out with *S. oryzae* genes. The genes with minimum 50 % identity and 70 % query coverage were considered as putative candidates in helvolic acid biosynthesis pathway. In addition, a homology search was also performed against NCBI non-redundant protein database to obtain homologous sequences in closely related fungal species. The protein domain based search was performed to identify putative genes involved in Cerulenin biosynthesis.

#### Prediction of repeats and simple sequence repeats (SSRs)

The *S. oryzae* scaffold sequences were subjected for *de novo* repeat prediction using RepeatMasker [26]. Reference based repeats analysis was done by comparing to reference repeat library database of RepBase (http://www.girinst.org/repbase/). The whole genome of *S. oryzae* was analyzed to determine the distribution and frequency of various types of SSRs using Microsatellite Identification tool (MISA) [27] (http://pgrc.ipk-gatersleben.de/misa/). The minimum length of SSR motif was set as 10 for mono, 6 for di, 5 for tri, tetra, penta and hexa motifs.

## Results and discussion

### Genome assembly and annotation

The *S. oryzae* isolate, Saro-13 was selected for whole genome sequencing based on virulence study, and was confirmed by mycelial morphology, colony characteristics and ITS sequencing. *S. oryzae* (Saro-13) produced sparsely branched mycelium with orange pigmentation on potato dextrose agar (PDA) medium. The conidium was single-celled, cylindrical and hyaline in structure (Additional file 1). The ribosomal DNA internal transcribed spacer (ITS) region of *S. oryzae* isolate Saro- 13 was sequenced using Sanger sequencing platform. Then, the ITS sequence was analysed by BLASTN to confirm the identity of Saro-13*.* The top 20 hits with e-value of 0 confirmed the identity of Saro-13 isolate as *S. oryzae* (Additional file 2).

Saro-13 was isolated from major rice growing region in Cauvery canal irrigated area of South Karnataka, India. Sequencing was carried out using Illumina NextSeq500. A total of 20,963,198 (paired reads, ~100x depth) raw reads were generated and the read length was 151 nts. Discarding low quality reads resulted 17,854,048 reads which corresponds to approximately 82x sequence depth of high quality data and these reads were assembled using SPAdes genome assembler [11]. The assembly process resulted 5,856 contigs with total consensus genome size of 32,778,109 bp. The maximum contig size was 89796 bp, and minimum contig size was 209 bp. The average size of the contig was 5597 bp and the N50 value of contigs was 18.07 Kb indicating good quality assembly for further downstream analysis (Table 1). The simple gene structures of most fungi facilitate accurate gene prediction. Moreover, majority of fungal species lack EST data to use them in gene prediction process. As a result, gene prediction in fungi heavily based on either *de novo* or comparative gene prediction models [28, 29]. The *ab initio* gene prediction using Augustus 3.0.3 revealed that *S. oryzae* genome harbors 10,526 protein coding genes. Out of which, 9658 were annotated with Uniprot fungal protein database and remaining 868 genes did not find significant annotation (Table 1). The average length of gene was 1689 bp with 294 genes were spaced at every one Mb of genome. This indicates that *S. oryzae* genome is gene dense like other fungi. An average distance between genes was 1.08 Kb with GC content of 45 % in the coding regions. There were 29,293 exons comprising of 15.97 Mb of total exon length. The average length of exon was 545.74 bp with 2.78 exons per gene. Overall, 18769 introns were present in 10526 genes with total length of 1.71 Mb and average length of introns was 93.85 bp (Table 2). The average exon and intron lengths and number of introns per gene in *S. oryzae* are in concordance with other sequenced Ascomycetes fungi like *Neurospora* and *Magnaporthe* [30–32]*.* The structural uniformity of genes among Ascomycetes fungi may provide a unique opportunity to study their evolution.

**Table 1:** Whole genome assembly features of *S. oryzae*

A. Assembly parameters	Value
Total genome size (bp)	32,778,109
Number of contigs	5856
Maximum contig length (bp)	89796
Minimum contig length (bp)	209
Average contig length (bp)	5597
Total number of non-ATGC characters	0
Depth of genome coverage (x)	82
N50 value (bp)	18070
GC (%)	53.49
B. Protein annotation	
Total number of proteins/genes predicted	10526
Total number of annotated proteins/genes	9658
Total number of un-annotated proteins/genes	868

**Table 2:** Details of coding genes, exon and intron in genome of *S. oryzae*

Gene details	Value
Number of coding genes	10526
Average gene length (bp)	1689.11
Gene density (number of genes per Mb)	294
Average distance between genes (Kb)	1.08
GC content (%) in coding region	56.61
Mean protein length (amino acids)	506.25
Exon details	
Number of exon	29293
Total exon length (Mb)	15.97
Average exon length (bp)	545.74
Number of exons per gene	2.78
Intron details	
Number of intron	18769
Total intron length (Mb)	1.76
Average intron length (bp)	93.85

### Protein family (pfam) domains and gene ontology (GO) annotation

An InterProScan pfam analysis identified 2,820 protein families containing 7718 proteins in *S. oryzae*. Large number of major facilitator superfamily (212 proteins), fungal specific transcription factor (175 proteins), protein kinase (137 proteins), fungal Zn(2)-Cys(6) binuclear cluster (122 proteins), sugar transporters (120 proteins), short chain dehydrogenase (97 proteins), cytochrome P450 (93 proteins), WD domain G-beta repeat (72 proteins), FAD binding (67 proteins), methyltransferase (57 proteins), and pyridine nucleotide-disulphide oxidoreductases (50 proteins) domain containing proteins were enriched in the *S. oryzae* genome. Majority of these gene families are known to be involved in host-pathogen interactions, indicating *S. oryzae* emerging as a very important plant pathogen to study arsenal of pathogenicity genes.

**Fig. 1:**
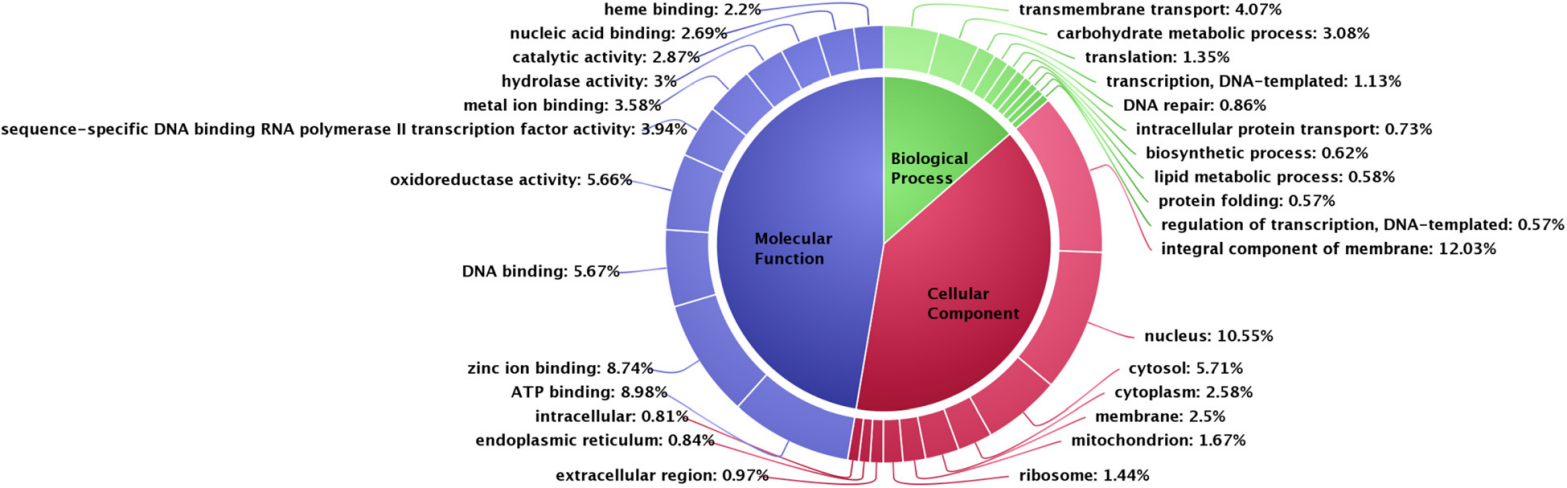
Gene ontology (GO) annotation of *S. oryzae* genes

The gene ontology (GO) annotation of 10,526 genes of *S. oryzae* revealed that 12.21 %, 39.10 % and 47.33 % of genes were annotated with biological, cellular and molecular functions, respectively (Fig. 1). Among biological processes, around 4.07 % of genes were involved in transmembrane transport, followed by carbohydrate metabolism (3.08 %), translation (1.35 %), transcription (1.13 %), DNA repair (0.86 %), intracellular protein transport (0.73 %), biosynthetic process (0.62 %), lipid metabolism (0.58 %) and protein folding (0.57 %). With respect to cellular process, 10.55 %, 5.71 %, and 2.58 % of genes were found to be associated with nucleus, cytosol, and cytoplasm, respectively. Similarly, in molecular function around 8.98 %, 8.74 %, 5.67 % and 5.66 % of genes were involved in ATP binding, zinc ion binding, DNA binding, and oxidoreductases activity.

### Orthology, multigene families and phylogenetic relationship of Ascomycetes fungi

**Fig. 2:**
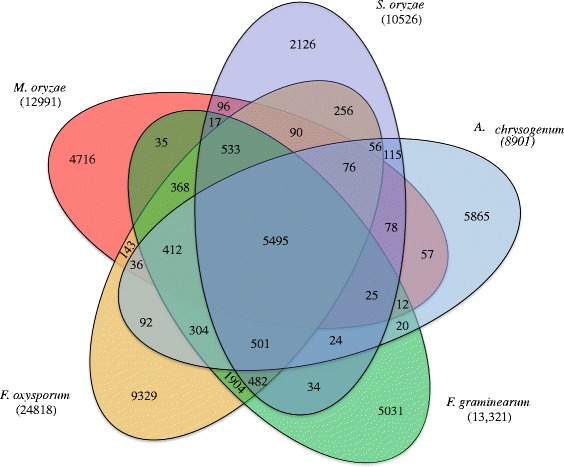
The Venn diagram illustrates shared and distinct orthologous gene families in Ascomycetes fungi. The proteomes of *S. oryzae*, *A. chrysogenum*, *F. graminearum*, *F. oxysporum*, and *M. oryzae* were clustered using orthoMCL. The number of genes in each species are shown in parenthesis

The orthologous genes are resultant of speciation process and clear delineation of orthologous relationship between species helps us to reconstruct evolution of species [33]. Moreover, orthology is the most accurate way to identify differences and similarities, transfer of functional gene information from model organisms to uncharacterized newly sequenced genomes [34]. To predict ortholog genes and gene family duplications among five sequenced *Ascomycetes* fungi (*S. oryzae*, *M. oryzae*, *A. chrysogenum*, *F. graminearum* and *F. oxysporum)*, we clustered their proteomes using orthoMCL tool. The clustering of proteomes resulted 13185 ortholog groups (Fig. 2), of which 5495 were core orthologous groups (COGs) among *Ascomycetes* fungi. Among COGs, 3246 were single copy ortholog genes indicating they are putative essential genes. There were 480 orthologous groups consisting of 1159 genes found to be duplicated (more than one copies of gene) in *S. oryzae* genome. The largest multigene family was encoding polyketide synthase dehydratase (PF14765), followed by ABC transporter (PF00005), ABC transporter transmembrane region (PF00664), Aldehyde dehydrogenase family (PF00171), Fibronectin type III-like domain (PF14310), PA14 domain (PF07691), Copper amine oxidase (PF02727), WSC domain (PF01822), OPT oligopeptide transporter protein (PF03169). The polyketide synthase dehydratase gene family is known to produce secondary metabolites and essential for fungal virulence [35, 36] to invade the host. The ABC transporters also play a vital role in pathogen virulence [37] by exporting noxious extracellular toxins and impose survivability to the fungus during adverse environmental conditions. The aldehyde dehydrogenase family of proteins are involved in production of indole-3 acetic acid (IAA) in fungi, which is very important during host-pathogen interaction [38].

**Fig. 3:**
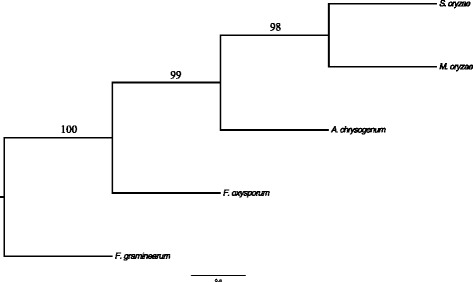
Maximum likelihood phylogenetic tree based on hundred single copy ortholog genes. Genetic relatedness of *S. oryzae* to other ascomycetes fungi based on single copy ortholog genes. Randomly 100 single copy ortholog genes were aligned and 1000 bootstrap maximum likelihood tree constructed based on WAG model of evolution

Phylogenetic relationship between *S. oryzae* with other Ascomycetes fungi was inferred based on protein similarity of hundred randomly choosen single copy ortholog genes from orthoMCL analysis. Based on WAG model [39] of protein evolution, *S. oryzae* was closely related to *M. oryzae* (causal organism of rice blast disease) followed by *A. chrysogenum, F. oxysporum* and *F. graminearum* (Fig. 3). The closer relatedness to *Magnaporthe* implies the shared gene arsenal required for adaptation to same host.

### Pathogenicity associated genes/factors in *S. oryzae*

To our knowledge, pathogenicity genes/factors are not determined so far in *S. oryzae* genome due to lack of genomic resources. The *S. oryzae* infect aerial parts of the rice plant, especially uppermost leaf sheath enclosing the young panicles. To identify putative genes involved in pathogenicity, we analysed *S. oryzae* proteomes for pathogen-host interaction (PHI) gene database, secretary proteins, carbohydrate-active enzymes (CAZymes), secondary metabolites, transporters, and transcription factors that are required to colonize in the host tissue.

### a. Putative Pathogen-Host Interaction (PHI) genes

The PHI database has collection of experimentally verified virulence associated genes from fungi, oomycetes and bacteria [21]. All 10,526 protein sequences of *S. oryzae* were aligned to PHI fungal genes using BLASTP (e-value 10^−10^). We identified 953 (9.06 % of total genes of *S. oryzae*) putative PHI genes in *S. oryzae* spanning across 59 different fungal species. Highest number of homologs was found in *Fusarium graminearum* (483 genes), followed by *Magnaporthe oryzae* (145 genes), *Aspergillus fumigatus* (66 genes), *Candida albicans* (36 genes), *Botrytis cinerea* (20 genes), *Cryptococcus neoformans* (18 genes), *Fusarium oxysporum* (18 genes), and other fungal species (167 genes) (Additional files 3 and 4). We assume that these genes are putative candidate pathogenicity determinants to induce pathogenicity in *S. oryzae* as their role in pathogenesis is already proven in their respective host species (cross-species pathogenicity) [40]. These preliminary results pave the way for future researchers to dissect pathogenicity genes in *S. oryzae.*

### b. Secretory proteins

The secretome analysis of *S. oryzae* proteome revealed 391 proteins harboring signal peptides (SPs) (Additional file 5). The aspartyl protease domain (Asp) containing secretory proteins were enriched in the *S. oryzae* genome and are mainly involved in proteolytic activity (hydrolysis of peptide bonds). Another class of secretory proteins like Tyrosinase is known to be involved in melanin production. Other important domain containing secretory proteins like hydrophobic surface binding protein A (HsbA), cupin, fungal hydrophobin, and lipase were enriched in the *S. oryzae* genome (Additional file 6).

### c. CAZymes

Carbohydrate-Active Enzymes (CAZymes) play a vital role in metabolism of structural components of cell wall and storage glucans in plant pathogens. CAZymes are responsible for breakdown of cell wall components of host to establish successful infection process. Pathogen uses the host as a nutrient source and deploys CAZymes to degrade plant storage compounds. Analysis of CAZymes revealed, 3201 proteins encoding CAZymes, which were distributed across 176 CAZyme protein families. Out of which, 295 proteins had signal peptides and remaining 2906 proteins lacked signal peptides (Additional file 7). Further classification of CAZymes based on their catalytic activity showed, highest number of proteins (1115) encoding for glycosyl transferases (GTs) followed by glycoside hydrolases (GHs), carbohydrate esterases (CEs), carbohydrate-binding modules (CBMs), auxillary activities (AAs), and polysaccharide lyases (PLs) (Table 3). The GTs had 52 gene families and they transfers sugar moieties from activated donor molecules to specific acceptor molecules by forming glycosidic bonds. The GHs are involved in hydrolysis of glycosidic bond between or within carbohydrate molecules, and 71 GHs families were identified in *S. oryzae* genome. Similarly, 27, 13, 10 and 3 gene families were identified in CBMs, CEs, AAs, and PLs, respectively.

**Table 3:** Overview of CAZyme and number of gene families in each CAZyme categories

Family	Number of CAZymes proteins			Number of families
	With signal peptide	Without signal peptide	Total	
GHs	110	932	1042	71
GTs	8	1107	1115	52
PLs	4	7	11	3
CEs	39	377	416	13
AAs	48	222	270	10
CBMs	86	261	347	27

*GHs* Glycoside Hydrolases, *GTs* Glycosyl Transferases, *PLs* Polysaccharide Lyases, Carbohydrate Esterases, *CBMs* Carbohydrate-Binding Modules, *AAs* Auxillary Activities

### d. Transcription factors (TFs)

Transcription factors (TFs) play a vital role in signal transduction pathways by acting as a linker between signal flow and target gene expression. Mining of specific repertoire of TFs in the genome gives us an overview about active pathways in the genome [41]. Around 351 (3.34 % of total genes) protein sequences encoded for 7 different classes of TFs in *S. oryzae* (Table 4)*.* Among seven classes of TFs, Zn2Cys6 (288 genes) were majorly distributed followed by C2H2 zinc finger (38 genes), bZIP (14 genes), heteromeric CCAAT type (7 genes), MADS-box (2 genes), Myb (1 gene), and Grainyhead/CP2 (1 gene). Similar level of distribution of Zn2Cys6 TFs in Ascomycota was reported by Todd and co workers [42]. These TFs have multifunction in fungi like controlling cellular process like fungal fitness, sugar and amino acid metabolism, gluconeogenesis and respiration, vitamin synthesis, chromatin remodeling, nitrogen utilization and response to drug and stress [43]. These TFs are also known to control calcineurin signaling pathway that is more important for fungal pathogenicity. It is reported that immune-suppressive drug cyclosporine inhibits calcineurin synthesis in other plant pathogens like *Magnaporthe oryzae* [44] and *Botrytis cinerea* [45] affected the formation of infection structure resulting in reduced pathogenicity. Another major class of TFs is C2H2 zinc finger, which are most common DNA-binding motifs, around 38 genes contained this motif in *S. oryzae*. The basic leucine zipper (bZIP) domain containing TFs is third largest family in the *S. oryzae* genome and they are known to regulate cellular growth and differentiation. There were 14 genes encoding for bZIP TFs in *S. oryzae*. Deletion mutants of this TFs showed defects in mycelial growth, development and reduced pathogenicity in *Magnaporthe* pathosystem [46]. The repertoire of TFs signifies that *S. oryzae* genome fosters diverse classes of TFs required for activation of most of the fungal pathogenicity genes.

**Table 4:** Distribution of transcription factors in *S. oryzae* genome

Type of TFs	Number of TFs
bZIP	14
C2H2 zinc finger	38
Grainyhead/CP2	1
Heteromeric CCAAT factors	7
MADS-box	2
Myb	1
Zn2Cys6	288

### e. Cytochrome P450 enzymes and membrane transporters

The cytochrome P450 enzymes in fungi carry out a wide range of bioconversions of complex polyaromatic hydrocarbons (PAHs) and steroid compounds mediated by monooxygenase enzymes [47]. There were 93 genes distributed across 82 various cyp gene families [48] in *S. oryzae* based on fungal cytochrome P450 database (FCPD) (Additional files 8 and 9). Genes encoding for plasma membrane transporters will help in assimilating the products degraded by CAZymes. The protein family classification of *S. oryzae* proteome revealed 212 genes encoding for major facilitator superfamily (MFS) and 120 genes encoding for sugar and other transporters. As compared to other gene families, MFS membrane transporters were high indicating their role in transporting small solutes in response to chemiosmotic ion gradients during pathogenesis.

### f. Pathway analysis of helvolic acid and cerulenin secondary metabolites production

**Fig. 4:**
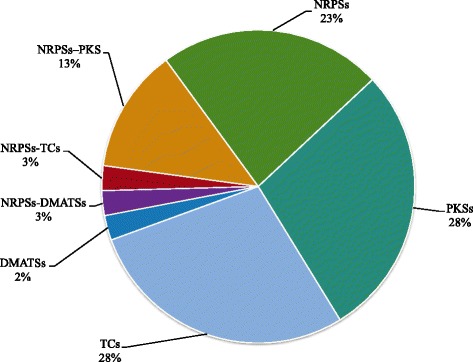
The secondary metabolites gene clusters in *S. oryzae*. NRPSs-nonribosomal peptides synthases, PKSs-polyketide synthases, TCs-terpene cyclases, and DMATSs-prenyltransferases

**Fig. 5:**
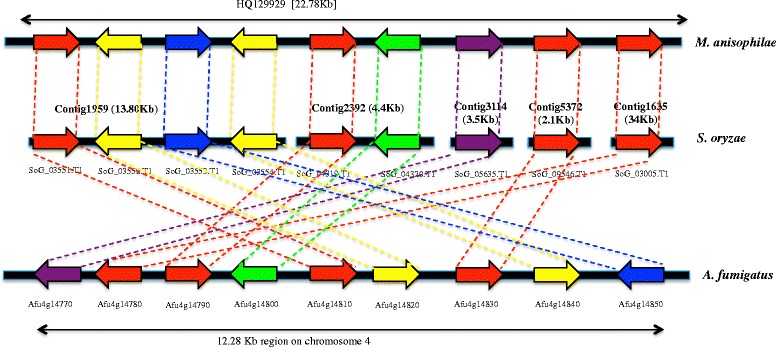
Organization of putative gene clusters involved in helvolic acid biosynthesis in *S. oryzae*. Genes are compared against *M. anisophilae* and *A. fumigatus*. *Red arrow*: cytochrome P450 genes. *Yellow arrow*: transferase family protein genes. *Blue arrow*: 3-ketosteroid-delta-1-dehydrogenase genes. *Green arrow*: SDR gene. *Purple arrow*: Squalene-hopene cyclase gene. Homologs are shown in *dotted lines*

Secondary metabolites (SMs) are small bioactive molecules and they are essential for fungal growth and development. At the same time SMs provide protection against various environmental stresses. The biosynthesis of SMs is catalyzed by either nonribosomal peptides synthases (NRPSs), polyketide synthases (PKSs), hybrid NRPS-PKS enzymes, prenyltransferases (DMATSs), and terpene cyclases (TCs). The catalytic activity of these enzymes results in production of SMs respectively like nonribosomal peptides, polyketides, NRPS-PKS hybrids, indole alkaloids, and terpenes [49]. Searching for SMs revealed that *S. oryzae* genome is enriched with PKSs, TCs followed by NRPSs, NRPSs-PKSs hybrid clusters (Fig. 4). Several studies have reported that *S. oryzae* produces helvolic acid and cerulenin SMs [50–53]. The biosynthetic pathways of these SMs were found to be different and concomitant production of these two metabolites might have synergistic effect to invade host by changing the cell permeability leading to leakage of electrolytes in the host tissue [52, 54–56]. So far, the studies on helvolic acid and cerulenin metabolites were restricted only to chromatographic assays and gene and protein level information of the pathways involved in their metabolism is unknown in *S. oryzae*. Based on our SM analysis, we hypothesize that PKSs, TCs and NRPSs could be the putative enzymes involved in the biosynthesis of these two metabolites in *S. oryzae*. We critically examined the proteome of *S. oryzae* to screen candidate genes involved in biosynthesis of these SMs. Helvolic acid is a steroidal antibiotic, known to be controlled by cluster of genes in *Aspergillus flavus* [57] and *Metarhizium anisophilae* [58]. Initial BLASTP searches of *S. oryzae* proteome against *A. flavus* protein sequences identified nine candidate genes in *S. oryzae*. The structural analysis showed these genes were single exonic genes arranged in clusters. Among these gene clusters, four (SoG_03551.T1, SoG_04319.T1, SoG_09546.T1, and SoG_03005.T1) cytochrome P450, two (SoG_03552.T1 and SoG_03554.T1) transferase family protein, one each of short chain dehydrogenase (SDR) (SoG_04320.T1), qualene-hopene-cyclase (SoG_05635.T1), and 3-ketosteroid-delta-1-dehydrogenase (SoG_03553.T1) genes (Fig. 5 and Additional file 10). The structural arrangement of gene clusters was more similar to *Metarhizium aninophilae* strain NwlB-02 (NCBI Locus ID: 129929) than *A. flavus*.

Another important SM produced by *S. oryzae* is Cerulenin and its biosynthesis is closely related to fattyacid synthesis [56]. The structure of Cerulenin is (2S), (3R) 2,3-epoxy-4-oxo-7, 10-do-decadienoyl amide concluded based on mass and NMR spectroscopic methods [59]. We looked at the enzymes involved in Dodecanoic acid pathway under fatty acid biosynthesis. There are six enzymes (FabD, FabB, FabF, FabG, FabA and FabZ) involved in biosynthesis of trans-dodeca-2-enoyl-[acp], an intermediary product of dodecanoic acid pathway (Additional file 11). The major protein domains of these enzymes are acyltransferase, oxidoreductase, and lyases. We identified putative candidate genes involved in Cerulenin biosynthesis based on protein domain annotation. There were 97 short chain dehydrogenase (SDR), 24 enoyl-(acyl carrier protein) reductases, 12 acyltransferase, seven beta-ketoacyl synthase, and 25 oxidoreductases genes found in *S. oryzae* genome. These genes are of future interest to understand its biosynthesis since Cerulenin is mainly used as antifungal antibiotic and anticancer agent that inhibits fatty acid and steroid biosynthesis [60, 61]. The knowledge of these pathway genes can be utilized for therapeutic and industrial uses, by exploring genetic engineering approaches to convert pathogenic strain to non-pathogenic strain for commercial purpose.

### Repetitive DNA content

Repetitive DNA is an integral part of fungal genomes. Repeat sequences play a vital role in generating genetic diversity, genome expansion and might also be detrimental to genome with respect to genome stability [29]. Repetitive DNA analysis was carried out on contig sequences of *S. oryzae.* The *de novo* and reference (by taking *Sarocladium* repeat library from repbase) based repeat analysis showed that 1.09 % of genome was repetitive. Interestingly, retro and DNA transposons were absent. However, repetitive DNA was limited to small RNA (0.03 %), simple repeats (0.70 %) and low complexity repeats (0.37 %). Then, we performed reference based repeat analysis by choosing repeat databases of closely related Ascomycetes fungi like *Aspergillus*, *Colletotrichum*, *Fusarium*, *Gibberella*, *Magnaporthe* and *Sarocladium zeae*. However, we did not observe significant increase in content of repeat elements. Many genome drafts of Ascomycetes have been assembled using short read technologies, and have reported repeat percentage in the range of 3-10 % [30, 32, 62, 63]. Thus, we believe that sequence read length (151 bp) might have not imposed a significant bias in repeat resolution. Low content of repeat elements is surprising since most of the pathogenic Ascomycetes fungi are known to harbor little higher percentage of repeat elements as compared to non-pathogenic counterparts. The low percentage of repeats can also be attributed to repeat induce point (RIP) mutations operating in the genome [64, 65]. Genome sequencing of other Ascomycetes fungi like *N. crassa*, *F. oxysporum*, *A. nidulans* and *A. fumigatus* showed lower repeat content coupled with RIP mechanism. Thus, these fungal species and *S. oryzae* which are closely related, might be sharing similar phenomenon like RIP in their genomes [65].

### Identification of microsatellites in *S. oryzae* genome

Microsatellites or SSRs markers are highly useful for molecular identification, genetic differentiation among individuals and populations in fungi. The genome-wide identification of SSRs in *S. oryzae* was performed in order to enrich genomic resources for population characterization. The scanning of 32.78 Mb *S. oryzae* genome revealed presence of 13,212 SSRs. Of which, 10650 were simple and remaining 2562 were complex types. The major proportion of simple SSRs was mononucleotide repeats occupying 50 % of total SSRs, followed by 22.06 % dinucleotides (2349), 18.49 % trinucleotides (1969), and 3.16 % tetranucleotides (337) repeats. The remaining SSRs were complex type, with 0.82 % of penta and 0.57 % of hexa nucleotides.

**Fig. 6:**
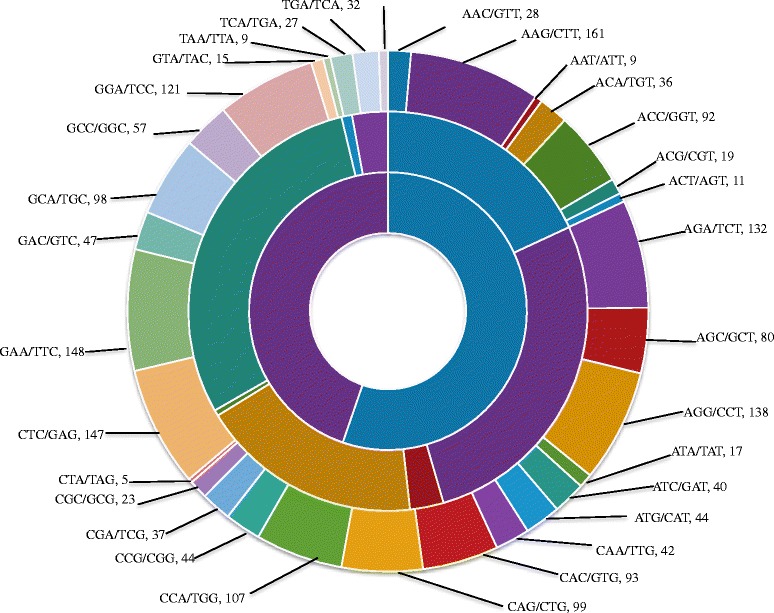
Distribution of mono, di and tri repeat motifs in *S. oryzae.* Inner, middle and outer circles represents mono, di, tri repeat types, respectively

Among mononucleotide repeats, 55.28 % were poly ‘A/T’ (3232 microsatellites), and 44.72 % were ‘G/C’ (2615 microsatellites) types (Fig. 6). Among 2349 dinucleotides microsatellites, ‘GA/TC’ type (29.54 %) of microsatellites were enriched in the genome followed by ‘AG/CT’ type (27.42 %), and ‘AC/GT’ type (18.09 %). The ‘CG/CG’ type dinucleotides microsatellites were present in lowest proportion (0.47 %). In case of trinucleotide microsatellites (969), around 8.18 %, 7.52 %, 7.47 % of SSRs were of ‘AAG/CTT’, ‘GAA/TTC’ and ‘CTG/GAG’ types, respectively. The ‘CTA/TAG’ type was lowest (0.25 %) in the genome of *S. oryzae*. The poor distribution (3.16 %) of tetranucleotides SSRs was observed in the *S. oryzae* genome. The maximum number of tetra nucleotides repeats was of ‘TTTC/GAAA’ type followed by ‘AAGA/TCTT’, ‘AAAG/CTTT’, and ‘TCCA/TGGA’. The overall analysis showed that the relative abundance of tetra, penta and hexa SSRs types were low as compared to mono, di and tri SSR types in *S. oryzae* genome. The similar observation was made in other Ascomycetes fungi like *A. nidulans*, *S. cerevisiae*, *F. graminearum*, *M. oryzae*, and *N. crassa* [66]. Hence, SSRs identified in this study will have immense importance in the immediate future to study population diversity, evolutionary pattern and understanding the virulence pattern of *S. oryzae* in the rice growing regions at global level.

## Conclusions

Rice sheath rot disease caused by *S. oryzae* is an emerging disease in rice growing regions. Lack of genomic resource for *S. oryzae* motivated us to takeup this sequencing effort and report the first ever genome draft of *S. oryzae*. The whole genome sequencing and *de novo* assembly revealed 32.78 Mb is the genome size of *S. oryzae.* This genome of this fungus codes for 10,526 proteins based on *ab initio* gene prediction algorithm. Furthermore, functional annotation of proteins showed that 73.23 % of total genes distributed across 2,820 protein families. The gene ontology annotation showed 12.21, 39.1 and 47.33 % of genes were involved in biological, cellular and molecular functions, respectively. Comparative orthology studies revealed 8,400 genes were orthologous to other Ascomycetes fungi and remaining (2126) genes were unique to *S. oryzae*. Multigene families such as polyketide synthase, ABC transporters and other pathogenicity related genes were distributed across 480 orthologous groups. The expansion of these gene families through natural selection denotes survival advantage of this pathogen for acclimatization to diverse environmental conditions. The overall analysis showed that *S. oryzae* has large sets of pathogenicity-related genes encoding secreted effectors, proteinases, secondary metabolism enzymes, transporters, carbohydrate-active enzymes, cytochrome P450 enzymes and transcription factors. This diversification and maintenance of more number of arsenal of diverse virulence factors may be required to colonize a wider range of host species by *S. oyzae*. More interestingly, helvolic acid biosynthesis pathway genes were found in a single cluster encoding for cytochrome P450 monooxygenase, transferase, short chain dehydrogenase (SDR), qualene-hopene-cyclase, and 3-ketosteroid-delta-1-dehydrogenase. Genome-wide identification of microsatellites revealed that around 43.71 % of SSRs were di, tri and tetra types, which could be explored in pathogen identification and population dynamic studies. Prior to elucidation of this draft genome sequence, very little was known about molecular mechanisms involved in pathogenicity and research in this area was limited to metabolite studies. Indeed, the availability of this genome in the public domain from our sequencing effort will now allow the researchers to carry out accelerated and rational experiments to dissect Rice-*Sarocladium* interaction that may help to articulate better disease control measures.

### Data availability

The genome assembly/contigs are deposited in NCBI/DDBJ/Genbank genome database under the accession number LOPT01000000. The raw sequence reads are deposited in NCBI SRA database under accession number SRX1639538.

## Additional files

Additional file 1: Morphology of Saro-13 isolate used for whole genome sequencing. (PDF 13621 kb) Additional file 2: ITS sequence alignment of Saro-13 isolate based on NCBI BLAST. (DOCX 1263 kb) Additional file 3: Pathogenicity gene orthologs in *Sarocladium oryzae* in comparison to other fungal species. (XLSX 34 kb) Additional file 4: Protein sequences of putative pathogenicity genes in *S. oryzae* genome. (DOCX 718 kb) Additional file 5: Secretary protein sequences of *S. oryzae*. (DOCX 266 kb) Additional file 6: Secretary proteins of *S. oryzae* and their pfam domains. (XLSX 48 kb) Additional file 7: Protein sequences of CAZYmes identified in *S. oryzae* genome. (DOCX 988 kb) Additional file 8: Cytochrome P450 gene families in *S. oryzae*. (XLSX 31 kb) Additional file 9: Protein sequences of Cytochrome P450 genes identified in *S. oryzae* genome. (DOCX 184 kb) Additional file 10: Protein sequences of putative gene clusters involved in Helvolic acid biosynthesis. (DOCX 134 kb) Additional file 11: Biosynthesis of Dodecanoic acid pathway. (PDF 47 kb)

## Abbreviations

AAs: Auxillary Activities
ABC: ATP-binding cassette
ATP: Adenosine Triphosphate
bp: basepair
CAZy: Carbohydrate-Active Enzyme
CBMs: Carbohydrate-Binding Modules
CEs: Carbohydrate Esterases
COGs: Core Orthologous Groups
GHs: Glycoside Hydrolases
GO: Gene Ontology
GPI: Glycosyl Phosphatidyl Inositol
GTs: Glycosyl Transferases
IAA: Indole-3 acetic acid
ITS: Internal Transcribed Spacer
Kb: Kilobase
Mb: Million bases
MFS: Major facilitator superfamily
MSA: Multiple Sequence Alignments
NMR: Nuclear Magnetic Resonance
NRPSs: nonribosomal peptides synthases
PDA: Potato Dextrose Agar
PDB: Potato Dextrose Broth
Pfam: Protein Family
PHI: Pathogen-Host Interaction
PKSs: polyketide synthases
PLs: Polysaccharide Lyases
QTL: Quantitative Trait Loci
SDR: Short chain dehydrogenase
SMs: Secondary metabolites
SPs: Signal Peptides
SSRs: Simple Sequence Repeats
TCs: Terpene Cyclases
TFs: Transcription Factors
TM: Transmembrane
