# Supplementary material for: De novo genome assembly and annotation of rice sheath rot fungus Sarocladium oryzae reveals genes involved in Helvolic acid and Cerulenin biosynthesis pathways
Source: BMC Genomics. 2016 Mar 31;17:271. doi: 10.1186/s12864-016-2599-0 (PMC4815069; doi:10.1186/s12864-016-2599-0)
Supplement: Additional file 1: — Morphology of Saro-13 isolate used for whole genome sequencing. (PDF 13621 kb) [file 12864_2016_2599_MOESM1_ESM.pdf]

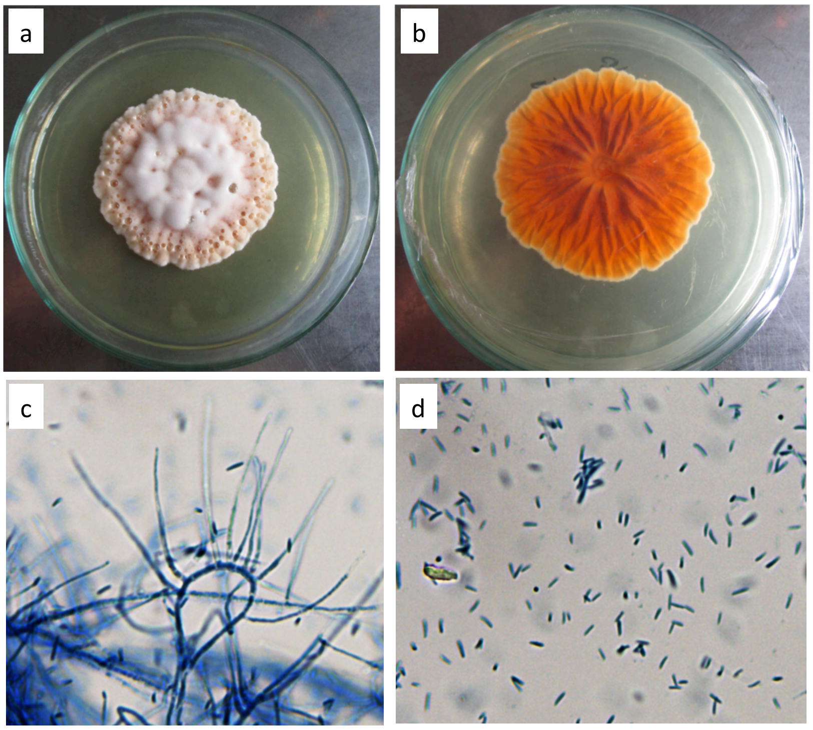

Additional file 1. Morphology of Saro-13 isolate used for whole genome sequencing. The upper (a), lower (b) sides of the colony morphology, branched mycelium (c) and conidia (d) are shown in figure.
