# Supplementary material for: De novo genome assembly and annotation of rice sheath rot fungus Sarocladium oryzae reveals genes involved in Helvolic acid and Cerulenin biosynthesis pathways
Source: BMC Genomics. 2016 Mar 31;17:271. doi: 10.1186/s12864-016-2599-0 (PMC4815069; doi:10.1186/s12864-016-2599-0)
Supplement: Additional file 2: — ITS sequence alignment of Saro-13 isolate based on NCBI BLAST. (DOCX 1263 kb) [file 12864_2016_2599_MOESM2_ESM.docx]

**Additional file 2:**

**ITS sequence alignment of Saro-13 isolate based on NCBI BLAST**


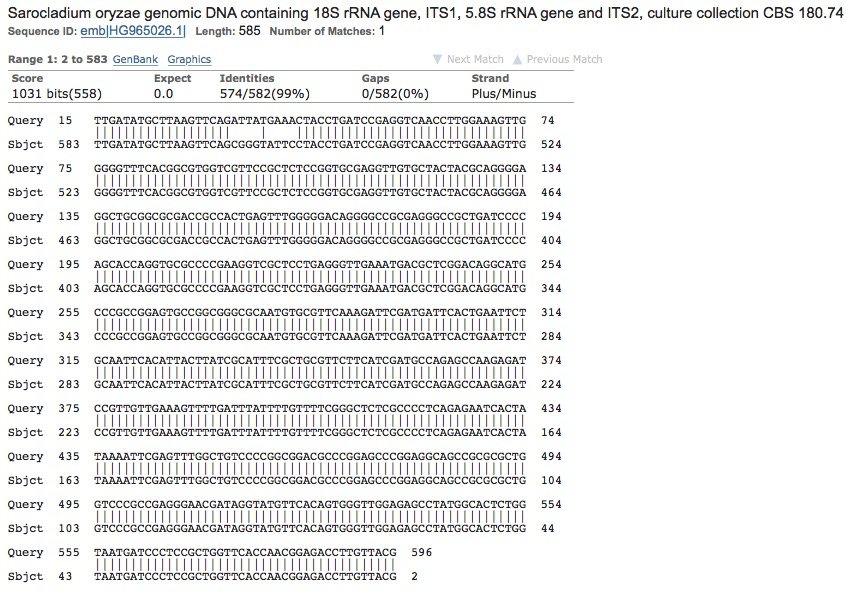


Maximum score = 1031

Total score = 1031

Query coverage = 95 %

E-value = 0.0

Identity = 99%

Homology to NCBI accession number = HG965026.1

**Phylogenetic tree of Saro-13**

**
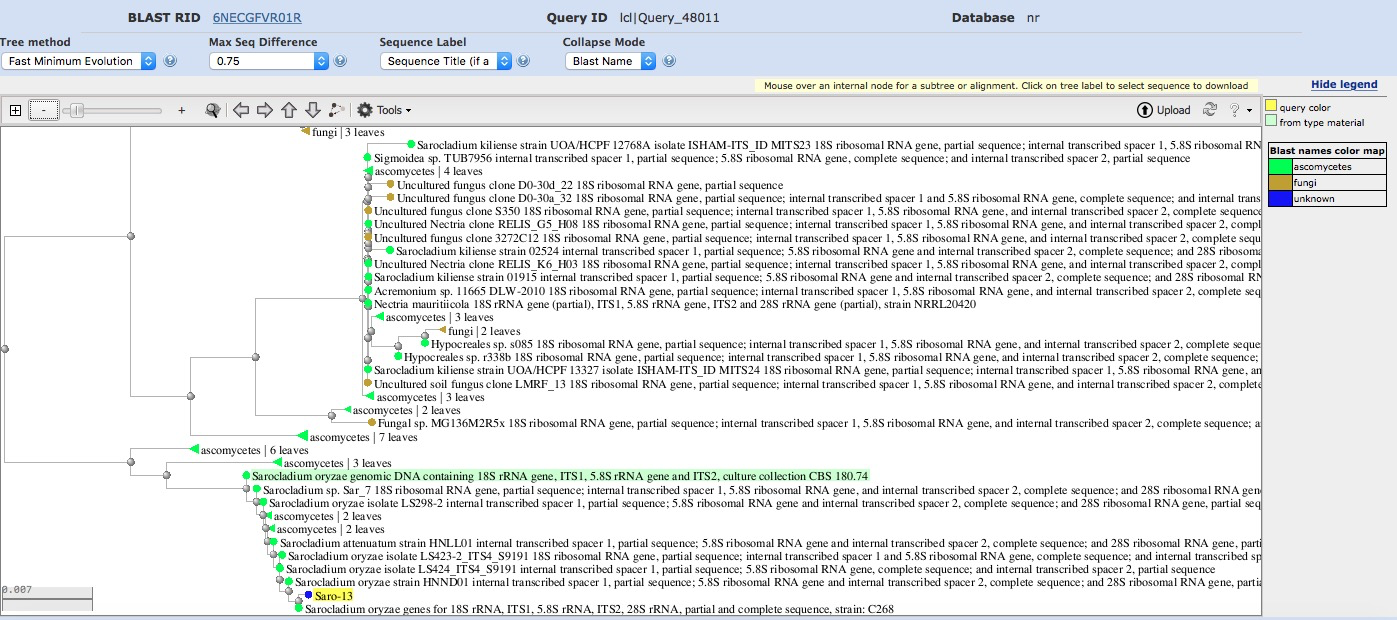
**
