# Supplementary material for: De novo genome assembly and annotation of rice sheath rot fungus Sarocladium oryzae reveals genes involved in Helvolic acid and Cerulenin biosynthesis pathways
Source: BMC Genomics. 2016 Mar 31;17:271. doi: 10.1186/s12864-016-2599-0 (PMC4815069; doi:10.1186/s12864-016-2599-0)
Supplement: Additional file 4: — Protein sequences of putative pathogenicity genes in S. oryzae genome. (DOCX 718 kb) [file 12864_2016_2599_MOESM4_ESM.docx]

**Additional file 4:**

Protein sequences of putative pathogenicity genes in *S. oryzae* genome

>SoG_00003.T1

MASSTRQFLREYKLVVVGGGGVGKSCLTIQLIQSHFVDEYDPTIEDSYRKQCVIDDEVAL

LDVLDTAGQEEYSAMREQYMRTGEGFLLVYSINSRQSFEEITTFQQQILRVKDKDYFPMV

VVGNKCDLENEREVTRQEGEALARSFGCKFIETSAKSRINVDKAFYDIVREIRRFNREMQ

GYSTGSGGGTGANGAPKPMDMDDGEKEAGCCSKCVVM

>SoG_00010.T1

MSPSRSLSRSSPEQWWNPYRRIAWSKRNQTWDAADLESNQQTSRGRDPKRSHTTYGNEST

REDVTLHDHGVLPPTGRPTDLSTADTMSAEPAEMNGGAGPELRQRKVDEKTNGSAAAPGD

NREDTSSKTSTPPRTGNGKRREKTEKQGLIRHVQPKTPFTVGNQIQRTILNSWINLLLIA

APVGIALNYVSSINRVVVFVVNFIAIIPLAAMLSFATEEIALRTGETLGGLLNATFGNAV

ELIVAIIALIDNKIRIVQTSLIGSILSNLLLVMGCCFFFGGLRREEQFFNETVAQTSASL

LALAVASVIVPTVYDSAIDTPTSDVAKLSRGTSVILLLVYAAYLLFQLKTHSAVFSEESQ

KVPAKPWRRNSLGPGAISSGLAMPGGLMGPAMPNQDEHERLSKLLVNPPGNGDDEDDEEE

PQLHFFVAIATLAISTAIIALCAEYMVDSIDAVTKQGGLSEEFVGLILLPIVGNAAEHAT

AVTVAIKDKMDLAIGVAVGSSMQVALFLIPLLVVIGWGMGKDEMNLSFDMFQIAVLFVAV

LLVNYLIGDGKSHWLEGFLLICLYSIIAVCAFSQVCKT

>SoG_00013.T1

MSAGRSIDAIAHDFTSGDLQAQVGSDADMMARRWARMVERGCCTCATYLPLIFVYSLTTW

GVWVLVEIGKYSTKSAWVGTTSSVVSVILFGLLNWSYTTAVFTNPGSTTNSDGYGLLPTQ

NAPKATSFTVKSNGEIRYCKKCQARKPDRAHHCSTCKRCVLKMDHHCPWLATCIGLRNQK

AFLLFLIYTTLFCLFGFAVSGSWMWAEVIDSPTVDSLLPVNFIMLTVISGIIGLVVGAFT

SWHIMLACKGQTTIECLEKTRYLSPLRKPFNQIHNPSNAMPQVAQQFVDFHANALPGITR

PEEGEERRTAELPRSYPPDGSRPVQMSYSERERDQTRRRYEEYLDEQDSEKLPNAFDLGW

KRNLTHLFGPSPVLWAFPVCNTTGDGWAWDPNPRWLETRERLRIERQQQRDREINAGWGL

GADSVPDFVPQRTEGSGRHYLNAPGEQYQPGRRTPSKADRVLGRDPNLYADSTQNLAMRR

LSPRGRTLEEELDDTDDEGAGMEDSQSAAEIGKTSQAAHAVSRHEAERRAMDVVTNGGGW

GRGASGMLRTNSGASSNGAKRTLSPAFQDDGVD

>SoG_00014.T1

MLIDTNQYPNATQTTSLPHSNMLQHSRSNQILNTFAARPSAPSSVSAISGSSVASSSRQG

SPIMTPELATLTNSSSIQASPEQLSAKDDDSCPPLFQLPESIVSRKTSSTSLGQSLKASS

YVASDTPPVPAAKGGIIRRLSSRASRTISSRRRQSSAAPASRDTSVGPCIFRRRSDSNTT

APPEFPSATTDSESDAEERDEIGSLRIFGLEEVSREPSTPQLNTAGAMAGPVIPFFVQQG

TRLRKVSKKSRSKRISLAYQLETHTLVWDKSRPNKSIHVDEIREIRTGSDVQQYAIDHSI

AEAERTGWFSIIYSDPVKRKTKLMHLIADNLGTCTAWVNFLDAMLKYRQEMMTSLMSFKP

DAVAEYWQRETGKQPGTQHGSDSTGELDFASIKRVCSNLHIYSSHAALEDCFDACDMRRK

SKLNFEEFLAFVSRLNQRRDVQRIIRSVASSPEIGLTFSEFLRFLGEFQKEDITNVQSWE

IVYRTHAGIAANVVLDESVIMTEAAFISFLSSARNGPLEDIPQNYKLDHPVSDYFISSSH

NTYLLGRQYLGESSVEGYIAALGGGCRCVEVDCWDGSDGQPIVVHGRTLTTSISFGEVMR

CINKYAFVSSQEALWISLEVHCNPGQQAIMANTIRETFGARLVTEPLPDREITLPSPEEL

RGRVLIKVKKARDDTPHAEPAGRRRGNSLNSVNSPLARPSMSDTSSFLAPSQSLPQSPLL

SPSLVSRRLVSKSRVNTIAEGEVPDIASISTSTSDNESGSELGSSRHSANKTVPVLGQLG

VYCAGLKFHGFDHPGAKTFNHIFSFMESSFARHSQSKEKKMALEIHNMRHLMRVYPDGIR

LGSSNFDPIAYWRRGVQMAAVNWQTFDLGMQLNRAMFQGGVDQSGYVLKPPELRGIQVGP

YNLDVAVRGKKQRSIVSFSIDVLSAQQLMRPANLPANKSMDPYIEVEVFHANDKRGKDAS

QSNTHQLDSPLKFQTEVIRENGFNPMFIDGHFNFRVTTQYPELVFVRWSVKLSNDGENSK

SKDRTPVATFTAKLCNLKEGYRTLPLLNHAGEQYLFSRLFCKITKHPVVKTMVDAPMAMP

EPGKLNRLGKALGRINTSPRSTIERSNASEKTSMEKSSFES

>SoG_00027.T1

MTASAILSDLPPDATAALASAGSFDPSEKVVIRFKAVGSAPALSQDVCKITASRRFEEVC

FKNGNDQLIVAYSMTPAFG

>SoG_00031.T1

MLSQASSTMRAGTRRMARPSSSTASSPLSQSRVQYTARSFSTIYSLSPYSSHSRSTPVSV

LCAKQHKHNYATTTNPNPPFGKKNASNDKPSRIGLIGGRGFTGQAMINLFEAHPYMDLKY

VSSRELAGQDLEGYSKQKLIYEHLSPEEAAQLEQNGEVDCWVMALPNGVCRRYVEAIDQA

QKDSGSKTIIVDLSADYRLDNTWTYGLPELTPRSEVESANRIATPGCYATAAELGIAPLV

EHLGGDPVIFAVSGYSGAGTKPSSKNDVKVLKDNLIPYSLTGHTQEQEISHRLGTSVSFI

PHVASWFRGIHLTINIPLNKNIPSRDLRQLYQDRYAGEKLVKVVGEAPHVKSVSGGHHCE

IGGFSVDSTGKRVVVCVSLDNLNKGAATQCLQSMNLALGYAEYEGIPA

>SoG_00044.T1

MCFGGRDKDEAGGARSREIDKVIRQDEKKMSKEVKLLLLGAGESGKSTILKQMKLIYSQG

FSKNEKLEWKPVVFSNVIQSFKTIQDAMQELAIDFEDPANEKNMAKVLVEREISPEEPMP

VEYLEPVKALWQDGGVKSAIAKGNEYALHDNLAYFIEDIDRIWAPEYVPTDQDLLRSRLR

TTGITETTFDLGQLTYRMFDVGGQRSERKKWIHCFENVNCLLFLVAISGYDQCLVEDKDG

NQMNEALMLWESIANSHWFTKSALILFLNKMDLFREKLARSPITEHGFTDYHGPADDYKA

ASKYFLDKFRALNRNTEKEIYGHFTNATDTNLLKITMGSVQDMIIQRNLKQLIL

>SoG_00045.T1

MIAVPLLLICGLSGLAALGSASSGSGIARQESKEMILDDAYFYGESPPVYPSPNMTGVGD

WAIGFNKARDAVANLTLEEKVSPAIEAICCSGMIPAIESIGFPGLCLSDAGQGVRGTDFV

SSFPSGIHVAASWNKNLTYHRGNAMGGEFRSKGVNVLLGPVVGPMFRVARGGRNWEGFSP

DPYLSGTLVALTVAGVQDRGVMTSTKHYIGNEQETNRNPSTADNVTIEAVSSNIDDRTMH

EVYLWPFQDAVKAGTANIMCSYQRVNNSYGCSNSKTLNGLLKTELGFQGFVVSDWDAQHA

GVATALAGLDMAMPNDRGFWGDKLVEAVKNGSVPGQRIDDMAIRILAPWFLFGQDKGFPK

PGIGMPQTMTDPHEIVDARDPAAKPIILSGAVEGHVLVKNTKDTLPLRKPRMLSLFGYSA

KSPDLFAPADGLLFTSWIIGAEPASPAAIFTGSSEGDYEAIAINGTMLNGGGSGATTPAN

FLSPFEAIKTRTFQDNTALFYDFRSAKPAVVPVSDACIVFGNVWAAEGYDRPSLRDDYTD

SLILSVADQCNKTIVVFHNAGVRLVDKFIDHPNVTAVIFAHLPGQDTGPALVSLLYGESN

AWGKLPYTVPRNESDYGSAGLRDPSRPEGKFGRFPQSNFEEGVYVDYLHFDRANITTRYE

FGFGLSYTTFEFSSMSAELVDGAKTAELPTGAVISGGQADLWDQLVRVSVLVRNTGNVAG

AEVAQLYVGLPSGEGKADAPRKQLRGFEKVNLEAGQSEELAFTLRRRDLSVWDVTAQRWR

LQPGEYTFWVGSSSRSLPLNGTVQIGA

>SoG_00065.T1

MGPAAALAATPTGALNGSANASARSSPAVGNPSGPTSKSNKKAAANCFNPASANVPLSSL

SSAALDLTSVERRGHPTEFREPTTQPKVRPSGISDAPTYFPSEDEWRDPLAYIKGISAEA

QEYGICKIVPPTSWNPGFAIDTEKFHFRTRKQELNSVEGSTRANLSYLDGLAKFHKQQGS

NLHRLPYVDKKPLDLYRLKKAVESRGGFDNVCRQKKWAEIGRDLGYSGKIMSSLSTSLKN

SYQRWLCPYEEYLRIAKPGVHQQLEAEYGGPLTPSPANTPMKRSNVNTPLSTKADSPARQ

ASEILQASVNGIKEEKDRDTPMVDATPLPKPATSGFTPVNSGGFRAVNAGFTSVNRQSTA

DRRSFTPDTKRFDSPSGEAKNLDSRPPSSLAGATSGKRMSGIEGSADSSAKDGEGETGDG

ENSSRRSKRLKKSAVPTVAGSHMSVFRPSLPRIPREESVTPGEKCETCGKGEDTGFLAVC

ESCDHIFHGSCLDPPLTRKPEHEWNCPRCLVGDGQFGFEEGGLYSLRSFQQKANDFKRGY

FENKMPFDAELNCHRPVTEEDVEREFWRLVADLEETVEVEYGADIHCTTHGSGFPTVEKH

PNNSYATDPWNLNVLPFHQDSLFRHIKSDISGMTVPWVYVGMIFSTFCWHNEDHYAYSAN

YQHFGATKTWYGIPGADAEKFEAAMREAVPELFETQPDLLFQLVTLLTPEQLRKAGVRVF

AVDQRAGQFVITFPQAYHAGFNHGFNFNEAVNFAPSDWEPFGLSGVERLQAFRRQPCFSH

DELLWTAAEGSSTTALTIQTAKWLAPALQRMVKRETAERDEFWARHAESPPHNCNPSEHG

GSGCALDISYDDKDVKDEDEQCCSYCKAFAYLSRFKCLESGKVLCIAHAGLHACCSLSEQ

QRCSGEGHVLVLRKTSEMMASIHSKVVEKAETPQIWEDKYESLLDGEIAPSLKSLRALLH

EGERIPHALPSLPALKQFVDRCNEWVEEAMNYIVRKQQNRRKNDKSWQSGSRKSIGNAEH

DQKERDARHVSNIARLLNEAERIGFECPEILQLQERADAVRVFQSNAAKALESTTSISVE

TIEELLEEGRSFNVDMPEVDHLSKVLDELRWNQKARASRGVFLTLKEVEDLIEEGKRLGI

QNWNDHFQYFREQLRAGQAWEKKARELIYADMVHYPQLEALANQAQAHALPVSQETLNAV

DQIMQRQREAHRQIMDLTQRCRDPDFRKRPKYAEVVDMTKKLEDLNSKPKGTIDLETEKK

RHEDWMRKGKRLFGKSNAPLHILKSHLEYVLERNSDCFDIDHDTPRHPGEPNSRETSIEP

GSSKLDDGKARQVFCICRKVEAGMMIECELCHEWFVSPPITSRRTKLITGRYHYKCLKIA

RGKVKEDDKYTCPICDWRMKIPRDASRPKLEDLVALAEEIPGLPFQPDEEDILEQILDNA

QTFRDHVARYCNPLVSTSAEAETQRFYLRKLEGAEVLLAYETNFFRQELHKWCPVAPEAP

PILEVSLSTRKPRPTKLMKMLVEYGVDNPDDLPESVKGKANSLRRKAANAEAAAAAAAAT

HTSIASSAAMLPHTADSALAGRRTSASHSTDGTAGGSEKNIDPSLSSGITTSPQLRLHSG

SDTSPAVDRRAGDPMDLDHPQPDVLGTIGPRVVMDDSMLSLEERLLAGKEINLQSDAERR

KALEILSRTEMGKRKAEEIFGPDVWNPSHRDGAGASIDGDIEMKEDDSKVDEMFRQMTHQ

DEDEEAKKEPEQAKSAIAEPNKPAEDMSTRDQSVVGKPADEKPEEAKPPEETSAGERTAA

ESKDASGVV

>SoG_00067.T1

MAESAGGIDRKADEKMEFSTSKEVTVHPTFESMSLKENLLRGIYAYGYESPSAVQSRAIV

QVCKGRDTIAQAQSGTGKTATFSISMLQVIDTAVRETQALVLSPTRELATQIQSVVMALG

DYMNVQCHACIGGTNVGEDIRKLDYGQHIVSGTPGRVADMIRRRHLRTRNIKMLVLDEAD

ELLNKGFREQIYDVYRYLPPATQVVVVSATLPYDVLDMTTKFMTDPVRILVKRDELTLEG

LKQYFIAVEKEDWKFDTLCDLYDTLTITQAVIFCNTRRKVDWLTDKMREANFTVSSMHGD

MPQKERDSIMQDFRQGNSRVLISTDVWARGIDVQQVSLVINYDLPSNRENYIHRIGRSGR

FGRKGVAINFVTTEDVRILRDIELYYSTQIDEMPMNVADLIA

>SoG_00070.T1

MANSPHGGVLKDLFSRDRARHDELLAESESLPALILSERHLCDLELILNGGFSPLEGFMS

QADYEGVVANNRLANGILFSMPITLDVDQAQIDALGIKPGARITLRDSRDDRHLAILTVE

DVYKPDKVKEAIDVFGSDDDTHPGVKHLFSIAKEFYVGGKLEAISRLEHYDFLDLRFTPS

ELRSHFDKLGWQKVVAFQTRNPMHRAHRELTVRAARSQQANVLIHPVVGLTKPGDIDHFT

RVRVYKALLPRYPNGMAALALLPLAMRMGGPREAVWHAIIRKNHGATHFIVGRDHAGPGK

NKNGKDHYGPYDAQHLVQKYQEELGIQMVEFQEMIYIPGKDEYMPANEIPEGTQTMNISG

TELRNRLKTGKEIPEWFSYPEVVKVLREQNPLPREKGFTVFMTGYQNSGKDQIARALQTT

LNQGGGRPVSMLLGETVRSELSTELGFSRQDRDRNISRIAFVASELTKAGAAVIAAPIAP

FEDARTQARELIEKSGPFFLIHVATPLEYCEKTDRRGIYAAARKGDIKGFTGVDDPYENP

AKPDLVVDLSKQNVRSIVHEIILLLESRGLLDRV

>SoG_00081.T1

MSGPPPPQKGGLSLYDDLLDPNEKPATISSAPVLYNSQSSNTATEPSSAPPKKPIDPALR

FQPIRRPQQKPSKPKTVTKTAPAAAAPVSDASSQISAPPAQQQPKTTLADWAATEDDEWR

YGTGEKRQRGGRKRKKKQHQPVDTDWDDIYDPTRPTNVDEYLRSDEKIDEVREWKSLLYS

HRREREQPSDLSDDSEADSRPAASSQYTFLRCFLTVLLTFPSDRFAPPASYNFAPPPESP

PDPSVDALPPPPPTTKPTTNNSSTISAAPIRYTQPSPKPTDLDDDGSYSPPPAITASSAP

PQTSLPGQAGFASRLMKKYGWTSGTGLGASSSGIVNALHVKVEKRKKRSDAEGGGWADPA

NKGKILGGQRAPADAAKEGEGRGKMSEVIVLENMLENMPNLQEEISAGLGQEIGEECGEK

VCRCESTSTVCCVLSYQLTTANRTKYGRVERLYIDQERRRVFIKFTDQISALRNWKLETD

EVQAVTELDGRVFNGNAILPKYYDADKFEQGQYS

>SoG_00084.T1

MPSRTSESQDWDIHQDRISDDAVTKSATQAAYGDDSIPSHHLGVKPLGNLYLSDTPNARI

NIGTLQGLPDEMLMLALERLDGMSLQFLASTNRFFYALCESEDLWKSIFLQEQVKRVDWR

GSWRSTVLQFPSDYQADIDCGNVFSDVIHRPFACANISLRPYAERIPKRNQIRRFENLTY

TEFAESWTEQPFILTECIKDWPVLKQWSITSLLEDYAKVVFRAEAVDWTFATYCRYMSRN

HDESPLYLFDRSFAEKMNLGVGQEPGAAYWRPECFGPDLFELLGDERPAHRWLIIGPKRS

GSTFHKDPNGTSAWNAVIQGSKYWIMFPPSSHVPGVYVSQDSSEVTSPLSIAEWLLTFHE

EARQLPECVEGVCHAGEILHVPSGWWHLVVNLEEGIALTQNFVPQSPSLKLVSHVLDFLK

NKPDQVSGFRDDIENPFALFAERLRQHKPDMLETALQMVEERSTKKRKWDEVVVHKEEES

GGFSFGFGGDDDDEIP

>SoG_00091.T1

MKRGESLALIRTWHQEYGKTFKVQLGRPRVITIEPKNVQTVLALKFKDFELGERNKALSP

LLGQGIFASDGQVWEHSRALLRPNFVRTQIADMHVYERHVSNLIKRIPKDGSTVDLQTLF

FQMTIDSATEFLFGESIDSLGAGDSQPKFARDFNLSQEGLAIRTRLGPLMFLHKDRAFSE

ATVEARRFVDQFVQRAVEYRASHSKEAGASDASEEGYVFLYELSKRTADRKMLTDQLLNV

LLAGRDTTASLLSITCFTLARRRDVWDKLREEVLALGDKTPSFEDLKSMKYLNWVLNETL

RLYPVVPLNSRTAVRDTFLPTGGGPDGKHPTFVPKGVDVVYSVYSMHRLPEVYGPDADEY

RPERWATVKPGWAYLPFNGGPRICPGQQFALTEACYTMVQIVRHFKSMESRDDRPFVEGL

TLTLASENGTKVALTPA

>SoG_00098.T1

MHNGTSAATTTTTTTFVDQQNHHGNEDVKLASSAHGGDQDSDDQSDDDNGEDGGYRGRKR

KRPISVSCELCKQRKVRVHLPPPFYRLSTDILDKVKCDRGHPSCGWCTRNGAKCEYKERK

KPGLRAGYGRELEGRLDRLEEVLKSHADILAVLAPNHPQQMLQGSAPSMSSQRPESRDGA

NMMGNPAHALSPRAGAAMFLQRSAGFTPTNQHMGFDQQPPALPGSFQPHMPMPDVSPAAS

MPTGPSEATPQDYYTGGQSGSLSMPASTPVTGDQDMPPYDLLYALVDLYFRHINTWCPIL

HRKTTLDALFGPSTLSETDRILLHAIVATTMRYSTDARLTEERRRHYHQISKQRVLLYGM

ENSSVQALQALVILALDLCGSSNGPPGWNIMALITRSVVQLGLAVETTSALVSPNFTSIY

TLRAMVLPEPRDFIEEESRRRLFWMVYLLDRYATIATAFEFALDDKEIDRLLPCRDDLWM

KNQKVETRWFRSDTSAEDPDEQHQINNPENMGAFGWYVEILGILSNIHKFLKQPVDISAL

SDVEQWQLRYKELDQCLGGWQFSLPNELRMEKLYQTGNKNLNCGWVMLQATYHTAVMRLH

SSAAYPTTRSPIFTPSYSASQRCHAAVESIIAIGEFVVKQNMLSKLGPPFAFTLWVAARL

LLVHGSTVDNKLSPHVGFFVDTLREMGRWWRVAGRYCELLERVLDERGEGERSGEGVMVG

SVRILADMRRTAFDLDFLISRQPRRGTAMGQKMGMEGTKTPRLDDLEYLDVFDFFNVPRL

NGPGMVQGILQQEDMEQAGNGRNGGGGGGGEYNITNFIVDANSDWLFKQDGMA

>SoG_00102.T1

MATGSSGPPWFYPNETSSPSIETFRFKLTESFQQMMFVSGETAEPSVETTGIIEDIVRQQ

VIELLRTCTELAARRGSKSIGMNDLMFQIRHDSAKVSRLKTFLSWKDVRKNVKDSDDKNA

DADLVAGDDPVGVAGGPVDDAAKKNKKAKLTLPWEPVSFYNVEVPERDDEEDEEEEEMNY

ITRQRLHKADERTKLMTREEYVTWSEYRQASFTYRKGKRFREWAKFGIVTDSKPSDDIVD

MLGFLTFEMVAMLTESALRVKEQEDAARAQTGEDNAEGGKKRKHQQGLFDPPTKTAKDEG

DAEWNEVAAAYDTEHLLIEVALISVAEAACLFAFWCFGNLVTTGPGREGIHSVFSALNGT

SGNGEGILEIWSTVLLA

>SoG_00103.T1

MNGTRDTTAPRPKRKAAGSPNQAPPAKRATNGKVSEEDGSDVEFVDYDDEADLDLHAQAA

MYIGTPTSLGEWQDTIQKVVRNVVAIRFCQTCSFDTDSALTSEATGFVVDSERGYIMTNR

HVVGSGPFWGHCVFDNHEEVDCYPVYRDPVHDFGILKYDPKAIKYMQVDGLSLSPDQAKV

GVEIRVVGNDAGEKLSILSGVISRLDRNAPEYGEGYSDFNTCYYQANAAASGGSSGSPVV

SKDGSAIALQAGGRSDGASTDYFLPLDRPLRALQCIQQGKPVTRGDIQTQFLLKPFDECR

RLGLSPDWEAATRKAFPDETNMLVAEIVLPQGPSDGKIKEGDVLIKVNGELLTQFLRLDD

ILDSNVGNTIKVLLQRGGEDVEVDIEVGDLHKITPDRFVSVAGGSFHDLSYQQARLYAVP

VKGVYVCESAGSFRFESTDNGWIIQTIDHKKVPDLAAFIETMKNIPDKARIVVTYKHLRD

LHTLNTSVIYVDRHWSSKMKMAIRNDKTGLWDFKDLGDPLPPIPPKRRSAAFIELDHMPT

PEIADLIHSFVHINCTMPLKLDGFPKNRRWGMGLVIDAEKGLVLISRAIVPYDLCDITIT

IADSIIVEGKVVFMHPLQNYAIIQYDPSLVDAPVKSARLSTEPLTQGAKTHFLAHNRIGR

VVHAATTVTEITAVAIPANSGAPRYRAVNIDAITIDSNLGSQCNSGVLVAPDGTVQALWL

SYLGERSPCSQRDEEYYLGFGVPAVLPVIQAIQKGEKPKLRSLSAEFRAIQMSQASVMGV

SDEWIKKVTQANRSHHQLFMVSKRTFERVEPAVSLVEGDIVLTLNGKMCTTISDFDVMYG

EDVLDAVVVRECEELHLKLPTVSTDDVETDHAVSFCGAILHRPHQAVRQQISKLHSEVYV

SSRIRGSPAYQYGVAPTNFITHVNGQPTRDLTAFIEATRKIPDNTYFRLKAVTFDNVPWV

ITMKKNDHYFPTTEWIKDSNEPCGWRRVTYEGTDVFQGESTDGVPTVAEESEVGVGVDVD

EVA

>SoG_00109.T1

MKSPDASTTSPPPIEVTICEAPQHIIIRISDRGGGIPRDELPYLWSFSKGSQRHKRLRDL

SKVPKMAATMQELHTEAELGRADMKTPPHQSSLSSLSSSRPPHLRLGMGLPLSRVYAEYW

AGTLELHSLEGYGVDAFLQISRLGNKNEQLVTRAAMDAV

>SoG_00126.T1

MPGASVAVLPPPAASTPSSRKASLAPERKYKCQFCNRAFSRSEHRSRHERSHTKERPFKC

MKCRSTFVRRDLLLRHDRTVHAKDGGVPLHSDGKRRAGPKTRAVGGPSKSSLALDPGALE

QMEAGNDNIFDVEAAAMLVADLHHKATASMRDESNYEDSPSMSYSPHNSSIMESTVTYPS

GAIALPQVQWDGFMSQSVTEPKAHSITSSAGSFDSPQAFAAASGRHAHPNQLPPLSGRSN

GLVPAIHAMISSLPNSGAGTPVPQSPARGESAQAGFRVPQVANDDERHMILDNIRKHDIE

RSIIDSFRLPGLGSLNRYLSTYFGLFHHHLPFLHPTSFQPSQVSSPLLLAALSIGALYAF

DQEQAYMLHIGSKMLVNQFLQNKESFSSRKCPLWTMQATLLNMVFASWSGDPKGLEWACS

IKSLLANMVAGNKYELKLRQEARGSAKPSRAEWVEDEGCRRTYYAVYIFFGLLTLTYNHT

PAIGFNEFEDLQLPSTEALWNLDVTDEATWPEKLQSSPIVSFIDAHDMLFQGENMRYSAF

GARVMINALFLEVWYHKRSPEALQDVVTEYKLRLALETWEKSLEFCEPENSTVPLSAPHK

GHPLIFNATAMLRNARVRLEVDLKSVQEALRYHDPYEVGAAMSNARDRVKRSSEMIKVIE

ECYNCIQTAVVQGIRWVARTSPTNWSVEHPLCGLDLMIILSLWLYRLEHDEEPATQEELI

MYNKVRQLFAKDIAESLIGNLSSVVARLWGEMLDEVVVWGITRLMGESFKLHAQALVGYV

DDPEASSNPSTPSMTSQGADEDSVY

>SoG_00177.T1

MADGEDSTATPVASSEVQSRLISQDNDEQYQNTRDFVRQRSLELIRSIGDENPFLSSNPC

LDPKSPQFDPKAWLTALLGTRTKDPERHPRQDVGVSFRNLSVYGDKDSITYQNDVLTWPI

KFPKLFLNALGNRSQGTPILHDFDGLVQSGQMLLVLGRPGRFVLGSETGPSTTLTDRFKQ

NSGVSTLLKTLAGVTSGLRLDSKSIVNYEGIDFETMRKTFRGEAIYQAEEDVHFALLNVA

FTLEFAALMRTPQNRFPGVSRSAYAKHVRDVVMAMYGITHTASTKVGNGFVRGVSGGERK

RVSIAEVTLSQSAVQCWDNSTRGLDAATAVDFTKSLRLSTEMTRSTTIAAMYQAAEDSYN

MFDKVTLIYEGRQVYFGPVGQAKSYFTDMGYHCPDRQTTADFLTSLTNPAERTVREGYQT

KVPRTAAEFAEFWRKSEPYADLRKEIQNYESSHPINGKEVEKFLAVRQVRQSYLMRPKSP

YTVSVPLQIWYCVVRGYHRLMQDLMFFQFTVSQNQSTGLIVGSVFYNLPTNAESISNRLT

LIFFAVLFNALAASFEIIPLYASRPVIMKHSRYALYHRFSDAIANMIMELPNKILSNLFL

NIPLYFLSNLRREPGPFFTFLLFGFLSTLVISNTLRTLGHVTRSVYQALAPYALIITAMV

LYTGFVLPVRNMQGWLRWLQHLDPISYAYESLVINELHGRRFECNTFVPTYENAGALQRT

CGTRGGAPGSAFVDGDDYLRTQLGYDPVHIWRNFGIIVAFIVATFIFYLVVVETIDVDGA

QADTLVFLRGANRHAGTNGPVDEESGPNISIQEKNSAELSNDSEAVDQGDHPSTPHTPGG

LFRPKSALARSEARVFQWKNLCYDITVDKENRRLLDEVFGWVKPGSLTALMGATGAGKTT

LLDVLADRVSTGIVTGEITVNGLPRPADFQSHTGYAKQQDIHLPTATVREALRFSAVLRQ

RDASSKAEKFAYVEEVIKTLDMERYADAVVGEPGYGEHVKRLNAEQRKRLTIGVELAAKP

ELLLFLDEPTSGLDSQTAWAIGDLLRKLADQGQAILCTIHQPSALLFEKFDRILLLEKGG

KTVYFGDLGVDSRTMINYFEGHGAPTCETHMNPAEWMLNVTGAGIGGSAGVDWAEIWLGS

KERVERFAEMEEMGRHVGSGDGAAKVRESKETYATSFTTQFRYTLVRIFQQYWRTPSYIW

SKLLLVIGVSLFIGLSFQGMSLSLQGLQNQTFSIFLLLTILIFMAYQTMPHFIVQRSIFE

ARERASRTYSWFVFVLVNVVVELPWTLLSALFMFVTWYYPLGMNGNAQGDGAERGGLMFL

LLWAFMLFAATFTDLIIVAIETAELGALTALTLYISCLAFCGIIMPRSGLPGFWNFMYRV

SPLTYLVGGMLSAGISGNDITCSDLELLRFEAPANVSCAAYLAPYMRQAGGRIVDGGGGD

LCQFCPFATTDAYLERVEIYYHQRWRNFGLQFAYIAFNIVAALGIYWIARVPKGGGRLKS

RK

>SoG_00187.T1

MPPQTDVRGETEAPPTVITTGPEPETNPNASGTSTPAVRFSSAVDVAPDPPAVTTTNDHD

DDAQDDGHQSFSDVAADQLRALTKSIHGRPLQSKRLTTSYHFEAFSLPASRVQSREDDSH

DSTRLPTPSSTHRQSPHSSPRMSAISSPPLTPSGSGSHGSTEQKQENKEPSERFPGTEIP

TITPEPSSSSHERHGSGDQRVLSHRPRSEGSDIRRASSAEHAPHNEQSHRRGMFKVGPGS

VPGSRESSPSRSSASHFYSKPATPYGDANDPYAKGKRPAQQGFNRQSIDPRFVFSRKKKH

GSPGSSKTNLSEKRNSGIFSGNDSTVSDLGHHPQGSMADLKRFFRKSGHHKKRESSPAPS

GTRTPPTSRSTHQLPFDEHHDLQSKYGKLGKVLGSGAGGSVRLMKRKEDGTVFAVKEFRA

RHPYETEKEYTKKVTAEFCVGSTLHHGNIIETLDILQEKGRWFEVMEFAPFDMFAIVMTG

KMTREEISCCFLQILNGVTYLHSVGLAHRDLKLDNVVVSDKGIMKIIDFGSAHVFKYPFE

TDVVPAKGIVGSDPYLAPEVYDSREYDAAAVDIWSLAIIFCCMTLRRFPWKIPKMTDNSF

KLFAAEPTPGHDPNKLIARAKSTNDLSNTRAREFLTGDEGKDRLPHSQHHQHHSHQHESS

HPKDEKSEDGSAAPANKSSGEKKETIRGPWRILRLLPRESRHIIHRMLDLNPKTRATMEE

ILRESWVADTVICQQLDDGTVIPAEDHTHVLEPHSSQGQQK

>SoG_00190.T1

MGDGIKYNEEQEPKEALWKPSAHDVPEAPMTYSSGQSACLIISLGGLTCSACTDAVASAV

KALPGVSKVRASLTMQQATVIAHNNVRLDHGKILRAIEDLGYEATIGPRAPNEVLDMLEH

KQRIASLGATCSRFTQCAVVLQALAWGRHVVPGSSTSSQSFQGFLDVASIFVMIYVQFSP

GYEIHRDGGKWWRTGRLNMNTLTSLAMCLGTALPLLECFAEGRYGRHPDYTMAVRLGLVV

AAGKYMDSLSRTAAARDIGAVYKQMLETDTATLHPDMQVVPSAYLQPGDKIVIEPHTIVP

CDCYVLEGTSLVGMSIVTGESLPVKKAVGSFLLGGTRNLNVRLICAAQKEKGESFYTNLV

QSAVERASASSSEDQWIDTVTRYFVLSAIGISLATTLIQGYSSIGALPLIDIIHAAMLRT

VTIPTCACPCALSMAIPSATVAAVACSRGILLTGGIDTIDKLSQSHCVLFDKTGTLTHAR

LDVGDFLVMDHSHWSTSILWDYACALESQVVTDHPIAHAIFSSGLKELGSTWTDGQHLRA

HRNVEPALGRGVKGDIQLPKLPWCEVVIGSARFLADCGYNLIKVPAVNTEGIIVVHMGVS

KQYAGTFLITDVIRDEARDVVQSLIADGIRCGMLTGDTEEAAARVSRQVGLPLAKARALP

DTKQGCIKDLQNKGYIVTMVGDGLNDAPSLAPADVGVNFRKNAASATISGAVTIVNSKLS

PLPALYRMARMTMDQVRFNLCWIFAYNVVALALATEVIAPFGMRLSP

>SoG_00191.T1

MSHQDTLQETKPLVVSGPRTPALWTKTLSTLIDEQAALYQDADAIVVPWQSARLSYRQLS

YRSKLVARCLLARRLRHGDTVGVMAGNRSEYLEVFTAAGRIGCIAVVLNNTYSPDELISA

VTQAECKIVFMGSHVGNRSLQDHIGRLLKGKALTPKVVCFGDSSRPEVERYADFVSRYLP

QSVASEAKLKEAERTVNATDVLNLQFTSGTTAKPKAACLTHSNILNNARFVGAAMKLTQA

DVVCCPAPLFHCFGLVMGFLATICHGSSIVFPSDSFNAEATVKYAAQERATALLGVPTMF

IAEIEALEEKKYDISSLRTGLAAGAMVTPALMKHIHDKMGIKGMLIAYGMTETSPVTFIT

SLDDRIEKRYNTMGRVLPHTAAKVVDSEGKALPVGSRGELCTSGFALQKGYWKDDERTRQ

VMRYDSEGVLWMHTGDEGYLDGDGYGHVTGRIKDLIIRGGENISPSDIEDRLMEHESIGE

CCVVGLEDHKYGEVVAAFLGQAKSNPTRPSDQAIRDWVSQRLGRIKQPKYIFWVGEPAVG

PIIPKTGSGKYQKHLVRALGNKLVREGAVAKL

>SoG_00207.T1

MVFDKTNEPRGDGLSDMDIQPVSLEKRGRTPSDATTIRPPTASSDPFATPSLRSRSSTLA

TVACPPPVNTSEALRPDPGTEADFVVKDNPFAFSPGQLGKMLNPKSLDAFRALGGLKGIE

RGLQADITTGLSVDETTVANRISFEQAVGDLQKKPEMSPHHSTGSGAYADRGRVFGRNVL

PAKAATPLYKLMWNAYKEKVLIVLTIAAVISLALGIYETVGAEHPPGSPTPVDWVEGLAI

CAAIIIVVVVGGLNDWQKERAFVRLNAKKDDREVKVLRSGTTSMINVAELLVGDVVHMEP

GDIIPSDGIFITGHEVKCDESSATGESDALLKTGGEQVMRMLESGSKSKDLDPFVISGAK

VLEGVGTYLCTSVGTHSSFGKIMMSIRTTVEPTPLQTKLAGLAIRISQWAVTSASFLFFV

LLFRFLANLGSDDRSPAEKASFFMEIFIVAITVIVVAVPEGLPLAVTLALAFATTRLLKE

NNLVRVLRSCETMGNATTVCSDKTGTLTTNKMTVVAGTFGSSSFAKSEPIQAGAQSSVSS

WASGQTQSAKDIIVQSVAINSTAFEGEEAGQFCFVGSKTETALLQFARDHLGMDSLPLVR

ENEKTVQIFPFNSSKKCMAAVIQLRSGAGYRLLVKGASEILLGYCNAKASMDDLSESPLS

ASDNMSLRSTIEVYARQSLRTIGLVYRDYPSWPPAGVHASEDGHVQLGALLKDLIFLGVV

GIQDPVRPGVPEAVESARKAGVKVRMVTGDNVITAEAIARECGIYKQGGLIMEGPVFRTL

SDEEMTRMVPKLEVLARSSPEDKRILVRKLKSMGETVAVTGDGTNDAPALKAADVGFSMG

ISGTEVAKEASQIVLMDDNFRSIIVALKWGRAVNDSVQKFLQFQITVSITAVILAFVSSI

SHPEMKSVLTAIQLLWVNLFMDTFAGIVLATDPPTDRILDRPPQKKSAPLITLNMWKMII

GQVIFQLAVTVTLHFAGGKMLGYDETDEHRALQIDSMIFNTFVWLQIFNELNSRRLDNGF

NCFTGLQRNPYFIGINLFMVGCQIAIMFVGGKVFSVTAMTGVQWAISTVVPALSLPWAIL

VRLFPDETFGKVVKVVTWPFLKIHAGLAIIFSPLSRLVSRKKKQGGAVADQEKGRS

>SoG_00220.T1

MAENDPKPNQEQDINPWSVAGAQNEAGEVVAIDYLALSKKWNTSLVDEAILERFEKVTGH

KPHRWMRRGLFFSHRDFERILDRYEEGKPFFLYTGRGPSSGSLHLGHTIPLEFTRWLQEV

FDVPLVFMLTDDEKALFNEKLTFEAALEFAMENARDIIALGFDVKKTFIYSDLKYISNHI

LMNAWEFAKCIPFNQVRGAFGFDGSTNIGKIFFPAVQCVAAFATSYPEIWSDEPAKTRSK

EIASIPCLIPMGIDQDPYFRLLRDNAHRMRYPSPKPALIHSKFLTALQGAGGKMSSSNPN

SAIFMTDTAKQIKTKINKHAFSGGQETLELQRELGGNPDVDVSYIYLTYFEEDDEKLAQL

HKDYKSGALLTGELKKLAIESLQKVVGDFQERRAKVTDEVLESFMKPRKLVWGGNPNPKP

KEAKKDAKETKELPNLDFARYKASQRGANAYAVMPPSPSYERPLSPAHDSDSDLEFDLQE

LDPISTQPARGSAPAPSDAEPKSSRIALRNLRMGGPRRANKRSHAYGELGKDRDDGDEEA

LLADDGSRQRWSDGHGEADEPLLGDSQPSRRRQRLSSFSSRVRLPSFMSGGKSQAEEDSE

EQEDDDSSSSRVVVVGASQPTRYPSNMISNAKYTALTFLPITLYNEFSFFFNMYFLLVAL

SQAIPALRIGYLLTYIAPLAFVLCITMGKEAWDDIERRRRDTEANSEEYKVLEFGDPQSS

FSTSRPRRALRSESYKKKSKRQSRSRHDLSDIREEAEDDQGEYAQPSSQVREVSRKSRDL

KVGDVLKLSKGQRVPADVAILQCYTTDGPSQVAAKEPTEEESLLAFGDGEPSEVSKGKQP

AREPTSDDADNDSTGETFIRTDQLDGETDWKLRLASPLTQMLPAEEFVRLRVTGGKPDKK

VNEFFGTVELLPTRQAAMSQQAVLPSSEDESDSKTAALSIDNTAWANTIIASQATTLAVI

LYTGPQTRSALSTAPSRSKVGLLEYEINSLTKILCALTLALSIVLVALQGFESTEDKQWY

VKIMRFLVLFSTIVPISLRVNLDLGKSAYSWFIHRDPGIPGAVVRTSTIPEDLGRIEYLL

SDKTGTLTQNEMEMKKIHVGTVSYANEAMDEVTAYVRQGFHIQLTTDPATHSMLITPSST

YSAPANAGATRTRREIGSRVRDVVLALALCHNVTPTMDVENGEEVRSYQASSPDEIAIVK

WTESVGLRLAHRDRRSMALESTDTGRPVVRVRILDVFPFTSEGKRMGIIVHFHTDIKNQT

PDLTSGEIWFYQKGADTVMGSIVAANDWLDEETANMAREGLRTLVVGRKKLSYQQYQEFG

SKHQEASLALTGRDAGMQRVVSHYLENDLELLGVTGVEDKLQRDVKPSLELLRNAGIKIW

MLTGDKVETARCVAVSSKLVARGQYIYTVAKLKRKDNAQDHLDFLRSKTDACLLIDGESL

TLFLTHFRLDFISVAVKLPTVVACRCSPNQKAEVAKLIKEYTKKRVCCIGDGGNDVSMIQ

AADVGVGIVGKEGRQASLAADFSIEQFYHLVKLLVWHGRNSYKRSAKLAQFVIHRGLIVA

DWLLVGYATIYTAAPVLSLVLDKDVDENVATLYPELYKELTLGRSLSYRTFFVWVFVSIY

QGGMIQGLSQILVDVDGPKMIAVSYTVLVLNELLMVAIEITTWHPIMILSIVGTFVAYIG

SIPFLGGYFELAYVITWGFVWRVLAIGAISLVPPYVGKIIRRTMKPPSYRKVQST

>SoG_00227.T1

MVQNKTFVYKKIPTELPVAGEHLVVEDRPIDLEAPLAPGGILLELQYASFDPYLRGKMRD

PGTNSYAAAFNVDDPVVNTCIGKVVRSSSDDYAEGDLVLAYVGIAEYAVVPDPKAQMVRR

VQNPHGLDLGHFLGALGMPGLTAWSGLHRIGRPKKGETLFVSSAAGAVGQIVGQIAKREG

LRVVGSRSTP

>SoG_00253.T1

MSQAVKRACDACHRRKVKCDGINPCRNCSSAQLSCTYNAIPQKKGPKGSRAKVISELRET

QRQTSLSAKVQNRINGIVCPQANQGLTPTPGLVTGELVKECVHFFFEHMYGQLPILDRAQ

VEQQILYMEQNRDAYCLLTALSAFVMLQPGMSMPSGDPFNLDMLPGANIVSSQLLLEECL

RVRKGYEFLDSITLNTLATNFFIFGCYHGQEMVDRAWFHHREATTMIHIIGMDKEEFYMS

LDTTESSRRRRLYWLFYSLDRSYAMQRGRPMTLQATINLPTTGDDPSDAMSHQINGFISM

VNLFRPFDSSFLATWNKTRSQLSAQYLSGLQKQLVELVQSYQCQDSNITDLHINQQWLRN

TVWQLTNGNVGGDENMPFQYPVGMARELVVNMASQFSNPANELVNAGLIEKLIEIVYSMN

DYLSVQPASRDPFTLGPRQYLEQLLSIVAVVRNGDFRFLPLLLNKVTEILPRLANPMLLN

APETANMANIDIFDGFGNAGMAQPPPQLQIPLDEYDRKFPVEDYDKQYSMDMQQHSTPDS

STNSQHSGAQPTPQGNDMKGSFVSSPGIMSPGVEYSHGHNMNNFACTPISDMVMSPLGNQ

PNPLNGGQTQHQHLANQQINRNSGIANGMASPHPMNGSMYNMRQPTQRQQSFHLQHPPQM

RTVGDFQGLQRGGADNGGGMVGMAQMAGELDFSGMR

>SoG_00260.T1

MADLPPYNGTKDHADSGWDTGVHNLNIFYDTGNMAWITVSAALVLLMIPGVGFFYSGLAR

RKSALELLLLSMLSVAVIAFQWFFWGYSLTFSRTGNSFFGALDHFGLMNTMGQDVGGIPD

ILFCLYQGMFASITPALAIGAVADRGRILPALVFIFIWATVVYDPIAYWTWNANGWLLNL

PSYDFAGGGPVHVASGTCALAYSLMLGQRTGYNKNHGLPYRPHNVTNVVLGTVFLWVGWF

GFNGGSALGMNLQAIFACYITNLSAGIAGLTWVLLDYRLEKKWSTIGFCSGAISGLVGIT

PAAGYVPAYGAVVIGVVSGIACNFATKLKFLIGVDDALDIFAVHGIGGMVGNILTGIFGA

SWIAGLSGAEKDPIGWVEGNWIQLGYQLAGTCAAFGWTFVMTCIILFLMNLIPGLSLRAT

PEEEEMGMDDCQLGEFAYDYVELTRHVADSASISDMPTGAIASSQTSTERPKEAV

>SoG_00272.T1

MSALHRNTPLHKRAAGGILFRVLGQEHATMESYSSYREPAGRSPVERSRGKDDSRGFDRR

DRRRSRSPAMVDRYEPRSRRDDRDRDNRRRASPPPTNIDRYVPGQDGGPAASVAVNPLAD

PSQLPYQVGFSYFGEWWRMEEKIKDDLERARTGRRREPRAPEDREKEKGKIQAAYDAYKE

ELQAKMARTFVAEHKKEQWFRERYVPEVRDEIRSRLNEFRRGAYSQWEQDLESGTFDEFS

LEGMPKGDGNGAGGSADKEEGEATSNEILGVGDLVPTNGADIRDENQFQPTLLIKTIAPH

VSRQNLEEFCRENLGEEEGGFKWLSLSDPNPSKRFHRIGWVMLHPSSEAPLSGDTDDDIK

LDDEGEATEPPKPVSTAEKALEAINGKTVKDEVRGDFVCHVGIHNPPVTPRKKALWDLFS

APERIDRDLHLIHRLVNKFEEEFGSDFQAILRIEDKVEELRSAGRLQPPAVPTPAKKVKQ

ARDLDMDAAMDGEEDGMVEDDDQGGVEEDVDVDDEEVMAKKKQLDLLIEYLRRVFSFCFF

CVFECDSIHELTRKCPGGHLRRPRNTLSSAARAVARASANGEPFPEKKRNEPLEEGEEPT

DGERKFKATSKAEQQLSRAFNWVKTFEDKLLQILEPDSIDLRKLGGREVESGIEEEVKKH

VKQEDEHKWRCKVPECNKLFKEEHFWRKHIDKRHGDWIDTMKQELELINAYVTDPSHIAP

SRTDANSNGHFPPANGQSNAGTPRGFNLQNFAMNGMMGMPGFPMNNFGTMFSAMQGSKWA

GPEDRSGAGGPIRRGGMAGGRGAYRSGPYDRRPGGRHDGGGGRGRQGGSRWGDGAGAGAG

GPREAVQGRSLKSYEDLDHVAGSGSGELNY

>SoG_00274.T1

MRVLDLAWLLGASCVLAAPQPAAAACSTVTLTSTVYVTGALPKTTTSAKTTSQTTSSAST

APTNIKPASGYRNILYFTNWGIYGAQTFPKDIPADKLTHVLYAFGDINYNGEVISSDSYA

DVEKQMPGQTIDWNSPVKKAYGCVGQLYDLKKQNRNLKILLSIGGWTYSQAGKFKSPAST

DANRQKFAASAVKLMASWGFDGIDIDWEYPDNKQEGNNFVLLLKALREALDNYAKRNNQN

YRYELTVAASAGPSKYDLQDLKGMDNYLDAWHLMTYDYAGAFSSLTGHQSNLYKDPNNPG

STPFNSDSAIRDYISRGISPSKIVFGFPLYGRSFGNSQGLGKPFSGPGKGPLEAGMYLYK

NLPAPGARATFSDVTGATWSWDSSTKELVSFDGPKSTNFKADYIIKKGLGGSFFWEASGD

QKGEKSLVGVMAQRFQGKMQQKANMLSYPESPYDNIKNGQKS

>SoG_00278.T1

MSQSKDFTVAVVGGGIAGLTLAIALDQRGIPVTVYERASKFGEIGAGVSFTPNAVRAMQG

CHPGIAEAFNKVCTRNGWPSKQKVWFDYVDGVESQTRFSITNNLGQNGVYRAHFLDELVK

LLPPEKASFGKCLEEIVDRPGEKVMMKFADGTVAYADAVIGCDGIKSKVRQFVVGADHPS

VKPSYAHKYAYRAMVPMDKAIEAVGEEKALNACMHMGQGSHVLTFPVEHGQRLNIVAFHT

TEDEWEDWERTTKLSTRDHAFRDFAGFGTDIQNLLKLTEEPLNVWAIFDLGDNPVPTFTN

GRVAISGDAAHATSPHHGAGAGLCIEDGVVLAELLADPSVRTASDLAPVLKVFDQHRRER

GQWLVQSSRWIGDCYEWQAQGIGRDLEKIEREIRERNSIIADFDIEGSIDTARKDLQAAL

GCARL

>SoG_00283.T1

MATAYLAQPYAPRARPSYHHSHPPSPPMEESKYSLPSISNLISLADAGSPTSEAAPSQLS

PRPEGPSAQRHRLETSAVKPDSRPGSGYAPGLQGSRGLPPTPPMSVDASFESRNSPSAQS

TSQLSTASGPTGYYHETTPPLEADARRPPPAPRSQLPHYSQQAPAPAPYHPGPPAGLYAQ

APPPSQSLPQVHSLFYQRPLPQAFPPPMPVAAGGSNPWQHHHYLNPSHGPSYPQSQDRYI

CQTCNKAFSRPSSLRIHCHSHTGEKPFKCPHAGCGKAFSVRSNMKRHERGCHSFEVTPGL

AR

>SoG_00304.T1

MSSIKNIAFAALFLAADFAAAHSVITAAVGNAGGSGMALGVDTSTPRDGTRRQPFQTDST

RFRGNSAATVGETLAGGANNIQQGTAAIMAETGSQLPQITPGGNVQMTIHQVNADGAGPY

TCMINADATGNNWQNIPVTTNVAGNNRGRNRDGQMGDFPLVAAIPQGQTCTGTVNNANNV

CLVRCQNPARAGPFGGVVPVQMAQGGGNAGDAAGEKPAASAAAAKPAATKAATKAATNPR

SLKGRAVAYFA

>SoG_00316.T1

MDTNMEDVSRAPADVSPVQAEPATIPTLDGWIEALMSCKQLAESDVQRLCDKAREVLQEE

SNVQPVKCPVTVCGDIHGQFHDLMELFKIGGPNPDTNYLFMGDYVDRGYYSVETVTLLVA

LKIRYPSRITILRGNHESRQITQVYGFYDECLRKYGNANVWKYFTDLFDYLPLTALIDNQ

IFCLHGGLSPSIDTLDNIRALDRIQEVPHEGPMCDLLWSDPDDRCGWGISPRGAGYTFGQ

DISEAFNHNNGLTLIARAHQLVMEGYNWSQDRNVVTIFSAPNYCYRCGNQAAIMEIDEHL

KYTFLQFDPCPRAGEPMVSRRTPDYFL

>SoG_00331.T1

MGGLRSRIISTLLLCVSSLGLSQANEYVLSAPRGSRGAVASEAVECSSIGRDLLAKGGNA

VDALVGTVFCVGVVGSYHSGIGGGGFVLVRDKDGNYEAVDFREAAPAAAHEDMYKDNVLS

SMIGGKAAGVPGEVRGLGYIHAKYGVLPWKDVMEGAIYIARNGFRVSADMERIMHDAMNA

YNYKFLSEVPDWAEDYAPNGTLVKTGDIMTRKRYADTLEKIASEGPDSFYRGEIAEAIIN

ALDRSDGIMTLQDLANYDIISRNVTTTTYRNLTLHGIGSPAGGAVAFHLLKTMEHFPPDT

FSEDEPLTNHRLVEAMRFAYGARLQLGDPDFVENVGSMEKEMLSDERAKETRKRILDDAT

QPVENYLQKKVFLRDSHGTSHIATADASGMATSLTTTINLLFGNLIVEPKTGIILNNEMN

DFSIPGEPNEFGFPPSVANFIRPFKRPLSSCTPMIVTSNEHSNPPSFLATLGAGGGSRII

SATAQVLWHVVEHRLTMADAIAKPRLHDQLIPNVTALEHAMRESESVVDGLVDRGHEIVW

VPPTKSAVQGILRGADGVFEAAGEYRQKNSGGLTL

>SoG_00336.T1

MLQPRLQLSVLRASFQALSLNQPATRTYAAVARKSAISRKKTTTASDDVADEAVVKAVEQ

PKSGSSIVRTYKPRTPGVRHLRRPINDHLWKGRPFLPLTFPKKGHAKGGRNVHGRITVRH

RGGGAKRRIRTVDFERRRPGPHLVERIEYDPGRSAHIALLTEQATGQKSYIVAADGLRAG

DIVHSYRAGIPQDLLDSMGGVIDPGILAAKTAFRGNCLPMHMIPVGTTVFCVGSAASRGA

VFCRSAGTSAVVVNKNEETKDDGTKVMTGKYVEVRLQSGEVRRVSKDACATIGVASNVHH

QYRQLGKAGRSRWLNIRPTVRGVAMNKVDHPHGGGRGKSKGNRHPVSPWGVPTKSGYKTR

RKNNINNWVVTPRPRNHGKRRSKNT

>SoG_00353.T1

MATTAPRPRDLRPCDNCRHRKIRCLFATDDSVNCVLCQSRATTCTYVREPPRKKRALSKD

KDLDNTAGTSPQSRQHRTASQSVSAGPVFAKLKRKSTQDGTSPVVRDYSQLPGQSLLKET

LGHQNRQSSSVIGASSDFDPCLTQSLPWDERGVFSGFRAPHVLRRANASTQFFLRPDTQT

EMDVELANLDMIESFVSPHGPELVKIYFRIVHPTFPILHKNVFLEKHGRSYRELTPIGLG

AVYVLALNWWSYSQALSSFPKPDAKELEEIVLRMLVDAHRRPKISDLQGGLVLMQSPNVD

SWAMTGHLVAMAQNLGINVDCMDWQVPDWERGVRKRVAWALFMQDKWGALVYGRGSHIRA

DDWDIRPLDSSDFPETAKDDSNEEGSAEIEKGKLTFLHMVSLTQIVAEILDSFFTLKAAR

RASTIHDVLETAKPLQIQLKAWHASMSSSLSLEEIVPMKLSSVGYLHLAYFTAEITLHRA

IMRSHDNATPSPPDHHLYTITRQAAEARFISALEFVRRLKAEHFQSFWYFSSSVSLAIIG

IFGAVLCSAAATQTERERYVKKLAEYRWLLRLSANSASFMQYAVGVLDACSQLLEEQAAS

DGASMPHAVTEEVVGLGDWAIQGSELGRAAEDMSGSGALDDDAFAAFLMHENGLALLQVE

QSEWLQGRVPDFDCADMSRFQQTPTK

>SoG_00357.T1

MSSSASTPKVAQAAAFPDGTTDYKPLRSGQEWDSKKVHIADQPITWGNWYQHVNWLNTTF

IIIVPLIGFISCYWVKLHPYTAIFAVLYYFWTGLGITAGYHRLWAHTSYKATLPLKIFLA

AGGSGAVEGSIRWWSHGHRAHHRYTDTDKDPYSVRKGLLYSHIGWMVMKQNPKRKGRTDI

TDLNEDPVVVWQHRNFIKSVIFMAVIFPTLVCGLGWGDWWGGYVYAGILRVCFVQQATFC

VNSLAHWLGDQPFDDRNSPRDHLVTALVTLGEGYHNFHHEFPSDYRNAIEWYQYDPTKWA

ITFWKWIGLAYDLKQFRSNEIEKGRVQQLQKKLDQKRATLDWGTPLEQLPVVDWDDFVEQ

SKNGKALVAIAGVIHDVGDFIKDHPGGKALISSAIGKDATAIFNGGVYNHSNAAHNLLST

MRVGVLRGGCEVEIWKRAQFENKDMTYVEDSTGQRIIRAGNQVTKIVQPVASADAA

>SoG_00372.T1

MATIKPIESRTVHQIQSGQVIVDLCSVVKELVENSVDAGATSIDVRFKNQGLDVIEVQDN

GAGISAANYESVCLKHHTSKLSTYDDLASLQTFGFRGEALASLCALSTLTITTCLASDVP

KGTGLSFEASGRLQGTSVVAAQKGTTVSVEKLFHNLPVRRRELERNIKREWQKVIALLNQ

YACILTGLKFSVSQQPSKGKRIILFSTKGNSTVRENIINIFSAKMTNQLVPLDLQLEMKP

TRSGHGLRVSNNDETQLDASKEVRVVGHVSRPVHGEGRQTPDRQMFFVNGRPCGLPQFAK

TFNEVYRSYNSTQSPFICADIRLDTDMYDVNVSPDKRSIMLHDQNQMLDTLRASLISLFD

DHEYSMPVASMSRNTVAKPDPRPSQPSTFRFRKQDPEPASPSSAESEVDNSDGNLNEEDE

NPSPEQQRTNIDLSNFTNGSARRIPSANSISMGGPHSQGLLRHWVASKTGEPSRGPRPPA

ELAGGSSSDSNRSRGRYIAAVASPQSSGVDGNAETETEKPTGDNIGSPEQRLSETCGAED

DLVHQSVTSRASEATPSQGDSRVASDSEDGQRHSRYSKRAAPQLATLTIGDETTTSWIGS

SAKRSRSATPEKPQLSFGSRLSQMFAAGGPSSSTRGIGGTRQTLSTSDSSENEDEGNSDD

SDEAPREATPAPPHSLDNSGASSSGHEDSLFVPDEMGPDDEPATLPKMHVSEEAQPRAAA

FEAARRRKDTTTVLKQVLEMSEDNVTNAVAKWKARTVKHQGDACTLDNVEDITHPDAEAK

LSLVISRGDFSRMRVAGQFNLGFIIATRPRNSMLFGSETSEGDELFIIDQHASDEKYNFE

RLQSETTVQSQRLVQPKQLELTALEEEIVMHNLPAIEANGFKVHFDISGDSPVGARCQLL

ALPLSRETTFDLADLEELISLLGDSSNESSHVPRPAKVRKMFAMRACRSSVMIGKALTQS

QMYGLVKHMGEMDKPWNCPHGRPTMRHLCRLQSWDTKGWKNDIGVASGSSWAEFMGT

>SoG_00375.T1

MDDSATYGSPSHGATPDESSVLSRPPLQQDENNAGEGEDVQMSEGVSADTNVKQDSNTPA

PAGSGGDLLDVPEGPPAESTGGADEEMGDTAEESKADGEGENGAANAETQSTKSKEALES

AAREHLISQTHAIVLPSYSTWFDMNSIHDVERKAMAEFFNSRNRSKTPAVYKDYRDFMIN

TYRLNPVEYLTVTACRRNLAGDVCAIMRVHAFLEQWGLINYQVDAEARPSHVGPPFTGHF

KIICDTPRGLQPWQPSADPVVLEGKKNADTDAKAAAATGGKSEMNLEIGRNIYEANTRGT

PINKAEAKVNGDTPSTNGVSGAEAASKTSITKVNCHQCGNDCTRIYYHNAQTDSTTKSSY

DVCPNCYAEGRLPANHSSSAYSKIENPAYTASPDRDAPWSDAEILRLLEGLERFDEDWGQ

IAEHVGTRTREECVLQFLQLDIEEKYLDSEAAINAPTGLSMLGSQHGQLPFTQADNPVMS

VVGFLASLADPVSTAAAASKSAEELKKNLRKQLESGSEGSFQVNGKDKEKPESMEVETTT

TTTTTTTTTTTKTSLATIPLASMGARAAGFASHEEREMTRLVSAAANITLEKLELKLKYF

NEMEAILRAERRELERGRQQLFLDRLAFKQRVRNVQEGLKAAAAVGGEQGARLAHEIMDD

GERLGFQPATGIPAVQPLSAEGQIKSFEA

>SoG_00376.T1

MYASARARALSSAVSRVRITPKVGPPARCGFAPLLRHTRDQQADMRKQPLTMAPARRSQS

SLPAGYVEDKSKGPMLRFQESLPRLPVPALEETAKRYLKSLHPLLSESEFEHSKKAVADF

IKPGGVGSKLQEKLVAKREDPNTKNWIYDWWNDAAYLSYRDPVVPYVSYFYSYRDDRRRR

DPSKRAAAITTAALEFKAQVDAGTLEPEYMKKLPICMDSYKWMFNASRVAAKPADHPVKY

APESHKHIIAIRKNQFYRIEYEVNGQQLTTRELEQQFRKVYEQAKSVPSVGILTSENRDI

WTDARETLLKAGPENRKALETIESSAFVVCLDDASPVTLEERAHQYWHGDGANRWFDKPL

QFIVNDNGTSGFMGEHSMMDGTPTHRLNDYINDLIFGNKLNFDKESTSSPSVEPQLVEFK

ITPEIQSEIDRATKDFNEVIGKHQLAIQAYQGYGKGLIKKFKASPDAFVQMVIQLAYYKM

YGKNRPTYESAATRRFQLGRTETCRTVSDDSVAWCKSMADESLTDEKRIELFRKAISSHV

EYITAASDGKGVDRHLFGLKKLLEPGQEIPAIYKDPAYAYSGSWYLSTSQLSSEFFNGYG

WSQVIDDGFGIAYMINENSLNFNIVSKGLGSDRMSYYLNEAASEMRDLLMPTLEAPKAKL

>SoG_00380.T1

MDSFMLQDEGVRDRIRQAEEFLDPNDPRARSYRSDIILMLQKNQRRLVVSLDHVRNHNSE

LAEGVLRQPFDYSLAFNHALKKIVETIPQARPDQTARETVYYCAWAGSFGLNACNPRTLS

SQHLNYMVSLEGIVTRCSLIRPKVVRSVHFNEAKNKFHFREYQDQTMTNGVTTSSVYPRE

DDEGHPLITEYGHCTYQDHQTISIQEMPERAPPGQLPRGVDVILDDDLVDSVKPGDRVQL

VGIYRTLGNRNTNHNSALFKTVLIANNVVLLSSKSGGGVATATITDTDIRNINKVAKKKN

LLELLSQSLAPSIYGHDYIKKAILLMLLGGMEKNLENGTHLRGDINILMVGDPSTAKSQL

LRFVLNTAPLAIATTGRGSSGVGLTAAVTSDKETGERRLEAGAMVMADRGVVCIDEFDKM

SDIDRVAIHEVMEQQTVTIAKAGIHTSLNARCSVVAAANPIFGQYDPHKDPHKNIALPDS

LLSRFDLLFVVTDDIEDARDRQVSEHVLRMHRYRQPGTEEGAPVREQAGQFLGVAAQSQV

DTQQNTEVYEKFDAMLHAGVTVTSGKGSNKKPEILSIPFMKKYIQYAKSRVKPVLTQAAS

DRISEIYVGLRNDEMEGNQRRTSPLTVRTLETIIRLATAHAKSRLSSRVEERDALAAEGI

LRFALFKEVVQDESRKKRRRTGTVDFASSSEEESDDDDEDPREATDGTYRGAANGSSSRP

TRASTRARTAASSTENAEPTEEPEEDETLTGSTPRRSRRTQRSLHGAWQSTSFASSLPSS

QLPTQTQEDSETEQDLVTVAAELAIGDDTPISAERAAVFRTALGQLLSTDLFVDDAASVT

AVMDAVNAKVTSHDGGSFSKSEATKALRKLEEDNHIMLSDGETVYKI

>SoG_00387.T1

MTEVGAADLSRLLHSKRNEYDAIVTSRKRKLRELFAVATHAESLPRDGFTNPDATEPTPS

ESQFLQANDILQDKLFDDATLPKRSEPNVGFFVQSLHNLLFPPADPHPAPTAVATKIDIP

TPSQSVKQPQPIIPNARQVQPAFAGRLTPASSVSSPLSAPVDSKSSSVSPVNGGPHISAA

PKAPNREARPSPPSLAPVAKATDTKTKQPPQVDFPHGTKGPTSTAPIPKVPVPVNGGRNG

GPNSGPNGSPAPVRGAAEHVKASDAVSSPGSTAASAATPHIRTASTETSPDKDVPPKVEP

VQEKPRPAPPGIAGSSTTQDTEESQDASSMAVDSAEQQLLQESRLSAQAATPRAPSGEPA

KSPAAAHPTTKTPAPTPAPSSTSTTKISEIPDSQEELPDKMEIDNAPSAMETQAPAQPPQ

DSEPSVQATPTASQLPQSPPKETVQASERAVTRVSSGAMRLKSVNEIVGAAPKPTTPAER

PTTAEPENQLTPLTSTSHSPSIKFRHGRPQSRGQVSAVLFGKQPKRSEEKTLAPDEKEVI

KPEDDYFTPLFVQGFTNSSSWMQPIEKLLYQANKTIATPDALLALQDHQACKVLRRVYHL

QQNDKWSLRQPKRCPEPTRQATHWDVLLKEVKWMRTDFREERKWKMALARNLAYACAEWH

AAPAEVRKAMQVSAHIPPKAEPATDVSMTDADEDAPENQPTPDLISSADSPQNIEELSDD

FAETVAPSAIFALQEDDLVFGLRRSPTADRLLDELPIFSSPLQVPKVDPLNPDFDPDALW

RRPALPLSKYVEGQMKVVTPVPPRKRSRYDYEDESSDDETEMVFGSDDRAHHVRVPPASD

AVALFKTEMKHTRDRLHAGHQFRPPSEHPMPVQSFYECRSPSQWTQSEDDELRSLVREYT

YNWPLISGMLSSRSQFTSGAERRTPWECFERWINLEGLPADMSKTQYFKAYNSRIEAAQR

VIMQQNQLAAQQASASGNNSTPLRRRPSTPLRVERRRNQKHLTLIDAMRKLAKKRETQQQ

KQQQTAAQSAANKKPIDTTNQRLPTKTPRDYSLLRWERDQALAERMAQYAAKQDAQRRAA

LQAARGQGQVVQAAAGQAQAAQGGVPAAQQINHAGAVNGVNGVPRGNITSHVAAAAVAAA

AAQQGRPRLPLQAPVNGMANNGGVQAHMNSGLAAPVAHMNGQPQMQAALQVQQQQLSQQQ

QQQQQQQQQQRVQMPTGQAELLLRAQRMAEQQRNAIQMQQHHPQQLPQHQQHLQQQQQAQ

AQQNNVGTPGSQGTQGSPPIRNGVANMNQQSFMNNAQNILAQYGTSGGHGAPKAGLHMPS

VAAGSPGQAQVRPQPQFPNNISAQLAQLEANFLAKNPSLTPEQTRQLATEHLTRAMMQQR

QSAMNAAAGGAAAAAALANAQPGIANGMTATTSPHQYAALLRQQQQQQANQAAQSASPVP

QAQQIAHQRQSSEGSTPSVPK

>SoG_00415.T1

MAASDDLINFDLIEGQKENIQSLPGGRSAKKLAELYSPSPLHKLATPTPTDTKNINDCIR

AEYEAEIAAISESDDPLDVYDRYVRWTLDAYPSAQATPQSQLHTLLERATKAFISSAQYK

NDPRYLKLWMHYIHFFSDSPRETYMFLSRHSIGETLALFYEEYSAWLESAGRWSQAEEVY

KLGIEREARPVARLLRKFKEFDERRAQLSVEPSEPSSPALPTVRPALAAKVDPFGAGARV

ADPQAQRQGSSSGAPRPAKSKLAIFSDADAKAPALSSKETGSKGWDTIGSLSDRRKENTV

EAKPWVGETLKAGGKKTAAAKMAVFRDPNIVVVPSKNQVTMHPQTGKRERIFVNLAAVYP

TPEEAGTELSFEEIIAANRGWLDYAWEEDLVDSSIIPDPTEEVGRDVMKKLVIHHDPPAF

YDENGAVQELPPQPKGGRKKKVMEVNETQIIKAKLDSPSGPKLRKKNTSEPTMTIHTKAA

TDDIYDIFNAPLKSKEQQDESGDDDYMTDGDYTTDGESTGTTRQIEESEQGGDEDEISDV

KSVSEWSDFSTRRHVPNVNSPEAGAEDVEQDDGEFSDLMDPQQLPQSQKSILRDEQAGEE

GEEEEEGEEGEEEDWIPDHEDLVEPASDSAPRTRTIYIPVPPEDYEPPTRPYRDPAEVAN

NRLPFMTPITERTEISLEVDVDNEAPLKTPSRYDGRAVMRPSSEYEPLSSPIREIVQEDL

PAPRIPDSLVPKMPANERKAFASAKPKPHKGPIIRETLCNPVDEVVRSEILEKMHPPLTS

YGGFYDHRDTKYERGGEIRKYIKALTRTKGGSDRTSGIPEPAVIELPGVSSSYTVRRELG

AGAFAPVYLVENSKPDGDENDEDAVVAMGKGAFAVHHRSRMEALKMEAPPTPWEFYMMRL

AHTRLGPHHRASASLSYAREMHLYQDEAFLFLPYHPHGTLLDVVNFFRAEPSGVMDEQLA

MFFTIELLRTVEALHSKNLLHGDLKPDNCLLRLDSLAEEEHVLTSQWNADGTGGWSSRGV

TLIDFGRGIDMMAFMPNVEFIADWKTTAQDCAEMREGRPWTWQIDYHGLAGTIHCLLFGK

YIETTRADAGGALRGGRRYRIRENLKRYWQTELWAGCFDLLLNPAAHAADRESSGKMPLL

KGLKEVREGMETWLEQNCERGVGLKALMGRIEGFVKSRR

>SoG_00420.T1

MIATTIHQQASSSALRALPLHRPSSAALHFANPASFTRLSCAASLRNPPARLFSTTQPAQ

IRDFFPQKETKHIRKTPPVWPHHGHSMEEMLAVTPAHRPPRGLGDWAAWKIVRIARWNMD

FFTGMSRKQASDPKNPTTAIVADEPFTESQWASTFPTFKFIRFIFLESIAGVPGMVAGML

RHLHSLRRLKRDNGWIETLLEESFNERMHLLIFMKMCEPGWFMKTMILGAQGVFFNSLFI

AYLLSPKIVHRFVGYLEEEAVHTYTRAIAEIEAGHHPKWTDPKFEIPDIAVQYFNMPEGN

RTMKDLILYIRADEATHRGVNHTLGNLDSKEDPNPFVSDFKDMDRPKAALKPQGYERSEV

I

>SoG_00422.T1

MDQQGAAGQRGRSASHGQHHANNINHSHTPPPSASFSAQDSSVGLGLGLDQNAHQNLNAF

GANAYLSPQQQQQQQQAYSQNNISDPAVFDPNASFGQTTAGIVDATLSFDGSQSHTAFLS

QELNDADFSLFPTGGQSDNFDTPLFEQSSTLNPNDINTMASQHHQSPTPPHLLQPDAPQP

GSAHQSPSFNQHQFSSPPGTHSRNVSLGPEAALLPNQMGDWNQPQFQGHRRTPSEYSDVS

SVGHSPHIVSSDTFDEQTGHSPLQRASDGSMYQDVLQIGQFSLSDAQGSPGLHGRSPSHS

PAISPRILPQQMPDMNQHQGFGLATHHPGFPSTTYGGMAMGNESYSNQSLGGASDVSQMA

APAINVTLAEPNNLKAGTFNQIKGELDQDSLTPPDRGRPRSRPRSVTDPFAPGSGLANRS

NNSSLSPMGASLSPRSDTSRSLSPSSGISPSRRRQSTSAVPNNVIALRLADPEYQNSQEA

GSTKRVQKHPATFQCTLCPKRFTRAYNLRSHLRTHTDERPFVCTVCGKAFARQHDRKRHE

SLHSGEKKFVCKGDLKTGGTWGCGRRFARADALGRHFRSEAGRVCIKPLLDEEMLDRQRQ

WHEQRMQQNMQNLSPQPGMGMDNGSSYPMDAGGNQWLPAALLAQYPALAQVNWSAPEMTS

GIEDELSGRSSFDASDFDDGDDGGYVSGPGTGYGEGGLGQNFGEIGYASDFGGR

>SoG_00425.T1

MSMRATKFVQTPGIMESANNYDAPLPLPARFAHIKSDLIRGKHAKVQASWFRLLQALRDE

ISEIAATGSSIIPSIDCRDMSNPALMGEFSNQLRRRGAAVIRAVVSPEEASEWNIDAKAY

LADTTSSAGSPTSRDHHLQGVYWSPAQIKGRVHPSVLATQRQLMSSLWHNSRAINNREDP

TFNTRSPPSDSTLLSTNYPVSYADRLRIRIPGDPSFYFNAHVDGGSVERWEPDGYGLADT

YARIWEGNWEQYDPWDSGTRLKVTSDLYNGTGTCSTFRMFQGWLSLSTLTPDDGTLLLCP

MIKLSTAYFLLRPFFSPIRDCPSHKDYLLPCNWKLDDPPTSIIHGARPSYTQELNDLLHP

HLGLSQSLIQVPQLNPGDYVVWHCDAVYAIDRHQRDEVDGATVMYIPACPLTQTNALYLA

RQRKAFLLGQPSPDFGSGRGESTHLSRPGVQEVHAAGGEDGLKAMGLMPWEEDEALTEGE

AEVVEMANRILFPDRYEMP

>SoG_00453.T1

MFRDESTKVMQKARAQWGADDEGSSSATAATSALAPMPSSTVGSPTSAMTRLLSPAAARL

PPIPVNSGFTFLQSPPTISPSITPNLEEQGTSFYMNRYLIGHPDEPRSVEELAEIPWIWS

PILRDGIVAVGLAGLSNLRRDTELMTIARARYGQALRQAGALLTSNATPSHEAMRLIVTL

AMFELVKGAFQNEGTRATTHVLGGTAMIRSWIPTRGPNAALSTPLYKGLITPNPSYRGVR

PLIQMAFTMFIPSYMSGTHVPDTLHDILKFCAQVGSAMDLPSLELGRLMAEWVEISAFVK

TKVLSDGRPAAAPTLKRMMDLDNGLVAWAESLPELWRYTVAHGEGLPSDAIFEGEWHVYH

DMWTARMWAHYRWTRLLLQEAILNLIDSCPTSSLPLVSVSEHQERFETIHNLSRDTLVST

PVFWRHPSLVGLGDKQPIMVEKIGGGGSGAAGIPVTIFQIQAAGCAPGVPISYWEWTTSI

LECIFGDMGMVHAKSMRESMILHRERMQKYTTESMLAPGSSMG

>SoG_00456.T1

MTALSSSSSPSTILRALLQPYITTRSLRTSKLVAETIPSTPIKVLRTVPSLRTWRRPHVL

NHRSVALVPTMGALHAGHLSLIRAAARENHHVVVSIYVNPAQFGRHEDLDSYPVTWEADS

KILANLDRELADDGGNLGRVSVVFAPTTKDMYPGGFPGQDVDSKGSFVNITPVAEVLEGK

TRPTFFRGVATVCMKLFNIVQPERVYFGQKDVQQTVIIKRLVEDFMLPIDVVVCPTEREP

EGLALSSRNVYLGTRRRRVGLVLSQALRAAESKFQSGELSRRAILGVAHEVVDRTLAEQM

ALGPEARALFEVDYISLADPQTMIDIEEVDPAKGAVLSGAVKMLQIEKAAEGEDVGYSGG

PPVRLIDNIVLKPTPP

>SoG_00467.T1

MASAFVPGLRRLALRSIPTRLPTTSCSQCMRQTTPRISNVLRVVRSQRAAYSTTTQAAAA

AVDAQKIADRVVANAARASRDFKKTTTRKSAWPETNSKTVGYWLLGSAVSVFGIVVFGGL

TRLTESGLSITEWRPVTGSLPPMSAADWDSEFEKYRASPEFKLLNPHMTLGEFKKIYFME

WTHRIWGRVIGVTFVLPTLYFIARRRVTRKMAANLLGISALIGFQGFIGWWMVKSGLKDD

LFAPGSHPRVSQYRLTAHLSTAFVCYSWMLLSALSVFRSHRFLASPQAAIDAVKSIRNPA

LRIFRRSVFGVSALVFLTAMSGGLVAGLDAGLIYNEFPKMGLGFAPPSSELWDEFYSRKE

DRSDLWWRNMLENPSTVQLDHRILAMTTFTAILSLFAYSRTGRVAAALPRDARKGAMGLV

HLVVMQAALGISTLIYLVPIPLAAAHQAGALAVLSGALVLAHRMHVPAPTVRLIQRRLQQ

LKQ

>SoG_00484.T1

MGDLQGRKVFKVFNQDFIVDERYTVTKELGQGAYGIVCAAVNNATNEGVAIKKITNVFSK

KILAKRALREIKLLQHFRGHRNITCLYDMDIPRPDTFNETYLYEELMECDLAAIIRSGQP

LTDAHFQSFIYQILCGLKYIHSANVLHRDLKPGNLLVNADCELKIADFGLARGFSIDPDE

NAGYMTEYVATRWYRAPEIMLSFQSYTKAIDVWSVGCILAELLGGRPFFKGRDYVDQLNQ

ILHILGTPNEETLARIGSPRAQEYVRNLPHMPKKTFPSLFPNANPDALDLLDKMLAFDPS

SRISVEEALEHPYLQIWHDASDEPVCPTTFDFAFEVLDDVGDMRRTILDEVMRFRQTVRT

VPGGGVGGAQGQQAQAGQVPMPQGGGQWTAEDPRPQEYIGQHGGLEQDLAGGLDAARR

>SoG_00495.T1

MTKSDFDHRASSHTNASTDSGHSSVTVKPSSTRRPAHPPARINTSRKRRSNSRSEESDSH

VSPTTSIAATSGVQTDPNLLRDCNMSELGNHRRQLSVLDTDRVPRIKQQPPTGGPSPQIA

PWMSSSNSSSNQPMQTSFYNDSTESLPSTLAGQSLPSPGHLRTGSRSGFVLVDKDANGTP

SFTDDRRPSVASIATTASSTGSKTSGTRGGLRKLQGFFGEEFPGRDGSDGSLPTSLASKD

HRGRSYSHSRSHRGRQFSNATDPTREASPSPARPRTPAPEVVPFLYQDNTDILRYGEAPV

REELTGPDKERYLVDNPPQVPPKTSSSSRSGHSGVHLPGHHHRHNKSNDDPRALRPSVSR

EDSAHAQYAKDKSGTSPGMFYTRSRGQSPTPSASSAPSHTLKHAQTDGAAAAHGKRGLLG

RLRRHHKDRDGPDSASKLRDLPPSSRSLQPKKSKTDYGPELSPSAFPSSTADLSDTLIQD

GRPGAQQRGATFNNKFPFSKKGRGPRPLDFGDDAIGPTDRYDKHMYHLDTNLNDMEGILT

KPPPLTPMDTSFVNSIVPDRVPEPVAATTTATVATAAATAAAAAPDPAPKSRWDAPDSWA

VRRNTEENADIAPDIDDIGSPPRPEEKKTPYCIRIFRSDGTFSTHSMPLDSSVTDVISQV

IKKTYVVDGLENYHIILKKHDLIRVLSPPERPLLMQKRLLQQVGYEEKDRIEDVGREDNS

YLCRFMFLSARESDFHSKTADLGVKPGQKLSHVDLTGRNLITIPISLYKPAPGLVGLNLS

RNLSLDVPRDFMQSCKSLREVKFRNNEARRLPASLSRATKLEILDVANNRLEQLEHAELH

NLTHLRQLVLANNRLTHLPPHFGAYRSLRSLNLSSNFLELFPPFLCGLTSLVDLDLSFNT

IANLPAEIGNLKNLEKFLITNNRLAQYVPSTFRNLTNLRELDIKFNGITTIDTIAELPKL

EVLYADHNSISSFVGKFESLRQLKLNSNPLNKFEITEPVPTLKILNLSNAQLASIDTAFA

NMTSLEHLNLNKNYFVSLPQQIGTMSQLEHFSIAHNSVGELPPQIGCLQELRFLDVRGNN

ISKLPMEIWWASKLETFNASSNVLENFPRPASRPPRVPGEDLPGPPPAQNGRSMTLGTLS

STPSSEELTDERRPSQASSTLLSVGPSPISEADRKGSVVSVYGKGGRKTSVVSRTTTQNS

VASSKNAPPPPATRKDSSVAAKYSNTFAASLRNLYLADNRLDDEVFEQLTLCTELRVLNV

SYNDEIGDVPQRSMKSWPQLVELYLSGNSLTNIPADDLDESSLLQTLYINGNKFTNLPAD

ISRARQLAVLDCGSNQLKYNISNVPYDWNWNLNPRLRYLNLSGNKRLEIKQQGWQGPGPG

SQQKEQYTDFSRLLNLRILGLMDLTLTQPSIPDQSEDRRVRTSGSLAGHLPYGMADTLGK

NEHLSTIDLVVPKFNSSETEMLLGLFDGQALSSGGSKIAKYLHENFGFHFANELRSLKSR

QNETPGDALRRAFLALNKDLVTIAIQHQEERPKTVHKGSAQPVILNKEDLNSGGVATVVY

LQGTELYVANVGDVQAMVIQNDGEHKILTRRHDPAEPMERSRIREAGGWVSRNGKLNDLL

DVSRAFGYVDLMPVVQAAPHVSNMTIREQDDIILMATKELWEYLSPGLVADIAKAERQDL

MVAAQKLRDYAMAYGATNKIMVMMISVADLKRRVERSRMHRGTSMTLYPSGVPDEAQVLP

IRKRGKGRAEILDSSLNRLDPEIPAPIGNVSIVFTDIKSSTNLWEMHESAMRSAIKLHNE

VMRRQLRRIGGYEVKTEGDAFMVSFPTATSALLWCFAVQLSLLEVSWPSEVLNSINGQPV

FDKDNNVIFKGLSVRMGIHYGDCVSETDPVTRRMDYFGPMVNKASRISAVADGGQIAVSS

DFISEIQRCLESYQDTERNGSQGSEDAFEEDSYASAIRKDLRSLTSQGFEVKEMGEKKLK

GLENPEIVYSLYPHALAGRIEHHQQQERRDDTVDRPAVLGGNSELSFDPNLIWSLWKVSL

RLEMLCSTLEEVRGPGLQPPETELLERMKTRAGEVTEGFLVNFMEHQVSRIETCISTLAM

RHMAMGSGILRELNDLRAPMSSVLDHFVKEQKKLAKYRARYGDISSDESSDSEAEDRDQG

RITEIPNNSDEGSDTAGDVFYILGLTFSHAPHDPDLWHHPGPKPPPTCNPTCEVPHSVNQ

PRQAKDGSIIRFGCCRRLLARPQQFQTGEAVDDANDLPLAESLGRIFHERVVEFAPDPGP

GETDLVFCPVEEEDRLGTAGSGLGASDVGGVEEGQGEAVRVLPEEGEPGHAGEDVGVDAD

DAGFSGGDVVELGEGVAAGVVERGEEEHGGARCRGEEDGHCERALQEGGEERLGGADGDV

VRAEGRNGGEGRDDVEDEASLFVRLEGVDDEEEDGRREEQACLGGCSGVDEGVDDEGPER

ALPEVGVEGDGEEEGGEEAAGEGREEDPEGERRRPGRVDELDGEDGGEEKDR

>SoG_00503.T1

MSRKTGDMTPNEVAQHEDQIQYGSVGLSREELDKEELFNPLNENKKQPSGGQQRKRGPRG

ANKLSPQEQRRYIIERFIGRWRKEVGNDFYPALRLILPDKDRDRGVYGLKENTIGKLLVK

LMKIDKNSEDGYNLLHWKLPGQTSASRLAGDFAGRCFEVISKRPILTEVGNMSIAEVNEQ

LDKLAASATESDNLRIFEVFYNNMNAEEMMWLIRIILKQMKVGATERTLLGLWHPDGEAL

FNVSSSLRRVCWELYSPELRLESDEVGINVMQCFQPQLAQFQMPASFQKMIDLLRPTEED

PEYWIEEKLDGERMQMHMVEDPSHPGGKRFCFWSRKAKDYTYLYGNGLRDENSSLTRHMK

TAFAKGVRNIILDGEMITWDMDVDKIVPFGTLKTAALAEQQNKSGSNATGHRPLFRVFDI

LFLNDKQLTQYTLRDRRNALEKTVKPVHRRLEIHSYTPATSADAIEPMLREVVANASEGL

VLKNPRSMYRLNSRNDDWLKVKPEYMSEFGESLDCVIIGGYYGSGKRGGQLSSFLCGLRA

TQNHIRAGANPEKCFSFFKVGGGFRAEDYAEIRHRTEGKWIEWDQKNPPSEYIELGGGEA

RQHERPDVWIRPKDSIVISAKAASVGPSDQFAKGFTLRFPRFRRLRLDRAWDTALSLEEF

VELKKRVEEEARDKAMTVEDRKRRAPKRIKRELVIAGADAAPVAFEGPKTKVFAGLEFCV

LSDSGKPFKKTKTQLESIIKENGGAVSQRAVPGSDMVLIADKKVVKVASLIKGGDVDIIR

PRWIRDCTEQNFDAGTPLPYEESHIFHATDALTRAASLNTDQYGDSYARDVPVDELREIL

QGMPKIELEDVFDKAQFVEELEARGKGLSELRNWMFSRLVVCLRPATTSDERKAGRLAAL

VRYSGGRCVDDREDQGITHVVVVGDDAMETGEVADKIRREISGRRRMPRVVSGKWVEDCW

SEKTILDEENYIVQ

>SoG_00531.T1

MSSTWMNDAVTNHNGNSFPHMNDPNAAATGAMMDPSAFMNSPAQFNPAQFANQQPPQQGQ

PQQQQMAGAQNGPMRNASPSYQNPVYQTNSVIPSKRPRPREDSLAGSPRQNPGMLPTSRS

ETPQQHNFPGFQPGTVQQHNPNQFPHLQANGSANASPSPIMGNQMRPGSVPQRVATASPH

PFSPSAQQFGAQTSPIPSEQGTPQPYMQTMPPGYNASFAPSPSGPRPAANPNGMPGSQMT

PQQLAQMQQQMGHMPNAMYAQVQQQQQQQQQQQQQQQQQQQLPSQQTGQQAQPQPGQTRQ

QQMTDQQRMAAYQMQLQQRLQGNMQGSPHMQGQTMGRGVMNKPQMAGLPNGQTPQGGMRP

QQSAQQQQRPQQGMNPESFMKNLVNLMNAKGLPLDPNPMVGDRPVNLMVLFQAVSSKGGY

KLATGGQAWGPIAQMLGLPAHIPTVPQNLKQVYERNLLKFEEVWTAQQKQRMMQMQQQHQ

QQQQQQQQQQQQQQQNQQHLPPSHPQPQQQHPHQQQQHTPNMTHPGTPQRPMPPQQPGHG

QGQMQPGLPAQGQQTPSKPGQPVINGFSTPQMQHQQPPHTPQQVQQPHPQAMAGHSRNSL

SRSQEPSTGNDYAVQSPAHVKTGSMSLPGGELRPQSLNGPGSEQVLPRPTPKSDDYIPCT

REISTWGGVDTVAASTIGQQLETWRPDVPHANELGHIDILALMRSLQCGIHGEVRLALDT

LASVSISPNPNHAVQLAYCEELVEVITELAEEQLDVLTEHTVEVSDEIQLNSYEDLLRFC

HQEKLAVREEPVFGSKEYVLDRAVDRLVCITAILRNISFPGEGHPNHNFLAEESVIKLYC

SIIRYLGTRTMLLRTHANTLDFMKDLVVLLSNISGCIELASREHALCLLHFLLAFAPMPG

PSLTTHGMDFPPYDPSINPYLPHAVDALAKLLARDEPNRTHFKTIFALDAANSPPHELLT

RTFGLAVSPIPTKAQEQDRHPQHPPLLEMRKPFLMQGLLAADILVSLAPNYESGLVKTWL

STSDGQFAANLFRMIRELSRQYDAPQVYTRNAPRGQARKDPELVYIVMVALSLLRRLAEK

ARDPSTPGSSIPLDFLPSHDILFEATTLQSPEWTREGLLQQLTSFYNLAT

>SoG_00534.T1

MASVRSMGLHHPSGLQHAIDEEILPPNPSSSLYEWEIYPPRTPDDVEDELLITATCVIWS

RGGTYQTSYSFELEKESITKALLAFFPVSNSQHHARGTQIPGIHEKQLEKTLVVILKTQA

HIYSLTGTSHIVHLPFEVQTACAAPVGVILQRKQRSENLAPLALKFPKVPPESFMSSQLS

FLTSSQQTTFSVETLGNPKSMHLGQSLTIENTWDRPVEGQDSHWPRLVCLVDPLSEIGLV

VTDKNSPGGAMQKPKAQIKTQFLDPAEEVLHIEDVSVTGSPESVTLAITANSQSNSYTIW

RLTYLDQEDPFLGQSKKSKERATRRRSSMAPNAAAAHLATPVQPGYSRDSFGVPMPGKRA

RKSEKVEKSDHALDLVTSLEQQDHETGVTRRSSRRVSSMLARADLSASHERTSFTEQPLG

STFGHASRRSDSYGGRQARLSSSQAQQIHPSLGSLLEAPFDVGLDEGFHNMGLNDRDLDG

LQREIHMSKIHTFERDCSNARYSFTNQARSNQPKVFILQAPPFATNKYYRGQLLVGIQDA

SEKRLQLVVLYLRAKAQADSAQKQGQTPGESNGVVVIPGELRNAQNVVDSCKLVDGDQSA

ILILSESMSGQHELSTQAPWGELTKVSLSLLFVDDERHLQFRGRKIDRDIKQRKSEVIDL

ADGSIVGVRHPRQNGVVDVLDASGRLHQLRLQLQPTSPQVRRVLGACKSILPDSLGERIH

AGWLHVLQWLQHQDNEHTDLEWSAVTTLLLAMTLNLGRVVGKPLHVAKVPVRRRRPASGS

FSSIKDSEDWKSMERGEAANALGYPPWMMNGAWDWALDDEAEDRYRSTSDLLVPAKYITR

HVAWAREFAASAAGESALGSSGYLPTALGKPLEARIKGLSDLIMGLHLLLEEQKLNIMTP

EYTSPGRADLRAVLCQISRWLKWQDFLTIYEVGMQEELDPRNDSELVLKPAIPQPSRHPP

DILSWIQMRLTGSRKEHFLTARDIFYASARLPETDRSLDDRWDAVFPRTFMFKRFFKLVR

SHTTAVQMVEIMRDCGITPPVLETLPEALVVPLRDAISLCQPRPPTGWSTELLDLVSRSD

ISLLLKPGQKPRSTASNILAPTHQAGWDYRMLCQSLEETNSLGYDEGEGTERQAVIRALF

KDDRRLNEAQDLLSTHKARIVRLDPQPNWQESEYLEKQKELVSRVATGTLAIPAGRGLLY

YGLRYPLLTQKFHIGGFNLNCVVKPANVTVGVDKSLFTEEKVNWGFFHQGVAAGLAISPK

AQGIDTSWILYNKPGALPNSNELSNRHAGFLLALGLNGHLKDVAKWVAFKYLTPKHTMTS

VGLLLGLAASYMGTMDSLITRLLSVHATRMLPRGAAELNLNAMTQTSGIMGIGLLYANSQ

HRRMSEIMLSEIEHIEEEDEEDPLRSECYRLAAGFALGFINLGKGSDLKGLHDMRVTEKL

VAHATATKNVDIVHVLDRAAAGAVMAIALIFMKSEDQIVARKIDVPESVLQFDYVRPDIL

LLRTVAKNLIMWSRIEPTTAWIQKSLPAPYRPRYKLQETTRLRSTDLPFFSILAGICFSI

ALRFSGSASPKVRDLLLHYLDQFMRISRIPSTSRGVANAAPPYDEELARTNARMCQDILA

LSVSIVMAGTGDIPVLRRLRALHGRDDPDVPYGSHLAAHLAIGSLFLGCGTVTFGTCNQA

IAALLVSFYPVFPTSVGDNRSHLQAFRHFWVLATEQRCLVAKDVIDGQPVSVSVQVNMRK

GASAEPVLYRTTPCLLPPLDQIASVSTVCGPQFWDVNIDLTNAEVREAFTENQSIYMRRR

PPRASPFSSTLGALGSDSQGSRNPLRWLFALESLRSVSYAEREAILENGDQDQETGRAVD

ARLEMERGILAGGDRERLEGAKLVFEWEAARERLLRNSSSTQDQGPDDGKDESSVWWLRD

SVIEDLKGKVWLAAREAEH

>SoG_00537.T1

MPILAFVLPFLVAMPSSISVPILNAVGFSTAGVAAGSAAAALQAGIGNVVAGSAFSVAQA

IAALAI

>SoG_00555.T1

MSQTEFNILVLPGDHCGPEVMDQALRILDVVEECRPGVKFNRTFDLVGGSSIDKHGVPAT

DEMLQKAADSDAVLFGSVGGPEWASANPNPEAGILALRHRLNAFANLRPCEFLAPSLVGA

SSIKPDVVRGTKFLVVRENCGGAYFGHKVEEEDVASDLWVYTRSEIERSSRVSGAIARIM

GQNGDGTGGPATVWSADKANVLASGRLWRRVTEEVLAREFPDVPLKHQLADSMAMLMVKR

PTMFNGVIHTDNTFGDILSDISGGIVGTLGTLPSASIAGVPGEGRCKGIYEPVHGSAPDI

SGQGVVNPVAQILSLAMLLRYSCLMLEEASCIEKAVQKTFESKEAGGLEVRSKDMGGSAG

TVEVGDAIVNELRSLLKK

>SoG_00560.T1

MADIPITPPSRSLKGKTAIVTGAGCAGEGIGNGRAISILLAEDGCNVVCLDMNIDWAGKT

AEMVNAKPGRGTAVAAQGNVTKAEDCEAAVKLAIEKFGRLDILVNNVGVSGASGTAVEVD

MEQWSKSLEINISSMVLMAKFAVPAMEKNAGEVKGAIVNMGSVAGLKGGTPHLLYPTSKG

AVVNMTRAMAAHHAAQGIRVNCVCPGMLYTPMMYAGGMTEEARAARKARSLLGTEGNGWD

AACAAVFLCSDHARWITGVILPVDAGTTAAVGIGMPKSASVNGSA

>SoG_00568.T1

MQSTGRTNQHHFSVQDLNGDERDEEKPWNFQELPRWKRSKRCPSLPRVPCADNETEEVER

GRTKALPALSLPKNRSTGNLALARSPDPSTATAADKSRYRMSFDFSQAANIDPESAIHRS

PLMADHEHGLGLSGLRRIRQHHPPSRAPTLPNSTASSRSPSVVALSRSTSMSAMLAANSS

FPLSTGPSSPSFTEDLSRFPSESLHSFSFAHQSEDFLHNRENVLKRSIEYMKDRKGWSVS

SMQAGLASAQARATGDIEAQHMLELLARAQLIEAGNLPNAESSLANPGPLTGPAEVSGEN

IFEKHFIPRTSSPEPLPATSNSTATQPPSKSQRSQLEGSTPRAKAEQIPKHKEVLASEDS

SRTTTGESTATADTSPPASRPTSLKRTMTDTHGITMQTKLMDTLAQPYVLGQQPIPESIA

SPTFQSFTPTAPTFPAALGPVAHGHTNRWVPAAQAIFTTEAKPPWTIIAANDLACLVFGV

TKAEVRRMGILEVVQEERRAWLERQLLRNEEDDVSEGGDAKKGTPAASVATALLGGRGGI

TAQLLSKPNSRAQPPKTPPRRAQTVHSGDPSPPKTRGSANHRSNLSRGVLLCGDVVPIQK

RNGATGSASLWVKEKRIGLIWVLEEIHEDVANVALDEEGIVQSISGAAHAIWGIDDRQKG

FDIAKLIPRIPRQGINPKEGDVDFAEIARRRYFTATNSKKTNLPCTVEQVRGKIELKVST

FPHMAGIVVVDPENLQIRSSNTAFCSALFGFEKPDGLHITTLIPDFQKILQTLTQEEGLQ

LMDGMVIPEHRFRRASAFLALKEHRPDAAAAFLQPDGLQAKHRDGSDLKIDVQMRIVKSE

KQPSIPEETVHEEVSESEEDQEGHDLFPIQRSEIVFALWVTYSRHIHSSQGHLGTASPAA

SGTSTPLHQPSPGQTVVNSPMELSSDGEESKNKKKPEPKTTPLSKQLKDAALSAAAKLTG

HSKLAPKTEEAPVAETPSDPHHKKTIDDYTILEDMGQGAYGQVKLARNKRTGKKAVLKYV

TKRRILVDTWTRDRKLGTVPLEIHVLDYLRRPEFRHPNIVEMEGFFEDDVNYYIEMVPHG

LPGMDLFDYIELRANMDESECRSIFVQVAQAIHFLHTKAMVVHRDIKDENVILDGEGNIK

LIDFGSAAYIKNGPFDVFVGTIDYAAPEVLAGKPYGGKEQDVWALGILLYTIIYKENPFY

SIDEIMDRDLRIPFTMSDESIDLIRGMLNRDVKERADINQVIEHPWCKVQV

>SoG_00588.T1

MATVSHLPNGRVVPMRVIVAGVHRTGTLTQANGDVDGPQWSRAFAAKYSGKGSFTRADWD

KLLGHFQATCDVPAAFFSAELADAYPDAKVVILNRDPEAWYDSVLNSIYKYFFSSSLWGK

MNLAYRLAFDPTTRGAVQMSSWFGLAMPYNHGTQKDKAIAWFKAQYDEFRERIPEERRME

YSIKDGWEPLCQFLEVPVPTVRDEKTGEVAVAPFPHLNDREVFAANAAFMKTSGSKRAND

NFFKAVGKATVFGAAGFAVYWAWKSRLGGRV

>SoG_00604.T1

MAVLSTLPSIRTLAVASIAFFVIYRITSRLSLAARRRALKRRTGCQPPVDLDALPSRAAV

ARRYWPFDWLLGLNIGNFVYVGELARQHRFYPARMEIWAAAAPSKTCRTKLANLEWTLTQ

DVENVKYVMATDFDNWAFVPGREHGLGQFLGKGIFTTDGADWAHSRNLLKPNFTRFQVSN

MALFERHLQELLAVIPLDGSTVNLGPRFFCMTMDIATEFLFGASSDTQRRGENKEFSDAF

GYCQDHGLALLRLGKLGSWLPHPRKFRRARKLVYDFVDGYVDAALEEKRSLGDLKQDASE

KGRYVVLHELVQQTSDRVQLRSESLAILLAGRDTTASTLTTLWFILALYPEVWRKLQAEV

ATLDGRHPTFEQLKELKYLQAVLNEVLRLWAPVSLNSRICLRDSVLPRGGGPDGSAPLFM

EKGAQLSWTLYSYHRQRDVWGEDAEEFRPERWLGEGAMRFGWHYTPFNAGPRICLGQQFA

LSQVGYVTVRLCQEFDGLELRGELREWQENHSLIISHSEGATVSLRKRE

>SoG_00644.T1

MLPTVDLGLKLTSTLPEPPHPITPTAFGLQRRLLDHGSRRPSSTYNYHTTSDPLEEVVNP

RPQLHRLSSFPKPRTVNTRDMVMATAAVECYTHHVNNNVLIKKSHLVEPLQNHHHGPIDP

KTLIGEALHRRAEAVDHELCEPGDEDTFFVADLGEVYRQHLRWKRNLPRVKPFYAVKCNP

DPRVLRLLAELGTGFDCASKGEIEQVLGQGTSPDRIIYAQPCKTNSYVRYVKAAGVKQMT

FDNADELYKIAKLYPGAELFLRIMTDDTSSLCRLSMKFGAAMDTTDSLLNTAKELGLNVV

GVSFHVGSGASDPMAFEQAVRDAHAVFQQARTYGFTMRTLDVGGGFSGETFEAMAAVLDS

ALDQFFPAGSGVDIIAEPGRFYVSSAFTIACNVIARRTVEDPTLDATGYMVYVNDGVYGN

FSSIMFDHQNPEARILRSGGQNMYNTVGSGPCPAGEGVEYSIWGPTCDGIDRISESTRFD

RVLDVGDWLYFEHMGAYTKCSATQFNGFSNSHEVIYVCSEPGARALLNL

>SoG_00651.T1

MLHKPRQFATDGFQVIDTSEKVEEERLPMYRQDKYYPVRIGDVMENRYQVVAKLGFGTTS

TVWLSRDLSNHEMEVFDRLRRVKADEPGWKHVRVCEDVFTLQGPDGEHDVLVMNPLGWSL

QRFQNFQPTRAFEGLFVSEAIQQVLLGLMLLHEADIVHSDPHADNVMIGIDDRSVLSRVE

EKELNEPSPRKLVEDRAIYLSRNFRSGPGPVTISDFGQARLGSAHRGIAMPVQYRAPEVI

LNMTWGKSIDAWDLLENKPLFRIYDQKSQDENDAQHLAAMTALLGPPPPEFLKRSIDTQK

YWTEDGQWRGPADLPPETDFESLITNLEGKDRDKFAHFMACCLAWETDDRLTSLEAWFHP

WLRGGELPELTQEHGT

>SoG_00677.T1

MPRAEAGSVKDIANRQKLKGLQRLRFYCQVCEKQCRDANGFKMHTQSESHVRKLLLVGED

PKKFLEQYSKQFLSDFLQLLRTGHGEKKIGINRFYQEYIANKEHVHMNSTKWSSLTEFAK

YLGREGICRVEETEKDGLTISWIDNSPEALRRQEALKRKEAQDQGDDELEQRLIREQIKR

AQASAESRKKDVDEDPEDRELKRQDGERIKLSFGAKPAAKPSETASPSPASGGESGADKT

ASVDSTVKESDATPALAKPSGFGGGGISMKLGTKPPQTKNVFAQAKKNALSSGGAKKPSA

FGEPKKMSEAERIMKEEMERKKRPAAGFGMPGGKKQKNY

>SoG_00708.T1

MARGYSPPDSPLSSVVESENYDEEVHDDEDGESRPSKRQRLEAGSTTSSAVIPDVEPEHP

EAPQLDGMSDLSSDTSGDIPSSPVNARLDEEDFQDQVTVCDWDGCPAGDQGDMDKLVEHI

HNSHIENRQKKYTCEWRTCNRKGLPHASGYALKAHMRSHTREKPFYCYLPGMSTNVKRLS

TKSNCISNKPFLTECDRSFTRSDALAKHMRTVHETEALRPSDPVPKSQQPTGKSGKLKII

IKTPNSHNGGEDGTDGSMNGEEANAEYFTALTSELFSAKELALPVDELYRKCYWESKWSG

EIGEALKKECKKWEEQYRQEWLEKEVLLGQVVQSEVDWHERRQAILSGVADVNVSAAAAA

AAVTGKPEEAGEQANGVAKEETTVKEEKASEVAPEAVGAAA

>SoG_00751.T1

MAQHGFNQFGSGHNNGSIDPNDLAMGNGYSSSFANNNFNNNSGNNAFSSGSAVFGDDELL

DGLASPGDMTGMQGQDFSGMNAGFSQNTYTNHRNNSGLQIDPSQMNGYSNTPDGDPIHSP

YNASLNAQFRNLHTGSLGTSLHSPLSYSGSPLAGGDMNVDGQDPNYLNAKARARMSQQMQ

RKGSSNRSPMTPKSNLHGVPIGSQESPGFGPQTIRNAGHDKSPSGQWIGTPGSIPASYNS

GFSSPMQQGLMPMNEVMMKGGTSMPAKLGVQSSGAVSSQEMKRKRRRESHNLVERRRRDN

INERIQDLSKLVPTHRLEDEKIRKLIQNGTPLSPTLTGLQSPSQATSGLAGPGARRAAGA

TAGSITTGLPIEDKDKGPNKGDILNGAVSWTRDLMWMLHLKLQQQEELMNQLTELGGHFP

FEFTEDERRMQSELLEAMSKLEPDTLSYTRTAGSGLRVPNHTDYKGDPVNGMGGSSGLDT

MGITPEPNGTADLNDPNSFWRDDDHSGRQSLDFKEEDEYDMDLVN

>SoG_00762.T1

MKLDFVIASTNLKANTSLQDLRVGNKYRIGRKIGSGSFGDIYLGTNIISGEEIAIKLESV

KAKHPQLEYEARVYKSLAGGVGIPFVRWFGTECDYNAMVLDLLGPSLEDLFNFCNRKFSL

KTVLLLADQLISRIEYIHAKSFIHRDIKPDNFLMGIGKRGNQVNVIDFGLAKKYRDPKTH

FHIPYRENKNLTGTARYASINTHLGVEQSRRDDMESLGYVMLYFCRGSLPWQGLKAATKK

QKYDRIMEKKMTTPTEVLCRGFPNEFAIYLNYTRSLRFDDKPDYSYLRKIFRDLFVREGF

QYDYVFDWTVYKYQKNAQAIAQAAGQNNGEDDKDGRNASKNATGGQSGSAKPNAIPSSRR

KMLERGAGAGPDTPDTNRAIGGSDRM

>SoG_00766.T1

MSLSKLITLAALAGARLAIAQASGSADAHPGLTTYKCTTSGGCVSQKTSVVIDYQYHWIH

SPSGSQASCTTSSGVDKTLCPDQATCDKNCVIDGTTNYTSSGVTTSGDTLTMYQYVQGAN

GLQNASPRLYLLAADGTNYEQLKLLGQELTFDVDLSTLPCGENGALYLSEMAANGGTKGQ

AAFGAGYCDAQCPVPTWRNGTVNYSNQGFCCNEMDILEGNSRANAFTPHPCNSAGTDCNK

GGCGLNPYSQGQKNYWGPGGTVDTSKKMTVITQFITDNGKTTGTLSQITRKYIQNGREVA

SAIQGGDTITTAQCNQWDPNAATFGSLPTMGQALGRGMTLVFSIWNDASQFMNWLDSGNN

GPCSATEGDPSLIMKNFPNTHVTFSNIRWGDIGSTTGNTGGNNGGSGTTTKKPATSTVPS

TMSTATKPATTTTAKTSSMQAATTTKPSGAQQTHWGQCGGNGYTGPSVCESPYTCKAQNE

WYSQCL

>SoG_00768.T1

MLSLSTLALALTAAAGALSQSAGCNSGGGLQSGRASINLNGQNRDYILRVPDGYDGSTPL

RLVFGFHWLGGSMNEVANGWYGLEGLSQGGAVFVAPNGLDAGWANSGGRDIAFVDALVEH

LTGNLCIDEEQIFATGFSYGGAMSHSVACSRPDVFRGVAVIAGATLSGCDGGRQPVAYLG

IHGVVDSVLPIDAGRQLRDQWLSTNGCQQQNAPEPGAGSGEHVKTTYQCSNAPVTWIAHG

GDHVGDPNNNGNYWAPGETWEFWNNA

>SoG_00773.T1

MSRQNPPNNAQASRKISFNVSEQYDIQDVVGEGAYGVVCSAIHKPSGQKVAIKKITPFDH

SMFCLRTLREMKLLRYFNHENIISILDIQKPRGYDSFNEVYLIQELMETDMHRVIRTQDL

SDDHCQYFIYQTLRALKAMHSANVLHRDLKPSNLLLNANCDLKVCDFGLARSAASQEDNS

GFMTEYVATRWYRAPEIMLTFKEYTKAIDVWSVGCILAEMLSGKPLFPGKDYHHQLTLIL

DVLGTPTMEDYYGIKSRRAREYIRSLPFKKKVPFRTLFPKTSDLALDLLEKLLAFNPVKR

ITVEEALKHPYLEPYHDPDDEPTAPPIPEEFFDFDKHKDNLSKEQLKQLIFQEIMR

>SoG_00789.T1

MSDPPSPQQHHPSPDPDSPNATAHAYASNGLEILQAASDAAQAAAAAINNPQALQSAAAE

AVAQQQLDQQQHHHQDHQQFQQHTQHVGNAHFALAEDLSSAGATTAAMMARDPAINPKLT

RLRRACDMCSMRKVKCDDQNVPCRPCRELGVDCTNDRPTKRRGPPNKHAVAAKAAKRARL

ELEASSTSPGTAFPISPTPQTAAKTLMEISTDAPASSLDAEAIAPIPVLELLIDDFFTYI

HPLAPFPHEPTFRQSFANREDRTRPEFLGLVASMICALVASFPRSAREHLKAQHSTHLFP

RAIVMMERCRDVALATRGARWALKEPKTLDDAATSYFLGLGSGYSHQWNISRHFMSETLT

LIRELGFSRPKHPGELPTFGNDTHSPDPLPFNHVKDQIGTRIFWCLLLGVRSFSQLGPGQ

ADVVIAPSTPSLPYPAYPENVDDICVLANEIIHQPEGTVTLLTGYRFAIDIYTTMNGVVS

LELAYGMSTLPWSDQRLLLRDGLLAAKSVIDNLPPELQLHPSPTSEAANPLAALDDSGMQ

YAPPAWPASQPAHDVRNVIKLHPHRRRHLQYEIQKANIFVSQLATRSYFVEMYFNLRDVY

LAEQAQQRDHDGATNGLDVKSTDPDTDENDPEDKQVLEFMSSERELIVQNLLTVLGSISQ

RNLEPNGASLINKIRQVASTLLNDRPERKGPFAVKSEEALSQLIDVLMKLEKSGPGPGAT

AAVPDDGGSFGQQMTAQDEEEELRNWADLRDYQLRFALHGGFGGNL

>SoG_00793.T1

MDGPHYPSNGIIGSSAPQTYPSPTTLSPHQLQSGSPLPQTLPPLQPSVSAMQQPLYGSHP

HTPRTPGTPNTPNAQAPNGNGNGQNNGPAHQQQPQQQSYQPQTSQPQGQGRPGPYTMAQN

PYAPHQGYATSGPMMPQTTTAASHPQPIAPAPAGGRGPPVLRPMPPAGMPGSMGPLPHAP

GGHMLPTAEWKNSAWMPQPDAPGDAPTHVVGSQGRRGILPSAPGRPAAPAAGTGAGKNTV

IPVKDADGKFPCPHCTKTYLHAKHLKRHLLRHTGDRPYMCVLCRDTFSRSDILKRHFQKC

SIRRGNPTGATHLSHPLAHARRNQNNNVAAQQKPAEGDMNHVNGMNNMPPSDGMVHPFGM

VPVSDGMNNMSNDQNQLSRSGSMGNGAAPDRRMPGPGMGNSQPYPSDVSNSMTSQQMPYS

MPPGQNGMPMYGGSNTNQQSSLDWSQMFQAGAHQTYVNNSPFPPNLGQTQIATKTEPKHE

TDRTTDERNGEPLVYHEWGIPSTLPNAFDDLSHQILNFLYPPDEAVDPHLSGMNLHFSSD

NIKDFLDQYHHFHVHTPLLHIPTFRIMEARTGLWAAMCCIGACYSDRVGSSIVREMMDVT

WSALQRDNKLTSNSSPSFSSDATRSEIEDLQSFLLMIIVHLWNGTPVQRNRARQSYPDLV

DKARRLNLLHVVSDPHNQSPLHLPDFDPSCLKPEHFNWDSWVDQERRIRLMLGIYLCDTA

MGLYFNMPPRFDPAEIHLPLPCDDAAWDASTPELCAASLGLYGPDSARQHNPYGTQRAKQ

PRLDWALQALLHNSREVQPGTTNLYGKFVLIHALLALIRRTQVDGQVYQHQTPPRNEWTK

LADARGERSSQGHGAADPRGHDALSIALDKFKEMWDVDIRDQFPPQTSGVNNPRRHGFSR

DAIHFYWTARYLMKFTTREDLEMPADQRLVLVFEILKGVRSWVTSDAFERGEELGSIGEI

DDQFAIMDLDLTKLFKPLSSRTREEDTSTPSVNTV

>SoG_00798.T1

MQGPGGRLTSLSSRQPHIFPTTSHISLTSAPGPQPRLRQPPKLSEASGTTWPISAPHEAT

SRPGEPRSCWTAPTGHLPPVFQATGNPMESPGPQAPPPLALRGAFTFFKLPPTSSTALSP

PSFSSHLFSCISILSPFHSQACLELLNPSRELYVFIYIQGKEPHPPHPTHPPPRSRSAIL

CSVSIVGGLPFDTPHLYTTVTMAETFEFQAEISQLLSLIINTVYSNKEIFLRELISNASD

ALDKVRYKALSDPSQLDSGKELRIDIIPNKENKTLTIRDTGIGMTKADLVNNLGTIARSG

TKQFMEALTAGADVSMIGQFGVGFYSAYLVADQVRVISKHNDDDQYVWESSAGGTFSISA

DTEGEQLGRGTAIVLHLKDEQMDYLNESKIKEVIKKHSEFISYPIYLHVQKETEKEVPDE

DAEVEEVKDEEGDDKKPKIEEVDDEEGEEKKPKTKKVKETSIEEEQLNKQKPIWTRNPQD

INQEEYASFYKSLSNDWEDHLAVKHFSVEGQLEFRAILFVPKRAPFDLFETKKTKNNIKL

YVRRVFITDDATDLIPEWLSFVKGVVDSEDLPLNLSRETLQQNKIMKVIKKNIVKKSLEL

FQEIAEDKEQFDKFYSAFSKNLKLGIHEDSQNRSILAKLLRFNSTKSGDELTSLSDYVTR

MPEHQNNMYYITGESIKAVSKSPFLDTLKEKNFEVLFLVDPIDEYAMTQLKEFEGKKLVD

ITKDFELEETEEEKKAREAEEKEYEELAKALKNVLGDKVEKVVVSHKLGSSPCAIRTGQF

GWSANMERIMKAQALRDTSMSSYMSSKKTFEISPKSTIVKELKKKVEADGENDRTVKSIV

QLLFETSLLVSGFTIDEPAGFAERIHKLVQLGLNIEEDDAAPAEAATETPAVATGDSAME

EVD

>SoG_00802.T1

MDFAQLRSNGHVLSPVPARPVVLTPTSASTTTTGFPSPTDATAPNRVSHGLVRSISDEYD

DGGSAGPNSHADEPPKKKQKRNKPTLSCHECVEQVGHIAWLSAGIKRQTDCTYDHVANLL

EYVWITTKSTLDQSTDCERRETNRTAANGRRMTKPPKKKPAGQSGKDVIPNIADRGFLHG

RDASSRGAIALSTGLMSNIPYTFPSSSNVFGIGSEHPFANYWTCEGGLPEVISVLPEKIQ

ADILLGRYFECVDPVYPMIHRQTFYADYEHFWSLSQDDKNGTDADFVGLMFVMLGLGTQF

VTSTSPKEKKQTAEFYASAANQALRMFSYLSQASVRSLQALVLMVYFLINDNHASDGWSF

AGVVVRQAYAMGLHRDPNIVTPEASPFEKQQRRKLWQAVLLQDTFLTVLLSLPPSATHTD

VNVDDLLDDGLSIASSDPTDVAYIRGSWTLANLVQETICSPRSLDLPICTTARHKSKLVA

DFRSVYRSFPDVFRSWDPDSLTVLAGTNKRVVRQTLFLTSNYFHNLMLVHASESEDVPVN

VRGTLEAAHDAINAFFMLFTLLEPEARVWWHFNHRAFLEALCMGNVLREAAKEPGGDDIA

ARDPLFARAKSDICEFPPPTFLGLNVAGQAGS

>SoG_00821.T1

MGVGNKRTLVKTRRKTRDVDQIKADLLSPKHLAQFKKTKAEEDLPGLGRHYCIECAKWFE

TETSLLGHRRGKPHKRRVKQLREDPYTQKEAEAAVGLRTDNGKDYTTAKSGDIDMAV

>SoG_00827.T1

MSGHPGSGGTGGRNDYHDQYDDGYGQQGGGQAGYYQDDQHYDNGYDARGGYDNRGQGGAY

DNRGGNDGYYDESGYYNADPNNPYHQDGGYYDGHDQYQDDYYNNNGQDGYYDQYDQQGYD

RQRGRHGSEEDSETFSDFTMRSDMARAAEMDYYGRGDERYNSYGDQMGGRGYRPPSSQIS

YNGNRSSGASTPNYGMDYGNVLPAGQRSREPYPAWTSDAQIPLSKEEIEDIFLDLTAKFG

FQRDSMRNMYDHMMVLLDSRASRMSPNQALLSLHADYIGGDNANYRKWYFAAHLDLDDAI

GFANANGKAFGRRKKKGKKAKKGQDEAEALAEIERDDSLEAAEYRWKTRMNRMSQHDRVR

QIALYLLCWGEANQVRFMPECLCFIFKCADDYLNSPACQALVEPVDEFTFLNNVITPLYQ

YVRDQGYEILDGVYVRRERDHKHIVGYDDCNQLFWYPEGLDRIVLQDKSKLVDLPPADRY

LKLKEVNWKKCFFKTYKESRSWFHLLVNFNRIWVIHLTMFWFYTSHNAPSILVGPSYQQE

LNQKPEAAKQWSVVGFGGAIASLIQIIATIAEWAYVPRRWAGAQHLTKRLLFLILVFIIN

VAPGVKVFMFPTPGTVDLIIGIVHFVIAVLTFLFFSIMPLGGLFGSYLTKNSRRYVASQT

FTASWPKLTGNDMAMSYGLWLVIFGAKFGVAYPYLTLSFRDSIRYLNTMKVRCVGDALLP

GKDILCKNQPTILLIIMAITDVVFFFLDTYLWYVLINTAFSVARSFYIGSSILTPWRNIF

SRLPKRIYSKVLATTDMEIKYKPKVLISQIWNAIVISMYREHLLAIDHVQKLLYHQVPSE

QEGKRTLRAPTFFVSQEDHSFKTEFFPSYSEAERRISFFAQSLSTPIPEPLPVDNMPTFT

VMIPHYSEKILLSLREIIREDEPYSRVTLLEYLKQLHPHEWDCFVKDTKILADETAQMNG

DEKDEKDAAKTKIDDLPFYCIGFKSSAPEYTLRTRIWASLRSQTLYRTVSGFMNYSRAIK

LLYRVENPEVVQMFGGNSDKLERELERMARRKFKLMVSMQRFSKFKKEEMENAEFLLRAY

PDLQIAYLDEEPPLNEGEEPRLYSALVDGHSEIMENGMRRPKFRIQLSGNPVLGDGKSDN

QNHSLIFYRGEYIQLIDANQDNYLEECLKIRSVLAEFEEMKTDNVSPYTPGVKSEQRFPV

AILGAREYIFSENIGILGDVAAGKEQTFGTLFARTMAQIGGKLHYGHPDFLNGIFMTTRG

GVSKAQKGLHLNEDIFAGMNALLRGGRIKHCEYYQCGKGRDLGFGSILNFTTKIGTGMGE

QLLSREYYYLGTQLPLDRFLSFYYAHAGFHVNNMFIMLSLQLFMIVLLNFGALKHETIAC

DYDRKVPITDPLFPTGCANTDAIMDWIYRSVMAIFFVFFLSYVPLIVQEIMERGVWRAVL

RFLKQFFSFSPFFEVFVCRIYAISVQQDLSFGGARYIGTGRGFATARIPFGVLYSRFAGP

CIYFGIRLLMMLLFATVTCWQAALTYFWVTLLGLTISPFVYNPHQFAWNDFFIDYRDFLR

WLSRGNSRSHSSSWIAFCRLSRTRITGYKRKALGDPSAKMSADVPRAAIANIFFSEILAP

LFLVVVTVIPYLFINAQTGVHAYNNDDKKPQPTDSIVRLLIVTFAPIGINAGVLAVMFAM

ACFMGPVLSMCCKKFGSVLAAIAHGLAVVFLLVFFEAMFLLEGFNFARTMAGMIAVMALQ

RFFLKLIISVALTREFKTDQSNIAFWTGKWYSMGWHSVSQPAREFLCKITELSMFAADFV

LGHWLLFMMLPMISIPKIDMLHSMMLFWLRPRYVLFAEILLANLDIDLRSSRQIRPPIYS

MKQSKLRRRRVIRFAILYYVMLVLFLALVVGPAVAGDKIMPSMPSMLKPTGSLAGFKLVQ

PWNLNNTDTNSTMLTGTGRPGYKTESGNAKATAKVKLF

>SoG_00846.T1

MSAKAIHEADGKAVINYHLTRAPVIKPSPLPKPTKHNPPSRLASLHFNDDDDVNAVLDQA

EVSFPWLLQAGSKFVAKPDQLIKRRGKAGLLALNKTWAEARAWVAERALKEQKVENTTGV

LRHFLVEPFVPHPPETEYYININSVRDGDWILFTHEGGVDVGDVDEKAEKLLIPVDLTEY

PSNEEIAATLLKHVPKGVHNVLVDFITRLYAVYVDCQFTYLEINPLVVIPNEDKTSAEVH

FLDLAAKLDQTADFECGVKWAVARSPAALGMTAVASTNGEKVNIDAGPPMEFPAPFGREL

TKEEAYIADLDAKTGASLKLTVLNPNGRIWTLVAGGGASVVYADAIASAGFADELANYGE

YSGAPTESQTYHYARTVLDLMLRAPLSDKGKVLFIGGGIANFTNVASTFKGVIRALRDYA

TQLNEHNVQIWVRRAGPNYQEGLKNMKAATQELGLKAKIFGPEMHVSGIVPLALVPGKWE

EANVEEFKA

>SoG_00847.T1

MAPTASNGANGAKGGLSANDNIQRFAAPSRPLSPLPEHALFNEKTRCFVYGLQPRAVQGM

LDFDFICKRKQPSVAGIIYTFGGQFVSKMYWGTSETLLPVYQQVDKAASKHPDVDTVVNF

ASSRSVYSSTMELMEYPQIKTIAIIAEGVPERRAREIAHVAKKKGVTIIGPATVGGIKPG

NFKIGNTGGMMDNIVASKLYRKGSVAYVSKSGGMSNELNNIISNNTNGVYEGVAIGGDRY

PGTTFIDHLLRFQHDPECKILVLLGEVGGVEEYKVIDAVKQGVITKPIVAWAIGTCASMF

KTEVQFGHAGSFANSQLETAAMKNKKMKEAGIYVPDTFEDMPGVLKSVYDKLVKDGTIVP

QAEPVVPKIPIDYSWAQELGLIRKPAAFISTISDDRGQELLYAGMPISDVFKEEIGIGGV

MSLLWFRRRLPTYASKFLEMVLMLTADHGPAVSGAMNTIITTRAGKDLISALVSGLLTIG

SRFGGALDGAAEEFTKAFDKGLSPREFVDGMRKANKLIPGIGHRIKSRNNPDLRVELVKE

YVIANFPSHKLLDYALAVETVTTSKKDNLILNVDGCIAVCFVDLLRNCGAFSPEEAEDYL

GMGVLNGLFVLGRSIGLIAHFLDQKRLRTGLYRHPWDDITYLLPNLRESGAPGAEGRVEV

QM

>SoG_00858.T1

MSLTNCRFYEEKFPEIDSFVMVNVKQIAEMGAYVKLLEYDNIDGMILLSELSRRRIRSIQ

KLIRVGRNEVVVVLRVDKEKGYIDLSKRRVSPEDIVKCEERYNKGKLVHSIMRHVAEKTN

TPIESLYESIAWPLNKKYGHAIDAFKLSITNPEVWNDITFPSTPIADELKLYISKRLTPQ

PTKVRADIEVTCFGYEGIDAVKTALRTAEAANTEDTQVKVKLVSPPLYVLTSTCLDKNAG

ITRLEEAIVDIRKSIEGAGGQLTVKMEPKAVTESDDAELQALMEKRERENAEVSGDESVS

DSDDHMPDTI

>SoG_00859.T1

MAQDASTLSRKRPQSPDATEELAGKRQKLSEGHTTVSSGFAGLNAIANKISQAFRYSSEA

KQTHAYPPPPPAVQNGVHSPTTAFSPSASPRPMGLPPPAATSPSPAKYRPAIKLAALRGT

KWDDGSVPKSPSPKKTTPRRKPAGAASLQGTPTKATPTPNKGSPLKRASAVGDAESGDEM

SASGRTPASTSKAAGAKRLFSSSKAATPKSILTPKKRRGRPPKNVKFGSRLDDDEVFFED

ARKPGRPSKASKAYADEDEGEGEIVCGICARGHSKAPNQIILCDNCDFAVHQECYEVPEI

PTGEWLCKSCAQEDVLKTPKKLDDVAEIVRDVEVPDIPNLDKHVRALQRVLLDRCTGHRP

LRIFGQQEAQDKARQLVEQTVVAGEGNSMLLIGARGSADAAIQLLEGIISDLSKEHAKDF

HVVRLNGFIHTDDKLAVKEIWRQLGKEMDVDDDLLNINNYADTMTSLLALLSHPSEIMGT

GDGVTSQSVVFIIDEFDMFATHPRQTLLYNLFDVAQSRKAPISVLGCTTRLDVVEMLEKR

VKSRFSHRYVYLHLPRSLPAYWQMCKQGLLVDEQDAEAEGIDTQLEGYAEFRNYWSHKIE

ALYKDRAFQDLLQYHYYTSKSPTAFFAEWITALASISGSDLTLTIPPSPSADGASLLPPD

SRMHLLSTLSELDLGLLISAARLDIIAHTDTVNFAMAYDEYNSLVGRQRVQSATSGMLAL

GGVARAWSRGVAGIAWERLVSLGLLVPAGIGGAKGLGHGGLEGKMWKVDVALEEIPTGVK

LNAMLAKWCREI

>SoG_00870.T1

MPGREGQVASPGEVDDRDESARLGSPYNDAAVEGHGAEGGPQDASPTSDGGPARKRRRSR

KGLDKRFECSEEGCGKSYSRAEHLYRHQLNHNSKQTFRCDFPNCTRTFVRGDLLKRHKDR

HTNKGSQLSRRDSVISHVAQAGQTGGLSSDQQNASHSYPNGRQSQAIYNSPHEGSATPFT

PGSVTQPDVFGNSTPLQVAGEFSHDARYAQASPHRQEMDMTTNHSRQVSGSRMGSYGVMS

PVSNQRGYSGQQSQMLRSDSFVPQQSVQPINLPPAQYPTFQTMGSRAEETAYSNHVAQDY

SEASNHMMLDQMAMPGTAPVFGTDGALNKSPYVGMPLDFMNYLFNSEVHAGHGSPMSSPI

IPVSYTNYGDLAGFYMSNEHGASGYFPTGPQQVMAVNNLLEQNVPENTISEEKGQEIFDF

IKERFKEKDQAPIERQREGILDGDRSDPEHMLSTRMMKAYISSYWYHFSDQMPILHKPTF

SPEKTPTLLLLAMMTIGAACLDRTHGPKVTKAGAQLSNFLAWYLRWEVFMDVNSRPPAKL

WIFQTLILLELYEKMYSTRELHERAHIHHATTITLMRRGRSLIGKSAMDSPPNPRDSANG

SRHSSTSGAARTPEEWWNDWITSEATRRVAFAAFVIDSIHATMFGHSAFMVAHEMRLPLP

CDESLWRATSSAEVGRVESNLMSRGIKPMSFLEGLKRTLSGQEVRTNSFGRTVLMAGLLS

VTWHMHQRDLQVNVLGGGVVVALGGRDKWRATLTKAFDEWKSDFDKALLRSESAMEQYRY

DKNEFNIVFESRTVLHHLAHMAMHADIVDCQMFARAKRLLGRTIGPQEFNSAKRRIKDVW

APSAKARDATFYALKFLCSVLVPESTEHGITSGHFYEEQYEARDDVLLNRAWVLYFAALV

VWCYGYALEGPCWKVPAPTTREEKHQQMREYLLKFGGIADPNELRPLTGVNQNTALLMVL

KDSFETTRWELLQEGSNLLNNCIVLNCEGALG

>SoG_00879.T1

MIPSRYVRLARPPNIFSIASKCPLAARLGRCTYSSEATAGKHNRFSGSATGVPQGRDEDE

SGGGVSGFKQQRLADLARAKEGEAPLITDSYPRLKSDPLRMSNPAFRSRFAQLEQSRSRQ

SEPERITEIENLEPLPDADESSSNPDAIPTEEDFNDGFHQAEREATVLRATLAGRVRAKR

VVGSGLIFVDIVNDFQKVQVMINKKKCFVTEHRQAFKMWRNLIQVGDHISVTGVSRLTDA

GELTLEAESLPELLSPTMEQIPERLTDSKTKMEERHLDMLVNKQTTDVLRLRSAITKHLR

DHFNSNQFLEMQTPILADNAGGAIARPFKTRATEFPDRELALRIAPELWLKRLVVGGIDK

VFEIGPAFRNEGIDGTHNPEFTSCEFYSAYSNLQELIEQTEQILFGMASECQKLISTDLT

SLPEIDVDMFRGPFKQVEFLPSLEAALGIRFPNLSAQGALPELIAILKLAGVDLCGDTPK

SLHKLLDRLATLYLEPMSFEQPIFITNHPVCMSPLAKGFLCPTTYQLVSARAELFVGGRE

LANMYEEENDPAEQSRKMNTHRRLVNKPGDEMGLRPESEADLEEPEEVDDEIPPVDQAFL

RALEYGLPPTGGWGCGVDRLVMLFSGTDRISDCLSFGTLRNVVNRNNA

>SoG_00885.T1

MRAQSERFGTEIVTDTVTTLDLSSRPFKFSTEFNPGETHTADAVIVATGASARRMNLPGE

DTYWQNGISACAVCDGAVPIFRNKPLFVIGGGDSAAEEATFLTKYGSHVTVLVRRGELRA

SRTMANRLLAHPKVTVKFNTVATEVRGGEDGLMSHLVVKDVQTGKEEVLEANGLFYAIGH

DPATKLVKGQVDMDEDGYIVTKPGTTLTSVEGVFAAGDVQDKKYRQAITSAGTGCMAALE

AEKYLADHEMDERADDRKD

>SoG_00888.T1

MARPSSSRGKSPQPPRNRGKSPQPSPMEDQPAPVAPAASLAPATAATPSLKKKKSKSKLN

AASSYKSDGVEDNDVFLLPSSDYWIMLGMTVLAAAVRVFRIYQPSSVVFDEVHFGGFATK

YIKGKFFMDVHPPLAKMLIALTGWLAGFDGSFDFKEIGKDYIEPGVPYVAMRMFPAICGI

LLAPFMFLTLKVLGCRTMTAMMGSGLIIFENGLLTQARLILLDSPLVAATAFTALSFNCF

TNQHELGPSKAFSPVWWFWLVMTGLGLGITASIKWVGLFTIAWVGALTVLQLWVLLGDTR

NVTLRIWSKHLMARAFALIIIPVTFYLAMFAIHFVCLVNPGDGDGFMSSEFQATLNTKAM

KDVPADVILGSRVSIRHVNTQGGYLHSHPLMYPTGSKQQQITLYPHKDDNNVWLLENQTQ

PLGPDGEPINGTLAWDDREPTYIKDGMILRLYHIPTDRRLHSHDVRPPITEAEWQNEVSA

YGFKDFPGDANDYFRVEIVKKKSHGAVAKERIRTIETKFRLVHTMTGCVLFSHKVKLPDW

ASEQQEVTCAKGGTLPNSLWYIEYNEHPKLTGDVEKVNYRKLGFFGKFIELHKVMWKTNA

GLTDSHAWDSRPESWPILRRGINFWGRNHTQIYLIGNPIIWWASTLTVAIWVGFKAIAVL

RWQRNCNDYANTTLKRFDYELGTSVLGWALHYFPFYLMKRQLFLHHYFPALYFAIIALCQ

LFDYATARVPVLSQAQKRVANRVATVTFLALSVSAFFLFSPLAYGNQWTKGECKRLKVLG

TWDFDCNTFFDSAWLRRHGHMSTESSRATSKQTTRGIYDQYAEMSNAPTISSSVTPDAAK

PVQPNNQAKEAAAQSPKGELPADISGAPAVPQGARVLRKEEKLEYRDQDGNLLNEEQVKE

LEGKVEFQTRYETRTRVVDEAGNELEMPEGGWPADYSPVAPPHPDVQGVDKETVRAEGEQ

EVPKEVDASKDGEKEAEKSQAKPASEGKKATGHEEL

>SoG_00892.T1

MPPPPVPSKSIPQDPKVTSGDNGAEVNGTRLFKELHLGLFEIGKPLGKGKFGRVYLARHR

SSGFICALKVLNKDEIRREGAEMHVRREIEVHSNLRHPGIVGFHGWFHDSRRIFLILDYL

PGGELYRVLRREGRFSERRAAKCAAQGAQSLEYLHSKNIMHRDIKPENILIGLHDELKVA

DFGYSVHAPSNRRDTLCGTLDYLPPEMLTSSKAQYTKAVDQWTLGVLTYEFLTGEAPFED

SPAMTHRRIAKGDMTPLPASLSKEAKDFVHSLLVMDATKRMPLSSVMLHPWIVRNCKNK

>SoG_00909.T1

MRPEVEQELAHTLLVELLAYQFASPVRWIETQDVFLAERNAERIVEVGPADTLGVMAKRT

LASKYEAYDAAKSVQRQILCYNKDAKEIYYDVDPVEDEPEAAPAASGSGSQAPAAAAPAA

APAAAAAAPAPSAGPAAQVPDEPVQAVDIVRALIAQKLKQPMADVPLNKAIKDLVGGKST

LQNEILGDLGKEFGSTPEKPEDTPLDELGAAMQVTFDGNLGKQSLSLVARMISSKMPGGF

NITAARKYLETRWGLGPGRQDGTLLLALTMEPAARLGSEGDAKAFLDSVAQKYASNAGIS

LSTAAAAGPAGGAGAGMMMDPAAIDALTKDQRMLFKQQLELFARYLKMDLRSGDKAFINS

QKSEKVLQAQLDLWNAEHGDFYASGIEPAFSPLKARTYDSSWNWARQDALSMYFDIIFGR

LQVVDREIVSQCIRLMNRSNPKLLDFMQYHIDNCPTERGETYKLAKKLAEELMENCKDVL

NIPPHYKDVSIPTGPRTTIDARGNLNYEEIPRASCRKLEHYVQQMAEGGKISEYGNRTKV

QSDLSRIYKLIKQQHKMSKTSQLEIKSLYGDVIRSLAMNESQIIPKDGPKGRKSGLKGTS

SNKGKIETIPFLHLKRKTAHGWDYSKKLTGIYLNGLEEAAKSGVTFQGKHVLMTGAGAGS

IGAEVLQGLISGGAKVIVTTSRFSREVTEYYQSMYTRFGSRGSQIVVVPFNQGSKQDVEA

LVDYIYDPKNGLGWDLDYIVPFAAIPEQGREIDGIDSRSELAHRIMLTNTIRLLGAVKTQ

KSDRGFETRPAQVVLPLSPNHGTFGGDGLYSESKLGLETLFNRWHAESWGNYLTICGAVI

GWTRGTGLMGGNNIVAEGVEAFGVRTFSQNEMAFNLLGLMAPSIVDLCQDQPVFADLNGG

LQFIPNLNTTMTKIRKDIMETSEIRKAVSKESALENAVVNGADSEVLYQKKTIEPRANIK

FDFPKRPDWKTEVAPLNDKLKGMVDLERVVVVTGFAEVGPWGNSRTRWEMEANGEFSLEG

CIEMAWIMGLIKNHNGPIKGKPYAGWVDAKTGNPVDDKEIKAKYEKQILEHSGIRLIEPE

LFGGYDPNKKQLLHEVVIEEDLEPFETSKETAEEFKREHGDKVEIFEIPESGEYTVRLKK

GAGLWIPKALRFDRLVAGQIPTGWDAKKYGVPDDIISQVDPVTLFLLVSTAEALVSCGIT

DPYEFYKYVHVSEVGNCVGSGMGGAAALRGMHKSRYLDQPLQNDILQESFINTMPAWVNM

LLLSSSGPIKTPVGACATAVESVDIGYETIMEGKARICFVGGLDDFGEEGSYEFANMKAT

SNTVDEFAHGRTPQEMSRPTTTTRNGFMESQGCGIQIIMTGQLALDMGMPIHGILAMTTT

ASDKIGRSVPAPGQGVLTSAREHQGKFPSPLLDINYRRRQIERRKKQIKQWQESELEFVH

DEVDAMKSQGATFDEKEYAQDRIAHIEKEAKRQERELLRSMGNNFWKQDPSIAPIRGALA

TWGLTIDDLGVASFHGTSTKANDKNESSVICQQLRHLGRSKGNAVLGIFQKYLTGHPKGA

AGAWMMNGCLQVLDSGLVPGNRNADNVDPVMEDFDLIVYPSKSIQTDGIKAFSVTSFGFG

QKGAQVIGVHPKYLFATLDEQTYATYCAKVEARQKKAYRFFHNGLINNSLFVPKASSPYT

EEQLSSVLLNPDARVTEDAKTAGLTFSANFMKQSEKVVPSEKAKETQKVMEALTLKVTDK

NSKVGVDVEDISAINIENDTFLERNFTKDEISYCQSAPSPASSFAGRWSAKEAVFKSLGV

PSRGAGAPLSDIEIRRDANGAPVVQVRHCPIPKKKKKTKKN

>SoG_00910.T1

MYGTGTGPQTGINTPRSSSSLRPLTLSHGSLETSFLIPTSLHFHAQQLKDRFVASLPEAT

DELAQDDEPSSIPELVARYLGFIAREVEEGEDDSQGSYEEVLKLVLNEFERAFLRGNEAH

ALAATLPGIESKKLEVIRSYYAARATSNRAIRPHESALFRAADDGSARIYAIFGGQGNIE

EYFEELRELFTTYSSFTSELIATAAELLQTLSSHSSAEKMFPKGLDIMNWLQHAELTPDV

DYLISAPVSFPLIGLVQMAHYEVTCRVLGVDPGRLRERFSGATGHSQGIVMAAATAAADS

WDSWREIVTSTLTILFWIGTRSQQTFPTTSMTPTMLRESLEHGEGAPTPMLSIRDLPQSE

VQKHIDATNQYLPADRHISISLINSPRNMVVTGPPTSLYGLNTQLRKFKAPTGLDQTRVP

HTQRKVRFANRFLPITAPFHSQYLKEATSLIDEDLKEISIDAKSLGMAVFDTSSGRDLRE

VDGNIVPALVRLITRDPVHWEKATVFPGATHILDFGPGGVSGLGVLTSRNKEGTGVRVIL

AGTVDGTITEVGYKPEIFDRDEENAVKYAIDWVREFGPRLVKTSSGKIIVDTKMSRLTGL

PPIVVAGMTPCTVPWDFVSAVMNAGYQTELAGGGYFNANMMTAALNKIEGAIPAGRGINV

NLIYVNPHAMAWQIPLLARLRAEGVPIEGLTIGAGVPSIEVAQEYIETLGLKHISFKPGS

LDAIQAVINIAKANPTFPVILQWTGGRGGGHHSFEDFHQPLLQMYGRLRRQENIILVAGS

GFGGAEDTYPYITGEWAKAYGYPPMPFDGCLFGSRMMVAKEAHTSKAAKQAIVDAPGVGD

EEWEKTYKGPTGGVITVRSEMGEPIHKLATRGVRFWSEMDQKIFSLPKEKRVAELKKNRE

YIIKKLNADFQKVWFGCNKEGKSVDLEDMTYAEVVRRMVELLYVKHQSRWIDPSFMRLTG

DFIQRVEERFTTESGKPSLLQNYADLQQPFEVIERILAHYSEAENQIINAQDVQHFILLC

KRPGQKPVTFVPALDDDFEYFFKKDSLWQSEDLDAVIGQDVGRTCILQGPMAVKFSTVID

EPIKDILDGIHHSHIEFLTRDFYSGKPASIPAVEYIGGKIVESEIPLDVEGLTVSYDAHK

NTYRLASSVAATLPSLDSWLSLLAGPNRNWRHALLMSDVVVQGKKFQTNPLRRIFAPARG

LFVEIDYPNDPSKTVIVVKEQPRHNQYQDVIEVRLTGQNEIIVNMIKDTTALGKPVALPL

KFTYHPDAGYAPIHEVMDGRNDRIKEFYWRAWFGDEPLDLDALVTSKFDGGKTTITAEAI

NDFVHAVGNTGEAFVDRPGKTVYAPMDFAIVLGWKAITKPIFPRTIDGDLLKLVHLSNGF

RMIPGAEPLKKGDVVSTTAEINAVINQDSGKMVEVCGTLVRDGAPVMEVTSQFLYRGAYT

DFENTFQRKHEVPVQVHLATTKDVAVLRSKQWFNVDEMPSEIDLLGQTLTFRLQSLVRFK

NKTVFSSVQTQGQVMLELPTKEIIQIASVDYEAGESRGNPVLDYLERHGSPIDQPINFEN

PIPLSGKKTPLQLRAPASNETYARVSGDYNPIHVSRVFSNYADLPGTITHGMYSSAAVRS

LVETWAAENNIGRVRSFHASLTGMVLPNDDIQVNLQHVGMVSGRKIIKVEVSNVETEDKV

LLGEAEIEQPVTAYVFTGQGSQEQGMGMELYASSPVAKEVWDRADKYLQDSYGFSITNIV

KNNPKELTIHFGGPRGKAIRQNYMAMTFETVAADGSIKSERIFKEIDETTTSYTYRSPTG

LLSATQFTQPALTLMEKASFEDMKAKGLVPRDSTFAGHSLGEYSALAALADVMPIESLVS

VVFYRGLTMQVAVERDEHGRSNYSMCAVNPSRISKTFNEEALQFVVNNIAEETGWLLEIV

NYNIANMQYVCAGDLRALDTLAGVTNFIKKQQIDVEEMRTNIEEAKDALRQIIRGCAEVT

LKKPQPLELERGFATIPLRGIDVPFHSTFLRSGVKPFRSFLLKKINKTTIDPSKLVGKYI

PNVTARPFELTKEYFEDVYRLTNSPRIGAVLANWEQYSQEEPTTNGQAGSDSGVSVAYDG

PGAAA

>SoG_00922.T1

MTNSNLVAELKRQIAEHEAASARLQEELAYQERLENDGHFGSSKLPARSLSTAGHAMATQ

SRSQASNDEPAPKRKKRAMSQQVTHQMSRNTSNRPETGPFLQTGAALQPLPTPSRQGLNV

LQTQDKMHHHPQDGPAMPDVGVRPDVFLAGLGPDHPYIPALGVEFSPVDLSNTLSVSKCP

SMSSGWSAGDGLTPMTRQNSLFDDNMVSGLDMSRWASGSQSSRGFDDLMFSQGHQAKNPC

VEQDFLGVGANLASTDMTRSFSSSLQSQMLLSPPDSGTDSTAMERSDSNETTSSVRSTAS

SKRRHKEALERVIKASRGTLLAPKPHPSGAEKPRAVLPAKKEGKVAVPPKPVYARRKPTK

VYCGQCNEHPDGFRGDHELRRHCLSKHEVTVKKFICRDPASVGIPTNLKAIKPLSDCKAC

RAEKPYGAYYNAAAHLRRQHFKPRSTRSKGKSEEKRGGKGGGDWPPMQELKDWFEVRRVP

LNQAEGASADHEDDGSDPTDGDMEELDPMAYSLESTTTPYDIVPMGDGNMLSLDATAASA

AASFGLDQFADVSSVPEVMTGFVDEGQLYDSTFSAVNAVISSNLQDMGQLSW

>SoG_00942.T1

MSSDGSSSEKPANPSLKRVDLDGHDLPPSPTPSSPRNGRRRYALATELVYTDAKDQYGAS

SIPIYQSATFKQSSGRGGQQEYDYTRSGNPTRTHLERHLAKIMNANRALAVSSGMGALDV

ITRLLRPGDEVITGDDLYGGTHRLLTYMATNQGIVVHHVDTTDPSSVRAKISDKTAMVLL

ETPTNPLIKVVDIPGIARAAHEANEKALVVVDNTMLSPMLFNPLDVGADIVYESGTKYLS

GHHDIMAGVIAFNDNAIGDKLFFTINSTGCGLSPNDSFLLMRGVKTLAIRMEKQQANAQA

IAEFLENHGFRVRYPGLKSHPQYDLHWSMARGAGAVLSFETGDPAMSERIVEAARLWGIS

VSFGCVNSLISMPCQMSHASIDAKTRRERQMPEDIIRLCVGIEDADDLIEDLSRALVQAG

AVTVTLDGFYAKGAAEEIGQTPLTVQ

>SoG_00949.T1

MKGEILHLHLGQAGTQLGNSAWELYLLEHGLGPDGRPDPNAKDVGDPGSFETFFTETSGG

KHVPRSLFIDLDPSPIDEIRTGSYRQLFHPEMLISGKEDAANNYARGHYTIGKEMVDSVM

DRVRRVADNCNSLQGFLIFHSFGGGTGSGFGALVLERLATEYGKKSKLEFAVYPAPRTST

AVVEPYNAVLSTHSTIENSDCTFLVDNEAVYDICRRNLDIPRPSYEHLNRLIAQVVSSIT

SSLRFDGALNVDLNEFQTNLVPYPRIHYPLISYAPVVSTTKSQHEGFRVPDITFQCFEPN

NQMVVCDPRNGKYMAVALLYRGDVVPRDCNAAIAALKAKASFNLVEWCPTGFKLGINYQK

PMAVPAPPEEGGLASVERSVSMLSNTTAIAEAWARLDYKFDLMHSKRAFVHWYVGEGMEE

GEFTEAREDLAALEKDYDEVAADSFEPEEEAEY

>SoG_00957.T1

MRVSTLIAVLPMAMAAPQVKRTSPAPLIIPRGVDLIEGKYIVKMNDDTVSASIQGAIDKI

NADADFTFSQQFNGFVASLSGDELTNLRYDPNVLYIEQDAKVTMWATQEDAPWGLARISS

KSPGGSTYTFDESAGEGTCAYVVDTGIDVSHPDFNGRATWVANFVDNSDRDGQGHGTHVA

GTIGSTTYGVAKKTKLFAVKVLDDNGEGSNSGIIAGLEFVVKDAAQQNCPKGVVVNMSLG

GGYSSAVNAAAASISRAGLFLAVAAGNSHADSKNFSPASEASACTVGATEKDDSLASYSN

FGSPVDVLAPGTDIESTWTGGGVKTISGTSMASPHVAGIAAYFLGQGQSASGLCEYIASN

SLKGVVSGVPNDTKNLLINNGGRNNTTTLPDRPARPARPGRPNRPARPF

>SoG_00974.T1

MADKEPTAAENAAARAKEAEEQAALPYKWQQTISELDVTFNVPGNLKSRDLVIEIKKQKL

SAGVKGEEPVISGDLPHPIHVDDSTWTLSTNADGTKTVEIHLDKVNKMEWWAHVVQTAPK

IDVTKIQPDSSKLSDLDGETRGMVEKMMYDQRQKEAGLPTSDDQKKADILKKFQEQHPEM

DFSKAKIQ

>SoG_00986.T1

MDDLEKLEELSLVSKVVSELQNHLGMNNKTLAEYLIHLRVESKDFEEFRDKIKQTGSALP

ASLVESLDRLVRNLHPKLKAQAKTAAAEPHHRTFKEKEQTFSGLALPDKAAAPDGGADAI

DDTLALLEGLESKAGGDRGPRKRSRSPYEDGPKDSRRRKERNRDRSRSRDRKRRDRYRER

SYSQDRGDEDWRDGHRDSWKRRDRHRGRDHDDDRLNRSPEPEIDDEPQMHKVYEGHVTGL

KPFGAFVNLHNVRGKVDGLVHVSRISESRVEDPSDFLSQGQRVFVKVTNIEGKRIALSMK

DIDQETGRDFEPQARLTSGANMEALGGGGRNGFSNPSAAGPVGGISAPGRARKRMTSPER

WEIRQLIASGVAKASDFPDLEDDYNATLRGDGDMELEEDVDIEVREEEPPFLAGQTKQSL

ELSPIRVVKAPDGSMNRAAMSGSELAKERKDLKQQEALAAKKETPKENLSSQWQDPMADP

DKRQFAADLRNARNNAAPQRAPEKKPTRSSNPVLVKRSNMSMAEQRESLPVFAFRSQLIK

AVEDNQILIVVGETGSGKTTQLTQYLAEAGFANNGVIGCTQPRRVAAMSVAKRVSEEVGC

QLGHEVGYLVRFDDMTSPATRIKYMTDGMLTREILLDPDLKRYSCIMLDEAHERTINTDV

LFALLKKATKRRPDLKIIVTSATLDADKFSSYFNECPIFTIPGRTYPVEVLYSKEPESDY

LDTALLTVMQIHITEEPGDILVFLTGQDEIDTAWEILNERMKALGPSVPELHVLRMYAGL

STEEQNRVFDPAPPGTRKVVIATNIAEASITVDGIVYVVDPGFVKQKAYDPKLGMDSLVV

TPISQAQANQRAGRAGRTKPGKCFRLYTEAAYQSEMLPMTIPEIQRTNLSSTILTLKAMG

INDLLHFDFMDPPPINTMLTALEELYALSALDDEGLLTRLGRKMANFPMNPSNAKVLLAA

VEHHCAEEVLSIVAMLDEQSVFSRPKEKQTQADQKKAKFHDPHGDHLTLLNVFNSWKQNG

FSREWCFENFINWRAMRSAKNVREQLVGIMQRYKHPITSCGRNTQKVRQALCSGFFRNAA

RKDPQEGYKTLIEGTPVYLHPSSALFGKQAEWVIYHTLVLTTKEYMTHTTVIEPKWLVEA

APTFFKVAGTDRLSKRKAAERIQPLYNKFQGDDDWRLSAQRRGGRGGGGGTWG

>SoG_01009.T1

MTDKQTQNSEKPLHKSSSEGLSSSNPSNMDHHTASEHEPAGEQKQQQQQQNEKEQEQKQN

DDAADASQGWVMDTSDFPGPRALTLIMIALFLALFAANLDTTILATAIPYITNEFRTIRD

VSWYGAAIMLVSASFQSTWGKIFKYFPIKLTFLFSILIFEVGSLICALARNSTTLVVGRA

VAGLGASGVTAGVFILIAFSAPPKYVPAFMGLGGATYAVASLAGPLLGGVLTQSATWRWC

FWINLPIGGVTAAIMVLVYKTPKAASPQPATMRERILQMDLVGTFLIMAAVVCFILAFQW

GGSFKEWSDSTVIGTIVGFVLISALFVANELYMGDRAILEPRLMRIRRVWSNCAHVFFVS

GGFFIFIYYLPIYFQSVQGASPIASGVRNLPINIGCFLSIAAGFAVSVYGRSWAPLMATG

SAIATVGAGLMYTFGLDTSAGKYVGYQLVTGMGMGMTLQIPLMANQAVVQPMDISAVSAI

TLFFQIIGGAFSVACAQAAFASTLVRRVAVHAPGVDAKQLLHLGATQLRDEFSGEVLQGV

LKAYMDGLRVAFAIAIALLGVGFLFTLVPKWNDFRPNQQQRKPSDDEKDSSDAA

>SoG_01014.T1

MAEMTHTSSMSSFQDVKMDHDIESGYASAQSDYSPSSSTASLPLVTLTKAHVEHLNRQLE

PMHPMDILRFCKVLFPNLYQSTAFGLTGLATMDMLSKIQAETPDAPTVDLIFLDTLYHFK

ETYDLIDRVRERYPNVPIHIFKPDGAETAEQLEETYGAELYHTAAEMYDWIVKVEPLQRA

YQQLKVTAVLNGRRRSQGAARGSIPIIELDDERGIIKINPMATWSFKQVNDYIKENNVPY

NALLDQGYKSIGDWHSTVPVKDGEDERAGRWKGQDKTECGIHNKKSRYAQFLLDMENKKR

KEELDDASRSLGQIVQPV

>SoG_01024.T1

MDTLGALGGEWEPSERSFLNLLKGALEYSVSTEARATKIANDVLHFYMDNTPEGEVGGML

FELWGVWIEVAYRIPHGHEWHRCMALAVDLIRQDQRASDRGSKASREHDELPELFMSVRE

RWEYKPFEGAAHDHGEWKNLNSFMSLVVGSGFVQQIHLPLWEISLALEEPSSKELALMDY

RVWVAAEWIINCREVLKGEMRKPDEELTVESREILGIGTLCDDSIKPRSWQRWEFWGRQL

AEILEESDDIGLEEETKKHVEKALEVVRSTXPLAATLTEHTERHNFQAFCCPAAGPPAAP

QTWNRPRSRFVELIATSAQALASLLVPASSSIPSPPCFFSLLYFGAIYARLTHAGTSLIS

NTIIDSPCDRAQPGASEGAILTTSLRTPSDINVCRGLSTSNMPELSPRGVRFSAGEEDMR

VERLKDPRLSTVALESEDSSPESNGDPNANDQDDDLGGMDRYVDASRHMGSISSLTPSAS

LNNVNKSQRSNGGDDGTSFNGAWPQRPLGPTRTPSSTYNPGSSRKPAPPQSAPSFVDSMR

SSSKTRPRQSESSRFRAQERAYIQKLRQDYSGEYFASYETTNGNDSDSEGETPSSEGPFD

DRYDEETIMFYGNETLQPTEEDLQNKENRERLEWHGMLAAVLMGNVVTQEKQRLHGSSDK

EVGKTAHKTELWVGVRAKVCGRRLPVQKRMVEDARREVDRMLDDIINFSVKGAREVGKPP

YEQVKDIVGKIEKCESLYPSWKSLKAEHKAADSAPFNEAYDSILSWYNTNEMINTELAIL

KKWVGNDEMDFSRTKQRSPAVHGISSDETSFLDRLMKEDGLRSLYNDDEKSTQKGMLRPI

SAIISKAKQTLIKNSGPFEKRHLPPYLEELLTLISFPSRLIEEITRTRLAYARRVKESAQ

QNPLMQDQMISQFQLLLRFAIRIKAEYLAIAAPEPGWELPPLIDESFDQVILDALKYFFK

MLNWKLSGNRNTFKEAELLFEQWGFANEIGGHLLRGEIEVAEQFSSLTFKALNRLSQAFE

RELQAKPKETAADMSKRYKACLDSVRVRHRMLQRFSRNLSEQYEHCCDFSINFPLDEMQR

IYDQLLTTGHFHVTTGVFEQDGVYILASPGLRERLDDIQALMAVTSKETFDPELSDQYLL

ILRPEEPLTWCGPSISLPLREQNIDLKRGQVRLCATGSTALPISRRIFLDAVDTHIDLIQ

EHRSNIHKVNTRLTETRRVAYKLSNTFMDSVEIVRKQAEGKDCQELVQTCFVFATEFGQR

SLQSMDTNRRQMNNIKLTKLALDWISFIVDDCVASDRKTFRWAVQALEFAMRMTMGRHIL

ALGEDDYALMRDKVAGCMGLLISHFDIMGARSTLAAQAEKEKLEAMVGHFKRMDHNGMLL

DDAEASKSITEQRMDSIELVDGFRREKEAERQALGRVLEANTEADRSLAYLSSSATNITM

RWQQGHYVGGGTFGSVYAAMNLDSGHLMAVKEIRLQDPKLIPTVAEQIREEMGVLEVLDH

PNVVSYYGIEVHRDRVYIFMEFCSGGSLASLLEHGRIEDEQVIMVYALQLLEGLAYLHES

GIAHRDIKPENVLLDHNGIIKYVDFGAAKVIARQGRTLVSDLTSTKPNKSMTGTPMYMSP

EVIKGENPGHPGSVDIWSLGCVILEMATGRRPWASLDNEWAIMYNIAQGNPPQLPTHDQL

SASGIDFLKKCFARDPRKRSSAVELLQHDWIKAIRSQVVEPATPSDSSSSVQSTPMGSWP

SSRGSAGPDGFY

>SoG_01043.T1

MAEANGIPTPKRSASPLQPSEIPEAKRSRLHQGTESQSQQTTALTNGEVDTLLADQAVES

SALTQHHQIRCTIQRSIALVLKHDGFQSATPEAMESFTSLVEAYMESLIEETTRFAHASR

RDRPIPTDFESMLRLHNLPISSLKPHLKNPLSQSQLAPKWDLINPQDEDAYTTLPLLGEE

LSGQPDKDSMQHVPSFFPDFPSRHTYRFTPQEDVNTRDSQKIREEAARTAQQGEDALRKL

VRASKMRKQKEVKTLVEKDVQGKERFRLWEATMRRFMGADRRSDAGEQVEIADHSMIVNA

DAIFARKEVSRFGKRAAAGAGVKVGKA

>SoG_01044.T1

MAEQLILKGTLEGHNGWVTSLATSMENPNMLLSASRDKTLIIWNLTRDESQYGYPKRSLH

GHSHIVSDCVISSDGAYALSASWDKTLRLWELASGTTTRRFVGHNSDVLSVSFSADNRQI

VSGSRDRTIKLWNTLGDCKYTISDKGHTEWVSCVRFSPNPQNPVIVSSGWDKLVKVWELN

TCKLQTDHIGHTGYINTVTISPDGSLCASGGKDGTTMLWDLNESKHLYSLNANDEIHALV

FSPNRYWLCAATASSIIIFDLEKKSKVDELKPEFPAAGKKSREPECVSLAWSADGQTLFA

GYTDNIIRAWGVMSRAKSREEENCARLEDGGKDEERSGLW

>SoG_01050.T1

MDGKRHPSSFQQLEKLGEGTYATVFKGRNRQTGELVALKEIHLDSEEGTPSTAIREISLM

KELKHENIVALHDVIHTENKLMLVFEYMDGDLKRYMDTNGERGALKPATIKSFMYQLLKG

IDFCHQNRVLHRDLKPQNLLINSKGLLKLGDFGLARAFGIPVNTFSNEVVTLWYRAPDVL

LGSRTYNTSIDIWSAGCIMAEMYTGRPLFPGTTNEDQIIRIFRIMGTPTERTWPGITQFP

EYKPTFQMYATQDLRSILHAIDHTGIDLLQRMLQLRPELRISAADALQHPWFSDIHMAQQ

AQQQQAQAMQARAYQQQGVPAQNFDGY

>SoG_01059.T1

MPPSARRTQRARRDVRKEASTSSDAVADSSPSRPAKRRKKAQDEDPAPLPDDDQLVTQIT

QQLKTQPVQASKDHANAIHEANGDGVKAYAKVAAQDWTYYITRLLVNIGRAQEQGHPDEG

DEDQVHIDLGPGKMVSREHAAISFDSKHEQWILNIKGRNGAKVDGVPLKASTPHVLSSGE

VIEIGGVEMMFVLPSEISPLHVDPAFLQRCGLSTDTPQARKVRRAPPVAPTPPQLDRPGT

PSQDQTPSRVGKSPGMMSTPAVMMGANGIDLSSDENQHIKPQYSYAQMITQAILSEDDGK

LNLNGIYQFIMNSYAYYRHQQTAGWQNSIRHNLSLNKAFEKVPRLTDEAGKGLKWQIVPE

AKDDMVRNAYRVGRGGHRGSSAPSSPNQLNYITHGPRDMAARGPGSARKRRTSPLASPSP

RPPFNAGQSTPERPSARALGRNAGLAVDGSPLPRNQKNTPAASYSSFNPQSPTLTSSYLQ

DDNTSFVTPAPPRVHPKLAPPSTAQRPSQHMPTSSPAPFWKYADIGSTPLKPITTYELSP

SKMDGPAPPPSSSPPPPAQKSLPSSPSKPQKVMTEAEPEADEEQGFDLTKGFQSIGAYHA

PVGRGMPVPNALQSRT

>SoG_01068.T1

MAMTTAHMSLSDAHYDETDSLVSRNSPYMTPIHPKLELSPSPSLDLPVAKVRANPGNAVL

VSYLGNGRNADVVLAASNEIEEDDEYEDCDNDGYSTANESTTCASQFRDDRTGADLPKSD

LRNAVASPELQLLATESLKAVAADPAPSSSIPRLTADLSASTRHLSIRDDLPPKIMHPSP

DSVSSPTASSTSESYFRRRSIIPAAGVMGDGALPPLQIDYPKSDLSVRSLPSITAQLGDL

KNLSTERTTYAESDVCSPRSHVSVYPGSPQARLPPISANHTSPPLSPHDSYPRTIGSPTS

LSTHSSQYHYSSNGTWPSRSNPDYSSSGTGETPNTEQSASTPATSATSVADLMSLDNISN

PMGPGYPCTEPGCNAPPFQTQYLLNSHANVHSSARPHYCPVPGCPRGETGKGFKRKNEMI

RHGLVHDSPGYVCPFCPEREHKYPRPDNLQRHVRVHHVDKDRDDPLLRQVLAQRADGPSR

GRRRRGATS

>SoG_01085.T1

MPPPKCHGDEPVTSVTGERSTVRLVLLAESAVYLCAHNPEGPEQEELEEALQAAQQGNTT

LTAASRFERSPLPDAATTRGRNLALSHSVVSPQATANSPLSNPAIASPHDCPVEPSARLS

RCQLGSNWYFKGVGILSSRGREWISDGAGQRSFLERFHVFECPVHIPPARASWAWPEQPM

LLPAESISRRLFSAFLDTNTYAMFPILHKDLFESTISRAYAVTASESALRASAEACVWAV

LALAGCTDRFQQITLIPQPEQCIQQATRLSALTSHSSSLDNLQTILLVWVYQKTKGRCQE

ASTTLAKACRMVCDLGGHFHLSNLTMLPQHIPIIDEPGRQHIRQLFWTCYCFDKDASLRT

GSPPLLTSDLCDMTSCTSHNRVDASCCHGCRDISLAKIKEHAIQVLCSPSAFRYTDGELA

AYVRQLDDELEEWRMSIAPPYRPRLSIPPDSTLTMPLAASVRDRTHMIKLQLDYLFTMIN

IHFLVRKCGNFERNLPDDLHSVVHSSADLSLEASRSIFHFLDTAVDSWKEDSVWIVSHYT

LIAAMPLFINILIHPVGFPADKDLQILSSVGSIARRVPTDRLFKDDIEQVQEVVEFVMEL

VRLGHSAAWKAKKGERELDLDIIYREEPDAA

>SoG_01086.T1

MSNNLLDPKALLSPRQRAPSEATSTLSAEQSFITGTTTVTATPSFVDVDLKQALKPDPGT

EADFNVPDNPYDFSPGHLNKLLNPKSLDAFRALGGLQGIAKGLQVDIKSGLSVDEVDANV

RTSHGSKAPRSRVFGRNQLPPRKPKSIWRLAWITFQEAVLVLLTVAGVISLALGLYKTFG

VAKKEGEGAPIDWVEGVAILAAVIIVVVVASHNDWQKEKAFVKLNTKRDDRDVKVLRSGK

SMLVNVNDLLVGDVLYLEPGDVVPVDGIFIGGHNVTCDESSATGESDALLKTGGAEVFQA

SQSKNVDHLDPFIISGSRVTEGMGTFLCTSVGTHSSFGKIMMSVRSDIESTPLQKKLEGL

AVAIAKLGGGASVLMFFILLFRFCANLPDDHRSPEDKASTFVDLLVVAIAIIAVAVPEGL

PLAVTLALAFATSRLLKENNLVRVLRACETMGNATCICSDKTGTLTTNKMTVVAGCFGAS

SFTTDVSAWATKLSEKMKKILTQSVAVNSTAFEGEQDNVTTFIGSKTETAMLQFAKDHLG

MQAVSEVRANEQVANIMPFDSARKYMATVIQIPTGYRVLVKGAAEIVLGFCSNQIDAATG

GIEPVDRQRTEEAINEYASKSLRTISLAYKDCDAMPDLKELDRMTLIGVVGIQDPVRPGV

PQAVQNARRAGITTRMVTGDNVITARAIATECGILTDGIIMEGAEFRRLKEDELDKTIPR

LQVLARSSPEDKRILVMRLKAMGETVAVTGDGTNDAPALKAADVGFSMGISGTEVAKEAS

EIILMDDNFTSIVTALKWGRAVNDAVQKFLQFQITVNITAVILSFVTSMYNDNMEPVLKA

VQLLWINLIMDTMAALALATDPPTDAILDRPPQPKSAPLITMNMWKMIIGQSIFQLVVVL

VLYFAGDEILGYNSNIPSEKLQLDTIIFNMFVWMQIFNELNCRRLDNKFNVFAGVHRNWF

FIFINLIMVGLQVAIICVGNRVFHIDPDGLDGPQWAISILVAAFSLPWGILVRLFPDRWF

AAIVHFIAPPFVISYRFMGRGFSRFGRLFRRKASSTAESAVTDGEKGEGSNHVVAPIILS

PPSEKA

>SoG_01108.T1

MSFSHGSEGGTLALPSPTHAHHMDVSSAVRTLRRSMSRSPSTKYLSRSNSNSSDGTTSQQ

ISPQSPCRRFGLTPHRRASPSAHAQTAPPAAIYPPATNASQSPSFTPLRPSGRLSLRSVK

SAKTSTPQRPLSRLRASPKSPLRRALNTNSDSGNSSLSTVFSAIAPTTGQENVASFARSP

PRRRNLEKPSRHSLHLDVSGVSQHAFLKALDANKSPSMTSTGALKRSDATMNLDQPNQGS

PVAKRRSLHGFSTLVHGDDFIAASPGTVPAAPSFEIHEDANTEYELTTAAEAPRSDFAQS

ASAGITVSQRSSSLRKTTLQQRSLSRRSGERHLAQLAAEQASPARSRPRLSSDHFLPPTV

PRDSPFNSKIPLPSTSNHSIEASKPHPLSKTLTTSSSGGSLEEEQPPFYASFKSFEAPKP

HPFSKSLPVGATRPSAPKPLHERVVATPNGSGQLWLGAFNSTGLVSKVNRNLEEEAAKKI

IPPDTPCKKHSNPFATYPPKFSSVLKKRENNRKSFGGIPSTPFSSTPGPAPDTFGKPGKG

LSIFQRGNALRNSRRGSILGLDDEDRKLLGETSDSGCTGDSDVPPTPTRNTLTPSGSNLS

QQSLESPSANRTQAAPMSAVRPPISRESTSKSTPGMFEVPADADGVPTGENVPASNGLGQ

ATVPSMLPPTSFGNSRAQQGCRVPAPILSVTTFSHSVASDAKTTLANPASPVDGRRTPQT

PQESYLPWDTSRLSISQAANGVPAESMPPPMTPTGGRDLRSSTSIFVTPANARTQNLDID

SSLTGRFDKVEYIGKGEFSSVFRVSVTNHRQNALDALSGTEAPPSSAQGSIFAVKKSRYP

FQGPKDREAKLREVRALQALTHAEHVVQYVDSWEHNYHLYIQTEFCEEGTLDKFLGNVGR

GGRLDDFRIFKMLQDLCLGLKEIHDAGFMHLDMKPANILITFEGALKIGDFGLAQPVTSS

EGVDVEGDREYMAPEMLKGNVSGSADIFSLGLITLEAAANVVLPDNGPTWIALRSGDLSE

VPSLTWTPSVESRRTGHPTDSGLSDELGAVRTHDGGNLFGSLKRSELQQPPDFMVDPTHP

SSLDSIVRWMTAQEPSQRPLADQILELEGLRWIAEHRSAPATVYEGSWGPAETMPVSIML

DSDSEMIDV

>SoG_01110.T1

MLSPPEAASHSSLQLRSHHFCLAFSLRHVSQHFSTKLDVMELRFDSHLFHREPRWLLTPP

PRRTPRRVTFSDQVDMIGSSFLPPRFRGEPTVSKPVAPTWMSKRVNPFLQFLARLACSHP

IHTVVVVAVLASTSYVGIIQERLRESTASISGKADWYALLDGSRSLQIAPETAWKWQNVE

DPKAAAQGADIEHLALLTLVFPDTLSNDSPSNAPRSHAVPIPQNLSITQLPPTENPLTAY

SQDSILAYSLPYADAPAFVANTQEIPNEDVKEKDFTRGLEQKMWIMKAARVNTKSTIVKW

VKNTWAEFLDLLKNAETLDIVIMVLGYISMHLTFVSLFLSMRRMGSNFWLAMSTLFSSVF

AFLFGLAVTTRLGVPVSVILLSEGLPFLVVTIGFEKNIVLTRAVLSHAIEHRRNQIAQKK

SQKSAESSNQNLIQYAVQAAIKDKGYEIIRDYAVEIVILVLGALSGVQGGLQQFCFLAAW

ILFFDCILLFTFYTAILSIKLEINRIKRHVDMRMALEDDGVSRRVAENVASSNDDQDSKE

TSLFGRKMKSSNVPKFKILMVTGFVLLNLINLVTIPFRGSSSLRSWAAGLGGVVSTPPVD

PFKVASNGLDTILATARANSQKTLVTILTPIKYELEYPSVHYALKSSTGGPGVDGPPYHP

LNDYGVGGRMVGSLLTSLEDPILSKWIIVALALSVGLNGYLFNVARWGIKDPNVPEHNID

RKELANAENFNDTGNATLPLGEFVPASPPPRADLATPAITDDEDDASKGAKFIPSSTEKR

SFAELEKMLMEKRVPEMNDEEIIAMSMRGKIPGYALEKTLRDFTRAVKIRRSIISRTKVT

SEITSNLERSKLPYENYDWERVFGACCENVIGYLPLPVGVAGPLVIDGQSYFIPMATTEG

VLVASASRGCKAINSGGGAVTVLTADGMTRGPCMSFETLERAGAAKLWLDSEAGHSVMKK

AFDSTSRFARLQTMKTALAGTNLFIRFKTTTGDAMGMNMISKGVEHALNVMATEAGFDDM

VIISVSGNYCIDKKPAALNWIDGRGKSVVAEAIIPADVVKGVLKSDVDSLVELNVTKNLI

GSAMAGSIGGFNAHAANIVAAIFLATGQDPAQVVESANCITTMRNLHGSLQISVSMPSLE

VGTLGGGTILEPQGAMLDMLGVRGPHPTNPGDNARRLARVIAAAVLAGELSLCSALAAGH

LVKAHMQHNRSAAPSRSTTPAPQASMTPVSLAMSNAQDKPSSAATQRSKR

>SoG_01134.T1

MHARDATGRKVSLLNEEGHASAPSRPVPYSRPMSFSAVNHTLTQPYPGSRSSSSSPNTPE

LMRSDSYDSHMSNDAISPLTPSLDFTYPRTSVYPLGGEYSHESQPLKRPAYADSSRSNSY

EDDSSVTSPLPERSGKRYPCRYRETHGCEKTFTTSGHASRHSKIHTAEKAVQCTFPGCQK

KFTRADNMKQHLETHYKDKSRSSGQRAQKAAALADARRNSSTGRIRTTIARDSSQWESTE

QYPLPTPPLPSPNSTAAWDMRAMNLPLLNRPVAARTPSSGLDALAMAIACQEGGR

>SoG_01136.T1

MATRIDRRDDAASHDLVRGVILGWKSSIAALNVLEIFTCSDGYVEGSLQYHPLPKFLASA

SASASTSTRGIAALAELMPRNRPVGVSTTRRRNRGFLGSDSSALSAEDAQDRNQLRYELD

LNSWNLRIWGVAASGFLTDSYNLFSTNVILASVGFVYWPHGGAWRGLLINFFTLFGSVLG

QLSFGFLADWFGRTRLYGIELVLVIVSTIGVATSSRGYNDMDFLALFTWWRFVMGIGIGA

EYPLSAVITSEWSSTKSRATMISSVFMMQPIGQALAQLVGLWVLLGFEDMKNLKEMRCGL

DLKNEEKCREAVDGIWRIVIGSGAAPALLAIIFRFFLYDCGLYTLEVRNKPGIAIEDTQR

VYGQPSAQTTGFRFGTPSAVELTNSHPAPAASSHFPHENGSPSGVDHDSAPRPSPIQFSK

EDLYNYFIRDGNWTYLLGTSATWFFLDVSFYGLSLDNRLTLSDLWATTSPTPIDSRLECW

NSPLEGGVSAVPNWVKDGLPVWQTDSTHPCNTIYDVLIEQTKQYLLTVSLASIAGSACFV

MCANRIPRRLWLTASFLVLSLMFMVTGGVFYAVHREKAAPATVVFVALCHFLFNFGANTL

TFMIPAEIFPTCYRCTCHGISAASGKLGSLVAVLVVYGINSTWSSVTRQGTIFIIFGSFA

GVGAIFSWAYLPDSQRWVEEEEGKRFLETKDLEELGEGRVRARQGGETITLGEKWAEIKR

KRREAARQVQGQERDSSPTPLLASVIASCFHSSLIRRRRPVLPVHLGMTNPGGGFSARTP

LVSSYSSFPLTVLAIAFSSMRASSQALHLADGWICSGSSGPGLADASPGSPPGGEEAAAP

GGEEEEEEEEGPRSFSVLRRRANGAK

>SoG_01141.T1

MAKYLDTIYVSGWQSSSTASSSDEPGPDLADYPYTTVPNKVGHLFMAQLFHDRKQRQERL

SVSKAERAKMQNVDYLRPLVADADTGHGGLTAVMKLTKLFIEKGAAGIHIEDQAPGTKKC

GHMAGKVLVPISEHINRLVAIRAQADIMGTDLVAIARTDAEAATLITTTIDPRDHAFILG

CTNPNLPALNDLMMAASAEGKTGAELQRIEDEWLASANLQRFDEAVLAAIDTSGSGNKDS

LKKSYKEQAKGKSNKEARDVARKILGKDVFFDWESPRTREGYYRLKGGCDCAINRAIAYA

PYADAIWMESKLPDYAQAKEFADGVHAVWPEQKLAYNLSPSFNWKTAMPRDEQETYIRRL

ASLGYCWQFITLAGLHTTALISDRFAGAYAKQGMRAYGELVQEPEMELGVDVVRHQKWSG

ANYVDELQKMVTGGISSTSAMGKGVTEDQFK

>SoG_01142.T1

MAPRGFDDEELTISLSSSHLRRQQQQQQQQEEARRAREEGSRQPPMPTIADGVAMDAPPA

TERVKAKTEQRIDAYKVIRTLGEGSFGKVKLAVHTSTGQQVALKIIARKKLISRDMAGRV

EREIEYLQLLRHPHIIKLYTVIKTNTDIIMVLEYAGGELFDYIVANGRMKEGEARRFFQQ

MICAVEYCHRHKIVHRDLKPENLLLDDNLNVKIADFGLSNIMTDGNFLKTSCGSPNYAAP

EVIGGKLYAGPEVDVWSCGVILYVLLVGRLPFDDEHIPSLFAKIARGTYSIPSWMPAGAV

NLIKKMLVVNPVQRATIEDIRQDPWFMTDLPPYLQPPVEEFFNTGMDPAKAIEKSDIAPH

ASEVVQERLHNEVTEKISKTMGYGKGDVEEALKAEEPSAIKDAYLIVRENKLMQVSQNPD

ALIVDEPEGAGSSPLMSLSSARSGMSQIPGQVRPYVSKVGILPSSIPTYHKDYVERLKSG

ILESHSGTDNPDEEPAPARTEAEKEEVSRRLKPHTRSQIRLDDAAKPQSMTPVTPKKNKP

VRWQFGIRSRNAPWEALLTIHKALHKLGATYLPDEDFELAHRKEPEGSRSGDSSFVREHD

GGPVGGGSTASLDSTQGYKLPADPWHIKVRWDSSTIRKHSQSPSQGGEKDKYGTPDSFHV

YSPEEASKKSPFVAMHLDIQIYEMEHGVYLVDFKCSGYETSQGLLLEEKDVTSPFPFLDL

AAKLIMQLAEAD

>SoG_01146.T1

MSDAFAPRSMKRKNVKGLALTPAAPKPPPTAETSFVDPGESNRDEQLEIGIEYKLDLRPE

DLEIVKELGSGNGGTVSKVKHLTTGTIMARKVIHVEAKKEMRKRIVRELQIMHGCHSDYI

VTFYGAFLNYNNDVIMCMEYMDVGSLDRVSRVFGPVRVDVLGKIAEATLGGLTYLYTKHH

IMHRDIKPSNVLVNSRGSIKLCDFGVSGELINSIADTFVGTSTYMAPERIQGEKYTVKSD

VWSFGLTIMELAIGKFPFGASEALSDGDGAPAGILDLLQQIVHEPAPKLPKSDAFPSILD

DMIQKCLYKEPEARPTPQELFDRDPFVQAAKRTPVDLREWAVGLMERDNRKSHLAPQLSP

STQELLRSGDSPIFPPQHEERSLQTPTSGEIPIAGTGFMSPSEALGHGSRSPTRNGSGRT

PITPHPGLGQRSTTASSIPKASIYPTSAGPTPNSASAATFSLPMRPGPPGGPLPPPPPRK

ETPDESRREYRR

>SoG_01147.T1

MSSMRDLIDNQAELDSEEEDESFDDEAGDRPRKDRDGRIEDSSDEEEDDDDEEEARKIRE

GFIVDEDEDEEDGGESDGDARPVPKRKREHRDREEEAQLDEDDLDLIGEQFGERPKPQSQ

SKFKRLKRGHRDEEDLNRGPRGLEDIFSDEEEEGGDRYSRPNARAAVDEFDGFIEADFSD

DEDERRRQREDAEVARPRDRGMGGVVDTSGLDKDALEDMEAIFGDGEDYAWALNLEDDEE

QAQHEEQAIELKDVFEPAQLKEKLLTDEDNAIRFTDEPERFQLDRKAFKNLQLTADQFRE

EGKWISNQLWPKKGLAADLQQPFHKAVSKVLEFFIVDEVEVPYVFQHRKDYLLHNRKSRS

GRDEDGGDYHDEPAKLLNQDDLWKILELDIKFRSFVEKRNALEKTYDNLKSLDVQDSMVE

EMIPEATAMEELQDLQDYLQFRYHKELKELALTNGDSHHAKRAGGKSALLDHIRSSRAAF

FVKAYGLTADQLAKNALRQGKKVSPDDESQYPMDLADSLTDDMFSTGDEVISAARQLFAE

ELYMSPRMRKYFRNSYYQAAEISCRRTDKGLKRIDESHPYYQIKYLQNQAIADFVHRPEL

FLQMMRAEEEDLIEIQIGMPRQLNFCRQLYQEFESENFSDRAEQWRDERKKVVDIAYSRL

EKIIAKNVKEVIRTFCQDEVLKTCREEYAKRLDQAPYKPKGMVLGTLPRVLVFSNGMGDP

TREPIMWTWVDEEGRMLEQGRIAKLDRDGYWKDEVAKAVERFQPDVIGVSGWSAHSHRLV

HALDDLVVEKELKCAEFEDMENGGYNTEPLEVVVVNDEVARLYKDSPRAAAEYPSLNPVT

KYCVALARYMQNPMKEYAAIGNDVISLLWHPCQSLLPQDRLAKYLESAMVDMVNLCGVDI

NLAMTDSYTANLLPYVAGLGPRKTTSVIKAISANGGSVNTRDELVGDPDSNKLPVVGPRV

WNNCASFLFIDYEAGNPSSDPLDNTRVHPEDYELGRKMAADALELDEEDVKAETDEHGAG

AIVQKLFRQGEQDKVNELVLEEYAEQLLRNFSQRKRATLETIRAELQAPYEELRRHFAIL

TNSEVFTMFTGETKDTLCEGMIVPVNVRMVKDDFAIVKLDCGIEGRIESHEVDANGGLRD

LLSQGQTLQAKIKVMNYKDFMANLSLREEDLRIPYRRPANYGADGWNYAQEAADKDELRV

KDQKTGRTQRVVKHPNFRPYNSTQAEEYLGSQPPGEVIIRPSSKGNDHLAVTWKVADAVF

QHIDVLEMQKENEFAVGKLLRVGKYTYSDLDELIAEHVKAMARKVEEMMRHDKFQNRSRS

ETEKWLATYVEANPTRSAYAFCLDTKHPGYFWLNFKANKDVQTLALPVRVIPQGYELKGC

QYPDMLGQEELRRFFRANDDEASEAAAAAASGGGISNKLS

>SoG_01150.T1

MSGQFSSPFGANANPFGTPSAAPTNTIVEEEENDTVGTPGEARFGTGSQPGFSGPFGGGD

ASADTPPTMRGPPDPSSYPAQYNFNRRTSVSAESLKPSGDVSDNWSPPFAEKSAEQLQRL

KKAVEGNFLFSHLEDEQTQKILGALVEKPIPAKGIKVRTAISGTQHPCKYVTDISNQVIS

QGDAGDYFYVVEKGSFEVYVNSTGSVQPGPDGMGEKVGTIEAGGSFGELALMYNAPRAAT

IVSAEAGCMLWALDRMTFRRILMESTFARRRMYESFLEDVPLLSSLTPYERSKIADALET

QKFAAGEEIIKEGDPGQSFYLLESGEADAVKGDAKVLHYKKGDFFGELALLNDAPRAASV

VAASDVKVATLGKNAFQRLLGPVEGILRRTKYEGVKSGVEEMDPLHTG

>SoG_01153.T1

MRDTQDDAQVGLHASQYCLPLRGSQDGSDADLVTLSKPMNAGRSKRFPEHLHLSQGVIRR

LRSPTESSMSTLSSISSSTHVPDSATTCSLSTLASPISTSYASEVIEQFAKHTINSSRAS

SRILHRHRGSNATCSTFVNDEEEALYLSRRKELDLDIASIHSTDRVAKPTEQESHPLSDA

TVEELPSLNIPRTARPVSSPRPRSSDAWETTSTASDDFSLDDSDAVLDYTLQLVYGIDSN

ENPIPRSRLQRLTRDFLSGIGNAMREECEDAEQVPHRSSTGFSSSTITDGNGGDDGQKGE

KRKKLDQSDKAGDEFSDHEGYNPLPNKRMKPTPNDDNLRLSCPFRKRNPHRFNVRDHHSC

AMTYFPKFAELRQHIVKQHKRDDPSAFVCDRCNTDFPSRKGLREHQRLPKEEMCDIAEHN

PESGIDGPTSNKLLSRKRASGASPYVQWREIWNILFPDDDDSAIQPYDFTPVIEHFEISE

NFERSFKFLQGSLRDMMENPATLETLAAKFHQCFIETVGQCILNAQKMPYTNRSNKRSEP

SRLSHSITAAKRGSSRPDSGIIIDDGSEESGSIVNTALRHNSVRTVRSAQRDSTRTVGPR

EILPSSQSIFTGLDETLLEQLSKGEGNSVGYGGTASMDPVSVQAWNNAVLSQQMTPQSMD

SLQPGPYSTMADMSGSGDMVYPEDAFYQMFGAIPAGFPGFAGQPRE

>SoG_01155.T1

MSTSVPHPLDPLSGAEIQTAIALVKKTYGDGFFNVVSLHEPRKAEMLAWMESPATAPRPA

RVADVIVIQKGGKVYDGLVELAGPKITKWELIEGAQPIITMEELQLVEHICRSDPKVIEQ

CVISGIPKEDMHKVYCDPWTIGYDERHGSNIRLQQALMYYRPDPDTCQYSYPLDFCPIFD

ADKQAIIAIDIPKIRRPLSKVEPIDYTPRGVEKRGGYRKDLKPINITQPEGVSFKLVGRE

LQWQNWKFHIGFNYREGIVLNNIRYTERGTDRPIFYRLSIAEMVVPYGNPEHPHQRKHAF

DLGEYGAGYMTNSLALGCDCKGSIHYLDAEFPTRDGNIKTIKNAICIHEEDAGILFKHTD

FRDDSVTVTRARKLIVQQIFTAANYEYAVQWIFHQDGTIQPEIKLTGILNTYALNPDEDP

GPWGTCVYPQVNAHNHQHLFSLRVNPMIDGTNNSVFMVDAVASDAPVGSKDNFYGNAFYA

KKTKLNTEGTSRTDYNGATSRTWEMCNESKLHPYSKKPASYKLVSREVPGLLPKEGSLVW

KRAGFARHAVHVTKYRDDELWPAGRHVPQTSGEPSRGIPEWIGDGTTSTENTDIVLWHTF

GVTHMPAPEDFPVMPVEPITLLLRPRNFFSNNPVMDVPPSYSSTPSQVAGTASGVVNAAD

KQSTYAFAESAGRDCCSKL

>SoG_01158.T1

MGGLPEDVEQSASVAVPARQNAQGAVTSTANDALPTPTEDHIPASRAVRDLKSPGVARVE

AISSAITFTDRIFIFLGIFLVAYVYGLDGTLRYTYQTYATSDFGQHSLLSTVNTVRAVIA

AAAQPTAAKIADVFGRVELICLSIFFYVLGTIVEAVATNVKTFAAGAIIYQVGYTMIILL

LEIIIADITSTRSRLFFSYVPALPFIINTWVSGNIAEETINNTTWHWGIGMFAIIYPVCA

IPLILSLLVVSYRAKKNGLLDNWRSSFQIFGVSNVFLELFWVLDVIGIILVIAVFALILV

PINIANGSPDSWKEAHIIAPLVVGICCIPAFILWEFRAPHPLVPFHLMKDRSVWAPMGIA

CLLNFAWGMQADFLYTVLIVGFDFSIKTATRITSLYSFTSVIVGPLLGVLVYHVRRLKIF

IVCGTALFMVAFGLMIKYRGSASGAGKAGVIAAQVVLGVAGGMFPYPAQASLQVFLRHEN

LAIMTALYLATYNIGSALGNAVSGAMWRQILPGKLDQGLGNINATLATAVFANPLSAADT

YPMGTPERVAIIDAIQQVQRYLCITGISLCVPLIVFAICMRNPKLNEKQTLAKEQETDSE

TEADPDAKLSAERA

>SoG_01179.T1

MPASHQLPHDDSPRPPVDQGYDAVTPAIPSGRTDYNFETTTDLPSYFSRADPLISRNDPD

DLPLLRQDGQNPYSTSLPGPMARRESLSDIRASNPDLALTGNIISATFTIPHSFVYRKGG

SWDLSLRRGQSALFDSLSHLSSDATPWNHTVVAWTGEIESPSDVPLSPPTTPGNTSSSAS

SHNPLSAPVPVDAESKLPTPPPSDGLWLPKADMENLEYQLSHSKTIRTVPVWLADEDEIF

DDGMKLKDQARWRRYAEHDLYTLFHYKQHEPTDGRAERLEWADYYRMNQKFANRIMEIYK

PGDIVIVHDYYLMLLPSMLRQRIPNIYISFFLHCPFPSSEFLRCLSRRKEVLEGMLGANL

IGFQSYSYSRHFLSCCTRILGFPSDSLGIDAYGSRIQVAVFPIGIDAAKVLSLASADKVT

EKYNALKAMYKDKKIIVGRDRLDSVRGVAQKLQAFERFLEMYPEWRDKVVLIQVTSPTSV

EAEKGDAETKIASRVNELVMKINGQYGSLGFSPVQHYPQYLDQNEYFALLRAADIGLITS

VRDGMNTTSLEYIVCQKDGHGPLILSEFSGTAGSLSDAIHINPWDLSGVAEKLNSALTMS

DERRMAMQSSLYRHVTTHTVQSWINKFIQKTYTVLSESKSTNATPLLDRSQLLQRYRAAR

KRLFMFDYDGTLTPIVREPSAAVPSERLVHVLQILASDPKNAVWIISGRDQDFLHQHLGH

IKELGFSAEHGSFMRHPTEEEWENLAEKLDMGWQEEVIAVFQKYTDKVPGAFIERKRCAV

TWHYRLADPEQGIHMSRECHKELETTIGLKWDVDVMPGKANLEVRPTFINKGEMAKRLVH

FYNDQADETPIEFAMCLGDDFTDEDMFRSLNGLSGSVLKDEHVFTVTVGASTKVTLAKWH

LLEPADVIECMALLAGAGGEGQKEWLGEVNLAAVSAVEGHIPEEEKLQME

>SoG_01189.T1

MGACMSSNTEEMEQKKKSQAIDRILEEDSKKLRRECKILLLVRPAGSGESGKSTIVKQMK

IIHLKGYSEDELYNYRATVFKNLVECAKAVVTAMQQLEIEPANEDNKRYAQYLLDYQTES

GPQAHIDPEVGIAVQSIWSDPAKDQLMEHQTEFYLMDSAEYFFQEVSRIVAPDYLPNEMD

VLRARTKTTGIYETRFQMGQLSMHMFDVGGQRSERKKWIHCFENVTSIIFCVALSEYDQV

LLEESSQNRMMESLLLFDSVVNSRWFVRTSIILFLNKVDIFKQKLGRSPLGNYFPDYSGG

DDVNKAAKYLLWRFNQVNRAHLNLYPHLTQATDTSNIRLVFAAVKETILNNALKDSGIL

>SoG_01190.T1

MASLPTRERRPSSGAPIVDIQGAIGPAGISRPKHKRTVTGLAPGEIKNVEASIPEPQREA

WKRSQIKAFETPEDFQKEVVRHVETSLARSMFNCDEYAAYAATSLSFRDKLVLDWNQTQQ

RQTFRDPKRVYYLSLEFLMGRTLDNAMLNVGLKDTAKAGLNELGFRIEDVITQEHDAALG

NGGLGRLAACFLDSLASLDFPAWGYGLRYRYGIFKQEIIDGYQVEVPDYWLDFNPWEFPR

HDVTVDIQFFGSVRKDVNAEGKTVSLWEGGEVVRAVAYDVPIPGYDTPTTNNLRLWSSKA

SHGEFDFQKFNSGDYESSVADQQRAETISAVLYPNDNLERGKELRLKQQYFWVAASLYDI

VRRFKKAKRPWSEFPDQVAIQLNDTHPTLAIVELQRILIDIEGLEWNAAWDIVTSTFGYT

NHTVLPEALEKWPVGLVQHLLPRHLQIIYDINLYFLQSVEKAFPNDRDLLRRVSIIEESQ

PKMVRMAFLAIVGSHKVNGVAELHSDLIKTTIFKDFVEIYGPDKFTNVTNGITPRRWLHQ

ANPRLSELIASKCGGNGFLKDLTKLNQLEKYINDTEFRKEWAEIKYANKVRLAQHIKKSA

GVTVNPAALFDVQVKRIHEYKRQQLNIFGVIHRYLSLKAMSPEERKKQQPRVSIFGGKAA

PGYWMAKQIIHLINAVGSVVNNDEEIGDLLKVIFLEDYNVSKAEIICPASDISEHISTAG

TEASGTSNMKFVLNGGLIIGTCDGANIEITREIGENNIFLFGNLAEDVEDLRHAHTYGSH

TIDPDLAKVFEEIQKGTFGDSHDFQAMINAVKDHGDFYLVSDDFHSYIETHAMVDEAYRN

QDEWVEKCITAVARMGFFSSDRCINEYAEGIWNIEPVVARD

>SoG_01191.T1

MAEFVRAQIFGTTFEITSSSARDQLTNQNVAIKKIMKPFSTPVLAKRTYRELKLLKHLKH

ENVITLSDIFISPLEDIYFVSELLGTDLHRLLTSRPLEKQFIQYFLYQIMRGLKYVHSAG

VVHRDLKPSNILVNENCDLKICDFGLARIQDPQMTGYVSTRYYRAPEIMLTWQKYDVEVD

IWSAGCIFAEMLEGKPLFPGKDHVNQFSIITELLGTPPDDVINTIASENTLRFVKSLPKR

ERQPLKNKFKNADPPAIDLLESMLVFDPKKRITATEALAHEYLAPYHDPTDEPVAEEKFD

WSFNDADLPTDTWKIMMYSEILDYHNVDANEATMADGFNGQ

>SoG_01194.T1

MAPAEAEKSTKKYDLEDVLDTNTFDQILEMDDPDSDEFSRSIVTGFLEQAEETFVEMDDA

LSNEDLIKLSELGHFLKGSSATLGLNRIRDGCENIQRYGKQETQTGEPIDDPEKCLVLIA

ETLTQVKKDFDFAQKVLRDHYSEKAIA

>SoG_01211.T1

MLQSQLDPLPRDLPFRIISKTVGRGAYASIKKAIPLDSSEPVFAVKLIHKGYAVKHGRIS

AKQLAMEVSLHSHIGQHPNIIEWFASGEDAVWRWIAMEFAEGGDLFDKIEADVGVREDIA

QLYFTQLVSGVSFMHSKGVAHRDLKPENILLSQDGQLKLADFGMATMFEYKGQRKTSSTL

CGSPPYIAPEILACGRVDRRAGTGPKYSPDLVDLWSCGVILFVLLVGNTPWDEPSQGSWE

FQEYIRTSGRSTDALWERIPSQALSLLRGMMSVDPRKRFTFAQVRQHPWYTRHNSLITAD

GQVSDPIHLATQMLENLHIDFSQQVSSSQRPVSDDMDVDTGLNAGRFASTQPETPIADTD

WDWERPALRSMAAPSSSLPTAQADDARRMLLNHLADEPSMSQFAKKAGPTMTLTQQARRF

RDICPPESLTRFFSHVPPAHIVQMLSDALHQLNVPLGGVPVPNPYGNPMATLKVKALDGR

QQELRGEIVVDRHQLPDGNEVLDVRFVKVKGDPLEWRRFFKKVVILCKDGVYTPDS

>SoG_01214.T1

MAEAHQPATEMTPSSLPDLTRNRLPTLFEVLSRRTLPPVDLFSFYIYMRDQQRSVDYLDF

WLDVAQHMSLCRHYVRELRRSVLVGTPDIQSKRSSAILENMGDLEPRAAGPSMYATEKEK

DQDAQMSAFLREEQAPPAPQSDSSRMRPSPQFSNSHDITTDSNSPAHTVARQDIRASAEK

ILYTFLLPGAEREITLPGSITQDVTTSIEEYGRDDPEVFDVAKDYVFQAMERDAFPGFLR

MKALGNLIPPTLIMRLIVGLLAMFGGFWAAFILIFLDKSRQTRCWLILPFTIGVYLLASY

QYSLDPIMALIGFSEYTPFNFSRIREPYVRKLLAKRAVMVLAVTALVDAALCLLFILVPG

KRL

>SoG_01216.T1

MGQTYTPVAQGERRQSSGSALAAPKAVHMATSTLRVGGMTCGACTSAVETAFKGVKGVGT

VSVSLVMERAVVTHDPQIISAHEIQEVIEDRGFDAEVLSTDLPSPVVARFESHVTDDNAE

AASATTTVAIEGMTCGACTSAVEGGFKDVPGMKNFTISLMSERAVIEHDPKLLTPVQIAV

IIEDRGFGAEILDTVTTSSARTGANAPAQSNVATTTVAIEGMTCGACTSAVEGGFNGLDG

LLKFNISLLAERAVITHDVTKLTAERIAEIIEDCGFDATIVSSQLETSGPGSGTSMAQFK

VYGCSDAATAEALETSLLALPGVTSATLNMATERLNVTHQPNVIGLRGIVEAVETRGLNA

LVADGQDNNAQLESLAKTREIHEWRSAFKISLAFAIPVFIIGMILPMCIPALDLGKLELF

PGLFLGDLVCLVLTIPVQFGIGKRFYVSAFKSIKHGSPTMDVLVILGTSCAFFFSIFAMI

ISTVFPPHTRPATIFDTSTMLITFITLGRWLENRAKGQTSKALSRLMSLAPSTATIYADP

IAAEKAAESWAKSKDVPETPKTAEASGGGSSAWEEKIIPTELLQVDDIVLLRPGDKIPAD

GTLVRGTTFVDESMVTGEAMPVEKRVGDNMIGGTVNGDGRVDFRVTRAGRDTQLSQIVKL

VQDAQTTRAPIQQLADTLAGYFVPTILILGLTTFLIWMILSHVLPNPPSIFLKDDSGGKV

MVCVKLCISVIVFACPCALGLATPTAVMVGTGVGAEHGILIKGGAALERTTKVTQIVLDK

TGTITHGKMSVASMNIVSQWTRTDTKRHLWWSIVGLAEMGSEHPVGKAILGAAKAELRMG

TEGTIDGSVGDFKAVVGRGVTTVVEPATSDRTRYQVLVGNLIYLRESGIEVPDEAVEDAE

QLNSAFKSSSKAKAITSAGTTNIFVAIDGQYTGYLCLADTIKEGAAAAISVLHRMGIKTA

IVTGDQSSTALAVAAGVGISADNVYAGVSPDQKQAIVAQIQEAGEIVGMVGDGINDSPAL

ATADVGIAMASGTDVAMEAADVVLMRPTDLMDIPAALSLTRTIFFRIKMNLGWACVYNLV

GLPIAMGFFLPFGINLHPMMAGMAMACSSVTVVVSSLLLKFWQRPRWMNEEAGRTGGERW

VMGSGLVGWVKETFTSRGRGRGEEVGYVPLENLEDGR

>SoG_01240.T1

MPVASSRPSRRAATRRAVIDSDDEEDQIANTTRRADEDEEDFEPQAAQSPKRTRRSTRQS

SIAPAQPPVAPRGRGRPRKSVATDVSVTDENSIAESEAASEADRTLKADPETPPKRRKMN

TSSIAVATPANKALPTPQSMAPSDFTTVTATPISDITATSVNTRLLDETPATVKPIRAMD

AIMENPIDIKLKSRTMNTMPLVEPEAPKSRIVLTYLILTNFKSYAGRQEVGPFHATFSSV

VGPNGSGKSNVIDSLLFVFGFRASKMRQGKISALIHNSAQYPNLEFCEVAVHFHEVMDQP

DGTQQIIPDSDLVISRKAFRNNSSKYYINNKESNFTTVTTLLRDRGVDLDHKRFLILQGE

VESIAQMKPKAGNEHEDGLLEYLEDIIGTSKYKTPIEEAATEVEALNEVCVEKSGRVQHV

EKEKSSLEDKKDVALAYIRDENELVMKQSALYQLYTHECKENLAVTQEAITEMQAQLEAE

LEKHHGSEQVIKDLEKQYAKGAKELDAQQKLAQSLTKELAKFEQERVKFDEKRKFLDDKK

KKLEKTIKNAEKTSSEANETIEQCTEEIETRTKEIAELEEQVRAEEAELANIRDSLKGKT

QAFSDQIAAKQKSLEPWMEKINQKQSAIDVAESELNILQEKANAGAKAREELEVKATQIE

ESKTAKAKELKACEKQKGELVAEKEKFESELEQLAGTEPKMRAKISNARQKADEARSSLA

SSQSRGNVLTALMRLKESGRIGGFHGRLGNLGTIDQKYDVAISTACGALDNFVTETVEAG

QQCIEYLRKNNLGRGNFICLDKMRSRDLSPIQTPEDAPRLFDLVQTKADKFRPAFYHALQ

DTLVATDLAQANRIAYGAKRWRVVTLAGELIDKSGTMSGGGTSVKKGLMSSKLVADTTKE

QVAKFEEDRDAWEAKFQEFQEYQRECETRLKELEKEIPQLETKMQKLSLEMESATKNLAD

VQRRIKDVSKEHSFSADDEKRQSALRSEVSKLKRDIENLHSETASVEAEIKALQDKIMEV

GGEKLRAQRAKVDGIKEEISSNQEETSSAEVRKAKAEKQKVKLEKDRAKAAKELDAALKD

LEKLDSDVNNQGEKADELRAKVDEAEESLGAQKEELSALKSELDEKSAELNETRAVEIEM

KNKLEENQKALVENQKQLRYWDDKLSKLALQNIEELIGEPDKAPEAPQTEEHNEGAAGDA

ESAQDEAASSDLEMVDARSQQTTSDAEMVDARSHQTTESSDQDGDSDSETGDDTMVIETR

AARYELPQYTPDELAGMSKKTLKGEIAALEEKTQNVNIDLSVLAEYRRRVEEYAARSSDL

NSALAQRDAAKKRCDDLRRLRLEGFMEGFGAISLRLKEMYQMITMGGNAELELVDSLDPF

SEGILFSVMPPKKSWKNISNLSGGEKTLSSLALVFALHHYKPTPLYVMDEIDAALDFRNV

SIVANYIKERTKNAQFIVISLRNNMFELAARLVGVYKVNQMTKSVTIENRDYIARPQPAQ

RQAPRQPIGVGNTTTLALR

>SoG_01273.T1

MGAAVQDEKRSTWPSPAERSTSTNTSSQGEAIVSLGEGEKEPTAHPERTPTFKDYIRVFS

YATKWDSLAYAGGLVASIGAGVTFPLMNVVFGQFVQQFSDFSSLETMDVSSFRENVDQLA

LHYLQCLFQQSIHVLDTMPPGYATGIITGTANVLQLGISEKLGVFVEYTSTIIAALVVAF

TRSWRLTLVTSSLLLFVLLVVSILLPLIVKRTARQAKAEAKAASVASESFASIRMVTACG

AESRVVEKYARFVEEAKGHARSTSPLVSLMTGLMFLGAFGSFGLAFWFGIKLYTENNLDN

VGTITIVLLSVMMVVFSLERTSTPLLAVGKAMVAACEFFVVIDAPQVPKGHLKEPDVTST

ADIVFEKVTFAYPSRPLVKVLDDLDLRIEAGKITAIVGPSGSGKSTIVGLIQRWYSLQGH

YRLEKVVDLEKKKKKEEREKRRKNKGKSEDDSDIEEEENESGGILEGREALTEPVQLKGT

LMTSGHSLEDINLRWWRSQIGLVQQEPFLFNETIFVNVSNGLVGSQWEHESEEVKRMLVK

DACAEAFADEFIDKLPEGYDTVVGDSGAKLSGGQRQRIAIARSIVRKPKILILDEATSAI

DVRGERIVQAALDRVAKNRTTITIAHRLSTIKKADRIVVLKHGRVVESGTHQSLIASEGG

VYAGLVNAQALSLTEPDESESAQQDDTSEELAHEKSHSRSEGAADGSSQKTQLKDRGIFQ

SFFLLFYETRDTWGFKIATIIAAACAGTAIPLQAWLFAHCIDAFKYSFDKHKLESEGSHW

GLMYLILSIGVGVSYFCCYFASTQLASIVRAKYQTQYFDAILHQKIPFFDEEDHSQGTMT

SRAATDPQRLEELMGTNMAAVYTGIFTLLGGVIIAFSYAWKLALVSAVVILPVLLAASFW

RFRYEIQFEKMNNDVFAESSRFASESIGAFRTVAAFTLEETITARFETLCRGHVIKAYRK

ARWVSLIFGFADSITIGCQAVLFYYGSKLLASGEYDVISFFVCLLASMNAGESAGQALSF

GPNAAQATEAANRILRMRESRLRNNPAGGAVIPNAEGGVKIEIRNLKFKYPTRDTPVFHG

LSLNIEKGHFAALVGASGSGKTSIISLLERFYDLERGQILCNGKDIADLNVYAYRKHLSL

VAQEATLLKGTIRENIVLGVDMDSITDEQVHQACKDASIHDFIVSLPDGYNTNIGARGVS

LSGGQRQRIAIARALIRDPTILLLDEATSSLDSESERLVQAALERAGKGRTMIAVAHRLA

TVQNADVIFVLGEGRVLEQGTHVELLKKRGVYWQMCQNQALDR

>SoG_01301.T1

MSSNRNYDFLVCKPVPTEHHAPPLLAAPYHAKHVLLLDPTLRIKLLLIGDSGVGKSCCLL

RFSEDSFTPSFITTIGIDFKIRTIELDGKRVKLQIWDTAGQERFRTITTAYYRGAMGILL

VYDTTDQRSFDNIRTWFANVEQHATEGVNKILIGNKCDWEEKRVVSTEQGQALADELGIP

FLEVSAKSNINIDKAFYSLAADIKKRLIDNQKNEAPSASGVNVGDKGEGGSGSKCC

>SoG_01317.T1

MAEDRYRQDYPQNPQHSEASQSSIPSELHRHGAGTPANMAASVTLPSIHDPRSGGYGPPP

PPGANPSRGYGPDPRYEPSREGPNGYPPPHQPPPPPGSHLPPQYHQGDPRSPAYPPHDQR

GYYAEQRHPAPYPHDSYRQDGYYYPGPPPPAGYPPYGPDGRVAYGPPAQGQPQAAPRQRT

SIACRYCRKRKIRCSGYQSAPGGKCQNCARMGQECIFQPVSSSSSAAFIPVSAVPGGVPP

GTPLYGAYGQPLAPAHHQPYQPGPPPPSGPHAPPHHVAPAPYYQPGAHSPTESYSSYGDP

RAEDPAIASRRRRRTSEENDEGYRLPPPRTSGEDDPRRRSPAEFSNNSSPSGFGHSQHSA

GARHSPRGPPLPQPSAGPTQAPSNPGGRSPGVANGSSGASTPAASAQSGQASQQGGGASV

MSLSNLVDKNDIDQGMINRLNRPKAPGAPPSNASNASR

>SoG_01327.T1

MVNKILATTMVAHAIFLATGCLSLGFSLIVRNMMDKPPTNGEEATRNLLYKEFPLNAGIA

NGVFIIVAFVASLPGLLMPMRGWLKASGYMVTVCGLFALCVGVYLWVMTLNLRDNFFNTY

LDQEPAVHDLIQTRFECCGYINSTTPAFVTNRICPSPAAAGLLRGCATAISSYSNRFIDT

IFTAVFGFVGVDAILILCIACLAKDRKERERYRHIDEKTGYRQI

>SoG_01329.T1

MAQNRHWEQDKDATVYIGNIDERATSAIVYEIMLQMGPIHNIHMPRDRVTQSHQGFGFVE

YRSPQDAEYAANVMNGIKLYGKSLRVNKASADKQKGAEVGAELFVGNLDAMVDEKILYDT

FSRFGPLLSLPKVARDDSGASKGFGFVSFVHPQQGGL

>SoG_01355.T1

MAGPSSPNPQPHKRQRQSSQEDPSPRNGRPASPTEQSEVSSGGNTTAAAGLTGKPGQSSN

FRNVSACDRCRQRKNRCDQKLPRCAGCVKANAECYGYDPILKMKIPRSYVFHLETRVKTL

EKLLDANGIPYPPGDKLDMLTRRVRDDSESTTAGDHGAVDGEGRDAPKPTNQAIESLKGK

KGGQDPTMLNIVSPSKPKSLASTSGVSFARVVFAAVQYSVSDQNGSQDRTASRHASGSAG

SGTSMRDSFFGLHTKPSIRPAPWPDKEVASRLVTLYFEHANPQIPILHREEFMQMFEQAY

SSPEPPSGSRELYMLNMVFAIGCGVIVGEVVKTEESSSSSKMAIDHSKHQGQPEEYHASA

IVHLEACLSNSGGGLEVLQAVLLLANFALLRPVPPGLWYIIGVAVRLAVDLGLHYEEGND

IDWGLPDDAEETKPSARRDSAQERGRRLWIRDKRRRLWWCTYSFDRLVSTCVGRPFGISD

QVITTEFPSVLDDAYITPGGFLDLSGEESQPSYKHVAHHYFRLRLLQSEILQVLQYNQAQ

VARSSGQGRLYPEQHDDLPCAFLVQFESYRAWRMDIDRRLHEWKTSAPTRQETGVAFSTE

FLELNYWQAIIMLYRQSLSVPAMFEDEYNTSNEVDSPQAFTAELREDEEAIYLKVAEAGH

KILRIYRQLHLSGLVSYTYLSTHHLFMAGISYLYAIWHSPAVRARLDLNEVDFTILAAKS

VFTDMIDKCPPAETCRDAFDRTAKTTLKMASSSGGFGNAAKQVRLQKPERTSWSQADATP

RPQPPGRQQIRRRQQQQQQQQPDPQQFQFDLSLSDTLPSPTISAGGDLGSQGSPSLLKSK

SFEGSEYVGSGMSSKQHPRSNPGGMDSNSISQDGSAANTVDTSMANTSPMMDRRHAGSNT

MMGQQFGLQGAMDYPDAQTIEFLNSLGGPNGSSSAGDFTGGSIDQAQLDLGFGMNWEGLA

NDYSEGQQINPFDTFFFGGQQGQQ

>SoG_01365.T1

MGVIRKKTGNSRGEGGVKYVCDICSSDITSTVRIHCADSACTDFDLCVSCFSKGESRNSH

DPKTHSFKVIEQNSFPIFDREWGADEELLLIEGAEIYGLGSWADIADHIGGFREKDEVRD

HYLSTFVDSPKFPLPKRCSPHDSELANEIPREEFQARKKQRIEERRDAAKNAPALQPKTK

PTASVPSCHEVQGYMPGRLEFETEYANEAEEAVQHMQFDPGDGLNPRTGELEPEMELKLT

VMEIYNSRLTQRVDRKKVIFEHNLLDYRENIKNEKKRTKDEKEILNKAKPFSRVMNHQDF

EDFNQGLLDELNLRLAIAQLQEWRSLKIGDLKSGVQYETEKANRIQKSIPMGSMDRERLA

TAQRSKQPPPPEPVSGAALLVAPELPFRPSTNGQAKTNGDALTNGTTNGVNGHAKPKTGS

APQPIPGLQPLPLKEDCPDYNLLSKEEKELCEIIHVQPKPYLMIKEQIIKEAIKCGGSLK

KKQTKEICRLDSQKGARMFDFFVTSGWIGKA

>SoG_01372.T1

MAQFPLPPTNTIDWSNVGFRVREVNGHIESTYSRTTGQWTPLRFVADPFMRIHGMAPALN

YGQQAYEGLKAFRDPGDQGIAIFRPDRNALRMQHSADVVSMPRVPVDMFVDAVKAAVALN

AEFVPPHDTGAAMYIRPQMYGSSAQLGLTAPEEYTFCVFVLPTGVYHGTHPVKALILDDF

DRTAPKGTGNAKVGGNYAPVLRWSDGAAKDGFGITLHLDSARHEEIDEFSTSGFIGVRRD

DADGGVTMVVPDSNCVINSVTSDSIQEIARSYGWTVERRPIKYAELPDFTEVMAAGTAAA

LVPIRSITRRSSKGLPAGEKVRATDEAETVSFLPDDQEEPGEFCVKLLAHLKGIQLGKQE

DQFGWRFKVEGKDGRVDGAKEAQAGGEATPPVSQME

>SoG_01385.T1

MSANYWESTQRQHWLFTKDQLASMRQKLDDENAELARQYPLPEQRHLAIFFNQQLLRLGK

RLSVRQQALATAQVYLKRFYSRVQIRSSNPYLVLTTALYLACKMEESPQHIRLVATEARQ

LWQDFIGLDTSKIGECEFYLISEMSSQLIVHQPYRTLTALRPELNMTEEDYQMAKSVIND

HYMTDLPLLHPPHIIALVGILLALVLRPIAPQPGGQNSSASSTAAGLAAAQAALHAAHMG

SSGGPGGPGSMGGSGAGDGKDRPQQETRVGRVQQFISWLSDSNVDVTAMVDATQEIISYY

ACYEAEYNDKNTKDQIHRFVKARHLDK

>SoG_01387.T1

MERSPCHVVYVNRKAGQDILISATPSYDTPAECGSATDWTPEQVRQEVQPLLDTFGDVHV

CHSGASCIAKLVELQEAAQIDIMPTLVLIDTPNDANFVNLGGRLSSTGPTKGSTSDFYAP

DEDVYGLSLLQKLVTEAHLRGVSKLVVPVPVIAPVDSLPAASTEPMTDGTVEGANASSGF

RPETRHLVKRCLDLGAADVIISPLSAKCITSLEICAYKAHRDAAKEQQAILEVRRGRKRS

WVGVNEEKPFAYLREAMVSGLMSGICRMNADDDSQLISAHISVSSERQSVIASAVGPWHF

DAHAFTDDELVVAAMFMFKHALGVPEMEPWRIPTDQLVSFLMACRAAYNSFVPYHNFRHV

IDVLQATFSFLVHVGALPSYPGGANAVMAANRSPLATMIGPFEALTLLVTAIGHDVGHPG

VNNGFLITLNAPLAQLYNDRSVLESFHCAAYSQILRRYWPTVFMDKQMRGLMISSILATD

MGLHFDYMKKMGDLQDKLHENNSTDGWTGRQTEEYKALACALLIKCADISNVAREHDTAL

KWMHILSEEFSRQASMETELKIQSSLLAPPKKDDASLARAQLSFMNMFAIPLFQGVADVM

PGMQYTVDELELNRDLFQKKVSDDREKDILQSKEDPTRKRLLHEGTFSPRTMSFAAESGV

ESKHKDSSAQDIERFQALAKLTESLAEVPTQERVQETSPEEMDRRDSQLSPPRSAPTMKP

PHVPSTNDHDYKEINGAVSTFDAVRELANSDPFRAKHESQDSCIDTTASPHSAPRRSETT

EGSASGQTGDWASQATSTATGKMPLSPSTQGTSLVSSESMERQGCSLPGMRLATSAPNSS

TNTTMRKESSHTDVDSNSTASIGMTDGKALKKKPSRFRMKDFPFFRKHHKSSSPSQSATD

APGC

>SoG_01390.T1

MAPETPKGVSSRLLTMKFMQRAAASGSSAGTPDSASQTSKKRKLDNSSSNGKPTFNIDQA

AVQAAINEQQATRQAALDRHVTGDTHWVLNTSLESTSSKENNTAPLKIKYIGYGGVDSSD

ESGDQGDGAQVGRTSTSNYKSGAKKQKVESNQSTGDGDPSESNSDHSETEASPAQRSSAQ

NSRPSSRSGSRSRSHSRHRAESSKAKELRDKRKKKEVPLSKITSISSGGNRNFSPQSGRG

GFKGGR

>SoG_01402.T1

MARIKKKGTAGAAKNYVTRNQAIRKLQISLPDFRKLCIWKGIYPREPRNKKKVNKSSTNS

TTFYFARDIQYLLHEPLLAKFRDQKALEKKISKALGRGDVTDASRLEKNAARPEKTGKPS

YTLDHVIRERYPTFVDALRDLDDCLSMLFLFANLPSTSTVPAKMIARCERLCLEFQHYLI

TSKSLTKSFLSIKGIYYQANIQGQDILWLVPYKFNQRVVGDVDFRIMGTFVEFYMTLLGF

VNFRLYTSIGLKYPPKFDAVKDENAAELGAFTLEGKALVGANEEQQKQIEAAQHRPDPKT

QAAVNKMLTQLEEGKDKNETAETSAEATEEADAGAIDKFEPTAPGGDVLPQPVASASNAG

SLFSNFTFYLSRETPRQPLEFILKSFGCKRVGWDAVLGDGAYTTNELDSSITHQIVDRPP

IQAVHDEDEEGAEDNQTSQKLAANRRVPGRIYIQPQWVWDCINDGELKEPHLYAPGASLP

PHLSPFVRSVQGAYDPTIPLEEQEPAAEALARESDDDLAVKDVDGAEGMDIADSDDDEDD

DFGGFSADEQEADEEEAEEEEEADDGSEDDADQRQKELEAELTGASVSSSKPKDKKAKAR

EEARKAQAKKDKEEAEDVERAKGMLSKKKRKLFEQMQYTNNKKSAEDEKLRAKRRKFEKE

QAQKKKGKKA

>SoG_01403.T1

MSRKKPADEWGDLDSDDIASVASDELHAHRPNRWTGPASTWRNLTKDERMLWKSMERMRS

EDLAVHLYNAFALKRQATEPEGRKFLTVVTEDGKETVWQPPKAWTAWPLKPSQLSFTTLL

NHPSDQHDHLTFSQPTYPMPSTSLHEELNANILRIAKDRFRRRMAAQAKRKDGAGPIFAS

IEGPASDAAPSSPGSEVSYRSGREEKRGRRAVSDMEVDVEEDGRSSKGKVDEKRKRRTGR

PQVSADDEYSEEILRAPVRHILTQLDKTLTILHHGRVAMASHLEEPSDYEDDTELEPGGP

STPRTRKRRAKSRSRSRSRSRSQSRSRAASRPRSHSATPAPPSRPTTPGGTARPRGRPRK

VHVPLEGETPEEMAVRVAKQSHRANPATVARRQDAAFEAWLQKGEADEQREKERTQGLRL

TADTGTGTAVAAAAGTAAGEGKSWREQKLDRMGLRDWGDVLGAAALAGFSEEVIDRTMKR

CVKLFGQGMVMRRFTEVPIKAGPGGREQRGDGGGGVVERVYRPGEVRAEDTDFEDDDNDI

LHPGNRKTASVSAPASRLHSLSRYSSLAPTRSPTPSSSAHNHHNIKPTTARLPIFPSATP

PPILSSPSRSRSRSRSRSRSATADMYCPEPSCPRSARGFDRRANLERHVRLVHGGRRVED

WEVDSDDDVVGGVRVDGFLREIVPETGWFKKRRVRSRRVARREAGDDDDDGGGGDGAGSE

SA

>SoG_01419.T1

MSVLSKPLAAALALAAGVAAHGHVDHVIINGVRYDGYDSPKFHYTPNPPNVFGWTIEQTD

DGFVEPNAAGHPNIICHRDAVPAKSHVELAAGDTVFLQWNTWPESHQGPVVDYLANCNGP

CETVNKNDLEFFKIGNAGLIEPGGAQSYNGYWAADQLIDTGDAWSVRIPPNIAPGNYVLR

HEIIALHSGGNPNGAQFYPQCFNLKITGSGTAKPAGVKGTSLYKANDPGVLFNIFTNVQS

YPIPGPTLAPGGVSWVPQTRTSATATSSATPHGGSGGGGGGGGSPPTSSAPQPTTTSGGG

GGGGGGNVSSPPPGSEAFVVVRVGPGQLAAQAAHAAEVTTTTRSASDTPSIRFEPWTEAH

GPSVEADG

>SoG_01436.T1

MPHPGLPPTPGSSSDIKGQDGSQHLNSLHLAFELPPPALHDASRTSPTEYKPSKNDSKYP

HPVGDSPRAPFPMQAAEAVKSRRRSSAAKEANEGVFALPPPPTRSRKIIQMKPKAVNDEE

VEKAAAKSAKANGKGTTQSAKASNSAAKDTGKKKQPSATSAAGRKIARKTAHSLIERRRR

SKMNEEFALLKSMIPACTGEMHKLAILQASIEYLRYLEDCVAKLKAQHDDQAQHNPMAHA

LPSISEFHPTFREEPVDVEMSDSETASPTFTATMDRTEPSPSPALLAQDSRHRQHSYSSV

STDQRHYSFSTSATTSPAFGPQHGTPYAPSNASAVGSALTSPALGPQSDLDHEATAALLM

LNSDRRGTISNGRGLSVRDLLST

>SoG_01439.T1

MAIARPIRGLLFVAIIVWCVFLWQVFLSSAPVISGPEGKNVNFDRDPNLDATGEPQGKLT

RTSEGYAVDAENPERINATILALARNDEVDGMVQSMIDLERTWNHKFNYPWTFFNDEPFS

EDFKKKTSAATKAQCRYEQIPNEHWATPNWIDDQIYEESAKVLKESGVQFGNMTSYHHMC

RWNSGMFYKHPALKDMQYYWRVEPNVHFFCHVDYDVFRYMQDNDKTYGFTINLYDDPKSL

PTLWPETVKFLAEHPDYLHDESAIKWLTDDTARPQHNQKAQGYSTCHFWSNFEIAKLDFW

RSQQYEDYFDHLDRAGGFFYERWGDAPVHSIALGLFEDRRKIHWFRDIGYQHTPFFQCPN

SPKCSGCTPGRLTDGEKWLHEDDCRPNWFKYVGMD

>SoG_01441.T1

MWELIAMSCPTTLSHREALQVFCNERIQPPLAVSERATPLQATPLSACTLVQRLEPGDLF

RSNGKGSGWLHSSLPPRCAFSPQGQHFSGTNQQPLEGVPLACQRIRTEFQKGLRSLGNPR

RSGSALKTLKQSCPTLSSPGLLRRSLPMGSIAAADFRQHLVFPGTPPELVSPSAASYASL

SAPSEAQTVGSGVSTSPTMSIHDKAPAGDVSPAMRREDSSHSRPDSSSASAASRQQLPSL

SSLFGPPSGRPMNSPASDRPGSFPTPSLLDRPRITSTDRASGSYFPQGIPAPMSQPRSTL

DGKLDSTERAALQPLKRTFSGSSSPGYRDLANARSESRAEADSVGKWPTQHDLSRQEYTL

SSKEQHLRMSNEPFRLHFSAPRDAQISPYADQRSSHTNPPPTPTSTINSEGMSKDGLGPK

IWTGSHFLPRFVRAADVPGEGLCYFYDDGSHCKTVIDGEAVNAHWGVTKAGKPRKRLAIA

CMTCREKKIKCDPDYPRCVQCEKFGRICKFKNAPRGGHNGSPTTPPAELDDMRKMGGQMG

AGEYRIGDSESGSPISPRTALPNRSPEEGSHKRLKLEGSRYQTVADPHTSTSYSLDHSKA

EFPAHHRPRELPRIPDNVLNRAWQTDPYVSDPQSIMAVVSHFFAQIEHTMVIRFLPQDAT

MAWLSTSAHRKTPEDLMILYSMLAVGVALSNGPRHIAFEYAQVAHYAQRTLQQDCLQLVQ

SRIVLSLYYLSTQRLRDATDLMAAATAAGASLQLHLELDDSAESKRHSFPLNMTRVGYAE

SRRRTMWSLFMLERLSGFFPQQPAIINAEDICIRFPVETRAFEKQLDDFAPIFDSSQSAS

RPLASTGCLIEMVHLWSSCQAAVFRQIRRLVWSEFELDKFRSLLDRLEKWRGRLPESLIF

GPQNLEGAILVGKADAFLWMHLLYRHSIIALTRYGGIPNQMSRHERMVAAYQCRDNARLL

LDILGTGHAHRGIPSFWNSMPPTTSLLVSEAVEVLSCGGSIASLDGVIKVIDSARPAMET

LGMVWENPRVTASAIDERLSKLRRIKDHGSEIPVAGAGYRIIRGSPGPSCNGVVWQLEDG

PRTSSRELDILFHVQH

>SoG_01444.T1

MTATIEPRLIHLLNESTTPQLPSADLPPLAQLPFPKSSDLPLPPIDSDPIYRSERNGLDG

LSLINSSAPFANDDFIQGAFRDENGRSIASGSRSHPLQSLLSDSDPVEPQPTSLSKILDD

TSKIAEESTKKKRGRGLNAKDDFMQLPQPVKKQKAAPQTHVMPPIINGLHEPPPDAALFP

PIASGSTFPEQESSKLSFLQDLGYGTEERSQAMPSQLTAREEVDNEDKTAGKVKKRAAKP

RSKWSEEETKQLLLGVSKHGVGKWKNILGDPEYHFNDRSAGDLKDRFRTCCPDELRNLKK

SAPKQSSHLEVVPSKHGPKKGRDLDKILISVSEVEPEQGGVASAEGTPSTAAAPKKSRAH

RKKMKDLADLGIVAPFRPSGRRERRPFTDEEDKQILEGLEEYGPAWTKIQRDPRFNLSSR

QPTDLRDRVRNGYPSVFQRIEKGAFQAKCTSSRTNDIMEPSVVTSIGNRLEWATVIDPQT

NRALPREDVSRWPLPILDNTDMPLSPHQLEFGDGSGQNIMGGEMDISRLLLDEPSLN

>SoG_01449.T1

MSRNGTPSLPRRAPQWSHLRQRSRSSLDRASSAADIPASPSSASTRSTVKDERRSVKQRI

CTLGIIEGHSRDEVVLNYERLGGGIPPKTLMGITMIKGDSARTGAGYGSMNKQSGNDHSV

PSRGMSWSQNDHSEAVIDRYVFVAKDAPKHMKDRQPDADVLVVKQIADAFGMKKGCHVFL

EVVCSPLPHHDRDSGANSGVQIDGDNPVIEATHIELTFKDQYLSRSDLWRLTIGDLTNRT

IYKGQSILYMDTIKAQVTAVFVGGQRVYSGFFTRDTRPIFRSESARYVLFIQMAKEMWDF

DSESSGEIVFSKVVNGFLPALFKRWATLRVKHLVSIVLFARVEYDTGLTTDLASNALHSD

YYTGIQPTGTKRPYKDFYRVVVSEMASGEWTEILHRLKKEFNFFQRDISSHHHQANVDLG

LTQGDLPKNAQSRITAVPALSMHGNVLEAIHLASSQFAHDYIDRDLTRTGISIAVITPGT

GVFEVDYETLRRATEAMVGNGIGIDLICMPQMPLHSVPLFKYKNPHYSDDRNQPHHLGAS

RSYQSRDSTPGHPTPVVGSYQSFTGSFSPSKGMSNSLTRRMDSLASAGTNEEWCYALPQW

LHVSYWTGNSDEALSYAGVALSVSNKVMKDRGDDFTMRCRMYDLQMRSLLETNEIETAPL

LTDPNFPASIQESGTSSKAWRNGIDEVVYIPNKHIPDSLFDHVYGFQKFVPDRLVKPGEP

SVWKKLREFDNSQAKPPKSKRYCRSTRQDQDLEEDTGRKLSEGVRLFGSSLPERNIAHQQ

VRKQSVNIPRPERPRIAAVVKPTDTQPVVTTATNSRTPAATAKLPRLMRQISLGQRGFGV

AAPKVVAAEVKTETVNASGVSNPPPDTRRPTTPRVLSDMRPASPMTISSQTSSLLAVGTA

NKPQRGREDHEFVPSTPSIPIGRKNPETMRQHSSNPLRNSSMASMASTVQKRQGKHDDDQ

DIKYSDALRAEDAHKVSINKLRAGPVVELLAALSPTTAVTPWLLLLNPSNPEGHRIDDAL

LYSRWQHVFPSTSEMKVQKWKSLCCPASVPLTTEYFPPKTAFDTEYQRHPYTLDQNVDDD

MVEEPRNRKEFIRELISLRFSQGFQVIIGPAVARAFGQKQIKIADIFSRDQPLEDGTSVF

MSVGNTIHQLSCVNGTEVEVNIFVRKPPESSASSDDFTTTYKPAIRTLFDTTYETRHIEI

LTPRPERNWNTIDSYLAGHHDEMMDSLRFWRARFVLIPVSLRSSAAPRAHASDNPEEIRL

EGIKRLAHLWQRNRYIPPSEQWLNTPSKRNDANLLNIVYKTEDSSIVIAAELESLPLVEG

QEAGPRKGLLSRKERFRKANLNLSALAEAMQQPVEQGGVPLRNRRWHLRLHSNSFIGSDM

TTWLLDNFEDLDTREEAEALGNRLMVQDDGKLKDKDRDEDKKGGDGKDEKPRGLFVHVEK

RHHFRDGHYYYQISSDYAKSQPGWFGSKRRDASVPPTPMGENSPRSALPRQTGNESPASN

STTPTMTGVHGRNKRPRVYLSTVIKYDVDHRKKSYRPERVDLHYDRLHNPDNCYHIRIDW

MNTTSKLVEDVVESWSREASQYGLRLVEVPIREASAITEANPFRRPYPVKLAVRPPEKQP

DAYLDPNSSGPQTTQRKFFYQTAILRRLDFVLDMEAASNFPADVDVRFSWGKPDYHYTQY

IHRSGRLLAQITDENHFILVANRLYNNQAYRDHQNRSQAAEQDRGGSSRMVSSMSSYSAI

NLPEPTPISSPMAKPAFHHYSPALKPSEPSPKLSGPPPPPEPEQLIIEVETFCSDRATLE

AFYKETMEKGEGLTGTPAVAPVGGLEAVPEASIPTLGLPPGVLGGDSHNTANLRMGSPMS

FLRRSSVQLDGMGLGTGLPKPKN

>SoG_01451.T1

MATPSSIPANSSRDTVKSIHRITRENRKLWYQLSVLQQPERARACGAGSKGISNKLTNSM

NIAHSDRRPVDPPPVVELRVIEGESIETGKDITFDYNANFLLFASLENSRPMARGRVQTP

ASQSPPILTGVPASGMAYLDRPISAGYFIFPDLSIRHEGNYRLNFSLFEETKEDKDADME

SAESDLPPGFDYRMDIKTDPFQVFSAKKFPGLMETTSLSKCVADQGCRVRIRRDVRMRKR

DNKHNNYERREENYASRAPRAVTPASEDPHAAALRARSLSNSSQQRVPYDGDAHRRPSVA

DSYAAPPPPPGYDTTTGPSRGHLAFGDPNAPQYAAPRQPPSHVAPVSPGGPYTPSGYTTP

TCPTYPTLRPLSRLGQGSSAPIGQEGIDRRPSIHVPSSPGYSSMDSRSRRDSAASYTSTS

IVPPSMPTPAPSNSSYSSRAPLKIASLVLPPPPPANLPIIEAQTEPEPPAPVISTGGKRK

HDYTFFNSSPRLHNGQRPQESHFYNRSHLLPEPHDASYPRADGSLHMAQFPTMRD

>SoG_01456.T1

MAENGTTIPQEATNAETFTEDKGKGKGKAAAEEQPQAMVEDDDDDSSDDEDEDKEEPAAF

AADEDGMEEIDLNNIVDGGRRTRGAVIDFAKAAEENPADDDEDEDDDDFQPPDEDTAMSG

>SoG_01460.T1

MPSINTLAQVMAVLPAIVSAAVNTFDYNSISLREVGARNTLDWRIWLEKDGEPISFWHDI

PLYPDESNQQIINFYVEIPRWTDAKIETKRNEPLICDVTDQRTPTALDPIFHDDSKGEPR

FVESVWPHKTYPVLYGSVPQTWEDPNFKHALTGFPGDNDPVDLFDVSGIEEGYVGQVKQV

KILGALAMIDDDATDWKVMAIDVRDPVAELVNSVEDLEKHRPGLAKSMHDWYIYYKVARG

KPLNTIVGGAYVNASVAASVVHESHGFWTDMMTGKTQADKISREQTSNKDWRKTYVNKEV

ATKKFGIPKKSKVQEAKPKPERYQHWYYLDSEFKPIELKKSEKKLRRNGNRIEEC

>SoG_01466.T1

MQQQQSTSKPTHSKSRNGCLRCKAKKTKCDERKPACARCEDRGFQCPGYALDVRWSHKHQ

MFRDGTASPARQRSRNAGRGTQRNSNLGTPGVSNQGAAMPGPAVEHNVQRLESWPGTSST

PSQWDFIMPQEMIFDLEEASTASSNFLLPPQEQPNPFTLLVGDADLMSSAWTVENDSSPS

TSHEDAPDGNVLHESNTITMENGCSTTTRNETESLLQVDVLQPKSRPPLVLQEARTGLFP

STPREIFNLPTALSEYFFREVITLYCLWDSKSNVMRNIVETMWQSSGALHHTIQSMAAAC

LSEDFPHLQSVARSEHVQALELIHNKHKQTTSLTSKNAMLLASQLLGHTSSWLTPQNLAT

DIFRASCSLLNDISSEVPPSGDASLSFFSDAMDYWAMLLVYLTDSSQLGDYRRNSPSVGA

AGPSQTAEPHPYSGISHEMVRILTDTGILIFQYRKHMAGVKFMTEKDLDVFKQALREARR

LERVLLSNRTPDLSQFRDPGDPKTPLKHLELIDEAYRCTGLLQLYRVFPDLLNERWAPWD

RDLLLRPMPSEGDPPTVGERQTWLTKLAMHILSILREIPFESRTRSAQPFVMVAVSSELR

RDPQHLQQQQQQQQQQQQGFDGDFQAATANAGGIPIDRTSIEVARARKFVGSRLAAYTHI

LPLRKSRVIFELINNVWSAMDAGQQDVYWLDVAYEKGLGTMMG

>SoG_01487.T1

MAKRTWSESQNGVGGGPAHVEQNGVYELDGKTLARKSRALSSAGGINDAQLPIPQISRKI

KACASCRRHKIKCLMDESGPPCRRCAERNLGCVLTKNLQSIIDEKSQYSEAIVQDLEHMH

SALKELVAKAGMPDLPPLQSASLRDSAGSPPNDENSVIISQQVTGASRQDDQGPSCDNSP

KISPEDDGLPYVPIQSLYALTKMRALRSPEESHMQQTCRMRNVNDFIARGAVKLEDAERL

FSLYRDRLDAFMYGVGCKYRTLDEMRRRSTVLTAATLTVAALHDAHSDNTYGVCSSEFRR

LMERSMFERRVDRDYLRAMAVAAYWLSDMSWMLSGYAIRRAAECNLHTSHSRLLSNPSEE

AADCARLWYILYICDQHLATLYGRPSIVQEDASIQGWESFLASPVSNHEDKRLTSQVALL

GILRSIRELFGPDNGQPIPRVYLNQIAHFNRQLDQWIGHCRAESHHNIGSFPRKGAMLHF

HFAKLHLYSHIFRGLPNDSPVPHYFMESATNAVSAALAILDFIRSDPDVAAGIVGMPSYL

HSMTAFACMFLIKVAIKYGGDLIERERVFDVTTALVSQFRSLSTGKWHLANLMTGGLEKM

AATLQVAVPHNYTNGTNGVEQVVMNGTNGLVNSMGAVPQGADMFANVDGELFFDYGMSFG

LSPVFGVDGTGFNMAGTTPQVHGFGEVDYTQVTPRSG

>SoG_01488.T1

MEYTRLGNSGLKISKVILGCMTFGSPTWETAPWTLGEEESLKVIKAAYDAGINTWDTADT

YSNGKSEEICGKALKAFNIPRQKVVILTKIFNPVMDDDSRPASINDGPLVNQMGLSRKHI

FHAVDKCLERLGTDYIVLTPRVKPPDVLQLHRLDRETPPEEIMRALHEVVQSGKVRYIGA

SSMYAWEFARLQHVAKFNGWTEFISMQPFYNLLYREEEREMIPFCKATGVGIIPWSPIAR

GLLAKPLGEETQRSKEDKKKEAWFKDANLDIVGRVEEVAKKKGVSMALVSTAWVMHKGCW

PIVGVSSKKRVLESVEALKVKLTDEEVKYLEEPYQARAIQGM

>SoG_01498.T1

MAQWPNDGQLPQFAESNYFDPSTGYTFNHDVLDVHDPSFSQSLPQTHPHASSQHIFDSGQ

SPHPHQQLPQQYVQSEDQMQTLPAADETTLDPSALTLDAFFMSNGGWRPPEPCNYCRRMR

LQCFMLQTTAANPNPVTSCSSCVALFRQCSLAERAKRHHSAFETSAPVIGQLHGVSEEDG

SALAESVALGLSDQPQLHLQTQPPAALLDDLDDDDEIILSSFQQASDLPKFSGKRTSARM

VKRTRALRNWFARHQEHPYPTDDEKAYLATESGLTRTQVTNWFTNARRRQRQSMRAVAKQ

SYFPQGSPMPQSSPSQMSPLERWRNSPPEQEAVDPTALQDALNGLQNDDVHAGEGRYRAF

RGSVDAHEFSSGSASQASSYNGNRSHAWPDGSSNSGSSAAFSQKSADGFGLISPADGYSS

GEASHVGKIPSHVSSGGFLQCTFCRRNFKKKSDWVRHERTIHLPELDTWICSLNNDGNHP

HLSWGMNQTQPSCAYCGNENPSADHLRTHEFESCSERPVSERSFARKDHLWQHLQKFHGC

KRWDGWALDLGKMQKNNDAVRSRCGFCGLKMDSWKMRAQHISAHFRSGLTMASWSGDSGI

EEIGEMARNS

>SoG_01500.T1

MSSSLFMRRAAFGARAFSTSVPRPIARISIIGNLADSPELVATSSGREIIRYSVASNSGP

SNNRQTSWFRVTSFAEGPQRDYLLGVPKGATVFVEGNATINTYQDAEGKTRSGLNVTQRS

IEVVKRPQGAVSEQTE

>SoG_01503.T1

MDAILRQSKAVCPFLKTASPATLRAMSTAARPQASPCGGTMSKLQLYAHRCPVMGKALAV

QSSKHGAAATIRALSSQSKAKIHTTANKEARAVEGSVFDRRDKTPLYPNVPPSARRSAPT

SVLPHPVTSTGKFNYEEFYNNELEKKHKDKSYRYFNNINRLAKEFPRAHMANPEDRVTVW

CANDYLGMGRNPVVLQKMHETLEEYGAGAGGTRNISGHNKHAVELEATLAKLHSTDAALV

FSSCYVANDATLATLGSKMPDCVILSDSLNHASMIQGIRHSGTKKIVFKHNDVEDLEAKL

ASLPLHVPKIIAFESVYSMCGSIGPIEAICDLAEKYGAITFLDEVHAVGMYGPHGAGVAE

HLDWEAHKEGMPRGTIMDRVDIFTGTLGKAYGCVGGYIAGSAKLVDMVRSLAPGFIFTTS

LPPATMAGAKASIEYQMEYDGDRRLQQLHTRAVKEELEQRDIPVIPNPSHIIPILVGNAE

VAKAASDMLLQDYNIYVQSINYPTVPVGQERLRITPTPGHTKEYRDELVAALEEVWTRLG

IKRTSEWAAEGGFIGVGEKDNVQEPLWTDRQLGVEQASKEILASGRAAAGNLTETLLAQE

AGRPSAVAGAA

>SoG_01515.T1

MRLMPSQSLLAASCVQSRLLGTLWLILAASRGASSTTTPDQPGPLVEVHGKTLDGIQQGY

EPEFEAFDRGILGRAPLGVTALKNNVPESLNLSPGTTACYMVEKSTFLGVKASSRRQTEG

SEGSNEGGQGDKSVIGRASNQTFYLSANTCLQPRSIQNDNPTAQPPQLFITISQSKDGGC

ARALSDIPANEKKQFEEGAVMFQINATDDVFIGIVAPDVDKTKFGEVYNFEVAASSDTYY

HTYSKGNNSELLWMDSDSSAALLVTRALTSDPSQFQTVMKSNPPFMLYAENQARNRTGGL

RASVCGLKNNAQILANWKGDGMLNRLCRTGMTTRGPGGYPKQQFYFQGLNATSSYIGILY

KPPASTGKRDVNDAGGGGTVFPAVSFQTVQGKNCKVPDGAGFEFCDEVDWAMPGNDKLNN

TELVKVYDNYARDMYANFQKVLAQVPCEAPPEQAYSLTRNCSDCERAYKRWLCTVTIPRC

EDVSSSNPHAVLRNVGEPFPNGTMLPQELRDELGENVYFNGSRNKQLDGEIKPGPYKEIL

PCDDICYDVVQSCPAAMQFSCPRPGWYGFNISYAKRGDPNDQELKCSFPGQSRTRTNLAA

GLLAPGPYVMTVLLLAALFAVVI

>SoG_01520.T1

MLSHASILNNGRLIAERMGLSPEDRIVVPPPLFHCFGSVLGYMATATTGAAIGFPSPAFD

PHATVRMCSEWDATGLYGVSTMLVAVLEVLDSSTSPAPRNLRKGIVAGSSVPAALMRTVQ

SRLGLEDLVICYGMTETSPVSCMTTPHDPFSKRTTTVGTPMPHTTVKIVDPADPSSILPL

NTRGELAASGYLAMKGYFNDPEKTAEVRRVDADGRVWVHSGDEAEMDEDGFVQITGRIKD

LIIRGGENIHPLEIENCLFQLRGVKEVSVVGVPDQKLGEAVAAFIVPVKGWETAEGHGGS

PGGNVLGKMDVRKWVAEKLSGHLVPRDIFWVDDYPKTASGKIQKFKLREMAVELLKAEAS

A

>SoG_01543.T1

MPQPIPTASRLLDLFNMKGKVVVVTGASGPRGMGIEAARGCAEMGADVVITYASRKEGAV

KNVEELIRDYGVKAEAYKCNVSDYEDVQRFVDEVVAKYGKIDAFVANAGATADAGVIDGT

VEQWNKVINIDLNGTAYCAKAVGTQFKKQGHGSFVITASMSGHIVNYPQEQTSYNVAKAG

CIHMAKSLANEWRDFARVNSISPGYIDTGLSDFIDQETQALWRSFIPMGRNGDAKELKGA

YVYLCSDASTYTTGADIVIDGGYTCR

>SoG_01544.T1

MVTTAPFQSERAHPERRPSMPTRYHDEEPEQIVDHEAGNAEIHRIASQLTFQASPTPALF

PVKPDSNLDPQSSDFDPRAWAKAFYEARNEAMDGASPRQAGFAFKNLNVFGYGSSVDFQK

DVTHIALEAWNLASRAFGQSKKQRVDILRDLEGLVEPGEMLCVLGPPGSGCSTLLKSVAG

DTHGFHVDKSAMMNYHGIRPEQMKKEYRGEAIYTAEVDNHFPHLTVGDTLYFAAQARRPD

ALPNGASPHDYAEHLRDVIMAMFGISHTRNTRVGDDFVRGVSGGERKRVTIAEAALGYSP

LQCWDNSTRGLDSANAIEFCRTLRTQADVLGCTSLVAIYQAPQAAYDVFDKVIVLYEGRQ

IFFGRADEARAYFEELGFVCADQMTTPDFLTSMTSPVERVVREGFKGRTPKTPDDFARAW

KSSEQRRRLIDEVDAYVEKHPFDGEDHKRFALSRKQDQSELQRRRSPFNLSYFSQIKLTL

WRNFVLLKGDPSIPLTMLACNISEALVIGSIFYNLPQNTSSLFKRGMLLFWVILMNALGS

MLEIINLYAKRKIVEKHARYAFYHPSAEAISAIISDLPYKIVNTLCIDLIVYFMGNLNRE

PGPFFFFLLFTFAVAMTMSWLFRFLASVTKSLEQALAPSTVMLTILVLFCGFALPINTMT

DWLGWIRWINPIFYGMESLFINEYVGRDFDCSAIIPRGPGYDDVASNERVCSVAGSLPGQ

AFVAGQAYLEATYSFVVSRKWSNIGPIAAFAILFLCLHLITLEFVMSERSKGEVLVFTRS

AMKKKLKACKATDDLEGNLDTTPEKPARMDDSSSDGLGGVDKQTSVFHWKDVCYEVQIKN

ETRRILDNVDGWVKPGTLTALMGVSGAGKTTLLDVLASRVTMGVVTGAMLVDGAERDSSF

QRKTGYVQQQDLHPTSSTVREALVFSALLRQPAKYTKAEKVAYVDNIIKLLEMEEYADAV

IGVPGEGLNVEQRKRLTIGVELVARPQLLLFLDEPTSGLDSQTSWSICNLMEKLTNSGQA

ILCTIHQPSAMLFQRFDRLLLLAKGGKTVYFGDIGRNSHVLMDYFVRNGGPALPEGANPA

EHMLAVIGAAPGAQTNVDWPNVWRASPEYRSVQDELERLSSSKGLARVDTTASKAAEKAA

ALREFAAPQRVQLMQLTKRVYQHLWRSPNYIYSRFIMSAGLALGCGLALSNVDNTMRGMQ

TQMFGVFFLVMIFQQINTQMYPEFCEQRKLYEARERPSKTYSWRVFMLANIVAEATWNAV

MSVFTFVMWYYPIRLYRNAQWTDEVHSRGITTYVIVLAFFIMTSTFAHMLIAGLPNPDIA

GGVLTLIFIVTFALCGVLAGPNDLPGFWVWVYRVNPLTYVVEALLGTSLANAPVRCSAKE

IVYFAPPNGTTCAEYMQGYISAAGGYLTSSSSSSDHCGYCAMENTNQFLSALNVDYANRW

RNFGFLWAYTAFNVCAAIFFYWLVRVPKKSRVATKKDS

>SoG_01549.T1

MDTNASNNRLYLNFNGNDRLGAAPNDRTYPTTPSTFPQPVFPTSSQPGGMQSQQGYNAGY

APNAYFQQGQQYQQQQQQQPYGGQQMNDYGNAQANAYQPRSNTPGTNDPNTGLAHQFSHQ

NLGGAARNAQYNSRGPSPSQRPRTAGATGQPGAPSYANYPAVPSQSAAPAQSFQPAPERN

PDRYGSNANSNQKKCSQLAADFFKDSVKRARERNQRQSELEQKLQDPNQNPARREQLWST

AGRKEGQYLRFLRTKEKPENYTTVKIIGKGAFGEVKLVQKRGDGKVYAMKSLIKTEMFKK

DQLAHVRSERDILADSDSPWVVKLYTTFQDAYFLYMLMEFLPGGDLMTMLIKYEIFSEDI

TRFYIAEIVLAIDAVHQLGYIHRDIKPDNILLDRGGHVKLTDFGLSTGFHRLHDNNYYQQ

LMQGRSNRPRDRSSVAIDQINLTVSNRSQINDWRRSRRLMAYSTVGTPDYIAPEIFTGHG

YSFDCDWWSLGTIMFECLVGWPPFCAEDSHDTYRKIVNWRQTLYFPDDITLGVEAENLIR

RYEESSVLH

>SoG_01555.T1

MDSTVEKIAELFAEKNHTLFLGRGAQFPVAMEGDLKLKEISNIHAEAYPAGELKHGPLAL

VDIDMPLVTVAPNNELLEKLKSNLQEGITWCS

>SoG_01569.T1

MHERDDRQQIPDRGRRHDMRDDYDSRREDSYRRQEYSTSPGAQHRSRSWRRPGGDRRHED

GEDDHHRRRGGAPRSDERRRDFDVGNYSEMYDEPRRGDERPHRDDYDDRQRDYDRSRSHS

RHRRRSWSRSPTREAGQPNDTVILEGLPHSVSTNELRESLVHHSIAMEFPNIDIRVQSSR

GQRRAFVQFESLDNAVTFVNEHYPKLLVTLQEPTDAAPDGQFEAYLHYARRREEWDTRNQ

GNDNGNWNCPSCNYSNYPTRAKCKVCETSPMDSNWQQSLTGAADASNTPSQILVIYPLGS

FVNEAMLAADVKRLEREKPERSKTTTESGAPKLKSTAPGAVSSSLGAREGSLHRVFLMRD

VNTDESFKYGFAEFWTLEDATAALAKFNMARSFSVAGCAVTIATIHMGVFLPEDRELDDY

LERQSFHPLFNPSLRVRYRDPHVYPSQMVVTTEPPHGDQKRNVDEDTADPKKGKKRKAEG

PLSSSATKKTAAPMGGQISLWQKKHDEIHDGTVSGDGPGQTQTAPGASPPKIDQNAPIKI

SLSGSTIGGSKSSPNEDRSQSPPDKNGSKNAAVSYVDRERLMCLICMRKYKSVDEVNIHE

RSRNHKNATEKDELVKAALPRLAIRDKRRAKQASEEASDQQDQQQYRDRAKERRQAFNQP

TKPAEQSTNTTTNNGKSSKSDGKPPKPEDAKLAAPAVSKGAGMLAKMGWTAGAGLGADGS

GRTEAITTMAYQEGAGLGAEGAKLGDAAAVAERKTTGSYKDYVNTVQDKARERYNNLG

>SoG_01573.T1

MPTVPAPSSTYPLPTVEGFTYSLANSTTSAEAPKHPIVLAHGLLGFSELPLPIPYLPPVQ

YWHGITSALAASSVKTYTPSVPPSASIAERAEALSESITAQLTGSEHRSVNIVAHSMGGL

DARHMIAHCPPLGVDVVSLVTIATPHRGSPFADYVLEPGAGPLYLPRLYGLLRSAGLSTT

GFSQLTTDYMTSTFNPSTPDSSSVRYYSYGAAVEQAPPLLSPFRTSWSVVEEKEGDNDGL

VSVRSSQWGEYRGTLMGVSHLDLINWSNRLRWTVREWMGMRKTFNAVAFYLDIADMLAKE

GL

>SoG_01576.T1

MRTQSWVLASAFALVATVASAEPERPRIYFPRAVKRQIANVTTEDLPEPASTTNIDSTTR

RGLFDDLLDGVLNPDRTTTSDGARTTPADKGKGDDKKTDGGIIVGPTGIKIPGLEPRPSS

KTGSDSATSTGTPKATSPSSSKPDAKSDTDAPTSPGSRSSSPSSSSSSPSSTKLIDLNPI

LSSLLPDPTSSNSTDSATATATATATATATATSGGHSGSSGIIDLSSLFPPVSETTTSAS

MSTSSSGSVSTTSSELPLTPTPVPSETSTGLPASTSAAPAETSSSLLPSTPLTPLPSTTT

GSSEASATKTTEATTESAATSLVSVTSAADLPITPTSQSQVPVFSTPEPEPTTLSTVGPS

RVASTSLAEPTSTEKNEENWLPTTIVQAPTSFTFVRPTAEPTGTATAIPSNIPKVILPDE

PDIKQPEGTTAIQIGFLYPLNWGFVSKSGVAAAQVFKWLPDALAFAGEFPINNVVVTKLV

PLDTTSKWGYITTIAKVYYPTDQLPGLQMQMGVANSRLYNNDKEIVNSLTANINRNIDLF

GNIEEENGAANNGNPDSGSGSDSTFSDNDTGNTTAQERARTAGIAVGALSLAGLYGAAMF

IVARRYKRKRQGHQRASSLTSSQASSEMRYTGAGSPALMGGALLSRDISSYGGYAAGGRD

SHGSGQSGSARTANISAPVATENSLGWN

>SoG_01630.T1

MSSQPLLQTAPGKRIALPTRVEPKVFFANERTFLSWLNFTVILGALAIGMLNFGDRVAFI

SAFLFTGVAMLTMIYALVTYHWRAKSIRVRGQAGFDDRFGPTFLAIILLLAVVVNFVLRI

MDQSKKQKSGLN

>SoG_01638.T1

MLAVRQRPRMGSSQPPTDEFLPFGYDFNAPPEPLSEAPEPAPGNPLLSETDSKLLSSFFD

DMTADHYSMPSFGEGLNFSSAWFDLPPQFMGSATSLGQQPDLAAVSPGALSSPIDQSVMQ

RQVMMPNMMPPPPPPPPPPPAVQAASQIQHHPQQQLQYQQQHSEDVLHAAATLLQNGAAA

ARGDSQLDFAQSRRAMGPPVGHLRHQPMEEFKEEARRAHMTEFPEDSNFMDWMGTAALQP

QRSHSRLLPISEYQWGSDSNFNTAKAYTPGSHKDSVESQHRLQLDVLNCLEPSKSANSTR

PNSPGPSNGKPVPNAPGNQTLKPAEDPEAPPRKRRKSKIAKTESNADDEEEAEETSSSKA

SRRRRPKSEQGQSSSPPAPTEGGKRRKSNVNGGKPTRENLTEEQKRENHIKSEQKRRTLI

KEGFDDLCELIPGLQSRGLSKSTMLSMAAEYLEQLLLGNKELADQLAALEGR

>SoG_01643.T1

MELFRLRLNCIDHYQAIPTRYDPQLRKDIRPSQLSKGPKVPVVRVFGSTETGQKVCAHIH

GAFPYLYVEYDGGLTQDEVGSFIYRLHLSIDHALAVSYRRDQYGENARYVARITIVKGVP

FYGFHVGYRYFLKIYMFNPIVMTRLADLLQQGVIMKRKFQPYEAHLQYLLQFMTDYNLYG

CDYLDTYHIKFRAPLPNHDEGSNSSHLWHNWSVSENQVTDDPTLPRMSHCSIEVDICVQD

ILNRKSIKERQLHHDFIERYAPIPADMKLVHSMAGLWRDETKRRKRQLPLDHQGASPFPP

EVMVSMSADPRESDPQGWIHEEEFRDQIQELIDQEGKELDRKPDFHNFAATDPLGSSVNT

ALQAVEDLHPSSLMPALGLPSDVDAQDMDIASSIEVDDKKAAQVVADEHDFFPDDSDEEA

LRDLINAEQLEADKALEGKKPKSSGSGSGTSQSSHSPDRDDWRTYAGICESGLQTGQLPT

MPFSDVLLDIADNEGLIARTINLDAVKKLAANAATRTEVLTIGQIARKRAHEEMERSAAS

NASNDGVADNAISPRTPKNSFPQPSPNRKRSPDNASRIGSQKSPSQTLNFPIVKDPDDPN

TRLRLSQQSASSSSQPDAKRLKHVSFDSSSFGQTACVDPPSSSALVPDQMSSQSDLMPSS

SNSTELNIAPFSASARTSVIAALPPSQSSVLGTFGDHLLPEVLYRDAFYGKEADVPARAR

EYAGREFRLEGNTLPFLPAFDPTASSPANFGIRGEAPDKAAEKLAYSRQSRQCSWRSWEI

AQPPPSYQEVAQWMDDRTSAATEQGKSVRDIRSTQKMAMSQIEGATPRNKHGFKYTQRSK

VTSVEHEIQYMSTMSLEVHVNTRGKFVPNPEQDEVQCVFWALKSDETILSSQDPSTAIRS

GFVILDPDGTLEPRVRKQVKGEVVAEETELDLMTRMIEIVRTHDPDVLTGYEVHGSSWGY

LIERARFKYDYNLCDEFSRMRSQSHGRFGKENDRWGFNTTSTIRVTGRHMINVWRAMRGE

LNLLQYTLENVAWHLLHRRIPHYSWKALTSCYKSGNSRDLGKVLRYYQVRTKIDIEILEA

NELIARTSEQARLLGVDFFSVFSRGSQFKVESIMFRIAKPENFILVSPNRKQVGGQNALE

CLPLVMEPQSAFYSSPLLVLDFQSLYPSVMIAYNYCYSTFLGRITNWRGMNKMGFTEYKR

QQGLLSLLQEHINIAPNGMMYCKVDIRKSLLAKMLTEILETRVMIKSGMKKDKDDKVLQQ

LLNNRQLALKLLANVTYGYTSASFSGRMPCSEIADSIVQTGRETLERAIAYIHSVERWGA

EVVYGDTDSLFVYLKGRTREQAFDIGNEIAKEITAQNPQPIKLKFEKVYHPCVLLAKKRY

VGYKYESKDQVKPEFDAKGIETVRRDGTPAEQKIEEKALRLLFETADLSQIKAYFQKQCS

KIMHGSVSVQDFCFAREVKLGTYSNKGPPTAGALISTKRMLEDARAEPQYGERVPYVVIT

GAPGARLIDRCVAPEELLQNPHWQLDAEYYISKNLIPPLERIFNLAGANVRGWYDEMPKV

QRIRHATGSAKKTTLESYMQSTNCLICKSRFPRSESTGALPG

>SoG_01649.T1

MSFPTLRGTSGVLRRTVGRPVVACRNVARPCVAARYSSSMTLDGQQQVVLPLTVLWDIQC

VADSGINSSSPATSTRLIPPSLTSSSRYVASTCAISASRADIVFQLQEKNRQKHFINLIP

SENFTSQAVLDALGSVMQNKYSEGYPGARYYGGNEFIDKAERLCQNRALETFGLDPKNWG

VNVQPLSGAPANLYVYSALMESHERLMGLDLPHGGHLSHGYQTPTKKISAISKYFETFPY

RLDESTGLIDYDKLEENALLYRPKIIVAGTSAYSRLIDYKRMRQICDKVNAYMVADMAHI

SGLVAAQALPSPFEFADIVTTTTHKSLRGPRGAMIFFRKGVRRTNPKTKQDEMYNLETPI

NNSVFPGHQGGPHNHTITALAVALKQAQSPEFRAYQTQVLANAKAFAKRLSEEKGKGGLG

YKLVSGGTDNHLVLADLKPQGIDGSRVERVLELVGVAANKNTVPGDRSALVPGGLRMGAP

AMTTRGFTENDFVRRT

>SoG_01680.T1

MYQGGHRHGERPYQVPPPPPMPNPGAGGHQMNGMTQFPPPPPRYPSGGAPAPAGMVAPPP

PGPPPGSAMGQQAPWHSNFGRVYDARGALVPPPPPSSGQHQPYNPQHHAQLAGQPMSIPP

PPPPNEQMSATYIPHGDTYGEVAGFPAFGPDDWAGASGATMPAPNFYTTASNPQSATDTY

QTTSLDSAQTRGLSNASSATNTTSTSTSNIPPEVAAQWPLETVLLWLAKNQFSKDWQETF

RGLNLYGAQFLELGTKTTGRGNLSLMHKQVYPRLIHETTSSGTGWDPAREREEGKRMRRL

LRNVNAGTTVEGYGLGNHHTHNRRESGTSAPPASLPSAGTDQAESPNPLQAPGPGFSAKR

YSQSRSTTMPTLTNNTMSSDSGYRTALKGFDGDRKNSPNHGEGNDAGPFRGAAMRTDSPE

GSPALSSGTFAPAMSASPNTTKFPHRAARLSMDSQSSNAAIYGSGVPAEAASMLSRSLNL

NEALASARSPVERPRQSPVDSGSAGTEPPSSAKGQGFLSFLSRKKRQKEDGSHPSPDDLE

SPTLEAFAKPPFGNRSATASETNLDRSSHSSIPRGRSDSQGKRGPRLFVLATMDYWTYRM

CDVTDAETAADIRQVVCMNLGVGNFDGSSIYLTELGKFDHSEPLDDQKLLLQRKTRADAA

GTLKFFVAPAWNQTNTDGSTKAGSGVPLALSPGYLPPGASMDAEAYDQLNGRQRSSSSPP

SSRSNTLLGEQVDGQALAQEASEYRAVMERKQREYLAKRKQAGKGQSPTAPEVASGYGIV

GRNVDFDQPRNSPFEDKKPDQLFPQRKPPAPPSDPSATLIKANSLSRKPGHNGRASSGSM

EGVPTPRHPTTWSPENEPDRARKARYQGASNAAVSGIGAALVGMGRNLGAVGQSSANGPR

GVSPGRAPAATEDAPQSGKRPSPTTSHSKLDSGTGVRKPKSGSPGTLTWSPGSVAFMVPD

YVRGQPARLEEWSPPNSPTSAHAELPPAIVPPTSQSSPSMQQRSTFPLESNRRRSGQEVE

KGDRDVQFNSPRPTARQAVPPQVNDSDDDSDDGLFAIPLASRNKGKAADKPGHAQRPSLT

VKTQRSKKNLSVSFNSPQSSVLEGTDETPGTRTTGSSRRTPMTPASDTWESEQSEGKLNR

RKSFIEKDVWANRPPTDALLNNLDDFFPNLDLDQPVLEDGVDGEPGPSPIPESDENGEGA

APNSATVQDPRASASAIPPSRQPSLYTNDGDTLGSDESTLKALERPSSYQGVAQRSMRRS

GGLGRMKSIREVARGAHEANKRYTNASQDMGKASGSGPGNMSSNILRRKSTKMFNANIVQ

IRPDQRGSMVLPPVPQDTLPKRQTTFRWFKGQLIGKGTYGRVYLGMNATTGEFLAVKEVE

VNPKAANGDKAKMRELVAALDQEIDTMQHLDHINIVQYLGCERKETSISIFLEYISGGSI

GSCLRKHGKFEEPVVSSLTRQALSGLAYLHREGILHRDLKADNILLDVDGTCKISDFGIS

KKTDNIYGNDKTNSMQGSVFWMAPEVIRSQGEGYSAKVDIWSLGCVVLEMFAGRRPWSKE

EAVGAIYKIANGETPPIPEDIQETIGPLAVAFMMDCFQV

>SoG_01700.T1

MAGFNDAPLIQVIAVPAVCLLIAFLGYGSQLIFQSSTLDPGPPSRNETIVFNILLLVLWY

TYYKAVTVDPGRYVFPEVVEADGRWCKKCSAPKPLRSHHCRHCSRCVPKMDHHCPWTANC

VSMTTFPHFMRFLVYTNLSLWALGRLVWQRFYALWESRHLPAYLGPSLPSLIALAVVGLV

GFVTSLMLGIMLITTTKSWIFNMTMIEGWEVDRHEVVADRAGRDWWDIVGLDGDKVRFEK

LEFPYDIGIFANMSQAMGTSNPVMWFNPFASNPTIAKDGKGAGWTWEENGFNRIEGLWPP

PDPDKVRRAGRPWPAARRDYDDELRQAYLNPDAIDGSGPTPEERKSAFLKRQAADLKRQR

QLMAELEEVDAYDMMDEELNGHEGETYDQGGWFNGDGERLRDYGVDEEAEADELEDEHGQ

DDDIPLAELLRRRKVRRKDGGASEM

>SoG_01703.T1

MAYEPRGDRGGGDGQDGGFPKIRGRRPVTDYSATILHWQHNRVPNYKGGYIGEAERPSAS

YIVDMIPPAGRPNRPADSIPSKHLHSSLNKIKHPVNVVRWTPEGRRLLTASTSGEFTLWN

GTGFNFETIMQAHDSAIRAMEWTHSDDWLVSADHDGVVKYWQPNFNNVQSIDAHSDPIRD

IAFSPNDSKFITASDDSTLKVWDFAKGEMESKIEGHGWDVKSVDWHPTKGLVVSGSKDHL

VKLWDPRATPRCLTTLHGHKSTITKVLFEKVRGACFATSARDQTARVFDLRMMRDICLLK

GHEKDITTLAFHPIHPNLLTTGGMDGSIYHYLLDTPNPPAGQALTVSPYDSPDPDSVPAQ

SIWPAHKVTYAHDYAVWSLDWHPLGHVLASGSNDRITRFWSRARPGDTDVFQDRYHIGEA

AAEAQGTWDRRGNRRQRQEEEQLEFEDEMDALPDQNASRPAVPGLPGIPGLPLGGGLPGL

GAIPPPPVIPGMASGAAPPPPLPFPLPGLNGAPPPMIPGFDPENPPDPQMLLEIMKKAGM

QIPPPGALPPGFIPPPGSIPPGGLPPPGNFPFPPPPPPPPPGGTDDARRRGPLPSQEDSL

RQEQRAGRYTRAR

>SoG_01707.T1

MASAKVDMPHRERNERNERNRDRDGGDSYRPAPRERTPPPVRTEEEKQAAAKAEYEKLLT

MRSGGTYIPPARLRALQAQITDKTSKEYQRMAWEALKKSINGLINKVNTANIKYIVPELF

GENLIRGRGLFCRSIMKAQAASLPFTPIYAAMAAIVNTKLPQVGELLIKRLVMQFRKGYK

RNDKAVCLSSTTFIAHLINQQVQHEMLAGQMLMLLLNKPTDDSVEIAVGFCKEIGQYLEE

MQPSISMVVFDQFRNILHEADISKRTQYMIEVLFQVRKDKFKDNPAVKEELDLVEEEDQI

THRVELMGEVDVQDGLNIFKFDPEWEEHEEAYKKLKAEILGEGSDYDDDDDEEDEDESSD

EEEDEETKAIEIKDQSNADLVNLRRTIYLTIMSSADPEEAVHKLMKINLPAGQEPELPSM

IVECCSQEKTYTKFFGLIGERFAKINRLWCDLFEQAFAKYYETIHRYENNKLRNIAQLFG

HMFASDALGWHILSVIHLNEEETTSSSRIFIKILFQHIQEEMGMPKLKERMVDETLRPNL

EGLFPRDNPRNIRFSINYFTSIGMGPLTEESREYLQNMPKPALPAPAAADSDSDSVSNPD

PGRSLDAGARNGQAGDALCPGKAPTGMAGAVALIRLTRDRGLAARHDRDHLHPADGMLRI

LPARRRSVEVVHPAQVYLVHHHLVMVSVAANVAPHTAPMTAAPAPHHLVVANDEDLTRPA

EAEAEAPVAAGVEHRRRVKIEDDREAAARVPTAEIPLPSAAAAAVPLQRQGKAELRVRHL

TIRGRHHPRGEEGTIPTPGRDLHLEARERGGCRVRWVKEGGAGA

>SoG_01711.T1

MDDLLIQSRPPPAIRIRSSKSLPRLDAKSKTHSLRGRGTLSTLGRLNATLEQVNDAWLSW

KEGTTPEEREQRRCLEDQRQVCILRMTTAETHRQWESAARELDQLEGNEEWKRDAMDGDY

NPQLIAERLRELDEARASGDTRAMMHLVRTAMSRDLGGMGNVDLYRHSYVGTKDLIERYV

DSAIATIDAVVNQGSKHPRMEHRDLLDSMLYARQSFGRSALLLSGGCTFGMAHTGVLKAL

FNAKLLPRIISGASAGSIVCAVMCTRTDEEIPALMEGFSYGDLAVFEKEDGSESGMLLHL

RRLLTEGSWSDIQHLTRVMRHMTSDLTFQEAYNRSRRILNICVSTQSMYELPRLLNYVTA

PNVLIWSAVAASCSAPLVFNASPLLVKDPLTGEHQPWNPTPERWIDGSVDNDLPMTRLAE

MFNVNHFIVSQVNPHVVPFLSKDDHLTPGHDAKRRAKPRSGEDMEWVYTLTALAKDEALH

RLHFMTELGIMPNLATKFRSILSQKYSGDINILPEISMHDLPNLLSNPTVDFMLRMSLAG

ERATWPKLSRIRDRCAIELALDRAVHRLRTRVVFSESQKDLRKMRSSVAGLHTMANGNAA

APDPGTATTVTSPTPPTLAKATPSPPQGRQRRQSGGSYQMLMHRRQQLDGAGSESDNETL

VGMEDQQQQPPVVAGPEVQLQGVGSGLAGSGIQMKPRLRRAVKSYQQVPYRGSLSSLVPN

T

>SoG_01756.T1

MSIVNKFITRTAATDDDNIDHIGTETPGSGVATPQPDLQDKRLPGIMSYFGQVRQDSALC

HGFPTACSPPGESSCLTKELAQNAMPISVKSQHLDVGQVLKADTRTLPDLKHLVISHAQP

NAELNTVHGGAGHLVPYSIPVISQSPSSRWPNEANAQEPISTCDEALEGLAGNGGSVSRK

MEGSNSSSILRKPTAVLSQAVKSPVAAHSADNSIPPLLTRPRPPGKWFSLDGFMELTRGV

MFKSGQSTPTRALSTAQPSQSEGAQSSGRNSHDDGTASGTQTPRGSGGAQVPASKGKLTI

KIKEARGLRKCRDPYVVAVFQRSELISGGPRPGENEEPLNAATSAMAGIPIQRQGSDSGR

PMAIPMRSRQSSNTSISDYNTFRNRPRRQSFTNPTWDAEAVFDVVESDMLVDVSVYDHTT

SGEEFLGHVDFQAKKDAEGVVRGWFQLRGHADTMAENAPTGELYVEALYQRSEKRHFGPS

DFEILKLIGKGTFGQVYQVRKKDTQRIYAMKVLQKKVIVQKKEVAHTVGERNILVRTATS

DSPFIVGLKFSFQTPSELYLVTDYMSGGELFWHLQKEGRFDERRAKFYIAELILAIQHLH

QNDIVYRDLKPENILLDANGHIALCDFGLSKANLTKNDTTNTFCGTTEYLAPEVLLDESG

YTKMVDFWSLGVLVFEMCCGWSPFYAEDTQQMYKNIAFGKVRFPRETLSQEGRNFVKGLL

NRNPRHRLGATDDAEELKRHPFFADIDWDLLAKKLITPPFKPKLKSETDVSYFDPEFTTA

LEQNGSLNERAAALARGYAASTPLSPSVQANFQGFTFVDESALDENMGHRGRHDDEDMDD

AQDDDWDNMNDIDPRRANRMSGILKTGQDDQMVGGQHFDV

>SoG_01768.T1

MISISHLNTNDNIHIRRNSLHQASTTTTNSPNNDHLDVPRGWALDLPTASAIDLLQSQFD

ESADIESDFLPLSINMPADAWGVTLPQYQSKMPRGHHHQRESSLSSLGSAGPASPFNATT

SNPQIAVTTDFGNDALDMQPADTSSANTHYVAKSLGQYPGYHSVDASIPEMAYPVTIPGP

AGNGNQKNDRGLLPAPDFSNSSSRSQPTSVASSVGGDSPATPTVQEPDVTNKRRKSMYWN

HPRCEGDIDSLLEVANAFSSVPKLDRTMTDIYSDELYNPNFAITSTTSPSQSQLAMSPAN

EVFNQRLNAANSQHLSARQSPVMSAGSRERSPFRTSSPLAGALHDFSAPSSSARGMPFNS

AQLVGENKQLQHESRLQHHMAGGRAPETPSTISPKDAILDFPESEDNRNFPLFTSEGHDF

ADQFTKAMMPQPVHGIPASHDSQVAYLNTNMPSVSGLQVPQQYPFVAQSPSDTPPRLSSG

PSSTGSNGSTPIRLGRPVSTSADGGTYTCTYHGCTLRFETPALLQKHKREGHRQAHGFGM

SRPHDMGVPSSLIGSQAGPHRCNRINPSTGKPCNTVFSRPYDLTRHEDTIHNARKQKVNC

DLCTEEKTFSRADALTRHYRVCHPEVEVPGKRRRG

>SoG_01775.T1

MADSDGEFVADDMSDDDIMDHDVEAGDTVNEDNTRLSRSGGGAGALASSRSRPKRRRKGE

GGKGGKGGAKAWEQSKRSWETNLPDEDQDGVLNIAALEAEKRRRLMRDTTPLQRGIIRHL

VLVLDMSFAMAEKDLLPTRMRLTLGYAAAFVKEFFEQNPISQLGIVGMRDGVAVRISDLG

GNPAEHLEKLKGLESQDPQGSPSLQNALEMCRGALFHAPSHGTREVLIIYGALLSSDPGD

IHETVAHLITDRIRVSIVGLSAQVAICAELCARTNAGDDSQYNIAMDEVHFRELFLAATT

PPVTRTQEQSTASLLMMGFPSRVLAPNGGTPLCACHSRPCREGYMCTRCGVRVCRLPSEC

PACGLTLILSTHLARSYHHLFPLRNWVEVSWADAAKSAACFACQCPFPVPPSKGGQVAVD

RKEAESDAAKKKGPKGVSESSRYACEICGNHFCIDCDVFAHEVIHNCPGCQSNVAGEGAA

TETNGADAHSNGAMEIDG

>SoG_01776.T1

MIDHVLGRPSSKSRRLQVLAVLSFWGFYLYKGNRHGPPPAKPLSRLLSKRLTAWQTVLIT

MMYLYAARNFSALVGLASPEPMANMYDATFFRATWVLTALDAGFWTAMKIRSKWLRDLAS

IVFSVFYLFAAERADEKVRKVRGMLTVEHLRVSWNKGTTPYLRFFQGLMRPRFTRWPPRQ

IRIPRPSSSDYKEHVDAWLYFDGPLTALSNQNKLILDIPGGGFVAMDPRCNDDKLFAWAA

RSGLPVLSLDYKKAPEYPYPFALNECFDVYSTIHQTRGRCVGMSGKQTPRIIITGDSAGG

NLAVATTLMVLEASSVTRRRLSSRGDLPPPDGMVCFYPALDMNIGNWMTDEQMSLIRDRK

MRKTNQSIVKRKSMQYNQLAGTPHHSDEEDDAPVAKAEPTTMENLEVSYEVVRPQESEDT

ESQNGKPKSPKQGKTSPGGKRELTGSHHSEPLSTRLATSSIISYFNDRVLTPELMRAMII

LYIGPHDRPDFMRDYLLSPVLAPDELLARFPKTFFMTGERDPLVDDTVIFAGKVRRAKEA

AARRGRGSSSSRFAKSLSEEGYDTPEVLLIPGTSHGFMQFPSVYPPAWKHFERCAAWFEL

LFANADAQRRRERAEQVRAKAAAAKGAESSEEDRPLEISMTRMRSSTAESSGEKDGQVDK

KINGNGKKGKMLSKSKSLVKLSSEDDLLHRRMKGLTSGLTGTIDPE

>SoG_01779.T1

MHRSWVSELLVSLLLAATPAQGSLDCEKIRVDGHTFNLKPLGGPHSVVTSRWEASSEAHF

NTTYTVDICQPLKKSGKAEKTEECPNGTHACAITHMIKGDTDSIAKVVAIAGRLENAGGS

QFDFKATRLKTSESNSDSSKEGVRLLLQGGKHPLEGPVKERKAQQAIIEFLCDPEKEGTE

GEWVSEDKYERLARRDDEGKDGEGKDDEGESSIEHQLKNDNAALIWESFKSEKDADVLRM

TWHTKHACEDKRSDGDNGEEPISTSWGFFTWLFIIAFLGIAAYLIFGSWLNYNRYGARGW

DLLPHSDTLRDVPYLIKDWVRRVLNTVQGTGSRGGYSAV

>SoG_01784.T1

MKLLHLLLGASISISTAEAVENPHVRARNLVAAKRPRSAPAPAAPRGLRPSQAIPSFLTN

KTKEFVVDGKNIPQVPFDIGESYAGILPIQKGGSKTDPNQLYFWFFPSDNPAAEKEIVIW

LNGGPGCSSLEGLLQENGPFLWQAGTYAPAPNPYSWTNLTNVIYIDQPLGTGFSPSTPDA

PLEITNETVVATQFMGFWQNFIDTFDMKGYEVYLTGESYAGMYIPYIAYNMLEKNDTHYF

NVKGIQINDPVIGHGHVQTTVPAVMHLNAYSNVFNLNETFMKEMNERADSCGFTSWMEKA

LTFPPTGPLSVPAKAEEPGCSVWGDIINAATLVNPCFNIYHLLDFCPFPWDVMGLTPSAG

PDNFFNNSDVQKAINAPPTNYVECGDPNLNLQDDTSPPSSFGPLPTVIERTSNVIIGHGM

YDYLLLVNGTLATIQNMTWGGKQGFQDRPKDDFFVPYSSILAQFLDQSQQQSFPEPPVGI

VGGAGFMGVTHTERGLTFVTVNNAGHEIPLYNPGAAYRQLEFLLGRIKSLTQQGDFTTET

GNFT

>SoG_01787.T1

MSTVPPDQHRNTLMRTSSAAGTMAAPSAKPKAASAAKAANNGAAKSKSQMHRRSRTGCYT

CRLRRKKCDEGNPMCTACRHLGLECEYKRPMWWSNNDHRRKHKDNIKMIIKRKKLSEKSA

HSISTSVSSPPGLSHSLPTSATFTDPLDRNRSASIDSHFSTAFNFNSPPTEYGHFGGQMP

HEFMFGNYSPYEIDVKTERQMFVNDVPTLRESHVSTFSTYQTPPPPGTVLSQGPINPEWT

QQAHHEHRRESLAEEALDVNFFDFSHGQPTDASQFKVELEEKDQRLLDHFIQHVIPTIFP

ILESNQHGSVTSSMILPALQSNSIYLHCCLSIAGQHLKACHGDDGSSIDQEIMHHRYATI

LGLCEALKRDEDHQQILEVTLAVIFFQSVVGRLDDGLLDVPWHQHFQAAISLVQKLDLPR

LVSNPAEPMAQTPFNMTLTAWIDILGGSIQGASPAFAHTYREKHLSKLNPHLGLRELMGC

DDRVMYLISEIACLESLKKDGMDDFTLCQHVSSLGQEISNTEIPEAPRIPFNTNGTLSPK

QLSRNITQAFRLAARVYLCSLVPGFSPSQSSPVALIEKLVGVLQHIPCGPEGFDRSLVWV

YLIAGSMSLPGSSFRGFFEERVMQLGEKANCGSFGRMTTLLRETWLQNDALASVSPPSSP

GSDAPRPYIHWRDVMQAKGWELLII

>SoG_01798.T1

MAFQPAAVHQEPSTPTQKLSVLHGPSDPPLLDLTLGELLSLQTYQHGPKECLVFPWTGAR

WTYNDLHQQSSLLAGALLDMGIGTGDRVGIMAGNCEQYASVFFAVAKIGAVLVILNNTYT

ATEAMYGLEFSDCRVFFTTQRIGRVDNTKLLGELESRRVNGEYSPKVVILRGETGKHTTT

YDQLIQSSRRQDHERLYKTMSRVLPHQVVNLQFTSGTTGLPKAAMLTHHNLVNNSRFIGD

RMRLSSNDVLCCPPPLFHCFGLVLGLLAIVTHGGKIVYPAEVFDIDSTLRAISDERCTAV

HGVPAMFDSLFQAELPKDFNCERLRTGIIAGAPVPRYLMELLVNKFGMTEFTSSYGLTEA

SPTCFNAFTDDALDRRLTTVGTLMPHAQAKIIDREGNVVPVGQRGELCIAGYQLQAGYWN

NSEKTNEAMVRDSAGVLWLHTGDEAVFDEQGYCSITGRFKDIIIRGGENIYPLEIEERLA

AHPSIIRAIVVGLKNKHYGEVVGAFIELSPDCPRDRRPSDLELKEWCRRKLGGHKSPAHV

FWLGEDGVPAAVPLTGSGKARKFEMAKLGDELLARRGSAKL

>SoG_01799.T1

MRNVDGSSAGGLLTTLETQTNQDTTSKVEEASSGSQTPLPSSTADLDTIWSWNSVVPETI

RRCMHDLFRDQAAQRPDHLAVQSWDGSLTYSQLDELSTKLAMHLLDQGVKTGTRIPLCFE

KSMWAVVALLGAMKSGATVSLTDPSQPEARLKTIVEQTEANIILTSAAQSVLGRRIAGNA

KVVPVSQEFLDKTSPVAASDSLPIVDPASPMYIIFTSGSTGKPKGVVLSHESYTSGAVPR

AKAVGYTASSRVFDFPSYAFDVTYDCMLCTLSVGGTICVPSEESRMDDLSGAIRSSKANM

VHMTPSVARVLEEDIIPSLDVLGLGGEAVGARDAAEWGKHTSLVIAYGPSECTVGCTINN

TVYDSTGIGRGVGGVTWIVDPDNHNRLMPLGGVGELLIEGPVVGVGYLGEPEKTAEVFIE

SPTWLTQGHGSVAGRQGRLYKTGDLVRYEDNTTNGSIEFVGRKDQQVKLRGQRVELAEVE

HHVRSCLPPGVKVAAEVVKPERGSPTLVAFLSESGTAQPGADMEAAKPSSELTEALAAVE

SSMAEKVPRYMVPASFITLNTMPSLVSGKTDRKRLREFGALMIQGSPGRAPLSEEENEEP

QTELEKKLQRAWHKVLGSNAVIFRGSSFFSLGGDSLRAMRLMATARSEGITLTVADVFGN

PVLRDMAKKADICMQEVSADVAPYSLLESSWHVSSAQKEVALMCGIHADQVEDIYPCTPL

QEALMALSAKVKEAYVAQRVVELDSIETAGRLINAFATASKESAILRTRIVQVPGRGLFQ

VIVRDDFNFHQGDDLSLYLASDRDKAMTLGQPLVRYAIISDTLKGKVSFVITMHHAIYDG

WSMPLVVERVNKAYHSKTLTRPAEFRSFIRYLSDLDRSASASYWRENLKGASSNQFPRMP

KAGYQTKADSLLEEYIKVPGTLPVNTTVATLIRAGWALVAAQYVGRPDVVFGETLTGRNA

PVVGVEEIEGPMITTIPFRVRIGSDATIAEYLQEVQSQTVAQIPHEHFGLQHIRRLSPDA

LGACDLATGLVLHPSGEDKEETIESSPADLLVPAGDAEAAQEALKFNTYALMLVCSIDPK

GFLAMASFDSNTVEVPLMQRILGQLARVVEMLCDPNVSSVSEIEALMCSDLSSSRSMRDE

GALLTLPDLPPFEGAYIVDEQISATVLPLGAPGKLIIRTSSTVDLPEVQTPPFFKRADLP

LPSGHFYDTGKLAVISLSGTISVLEAPIAAPPSAHRPNIRTKRLESSFTRREQVLRTLWS

HVLQVPEADISRDDSFFALGGDSIFAMKLVSEARAKGITLTVMHIFDNKLLKDMSKVMVE

DSSAGKRDELVVPPFGLLDLDNKDAFINSVIKPQLSDSSWAIQDVLPVRPLQGIAVKGTV

NIPRYSARYELIKFNTDMPTAGIRKACQELVARNEILRTVFVEHQNKMYGVVLDSLEVPF

VEYNVDGDVNAFCHQVCRLDVMTRMPLGSSFVKWFFVRGDKGSSLIFRISHSQYDENCLP

IMLQQLSAIYENKPIPKSSSFSRFVAHIIRENIPASIPYWEDLLSGASMTMLEPDTPITQ

RSHFAIEESVDLTGWSSDTTLASLPTAAWALTLARQCETDDVLFGEVVSGRKTDFPDAHS

VTGPCWQYLPFRLVLSRDWTGHDLLRAVQSQHVSSAAHEGMSLSEMAELCNIPGLKRTDW

FGSVVHQAVKPVKSLEIKDAGGETETVYVHEEPLREWSVQAFFDERELTIEVITLESWKD

HAADLLKDITATARHLINNPSMKLF

>SoG_01801.T1

MASVARLAGRGAKRLCLRPAPFAAARSISTTAQRRYATPDLATRLMPVDSDFGDPKDPYG

IARDVVNAARKKADNSVADRKVRHYTVNFGPQHPAAHGVLRLILELNGEEIVRADPHVGL

LHRGTEKLIEYKSYLQALPYFDRLDYVSMMTNEQCYSLAVEKLLNIEIPERAKFIRTMFG

ELTRILNHLMAVLTHAMDVGALTPFLWGFEEREKLMVRTIMMIYLENSDQQLTPILQEFY

ERVSGARMHAAYVRPGGVHQDIPAGLLDDIYQWATQFGDRLDETEEMLTDNRVWIERLRG

VGVVSAAEALNLSFTGVMLRGSGVPWDIRKSQPYDAYDQVEFDIPVGTNGDCYDRYLCRM

EEFRQSLHIVHQCLNKMPAGPVRVEDYKISPPPRSAMKENMEALIHHFLLYTKGYAVPPG

ETYSAIEAPKGEMGVYIVSDGSERPYRCHIRAPGFANLSGFDHVAKGHLLADAVACIGTM

DLVFGEVDR

>SoG_01817.T1

MRHPQRPAFSGLFVRGYATAGGAADHLSSSIKDSDPRIAEIIKKEEHRQRHFINLIPSEN

FTSRAVLEALGSVMQNKYSEGYPGARYYGGNEFIDEAETLCQQRALEVFRLDPEKWGVNV

QALSGSPANLIAYSALLNTHDRIMGLDLPHGGHLSHGYQIPSKKISMVSKYFETLPYHLD

ESTGIIDYTRLRQSALVYRPKLIIAGTSAYSRLIDYAQMRQIADEAGAYLLSDMAHISGL

VAAGTLPSPFDHSDVVTTTTHKSLRGPRGAMIFFRKGVRSVDKKGKEIKYDLEGPINASV

FPGHQGGPHNHTITALAVALGQAAKPEFRKYQETVLSNAQALARQLSDVHGYKLVSGGTD

NHLLLVDLRSKGVDGARVERVLELCGVASNKNTVPGDKSALKPGGLRLGTPAMTSRGFNE

ADFERVADIVDRAVAITSTIDKQARSAAEEQGVKNPGSVRAFLNHVGDGTAILEIGALRE

EVAAWVGEFPGPWTVPP

>SoG_01833.T1

MLKKQQQQPNMAQLGIPRGSTPPLESRASTLADEDLRTDNNKTDASVLDDNTVVDTASRT

SGDKDPEKAQQEAAPAPGELREDEYPSGYALISVVAALVLSIFLIALDMTIVATAIPKIT

QEFKGLDKVGWYGAIFFATIGAFQSTWGKAYKFFDLKWTFLTALLIFEVGSVICGAAPNA

EALIVGRAIAGLGAAGLGSGAYTIIAFSAPPSRRPAFTGFLGASYGLASVLGPLLGGAFT

DHVSWRWCFYINLPIGGVSAAIIFLFFSTPRTAIPAKATWKEKLLQMDPAGVVLAMGGVI

SYTLFVEYGGIRYPWSNSTVIGLGVGFILIWIAWFAVQYFNGERAMIPPRLFKKNATFVM

YAFFFASAFFQAIYYLPIYFQSVHGSSPTNSGVRNLPLILAVTIGTVASGVWISAQGWYQ

HLLIGGAMIATIGSGLLYTMGINTGVGKWIGYQIVAGVGFGAGFQVPMIAVQGTSDPADI

SAVTGMVLFAQSIGGAFIVSGSQSAFLNTMVKYITARSSEITLGDLVLTGASEIRNAFTE

AQLPLVLDGYMEGIRVVFAMTIAITGVATLISFTTRWTKLNTANLTGAA

>SoG_01858.T1

MSSSEDKYETLEKIGHGSFGVIRKVRRKADGFIMCRKEISYLRMSQKEREQLHAEFQILS

TLRHPNIVAYYHREHLKISQDLHLYMEYCGNGDLGRVIKDLALKGQRAQESFVWSIFSQL

VLALFRCHYGVDPPEVGSNVLGLTQGFVAGTPKVPAGTMTILHRDLKPENVFLGEDNSVK

LGDFGLSKMIKSHDFASTYVGTPFYMSPEICAAEKYTLKSDIWSLGCIIYELCAREPPFN

AKTHYQLVQKIKEGKVAPLPEVYSAELNQVIKDCLKVNPDRRPDTTQLLNLPVVRLMRKE

KEVVDLNKSIRSREEALRKKEKELNEALANVNREKEAMRHEIDSQLRREWEVKARLEIDR

LTNAEIEHLQKRFEEEVQARVQAELQKKTVTFKIEGERSSTPQDDFSSSSKSDYPQSSIG

ASGDEFPSTTDITEYSSLDSPDTSRELKKPNTRTPFGRAQTMYAGHAGTPMDIEMMSPSP

AAIASLSLSPRRGGATKAPSANTGNIFAAANARDPDTRWDIPRDLGLIDSDDDDIMPSPT

RNIKSTKNPFTSKTRPVLTSQKSCPLNRLKTKSSSSGLISKQTGQVATPAGTASASTSPT

RRISKIPSAATLQSDGSALSNSPVLGRKPPAKNGASGSDSDSSSALGKVTAKNNLKGRTL

VELQQARAGGRPLSAVMGAPAAGAGENNGSPKRAFRDRIERRLSVESVAVWDPERDEMPS

PFLVRGRRAIIKSDS

>SoG_01863.T1

MATRSLLSRGSPLGSRCVAPAARQSCQIRPALPARYKSYKSTNPRPVRKQDNFENPLPAG

LKTARRIQQLNNVPGGIPAQWEDFTVRHIGPRDDDVPKMLKELGEGAETMEGFLNQVIPE

SVRLPPWEPGLPSSLSETRMANLFNELAETNAKDQIWMQGGGYYPTETPPVIRRNILESP

AWYTSYTPYQAEISQGRLESLLNFQTMVSDLTGLPIANASLLDEGTAAAEAMTLSLNTLP

ASRAKLDGKTYVVSHLVHRTTVEVMKGRAEGFGIDIKVIDLSAPDALAQIDELGNKLVGA

LVQYPDAHGGVQDHKALADKVHEHGALLAAATDLSALTVLTPPGEWGADIAVGNSQRFGV

PLGYGGPHAAFMAVKESSKRRIPGRLVGVSKDRLGRPALRLALQTREQHIRREKATSNVC

TAQALLANMAAMYAIWHGPNQLRNMAIKNVLYARAVAELFRHYGHEVISAPEGSQILSDT

VTIRPRGGRAANASFRRCFEKQKLNPGRLAQDGAWTFACLAEFGPEVFWEIAYALSNHQF

EMDPTSDGVSPRARNYKVAETYKSAPIRDIWNQAFSTSAETFVESIPESLRRTSSYLTHP

VFNSYHSETEMLRYIHHLQSKDLSLTHAMIPLGSCTMKLNATSQMELIGNPKHANIHPLA

APHQAASYNIMMSNLKSHLCKITGMNAATLQPNSGAQGEFAGLRAIRQYLNQSSGGKRDI

CLIPSSAHGTNPASAAMAGMRVSIVKCDPETGNLDMADLRAKCEKHKDELAAIMITYPST

FGVFEPDIVEICKTIHDYGGQVYMDGANMNAQIGLTSPGALGADVCHLNLHKTFCIPHGG

GGPGVGPICVKSHLQCFLPEGKGQVPIASAANGSASILPISWAYVSTMGTNGLRKATEMA

LLNANYLLARLKPHYPIVYTNANGRCAHEFILDTRPFKATAGIEAADVAKRLQDYGFHPP

TLSFPIANTLMIEPTESESKEELDRLADALISIREEIREIEEGRQPREGNVLKMAPHPQV

DLIEGDGEGAWNRPYSRSKAAYPVSGLKERKFWPSVARVDDTYGDTNLYCTCPPIEDYVD

EQ

>SoG_01885.T1

MPSNMFSRLGGQMAAGRSFYEELRARDDGIEVDDALRLDEENLRHHFSDDEAATGIGPGD

SRITVASGPIGVHARPLGQLQPHLEAGGTKWPPLDDDFDNDVPTSLLIEPNDRGHKKQQG

NQRYPQSGGSEAGPPTKQAQEMWNAATTQQRVHSDDLHARPIRGRPRSVLKAVKTENAKD

QAMWRWVNVSNLDSFVRDVYNYYEGGGLWVIQSSNALQLLKTLFVATLLTFLGQCVDYSK

LPQSKSLNQVVVPQCTRKMSSLWSIGIWFYSFYFIWKSVQLFFETRRLLHIRDFFVHLLE

IPDQDMQTISWQDVVARLMALRDHNPKTAANIPSRLRGFLGSQSKERLDAHDIANRLMRQ

DNYLIALINKDILDLSLPVPFLRNRQLLSTTMIWYLQYCILDMAFNRLGQVTQDFLRADR

RGVLSQKLRQRFIFAGIMNLMFAPFVLAYVVVVSFFTYYNEYQKDPKMAAARKYTALAEW

KFREFNEVQHMFYERLHMSYPFATRYLDQFPKRVTEEIARSIAFMSGAITAVLAVLTVFD

SELFLGFEITKDRTVLFYLGVFGAIWAMTRGIVSEETTVFNPEYALRNVIDYTHYMPKHW

EGRLHSSEVKQEFSELYKMKVVILLEEILGIITTPMLLLFSLPKCSDQIVDFFREFTIHV

DGLGYVCSFAVFDFKKGVGDTGQHEHGHDVREDYYSTKHGKMAASYYGFLDNYVINPKTG

IPGHQPPLARQQPFFPPPIFAGLNSPSLMGDLQASLGGRGETGRSRSRVQGFGPVPSRTL

RGGKAGMAQPSPVASILLDPHHQPHSASLVRSLHQSRHPRAVYRGEGQIPEEMLESRGQS

QIGIDDDLQEGTGILDESTWETSPGKGLSRENSAADPGEPEAGVLGMIYQLRETQRNRRG

TSMV

>SoG_01888.T1

MSSQVAQAARRVNHDLRGIVVSAGLMQKTVKVRVGGQKWNKIVNKWFADPKHYMVHDPNS

SLRTGDVVSIMPGWPTSQHKRHLVKKIIAPYGVPIEERPPIPSLEDLITEREAKKAAKDE

RRAKRQEDLEKDREEKSRKLHERKEEKRLAQSGFWTESKTFVEKQL

>SoG_01892.T1

MGAQQSSSSAGAGHANLSSASKTCYYELIGVSRDATEDEIRRAYRRKALELHPDRNLDDV

ETATRRFAEIQSAYEILSDPQERAWYDDHRDAILSGQDGSGDGTEATTFRNVRLTTAEEI

MSLLRKFNATVPFNDEPNGFYGIASETFEHLALEEEVAAEHADIDSPNYPTFGSSDDDYE

TVVKPFYSGWAGFATMKSFSWKDKYRLGDAPDRRMRRLMEKENKKLREDAIREFNDAVRF

LVTFVRKRDPRYVPNTQSDADRQKSLRVAAANQAARSRASNNKKVSEYHLPDWVQQSHGQ

DEFFSEESEEMEEEVEILECVVCDKTFKSENQLEAHERSKKHQKAVQQLRRHLRKEGKDL

DLDTLEPSEPEATGNKGVESLDGQDLDFAPDEGNGSATQDPDEVSVAPSNSDSADPAPVS

VANLSISDDTSDPIKKLGTQESTSTQPRETAIDESCVKRVGKAKLKRERKAAAQKQNSGE

CAVCASSFTSKTKLFNHIREEGHAAALPVSLGKGKKNIR

>SoG_01893.T1

MANVDLGRYRRIVQMFWDPEPTNDTSLDQPVWCLGRSYKLKPTQERKEAGPVKRANTAGD

QSRRSPSTIPQSVTSDTDPSSGILASPANAPDTPPESNSSSSPSSLAYGEPDQDNGRWPQ

GFIDDFESRLWMTYRSDFEVIPKSTDPKASASLSFSMRIKSQLRDPAGYSSDSGWGCMIR

SGQSLLANSILSLRLGRDWRRGMKPLEERSIISLFADDPRAPYSIHNFVRHGAEACGKYP

GEWFGPSATARCIQALANSTEQNLRVYSTGDGPDVYEDSFMKIAHPQGESFHPTLVLIGT

RLGIDKITTVYWEALVAALQIPQSIGIAGGRPSSSHYFVGTQGSFLFYLDPHHTRPALPY

HSDPTDYSDEDVASCHTTRLRRIHVREMDPSMLIGFLIRSESDWADWRRSIKHVQGKAII

HVSDKAPMMYGSAEGREGAIDEVEPLSDDEQTVHGT

>SoG_01896.T1

MTGGGKSGGKASGSKSASSRSSKAGLAFPVGRVHRLLRKGNYAQRVGAGAPVYLAAVLEY

LAAEILELAGNAARDNKKTRIIPRHLQLAIRNDEELNKLLGHVTIAQGGVLPNIHQNLLP

KKTGKAGKDSSQPI

>SoG_01901.T1

MKFSYTGFLLFASSALGASRTKAPSGCLTVSPNGTYKTVQSAVNALSNSSTSDQCIFIDR

GTYNEQVLVSSRSAPRFTIYGYTTDDRSPRANGATIVFGLSQKDGLNNDGTATLRVKANG

FRLYNVNVKNSYGQGSQAVAVSAYADSGYYGCSFVGYQDTLLSNVGRQIYVDTQITGATD

FIFGQQATSWFERADIRVVSASLGYVTANGRDSSSNPSYYVFDHSTISAADGNSVPNGAY

YLGRPWRSYSRVVFQRTSMTSVINPAGWRIWNTDQPNTSNVFYGEYQNTGAGSQGTRANF

SRKLSSPIAIGTILGSDYTQQKWFDASYFAGKGADVGST

>SoG_01909.T1

MIMSGKKAVHFGAGNIGRGFVACFLHNSGYEVVFADVMDALIDKINSTPEYHVIEVGSEG

TTKSTITNYRAINSKTHEEDLINEIATADVVTCSVGPNILKFIAPVIAKGIDRRAEGESP

LHVIACENAIGATDTLAEFIKSPQNTALERLDDHHMRARYANSAIDRIVPAQDPNAGLDV

TLEKFFEWVVDKTPFQDIGIPSIEGINWVDNLEPFIERKLFTVNTGHATAAYHGYNRQKK

TVYDALQDKDIMAEVRGALEETKALLVSKHGIDEASQAAYMNKIISRIGNPHLEDANERV

GRAPLRKLSRKERFIGPAAELAENDMSCKFLLDAIEMAFRFQNVESDEESKELAKIMTEN

GPEDVVTKVCGIRSNEKLYPSLVEVVKRVQSDSQE

>SoG_01993.T1

MTANGTNGIKQHVEGSFLFTSESVGEGHPDKIADQVSDAILDACLAEDPLSKVACETATK

TGMIMVFGEITTKAKLDYQKIVRDAVKDIGYDDSAKGFDYKTLNLLVAIEQQSPDIAQGL

HYEEALEKLGAGDQGIMFGYATDETPELFPLTLLFAHKLNAAMSAARRDGTLPWLRPDTK

TQVTIEYKHDGGAVIPQRVHTVVISAQHAPDITTEELRKELKEKIIKKVIPEKYIDDNTI

YHIQPSGLFIIGGPQGDAGLTGRKIIVDTYGGWGAHGGGAFSGKDFSKVDRSAAYVGRWI

AKSLVAAGLARRALVQLSYAIGVAEPLSIHVDTYGTSDKTSDELVEIIRNNFDLRPGVIV

RELNLDRPIYLQTAKNGHFGTNQSFTWEQPKQLKF

>SoG_01994.T1

MSSKFGVMVMGPAGAGKSTFCAALITHLQLNRRSAFYVNLDPAAESFEHTPDLDIKELIS

LRDAMEEAGLGPNGGLIYCFEFLMENLDWLTEALDSLTEEYLIIFDMPGQIELYTHIPIL

PTLMRFLSQPGALDIRMAAVYLLEATFVVDRAKFFAGTLSAMSAMLMLEVPHINVLSKMD

LVKGQVKKKDMKRFLTPDVALLEDDPLERTRRIEEGPAGEDDESQPVDDKKQVMKGASFR

RLNRAVAGLIESFSMINYLRLDVTDEDSVGAILSYVDDCIQYHEAQDPKEPHDGEFEEQD

QA

>SoG_02002.T1

MASNGAGAWKTPIVTSPRNKKNDTSFPGLRPQGQDPGSKIEYEELQQNEVMALEAIYADD

FVNHTSVQQSAWKVGRHRRNGGLEIKASTNEDFAVTIGFVLTATYPRTPPVLSIKNNELP

AAITFKVQKFLETEPRLFAREPQEMVDKIVEGVRDILEDAALAKAQGALLPSLEQERERH

EAALAKQAEEQKQEDERKRLEETREEERVIAEMLQQQLDRQRQKHRESKHGKRSNGVGDH

APTMSADNPPDKVDFDQTCNAVDKAGNAMSFRSVVGKTDPRHGPVSVVYSVRPLLPSGQS

SSAMALKEAVLRPKEKDSKDFKKQLQSLESHLQDLKAGKKIQHRHIVDVFDFKVQSGLVH

DLSASNVWTISILGPLAEKGSLEELLELAGRLDIGKVRSWTRDLLDALNFLHNRNLAHQD

IHPGNILLFRETTGEIIPKISDALYQRELHTISARKQAPPGFGSAKSAYWLPPEIAATSK

PQYTHKTDIWDFGVVFVQMIFGLDILQKYSSPRNLMESLFLSHSLQELVGRFFKEDKQKR

PRAFELGASEFLATDAPVLQEEVAGVMSSSASHTSMPAMPGKFRRDSTTRGIASSRFTED

FVEEGRLGKGGFGEVVKARKKLDGQIYAIKKITQRSQASLTEILKEVRLLSQLSHPAVVR

YYNTWVEEIPNDTDTEGETSTEAATETQDTASAGVDIQFGTSTGGLDFMSSNAGVEFDYS

EDSESDADYDESTDEEDSTDVEAGDQALSPEKDRNSFLQKRARFQRQYRTVLYISMEYCE

KRTLRDLISRGLHKNATEIWRLFRQILEGLAHIHSLSIVHRDLKPENIFISISSDGVDNV

KIGDFGLATSGQFSIDKSVTNLGESDDMTRSIGTVYYSAPEIKSSANGMYSTKVDMYSLG

IIFFEMCYLPMIGMQKADVIGHLRRPKPVLPADFKPGDKQTDIVLSLVNHNPKERPTSAE

LLKSGKLPVQMESETIRRTLAGLADPSSPYYGKMLSTLFARPVEATKDYAWDMFATTISP

AELLNQGVVKQALTAIFRRHGALECTRSSIYPRSSHYGDNVFHVLDQNGTVLQLPFDLTL

GHARMLAKQSSGPVMSRTFTFGSVFRDRQDTGQPQIFGEVDFDIVTTDTLDLALKEAEVI

KVLDEVIETFPSLSSTQMCFQLGHSDLLQLIFEHCAVEPANRRAAADVLSKLNIHSHTWQ

KIRSELRSVAGVSATSLDELQRFDFRDTPNRAFSKLKTLFEGSDTYQRASPTIAHLKEVA

EYCKRLGVSTKIYVNPLNSLKENFYVGGILFSCLYDKKGRDVFAAGGRYDNLIKEQRPKM

GGQLQERHAVGFSLAWERLARVPKSGGKAFLKKAEEDTTGLFNGKRCDVLVASFDAALLR

SSGVELLQTLWAHNLSAELAKDARSPEDLMAKHRDDSYSWIIIIKQDAIIKIKSVGKKDV

ADVDIPATQLISWLRGEVRDRDSRALTKVRGGSSDNTGAGIDKEHEQSVKVLVSQTKSKK

FNRRTVVEQAQASAATLVESFLNGPILAVETTDQVMDLVQDTSLGEPESWRKVEHAVTTM

EKKYVREIHDQLEAWRFSYEQESGPRHAFVYNFRSGHTVYYDLGS

>SoG_02020.T1

MALANPHSREHFALTMISNPDASSAPNLLHDAGRALPAHPPQQGRDQHREIDYSPRLRAR

TAMTPKEDNGPEGQLSYPSPGADGMDAGPYYTTTGREDDATQEMHDQGAEHVPEHNGSDL

HDPNATHHDPSHEAHHDVHQDQQNGRPANLEELQLAAQLGQGLTEAPIMAGPEASMEDPN

LRSILPHPEPEHQDHNNQSQPQYMGPEPSAAEAMAAHGMPVPVPPQIPPHYSLPNDHVPP

RKRSKVSRACDECRRKKIKCDATSDSGENPCTSCAKSNVRCLFSRVPQKRGPSKGYIKEL

ADRINSIESKLQSEDGLGHEDLEKFFAQERQRSSTGPGEDASRKRPFSSISGNEFSTPTS

NHRQVPWGSEARGPQSESFATYSNTSLAPQPSPIKPDVASSKQTVAGDDMPMPDDEGVPE

VGDNILHDYNTLIQPLYPVLPDDRSKLQSLLVQSPSPVRTGFLNAVMAIAQPAGGNAKLA

SSLLNDWENSDNPRTPAANIVHAQALILLVIDADMRGSATLPFLLARAVTLANSMKLWKM

ADMAAAAEPDSDMQLCVRIWWSLVLLDRWHAAGTGKPTLIPDSSVVIPAGLDAIVGESRY

YLIRLSKVLGKVAATIASLPPSGTAEPMAATILSDYVENFREDLPSHIDSSSFPLVHQAY

WHARLLVTLLHPVISGTPNRLLWPTSELITLLTSEPDVKTPLTHHFTSLLVLALTRLSQL

DESKESALQLARDVVDQPGAAVWDDVRERLARLTRPISSVEAAASQGLQHLADLATAGQG

GAAVGEETGLPGQGSSLAVGYLELVVTNALHPHQSTGIPGSLESPSPDHGRAQPSPISHF

QSNSTDST

>SoG_02038.T1

MQNARGILSCASCRQRKLRCDRGEPCHNCETRKIECKYAPIPRGRVVVQRQDDNRQELES

RIRQLEKLIGNLASQNGSNGSASTVAASTRSYDSGSAPGPAPPSVTSTASSQSEIKSGRL

IENDNQMIYVSPDHWAAIHDEITKLRAHVELDSSEADVIAEQVAPDRGLMLLEGLCPPCS

LEEILFDIPARPIADRLVSKYFTSRDRLIVLHGPNFLREYREFWDQPAAAAPAWVAMLFG

VMLGGAFLYARACEALPEGMASPGEVLENFQRRSAECLVLSNYSTAPGKYTLEALLLHGH

SEFIRRPDAQVGLWVLGGVAIRLALRMGYHRDPSHYPHFTPFEGEMRRRMWSNIRQLDCL

TSYQVGLPSMLQNRHVDCLPPRNIFDEEISVESTELPPSRPETEMTPILYQIVKVRLLAV

FDEIFDQTSLTEMSDRYCGRVAELDRRLSETIASVPPDLRLATPQDSLMVHPNVLIRQYN

IELLGQKARCILHRQHMARSFQDPKFDPSRKACLGAAMALLKYQMDILAEVQPGGLLFNE

RWFVTQLEHSDFLLACMVVLLEVSNRGKRRDASRGLEGMYSTTQLMDAVRRSQEFWYIMR

DRNRKAMQAYKTMSGMLTRIMVDPEHNATAPTNTGSSGRKDENSLQESVSPLTSGAGVAG

HLGQSYDHHMDPSLFDPSMMEISDMLISPALVDWESWESYMQRPLS

>SoG_02044.T1

MAPFDPSVITKAGDSEAVPELLKKLNLLADKEGDEARQDMKDLARNLWLALETPREAMIR

QCWAEPGAMAAITTCQHKGIWNYLVENENGPFNANDVAKVKGIDPPMICRMMKHLSAMGY

LKEVSKDTYNLTNHTRSLSVPIIGDPYPCMMVGCYGAINNFHVWADKNDWKTPSGPVGGP

LQMAYKTELNFFEHLQNVGCGEQFNSMMGGYHQGRASWMDSNFYPVQERLVDGFDRDNKD

AAMLVDIGGNVGHDLEEFGRKHPEAPGRLVLQDLPVIIGQIKKLDDRVERMEYDFYTEQP

VKGARAYFMHSILHDWTDDICLKILANITDAMKPGYSKLLINENIIPDTGAHFEHTALDM

MMITLLSSKERTRADWEDLLTRAGLKISGVYTVTRSAESLIECELVDGATNGSSNGANGA

NGH

>SoG_02046.T1

MLLSQTWTLMKKNFRWLLFRHAFLCIWMAFLLPLFLCTFFAFSRNLFVPPATYGIGDASP

LRSLSNALRASSGRNKVILVNNGFTGGEIDRALGRFGDQVEASGTGTQVIRVQREDDIYR

ECRSSLRGVTTCFAALVMYSSPTEGDGSIWNYTIRTDAEFARSPLRVEIRKTNNPVQVYI

LPVQRAMDGIIANLNNTADAGVLDNTRELPFTDRTQEQREQRIRQIFHEAIVNFMGVLFL

SAVLWITYHMTGYIAAERESGMSQLIDAMMPVRQPWMALAARIVSHHFSFTIIYAPAWII

GSIIVKSGVYANTNLGIILVFTVLGGISMASMSVLYASFFKRAQLSGITAILATALLGIL

AQSLTDPGTGIVAVLSALFAPCAFVYFLCTISHFEEKRRPGSLTEMPPDSSWQIPAIVFW

VFLIIQIFAYPLIGALIERHLYGTSTKGRQVIVNDTSVLGDSAVQLQGFSKTYKPSLLAR

LLPFGAKPQPVHAVKELTFDAGRGQIIALLGANGSGKSTTLDAIAGLNKLNSGSITIDGT

GGLGIAPQKNVLWDDLTVEEHLRIFNRLKAPKAQRATREEITELIAAIDLSHKTKALAKT

LSGGQKRKLQLGMMLTGGSAVCCVDEVSSGIDPLSRRKIWDILLAERGRRTLILTTHFLD

EADLLADHIAILSKGTLRAEGSSVELKDRLGGGYRVHVPKHAHLPPQMPHIEGVRKKNSL

DLISYIAPSSFLAAEVIKALEAAGIHDYRFSGPTIEDVFLQLAEEIRDEEAFRGTDRTAN

EKEKIDDDDDSMTTPNREGLKLMDGQRVGYVKQARILFGKRITILKRNWLPYALAFLLPI

LAAALTSLFIKGQTPPGCSPEDQASRSENENAFTQASDSNKRLMFLAGPSSSLSQAEFIR

LFTPIFGRSLSGGSSGGEAETLAGITLVDSYDNFTRTIEQRRDRVSTGFWLGDQSNPPTF

AYVANLYVASGIISQQLLDVLRTNVSISTQWSPFEVPFSPSTGDSLNLIVYMGLALALYP

AFFALYPSNERRRFVRGLQYSNGVRPFPLWISYLLFDFIFVIVSAALVTAIWVGMSNIWF

HIGYVFLVMFLYGIAAALMAYVVSLFTKTQLATYAWAAALQAIGFLAYLIAYMCVNVYVA

AAKIDNTLLIVHFVISAFFPIGSVMRALFLTLNLFSVACDDTVLSTNPSGILYFGGPILY

LILQSFILFGLLLWLDSGSAGTSIRGLFGRSKAPENSEASDEELANELTRVESSGAREYP

LRVKHLTKSFGKYTAVENISFGIKRGEVFALLGPNGAGKSTTISMIRGDIKPSRNGGDVF

VEDASVTNELTAARRNLGVCPQIDALDSMTVREHLEFYAKVRGIPDVPHNVTAVLQAVGL

QSFASRMGHALSGGNKRKLSLGIALMGNPTVVLLDEPSSGLDAASKRIMWKTLADTVPGR

SILLTTHSMEEADALAGRAGIMARRMLALGTPDDLRERFGDALHIHLVSKTAPRTTDEEM

QRIVQWVHATFPSADVEENTYYGQMRFSVSASDVRRARRATQVDQITQHGTVAASDSEGT

SRSAIGNLAVILEENKEALGVGHYSVSPTTLDQVFLTIVGQHNVKEENSGVAQGTPLWKK

LLFLKF

>SoG_02050.T1

MAVIYIDEEKGVDAPDTQGTEAAPFKSLAQAYLEHGADNEYKVKKKDDEEYKPAAKAAMK

KAVAYSDQQRKKKEAAAKRADKEAAEKAALEAALEAAKNIKISEDPALPKAVNIALQETD

SKIIGTLRKSKDEPAEGVVRVCVQGRVQRVAKQGGLVFVVLRRGLNYMQCLLSGELAKTY

DALTLARETSMEITGELWEVPAGLHAPLNRELHADYFRIIAKAPGGDDAFTNKVPEDGDP

AHSSMLDLRHLTLRHEKPATVMFVRDVLEGAFNKAYRELQITKVSPPALVQTQVEGGSTL

FKFDYYGETSYLTQSSQLYLETCLPSLGDVYCIEKSFRAEKSLTRRHLSEYTHIEAELDF

ITFDDLLSHLEHIICRVIDLTLENPTAAAAIAKYHPEFKKPQRPFMRMRYSEAIDWLREK

GIKNTEGTDHVFGDDIAEAAERKMTDEIGRPILLTHFPVEIKAFYMQKEKADPRVTESVD

VLMPGVGEVVGGSMRIWDHDELMAAYKREGIDPAGYFWYVDQRKYGSTPHGGYGLGTERL

LAWLLKLWTVREACLYPRFMGRCTP

>SoG_02067.T1

MPSAPVDSKPASAASSKPSSLKSQDAPARSPHSTPQNGTSSPVNGATSPPLTPDGSLKPL

EPKASVKERFTRMFSNKGDIPKVTPPASHNAAPSSQQGESSGRSRGSSLNSASGPVPAPA

TPATATAAAHAPAAAPAAVAASQRDKTSQRKESYSDQGSERSAVPAKPQRFVLLPESQGS

GHEHHLKSSRRQEKLSDMWRSLIGKKHEPPPENDLSLVSSWVDTLRQEKEAASEEKKTGV

AAPTTLVEKYGKCQEVVGKGAFGIVRISHKKLTTGNGEKLFAVKEFRRRPEETEKKYSKR

LTAEFCISSSLRHPNVIHTLDLLKDAKGDYCEVMEFCAGGDLYTLVLSSGKLEVQEADCF

FKQIMRGVEYLHEMGVAHRDLKPENLLLTTRGAIKITDFGNGECFRMAWETDAHMVSGLC

GSAPYISPEEYTDKEFDARAVDVWACGVIYMAMRTGRHLWRVAKKDDDEFYARYLAGRRD

EEGYGPIESLHRARCRNVIYSVLDPHPSRRLTAAQVLRSEWVREIKLCKAGEEGL

>SoG_02072.T1

MKRSRGSEEDFSSDSDSLNPPGTAKSVSEFHRVSKIAELDAAVLDTNDETVAAMKCYLPP

HKEPLTFGTYEDYELHYRSYHTNRCLDCRKNFPSEHLLSVHIEECHDPLVIVRRDRGEHT

HMYPKNYFFGITKDGIDGRRSLLNDGPHSRRRSSASIPSKESRPRKNLVESQSLNQPDGH

EQAASKGGTKPKDGSTGASVQSDTAMMDLTGAMSALNFVPPSVRFGKGRAGFAKR

>SoG_02075.T1

MGRVIRNQRKGRGSIFSQCADSSLLLAAANTRLNKAPAKFRNLDYSERHGYLRGVVKEIV

HDAGKYPDFLTQRTRKHRTRSNLGLTVFLITGRGAPLAKVVFRHPYKFKQVTETFIANEG

MYTGQFIYAGKKAALTVGNVLPVGEMPEGTVVTNVEEKIGDRGTLGRTSGNYITIIGHNP

DEGKTRIKLPSGSKKVVHSSSRGMIGIVAGGGRTDKPLLKASRAKHKFAVKRNSWPKTRG

VAMNPVDHPHGGGNHQHIGKASTISRYAAQGQKAGLIAARRTGLLRGTQKTKE

>SoG_02094.T1

MPDASIPDIGRSSSHLQLQQQQQQQQQQQIADTALSDDNAAGAKKTSSSSAAAAASAAAE

EEAKKARACEACRGLKVKCEPDPNDETQPCKRCKKAGRSCVVTAPSRKRQKKTDSRVADL

EKKIDALTATLQARSSVAAGHQGTGLGHGHGTHSIAEEDAVAAARTDAQPWGGVPPAKAW

HSPSMPSHQDRDRQSSGAGVARGTSSLGTAPPNAVAGQKRKDRDSVGSLDQISERTRQAY

SQPAPVSYKSRNPDFVERGLITMEQADQLLRKYNERMLPHFPAILIPPGTTAAELNRTRP

VLFASIMAAATTDQPALQKTLQRELMICFAERVFLAGEKSLDTVQAILVAVIWYWPPENF

EELKFYQLIHTASVMAIDIGLGRRGGSRRAEPPFGLRDPASKKPAPPDALTLECRRTWLA

CYLLGTNAAMALHRPVLIRWSSFMAESAELLEKSPDALPSDRALAQLVWAHRLSEETGIH

FFSEDPDVRINLADPPTQFLLRGLERELERRRAAIPQEKQTATVSMTFHTVNLFMHELVL

NSSAASDQGRPPFSTESLTVNFIGSDSPLSAAQISSLTACLVSIHGVCNIFLSIPIDSAR

CLPVYFFVRVAYALVILIKMAFSASKAGSELGRIIGPDDIRVQYYLDALVGKFRELSMDD

QCRPAAKFLIVLAMLRTWVMRQVKDGTFSVSSKSSEPLQAARHKQTEDPNIISPPPPPPP

PSTHRSSPSQQPASTPLHLLSEVASNRNNSTSAGTGGSSSSSSSGVPRPELITSQFFKGI

SRVPQPFFANDSATATAGPASAVAADSTSSTASAGSGATGGADINMNAWMPGGMGFAPPM

PLDPSIMNGSLDLEGLDFATAEPWFTDVFNVMPNMENMFYF

>SoG_02096.T1

MLSNAILALGALAATVNAAPASHSSHGSKSRATNCEFTTLSALENGKSNCDTIILNGISV

PAGQTLDLTGLKTGAKVIFEGNTSFGYSEWVGPLIAASGNQINISGASGHVIDCQGQRWW

DGKGGNGGKTKPKLFSTKGLNFSTIQGLNIKNQPVQGFSINSVNDLNIIDVTLDSSLGDT

LGGHNTDGFDVGNAQNIYISGATVYNQDDCLAINSGNNITFTGGTCSGGHGLSIGSVGGR

SNNNVVGVRILKSSVSNSENGVRIKTISGATGKVNNILYQDITLKNIANYGVVIEQDYLN

GGPTGTPTGGVPITQVDLEGVTGSVKSSGTNVYILCAKGACSNWQWNNVKVTGGQQHKTC

ENVPSPAQCS

>SoG_02113.T1

MAGRMVLYKLVVLGDGGVGKTALTIQLCLQHFVETYDPTIEDSYRKQVVIDGQPCMLEVL

DTAGQEEYTALRDQWIRDGEGFVLVYSISSRSSFSRIKRFHHQIQRVKESCASSPSYPGS

PISAASASLPIPIMLVGNKSDRVTEREVSTQEGHALARELGCEFVEASAKNCINVEKAFY

DVVRILRRQRQQVARPAAGAGGRRQTNSDSTPHRDKGRYRRERDGEKRGKSCVLL

>SoG_02115.T1

MALSPVYREPSQALPSIRGPTHDRFGSHHDSPFSSSPAQAIPGMRSHHEAPPPLPPPRFP

LYGGAPQPEEMRRLERDRSHHGYAPSSINSGYESMASSFADDRPAFKRRDTSSPNGDEGY

ASFSTDRSGRSIPKEFGISHNSFKFQSSDLLVDSMKKKLDPARHCERSSKPSGLSLSLDP

WPRSQPTDSRAPPTLSLPLHVPGRPPLESPSRWTDGPTYSAMSPRGFPFPHSPREQRSPR

DSDAERSPQLFTRRKNSDDAASTQGSYAGHSPDEMDVDDNSSLKRLRLDEGYSAASQKRR

APSPREDEVMIGVPYQVAVTRKSDFGPRGSPTPRLSTGLSEPHPSALSLSRSNSYVSTPA

ASGGAPSTSALGRRSPRGLSPNTVSPTSTQSPYTPTMSADPSPRALPSRSLPHGRTTSST

SPRKIAEMQKPGNPKIQGFYMCDCCPKKPKKFETIEELQAHEAEKQYECNYCGNRFKNKN

EAERHQNSLHVRRHSWSCSALSGYDRAFHDSPNRPGEADTCGYCGQDFPRSGMGPGSGAL

NGGVMPRYATEKDIAERNAHLQGVHKFRECNSSKKFFRADHFRQHLKHSHAGTSGKWTNM

LENACMLEEEPPRS

>SoG_02122.T1

MIDDTGSTPILMDLGSVAPSPIPITSQSMALQIADTAAEHSTMPFRAPELFDPQTGMVID

TKTDIWSMGCTLFACLVGKTPFEMRSDETGGTLSLCVLGGDWRFPDEGKQAVKRTNSMRP

QQADGPNAAGDPHSIKISEPIREIVRRCLKVEPAERPDIDELIDMVQGVIEELPEDGA

>SoG_02132.T1

MRVTALASVFVAALGGAHAVAIPEAVPAAQPDRTLSARQTYTSTDPKWTDHDQFRNSLIN

RHNQYRGEHGVGGLTWNRTLSGFAAQYLNKKGNGLNQCPDFAHSGGPYGENLAIGYGTPT

QAEVAWGEERKQYDFNKPGFSSATGHFTQMVWKDTTQLGCARKFCTSGNPYRGWYLVCEY

YPRGNIIGQFDKQVLRGSYRQKRDEELVPREEDEGEEAIEEVAPPEGVEELSGEPLVFFQ

AA

>SoG_02144.T1

MALSPVVTQTLDRSKLAGSPLKIVGTSAQTSANGTPLPAPTGETISSLPPTLDLAEQMND

EEKRKYVKGKKLGEGTYALVFLGHVRRDPSQLVAIKKIKVQKEYTEGMPPDAVRELKFLR

ELQHPNIISLLSVFSSRDQNLNLVLEYLPLGDLEELIRDVDSVRYGAPDIKAWMGMLTRA

IWFCHENFVLHRDIKPNNLLIAADGEIKLADFGLARSFADPHINMTPNVITRWYRPPELF

FGCKHYSGAVDIWSVGTVFAELVLRRPYLPGNTEIDQVRLICENIGTPTESNWPGVSKLS

EYTVVEGHALKTRQEFEMRFGIVGRDGVDLLMKTLSLDPKKRITARGMLDHPWWHAEPQP

TKKRDLPRKGGGEEKMGADLKRRPGKVEDDRGSKVARKLDFGAMK

>SoG_02152.T1

MTTAAQEEFNDLVAKNTPRETIHPEDRDDPDRQYHDDPTEEDDFRNAQIEAAMRSNTLTA

PGAAPDIHLPPTSFDAGHSTGVKGVIADARSFEAARRSKWRSRIQTARQSIFGSDSVDRR

RRAGSSNSNNSLSSSPSDSGTAGTDEEAFLEEWRERRRKELEAEAARGIRNRRTSPSARM

YGRFDEVDAMGYLDAIEKVGRETKVLVFVYDHECEVSSTVESALVPLVREHPTIHFVKVH

YDDIEFDPAAVPSLLAYHNQGDLFANMTGLIEMIPDEDSFGTAALTKILQQNRVL

>SoG_02164.T1

MSGLPPVYIVSAVRTPVGSFLGSLSGLSATQLGAEAIKGAVSRAGIDPNSVDEVFFGNVL

SANLGQGPARQCAINGGLPQTTVATTVNKVCASSLKAIILGAQNIMLGTSDIVVAGGTES

MSNSPHYLPNLRTGAKYGDQTLVDACIKDGLTDAYKKEHMGLAGELCASDNDLSREAQDE

YAIGTYKRAQAATEAGLFKEIIPVEVPGGRGKPNISITKDDEVSNLNEAKLRAMRPAFKP

DGTITAPNAAPLNDGAAAVVLVSEAKLKELNLKPVAKILGWGDAEREPERFTVAPALAIP

KAIKHAGLTDKDVDYYEINEAFSAVALANIKILGLDPEKVNVYGGSVAIGHPLGCSGARI

LATLTSVLKEKNAKIGCVGICNGGGGASALVIENLQ

>SoG_02167.T1

MASFKLSPLTMLLGAVFFFSAQVFAASAVLGVDLGTEYIKAALVKPGIPLEIVLTKDTRR

KETSAVAFKPTKGTPQDARFPERLYGADAMAIAPRFPADVYPNLKAVLGLSVDDSVVQEY

AARHPAMQIFPHPSRNTVTFKSKTFNADEEAWMVEELLAMELQSIQKNAEVTAGDGSIVR

SVVLTVPPFYTTGEKRAIQMAADLAGLKVLSLVSDGLAVGLNYATSRQFPNFSEGEKPEY

HMVFDMGAGSTKATVMKFQSRNVKDIGKFNKTVQEVQVLGSGWDRTLGGDALSNLIVDDM

VAQFIESKGAQKISATSEGVKAHGRALAKLSKEAARVRHVLSANQNTGASFEGLYEDVDF

KYKITRAEFEAMAEAHAERVGATVNDALKMANLDIGDLTSVILHGGASRTPFVQKALEKI

VGAGDKIRSNVNSDEAAVFGAGFRAAELSPSFRVKEIRISEGAMYASGMEWKGNKDKMQK

QRLWSPISPLGGAPKEVTFSEHKDFAIAFYQQVAAEDKIVKTLTTKNLTATVTAFKEKYP

SCDDTEIFFRVGLKLNGDNGEVEVSKAAVECEAEVKEGFVDGMKNLFGFGKKDQEPLTGD

DAEADSVKAEGSEEEVKDGAAKSSATDATGSSSSATDASASGSTEEAKTEIKKKLVGIPV

EVELLQAGAAGLTKEEVAKSKDRLKAFTIADKARVAREEALNQLEAFTYKIRDLLENEDF

IAHSSEAERSKLSEQGSTISDWLYDEGSDASKDDFKAKLKQLQAIASPIQKRMEEAEQRP

GLISSLKEALDQTKDFTGKMLKQMEEYEAWHASSSASKSASSTIASSESTEAPAETPTGE

FDGLGDEASATPKSRTMEDVTEEIGPIPPLYKREDLEEVVAVETNIRQWLAEQEPKQEAL

PATADPVLLIKDLKSKREKLDKAGVDLAMKGVRNFDKKSKKANKSASERKKKTKTASADN

AEKTLKFEDFADSEDGPKVYSGDDIEELMKKVQREKEAWIKAEEAAAKAKGESSGKEHDE

L

>SoG_02181.T1

MEMLWTDPQEEEGRGPSKRGVGMQFGPDITKRFCEKNGLEAVIRSHEVRMDGYEVQHDGR

CITVFSAPKYCDSTENRGAYINIGPDYKLHYEQFDAVPHPDIKPMAYAQSSLMSSLM

>SoG_02197.T1

MKLGKNQARIEGGIASSQVDVKHSKRGSAPLIGNHVNPMSQPARHGTACIVCRRRGRKCD

RTLPFCLNCGKRGVTCEGYITRWPGVAARGKLAGKASPVLDDHGPAAATRSKRLKVIHDP

EHLNKVVTAGSAFPSEHPSQKIREYRGESDQFDETKVDRLVGHYVKDLSGIFYLGGGGGA

RENPMFTYVLPLVDTVPPIRYALAASAACHTAARTSDDALDQESLSLRVHATRLLREMLG

SSARPEYGTMLPSVLMMAQLDMCSGDCTEFETHMQAGVAICRDSESSHSANNYYFEQRLA

WLDIISSTTSDRLPFFTVDELKAALSRRPSTNAGGRDWGYDVFPCPVDLCEMMLDAMTLS

KTRASSTIEPSSEEVAEQARRIMRQLQDWGDVRREPTEPREHLVQAWRLGIMTYLARLFP

SATDSSLPVPSSKATEVIQHAQGIQLATSFSYPLLWPLFQAAVTLDGSAAVEEKTWIRRY

LTKSLEAVGCRHFSNALDTLDFVWANRDRLPFSSTGAYGRTIMLG

>SoG_02204.T1

MYIIGNPPAQPGEGLERLSPRPTNPPNPRIPFTSTPRAVPCRRRMSSKVRSAVCHRCASI

KQGCSGGFPCERCVRLGVPCQPRSAAAAAVVVRSGSSPSLKDVMQGEHWQPLPRARIRRV

HTGCLTCKKRKKKCDETKPSCGNCRRLCLSCSWSPDRFAAARASVGSLAGDGMAGSSSGW

PLDQQSLDQPQQWCFDDGVTAIDGQQIRASQEFSTSLPSTDGGLDASMADLLCQLSAVQG

PFPVDESSTTTTATFDISWTLSPNAQEFGMLPESPSALSTSSSSLSPGDASGDSNLSLTL

HRPSMMPDMTSPLDKALLSHYSTVVTSVLARCPNPSSNPYLVHLLPMAMSNPIILHSVLA

LSATHWQRLLPEMEDRAVLHRGKATQSLALLLPHIDASTIDIALVSCLLLCMTELFDGSS

TGWTLHLQGAKRLFATLREKKRAGSHVRFLVKLARFLDSAATTSTCKPPLIEKQQQQQDV

RALDLEEEEVPLPGEDAAIYGIPKELFHLVDRVNDLASKRGTRVDEASENAFRKQASIVR

EQLDNWALDYGGLAGAVASLGLNRSGGGADDDVLHATTAEGMRIAHRRGGAEDPVRQPAG

DVPPVPAGDGGRRVRAARGQDRDTRQARGDGEDLRVRVHPPEQGARGEGVAATQGGGRWR

GEGELGEDKVRGDGRAGGVLKRKIPG

>SoG_02217.T1

MPAHGSNQPQATEYTLQGVMRFLQTEWHRHERERNSWEIDKQEMKSRIANLEGQARRADA

TQKALRKYVSILERKIKEQAIQLKGAEAGELADAQQEARAAKIQEKLKSAAAQKLSVPGV

DGFEMEQPDDEAQRQDLKTFLDQCQSEFMYLMITPANPQPPRDSPPLPMIEDLREAEAFN

MQHAHVMDRQYPMRPAQNHVQELNARSQPGPNHASPMPPNPANFPPKPSDLQAQGMVRNT

SEHQPVMYTPANEWQPSVTTRQVEEPVPDGYPQPGVALGGLEAPVELKEKIQPEADADGW

EFPEGTFPEPGTGQPPSNATNRPDTDVFPNAENPPKSPNRMANSHRRKGSMSRRRSADHD

LSLSSSQNVDNGNFKLRFGLRGHLDTVRTVIFSGGGSPGEPEICTAGDDGTVKRFHIPRL

DSVSGAAASDLDVTANFTHRGHAGGVLALTSWSPSPNFSTGGRAQGDGWIFSGGQDATIR

VWERGRVDPKATLEGHTDAVWALCVLPTTLGAIFGSASTHGSADRILLASGAADGTVKVW

SVSAPPQLSSPQPGSNRGVSGRGGRVRGNSMSSGSGFSNSPQPSVASNSPFNHTLVHTIR

RSGDADASPTCIAPLSPSGETFVVSYSDATVIVYDTRTGDQLGSMDSTETYDQSMRTSIN

AVVATTIGLDQAHQQHHGTMSDEETASGPTGGDRSAGSGVEGTIISGHEDCYVRFFDANS

GQCTYNMLAHPDAISSLSLSPDGRELVSAGHDASLRFWSLEKRSCTQEITSHRVMRGEGV

CSVVWSQDGKWVVSGGGDGLVKVFAR

>SoG_02236.T1

MSAVTYTLPALPYAYDGHVSINQALEPSISKQIMELHHSKHHQTYVTNLNNAIGSYAAAV

SSNDIPAQIALQPAIKFNGGGHINHSLFWANLSPASSPDADPKSAPTLVAEIERTWGSLK

DFRAAFGKVLLGLQGSGWGWLVRDGPALRIVTTKDQDPVVGGEVPIFGVDMWEHAYYLQY

LNGKAAYVENIWNVINWKTAESRFVGGRDDAFKVLKASI

>SoG_02242.T1

MRTHSPPEDRDSSPQDQEQTEPIRRSKRRKYTAVAWRKLKCIPSESDSRCERCVSKGHTT

CDYDTVRRENAESQKSRKNDSETIEALRQDVQSLRDMLTDWISVARRPSGSTNADFVSPR

LPQVAGSASMASASTAQGGPSSVAPDTSTVTTQTPRSDTSLVDVALPRRTTQSRSNRNAP

QEPRFIGPTRSAYSLEVSERALSRLGIPKYELPPPSGQQSPAASPPPTSTSSEGYFWERC

DSAEFIRMITVFQEEVESVYPCFQTGGLIEHAAEVLELGRMSEEEAQQAIESGRSPVSIK

ELHMAKLAVATTIVSEGHGKSETSTKLVEPVEHSVLSILKPSREIIDLQLLIALSIYYFY

LDEDLLAWRTIGMAAREALVMGLHRKPILMETYRDKEEQNIAVRVFWTIYVLDRRWSFGT

SLSFALADRDIDAELPEPHDDCPYLHCMVGYGRLCTALWDALIPFGDRSHNGNPDAMTEH

LDLKTQEWLESIPNELRLSHPRLGLAARAQPPVLQRVRAMLYLRGNHSRILIYRYYLLGP

ERIKSYPRNAWLAVEIAQDSIHVLVHLNESSDIYRRQQAMFNYFLLSALAVLFLAVCNDP

ETFAGPCKRSLHSAIDLLRYFSKHSLGSRRLWSSIRGIVARLRRLEMIHAEEKQLQEEGE

ATTMHQPGKDAENAQQPVAAGTTSGMTMKSGDSGLAGVPVGHTSSYAPLREPTGTYDARE

GMYYADGTQQGMATAPDFSDVGDDLINLYEIFEQGRTLAPQMDLTNFWANGIHAPSYLSE

DPGDLQFNWLVQ

>SoG_02250.T1

MDAHHFDDFGSFPFDGLPDDDHILSLADQHSLRIDPPPVHAFCQAAGIGSASTPSFSKGP

ASISSANVAFDIAHSHRTSSIDRQDDDGLTVITNEKQTPASGTAEESNAGDDMSVSHRGR

ADGTDLGGKPKDEKSDNTPTWSDLKTKAGKERKRLPLACIACRRKKIRCSGEKPACKHCL

RSRVPCVYKVTARKAAPRTDYMAMLDKRLKRMEERIIKVIPKAEQAATATVPRAVLKPSI

PGTNPTSKPSSRKRNAGEAFGGLDAFLKSSSSNEQVETEKANAQRIKEAEENHLFQEGAD

ALPSKEVQEHLAEIFFDNVYGQAYNLLHKPSYMRKLKNNQLPPVLVLSVCAIAARFAGNP

KFHPDDRKFLEGEEWASHARAICVRRYEWPNITILTCLLILGLHEFGTCHGGRSWALGGQ

AIRMAFALQLHKELDHDPSMPGSKTPLSFIDREIRRRIMWACFLMDRFNSSGTDRPMFIR

EETIQICLPVSERCFQLDMPVPTETLDGRVLSLDASKDGQQSDLHENMGVAAYLIRSVAL

WARIITYLNQGGRDADPYPLWDEKSGYAALCHAAEGFQGKMPEVLRFTTENLALHDTENT

AKQYLLMHITIQQNILFLNRAASMTKAHNGKTPPEAFISETVAKTFDAARRISFLLEEAE

KTSQFVSAPFAGYCAFTATIVHVMGIRLGNPLAATNTSPNVEVNIRYLNKMVKHWGMFYW

MVEHVRSEYRAALDAARAGKTGKDGGVSLLQYGDWFNEYPHGLADSDIMDPVNNNKKEKG

ADGVMEPKSELQSVEEFFTTLSPQNQEKSDGQKGQGPSKRKQAARKQSAATIRTGQGKHA

PAETSQQVASQISAQLQLQQQQEQQQQRRFSGQHHGQATGPPAYNSMSPASAQAGAFGVS

PISPVAIQNQFAQQTHGHGPDRAGFFASGMMIPQQTNPLLQSMEPQMMLDGFTLEANGVL

GAQGMIDGNPDWNTMQMAAMHNQRGMKRDQGAAMGQPHGGNGRDMMGGFDPQDTSWFLPF

NMEPPAPNTDVSMDASNLDAFNGLFNSNGNGMTTPNPLGGLQQGQ

>SoG_02252.T1

MSLRPRALPLLRSLSKPQSRCFGFASSAGGNGPTMDIPMPYIEETSSAGRKTWDIFSKLL

QERIICLNGEINDYMSASIVAQLLWLESDTPEKPITMYINSPGGSVTSGMAIYDTMTYIK

SPVSTICVGGAASMAAILLAGGEAGKRASLAHSSIMIHQPLGGTRGQASDILIYANQIQK

IREQSNKIMQYHLNKAKGFDRYTLDEVNDMMERDKYLSPEEALELGVIDEILTKRPEKEF

KDGESGEQSGQTSKT

>SoG_02258.T1

MSSPEIGSKTNVDGRHVENGHELDKIGTNDVPTKDRSLQPPELVRNMTPEQRAELEKKLV

RKIDMRLLPMIILMYILNYIDRNNIAAAKLAGLIEDLSLTSTEFATCVSILFVGYILMQV

PSNLMLNKLGKPAIYLSTCMVIWGIISTATAACQSFGGLVTARFFLGFIESAYFPGCIFF

LSCWYTRSELGVRTTYLYSGSLISGAFSGLISAGITKNLDHALGLRAWRWLFIIEGAITV

AVAIAAFFILPNFPRTTKWLTEEEAALAIWRLEEDIGQDDWVSTEDQSIWHGFKLALVDV

KTWVLLVILFGNVSAASVTNFFPTVVRTLGYDDIKTLLLTCPPYILGVITTLLNAWHADR

TGERFWHVTLPLWLAVVCFIICAATTNVAARYVSIMLMISGLYSGYTTALAWISNTLPRP

PAKRAAAIAFINAVSNATSIYSAYLYPDSAKPRYEAAFIHNCLLAAVAIGAAFVLRTMLA

RLNKRLDRGEHIEGVGNAAPGGATENGFRFRL

>SoG_02259.T1

MDRDRRQKIDVVTISDTEVADSGGNEDNISAHGVNRAKKRKVEDEILEKVQLTDDSDGPV

KKPKKRRAKRKSAGKKRRSDVHGDEPPDNNDELLEDLPGYLQDRRREFDEQRKLHHENAL

MIPPDYSGIPALNDVELGNLEERPRFDESSGVKPSRPYKDIELPQSAGLIPASIARYLRD

YQIEGVRFLHQKFVYQEGGILGDDMGLGKTVQVAAFLTAAFGKTGDERDNKRMREIRYHH

DRWYPTVLIICPGSLIMNWKNELDRWGWWHVDVFHGSRKDDVLTTARVGLLEIMITTYET

YKNCKSAINMVQWDAVIADECHRLKDRSAESTRAMKEVNALCRIGLTGTAIQNKYEELWT

VLDWASPGHFGTLSEWSHAITKPLTVGQSHDATEAQLGLARQVAKKLVQNLLPKYFLRRM

KTLIADQLPKKTDRVAFCPLTDLQLEAYQNFLDSEDMRVVRNASMPCEHDSKKGWCCEKY

VPSGTRWQNVVFPGMVTLQKLANHLTLLTPNTTDLDEKHQSELDRLKLCLPEIWKELYQH

RDQIRYLVHPEYCGKWKVLKRLLAYWEKSGDKVLVFSHSVRLLRILQHLFSMTHYSVSYL

DGSLSYEQRQEVVDTFNADPTQFVFLISTKAGGVGLNITAANKVVIMDPHWNPSYDLQAQ

DRAYRIGQTRDVEVFRLISVGTVEEIVYARQIYKQQQANIGYMASSERRYFKGVQQDAER

KGEIFGLQNIFTYHSESGLLRDIVNKTNIAEAKAGVSLVDVNMDEAVRDGENLGAIKNEP

KDDDGDMGMSQLAALITSEDPKNAVKSAERSGGDVIQAILSAAGVQYTHDNAEVVGTSKV

EEQLSRKAARLSTAGDEAAGQSVLFAGSQDVDEDGLEYNPPEDVQLRQFCEMAKEFGFAS

ATQFALVVEGWNQEKRRNCLDAFYKRRAPILAEATSSGEAEADVMEKAEDDKDNVRDLKS

EDLKWDEGKEDKKDVSLGVRHEPKEEELKEDFKLESMEKAKAEVKNEAGLGPADDKDVAK

HERDVKPKAETKPFKFEGGDSRRTSIFLSSDDEDDEL

>SoG_02273.T1

MSTTVPRPGPANLGPNAGLDEWLEEAKQCHYLPERAMKELCEKVKEILMEESNIQPVCTP

VTVCGDIHGQFYDLLELFRVAGGMPGETNVQAPKTATTVISSDDIEPPTQITNPKLKKKI

KPSIDGDSIGGGDGDDDNGDMDAASQSGDSGVEVTSAPAQASSQSAETRFIFLGDFVDRG

YFSLETFTLLMCLKAKYPDRIVLVRGNHESRQITQVYGFYEECQQKYGNASVWKACCHVF

DFLVLAAIVDGELLCVHGGLSPEIRTIDQIRVVARAQEIPHEGAFCDLVWSDPEDVETWA

VSPRGAGWLFGDKVATEFNHVNGLKLIARAHQLVNEGYKYHFAENSVVTVWSAPNYCYRC

GNVASIMSVDNDLSPKFSIFSAVPDDQRHVPASRRGPSDYFL

>SoG_02293.T1

MANTEKILQGVAVLGKVDEVHRKILTPDALAFLALLHRSFDTTRRSLLERRKQRQADIDR

GQLPDFLPETKHIRDNDTWRGAPPAPGLVDRRVEITGPTDRKMVVNALNSDVWTYMADFE

DSSAPTWDNMINGQVNLYDANRRQVDFKQGAKEYKLRTDRPLPTLIVRPRGWHLEEKHFT

VDGTPISASLFDFGLYFYHNAFQTLKNGHGPYFYLPKMESHLEARLWNDVFNLAQDFIGM

PRGTIRGTVLIETIMAAFEMDEIIYELRDHSSGLNCGRWDYIFSVIKKLRQNPRYVLPDR

SAVTMTVPFMDAYVKLLIKTCHRRGVHAMGGMAAQIPIKDDKAANDKAMEGVRADKLREV

KAGHDGTWVAHPALASIATEVFNKHMPTPNQLFNRREDVQVYQKDLLNVDFPGEITEDGI

KKNLVIGLGYMEAWVRGVGCVPINFLMEDAATAEVSRSQLWQWVKHGVKTAEGKKVDKAY

ALQLLKEVADDLTSKGPQGNKYQLAAKHFSGQVTGEDYADFLTTLLYDDITHLGSPKSAS

KL

>SoG_02294.T1

MSSSATENQASASSTSSNDSKSTTPAPSSTTSNAPSSTADEPLTCRWSHCNQKFPTPETL

YEHICERHVGRKSTNNLNLTCQWNSCRTTTVKRDHITSHIRVHVPLKPHKCEFCGKSFKR

PQDLKKHVKTHADDSVLGPARQDTGGLNYRTQPKGPSSYYDHTGQIRTSAAAFPHQGGHP

GGYYAPQPSTNYGLYFNQPTMNTPRAEHLGYSAAGGYDRKRAFEMVDDFFGSAKRRQIDP

SSYAQIGRSLLPLHGSLSIPSGPLGAEAQYMPQQSAGPGVMHAAQAPGHNHPLAQQYYLP

LPNARTQKDLVQIDTMLGQMQDTIYDNASHATAGVQIHDGYGGYRHSPSPPMNPRGPTHH

GGMPVHADGYHAVSAASMNSPLTAISSTGTPAVTPPSSALSYTSGQSPSPSSSGMSPQSR

HSQTASSVMYPSLPTSLPAVSQGFGQSTTTTLGPSFDGNERRRYSGGMLQRARGAPARAE

LPTSTPRAPESKSSPVSVGSPSSESDVSESTREREEQYDRWLENMRVIETLREYVRGRLE

RREYDEESNSSEAPKSPRDPPTDAMDVDAKSPVPQKKELPGPSSLYPILRMPPKA

>SoG_02298.T1

MYIPSAHAETDLAVLRSLIRANPLGMLTTAIRSPNFPLLQTSHIPFILDDHVASGKGENG

RLRGHLARQNPHSKAMIETLTDNPKEGNILEEEVLVIFTAAAHHYITPKYYTETKPTTGK

VVPTWNYAAVQVYGKAKVFFDTKAEETSSFLNQQIRDLSHHTETNIMNYTGRDGQPAEWK

VSDAPERYIDLLKKAIIGIEITIDRLEGKFKMSQEMVKGDRQGVANGLASLGSDDARWVS

QTVQARSDLKESQKGLDG

>SoG_02301.T1

MRSFALFTTLAAGASAHGIIRNVVGANGVSMPGLGVADGTPRNCAANACGAQADTCIIRD

AEIAAGKSPLGWTQGNGEVTPEKVVAAFMGKGNSAPTNQGTSGATGVEDNIPKNIKGQRT

KRYEEHMNQMRDVFSNIFNLPGIGVLGLGGKATSYPVETIVGDTAGQGAAKGMPTTNDKG

EVGLIYRQVNQDGAGPLTAAIDGTSGGKDASAFKSAAIPVNMPGVGIGGLSLATNTDFPI

TVRMPEGMTCEGEIAGVKNVCVVRVRNQAAAGPFGGSAAFVQSEQSRKNAISYRLKKRFD

IGRMVEE

>SoG_02316.T1

MVLYKRKPVQFLPPETVEDEDAEVWYIPQTGEIFASYENYLSRMDFYKQRRFNDQISGHS

GMTFFEALKNELAGGEEVEAAFPEALKGPVLRKAQFQTVSRLDHLVDMIFDTFKNDYFPG

ESVTVFYPDDDKRLAGLVRDKVTIGARPQADGSMSQPVTRYLIFVVGRDEESSFEETQVS

RERGVFTKSMIRSFMRKTVSREAWNGAPWLVKPDYAAQYYIDTRIPAHLRYDTKLMERKQ

LQAQKRASLPNDHSAGGPVRLPELKPAAKLQPKKATGGAKGKWPPDMAVHGANPAIPEMP

KIPREPTPPPPPKYPIEDLQLELRENVVRPALEFYCHDPPIKAANGTTNGTSRYSQVDME

SVGPLLETWDTLNVYCEIFKLDSFTFDDFVEAMCVASEQVVVQLFEEIHCSVLKILVDSE

ADGGKVRITLPQLEEEDDSDEDGEEEDEEETPEPQPKPTGRATRSSLAKAEAERLAAAAE

EESLRAELETKHRAEELLQDYDWIDELRKRNFPNGGWERIVVGLFHQLSKNERYEQRCEE

LLLQLVPPVEEPSQEAVSLKYANLDLNHRVKALQMMCMLTMETKTVRGYMDECSETMTKY

RKDKIEWQRQKRQALEDLRQLNEQRRDLQPPETAPNSPKPESVKEEEGESRIDADASQVD

KEQEEGDQTDQDVQSRRKRRKPLTEKQRKQEEERESKAKLKEKEKKEAAVPPPSKQLLKI

LKEIQKQEAIIKNCEDEVATIENDLREADCPRTRVMGKDRFWNRYYWFERNGMPYAGLPD

SSTASAGYANGCIWIQGPDDLEREGYIDLPEEQQKEYKEKFGMTIPERKAKEEGGTSVFT

ARQWGFISDPAKVEELINWLDPRGFNELKLKKELVLFKDRIVEHMGNRRKYLAGNDNADG

GEEEEAMTTVSKRTSSRIREKTPEPPNHLCLRWENTMVLEELGHLHSEAPPKPRQRKQSK

KREAAVAEATTVPAKKTRRK

>SoG_02321.T1

MASSVFFKFKSQKEPTRVEFDGTGISVFELKREIITKSGLGDGTDFDLSIYTDDNSEEYD

DDTTIIPRSTTVIARRQPALKPGAGRAARYVSGKAPINAKNAGRREQSSKATASKPSSNA

MSQMNAAMTEEEKMAAVFAAQSEQWNAQQEEMSQYVYVTASQREETNRNSQTPVFKPGTK

RPPTNVPDRDPPNGYICYRCGQKGHWIQLCPTNDDPEFDNRPRVKRTTGIPRSFLQKVDK

SVILAQTDGDETKRPSGVMVNADGDYVIAEPDKVSWEQFQAKTNTASAAKEAAANDDDEA

QKRGLACPIDKKLLIEPMKTPCCKKSFCNDCIIDALIESDLVCPSCQTENVLIDDLLPDE

EVAKKIQEFVKEKEKEKEKETAKSPRPTSPEADTSGEVEKAKTEEKKEEKNDASPTAEKE

AAPAKASPVADIPSSPSHGALVKSPTSTPVEAKEASSATDKADKLGTNDITTTEAKTDKS

KSKKRPADDFLENPKIPKGPRAMQQQQQQQFNQMPNMMNGMPDMSMGNMMFNGMPMMPNM

MGMPGMMNMPNMMNMPMMMPNMNMGGYNGMNNMSNMNMVNGWDMNAMPAMTMNGGHGMMG

NGGGNNFPMRHNFTQPQQDDDAYFRKPVNPHRHQNKGRRARPSDYREL

>SoG_02324.T1

MASGHRTRTSEDYGVFDDAKTYYATDERHTNRSGVRTRTFSQNTLLKQFDNGSSKPPFRR

GSHDEVGSTSHRRFLIQVDPTLENLRAQEDTDDNMQITIEDSGPKVLSLRTAASAGHNKF

DVRGTYMLSNLLQELTLAKEYGRKQIILDEGRLNENPVDRLSRLIRDHFWEGLTRRIDAS

SIEVAARDPKDWTDDPRPRIYVPVGAPEQYQYYKKVAEERPEMRLDVQLLPAKITPDLVR

DMNDRPGLLAVATEERTDPTTGKKTLEGLPFVVPGGRFNELYGWDSYMESLGLLVNDRVD

LAKSMVLNFCFCIEHYGKILNATRSYYLCRSQPPFLTDMALRVYEKIKHEPDSKEFLRRS

ILAAIKEYHSVWMAEPRLDPITGLSRYRPEGRGVPPETEASHFVHILKPYIEKHGMEFSQ

FVRAYNYGEILEPELDEYFMHDRAVRESGHDTSYRLEGVCANLATIDLNSLLFKYETDIA

RTIRSVFGDRLEMPEEFCRGTPYTPGELLSSAAWDRRAKRRKLMMDKLMWNEEEGMFFDY

DTVKQERCTYESCTTFWALWAGIATPKQAAEMVRKGLPRFEVSGGLVAGTEKSRGEVGLD

RPNRQWDYPYGWAPQQMLAWTGLLRYSFTDDAERLAYKWLFMITKAFVDFNGVVVEKYDV

TRPVDPHRVDAEYGNQGLDFKGVAKEGFGWVNASYVYGLQIVNAHMRRALGTLTPYPTFV

KAIELNDEKALADLQLK

>SoG_02353.T1

MAAGNPSIRPKGREEQQQQQHQHRPQQHMTPPSSQRWDDETRWGSNTDGPGMEHVRSDQG

PEQQPAPSAGSRESSASSEDGPPQQNSAATPSQRKKRKMQQYQKISAEPACGWCASHNRN

CVYLERQKPGSRSGFNVELEAKVNRIDALLQVLGRRIEEHIANDHPAPSVQAATPGAPAA

YRSPPQAIHDASSSEFAHGPGPSSLGRATPMESGRTASTPGGGGGGLVDHYSRMDASSEQ

LGGGVDIQPPAGFTSLPYSPTNTATPRSLSTTPDLPPQDMIYTLVDLYFKHCNTWCPILE

RQTIFGAFFGSTSLSEPDRVLLHAIIATTLRFFKDPRLTPQMKAHYHATSRRIVQTYVLD

HVSIPAMRALLIICLDELGTANGPKGWNLLSLLCQNVKQAGLCEEISVYLLADADDIPRI

GSVRRVVAGRPESWIEDEGRRRLAWMVYLLDRYCATATTTFEFMLDDRRMKRFLPCSYDL

FCRNVPRETRLPSKVLEPLSDNPSNTNCNNAAQSDSLGSFSYHCEVLRIMSEVHNFLKTP

VDVTSPAEVAGWRNKHQLLDAALDRWLQSLPSEYSRISALCHSDPASRVANWFMLHSAYV

TAVIRLHSSAAYPTVRTEIFVPSHYAMQRCLSAVQSLRDLSRDVHEANGLDLLGPPFAFS

LWVAARLLLVHAATVGAPVDDKFDFFVETLVYVGQYWEVANNYARILKRVVQRGQEGDLS

LSDMRWRARDLVTLTGSPRPSGLDPTSTQASSLSELDSIDVFDFFHSPKVTPEVMAKTNF

GQTNFLHPSSSAMGGGGRQGPGAVPDPEADWLRMSQVYQ

>SoG_02367.T1

MAPPPEIAIPSTSVSSESSSKPYTLYNITLRLPLRSFVVQKRYSDFATLHSTLVSQVGEA

PPASLPGKHWLKSTVKSADLTHQRQVGLEAYLKAIAESPDRRWRDTSAWRAFLNLPSSST

SNSAVSAGGMVANKAAGAADPGTWLDMHRDLKQCLHEARQSLARRDHASDSGNTSAAAEA

GTAAKRSIVKAGTSIAMLADGLRKMQESNRLGDGEIRRRRDLIATARMERDGLDKLANSM

PSAMGSSSTSKGQPSSSDKSALLSGGRPSGGRVLGGPIPETDKTRELDNQGVLLLQRQEM

ASQDQALDELAVIIRRQKEMGMEINDEVERQNEMLEQLDQDAGRVQGKLKVANNRIKKF

>SoG_02373.T1

MADMNPELQAKLEELERELEEGDITEKGYQKRRANLLAQYLGQPIAPQSAPPSAPRGGLR

LHQPDDLPYASNDGHRATAFNAIASAGGSLANSPTLTGFPQDQGFERPSSGYGQPESHAA

GLLRPGGPIAERPAVVQRDSLFLTPGSNDGGTRTGTMVSGDYAFNPEQHGAYMDQHQSMA

QQQSGYDGRTQTLGNDGRTGTLLDSQGYFSDFAGQQHYDQNQGPEYGGAHRYSMGEAFSP

TAAMAPPMLTASDLPPPEVLEYQMPLEPREVPFAICDPHDSNTAMSKFDNMAAILRHRAR

TTAKVPAYWVLDNKGKEIASITWDKLASRAEKVAQVIRDKSSLYRGDRVALVYRDSEVID

FAIALMGCFIAGVVAVPINDLQDYQRLNYILTSTQAHLALTTDQNLKAFQRDITAQKLTW

PKGVEWWKTNEFGSYHPKKKEEVPPLSVPDLAYIEFSRAPTGDLRGVVLSHRTIMHQMAC

LSAIISTIPGNGPGDTFNPSLRDKNGRLIGGGNSSEILLSYLDPRQGIGMILSVLLTVYG

GHTTVWFDNTAVEIPGLYAHLISKYKATILISDYPGLKRAVYNYLAEPLLTRNYKKGSDP

NFQAVKLCLIDTLTVDSEFHEVLADRWLRPLRNPRAREVVAPMLCLPEHGGMVISVRDWL

GGEERMGVPLKLDMASEEDSDESEKEEKPAPSNGFGSLLGGGTTQTTEQRPKTELFEVLL

DREALKTNEVVVLAVGEEARKKASADPSTVRVGAFGYPIPDATLTVVDPETGLLASPSSV

GEIWVDSPSLSGGFWAQPKNTELIFHARPYKFDPGEPTPTPVEPEFLRTGLLGTIIEGKI

FVLGLYEDRIRQKVEWVEHGQPLAEYRYFFVQHMVVSIVKNVPKIYDCSAFDVFVNDEHL

PVVVLESAAASTAPLTSGGPPRQPDMALLDSLAERCMEVLVQEHHLRLYCVMITPPNSLP

RVIKNGRREIGNMLCRREFDLGNLPCVHVKFGVEHAVLNLPIGIDPMGGIWSQAASEARL

DYLLPADKQYSGIDRREVVIDDRTSTPLNNFACITDLIQWRVARQPEELSYCTIDGRGRE

GKGITWKKFDLRVAAVAMYLKNKAKVKPGDHVILMYTHSEEFVFAVHACINIGAVVIPMA

PLDQNRLNEDVPAFLHIVSDYTVRAVLVNQDVDHLLKIKPVASHMKQSATILKIPMPSVY

NTTKPPKQNSGLRDLGLTIDPAWIRPGYPVVVWTYWTPDQRRIAVQLSHDTIMGMCKVQK

ETCQMTSSRPVLGCVRSTTGLGFIHSCLMGIYIGTPTYLLSPVEFAQNPMSLFVTLSRYK

IKDTYATPQMLDHAMASMQAKGFTLHELKNMMITADARPRVDLFAKVRLHFAAAGLDRTA

INTVYSHVLNPMVASRSYMCIEPIELWLDTKELRRGLVVPVDPDTDPRALFVQDSGMVPV

STQIAIVNPESRMHCYDGEYGEIWVDSEACVKSFYGSKDAFDAERFDGRTLDGDPTVQYV

RTGDLGFLHNVSRPIGPGGAQVDMQVLFVLGNIGETFEINGLSHFPMDIEYSVERCHRNI

VPSGCAVFQAGGLVVVLVEVSRKPYLASIVPVIVNAILNEHQIIVDIVAFVAKGDFPRSR

LGEKQRGKILASWVTRKMRTLAQFAIRDLDPSAMGDGTGATDGSDPNRMSMVSSVRNSGG

PAGVSSLRNVEQAPQILEQEEFEQQMDHIANMPPAHTMRDEPTTPTGYTLQDGEVDYDDV

TTPLANPQSHARYQDQQYEANQEPPRPGPKPSTRDGESSTPHIRLPGVDGRESIDFWGKG

VVPKDGETDEEDWTADAIMHMNLAGDMSRPQGPER

>SoG_02375.T1

MGKVENPALQVIVLGAGGGPLESNTTAFLVRSIAQEWRKGSVIAVDGGVYLSVITKLLEE

AVCSPMPTRPYKIETGPFAGLSLPYSSIEANAGHITSALIDAHLITHPHLDHISGFVMNT

AGLPGTRPKKLAALPNTIHAFKTHIFNEVIWPNLSDENGGAGLVTYLRLVEGGSPAMGDG

DGKGYSEICDGLLVKGWSVSHGTCVEKHGRRGSVSSTSTRFGSHDASAQTPRPQYSHPHQ

QSGARRPSLLSQAMMPGSGSPQAAETERASTCVVDSSAYFIQDRETRREVLMFGDVEPDS

ISLSPRNLNIWQEAAPKIASGTLAAIFIECSYDDSQSNDRLFGHLKPVFVVEELNALARE

VASARREMKAAESRKRKRTSIGADDGRARSTPKYGMTPTTEIPLSPKSTFTKPSRGIDST

TTSQPNTPHLATPTEGLSLGDVEPFAPNPHRQLDGLKVVIIHVKEKLMDGPHRGDLILEQ

LLEHNEEVGLGCEFVIAKQGQSFYF

>SoG_02377.T1

MGILEEVAAHPLAQSFQSSSLSFQISIVLAALIGLSVVYHVASQLLFKNPNEPPMVFSWF

PIIGSTITYGMDPPAFFKENREKVHSLSRTVSCGHFGDVFTFILLGKKTTVAVGPAGNDF

ILNGKLKDVNAEEIYTNLTTPVFGRDVVYDCPNAKLMEQKKFSSTQFMKIALTTQAFQSY

VPIISNEVQSYFKKNAHFKKTTDVCDIPKYIAEITIFTASHALQGSSIRNKFDESLASLY

HDLDMGFSPINFMLHWAPLPWNRRRDIAQQTVAKIYMDTIKERRAAGDHDGEYDMMKHLM

NSTYKNGTKVPDHEIAHMMIALLMAGQHSSSSTGSWIMLRLAQNPHVIEELYQEQVKALG

ADLPPLTYEDLAKLPLNQSIIKETLRLHAPIHSILRAVKQPMPVPGTKYVIPTSHSLLAA

PGVSATDPNYFPEPDKWDPHRWESDSPLAPKLPTGRDEDEEKIDYGYGLVSKGSSSPYLP

FGAGRHRCIGEHFANVQLQVIVAEIVRLFKWENADGSNSIIGTDYQSLFSRPLQPANIRW

TRREA

>SoG_02380.T1

MAPRRRSALFQQVWTLAAKSIKIALFHQTRSTFYTAFLLPVLVAVYLGVGQRLNQPQSDF

GIAEPRAVRSLQEALSAAGSGRETVLFVNSGHDNGAIDSVISSLSNAVRSAGKNATTVRS

EDDIGYVCRSSIRGTSGCYGAVTFHSSPEEGSGGRWNYTIRADAALSREFFHSKDNNDAQ

IYVLPLQRAVDNAIRKANGSSSNGDGDLPRVDEYAYTALTEDEREDKQRREYQQTFIDFL

GVAFFVGLVGVAYHLPGMLATERERGMSQLIDAMILTRYDWEPQLIRMLSFVLAFSMVYV

VGWIACGAVAGAIIWKETSVVIPMVAFVLGGVAVTSMSLMGATCFKRAQLSGAVNSIVYL

LFGVLAQALPNPGTPAVVVLSILFTPSCFVFFVQSIARFEAEGRPANLVRAAPGANHDVP

GIFFLVMFIVQSLLYPVIAGFLERAIHGVSSEHRRVFHAGPGREPPVDTLSIDGLTQIYR

PSFFRCLFSFVSRPRPATVAVDNLSLSAKRGEILALLGANGSGKSTTLDAVAGISRFTRG

NISIDATGGLGYAPQQNVLWDDLSVEENIRIFNKLKAPHSKATLTQNRELARAVGLESKY

KSKAGKLSGGQKRKLQLALMLTGDSAVCCVDEVSSGIDPLSRRKIWDILLAERGSRTIIL

TTHFLDEADLLADHIAILSKGSLRAEGSSVELKDKLGAGYRIHVLDPHKVKSPPKIEGVE

SRVSHSSILYLSPTSRLAAHVVKVLEASGIEYRLSGPTIEDVFLQVAEEVRSEGDLMRTG

GEKVRPARDAGKSHDLQNGRRIGSFQQVKVLLIKRLIVFKTNWIPYLIAFAIPIIAAGGT

QVLVSDEPPTGCSPLQRSSGNRISSDYGDIFSNLNVVAGPRGELEGNGVQQTISRMVSSQ

PGVNGGDNKDILELVTSLPSFESFIQENRKNVTPGGIWLGDSSSPAILAYRADRFSVRTS

LVVQNLMDVMLSNISIVANYNVFEAPFSSSTGDALQLAIYFGIAMSLFPAFFGLYPNHER

RLHIRGLQYSSGARSFPLWIAHLSFDYAIVLVTMIIAAVIFYVSSDIWYQGAYLFPVFIL

YGLTSLLLSYVFSLFLSSQLGTYAACTAYSMVGHAVYFISFLYIITFSSEQKTDFNVLAG

HWVIAAFFPVASLVRAMFVALNAYSTSCSGFELRSYPGAMAAYGGPILYLTVQAVLLFIF

LLWHDSGRRMPFASLFTRRKNTAVNSEDNRDSEVILTESDQEIQNELTRVTDPENRDGLR

VEHLIKEFGDFTAVDNVTFGVEHSEVFALLGPNGAGKSTTISLIRGDIQPSKNGGGVFVE

GISVSTQRAAARANLGVCPQFDAIDNMTVEEHLRHYARIRGISDIDRQVDAVIRAVGLEQ

FPNTMAGHLSGGNKRKLSLGIALTGNPSVILLDEPSSGLDAAAKRIMWRTLDALVPGRSI

LLTTHSMEEADALASRAGILAQRMLAVGSVDHLRQRFGDTLHVHLVSKTAPHSTPEEMEH

MRGWVQQALPSAEIQSDTFHGQMRFSVPAAEISARNGGTGSAVGQLVVLLEDSKEQLGIS

HHSVTPTTLNEVFLNIVGKHDVAEEGYSSRPKKNKKKGWWSKMLWLMKP

>SoG_02399.T1

MAVAAAHPLAGAPPPFRLPSFRFSKTPLRLPLSHLINRVHSPHQTSLNPSLSLFLPLLLQ

NLNHPKSAHRRRTILTERNPQAMDFTNSYPQFAAGQPYGHFVPVPPLTPSQTVNSDDFNQ

SPPERFENFNGNGFPNFDYQSFNVAPAQQTPFTGPPTPPTHGLFGTGQQQQAQQQLQNRQ

QAQQQQQAQQQRHSPLSSAASNGPNDVMIKSDHGDDSLRGPGGSEDDDNVTPAQSRRKAQ

NRAAQRAFRERKEKHVKDLESKLANLENAQQQASVENQRLKADLQKMSTENEILRATGGS

VQNSHSPEPLSTGPMTYEPKDFYSNLLKGHNNQTLSHRIAYSDEGERLLGAAATWDFIVN

HELFKAGLVNIAEVSNNLNHRAKCDGQGPVFAESTILDAIRHSVASGSDDLL

>SoG_02410.T1

MDAPAQSQRPSASRRGGSRNEGRGRRGNHHGRGNGRGGRRGGGSSQSQPLNSTPPAPPTD

TASLANSETQSTGRGRSSRRARGGQPRRGGGPSSRGAMSSGRRAFGGRLTQEEANEDGTS

DPSLSAEAAEFVPGQPVVARPRPSATRHSQPSRPNLPKSTANDLGTRIHEDISNNNYECT

ICTDEVLRSSKIWTCTLCWTTVHQKCVQHWYRNQKAQWNEQQAKNPAGDFKWRCPGCNSN

LTEEPAAYHCWCGKELSPSPASKALPPHSCGQTCSKPRSTCPHPCFLQCHAGPCPPCDLM

GPLQSCFCGKNTSQKLCRETDYENGESCGETCGDLLPCGEHVCPRPCHSGLCGDCETVVE

ARCFCGGETKAVTCSSREDPQDSYDAKEAVFFEGTFHCDKPCERLFDCEKHSCSKSCHAQ

DEEATHCPFSPDAIITCPCGKTNLTKLIDSPRRSCEDPIPRCNEPCEKTLPCGHACQDKC

HTGDCGYCSLVVDIACRCGRTSTPSLCHQGDMQPPMCMRVCQANLNCARHKCGERCCTGE

KKAVERQAAKRKQRLAGPDLPVEAEHICIRECGRPLKCGLHDCQQICHRGACATCPEAIF

DEIACDCGRTVLHPPQPCGTQPPACQFPCQRRPECGHPSVDHHCHGDDKACPKCPFQVKR

WCACGKRNMANQPCHLQEAHCGLVCGKKLKCGLHACRKLCHKPGECEDEGAQASKCSQMC

GKAKLFCDHKCQQPCHGATPCNENIPCTARATIACPCGILSQEVKCAASASNPTPERQEL

KCDDECLRQERNRRLAAALNIDPATHTNDHVPYSDTTLALYRDHKVWAEVQEREFRVFSQ

SPSEVRLQYKAMPREQRQFLHALADDYGLESISEDEEPFRYVVVYKGQRFVSAPNKTLGQ

CIKIRDAQAAEAAASAAASRNPSPPPPPVFNEDPFNAFLLLSPRFGLTADEVTECLKPDL

AKASASFTFSTYFLPTDEVAVRATAHYSSFLHPSAVEQELASLKPRLAETLGRTGLAGSV

VLCHLSDAEEVTRREVKGGVQMQDGSGWSAVATRGAAGKKTVAEESSAAKGTGRKLLGLR

KKKVGEQPKEKAWAALGTDVEC

>SoG_02415.T1

MSTNGAVGAPEALANIEQALRLVHDPASSNESRRDAQAYLETVKENPEAPLHGYRLASDK

TQGSVVRHFALSILEHVIRYQWASYSEEQAEALRNWVVELNQAVSPSDPTFLRNKTAQLW

VEVAKRSWGSEWTNMDTMLVQMWEVPDSSVHKEAVLFILETLSDEVFTGDDAVVAFREGV

LSKACVEIFTPLPVLLESFPNRQAGPDVRHGPEGWLSRLSAFLEYCLTCDAKDNEEVKSC

AVKGFSAFLSLMPWAIPKAIAAAQCVAVMCLGLKSSNVEAQKLALEALYALFGRTNFNDE

EFLALVAPMYSQPSVELLRNMYDWASVDPEDIDDDKYQVQKKLAELLSPLGDYFQRKFSK

LPPDAARTEFMQLLVHVAQSQSLMVSIPVLVAWNVILGRGALGEGVFRENGMITTVIGPL

LELSCSRLVRYENLPEDSNDPTFLFLMEDTDTLPERHAFLGNYRRYSSMIIESIVQLQLN

EAISHVLSGTEEVLLHLYDGQAPLDKQCYSKHSMPVLRVDARFTVIESCLKGYMKWRKQH

RDEPENKQQCADLEANLEAWCQKLLAMTFEDPMIRKRTLQLLVYFSTTALKHNTSFMLKV

LEHILMTWPTVEPEYRAYNDAIKDLQSESMVELQRLASEMPDHLLDVYEQIEGRVNEMIS

SGTLDEKRSIAYKSFLFIIIHRTSKVDTQSKIQKLREFIDPVKAQWQSEPIRTSLKSYNS

FCQLLGLDKAQTYLASKRAHEIADWGSTMLDEEGLALQAELEERLKALPLRPTKSFLAFS

VERLEKSSSAFQASYVLWQEGFSNILADLLEFLGFAHAAHNPDNWSGLPSDMRGMVHRVL

SDRFWQAGISDGSKDDFYARVMDKKNTMEGLASSIRGSVRFVRETAYAIIYCMSRMDGQF

YGCEGLSGPLSRALFADSIWLSTHQQSNLLNLVRYLVDDCPVDYRDQFLPQILAACFQQM

DTKINGEWEKLAQQQTVAAGGDDALKEEMKAESILRQVTYTAVVMVADFLDPNKPVPRPR

KLETGEMQANGAEHSSLRKFCLSRREVAEPLLLFCLHGTRMRDHRCCSMVLRLFVSLIPE

FRSDESRDPNVANGAVGENPTAVAIRDFIALDVLQACITSFHEPYFVELQKDLATLIASI

LVYYSPVTPKPREVILSLPNINPAALERLTPYVTKPNAHIRQQRAIVLDLLKDLKGVSVS

EMGKLQKSGLGGAKRSGGVRGSDRGNNNGRSRMAQQFMQSQTEGEGNMAARGAAAAAAVA

AGGDRKTTPDGLEGVSTLFELQG

>SoG_02429.T1

MAPKNKAQGSGKKPSAAKVVEDKTFGMKNKKGASAQKQIQQMTNNLKNSGSQEEKRKAAE

KAQREREKKAAEEAKRETEALLNKPAQIQKVPFGVDPKTVVCIFYKKGDCEKGRKCKFSH

DLSVERKTEKKDLYTDARKDEEEKKKAETSADWDEEKLRSVVLSKKGNQRTTTDKVCKFF

VSAIEDGKYGWFWTCPNGGDKCMYKHALPPGFILKTKEQRAAEKALMDKSPLKTLTLEDF

LESERHKLTGTLTPVTPETFAKWKQERMDKKAAEEQARKAKDNTGRALFESGKWRDEDES

GSDDDDDDTWNLEKLRRETEAIQTRKEEERLLALYGGTTNGDSTPATQEGTATPQPVA

>SoG_02442.T1

MIFEHLEDKPTFDLLGTEPSQLPKSVDSVSLDSSKGTDTSGKSDDGKPVKRGRKSRPKVK

TGCAVCNCTQCVRSKKECMGYPPPPRSSRPEYEVRIAPKPSESGRAHSSSISSAMAPSHL

IPTIDEPPYPSLLGSGAALPSRGTGRRRGTHSSASSPIPESAASSHDQNSTLPVMGTDRF

YFRIFQDHTANELSGFFDSSFYTERVLAACRNEPAIRHAAIALGALHKTLEQSWGASGAP

PGPPKERGEQLMHWQMAVRQYSEACNVMMASRDPSESSLRTQLMASILLASFDSFIGDHR

QAIVQIQNGLDLLERIRAEQRLPAYGLATDTAEEELTVIFMRLAIQAKSYDLGFHFPEPY

VVRFTSPAQHQALLQQLSQQHPQPELGVDAYLSHLSPGSIYESRSTTPSGSPPPLQPFAS

VHEARVAHDRLHERMIKLQESLHMRPRELAQMAITETWVRASSSFKSQLASWSEAFQPLL

LSRHGPHVSSRERAAINVLQMAHLTASLVSEVQFTGSELVFDKYTGIFQSIVDLGLEVIQ

EEEAFGAAACQAESAYCPHQRQLYPDYASPSLFSACHVKPIFSMDFGIVAPLFVVGTKCR

LPILRRQAIELLRGCSRREGMWDSNLTALIAHWIMTLEESPSEVHNNSFPEFASHPASKG

YDELAPTSLPEFLHDPGLHLTGQPYAQVPEEKRVMLKSVDFDLRARWANVVVGTRSVPDG

LPDPRFRSTQLTW

>SoG_02459.T1

MAADKATATGADLGDGLRKRINVPAAPAPGVLQPQDNKKLAKKEPTFFESFDAWEPIFAP

ILFTLLAIGTRLWKIGISNIVTWDEAHFGKFGSYYIKHEYYFDVHPPLGKMLVGLSGVLA

GYNGTFEFKSGEKYPEELNYTFMRAFNAFFGIVCIPMAYYTAKELRLKKPAVWLVTLMVL

CENSYTTISRFILLDSMLLCGTVATVLCWAKFHNQRHRSFEPEWFFWLFMTGLSIGCVCS

VKLVGLFVTALVGLYTIEDLWNKFGDTKMPLTTLAAHFVSRVVGLILVPFLIYLLSFALH

FAILNKSGPGDAQMSSLFQANLKGTEVGKNSPLEIAIGSKATIKNMGYGGGLLHSHVQTY

PEGSGQQQVTCYHHKDANNDWFFYPNRNEPDYDSTSDEIRYLGDNSVVRLIHAQTGRNLH

SHDIAAPMTKSDKEVSAYGNLTVGDDKDHWKIEIIRDAASRDRSRLRTLTSAFRLKHTAL

GCYLRAGNKNLPQWGFKQIEVTCTKENNPKDTYTHWNVEAHTNEKLPAADAGAYKSPFFH

DFIHLNVAMMTSNNALVPDPDKQDDLASHWWQWPILHVGLRMCSWNDDVVKYFLLGNPFV

YWGSTVSLGFVAAVVAWYLVRWQRGFKDLNAQEIDQIHYAGIYPVAGWFLHYLPFVIMAR

VTYVHHYYPALYFAILTFGFLTDWLLRNRIKEVQIVMYGLFYTVIVGLYIYFIPICWGMT

GSNKEYSRLRWFDSWRVSDPQ

>SoG_02482.T1

MSILPRGVKNVLAKSPSDVVILSSLRTPVCRSYKGSLKDAYPEELLSAVLRATLEANPNL

PPSVIEDVAVGVVLSELGGSKAARMAMNHAGYDNKTSLYTVNRACSSSLQAINAVAAQIR

TGMISVGIGAGMESMTRNYASKAIPVDLWPALKEDPNKDVRDCIMPMGLTSENVAERHNV

SRADQDAFAVKSHENASRARSSGAFAQEIVPVTTRFQEVDKQGNKVGEEKTITVTQDDGI

RDNASIEGMAKLKPAFKADGKSTAGNSSQVSDGAAATLLMRRSTATELGLTSSIKGKFVS

AVTIGCAPDEMGIGPALAIPKLLSQHGLVNEDIARWEINEAFASQAIYCLRELGLEGAWE

SGKVNPDGGAIALGHPLGATGARMASTLLHGLARDGGEMGVMSMCVGTGMGMAGLFVRE

>SoG_02485.T1

MADAAFEKAEEAVAAGRSITQVNEAAPVSSPHIEREDDEPRDVALTRVNTGVSIEQAEAD

FQELQREFTGVSRASRRHSKKPSHADLEKAGPVNSSDDESIFDLETALRGDVDAGREVGI

RAKHIGAYWDRLTVKGMGGTTNYVQTFPNAFINFFDVISPLMRLFNLGPKPVEATLLDNF

RGVCKPGEMVLVLGKPGSGCTTFLKTITNQRYGYTSVEGEVLYGPWTAQEFNTFRGEAVY

NAEDDDHHPTLTVEQTLGFALDTKMPAKRPGNMSKENFKQHVITLLLKMFNIEHTRKTVV

GNHFVRGVSGGERKRVSIAEMMITNACILSWDNSTRGLDASTALDFVKSLRVQTNLYQLT

TFVSLYQASENIYRLFDKVMVIDEGRQVYFGPASEARAYFEGLGFAPRPRQTTPDYVTGC

TDEFERQYAPGRSAENAPHSPDTLLEAFAKSGFSKRLDDDMAQYKAALDDEKHKHDDFLA

AVRESKRGSSKRSVYQVGFHLQVWALMKRQFTLKIQDRFNLSLSWIRSIIIAIVLGTLYL

DLGQTSASAFSKGGLLFVALLFNAFQAFSELAGTMLGRSIVNKHKGYAFHRPSALWIAQI

IVDQAFSASEIMVFSLIVYFMTNLIRDAGAFFTFYLLILSGNIAMTLFFRIIGCVSPDFD

YAIKFAVLVITFFVTTSGYIIQYQAQHAWLRWIFWVNLLGLSFSSLMQNEFSRIDLTCTA

DSLIPSGPGYDDINHQVCTLPGSTSGTTFVSGSAYLAQGFSYYPGELWRNWGIVMVLIVS

FLCLNVILGEIVNFGMAGSSFKVYARPSKERKALNAQLLEKRDARRHDRSNEEGSEVTIK

SESVLTWENLNYDVPVPGGTRRLLNNVFGYVRPGELTALMGASGAGKTTLLDVLAARKNI

GVIYGDVLVDGVKPGKQFQRSTSYAEQLDVHEPTQTVREALRFSAELRQPYETPIAERHA

YVEEIISLLEMELIADCIIGSPEAGLTVEQRKRVTIGVELAAKPELLLFLDEPTSGLDSQ

SAFNIVRFLKKLAAAGQAILCTIHQPNAALFENFDRLLLLQRGGETVYFGDIGEDAHVLR

DYLKRHGAEAAPTDNVAEFMLEAIGAGSRPRIGDRDWAEIWNDSPEFRQTKEFIVKLKEE

RATVGRQSDHSQEKEYASPMKHQIKVVVSRMLRSFWRSPNYLFTRIFNHFAVAFITGLTY

LNLDDSRSSLQNKVFVMFQVTVLPALIISQVEVMYHIKRGLYFREASSKMYAPSTFAIAI

TLAELPYSILCAVVFFLPLYYMPGFQTESSRAGYQFLMVLVTEVFSVSLGQGLASLTPSP

RISAQFDPFIIIVFALFCGVTIPYPQIPEGYRVWLYQLDPFTRLIGGMVTTALHQLPVVC

RPNELNRFTAPENSTCGQYMEPFFASGGAGYLVSNDTSSCEYCAYKVGDQFYTHLNISFA

NRWRDLGIFVCFVVSNLAILFLAVSVFAIY

>SoG_02486.T1

MSDRRRVGDYVWEAVIYAGVLSAGYYLLQNLARPMIQSLQDPDKEKHDQARQKAEAHLER

LSRRRARGGDGSDDDTDDSRHRRRIQELKLNEYENLIALEMVAPEDIPVGFNDIGGLDAI

IEELKESVIYPLTMPHLYSHSAPLLSAPSGVLLFGPPGCGKTMLAKAVARESGASFINLH

ISTMTEKWYGDSNKIVRAVFSLARKMQPAIIFIDEIDAVLGTRRSGEHEASGMVKAEFMT

LWDGLTSANASGVPDQIVVLGATNRIHDIDEAILRRMPKKFPVPLPGRAQRRRILQLILE

GCKKDPEEFDLEYVANLTAGMSGSDIKEACRDAAMAPVREYMREHRSQDARTMARVDPSE

FRGIRSSDFLARRGTQPPEKQPVRRSSARSAKDASDEFEDVEETIGQD

>SoG_02489.T1

MVSLKSLLIAASAVTTALGRPFDFLDERDDNSTSVIEARQVTGNSEGYHEGYFYSWWSDG

GGYAQYRMGSGSHYQVDWRNTGNFVGGKGWNPGTGRTINYGGSFSPQGNGYLCVYGWTRN

PLVEYYVIENYGSYNPGSNAQHKGTVYTDGDTYDLYITTRYQQPSIDGTQTFNQYWSIRR

NKRSSGSVNMQNHFNAWSQAGMRLGNHYYQILATEGYQSSGSSSIYVQTH

>SoG_02495.T1

MDHWQQQHHHQQPPPTTQQHYHQQQQQPYVDAAGNPSRRQNGTTQQLPRDYVPQQHHLQQ

QQPPPPQQQHHQYPPASSSAAHASYKYDQYRGGAPVSAAHPASAGASAAASPLSVGPPQL

RDGNGDVPMHDAHDPHAGIKYPMRPHHQSHPSAGRVPTLQHPNQEPSAAAQRYSPMEALS

PASPYGSKPVQYGAPTSQRQSPTKPGDYPTSPYFAGRSQGQQLPPISPYTSAPDGYASSA

VANFDGQFNDPKSPRRHMAPQMPPQKGPVPEFKNVRALSDLRPKNSQQPPFRRANPEGGF

ISPLQALTCHLPATYRICNPNFKYETSRNPRRVLTKPSKGTKNDGYDNEDSDYILYVNDI

LGSEEAGHKNRYLILDVLGQGTFGQVVKCQNLKTQEVVAVKVIKNRTAYFNQSMMEVSVL

DLLNTKLDKNDDHHLLRLKDTFIHRQHLCLVFELLSVNLYELIKQNQFRGLSTTLVRVFA

QQLLNGLALLNKARLIHCDLKPENILLKNLESPIIKIIDFGSACDERQTVYTYIQSRFYR

SPEVLLGLPYSSAIDMWSLGCIVVELFLGLPLFPGSSEYNQVSRIVEMLGNPPNWMIEMG

KQAGEFFEKKQDEFGRRTYHLKPMEQYAREHGTKEQPSKKYFQANTLPEIIKMYPMPRKN

MKQSEIDREMNNRIAFIDFVRGLLNINPLERWSPQQAKLHPFITQQKFTGPFVPPMNLKA

SSLNRSPAPGTQQQQQAEALSKQRAQAAQASANSAAQGAYGALNQYGPPVHGQPPPMYGA

NNAVYSSGGSHSNVPPSYGTQGSQYGPMVMPQQPQQMPQASYGGPNPQQNMYQHQPQQPP

QGMRNNRQRASTMDQQQSGIPAAIQRVASHLDPSQPIRLQPSPAYYPPPGEGMPGVDQGA

NRQGRRGSRAQQGGRGNRDFIRNLEERTLEEGFMGNQPNPWH

>SoG_02512.T1

MAEVDPFDSALDLLRRLNPKHTTEHLNSIISLAPDLTEDLLSSVDQPLTVKRCKQTGREY

LLCDYNRDGDSYRSPWSNQFDPPLDGEGGVGGVGAGGNEGAGEGAIPSERVRKMEVGANE

AFDLYRDLYYEGGVSSVYFWNLDDGFAGVVLLKKSATPGQSTEGVWDSIHVFEAIERGRS

THYKLTSTVILSLSTSGSTLGDLDLSGNMTRQVEQDLPVDNDESHIANVGRLVEDMELKM

RNLLQEVYFGKARDVVGDLRSVGSLSEGARDRDAQREIIGSMRR

>SoG_02519.T1

MYPGQGYNGSGNHHGGGYGRPPPGPPPPQQYYGHPPSGPPPPGPGYGQYPPQQGWGAPPP

GPPPGQYNYGAPPPGPPPLGPRPPVQYESAHGRPPPGAPPQHLDAYGYPVQRAGYASHAR

SGPPPPQGAQQFGHGAPGGYTFQYSNCTGKRKALLIGINYFGQDGELRGCINDVHNVSSF

LVERYGYKREDMVILTDDQPNPVQQPTRANIIRAMGWLVANAQPNDALFLHYSGHGGQTE

DLDGDEDDGYDEVIYPVDHKQAGHIVDDEIHFRVVKPLQPGVRLTAIFDSCHSATVMDLP

YVYSTKGVLKEPNLAKEAGQGLLGALSAYASGDIGGVAKSVFGFAKQAYMGDDAYKKTIE

TKTSPADVIMWSGSKDDQTSADATIAQKATGAMSWAFISALKENPDQSYVELLNSIRELL

ESKYSQKPQLSCSHPLDTNLKFVM

>SoG_02526.T1

MNEDEAIQNIYKKIEREKVLINGANAMRAQTNNEGVRSRLDSQMRDARRNLQFFEEKLRE

LQMRRVNQGMGDVSLGAAGEDGAPPAPPPKDSDGNWSSGDVGNYGNAQYSQIGGHGDLMP

PRHPYAPPGPGSSMPKSRPNFTKLDLIKYDTPHLGPRIQLMLSQIQFKLNVEEQYLKGIE

KMIQLYGMEGDRKSKADAAARRVESKQKIVLLKQALKRYEELHIDFDSADAQDDDSINTP

NLRKPLSGQLAIRVLAVKDVDHAMTGRFTRGPETFVTVKVEDTVVARTRASRNDRWEAEY

HSIDVDKANEIELTVYDKPSEHPMPIGMLWVRISDIVEEMRRKRIEAEMNSSGWVSADRM

GSTGGAPAQFPMSPSQGGFNNPASPSGAGQEGAFGAPGPQPQVITGPIDGWFNLEPTGQI

QLEMTFNKTNVDRRPVDLGLGRKGAVRQRKEEVHEMYGHKFVQHQFYNIMRCALCGDFLK

YSAGMQCEDCKYTCHTKCYSSVVTKCISKSNADIDPDEEKINHRIPHRFAPFSNVTANWC

CHCGYILPFGKKNCRKCSECGLTSHAHCVHLVPDFCGMSMAVANQILEGIRTQKQRQAKG

TSLTEKTLRAGRMSPTSTHSPSPSLSGSIAASFPSGSQEAADAARAMYGTQSSQQRPPAP

DRTSSSSTAAAAASAAMSSQAQRPSDYGRYGGHEQPQQPQAQEDPYGQGQYQPQQRRYNP

ADYANVNPQYQSQPPQQRPAQQIPPQQPPAHQQMPPPHQQAQQQVPYQQPTPAPKPPSQE

PVQAPVASAGQVTAQRKALPLATDPGTGQRIGLDHFNFLAVLGKGNFGKVMLAETKRSRK

LYAIKVLKKEFIIENDEVESMRSEKRVFLIANRERHPFLTNLHACFQTETRVYFVMEYVS

GGDLMLHIQRGQFGTKRAQFYAAEVCLALKYFHENGVIYRDLKLDNILLTLDGHIKIADY

GLCKEDMWFGSTTSTFCGTPEFMAPEILLDKKYGRAVDWWAFGVLIYQMLLQQSPFRGED

EDEIYDAILADEPLYPIHMPRDSVSILQKLLTREPDQRLGSGPTDAQEVMSQPFFRNIVW

EDIYHKRVQPPFLPTIKNATDTSNFDSEFTSVTPVLTPVQSGKYQALLTLGFEGAIANTI

CSIRNITRYWERSYDQGRTKCLVKEAAIEVGHGSEECDMARSWSHQDDRTVESQMNQHVV

TSNLIASRNGIDDDEGYSESSKGSGFA

>SoG_02529.T1

MLRSKDLVSRQALIDALAAKQKAMGYMREVLGALDPAGSEVALAAVHFFLRWDLIDVNKS

DRRSWMSHLEGASSIMALLTPGNPGSAASRLLRDTVIADCFIYHILGSTLTSGGLATKIA

RYAFELLPVMQRVEITSYLSCPPGILQIILSASQLSDDTTFPDSRLTAADEALVLIDQAL

CFDIDQWAAQLRLLPNITDMESRVHIASAHRSAACLYIVQALPLVRSVRPVDTDMLVEDI

LAHLAAIDADDPYFKASSWPTFIAGAETRDAEKRTWALQRLMILWERVRWGYIFTAIEML

KATWAMQDARGGEDAGVNWLQDLKSMGFDSLIV

>SoG_02530.T1

MRYAFITSALIAGASAHGLVTSIEGANGVTMPGLSVADGTPRDCSSNGCGSQADTAIIRN

REMGSGRASALGRTQGNGPVNAAAVIAAFMGNAKGAAPTNNGTEGATGVEDDLSALQKAR

AQRREEHKRQVGNLFGGLLGGGNRNGAAGNRAAAGNRGGAGNRGGGRAGAVGLGGLLGGG

GNRNNRGPETMIADTTGMGSAKGLPTASDSGEVSMVFRQINQDGAGPLTADIDGTSGGTD

PNAFRRARVTQDVPGLGIQGLSLATNTDFPLKVQMPQGMTCDASVGGANNVCIVRVRNGA

AAGPFGGSAAFTQSKAARKRAIAYRLKKRMELDREELDSEELDLD

>SoG_02532.T1

MPGLPASVDLDECIARLYKKELLAESVIEAICAKTKELLMRESNVVHVRAPVTVVGDIHG

QFFDLIEIFRIGGYCPDTNYLFLGDYVDRGMFSVETISLLVCLKLRYPDRVHLIRGNHES

RGVTQSYGFYTECSRKYGNANVWHYFTDMFDFLTLSVVINNQIFCVHGGEYAFWQSFFLS

LDEPELTHKPGLSPSIHSIDQIKIIDRFREIPHEGPMADLVWSDPDPDRDEFSLSPRGAG

YTFGAQVVKKFLAVNNMNHILRAHQLCQEGFQVLYDDRLSTVWSAPNYCYRCGNMASVLE

VSDAGERFFNVFAAAPENDTHKDIQPGSEKAADGNTMPDYFL

>SoG_02534.T1

MARSTGRKLPSRHNPLMLDNVPEHFELVARRRLGQTKLTPKMIGEDVADDDTAELPHLDY

AHLRAPLPKGIISGIFKGSPASYFLMRRSHDGYVSATGMFKATFPYSEASEEEAERMWLK

SQATTSPDETAGNIWVPPEQALALAEEYKIVPWIRALLDPTQINPSGYVAGQTITAPPKF

DISKAAVNLAPPTPSSIPRSTRGRRSVSPTKESKRGTASPRKRATKSKVKAVDSVVETTD

LVNGTSTTKQDDIVMKTSEFEPAVVLDPREEDSKVKIDVKETVTKTKKGAETKTTTTEVE

LPLPTAGEPPSAEEISKMMEAAKEMVKAQKEAEGTPAKTPSKETPAKKSKRKAGDISVGK

DEKEAADDESKPEKEEEPRAKKVKTEVELRRAKVKNRALLGIGATVAVGYVHWFSRRGHD

LLTVLLQRSGAMARQLPLIIQPGSHCWAGEVKSFLCSQPGGGYG

>SoG_02537.T1

MGDIAVENPANSVPPHKKAAPSAIPTIDNFEGLPADGGDEYANLKKLQRQLEYIQLQEEY

IKDEQRSLKRELVRAQEEIKRIQSVPLVIGQFMEAIDQNTGIVQSSTGSNYVVRILSTLD

REKLKPSSSVALHRHSNALVDILPPEADSSIAMLGADEKPDVTYADVGGLDMQKQEIREA

VELPLTHFDLYRQIGIDPPRGVLLYGPPGTGKTMLVKAVANSTTASFIRVVGSEFVQKYL

GEGPRMVRDVFRMARENSPSIIFIDEIDAIATKRFDAQTGADREVQRILLELLNQMDGFD

QTSNVKVIMATNRADTLDPALLRPGRLDRKIEFPSLRDRRERRLIFSTIAGKMSLAPEVD

LDSLIVRTDPLSGAVIAAIMQEAGLRAVRKNRYNIIQSDLEDAYSSQVKGTSDENKFDFY

K

>SoG_02555.T1

MAMSLRHTSRAFGSVKLTRVFQPRQPVARVLGARGYATSEPDLKTVLKGVIPAKRELLKK

VKAHADKSLGEVKVENTLGGMRGLKAMVWEGSVLDANEGIRFHGRTIKDCQKELPKGTSG

TEMLPEAMFWLLLTGQVPSTNQVRQFSRELAEKAAIPKFVEKMLDDFPTDLHPMTQFAMA

VSALNYESKFAKAYEQGLNKADYWEPTFDDCISLLAKLPTIAAKIYQNAYRGGGALPAEV

DLNQDWSYNFAAMLGKGGKENENFQDLLRLYLALHGDHEGGNVSAHATHLVGSALSDPFL

SYSAGLQGLAGPLHGLAAQEVLRWILQMKEAIPSSYTSQDVHDYLWSTLNSGRVVPGYGH

AVLRKPDPRFEALMDYASTRPEIAQDPVFQLVKMNSEIAPEVLKKHGKTKNPYPNVDSSS

GVLFHHYGFKETLYYTATFGVSRGLGPLAQLIWDRALGLPIERPKSINLEGILKQVEGK

>SoG_02558.T1

MNIQPPAATTVVRGFQATPVTSADEDSDYNSGIDTPRLKSAQRGRTHRIDDVISANCSPT

LRPAASPSISGIAKLRMQMEPLSLEEASRSSSMSRNGSYQGRNVRYYKGMANSRSHSSDG

ARSDAGSDGSESYEVNLEHDFVSESVRDRNGFMDQFEGGLNPKRKMTTEDFETLRCLGKG

TYGTVHLVKQRLTGRLYAQKQFKKASLVVHKKLVEQTKTERQILESVNRHPFVVKLFYAF

QDQEKLYLILEYGQGGELFTHLNTEKMFPEPVAAFYMAEMLLAISHLHNDLGVVYRDLKP

ENCLLDADGHLLLTDFGLSKVSAEEDDDSCKSILGTVEYMAPEVILGKKYGKAVDWWSFG

ALGYDLMTGNPPFRGQNHAKIQDNIVKQKLALPYFLGPDAKDLLTRLLRKDPNKRLGASM

PKDLEAIKKHRFFRKIDWKKLAARELEPPIQPMITDPELAENFSPEFTELSISPVVTRDP

GFFNLAKDDPFGGFSFVAPSSLLEGNAFPMAAAP

>SoG_02563.T1

MSMTATDCTVVRDGFPRPFPDTPENVVEQFKMNGKVVAVTGAADGIGYAVAEGMAEAGAH

VALLYNSNDAAIQKAAALAEKHNIKATAFKLDGAHTRPTSTASIPRLLTRAQTVSNSDQC

QKAIADVVAEFGKIDVFVANAGMAISKPILEQTLPEYQKQMSVNVDGVVFCSKYAGEVFK

RQGFGNLIITSSMSAHIVNVPVDQPVYNATKAFVTHFGKSLAREWREFARVNVVSPGFFD

TKMGAGPLALNEAYRMAVLGRQGHVKEIKGLYLYLASDASTYTTGSDIIIDGGYVLP

>SoG_02585.T1

MPPRRSHKKSRAGCRRCKNRKIKCDEVHPRCGNCVKHGVACDFEHPDLRDELTPAPTPSL

NLPDSPPQPVTPAATLNIPQTPKPFAVQPSPSPMTVTPLMIQPSLPRMSRASIQVDRLME

LRLLHHYTTSTCHTLHTGAPTTQDIWQKAVPELAFRSGTFLMDAMLSVAALHLRAQEPKD

AALVRASHAYAASTLAEYCKLLDNGITADNAEALFLTATLIAFQASGSRIFLKEDADSNA

TEPGSRYVLPLPWFHAFQGVKTVVASSWQWIRTSSTVKTVIDAQPSFQLDMNPTGPHSFF

GHLLDDLESELATEDPAKIMSSRQAYSHAVCVLNWAHKNPTPGASLAFPATVSRRFIELV

EAKKPRALAILACFFALLKRLDGVWWLQDVTRREVMGLVGLFEPGSSWWRHLEWPIRIAV

WESIPIPAEVWGASCDGDGMSEVGLTDTMLNHVEMMADLVKQKYALPQVQVNGDIMLSVA

SPD

>SoG_02594.T1

MHQQIRAGPRVSSPASSPQTNPSRTNNPREATLPTRPRATSNVSSKDTMQSPTIDQPPSF

PGLPAESVRKLDQIIQNFFVKIAVIVLDSRIKVKATRGANGARRVNKWFQLETDELDDFK

EELRTWKASGSLENRPPPLIIELYLDTSTLKENQSLVIVDDDGKRWDVMERLNSSGSSAE

DETRGIPKKNSEVIIERWRVELTPLQDVPTEDFGPLLPTIYKKAIVFFRSLFVTTRLLPA

WKFASSGPSRTANPALIPRCRIRMSDETTGQPDALRHPIDGRPDPVTEYMFGDLEVPVGR

LSTSVTYRSDCSFRVDDSESLLSSRFMGVDENFFKPSLPQRAESTRTAEIGSLRDRPDRP

HRPDLADIRQTYGSLSTFHGEGHIGTSPMSALRSVKPPGSDTDSPPPSLPTHAIPEPPHS

LPASGRATASRPVARTGEGSSRRPSMSFQPFKAGSLSQSPGPRMMDHESPSSPHSLIRPG

LSALQQPRNRSSLTAGMPASLRGGPPPSTSAGDTAVVGSPRPVSTSGSRYSSSFTHRRGR

LSFGGASRAGDDEQGSSGRQSLASSVAQPGSGLLAETGGTSSGSLQADDDNISEFLKALD

SKKTLKSFDQPKRGESATNRTVAQLSKFHNMRESNNALTDSMTSSMQLQRSSSSSSRQLT

SVPGMVAPASLSASSSPGKPLSPHTPHTPAIPSRLSENSVASYSQGHSRASAREGRASGQ

APSTSRQSTITQGGTATAIDIPLSPRLGSYQRRSSSVAQQSRNTADDDENELAFARRSTS

LDTDTREPPTMSVLHDMHLQLEEESTLREDPTSSLQPAAEIQAGETGRRLRHRASEEAPP

DGLITASPSSSPFGRRRYTGLAGRQQRPTPSHSSRGSFTGSITRAGRDEDSVSEEPLVFD

LSEMDAQGRRSIEEGQGSGSGPSSSTRGSYEQRGITRRGW

>SoG_02602.T1

MIMSNARAAEDELQQSVMLSAEDAEYETADQGNDGSTTPGNSHAHDGETDGSDSGSSVSD

PEDRESEDRDASGEEVDDDVDASGDDEIDLVPQNAPNGAAGDDESEESGSEDGDEAVGAV

KSRNSLSEESDQESEVSSHQSAVEEESDEAPWEQDDEAGDDDAESEEAQANVCFFCKQDE

ENDPSEDFEAFLACRSCKNHAHQQCAREEEALEPGTSDKRWRCPDCDAITPKAEERDDSD

VDMEDDELAGPEVDKTSGATEDATRHGSAESTSSAVASRPRRKRKSPSAEPEDDTVSLRK

RRRNASSVPGSVTDPDAEPSGDQSRSQSRALRLKATRPSLVTIEKRSRTSLILTIRAPPD

KLKRHMVSKRTLQRRAAGARQRAAAAAAAAAEAQANVGIPALLETPFTADSYSQPFYSFF

DKETDDAKGKPYGGILTDAEADTTRTLPQPEDRRKFDQAKQKAEDEWRARVLAMQAQSEA

PARKSKKNDHASQIECIEFGGWEIDTWYAAPYPAEYSRNRVLYICEFCLKYMNSDYVAWR

HKLKCGTKHPPGDEIYRHGSVSFFEVDGRKNPVYCQNLCLLAKLFLGSKTLYYDVEPFLF

YVLCEYDDNGYHFVGYFSKEKRASSQNNVSCILTLPIHQRKGYGNLLIDFSYLLTKVEEK

TGSPEKPLSDMGLVSYRNYWRLILCRYFIRTLEQDNYKREGLSIKRVSEDTGMTADDIIS

ALEGLRALVRDPQTKMYAFRVDIEYCRQYVAKWEAKGYVQLKPEALAWTPYVMGRSNAVN

FELGPPINTIAPREDEEAKLDEGPNSLAQGPKPASHGLGDATARLEKAIEPKTEGDETNG

KGQKFAEADKENTTPGQESAEGESTDSWSLPYRNIPPTRFEVFPAVTHGRRNDRQRASRP

SAPRTSSSAPRPKRPSGSSRRSTHPRPKSSSSRRKSGGTGRGPGRWPKGTKKADYGNADS

GPGLPPGWTAKPGQSKSGSAGEHDRAEAEDDESAQEEVRVFVAPLETNGANGSPSKGGPA

VAAVEEEDAIMAEDVDAEGETE

>SoG_02622.T1

MAHMGFQMPGMPPPQLHQPPQIFGMYGPDGLPQLQHLPPDLAAQMFPDAHLYADDPHDAK

KRRIARVSPQSTQLYQPDAPACDMCRKKKIKCDGKMPACTHCTTYKTDCVFTQVEKKRTP

PKGAKYIEGLENRLGRMEHILRLSGILGEDDEDDLGEIERRLSEKQRDSAGAPSAPTSPS

QAPSGHGTPQTAFTSPEPGANKDKDEKRKSIDPSAAEEDKQDSEEVSALSEMMSSLVTNQ

FGETRYIGSSSGFSIFSPKGVSWVNERTGDDSFQKMISTVSVSDYKWTNWKPEVFSDLFQ

RPVFRPLPPKAEALSLLKDYFENFNCMFPLFHQPTFMHLVERQYSPDPYQGSGWWASLNI

ALAIAHRLRVMSNLVGPDEDEKAWDYLKNAMGVFSELTMRNTDLLSVQALLGMASFMQGT

PNPQPAFQLVAVAIRLAHSIGLHKRGTGFNLNPIEVEQRKRVFWIAYMLDKDLCLRAGRP

PAQDDDDMNVELPDADPEDQIGNIPLADGKGKMNLFRVMCEFTVIESNVYKRLYSTKATK

QSTGELLKTIGELDEQLEEWKDRIPIDFRPEHEIKASHTPLILHVVMLHFTYYNCLTTIH

RMSIHRGYWTSRLSDYAIKGLNSGPVNPRIFSSAALCTAAARASISLLKYIPQGDFSCVW

MILYFPVSALVTLFGNILQNPLDARAKSDTKLMALVVTFLSMLGQEAEQGGVHRMLAICA

EFERIAKLVIERAEKEQSSRRKRKTADSKPAAVTASSVASTTPRQASMSATPGASAGGVN

NTQANRESSQQLSPPNHGPASERGQSHSPMITAANEPSPSMASVGWPQEYSIPQAGGDFD

SFNGMDFTQTGLSGPPGASGMPSAPFQPGNPLLPQDLFSLPMTLDWSWAEMSGGAYPTVE

NGNFENFDQHPRQGAM

>SoG_02644.T1

MEPSHLMNRETIIQHPPPPPPPPPRALINVLILARTGRTMATPPSLFHALLRPTILQILR

ATGYHSTRPAVLDSVTDLAARYLSLLCESTARHAFHNHGDSADFDLVDVRLALQDAGALL

PQKVATEEEWAGEEDLRGVEEFVRWFAGQRMKEMMTVGAGDGETDATDYLNGSF

>SoG_02647.T1

MAAVASNRPKYALRNAPSPPPSSIANPANRQDWADDDDNEETSTDLPPPQTVSNKDGTKT

IITFRYNDAGNKVKTTRRIRYTTHTETVNPRVADRKTWSKFGASAKDGPGPATDTTSVGE

NIIFRPSVNWRKDEKDGDADAGAKAMKAQLKDKQVKCRICSGEHFTARCPYKDTMAPVGE

GAATADVAAGMPEEPAAAGPGGKKGSYVPPALRGDRGAGERMGGSKYGERDDLATLRVTN

VSELAEEGELRDMFERFGRVTRVFLAKDRETGLAKGFAFISFADRGDAVKACEKMDGFGF

KHLILRVEFAKKAA

>SoG_02661.T1

MAGGDIKKGANLFKTRCAQCHTVEKDGGNKIGPALHGLFGRKTGSVDGYAYTDANKQKGI

TWDDKTLFEYLENPKKYIPGTKMAFGGLKKEKDRNDLIAYLKDSTK

>SoG_02674.T1

MNRDASLTAGRMAAPRLQEVSGSHTLTKHLAIEDAKNTQKLVEDSCAIVGHDSPPYILSE

LIGKGSFGRVYKASRKTAPTQVVAIKIIGIEDADSFNPGTSDTLRDVLHEINTLKLLAEG

GAKNVNSIIDTLLVGSTIWLITEHCAGGSVSTLMKPTSGLPEKWIIPILRETAVAIQWIH

KHSIFHRDIKCANILITEGGHVQLCDFGVAGIISSRFDKRSTVTGTTAVDGPGVLRLQSV

LWDRG

>SoG_02675.T1

MSPSSTGDSPPVPSGTVRQFPLPARDNFTIEMPSSLAPMPNGSSILKSPSDLKVQRTSSF

SRDGILGAAQKARHLSQSSEALSNGALKDNSDDNSNPLKRRNTDAGVDYPRRRATIACEV

CRSRKSRCDGTKPKCKLCTELGAECIYREPGIKLDAGDKLILERLNRIESLLQMSMVNGQ

PNGININSDSPSMSNGTALSGDNVLLSGNGNSFVSVIPNGGFGTWTSQPQGTNISTMPKV

HTNAAMHLLQWPLIRDLVSRPYDPQILLQLEMAREPLHSLTKTPCVDLSNTQAYIEAYFE

RVNVWYACVNPYAWRSQYRTALSNGFREGPESCIVLLVLALGQASLRGSISRIVPQEDPP

GLQYFTAAWSLLPGMMTTNSVLAAQCHLLAAAYLFYLVRPMEAWNLLCSTSTKLQLLLMA

PSRIPTQQRELVERIYWNALLFESDLLAELDLPHSGVVQFEENVGLPGGFEGEEGEQVGR

DELWYFLAEIALRRLLNRVSQLIYSKDSMASTTSLEPVVAELDYQLTQWYESLPLPLQFP

FTRTVLQDPVQAVLRLRFFACRTIIYRPYILAVLDNEQAVLDPAVRESCHKCLEASIRQL

EHIVEHHAGHMPYIWQGALSMVSQTLLVMGASMSPSLLSILLTLVPHREALDQIINDVVM

EVERYAFLAPSLSLAAEIIKEAEYAWGVRIQGNIQQHAASRSSLKMQVDGASPRTGWQSY

RPKNGPTPASQKMTGRTKQTDQLAPGLPRCVRAKRMVEFNYRSAGGSALEGCVHPESDDS

PCMGEPRTFAPSQAKCFVRY

>SoG_02676.T1

MLRSRHTSRALKALGQTRSFTTTSAAAAVQALKKVPATTRNQATAAAASQAARPVPSTAF

NTDKSHVQPLVNARRQEMDESFIGKTGGEIFHEMMLRHGVKHIFGYPGGAILPVFDAIYN

SKHFDFILPRHEQGAGHMAEGYARASGKPGVVLVTSGPGATNVVTPMQDALSDGTPMVVF

CGQVVTTAIGSDAFQEADVVGISRACTKWNVMVKNVAELPRRINEAFEIATSGRPGPVLV

DLPKDVTASVLRKAIPTDVTLPGLPSAATRAAVELRDRQIQESIKRVADLVNIAKQPVIY

AGQGIIQSPGGPEILRELADKCSIPVTTTLHGLGAFDELDEKSLHMLGMHGSAYANMAMQ

EADLIIALGGRFDDRVTLNVPKFAPGARAAAAEKRGGIVQFEIMPKNINKVVQATEAVEG

DVATNLRHLMPHLQSKSMEDRSAWFGKINEWKAKWPLSHYERSDRNGKIKPQTLIEELSN

LTADRKDKTYITTGVGQHQMWTAQHFRWRHPRTMITSGGLGTMGYGLPACIGAKVAQPDA

LVIDIDGDASFNMTLTELSTAAQFNIGIKVIVLNNEEQGMVTQWQNLFYEDRYAHTHQSN

PDFIKLAEAMKVQCRRVEKPDDVRDALKWLIESDGPALLEVVTDKKVPVLPMVPTGSGLH

EFLVWDEVKDKKRRELMLERTCGLHGH

>SoG_02686.T1

MSTSAPSNADDPTRRAIRCDKDWPCSNCRIAKRTCTSTGAGQRPKESRQRVLISSQYEKK

IDQIESRLGSIETLLRGLSIQSGCPSGQETFTTKACSFDTPQSSNNSGPAVNSADYDSSD

HNSEYGGDTGLHSQANFASEFLEHAVERTSLTDVNPKMSEALTTLRQLVALGNRRSISHG

PRFPLQRPLPPGGVTKLSLPSQVIVTALLKENKHIQDLPGMVRDVYFATEDFSEATFAIV

NAGLHMLFIEAIAYGSTVASKGELENCLRISRANLETCLLHMPLFQSSKTENAQALYLGA

LYAIDVCRPSVAWQLTSMAAQLCQTGGYHRAECLKSDDPKVARTKTLIFWQVYTLDKNLS

LRLGRAPTIADYDISIPREYNLHGLIAQGANADYAGLWLKIAEIQGRIYEQLYSPTALKS

SGEELVQRARVLAAECEAMEREAQVTREYTNEFFKLSKRSELLELFIKGDELQFLATWSL

VQRVIPAPENAVSRFNEGCLATARRAMALHQDCIRLFKMGNHVQSIYIHWNLMLTPFAPF

FIIFCYVIETTSREDLAMLQDFASTLELAREASEVSRRFATLCRVMCEVLALYVEAKSQQ

SNNQNLRPIGDEFEMYLNQLGFMPNMAHPDQGNTPIAGASGQMSASQQPAMVGEWYSGSM

NMMGLVEEDLSSMAMGFPAGMGGMGGDGMGM

>SoG_02687.T1

MPPRHQTLPAAQTSSAREAQKSFYCELCSKGYSRMNDYEAHLSSYDHSHKQRLKDMKAMV

RDPNAGARARKAEAKADGIINIKLSGQDQQSGPSTSGGFKKGGFKKSGFKSAFAPAQGAA

TAPPSSTTATKPATNDSAAAVKPSLTKSLVQEESDTEDEGYEVYDPRFPTD

>SoG_02705.T1

MATGELPATALPGRVLGPITKFLPGPGTHVYAGQVVSSLMGHVSVSQPTQPAGPAKRLNK

LTTPDASTTQNLPTLSVARHGRRREVLPDVNNIVLARVLRLMPRQAIVTIQQVGETVLQT

EWQGVIRVQDVRATEKDKVKIHESFKPGDIVRAQVISLGDQANYYLSTASNELGVIMATS

EAGNDMVPVSWKEYKDPETGISELRKVAKPT

>SoG_02710.T1

MSSHRPNAFNSLRMGEVIREKVQDGVTGETRDLQYTQCKIVGNGSFGVVFQTKLSPSGED

AAIKRVLQDKRFKNRELQIMRIVRHPNIVQLKAFYYSNGEREFVPETVYRASRFFNKMKT

TMPNLEVKLYIYQLFRALAYIHSQGICHRDIKPQNLLLDPNSGILKLCDFGSAKILVENE

PNVSYICSRYYRAPELIFGATNYTTKIDVWSTGCVMAELMLGQPLFPGESGIDQLVEIIK

VLGTPTREQIRTMNPNYMEHKFPQIKPHPFNKVFRKADANAIDLIARLLEYTPTERQSAI

DAMVHPFFDELRDPKTKLPDSRHGSSQLRELPDLFDFSRHELSIRPDLNQQLVPPHMRSV

LASRGLDIDNFTPLTKQEMMAKLD

>SoG_02712.T1

MALSTMDSLSSALLTNPVAAALGDALNSFSERRAKLGLSNPGTIENLSREVQRDVFLTNQ

MFTGLRADLTKIFSMNPMFQVSHAFAMGGERMNPYTFAAIYGTSKVFAQGSFDNEGSLSG

RFNWRWTDKLVSKSQIQISRDDQNMAQFEHEYTGNDFSASLKMINPSFLEGGVTGIYIGS

FLQSVTPKLALGMETLWQRPSLSQGPECVTSYCGRYKSNDWVATAQLQAATGTFNTSYWR

RLSEKVQAGVDMSLGLVPSPGGLMGGGLQKEGVTTIGAKYDFRMSTFRAQVDSKGKLSCL

LEKRVAPPIMMTFAADIDHFTQSAKLGLSVSIEGVPEELQEQQEMLAAQPSPNIPF

>SoG_02721.T1

MSLKQEIETWVAALARYDNNEFEEAVNEFEKISDTSKILFNMGVIHATLGEHEKAVEAYQ

RAIRLDQYLAVAYFQQGVSNFLLGDFEEALANFNDTLLYLRGNTMIDYAQLGLLFKLYSC

EVLFNRGLCYIYLQQKDAGMQDLAYAVKEKVVEDHNVIDEAIREEAEGYTVFSIPVGVVY

RPNEAKVRNLKTKDYLGKARLVAASDRSNAFTGFAGAEIRNAAKTESKDDRPTENVSYAA

TNLVKPGLQSRRQQSEPPTNRSNVFPPTPPPENDRPSRGASVRSGRGPMPAKLTIQTQEL

GRKYEKAGSPESRSARPGRSASATPARQYSTRDGPQRSRTARPIDEETEDAYPDELYDMY

SAGGVRASRGSRGQAPRQRQQQKYIEEEEGSDYDDGSFDEGDFEMISTNRRGPGSVSSGG

RAGSRRPEVRKFRVKVHAADDVRYIMIGAACEYPDLVERVRDKFELRRKFKIKVKDDDGD

MITVGDQDDLEMAISGSKANARKQRLDVGKMEVCVLVTPTNLHSSESLTCPSTALGD

>SoG_02745.T1

MSFLGGGAECSTSGNPLSQFQKHVQDDKTLQRDRLVSRGPGGQLNGFRSQNVGGSQDEMM

NGFLNGAPSMQQEMPMQGGPAQLPQHHPAQLRASSTSPTWAHDFNSQPGMEATHKPAGVP

FLNADEFARFQNMHSQSPMSHPEAMQNNVSPQMQQSRPMMGMGMGMGMNYGQPMFQPMYQ

NQQYHQQQKEPEGKGKGRLVELDDNKWEEQFAQLEVQDKAEAKAKEDEEANAAERELEDM

DKGIQSETNEFGDFESIWRGIQAETAAARSMVNEEQFFDQFDAQWNKDSLTDLSHLDSWG

RYGEPIPEQYRFEEENIFQDSKNAFEEGVKIMKEGGNLSLAALAFEAAVQQQPDHVEAWV

YLGSAQAQNEKETAAIRALEQAIKLDPNNLDALMGLAVSYTNEGYDSTAYRTLERWLSVK

YPQILDPSDLHPPAEMGFTDRQQLHDKVTKHFIKAAQLSPDGEHMDPDVQVGLGVLFYGA

EEYEKAVDCFQSALHSSELGTSNQQEQVHLLWNRLGATLANSGRSEEAIAAYEEALNRSP

NFVRARYNLGVSCININCHHEAACHFLAALEMHKSIEKSGRAQAYEILGEGSGARVDETI

ERMSAQNRSSTLYDTLRRVFSQMGRRDLAEKTVAGVDPAIFRPEFDF

>SoG_02746.T1

MKSFFGVQAATLASVLLARSHAAVVEHDFNVTWVTANPDGMLDRPVIGINGQWPIPRIDA

NVGDNVVIHLHNQLGNQTTSLHFHGLFMNGTTQMDGPSQVSQCPLQPGDSMTYNFTIQQP

GTYWYHSHTISQYPDGLRGPLVIHDPEFPFRKDVNEEIVLTLSDWYHDQMQDLIPPFMSK

GNPTGAEPVPQAALMNDTQNLTISIQPEKTYLFRVVNMGAFAGQYLWIEGHNLTIVEVDG

VYTKPTVAEMIYLSAAQRCSFMVTTKKGETENFPIMASMDTSLFDSLPDDLNYNVTGWLV

YDDKKPLPDPQPVDSFDDLVFDDMTLVPYDDMERLGAPDRTVELTVRMKNLDDGANYAFF

NNITYKSPKVPTLYTALTSGDLATNPAVYGDYTHPFVLEKGEVVQITLNNQDNGRHPFHL

HGHHFQALYRAPKAGGDFNATENPDDSFPKTPMRRDTVVVWPRGNIVLRFRADNPGVWLF

HCHIEWHVTSGLIATFVEAPLELQKNITIPQGHIDVCKKGNYAVSGNAAANTVNLLDLSG

QPTPPAPLPAGFTPRGIVAIVFSGVAGVLGMIVVAWYGLSGAAIEAGKPAGGGVGLVSQH

EPDAAEEAASHEGSASDAAGAVKPVETSRATAQ

>SoG_02747.T1

MSNVFAVPIFVVVFREALETVIIVAVLLAFLKQTLSGPGSDEKVYKKLRKQVWLGLLTGF

FICLVIAAAIIGTFYTVGRNAWEANELYYEGAFSLVAAVIITIMGVALLRIGNMQQKWRV

KLAQAIEAPLKTSKGPRAWFGRAMEKYTMFLLPFITILREGVEAILFVSAVSFSAPATAI

PLPTVVGLIVGGIVGWLIYKGGATAKLQIFLVASTCFLYIVAAGLFSRSVWYFEQQKWNE

VIGGDAAELGMGPGSYDIDKSVWHVNCCSPEFNGGGGWGILNAIFGWTNSATYGSVISYN

VYWIAVITGFLVLRYKEVKGHWPLLKGKGKAKATKPEVHDASANSPGRMEENVGISEKSA

TTAAGGEVRP

>SoG_02752.T1

MGFFETFTLALAASSVGLVAADGLNARAKVLGKYMGTEYNVGELSESTFMNIANNLEEFG

SAVPGNEQKWDATEPNRGQFSFSQGDRIANNVIQAGQLLRCHTLVRNGGFNNQTLISIMQ

NHIQNVVTHYKGKCSHWDVVNEALEENGSYRSSVFYNTIGEAFIPIAFRAAAAADPNVKL

FYNDYNIELAGAKSSGAQRIVRLIKSYGARIDAVGLQAHLVVGQVPSYTQYQSNLKAFTD

LGVDVAYTELDIRMDLPSSSQKLQQQATDYGNVVKACALTEKCLGVTMWGLSDAHSWVPG

TFPGTGDALPWSSSYQKKPAYSAILNAWGSGGGSGSSSSSTAQGTITSGPATTANTLTTR

AATTTSAGNGGQCAAMWGQCGGSGWQGPKCCAQGTCKYSNDWYSQCL

>SoG_02758.T1

METAAICAKQGLTPPTDSREETESPPQSVTYARAACTECARRKQRCNREWPCNHCKKRKV

GNQCRYHHDASVGGGKPSPPTQDGRKKRGRSEEEGQSEIDNVGDAVDYGGDGFEALGYST

AHLFAGLTTRNEATPRKPLPKQYFKDAESCPQLARALQVLPPRPHTDHLVQNFLSNVNFH

YYIIYGPDFLEEYQAWWKDRAAHKPLSIQWTCLLIMICACSCQYTDKELQRRLEEDLAES

VQRLTERYHGAARELYSIISVGHGHLLNVQVLLHSCYWYKGEIRFVECWHVLASAIREAQ

ELGIHQEQLAGPLPEFEREMRRRVWCILDTWDWQISSLLGRPMIIDRSDCEIGLPSLSLE

DFDPSPLLHMKLQSQIIGKMAGTFGLPKYVVTPEDVQKYQRMLEIWMQSFPACYDFDRPD

FSRDKKQPWMKLHRHYLHTMSNSMMLDPIRAYLAKDMSRQSPQDELQIRRDGIKYCLRLM

KALHGFFGHVYPRDSKFHFVIFCVFDTASILCSTIIHDQDDSAPKKDEMLEAIDGALGML

KQLSDVIDNAKTSYQVLGRIAARARVAAGRLETSGQAKRTRFTSVFDTAPAAMTNTPSSD

RIDGSVPTMPQDSNANFYTQPNAMAFQPLPATAAKEQNGHHNEFQVEQVSPPMGYPITEP

YLMPNFMPQRAYDPNAPAVYGMDPVMQHTTILNDHDVIPSYPDGGAMNGQGLPGSADVSH

VDVSNITEEELGQLAAMWQYQTLNLDFINPQP

>SoG_02762.T1

MNGLASPIEGKSKLPFVWPEHLNLALLNRLPERYHVGRRRSRSKSRRGEEDITSLQTSFN

MWDGVRDLQKHHFKTSDLQYVFLICLTLFSLYVAPPAPGIKLLALMATAWVLLMPATRQF

FLPSSMIWVWLVYFFCSRFIPYDYRPHIWVRVLPALENVLYGANLSNILSAHKHPILDIL

AWLPYGIIHFGAPPVCCLIMFIFGAPGTTPVFARAFGWMSILGVTIQLFFPCTPPWYENE

NGLVPAAYGMPGSPAGLARVDEIFGIDLYTTNFTNAPLPFGAFPSLHAANAVLEALFMSH

CFPQFSTFFAIYAGWVWWATMYLSHHYAIDLVSGGLIAAAFFYTCRAYWLPQRQEDKLTR

WEYDYVEIGDRHKPRDEEFGQFLGLGLIEPRRASSSDGWTLGSSSSGSFSSGTMSPTTSE

ESSPGLELLRGEAWHGTVPPRDVELSEVVSR

>SoG_02781.T1

MARSLPLVAAALLGLASAQTPDNTPEVHPSLTTWKCTKDGGCKAQDTKIVLDSLAHPVFQ

KNAPSFNCGDWGNAPNSTACPDAKTCQENCVMQGIPDLSQHGVVTTGDELYLDMLRDDGS

VISPRVYLLSPDEQTYEMLELTGNELSFDVDVSKLPCGMNGALYLSEMLPDGGKSDLNKA

GAYYGTGYCDAQCFTTPFINGEPNLEGYGSCCNELDIWEANARATHLAPHTCNQTAVYKC

AGAECKFDGVCDKNGCGYNPYAQGNPGFYGYEKIVDTTRPFTVVTQFPANAQGELVEYRR

FYIQDGKKLDNPPVRDANGTSTGKNWMDDPHCVATGAKRYMDLGATKGMGEAMARGMVLA

FSVWWSEGDEMKWLDQDKAGPCKPGDGAPSNIRKIQPDTAVTFSNIKWGEIDSTYKLRDK

CKRTHIQHRI

>SoG_02796.T1

MMSSSKMLRKKKNVKKGIQFCLMVCGASGTGRTTFVNTLCSQGVLQHKESDDANDAHVEE

GVKIKPVTVELELDEEGTRISLTIVDTPGFGDQIDNEASFAEIEGYLERQYDDILAEESR

IKRNPRFRDNRVHALLYFITPTGHGLRELDIELMKRLAPRVNVIPVIGRADTLTPSELAE

SKKLVMEDIEHYRIPVYNFPYDVEEDDEDTVEENAELRGLMPFAIVGSEDIIEIGGRKVR

ARQYPWGVVEVDNPRHSDFLAIRSALLHSHLADLKEIVHDFLYENYRTEKLSKSVDGAGG

IDNSMNPEDLASQSVRLKEEQLRREEEKLREIELKVQREINEKRQELLARESQLREIEAR

MQRETTAQASPAPEANGDHQQ

>SoG_02802.T1

MIAHDGKVTLLLTTDLDQRSGYRHDSIALSTIAPSETSRHWSNASTQPPPLFAGTSRTAQ

FLSSLEEPDAAAQLPAFVRPLSSKIAADDVAYLFAKGALTLPSVPLQNALLQAYVEYVHP

YMPLMDLHSFLSVINSRDGLNGQCSLFLYHAVMFAATAFVDMKHLREAGYSTRKAARKAF

FQKTRLLYDFDYELDRLVLVQALLLMTYWYETPDDQKDTWHWMGVAISLAHTIGLHRNPG

TTSMAPPRQKLWKRIWWSCFMRDRLIALGMRRPTRIKDEDFDVPQLEEDDFEIMALPDNI

TVIPPECALMRDVAMQRELAVMCIYKAKLCVTISHMLKAQYSVLIRDHMKPENTTNSTMM

LFPNKEMDNLDKINEVDMELLAWAESLPACCQYRTLTPMDVQNGKSTMAVQRTLLHMVYY

TTISALHRPQFLPSSPLRIPTASRSVQEMSRVRVRGAAMHITRMATELHHLRLERYLPTT

GVTVILPAMIIHLLEMKNPSVEARERATRGFRQCMRVMEKLREVYAAADYATGFLDAALK

KASINVNPNIAPSSLAILKSATNYSAQTPPPDNLPYMTASETLFNEKPSAGEARMPMMPP

ETINGAALDLSSGTAGNLNGTAGTNSPPHTDLGSAADLTPSASGGSDVGNGGVEQLDAMD

LDFMQGHDEFDWNAVAGTDFDVDQWLQFPPEGVNGGGEDGSAVAGAMRRDQDVVMATGQT

VGWDGSMATGGRRDAAPALA

>SoG_02812.T1

MGFKTFGFAGGRADTWEADESTYWGGETTWLGNDVRYADGNPGSAASGVVDADEKHAHKD

IHNRKLEEPLGAAHMGLIYVNPEGPDGNPDPVAAAKDIRTTFGRMAMNDEETVALIAGGH

TFGKTHGAASSDNVGPEPEGAPIENQGFGWVNSHGSGAGPDAITSGLEVTWTKTPTKWSN

QFFEYLFKYEWELTKSPAGANQWVAKNAEPIIPHAFDSSKKQLPTMLTTDLSLRFDPEYE

KISRRFLENPDQFADAFARAWFKLLHRDLGPKARYLGPEIPKEDLIWQDPLPAVNHPLVN

ESDVAALKQAVLQAVPDVSKLVATAWASASTFRGSDKRGGANGARIRLAPQKDWKANNPQ

QLAQVLGALENVQKQFNAQGNGKQISLADLIVLAGNAAVEKAAQDAGHNVSVPFTPGRTD

ATQEQTDVESIGHLEPFADGFRNYGKSTSRVKAEQYLVDKANLLTLTAPEMTVLVGGLRA

LNANYDGSSHGVFTTRPGVLTNDFFVNLLDMSTQWKSAGSDDVFEGVDRKTGAKRWTATR

ADLVFGSQAELRAIAEVYGSADGAEKFANDFAAAWVKVMNLDRFDL

>SoG_02825.T1

MPSFADSFWSSDYAAGLGVLFGKLNQGIQENRQVLAIARLRAEAEDTYGQRLSDIVPSAN

KITGGFGQDDGATARKAFDGMRGEMEDAARTHQRMAQSIRDLVVNPFSRWCDAHEDRVSA

SQEELQSRLKAHDRQAETVKKLRSNYFNKCRLVEDLEEEEKLGFQDPEATSTPPSGQAIP

EIKVQPHKDPVDEEDEELYEIGDEVYQPEQMKKILSHMLSTITMGETKVPILGTYTNTSC

GSDIVEYLQRSMGSTSVSYAERIGQDLISNGLLRLIGNVGSTFANSSKMHYQWRPKAFKL

AGLPEKKQALSRTFSMPTAGSEGSDSPVVGTVSEYLEKWNMLPNQYPNETPGQKLQREAR

EADTRYKAGVRRLDELRCELEEAIMLHLKFLERCELDRLKALKTVILDFSGTLGNVIPSL

QSTVDKMVLFQETIQPEGDLRYLLENYRTGSFVPKVVVYENYYNRADDQTFGVDLEARAR

ADKKRVPVIVTAILTYLDHHYPELEGDEARRGVWLVDVPLAQTHKLRALVNDGKNFTPDL

FANFDIPTVASLLKLYFLELPDSLVSSHVYEIIRTIYTSQSTDTSEANRIGILQQTLGQL

RLTNIATLDACMNHFTRLIELTSADEEYVAAFSANIAPCILRPRQETSLTMEEKHAYKLV

RDLLAHKEPIFSELKRQSSLSHSGSIGKGPAVGGIGNRPRAISTDESNRKALMEERNKAL

LEKVSSSRGRAVSPAPSPRGTHRRDRSTGGPEGRFPIQVASPTQSGNERHRSSLGSAVKR

SSLEVPGADGVSSPDLNGSGGSLEDSPAPSEKGHARGRSTTKFVGGQRISVPVSSENEPP

ATRGVTLEDKPMED

>SoG_02851.T1

MSTQITNPPYVYIVINSPFRSNGRASWRAAGQEACKLDKRRHHVEAWRGSELGCLQDEAE

SMAAAAAEAGREFDREAFQAQGTRDINKEAARQEREATYSIGNACWKGDSSISPIRGLLA

TWGLTPDDISVAYMHGTSTVLNEKNELEIMQRQLSHLGRSRGNRLDHPD

>SoG_02861.T1

MVAFTNIALGLGAAAALSLAAPAPAPAPAKSPLEERGPMNFVMGYDHPLSKRFGNFSVRA

IGPRSDTNYKQDYRTGGSVNFSPGTNSFSLNWNTQQDFVVGVGWNPGGHSPITHSGTFSV

NSGLGSLGVYGWTTNPLVEYYIMDTNVGINTGGSQRGTVTSDGATYAIWEHQQVNQPSIQ

GTSTFNQYISIRQSPRSSGTITVDNHFKAWAALGMNLGTMNYQVIAVESWSGSGSAQQSV

SNTGSGDSGGGGGNTGGGNTGGGNGGGNNGGGGGGNNGGGGGNNGGGGGSGGGSCSAIWG

QCGGNGWTGPKCCSSGSCHFQNDWYSQCM

>SoG_02869.T1

MMAQHASPYMFLNSEAKKQAAMFPNFPAMPSTPVYSRPGSSSSQPPTLYSNGPSVMTPAA

SPHPANSKPSIVLDTDLGEASYFPSTPPLSTAGSTIGSPKSLDVLQTPMNPMFSGLDDLE

MGKDGFESGEFSILDWSSCASPPMTPVYIQSQPGRAPSLTSTTSDLLSATSAPSPAAYAR

SVVSELDVDFCDPRNLTVGAGSANTTLAPELSLDALEGLKPAAAKSAAAAAPAQPTFDFS

SHLPNGTLFEDLSDIESDEDFVNSLVNLGSEHDSTDCGRPRACTGSSVVSLGHGSCFGDE

ELSFDDSEAFQFTIPSPPSTTCSMDGEHPAKRVKKSHKKENRTSAPVMTAAASGDAEVVE

DAQGSPKDTAASDSNASSGSDGSAPLPAPVNRRGRKQSLTEDPSKTFVCELCNRRFRRQE

HLKRHYRSLHTHEKPFECNECGKKFSRSDNLAQHARTHGSGAIVMDLIENGEVGSFDGSV

MMPPVPGSEDYSAYGKVLFQIASEVPGSASDASSDDASDGKKKRKRSD

>SoG_02875.T1

MAANTASGAVDQLASDLNNTSLDAKAPAIDTTVGDASEDAGPTPNSAAPHPQNSASLYVG

ELDPSVTEAMLFELFSQIGAVASIRVCRDAVTRRSLGYAYVNYNATSDGEKALEELNYTL

IKGRPCRIMWSQRDPALRKTGQGNVFIKNLDVAIDNKALHDTFRAFGHILSCKVAQDENG

NSKGYGFVHYETDEAAQNAITAVNGMLLNDKKVYVGHHIPKKDRQSKFEEMKANFTNIYV

KNISGEVSDDEFRDLFAKYGEVTSSSLARDQDGKSRGFGFVNYTTHEAASKAVDELNGKD

FRGQDLYVGRAQKKHEREEELRKTYEAARLEKANKYQGVNLYIKNLNDDVDDEKLRQMFS

EYGPITSAKVMRENYSEESEEKKEEEKKDEEEDAKDKENKKDSSSEEEGAEKKSEKKGDK

KLGKSKGFGFVCFSNPDDATKAVAEMNQRMFDGKPLYVALAQRKDVRKSQLEASIQARNQ

MRMQQAAAAAVGLPQQYMQPPVYYGPGQQPNFMPQGGRGMPFPQPGMGMPAPQSGRPGQF

PAYPQQGGRGGAPQQIPPNMYAMPGQFGPQFGQPGTPQFMAAMQQAAMGGGRGGPQGGRG

MPGAPPNGMQGYPPNNRQQGGRGNGNGRNGNFPQGGRGGDANQTSLIQAQLAGAPPAQQK

QILGEMIFPKIQAINGELAGKITGMLLEMDNSELVNLIEDEAALKAKVDEALAVYDEYVK

SQAPAEGAEAKKEEETKA

>SoG_02888.T1

MLNNGLATPLFLAIGFAAALADAGSVSKRDIRDEYPSRPYYPAPFGGWLDEWTESYAKAK

ALVDSMTLAEKTNITSGTGIFMANISSRRCNGNTGSALRVGFPQLCLNDAANGVRQADNV

TVFPDGITVGATFDKKLMYERAVAIGKEARGKGVNIWLAPAVGPMGRKPKGGRNWEGFGA

DPVLQAVGARETIKGIQEQGVIATIKHLVGNEQEMYRMYNPFQYGYSANIDDRTMHELYL

WPFAEGVRAGVGAVMSAYNAVNGTACSQNPYLVNGLLKDELGFQGLVMTDWLAKMSGVAS

ALAGTDLDMPGDTQIPLFGNSYWMYELSRSALNGSVPMERINDMATRVVAAWYQMGQDSE

SYPRPNFDTNSYDREGPLYAGAWPASPRGIINEFVQVQADHDVIARQVAQEAITMLKNND

GLLPLSTSRSIKVFGTAAQVNPDGPNACADRNCNKGTLGQGWGSGTVDYEYLDDPIGALR

SRASDVTFYNTDKYPSNAVVGDDDVAIVFITSDSGENSYTVEGNHGDRDASRLFAWHNGD

KLVKDVANKYKNVIVVAQTVGPLIFEEWHDIPSVKTILIAHLPGQEAGDSLANVLYGDAS

PCGHLPYSITYKEDDMPESVTKLIDFALFNQPQDTYSEGLYIDYRWLNKAGIKPRYAFGH

GLSYTNFSYSDAKIEKVTQLDTVPPARKPKGDVLNYSQDVPDFKEAVQPDGFSKVWRYIY

SWLSERDAKNAAADRESKKYPYPQGYNEDQKPGPRAGGGQGGNPALWDVAYKLSVKVTNT

GKKHSGKASVQAYLQFPKGISYDTPVIQLRDFEKTSTLAPGESETVELTLTRKDVSVWDV

VIQDWVVPDVDGAYKIWIGAASDNLGTVCNVDGLKCESGVSGPI

>SoG_02891.T1

MASDSSDDDRPLARSNGHHEAMDRAIPNGGNSRAGISIRNGPVDEMDIDSSLNGTAKRKS

RGSLSKVNYKDGSDSEGEPLAKRPKRKPGDDSDSDVPIKSRVKKLPPSIKETSLLESSDD

EKPLGQKLAAKKAAIERKAEKVAKASRIKEDSDDEPMMKSSANKRASNGTAAKRKSNGIK

DESDSDVPISRKAKAKSATPAKKGKDVKKAKDTTPAEEDDEHAWWNDPTGGDDSIKWRTL

EHNGVLFPPEHEPWPKNVKLKYDGTPVTLGLEAEEVATFWVAMGPPETSQHVQNPVFRKN

FFGDFQEYVKKYGATDAAGNKVDIKELEKCDFSKVYEYWQAKVAANKNLTKEEKAAKKAE

KDKLEEPYLYCMWDGRKQKVGNFRVEPPSLFRGRGEHPKTGKVKRRVMPEQIIINIGKEA

KVPEPPPGHQWKEVKHDQTGTWLAQWQENINGAYKYVMLAAGSDIKGQSDFKKFEKARSL

KKHIDKIRKDYTRELKSPVMADRQRATAMYLIDRLALRAGNEKDTENEADTVGCCSLKYE

HITLEAPDKVTFDFLGKDSIRYHETTTVDLQVFKNLKLFKKSPKTDGDDLFDRLTTAQLN

KHLQSYMPGLTAKVFRTYNASWTMATLLKELHKNPLSRGNVMDKVKLYNDCNREVAVLCN

HKRTIGAAHENQMAKLGDRIKGLRYQVWRTKKMILDVDPKQKKKKGAEYFELDSDIDDEW

VKKHQEFLVEEQRTKIEKKFEKDNEKLKANKERPLPQSELKERLQVVKELESKFKKENKT

NKVEAEGRGPTVEKFEGAIEKLEERIKTLQTQAEDREGNKEVALGTSKINYIDPRLTVVF

SKKFDVPLEKLFPKTLREKFRWAIASVEDQDDWEF

>SoG_02895.T1

MLNRLHGQPESYDKKSKYKFGRTLGAGTYGTVREADSPFGKVAVKIILKRNVKGNEKMVY

DELEMLQRLKHEHIVKFIDWFESRDKFYIVTQLATGGELFDRICEQGRFTEKDASQCIRQ

VLSAVDYLHRNGVVHRDLKPENLLYVTTEPNSDLVLADFGIAKTLDSKEDSLQTMAGSFG

YAAPEVMDRQGHGKPVDMWSLGVITYTLLCGYSPFRSENLKDLLIECTQNTVVFHERYWR

DVSEDAKDFILRLIVPDQHKRWTSEQAMHHRWLSGETATDHDLLPDLRSYRARQKLRRAI

EVVKLRNRIKALKEKDEDPENSDMADLDDEKEGGDGARLHALGLFALKDAKAKQESLQVE

ENLAKEAKRRSGQFSG

>SoG_02911.T1

MLEFRTQGYNPYAVKYSPYYDSRIAVATSANFGIVGNGRVFALGLTAQGVQVEKTFDTND

ALYDLAWSEINENQLIVACGDGSMKLFDLGVNDFPVMNFHEHKRETFSVCWNPVSKDTFI

SSSWDGTVKLWSPTREFSLKTLPVGNCTYSAAFCPSNPALISAVSSDSHLRLFDLRIPSS

AKYHLTATIPVHAPPQNPAFPSPQTGPTTPPAEILTHDWNKYNDTVVATGGVDRVIRTFD

IRTPTGGPTAILQGHDYAVRRLAWSPHASDVLISASYDMTVRLWNDVSRRQVDPSMGARA

GMQMGVMNRHTEFVTGVDWCLFGMGGWVASVGWDERVLLWDSNMLMTR

>SoG_02912.T1

MDTFLTRHKRKSSGSPDPLPGHNDSEDDEPTEFKLAVLASMFPEMTQDVLLDVLLANEGS

VPASSAALRAAATIPRKAGPGAIGHQQSLKSFATMAESLSPQKKKIKSKKGSTLHLYSPE

DVAEYTPCTIIHNFLPTEMANDLLREMLKEAETFESSTFKLFDNVVSSPHTTSFFVESYD

DIRKQKTEYLYNGSSLTDVRRITPQLSKVKPLVQDAVNREIQHRIKTRYPGGKKLKYQSP

HPWVPNSAFCNCYDGAQQNVGWHSDQLTYLGPRAVIGSISLGVAREFRVRRVLPKDGDSK

EALDPDAEGQISIHLPHNSLLVMHAEMQEEWKHCVTPAPSIDPHPIAGNKRINITYRDYK

ANMHPRGTPKCSCGIACVLRVVQKKKENFGRYFWMCHAGNIPDKEGCSFFSWAHFDDDGN

PKLADVSQDKKARRQSRSFD

>SoG_02932.T1

MSEVASRSSARGGRGSARGGRGGFAGRGGRRTNGDSKADDTTTGAFDDEGDFAELRKQYG

EKTSVIREMFPDWSEVDVLYALQETNGDENEAVTRIAEGTISQWDEVSKAKKAPRKPKET

AAPTTSADTTGGAPRPARGGRIASEGGRGRGRASDRGGRGGRGGRPAQAPATNGSRKENH

QLSVSTEESTAWGETKANAAEPEAPATAPEPTKPAAPATKTWASMLRQSAVKPQPKPKEP

VAPAVQPEEPTPAPETTPAEPEPVEAPDERTPVAEPAEPAPAPAPAVIEPEVALEPSKDE

LTETNLEQVVDVSIPPATGTAASTAADSWDPRQNPTSTSATPLSASYQQHQGQAVPASGY

AAAAIKATAERTARVPSYQRRVLDQEEAVRMPGNREVDRAAVQFGAFNLNDGEEDLDGDR

EEPETRAQPPAESPVAHPRASLPPANQPAAVPDAFAQKQPGAPGAPGAPGAPSGPAASNA

QFGRYGQAAPQEAVAKPQDPFSQQAPTSQPPFDNFSTPASQPPAQQPGGAFSSAPSEYSS

YYTAGQHDRNPYNNYYGQQFGQQGGQGQPDNASSQQRPFGGYTATQQNDNLSQYPQSQPR

IGSGAADAAQNSGSNTPNPTTQAQQPQQQQQAGPGSQPQSHGQQYPGYNHPYYSNPYYHQ

YYSGYGQGSFGPYGGKGGMYGQPYGVSPNTPYDHASSPGTFNQSSGHRDSGLGSGLGEYG

RGSAAQSGSQPGLGGSSSFGSMHDSFARGSSAFQSQTPSFGGQNQPGSNASTDELKPFPD

SKAPSGPSPSLGGARPGSAANNGPGAGLPPPQGSQMGGGYGGYPGHLQSHGLHNNSGYGM

GAGAGGSQHGNTPYGSYGQGFGSGGYYGGGQQQRGGWGGNYH

>SoG_02949.T1

MPILGVADAGHIGNLRLAVQNVADVALNVTRGARHENDLGIGASLTRSSSSRSHELSHSF

QIPASARRSSFTLNAAPLANLSADIRSSVFDLEDMALFHHWSFTTCLTICFSRNTLKIWQ

RVFPEIGFKYPFVARALLSVTALHLANETPDAGTREQYINRAVTHHDVGLSSFRNAVKDI

TTENSEALFVWSILNLIYVFGMLDHGPLAPSTADKDQILGAEWIPMMHGIRAVLLPARGH

LSGGRMGTFFSLGSWAKLDYDESDAWDLELQRTREIWRESTDKDIYEEALKMLSRCQHFM

ARFKHMSEEDLTQWGYNQAWSAPFIFIEVAPEAFFKLLRQRQPPALVLYAHFGALLSSTR

SHWFIGDLGKKMVQVVDEILGPYWRPYLTWPLEYISRGLIF

>SoG_02959.T1

MQSSRAKGDAHGRKAKLANSYQELLEMFRADLVLPTMSSEFASKDLKSVGNYTLGRLIGK

GSFGKVYLASHKLTNGSKVCLNITLRSTYLQYGGTDWAAQVVLKSANKDDSNLAREIHHH

RQFVHPHIARLYEVIVTETLVWLVLEYCPGDELYNHLLEHGPLPVHKVQKIFTQLVGAVS

YVHMQSCVHRDLKLENILLDRHENVKLVDFGFTREYEGRANHLQTFCGTICYSAPEMLKG

EKYAGEKVDVWSLGIILYALLCGELPFDDDDDNVTRTRIISEEPTYPDHLAPDAVSLIKS

LLSKRPFPRPSLPDILSHPFLAEHAVAQQAILNKGVTPPFGTALETDCLQRMRSAGVDID

AVIESVLAQKVDALAGWWTLLLEKERRKMQRRERKRRERDSESKSLRRLSAASSRLERMG

PMLQDVDEDGGLTSQFIRLGEMQTSRTRGRSERRSAHYYGDLGIPDLPQVSEVGSGQNTP

DDIPPTPVDKDSIRSVSTSRHRRPIPPPKEGVIRSARSRGSTLHLVTTSEALGGTSSNSS

QANSQPKARKKPSQAIIAHWKNWTHWIFENTTRRKKGHERRTSRSVPDLHNKDGNGIDKD

GKVSPRPQTSKYPTTGSPGAPPTAALPKGVVANGHVTRGQATPSMQGARGTRIPSGQPPG

PPRISTAYKRQSLSPTPLTPRSTMRRSSAGLRGRKSTSSSLSSVRSIHHHHHSHSKASST

SSTGSVSTTKTPLHRGHSPHHSSVKVLPATPSTSSPFPSNIRLVRASPAPLALWNEGMPG

SDGAPPGSPNPFASGVQFAKRKKNLFKGPTLNFGTSGGARAANSSSHSRNTSASGLGRRS

GEITIQEEDEDYVGEDAEEIEEVEAFNPIVRRPGEIVEEEIIEDGEATPTRLRAVDPVPQ

DHDKRRDDGRIEEQEQPKEEQLKSGQAISDDNALPRDQPASVEARA

>SoG_02968.T1

MPPVDNPSGDIDDQPTVVFFHPDLGIGGAERLVVDAAVGLKSRGYRVVIFTNHCDPSHCF

DECRDGTLDVRVRGSWLVPPSILSRLTILCAILRHLHLLLSIYFSSELSNLRPAAFFVDQ

LSAGLPLLQYLNPNIPILFYCHFPDLLLARGRETSALKRAYRIPFDWLEEWSMGFSQAIA

VNSEFTKRVVEKTWPGLTGRVSTKVIYPCVDTDDAQTSDMPLPDELKNQKIILSINRFER

KKDVGLAIKAFAALPEGKRKDARLVIAGGYDSRIAENVEYHAELQDLATSLSLTHHTTSS

LQTPPPPTPILFLLSVPSQLKQSLLSSARLLIYTPSNEHFGIVPLEAMLSSTPVLAANTG

GPVETVLDGETGWLRSPDDTPAWTAVVSRALQLSDAQIGDMGRKGRDRVKRMFGREKMAE

RLEATVDEIVELKRPPPVLNAVLNFLGITLVFVLGLGTANVMSRLHGKA

>SoG_02969.T1

MNPSSNNVPDQGLLQNRGDSRSPAPLQTQLPPAPSIPSKTAQSTPMSSPGLFSPTGIRHN

AAAAHISLSESTTPSGFGSLHPLQNHRVRETHKANIDSDNVTGRKSINQYEVIEEIGRGM

HGKVKLARNQQTGENVAIKIIPRFSKKRRLGKLSGLAPQDKTKKEIAILKKIRHPNVVAL

LEVIDDPELKKIYMVLEHVELGEIVWRKKGLPHICIFERRRIEREMRGEPPSADEERYMQ

LLERRQRIKQMKRERMAQHYPDASNYWSVEHGAADEASSYGQHSRIASQDDFAIEESMPG

SRSSSRAPTRSLSNRSFNEAPFELEEDVDWDDDMETPGPLRSNHTSSAGLEAGSFDIPGE

GEYRGRSPSMADSIISHMSSIDFPHQSHDAFVDDFSYVPCFTFQQARSTFRDTVLGLEYL

HYQGVVHRDIKPANLLWTKEHRVKISDFGVSYFGRPIRDGEADETVSESEAQDFDDDLEL

AKTVGTPAFFAPELCYTDVNQEQPKISEQIDVWSLGVTLYCLIYARIPFLADDEYQMFKK

IATEEVYIPTRRLKPVDPSTSPVVVSLYKRQNSDPYRDDNDLEYEDVDHDLIDLIQQMLI

KNPEKRIRLRDIKRHPWVTSDISNIIGWLDDSDPARPTEGRKIQVDEKDMSYAVVPIAIL

ERARTMTRKVLNKVMHPLGDRGDSRSRHRASSSAASSAGDSMANNVPTTPGQRNERRRSI

LPDDYFATAIQDSLMQAETSTAPSQSDGATTPNQLYDPLATVLRASEIPREHARSGSAAA

AFDFSAIPSRTMSSGHRHGYSVGRLPRHGQHLWMPSGRHTTPTTPFGEVRAQSPAIPANP

AQAVPQMRDVESSQEELSRSKSVDRGLFASTDKRAQAQVSLNKAAAPGSIMTPIQSSNMA

GKSSQARATVLASPPHLVQQRSDNAAAYAYHEPPSDSSIHANFRDTVEHRPQTAQKSSHE

HTLHGLGSFAYPEHFAQVQADQHRRHQIELAHAQKNSSPLRDGSSTMTTSALWASQQRLP

AQAEISSTTVQSSSTESMGAVGTPLTSPSEITSPISVRPDGKNSNESMAAFQSDPSLPAL

LSGASSVSADIEGELLGRPGIVEGQPSMLLETTDSLTPPAMMKEPSTFPIQEVFANPPAM

DSGSLAVHLANAHRPHSPSSRSHTPPAECHDGDDEDDSGSDDGLLIMAKSKKKPISPYSP

AGSPSPSPFEPRRRDTNISIMSTETAKKVSID

>SoG_02971.T1

MTTDTANEAETRANVGKQAETLPARKQIRFVNNQGQPPSKRRRINAACLTCRRRKTRCAG

ERPLCSTCTKNGHECLGYPEERRDGPDGSEPKSAQDDSNETDGDEQELAEKAHHTRPSQS

EPVGRVNPVTAKTKEEAVPSTTDMSAADATAATAQPFIPAHQRAVNSISDSDPLSPTLHR

NSHSHRVPYFRYFGPTAIVPGFKQMVVSVRDRHHSGSHSAISPLSTPSGVQRASTAGSDV

FMEELPVYDVNDPTPVHPIIISLVNTFFTHLGCNYPFLKQRKFLQMIKEKTVEPILVDSV

CAIAARFSDLPALTGGNEKMPRTDRGAVFAQRAKAATVDTFPAPSVGAVQACLLMAYEGF

GANQDSALWMYLGLAIRMAIDLGLQKRVGIRYQGDQDPWFTRQRSRQNGEDSSPDLKKGE

ADALTLEEQKEIEQERIDTFWAVFFLDRVISSGTGRPVTLREDDFELSYPQSHMETLTKW

PAPFSALVEIIHLYGRVSDVLNNIRDIKDLTQEKWDRLRKLEHQLTRVYKNWDPRLQFNV

NNFKAYLGQGQGTTFILLHFWFHALFIILHQPTLMTPFGDLRSESQLLSDSRELSMSSAK

TICDILAFADLIDPNSFVGNPFTSQPIYIAACAFLMESSANASAAPSREGSQPRTEVPAT

KSDGAGKSSTASRHSRHSLLASAANQNYQRCYSSLQHLHTYWGGIKYILTALDQKSKGIW

DVETFTTEEYESTDIRRTRELGSQFPFEGHATSPKMTGPPIAWSLAGTANSPNSSLTLMY

QNVSGINAASQSQGLAGPMSMPSPGNMVYDPIRQNSSEGSMLPPSVPQPNIFNACAERLT

TQPQVLGFDGSGNRNGTRPDDANNGGLGKIYMPPNFTPGGQPSASFDAFSVSPASGSLPD

GGGGHHLGGGIYGQAAFPVSAWGMTGMDAITFDSQDIDIGALGLQGQELMGGWLDYIPGD

MRGLYGEDHMGHQGQ

>SoG_02976.T1

MLLSSRGKPYVCWRCVTRRTSAFTTLPASQHRRLNQTLAGPSKLGADTGPLRHHNASANS

DNHRAEANTSSKKPKSSIRDELRAWGSSDQAPEVTTRPLDVSLSARVNNRLTRTQGDSAS

LDEGGSEHVGDVFGFSQDEEVGIAGTSDRQVGDLVELATAASRWGTLAIYLGFFSGRHHF

YSHNSKWLMSAGYPAVFSVAKFASDEELASVLRSIPMGLSVEEYQHMQKQNQGPSRSDGA

ELLKKMRDFRLASELIHQNNIVTLDSARELLAHDTEVRHLSLFEITDILLPKARNDDGSF

PASALYSVLSVLGREDIAFRALSPSRDCHRQDYMFEIVSKATVQSIKRAVAAVRQYTLLN

TNTKGIMHEKHAKANALNAFVSKACAVVTQSRMNRGWTRHGTLEPCQGFELDQPEWKHSN

MDIINFLQWWASFDLFTTSSHYHSYGSTILRATGLYEDAPLDQRTAWTFLQEIGHIISSE

IPSRFRIRLPGATFGKGGGITRKTLSDAAILDSQQADIAADRRIEISQAVLCIDHPDTVI

IDDGISLERTDKKDEFWIHVHVADPASRIKSKSELAGFLELVPENIYLPGHFEAMLPSQL

GKNARDPKSRSLLQELSLHQGSPTLTFSAKVNAAGDMLDYDIKPGKLTKVSYLDPGEVSR

FCDEPKGMTAPDIKLSVGEAYGEETYAEPRSMVTPEQLDEPIQDDLRTLYKLSRALKVKR

LAKGAWPIFPPRPSVSVQFDPKTRAAADNASISDNQTPSIDVHSDDTPPHKTIQIPADPY

INVEYQTSEGCSVVSNTMVLAGQVAARWCSDRGIPLPFRRDTASAMNYDKALTYVKEQVY

PLLLKGIDPGREHRAMLSALIGGTELSTSPGPYFILGIDEYAKVTSPLRRFGDLLAHWQI

HAAIGHEFRTGEKVNPSTVNDIVPYPEESLAHTLILLQMREKMIRAMARGSRDWILIALV

RAWRANDGSIPDRMRFTVTTRGTSGLLGQLDYFDLPASMDAVQIGGKVLIQDISVGDQLE

VELADINVYDGVITVKALEYLGRTKNSPPMPSFDARPLLPAGAQEQGFGIFPTSHQTSSP

STAS

>SoG_02980.T1

MSSPYGFQGSTGPSPSATSSSHTDASSASPDASNNGALFATGDSSRSQPPREQSAVPAAC

LGCRSKHLKCDGKNPCSRCVSSESQCLYVASRRGYKGPRKGAASNPNKRQATSPPEQDGG

STAGCPMGGGFVVTTLDGGSNNANLIQSYKTQPPGTTQQVGNIRQTTTEVPLYERCFDAF

YHFFHAAHPFVLPKNFLLPLLKEVAMEPLLAAMRWAGSLYLDKVDAQTKARLLEEAMHLL

KHSPSRDGFLVQALMILIVALDGQCKQEHARELLERAEQLALEIHLNTRSFATTYGRGLP

VLEESWRRTWWDLFIIDGMIAGVHQVTNFFLYDIPTDVALPCEELEYLSGVIPTPKTLAD

LEDREFSGVDVEYSSFAYRILCGRNLGRFMRLSPIQGPNDENLNRIETDLTNWRLHLPKS

KKDALGADGRPDEMMFQAFMMLNATSLLLHQQHSQLDSSPARTVTSCAPYRPIPSGEEAY

NIHTHHTLIAANEISKLITHRTPLLTHTHFFTCVITLSSIVHLSRWAQHFYLPSTGNSSN

ALLLPAAYQQTLSQGVDDDLRGLIRLNIGALAELSTVWGAARTAKGQVQGVAQEIYRGKK

QRQQSPNYWYGFSQEEMVNSIAADDVIIGDIQSLQGLSGLPELLG

>SoG_02989.T1

MGCGMSTEEKEGKARNEEIENQLKRDKMMQRNEIKMLLLGAGESGKSTILKQMKLIHEGG

YSRDERESFKEIIFSNTVQSMRVILEAMESLELPLEDQRMEYHVQTIFMQPAQIEGEVLP

PEVGSAIEALWKDRGVQECFKRSREYQLNDSARYYFDNIARIAAPDYMPNDQDVLRSRVK

TTGITETTFIIGDLTYRMFDVGGQRSERKKWIHCFENVTTILFLVAISEYDQLLFEDETV

NRMQEALTLFDSICNSRWFIKTSIILFLNKIDRFKEKLPTSPMKNYFPDYEGGDDYAAAC

DYILNRFVSLNQHETKQIYTHFTCATDTTQIRFVMAAVNDIIIQENLRLCGLI

>SoG_02998.T1

MSEPTEQQPPAEQPAGEQGAAPTSKKGAKKAEAKAKKEALKAARAAELAAAAAKVTLEDD

PAQDNYGAKKTVNGLFSKDAEDVEIRSLNESHIDKTVIVRAWLQNSRVQSAKMGFVELRK

GGNWNIQGVVMASEQEPLVSRRMVKWITSINPESFIAVEAKVKKPLEPVKSCRVSGLELH

ITKCYVLSSAPAMLGMTMAAASAPIVNFSDEKAAGEETEKAPEAVAESTIPAASMLTHLD

NIAMHKRAPIQQAIMDIRIQVKRLFRSYLEERGFKEFEPPALIGAASEGGSNVFRLGYFG

EEAFLAQSPQFYKQFEIAGGRERVFSISPVFRAENSNTPRHMTEFTGLDLEMEIKDSYQE

VLLILEGVLLSIFRGLQEKCADEIETVRSVYHSEPLLLPEPGKEVRLTFAEAQKLLREEG

PAEFANVSDDEDMSTAQEKALGEVVRKKFNTDFYVIDKFPETARPFYAKIDDESPKEGVR

VTNAYDFFIRGQEVLSGGQRIHDPVELEERIRAKGVDPNSGGIKEYLAVFKQVGVPPHGG

GGIGLDRVVAWFLALPSVHLAAFYPRTPKRLMP

>SoG_03002.T1

MATSSAKRRKLAHAAAPVEDHESSSRQAQKSFFKNASNWNLEQDYESRPRKGKKREKESN

RLPIKTSDGRIEQLQGLQEDAESVESDTEWLEGKDDAVGSEDEMQDGASDEKEGEEEEPE

LPVQQQIVQAQEELAKLAMALNENPEEHVGAFKSLAKIGAETKVPQIQMLALVTQMTVYK

DVIPGYRIRPAEDMAKEQLTKEVRQLRQYEQALISGYQNYLKELGRCAKLPSPRSSQGPN

VALVAVTCACTLITSVPHFNFRDILLRILVSRLARRHIDEAGKKALEALETLCREDEEGR

PSMEAVALLSKMMKAKDYNVHESVLNLFLSLRLLSEFAGKASHESVDRPEQKQKKKQREF

RTKRERKALKEQKALDRDMANADAMASHEERERMQSETLKLVFATYFRVLKLRVPHLMGA

VLEGLAKYAHLINQDFFGDLLEALKDLIRYSDEEAERDFEADKDEDDEQEEEEESGEDGS

DDDEDDEDGNNVELTRNLSREALLCIVTAFALLAGQDAHNSRSALHLDLSHFITHLFQTL

LPLSLNPNLELTRLPSASSSSTSKINVHTTTVLLLRSLTGVLLPPYNIRSVPPLRLAAFT

KQLMTVALQLPEKSCQGVLALLGDVAHTHGRKVSSLWHTEERKGDGRFNPVGDSVEGTNP

FAATVWEGELLRKHFSPKVRESAKLLEKSMAGTGGNN

>SoG_03006.T1

MDRPSTPLGARPPDYSLPSYDDEHDTPNGTNSAAVRLLTSVDDYDSRPYVPPRSPCLPNE

ASSIPSSSFSLVPPQQRSTAVPRGPSTTARKPVPQKLPVPLAPLLPSENSEEKRRETKSS

ASFTTAGQHLRTLKQKEDLVSQRASSPTQRGSLLHEDQSNPRRSSCLTRPASVKLQKRNK

PKQVVISEFVESLPFIPEGPSDRLAQRSSSAAPHPHHSLSSSSPPPPSPPRPVHGNPPKK

NSVTMNPPKYTRRSPRKLPSPPRQTRQSYQPSVNSSHSRPGSIMGDVPTMPPPDSTYVPF

AGRDTAGSPQRPWTPSSRMSDYSRPPPSNVSYEPADINGSPRPGTPSSRYGGSPRRPLPP

APLFSNPGRNSQAFAEDATIDIPLDEPNGRHGRDNDDVFGPESDLSEARPLPVDRNSYMS

ESQVTLNQDPEDDDAEYYDEKTAHYGPAPDGAQERRGVRAPQTSTREVQLINGELILECK

IPTILYSFLPRRDEVEFTHMRYTAVTCDPDDFVPKGYKLRQQIGRTTRETELFICVTMYN

EDEIGFTRTMHAVMKNIAHFCSRSRSRTWGEAGWQKIVVCIISDGREKIHPRTLDALAAM

GVYQHGIAKNYVNNRAVQAHVYEYTTQVSLDSDLKFKGAEKGIVPCQMIFCLKEKNQRKL

NSHRWFFNAFGKALNPNVCILLDVGTRPGTNSLYHLWKAFDTDSNVAGACGEIIAMKGKF

GTNLLNPLVASQNFEYKMSNILDKPLESVFGYITVLPGALSAYRYHALQNDETGHGPLSQ

YFKGETLHGQHADVFTANMYLAEDRILCWELVAKRNERWVLKYVKGCKGETDVPDAVPEF

ISQRRRWLNGAFFAAVYSLVNCRQIMTTDHTLARKILLSIEFVYQFVQVLFTYFSLANFY

LTFYFIAGGLADKRVDPFGHGIANVIFVILRFTCILLISSQFILSLGNRPQGAKKLYFAS

MIIYAIIMLYTTFACVYIIVSQLTAKDGEKVEIGDNVFTNLIVSTVSTYGLYLFMSLLYL

EPWHMITSFVQYFLLLPSYICTLQVYAFCNTHDVTWGTKGDNVMKTDLGNAVGKGQTVEL

EMPSEQLDIDSGYDEALRNLRDRVEVPSSPPSESQMQEDYYKSVRTYMVLIWMITNGILA

MGVSEAYGSSGIGDNFYLRFILWSVAGLALFRAIGSTTFAVMTAVSFVVDGRMKMSLKAP

KWMGGVGGKISDKVSSVGSSMRS

>SoG_03013.T1

MVSSSLLNLDGRVSRPPVDCVSPFQASSASTATPSRHAFDQRVDAIKAPKSDINALILDY

LTMEGYPNAAANFSKEANLPPQQQTSSIMARQEIQNLIHSGKIEIAIEALNEYDPEILDD

DKALHFSLLRLQLVELIRSCKAVDGDISPALKFATEQLGPRAPTNPQFLEDLERTMALLL

FPNDSLEPHIAALLQPNLRREAADSVNKAILERQSKRREAAIRSLVKMRAWAENTAREKG

IALPAHLDIGLHGDETDNGNGPSSSNGHEPMVTT

>SoG_03030.T1

MPTEESKPIEPAVAEADAKLKANQAEAESEGESDDEPSTAADGSAAAGSSSSASKKKKKS

KRKKAKELLTGSSGDPEQDMKKAIGGLTPQQLRDLMALNPALAQEVAQVAGTSNPSAEQA

ADLLKRLNLQEIMTGLAAGGKNVKDMASYKFWQTQPVPKFGEEDKNKEDGPLKIQTVDDI

SKEPAALVAGFEWVTVDLKSDEEVKEVYELLNGHYVEDDDAHFRFNYSPSILRWAMMAPG

WQQRYHIGVRASQSRKLVAFISAIPVHLRVRDKVVLCSEVNFLCVHKKLRGKRLAPVLIK

EVTRISNLDKVWQGLYTAGVVLPRPVSTCRYYHRSINWQKLYECGFSPLPPNSKPAYQVR

KYALPDSTSTRGLREMKEKDVDAVLGLLKRYLARFDMAPEFTRDEAVHWFVPKIEPGGEQ

VVWTYVVEDNNKNITDFFSFYCVESSVIGNAKHDVVRVAYLFYYATEAGLTEPFDQAALK

TRLNALINDALILAKRAKFDVFNALTLMDNALFLEQQKFGAGDGQLHYYLFNYRARPIGG

GITESMSLDEKNRSGVGLIML

>SoG_03034.T1

MTVASDLTQRASSRASSHAPVALEDRFEVIKEIGDGSFGSVVLARVRTAGASVARRGTVI

AIKSMKKTFESFQPCLELREVVFLRTIPNHAHLVPALDIFLDPYTKKLHIAMEYMEGNLY

QLMKARDHKCLDNASVKSILFQIMQGLEHIHSHHFFHRDIKPENILVSTSSHQDSSNSFR

RYSALVTPPSTPPTYSVKLADFGLARETHSKLPYTTYVSTRWYRAPEVLLRAGEYSAPVD

IWAVGAMAVEVATLKPLFPGGNEVDQVWRVCEIMGSPGNWYNKAGIRVGGGDWREGTRLA

GKLGFSFPKMAPHAMETILQTPQWPPALANFVTWCLMWDPKNRPTSTQALAHDYFFDAVD

PLRPKSSASKILGRKQSDLGRNSKDSSAVTPPTSKQSWFRKSLIGRVEVAEPIIPAVAPQ

LKDPLARPTPIQPTTITEITAQIQPQKQRAVNAKRSTWTNGASNMAPMPILPSIRPISPL

SDAVTARAHEAQANLQPEKSTKIGRQLSVASSTNNYTEMHRQQAERALNGGLASPPNGQK

EGFFSHLRKRARRLSGRHQTPVSPAYDDLEAGAGCGPWNSNRSSMIVDQQTQVPIPKSEV

YESLDRALRDVQNSLDSRQPMASNSGSNTTLKRHHSLPGQQARSVDNLIGAVRTGPISSR

TRRAQAAHGVQQYEAPDEEDELLDEVLSSTHRAMKRMDQNSKPLRQSASNLGLSNPYPTP

SPSASGNILFSDANEALAPRPLDLSKKTEGQPKWPTPPYEESEWAASASASIWAAGQRF

>SoG_03041.T1

MAVLWRAIRLNASVVATQQALASITRSRSLLRPVATYRNYVATSPRRLPQAGQSPPKPGA

YDLQSSLDSAARANPQTLTEKIVQLHAVGLSPGRRVRSGDYVILRFMEMGALSVSDNRQV

VMALDHDVQNESEQNLRKYRLIEEFANKHGIDFLLTQLTWGWKTDPAKTGIGHQIMIEEG

YAWPGTLTVASDSHSTIYGGIGCLGTAIVRSDAASILATSTVFWQVPPIAKVTLTGVLPP

GVTGKDVIIALCSFLKSDVLNMCVEFTGSEETLASISVSERLTISNMACEWGALSGLFPV

DNTLVSWYRARASAAAITDSPTKSRINHERVDKLLETSPVADPGARYAKEFYLNLSTLAP

FVAGPNSVNVANPVTKLEAENIAVDKAYLLSCTNGRSTDFAAAARVFREAGTDGKPAKIH

PRVKLYLAPASVAEQRMSEEAGDWQVLVQSGAELLPAGCATCIGLGRGLLEEGEVGISAS

NRSYKGRMGSPKALCYLASPEVVAASAIKGQIASPGWYLKPDGVEKVIVGEGTGDFVADK

ARSTEDAFEKLIGEMESMVSAAEGAAEATASPSASEDEVLTDVLPGFPEKIEGEIVFCDN

DNINTDGIYPGRYTYQDGMTAEQMAEVCMENYDTNFRNVIRPNDILVAGYTFGTGSSREQ

AATSILANKIPLVVAGSFSSIFGRNSINNALLGLEMPKLVERLREVYKDEAEKPLTRRTG

WKLLWDVRRSQVTITEQDGTSWVHKVGEMPPNVQEIIAKGGIVKWVQSTLQAA

>SoG_03043.T1

MIKEDRSVGSASDTSAENREAQVKQLAKQFSRQSVHSTTGQNPFEATPGSALDPNGENFN

ARAWCKAMLHMQNEDVRCHPARSLGVSFSDLGAYGFDSDTDYQKSVGNVWLELIGTVSEA

LSIGRKKQKVQILQNLEGLVEAGEMLVVLGPPGSGCSTFLKTITGETHGFHVDKNSSLNF

QGKSLPAVAPI

>SoG_03044.T1

MSAKQMATEFRGEAIYTAEVDVHFPKLTVGKTLYFAARARAPRHIPGGATVDQYATHMRD

VIMAMFGISHTKETIVGNDFVRGVSGGERKRVSIAEACLSNAPLQCWDNSTRGLDSANAV

EFCKTLRMQTDINNATACVSLYQAPQAAYEYFDKVLVLYKGREIYFGPIHSAKQYFLDMG

FVCPDRQTDADFLTSMTSHIERIVRPGSNAPRTPDEFAARWRASPQRQQLLQDIEKYNAK

YALGGEYLDKFKDSRRAQQAKVQRVSSPYTLSYVQQIQLCLWRGWQRLKADPSVTISSLF

GNSVMALVISSIFYNLDSDTSTFFQRGALLFFAVLMNALGCGLEMLTLYAQRGIIEKHSR

YALYHPSAEAFSSMIMDLPYKILNSITFNIILYFMTNLRREPGPFFFFLFTSFVLTLCMS

MFFRSLASLSRSLVQVLPFSGLLLLALSMYTGFAIPTDYQLGWAHWISYINPINYGFESL

LINEFHNRNFPCVNYVPQGPSYSGVESDNQVCSTVGSVPGQPFVNGDAYIESAYGYEASH

KWRNIGIIFAFMFALGAVYLVATDFITEKRSKGEILVFPRGHKFLEKKKMGDDIEKNGSF

GRTTVENSPDSENLPMIQRQTAIFQWKDVCFDIKIGKEERRILDHVDGWVKPGTLTALMG

VSGAGKTTLLDVLATRTTIGVISGEMLVDGQPRDESFQRKTGYAQQQDLHLSTATVREAL

EFSALLRQPAHVPRQEKLDYVTEVIKLLAMTEYADAVIGVPGEGLNVEQRKRLTIGVELA

ARPALLLFLDEPTSGLDSQTSWAILDLLDKLKKNGQAILCTIHQPSAMLFQRFDRLLFLQ

AGGRTVYFGDIGENSHILIDYFVRNGGHPCPPDGNPAEWMLDVIGAAPGSHTDINWFETW

RNSPEYAQVQAHLDHLKHERSQLAETSSATDPKRRDPASYREFAAPFWVQLWLVQCRVFQ

QIWRSPIYIYSKALLCVLSAMFVGFSLYQSPNTVQGLQNQMFGIFMLLTLFGQLIQQIMP

HFVAQRDLYEVRERPSKTYSWVAFIISNIVVELPWNTLMSVLMFFVWYYPTGMYHNAAAT

DEYALRGAQTWLMVWTFLMFSSTFAHFMIAAFDAAENAGNLGNLLFLLCLLFCGVLATPD

QLPGFWIFMYRVSPFTYLVSGLLSAGTANAFVECAKNEYLHFDPVNGTCLEYMQTYMSQA

GGYVENESATSDCSYCPLKDTNVFLEGVSSSYSEYWRNFGLMWVFVLFNIFAACMLYWLV

RVPRTKKPVTKTQ

>SoG_03071.T1

MSDLSTYTSVFRANSGYRAKRNRQPLSCTTCQRRKTRCDRQQPCYACWQRGDPDNCLFGH

AAGDDPQTGARNEHGAAASSGSRKEVQARLTKLESLVKELASSSDGNARKRLRGDRIGRD

GNVKPRDSGSDIAMSGKSLHVDRTDGAYRGATSWSSLVANIRDIQHLLDTDGEEESERAE

NLPSSRDVVAGAEPPQPVAECFLISPGKIDLDGILKALPPRQDADKLVTTYFNAKFVAIP

FIHTHQFRRQYDRFWEDPSSASYLWVSILFSIMSLGTALLRYSPHVTLSGGANAADSGNY

VILAAQCFVAGEYFRAKPLSVEALLIHVHSRNVCLDDSHPSVWIFYGLALRLAQRQGYHR

EMTKVSARATPFAAEMRRRVWFVLRSMDLLHSSQQGMPPMVDPALCDTQCPNNLTDDDFD

EDTIILPPARGTTDPTPLLAYITKAPLCSILDRVLRHALATGPRLYSDILALHAELEAWH

DSVPACLRVRPIRNTSFTDPGYTIMHRIVLETMYLKTLCILHRQYLGAESRRNPQYGTST

QSCKEAAERLISIHVELHSEIQPGGRLYDERYMVNSLTLHDFLIAAMILCVELCEGDHNH

SSPDRKRQISVLRSATDIWENRARTSQDARHATKVLKAILQKVEAPASSTLWYSASLETS

ATVPIGVQPKTGSSYSAHEWHMSLEDDKAMFDPFSTDHLILDWVGRFHILASATNMHNLI

SEDGLWRDAGTVPLEPCHALGDEALKKRGCSFGDMVIIQRLALDYGSFRAANLKPGLGCT

LRCWCRAGLPAQAATSWLSPALPPSWAPEISAVACYNVRPLKRGDRSYHSRNMPSLRRRR

LH

>SoG_03072.T1

MSAFTALNGGSPKGSEPPATFADSNNAPEDRPRSSAAQSQAGTENSAPRDGWTSQGQEGS

ALQTSPKIDADSSLKRKRSHSTEARREHLIQERTPDTATVPSHGDSRDPFDTPQRDRDQW

YGHQGREERSHHEASQSSRTSPGHAEEHTGDALRRATQNERSEYEQTSPEGEERSAGAYG

SPHTTAQRQGPILQHDPKKRKRNFSNRTKTGCMTCRGRKKKCDETKPECINCVKGGFVCA

GYPAQKGAQWQKPSEKTPTVPLESKDPSYVPPGAYGMPQQGPYASQPGKREPLPPYRGQA

LRIDPPQGRPLTTEDDRPTASTIPSASVASPDNKLSAISSYTTGNVFPTPVSANTQPPPF

SERMGKEYQRVPPLHDLARNEPETPHPGSHLPQINILHASRKNSPTQGGQQPSSNPQVAA

QLALSHPGFPARRTQKEDMLSGRSYYPFDKELCLERERCASACWRFNNLTIPSHGVSHEE

RGRLFMDILQPREAVRVSPTELSPVTNVGRVGREVAVETPFTCDYGYNITIGSHVAIGRN

CHINDVAEVRIGDNCVIGPNVNIYTATLSTDPKRRMGGQGPQTGKPVIIDQDCWIGGGAI

ILPGKTIGKGATVGAGSVVTKDVPPFTVVAGNPARVLRGIAS

>SoG_03077.T1

MADEGVAEHYQVLEELGRGSFGVVYKGIERATGEVVAIKHIDLESNDDDIQDIQAEIAVL

STCASPFVTQYKCSFLRGHKLWIVMEYLGGGSCLDLLKPANFGEVHIAIVCRELLLGIQY

LHNEGKIHRDIKAANVLLSETGKVKLADFGVAAQLTNIKSQRNTFVGTPFWMAPEVIQQD

GYNFKADIWSLGITAMELANGEPPLCHIHPMKVLFQIPKNAPPRLEGNFSKDFKDFVAQC

LTKDCDRRPSAKELLRHRFIRSAGKVEALQELIARKQMYDANQNRQKHPIYYQETLQTIS

PKDDEQEWVFDTVKSVAPPKRPTVRHRKPSSIFAADEAMRKLDLKDAPLGTSSPAPAHGT

VRRSTVRRAPSLAQVSSMHRNGSPRGSIAPKKPLQPDMSFGNSGSTMRLFRRVPSDSSTN

GQLGRPTSPDDVFQDENLPPSIATPVEPYGKEAILGRRLYNKALEPTLAELHAQTSAMQK

REALAKLSDAFAALDAVDPEGAHHLMTNLVATMSQDKKLNACFLQQAVQKTPDDGTPQGT

VLIKPSTPALSPSKLVLAPNNPHLKSHRRRQNESPALNEKDYERSMLEHKYPGREAKAGM

EHCKQLSEVLYSRWSDGLRLRWPAT

>SoG_03097.T1

MCQIIISKLQELFAALKQRSRELVTILRITALLPRSKNTPALSSTITRRLDLFIARREVQ

DAGSLADFMANLVEANHEEKLEVLAALDVKVRITKVLELLDRQVGGIKNSVKITTITSMP

VGIADRSSDNTNQRPRLPPNIGALGFFAPPGQMPGMPGGNGEPKDDQEPNELDELRKKLD

AAKLPPDAAKTADRELGRLSKMAPMNQEFQVTRNWLEILSEIPWQAMTDDRLGPETLTRA

RKQLDDDHYGLEKVKKRLIEYLAVLRLKQSNNDEIDERIRKLEAETSPTDHSTEEKNEEN

TPAESRIAKQEQEAAKLHILKSQRMVDKSPIMLLAGPPGVGKTSLAKSVATALGRKFHRI

SLGGVRDEAEIRGHRRTYVAAMPGLIVQGLRKVGVANPVFLLDEIDKIGVASVHGDPSAA

MLEVLDPEQNHSFQDHYVGMPVDLSKILFIATANSLETIPAPLLDRMETIYLPGYTTLEK

RHIAMQHLVPKQIQVNGLAEDQVAFSQDVVSKIIESYTRESGVRNLEREIGSVCRAKAVE

FAEAKDAGRLETYRPQLAVEDLEEILGIERFEEEIAETSSRPGIVTGLVAYSSGGNGSIL

FIEVAAMPGNGRVQLTGKLGDVLKESVEVALTWVKAHAFEMGLTQDANVDIMKDRSIHVH

CPSGAIPKDGPSSGIGQAIAMISLFSGKPVPSTMAMTGEISLRGKITAVGGIKEKLIGAL

RAGVKTVLLPSQNRKDVKDLPQEVKDGLEIIHVSHIWEAIRIVWPDSRWAADHQYAGIES

RL

>SoG_03132.T1

MSSTPSRPCDTCRIRKTRCVKADDQSVCVLCAFHGKSCTFFRGPPARRPRAARRAAAATA

TATATATAGNSVTTPTATTSAATPGNASQHHDAGRFERRGSLGSFSPEMVAKSMTLADGG

PGAAQQQQQQQQHNSDGNAPSPDMTFTPEDANENAALSLLTGTLGLDLDTHPEYIGQSNY

HEPALLDLYRHGMEASAQAPATPGANSGANHGNEGSGGRRASMRRTRRMDDRTMFLICPD

DETASEAQRIADCDAIEDSVRPLGKALMDLYFRIVHPSFPVLHKGVFITKHAISHRMFSP

PLLAAVYLIALDYRLYDSSMAGGVAKPAHSNGQAALEALASRTMADDLKRPKLSTLQAGL

LLLQRHDSALQPSKWIFIAQLVAVAQELGLHLDCTSWSIPEWEKGLRRRLGWALYMQDRW

GALTQGRPLLLHDDDWDLRPCAPSDFPETQADEDPDVDGSVGIEIGRENFMRQAQLATIL

SDVIANFYTVGATKTRGRLDQLGAVNAVNLARPLVLRLRDWHMSLPVELAVDNIQFRRLS

ANASLHLSFAAVSIALDRALLRILTPSSPPDLTYAVRSTAKTRVTTTIGFLKSLQPEHMG

ALWGGASAHQVVSVGSLAGLLWATTEDGEEMRWCAERMDELRWVLRTRSPRIHQKTTLLL

NSPPSLNTTIYNTMPLHHYGLPVGSHYTAMRDFYVAILKPIGYVVVMELPAMEFCGFGVA

GGNPEFWLGGGKREGGLETFKEGSDLGERIAPIHIAFKGETDQEVDEWYENAIKCGAVDN

GKPGLRPQYTKGYYGAITLVKEGKLTSVPVNRMPGSQADQVTQGRDEVYAAELQTNEGAL

LVAHAREQGPGVPRRRARYGEAVYAGRQDHVRPGVAVLRGETVNDLRAFIHVLPEGLGAG

>SoG_03134.T1

MNIKVPPNPEAPVTIQDWVQLQDGGEGLSQIKAIVEAERASKTVAWITLASTEQLDAQWR

RLQTRVQEGASLPLRGVPFAVKDNIDAAGFPTTAACPAFAGPPATTDAPVVANLKNAGAI

IVGKTNLDQFATGLVGTRSPYGAVPNSHDPTKVSGGSSSGSAVVVARGVVPFSLGTDTAG

SGRVPAGLNNIIGLKPTRGALSARGVVPACRTLDCVSIFAMTTQDADTVLRVAESYDAQD

AYSRRRAADVKLTLDDAPSIAICSQPPWFGHTQQEAAYNASLDKARGLGWKLTKAEFAPL

FSLANLLYEGPWVAERYEAVRQFVESVPAEDMDPIVRGIIMKATNFNAADAFAGVYKRQD

LTRKIEEAFGSYDLLLVPTTPTFPSIADLQREPVIENSRLGTYTNFVNFLDWTALSIPAG

YRSDELPFGITLIARTWQEPLLLKLADQWLAGSPRTLGATGVIVTHEEPSSVKESEGPHA

GRIPVVVVGAHLSGLPLNKDLVSRGAVLQAASTTSPNYRLYALPSNGPVRKPGLRRVGYG

EAGVAIQVESWLLPKPQLASFISTIPHPLGLGSVELADGTWETGFICEPIGLEGATDVSE

FGGWRAYIKHVNTQSPAVDGIPQPPSGIKKVLIANRGEIAVRIISTLKSMGIASVAIYSG

QDSTAPHVRLADQAFQLTGHSVLETYLNTTQILDIAAASRADAVIPGYGFLSENADFAEA

VEQAGLKWIGPTPKQMRDLGLKHTSREIAAAAGVPVVPGSEKLLMSLEEAIQAAEKIGFP

LMLKSTAGGGGIGITRCGDLEALREAWDSTQRLAKANFGNDGVFLEKFISEARHVEVQIV

GDGTGRVLTVGERDCSLQRRHQKLVEESPAPGLSADVRQRMASAAASLAAAVRYRNVGTV

EFIYDKQSNNFYFLEVNTRLQVEHPVTESVSGLDLVKCMIDVANGACDDLFKEGTSAAVV

AGSSIEVRLYAESPLQQFRPTSGRLLDVKFPSTARVDTWVAKGTEMSSSYDPLVAKIIAK

GSDRDEAIQNMVTALRDTRVYGIETNLEYLLQLVGAPWFRYADYNTNLLNTLPIEATAFE

VVEAGPSTTVQDYPGRKGLWHAGIPPSGPMDNYSSRLANRAVGNASDAAVLECSVQGPTL

KFHTNGVVAITGATCNVKVDEKEVTQNEPISVEKGQLLSVGVATAGSRMYVAFRGGVDVP

LVMGSRSTLELGKFGGFNGRKLQKGDIVRIGNDGAALSGLPAPVMPVPIPTESNAVWKIG

AVAGPHGAPDYFTPDGLETLFAAEWKVHYNSNRLGVRLTGPNPQWARSTGGQAGLHPSNI

HDSPYSVGSVSFTGDEAVVLTCDGPSLGGFVVWCVVVEAEMWKMGQLRPGDRVKFELMTA

EAAIKANDDELAHAIGSPAAVACNAGAVEAGGMAEIPSAIVGQFVRGSQKITIRQAGDHA

MLLVFGETDGFDLRQSLAIFAFIDEHKRNPIPGVEELSPGVRTLHVIYKMGLLPQEVLQS

LQSHDYKVPSRLPSRKIQLPFAFDDETCKSAVERYRATIRSKAPWLPSNIEFLRELNGLE

KGDVGKLMHEAEFLVLGLGDVYMGSPCAVPLDPRHRLMGTKYNPSRSFTPRNAVGFGGQY

MCIYATDSPGGYQLVGRTTSIWDEQKLEYKYGGPVVGGTAGLTAKPWKFRTFDRITFYPV

TEAELDSATDDMVRVEESETLDLDEYEAWLERESDSINKITQARQEAFSSAPYLQELLEP

APDMPNDGHGRVAIDGEVLPPGEAVRALVPGRCFRVAVKEGDEVRVGDALAWVESNKMEL

KIASPVEGRVALVRRAAGDLVDANEVMFVITMK

>SoG_03135.T1

MASFRLLYAAIAALCVAPVLGNPTTRDQDHESQGLRKKVFDWTITWEDYAPDGFTRKMFL

INGKSPGPVLEVNQGDTVVVNLHNKSPQDTSLHFHGIEMYGTPWSDGVPGVTQRPIHAGQ

SFKYEWTATQHGSHWYHAHTRGQIEDGLYGAILIHPKKAIANPFELISKDKKTIKDLERA

EQHAHPLLISDFTHLTSDDKWQKTLESKIEDTCYDSIVFNGKGRVECLDPGLVAESITDL

QKTYLALANATMTDKSCVPAEALNLIIGGGAGNPDVWPEGVFYGCVPSKGSLETISVNRK

PSKDGSQWVAIDLIGAINFITAAVSIDDHDMWVYAMDGSYIEPQKVQALVLTNGDRYSVL

VQTKKPGDYKIRVNANSAPQVITGHALLSVDGPREGSQQEDGTAHIDLVGNPTSPNVRFF

NHLAAAPFPPSPIPQKASALYVLNMLADGASYLWAMNSTRLMPDEIETLEPPVLFNPRPD

VANNVTITTRNGSWVDLVFYASNFPMPAHPIHKHGNKMYEIGRGKGDWKWQSVEDAVEEM

PGSFNLRNPPRRDAFTSPEAVEGRAWTVVRYHVTNPGAWLLHCHVSNHVVGGMMLVIQDG

VDAWPEIPEEYQLS

>SoG_03159.T1

MFPCSALLALAAQGALAWLPESRDLAAFNQTARFAELGKRFEPSLPSGVTKIRGVNLGGW

LISEPWMMGDVWKVTMGCGSAGTELDCMLQNYAGGNRENGNQAFAKHWQDWMGPDTVQSI

HDVGLNTIRIPIGYWSYSDVVNYDTEPFADPGPMLEALDAVVGKAADLGLYVIIDLHGAP

GAQQEDSFTGQTQRPAQFFNEGNFDRAERWLSWMTKRIHTNNAYRTVGMIEVLNEPVSNH

DRNSDGSSRYPAPGQVPGLLESYYPGALAAVRDAEASLSPPVSDDKKLHVQFMSEKWGSG

DPRTVSQIMNDAAVAFDDHNYIGFAVDDNGDQYKLMHSACTDSRVVDGESLIIAGEWSMT

SSVGWEGDDGKAFFKRFFTAQQQLYEKPGMAGWIFWTWKTELNDPRWTYSFATYLNLVPT

DAAGLEGNVYQDVCAGFT

>SoG_03183.T1

MHPFGGVIALLHSYLLIEVLLPGQVCDRRIDTTAQVLKHHDCCRLAPRRSGGVSIRVAAL

LRFAWAGYGAAGLGAIPRGKIVSLLRLLSTPSARAREAARRSTGAYSSTTEHTTPPPPPP

ISQTPVRQARPGTQAQCLAGQYDNAATEQHLNCLSNGTILKSLNVFNNKFPELAILHLPT

LMSAWQSPSGPSIETKVLVAAVLAVTKAQLCALNLFWANDLLPKEAYASFARSALLTLVL

EAPNIQVAQSLLIFTLYDWGTREFHRAWIYCGIAVRIVQALHSMRVAPCAPEKSWKADNN

PLAEAIETRTYWACSIMDNMIHSGTYNPPMLPLSEMERLNIARPCSAVEFAFGSDGSVPS

ALYDSREPRNPEGPHDITQSFETLVHGFDIWRQVTTFMFNDGRRAPGMCRPENCPWMPTS

PWHACRTRLETWRRNQHRNMHYPATSVAVHMTLCYGETFTCLNLLYYLCTLMLHREYFPW

LPNKDSVPRAPVDPPHLEAEAPPNWWEDSASELFGAAENISLLLNEASECGVHLMTPFAG

FCAVSAGFCNIYVAKFPKMNLGRSLRAKEMTDICTTYLKEFRQVWSIADSWMKTLKYTNL

LYERLAANESRYRGRTRYDYDILHHSVHEFRGVDRSDQQDREINEVEASIDQLPSLGAAN

EDVPSTDMLLSQLMTEISGNLDEQGVWSNWWPQMGDMDDTTPNNEMAC

>SoG_03188.T1

MTFRKRPRPRRPRLLLSVLAVASLALTPVAAVQVPFTNCLSASYQNNDPTLLQWVPLYAE

AKFDTESSSHNLEVVVWGNVTGSRERVTLPPAGSPYWDDSNETRGKIIQTPEPDVKDPKA

TTLFRKVNVATFEPWRQPVDFCREGLVNGRCPLAPVFPTANFRFPDDLPSMNLTHNFSSS

YAFTSFTNTLLLIYGDSAATNIGCVSTTVTPDLGNIAALLKFLPLVVLLLTGVAVVVAAI

YSPWGTTDVFHWTSNYGRDADLLRLVTPGFGDCLQYIQFIALTGGLSLSYPGFYQPVVSQ

VAWSALMFNESFVANAAPWKSVVDGLYVTNATYGMAELGQLVGMAESEDIWAGMMVWLCV

IIAAVSVLIQIGFLIQWVYRKILSTPEEDLRSKNIPFLVGNVVRIVFNFFLLPIVALSTY

QLVIAKDSPAFTVALSVLTLVVLVGFSSWLLFLIIRTKPKSVLFDDLPTVLQYGPLYNTY

ADEVAPFALVPVLLTFIRGIAIGAVQPSGIAQVVLLAICEVIQILTIYAFRPFSSPTSMN

VYHTIFSGVRLITTMLMVAFTPTLGVTDGSRGWIGYVILLLHGCVLVFAFFLASLITMFE

VVARMLGAGGDDARGLQRGGLSKIFGMRQLSRRVINREGPSRASQISTSAMLDAEDGSKT

GYAMPSGRVRSESAASVGGLALHNRNRTSSAMDSIDLFPGGHRHVDSGSSFGPGTPGGTS

NFSYLPSPTTARGPTVTAANAAPEASDPYYRPPRRRRETLGDSIDSEVQRGSLHIDTQRV

NQNPGPALEPPDVAGEISRNPTPAPGAQGSNAAFNFPANRPDYATREVDFYYGVRGPALN

SEGPGRKLGTGPADPTGPVATATGWIRTMFGGKSKEKGKGFEVVRSARMPPAMMARNGGF

GDETPPEGIPVAMGVLRNGPIDSDDDEPTTKASPKHRQGDLLTDLGEPGRGDEEDAESPI

TENLPRGFGHDRKGPIPPHLDLPEIPRKSSKRHSGIIDPLSPSLPPVETTGRLSVDFGEP

YTTGSSISSQHGGRMTRLPFERSGSHETASSGDVLGDLTAADIPPSVNDRPASFGRVSQH

GINRVESGMVDVDLLGSSAEVIKGSGDPRTRTR

>SoG_03195.T1

MTPFSALLLFVAILACEAAKFRMARADFPQLVVQRQNGTSNLAYSPPVYPSPWMDPQASG

WEEAYVKARDFVSQLTLLEKVNLTTGVGWMGERCVGNVGSIPRMGLRGLCMQDGPLGIRF

KDYATAFPVGMTAAASWSRNLWRDRGKRLGRTHYQSGVDVTLGPAAGPLGRNPTGGRNWE

GFSPDPYLSGIAFADVVTGIQSEGVVATAKHWLANEQERFRQAGEARGYGFNISESMSSN

MDDKTLHEVYAWPFQNAVHAGVGAVMCSYQQTNNSYGCQNSKLMNGILKDEFGFQGFVMS

DWQAQHAGVSTAAAGLDMTMPGDVTFNSGTSFWGGNLTLAVINGTVPAWRIDDMAMRIMA

AYFKVGRTVENQPEINFSSWTQDTIGPVHMAAGENIEQINFHVDVREDHAHHVRESAAKG

TVILKNKGALPLAKPKFIAVIGEDAGPNSRGPNGCPDRGCADGTFAMGWGSGSVEFPYLI

TPDSALQAQAVKDGTRYESILDNYDWEKISQLVRRPNATAVVFANAGGGEGYINVDGNEG

DRKNMTLWRSGNELIKNVSAINPNTIVVLHTVGPVEIDEWYDNPNVTAIVWAGAPGQESG

NSLVDILYGKRSPGRSVFTWGRALQDYGVDVLHEANNGGGAPQQYFEEGAFIDYRHFDRD

FPEGSAKAPIYEFGHGLSWSTFEFSNLQVEKRDVRPYKPTTGMTIAAPKFGNFSTNLADY

TFPANVRYIYQFIYPWLNTSSSGAEASTDPHYGQTADQFLPPGALDGSPQPRHPASGSSG

GNRQLWDIVYTVTATITNTGSVMDDAIPQLYLSHGGEGEPVRVLRGFERVERINPGESRT

VRMELTRRDISNWDTASQNWVVTPHAKTIWVGSSSRNLPLSATLP

>SoG_03218.T1

MELFADVVPKTAENFRQFCTGESKNAAGRPQGYKGSKFHRIIPKFMCQGGDFLNGDGTGS

TTIWGHKSFADENFNLKHDRPGLLSMANAGPNSNGSQFFITTAATPFLDGKHVVFGQVVD

GMEEVVRKMENTKTGYRGKDMPNLDVVIAQCGEM

>SoG_03228.T1

MEDDDSIIPYVRPRIANACDGCKARKVKCDGKQPCSYCARRKRSHTCTYSPQKRRKGGSS

RGAPTPASRSQSDSLMPDASSSTHENAGSPLARGGHGAERDRRLSEAASTEDDTEVPREA

RLVCDAQGKLIFIGDCAPLSFFQSVRQLVTTRVEPNAFAPGSSRFSVLEVAAPSQQHSRP

ANYRTPAVWANTVSDAVLRYLSITAGIVDLFENARLIDDVQIWASLDRKPQGPTSIINYL

VLSIGLRTVDDARAEEYFEYARDRALLDLSMDLSVATVQAFTLLTMYMLCACQINGAFLY

FGLAVRAAYSIGLHRSEVNARLGSGIHVQRDRLWKSLRILDLCLSTSMGRPPAISDVDCT

VPYKADEDSGAEPLDLLNASVQILLVIEQIVEQIYSRRKISLQLTEGLSSQLREWAVTWL

ERLKQVETDNNWNTNYNGACQIMATYYYGVMLVSRPFLMYELYRNLDGPHATGRASPTTG

KSSLADACIDSATLMVEAIQDGISRNAFTGASRTNSSMPLLVPWLFAASLVLGVGLMGGF

GRTLEKRCQQSIEALDHFAQHNAHAEQYSLISQSLLSVSLQHLERKEMMEQVQRKERSRQ

LFGLPTLGGSPAASPGTPRAAPQMGTPAAAAASRTREVEDVPAARDPMQNWSQPPNASPS

FAQIDPAFFGMMENHGMQPPMDGGYWDGAAGSNVDEASTLNLFALIDSGGGIDLAHHL

>SoG_03233.T1

MFSSPLRAAARPCRLTRPHLGASTNVKLRRPRHALNSCHPATSTTAQRRYASDNSAPWRP

VSVLDEWVAKEARPISLRQLMVFGRSLTEQRLISSANYVRTELPTRLAHRIRDMQQLPYV

VVTNSHINDVYNLYYDAFDTFRKVKEIKNLEDNDRLCDIINSNLKKHLTVIPKLAMGIHE

CEGLIDRAEVDKFMNVILISVRPARNQVGRAHV

>SoG_03235.T1

MATPSAIKIQEPNAAARNDAKPAMQDPNAGTFSLDPEDALTPDPGTEDMYRVENNKFAFS

PGHLSKLLNPKSINAFYAMGGIDGLEKGLRTDRVAGLNSDEQELDGAVSFDEVAPKGTPR

YGKAGDTIPESNTEAAVYIPPPTDPNPTGAFAERRRVFRDNRLPPKKEKTLLQIAWQTYN

DKVLILLTIAAAISLALGLYQTFGVKHEEGEPSVEWVEGVAILVAICIVVIVGTVNDWHM

QRQFTRLNKKSNDKTVKVIRSGKSQEISISDVMVGDLMHLETGDVIPVDGIFIQGNAVKC

DESSATGESDLLRKTPAAEVSEQLRSPEGEKIHKLDPFIISGSKVQEGNGTFIVTAVGVN

SSYGRITMSLRTEQEDTPLQKKLNSLADWIAKLGSAAALLLFIVLFIKFCAQLPGSPDSP

SEKGQRFMQIFIVSVTVVVVAVPEGLPLAVTLALSFATVKMLRDNNLVRVLKACETMGNA

TTVCSDKTGTLTQNKMTVVAMTLGKTSSFGGTDKPLNQSEKIPQPASDIRNVTVAEFAKE

LGPGVQDLITQSNILNSTAFEGTQEGVNTFIGSKTEVALLTFCRDHLGAGAVQEIRSAAQ

VIQVIPFDSKYKFSAIVVKLASNKFRVYVKGASEILLYKCSQVLGKSSADGLTTEPLSDT

DKNMFTGVISSYAGQTLRTIGSSFRDFEAWPPPGAESNENPRAAHINSIHQEMTLIAIYG

IKDPLRPSVIDAIKDCRHAGVVVRMVTGDNIQTGSAIAAECGIYTPETGGIAMEGPEFRR

LPKAELEDKVRNLQVLARSSPEDKRILVRTLKDLGETVAVTGDGTNDAPALKMADIGFSM

GIAGTEVAKEASSIILLDDNFASIVKGMMWGRAVNDSVKKFLQFQLTVNITAVVLTFVSA

VASDEEESVLNAVQLLWVNLIMDTFAALALATDPPTRSVLDRKPDRKSAPLITVRMAKMI

LGQAIAQLAITFVLHFAGRDLMGYSNSPEDTKSLKTLVFNTFVWLQIFNEFNNRRLDNKL

NILEGLHRNYFFIAINLIMIGGQVMIIYVGGQAFKVAPLNAKEWGLSVGLGAISIPWGAL

IRLMPDSWLSSCLPWFMARIWAKKAVEEKSLNVNDTDSLRPPLRMMSSLRGPRVQQHIGF

RARMHHIKEKTKEKMAAAHEEEAAQTSSESNATKSS

>SoG_03236.T1

MVAFSTVLAACAVIAGASASPAADLGKRQVTPNREGYHEGYFYSWWSDGASPVTYNNGPG

GSYSVQWQRGGNLVGGKGWNPGTSRSISYSANWQPQNNGNSTSFKYLCIYGWTRNPLVEY

YVIESHGEYNPGGQAQQRGQVQHEGSTYMLYESTRHQQPSIDGTQTFQQYWAIRQNHRQR

GTVDMAVIFRAWENAGMRLGNHYYQVVATEAYNSAGQASVTVESPP

>SoG_03238.T1

MSPEEERQAALNAYRAKLIESREWEAKLKNLRLEIKDLQKEFDKTEDNIKALQSVGQIIG

EVLKQLDEERFIVKASSGPRYVVGCRSKVDKAKLKQGTRVALDMTTLTIMRMLPREVDPL

VYNMSLEDPGQVSFAGIGGLNDQIRELREVIELPLKNPELFLRVGIKPPKGVLLYGPPGT

GKTLLARAVASSLETNFLKVVSSAIVDKYIGESARLIREMFGYAKEHEPCIIFMDEIDAI

GGRRFSEGTSADREIQRTLMELLNQLDGFDYLGKTKIIMATNRPDTLDPALLRAGRLDRK

IEIPLPNEVGRLEILKIHASGVVVDGDLDFESVVKMSDGLNGADLRNVVTEAGLFAIKDY

RESINQDDFNKAVRKVAEAKKASSHTISIKYLKHS

>SoG_03239.T1

MCGIFGYINYLVEKDRKFILDTLINGLSRLEYRGYDSAGLAIDGDKKNEVLAFKEVGKVA

KLRQLVDESNVDLGKVFDSHAGIAHTRWATHGPPSTINCHPHRSDPTWEFTIVHNGIITN

YKELKTLLASKGFKFETETDTECIAKLTKYLYDAHPGIGFTELAKAVIQELEGAYGLLIK

SVHYPHEVIAARKGSPLVIGVKTQRRMKVDFVDVEYSDENAALPAEAAAHNAAIKQKGPG

FLSPQNALLGAPDKSLLHRSQSRAFMTDDGMPMPTEFFLSSDPSAIVEHTKKVMYLEDDD

IAHIHEGSLNIHRMKKADGSSNVRTIQTLELELQEIMKGKFDHFMQKEIFEQPESVVNTM

RGRLDIANKTVTLGGLRSYMPTIRRCRRIIFIACGTSYHSCMAVRGIFEELAEIPISVEL

ASDFLDRQAPVFRDDTCVFVSQSGETADSLMALRYCLERGALTVGIVNVVGSSISLLTHC

GVHVNAGPEIGVASTKAYTSQFIAMVMFALSLSEDRASKKARREEIMEGLSHVSANISKI

LELNEPIKQLCEKFRNQKSLLLLGRGSQFSTALEGALKIKEISYLHCEAVMSGELKHGVL

ALVDENLPIIMILTRDDLFKKSLNAYQQVIARGGKPIVICNPGDDEFKSSEAEKIEVPKT

VDALQGLLNVIPLQLIAYWLAVLEGLNVDFPRNLAKSVTVE

>SoG_03249.T1

MKVNISDMVTGSDVVNGGANGSAPSYVNGDGPLAHRLKMERKASSPMAPAFMVSAPGKVI

VCGEHSVVHGKAAIAAAISLRSYLHVTTLSKSKRTVSLRFPDIDLVHTWNIDDLPWAIFN

SPAKKKSYYSLVTELDPDLVDALQPHIGSVNPDAPEKVRKIQQNSAFAFLYLFLSLGWPS

FPGCLYTLRSTIPISAGLGSSASVSVCIASALLIQLRTLSGPHPDQPPEEARLQVERINR

WAFVAEMCIHGNPSGVDNTVATQGKAVVYQRTDYNKPPSVRPLWDFPELPLLLVDTKQLK

STAHEVAKVKRLKQTHPKLVGTILDAMDKVTVSAAELLADEAFDHEDEEDLSKVGELMTI

NHGLLVSLGVSHPRLERVRELVDHEGLGWTKLTGAGGGGCSITLLRPGVDHAKLQKLEDQ

LEEENYEKFETTLGGDGVGVLWPAILKNGTEEDEEGGMEIDLEKFLNAEGTEGVERLVGC

HGPGGGEREGWKFWRVESR

>SoG_03270.T1

MLASPTPAPAGPVRRTSQRQALRRPASRSMLTRSESQQALVSGGAGPGPAIETRANQSKQ

YRDDSSEDEIPVPMKLSALTKALLNDPDEPVARGPSPPRTRRQASVLNSSTNSAQSVEER

RHLRSRSNQPQDSSRMEREASPGKSREHSPVRKRVVRLSRDSSNLNHMGPISTKRRSTSV

SRGMQKDSNRPPSRPSSRTESHGEEKSELQRDVNTPSQAPLRVVRISSSSSVKRRLAARR

SSGLNPDTSGADGSALDLGQDYHTRKEQVDSAARIGSSASKSSGSRYPSSSLRHRSEENP

ALHSSMRIKRVGKTPGGFLSGPARRGKRRQSDEDGGEEMGEAEPLFSSHERGVPAGEDGG

VMSHVRNFNSGSPVSSSAAARASHRRQASQADIQLGSRQPSPRGLELSRKDGILESLDVG

FPSPKGIEMVVRTHGKDQSDARRSIRPELPSNHDQENEVPGSWRRSKPSVDLIMEKIPSR

PQQAEVPAPKPISSPERKPLAAIAKNTPSHAVPPPPPKMSVLEAATSNAGAAATTQAKQR

RNVLRVNGKVYTRLDCIGRGGSAKVYRVSAENGHMFALKRVSLENADEVVLRGFRGEIDL

LTKLNGVERVINLFDHELNSEKKVLYLLMEMGELDLGSVLKARHALDESKFDPVFVRFYW

KEMLECLQSVHHYAIVHSDLKPANFVLVRGRLKLIDFGIANAIADYTKNVHRDTQIGTPN

YMSPESLLDSNSPANNSGARMNGLPKLMKLGRPSDVWSLGCILYQMVYGAPPFGHIANAM

ARCQAIINNNHTIEFPLHAPCGTRVPNSVIKTMRGCLIRDPSYRPTVEQLLKETDAFLYP

RELPNDSLPVDREMLGRIVQDAVRKYIDGQDHPDPRKRPNREDLRKEFSDDYWHSLRKAA

VAKGVMW

>SoG_03273.T1

MSGDKMEVEKVEEKMATMEHSEQHYFKSYDHHGIHEEMLKDEVRTRSYMNAIMQNKHIFK

DKVVLDVGCGTGILSMFAAKAGAKHVIGVDMSTIIFKAREIVKKNGLADKITLIQGKMEE

IELPFPKVDIIISEWMGYFLLYESMLDTVLYARDKYLEKDGLIFPDKAIIYFAGIEDGDY

KDEKIGFWDNVYGFDYTPLKETALSEPLVDTVELKTVVTDPTSVLTLDLYTCTTADLAFT

VPFSLTVKRDDFVHALVSWFDIEFTACHKTIRFSTGPHTKYTHWKQTVFYFKDVLTVQDG

ESVQGTLMVKPNSKNRRDLDVKIDYLLEAGDANRSAKGSCEYRMC

>SoG_03326.T1

MEAPPDPQRQRQNRIAGRQQRKRTARACDVCRRQKEKCDGGVPCRRCSRIGHRCEFSGRA

AVSQATGGDAPAPAAIDFGSEPVPQVVVVPRPRSAAASASAERSPAALPQRVAYLEKIVQ

RYVGDVSFDSESLRSLAENIDRERSPPRVALATNDSSESSELVGVDKENFTIEPLDNNIA

HYSGEFSHWNFSMRIKQWIEQSVPRETHNSTHYKEYYRAEDLQSPSSVTTSLSSLPPKFV

ASFLLHSFFKHAEANYWYVEKEWLADRFDAVYENAGSLSRRDVGAVCVVFMVLAIGTQHA

YLESDSGRLPEHCSHDQADSGLFSEDTVGFMFYQQACRLLPDVITISSLESVQACLLIGI

YTLPLDAAGLSYIYLNLAVKLAIQNGMHRKYPGEGLDACIRETRNRVWWTAYTIER

>SoG_03330.T1

MAQVVGRSNNRYPLTENANRVNTAGYGHGGQSSKPRHDYIVVTSNNTPAPRNNYNIPVNH

PAAQNPAQNPAQNPAQNPAQTPAQTPAPTHDAAMDSRAMSELKPPSDSEESKRHSQASYS

SNASRSSRYKSHIGPWQLGKTLGKGSSARVRLCRHNVTNQLAAVKIVNRRMAYLVQDSSL

AALSKWDKSLPELDGEMRVPMAIEREVAILKLIEHPHIMKLYDIWENRSEIYLILEYIDQ

GDLFTYINSHGRLSEEVSLYFFRQMISAITYCHSFNICHRDLKPENILITADMQIKIADF

GMAALHQTDNHRLATACGSPHYAAPELLKNKHYKGDKADIWSMGVILYAMLSATLPFDDP

DLRVMMGKTKRGVYEMPKGLSHEAEDLIYRMLQTNPENRITLREIWRHPLVQKYSYLDDL

GENSGQPPDTRKGFQYAPVPRQSLDAQLIRQLRSMWHMFSEQQLAQKLTSNEPNDQKAFY

WLLYNYRDQQLEDFKPELAHSMSDYHHLQPGVWKKRVSTCEFSQPRLDGHGRSVSRFTVI

SNAAETEVGTVQSYDPYRGSRVLHPSQSQVSQTKVTVHRDAQSPGSYSAQASRMRSGSNA

RHRRLGSVRSSINGRPASSRGSVTSMRSARHNTPSARGPNLRHKRGVDFSHVRDRANSVG

RAQKASTRMASLALDEVPSLAPVEPLPEVPRVRESASPDLPTLTTGQTHPKAVGGPAPLA

VAPDESLALNEELRHFSYNIAKDLDEAFGSSLIEATSFGGSLTDSDGRTRETSPLSLTFD

SMSISTPPSEVSVKPWDTRPLPPLPNQRALSSHPVVSEAPVVAKAEVAVRVVPSSLQPPQ

ADRRIVSAPAYPQNSAVKPTSLPSINENAGAVFNSQDKARIVSAPPHTPPKRRAERLASA

EYLSKVEHSIRVVHSPTAYSPVKVPAPLNVRKASGTEDFGRSLHQKLAYHADSDEPAHES

SSQSSQDGGKQKKKTSWFKRVSKAESETRTREAQTTQVEQPLSPVDFDDILRPDHGQLVA

TKKKSITFSFWKGGKNKEPKMKIDETPEQSRNSRGQPAVLKKAEPSNWRDSRSSSGMRVI

EVKQNWLARLFKVKPAVSHICMTLSRKRARQEVAILLREWRKYGIRGVSIDKQRNIVFAR

VAAKNHLNMKEVEFAAEIIAVIEHGKKQPLSIVRFTQERGAASSFQRVVDTMKAVFDSRN

LLVADKNRRKMMIKTLNSHE

>SoG_03334.T1

MDLDPKYDDYDFPATAPDPQPGHPGNLTEQHIAQVHQLRMMLEAEGYTERLDTLTMLRFL

RARKFDVNASKQMFVDTEKWRKEINLDALATDWDYPEKGPLAEYYKQFYHKTDKDGRPIY

IETLGGIDLNAMYKITSAERMLNNLAVEYERVADPRLPACSRKAGHLLETCCSIIDLKGV

AITRVPQVYSYVRQASVISQNYYPERLGKMYLINAPWGFSTVWSVVKGWLDPVTVQKIHV

LGGSYASEVLKQIPAENLPVQFGGKCECPGGCENSDAGPWREAEWAKPAKRDLKKKDGEA

IENKPAEIEKPEATAGTETEAATATDEAKQPAAA

>SoG_03340.T1

MAKAQTGYLQDLLTKAPKTGIEYNRLGKSGLKVSKVILGAMSFGSKEWRDWVLEEKEALP

LLEHAWKVGINTWDTANLYSNGVSEEIIAKALATYKIPRESVVLMTKCRWATSGPGEPQM

QVWDATDNSGRNVNRLGLSRKHIFDAVQASVKRLGTYIDVLLIHRMDPDVPREEIMKALN

DVVEKGWVRYIGASSMPAWEFQALQNTAKSHGWHQFIAMQNYYNLIYREEEREMIPYCED

AGIGCIPWSPLARGALARPWSQMKDESSVRARSDVAIDHLLASESPFEKDIIDTVEAIAR

ERSLPMAVIATAWCIRKGVNPIVGLSSVERIDEAVLAANTRLSDEEVARLESGYRPRAVS

GY

>SoG_03344.T1

MADRPPSQPKAENPPQNETQLTSTGGVPPPTATLDHATSTPQEVPVVMPTSTSPTPSPGR

DTPVGSDAAFESTTIDPNSKGKEPVGRPLVDLTDPDPIRGNNFAFTPNQLHKLQSERTLD

ALEAFGGIRGLAIGLRTDPVAGLNADEGKLEGGVSFEEAVAAGREDRTPIRRELSALPES

HQPHPLRLLEREDNKGFEDRIRVFGKNKLPKRQQKSFLMLMWIAFNDKLLILLTISATIS

LVIGIYQSVDAEEGTSNIEWVDGVTVVVAILVIIFASAATDWQKNHKFEKLNERKEQRDV

LVVRSGCDRQISVYDVMVGDVMHLEAGDVVAVDGVLIQGSNLQIDESSLSGESELVHKVP

ADRLAHHRDLDANPFILSGTTVARGVGRYLVTAVGASSQYGRMLMSLRSDVEETPLQAKL

GRFGKQLITFGGVAGTIFFIILLIRWLANIDRLSGKTPSEKAEQFFEILILAVTVVVITV

PEGLALNVTIALAFATKRMLKDNNLVRLIRSCEIMGNATTVCSDKTGTLTQNKMTVVAGR

IGLEGRFDDIDVPETDRNRSYANPLSSKTNTSGRLIATLSQDVKDLLRVSTAVNSTAFES

EENEFVGMSTETALLKFARENLGMGKLREERANNPAVTMLPFDSSRKWMGAMVKLPDGKY

RLFIKGAAEIVFEYCAYSISDPTYKLQTDHMDKDVRQNYRRSIQEYAKKMLRPVVLAYKD

FEASEVFGDPDDEPDSVNLEWLASGMIVLGVFGIRDPLRPEVLQSVRTCQEAGVMLRMVT

GDNYETAKAIAIECGIFTPGGIAMDGRTFRKLTKDQLDAVIPRLQVLARSSPEDKLLLVT

HLKSMGETVAVTGDGTNDALALKAADVGFAMGVQGTEVAKEAASIILLDDNFASIISALS

WGRTVNDAVRKFIQFQFTINIAAGTLTILTELIGSAIFTVVQLLWLNLIQDIFASLGLAT

DKPSKDFLLRKPDPRNSSIVTITMWKMILLQAVYQLAVIFTLHYGDFSVFNPTRSPAYLR

TLTFNTFIFMQTFNQHNCRRVDNNIGIWYQGVLRNPWFIGVQCVTIAGQFIIIFKGGEAF

DTVPLTGQGWGWTMLFGALTIPLGALIRQIPDIWVLHFFHAVRDIFMFLTWPLRKLFGPC

FRRLKLHRTRRRERKKKDDETASQETQETGEKTEGKLSPMETMMIEAGRAMIQPMIIHPA

HLQVGDNGEDLDISPEQRRALERAAREQALREAEEAERIVDLPGLIAAARARRDTGPHRV

EIHPSTMKKDPILMGKRKNTHLPPSQDPDILRFMRRLDEESMGAQEAAELRARRIRAAAR

APTMGKTRERNGLSWEAMVRSKRR

>SoG_03346.T1

MSLIPFHPQEGREIVLRHRNALVVRDPTSHRLEIRGLSNCPTCHRPWQRSSSPDRHFDRS

AQLSRDPYVDPNYFRMLRPAGDDFIPDRPPSSPVRRLAEPALPTNDNLQSSYVEIEDEER

RGWRSSSSQAPQTSSKIKRESFSQNYFNDFFIEEGILGKGGKGVVLLVQHQLDGCPLGLF

ACKRVPVGDDHAWLERVLVEVRLLAQLAHPNLVSYRHVWLEDAQLTRFGPPVVCAFILQQ

YCNSGDLHKYVLGEQNRETSTEELKAQMRRRSKGQAELPRMDNGKPPLPPEEIYSLFKDI

TSGVAHLHAAGYVHRDLKPSNCLLHREGGFITCLISDFGEVQPSDAIRKSTGSTGTISYC

APEVLQLDASGKYGNFTFKSDIFSMGMILYFMCFGSLPYQSANSIQEELEDVDELRAEIS

DWQGFEDEKRLRPDLPSKLYALLKQLLSVNPAQRPSANDVLKALTSEGRFDGWTKSGRAA

TPSFGLQRVQNLDSPAAPSTPVPEPRKQSRRSSETQDLNPAWPQPALSEMGNEHDNMHGL

RRRNNAMTLTRSHADFSPVLPANISEDPSIDGHDGPLISPLLLPSPPSTSLLARLSRTAR

LGAHRLSQITGIDSTTLEYALRLSLFLIKISSVAQPCWPFMPTLQVAIPLVLMAALDLRT

SPSQAPHTRRSGVRRRAVAMSAVLLALHVSLLWLARSRDFLCIAVQHDGWPDW

>SoG_03352.T1

MSWRNQGITGSNNIPLGKRRFGGEDDEEAPAVDFKRGRDPEPRSEADGPRRRKKRNRWGD

VSENKAAGLMGLPTAIMSSMTSEQLEAYTLHLRIEEISQKLRINDVVPADGDRSPSPAPQ

YDNHGKRINTREYRYRKRLEDERHKLIEKAMKTIPNYHPPQDYRRPTKTQEKVYVPVNDY

PEINFSMIANPFNPDPNQSSVPLPSPGFDLVLGGSCTLGLLIGPRGNTLKKMENDSGAKI

AIRGKGSVKEGKGRSDAAHASNQEEDLHCLIMADTEEKVNKAKQLIHNVIETAASIPEGQ

NELKRNQLRELAALNGTLRDDENQACQNCGKIGHRKYDCPERQNFTASIICRVCGNAGHM

ARDCPDRQRGASWRNNDSRPAGRIGGGDAVDREMEQLMQELGGGSGAAPARIEAGPGGDD

RGGDNLKPWQRGPTGAAAPWRTRNDDDQGGSRGGPAPWHRDREDRGRDNQRGGDNYHGGG

QQGYGSTGGAAPWHQAAPQAPGTQGGYGAYPAYGGYGAPPGMGAPPGMPQAGLAAPPGLP

GGIDALIQQYTGAGAPPPPPPSGEAPPPPPGDQPPPPPPPA

>SoG_03355.T1

MQHHHAPYGYPSPPASPSSYDNAKFESQGYAARHYQPRCVPLAPEERLGKLLQGKLHLTD

ILGTGAYGVVYSAYDIDTGSRYAVKCLSKFNPDGTLLERRQALYQQREIRLHYLASGHEN

VVSMLKIVDDVDCIYVILEYCPEGDLFLNITERGRYVGKDDLAKKIFLQILDAVEYCHNL

GIYHRDLKPENILVTDHGDTVKLADFGLATSDDRSEDYGCGSTFYMSPECLQPNSRKPFY

RCAPNDVWGLGVILVNLTCGRNPWKQASYQDSTYRAFARDSTFLKSILPLTDELNDILGR

IFNPSPEHRITLPELRNRILNCSQFTVPAMCVSPPTPPVSPDHITAYVAPEEAIVDDCDY

DSPLSPASTNSDEGSLTSSAPTIDDLDDEVFIDCQPPQADLGQDMNTVAFESDMSEGSPT

FHAQEFMPQQHYTGPVPAAPIVVHSQPTNSQPSMQAQPHVSVQAPCQPKSYFHFWDVVKY

VQQTPMLPNHGPFHQQVSFFPVQGY

>SoG_03373.T1

MLGRLLHLGSGAPVAGQSQQQGAPSRPVASLESVHEDIHTRNLLFPDAHTLFQHRNDQVF

PLSTAPTTPSAASPASFDYTDENGIDAKDVRVIIMQDALGPTNTSVLFDSHPAPAVPQSE

RTSPVLPQESMRRTSVSRKSSLVGQSTRPVIIQSDSPQPRPGAFDRRGSSHGRTQSYAET

ETQRAAREYREELATFSSCIFGNSELMSYKGTSTKVHVVPSDFRGPESTISSILGDGRSS

IGRSSARSSKLSQSFSSQAISPSTRSPFANHAPTPRHKKVLITRLFPVNLPVEDLDSYMT

PQSRFSDENAGFPFPQTSDDGNTKKKKPQPRQRRTPMYAIVLVVQLPLSRAVTGPRASTL

RDSSSYTDGDMFPSSYSSARPSAWSMSGSGTLGDATDSVYSLDLEERIDLLTQHWDIIMR

TMTHLQSISASTITTRLRQADAASPEPYSQSSAPNLSTPAPSDRRATEFRAKQPKSTTKL

VSLGSNCLSDCVDLAQEVETARNRIVTGLCASRVVTGQGRWGIWRDEAIWTSRWTSTVDR

EQFFYNLLTGFLATHTDWLQALCPPSYRRRYFQSRQKQSEEDLSLPSRTIIVSDDKMAAR

RLIFLLSAFLPANQHLPAARAHRPSTSASVGHYPSSPPTFVIPVLREEFLRRKINRRAAP

QRASHSRTASQSTRSTAIPAQLAHLSMERAHERRPSDAASIRTASLAMPGSDIVSRKSSA

ATTTTIMPEATMPHFTSVQRTDSRRRPRPGSSNSVAADDLKRSLTRVDSSGQTTAGNRNA

SQGSRWGSVISGLWNTRRRESSNLSTYSQASGSQPPSVKMNFGRPERMSDMIHEDALPEE

PAASDMQGTPQACNARDMVSTPREQTSRAQDLCTQPDRTPDPNGAFESPVKTSINVDDGV

IDVDVPFPDYITSFESAISSPSSSGYLSTPGLPGGLESFEHSARITFEGDLPLNAAGWLR

AFHPDFALQAIPPQDNIVEAVKAALRAEPTPAPAPNTVFEAGERWVDVGSVMIADTTTNT

VSRIIYRRLVRPRAPIDRSSSGNMSSGLYGSSLLTPSILPYETQLEEEWIEDIVERPDES

MVDAMNKVMDLQQDSSKVSSCTSSHTASDHHLQDGHTSMAHFDGSNSSRIPLETPRVQCK

SVVLSALEDTIRDVIEQRDKQGDRFASSRENPLRAAVRNWVADLDLLE

>SoG_03383.T1

MVDIASRIRAKLSRRRHSSTAPSLASSRSAIDEASSVTSQSQGHSLGLSCEGSSLGDAAT

TKQGNPIVAASAASAQDGVSKDDKGHSSERTWDADGYDGDQAIANAQSTMAPKASSDAAS

DPGAAYAGGRSDNGTSPRPTVASLSSATVPPTNSGNSPPNGDTPATSPHQSRAPRAMSTA

TSAETMAAIGDRRRLASSIVQGQAQPTSNLSSIHESSNPASPSNREAAIHEASEDAGGDG

DDENEDDSDYPHRQGATLRGLANAACAQGSSTPNSVPTPTPGFLIRSPTGSSSRPQTISR

QQSLVSNRQSALVPSFVLPTLTTMGDGVAPSDAPMVTRKIWVKRPGGSATLISIREDDLV

DDVRDLILRKYANSLGRTFDSPDLTIRIHPREQQHRDRTLGPEEHMCRTLDACFPGGQTV

DEALLIDIPRRTPKASPRAPLHPYYADDGRPSEAGEGYFPPVNSVPSPHLPLAVPVTASS

HNIPHSISVLGTGQIPPIPSPGATSSRRGYRDRPERPRLGRTHTSSPTVLGVGAPNPAPA

ALPAVNHGTQQFLTRPSRSRTHSDSSDQPGHPPTAPPLPTSPGPEPIARTATPPLRTQSP

RPGVRARRTKKAAPEPPTVQPGAFSGGVPPINVLIVEDNPINLRLLEAFVKRLKVRWQTA

MNGRDAVKKWRSGGFHLVLMDIMLPVMNGLEATREIRRLERVNSIGVFSSSPGSLPDDPN

GELAEQDRLENLALFKSPVIIVALTASSLQSDRHEALAAGCNDFLTKKVMEWGCMQALID

FDGWRKWKDFSQSGEDEASKKTTSAKPKTKKNRSSLTSAA

>SoG_03392.T1

MAANVDPPTKPCHNCRRQRLRCDRSYPRCNKCILAGKECLGYGQLFRWTGAVASRGKFAG

KTSLAADEDEKAGAKSSRESSILNPSSEKWTLSSDAPSPTGTENNLQLVHAMSAPVDEVP

GPWSLTDPLFQDLSYPYRYYLSYFTARVCKDLVSYDLPENNPFRALIPLTKSQPLLQHIL

VALSAAHMSILLRAPLPSSFRGGKKETYPSNELAAKVAANDSLVAKQKALQLMHYAVQNI

ESVGADVMLAAALFFVNLELIESGKHGWRAHLEGAGRIMALLPAGNPANESLRDYLLSDC

FIYYVLGSAFSPPKSGTFAHFQPDRLPTTLKRAAANSYLCCPPEILEVLYAASQLSATPE

DHEASVEEAAALIKRAEEFDIRGWAEGLHRQPQFRSIPVQSRIHAASAHRLAACLYASQA

VPNAVSHLGEEECERLGRELITHLSSVPDEDPNFKATAFPTFIAGAEAKDPKKREWIMNR

LRRLVFWCPWGFLYTAMETLEVIWKLEDEGKNQLGWIQTLKDPELNFLIV

>SoG_03397.T1

MSANANSNSKPRRSGDERTPLLNGSSSPRDDDNGGENHYPTQSSDTMVFLFNSEHTPGKD

HHNIAIRAFAHSWHVTKVTLLSNYVNFLLVMVPIGIVAGASGWNPTAVFTINFFAIIPLA

AVLSYATEEISMKLGETMGGLLNATFGNAVELIVSIVALKDGQIEVVQSSMLGSILSNLL

LVMGMCFFFGGLRHRGSSGNGTEQTFSSVMAQTTCSLMTLSSASLVLPAALYAILDQSDS

NEDEKNKSILILSRGTAIILLFLYALYLIFQLRTHSNLFDAENQNPEEHNEETPNLGPVA

AVMVLVVTTLLVAVCAEYLVGSIDDLVETAGISKNFIGLILIPIVGNAAEHVTAVVVAIR

NKMDLAMGVAIGSSIQIALGVTPFLVIVGWIIGRDMTLHFETCKSLYPHPPEEDNC

>SoG_03445.T1

MENTPWSDGVPGVTQREIPPGGSFQYKWTATQYGEYWYHAHHRGQLDDGDFGPLIIHPKK

DRQTPFSLISQDKTTLSAIEQAVANIQPVMLSDHRNIPAPEAWDLEVAANMELPCYDSML

VNGKGKIDCWSAEKIASLTTPQQKALLKIVNATSMTPKGCLPKEADAAILAAGRTVNLSA

IPAEIYDECTPTEGSRAVIEVKSNGCGSQGTWAALEIVGAYSLFSTVFSIDELPMWIYAV

DGEYIQPQRVTAIRVTNGDRYSVLVNIVETGDYTFRHASTLPVQLLSGQATLSYRDGKKP

ANKSGDSKPYISDAGLPLSSDVVFFNQTAQKSLVPFPVAQKADQTFVLGMGNTETAYMWA

LNGTSQPMALDAEDPILFKAQPNLMNNLTITTLNDTWIDLVFVTTQVPEPAHPIHKHGNK

MWLLGSGQGAFKWSSVDEAIKAVPQSFNLVDPPRRDGFSTMDAPHAPTWTVVRYHVTNPG

AWFLHCHIQTHLLGGMSMVIQDGVDHWPKVPSNYLEYGS

>SoG_03446.T1

MIRTIPAISDEFNTTEDVGWYGSAYFLTMCAFQIFWGRLYTFYDLKITYIVAIVIFEVGS

LLCAVAPTSTAFIVGRAFAGVGAGGVFTGSFVTIAFSVPLVKRPMYASYLGTVYGLAGLL

GPPVGGAFTSNLSWRGCFYLNLPLGAIVVTGLAFFFRSPAAAARMASLPPKEKLKRMDPI

GTVIFVGAITCLLIALQYGGQTYPWSNARIIVLLTLFGVSFITWVTWQVYLGPIATIPKS

IISQRSMAFTCFYSFTTGGVNFGLLYYAPLWFQAVRGADAVHSGLDIVTFIAGMTVTMLA

VGYILTKGGYSAPFMMACVCVVSVACGLLTTWTPRSSDAEVFGYLTLYGLGQGLGWQQPI

LIAQTMLPAADIPTGTSMTTVCKLLGGTIFVSVGQSLFNSKLATLVTQRLPQLAPGTLEA

IGAVELRSKLDPSLIDTVTSCYNDALRDVWWMLLGLTAASLFGTLGVEWRKVTDKVASQG

VPPLHQQEPMRELKRDRLPGLQTTSNSTSKANLASLAGAPESPESRV

>SoG_03447.T1

MSDQGHLYLFGDQTSDFVPGLRQLLRVRDSPLLAFFLERTHNALRLEISQQPGEVQSLLP

RFSRIVDLLSSYSIDADSNAALASTLTVIYQLGSFIRYYEDGSKSKPYPSGQNHVVLGMC

TGQLAACAVASATSLIELVPLAVEAVVIALRVGLHVTKIRELLEDGPAKHQPWSYIIPKL

SAEEASPRIEQFSQSANIPFGSRPFVGTVTPSSVTVFGHPDALKAFTASPYMSDAKSLPA

SVFAPYHAPHLFSQEHISEILSGLPSPVSHVSAKLRMISSTTGEPMKATTLYELLHTSLE

EILRQSLRLDNIAKSLSTSCNAVLHGKWLILTVGANAPQTLANALCENQAAAVTVEPISS

TQNVGRTSMPNDANVGRQDQSKIAIIGYSGRFPEAVDPESLWDLLHEGRDVHREIPPDRF

NVETHYDPTGKKKNASQVRNGCFINEPGLFDCRFFNLSPKEADQSDPGQRLALMTAYEAL

EMGGIVPDATASTQRDRVGIFYGMTSDDWREVNSGQDVGTYFIPGGNRAFTPGRINYHFK

FSGPSVSVDTACSSSAAAIHMACNSLWRNDCDTAIAGGTNVMTNPDNFTGLDKGHFLSRT

GNCKTFDDGADGYCRADAVGTVVLKRLEDALADKDPIQAVICGAYTNHSADAESITRPFA

GAQAAIFRRILNDAGHEPLDVSYIEMHGTGTQAGDAVEMRSVLDVFTGKSSGRSANDPLY

LGSVKSNVGHAESGSGVTSLIKVLLMMKNSEIPPHCGIKTKLNTGFPKDLTERGIRIALE

ATPWQRPADGRRRMAFLNNFSAAGGNSALLLEDAPSCSEDASLSAEDPRTCHIVTVSART

PKSLLANAERLAKHLSQNKQNMSLGSISYTTTARRMHHNYRLALTGRSIEDITAALEDSA

VSLANSSKPIPRKAPTVAFAFTGQGSVYAGMGKQLFDSFRVFREELRRLSEISMSFGFDP

FLGLIHPSNAEKDPDFQATPQATQLALVCFEISLARLWKSFGVTPSVVVGHSLGEYAALH

IAGVLSARDVVFLVGSRARLMQEKCSAGSHAMLAAKAPLSDISAISQEYGLDVACLNGPT

QTVMAGLVHDVDRAEKAMQQQLGIKSTRLDTPFAFHSSQLDSVLEEYQAITRGVVFRKPR

CPILSPLLGEVVTEKADFDSDYLVRHCREPVNLLSAVQDSISQKLISEDTICLEIGPDAI

VSRMIKSIVGTSTRTISGVKKNQDPWETLSDAVASMYRAGVDVQWSEYHRDFDEYCHVVS

LPSYQWDYKNHWIQYVHDWCLTKGDAPNMEVVAIAPAAASDVQPTYESLSSTCQKVLESL

HGTERSEVLVESDMSHPDLRAVFEKHKVNGAVLCPSSVYADIAMTLGDYLAKRNPRQANT

GIEVAEMATTKPLLMRNPGKPELFRISAEADWNTQAAKVVFYSVTTSGERSTEHATCTLR

FGDPEAWKSDWKRIAHLVQGRMRCLRDASGQADSSCHLIKRGMVYKLFENCVEYGDSFQG

IEGVYLDSKSHEATAWVNFQDKSTCFFANPYYIDSLGHISGFVMNATEAFDYRSQVFLNH

GWESIRCAAKLSPEETYQTYVKMNSSDGKKYVGDVYIFHGEEIIGVNQGVAFQSVPRKVL

NMLLPSPARGGDTVQTTRTKPSPTVASPEVKSQPPPRIPQSIPAASRVLPSDPRERPAPA

APELVLIMQAINIIAEEIGIAPAEMNNELEFADAGVDSLLSLTVCGRLREELNMDISSSL

FMDCPTVRDLKRYLGATMGPTPVSSYDSSSDGTQGSGRSDQGEESDATSIDDNFDTKHGR

EMVIMESSSGETPLISALCSILADEVGVQVEEIWSAPSLSDLGVDSLMSLQVLGRLREEV

DLDLPTDIFFHDNMVTIREKLVGTSTQSPMQDTSISKTALDSTLRTSSSLVDIPAATSVV

LQGSLKTAQKVLFLFPDGSGSATSYASLPRVAQGVAVVGMNCPYMKRPQDLKCSLADLTT

PYLAEIRRRQPHGPYHLGGWSAGGICAYDAAKILIAGGEVVESLILLDSPNPIHLEKLPT

RLYHFLSGVGVFGSGDPSKALPEWLLPHFLAFVDALALYEPDPFPPGRAPSTHAIWAADG

VYRSTGGKRLEKQADDTRELRWLLEDRVDFGPNGWDKLVGVNKLKMQVLQGANHFTMFNG

QQGQQLSRFIATSLGI

>SoG_03467.T1

MTRTDCSSRTLNSLPPRFVADFLVHAFFSHAETNYYYVDRRWVLEKLDIAYNHPSALTPR

DVGTVCVLFIVLAIGTQYAYLDSPVEGSQDNGLGGTFSEDSIGVSFYQQACKLVPDVITI

SSLECVQACLLLGIYTLPLDASGLSYVYMNLAVTLAIQNGMHRRYPAQGVDPVVRETRNR

VWWTAYTTQRRVSIFHGRPISFESQYMDAELPADHPDILPSRPSTHVPSMLATLQLHQML

GKISQEMDVLKSQGKSGGVESLNRVVELHAPLKQWWENLSETVCRKSLDPSPLMKRSDMH

LRLEYNLVRMFTGRPFIFLGVPNRGSTSASSSPADVASQSSPSKRTSQRRRSGSQNQSAS

RRSDAKDKLVKDCVEAALDVIDVCKALRNTIGLARASYTEFSACRSALLIIITQCLQHRT

PRLRQALREGLAMIKLMSTGGESARSEASLIEVFERAIARLDAAEGAVTTSTDSNYSRFK

QWEMLLRKKESQPSGSVNDGDPSPSSATFAPMPPPMNGFSAPAQNLGLDGADAHGIAPGT

APMDWSFASFPQTVDEFSSMFGYGFPNGTEASGVDASDPGRAGMWLL

>SoG_03477.T1

MPGTTETGAAASDAPATNAPGRLLLLSNRLPITIKRSEDGNYTFSMSSGGLVTGLSGLAK

TTSFQWYGWPGLEVPESEVDGMKQRLKEEYGAHPVFIDDDLADRHYNGFSNSILWPLFHY

HPGEITFDESAWAAYQEVNRLFAKTVVNDIQDGDMIWVHDYHLMLLPQMLREEIANTRKN

VRIGFFLHTPFPSSEIYRILPVREALLAGLLDCDLIGFHTYDYARHFLSSCSRILGTPTT

PNGVDWNGRFVTVGAFPIGIDPEKFVEGLNKPAIKERIAALKRKFEGVKLIVGVDRLDYI

KGVPQKLHALEVFLTEHPEWIGRIVLVQVAVPSRQDVEEYQNLRAVVNELVGRINGRFGT

IEFMPIHFLHQSVSFDELTALYAVSDVCLVSSTRDGMNLVSYEYIATQRDNHGVMILSEF

TGAAQSLNGSLVVNPWNTEELAQVIHDAVTMSPEQREANYRKLERYVFKYTSAWWGASFV

SEMTRLSAEGSQPKTLRNISGSVVGDIGQKVKQAIEGVEKLALGDSKDGGVIEEEKTA

>SoG_03482.T1

MSLPDPTEDLDWGGYVGGIHEIFHKNAVAHPDRACVTETKTSKAPTRTYTYRQIDEASNN

IANYLRDSGIQNGDVVMIFAHRSVELVCAYMGTLAAGAIVTVLDPQYPPQRQQIYLQVSQ

PKALISIRKATEESGPLAPLVEKYIDEDLGINIKIPDLRFTDDGVLTGGAEGSADIFANV

KERASTPPDVLIGPDSNCTLSFTSGTEGLPKGVLGRHYSLAKYFPWMAERFGLSSESRFA

CLSGIAHDPIQRDIMTPLTLGAQILIPAKEDIQHVKLSEWMRDWSPTTTHLTPAMGQILV

GGATAQFPSLRQVFFVGDVLTTRDCRSLRQLGPACTIINMYGTTETSRAVSYFEVRSAQD

DPAALEQLGDSVPAGWGMKNVQVLVVDREDQTKICPVGVVGMIYIRAAGLAEGYLNDPEK

TKEKFIDNWFVDNNKWVEADKASDKGEPWRKYYKGPRDRLYVTGDLGEYRPDGSVRVLGR

MDSQVKIRGFRIELNEIDANLGGSPLIRDSKTLVRRDRHEEPTLVSYIVPEIAEWKRWLE

TQGLQDVEEEGVEMGPCLVYLKRFRRIQAEVRDHLKSRLPAHSVPSIYIVLQKLPLNPNG

KVDSPNLPFPDASLMTEDASEEDLKSWEGLSETEKAIATQWSTLIPGLNAKMVRPGSSFF

DCGGHSLLAQQLLLDIRKQFRVDVTIGILYSDPTIRGLASTVDRLRSGQSVVVDNSNETV

YSDSLDELTKTLDAKYQSADPEARTPSSGAVFFLTGATGFLGAYLTKDILDRENTKLIAC

IRGAKDLKFAKERLIRSLKGYGLWQESWVDRISCVIGDLSKPRLGLDDASWKHVAETADA

FIHNAAYVHWIARYEQMMGPNVLSTIDAMKLCNEGKPKLFSFVSSTSTLDTDHYISLSDA

QTATGRGAVLESDDMMGSRTGLGTGYGQTKWVSEQLVREAGRRGLRGAIVRPGYILGSRN

SGVSNTDDFLIRILKGCIQLGARPRIINSVNAVPVDHVAHVVVASTLNPLPGLQVVHVTA

HPRLRMNEFLSALSYYGYDVPEVDYDDWKSQLEEFVSAGAVEKDQEQSALMPLFHMATSN

LPSTTRAPELDDRNAVAVLRTDADRWTGVDDSAGEGITRDDIGRYLRYLVEIKFISPPAG

RGRKLPDIDGSIAEALSQWGVGGRGGSS

>SoG_03502.T1

MMSASFIQRRAFSASARNLSKVAVLGAAGGIGQPLSLLMKLNPRVTELALYDIRGAPGVA

ADISHVNTKSTVKGYEPNASGLADCLKGAEVVLIPAGVPRKPGMTRDDLFNTNASIVRDL

AKAVAENAPKAKLLIISNPVNSTVPICAEVFKSKGVYNPKTLFGVTTLDVVRASRFVSEI

KGTDPKDENITVIGGHSGVTIVPLFSQSNHPDLSSNADLVKRVQFGGDEVVKAKDGAGSA

TLSMAMAGARMAESILRAAQGEKGVIEPAFVDSPLYKDQGIDFFSSKVELGPEGVEKIHP

IGEVDAVESKLLEACLGDLKKNVEKGVAFVASNPGN

>SoG_03507.T1

MAPLVDNPQIQHAELLRPLPTYLHAYVWPFTIAWPIFFAFYLSPDLYEKHIGAQEWTFVW

CGTIITFQSLAWLMTHWSVNLAARFTSTTVNDVKDAERIKVLPIANAGSGEICQLEREKD

GGKTKLSFLFQKRRFLWNPDTKSFSTLRYEIDAEPKPLIGQLQSSRGLEKQATITQLEHH

FGTNTFDIPVPSFTELFKEHAVAPFFVFQIFCVGLWMLDEYWYYSLFTLFMLVAFESTVV

WQRQRTLTEFRGMSIKPYDMWVYRTGKWIEVASDKLLPGDLVSVSRTKEDSGVACDMILV

EGTAIVNEAMLSGESTPLLKDSIQLRPSDARLEPEGLDKNAFLWGGTKVLQITHGNPDQE

RPKLASGVPAAPDNGAMAIVVKTGFETSQGSLVRTMIYSTERVSANNFEALLFILFLLIF

AIAASWYVWDEGVRKDRKRSKLLLDCVLIVTSVVPPELPMELSLAVNTSLAALSKLAIFC

TEPFRIPFAGRVDVACFDKTGTLTGEDLVVEGIAGLGLGHAEIDDKKEADGAHSNMTSIT

DSSLETSLVLATAHALVRLDEGDIVGDPMEKATVSSLGWTLGKNDTLHSTPKAGNATGNV

TIKRRFQFSSALKRQSSVAFVQGIDKEGRKMKGTFAAVKGAPETIMKMLVEVPEDYEETF

KHFTRKGSRVLALAYKQLTNDSELGASKINDLKREKVECDLTFAGFLVLHCPLKEDAKEA

VQMLNESSHRVVMITGDNPLTAVHVAREVEIVDRDVLILDAPDDHSKAHSEGIDLVWKSV

DDKVSIPVDPSKPLDPKILKENDICVTGYALAKFKDQVGWKSLLRYTWVYARVSPKQKED

ILLGLKDMGYYTLMAGDGTNDVGALKQAHIGVALLNGTQDDLTRIAEHARNTNMKAMYQK

QVDLMKRFNQPAPPVPVMIAHLYAPGPSNPHFNKAVEREAKKKNITPEEYAKQQGHTFQA

IEPTATPETSTDPRVAKQQAAAKKAAGFADKLTSTMMEAEMGDDEPPTLKLGDASVAAPF

TSKLRNVIAIPNIIRQGRCTLVATIQMYKILALNCLISAYSLSVLYLEGIKFGDGQYTIS

GLLMSVCFLSISRARVVEGLSKERPQPNIFNFYIIGSILGQFAVHIVTLIYVARLCERLE

PRSDEVDLEAEFKPSLLNSAVYLLQLIQQISTFAINYQGRPFRESLSENKGMFYGIVGVS

GLAFACAMEVFPEINEQMKLVPFTDEFKYNMTTVMVLDYGLCWIIEKVLKMLFSDFRPRD

IAERRPDQLAREEERKKVLAVKKAEEEEAKRLEKVAEWERKVEERRRKIEEWRAGRQ

>SoG_03517.T1

MAPYSNAGTASPSVGSNTPGHHQLQHQLPPPPRSASVAATDPNSNAGNTPAQQPQHKRVY

QACIPCRRRKVRCDLGSVDNPHDPPCVRCRRESKECFFSATRRKRKADDGEVSDQDEYII

RNGRKRPNNASRTPPPVDSSLYSDVPLTPGGSQGRSRPLRRPDDHGAKDHPDGSLPRGHG

DDHDHGDANQQLENVEAQTVMRRGLYGPHDALDLLYKAATDRSVASEFTKAKANSQANGT

PPQQSGFQHQRHESRSSSAAGGLNGIPKPVGRSSSFAGADSREHRMKRQMNRHASDVGGA

RPNMEQPQRADEQQPIDPELTEQDPSTQPGYNDAIRAWQRFRFVRAGWFTAQEAIQYIDY

YYKYLSPMTPISPPTFSDPSSHLTLLTEEPILSVTLLTIASRYKQLDGPGGHSRSYAIHE

QLWTYLRGMIERCLWGQEAFGSGFCGSGSATIIEEESKTSSTAPWRGMRKGSLRTLGTVE

SLLILTEWHPRALHFPPQDATDELMLPDFEAGANMHGGSEGGEEGAPRNNASGFGGKRIE

SWLEPAWRSDRMCWMLLSTGMGLAYELGVFDNIEEMIQEDGANRPEFLDEAYRARANRIK

RLLLIYTTQLAGRLGWTSMAPDELRRDDPAFARRSSKHPQQQPQQHSGGGANNEDATPGT

TYSALSHRFRYVPDMELDDQIIHCWAGISFAMHMGNEKLFRSRRHTQEIIQSGRYVSLLG

EFGPMLKDWEREFELYRLPPFIRCILRIEYEYVRIYVNSLSLQAVVERCTNNATGAAHNG

GSTDGDQQHQPTQEPAQPPQAQQPQQGQDQSGPPQLSPQTIMNYGKLPLGQLGGFTVNDQ

EYVREVVEGSRNLLNEVVTGLLPNGYLKHAPVRTYFRIISGAMFLLKTFALGAPRSDVKA

SIDLMDATVEALRGCVVDDVHLGIRFADLLESLTSRLRNRFIQAPTLQQVVTSGRGTSPV

AGAEAGAGGNAGGDWIGGHAQSLRDGLNGQFRPSSPAMEANNISATPFDLTASSFPYPGG

ASLGPSTPAAALDLNNTNNANGQQHQNHSQPPPNGGIDATLFDEWNNPGNEMWYLPPGPA

FFQNVGVGGDSSTVAMTSEGVNVGGLDLLDYMGMDSTQYGGTALEF

>SoG_03523.T1

MSKRSRNQSSGAATEASADFDYEIAPQTKKAKVEERRSLFVRSLPPNATSESLTDFFSEH

YPVKHATVVVDKQTNESRGFGFVTFADAEDALAAKNALDKHEWDGRRIRLEIAEPRQRDA

AKGPKPTPSGKPPGRDEFEKPPKLIIRNLPWSIKTPAQLAQLFQSYGKIKFADLPSNKGK

LKGFGFVTLRGKKNAAKALEMVNGKEIDGRTLAVDWAVDKQTWDQQQAKEAAADDDDDDT

SSEGESDADEDADEEEESDGGDKIPRKDDDLDADLENFMKNHMQNMEDEEDEDEDEDEDE

DGGAEINTNRPPRKLSTDNSCTVFVRNLPFTTTDEQLKGFFTHFGPVRYARVVMDKATDK

PAGTGFVCFANIEDCKACVKLAPNPQRPTSGPGTRQSILLDESADPDGKYTMDGRLLQVA

QAVNKEEASHLADTSIAKRNEKDKRRLYLLNEGSIGKNTALHKLLPESEIQMRLQSAQQR

KKMVQSNPSLHISLTRLALRNIPRNIGSKELKELARRAVVEFARDVQAGRRQPLSKEENA

RDGKDAKEKEKQRKLKGKGIVRQAKIVFESDKGSKVPEKSGAGKSRGYGFIEYSNHHWAL

MGLRWLNGLQLESDTGKKQRLIVEFAIENAQVVQRRRATEEKSSQPSKKDGKTAKNEADE

KPESEEAKPLSKRQQRRQVKKAKKKADRAEAAKEGLKKEDGGEDPKAEMQQKLIARKRLM

RKKKAQARGKN

>SoG_03530.T1

MVKAVVAGAAGGIGQFGQPLSLLLKTSPLVDELALYDVVNTPGVAADLSHISSRAQITGY

LPKDDGAKAAFKDADIIVIPAGIPRKPGMTRDDLFNINAGIVKGLIEVAAEVAPKAFILV

ISNPVNSTVPISAEVLKAKNVFNPQRLFGVTTLDIVRAETFVAGITGEKQPQKLTVPVIG

GHSGETIVPLFSKVQPSASIPDDKYDALVNRVQFGGDEVVKAKDGAGSATLSMAYAGFRF

AEKVLKAAAGEKGLIEPSYVYLPGVPGGEAIAKETGCDFFSVPIELGPNGAEKAINPLEG

ITDKEKALLAKAVEGLKGNIKKGVDFAHNPPQK

>SoG_03555.T1

MAASSEAGYLMSDAPSRTPRRQGFPGSSSSAIRPRGPPSENPDDDADGFADDQVPRSSRP

GASNGPIPKVEDKIGGIIQEHFQAFIESFVEDTSNAPAMTPGSSAMTSDKYYVAQIQGMR

TYQLSTFYVDWRHLATWENGSLADGIMRQYYRFLPFLTAALHEMIQKYEPQYFREHRQPT

TTTSTSAASHLGSASQSDASHRKNEHQQTDKLFSIAFYNLPLVSRVRALRATNIGQLLSI

SGTVTRTSEVRPELSLATFVCEGCRTVVPNIEQTFRYTEPTQCPNQSCQNRFAWQLDIRH

STFVDWQKVRIQENSSEIPTGSMPRTMDVILRGEIVDRAKAGEKCIFTGALIVVPDVSQL

GLPGLRPTAVRDDRNAPRGADAGGSGVSGLKALGVRDLTYRLAFLACMVTPDTSSTGQSG

AGGVADIVNTLTQNAAAEGNQSTEDAQAAVLASMNPSEIEDLRAMVHGDHIYSRLVGSIA

PMVYGHEVVKKGLLLQLMSGVHKSTPEGMQLRGDINICIVGDPSTSKSQFLKYVCSFAPR

AVYTSGKASSAAGLTAAVVKDEETGEFTIEAGALMLADNGICAIDEFDKMDIADQVAIHE

AMEQQTISIAKAGIQATLNARASILAAANPVGGRYNRKTTLRANINMSAPIMSRFDLFFV

VLDECNEQVDRHLAEHIVGVHQLRDEAIEPEFSTEQLQRYIRFARTFRPEFTEEAKEVLV

AKYKELRADDAQGGVGKNSYRITVRQLESMIRLSEAIAKVNCTEEITPEMVVEAYTLLRQ

SIISVEHDDVEVIDEEEDPALAAEDTQETLRAAAGTQDTAMGGDEAPSQKRQHKITWEEY

TKMVNMFVQRINDDESGSGDGVNGETLVSWYLEQKESEMEGEEDYHREKALAAMVLKKMV

KENILMALRGEGLTDGNTSAPTSSSAATANVVYVLHPNCAVEEY

>SoG_03557.T1

MGGTPAGDAAVGSDTPRQKSLATADTIIKGCIAWVEKNGQPRRAEILSIRDMKSGVRQFY

CNFDNFNKRLDEWVPVDRVDFTREVEWPNPEKEKPKESKKKAPPVAKKTVPKKGQKRPTN

KRDTSVISEANTPHPWTEFVENQDTRKSASVGPESETPARGSVEPSQTPGAAEDAETEDK

DDVKKEPAAFSREDEIEKLRTSGSMTQNPTEVSRIRNISKVQMGKFDLFPWYFAPYPESF

NQEEVIFLCEFCLSYYGDEFSFQRHRRKCTLQHPPGNEIYRDDTISFFEIDGRRQRTWCR

NLCLLSKMFLDHKTLYYDVDPFLFYVMCTRSDKGFHVVGYFSKEKESADGYNVACILTLP

QYQRKGYGRLLIQFSYELSRIEGKLGSPEKPLSDLGLLSYRQYWSENILDVLLGYHERGE

KVTIEAISTALAMTTQDVEHTLQALKMQIYHKSDHKIVIPEKLVKQREKQMQKARRQIDP

EKIQWKPPVFTASSRTWGW

>SoG_03558.T1

MLFGASSVGLLALGLQVALAEDGQIPLDDDYSAACPDYTQYSTHPHPPLTEGRYGLPFQR

PDPRCRTFRSDEIERVIEEVTSRMKDPDMARLFENTFPSTTDTTVKFHTAGEKDTGIFNI

GSFRSSWDSEAWQGPQSFIITGDIIAEWLRDSTNQLRPYQALANKDPKIKTLLLGAINTQ

AEYIIQSPYCNAFQPPPIAKLQLSNNHQEDNVHPAYEQSVVFECKYELDSLAHFLALGND

FHDHTKSTDFVNKRWLLAVETVLEVLEQQSAPTFDPETGAFKRNIYTFQRRTNTGTETLS

LGGVGNPLNSGTGLVRSAFRPSDDATILGFFIPANAMMSAELIRASAMLEAAGKPKLASE

FSKWGRQLREGVLEHGVVEHKKYGKVFAYEVDGYGSSIMMDDANYPSLLALPVMGFCDVD

DEIYQNTRKMILERSGNPYYLKGSGFQGIGGPHIGFRNAWPMSLLMQAQTTDDDKEIREC

LELVLRSSELGLVHESIDVDRVSAYTRSWFAWANGVFATTVLDLAKRKPHLIFKEGEGSY

EI

>SoG_03564.T1

MTAGLFHQYADGLLPVFKQLRYLIVGGDVLDPAVIGRVLKDGAPQHLLNGYGPTEATTFT

TTYEIQSVGEGGIPIGHPIGNTRVYVLDANQQPVPVGVAGELYIGGDGVAKGYLNRPELT

AEKFVADPFSADPAALLYRTGDLARWRADGAVDYLGRNDDQVKIRGFRIELGEIEARLGQ

CAGVNDAVVLARQDDNGPIRLVGYVIPEEGVTLSVQTLRSQLASTLAEYMVPSAFVSLVA

LPLTPNRKLDRQALPAPDADACASNWGWS

>SoG_03568.T1

MKKWKKIDKTLGLVAHVQSCLQNEVLELSSMGNLEIPRYAKVSPQIPSSLFLPELFIADD

VLTLIVLQELAIVADEIKKSGGTAIVNTNNTRNGDKIVQAAIDAFGGIHILINAASPAVQ

PKNFGDVSDGEWYAGTEAFIKGNYMTAHAAWQHFSKQGYGRIVNVSSDIGLHGASTQVLE

STVRHSQIGYSYTLAKEGVKKNILVNVLLDSGQASNRSAADEAATLVAYLVHEGNKKETG

QVYQVQNQKIAKLRWQRSGGLLLRADDTLTPAVLLKQWPQVESFDKGTEYPSGPRDFLEL

VKQGQKLARNQTVDPVSFRGRSVLITGAGSGLGRAYALHFASLGASVMVNDIADPAPVVE

EIRKLGGTAAGVKASAEEGEKNVEATIAAFGRIDVVVNNAGILRDKSFQKMTEPMWDQVL

AVHLGGTYKNTRAAWPHFVRQGYGRVINTTSVTGIYGQFGQANYAAAKSSIIGLTRALAR

EGASHNILVNVIAPNAGTNMTKTILSDEVSKLFQPAHVAPIVSALASPTAPASLTGGVYE

AGSGWFGKTRWELTQGPFEASGAEAASDKIASIVSSLQASSSKSYPSTLDEHRGLFQQGA

SSQKNVLENIKRAQSSSSLATRFTYEQRDLILYALSVGAQHTQLPLVYEGDKNFTPLPVF

GLIPFFNAKAHYDMDDIMSKYDLRLLLHVDQFLEIRSAIPRSGTLTTYPKLVQVVDKGKD

VLVVQGFTTVDERNTEVFYNETTVLVRGGGGFGGDPKLRDRGAATAANTPPSRSPDHVVE

EKTAEGQAALYRLNGDFNPLHIDPEFSAKGGFKTPILHGLCSLGIAGKHLFQTFGSYKNL

KARFTSPVIPGQTLRTEMWREQDKVLFQVVVLETGKKAISGAAVTLDGQPKSRL

>SoG_03570.T1

MSTPTERIQHLSAHLIGSAGSEKAALAAIQKKNPDDVVITLAIRTPLTKARKGYFKDTKL

EGLLVPLLEPSEQNVIQKSGLEPSQVEEIVLGNVLHKDAPFTLRASGLAAGFPATTAISH

VSRWCSSGLLAVEAVANKVAAGGIDIGLALGAESMSSNPDNGAPEYPTEFMAKQVIQDVT

QPMGWTSENVAKDFGVTREKQDAFAAASWQKAEAAQKAGYTADEIVPITTKWKDPKTGEI

REVVADHDDGIRPGTTKEGLSKIRAAFPQWPPSTTTGGNASQITDGAAAVLMMRREVAEK

LGQPIIGKFVKSTVVGIEPRIMGVGPALAIPKLLSKVGISKDSVDIFEINEAFASMLAYC

AETLEIDPARLNPRGGAIAFGHPLGCTGARQIVTALSELKRTGGRIAVTSMCIGTGGRDT

PHHVAIGDLSVSAA

>SoG_03575.T1

MNSAGPSDVSPEAMQARIQQARREAESLKDRIKRKKDELADTSLRAVANQAHEPIPKNQL

MKAKRTLKGHLAKIYAMHWSTDRRHLVSASQDGKLIIWDAYTTNKVHAIPLRSSWVMTCA

YAPSGNFVACGGLDNICSIYNLNQQRDGPTRVARELSGHAGYLSCCRFINDRSILTSSGD

MTCMKWDIETGQKVTEFADHLGDVMSISLNPTNQNTFISGACDAFAKLWDIRAGKAVQTF

AAHESDINAIQFFPDGHSFVTGSDDATCRLFDIRADRELNIYGSESILCGITSVATSVSG

RLLFAGYDDFECKVWDVTRGEKVGSLVGHENRVSCLGVSNDGISLCTGSWDSLLKIWAY

>SoG_03610.T1

MADDREYNGFNPKRRRIDSVRSQSRFSNASGSSTPRAQSRYAQTPAREQEIAKPTEEDSL

ALDRDWYGGDELGGHVFGDDSYNPFASYDTSTYDGQQQEMAKAQQMVGRFDVRQEQRNKE

NDAWETNRMLSSGVAQRRDMESDFDDQETTRVHLLVHDLRPPFLDGRTIFTKQLEPVPAV

RDYQSDMAVFSRKGSKAVREARQQRERQRQAQQATSMAGTALGNIMGAKEEDGDSALPIA

GEEDAVNGRPKGNKFSEHMKKRDGASNFSQSKSLREQREFLPAFAVREDLLRVIRENQVT

IVIGETGSGKTTQLTQFLYEDGYAKTGIIGCTQPRRVAAMSVAKRVAEEMEVPLGSTVGY

AIRFEDYTSEETKIKYLTDGILLRESLNEPDLDRYSCIIMDEAHERALNTDILMGLIKKI

LQRRRDLKLIVTSATMNAKRFSDFFGGAPDFTIPGRTFPVDVMFHRSPVEDYVDQAVQQV

LAIHVSNDPGDVLVFMTGQEDIEVTCELVQKRLDALNDPPKLSILPIYSQMPADLQAKIF

DRAAPGVRKCIVATNIAETSLTVDGIKYVVDAGYSKMKVYNPKMGMDTLQITPISQANAS

QRSGRAGRTGPGKAYRLYTEKAYKEELYMQTIPEIQRTNLANTVLMLKSLGVKDLMDFDF

MDPPPQDTISTSMFDLWALGALDNLGELTELGRKMSAFPMDPSLAKMLIMAEQYGCSEEM

VTIVSMLSVPNVFYRPKERQEEADAAREKFWVHESDHLTYLRVYDEWRLKRYSEGWCIEH

FLHSKSLKRAKEIRDQLMDIVKMQKMEMVSCGTDWDVIRKCICAGYYHQAAKYKGSGEYI

NLRTNLGVQLHPTSALYAGHPPDYVVYHELMLTSKVYVSTVTAVDPHWLADLGGVFYSVK

EKGFSIRDKRVTETEFNRKVEIEAKMAEDKRRDEERKLAEEERTAVKKKATGAVKKFTTQ

GAVKKPIVKRRGRGF

>SoG_03618.T1

MAAQDSLFVLPGDEIDPKLIPSHPKKALRLGPGLRHVAPNDILPTLAGQLVTDRQKNMIR

VETSGGRYVPRAGELVIGTVQRSTADAYFVALTDYTAPALLPHLSFESATKKTRPILASG

ALVYARVSLAHRHMDAELECVSSSTGKADGLGPLNGGMIFNISLGMSRRLMMPKGSGLVV

LEELGSAGLQFETATGRNGKFWVDSTNVKTIIAVGRAVQETDQKRLSVEDQKKTVKRLIK

ELS

>SoG_03619.T1

MEKSPRNVAAILRRFTISNHGLSKPNITRRDSLVKRRLGDGAIAGISVGSIVFCALLCLC

LYPVIIGTLKRRKRRKQREKHPQFDPEGGVTAANGFPAANTEDYGRRLSSADSVKPNENS

SRGELAPPDGKDHGQRNLAVDAGVQQTWGSQMVQGSVDSPATHMTGHDLAQHVGDGQMTP

FPTYDGGVYYPGADPSEPRGANADYYSPSIPSEAFGMYTMPSPTEQSESSRVRLRGSSLR

NGVKGLLRRASGRERTMSSTTSGDADTVGASGVTSLQTVFANQDPTDSPTEMSPATSSPP

PGSLPPSSQAFGSFSRGLSEEDHAQLPPATERSLRCSPPLNPGPGTVNPMDIMPPSSEPE

KWHRTDHELYLATHQPPSASVSPTAPSNAHFSPAANSLPPSSSPPALPLHFGEPTRHDTD

QTIKQEYGRQQPQDVEMDNAPSQDYLHPEPDPGRHLSYPSDQSTPFPGLASTNPSSHNTP

TTSQTDTPSPESSGIQSDFRHSASPAVSNPSPRHAPGHYACTVPDCGQVFDQHHKLNHHA

RYHAKQHRCEYPNCGKGFGTKTHLQRHINDRHLHTKKFHCAVPNCEYSRSGGKSFLRKDN

WKRHMTRIHHVESRDLPEPVEYEEMVDS

>SoG_03625.T1

MAAVPDTVRPAISDQPQPSYHTPARSYSTRSSRPSPPDDYYYPSNPAVTHDQPAVPRSQS

QYASASASARAQQYQHRRSASSSAVPSPAVVASHSRHLDIHHHQQQQSQTHHVSNPSDPA

QPQRYHRRTSSSVRPLQEALPENDYEASNLATYPKRSPSRDRPLPPSSSRNPPRPAADVE

SSRISHNRTASTRKSNHHRTSSQAQSHGQNSRSMAPSASNGASAPSGGPADGTHSSSGKQ

TRSRTTIPTQSGKWILGKTIGAGSMGKVKLARKEDGSEQVACKIIPRGSTDDGHQSRADK

ERADQSKEIRTAREAAIVTLLNHPHICGLRDVVRTNYHWYMLFEFVNGGQMLDYIISHGK

LKEKQARKFSRQIASALDYCHRNSIVHRDLKIENILISKTGDIKIIDFGLSNLFAPRGHL

KTFCGSLYFAAPELLQARAYTGPEVDVWSFGIVLYVLVCGKVPFDDQSMPALHAKIKKGV

VDYPSWLSTECKHLLSRMLVTDPKLRATMQEVMNHPWMTKGFNGPPDNYLPAREPLSPPL

EPEVIHAMQGFNFGSPESIRAQLTKIIDSEEYQRAVKLFQKEKELPQPNKDEQKRRGFGF

DFYKRRNSGTSRDTLTGPSSEALQLGNDPLNAFSPLLSIYYLVKEKQDRERTDGPAPTPS

TPREKEKERERDRDRDYRDHREPRDYRERVREEREKPVDALPEIAPPQAAVTNVTTYEMP

GEKPTGGRTRPRARTHGEDDVPDVVKQSPQPDHRPEHQPPKKESAASGLLRRFSTRKRRD

PERLDKDRSHPPVVQVHSPAEGASLMPRKSFSIRRGRRERDGSIEPRLRSGSSQPQHSEL

LSPPATAGGSRDSRRGGLGRSTSVNSAEMRRQKARGTAKEPPPTSGSDQSMAEEPSAGPS

LVHGHSKSVAYRAKSLGHARRESIQQRRMRREAAQEANVPEETDMEQEASGVSTERLDSS

DLAKPVFLKGLFSVSTTSGKSVPAIRADIRRVLRQLNVDFIEIKGGFTCRHMPSIDLNKV

QDPPGSPGPVSSGGGHRRRFSFGGLMRGDDREDVRDPSDRPPATPRTPGRSDRDRSDRDR

SYSNSETSVDSIPRRTNNGASRRAPGETSTQVQSDLGGSMVLEFEIFIVKVPLLSLHGIQ

FKRLDGNTWQYKNMADQILRELRL

>SoG_03630.T1

MFRQRTSSAKPGDSLLADFRQQFPQVAAATSATAAPGISQTVAPDATVAPADVTAENLAT

EGFRDEDPTPRASNAANEPWRFTPGLLDAHSFSYASFPNAPPGYYTPTPGGSNTLFHPQA

GDLHTPTIALGPALGTPLSMPTSGEALQAGPAILDISGFQTLQPHQLHHFNPFIQAAPPQ

GAFVPSSFVHQDTGYETMEHDGSPMGSDPSEGGRVTSIDSAFHPTTRMIAGVQPRTHAGL

AANHPLPPSAEKFRFHSTLNAPTAMIKQADEIPITYLNKGQAYSLTVADTHATMPLQPGT

KYRTFVRVSFEDVEQRQKPGVCWGLWKEGRGTNEAHQRGGRLQAVEYVAASQSAEGNNKR

TRVELEESSFDGFSVTWTPGASGAPELSVAVRFNFLSTDFSHSKGVKGIPVRLCAKTTPI

GPDATVPTGTKFTPEVCYCKVKLFRDHGAERKLANDVAHVKKSIDKLKQQIAQAESGIKD

FGKRKRPSVSKASAGDQRPGKVQKHKRTWSISSASSADGSGSKMTVEEDLHFKLQTLQDM

FTSTRPVSLLFLRGEDLDDPDKHPVSLPGEQSPLQKTGSVPREGPNWQARSGRSSVVDSM

VSPSPSSLSLASQASAVGQSGHWQGFDSVVGGEVRQADQPTKVGKTDDDGKLSGWIEALG

VDQSYRPPPERSPRPVACFYILNKSTPNAPNAEYYHAVYLLQRTLKDFTEGIATKWGLDV

SRILRMVRILDNGLEVLVDEDVIRELKEGQDMILEIQDCTEPPIPAKREWEMTVDGTGEG

APKGSTPPASGFVLRLRF

>SoG_03635.T1

MSGAELATTQTGGNNASEFPADLGEHRFANFIGMLQRLDRNSIAMANGETSMLEDPTHQD

VARWGKDGDTFVVEDGERFTRSILPKHFKHSNMSSFIRQLNKYDFHKVKPSSDSENMGNV

LEFKHPYFRVDSKDDLDNIRRKAPAPRKAPATEDYSTQHHMSVISEQLTAQAQQVQHLQE

LYAEVSQTNKVLVNEVLTLNKMLHAQRQAQYEMLNHLTPFHNESRNSGMMSQSLNSGGST

EAEDPPELRRARELLSSVSTDSLADRELERLHGVYASPTDSSAMIPPSMPMMHDPMNDLN

RYPVYPVGQTVGIDPFHSDHIHKIPYSLPQEAGGALLAEAPVSQPLVNPSTPTATGQIGK

SDSMWGPKKPNVFLVEDDPTCAKIGVKFLKSMGCDVEHAPNGAEAVSRISNVSRDHFDMI

FMDIIMPRLDGVSATLYIRGTHPAVPIIAMTSNIRPEEVNGYFDHGMNGVLAKPFTKEGM

WKSVKTHASHLLKNPPQNENHSYMLGGGAYFNTQMQGGNSSIKFDTPTPPSGTGGSTWSP

AQMQQPSPINNSNNSNNNGNDQSFSGMMNGTNQYGMTGGNRYSSLQNDSSRISDHDSPPE

KRQRLNAPQGSY

>SoG_03653.T1

MAPKQDTPFRSADMSMVQLYVSNEIGREVVTALGELGLCQFRDLNEDVSAFQRTYTQEIR

RLDNVERQLRYFYAQMEKTGIPLRKLDLDVERLASPSTSEIDELAERSQKLEQRVYDLNE

SYETLKKREGELTEWRWVLREAGSFFDRAHGNVEEIRASTDNDDAPLLSDVEQHNSAPDV

ERSFSGMNIGFVAGVINRDRVGAFERILWRTLRGNLYMNQSEIPEPLINPSNNEPIHKNV

FVIFAHGKEILAKIRKISESMGADVHQVDENSDLRRDQIHDVNNRLEDVQNVLHNTQATL

QAELNQISQSLSAWMVLIAKEKAVYNTLNLFSYDRARRTLIAEGWCPTNDLPLIRSTLQD

VTNRAGLSVPSIINEIHTNKTPPTYLKTNKFTEGFQTIVNAYGTATYQEVNPAMPVIVTF

PFLFAVMFGDFGHATIMLAAALAMIYWEKPLKKVTFELFAMIFYGRYIALVMAVFSLYTG

LIYNDAFSLSMTLFDSAWEFKKPENWSERMPVTATLNEKGYRYPFGLDYAWHGTDNDLLF

SNSYKMKMSIILGWAHMTYSLCFAYINAKHFRKPIDIWGNFVPGMIFFQSIFGYLVMCII

YKWSVDWAAAGSQPPGLLNMLIYMFLQPGTLDEQLYPGQATVQVILLLLAFAQVPVLLFL

KPFYLRWEHNRARAQGYRGIGETSRVSALDDDDEHRPNGHGDSFDEGEGVAMLSQNIDEE

HEEFEFSEVMIHQVIHTIEFCLNCVSHTASYLRLWALSLAHQQLSAVLWSMTLGPCLAMS

GAMGVIAIVIGFTAWFFLTIAILVCMEGTSAMLHSLRLAWVESFSKFAEFGGWPFAPFSF

DALLEESEDLKEYLG

>SoG_03676.T1

MALQSEAAIKGLGCSTAKRAMTEESDTPDARNVRIATPPPVSERVRLDARQIAETSSNRR

LRQSARHSDGSRSDHTDSEPFESSLFRELHRPHRESTPSASPHRKRQRINGDRFIPSRSG

QDLQASFSLLHEDGSPATASRQKKRTPHGELHFQKTEEANRTFSTLLRAELFESSVPQAS

SPAVSSDQGITGSSTTLPHDGTRSHTPPNNASTTSLPSSLTPSTPHKNLFSYMSPRHSHV

GGHLTPSKTPQSRHGPNLDTRAEIYSLSPVRFGSQQLLLSPRRQPRAVSKVPYKVLDAPE

LADDFYLNLVDWGSANVLGVGLGSSVYMWNAQTSKVNKLCTLDDDTVTSVSWIQKGTHIA

IGTGKGLVQIWDAEKARRLRTMTGHTGRVGSLAWNTHILTSGSRDRLIYHRDVRAPDQWM

RKLVGHKQEVCGLRWNCEDGQLASGGNDNKLMVWDKLSDTPLWKFSDHTAAVKAISWSPH

QRGLLASGGGTADRRIIFHDTVKGTVINEIDTGSQVCNIAWSKNSNEIVSTHGYSQNQIV

VWKYPSMTQVASLTGHTYRVLYLAMSPDGRVVVTGAGDETLRFWSVFGRRPGTREDGESG

GSKLNEWGIIR

>SoG_03687.T1

MFPTYLIPSRSSFHPSIVSFIHHSPTISIPSNHDSLSPNATKHDQLWTFKHLIFQQTQTG

LGKALERSSASHLSSSPPSLPPSTRSVTTVTTTTVIENPQHGQEPQESTIVGTAGTRKPS

FSSSSSSSRSNERRGGGSRGVSRHSQKQERRRRLSNTSASTVASHSSAGANSLHDEESIQ

QPQPPLITSFLTDNASAFIINWFQEVCPAWSGFDSDANWNRKIAIDLWQTSASVSSALES

MSASFLASRLPNLRQTSLRLMSRATEFIQAELQVVKSQPVLCTIPAGVMFAMFCMATTIC

WVDSRGLGLPFFRELRALLRRLNAQPPTSTAAPGRSSRELLEYFNRSMVYCDMLLAVVSD

DQKMGLSMDDMSPQIATAARESEPLQTRGAPDMLHPWTGISTSSSRLFAESVRLCRGSRA

RLRQTLTNTQACFSAALEDLLRAQALEEELLSLEFGGAQHQHQYQHQHQLSDTGDLSTPQ

SHLTMTAEAYRVSALLHLYQTFPELAFQRLPAPAAAAGGQLGQRPPNDAALWNDWIVPLA

LHLVRILEQIPPSSGSRVIQPLLYISAATGLRHSSAPASATAPAQCNHVVSSLFPSSMGH

GDGWGWRRQLQQPSLSESDTLTSYITHITSSSGKPTSILDHTTGSSSPSSDLSMEISQAR

HFIMKRLGMFETSLPPGPILVAKELVSTVWSSYDSDFGGNEGLYMVHWIDVMELNDLRSM

FG

>SoG_03696.T1

MSSDISEGPNQSKDVPEAVDDQKPTDLLQSIKPTKCGVCHSFPPKYKCPRCFMPYCSVAC

NKKHRENHPPDPEPVMKSTSPALPTSQPTVDAQPPTYSDPNNPFHILEHSDKLKLLFAKY

PGLPDQLRRIHDATLPPKPAPGTKPGIPDSLMQGIKKKETWNHDMGIKNGKAALRKAKTA

DGEEGEGVREYYELVLHTMNESSDREKASQFVQQQLAQEDTKLIERLLAQEKR

>SoG_03729.T1

MEDEQYQHPLLEGEFNEPPMSDQPLLKYFVIRQEVRLEVNFRDRRIDGETDVIIVTFDDK

IEDVLLDAADCSISTEKITVSEIRYVNGEIIEGQARPATAEFDDPYRKLSRLPKSNWSAK

HHDLRRLRARSLFYSRQTDVPAENREFEGCTPAYGSLRVGLKNKADQDRPRLVIRKSTSN

LDGTDTSSHKQYKITIPFSKANPRDGIHFVGVDSLDNRFPHMYSRHSFNPGTASCIFPCI

DDHGCRCDWRISIKYPRTLGDALHQALATQQQGAQRAYELAEEDKLREMSVVCSGFLMEE

SVDPDDEHKKIMTFEPEKKVSIQNLGFAVGPFEHIDLSSESRTEEDELKLGMNALKVHAY

ALPHRGDWVRHTAAALTMAADFMTFTFAKYPFSNFKVVFLDDMVDDTISLLSMAFVSNRL

LYPEDIIDHEIEVTRQLVYTLTYQWTGINMIPNTRSDLWLVMGLAHFMTDLFMKKLCGNN

EYRFRMKQLSDRLVELDIDRPSMYELGTKLHLDPQEVEFLTLKTAVVLFILDKRLIKASG

GHGLTRILQKMATKNVIDSSDKASILDTEKFRATCEKAAKFRLESFWNQWVYGAGCPTFD

VKARFNKKRLCVELTLNQIQAQALKKRGLTEDDFLRLVKEKRVDLRIEELQPLFTGPMTI

RIHEADGTPYEHILEIREDATRSTKFEIPYNTKYKRLKRTRRMKEKHLGANMDIATENAE

DALLYSLGDVLQSADDLRDWELIEWDPETERKMDQESYEWIRVDADFEWACDMKRNLEPY

MYVSQLQQDRDVVAQQDAMLYLTHGPLHPIASGFLVRTLVDRRYFHGIRTMAAQALPRQS

NIKDLPMLGLRQLMKAFREMFCYDNPSNPNQPRPNDFSDKKQYNVRCAIINAVASVRDSN

HRCPLDARRFILDQLLFNNNEENPFSDHHYIALLVKALATSLIPSKKDDWYAKQAKQVDE

DERQFLETALEQIERVLRRDEWTNSYQNIWTIAGLDAKQRLMKAEVIERNLPEFSQYLLD

GTADVVRIKCFEALIDLGAIMDSTFFTFLLYALATDRSPFVRNKLIHALSRGIAAIAFGE

HTVVTKKEPAPEEDADPLLLVQDSAQEIEARQEMFARKQNLEAALKALQKEMDETYHADA

RIYATAMRKALDHPSLGRTEVENLLILAGMLFEESSDWIMTLNLPKAWTIGRSTTQNPKR

VRTGQSHLILTATTDTSQLLMTFKSFYRIDPKKPLITQPAVAAAPPPPPPPVPLATIAET

PRPAPIARTGSIKINTKTSSSSQPRTSLPSSATSDLAPNPRQPSAPKPLTPVTSIKRPRE

DSGSPAPKRPKTEDPWEIPDDLATASRKKTKIVTLKTKDPRRLALLLNKPYIPSPAPART

SLPGSSSSSSVPRTPLVEGARKPLPGSGADSSHRKPLPENNTPHSAARKPLPSGDRKALP

SSKSPPPPPPPPSALRVNTSSSASPAPSGTPKPSPGMSTPSSAAPSGRKIIKIIRKPQAP

PPPPSS

>SoG_03747.T1

MSKQNEDLLRRPLYAYDLPPEVLESLSLKTNIDASDAIVESPAIRSPSSDGARTPSDLVG

TQSCSLCGLAFPTLQDQRSHIKSDFHNYNLKQRLKGSKPVSENDFERLIGDLDESLSGSD

DESDEDEDDGRQESTLTSLLKRQARLSEKQRAELDDGGGSDEDTKPGRRGSGKPPLIWFS

SPVLPENHYFGLYRAILTGEELRNSDLVQVLRKKQLDPIAVPKPSKDGALPEFVYKGPHV

FLCMIGGGHFAAMVVSLAPRQGKGSVAMNREATVLAHKTFHRYTTRRKQGGSQSANDNAN

GKAHSAGSSIRRYNEQALVQDVRDLLQDWKALLDTSELLFIRATGATNRRTLFGPYDGQV

LQHNDTRLRGFPFSTRRATQKELMRSFIELTRLKIREIHPDQEVAKPAAPSTKPSTPKSS

KSRLSEEEETAQLHTSQIQAFIRRSKLPALLGYLQKNNLSPDFEFQPPEQNYHAPRPLHL

AASQNSAPVVLGLLTRGSADPTLKNRDGKTAFDLSGERPTRDAFRVARSELGEAKWPWSE

ARVPAPLSKEEASKRDEHEKAEASEKEAERRKAEEARLAREGPRLVDERARSKGGKLLGT

ATEKTAQDRREEEGRGLTPEMRLRLERERRARAAEERFRKMQGN

>SoG_03778.T1

MTPRGSTPAAPPQPSTKAAPAAPKLNNELEMDSMPTEGGAQKPKQDDVMQLARLGDVAAM

EKLFETGGFDATYTDDEGITPLHWAAINNQYAMCKFLIEHGAEINKKGGESVATPLQWAA

QRSHYYVVNLLLQHGGDPLITDAQGYNTLHISTFNGNVLLIVLLLHQGIPVDVVDSYGHT

ALMWAGYKGFPLCVDVFLRWGANVQLTDEQGFTALHWALVKGSPACIMKLIEYGADRFAK

THSGKTPSVTASELNTEGAWHKALRECGYDDDGHSLTPPWPGASYFLQDKKGFINRFMFF

CPFVLVWAVISVLAHAPIYLGIPFAFIVGYGILWIAQQVLEYAPSGMGALHKTPWMAGIF

SGSLTLVAVNWLTTILPATTFWAANSNETHPLLNFIFAIFLGLTGFFYVASMRYDPGYVP

KMNGIAEQKAVIDELLKEWKYDESNFCTTCTIRTPLRSKHCKACARCVAKHDHHCPWVNN

CVGVNNHRHFFLYLICLTIGILTYDWVLYYYFTARSASASDSCNVLSPKFCSLLNADSYT

LVLAGWATLQLTWVTMLMFTQLVQVSRAMTTYENMYGIRSVSSTTAFTSTGTPLDPNHPS

LSANTDAHGGHGHKHGGGMFKRWSRLLGVDPFIETISGRGAATGKNKKKKNPYSRGCISN

CKDFWCEPTPIFGQKENGSAVLGGERVDYTMMYESPTLMNLASGRRARDGYEVVGTEEV

>SoG_03784.T1

MPNQAARTYRSHLQPACLACRRRKSRCHGESPGSCLMCQVHGTECVYPPSPRPSKDATRR

KRQKPEEDAEPVTKASRNTSKGVDVRSSLHSPEGQQNLHIVGPAGANDSQVLSDYLSRRP

GASSTSHNLVPVPASRSRSVFFTKVDRRPVGVNSHYNPAAEKLETIEKLLEPWCTALVDV

YFSNVNPCFPMIEQCSLRRQISQRKDGISPALMACLYAHTLVYWRCSQLLRRQHCPDDRY

IWNLANGALYSELHLQPSISVIQAILLNVGGRPTTSLVGNGALLGAAVSLANSLGLNRNP

LAWDISYSEKMQRMRIWWALLFHDRWSSLTHGTPPRISRDHYDVPAPTTESLYNERPTKK

EKDVAEVFIALMGLTEVLDDFLQRIYRLHSSEAPAPSALETSLNLWVESLSGSVRCAVVR

GAYFTVPGSANLRLSYLTTRLLSQRITLDATKQSAAADHDAVFACYFQSRQTAEEIVKTT

KELGPEQLGDFWPSVSAFVYPATVSFLLRCALEACESAKGIIEDPFFKIARELLEAVRAH

KESHSWDVADLCLTQHSEIMDKILSEAATNNHSAEQETDSWHDFLMSDTSFLDQFFPSFW

DPPNQESIE

>SoG_03804.T1

MARPSLIRADTIDLQDPEAPSAKDHHKHTTTGTTAPHQAETLREIAHETAEEEARSPRVS

WSNGDPLAGGGGIGAGTHSGETNMSTSASGKLQQQQQQQQQQQQEDSLAVAQNGGLSSSD

EGDMDADGDDLDDDMMDRISSSPSIEDGAFYNLSPLTTRVVPLCWPQRVSSLPLHLRSRS

PYLDSCSPQGPVLKALPFSQHLHAQRPPSSCSHHRHRRRKLSEPNTEPYEYATTVPATLG

IAYVQTEQGPKADSFFSAAQGGLDGDCSPKPLLGNLETIYEHDNDESGMPLMIPYTGFDD

DDDDDGDILPLLDSLFLYPNRPTQCLLNPEDIDFEFVYALHTFVATVEGQANATKGDTMV

LLDDSNSYWWLVRVVKDSSIGYLPAEHIETPTERLARLNKHRNVDLSATMLGDQAAKPKP

SFMPLRKRKKTVTFTEPTYVDYSDFDYSSDEEDIAELFGQQAAGASQQKQEQQQQQQQTA

AQSAANVEETADETAKVEPLKTRTATDLKPTEAVTADSGVEEKEEEPETRDSEEIMDASS

GLRQTRNGTIRNTDSFFKDDTVETKKMTLTPNLLRDDNQPRPSTDSTKDLKTRASLDKID

KELISDKEKKKLKEKEKREKDKKPSAIRSFFTRKDKKKASEDDDESLGKPSVDLGSDPRD

SEDPVGDEQASPEKASAPQRQTSKLQKPQPRGEPSPTRKAGGPQKPGTVELSSYLAESRV

NDVANVPPASMRLVDPDTKETQEASLGQVHASQERSASASGVRDDKLRMAKASQPRSASA

ALETRPQKTTKARTRMELDGSDPEETEESSFASPPDRTAPAAQGNPETQARDIRDNSQDA

DASRPQLPGAFPDSYQTATESGETTVTYLGDRDSDSPLPVSPISNPPALMGDTSSQDGVS

PEHSPSPELDPSAKIRTDQEQSKQEQSWDDSKLRAFFDEGDHIRDLLAVVYDKSDVVPAG

NDHPVVGPLFREQNAKLAEITTVRIDAAIFC

>SoG_03809.T1

MGAGYGTTELLKKQLAPSKLIFHFLFWTFHWSIFAYGWWKQAADIRLAGLNTLEYSVWIS

RGAGLVLSVDGMLILLPVCRTIMRVVRPKLRFLPLDENLWMHRQLAYAMLLFTILHTGAH

YVNFFNVEKTQIRPVSALQIHYGQPGGITGHVMLLCMLLMYTTAHARIRQQSFETFWYTH

HLFIPFLLGLYTHTVGCFVRDTPAAFSPFAGELYWKHCIGYLGWRWELVTGGLYLLERLW

REVRARRNTKITRVVRHPYDVVEIQFNKPSFRYKAGQWLFLQVPSVSRYQWHPFTITSCP

FDPYVSVHVRQVGDFTRALGDALGAGAAQAKLYDGVDPMGMYEVALQNGQQMPALRIDGP

YGAPAEDVFENEIAVLIGAGIGVTPWAAILKNIWHLRNSPNPPTRLRRVEFIWICKDTGS

FEWFQTLMSSLEAQSNEAARIPGSDGVEFLKIHTYLTQKLDIDTTQNIVLNSVGAEMDPL

TELQSRTNFGRPDFKRLFSTMREGIIDRTYLNGLEGSMRTTVGVYFCGPSVAAREIKAAA

KAADGQEVHFRFWKEHF

>SoG_03820.T1

MENTPTTSNATNGQNGAGVKSSDYKLKFCTVCASNQNRSMEGHLRLSQANYPVISFGTGS

LVRLPGPTITQPNVYQFNKTSYDTMYKELEAKDARLYRANGVLNMIGRNRDVKWGPERWQ

DWQIGVPRLDHATDKGAEGVEAGLVDVVITCEERCWDAVIDDLLNRGSPLNRPVHVINID

IKDNHEEAAVGGRGIVDLANSLNQAAVEEREAVGAAAFDAGGAASRASFDERVPEILGAW

QERWPKLPATWTLAWF

>SoG_03831.T1

MLFQGYLFALLCAVTSWYGMADAAPSELVKRASLTQVQNFGTNPSGVKMFIYVPANLQAK

PPIVLVLHACQWTAGAFFGTTKYGQLADQHGFIAIYGQTPTDGACWDVSSTQSLSHDGGS

DSTGLANMVRYALQKYNGDASRVYVTGESSGAMMTVSTHGVPNRRGMLTDSRKQQVMAAV

YPDMFEAASEFSGEPAGCFYTGSVRGWNSQCANGQVKKSPSEWAAQVRAMYPGYSGKYPR

MQIYHGDVDNILNINSYNESIKEWSGIFGYSGQATSALNNNPGNRLTKYIYGDRLQGIWG

HGFGHVVPTNETEALQWFGIIGSRSNSPTTTAAGPPRTTTTTSASRPTSTGGSGGGGGGN

GNCAKKWAQCGGQGWSGATCCESGSTCQVSNQWYSQCL

>SoG_03843.T1

MELVQACSQMADRISALEAENSVLQAQAASTGSGNSKATASAASTEDPSVAQLRLELAEA

LRSKGVVERRLLSAAEERDKLREKTKKDTRSLRVLESECTSLATRLKDRDHELREKRKLV

EDVQDEMITLNLQLSLAEKERDRVKADNKDLVDRWMKRMAEEADAMNLANEPHIHGS

>SoG_03897.T1

MTETIPQPVPASASPPTPSNHQFPLPKILQYPSSTPPQLITQGAEGRLYRSTYLLPDLPC

ALKYRPKKPWRHPILDQRLTRHRILSEARILAKCRRDGVRVPTVYALDENAGWLMLEWIE

GAPVRVRINERLGSRKVGIDKDTELAGLMRRMGVAVGQLHKNGIVHGDLTTSNMMLQQAP

PKDPEVDAQPGSSLDGEVVIIDLGLASGSISDEDRAVDLYVLERAFGSTHPRAECLFGEL

LDAYRSTFKQAPVVLKKLEDVRQRGRKRSMVG

>SoG_03906.T1

MRIAIREQLALLVLFAVLIALTIVSVPTWIYVNDHIATNLKDGLALTASLKAARISADLG

LIQTSCFTISSRVLIQDALSRFYQYNSTDWSAATNDIQSALSVGATTGLLQARIYSRNST

GGDPTGLLNVTAPNVAEVLLPYKDQNGQRIYLNDTAMGYPPMLYPNITYENLGRQNKVRH

NTSAFSANAFDGIPILANSPLLLGPLIINETAALLSVTIPIRDNMDTFVLGYMTIISLAN

SLIEVRDSREGLSQSGMVLLVGPVNPWNRFNDSNPPSNDTFQADRAAFAGVDVKFILPPK

TPDDTPSRHSERQYIGGSTALTFPLSDYPAALDSFVDRNSAVNNAAALLDTTNEQGVRVA

VGYARTQTPLVNWTILVEKSRSEAYEPIATLRNILLGTVFGTAGFIMLIVWPCAHLSVMP

IRKLKAATEKSVHPPGYDDSILESDYDDEPPTSGGTSQRSKKGFVANMMRIMKRKGRKPD

SHRDVARRMFKIPGKVDDRKHFITDELTELTRTFNDMSDELVKQYMSLDEKVAERTRELE

ESKKAAEAANESKTLFIANISHELKTPLNGIMGMCAVLMEDNDVTRIKQSLKVVYKSGDL

LLHLLEDLLSFSKNQIGQPLNLELKEFRLGDVRSQILSIFDKQVREGRINFSVNFLGNDD

GDFRSSWNGSADLSRLPALGPTGAGRLKDMCLWGDQHRILQVIINLVSNSLKFTPPEGKV

EVRIRCLGEHETPPEEESRASSMSKHSRTGRTRHRVGPGSTHSSSSGGRGSGAAFNGTGT

ALAINPMDPKATPHVQVRERSPTPPPITAKSYIFEFEVQDTGPGIPEHMQQKVFEPFVQV

VSGLSKQFGGTGLGLSICQQLAGLMGGAITLRSTMGVGTTFTMRIPLKYVQDRASSTASS

SIKSRPPSVDTLDPENQRHNAATPSKVPSAIATQPANIQPRLVGLSQPYFAADHPTQPKS

TEEKMAEIGRAMAKKEGQGKLRVLVADDNSTNIEVVSRMLKLEEVYDVTIAKDGQEAFDL

VKANMEMNQSFDVIFMDVQMPNVDGLQSTRLIREMGYTSPIVALTAFSEESNVRECINSG

MDEFLAKPIRRPALKQVLKKFATIPEEPETASLATRKTSPERSTGSTTSNSQQDSDFEKK

EAAVDVYTPMSDLGQTPPAKEG

>SoG_03913.T1

MVYCGKPSRGCQMCRTRRIKCDETKPKCNQCAKSRRECPGYKDEFDLVFRNETQATERRA

RKANKKAIAKLNKDAPESVQAQSDKPSFAPQSEQVAIKSPRIPLDQQAECHFVANWVLLP

RQGSTRGFMEHLVPLIKTESHCAHFRLAFDACALASLNNRVGSGRDFEKESLGLYTRALS

STFAALKDRATATADTTLAAILLLGLYENITARQMGMLAWGSHIEGAIQLVKARGRSILN

TQTGRWLFIAVRTQMIIHTLSTGTPPIMGVEWWIHDAVQDAHAAQCQKLNIRVGELRAEV

NRLMTTIGRSPEEIELMLDMIRKCQKVDQELVGWANNLPEVFRWQTVTWEDRVPDGDYGK

AEVYPGRVDIYQDLWVVSVINMQRCSRLILASMIVRCAAWVCSPVDYRTTPEYATAARTC

HEIITDVIASVPYQLGYFHRRKDLHSRIALSGFACGDEDTIKGLPGYFLSWPLVCIYGQD

YISDTQRTWIRGRLEFIGNHVGVRYAHILKQINVRIPSMLIRRDGLMASPYPTGHNFEKL

LSARNAKPTPGYAMNPLQQREAMQKAHIEKQKAELLNKVVGSGKLDEWTAKTWLALPTGQ

QAQPPT

>SoG_03920.T1

MEWPIRDIVYTAIVGVVMLMACLEWFLWLAAFLYCLIKVFQKSEHWSINVLCILVGVAFT

LLRCIFLPIMIVTLPLPSQVVRYWPEPMVAFLQWFAFWSFAILLTVPWLFCIYQLVTNQL

GRTKRIKQVLDDVTAPKVVIVMPCYREEPDVLVTAINSVVDCDYPPACIHVFLSFDGDQE

DELYLNTIDKLGVPLTLDSYPNSIDVVYRAARVTVSRFAHGGKRHCQKSTFKLIDKVYQE

YLKRNDNLFLLFIDSDCILDKVCLQNFVYDMELSPGNSRDMLAMTGVITSTTRKHSIITL

LQDMEYIHGQLFERTVESGCGSVTCLPGALTMLRFSAFRRMAKYYFADKAEQCEDLFDFA

KCHLGEDRWLTHLFMIGAKKRYQIQMCTSAFCKTEAVQTYRSLIKQRRRWFLGFITNEVC

MLTDWRLWKRYPILILVRFMQNTIRTTALLFFIMVLALITSSKKVDDLPVGFIAISLGLN

WLLMLYFGGKLRRFKIWLYPLMFIMNPFCNWYYMIYGIFTAGRRTWGGPRADAAVADSHT

TAREAAEQAEEKGDELNIVPETFRPALEARRAGIRTEPQGTAGVVRKRSVVRPPDTIDGK

FSARRRTAAGVYAYSDETGRTGTDGAMGSSRKHSEVTEPPRAAWEGDARYSLESLVGEPN

WTRMEDYMGEEDRRKYAMAQRAQQSRGHHRTQAVHLVGPPSTPRRPEASGQHGKRKNVDD

QYGSSMV

>SoG_03924.T1

MDPVPPDSKRPRIAAPGASLPHLQTSSQLPPPHPHQQHSQHLHHPPAPLPPPPSASYPTY

PSRAVEPNSAQARHDSDRRHHDQEPYPPMQDHYRHQPPPSPAHGPYQQHQPYAPPRDVVK

REAPYEDHSRRSSSTGHPLDGAPAVQPVPAHPHHTQLPPYADGQTRHMNYDHGPPSVPPT

PGGYRHPQAYPPPTPMPQYEPPGYPTTETYYNVYSSASKKKNTRASQACDNCRSLKAKCD

ETKPCKTCKDKGVECRYRDPIPKATDKAQADILDGLARLESILQQQGKTLQSQGTELGEM

SRRLQKLESACATRVPPIPIPVPVPVKPEPIIEEDHKVHESPARTNSNTTSERYSLSETS

RDVAPSTETAPSEATVHAVQVPEPYAGRVDPARINEAEMDDAAQMESSAEDEVEKDPGPP

VPPGEPAIPINHTTLAGLLLDWAAIKELTSRHLLGQGVRFVTEFPISVEQNRGALILYGR

GEDSPYQMIPRETPDHGTVDLPDDASDMTSPSPAPDYGQLGGLSPPDQIDYRGGVLTSDG

NPDFSEGQVWRYVESFRENILNMHPIIQNKVLDQWTRQFLDSLVPTNPRSQWPATSKAAF

AVENQHEATGMKRKRSLEPEVIDLEKTRHGRPNRSVHSALMLTIIALGKVCQYRKCIPDA

VHNVRDPVPHGSPRNPVTASPAQGSPMGVNSYGPSPAPGDGLARRPSTQQNAKGLGLKKN

YDAIPGLEYFAAATDILGNQLGAYNNMKNVYANIFASLYYGQLERPMQSYAHINSASNKL

LVILRPSLDKLRRLMRANTMPTETKYNQLCLAFWTCLQLESDIIAELPLPPSGLLSYEDD

MPRPDMSLLDHPPRVADSYNAQLYLRKHLNSIHRMFYAPTDPEKANPDEPFRNVELVAES

VSSMNWVSPRFRFEETDPPANDILAARLRAKYWGAQMITYRPFIRQILNFHHSMKVNPES

PNLPQGTEFRKDVDAPIIDPKTRSSNEINPTVIELAKKAIKALIESTRAFHNLDEVGNKR

PIITNIFGTAHAQWGNLLVLSACWRDPLLTKYIDPQLLQYLFRRTIRFLQQSATATSSLT

IDLNILKGLYQDFWPEDHPTGSSFSSHSSVATPMGPPHLPH

>SoG_03927.T1

MAAVLSSQGQGKDQGLDAKGKSLSSSESSSTRDDGENQRSGHYGSSDDHIFADKATADYW

RLKYEKAGYENRHRFDPELTWTAEEERKIVRKVDKRIMLWAWIMFCALDLHRRNINRAIS

DNMLGELGMNTNDFNYGQTIFLVSFLSAELPSGLISKKLGADVWIPFIMCGWSIVAGSQA

FLSNRAGFFAIKALLGLLMGGFIPDIVLWLTYFYKSNELPIRLAWFWTALSTVNIVGSLV

AAGVLQMRGIAGWGGWRWLFLLEGIVTLIIGILSWGLMPPGPTQTKNWFRGKNGWFTDRE

EFIMVNRLLRDDPSKGDMNNRQAVGLSRLWSALKDWEQWPLYLVGLTTYIPPAPPGTYLS

FILRQLGYSVFESNLLAIPSQFLFAINLLIVSWVSERIKERALISSLANAWIFPWLVALV

ALPASANPWIRYAVLTGLLSYPYCHAILVGWNARNSNSVRTRAVSAALYNMTVQSGNIVA

SNIYRDDDKPLYRRGNKILLGICCFNVVLMFAVKLFYIWRNKIRDRQWDAMTPEEKEHYV

LNTTDEGMKRLDFRFVH

>SoG_03929.T1

MASKGNSLRDRQIASLKKILNLNETVETSETEEAHANEFHSAVAPILDADGNPIWKVLVF

DDMGRDVISSVLRVSDLRSMGVTMHMHIASSRHPIPDVPVIYLLEPTAHNLQSITTDLQK

DLYSPAYINLLSSLPRVLLEDFATQTATAGTSDKIAQLYDQYLNFIVSEPDLFSLGMRKD

RTYWALNSASTSDSDLDQMIDRIVSGLFSVIVTMGIIPIIRCPKDAAAKLVAERLDRKLR

DHILNSKDNLFSSQRTGSSGTPSSRPVLILLDRNVDLTPMLSHSWTYQSLVHDILDVKLN

RITIETPNDESNPAKGTSKKSYDLTATDFFWAKNSGAPFPQVAEEIDSELLKWKEDTQAL

TRKTGVQDIESLMQGETGVNAQHLKTAITMLPEMRERKVTLDMHMNILAALLTGIKNRQL

DNFFQLEETVMKLAKPQILEIIRDADKGSEPEDKLRLFIIWFLNTEQEVTKSDYQGFEKA

LADAGADVSSLPYIRQVRATTKMTQLATINNASQQAASSGDLFGRFSAISSRLTDRLKDS

GVPAGLSSNFESLISGVKNFLPADRDLTVTKIVESIMDPSAAASSAIAKTENYLYYDPRS

ANARGTMPPPSAARAGAGSTPGGLPGSQGAGQSASFGQRRQGFSEAIVFTVGGGSMDEYG

NLQEWVARTGGDRGKKRIVYGSTELVSAGEFVRDELARLGKEVSG

>SoG_03943.T1

MAPQKHVEYEFGGPPPTSIGATGIVFGLPVLMNVLYLFCNDVSGCPAPALLEPRSLTWEK

LKPQIPWPEEGLWGFASWSVTGWVLAYYAVSLVLFRVLPAREVLGTKLLESGKPLRYRFN

AFSSTLVQLVPCAIGTYLHGSNFILWTYITANYLQILTASLILSFIISIGVYINSFSIKP

GNPELREVARGGRTGVIIYDYFIGRELNPRITLPFVGEVDIKTFLGMRPGLTGWMLLNLA

FVAKQHRMYGYVSDSILAVTAVQGYYVLEGQYAEDGIVGMMDFKSDGLGFMLTFGDIVWV

PFLYSTMCRYLSTYPVHLGPYNLAAVGVTFAVGLYIFRSSNSQKILFRKNPDDPAFNGMS

YIQTKRGTRLLTSGWWGVARHVNYLGDWLQSLPFCLPTGFAGYTILPAGAGAAAAAAAAA

AAKSATVATASASGGVATMLDGRAVIAGGDAAGWGMLFTYFYSFWFGFLLIHREGRDDRV

CAEKYGDDWAEYKRRVKWKIIPGVY

>SoG_03955.T1

MGRGLRNKAKKLKERVQHQFKDATSAERQKDSQSSSSSSSSSSQDHQEAPSAAEPAARVP

KEPQAAMAALEIETSDATSQSQSEGGRKRQNLHDQPDDEGGEWEVVGRPKKKPKKAPRPG

KNYPALEFSSSRSRLNSRLNLTNLRDLVTYIHADGPAPQWISVMHRPHFRKTVAIMVPGL

EEAMFKHNANFFTYNDRVNLIEERIATSPDDYYPRPLVKDKLPAALAPFADMFPHLWPVR

TPGDDKHAKMHSPITTFLTVPTAKDKVQKKGVKPATEPHGWQNTRTRITEFIATREELEE

NGYLIHPALIPEGERKDDFKPPEGWVVTRVKSLDEGVVPESEIEQGSITAGRDVLALDCE

MCMTGEDEFSLTRISIVNWAGEVVLDELVKPDKPIIDYVTQFSGITEEMLAPVTTTLADI

QEKLLDILHPRTILIGHSLESDTKAIRLAHPFIVDTSIAYPHPRGPPLKSSLKWLAQKYL

SREIQKGGANGHDSIEDAKTCLDLMKQKCEKGKLWGASDSGENLFRRLARAGTTYKAQGG

DEALGGLESGKSSAAVDWGDPAKGPGAGATHQLGCKNDDEVTTNIIRAVKGDPAGEEIRG

GGVDFVWGRMRELEALQGWWNKNRVENNNKNASKPPEEQALEAAAGALAAVGEVSPLEQA

LTSLSERISRIHAALPPCTAFLLYSGSGDPREMARLQQVHARWRKEYNTPGMKWDNLSVK

WTDTEEQALKKAVQTARSGIGFIGVK

>SoG_03957.T1

MSADDNSSYRPKSPDLSSFFAGPTEPVQHTTFLPAQSASPAPPQNNNNSHHHQPHQHQHQ

QNYSHHNPHYNDSQPVHNPYLQHQPPPLNQHPHYPTNPHPYDYPPTTQPVGYGYDSSFAG

APYAQPTSPRGIKTEELHPYLPPRQGPLPTNQAGAFLNDPVPHDSYPQQHQSAFNQPYTD

PTFPHFAGPSSYNPPYHPGAGLDQDLEPEMPQDQVSAGQAAAAPRPPPINLPDPSPVKTK

FPTARIKRIMQADEEVGKVAQQTPIAVGKALEMFMVQLVLTSADVAREKGSKRVTAQMLK

QVVETDEQWDFLRDIVSKVETHDKGSKGGNVKAESDSDDDEPKKKKGGRKRKTG

>SoG_03975.T1

MDSSQDEKSRPLGGAEAGTPDYGDCPARACTQDEAAAPLERIPTQEELIEEDEDEDEDDE

NEDEAAERSSSDDNGLMGQADRQVLRRIATDLSRRRSSVMELEHPGLTRTLTAIDENDPT

LDPDHQDFSLSKWVKAFIKRVQDKGISRTGTGVAFRNLDVFGSGSALQIQDTVGSFAAAP

LRLGEIFSFGKKEPKHILHGFDGLLRQGEMLLVLGRPGSGCSTLLKILTGELYGLKVGDK

SSVYYKGISLKQMRKEFRGEAVYNQEVDKHFPHLTVGETLEFAAAVRCHDRLLENGNRME

AARYISKVVMALFGLSHTNNTKVRSHTNSLFASPSLLLSLSLCMYLTWRIGNQVGNDFVR

GVSGGERKRVSIAEMFLSAAPIAAWDNSTRGLDSATALKFVQSARSASDIGEVTNIVAIY

QASQAIYDLFEKVTVLYEGRQIYFGPSKAAKRYFEDLGWYCPPRQTTGDFLTSVTNPTER

QAREGFESRVPRTPEEFEKRWRESREYRDLQAELNDYDREYLGENQESTLREFREQKTAR

QARHVRPESPYTVTVTTQVRLLTRRSWQRIWNDKSATLTHIGVECIIALIIGSIFYGTPD

ATAGFYAKGSVLFMAILINALTAISEIVSLYAQRPIVEKHRSYAFYHPAAEAAAGIFLDI

PMKFCIATVFNIVIYFMSGLRREAAQFFFFFLITYIMTFVMSAVFRTIAALTKTVSQAMS

LSGVMVLALVIYTGFAITVPDMHPWFSWIRWINPIFYGFEALVANEFHGQEFTCSSFVPP

YPNLSGNTFVCGTSGAVPGERTVSGDAFIAANYEYYYSHVWRNFGILMAFLVAFMTMYFV

AVELNSSTTSTAEALVFQRGHVPAHLQKNGKDIKADEESASERKMQESSHDSGDVKALPP

QKDIFTWRDVVYDIEIKGKPRRLLDHVDGWVKPGTLTALMGVSGAGKTTLLDVLAQRTTM

GVITGDMLVNGQPRDPSFQRKTGYVQQQDLHLQTSTVRESLRFSAMLRQPKTVSKKEKYE

FVEDVIKMLNMEDFANAIVGVPGEGLNVEQRKLLTIGVELAAKPELLLFLDEPTSGLDSQ

SSWAICSFLRKLADSGQAVLCTVHQPSAILFQSFDRLLFLAKGGKTVYFGDIGENSRTLL

DYFESSGARRCDDQENPAEYMLEIVNNGTNNEGKEWHNVWRESPKWQANRDEIDRLHREK

AAEASSAPPASKHGGSHSEFAMPFSSQLWAVTYRVFQQYWRTPSYIFAKFGLGIAAGLFI

GFTFYDANTSLAGMQNVIFSVFMVTTIFSTLVQQIQPLFVTQRELYEVRERPSKAYSWIA

FIISNIIVEIPYQILTGILIFACFYYPVVGIQSSERQGLVLLYVTQLFIYASSFAQMTIA

ALPDAQTASAIVTLLVMMSTIFNGVLQTPDALPGFWIFMYRVSPFTYWVGGIVATELHDK

QVVCAERELAIFDPPSGMTCGEYLAPFLQQAPGALQDPSATSNCEYCSISVADQYLAGPR

IFWSERWRNYGLVWAYIAFNIFIAVVTYYLFRVKKWNAKAPSFRIPFLSKKVPK

>SoG_03981.T1

MKKWSSFAVSSPTEIHQSASHPMLHDPQASLSTTNTRAIFLLAASKPPYGVYCNPFYKHA

GHGSVFTVDGSRSGALAENVQNFEYQPDTGIHGMVFEPRDEEYLYSADLKANKIWVHRRP

DLARAEVELVGSVDCPDERDHPRWVAIHPTGNYLYALMEKGNVLREYLVDPSTRLPVYTH

RSFPLIPPGIPDRWTQYRADVCTLTSSGRYLFASSRANSFDLTGYVSAFRLADNGSIERQ

ICLNPTPTSGGHSNAVSPSPWSDEWVAITDDQEGWLEIYRWQDEFLARVARCRVQEPGFG

MNAIWYD

>SoG_03988.T1

MDPYDPEGLPGSQDGFGDMSLFDASAAQNPASPIEGEGRKHKRDKKHKHKKSKKEKHAGD

PAPSAPADDAEELSIADRTLDVDDEARAIAEQLGSMASPTQAKRKRASADGSGGKRKKKR

REAAGADAVEEEAAEDDRVPASSGTRAEPEADIAATMDIDDATAQSHGNELDVASLALEA

FNEHLNGQANGVVADYEVPTAVMEDPADAPVAEASVGEAAMDASPKQHRSAKPKKAKPTF

FEQPTAELAPEDDEVGKDAMWDMPSPSAAPPKPRRSKPANRKSKGRQKLAHSMHGGSDGE

YGEAAPRQRRAKTEGFVQGRFSDLELDGIRQAVEQYRDDSGKTQHEVNEMIHAPGGTTAG

EEHRQLWDMIFEKCPDRYRQKIINVTRKNFHNFVARGTWTPEQEAELRYLIDLHGTKWSQ

IAGIINRHPEDIRDRYRNYIVCGDRQRKDAWTEEEESRLTQYFIEAAEAIDDLRRSQPDR

ELLKKSYEELIDWQNISERMDRTRSRLQCITKWKAMNLKTNGKDKVQALQPNSQITFKLE

KARRQLHDMPEPERYRLIMAIQATAAGTESKIPWQKLIDKQFRTMYKRPTQYLLWTRLKQ

TVPGHESKTVRDIAQELIQRYEQEGSLPSVDDDSMYDVREERELLSRLSSTGGAMAHPAS

QNDQNQLSAEFVMNSDDEGHAPAAAEPTLGSLMQIDPALIAAAAVEPLEAADALEAAQAL

EAAAAATETPQKKSKKGRKGKKAAVEASQEPMEDIPQEQPMDPAMLPEVTPADEEIDESQ

PRGKKKKSGKSKSSEGKDAALLETDLPPAPASDSVMDDMEDLPARVSPSLA

>SoG_03999.T1

MSQTFSTPPPSLPDTIILSSPSPHVLLVTLNRPKQLNALRREMHWQLDQVWNWYDGEPGL

RCAVLTGKGRAFCAGADLKEWNEKNHGEVQSGAAEKWTDNGFGGMSNRRGKKPILVAVNG

LCLGGGFEMMINADMVIASDKAKFGLPEVKRGVVAIAGALPRLIKVVGRQRASEMAMLGR

MYGAEEVRSWGAVNRVVSDDKVVDEALSWAREVVENSPDSVIVTREGLMGGWDGEDPRSS

THRVERGVYGAVVEGGENMTEGVVSFVEKRKAVWKNSKL

>SoG_04032.T1

MLMCAGFFAKTATHEVQHTRLSADFAKSEALQSWAIFLAEDSIPMGLKMHEAASKWGETS

SKTEMSFNLAFGTDKPFFEWLSSTPKATKKFSGYMKAVTATYGLALDHLIDGYDWSGLQN

GIVVDVGGSSGHASIALARKYGNLKFFVQDLPRVIQGVDTTSIDQGTRGRIKFEEHDFFQ

PQTVKDADLYLLRQILHDWPNVEATSILQNICSAMRPGARILIMDSALPTPGSISRVQEA

QLRVRDMSMMQTFNASERDLEDWKALMKNADPRLELESVSIPTGSSLALLLARFT

>SoG_04037.T1

MATVVPPPSKRQKREQLERTQIQQDVTAASGPAGSFKARFLDGDGQQMADVIEVPLADAS

EKNLSLLLNTLMMREKEDWLPYRFRIHIPETDIIVDQYPTDLLALLRSHGIENPYETTIT

LSAEPQAVFKVQAVTRMAHRVPGHGEAILAVQFSPANSARLATGSGDKTARILDADTGTP

KYTLSGHSHWVLCVSWSPDGERLATGSMDKSVRLWDPNTGKAVGSPLTGHAKWVTNIAWE

PYHLWKDGTPRLASASKDCTVRTWVVNSGRTEHVLSGHRSSVSCVKWGGTGLIYSASHDK

TVRVWDAVKGTLVHTLSAHAHWVNHLALSTDFALRTSFYDHTPVPQGEDARRAKAKERFE

KAAKVQGKLAERLVSASDDFTMYLWDPSQGTKPVARMLGHQKQVNHVTFSPDGSLIASTG

WDNHTKLWSARDGKFINTLRGHVGPVYQCAFSADSRLLVTASKDTTLKVWSMASHKLAAD

LPGHQDEVFAVDWSPDGKRVGSGGKDKAVRLWCN

>SoG_04042.T1

MAAGEKTGLAEPVEVNQIPYWRLVFDQAGVTDAVRNHTYPGSGTEEDPYVVDWLPDDPRN

PFTWTSFQKWFITMTVAMATLAVALVSSAYTGGIKEIIMEFQMSQEVATLGVSLFVLGFA

IGPLLWAPLSEVFGRQLLFFFTYGGLTAFNAGAAGANNTATLLIMRFFAGAIGSSPLTNA

GGVIADMFPAAQRGLAMSLFAAAPFLGPVLGPIIGGFLGQGPGWRWVLGFLAAFSGFFWI

VGTLLVPETYSPVLLRKRAAKLSKLTGKVYKSQMDITRGPVSLLNIMKVTLSRPWILLFT

EPIVLLLSIYMAIIYGTLYMLFGAFPIVYQQQRGWSSGIGGLAFLGIMVGMIFAVIYSIF

DNKRYIRTEQENDGMAPPEARLPPAIVGCVAIPIGLFWFAWTNQTSIHWAVSIAAGVPFG

FGMVLVFLSVMNYLIDAYTIFAASVLAASSVLRSLFGAAFPLFTSHMYANLGVHWASSIP

AFLALACVPFPFLFYKYGDRIRQRCRFAAESLAFMRKMKDAERARAAQEALAEEEEGEPT

LTGSISEHAPDQPQRPGAEAEVEAPVYEEIRTPTHQPNTRLSRTSSVASHASHASRASRR

FTMYDGNPYNIDRVNTNDEQFTLARTRSPNSQHHSPQ

>SoG_04054.T1

MRFIVALTSIAAVVSAARVGTPTRKDSPSRREPVASLCTKYAYYANNDYEVLNNLWGEEA

ATSGSQCTYYYGRAGDGVSISSNWTWQGAPNNVKSYIYANRLFKRPLVKDIKGLPTMATW

SYNTSDIRANVAYDIFTHKDADHPNYNGDFELMIWLNRYGGIWPITDSPTGKPVETVRIA

GYSWDLYTGYNGDMRVYSFLAADGPLHTFSADVLDFFHFLSKHYEYPASTQYMLSESTDL

FLPFPLLAFSTPCLSLFLFLSRLYIWRVDVAFYNFGTEAFTGGPANFDVPQFQADVHV

>SoG_04057.T1

MASAMAMRRLAAGPSSVRTFLPSLYRPFALEHRRWTVDCAFRHRWRTYADMGGIVFETGK

DANQGVLRDPLSCPYHRADWVCEGQGGLSLREQQVSRHCTSIRHALSAFLLQLVDHEYDA

LVVGAGGAGLRAAFGLAEAGFNTACISKLFPTRSHTVAAQGGINAALGNMHEDDWRWHMY

DTVKGSDWLGDQDAIHYMTREAPASIVELENYGCPFSRTEDGKIYQRAFGGQSQKYGKGG

QAYRCCAAADRTGHALLHTLYGQSLRHNTNYFIEYFALDLIMQDGECRGVLAYNQEDGTL

HRFLANHTVLATGGYGRAYFSCTSAHTCTGDGMAMVARAGLPNQDLEFVQFHPTGIYGAG

CLITEGARGEGGYLLNSEGERFMERYAPTAKDLASRDVVSRSMTMEIRDGRGVGAEKDHI

FLQLSHLPADVLAERLPGISETAGIFAGVDVTKQPIPVLPTVHYNMGGIPTRYTGEVLTV

DENGNDKVVPGLFACGEAACVSVHGANRLGANSLLDLVVFGRAVSHTIRDNFNPGDKLKP

VAADAGAEHIEVLDKVRTSDGPKSTAEIRLAMQKAMQTEVSVFRTQESLDEGVRKMTEID

AMFPEVGIKDRSMIWNSDLVETLELRNLLTCATQTAVSAANRKESRGAHAREDYPDRDDE

NWMKHTLSFQKQPHGKVDLSYRKVIGTTLDENECKPVPPFKRVY

>SoG_04061.T1

MDALRSVIQPITHNLPAPVRDLGVSLLGETCYTSLLLDVDLANQECLKLAVSKGLGIGII

AASSVVKVPQILKITRSQSAEGLSFLSYLLETIAYTIGLAYNVRMGFPFSTFGETVFIVV

QNVVISFMILKYSNRVGAAFIFLGTLLAVVPALFISDLVSMDILSYLQAGAGALGVAAKV

PQIVTVFSEGTTGQLSAFAVFNYLAGSLSRIFTTLQEVDDKLILYGFVSGFALNLVLALQ

MVYYWNAPSAKAKGKRREAPIKPVVSSASTTATPKKGATTRRRG

>SoG_04067.T1

MPSRSDILAPFASAFGRSHSPSPSPSQRSRTATPRSASRPEAAADSNQLTSEASSASSIM

HQDNVDSPAVSSAPTTPPQVPVIPDHRRLLGKMNDSAVDSNSSGRGTSTNSGRSSPRSSM

DIKRSSLDIMAASRTSIDSGRTGTSADWAVHPNEINTIAQLDRFPIGTEVNFRARIETQR

QLSKALDFLLLRDQTHSVQGVLSRENMDMVKWVQKLHPESLVEVGGIIKEPPQPVRSATQ

SKIEIHIQTIFLVNSASNAPFSNYRPPETLRNRMTSRILDLRHPSNQALFRIRSLVTRKF

RETLEDRGFVEINTPKLQPAATESGAAVFKVNYFGRRAFLAQSPQLAKQMAVSADFGRVF

EVGPVFRAENSNTHRHLTEYTGLDIEMAIEHDYHEVIEVIDVFLKSVFESVYAMPEVDVL

QKRWPSKEFKWLEETLILNFADGIQMLRDDGRDVEMEDLSTPDEIRLGQLVREQYGTDYY

ILDKFPASARPFYTHKDTEDPFFTRSFDIFIRGQEICSGGQRIHDLNTLRSNMAAAGMTE

DGMEDYLSAFELGAPPHAGAGLGLERIVAWMLELGDVRYASLFHRDPKSLPERLPGLPHP

EADTTKPHKPGQLPPLEKLIANYGDATNTSWLDERFNIWQHPTGAIVGYVKQDKFAMITG

DPLCDRSQYKDIARAFINFVTHELKLTPVWMLVSYDVQRFLAQDLAWRTLSCTEEQRVDT

DKHKSQTGGPKARRVEREGVKIHEVKPDEDFIKRAQEAVDTWKSSRKGKQMHLTEVRPWI

DLEHRRFFAAEKDGKVQAMVVLHKLAPRYGWQVKWALDFPGSVNGAIEVLIDFALSSVTG

KVTFGVGVSEKLTAGEHLHGIRARFLATTYSSIVDTLGLRKKADFRSKFGALGESVYICY

PKHGVGLRDLQQIIKFFQD

>SoG_04075.T1

MSAMDAQARPAPPKAAGLNNILNDDSPPNSNGNPPSHLRDSGFYSTAEASSKHTSAASFS

ANGLSPTGSGYHSAADKTPSPVTSNIPQPLVSPSASGMSVASMVSPTTPGSADPRRQERP

TSLESTGGTGSTLGQDLNPMMRRESVDSRINQGFSDMRLGNSPYASNNQSTTSIQNTLHS

QRNPRPGLENLSVHRISNGYQPSADRNPEVKTVRTAPAITGPATGTIARAAEPTKGQAWA

FPEEEIQRVSHQYDDSRRSSITESIASSQFTLESRLPPGQRRLDEAPEYGRMSGGGDFPP

VHHHTMQHKQLSDLQAEEAGGAGGNQPYSRTPELRVSHKLAERKRRTEMKELFDQLRDLM

PQERGSKASKWEILTKAIAEHQRQSDAIRALQQHLHNATTENDMLRRELAAMQGSRMGEM

GAPTPHASQQGPSGPSQPSSQPPYASDQYAGSRTELPPLRSISNGIPNGPDSMTGVQYEA

PRTNGYRGPEPRY

>SoG_04080.T1

MAEIRRKLVIVGDGACGKTCLLINICDARLGCIQSFNCCVTYLIFVPSVLLPSPSIFADS

SSRSVFSKGTFPEVYVPTVFENYVADVEVDGKHVELALWDTAGQEDYDRLRPLSYPDSHV

ILICFAVDSPDSLDNVQEKWISEVLHFCQGLPIILVGCKKDLRYDQKTIEELRKTSQKPV

SPEEGEEIRKKIGAYKYLECSAKTNEGVREVFEHATRAALLSRNGRSGKTKKKCMVL

>SoG_04123.T1

MGPNQHGKRPLSRQGCHQDPPVKRRASQACLSCRNRKVRCDVVNGGMPCTNCRLDNVRCE

VKETQRGRRPCSGRPSKKDTVTRPAKDLERNEIPQQDAQDVIQAQMTLEDEDEQEEGDAE

GDSEADEGSLIYCQPSRTPTTQQHSHSENPELSNCDEEQLPEEANGHAARLQSAPISPFL

VMSTPPRPGSQQHQIPQQPQRNSRGHQSRTPLPHGYHLLDRSTQLFPEAIRKPGLPLYIQ

PIPKHLDHQDVEYMESKHCLEIPDDELRDELLRVYVSIVYPLLPAVEIDEFIEAVMANDG

RNPISLLLFQSIMFVSVAFVDTEYLLARGYSSHKAARKDFFNRVRILYSLNYEKDRVALT

QSLLAMTYWYDAPDDDKDTWYWMGVALTTAQVAGFHRDPSSAVVAPRLREARLQRRIWWC

CFMRDLFLALGLRRPPRIRDTDYTVTMLCEADFNFGTHSATFGSLFRSSKFPCPDQTMSR

ELAAMSVELARLCVTAGHMLQTQYTSAGAYYGGSEYLGSHKPKRTSEQIATLAQCAAELS

DWIQHQNARAPYVPGDEQTSSDKGDERGKIIRFHRIQLHMHYLAALGALRRPSVFCQGPT

QRSHSEQTSLSAEVVLDPAIEMVKLAFDLQHNDQLRYLTTLAVPAYLAVSLVHLLDSRQG

EEETRNLSLGRLYQSVYVLQKLQSIYSSADYAICFLRSILANTKLRIPLLWMDDAATSRR

QDAPATKISMEMAPDEVAAACMYPSPSASAQLNKPVGDSPTGSSPPPVGVDQWELPWEQD

LIGGIAPFHELVAGSPFLGNWCDSDGMMPTAVDMGGTAPGFSGDISSRHC

>SoG_04126.T1

MSLRFDGQVAVVTGAGAGLGRAYALALAAAGAQVVVNDIGASLQGQGTDSKVADLVVDAI

VKAGGTAVANYDSVQHGERIIDTALKTYGRIDVLINNAGILRDVSFKNMTDEQWDIIQDI

HVRGAFVTTRAAWGHFRKQKYGRVILTSSAAGLYGNFGQCNYSAAKAAMIGFGETLAKEG

AKYNIHTNIIAPIVASRMTATVLPEDQLRYFAADWVVPVVSVLVHSSLKSENGSIFEIGG

GHVSKLRWERASGALLKADSSLTPGAVLANWAQVNDFSNPQYPNTTADLVGLLKASQSLK

SSPLAEDIRFDGRVAVVTGGGNGLGRAYSIELAKRGAKVVVNDLLDPNSVVQEIRVQGGI

AVAHKGSNEDGDALIQTAISNFGRVDILINNAGILRDKSFQNMTDDMWDRVQAVHLRATY

KCSKAAYPYMVKQKYGRIVNTTSTSGTYGNFGQANYAAAKTGIVGFSRALAIEGQKYNIQ

VNCISPSAGTDLTKGVLPEEVVKSRKPEYVAAMVLLLSSDQCPRDARGWIFESGCGWQAR

TRFQRSAGVDFPLTEPLTPETVLKFWEGIVAFTPGKTTNPEVASETRQRIMANIQKSKSP

PSASSYLDAIEKARACPPRLSSFSFSEKDTILYNISLGCTSSQLPLVYESDPEFQVVPSF

GVIPGTTAKRSFDLAELVPSFSYKKLLHGEQYLEVRKYPIPVAGTFQSECRLLDIVDKGK

ASVAVIATTTRDASTGEDVFYNETSLFLRDAGGFGGPAARRDAGDATAAYSIPKRDPDFV

VEEATSKDQAALYRLNGDRNPLHIDPQVSKSVGFQVPILHGLCTFGIATKHLVSTFGRIR

NIKVRFAGTVLPGQTLSTEMWREGNTVRFQVRIKETGKLCISSSGARLWPEPTGRL

>SoG_04145.T1

MADQHEVDLDSIIDRLLEVRGSRPGKQVQLLETEIRYLCTKAREIFISQPILLELEAPIK

ICGDIHGQYYDLLRLFEYGGFPPEANYLFLGDYVDRGKQSLETICLLLAYKIKYPENFFI

LRGNHECASINRIYGFYDECKRRYNIKLWKTFTDCFNCLPIAAIIDEKIFTMHGGLSPDL

NSMEQIRRVMRPTDIPDCGLLCDLLWSDPDKDITGWSENDRGVSFTFGPDVVSRFLQKHD

MDLICRAHQVVEDGYEFFSKRQLVTLFSAPNYCGEFDNAGAMMSVDESLLCSFQILKPAE

KKQKFGRR

>SoG_04184.T1

MPIDPEELERRAKAARAAVAKPRFIPRKERERLAAEKAAKEEAEKKRKLEDQERAHRESK

KQWLEEAEKDERRKRDLERRGANDHKSRNGGRDRDTQREEPGDWKDVREARNGNASTSNG

RKRTAQDIENENLLTRYLGPEVNKHSKFSAAKKRQRTAANKFNFDWDPSEDTTRDDDYGT

AVKPKIPKPSGSAVSVGNRFDEAAEQRALIKASAIRERDRETGEERAKGIMDDFYRSREK

EEQRLQKSSMGRKWATKSLADMTERDWRIFKEDFAISTKGGSLPNPMRSWQESGLSRTLL

EIVDKVGYSEPSAIQRAAIPIALGARDVIGVAHTGSGKTAAFILPMLDYISTLPLLTEVN

RDDGPYALILAPTRELAQQIESEALRFAQPLGFNCVSIVGGHSLEEQSHAMRNGAEIVVA

TPGRLVDCLERRLLVLSQCCYLVMDEADRMIDLGFEESVNKILDALPVSNEKPDTDDAEN

AQLMKSFSAGMNRYRQTMMYTATMPPSLEKIAKKYLRRPAIVTIGNVGEAVDTVEQRVEF

AGGEDRRKKRLQEILSSGEFSPPMIVFVNIKRNCDAIAHEVRRMGWSTVALHGSKTQEQR

EAALQSVRDGHTQVLVATDLAGRGIDVADISLVVNFNMATSIENYTHRIGRTGRAGKSGV

AITFLDGTEDPGILYDLKQMLSKSSLSKVPEELKKRAAAQPKFPKAGR

>SoG_04192.T1

MSPSAAEPVTNGSVKSLKPNIGVFTNPKHDLWVSETTPSAESVKTGKDLKPGEVTVAIRS

TGICGSDVHFWHAGCIGPMIVEDDHVLGHESAGEVIAVHPSVKHLKVGDRVAVEPNVPCH

ACEPCLTGRYNGCDNVEFSSTPPIWGFLRRYVNHPAVWCHKIGDMSYENGAMLEPLSVAL

AGMQRANINLGDPVLICGAGPIGLITLLCCAAAGACPLVITDISETRLAFAKELCPRVIT

HKVERDTAEDAAKKIVKSFGGIEPAITMECTGVESSIAAAIWATKFGGKVFVIGVGKNEI

NIPFMRASVREVDIQLQYRYANTWPRAIRLVESGVINLSKLVTHRFNLEEATKAFETSAD

PKSGAIKVMIQSMD

>SoG_04198.T1

MSDEKPIPGYDPKSSPDYIQFTCLPPGGALNRWSTVVTREHDFPGAQAMLYAAGIPDKET

MKNAPQVGISSVWWEGNPCNTHHTTVLEFGSIIKKSVEKSGMIGWQYSPIGVSDAITMGS

EGMRFSLQSRELIADSIETVTCAQRHDANISIPGCDKNMPGVIMAAARHNRPFIMVYGGT

IGKGHSSLLEKSINVATCYEAQGAFEYGRLQAKTDPGAPGRSSVDVLEDIERHACPGAGA

CGGMFTANTMSTAIESMGLTLPGSSSYPALSPEKRRECEKVGQVIKTTMEKDIRPRDLLT

RAAFENALVITMILGGSTNGVLHFLAMANTAGVELTLDDVARASDRTPFLADLAPSGKYL

MEDLYKVGGTPSVLKLLVAKGLINGDIMTVTGRTLAENIADWPSLDPGQQIIRPLEDPIK

TSGHIRILRGNFAPGGAVAKITGKEGLSFTGKARTFYKEAELDQALRKGQIKRTDGNLVL

IVRYEGPKGGPGMPEQLRASAAIMGAGLNNVALVTDGRYSGASHGFIVGHVVPEAAVGGP

IALVNDGDSITISAETNTITVDISDEEMERRRKDWVAPEMKIKRGVLAKYAKLVGDASHG

AVTDRW

>SoG_04199.T1

MSPIRPSPFPGLYAPAADVVDRHEYGVMKNRKPASTGGGRAWSEEEESYLLQTRMQKMPY

KHIAAHLRKTELACRLHYHQLSHGSGRRKRTASCSSGGSSEHSPVMSMAPMDRSRETQAR

SLSPPGTSSGYMHVPGNMQLPSIMNTNTPPRLPAILPKPMAMAPMAPSHIPNGQMMHAPP

PSEMQHIGLQPSAMDRTQSLPPSAPALRLDCNASPAPNHTANHVDLARLHSVYNAHRHTF

WSVVAKDYGHGASPATLEQAWKTGMCCSQSRAGSPITPGASPSSDEKNAYSRIQDKTRIA

SILTLDVEPRTA

>SoG_04209.T1

MREVISINVGQAGCQIANSCWEPDGYLTEERKAQDPDQGFSTFFSETGQGKYVPRTIYCD

LEPNVVDEVRTGAYRNLFHPEFMITGKEDASNNYARGHYTIGKELIEGVLDKIRRVADNC

VGLQGFLVFHSFGGGTGSGFGALLMERLSVDYGKKSKLEFCVYPSPQTATSVVEPYNSIL

TTHTTLEHSDCSFMVDNEAIYDICRRNLGLERPNFENLNRLIAQVVSSITASLRFDGSLN

VDLNEFQTNLVPYPRIHFPLVAYAPVISAAKAAHEANSVMEMTMACFEPTNQMVKCDPRL

GKYMATCLLYRGDVVPKDTHAAVATLKTKRTIQFVDWCPTGFKLGICYQAPENVPNGDLA

KVDRAVCMLSNTTAIAEAWSSLSMKFDLMHSKRAFVHWYVGEGMEEGEFSEAREDLAALE

RDYEEVAADSMGDEELEAEY

>SoG_04219.T1

MEAASSSFTSRRPAAVSLPAFSLPSPSSVIPRAAEGISPGLSSIHTGSSQGSQAQSNMAP

YNLGHMHGSWQTPGHSSYNLSSTAQQSPLNVGSYTAQRPSLYNQTSPMPYGTQRDSQSPS

GGNGDSLPGPPEHHVHQPFPSPLGGHAGSGAPSYQQTAQNGMIPSHSSSQSPAQSHNLPH

LDTYGNGRGAGGYPLSSTSSAQSAFPPYSSLQSQPSPTSGSPGIRALGSMAPPAPYRPPF

GGYGSLPGVNGAVLSNVHHPGGSMSMVGNMGVPSYHGHPMIYGQAPPTAQERPFKCDVCI

QAFSRNHDLKRHKRIHLSVKPFPCPNCTKSFSRKDALKRHKLVKGCDKKDADKGSPDGDS

ASIDDEIKRE

>SoG_04224.T1

MSPHIESVSNSGDEDSAPMLVGGKSSVANGNITTAANGNGTYTNGHGAVQRPKPQQQSAR

SASPYLKAASLDSAEDFDLICVGFGPASLAIAVAMHDALASGRALLPGNAQPKILFIEKQ

ESFAWHAGMLLPGAKMQISFIKDLATLRDPRSEFTFLNYLHSQDRLVDFTNLDTFLPARV

EYEDYMRWAASAFTHLVQYGHQVLSVVPDAAGQGAVRTFTVQAQDVSSGEVRSCRGRNVL

IATGGQPSIPASLPMKHSRVIHSSQYAQVVPRLLADAQAPYRVAVIGAGQSAAEIFNNIH

TLYPNSETRLIMRPEFLRPSDDSPFVNSVFNPEYVDMLYPKPASYRKNFITEARATNYGV

VRLELIEHIYERMYEQRRVLGRDESAWPHRIMGGRKIMSVEPTSEKSLQIRFARVELLES

DHPLSPEAEESFEADLIIAATGYRRDAHLDILRDTYDMLPETQGPIKSTGKTEYGWTVQT

SQGHHNMAVGRDYKVQYKPGTVQSGSGVWLQGCCEATHGLSDTLLSVLATRSGELVDSVF

VKDGE

>SoG_04282.T1

MIFPKVLLTIVTLGFHPVLAQQPVWGQXSCNASGGSPTTSGTAPGTTNPPSAGASRSQGC

GKAPISSGTRSVNVNGKNRQYIVRVPTGYNSNNAYKLIFGFHWRGGSMNDVAGGGTDGAA

WAYYGQQRVGQETSILVAPNGLNGGWGNSGGEDIAFVDAMIADIESNLCVNQSQRFAIGF

SWGGAMSYSIACSRAKVFRGVAVISGGVLSGCSGGSDPIAYLGIHGVSDNVLGISGGRSL

RDKFVTNNGCNRANPAEPAPGSGGHVKTEYSGCRSGYPVTWLAFDGGHYPGPVDGSGESG

ARSYVPGEIHSFFNKLS

>SoG_04289.T1

MASVRPNNSSRQKLAKRACDPCKVRKIKCSGVSPCNGCISAGIECTFLKAQSTRGPRNLR

GRTIEKIARTQREQDSSPKQTTLASSSGGNALKKLIDLLDVYATRLYPIWPIVEIEQLSN

AVKADPQDERAHHLAEAVALATVAQLKLASEWEVTAQKIEQDQQYNPGDIFDSLRVSFFL

HIYHENQTAGGVKSLLYLREAITKAQILRIDRESFFASLEESEQQMYRRVLWLLFVTERS

GVAMLHKLPVVLKPNIMFPWFGRANDHFQVLPAFLKLVHLFWIFDQSGIFELLRNSDSDV

LNMESLARSCLESLQKKLQESAADDMYANDVQRADMLVTRQWMRAVLWRAALRFGIVVPD

NHPLDVADEFLRLASQLPKVALESQGPTLEFKTYEIATAVIDALASSYPTGRMKQPHEVL

FGLQQILSSSRGGNKALLSLLNARMAAVTPDVRLFAQPSPVVEPYDSLFVEDMLPKPIDW

SMLTKTTGMMPPYSDEPNYHSIEEQTAQMSWPPLDLGILMRAHSPMTQEFLENPAVADDL

MLMGFSLGA

>SoG_04302.T1

MAVDGLFSADLISPDVSAVLPEGYRLRALRKTDYDSGFLDCLRVLTTVGDISQEQFEKQF

DEMEKQSSYYIIVIEDTNRTQNSVVGTGALIVEKKLYVQPNLPLFYDPPKFLSSVEEQLA

NSNLFLLHSIHSLGNVGHIEDIAVAKDQQGKKLGLRLIQSLDYIAAKVGCYKSILDCSEA

NEGFYVKCGFRRAGLQMAHYYNDDKKGKSS

>SoG_04305.T1

MSTLAIPPPLAFPPFRRETAHWDSFRPAVPSLPRLDTRNALPSIRELVPEHLLSSQPQER

ASSGPKTLLASPAAAVPGLQTSPGYNHISEGHKKRKLSEEEYRESERARQVPRLYHPSEP

TPSYPRRESPPAAAAAAAAPPPPSAAPSRPEAYYGESQAPRPLSSGKYGPSVEANERGEL

RQSVPSLMPSSSRPYEREGHSQYHHEDGYHSRVPAAAQARIASTEQQQHQQQPPPSSYRG

GEFTYSSYQSSSRFASAPSSATTYERTPFTAPPYGMQYGEYGRFGGDMGPGGLSGETKQR

KRRGNLPKETTDKLRAWFVAHLQHPYPTEDEKQELMRQTGLQMNQISNWFINARRRQLPN

MINDARAESEAMSGGRSLGKTELGRYSERGSRGDLLNLSEGEGRHYEEDMMMRRVHAHHS

RDSV

>SoG_04323.T1

MENPSSQPANYQFGDKFVLPENCVEYYLFVIDPELEARKKLSQLESIRQDALQFSQSITQ

DYIWQREEFNLELRNEDGLAYLYGTTDYGDAVEDEWLIVYIIRQLTMSNPSLWSRVADAD

GEFLLVEAANVLPRWITPEIDRFRVWIHNGQLCIIPVDEPAAPKNEQLSLPKAVRFLTDK

PSALVHSPFIQEEAFYRLEKYPGQIADAIHHSMVTIPRRLAFVIHTVPRAISAAVEAFYL

RDPISLRPILAKTRHLTFPPEDLVTTSVRFSRVLYAQLRSQRFEAPPQWRDIIHNSEEVK

DHEAHARLETGMKLTCGFEILAANAASSKSRIVREMAIVIDDLKEDGDGILPSDAEILGW

PHSQRNDEEQWLDINYEDFERELSGQKATPAAKGGDSGFGNAQTQADLRKIVSRFEAFLN

DDKAGLDGAELDDMDRDNDSENSGEGEMDDDSDSEDREISFDEAEFSRMMREMMGFQPMQ

SSSMDARPGEKGSREASVVDMDDAKEIEEIKDLTTQMESELKEHGALKLDSQGDRAIKDK

GKGKARASTGESPDESDGEVDIDYNLAKNLLESFKSQAGMPGPTGNLLGMMGIQLPRDEE

NNDEHNPT

>SoG_04374.T1

MKGTQVQEDASHHVSGQSNEPMTRPAHALTYEEVASELQTNTAEGLSSDEAAARLIKIGP

NNLGEEKGVSAFSIFVQQIFNSMTLVLVLALAASFAIQAWIEGGVLGGMILLNIVIGFFQ

DLQAQRTIASLNSLSSASARVIRNGHSDTEDAATLVPGDIIELKMGDVVPADARVIECVN

LEADEAALTGESIPVRKDGSLRFGSGFDSDCEDDDIGPGDRLNIVFSSTTITKGRGKAIV

FATGMRTEIGAIAAALRDSGNKRRLKKNADGSSSFMHYVEFFLGKVGDLAGEFLGLNVGT

PLQRKLSALFLTIFGIAVLCAIIVMAANKFSSRKDVIIYAITTAIGTLPVTLILVLTITM

AAGTKVMVERQVLVRNMRSLEALGGVTNICSDKTGTLTQGKMVAHMAWLPSHGTYSLGGT

NDPYNPEAAELYFTSDQPQDIKAGNVFDRKIDALQEERLHPSLKKFLDVAHLANLAIVKR

AVSGGTAEHIWSVNGDPTEIAIQVLAARFGRNADMLPSFDASEWKQIVEFPFDSSIKKMS

VLSQNVNTGEEMVFTKGAVERVIDSCANITTQDGGSEALTDHKKGEIIRNMEALARRGLR

VLALASRSSPRVVTEEEARHGNLKREIFEKDLVFRGLVGIYDPPRPETRPSVFKCHQAGI

AVHMLTGDHPETARTIAVEVGILPRRMDLLRADVASQIVMTAHDFDRLSDDEVDRLPQLP

LVVARCSPTTKVRMIDALHRRGRYVAMTGDGVNDSPSLHRADVGIAMGITGTDVAKSASD

IVLKDDNFASILNAVEEGRRIFDNIQKFMLHVLAANVGFVTALLIGLAYKDSTGVSIFQM

TPIEILFMLLVGGTFTETGLGFEAASLDILRRPPQSVRDISLSLSLSLSKTHKN

>SoG_04382.T1

MASNDPSVRQMMPPESQNAQGSQMSSFSTFAPQQFPQRETQKNYVFVDEHNRHKRLKVMR

ACEGCRRRKIKCDAATTNTWPCSACIRLKLQCVRPNGFDGAADPSAFEMIDPSQFQQMPL

AQQQMMQGQPKQGQPMYMQGAYSDAAYQTLPYDASAGHQDIHYTTVSPQAGMMEQQQQSG

GQNVFPTPPIQHQQSTRVEESSPEAYSPDAYQQQDLADLLGTLKVDEAGTASFRREEQPV

VDDDDEELEALPPLPVGRGMKIRIPPELMPDDETVLHYFDLYFTHVHPYIPVLSKPLFYQ

QWNQNRASISPLLLEAIFAIGGRLAEDPGEGQQWLALASRHADAFMDVPRLSTLQALLMI

LKAREAAPKRGYYYRSWMTIVQCVQMGKDLGLDEHFDDHQIGRGCDCSPTECQLRTRLWQ

AIFVCEVMVGAPQGRLDLAVDLDSVDFNVQRLLPGGDESEYHVSRNFTYFARVVRNVRKM

SKIYARLRKKKEWGIDPKFQQLNQDVHQYLSELPADLAVTFPADGSPPWLPSHVIGNIFS

YYYLTLILLHRPQLSFLDPSGADGSWKHHMLVCYDAAKCLCRLQEAIVANFGLTGLQCMQ

RGYSFTVYAGLSCIVLHLVAIVSPDTDLNTDARQFFTRHMRIMERVMEAWHMPELQKQVD

AVREAFSADTRKPFALKPSFPYGSPHPSNQSSPPRLATGYRPGTSAMDQQQQLQNYQQLN

YTNHPISPPISAGPDTKSESPGVQTGGMVMLSQGSQAPGMQQNLSLSDPPAWNPARLFEQ

WNSTFGTPEPSEPSPQARSLKLSSSPPGVPEVSTLQDMQAVNSTLPAGQQVSPQQFSTPQ

LPNFVTPAMWQESVASVYEGGLKRTWDYEGQQMMKRQ

>SoG_04388.T1

MQFYFVALLFGASALAVPTGGGGGGGGGGGGGGSGYQPCPSTLFSNPQCCATDVLGVVGL

NCGNPAATPSSKANFVKDCASRGQQALCCVLPAAGQAVLCQPPV

>SoG_04393.T1

MATDKGLEDVPEGQIESNYDETVDSFDDMNLKAELLRGVYAYGFERPSAIQQRAIMPVIK

GHDVIAQAQSGTGKTATFSISVLQKIDPSVKACQALILAPTRELAQQIQKVVVAIGDFMN

IECHACIGGTSVRDDMKALQDGPQVVVGTPGRVQDMIQRRFLKTDSMKMFVLDEADEMLS

RGFTEQIYDIFQLLPQSTQVVLLSATMPQDVLEVTTKFMRDPVRILVKKDELTLEGIKQF

YIAVEKEEWKLDTLSDLYETVTITQAVIFCNTRRKVDWLTDKLTARDFTVSAMHGDMDQA

QRDLIMKEFRSGSSRVLIATDLLARGIDVQQVSLVINYDLPANRENYIHRIGRGGRFGRK

GVAINFVTAEDVRMMREIEQFYSTQIEEMPMNVADLI

>SoG_04402.T1

MVKELAPRDEAPSGLRMDGVSIWWITFSTVWTAVLIGGMVFLYTRRQMPILRIRGLPLSF

GAVILLHIYWLAVQWGYTYGHLMPAYVEFWIMSIWFPFGIALFHASNSRFLYVAQAQKRY

LNSNAEPLKRTTGNKKSLWGRFRSMDYTNRMLLLVCMGMIFQFFLTVVMFLLSKKFHPSF

GVSGTEVTGSPNEQKVESSRGWEWWPSVFWQCFWAWIVAPLILWRARDLHDSLGWRFQTI

ACCLSNLHAAPMWLIALYVPGMAPVNRYWIPPQXARIALSIMLLEIFTIFVPCWEVVKAQ

TLKQETLDSIARWEARNKAGGAGTSVDTGSTYVSWKKVRSKRAASVKSANSSILTMDALE

HTLTKNPEPLQHFSALRDFSGENIAFLTRIAKWRNQFYPRVGSGERKSSIPPTEVDMREC

FEVALRIYIDFVSSRGAEFQVNLSSTDFRKLESIFEDAARVVYGEEISPDPATPFETANW

RIGSEKTDRPSNGSEDAIMSPTEQQGATMDNIRYWGEIPESFDEAVFDNAEISIKYLVLT

NTWPKFVKERRSIDSLSALEANA

>SoG_04412.T1

MSLTCRDAQDTGLPPNWEVRHSKSKDLPYYFNTIDKVSNWVPPPETDTEKLKYYMANKHT

VDREPEPEYIEPRKDQVLASHLLVKHKGSRRTSSWRQAKIERTKEEAMEIIKEYERRIRS

GEITLAELAKTESDCSSAPKGGDLGWFGKDVMQPAFQDAAFKLEPGEMSGVVDSDSGLHL

IYR

>SoG_04424.T1

MMSSATWDGQDHHMGAGTDDDFQQFLDMSAMGSMGDGMQFDFNAFQNGNAQQHIMAQARE

HTQDSIMGNADVADLIPRTDSMMPEPATTMATTMAGHMSATLAPTTADNSISDIDAQIQY

LQQQKFHQQQRQLHEQRVAFFSTPHGHSVPPTPQSFEMPPGSGNFYSAADMPPSGSFERP

YHQRAKEQQDMAFTPLVSPAVTPLDPHFNMDGGFTVPGAYFSPLTSPALRAQADHSSTYD

HSTHSHNSPIEHSPVAIDTAMTVDPQPPVSQTTGLDLSKKARKNSAAKARAKSSVKSSPI

SKPMRKKTGPSPALVSQVLTEVEEGRLTSMLPLPASSTDGSEDASVSPENLTDMPPPPVP

ARRSNSKSPYIHPQNGSMVATTIPTSAPSQATVAGPIGVLMEDQQPHPATPASLMKLPAS

RSKRTSTTSTPTSGTPTTETHDPSAIENMEALELPESISNQSAAQNAQKKQPKRIDTSVA

SKTPKMGPSSAKTPSLQTMASPLQRAQGSLSATQSPQLRPGSSGPSARKTPQLSSRESRK

GSVHHSPALLPRISPNIKPLLPGTPGMSAEDTASRLLMSKSNYQNILEGNTVPGVSYPSE

LSTNLTSKRTSHKIAEQGRRNRINSALQIMASLLPDKEDLGTAEDEDKKESKSANAANSK

ASVVENAIVHMKSLKQENTDLKKEVQELKEQLEKLQSDKS

>SoG_04439.T1

MMEDDTPDNITIASASYMGEDDRFDMSHMSTGHRKRKKEEQAQDFIQAQHSLYGDELLDY

FLLSRNEQPAVKPDPPANFQPNWFIDADNHTALHWAAAMGDVDVIKQLKRFNASTSVQNK

RGETPLMRSLHFTNCYEKQTFPAVMKELFDTVDARDSTGCTVIHHAAVMKNGRAYSPSCS

RYYLDNILNKLQEALDPSAFQQLIDAQDNDGNTALHLAVQANARKCIRALLGRHASTTIA

NNEGVRAEDLIMELNASRRERGPQRSSSPFAPDSQRHASFRDALANERTSSSRRTIAFQS

AAALSVQSRITPAIQDKFQELAKSYDAEWHEKDTAEAEAQRILANSQAELHMVRQQIADL

EGQLESDEAAAKVKNEANLATHQVLSLITHQNRIHVQQAVDSELSKMNGDASSADESASY

EERLALARHLSNLLADQRAAEVEYVDALSMVGTGDKIDKYRRLLRRCLDPKDGEELDANL

DSLIEMMEEDKPGPAAEAVDSNGAAAAAGPVGAEPMDFSVGA

>SoG_04440.T1

MVKAASNPATGPGVYSATYSGIPVYEFQFGVDLKEHVMRRRHDDWINATHILKAAQFDKP

ARTRILERDVQKDTHEKIQGGYGKYQGATPSMAQRRYPI

>SoG_04449.T1

MSRKFPLGQYKAGGGHPTGQRTGVQEKIDEALLSYVANLFNKYAGPDNKWSREQIGIFMQ

YIQSEDPCGAASSLLEKESMTLRDLLDYITSPSGNALDIAKPQDLSHPLSNYFISSSHNT

YLTGNQLSSDSSVDAYKDVLLRGCRCVEIDVWDGEERFLAGYGPDDEQNSKDRAAMKAND

PDAKPGPTYKVTFKDKMMIKAARWAMNKFDPVDPEGRTIDDRIADMMRGEPRVLHGFTLT

KEVLFRDVCRVVKEYAFAVSDLPLIISLEVHCSPLQQNAMCDIMEEAWGDYLLPGPTEEP

SRLPSPDELRRKILIKVKYVPEDKEEKVLEGDRTAEDDDSILEEVQQEDGTKRAKRVKAP

KITPRLSRMGIYTRGVSFKSLSQPEAGMANHIFSLSEKAVLETQRKDPIAFFAHNKDFLM

RTYPHGLRFDSSNFDPVVHWRAGAQVVALNWQSWDVGMILNEGMFAGSDGYVLKPPGYRS

ADSGPIPSKTLARVAVSVLAAQNLPLSNKGDSADKFTPCVRVGLHMEPDAMVAMISEDAT

AEQVKQVGYKGTTAKSKGTSPDFGGETIEFADIDGVVPELTFLSFMVLSDGDLAAWSCIR

LDRLRSGYRFVRLLDKDGMPCKGILLIKSDLTEVASEL

>SoG_04453.T1

MPGERLPSLASLPPGQAYSPGTSSPRVSSIASSNGSHADSQSSFTSAPSSLGPKTPSPTF

PGHAITGPATTIATYDAMNQGQPDMYYQHMSAPQPHTPQTVTSGAMSHYPPQQPQLLHPG

PTQYGHPPPYNSYGYANGLTSPPTAQPVPQPNVLPLPGGNPQGGVQHSYGGYDTTGQVSP

AGMKPRVTATLWEDEGSLCFQVEARGICVARREDNHMINGTKLLNVAGMTRGRRDGILKS

EKVRHVVKIGPMHLKGVWIPYDRALEFANKEKITELLYPLFVHNIGTLLYHPTNQNRTSQ

VMAAAERRKHDQGQIRSGPGLPSMPPQGTPQQHPQQTPQQAHQPMPLPGPQPPLGHVARP

SLERAHTFPTPPTSASSSVMPPNMGSSDHFNWQAQGMNGSQGTFPMPPDSAPSVTRSMPM

TPAATPPGSAVQSMQPYSAGGQSYDGSRQIYSAPASQQSPYQSTAPPPQDRMYSQNGSYS

KTEMGPPSNRPSVSGPSGDQPEAKSTNGALPSEQGSQPQTAEEDGDHEHGQEYTHDSAAY

DAQRNSYNYTAPGVGSLAADSNVSSEMNGSPNHHPSSGRATPRTTASQQPYYPQHGGYNT

PPRVQQTSSSLYNVVSNERQPANGASGNDVYAQQGDIAQPMPNGYASQPALNGNNGSMKR

GREEEEEIPRPGSGGPGSMGAMDLKRRKTMMESSVAAPAYDAMSRPASAVTAPRRR

>SoG_04495.T1

MPMAISPPLRPKNGLTKFTNCRLLRGLSLVEEDLWVSSVTGKIINSQASFYDELNLPDAT

IDLGGRIVAPGMIECQLNGAFGFNFSTLLDDMSQYGKKLKEVNRSLVKTGVTSYIPTITS

QRPELYQKALPFLGPSGTARLAEDGAESLGAHCEGPFLNPTKNGVHNVDVMREAYTLSDL

EDCYGAANINPTSAGAEIPVKMITAAPERGQMADLIPELRERGIIFSIGHSEATYEIARA

AVGKGATMITHLFNAMRPLHHRNPGIFGVLGIAESQPRPYFGIISDGIHLHPTTVKIAYD

AHPDGFILVTDAMHLVGLPDGAYPWTNGEYMCNIVKKGSKLLLENSETIAGSSITLLECV

NNFLAWTGHSIPQALRAVTATPAAMLGLEGVKGTLAPGADADLMILTDQEGGRGLKLNEV

WKFGCRVSRSD

>SoG_04496.T1

MLRAKPPVRNLATKSRYQSSRYLKDAAACRLVETGDGSGLAELPSWGEMVCAPLLLLLFP

LVLLPPRPAKPLISSGLPPVKQYQPPSFFTSAPQSPLHPPSTTTTTSDHERNSEFQYRGI

AARLPPAGTRPSQTSWFRPFAGTLITQDLRFQPHPTTELDVRAPPTPRAWPTPRSFAYLD

TALSSKTGRLSYRHLGFNSMPAMPSGPADAMGMNGQLPHGALPMDHFDHDLGFDDPLLDG

GALPALPFTPGYDFETFAPTFEDPFSTFQNRPFDTVLNNNNPDPLREEESPQEPDDKLLG

FSESVIHAAAVDESGNLADLSMTAELRGMFFVAEDVFGGESSDRPLELTCYRRNLWQCFG

QINLPRSVAGVINEQGQQITLVDLSASITARESIEGKPTEIISIPWKSANPQLAEESKVA

AAPSNVTVDLGSGQELDANRVSVPISWKRLQFKHATANNGRRKGLQQHYVVQITLLGKTD

NGDMIKIAEISSGPVIVRGRSPRNFDSRKDVPLTGDRKLERRNTSGSDNVVLKTERDVLQ

ATKHNFMQSTEWVTPQPYVQPSHNSHPAKKVALSTPTMTKPPVPSWSSDITSPSKAIGMH

QQHTSHGYQHQPSRGSVGKPSMPINLSLSDDDRSPNRSSADLASPQLGKSALTSGQNAGS

SPAEDADPLYEYFPLSLDDWFVTPAARPKTHLANVVARMPPVDAVYRPHVVHHTIVPPEI

KAQQVQSKSKRYFLPE

>SoG_04502.T1

MAPVSEHDDQLLSLDDPRLRFVHDDSPSSSHPPADASSHSYFASPNWMPFGNSGAPATTA

YTSTTLTTAPVSMPTSTWASGMLPMDGFPADMVFPPSDVNPAFVFGPSMHGEPQQQQQQQ

QQMTPVAPTSTNKPAPKQLAGMTTLTPALQEELRNIAMPPNLRYTSPNSASSPDSALGDG

KLGNLSSPDTLQPGQAKAGSRKRKSSDEPEDEDEEDEEGKPIKKTAHNMIEKRYRMNLND

KIAALRDSVPSLRIMSKSVRGEDTTEDREELHGLTPAHKLNKATVLSKATEYIRHLEKRN

TRLLDENNAMQARIAAFEKLFMAGAMNGSIPQQQPPTPMQYAHDAHGVGSPMPLPPDGGA

APVGMIQVPEDMKRIISAQMAAGRPYPVPEQHYYNQAPNVRIIQQQQIQQQQQMQQQKQV

PQSRWPTVGPYYGKLMVGSLAGLMILEAVREDELSNETPEGRGLFAVPVQMLGRMAHGLD

MHVMGYHVHSSLKLLLVLGTFLWLFIPSLFSSPDPKTKKLDVSTLRPLPSLASSIHVRRQ

AWLTSIQTVWVPRHNFFLEAAALLLKICKLSVRNAVGMQGYQMITGLTSEQETARVKAWS

IALDSQLAGGDVEINDSRLLLTLLASATLPDTPTRLMLKALHIRVILWGLSSNGLQQKAT

NAVAAKLARTRWVEARKLNRALILLRRDSNEQHDDELPEHLAALVELDSDDVLNADVIQR

AHNLAFNRETTHNVKQHIDGMDAVVDDLALGTPLDAVAAWWSTGALHTILADALARDDES

EKTKAEDINLAIQVAPIGSVARVRAVVARALLIEKHRGANIASALEAVGPGKEESPLSKT

TSIIESGSFIGSPDLRLALRCAMAIAHLRRLGDTKMAPQQKLIRLVDSIMTPSVVSSMSL

LGCTAAFQLMEALFEHKDSAGQFCLTLERLAGNLRLWMGGPSGSERGLDADVRHKVVDRC

LDITKDLVGMEVDTGYGTLSEEEQ

>SoG_04510.T1

MRAASNTQYFTGESLISTHAVIILLQKGFLSAPRPFSSPMPRSDEAAAFFHAVYSAVCEV

PHGKVTTYGHIAALVGTPQRPRQVGVCLKHLPSDPASRFNHDNVPWQRVINSKGIISPRS

QPEGTQNQAEALRAEGVEVTRSAMGEWMVDFGEYGWFPDVLPSEEAAEAEEEAGSVVS

>SoG_04526.T1

MASLPDSYESHPIPCLKLLHHPPSHPTVTPIITILLHRPSARNGFTDEMAASLASAYDLL

SRDPRVKCIILSSSDPSNRIFCAGMDFNAKHEIGSSATAHRDGGGLVTLAMARCRKPIVA

AINGSAVGVGITMTLPANIRVVSKDAKIGFVFSQRGFNMEACSSFYLPRLVGTSRALHLT

TTGAVYPATDPLLSSLFSSVVPAGEVYSTALGIAEQIASNVSSVAAHIMKEMIYRGPSSP

EEAHLLESKIFFDLYNGPDSKEGVASFLQKRKPHFSSTMEKDRPTFYPWWNPVDVKAKI

>SoG_04541.T1

MGSKLLEQLVGRLFLSAVKASSSRGQQNSRNTLPLRLPGSEPLSRPSRDRLGKALLHNER

PSTSDKTLSVACDSSLPELPCAKVALLTSPDLPITAVGPASELVCPHPASQPLLSLSSVS

QPTPLSVYDKDQSNSHIVGPANTNDSQVLTNYLSCVHGTSSGMRLVRMMPISWSKPVMFT

PVQHRTFGKSSTKALPYEKLHIMEKMIGKHTAPLTDVFFVKANTCLPLLEPTSFLTQYRN

AKDKISPALLACLYAHSVTYWQSSPQLHDVPPPDHQFLWSLASDALYAELHTAPGISTII

ALLLDLGGRPTTSLIANAVLLGSAVSLAHSLGLNRNPMQWDIPDGEKVLRMKIWWCLVIH

DTWLSFAYGTPPHILPAQSDVPPPEASFFNLGEGDEQAGSGDAVTVFIALSGLSAVLDNY

LRYLYHIRDDRTGPGSIEDLELKLNHWINGLRPDTRRIITRGNDLRAPGAANLRLSYLSI

RFLLCRLKLDKDRESKDVDFSTLGNRYMQVQSTAEDIVIFVQELADEQLNDFWFPMSAFT

FTSVTTALLRFAVETKGSQSTPGQNTSLQLAWDMILALRNLRDRVGWDLGEHCLQQYSSV

VEKLIEVPGTGLDSIVFQGFEGTIEDALAFDGEFSNLWGGAEPGL

>SoG_04548.T1

MKFSLLTALASLAAGTMAQVQLCDQYGYHAANGYYFNNNKWGQGSGSGSQCLLVFNTQGG

GVSWQVDWTWSGGQDNVKSYPYSGRSMSPKLVSQIGSLPSAARWSYQGNNIRANVAYDLF

TASNPNHDTSSGDYELMVWLGRYGNVYPIGSSKGMVNVAGAQWELFDGYNGAMRVFSFVA

PSQRTNWSGDLKAFYNHLTQNNGFPASSQYLITNQFGTEPFTGGPATFQVQNWNAQVN

>SoG_04554.T1

MDQDDVAAPAVSDAPRQKRSRVLLSCAPCRHSKLKCDRGQPCSQCFKKERVDLCTYAPKP

ENRRPAKGVAARLRRLETMVRDMMDNEGNVERLAMQLQQKLPDRLRGAEEVVTGLSSVVD

GHVVQGANTTTYVGATHCMAMLEDIEDLKEYFDYPEDDEEGDSPEGNPQDSEEWTILSAG

APMDRDQLLRQLPEKSIMDRLVTRYFESMSPSQHIVHRPTFTKAYKRFWEDRNSTSYHWI

AQLFMILALGIHFNRFQNPDEIEKDSFMPLEDRIKQYRACAGWALVRGRFTRPTFRTLPA

FLLYCESHFILNRAAQMTCYILSGVFMRLMLKMGLHRDPSKLANISPFEGEMRRRIWNMG

TQLETIVSFHMGLPSMISGIEADVEIPHNFHDEDLEEDCKELPPERPPSDWTVMSYPIFK

TKIMRVFDAIGRQAHALHAPSYADVLELDGRLTDTWRTVPFFMLVRPLEDCVGDHPSLLI

QRFGIGSLYNKSRCVLHRRYLAETFPKKEHDFSRQQCLDGALTLLKYQQVISEACQPGHL

LGQNGWFISSLIVHDYLLAAMVIYLVIQNEHYAAENSPHSWHAKDGNSPTKDELKDILRG

SYNIWSAVAEDTAELRKTADTIAVMLARLGDPVSELVAGAGKARLETTSTSNSASMTGTS

HDPRTEHSGSEALASASSWGYDTSSTRLDIEPTPFMIGQAMDSAPLDMPAMGFTSDQDGM

TTMEAAWMADINEMDWRCLDVSLAHSQTAGLNAEPGAGSTWMERMPMNNAGDANYQPLDF

WGIEAANPSGEQNNM

>SoG_04566.T1

MCGIFACHRNPGSAALVGKWRGNSSLRTRAHPTTPAITTSGKKNHPDVQKFKPTALKLAK

QVRHRGPDWSGNVISNHTILCHERLSIVGVESGAQPLTNSDETIILAVNGEIYNHRLIRK

HLKEPYHFKTASDCEVIIPLYMEHDIDAPNKLDGMFSFVLYDKTQDRTIAARDPIGVTTF

YQGWSSKEPDTVYFASELKCLHTVCDRIEAFPPGHVYDSKTGKTTRYFNPSWWDETKVPT

SPVDYTKLREALEKSVRKRLMAEVPYGVLLSGGLDSSLVASIAQRETLRLKKQALEANGG

VELPENPDLGEGLVGIDDDNNLSTVTYLPQLNSFSIGLPGSPDNEAALKVAKFLGTKHHV

MTFTIEDGLNALSDVIYHLESYDVTTIRASTPMFLLSRKIKAMGIKMVLSGEGSDEILGG

YLYFHGAPDAKTFHEETVRRVKNLHLSDCLRANKSTSAWGLEARVPFLDKQFLEYAMNID

PAEKMHTKERIEKYILRKAFDTSDDPKAEAYLPDEILWRQKEQFSDGVGYGWIDALKDNA

ELHVTDEMMKNPKPEWGDDIPDTKEAYWYRTMFDEHFPPHCASTVVRWVPKWSTQTDPSG

RAMAVHNNKYQNAA

>SoG_04578.T1

MRGTLALSAVAVAQAASFGIETVHNAAAPLLSSIEADTIPDSYIIKFKDHVDDAAVSTHH

SWIQNIHSQGEEQRVELRKRGHLSDVAEIFSGMKHTFSIGDAFKGYAGHFHESVIEQLRN

HPDVEYIERDTVVHTMLPIDGESKVTEDKCDGETEKEAPWGLARISHRDRLNLGTFNKYL

YTAEGGEGVDAYVIDTGTNIEHVDFEGRAKWGKTIPAGDADEDGNGHGTHCSGTVAGKKY

GVAKKAHVYAVKVLRSNGSGSMSDVVKGVEFAATSHLDNVKAAKAGKRKGFKGSVANMSL

GGGKTQALDAAVNAAVRSGVHFAVAAGNDNADACKYSPAAASEPVTVGASAVDDSRAYFS

NFGKCTDIFAPGLSIQSTWIGSKYAVNTISGTSMASPHICGLLAYYLSLQPAEDSEYSLA

PITPKKLKENLIAVATEGTLSGLPDADTPNLLAWNGGGCSNYSKIVAAGGYKANRAASSA

FKKIETAVEEKVEDISLEIIHGSKTFSGKAEKFANKIHDLVEEEIEQFLKEVRA

>SoG_04603.T1

MGKKKRSQPSVEDLLARPWCYYCERDFEDLKLLTSHQKAKHFKCDRCGRRLNTAGGQKST

GESSKTAFADLFGHIGLSVHMNQVHKETLEHVENALPNRQGLEVEIFGMEGIPQDILDQH

RNRIIQNFYQAQEDRRIATGNPLPGQKLQNSRPKIKYETAEELLARFAEWKVKRHNAPAN

GGAMEGVVPTNPSPVNFNQPHQPPPQGYEQNYPAQGYPAQQPYGYPTDGLPPRPGGAHST

GLPIRPETLPASKGDDIDQLIRMAEAGIRPQKAANEGEDAGDKKSKKDKKGRMVYDDAEI

SPEERMAQFPRYAFTPSVAAA

>SoG_04612.T1

MIAFAAAAVVHLRWIPAITGAAIPCHAGLVRFSGLSTAPNPVLARPVSLQLDNSTDYSYS

QQAMVPGTAGMFSFPQTEPQTDFLISSWGTTVEGAQSLQDFQEAGSAHSGEHEDPIFTSG

TSTPRGVRLDPAHNVEKWTNPRIASVSQGGHAMSRMDSNRSSASVLSRSSQLSHAHSTGN

ASAFRDGSQTDGSLPGMNNVLLDGTSGLPTQMYWNDYHPLELNSLGLGDGAYQVTDVNPL

QVVSNTHIPLGSDVIDSPLSWECFSTISRTSSPSTVDEAFAILPLSPHSSPEIACQSPSV

DRKPLVPEDFNILPGSQKDDTILNAVLPARRQSNETDARNHPLYKDVKPHADGLYHCPFE

NDPNEECTHKPEKLKCNYDKFVDSHLKPYTCRVASCNGAKFSSTACLLRHEREAHGWHGH

GEKPFSCTYPGCERAQPGNGFPRQWNLRDHMKRVHNDHGSTGGSPPSASAPQASKGRKRK

TDVQESQGTSARKATVKAMPAPQVSATSTKPLIEEWLEHRQAVESILRSGLNTPEDIGNI

NQISRVKEHLAAMAKMTTDFSTQPRTDIITAPRARAYTTTG

>SoG_04638.T1

MYGVVDSKGSLSIAVRQNVLVLPRHGQGPGHHMNDPSVSWLAQQGDGCASRTRHHSQPGS

SIHEVKLSPPPYYQDQTYACMGEVRPTNPKRRSLLHSASRIDSEAPSHVQVSSEYHPGHM

ASVAHEHLLSFTPTLPFLIWTSLNARRPVNNPTPELSSLAYSASANDDLLDSPTRQMEQH

TENLPEEPAVTAKTEEEHQWYFAFGANMLDEVLVKRRGIQPLRQEAARIPTHTLCFNAMG

VPYSEPGMGGLRAIDDETQDLPVHGVACLLTPQQMARVILTEGGGIAYVTEKLPAVLLSD

GSSITVTTLLARHQVPRSWERLPSRRYLDLLVRGAHEKGLPQEYIERRLIAQPAFEPNPT

LRFRIGKFTFESVWQRVSSLIQKGVRKYKDDEAPTNWPHMMAIQTLSNGSMELRDIGSAV

GLIWHQARSRPTSVAVSKQGRSLTYAELERASEGVATLLIENGAKAGDLVPLVTSRCLEM

VVCLLAISRVGAAWVPMEIDTWGADRIKAVSKRLDYDVSLVTDQEPRGLRNALCFDQIRA

AIDASLQPLDAEMTRTAETTSTGQIDAVGSSRLKDIAYIIFTSGTTGVPKGIEIKHESLL

NYVWPNHEESSMPFNLGVQPGDEVPLLFSVAFDAYYGVLFSTLCSGGHLLLSEPSTFVDD

VKSCNILPATPTLLGAIDPSDLTHLRAIFLGGEAPSPELVRKWSCPGRRIYNAYGPTEAT

ISVTMAELRPDHPVTLGRSIRSSQVFLLDEDMNESSVGEICISGPTVLALGYYKDPERTQ

ASFVQWRGQRIYKTGDMAKLTEDGLVFLGRKDFIVKNRGFLVNIEAEVIPGLLAYPGIVS

ATAAMHQQRLVAFVSPKVVENGLEIRKFLESEQDQFLIPDEIVVLEQLPRTSNGKIDTKA

LIRDLCQGHADGGDRERGRESSEGSCDAISAFRRVLADAMKISAASVNMERSFTELGGNS

VFAIKAVSLLKQHGLNVSLSSLFQEPHLSAIFSRVTEESSCANGLKTSADDGTSVGATKV

TTNGDHNEKRNNSFTLTSTQLGMIRSTMLEPPTGYMMLRISVADIQKNRDVFPRMMSRAW

SSILSSIDLFCCQFNLLEGKGQLGCEYYHDWEAISVDMDGLEEAVSRQSDQLLALAKHTH

SSQSGIFKPVTALRLVYNNDETSKTANCVLLWLVHHALVDGESISAIFEHVREELALLIQ

GGQGDIPGNQPASFVEYANAVDLHMNSARDEDEASTFWAAALEPVLAGTQINIIKSNNRP

GDASDGSTHERQLASSSIVGEEVLDVGMHVADFKTCAQAVEGMSAAVIFHAAWALVLQRY

SGNDTIVFGSAISARNFPIAGVEDIIGPLLNHCPVPINIPDRSSSRSVFLRSIRDLMLQI

IEHQWSFPRVLEQLSAGSHARLFSTALFLEYDLPGMTSRDARQGAGEAWSWERADWPEFG

LTMQVRNQAGQVSLRALFKKEQYPRPLIQGMIQHFRNIVVALVSPGDKTLRDVQQMMLGD

TETLSLVRNNPTYFDPYYGPQSLKELFEIGVDKWPNEIAMESPSKTLSYWHLDSITNFIG

RRITESVGAKGVVAVLGDSSIDWLVGAMSVLKAGGTYLPLDTKLPVERMVIMMATAGAHL

VVLPNKQCLQQYSALEEPKLFMDEMWEYESTASSASHERLPTTAAMDDYAYIMFTSGTTG

TPKGIKVTHRATLSHFSSEVTTLNARPGRRHAQVFSAGFDVSIAETFGVLCRGATLVLKD

PSDPFAHLARVHATMITPSLLSVLSVSDYPMLDSIYLIGEAVPQTLADTWSEHKTLWNFY

GPCECTIAALYTQLKSGDRVTIGKPLPRVGVYILGQDMMPVPVGVIGEIYLSGVQVMEGY

IGKGSEVATKRAFLKDPFLAGQPLYRTGDLGLWNEDMNVVFLGRTDHQVKVRGYRVELDE

IEHIIRKSSGSVQQAAVIVLQDRIIAFVSPEVVDVTSVQAALQQSLPSYAVPQNIFTLKH

LPTTPNQKLDRKALALIATELQKSDPLVSQQVKGTGAEARGKMLSLVESAWRSVLGLGEE

TPIGVDDDFMVIGGHSLQQIKVAQKICAVMGQTIPFGIFIRNTRLKSLAQAIEDFSEQLN

QSIGNNFLQFCSVPTQASPESLSHAELEILRMHRESEYPFTLNVAHIITMSGHIQVHLLK

QAMELVAAEHDILRTRFVLRNDETTWRTVIDPVFRVDVLEEPLTTSTVDSMVNTSFDIQN

DQLSRIAIIREASDCTKIIFVQHHLICDQKSLQIFFRQVAEAYHDLKTEADPVVRPSKAN

DYRSWAAWTAQQLEKPLDDARKKFWASAFSNMSPNDTCRIVNLESTSLRTPIHAQSGTLK

RPSHTCGLDMYLSLVARACGRVFGLRDILLGIPFIDRSEPGTDEILGLFLDTIPLRVIST

TQDDDATLQVRIHAALQDVLANYVPHHQIREVVNMQRLFDIMVVYNRFEERVTRDMDIPG

VQITTEPRRAQGSKFPMLIEFTEGEDDSLVVEFEYFSEAVSTAHAEELMDSLKSLVSACQ

>SoG_04642.T1

MSDLTSRPPIEPTRDQANPSQPYSANLRDAPSAIPPNVFYTLPVTVTSATPPPITTPFQT

TTIAPSPRLGGEDATVDSLTSQHYGQNQAEHPLRISTNIAPPNPQSNHRQDPANLTSPQH

SLTRIPSSSSLKPVSRTPSLKTAISNSLGGRSMSGSSSIIASPVIAAMGDLTPLPSPLMS

GSPGPWKLLGSGSPSLAQRREVARNAAEGSVLVTKSGESVDTAIYNAPKRKLYASIDAGK

PVPPALGGSSTDGASSQHTRNRSVSDYVPLEGMSAPLRPVVVSGSHIKIDIPEANEQHMR

REQHLAESRGLTPTMIKPPTPPPSESSRDSTEGVLGRKHANVELFEAFGRYDQKRRRWRS

LRLLGQGTFSRVMLATSQKAVSDDDGGSDSDGAVGSIAEDRVDRKSLVAVKVCEHGPKGG

ASEDRVEMSLKRELEILQSIHHPCLIDLKAWAIEPTRAILVLSYCPGGDLFDVATSQRQV

LKPRLLRRIFAEVIGAVQYLHERRIVHRDIKLENVLVNLTPQELADESIEWTTYPYSVTT

LTDLGLSRRIADDEKLETRCGSEDYAAPEVIMGQPYDGRATDAWSLGVLLYALLESRLPF

DPHPGMTDAHRMRSRTSHRIARVEWCWVEYAGDDTDHDGDEAKFEAKGLLSAMQITEGLL

KRARSRWTVERVAQEPWVKDAVQVDGGIRFREEKAGEEVS

>SoG_04656.T1

MTNCFFFFFFFSPFLLLDISKGSDSFLSLSLYEACALSPFSPTTLHHQYQHPSTVRVRVP

FEKKTTADTTSVRSEMANYDQPQVDGPPGEGRLSDQGADGNRLEKGSEKASELTAAGPTD

LEPKTEAPKEDEPAPARSITGIKVTSAISRICTEQKCSATVSVLTHPAKWFFCYASVLST

VFLFALDSTIVAALQASVIDTFKTQKDLAWIGVGFMLGNCAILPLGQAFGTFNTKWMFII

NLILFEVGSALCGAAPNMEALIIGRVIAGVGGCGIYAGGLMYVSMLTTNHERPLYLAGIY

SIWGVGCVLGPVVGGSFAESGATWRWGFYINLPIAAVFAPAYLLCLPSMDPAPNKTLMEK

LKVQDWVATTVFLAGCTCFSMAISFGGVVYKWNSGREIALLVLTGVLFIAFVLVTIFHPL

VPEEGKLLPVSFMKTKDLIILPLQSTIVAGAMFASIYYTPILFQFTKGDNALMGGVRILP

LVCLLVFFALLNAVFMPRLGYYTPWYVFGNALVVIGSSLMQTLAPDTSVARIYGYTILIG

AGVGCFQSAGVAVASAIAPPSDINRAVSVMTIGILSCQQKAQILGSLASLALTGCIFQNS

VLNKVGAIILERPGVSIEALLSGTSSAVFRELSSSEQAGVVSALTASVRNAFLYVVATSA

LGFITSLFLSRKKLYLANNQVAV

>SoG_04657.T1

MTSNTFDVVVVGSGWNGLINAKTYLDFEPDAKLIIIDEQASIGGVWSKEKIYPSLYAQIK

IGLFEYSCYPMRREGISNDGYISGATINTYLNEFARDFDLVRRTRLNTKVAKVERASGAP

NSGWRLAIEGSAEVVECRKLIYASGATSHPVIPSWPTSPGFSAPVIHSSDIGIHLDAVSK

IRKAVVVGSAKSAYDTVFLLLSKGIKVDWIIRGDGNGPLAIMPPTIFGVVNSMDAISTRA

MAALGSSIMRTDGFSYRFFQRSWLGRLLALNFWRTVTWIAARHAGYSRSADAENLRPLPH

GNGIFWANAGLGCASVPDFWKVFHNGDCTVHRTEIASLDSGAFVTLLNGERLETDHVFLC

TGFDKSYHQFDPALQAELGLVPDPAPDAREKWSRLDARGEEVVDELLPVLVKSPVPQSKH

DGVESEQSDLHEKPLHGCSRHYRRLIVPSLAAAGDRSIVFPGFIHSIYTPLVSETQALWG

VAFLLDRLKLPSQGDMEIEVATWNAWCRKRYLAQGRKHAYAIFDFLSVKTKRRSNSLVDL

FMPAYPRLYKGMVDEFKQVHGISKEADDHEGGLDD

>SoG_04659.T1

MPGLVDITKSSSSSSSDGFVDADPRLETASQNGNHEEYAAASFPGYAEKPLDEQLEPIAV

IGMGCRLPGEVKSANDFWNMMINKQTGQTPKVPPSRFNIDAHYHKNNDRPGSFGVLGGYF

LEEDLSNFDPGLFGITPIEAMWMDPQQRKLLEVVYEALESGGITLDAIAGTRTAVFAASF

TADWQQMAFKEPSFRHSLAATGVDPGIISNRISHVFNLNGPSIVVNTACSSSVYALHNAC

NALRNNECEGAIVGGVNLVITVDQHMNTAKLGVLSPTSTCHTFDASADGYGRADAVGAVY

LKRLSDAVRDGDPVRGVIRSSATNSNGKVPAVGITHPNREGQADVIAHAYQRGGNLDPRL

TGYFECHGTGTAVGDPLEVHAVSLAMNKNRAPNEDPLLIGAVKTNIGHSEAASGLSALIK

AILVVERGVIPAVRGLVNPSPSIKWNEWQVRAPTEATQFPPHLPVRRVSINSFGYGGTNA

HLIVEGATSLLQGRHPHAAGYTYDNKTEPRGGGKTRLPRRTKHQNRPFLLPFSAHDKATL

TRNIEAHAKVVHNYELIDLAHTLATRRSVLASKGFTVASQRTIGNVFTDIPSSFTFGDQK

RKYSKVGFVFTGQGGQWPRMGAELIRYSQVFRDTIQLLDGVLQELDDGPAWFIEDVLLEK

AATSPIADAEYSQPLCTAVQVALVQLLQSWGIRPAVTVGHSSGEIAAAYAAGLITARDAI

TAAYFRGLVTRDVVTDGSMLAVGLGADDVAKYIDKEREASVVVACHNSPSGVTLSGDSDA

IEKLRVKLDSDKVFARVVKTNGKAYHSHHMAPVAAQYEAYIQAARATSLEDEDDKPKGRD

DVVMVSSVTNTVLPRDARLDEKYWSSNLRNPVLFSQALQTVLSTPELSDIDLFVEIGPHS

VLSGPFKQIKAHVGANNIDYLPTLLRGDDGAVRLLQFAGELFLRNYPLDMASVVKAYAPG

GSSQGLTIVDLPPYQWNYTRPFWAENRTSREHRQPTHPRHDVLGQLVIGSSLTEPVWRNV

LRLRDLPWLRDHSLGGEAVFPGAGYFSMAIEAITQLNELSEKPSSIDSYVLRDVSIKKAL

VTPDDDDGIEVLFNMRPSTRANWWDFRVSSIGAGNISKEHMEGSISINTNPKGARTARAV

PEFAQRASGKAWNQALREVGFDYGPTFQDMEDIRFDGKRYESSCKTALKQSVDESLGESR

YVLHPATVDSVLQLSIAAIHAGRSQAMTYGVVPIQVDEITVWPPTQAQLDTKIATGYAWV

PRRGLRSFEGSAQITAGDGELVLEIVNVRTTSYEAAVPQKEETHFTDAAGPYGQMAWDLD

IDALSSATNLPSLSVPDLVRLALFKHPGLKVLEIGSEYALGALARHAHGSWTCVVDEVDE

HAVKETLKGYSFATTATAQSFLDAETGSSKKVTYDLIVASSLESIPRFAHGLLRSGGYII

TAASHGEEVQDVGEIFRGNEGASVYRSTSASSRTDGRIVHLVYHTTPTRILAQVKHALEK

LNWTVHVSGLEAVPSAKHVDLLADLEVPTLFNLSEKEFTAIQRVIAEASSLLWVTAGGVL

DGKQPEYALTQGLARSITSEQASLDFRTLDFDLDSVSVDKVVASIVRVATLQALAPDEVF

PTSRETELAVSNGKTYISRLVRNHELNRAFISSSHPTSAKYKPGERMRGKVREQGSVVFE

RTEERAVPDHSVEIKVEASSLTREGTLIINGSDYPTTFSHSVAGEVVRVGRAISSLKPGD

RVVGFNADTFSTFQTVPESLLAKVGLGSDLEVLAGSLVPYFNAVYGLEVAARVRANDIVL

ILGGTGTSGLAAIRIAQAHDALPYVEARSPEEAAFLQDSFGLEESQVIIGHSVPISTALE

NLTNGRPADVVFSGGTAVAATTASAAWRGISRFGRFVDSGRKDVLSRGSIDTLPISKGAS

YLPFDFLDVYEHQQSILVELLPRVIMLVNSTAGSSRITTNSVHLTDLDAEMSRFSDSFAA

PQPVITFGSTDKSISLLPPGRPNPSFNPNSTYLLVGCLGGIGRSLTSWMMQYGARRFTFL

SRSAADSPSAAKLVQDIQIAGAVVQVIRGDATSRADVVRAIQAVPKENPIRGVVHAAMVL

RDGMFHSMTYENWRASTAPKVLGVQNLHSALLGEPLDFFLTTSSVSGTLGTPGQSNYAAA

NSYLDAIARHRVASSQVATSIILPMVLGVGVVSENNEIEDALRRKGMYGIDEEKLLEAFE

AAIAAPREVDHVVVGLDPAILHRSLQEAEGDVFWREDSRFSLLTHAIDAAEAGSESSDGA

QSILATIKSATSPQAAVDAVTAHFIEKLARMLLLEVDHFEAHNSSIASYGIDSMIGAELR

NWIFKEYRIDIPFQQLLAETLSIDKFAKQVCAAAGVEFA

>SoG_04666.T1

MKSQGTGGSIVLVASITSHTVLPQHRMSAYSASKGAVRQLSESLAVELASDRIRVNSISP

GFIDTEMTQEVRNAQPHMNALMNETPPLKRIGTRDDLVGAVVYLLSDASSYTTAGDIVIT

GGLHVQMALDCKLAGI

>SoG_04667.T1

MDHHYQQQQWPSWAYAAANQSYSNYQTYADYPSYAHESMDPYSAHHHQHHLVHHHQMSRT

TESKPRLSKEEVEVLEAEFQKNHKPNSSTKKALAESMRVDNARINNWFQNRRAREKKENN

IRQYEARQRLEKEQHDADSTSKLDMVESRGLVASSAPFPGTQGSNWTSSKSGTPADDETT

EDAHSAKSEERTGLSLPISLSQMQAKLEAQEITTEILYTPTSENVSLSGSEQLNKSNDGS

GYFLSQGQKYTNDESSERTPCAPALRFPRHHIFPSAATDSPYGSDQDVEYLSQSSPGTSS

ARSPSADGDTTLRSPEDPLDIASRRNRRPPPLAIGGSRSYTGSGPRTATEHGGRAEFGRE

MRRVNSASGSVRVTKSSSVPRSPFHDRRMESLFHLNRSPVMTATKNNVAPPTPDTPIVAN

QAQNGEALTTSNHMSNGTKIQAGGFLAHDPTLRTPPTTPGIGDSFFSLNSAYNMSIPDQP

LVTPGISGYSSDFDVSSLPISAPQYVSAPTSSSGQSEASSCGAPMGPAYYGFSGGNAEYN

WASSSAGRVSPNPHSVQFMNMTTSNFN

>SoG_04671.T1

MLPIQSLDAQALRPGAPPGALPGADTAEVRQQKPKTLPCRYCSKRFRRVEHVQRHERTHT

KEKPFACTWDRCGKTFGRRDLLVRHEKLVHLNESGKEAQRPRKLSSGTSTSTTNGPHHAS

FSEGQMETDNVTLSRVQPQQARPHLQMSDQHQGHPQQPQAQHQKNQHYQAQLQQLQSSHL

GQQQQQQQQQQQRMYNHSGAKQSMGQIPTSDSRIPRSAACNLDLLSDAALASEVNPMQPI

LNDMSPPHTAHSRVKSYGESIGAFSDRSRDDASGLSAGFPPQAPQDAYNDYNLFMDDFAS

SNHFLPPHFDSGQQLGMWPGSANGGQRWGSKPPSHFPSRIGSMVPEGRDGEGQPRSHEDT

SRPPALRVSVMDHTLIKNRLDEFSSVLPKDFVFPSRHTLSRFLEGYFSGFHEHLPFLHLP

TLTPAETAPELLLAILAVGAQYRFESNRGYALWYAARAVALEQIRRRHSSEVHALLPTPA

AYSPHSTRPSPSMTYRHSFASAQNERQTTQDTHREPYSPNTPQARMETIQSVLLLFAVGL

WGVKTILQEALGLQSTLANLIREDGLHAESSHAGVNDWDGWHGLQHAAAAAHLGAEALPS

PAVSALARRERLAMARASPVDAGGRDHRARGVLKAVWPDAAGVADAPVLAWELRADPRIA

PAHLPAQADLHRGRVAVRHAGDAQAGRRGGGGTGSPRVADELRAPPSAPASRDGPVRDRA

GLDGGFAGVQRIGAAAPRVHQAVHGHAGEQVAGDARLGTGGVGSEQRATAHAQPETAPGG

VPGGARALDALCNFECAILLAKWLLTLGTIGDAGPQATPEEKNLLETIRRMLDETEFAVP

IDPSLGGGGGGGGMPGQMEMVTSDSAKLRQLAGAVVRLWAETFKGTHIFDMVKVMGMSMD

GFAALVEKPRDRRLGVDGSMG

>SoG_04680.T1

MRPEVEQELAHTLLVELLAYQFASPVRWIETQDEILVGKTAERLVEIGPADTLGTMAKRT

LATKYEAYDVAKSLQRQILCYSKDTRDIYYQFDTPPEEQAGEGDTLPPESSPAPTAPQPS

TAEVATNAAVPIAASAEVVDEPVKAIDIVRSLVAQKLKVPLLQIPINKNIKDMVGGKSTL

QNEIIGDLTKEFGSLPEKSEDIPLDELATALQATFDGNLGKQSGSLVARLVSSKMPGGFN

ITATRNYLHTRFGLGSGRQDGALLLAITMEPASRLGAEAEAKSFLDKVTTEYATNAGVSL

TSASSALETAGAGGGAGVMMDPAALDSLTKDQREHFKQQMELLARYLKIDLRRGDTALVE

SKVSEGVLQSQLDTWLVEHGDIYASGILPVFSPLKARKYDSSWNWARQDALQLFYDISSG

RSPSADRDTMNRCIQIMNRVDRRLIELIRYQVKSCPTLEGKEGHERAQQFGKRLLEKCEE

VLDEAPVYKDVAMPTAPRTSVDEQGELTYTEVPRESCTTMEHYVKRMARGSSVSQYGGRA

QVQTDLLRMYQLIRKQNLDMDSKTEIKALYGTLLENLADGAPGELSAQLESISEVVGVKA

TPEAIPFLHLRSKSDQGWEYNKCLTDTYLGCLDDAAQFGITYEEKTALITGAGKGSIGSR

VLQGLISGGAKVVVTTSRYSREVAEYYQSMYAKCGARGSQLILVPYNQGSKQDVEALVDY

IYDAKNGLGWDLDFIVPFAAISENGREIDSIDSKSELAHRILLTNLLRLLGCVKRSKESR

GIETRPAQVILPLSPNHGTFGNDGLYSESKLALETLFNRWHSESWSNYLAICGAVIGWTR

GTGLMSPNNLIAEGVEALGVHTFSQQEMAFNILGLMSPTVVELCQMKPVYADLNGGMQLI

PNLNEVMSQLRKDITTKSETRQAVQKEKRLEYEIAHSASSSYTSQQVERRANIKLGFPHR

PDWETSVAPLHETLHGMVDLDKVVVVTGFAEIGPWGNSRTRWEMEAYGEFSLEGCVELAW

IMGLIKHHNGPLKGQPYTGWVDSKSGEPVHDNEIKAKYEKYILDHAGLRLIEPELCNGYD

PNKKQLLQEIIIQEDLDPFETSKDTAEQFKREHGDKVDIFCIPETGEYTVYLKKGAALWI

PKALRFDRLVAGQIPTGWDAKRYGIPDDIVSQVDPVTLYLLVCTAESLLCSGITDPYEFY

KYVHVSEVGNCVGSGTGGASALRSIYRGRFQDQDVQNDILQESFSNTMSAWVNMLLLSSS

GPIKTPVGACATSVESIDMGHETIVQGKARVCLVGGLDDFGEEGSYEFANMKATNNTVQD

AAQGREPSEMSRPATTTRDGFVESQGCGMQILMSAQLALDMGVPIYSILAYSATASDKIG

RSVPAPGQGALTTARERASKFPSPLLDIKYRKKQVERRRKLIAEWKEEELESIHDELQAI

KAAGDTSFDETAYMEDKLGHINKEAIRREKELACAMGNDFWKEDPTIAPIRGALATWGLT

IDDLDVASFHGTSTKANDKNESSVVNQQLQHLGRTKGNAAIGIFQKYLTGHSKGAAGAFM

FNGCLQVLDSGLVPGNRNADNIDPVMKDFDLVLYPNRSIQTDGIKAFSVTSFGFGQKGTQ

AIGIHPKYLYATLDQKTYANYCAKADGREKRAQRFYHDGLIHNRLFVAKSQAPYTDDQMS

EILLNPTARVTEDSAKNLVYRP

>SoG_04687.T1

MTGRRDESERRQVCDSCRFRKVKCDRRQPCENCHIDGLRCQYLHSIRRKGAKTGQGRRQS

QLRRGLADSGKSVFQVMTTTMNQDATASPRVDTGNDCRLDDHPAARAQIQARGTTQQYSN

ALNTPSSLSDQLSEKTDAEPSGLLSRSLIAHIQLFLKALFPIMPVVDGDELLADAARLEE

LPPPRYALILALCAATRMQLGMDNVLHSQGPHAEVEVPLEPKLTGEMLACLAETSLRQYS

AIDDTTLDSLLASFFLFASYGNLNKPRHAWFYLNQSICLAQSMDMTHEAGYHHLPDAEQE

KRRRIFWLLFVTERTYALQHRRSVMLRSRIMKPRIIDSDCPVVMNDFLNHIRVFETLPWS

LYEWHDSGDSTQRKDLSLVPTINDRLCKIQAEQSVIPSQRFDTLVTQQWLRVCMWRLAIG

EKPSQTSLNFGGVLLPPTLPVDAGKIIMSALGAVSPRLRDCHGIGMEQKLFNIGVSLADC

AMLSNWNPCSSFEIGPQDLLYAVIGALSTARGSPSYLLPSLLQHSEKVLRLTDPTPHIDL

RLETPQGTQPVTRNAVVEDVSNVSTQGEEPVDSLSWLSEDELGLLDINTPSFAWDDAGLP

GHQVEATIGLSPG

>SoG_04705.T1

MSEEGSGSPTRTKAQTALAPVVEKPSTEPEQTDYQKKKANFITRTFWTFVMFAGFFAALF

MGHIYIIGIITAVQIVSFKEVIAIANVPSRARSLRSTKSLNWYWLATTMYFLYGETVIYY

FKHIVLVDKVLLPLATHHRFISFILYVFGFVFFVASLQAGHYKFQFTNFAWTHMALYLIV

VQAHFIMNNIFEGMIWFFLPAALVITNDIFAYICGIAFGRTQLIKLSPKKTVEGFVGAWV

MTIVFAMGLTSLMSRSKYFICPVNDLGANIFTGLQCDPNPVFFPHTYTLPQYFFLPENAQ

FSITLAPIQIHAFFFGTFASLIAPFGGFFASGLKRTFKIKDFGHSIPGHGGITDRMDCQF

IMGFFAYIYYHTFIAVYKVKLGSVIESAINGLTPDEQIELVKGMTRYLTNQGVVSQSLLD

CVDQAASNISS

>SoG_04752.T1

MANHPVVQRTAPIAIAPKPPRPEPPASRTDSFQRLELGASLHARSSAPGSMSRSVSASLN

GASVPPCHACRHAGTRCTMSSSDDDEGCIPCQVNGCDCSLVTSSSPQSRKRKLNGEQPDD

SIGKRSPGVSSRRRHPNSSLSSTAASSSFLEDMANVGGPTMLKRTLGLQDDRYSQYIGPT

TDFEPSLINLSSFDPHDESLLSRGTLRRVGDNDTFLMLPDYNTPGHEHVTQDLDAIEAVV

APHGRKLIDLYFRVVHPGFPIIQKHVFIEKYERSYREFTPPILAAVYILAINWWHHSEEL

SNSPKPNVRELERIIRSTLSDAMYRPKLSTVQAGLLLSQRPEGDQWAPTAQLVAIAQELG

LHLDCSTWKIPPWEKGLRKRLAWALYMQDKWGALVHGRPSHIFSSNWAVRPLTANDFPDV

EWDENDIEEKQDVERGRLLFTRVVQLTEILAEILDTFYTLNAMQSVTEAGPQGTQLVLSL

AKPIQLKLKDWYGGLPQSIRLDSSYGTGNIQNRLSSVGYLHLAYFATEITLHRRIIRSLA

STASTVDPYVQHICRSAAKARLISAMDFVNRLTPNHLRAFWYFASKTNFALIGTFGSLLW

ATSPGREEADWYRRRLGEYRWTLSVSSKPGESKGLTEFAMTMLDISTGLLKQLPEKPSMS

RSESTSDFAPMSGPGSYSGPGSIIGHFSNVPSRDTSNAQSPQSEDYDDSSDEEMDDSYAT

RM

>SoG_04761.T1

MMPPAIDLKQEDSSRDSESPGEVVQRAHRTCEKCTRTKKKCDKALPSCSRCTRLQTPCCY

DFVYAAPATTITEPTAAASILDPAAFDVTVPVIMSALSTRNVNWREAVDQYFSNINSWLH

VVHPTLLSSKLDSSSTSKNGSTSGESLPPDPETALLVLCMHLVTLYADSGRSQATDGQEM

FGNAEYIIAKRLFGLARGFNPPSLVLVQCCILLAVFEFGHGNFARAYVSVGDAYTMAKFL

GIRPGEYVEAERNDPIDPTDEERRSVYWSMFVLDRLLHVEEDLLWKPLHLPPHSSTDLLP

TFNVIWDPTVNRTITTSNRYPASISPSVPLGPFQRNCQCAMLYARALSWELDTYRPESQP

PSVTSFQELDFATRSLIEAMIAQAPRWGDHYSCFATLTSLLLLLYRRILQTTNPATLAEQ

MSSDVEVAKAVAGVNFTVRIIADTTSDFNEYLSHRPELLAPCSPVTPFSAYHCLRTLSTF

QSIIPEADARFHDIYSSLHFYANRWGVGGEFLALDFALPPSSFLFAYLFSLSLSLSLSLS

L

>SoG_04773.T1

MYQPQYTVPQQNMQQVQYQMPGLHAAAMAATAAATGPNYPYMHQESEISQTSPRMGGAGK

KEGRTSPRMGTSGRRMSQVTSPGVTGAQGMINHAAGRPGVAPPQMPPGQGMQHPASPEIP

APPSGAEESPLYVNAKQFHRILKRRVARQKLEEQLRLTSKGRKPYLHESRHNHAMRRPRG

PGGRFLTAEEVAALERDGKLEEITGVKPDGSNDHSAGSKRKSEAASTTSSKKAKTDSPED

NDEDEDES

>SoG_04788.T1

MATTTIAKLSEVDLRTRREKHHEADVLVVGAGIFGCAIAYGLANQGRSVLLLERSMAEPD

RIVGELLQPGGITALKKLGLGSCLEGIDAVPCYGYNVMYHGKETIIPYPPLDETGQVASR

WYGSSSEGKKQSGRGFHHGRFIMALRNACLQHPNISVVETEVTNTVCGDVSDQILGVETL

TMVNKETREKVKDCFFAHLTIIADGYASKFRKDVTNEKPIVRSKFFALELIDCQLPSPGI

AHVVIGNAFPVLLYQIGSRETRALIDVPNGLPEASPANGGVRGYINKCVLPAMPSGIREA

AERALSDGKIPRSMPNSWLPPSRQRANGMIVLGDAINMRHPLTGGGMTVAFNDAVILTDL

LHPSRVPDLEDQHAIHNAMETLYWRRKSLTNIINLLAQALYSLFAADDRLLRALQRGCFG

YFERHMTDGPAGLLGGLIHQPAVLFYHFFSVAFLSIWMHACDMMSSVLDIWKLPLAIIDG

VLIIWKACVVFLPVLFREWFM

>SoG_04795.T1

MSSVDEGHDEDASAAQPQIGTEPLACVSCRSRKLKCDRTKPACARCTKVNSECVYPESRR

KPTFKRRNVRELEERLAQVESLLKGVNDAAAVAAAAGGRGSSRASVGYQATVELEQEDGG

LFPSVSTIRTISDDLSPDAQLPFGEPELNQTAPPPGVDANEWQMMGLGFSEAPPPWDVIE

ELTDCYFNNHHHMVPIVHPGRYRSAMYGGPYMQPPMCLQYIIWAMAAQHHEKYSAYSDIF

YKRSRLYFERDEMKGDGEHFITIHHAQALSLLASYEAKRMFFTRAAMNTAKCVRLINMMG

LEKLDSPDQISMSLTLQPPKSWVEKEERRRTFWGAFAIDAHASVSTGWPSLVNSTDIQTR

LPCSEDAFLTGKEETAPFLEEVYDGAEYSSFASTLVSCNIFKSILTHVHHPKASDRPDDV

FSGRFWERHREFDNMLLSLLMFLPPPLKLHGSQKDAAAIYINLNIHASVICLHHAAIEQV

EKHKLPDTIRQASEMRMRSSANEIVAIAKMAAHMTHAFKSPLCSLSFYTATTVFVYQGKQ

DPVSGLSQIDRSNLELVLRLMEAIGRIHPITHAFLEQAWNDLDRNGLSHIVRVPGLSKYK

RQFPNEELSSIPLLTRTSNVSQPGHVSILPGRLPLEKPIGHKREVSVGHAMAPGEGTGQS

ARFGKMSALLDGECFQSVLGACSRNIDNVQSITHKRKRMSASPAPDRLAGQSTPFFQPGP

DLSFSLPDRTGSSSSSSPAYPSTGTDTQQSGSSHTSPSMMGLGNTAEENRIDLRQFQGRV

APPPQWDPATEPFCFGDVTDEMVNAALGQAGGGEPWNFLTADIQWHGQGGQGG

>SoG_04812.T1

MSDILSSIYTERSHDQSPAELSGMLHKLQLKLDSWTDALPSHLRFDPAKFESATFPPPHV

FSLHAMHHVLVILLHRPFVADGHLYSTSRSVSVDCFMKCASAASNIVILLRAYHRAFSSR

RAPYLISYATYVAATIHTRIAAKRSHDSSAHNNLATCLAVLQENQETNAAVNKANMIVQS

LMNRLGVKIGNVSARTLEMDFSDASGQEDSAERNSLEAAPKSASLGRAVRKAAGALAPGS

IPQGTPESDWMDIDGIIQSFLQENDGSNLNGLRQVCPSDSNLANQFSRPAWFQQEQQQMP

MRPEYTDSIQPVLPMQHHQPQMDTAVSDGYQWHEDPLFGLNGSSLDNFAFMTW

>SoG_04818.T1

MDGAKAVGLKKAFKAYLDGRRVVSTQHEARIFFEAVQLQSEPSECLELIINGKAGLKAVR

QAVRVSLDKEYVKKHVLPFLFNFSDPRVEALYDGSFLQQLIAVVIYPPTVWDNIMTIYRE

DTILLGQSESEAFAWMCHQIVLHPGPDLSDIADDVADLLREKPLFDLPWDNTRSIAYLIQ

KALQRKNGVTSPLDDATGQGGPGGRHDNDFKDFRETSIYPSADELSSTLKPFLRTPYQLA

EVSAALRPASHLDNQFRLYREDFLVELKEDIAAVIDPKQRKRLRGEVLRGLQLCLEDISN

PQRSHAFSLNVSFTSGLLFPKNLKKEEDRLHYLLENKRVIEHGNLGMLYHEKKVVAFAFV

VRHVNNLLRTPPVISLRFTTAKDLVSATESLSNQQNLQFAIVDSPWFAYEPVLEGLKQIK

EIPLQSELFGLHKRETSNASCFQPTEYIQQFATYCRDMVAKDKKVLIVDKKYRLDQAQAS

ALLFALESPLSIIQGPPGTGKSFIGALIVKLLLQSPSSKVLVITYTNHSTNQMLEDLLEI

GINSGTISRIGSKHKASAKTLGLCLEAARVQFQPKSHQWRHIHSLKEDIRELRADLDDAL

RRLGRKVSPTDVLNFLELDDSQESHEFWAAFQPPSLDGNFRVAGKNGKALQPESMLTEWQ

RGQDAGSARHHFTPRSKAIWDIPAPERAKFTTKWHDSLRREYVDAATNIASQIQRLQKKV

DTIFEEGKCTHATSSRVIGCTTTSAAKHMTLLKAFQPTCILVEEAGEILEPHILAPLCPS

VRQLVLIGDHKQLRPKCSYALSVEKGDGFDLNRSMFERLIMEGQRHETLQRQHRMAPEIS

QLVRSLTYPELLDGETTSNRPAIRGLCSRVLFINHSAPEASMNQIRDRRDADNKGSRENK

FEAEMVLKIVKYLGQQGYNSSNIVVLTPYLGQLRLLRDMLSKANDPILNDLDSAELLRAN

LLTKAAAKLSGGQIRLSSIDNYQGEESDIVVASLTRSNNNGDIGFMKAPERLNVLISRAR

NCLIMVGNMETFLKASNGQDTWRPFFTQLKDKEYLHDGLLVRCERHPGHVELLSRPDDFM

AKCPEGGCNEPCKEPLPCGSHVCPRKCHRLADHSQVVCIEIETIKCDKGHVYRVECCRKS

KSCPTCAQEEADLKRRIKQDLDLERDRLARQEKHRQELQDIKDKIARHNRQAEIEAEEKE

ELKDIAQHRSKLEALDRARDQKEVAKKASQVSPKAATSANAGDQIGAGRQGKSPAQAEWE

LMKEDEFAKSKDLDDLMAMIGLEEVKRTFLDIKCHVDVAIQQGVSLEKDRFGCTLLGNPG

TGKTTVARIYGNFLTSMGVLPGNMFEETTGSKLANMGVSGCQKLIDSVLNQGGGVIFIDE

AYQLSSGNNSGGAAVLDFLLAEVENLRSKIVFVLAGYAKQMESFFAHNPGFPSRFPLEMK

FEDYSDDELLEILALQVRDRFKGRMKIEDGIHGLYCRIVCRRLGQGRGKEGFGNARAVEN

ALTRIYHNQARRVKREAQQRRKKGMGSTPAPDPFQFEKEDIIGPKPSKALLQSKAWTELQ

GLIGLKSVKESVKVLVDTLESNYERELAEQPIIEYNLNRVFLGNPGTGKTTVAKLYGQIL

AQIGVLSNGEVIVKCPADFVGDVIGSAEKLTKGILDSTIGKVLVIDEAYSFYGGCGGKGH

GTDIFKVAAIDTIVANVHSTPGDDRCILMLGYREPMEEMFKNMNPGLARRFPLESAFAFE

DFDAMQLETIFRLKLSKQGFGATPKAQEVALQMLERARNLSNFGNAGQVDILLDNAKVNH

QKRVSTGLTKRKETLEAVDFDEEYDRGSKANVTHLFNDTIGCEKIISLLQGYQDTVRQMA

ALGMNPKESIPFNFLFRGPPGTGKTSIARKMGKVFFDAGILASDEVVEVSTSDIIGQYIG

HTGPKVRDALDKSLGKILFIDEAYRLAPKGSGGQFAQEALDEIVDACTKEKYHKKLIIIL

AGYEQDINHLLTANSGLSSRFPEVVDFQSMNPSACIDLLVKDLSKKKTEVETSGSGNSMA

YSCFSDPDFCDRVQVYFSRLCRLESWASARDVLTLSKSIFNAAIRDSGSGCVEINEAMVD

NELKMFVEEKESRNKAKTPSRPRVPSTALPTRSKDVSAVDTAPPATSTKVRTEAGTQSKT

GEESSEGHKNEDSDQQSRPDTDPTVHDVKPAVRDVGVSDEVWDQLQTDMQSEKQREEEYQ

AKVKAEKEAADEMRSMIVNELLAAEEKQRKEQERRRKELLEKQERIRKEILKEQERRREE

EETRRKLHHRGLCPMGYQWISQAGGWRCAGGSHFVSSHDASKL

>SoG_04836.T1

MAEAREGRRPLAMDPPHLTEFASQSYFAKYKQQQERQLQQHEIRDAPAFSIPQAPSKFIL

PLRESKAPEQQEAAKPAEQKREKRSIRRFMPKVSALHPKPVQSPSQPVAPTVQSRTSIES

TTNSIPLDQLFLALPNELQVQIISSLPLNDLLNLRLASKSWHSLITLNETPIVRYHLETQ

IPAYALRLYPLTEPSDATLHNLCGLWHRLHVAAKLAHMMCVWITKDIFLQQTEAQKQAFA

PQTERMRRRLIPLLFTVFHFFEMYRKLHLEHIEKNGGYGLRREVFTLNPIETEIMSMYDD

QTLLRVHEAFPLVISSFCRRLRPPTYVGRVERSLRGYLREKPADDVHSAILCIGGLREVG

KLWETKGYNSRRAAVDTWYNSLTKDAPIGSPAKQRRGLMSFARRKSTGQDREGRRFSLGD

SSQRNRSPSGSIDCDYAFDPNWVFNTSLAAGAPMSPLSRENAQQVLDDLPVLQQIWLTTA

EALILERKIVKSPHDIKRNQHVLLDLIREDGLNEEDEWLYGRHAPDSVKPSVAAIHDDMD

>SoG_04847.T1

MPSATGQNWEKYQKNFADDEVEEKKITPLTDEDIQVLKTYGAAPYGTAIKKLEKQIKEKQ

QSVDEKIGVKESDTGLAPPHLWDVAADRQRMSEEQPFQVARCTKIIADDNGDESKSKYVI

NVKQIAKFVVQLGERVSPTDIEEGMRVGVDRNKYQIMLPLPPKIDASVTMMTVEEKPDVT

YGDVGGCKEQVEKLREVVEMPLLSPERFVNLGIDPPKGALLYGPPGTGKTLCARAVANRT

DATFIRVIGSELVQKYVGEGARMVRELFEMARTKKACIIFFDEIDAVGGARFDDGAGGDN

EVQRTMLELITQLDGFDARGNIKVMFATNRPSTLDPALMRPGRIDRKIEFSLPDLEGRAN

ILRIHAKSMSVERDIRWELISRLCPNATGAELRSVCTEAGMFAIRARRKVASEKDFLSAV

DKVIKGNLKFNSTATYMQYN

>SoG_04893.T1

MASKTDTSKYIFNHTMIRVKDPKESVKFYEFLGMKVVRKLEFPEAKFDLYFLGYDAPGAL

YHGKHTMGREGLIELTHNYGTENDPDYSVQNGNKEPYRGFGHTCISVDNIQAACKRIEDA

GYKFQKKLTDGRMRHIAFVLDPDGYWCEVIGQKPLEETAHITETDTSTYRMNHTMLRVKD

IEKSLKFYQEVLGMSLFRTNKSEANGFNLYFLGYPGVLGLPKEGDSQANREGILELTWNY

GTEKDENFSYHNGNDQPQGFGHICVAVDDLQAACQRFEELGCNWKKRLTDGRMRDVAFLL

DPDNYWVEVVQNEQIAGKATDF

>SoG_04905.T1

MAGSEDAKEPIAVPQAGAGAESSDITTQENVSPRSQVARQSSTKNQVRHRASVACASCRD

RRIRCVVPQGQAECNQCRRTGTECIIKNDDERRRPISKAYMSSLSDRISMLEGMLLDKGV

QPPPAVHPPKTRHDNHSKAATEDQKPAPRPITTQHTSPSQGVPSPPDSRNEDFMLGGDMD

HNDAMMSSSSQILPGGLSKEDSPFRMLDAKQEDIVYRLLATKGNLSFDQLSGRLRFFGPT

ANSHVYAESPDQFDSREPPEQVRRAERIIRSLTTSTHDYLMNHFWEYYNSVLRIIDREAF

EADRDSQNPKFYSSFLHITVLAMGYRGADMDREDMKKISLGNRESTLHREAKYMLDIELE

RPGGIPSVQALLLLSDLECGVGRDNTGWMYSGMANRLAFDVGLHLDCRTNDMPEQEIKIR

HMVMRACTIYDKYWALFLGRPTSIKSQDIGMDLLQSAFSQLTSAFGEQTPVERDMTGEIY

EQLIELMDLAGRIVETRDHNVVKNSSASSIDRNNIFAIGEAEDNAYLHVVNLDRQLQNWY

RRLPDHLAWKPANVKTAPYSYFLLHQQYHVSMILLHRPWAKYGAITGDGASTGSHPSPDS

AHLNMGPDSAPQHPGAGAAVDHSMGMGDPHSMVHDSRTSLSRSICTQQAIRVARIFWQHR

QRFDGRKIFVTGIQHAGTASIALIAALAYQRNDADRRTYLGYLEILSDALNDMSYTYHPA

SRMDSLLKAVLAQIRTSMGEPPRSSGGIRGGLPLDGFSAGSASRTRTSGASDMTAPVIPS

RREAGDSDFVNPAKRRRPGNSRRASEYARPPPPFFPGAQPTPPHSFTAELHQRTNSQAEP

MMFHVGSADAAQMNLDFLGGSAVDMDAAGDDQMECSRHHGNGDDMNLMGHGSDATGVGWG

MGSMHSDHHHHHQHQHQHHHTFDPSPMGGGADWNSGPAGLSASSVLNHSAALTTGLMSTS

SALGATPRTAVDENDDDERGAHAMLNGGMSPGGNLNGLVQSVEKDGDVSRNHELDFFSFT

>SoG_04915.T1

MVQLTNVALVALSAIGAVAAPAEKRQQGTPSSTGQHGGYYYSWWTDGASPVTYENLDGGG

YRVNWQSGGNFVGGKGWNPGNAQRTITYSGSYSPNGNSYLAVYGWLQNPLVEYYVVENFH

PEYDPSSAAQDLGTVQCDGSTYRIGQSTRNNAPSIEGTSTFQQYWSVRLDKRVGGTVDMS

CHFGAWQKAGLPIGTHNYQILATEGYKSSGSAEIHVQG

>SoG_04929.T1

MAGTGGDATLFEDNFTITDFDQSKYDRVARISGTSTDSQTVMQLDINTELFPCSVGDGLH

VMLATTLSLDGSKDEEKGWRDVGKGGDAPATSADLFDYVCHGKIYRFEETYDGNTINAYI

SFGGLLMSIQGPVKKLTPLRVDNVYLLVKK

>SoG_04935.T1

MAPTIPSSAQGGMFHTFQGVTPRKPAAESSDGNKPVGSTGSKRITTPHACAECKRRKIRC

DGQQPCGQCLSSRAPKRCFYDKHRQRVIPSRKTLEALSQSLEECRSILKRLYPNHEVSAL

LPLSRQELLGMLDHSGDSVVALPSPPLNTSPTKTESGSPLGLDSEGSLASLEPLSSRDMD

WDEDQRCHETLPADTDDANCLSLHPDRQASYLGASSIKAALVVMLKVQPQLRSTLAPPLN

SVEMANNLPVIRQRSATPKDSSRIPWSWKGQTLIDAYFKRIHVFIPMLDEAAFRADYLEG

QRFDSPWLALLNMVFAMGSIVAMKSDDFNHINYYNRAMEHITLDAFGSSNIEMVQALTII

GGWYLHYINRPNMANAVIGAAIRMASALGLHRESLAQGGSDMAAAETRRRTWWSLFCLDT

WATTTLGRPSFGRWGPAISIQPPEFGVNQARDSAQHAGILPLIENIKFCKIATQVQDMLA

STPLLRTEDRCNMDGQLVNWYSNLPWLLRTTEPCAEPLYIARCIMKWRYQNLRMLLHRPV

MLSMASSGPGHQVLEQDLAAVETCREVAKQTIEDIAREWTRNQMSGWNAVWFLYQAAMIP

LVSVFWEWNSPRVPEWQKQIETILELLDAMEEWSLAARRSKEVVWRMYEASRLPSVVNGT

GNASGPSLPPTALTTTVGPDMLSLAEGDLHMSPIGLETDDMMSLLDQHGLWDLDGMQWGQ

QSQGSLPVDAGLCGLGEGMMQMDYGAMGGQPANLDPSFFVN

>SoG_04969.T1

MDNDVGPSQALFEETREVTTAAKRVLKKIDRVIIPLLFVTYILNFADKVILSSAAVFGLR

EDNYSWVSSVFYFGYLVWAYPTTILIARLPTAKYLAVNTFFWGAVVGTTAACSSFGGLGV

AEATITPAFMYLTSTWYTRDEMPTRVGIWFAGNSVGGLLSSIMAFGVGHIDDRVRPWRWM

YIILGVTTFLWAIPMLILLPDSISKAKFLDEGERLIAEERTVVAGTGSTENTRWRFDQFK

ECLVDPKTWLIFSVELLTQIPNGATQNFSNIVVKSFGFTSLQSTLVNIPYSLLTAAVIAG

SGWLAGSLRTVNCFLIIAVIIPPVVGSAIMYHRDRVPHAVHLFAYFLMAPGPAAMPLNMA

LVQSNYRGVTKKMTITAMLFLAYCAGNLSGPHFFRDAEAPTYPSAFRTIMICYALAMCLA

MVHRGYLQRLNGRRARDEGFEGSMASAGVVAGGKVPDPADTRDVAERLGELALRPEDYED

VTDWKAVGFRYRL

>SoG_04989.T1

MLLDLFYSFGNCLNCFPGSPTLKINNRSFKIQRLLGEGGFSYVYLVEDTSSHQLFALKKI

RCPFGAESVQQAMREVDAYRLFAHVPTIISAHDHAVATERGADEDAKTVYVLLPYYRRGN

LQDMINANLVNHTSFPERKLMVLFLGVCRALRAMHRYRAPVERMEMGREDDESVRTGATR

GKRTEEEEEGEQERPLMEGESQISNGGKVQSYAHRDIKPGKLSPLAN

>SoG_05007.T1

MPGILPMKVIKVGNSAQSRIAQACDRCRSKKIRCDGTRPTCSQCANVGFECRTSDKLSRR

AFPRGYTESLEERVRALETEVRELKDLLDEKDEKIDLLSRMHGNRPVSCAERKQSNASGA

SPRSPMDTGSSPPLREDTFRVQASPLLLGVEDSDSFFMGPSSGRSFVEAFKRKIQETGKP

CSDFTPEAFLHIQGSQPLITGEPPRSMKIPPRLFSDRCVNVYFQEWAPLFPVLHKPTFLR

TYEEFVADPEKVKCPSKLTQLYLVFSIAGLSLDSPDLPQLAACERQWTKLLESMVMDNTM

KTLQCLILAILYCTIRADFRRLLHYKALAVALSHRLGLHHSQKRFSFGALTTETRKKVFW

TLYTLDCFTAATLGLPKLLKEDDVQVEYPSDTDDDYLTEKGFQPTLPGESTRISSALALF

RATRILARVLEKNYPTATSYELSLQQMSQLESELDEWYNNLPAHLRLNFKQDKPSTDVTG

SRSPLLALVYYYIRTLIYRPAVGSSLGPKAAPALLAIGDASKHIIQIVQLLDERNMSFSF

CLNKADLLTLCGMSLLYQTIELKQDGRLMREDERLVNAVLKMLCNGAAPGSFAFAKVARL

LITVEDASVPGAVVEPPRPRSDTGASPASKSRKKSWSRTSRLAAVATASESDLLQQQEKL

RRMTMPSLASHRPEFYRAAPARQSFESLPSDDAAQAQRQRQPGQGQYPAIGSDGTPGRSL

SSSPSQARQMTLPTVQTHQLPTKVAAVSEWEALLGSMDGGINNVYDAIYGGPPLVEAAVV

ETISPANAADWSPDSWDLATFNLTSGPEFAAGAPNAPQSVLSLSDESLSSGEEIAPSELG

LSVSSADYNQMIAATCGNDMTGCEGERYQMENFEPFHL

>SoG_05010.T1

MSHNQNPYQQGPAQEGGYGYSQQNPYGQSNPYGQPNPYAQDDQYELQDYSINQNNDQPST

SAPHSQQEFLNRVQNLRNEIKSLTNDIDYIAQLHQRALSSTDGQANQQLEQYVTQTQIRN

TAIKDGIKGLERDLARTSDGSRNTKSTQLQSLKTFFKSELDKYQSIERDYQQRTREQIAR

QYRIVNPDASEQEVEEATQLDWGNEGVFQTALRTNRTGHASSVLGNVRARHNELQRIEQT

LAELAVLYQELATIVEQQDPVVENAENNAIQTNMNIEKGNEEVHKANEHAKRTRKLKWWC

LLIVVLIILAIALGVGLGVGLAKSATGN

>SoG_05015.T1

MADIADQHEQTSPNELDDAQNGAAANESRGVKRQRAAPDDDDDDDEKGNRERRKIEIKFI

SDKSRRHITFSKRKAGIMKKVSLVSPSPFCPDAISAVSPLGDNPGSMPSDQRDIVLQTGL

VYTFTTPKLQPLVTKAEGKNLIQACLNAPEPAAGNENGVDGGDQVESPEEPANQHLPPQP

GRPGMPQQPHMPGGYMPPGLPLDPQSAMAYQNYMHGRGGYPPPQSGMPPSGHHQS

>SoG_05021.T1

MASMSSQGPSPHPSPYDFDSTGDTDESWQYLDFNFSSSSSGLASVGFVPSPSTGSLAGYA

VVGHMSTPSQASGASPLPTAEYAGQDDFFTTQLPSSVQDITAGDFIPPTSMPPGWTQDIP

FLTPQAFLFPTEGSGDISQPVMDNINVLMDSFQSGTFSPTRGLEPGPSVQQGSDAAEPLT

QASLNNLQRLDTNEPWRPASSENSFEGIISFDGATSSSPPMRTVSSSPEFPSAKPTSASK

SPSRNRKVAADKVDKRKKKDDVSGNFVIVTPTSISAHSGRPNPYECFEHMRATQKGRKGP

LASATKKNALQVRRLGACFCCHSRKVKCDKERPCRNCTKLAVAVPQVVCWQFQDFLTVLF

PDFLRYHLKKEEVAKFVVDSVDEFRIGGIAHPCEVELYSGPTFASTLTVNANFFTAKSVE

ALQHWHLIGGSDRVDLMTNGSAPIGVDLSETTARDELRKRTKTYIQQLIKEPNFSAQVTD

NLRSTSLPRMVLNLAQRFAEQTDSAIVKRSLSIYAMHYVISRHLCITSQSIFALQPTGLI

PQDSLWVTPRVLARQIKAILDEHMQKEMQHVFDLFSKSLKPKHRSEWAPCLAAFLVLCMF

MEAVETATDSFVVMENEVNLRKGDAPKFKRSFALNICKEVENMPFKQFAYQFHNIYQTHS

KDANTKSFNPLFDSSFVENGELDRPAKELVAGLQELYYGETWRDMQFLSDDDLVISREEH

PFPRDPSLVYTGRLLAKFLLSFTNENAIFGSRI

>SoG_05026.T1

MPETVAKNATIHQAGGGASASQRVVETIHSVTLPRLKAIYTKYAGGNERWNKEQTKAFLE

DVQKHPVGFEEVAQKDEMDFNGFLAYIASHHASLTLEPKSEDLSWPLSAYFISSSHNTYL

SGNQLSSDSTTESYANVLLRGCRCVEIDVWDGDESDSDSSDDDEKGSKSQALPPKKTKGT

STFGKLKSSLPESLAAKLQKTGLGKKAEEKEVECSNPSAAPDDEQQKHAEGAPGVAVVEP

RVLHGYTLTKEISFRDVCVAIRDNAFTATDLPIIVSLEVHCSPAQQALMVNIMKEIWGEY

LVAEPETEPAALPSPGELRKKILIKVKYAPPGSSAPANDSEDDDRLSAEPLKKEEKASKS

SKIIQELSRLGIFTKGISFKSLTQPEASMATHIFSLSEKKFLDYHEKQREELFRHNRDYL

MRAYPSGLRIRSSNLNPPIFWGAGAQVVALNWQQTDEGMMLNEGMFAGTGGYVLKPAGYR

PSLKTKTAPNEVTRRKLNLSITFLAAQFLPLPQGDTRASGFEPYIKVELHVDGSKAQQLG

EHLKDKTDGHEREGEYKERTKTHRGCHMDLGAEKLEFKDIPHLLEELTFVRFTVRDDEML

RDDLAAWACVRLDRLGEGYRFVHLIDLEGKLTQGALLVKVEKTFL

>SoG_05029.T1

MDRVLERTPEGTSPRTFALNHLRPKSSFVGCSRISEYELMGKLGEGTFGEVHRARSRKNG

NIVAMKKIIMHHEKDGFPITALREIKLLKLLSHENVIRLEDMAVEHPSRPNPPPADKRKK

PIMYMVFPYMEHDLSGLLDNPSVHFDEPQIKCYMLQLLKGLGYLHENHILHRDMKAANLL

INNRGILQIADFGLARHYDGHTPTPGKPMGEGRRDYTGLVVTRWYRPPELLLQLRQYTTA

IDVWGVGCVFGEMLKGKPILAGESDSHQLDLIWDLMGSPTDEKMPGWKQLPGGHHLTPRS

RPGNLDSKFKGFGQGAVSLLAELLRLDWKTRINALDAMDHPYFKAKPFPAKPHEIPTFEE

SHELDRRKFHDRKANLPAAPRGGTVGVGPDVNGATAGFNSGDGFNGRNGMNGGSRHRGYD

DRRQGWQRDRGPRPPPGERPPYRDDADRGQRNRAPPRGGSDGDTYIPSYQGEAPPRRRDD

RPPRDDWRARGGRDERRDRDRRTRSRSRSPMKDKPRDRERDRDRDPYRR

>SoG_05052.T1

MPRSSHQDLYWKRYDAEISNDDLSLPKATVQKIVSEILPAQNGVAFAKEARDLLIECCVE

FITLISSEANEISEKEAKKTIACDHITKALEQLGFSDYVPMVLEAAAEHKEVQKVTQLRM

FVILMPLLVLTSAQGREKKADKFANSGMSMEELARLQEEQFAQARSRHN

>SoG_05058.T1

MSATTMRSVEELPVSPPDGASMLSDVLMGDSQMPASLDSRSTGRLNGRWNERHERHERQK

RRTSSSASTASFSSTDARMLYNQRGERVYIGSAASISFLQVLRRVVSEQIGPSPFSHNDK

AETMLEKASPDQKANEVSTPPALLPEITIAEKQDFARCCFAVTEGFIDIFDASELDSLIG

TTQAHSPMAPLSPMKRACGDLIISIGAQCKSISTAHSVGLPYFREAQRLVLAGMLEDPDL

DMVRAHLLMAFYLLGECRRNAAFMYLGIASRAALALGLHSRDSYSNISSPGDRLRLRLWM

SVRIMDKLVNSILGRPAATAGVYSDMRSVFEAFKTTPKTPAAEALSAAHGIVCIINDITR

KVYEKKEITVKVVEQFLDDIEAWKRGLPTALGTNPGSMDVSGTPSSQGSGSSLGNVHVSC

LYYFAVALVTRPILVSGLTTKATFGHQPAQLAGACLDSATFLAETCAEAYRSNLLQNNMC

IMKALLFAAGLILGFEIFTKRSIDHEIEAAFAGTTEVLGFLAASSPQAAHYLDILTSLSN

AALKRKASTRKNRYVSRIFSVGSATAEPPRLMEPPQSWEESLFIEDLPPLQGEEMEGWSG

GLGQMTGEGLSLDWDSLNISQWDSFPFQGAA

>SoG_05064.T1

MLSPPHVVPVTTSNDPSYRLVQETAVADIQSRIGDEPYTLGLFDTAGQEDYDRLRPLSYP

QTDVFLVCFSVTSPASFENVREKWFPEVHHHCPGVPCLIVGTQVDLRDDPSVREKLSKQK

MSPVRKEDGERMAKELGAVKYVECSALTQFKLKDVFDEAIVAALEPPAPKKKSKKCLLL

>SoG_05080.T1

MRSSVLLSSVVGFGLVAAQSVTTTIPPARGTQLSSTAIRVKGSYDGGMKYFDRSTRVCQE

QTETGRDDAMFIVEAGGTLSNVIIGPNQAEGVHCLGTCFLNNVWFKDVCEDAITIEDTGT

GYINGGGAFKASDKVVQLNGGANLEMKNFYAENYGKLVRSCGNCKNNGKARKIVSLLFSS

STLSFLPTETLKKKVSSLMSHETQTRPPKLTSAPLQTLNNVVAVQGGVLCGINTNYGDTC

TIVNSCQRQGKFCDLYTGNNSGKEPSKIGSGPDGKFCKASGQTKNC

>SoG_05082.T1

MGEPGPAEGVDTPMQDADVQPPNGMMDGLGDNDDTVSDQGSDYGSDDDSQAIDLESVRRR

GLLPTGVCYDDRMKLHMNADFSPNSFHPEDPRRIHRIFSAFKRNGLIYLGPPAEFPRIIK

DNPTKYMWRIACREAEREEICLAHDPGHVDTVESWNDMSSDQMRELTKQYDQGRESLYIG

SMSYQAGLLAAGGAIETCKDVVSGVVKNAFAVIRPPGHHAEIDHPMGFCLFNNVPVAVKV

CQRDFPELCRKVLILDWDVHHGNGIQNIFYDDPNVLYISLHVYQNGAFYPGQPPNPHTPD

GGLDKCGTGPGRGMNINIGWHDQGMGDGEYMAAFQKIVMPIAHEFNPDLVVISAGFDAAD

GDELGGCFVTPPCYAHMTHMLMSLAEGKVAVCLEGGYNFTAISNSAVAVARTLMGEPPPK

LKIPPINREAARTIAKVQALQAPYWQCMRAGVVDVLNLQEHNAQRLHDVHEQLYKSYENQ

VLVTPNLMHARRVLLVIHDPPGMYAQPDAIDRHLEPHNGWVVDGVKEYIDWAIGQNFGVM

DVNIPGYITREEDSDPFIPALRENDLQEQVRVLVCYLWDNFLQLYAADEIFLIGVGNAYL

GVKLLLINRRTCPTTATEFFAFSSGACLTSICPADSKSRISGIVSFVNGNLRPVKSETDP

DLSGWYRDNSKIFVSGAHACWSDPELLRKIEKRRFGKVVRSDKASLNAMMKRHTNEVQEW

IAERMEESSHGETTEEEEEEDEDGKGP

>SoG_05084.T1

MASLLLLADPSLIHPEISRPPMAILQHHQYDAQFNHHQQPPHAYPTSPSVASSPQLNLLR

NASVSPFLHSGPTHSAPFQPAMTPVNSFQHDPYAAMDPNARRPSLGGFISPSSADFGSGD

EAKEKQRCPHPDCGKVFKDLKAHMLTHQTERPEKCPIQTCEYHIKGFARKYDKNRHTLTH

YKGNMVCGFCPGSGSAAEKSFNRADVFKRHLTAVHAVEQTPPNSRKKATTGMAAGKKLTG

YAPDATGKCSTCSQTFPNAQDFYEHLDDCVLRIVQQEDPAEAINAQRLAEVEDDDEVHET

LEKNNLPTTTQPLSMDEDDDEDDDMDDDDADDSSKSTKSPTMKKKMNPHGGILKSKGLTH

SRGGVPTLQTKRGRKHRRDYPSSWGFDKGQMTMRKRVLCAFDGPRRLHKDEMMLSTEQEV

RLKLSDPKAYITDLDVQTLRRAEAFHSATDEEKGPWISDDPTEEQRQEMQQILLATAHMP

EAMI

>SoG_05101.T1

MDALLEAAGAALGVTHDEQVKEDVADQQEAENTGNRYGSSFRSSSGNAKWFVDGCSYFWA

VAQALEQAQESIMILDWWLSPEVYLRRPPALNEQYRLDVMLQAAAERGVRVNIIVYKEVE

AALTLNSKVEPEPEPELGHAELKERLNHLTNLDLAKASEDVLCSLYGTAKDVTLFWAHHE

KLLVVDQRLAFMGGLDLCFGRWDTNSEESAYQDVFKALTRAGHPIADVHPGNLNSILFPG

QDYNNARVFDFADVQEWDQNKLDRTKSSRMGWSDVALSLDGPIVSDLVNHFVDRWNFIFD

NKYAAYEGNKYESLTGAKRGSVNGSSSEEAHEAETQIQLVRSCSEWSSGHETEHSIFNAY

VDVISQAKHFVYIENQFFITATGEEQKPVANLIGAAIVERICRAHNAEEPFKVIVVMPAV

PAFAGDLKSDEALGTRAILEFQYNSICRGGHSILERLREAGIENPRRYIGFYNLRNFDRI

NTSKTMKDVEDGTGISYEAAREEQDAYVASPIHGDESGGGELSSSDRALPAQYQSQADLI

PDSVLDTVSSCYMESGQGLMDMPWDGNADDEIDAYVSEELYIHSKLLIADDRVVICGSAN

LNDRSQLGDHDSEIAVVIEDPTPVDSTMNGQAYTASKFATSLRRFIFRKHLGMLPDQKCD

QPDSNWMPVDQGHNEYDWGSPSDTLVADPLHADFENLWINTARLNTEVFSKAFHPVPNDQ

VHTWDDYKHFYAKHFRLPSGKKPKTESSESASAEATTEEECHEEGDADDSRYDYGHIVRD

EFPGGITEVKQWLGQIRGNLVEMPLDFLSGVEDLAKDGLTLNSLTDKLYT

>SoG_05104.T1

MEIDASRSRQSSQECSYTVDGGIPDAQFDEAMNSFLDDALNTDLDLTPRRHESPDGRVQT

GEPNDQEDPVYLSGDGDSGNEGEEGEEETHSTHLSTASEPEMADEPSYGLQKSSIASRQK

RRRNDQQFERPFKRKRTELTAGYLDLWNAEIREASVRGCTDDRDIDEDGVAAYRGSQLGL

AIWSPVEKRILYETLSRLGRNAIADISAAIGSKSVVEVEDYLYHVHYASEDRKSKLRSVL

LQPSDRAAAIELSPQCCHALDEAADTLSLLQEEKEKKREEKKWGASWQFTSSMFEVVSEE

DEGKPLDERLSELGGLFDIPRWLHLSSRIFMNSAVPSNNWNFVDEEPPSIWATAMEDFHS

LTVSITRRLVQTALFVAMSRIRAKKEAEPRILDVVRREDVEAALQSLNMEDKRVDFWRGC

ARRLRLNVQDNESDVLGKERKDGIDGRDYMTFDEVEMMLGPGNPEPEALGDAHAGTLKET

PNGDDSAAEDGATENEADVESSSHSDNNPLQLTAEEQVVRDEAEEVILYSGVDFPQSTRS

KDALLNRISGERRQEEYADRCDEQASREGEVKMWDLLGRPAPPSLRAREAIEQPLRSSLT

VDGVYSVGRSWRRALHYYAEWETNECASDGGG

>SoG_05148.T1

MWGVAIDGDHLRRYDSNNQRGEPNPWHQDQPYDTPETSSNDGERDESHQAEATKPGRAGT

WGEPDAGMTTEAAAKQCYEHLRQDLDQLHRTRTGDSRIARSTSRGAQSWSRASRRSSAAT

VTAQRVASRRSRQTEHEEDQEDSDLEVGQDHDEESEDDFELGEFMREGHFEKRTDGMSAK

RLGLVYENLTVRGSGSTAALVRTVPDAILGTFGPDLYKMITKFFPALSLRRPPQRTLINS

FTGVVRPGEMMLVLGRPGSGCSTFLKAVSNYRDSFAAVEGDVSYGGISAKDQKKHYRGEV

TYNMEDDAHFAMLSVWRTLTFALMNKTKKNEAYEIPIIANALMKMFGISHTKHTQVGDDF

VRGVSGGERKRVSIAETLASKSSVVAWDNSTRGLDASTALDYARSLRIMTDVSQRTTLVT

LYQAGEGIYEVMDKVLVIDEGRQIYSGPASQAKQYFIDLGFECPERQTTADFLTAVTDPN

ERRYRKGFEDRAPKTPEELEQAFRDSQHYKDLLADVADYKAHLERTDHEDARNFDGAVQE

AKSKRVSDKSPYTVSFFRQVAACTKREAWLLLSDTTSLWTRLFIIVPCGLIIGSLFYGQP

DNTAGLFTRGGVVFFSAVFLGWLQLTELMNAVSGRTIISRHREYAFYRPSAVALARVVLD

VPVLIIQVIIFGVIVYFMTGLDVEAGKFFIYMLFVFTTTMVVTALYRMFASVSPEIDTAV

RFSGIGLNLLIIYLGYVISKPQLLSTYIWFGWLYWINPLSYSFEALLANEVSDRQLVCAP

QQLIPQGPGIDPRYQGCAIAGATVGANSVRGSDYLAVQYEYSRSNLWRDFGALVAFGVLY

VLITVLATEIVSFSNGGGGGALLFKKSRKAKKGIETEEAPVDEEKAARPEAAIGRTGSST

SGTRQGDLPDTVPMEDEPMEKLVKSDSIFTWRDLEYSVPYLGGQKKLLNKVSGYAKPGVM

VALMGASGAGKSTLLNTLSQRQTVGVVTGEMLVDGRPLDPDFQRNTGFCLQGDIHDRTQT

VREAIEFSAILRQDSSVPHDEKIEYVNKVIDLLELNDLEDAVIMSLGVEQRKRLTIGVEL

AAKPSLLLFLDEPTSGLDSQSAYSIIRFLKKLSRAGQAIICTIHQPSSILIQQFDMVLAL

NPGGNTFYFGPMGESGEHIVKYFADRGAVCPPDKNIAEFVLETAARPHRKPDGSKINWNT

EWRNSKEAQAVLDEIDGLQRLRSTALAQGSASGPPKKEFAAGPWVQTVQLTKREFRQHWR

NPSYVYGKLFTAVIMGIFNGFTFWKLGNSIQDMQNRMFTCLVIVMIPPTVVNAVVPAFYN

NLAIWQAREYPSRIYGWFAFCTASIVTEIPVAIVSGTLYWVLWYWPTGLPTESTVSGYSF

LMTILMFLFMNSWGQWICAFAPSFTVISNVLPFFFLMFGVFNGIVRPYTDMPSVWRYWVY

WMNPSTYWIGGLLSATLTGQPVECNEAETARFLAPPGQSCGEYAGDFIKTAGGYLLDSNT

SGECQYCPYSSGDQYLATINISASDKWRNFGVFLTFCVTNWMLVYFFIYTVRVRGWSFGF

GWVFGHLGKLVNQVRSPFQRRKGESSSHDEKA

>SoG_05154.T1

MAEAVEAEQKAFREGGSKAEYERACEAAACATPEVSRGSSEAMDDAPSPGVQIGSYKNCH

LFADGVTSEVYRSGNIALKVIVNHHNLEPHNPQREVKILQRIKPPCIPLHEVFRDQEQRL

VLAFTYMPYTLASLLESGALRHEQITNIFQDIFRALQHIHSQGIIHRDVKPSAILLETPS

GPAYLSDFGTAWAPDLSSQSEPPDNKILDIGTGPYRAPEVLFGYREYGPPVDMWAAGVML

AEAATVPPTPIFESRAVHEDGNQLGLILSIFKTLGTPNKEIWPEAADFKVNPFELWTVFP

QRSWVNILPGIDSGIRDLVAKLIRFDGQRLTAEKVRFCTD

>SoG_05161.T1

METSIDDTLDRAQYPRPQEEMSQPASTSTARPKHHLVDFDENDPENPKTWSKAYKWYCTM

VVAVTCFVVAFASSVITADIAGVVEEFGVSEEVALLSITLFVVGFGVGPMAFAPLSEIYG

RRIIYTWTLLLAVIFIIPSAVAKNIETLLVCRAIDGIAFSAPMTLVGGTLADLWRNEERG

VPMAAFSAAPFLGPAIGPLVGGFLSDATNWRWLYWIQLILAGIVWVLISFTVPETYAPTI

LARRAKKLRAETGDNGYMTEQELDDRSLADRLRVFLVRPFQLLFGELIVFLISLYMSVLY

GLLYMFFVAFPIIYQKGKGYSAGKTGLMFIPVAVGVVLSACCSPWVNNHYMTFVHKHNGK

PPAETRLIPMMLSCWFIPIGLFIFAWTSYPELSWAGPAMGGFPVGFGFIFLYNSANNYLV

DSYQHQAASALAAKTFIRSFWGAAVVLFTEQMYDRLGDQWASTLLAFISLACCAIPFLFW

IYGAKIRARSKYAYGGDDAEESADELEKGEDQEAHHARDVALMRAISYVSNP

>SoG_05174.T1

MATNTRVLAIAAFPRVARHAQKPCARLYTGSTYDEDWNSRMIGTEWRQKMLTREEETHCQ

QSVGWGSAARLIVAGPRARHPEADLDLCYVTDDIIATSGPSQTYPQRAYRNPLDKLVSFL

DTRHGANWAIWEFRAEGTGYPDHLVYNRVRHYPWPDHHPPPFRLIPMIMASMRNWHHGGQ

LHSEAGRAGGEERLSEEEAKKRVVVVHCKAGKGRSGTISCSYLISERAWTAEDALARFTA

RRMRPRFGAGVSIPSQLRTISYVSRWTNNSKRYVDLPVEILEIHIWGLRDGVKVDVAGFA

DEGKRIEVLHTFNRDERIVVEGDPPEGGGLGEIVWQMAGYGVGVKKEAPKEAPLADSANL

EDNPEPKKHHSLKKKGTELLHRASSSTSLRSKARDASPSSSTSEIHEPGGKAVILRPTTP

IQVANSDVQVSVELRNKTSKNLGFTMVSSVAHVWFNTFFEGNGPEQDGKADDTGVFSIEW

DAMDGIKGSSRKGARALDRISVVWRRIGDENGDGKEIREPAEGEAVPELQAADWARGEEG

EVKEKELGLRKQSPGSADVSKASSLKDESRGGGGGDGVPGAGVVGARAEAGAQIGDVVDD

EDEWKGMRTSGPSGEELHEKK

>SoG_05218.T1

MGKPRRAMMAEAEEALTPPDTLTSDQSIVKVIKSEGNNVYTCELPNKKTVQLELAQRFRN

TIWIRRGGYVVAERYPPGSEEKRVAGEIINIVRDEKSWRKQPYWPKEFVKTSAFSDSEDD

SNVGKMPPSDSEDE

>SoG_05220.T1

MDAATIEETNRLRVSLGMKPLPVPGAENDARPDSEEDVDTLESRQAQGYENFRKLQEAEA

TKKRREERAAAIRKARDQAARFAKLEGKGLGEEDEKLDVDAKTWLLGQKKRQKKIEKARK

LEEELAEAEAEAAAAVQYTSKDLAGVKVAHDASAFLDGDEQILTLKDSMIGQDEESGDEL

ENFDLREKEKREERIGLKKRKPGYNPLDDEEGEKAILSHYDEEIEGKKRKNFTLDRDGAI

GELSAIMEKPSQESRKLQSISLDDIVGKSGITESHSQPQTLTSPQTGDDAAPSSDYLAAS

EIKVKKPKKGKKNKGTRKKQLDEDDIFPMEQTNGEADSMDIDSKDAAPKKKRKTTDEDLV

DDDDLQASLAAQRRSALKKRKKMRPEDIAKQLREQAEDEKALNDEPEGGLVIGEISEFVA

GLTKHDEEDEEERRPRKRRTTTRSPHRDNEEDEEMADVNHNYNPTQDESDLLEQAQRQAQ

EESSSAAIVDEEKTVGEGMGAALALLRERGLVADPATSSTAFQAHKDRTDFLLKKRLLEA

ELDEQTRLQRERDRRSGKFDRMSQRDREEYARQENHRRDLLQSRKMAELFAKTYKPTVEI

KYHDENGRELDRKEAFKEMSHQFHGKGSGKGKTEKMLKKIEDEKRREGQSLFDASGSSGG

GMSAATQQQVKKRREAGVRLQ

>SoG_05226.T1

MSSSDEGEIVENGAKSSKATSLPHPEGSGVDRRDRLSSRHSTPDYDTASRHSGSSRRSRS

PRGYKRSRDERDSYGRSRNDTRQGRGNYDDVRRDGYGRSQLPYDDIDRPSRSQDNRYVFD

RDRDRDRDRDRDRDRDRDRDRLPDKRPRHRSRSPRRYRPEDGRPSDRFVRDSQAGRRGPS

STESRRGDKRDHGSSVDLRNTVGKDSNLSRNGAKDTQGSSITNGDGSSRNESRPPQPEPE

EDYKEPEPIDEDAEIERRRKRREELLAKSSSATPLLLHAVGAGEKAREVTPSTPAETSSR

VSREESPQTPRTPGFGSPRSPGDVKMLSENDLMNTHNKTKPSDEDGPSAADYDPTLDMRE

DERRDEMRHGHVVLHGEPHILETTEQQEPERNADETKKSSGSDDEDDDFDMFADDFDENK

YATKPSKAVAPVEASDGDPTAPAENKGGILEGDDKDGYYKIRVGEILNGRYQIQTTLGRG

MFSGVVRAIDITTKKPVAIKIMRNNDALRKGGYTEIAILQKLNDADPENRKHIVKFERSF

DYKGHLCMVFENLSLNLREVLRKFGNNVGINLGACRTYAYQIFVALAHMKKCSIIHADLK

PDNILVNESRAGLKICDLGTAIDRSDAATAHTEITPYLVSRFYRAPEIILGMPYDYAVDM

WSIGATLYELYTGKILFTGDSNNQMLKNIMEIRGRLTPKLYKRGQLSSAHFDEQGQFISI

ERDKILNKTTVRTLPMVKPTRDLRTRVLAASSGLSDAESRELNQFIDLLEHCLTLNPDKR

IKPADALKHPFFTARSTGTKR

>SoG_05230.T1

MSHEISILRTYAKSEISNGLGRLIELRRELISWWEALPSDTFPKETSSQSAVTRMGMHLR

LEYYLIHIFVGRAFIFPRDSSRSNRSSSSSASSSNKSHPRSLLVNGCIEASMAVVDICRQ

IRSGIGLARASYTEFSACRAALLVLTTQCLQKKTDRFRRALRDGLSMLKEMSVGGESART

EVSLIEAFERAIARLDSVAAEGRADSAYSRFKKWEQLWKSDPAAMDVTGDRPHDNPMPMP

PQLAAASWRAAEGPPHRQGTVPMPSYTPFFGMDGNFASFPQTLDEFSSFLGYSFGPSPDS

TDNTGNRSTWIGP

>SoG_05233.T1

MASTRLSQLAEHLTFKESPKHPLDPLDAREIAAAVAVVRAQKGKLYFNTITLLEPRKKEM

LKWLEDTSKPRPPRVADVIATTKEGQVHDIWVDLGRRSIVKAEIAEGIQPTLIVEEMLSV

EEVIRKDPGVIEQCGIIGIPPENMHKVYCDPWTITHDPKYGTKIRAIQALLYYRPNLDDS

QYAFPLDFFPIFDVNKKEVIDFEIPPVRRPISLAEPNNYHMPDIEREGGYRTDLKPINIT

QPEGVSFTMEGRTIRWQNWSIHIGFNYREGIVLNNITFYDKFEGKERPLFYRLSIAEMVV

PYGNPEHPHHRKHAFDLGEYGAGNLTNSLSLGCDCKGAIHYMDAELPTQAGGVRTIKNAI

CIHEEDAGILFKHTDFRDESTIVTRGRKLIISQIFTAANYEYCIYWTFLMDGTVQLDIKL

TGILNTTAMNPGEDLEGYGAEVYTGVNAHNHQHLFCLRVDPNIDGQQNTIFQSDAVRSPY

PVGHEKNKWGNGFTAAKTKFTTVTEAQSDYNGATSRTWDIANENKINKHSKKPVSYKLVS

RESAPLMPKEGSLVWKRAGFARHTVQVTKYKDDQLHPAGRHVIQTSGDPSPGLPAWIAEN

PQESIDNTDVVLWHSFGITHFATPEDFPVMPAEPITLLLRPRNFFERNPCMDVPPSYNIT

STQVKEGKKGCACAASKESVRV

>SoG_05270.T1

MRPKFFRKRGEPEDITPVLGPIEAQAEASGIDHFKSLRQFEKLHKLDPNLPIDELNDVDA

AIATGNAEKGIEIEHALMEDNSPYPEVRAVVRNYDIDVPANTIRAWVIGLLLCTIGSGVN

MLFSLRNPSITVNTYVVQLIAYPIGRAWDMVMPDREWNLFGLKFNLRPGKFNYKEHVVIV

AMSNPVQAAYGGGSLYSTDVLMVQQLFYGQHFGWAFQLLFGISTLCTGYGLAGLARRFLV

WPAAMIWPADLVNCALFYTLHDHTRSDPAKTNGWSIGRYKLFLIIGSASFVWYWFPGWIF

RGLSYFTWICWIAPNNVIVNKLFGGYTGYGLMPISFDWTVISGYIGSPLIPPFHAIVNVL

IGIFVFFIFTSMGIHFSGTWFSDFLPVQSSESYDNTGAVYNVSRILDANNQFNETAYMEY

SPLYLPTQFALAYGLSFAAVGAVIVHVILYQGSDLWRQFRLARHQEDDVHMRLMKKYRDA

EDWWYAVLFIVMVAISFGVVIGWPTGFPAWAYVVCMLIPIVWLIPIGIIQGITNIQLGLN

VLTEYIIGYMVPGRPLAMMMFKTYGYICMGQALYFAQDLKLGHYMKVPPRVMFSSQVVAS

IWSAVLQIAVMNWALANIPDICSPNQPNNYTCPGTRVFYTASVVWGVIGPARIFSGSAMY

SSLQWFWLLGLVTPIITWLLARRWPKSLWRYISTPVIFGGQGMLPPATVYIYLCWGMVGI

VFNFFIKRRYTGWWLQYNYITSAALDCGLIISTLVIFFTLYLTSASHPNWWGNTTALQTA

DLTINAIKSRVAPGETFGPTSWP

>SoG_05307.T1

MADQQLDKDKRLGITWYDLTVKGIATDAAVHENVGSQFNIVKHFKSSRRKPQLKTIIDNS

HGCVKPGEMLLVLGRPGAGCTSLLKILANRRLGYAEVTGDVMYGSMNADQAKQYCGQIVM

NTEEELFFPSLTVQQTIDFATRMKVPHRLPSNVSSPAQFQQDSRRALLRALGIEHTSDTK

VGNEFVRGVSGGERKRVSIVETMATRGSIFCWDNPTRGLDASTALEYVRCVRAMTDELGL

SSIMTLYQAGNGIYELFDKVLILDDARQIFYGPMDQAKPFMEDLGFVYTDGANIADYLTG

VTVPTERRIEPGMESRCPKTAEQLRSYYEATELRRAMALEYSYPRSDEASLVTEKFQEAV

RSERDPGLSKGSKLTVSFYAQVQSSIVRQYQLLWGDKVTFFIPQILNFVQALITGSLFYN

APDTTAGLPFKSGALFFAVLLNSLLSMSEVTNAFGARPVLAKHRGFAFYHPAAFCLAQIA

ADLPLIIVQVTLFALPSYWMTGLKPTGEAFMTYWIITISVTMCMTAMFRAIGAAFSSFDA

ASKVSGFLMNALILYTGFLIPRPMMHPWLAWLFWINPLAYGYEAILSNEFHGQVMPCVDF

NLVPNGPGYNDQEFQACTGIRGAPVGASVVTGDQYLQGLEYSHARVWRNFAIVWAWWALF

VALTIYFTSNWSQVSGNSGFLVIPREKTGKMKHLAAVDEESQSRVDLPDSTGQRRGGSRT

DDEKTSAADSADTSSSANLDSQLIRNTSVFTWKGLTYTVKTSSGDRVLLDNVQGWVKPGM

LGALMGSSGAGKTTLLDVLAQRKTEGTIKGSILVDGRDLPVSFQRSAGYCEQLDVHEPLA

TVREALELSALLRQSRDTPIEQKLKYVDIIIDLLEMHDIENTLVGTTTAGLSVEQRKRLT

IGVELVSKPSILIFLDEPTSGLDGQAAFNIVRFLRKLADVGQAVLVTIHQPSASLFAQFD

TLLLLAKDGKTVYFGDIGDNGATVKEYFGRHGAACPQNTNPAEHMIDVVSGSLSAEKDWN

EVWLASPEYAAMTKELDRIIADAASKPPGTLDDGHEFAAPTWSQLKLVTNRNNISLWRNT

NYVNNKFLLHIISGLVNGFSFWMIGDTVADLQMRLFTIFNFIFVAPGVMAQLQPLFLERR

DIYEVREKKSKMYHWSAFVTGLIVSEIPYLVICAVLYYVTWYYTVGFPLGSDKAGAIFFV

VLMYEFIYTGIGQAIAAYAPNAIFAILVNPLVIGILVFFCGVYVPYDQIQVVWRYWLYYL

NPFNFLMGSMLVFSTFDAPVHCARNEFAVFNTPDGQTCGEYLADYMQGRLGSRASLANPD

DARDCRVCQYRTGADYLHTLNLKEYYYGWRDAAIVALFALSSYACVFALMKLRTKASKKA

ES

>SoG_05315.T1

MDHDTAARTHIKHKKRPQSRASTTSVQSGLTQVGAAEQQALPNAHNLYNGHWLANSHSRA

MSIPPQMSPEDLIMQAAGHMRGPPQDVHMDASMGGAMAHQMAYQQHQQAQHQSQEMHRHP

LPVEQFAGNASFTEGDTSMMEREDNIEGDSINHELAQPRSGSLRSSANNELEMRQLFSAN

RHRGLQDVASELHGNERGPNSERTRQVFAMLWINSVCSVGKGSVPRGRVYANYASRCATE

RITVLNPASFGKLVRVLFPGLKTRRLGVRGESKYHYVNFALVDDQPEARESQPPAPRLLS

EPRTNASQSFQSVSAGGNQRAASSAPPSQSLVPLPDGARNKKSAGAARRTHSLYNNPDLS

TLENMDATATKTILELAFMSQGEEDMGQSDFLALPSIEQYLPTGTDPDAAKSLSALYRSH

CTSLVECIRYCKEKTFFHLYTSFQGTLTMPVHKLFSHPALAPWIEGCDLVLYQNMMRIIS

KLTLQVLPKPVLDTLRAISERLITHIRESFQGQPAHVLRAKETPATLFAGLLDRALRVNL

TAHAAANMLSNPANRDQMYVEFIQLIKPRKVAECVPTRGMDDTVALLVNDMRDLLDPQNV

PWEIECLTIYGDLAIRAPKQSDRDSTANAGHNVLDKWVGLLQSLPERFPYASPSDIVWCV

ERIGTSVMRDITLSSGKSFGSWWVTKTWVDEMVCFLAEQGGFMKRTVTMTAPDAALEETS

ATKVNARQQSSRYSSGSDDLNLPNMSQSQPGRAPFPPVGKTKDAAVGMTNGADAHDDSGI

GIRTPDDDFTVDKFTFSPPPSENLGLGREASQGVA

>SoG_05320.T1

MAKKTVIGILGGGQLGRMLQEQAALVDVELVTLDAEGAPLRQINDNPKHVTGSFKDPEKV

RELARKCDVLTVEIEHINTEVLEEVATVGVDMGGGRRKKVPVHPSWETLRLIQDKYLQKE

HFGKAGLPIARQIQVESGEGMVAALQQAAKAFGFPFFLKARKGSYDGRGNFRVGGPEDFA

EAVEAMGKLPLYAEEFQPFNKELAVMVMRTETDEGQTKQVYAYPAVETIHEESICTKVFY

PPRGVPNSVSEEAKKLASDVVHTLKGRGVFAVEMFLLEDGKLVINEVAPRPHNSGHYTIE

AVPAMSQYRAQLYSILDIVHPSFKLQPRVSSAIMINILGGAQEQSHESLVDMTSSVYDDN

MDIFLHLYGKASKPSRKIGHITVTSYSPDVDLEQLASPLIKEADHIRRERLEANSAALRP

EAAASTARSQAIAPTASSRDTENPLVVITMGSDSDLHVLKGAFDVLEQFRVPYDFTITSA

HRTPQRMSELALSAAGRGIRVLIAAAGGAAALPGMLASETTVPVIGVPVKATHLDGQDSL

LSIVQMPRGCPVATVGINNSTNAAMLAVRILGTSDAGYRDAMAAYMHKLGEEVEAKAAKL

KQDGWKAYLDGQKK

>SoG_05325.T1

MSSPLRDNPASANRGLGPRANRKRSRTGDGSSPMAPGSSPMPSSPPAAFNITHGVEDDDD

IEEAADEIQDDLDDVDEMAEDDVDLFREGFEADYRDREDDRYEGVDLDDEGEYDDIELGD

RRRLEAQLNRRDREVARRQRIPAAFLPGDDEDGDIDLTAQPRRRRHHYDEDPDDAMDADI

MDEELSLEALGDVKAANLTEWVATAPVQRTIKREFKAFLTSYTDASGSSVYGNRIRTLGE

INAESLEVSYEHLSESKAILAYFLANAPAEILKLFDQVAMDVVLLHYPDYERIHSEIHVR

IFDLPVHYTLRQLRQSHLNCLVRVSGVVTRRSGVFPQLKYVKFDCQKCGVTLGPFQQESN

VEVKISFCQNCQSRGPFTVNSEKTVYRNYQKLTLQESPGTVPAGRLPRHREVILLWDLID

KAKPGEEIEVTGTYRNNYDAQLNNRNGFPVFATILEANNVVKAHDQLAGFRMTEEDEHNI

RALSRDPNIVDKVINSMAPSIYGHTDIKTAVALSLFGGVAKTTKGDHHVRGDINVLLLGD

PGTAKSQVLKYVEKTAHRAVFATGQGASAVGLTASVRRDPLTSEWTLEGGALVLADRGTC

LIDEFDKMNDQDRTSIHEAMEQQTISISKAGIVTTLQARCGIIAAANPIGGRYNSTIPFS

ANVELTEPILSRFDVLCVVRDTVEPEEDERLARFIVGSHSRSHPQSQLAQDSMAVEPDSL

AGETQRSAAAKKEIGDIPQELLRKYIVYARERCSPKLYHMDEDKVARLFADMRRESLATG

AYPITVRHLEAIIRISEAFCRMRLSEYCSAQDIDRAIAVTVDSFVGSQKVSCKKALARAF

AKYTLSRPGAGSAKGQSQPRRPNAVMA

>SoG_05328.T1

MRLDTWPLTANGKLDRRALPAPDLDALATRAYEAPQGEVEATLAQLWQDLLKVERIGRHD

HFFALGGHSLLAVSLIARMLQEIGSAHV

>SoG_05334.T1

MVINDSSLRDPAMSNTASSSVAVGTASANTAAPDDSAASSITVNTKPPANFPPPKTDKPR

PHVCGTCQRSFARLEHLKRHERSHTKEKPFECPECTRCFARRDLLLRHQQKLHQTSTPSA

RPRNRRESASGATPAASRARKNSIAGPNAAAAAAAGNGPMRPRANTISHVDGTAMQLIAA

ANASVARQLPNTHSRHPSLVGLPLHNLDHTFGGMAAAMGQRGVQHGLPKLETGQMNSTDF

STGLRTAPPMAVFGNEFDFGDFLYANAGTTINPNHLHYNDSPPSMAMEQISPFPTPMNDL

STPQLDDNFDWLTGFGHQINIASNENVIDGSSPSAISTTSQSGISDVMVDGSNHPPAGTS

SMWQSSLMGPPQMPNAFSMDLGSVFPDLLSGAPLSPQPASQKMNDPYFSTPPPSLSSLSP

SMIAGLNTQNLNQALGFNAGPETPSSLNGGNHGASPVTTITENTRTAIVNALSQCLPFGG

RKYSFTSQGSPQSPQFQSSPGNPAANNLPSTRDLQRYVGSYLLCFHPHLPFLHLPTLSFD

VPISPSGRPEGVGGSSCLILSMAAIGALYESEHTRSRELFEMAKKMIFFYLEERRKADVR

KADFRRATPGTDQSVHSHEGPIHTPVWLVQAMLLNVVYGHNCGDKTASDIASTHCTALVS

LAHAADLVRLVRVNSSDNQMNEDGNWHMKTEHDEQLEWLQWKTLEERKRTLYTVFILSSL

LVSAYNHTPALTNSEILLDLPCDEEFYSAESASAFAAKGGVKAANHNRMTFHDALGDLLR

SNERQQKQVAQVGGKSANGDMTQPDLKPSTFGCLVLINALHNYIWETRQRHHNKVWTNEE

TEKMHRHIEPALKAWQAAWASNPQHTLERPNPYGLGPLSADAIPLLDLAYVRLFVNLSRS

KDKFWQRDWDGVAEELARGTEIVQHAEHSPGSTSDATGTEQSDNSVQSSVFTDSPMTQVS

SNDTAAMKWQGQAGSSSRVTSRREKHLRKAAFYAADSLSMSDKLGVTFAEFTSRELPLQS

AMCAFDCVQVLAEWIATLQDRVGRYIGILGHDAVDLKAMPAIMLLEEEDVKLLDKVQHLL

SSVEMKINVEVMGGNMTGAETLLSTEGRKGYAAKILRVTALLLDKSAVWPVTHLMAQCLD

THAGHVKIRAEKSVASTD

>SoG_05335.T1

MADIDIANFPAAVAKADFRAVEPLLKQLDKYLTLRTYVRGYELSEDDKSIWTAIHGSKVT

LGVVRKGTYANITRWFTYIEQFHPEVKDAASGDKKKGEKGKANYNIGLKGTENGVVTRFP

PEPSHQTMKTPSTDQDCCRGYLHIGHAKAALLNDYFAHEAFDGKLILRFDDTNPSKEKQE

FEDSIITDLALLNIKPDKVSHTSDYFKELYEVCEQLVRDGKAYADDTDPEIQKEDRRNKL

PSKCRDRPAEESLAKLKEMKDGTDEGKRHCIRARIAYDSSNGALRDPVIYRFPNFADKEP

APHHRTGWDWKIYPTYDFACPVVDSIEGVTHALRTTEYADRNEQYHWFLEATKMRPVHLW

DFARINFIRTFLSKRKLTKVVDTGKVTGWDDPRLPTVRGILRRGLTEPALREFMLTQGPS

RNVVTMDWTKIWSINKRMLDPVVPRHVAVDEKDRVVTTILGGPEKPYFEEKLKHPKNPAM

GKRAVRFSSSILLEQADVTTFAENEEITLMNWGNAIVRKLTKTGDVITSAELELHLAGDV

KTTDKKVTWLAAEDSENKLVPAECWEFGDLITKDTLDKEDNLDDFLNPVTEQMTPALCDA

NLAELKAGQFLQLERKGYFRVDKALGEGPGGRAVLFKIPTGSSK

>SoG_05347.T1

MMYSSFWLMPVSAVLFMFGNIVRQLLPRRKEQPPLVFHWLPFIGNSISYGIDPPKFFSQC

REKHGDVFTFILLGKEITVYLGVDGNDFILNGKHKDLSAEDIYGPICTPVFGRGVVYDCP

NSKFMEQKKFVKFGLTTRALESYVPLISQEVKDYVSGNPIFDGQSGVVDVTHAVSQITIL

TAARSLQGEEVRKRLTGEFANLYHDLDQGFSPINFIMPRAPLPRNRRRDVAHAKMNAIYT

DIINTRRMSGSELGPDMIWNLMGCTYKGGCPLPDSEIANMMITLLMAGQHTSASSVSWII

FHLSSRPDITEELYQEQLNALAHGSNGLPPLEHSDLAKLPLLKGVIMETLRCHNSIHSVM

RKVKNSLPVPGTDFVMSPGQILLASPAVTMLDERHFREAQSWDPHRWDNQLSMEEEEADL

QGLEQQEISRGARSPYLPFGAGRHRCIGEKFAYVNLGTIVASLVREFKFSTVDGKKSVPA

TDYSSMFSCPVRPGVVRWERRKKQRLQTYFEVGEKAKA

>SoG_05365.T1

MIAASSFLPSRFRGQKPATKASAPSWFSNSITASLQTLASLTSLNPIHTIVIVAVLASST

YLGLLEESLFDLSETVSRADWSSLTDGSRSLRIGHETSWKWQPYDSEVSAPSGAHHLALV

TFVFPKSLSHSSPNSAPLIHVVPTPENLSITSLPSTANLLGAYSQDTALAFALDFDQAPQ

FAAAAQEIPNDIPGQETMDTERGREKKMWIMKAARVHTRSSVVRWFNNTWVGFVDLLKNA

ETLDIIIMVLGYLSMHLTFVSLFLSMRRIGSKFWLGTSTLLSSTFAFLFGLIVTTKLGVP

ITVVLLSEGLPFLVVTIGFEKNIVFTQAVLSHALTHHRSLGNGNSNGNAKGLPKVGESTT

NVIQYAIQAGIKEKGYEIVKDYVIEITILVAGAAFGVQGGLQQFCFLAAWILFFDGILLF

SFYTAILCIKLEINRIKRYAEIRRALEDDGISHRVAKEVAENNDWDRVDGTRQPATSIFG

RKMKSTSVPKFKVLMVSGFLIINVLNICTIPFRSTDSLTYISSWAGGLGGVASSPPVDPF

KVAASGLDSILEDAMRKSQETVVTVLTPIKYELEFPSIHYGLRHGKVEGESDHMANYGMG

GRMVGSILKSLEDPFLSKWIVVALAMSVALNAYLFNAAQWGIKDPNVPGRQINTRDLADA

QKFNDVDTPNLPPGEYKRPTATPSKPIIPASTDDEGESPATRAATEQAHANVSHSPEEIE

KLLREKRAHELTDEEVVSMSLRGKIPGYALEETLKDFTRAVKVRRTIYSRTKATEELTRV

LERSKLPYDNYNWAQVFGACCENVVGYMPLPVGLAGPLVIDGRSYMIPMATTEGVLVAST

SRGCKAINFGGGAVTVLTADGMTRGPCVTFETLERTGAAKLWLDSDEGQRVMKKAFNSTS

RFARLQQMKTAVAGTNLYIRFKTTTGDAMGMNMISKGVEHALNVMATEAGFDDMQIISLS

GNYCTDKKPAAINWIEGRGKSVVAEAIIPSDVVKTVLKSDVDSLVELNINKNLIGSAMAA

SVGGFNAHAANIVAAIFLATGQDPAQVVESANCITIMKNLSPSLRGSLQISVSMPSIEVG

TLGGGTILDPQGAMLDLLGVRGPNLARPGENARSLARIVAASVLAGELSLCSALAAGHLV

KAHMQHNRSAPPTRSSTPAPSSSATTPVSLAITSTVEKAARHPEQLGSPSPAAVERAKR

>SoG_05371.T1

MSGRSSSFQRPQNIGTRRPSITSRLSFAVSSAEQGEFSAPAERQIEEEIAEIKRYEDFTT

IDWVQDAAREQARRTARRRRTAGAYEHGQPGWRYRVWESYEAAQGWIVVTIIGAAIGMNA

ALLNIVTEWLSDVKMGYCTTAFYLNESFCCWGEENGCPQWRRWTGFEPLNYFIYICVATF

LAFVASFLVKSFAPYAAGSGISEIKCIVAGFVMKGFLGLRTLIIKSICLPLAIASGLSVG

KEGPSVHYAVCTGNVISRLFNKYRRNAGKTREILSACAAAGVGVAFGSPIGGVLFSLEEI

SSHFPLKTMWRSYFCALVGTAVLAAMNPFRTGQLVMFQVHYDRDWHFFELIFYVIIGIFG

GLYGALVIKWNLRVQSFRKTYLAKYPVLESSLLAMATAIIAYPNAFLRIDMTESMEILFR

ECEGGEDYNELCNPEKRMWNMVSLLIALVLRTFLVIISYGCKVPAGIFVPSMAVGALFGR

FLGIIVQALHEANPTSIFFSACKPDEPCITPGTYAFLGAAAALSGIMHITISVVVIMFEL

TGALTYILPTMIVVGVTKAVSELFGKGGIADRMIWFSGMPFLDNKEEHNFGVPVSQVMRT

TLVSFPMHGTTLHEVESLLAEGKYQGFPIVEDRSSQLLVGYIGQTELRYAVDRLRKDGPI

SPDSKCIFAPQSSTTLVPTTPTVTVTTDAMSSTSLDFSRYVDATPVTVHPRLPLETVMEL

FRKLGPRVILIEYHGKLIGLVTVKDCLRYQFTVEAAEHPKDERRVTAGQAQLWSMMLRTT

SWISDHLQTWSGGRIRLSGQFERESIPSRQQPGGRGAILDGDEDGVDEGVELESRH

>SoG_05372.T1

MPYNTSAILPRKEPTGTTQLPLSRVKKVISQDSDIHMCSNNAAFVITLATEMFLQRLAED

SHNQAKLERKPRRNIQYKDVANAVASHDHLEFLEDVVPKTVPYKKVKANALNTQAHLNGE

QAPETQGSSMATAAGRPSTSNGVASANTSPKAIVNGDAAASGAFSVPPRVEPDASRRVSI

GAVMGEDDPSEQLAMEMRQAAGTGRDSDVHMTG

>SoG_05374.T1

MGQGFSLATPSAGSTGIDVPQLRDVQYERSIGNARFMKSVRGRTENGIVLVKILVKPYAD

VKLDEYKKRILEQRKALADVPNALGFQRIIETETNGYLVRQYLYSSLYDRLSTRPFLEDI

EKKWLAFQLLCALRDCHARDIYHGDIKAQNVVVTSWNWLYLTDFSSAFKPVMLPDGNPGD

FSYFFDSSGRRTCYIAPERFYASGEGSVPPQEKMTWAMDIFSAGCVIAQMFLESEIFSLA

QLYKYRRGEYDPVITHLSVISDKDVREMISHMIQLDPEKRYSAEQYLEFWKGKVFPPYFY

NFLHQYMELITDPSSGNNPMSGSQKNMGESDDRIDRVYYDFDKISYFLGTQPEKTRGAQP

RLSSRLGLDHFPVQLSIPNHEHTVTADLEPPEDDGTLIFLTLIVSAMRTTARVTSRIRAC

DILLAFAERLTDEAKLDRVLPYLMTLIRKEETDMVIITAIRTITQLLQLVRMVTPINSHV

LVEYVLPRMEIVLGSKNRMTSPLVRATYASCLGSLATTAQRFLEMASSLRADGSMPITDP

EIEPGADAEANFESVFDNAGRQLFELLETHVKALVEDPDVHVRRSFLASVPELCMFFQEH

SNDVLLTHLLTYLNDRDWTLKCAFFDTIVGIATFIGSTSLEEFMLPLMIQALADREEFVV

QAALHSLAQLAALGLLSRAKLWEMVDLVSRFAVHPNIWIREAAAEFVSRSASYLEPADVR

CVLTPLVTPFLTVRNLPDFSELTILDLLQKPLSRPVFEQAVAWAAKADRSNVWRNLQQSR

LGSANALTVRPGQDRSSGALGKMTLTKEDEQWLGRLKNLGLRPEDESKLIALADFIWRIS

KMKARDPSPGDPGMANGIISLTALGIQPQTVFFNETAGQASGPSPDVEPQSGLYTIENAL

LDASMTIDESAGKRKRAMLNAHRRVHSVGPRDSAGRLLSPTGSGRASSQDTRLGSAVRTD

SEPSLQDGSYSLRRPIRHSASALNLLDRKDSNKSIPETGTSGTNAFGEMEGPFAQPLDPQ

AAGAAESDRKAGGKKPSRHTYEGGDPNIQRMLDQMYLDNYPRDIIEFGPMVQPVSKREGS

SSSSSRISAQGGSDVPWRPEGTLVATFSEHKGPISRAAVSPDHVFFITGGHDGTVKVWDT

ARLERNITHRARQTHRHAADAKVVALCFIENTHCFVSCASDGTVHVVKVDTLTASGVVRY

GKLRLLREYLLPEGEHAVWCEHFKQESSSVLLLATNNSRILGIDLRTMTLLYVLENPVHH

GAPTCFCVDRKRNWLCVGTAHGIVDLWDLRFRMRLRGWGMPGGKSPIYRLAVHPNKGRLK

WLCVSGGTGRGEVTVWDLEKMACREVYRTGGESARDVNAARKRAMDTSQSYEAWDVDEDK

PDGMLSRFAIGELDEEADAEDDGGVRALAVGTELGPTREVRHAFLLTGGSDRRLRFWDLS

RVEKSSIYSGLILPGSEELQPPKATYTSTQPGANLTVNTEKLPKSGASSAGGKSSSGISS

RSSRATVISRQQGQLLQSHMDGVVDLVVLERPYMMSVSVDRAGVVFIFQ

>SoG_05378.T1

MKFGTQLVNESVPEWSLHNLDYNSLKHEIKVHTTRGQATAIAIPGHQEASLRQFEEALYD

ELCHQHDRVDLFINSKADEVGRRLVHLSRSIERWLGKQPVEPGASQILRFHRRFARYERD

LLQCSDDIQALTRFANAQIVAFRKILKKYKKWTGSTTLGSRFVDNVLRDPKSFTRRDFSS

LQARHDEIHRTLTEACPGYSEPSSPSSDELPHPDGSRRVSQASGSQHFLPLPPPHHEEPH

SQFKYWNEYDDGSESGSPDTDYAIYINPDDGPAFPGLGYIQAIAGMPFEKAKQWFRKGSR

KDGEHKPLLGGNGVIGQGYNSTTTVQTDSEEEGYASSQDIPTQGYVGLYAFPSIAEQKVT

RYRERVLFLSTIGCFLASFILIGIAGILISTGRHRLRVEVDAGVTVAVMISLFCACSALG

MMFYRKDKLTIVHRVIVWSAFVSSCLLNGMLLLLVLGNTP

>SoG_05398.T1

MAKSTRFLGGNEPLQIFQDDMFEAGPMTSHAPMPTVTKPARRPLSSSSSNMILNPPPAAS

TSLSPHKFQSSSSPRSPLKATKGNKLNAVAMAPPSTKGITTDSLQKKPYLSKFKTGPYKP

NMDLMSSGKENMHPQIFPAPAAITLNIENYYQKPNGKRGLMEAASINGSRPVKKTKTEET

VLPPHDSFPPITDDGTKPPHSYAQLIGMAILRSPLRRLTLAQIYKWISDTYSFYHPTDAG

WQNSIRHNLSLHKNFIKIERPKDDPGKGNYWAIEPGTEAQFLKEKPTRKSASAAENIPVM

STRLEPPRPAVMHNQEPMLPPPRMPHHSQVALPPLPTSQMAGTELSSDATIVVSDSAVPE

EQGNQSADAEPPMDSSLYSPLPAALHSSPPIARSGTARTGTPPPLGRNVNSSVTRSHQRK

FRSMDDSGYISSLESSALRPRPTAHLLTSEAERPRLKRGRARDRGKGRAEEEIKLLRNSS

PYGPGYSSPLRQAHGQQMLPPLTPNVRFKPMARPPPSASPNTNLRIHRDNVKEILQSPCR

AGVESLVEMPWSPNFQLEDAAPGVDVGGMDSLDFSVFTDYDACNEFSFLGSVDNGSPVKR

SAKRQRLDRSISTSALDDISNSATRRSVTSAPLLKIPEQGPLDLLETPSKAFEGMSSPSK

MFQQSPLRNQSPCKFADLPDMSFNLDWHLAEDFMLAQPDATEISGLDLSQGFERIGSGNQ

VNKSKSKPTLGRSYSTNF

>SoG_05402.T1

MVSLKSLLLGSAAISSALAFPFNITEIQEMFKRDSELQPRTSPGTGTHDGYFYSFWTDGQ

GSINYENTQGGGYKVSWQNVGNYVAGKGWNPGAARTINYSGSFQTSGNGYLSIYGWTRNP

LVEYYIVETFGSYDPSSAAQVVGTVDVDGGTYKILQTTRYNQPSIDGTSTFQQYCTV

>SoG_05416.T1

MERHNPPSRQKACAACTKAKRRCDFALPSCVRCSMRSIPCKYPLRHASTTTARQRENIRS

IDTACSTTANSAQATTPLVDFYLGNGCNVGPEQHDLLSAEQIPTCTDSHGCINPILIDSL

HTYATESLGQSSSLAPPTAKHMKDMAHVVATRLQFAIEAFKKVPQTMRQASTLDTFSALC

GGTTNKPSTDAQAACALYVAKNEVNAPVIMRSIESYCEELLSSSPPATAFETLAYAHALL

LYQIIRLFDGDIVSRASADECIPALEDAALSLLTHVNFEEPLFPAPDLPLDPIGPTKVFW

KTWIFQESARRTMLFVFFFLQAYRMVSGGTLYPCDGKMGLVHSWTLSAHLWQARTPTEFA

KAWRDRNHFVVTNAEFHQVLGQAKADDVDCFGRILLSAMMGQDEADGWFAQRGGSLRDEC

YV

>SoG_05417.T1

MDKPTTTFVCGATGTQGGALASFLLSRALPVHCMARDPSSAAAQSLASRGAKVHPGSYDD

LDALAEALTGCTTAYMNFMPDFTDHTAELRWARAILRIAGEKGVKQVVYSSSLGLDDPLS

LPGVEEGTFTANVLLSKKSIENEVRAAEHVPTWTIFRPGWFMANLLNPFAQMMAPGLLSP

SGTWTVGFVPSTRLPTIDTETMGRFTCEAVLDPARFHGHTIGYCDELLSPDEIVDRLKKA

TGRHELSVQYLAEEDIQAQIETNPFLHGQYCSRAVGDMVNMDEVKGWGIELGSLEKFLDR

EKSRVAETYNVN

>SoG_05443.T1

MSSNKKGDDPLANHVSGQSNKPMSAPAHALTADKVVEDLSANADSGLTAEEAKKRLQEFG

NNEFGDSEGVQPIKIFIGQLANALTLVLILAMAASFGIQSWIEGGVVAAIIALNISIGFI

QEFKAAKTMDSLRSLSSPTAQAVRNGNNQTVPTVEIVPGDLVELKTGDTIPADIRLIEVV

NFETNEALLTGESLPVRKEISSTFPDDTGPGDRLNVAYSSSTVTKGRAKGIVFATGLYTE

IGQIAVALRGKKSRRREPKRRQDGSASLPRWAQAWVLTASDAVGRFLGVNVGTPLQRKLA

KLALLLLGTAIICAIIVLAANSWDGSQEVVIYAVATGLSMIPASLIVVLTITMAAGTKQM

VKRHVIVRNLKSLEALGGVTNICSDKTGTLTQGNMIVKKAWIPGRGTYSVDVGNEPFNPT

VGDVGLNKNQPKDIDFSSDSAAGENVTPKDLVGSDETLKQYLNVASLANLATVQQVEGEW

RGRGDPTEIAIQVYASRFDYNRLRLSTGNEAQWHQVAEFPFDSDVKKMSVIFVDKHANKQ

WLFTKGAVERVLTSCPQYMNGDELSDLTDTIKNDILSNMEAMASLGLRVLALASRTDIRQ

VEENEADLERSEFEEDLIFRGLIGLYDPPRPESGPSVRKCHEAGISVHMLTGDHPATAKA

IALEVGILPSRMSEIANDVAKSMCMAASDFDKLTDDEIDNLPLLPLVIARCAPQTKVRMI

EALHRRKCFVAMTGDGVNDSPSLKRADVGIAMGMAGSDVAKEASDIILTDDNFASILNAV

EEGRRMFDNIQKFILHVLAENIGLACTLLIGLMFKDQQNMSVFPLSPVEIMWIIMITCGF

PDMGLGFEVAAPDIMARPPVSLKTGVFTLELMLDMLVYGLWMAALCLAAFVLVIYGWGNG

ESEFGKDCNSNIEDGCETVFRARAATFACLTWFALFLAWEMVNMRRSFFRMQPKSKLYFT

QWCHDIWRNPFLFWSILAGIFTMFPIIYIPGLNRKVFKHDAITWEWGIVFVEAILFFLGV

EAWKWAKRIYFRRMDRKKTTDKSDLEERTFGHYFKSNMSRSGDEESGLGNGSDDGYEQKK

NAHSPEAAMTDTSATVNGALKAGSNEKAVV

>SoG_05447.T1

MDRNHMLATGPKSQAAALAPVPAHAKNAASSHERKSPQMPSQEILREASSFPPPYRQNTE

TLAATGLPARFGSSHNERDVMLPPLTTVTRGHGLRGDNHNGHPPPPASPWPVLSPIALPS

IARIDSPSAMDLDTGSNSVNSAASPDRYGDGRAPSVNLDDPDVRLAAEALGDLRADFVSS

PPSRSATLPLSPPASALPAGKKEEPQQEPLFSLLTTSHPFLATTIGGATSAYGGAKNYSP

RFKSGAEYVENYLTPLANTVGSVGRVTGVEGSVRWFLGAGRRNASSSSDLEAPGNSKKRR

KADKEGEMPSNKRVDNETEKHLASGQRDLLEQDVFPATPRGDRRLSLASTVDTLPAYDEM

RSPAYTETAESQGTPSSGSSSQTAWQSRLVMSTSGLGVAMSDESLRSLKYCLRWLRWAND

HMGGVIGNLKTTLDQYDSHQENGAAQLEETARQEEGVQSDETAIITDEQSRTELAARINV

LKGDVLKTLQDVINTVSKYGGGALPENARILVRRHLTSLPQRFRVATMVENNGQGGQADS

ESAMRDGAQKVLVLAKEGLDMVAQVSGVLDGTIVSAEEWCERMGKRKPRADDPSEQRGEP

SLLPQTEPQADVKMG

>SoG_05454.T1

MSESPDFMTAALYAQSSSSSSALSHSATASESPPDTSSSHHQPHESLNAPISPAAQAVKP

SQAPSEKPFHSKRPHKKSRAGCKNCKARKVKCDEARPTCRSCRLRKAECVYPELPPSTSS

TSSAPKSAAHSTLRATHRASVRHHAAAATTASSSNGASPSPSPTINLPYHHPSPQQTTLT

SSILADPVLASFDPSDNLQLPPPTDPDQSHIDPTYDITTPLIVVSEPLYKPLPTTTDTEV

KLMWWYTSQTSTSFSLSPDASFITPSVQIMRTSLVQLAFQQPFLMDSLYALTSLHLCQLD

PSGSTIDYQRAVNYRQRSYLGYRKAIETADPKTFPALLSNSLLLCALSSQNFREPDTPDL

YIIDWMVVWKGIGLIVDLISVSDLIASGLQALFYRPPLNLDDAATAVPNHLLFMISSIPE

LDPDYPDRDAYYSCLKHLGSLFSNLRTGGINPIMGLRIVTAFTFVPPRFVELARLRRPRT

LVILAYYAMFFKLIPIVWWTVGIGQRSLRDICRHLGPDWHHLLRAPMMAIHVSDPVAVSR

LILEDPTWEPPYDLSASEWEKQVKELSWVDNAGRKMVFQPLRDRMVVRESPDGPDLEPFF

DNDYKSKDQITRKYGTESGTPQDQYGND

>SoG_05462.T1

MAAATITATKSSSMLNPTAATGSEVTKGNVTFRSPLFLRRPSNPASSSINLLTYLFASES

PAARSNPLNVPFQATPAEQGRATSSTPASQSQKGDQPQRTSPRHLATELSDPIPEEACES

ESELAEPLTPVSHHSQQFQTHEDQHPQDIPAEPLAVSTPAKNALAPAQSHTRATATPSPS

KAADQDPPASISRRSTFTRAVSSLFKRSSSQLSQQGQGLPTVDTNETVSSTAPTSSSVAP

SASGGRRWSNNPSSNTTRSNSPPSPGSPLEMASTVNHQSNPLPQPSDFKNQKKVRAATGL

SFRGRGIKFVGREGAPRTRRAASFDASRPDAHHDRHHHESTPESYWPHLPDAGTGTKARR

ISISLPDDFTVDVGELMKEFEYQSKILGRHGRHLGKGAASKVTLMARKGFPGELYAVKEF

RGKAKSETKEEYEKKIKSEYSIAKSLHHPNIVETISLCTDHGRWNHVMEYCSEGDMFSLA

KKKYLMTEDRMVDRHCLFKQLVQGIHYLHSHGIAHRDIKLENLLITKESKLKITDFGVSE

VFCGTHPGLREAGGQCGINMDHETRLCSPGICGSMPYIAPEVLAKKDKYDPRGLDVWGAA

VVMITFLFTGPIWEKAVRGQPCYDNLVNGWEQWNKAHEGKAEEEIADDDYPFAVPFNKVN

PPALRKILLRMLNPDPTKRLSIADVYSHRWVKKIECCQPESHEDQNKGIDATKSTSGKKI

RCHNHLPSKNLDTSLIGNSNFD

>SoG_05473.T1

MAVLEAKRDEAQVHGISEEAENAPLSTIAAAVAEENLAIPHLEPDQDAPPAYGESHNQMH

FSQPGLDAGARVADDGRVNISISTKNNRLADLLRPVIQRQLQHEPTLPPGYIPSSLGGQP

GQVPPPKLNVVIQIVGSRGDVQPFIALGQVLKETYGHRVRVATHPVFQNFVEENGLEFFS

IGGDPAELMAFMVKHPGLMPGFDAVKSGEVSKRRKGIATMLVGCWRSCIEAGNGLGPAPK

PHARGATFNPHEDLPGDGINVPFVADAIIANPPSFAHIHIAEKLGIPLHMSFTMPWTPTR

AFPHPLANIQSSNTDDVITNYLSYTLLEMMTWQGLGDVINRFRENVLDIPHMPLTAGPGI

LTRLRIPYTYCWSPALIPKPNDWGNTIDISGFYFLNLASSFMPEPSLAEFLDNGPPPVYI

GFGSIVVDDPNAMTSMIFESVKKAGVRALVSKGWGGLGADDVGVPDGVYMLGNVPHDWLF

QRVSAVVHHGGAGTTAAGIKAGKPTLVVPFFGDQPFWGAMIARANAGPDPIPFKQLTAER

LATAIHVCLQPDTQERAKELGQKIASEKGVDEGGQLFHKHLDIDGLRCAVAPHRNAVWRV

RRSQIRLSPLAAAVLVDRGLIHYSDLKLYRSKEYVTESQPPDPITAAAGSLVMDLSGIGL

AVADMPRQLFKTARACSPHRDNSSKPKHAGPQSAGSGSSMQQALRGRSRDTSASKSSLSL

ISSPSTDAAASTILGETDSLTSQTSKVHDSDSTGQQPPLGTATPVSSKPAHPPKIEARHQ

DIPCAVGAEAVLEAGKGVGRIVAMSARTPMNFTLGLAKGFRNMPRLYNDNTIRPEEKVSD

FRSGLKVAGKEFGFGLYDGISGLVTQPLHGAEKEGAAGLVKGIGKGIAGIVTKPAAGKLF

NPLR

>SoG_05488.T1

MFFLVNKERRVTLHPSYFGANMKTLVSSKLLKDVEGSCTGTYFIVSIMDTLDISEGRILP

SSGLAEFTVKYRAVVLQPFPGETVGPLAKGDSGEAASYCCHRRLRNLG

>SoG_05490.T1

MVSQFLQYQHQQYRSAPLSVDTHHAQEYFKQEDTGILDDSILDHSAVDSGLELSPPMADS

RRESFAGMPAHSLFSPQSVDMQSVPSNNPFENQPASYVNLEHAQPGPFMVPGSSWPMTTS

NSGSGTPAQHFDSGMHGSMAAHNPFSSHGNMFSGLPSGNPSLPTSPQGNWTAGSGAKRAR

PESPTLRGHNDLRRGDGIRKKNARFEIPADRNLHNIDSLIARSTDEAEIKELKQQKRLLR

NRQAALDSRQRKKMHTERLEEEKKDFTQMYQNVLDLNDQLRKEIEQLQREKQAYADFVET

SNLDKEEMIRVHTLETAELRKKVGVLTEHIQRLESTSASAGAAQQSSYNNGFGSFEDMNM

PGSWDAGQFLHDYPVEPAQEVKPSMALVTSKKTEPQPATSNDKNSPPGGLLFMLFLVGAF

VLSNRSPQPIPAVSDDVRAASASLLNTVLKDAGVAQGSSSMQPLAPQPSGATSWSDPSTS

MPAAAVDEHQSTLVDFSDSLTQPTQEQTNEQLFSLSAVQYNGVHEQDFLQNAPPKSMSQG

RRNLADALAAMRMTNKHEGAAEVYTRSLLWDQIPGDVVRNFAKMVAECNNAQNTQQCDAS

TKT

>SoG_05493.T1

MPGVNGTPASEALDHTKALELLKEYETKDGLDVHQLMDTTKHGGLTYNDFLLMPGYIGFP

ASAVALDSPVTKRITLKTPFVSSPMDTVTEHEMAIHMALQGGLGVIHHNCSPEAQADMVR

KVKRYENGFILDPIVIDRSTTVGDAKALKEKWGFGGFPVTEDGKLGSKLLGIVTNRDIQF

EDDDNTPISDVMVTDLITAPHGVDLQEANRILAKSKKGKLPIVDKDGNLVSMISRSDLTK

NQHFPLASKLPDSKQLLCAAAIGTRPEDKTRLKLLVDAGLDVVVLDSSQGNSMYQIEMIK

WVKENFPNLDVIGGNVVTREQAASLIAAGVDGLRIGMGSGSACITQEVMAVGRPQAAAVY

SVSSFAARFGVPCIADGGVQNVGHIVKGLALGASCVMMGGLLAGTTESPGTSFVSREGKL

VKAYRGMGSIDAMQDKKAGNGGKDSQKSNAGTARYFSEGDSVLVAQGVSGSVAHRGPISK

FVPYLAAGLKHSMQDCGMQSLNELHECAASGELRFELRTASAQLEGNVNMEAYEKKLYA

>SoG_05501.T1

MENHVSGQSNKQLTKLAHALEVDDIVAQLEGDVDSGLSKSEAASRHSQYGPNELDDGPGV

QPIKILIRQVANAMMLVLILAMAVSFGIKSWIEGGVVTAIIVLNIVVGFLQEYQAEKTMD

SLRSLSSPTANVIREGSSINIPSTEITIGDIVELKVGDTVPADIRYFQALKFSIVPSRSQ

KFRIIESINFETDEALLTGESLPVAKDERTVFDEDTGPGDRLNVAYSSSSVTKGRATGIV

YAIGMKTEIGSIAAALRQKESKRRPVKRKEDGSASPARHAQAWTLTLTDAVGRFLGVNIG

TPLQRKLSKLAMLLFGIAIVCAIIVLAANNFSDNTQVIIYAVATGLSMIPASLIVVLTIT

MAVGTKRMVKRNVIVRNLKSLEALGAVTDICSDKTGTLTQGRMVTKKCWIPTRGTYSVGT

SNEPFNPTDGDLLFSPTPPSKQDPEKANDGEVKPFSDLLENNTELEDLLKIASLANLAKV

LKNKEGEWKAHGDPTEIAIQVFACRFGFDRQKFLDPRAPKVTEVGEYGFDSDVKKMSVII

EDNETGEQMIYTKGAVERVLESCDSIKWDSDTVVDLTDDMRNGILSNMEALASQGLRVLA

LASRAYDPSSGRRASREGPVPREEIERDLIFRGLIGLYDPPRPESAEAVKHCHRAGIKVH

MLTGDHPGTARAIAAQVGILPSDTKLLSQRSMQSMVMTAKEFDKLSDDEIDRLPALPLVI

ARCAPNTKVRMIDALRRRSAFMAMTGDGVNDSPSLKIADVGIAMGQNGSDVAKDASDIVL

TDDNFASILSAIEEGRRMFDNIQKFILHVLAENIAQACTLLIGLAFKDSSGLSVFPLAPV

QVMWVIMITSGMPDMGLGLEIAAPDILERPPMDLKAGVFTWEVCFDMLAYGLWMSALCLS

SFLLVVYGFGDGNLGSECNERYSDQCELVFRARTTTFVSLTWFALFLAWEMINPRRSFFR

MQPGSKKYLTQWCHDVWRNKFLFWAVIAGFVTIFPTIYIPVLNHEVFKHAPISWEWGIVF

IEAILFFAGCEAWKWGKRIFFRRRDRKRGMTADDLERRAFSDFEHESSDEISTGKKQ

>SoG_05513.T1

MGDGWKTIGHATPTDRIWRWDQEEQEKRMIESWDHEDRWLSLQQPILEGPDAKRFDNRLS

KQGILGFGAFGSVEKVEYRSVHLARKRIGRKGWKIEDLRHEGCNMRKLSHDHVVRLVGQY

ILPSRELCLLIWPAAVCTLGEFLEDLEHFRLGQGDREDILKRFGQLGLQDLRAVDNKSNL

GAQPARPFARCPLAFLRTVIGCTAKALAYCHEAQVRHNDIKPSNILLKPDRVYLADFGIS

KDVSNMEHTTTEGAPGTEKWRAPELYRPGKKSMQLADMYSLGLVYLNIATVLYDGRIDEF

DKVLKYDYYITRQEQLQQREERLGPYINNLSELSLANPPHRFRYAGQDTVGPKPLLDLIR

RMVSSTPKVRPQAKEVDEHLFLLGGVHQQYHGSCCRRSEEDVVDEWNKKLDSIMSENVRQ

RKRIEELEGRDRTYERRLENERRKQEQHMSSLQKRLEEAEQKYRQLAQESGRRRSGHFHP

PQGKRTAPAVPPNPAIPGPAMPHSQVRPTATTPHRPPLYIKAHSAQQPVASNRDPPDHPA

VRRITPAHRAVTDPVTQKALNGMANTSLEHRKNSTTSRIPLPITPTRVGTPSSLRDPSST

DVSLASSVFSRKSIDTPTPPVVTTPSTDVNDNEDKTAVRPKLSMQHTESWTSLGTTSPTA

TTTSSEPYSPELVSLEEATLGDLPKIRGQRTTNASKRLSWADVARKF

>SoG_05523.T1

MSAQAYAPSSATSASHPYTCNTCQVAYRNIDLQKAHMKSDWHRYNLKRRVASLPPITADV

FTEKVLQARAATTAEADKAMFERACEACQKTYYSENAYQNHLSSQKHKANELVSAKRPND

ETTSVISSTFSLGEPIPVRRSSIDSAAEEEFTHVVESLQKFGEERARTSPVKRPSNPHAS

SKAHIDDEDTSKDAGMEESSDATTAAQKEVDLEARLKSCVFCNYRSPTLPLNTSHMERFH

GMFIPEKSFLVDLEGLVAYLHTKVQEYHQCLYCNKFKSTAFGAQTHMRDKGHCKIPFDSE

EEQLEIGEFYDFRATYSDSEPSEDEDEGGIHNDQEKPRSAKLGALRSDKDLEEEEADADG

EGWETDSSASSLDSADLTAVPAEGHLHQFERLGKHPHHSRRDPRHRHQADGWHSHAHKHT

HAVFYDDYEMHLPSGRSVGHRSLNKYYRQNLVNYPTPLERLQRQAIEGPEADDENQVRSV

VSRDAAGMMGVSLQKRREVRQVEERGRKLEEFHAKRNEYKYGKKLNNQKSYYYRYQRGG

>SoG_05530.T1

MANFLASIFGTELDKVNCSFYFVSRPLPHGRCQLREARGDPRGNTPCSQIVPANILDQKI

GACRHGDRCSRKHVKPSYSQTILMPNLYQNPAHDPKNRMNPSQLQNHFDAFYEDIWCELC

KYGELEELVVCDNNNDRESCAFLPDDQVNVLVLTLSTKSDLIGNVYARFKYEDSAQKACD

ELNSRWYSGRPIYCELSPVTDFREACCRLNSGEGCVRGGFCNFIHRKNPSDELDRDLTLS

TKKWLKERGRDERSMSRSPTPEPTRKRYN

>SoG_05573.T1

MDFLQRLARFLDRPLFPWKKLIIGFNIGQYVFESFLTLRQYRILQQKQPPKVLSKEISQD

VFDKSQAYGRAKAKYSLLDGLWTQICDIAFIQFDVLPKLWSWTGDLLLKWAPARFTGEIS

HSILFVISFILVNQALSLPSRIYHTFVLEEKFGFNKQTPRLFISDMLKTNLLTFVMAPPL

LAGFLKIIQKTGDRFFFYLWAFAAAVQLFMMTVYPTVILPLFNKLSPLEDGELKTKTEAL

AASLNFPLSDLFVIDGSKRSAHSNAYFYGLPWKKHIVIYDTLLEKSKTEEIVAVLGHELG

HWKLGHLTRLMSISQVHIFYIFTLFSVFINNNSLYDAYGFHKDHPIIIGFILFSGALSPM

DTVVKYLMNVVTRKFEFQADAFAKQLGYASELSRSLLKLHIQNLSTMDADWLYASYHFSH

PHLSERLKALGWTGETEVTSKMDKEGVAQTTGRDEL

>SoG_05581.T1

MAQSGSMDDFVNWDQAGAAIESSQGQQKQDFPVVKTPSVSPEAPFDDNIDLVLANVIEDD

FSFHALQHFTNELSAPLQHIDPLLQELNSTVSNDGFTPMQWETPALPCAQCALGGYSCKK

IREGAYQGFCTSCVALMIDCSFGGVVPPNLNPASTTLPPVESTLACSNSGIATPLFEDGE

HHVADPLQPLSSVSALELFNMANGSSDSNSRANLQGPVTAPKVGARLSRESVRVLKNWLS

THNRHPYPSDEEKEMLQRNTGLSKTQITNWLANARRRGKVQPQRSTSPQVGNWSSGLEIP

ARRGTPALECMNPLQRWQNSPPEHEPASVTAIARAVTTSSSAVSSGLNSPFSSFPYTDDG

SSRSICNQSSASSIGTSKSSNGSLGSAYSHGSRNSFGSFGSAPFSSRNRRRRRRRAAPRQ

SPDGLKAFSGPLKTFQCTFCTETYRTKHDWQRHEKSLHLSLERWVCAPEGARVLRPETGK

VCCAFCSEPDPSDAHIESHNHSSCQERTLEERTFYRKDHLNQHLRLVHNVKFADWSMKSW

KVAAPEIRSRCGFCGIILDTWGTRVDHLAEHFKTGKSMADWEGDWGFEAPVLAMVENSVP

PCRASHESPRNAYELIKLELAYYAGNYQQQDPKDKPPSDEEMMIEACRIIFASETLSLQG

IATQVSWLRDIIMSNEELALKARFGPLRSATENRLATLKINGKDNLFEECPMEQQLHEFV

RAKRLLGLTAMDNELQEEACKIVGRVEEVSTNPSERVANWLVRLATSSTKWLAPFRQRAH

LPRSDDIKNDVIRSTDPTSIDSTIHSYSRLERELAEFLQTQRAMGTEPTDEDLQRQARII

IYEFDDGWNQTAADSAFWLNGFRSRHPPTEVSPAASGISSAFSHLRHTGSTASTVPSSTG

PVGPTGLHAAGRQGYNDPMSERMALNPSNCYHGLERELSRFVKSMMSPNNPNQHVPTDEE

LQHQARWITHNE

>SoG_05582.T1

MSQPRETGYSRTALPSRPVQSTTQGHGSQHLHPPTSNPSRERNLLPHPRDDQSVSPEPRD

SLMSDVMSLSSKASSPDSHVGESLAKANRITSSAATPSGQICSNCGTTQTPLWRRSPQGA

TICNACGLYQKARNAARPTSLKKPPNMVSTGASRQNSTKGTSPSTSKAATNIPGATYVDA

SQMPKGTCPGGGRCNGTGGAEGCFGCPAYNNRMSKSTHVNVKAKSGCGSQATESSREDSE

IDVNALQAQGPDTAVVIACQNCNTTITPLWRRDEAGHTICNACGLYYKLHGVHRPVTMKK

ATIKRRKRVIPADQDEPAEADTPPSQAPERTPERGTINDDGSINLGLRRRPDHPLAIEPR

SASLHTSRQTPPLSSTSDLAAYRQEGLRPQSMHSLSDSNRLAPMNPMPSSSNRQSNSPDS

FASPSRKRSFSTAETDAAQGDHGSNETPMRLSSIRSILNPQMGGDRDLSDHSLPPLRSPA

ATMPSLGSPSGSVSSRSHTPSTLQPSVLPGTANDADRLKADRRAALQREAERMREMLAAK

ERELMELGDN

>SoG_05594.T1

MLHIHRLEGGLDISTPLEGKHSPPSYAAADRGYGPPRGPSSAYPADVHAPPQAPQYPSGG

YRGGRGGGTRGSFSGRGGRGDSANGPPTSGANAVDVAERDREPGGSSRTRDDGTPENESA

SDGRPMLPPNRPPPTGPAAHNNSSSKFSFSFKASSKPTAAAPKPEISQKFNAAPKASLPS

SNHDREPPPSAPTEPASARARNDVRVPDGPRMAPRMRKVRKIMKRPKPRPTLPADLAASE

SVFYRKPGNESVVGSGTYGKVFKGVNVYTKGLVALKRIRMEGERDGFPVTAVREIKLLQS

LRHTNIVNLQEVMVEKNDCFMVFEYLSHDLTGLLNHPTFKLEPAQKKHLAKQMFEGLDYL

HTRGVLHRDIKAANILVSSDGILKLADFGLARFYAKHHQLDYTNRVITIWYRSPELLLGE

TKYTAAVDVWSAACVMVEIFARSAIFPGDGTELNQLEKIYNILGTPNRRDWPGLVDMAWF

ELLRPSVKRKSVFAEKYADKVTPAAFSLLEAMFLFDPAKRPTAAQVLQHAYFTTEEPLPK

QAVELASIDGEWHEFESKALRKENERKAREARRAEAAAAAAAGGGTNLTKDRDRKRSHEP

RESSSQRDAKRAHLDGQEPTTMVKSEEGSNSQGGVTAA

>SoG_05598.T1

MPQSAPTTTALPDSSSSPPTSHTKTTKTLAASQFPLKTPAATSSSSKKPVLARSVAPSSI

QRGPAVLKPNTRKPAPQNALSPAAVAVASTSPPADVMTMSAVISPAPTPDAPIMSMTSKE

WVIPPRPKPGRKPATDTPPTKRKAQNRAAQRAFRERRAARVGELEEQLEQQKSESDQRDA

TYKDTIRDLELEVQSFRSRCLLLENMLERERKERLQAETMLETMRRQADTRPAMRSNLPD

PSYQSSYAGTQQRSQHHQHQSRHNSSDSSRQEASNTLPPLANTPSAPAPTQGYFDSDASL

TCGGCEPSGRCACVEEVLNSSCGNCGTGDSCKCAEEADAVLNAGPTLKRSASPTSTAPVQ

KRARSSPDADSPVEVDFTNMFQRKSTVEISQPSQFDSSSAMSGPPSLDNTFLKDNCGFCK

DGTYCVCADTAMATPAMTPVDTLPPIASQTQTPPPSEADVVSQPFIMEMTAEGAVKLPKR

NKPKPTAAQAPSRGCGSGGPGTCDQCRADPKSGLFCRLMAANFSKGEGGCCGGKGADGGC

CKSKPAAKAAAPAKEKITLPSLPSLGLSCAEAYQTLASHRNFDRAADDIGSWLPKLRTMT

PASRAAATAKGGRMGPIEVEAASIMSVLKDFDVRFGRDA

>SoG_05604.T1

MAAEQRKLLEQLMGNTNQSRSSQISLTDPKACRSYIVGTCPHDLFTNTKQDIGQCTKVHS

EALKSEYEAMPERERLRLGFEYDYVRDLQKYIEDCNRRIDAAQRRLEKTPDEIRQTNVLL

KSISELSSSINSGLLEVEILGSMGEVSRAADELFRVRHATQAKGERERELKALSDTSGPS

GHQKLQVCDVCGAYLSRLDNDRRLADHFYGKMHLGYAQMRKTYESFPKEMRGRVRGGGGG

DDDTGPRGGRQGGGGYRGRGGRGHRGGW

>SoG_05625.T1

MVFYPPSWVPTPNLDVPDSIPIAEFLRNEKYGRRAIAKSRNPFTCGLTGHTRSVDEFFHR

SDCLARAISKRTGWQSNEGTCWDKVIGIFSFNTIDYLQIPYAIHRLSGICSPANAAYSVP

ELAHQLTSSGVKVLFTCVPLLDSALKAAEAAGIPNDRVFIIPMATGDKPVPFQTIEDLVS

EGEKLPELEPLQWAKGQGSRQPAFLCYSSGTSGMPKAVMISHRNIIANMVQYTTYESVGR

SLKGVETQVVLGLMPMSHIYGLVTISHCAPFRGDEVIVLPRFEIETYLAAIQRFKIEQLI

VVPPIIIRMLQTRDLCKKHDLSSVRFVYSGAAPLGEETIQELLSIYPKWAIAQAYGMTET

SVVVSSPSEHDILPGASGSLLPGFRAKIIDPNGVEITAYDTPGELLVQSPSVVLGYLNNE

KATTETFVHDDDGRWVKTGDEVLVTKAPSGNEQLVIVDRIKELIKVKGHQVAPAELEAHI

LAHPAVSDCAVTQIPDKSSGEVPRAYVVKSPEFASRSDEEIAQIVIQHVKDHKSSYKWIR

GGVEIMAEIPKSPSGKILRRLLRDREKARRMKEGAKL

>SoG_05628.T1

MNNYALEKPSERSRWTPLTRMLLSGEMTQEKQQTLSTREKFDRWMINEGYRRVFVFVFML

AHGLIFAFACVHFSMKDSLATARSIFGFTFITARSSALVLHVDVGIILFPVCRTLISLLR

QTPLNGIIQFDKNITFHITTAWSIVFWSWVHTVSHWVNFGQVAAKNNLGFYGWLLANFAT

GPGWTGYVMLIALMGMVFTSIEKPRRANYERFWYTHHMFIIFFIFWAIHGAFCMIQPDVA

PFCTSVGASAIGVFWQYWMYGGFCYLAERIAREVRGRHKTYVSKVIQHPSQVCEIQIKKE

NTKTQAGQYIFFCCPAVSLWQYHPFTLTSAPEEDYISIHMRCQGDFTTAVSTALGCEWKK

KGDASKVVGVNNDGNSSGVDPALRRVLPRVYIDGPFGSASEDVFKYEISVLVGAGIGVTP

FASILKSIWYRMNYPKKKTRLSKVYFFWICRDFGSFEWFRSLLLAVEAQDVEGRIEIHTY

LTAKIKADDATNIMINDANADKDTITGLRSPTNFGRPNWDMIFRGIRKLHTPAEAGVFFC

GPKGLGSALHVYCNKYTEPGFSFVWGKENF

>SoG_05644.T1

MMDSPRELKPSAARSERGEPMDTGAESAGETPSAATANQTSTGNTPQTTSGTASQGNPTP

GSSSTNPSTGSAPSGLPGSATAASSSSASQVSSKRRRGLGVVTPNACTECRKKRAKVRPA

QALRVLLLFHLGHNVRQPLTEKSLLIYHAQCDGNKPCGRCKSQKDVECIYEVPIRQSKEN

LRNEIESLRARQRSNEQVINALSRPDLWEEVLARLRNGQSVEAISDWLGSNLAPAQPQGS

QLPPFPRIHSDPIPRLGTGQGPTLAPISSFMSAASRAIPPSGPQLAPLNLGGIGGMSPIA

TQQPPPLRNDPVHGSPWNLSPHSHPGSNRSSSHPDAMNWQIDMPGPPQFRVASWASDTTP

PDHMKERMPRYRGLEQILSPLSEPQLRSPSGPWTNITTDVNLVLHLLALYFCWEYPTFAS

LSKEHFLRDFQDGRHRYCSPILVNALLALGCRFSTQPMTRADPNDPYTSGDHFFKESQRL

FYQENDHHSLTTIQALGIMSIREASCGRDSESWYYAGQSVRLAIEMGLHRLHDRGDEDEL

AVQAATFWGAFALDHAWSLATGSLPQCSCFPHLPPKPAIISDIEASLWVPYTDDGAPLQR

SCSQPSNVRSVYKCFCELSELVHESLYILHSPGKPLTARHLLKIYTQYLNWYDRIPEVLR

LGHNFTPAVLFAHMYYHFAILLLFRPLIKLRILGSKISPKDVCTQAADAIQGLLKSYSQL

YTLQRTPSFVPYFVLTSSIMHLAIGASEQGNASLSSTPKERIEKAAQLDPRIKESLKQGI

ADLAEMAPCHHFAEQALNILRYLAKKWNIEVDIDQGNSPPPHQDYDSLVKPYTASLNFFA

PNVSSGDFDCEWGFAEEEPETRATSTSTSTSTSQAPKDAVAGARGRKESSIIVTAAEGME

NPLFWPFPMQGRPILSSGKELAAAGFAML

>SoG_05647.T1

MTQSPSSSSVDDPAENTADEEDSEDYCKGGYHPVQVGEKYKDGKYTVVRKLGWGHFSTVW

LSRDNTNGKHVALKVVRSAAHYTETAIDEIKLLNKIVQAKPDHPGRKYVVSLLDSFEHKG

PNGTHVCMVFEVLGENLLGLIKKWNHRGIPMPLVKQITKQVLLGLDYLHRECGIIHTDLK

PENVLIDIGDVEQIVKKVVKNESSDKENNRNGRRRRRTLITGSQPLPSPLNTTFNHANMF

PSISGQSGASKPRTPEEQKHREKSAYVLQHLPLDPGFVDLGIRNLTEFRDMLSREVSGIS

LDKSSGSASAGDKRKADDAYALDVINVKIADLGNACWVNHHFTNDIQTRQYRSPEVILGA

KWGASTDVWSMAAMVFELITGDYLFDPQSGTKYGKDDDHIAQIIELLGPFPKSLCLSGKW

SQEIFNRKGELRNIHRLRHWALPDVLREKYHHREEEARRISDFLSPMLELVPEKRANAGG

MAGHPWLDDTPGMRGVRIPGLEVGSRGDGIDGWATEVRKR

>SoG_05650.T1

MITPRTLLLSALLLASNALAQVYQTPGRAKDAYSYTQPKDTVILGQYGHSEPIYPSPRAN

GKGPWQDAVRKAKAFVSQLTLEEKTLMVTGQTGPCVGNIYPIERLNFTGLCLQDGPASLR

TADFVSVFEAGVTIASSWDKEMMYNRALAMGREFRAKGAHIALAPAVGPMGRSAYSGRNW

EGFSPDPYLSGIAFEKSIRGFQDAGVQATAKHYILNEQEILRNPVIFPNGTVQYESISSN

VDDRTAHELYLWPFANAARANVAAVMCSYQRINGSYACQNSKTLNGYLKTELGFQGYVMS

DWYGTYSGVASIEAGLDMDMPGHIMGRTLNEDKQSYSSFFGGNITTGVNNGTIDTQRLDD

MITRIMTPYYALHQDDGYPSVDKSIVPLNTFSLPSTWLREWNFTGTSRRDVRDNHGELIR

KHAAAATILLKNKDKTLPLKAPKSIAVYGNDAGEVTEGPLNQGQFQFGTQAIGGGSGAGL

FTYLISPIEAIKARARQSGALVEAWLNNTMVLGRDSNAWAQTPLPNQPEVCLVFLKGWAA

EAADRESLNLDWQADELVESVTSTCNNTVVVTHSSGINILPWANNPNVTAILLAHYPGQE

SGNSIVDVLYGDVNPSGHLPYTIAHNATDYNAPLVTAINTTGSEDWQSYFDEKLETDYRY

FDAHNIDVAYEFGFGLSYTTFELSGLKLEAKASNITSRPEEREIQPGGNPALWETIYTAE

VMVRNTGDVRGSAVPQLYVSFPSSTPEGTPPRQLRGFDKIELGAGQRSTAKFELMRRDLS

YWDINAQQWLIPTGEFTIHVGLSSRNLIEKASVTVVSG

>SoG_05665.T1

MSSANQQNVIRRKLVIIGDGACGKTSLLSVFTLGYFPTIPTVFENYVTDCRVDGKSVQLA

LWDTAGQEDYERLRPLAYSKAHVILVGFSIDTPDSLDNVKHKWVEEATRLCAGVPIILVG

LKKDLREDPIAIEEMRRKSQRFVSTHDGEAAAREIGAKRYLECSSLSGEGVDDVFEAATR

AALLKFEKGEGGGCCVIL

>SoG_05671.T1

MAASNASAPAEGMLWGGRFTGGIDPLMHQYNASISYDKLLYKEDILGSIAFARANAKLGI

ISNHEFDEIERGLREVMKEWEANTFVIMPNDEDIHTANERRLSEIIGKEIGGKLHTGRSR

NEQVVTDMRMWLRERIREIESYLVAFLQVIAARAEADIDHIMPGYTHLQLAMPVKFSQWL

LSYGFSFASDLDRLREVLKRVNRSPLGSGALAGNAFGIDRQMMAKELGFEGILWNSMGAV

ADRDFVTEFLAWGTMFMGHISRFSEDLIFYSTAELGFKKNPDGLELLRGKAGRAFGHFAG

VYCATKGIPSTYNKDLQESWEPMLDHVKTISDSVQIANGILSTLTLRPERMRASLNPSLL

ATDIADVLVKIGVPFRETHHISGRVVAKSEELGITMDQLSVEQLQAIDSRFPDDIKDVFN

YEASVESRSAQGGTSRAGVLEQIEVLKGMLA

>SoG_05673.T1

MQISLSALLASGLITLVTASPVNLRLPLLGRDDCRCLPEDACWPSADEWKRLNDTVDGRL

VATVPIGSPCHEPNYDEDACKKLKDGWLLPQTHLPSSSSLMQPYFANQSCDPFTDKSTSC

QLGNYVVYSVDVAKPEHVAAALKFAKEKNIRPVVRTTGHDFLGRSTGAGALGIWMWHLKD

TSIVDYSDPFYSGPALKVAAGVIGAEAVEAANAQGLAVVTGECPTVAVAGGFTQGGGHSA

LSTAYGLAADQTLEFEVVTADGRTLTASRTQNSDLYWALSGGGGGNYAVVMSVTIRAHRT

GKVGGAKLAMSSAYTTPENWEKAIAEFHKLLPGMIDQGASVVYYVSSKIFAISPITLVNS

TGDVVRDEVLAPFTKKLAELLIPSQVSYSTLDYRDHYDTYMGPLPWGHIEVSAYQFGSRL

IPRSLLVDEPEAFQSAIRNLTNSGVLAVGSAAAYKSYTKAPNAVLPAWRDAYIQMQLTTP

WSNDPSKLGAMVEAQHRMTDEFNPQLARLTPGGGAYMNEADFREPNWKREWFGDNYDKLL

AVKMKYDPGNLLYMFKGVGSDAWNVEESGRMCRT

>SoG_05682.T1

MMPLAPPADQGPSPPAVPAATHIAPAPPGSLPMDDGSGDGRKAKRELSQSKRAAQNRAAQ

RAFRQRKEGYIKKLEQQVRDYADIEQTIKVIQNENGALRDYVAHLQARLLEVSGDFPPPP

HGLNLSHPAGTSSAAAAPMTSGEAPQDHVVSDAAESSTALEAEAVAVAGLAAQEQMAAHV

DRYPSPTYRPESGGEDTRSADEINRHLGATDGGAGQPQAV

>SoG_05729.T1

MSSPGGNEEPQRRPSGIPLQTINTSPPGEATNSRSSGASLTKPLNQSDSSSATTSANLLG

PSTYWDQPYDAHYHDIDGLESPIDPSALQAALPPNIRGPITHGSGRLIGAGSPYIDETPA

SDYSGSDREPLTAHPQPIAGSLAANLNDGQPRDSFQTVSDVDNTGRDAQGHRQDIEHGLR

PHQHHGFGMSLNPNEYRLSRSSSSAGALQRAGSIVRAMSQRVVNISGESEIVDNRASRHR

SSSPQPSLNQSRSGSRTQTPAHSREPSRTRSISMLGDTSYHSQAANLSPEKDDRQHLAPG

GPPYQVPLQPLPNPLRGKSLGIFSRDSHVRNWLCDLLVNPYTEPFILVLIVAQTVLLAVE

SGQSVFEPGHERPEFWGQQPTDWAMLGLFIVFTIELITRIIVSGFVLNAAEYSTIDRQKG

IRAAVADQYRAIFQPHQAKTARNHNSFKPTPSAFARSFTTILQGPQRLPETLEEQQRYQL

ARRAFLRHGFNRLDFIAVSAFWISFILGVTGAEKHRGLYVFKMLSCLRILRLLSITNGTA

VILRSLKKAAPLLVRVAFFISFFWLLFAIVGVQSFKESLSRQCVWVDPQDPNNLTAAFTN

ADTFCGGYLNATTGVEEPWVRMLTPGSLAELDAGTSEAKGFICPRGSLCLQQGNPQGGTV

SFDDIFHSLELVFIIMSANTFTDIMYWTMGSDYMVAALYFGAGIMIMLLWLVNLLIAVIT

SSFQVIREESKSSAFTTAEEPSTQVHAEARFRKPSFIQNVYTYSKNFWITVIIFGLVASS

LRSSSMSAARGRFIDLAEVVVTILLDLEMILRIAADWRGFHRSWPNLFDLSLAVVTSIIL

LPPIRGSRVYWWLSIFQIIRVYRVVLAVPVTRKLILLVLGNSSGIANLMLFVFLTTFLVA

ILAAQLFRGVLPEGSDVTFYNMFNSFVGMYQILTSENWTDILYNVTETTTAFGTSWIGAT

FLIGWFILSFFILINMFIAVIQENFDISEDEKRFEQVKAFLQRREIGSSSSNLALSTIFS

FGRTRQRKDPLDYGPALTEMLLKDSVVSDFLEESYNRGAGKSEQEPGPTRATTGLLTNDI

RPSYLSTLWGKLVRKMIKREPNPFYSNIRLENSSEHFDAREMAQQAVNATSARRKAQREY

LAKYPNYNTSLYLFPPKHKLRRFCQTIVGPARGLERIDGVEPNKIAWYSFSAFIYCAIVA

MVVIACVTTPLYQKQYRAEHPASSFIWYVWTDMAFAILFSAESTIKIIADGLFWTPNAYL

RSSWGLVDSVVLITLWINVVTMFVNDGAISRAVGAFKALRALRLLNVSDSARDTFHSLII

KGWWKILAAAFVSISLLIPFAIYGLNLFNGLLIACNDDDDIVSLASCFGEFGSTPFSNDW

PMLAPRVATNPYFNFDDFGASLFTLFQIVSQEGWSDVVFAASAITSRGMQPQSLASEGNA

VFFIVFNILATVFVLTLFISVFMRNYTEQTGVAFLTADQRSWLEVRKLLRQISPSKSSYN

DKGKAWKKWCHKRAIEKRGKWYQAVTVVLVLHLIMLVTESTSDPEWWRITRSFTFLGFIV

IYMANITIRIIGLGWTRYRRSSWDLYALIAVFGAFVSTAAMLIANSGNVQVRDETYVQVH

KFFLVAIVLMVIPRNDALDQLFKTAAASLTVIGNLLATWLVLFLVFAIAFTQAFSLTRFG

SNETNNVNFRTVPKALILLFRMSFGEGWNSLMEDFAFIEPPLCVEQENDFFRSDCGSKSW

ARFLFVAWNIISMYIFVSLFVSLIYESFSYVYQRSSGMGAIDRDEIRRFKEAWRSVDPDG

TGWISKETFPRLLGELSGVFQMRIYEPEDSIHSILEDIRGHDARGGRHLSIVSSTNATSI

DLDALNQRLALLNVSKIRERRRRFNLFYGEIMTTADVDRGIHFTNVLMTLAHYNIVTDSK

SLKLDEFLRRRARLQRVEEDIRRRIVQGFFNTLYLSREFKKHMRQKDAGRMTGIPEIMVP

DIYVGDDNIDDLTDDGKHRTSGLVTPASHLSVDLSQGYRGSRASGGSRGSSSARQSAWIN

AGQHPLSEPRAQYSNPASPSHQATTSAFSFELFDPDAQGSSTNEMRRRGSANSLTRGPDI

MDDTVWMDSIRKSTTIRRSGKTASYRFTDLG

>SoG_05743.T1

MATPNTTPPDQGEYLKPSVNKTQRVLACVLCQQRKIRCDRTFPCGNCKKHNARCVPATQT

RPRRKRFPERELLDRLRRYEALLRENKVKFEPLHKQDYGNGEAIGEDDGDESGAELDKAG

IASPSGSLKSENVTEARSIWQAISQESPSNPGKDTPIANAIESSIKSAWDQLMDGDAILL

GMNRSPVDLSTFHPDPVTAFKLWQIYLDHVDPLLKVTHTPTLQPRFIAALGQIGAIQPEF

EALMFGIYSMAVLSLSPEPCKEIFGMDRDDLLTKYQFGSQQALMNAGYLRSHDRECLIAL

MYSLASHPMGQPLP

>SoG_05766.T1

MEAIKKTFQRCKAQNRPALVTYVTAGFPKPENTPEVLLAMEKGGADIIELGAPFSDPIAD

GPTIQTANTVALDNGVTIGSTLGMVKEARSRGLKAPVMLMGYYNPLLSYGEERLLKDCHD

CGVNGFIVVDLPPEEAVSFRKLCSKGGLSYVPLIAPATSDTRMRILCQLADSFIYVVSRQ

GVTGASGSLNANLPELLERVKKYSGNKPAAVGFGVSTREHFLSVAAIADGVVVGSQLVST

LHKAPAGQEAAEVEKYCAYLCGRDNAVGADNTREVGIVEAMAEAKEPTGATVDAVITDAD

RLTDEQDSDLVAQLAALHGKIPDRFGEFGGQYVPESLMDCLSELEDGFNKIKDDPAFWEE

YRSYYPYMGRESSLHLAERLTEHAGGANIWLKREDLNHTGSHKINNALGQLLLARRLGKT

KIIAETGAGQHGVATATVCAKFGMECTVYMGAEDVRRQALNVFRMKLLGAKVVAVEAGSK

TLRDAVNEALRAWVVELDTTHYIIGSAIGPHPFPTIVRTFQSVIGDETKRQLREMRGKLP

DAVVACVGGGSNAVGMFYPFSNDPSVKLLGVEAGGDGVETERHSATLSGGSKGVLHGVRT

YVLQDKNGQISETHSVSAGLDYPGVGPELSSWKDAERAKFIAATDANAFYAFRLMSQLEG

IIPALESAHGIWGAIELAKTMKKDEDLVICLSGRGDKDVQSVADELPRLGPAIGWDLRF

>SoG_05769.T1

MRSKFKDEHPFEKRKAEAERIRQKYADRIPVICEKVEKSDIATIDKKKYLVPSDLTVGQF

VYVIRKRIKLSPEKAIFIFVEEVLPPTAALMSSIYEEHKDEDGFLYITYSGENTFGFESA

>SoG_05775.T1

MAGGQPRGLNAARKLRTNRKDQKWADLHYKKRALGTAFKSSPFGGSSHAKGIVLEKVGVE

AKQPNSAIRKCVRVQLIKNGKKVTAFVPNDGCLNFVDENDEVLLAGFGRKGKAKGDIPGV

RFKVVKVSGVGLLALWKEKKEKPRS

>SoG_05792.T1

MSVVSLLGVQVLNNPAKFTDKYEFEITFECLEQLQNADIDLEWKLTYVGSATSDQHDQEL

DSLLVGPIPVGVNKFIFEADAPNTSRIPDAEILGVTVVLLTCAYDGREFVRVGYYVNNEY

DSEELNAEHPSKPIIDRIRRNILSEKPRVTRFAIKWDSEASAPAEFPPDQPEADLVADGD

QYGADEEEEEEAEGEAVAAAEQTGDDAEMAGVDQENGKAPRGEAEEDEMSEDGSVDIEGE

SDEELEEEEEEEDGKEGDVAEGGEEANGDAMEVDAAEKADKPAPTPATQPVTVAS

>SoG_05803.T1

MAAVYKSLSKKESKKAEAPAANGVQKNKQRVLILSSRGVTYRHRHLLNDLAAMMPHSRKD

AKFDSKTKLYELNELAELYNCNNVLFFEARKAKDLYVWMAKAPNGPTIKMHMQNLHTMEE

LHFTGNCLKGSRPILSFDATFDKQPHLRLIKEMFLHQFGVPQGARKSKPFIDHVMGFSFA

DGKIWVRNYQINETEVSKLKPSDEKADEDDLSLIEIGPRFVLTPIVIQEGSFGGAIIYEN

KEFVSPNQIRADLRRNKASRHNARAEKQVERLSRKGELGLRTEGGVRLAKDELDTKMLFS

>SoG_05808.T1

MTTSTSTLNKVSYPGLALLALGVTAAMAATAQYTTNNTNNNNNNNNNNNNNNNSGGSKQE

STTPPAKTSHKKAHASPDIAGAGSVPPPATLTPAQVAIVKSTAPLLKEHGEAITSLFYRN

MITSNPSLNNVFNRTSQVTGAQPRALAHAVFAYASHVDDLAALSAAVARMADKHVSLGIR

PDQYPIVGEYLIAAVAEVLGDAVTAEVAEAWTNAYNVLANILIDAERELYAGFRGWDGWR

RFTIQEKVAESSEITSFRLVPVDGQPLPGYKPGQYVSLRVWVDELGCMQPRQYSLCEDPD

RAAAGAYYRIAVKREPAGAAGTPAGMISNRLHDEYHVGDEVELTHPCGVFFLDETAPKSS

PVVLISAGVGITPMISILNHITAATPSSSRRPISFIHGARTAGTQAFAEHLRSLAAAHPN

LDTTIFRSHSVEEHEVQGTHYHFAGSRINLAKVDAERKLFLHDPEARYYICGPLAFMHQT

QAYLREAGVDDGRIHMEVFNTGGV

>SoG_05821.T1

MATQQQLALERLEVITRVLRSRTSDETRIRAAASLRELVVFCHRDLSPEQFLAFYNAVNN

KITQLITHGSDSAERLGGIYALDALIDFDGVDVAVKYTRFTQNLKTILRGKDINPMQPAA

IALGKLCRPGGSLISELVDSEVNTALEWLQNDRVEERRYSAVLVLRELARNAPTLMYQYI

PTMFEWIWVGLRDSRQLIRATSAETVSACFRIIRERDQDMKQQWMGKIYNEAKQGLRMNT

IESVHASLLVLKELLEQGGMFMQDHYQQACDIVFKHKDHRDPTVRKTVVLLIPDLASYAP

NEFAHSWLHKFMVYLSGMLKKDKERNDAFLAIGNIANSVKSAIAPYLDGVLIYVREGLSV

QSRKRGSVDPVFDCISRLAVAVGQTLSKYMEALLDPIFACDLTPKLTQALVDMAFYIPPV

KPTIQERLLDMLSVVLCGEPFKPLGAPQPNTLQSVPIIAKDAKDPQAYEHRRAEVKLALN

TLGSFDFSGHVLNEFVRDVAIKYVEDEDPDIREAAALTCCQLYVRDPIVNQESYHALQVV

GDVIEKLLTVGVSDPEPNIRQTVLAALDERFDRHLAKAENIRILFFALNDENFAIREVSI

SIIGRLARYNPAYVIPSLRKTLIQMLTELEFSDVARNKEESAKLLSLLVQNAQTLIKPYV

EPMISVLLPKARDPTPSVAATILKAIGELATVGGEDMLPYKDRLMPIIIDALQDQSSTSK

REAALHALGQLASNSGYVIAPYLEYPQLLEILQGIIRTEGQRGPLRQETIKLMGILGALD

PYKHQQVEEQTPDLQRRAESSQMTDISLMMTGLTPSNKEYFPTVVINALLQILRDQSLVQ

HHASVIEAIMNIFRTLGLECVSFLDRIVPAFLQVIRSSSTTRLESYFNQLATLVSIVRQH

IRNYLPEIVDTLREYWNLSSSLQTTILSLVEAISRSLEGEFKIYLAGLLPLMLGVLEKDL

TAKRTPSEKVLHAFLVFGASAEEYMHLIIPVIVRTFEKQGQPLFLRKQAIDTIGRISRQV

NLNDYASKIIHPLTRVLESGEPTLRLAALDTLCALIQQLGKDFIHFMGTVNKVLQSHQIQ

HQNYELLVSKLQKGEVLPQDLSSGHRFADDLDESPFADLAPKKLEMNAIHLKAAWDTKGK

STKEDWQEWLRRFSTTLLTESPNNALRACASLASVYLPLARDLFNSAFVSCWSDLYEQFQ

DELIHNIESAIKSENVPPDLLGLLLNLAEFMEHDDKALPIDIRVLGREAARCHAYAKALH

YKELEFLQDTSSGAVEALIVINNQLQQSDAAIGILRKAQLYKEGIQLRETWFEKLERWEE

ALAAYNKREQEIPEDQPVPVEIVMGKMRCLHALCEWDALATLTGNTWANSSPEVQRKIAP

LATAAAWGLEKWDAMDNYLSSLKRNSPDRSFFGAILALHRNQYREALACVQQAREGLDTE

LSALVSESYNRAYQVVVRVQMLAELEELVTYKQSDEKKQAVMRRTWETRLKGCQRNVEVW

QRMLRLRALVMPANENMHMWIKFSNLCRKSGRMGLAEKSLKSLVGTEAPLESIIPYWNDR

PASGLPRNIPAQVIYAILKYQWELGQQPAARQKGVSEKTLYCLRKFTNDTAHRLDVAKAH

LAAQAGGEVNLSPELGFANHTDRNMVSPSTQRALYDQTVLLAKCYLRQGEWLIALNKDDW

QYTHVQDILTSYAHATKCNPRWYKAWHAWALANFEIVQTLSSRNDGQMSRADHSMVIDHV

VPAVRGFFKSISLSAGSSLQDTLRLLTLWFTHGGSADVNTAVTEGFTNVSIDTWLEVIPQ

LIARINQPNKRVQATVHNLLADVGRAHPQALVYPLTVAMKSWQNTRRSRSAAQIMDSMRQ

HSANLVAQADTVSHELIRVAVLWHELWHEGLEEASRLYFGDHNIEGMFATLAPLHEMLDR

GPETLRETSFAQAFGRDLKEAHDWCRQYETSKDVNDLNQAWDLYYQVFRRISRQLPQVTS

LELTYCSEKLLAAKDLDLAVPGTYHSGQPIVKIKSFETTFSVINSKQRPRKLNINGSDGV

SYAFLLKGHEDIRQDERVMQLFGLCNTLLANDSECYKRHLNIQRYPAIPLSQNSGLLGWV

PNSDTLHVLIREYRESRKILLNIEHRIMLQMAPDYDNLTLMQKVEVFGYALDNTTGQDLY

RVLWLKSKSSEAWLERRTNYTRSLGVMSMVGYILGLGDRHPSNLMLDRITGKIIHIDFGD

CFEVAMKREKYPERVPFRLTRMLTYAMEVSNIEGSFRITCEHVMRVLRENKESVMAVLEA

FIHDPLLTWRLTNAASPTGPNFRSERESSITGPNGARARRQSILDSDVAPSELIKASEPG

APPTSRSRARTNSSAAADGHLTNGGQEAESQNARAVEVLDRVSQKLTGRDFKNNEELDVI

NQVNKLILEATKLENLCQHYIGWCSFW

>SoG_05837.T1

MSRRQAQSNELATRLSVTIIPVIIIGTFGFATWAIIDHLCVRYLIRVRHETGTATAFLVL

YAILFPITIITYIRLFYTVQTNPGAVPWSDERQREEEERQRQKRSLPRRSWWKRMRRTDP

EEQPWNPPDANPDSPGLENFYSKNVFVCEVDGRPRWCSECRSWKPDRAHHSRELGRCIRK

MDHLCPWVGGMVSETGFNFFFQFTAWCFCYCVLCLVLSVYCLVKKKEESGDLDGVTIAGV

ALSAMFGFFAFGMTATSGSYMLQNKTTVDMLRKDKVHQLAVRVPRGTPSTSAYRTITYPL

SPYDGWPAPDTQIDGHDQAPLYSAPTNRDVQATRTFAILKTEPGENPWDLGPLENFKAVM

GNTIWEWLLPIRHSPCCNHDSMDSDYQLGPVVARMARRVDLPRKRSKPERVEMVETNGSG

R

>SoG_05842.T1

MSSGVVRSTALRAGGACVRCRKGKTKCVYENGRAPCKNCAKGMHECYLPSESMAHHHGQS

PARHTTTSSHRPQRESLPGAGAGASDSSRQPAVGATGARHVHAASEKLTPELIAECERVV

SKTFPACVAFHKPSFVQQIKSTTLDPSLIYGLLTCAARSSPTLIRRYGGPTAAAENYAAK

AINLINQNLDHPSLADIQALCLLIIHEWGTRNAVRAYVYLGQAARMIHMYRILDVHHTAN

ESDQFLRMESLRRTIWLVYILDCFLTSSPGRYPALTNSDLTNVALPCSDINFAFGNAVYV

KTLHQHLELSRDDPNNTAEVGEFGHIVLASMVWRETVDLLTRSFPDNYREDDCLAMIGKI

DALRASLPMQFRDKPGQINLHMTMGSGYTFAVLHCLLHCASVFAQRRRLLQDITSPTFNI

EIFRQTPRCHDIIDRLFTSCHGINSLLLAVEAGTEKDHNPCFPIFMLFSSFTASATVAYL

SLKGLTPPNAVETAAGIVKDGLRFMQDGTDGWPLMASWLRHLAVMQRVIDNDAAIVARHG

STPHAHGVKDEISSNADTNPDGMDYDGHNSNGAGHGPMAHSVSESGRSESEPPIALPRRP

GFAAINGGSGPGSTAATVSPPGNSTASRPQDQKASSPGAPSNGAPPISTSQDMTGPELCQ

AFERQLLEMDDLAAFMGGGV

>SoG_05855.T1

MFSKAAAHLEHGSTATLIHITRHQSCRRRALSRLNFRQLATVANTKPRRGSLALSSEARY

NEIGVQQLSSHVFNQIFPDGAEAPPADLVELSKDHLKRHDLLGKTTDNSDPIAFDLPPLH

GRTLDEHFYKLGCDAAEPFLSHAKQFARVDAPPKPRKWIRRSGWTKYYPDGRTEEVDAPD

EKMICFDTEVMWKESSFAVMACASSPTAWYAWLSPWLLGQSENDQHLIPLGDPTKDRIIV

GHNVGFDRARILEEYNIKQSRNAFIDTMSLHVAVNGMCSQQRPTWMKYKKNRELRERVAT

ETVDHDLAELLRNKSMNDEEELWVERSSVNSLRDVAKFHLNVSIDKAVRDDFGELDRDGV

LAKLDELLDYCAADVSITHRVYRIVFPNFLDVCPHPVSFAALRHLSSVILPTNKSWDSYI

ANAEATYQKLLAAVHGRLVELTQKALAMKDNPHEYQNDPWMQQLDWSGQEIKMVKGKKKT

DPPRPAARQKKPGMPQWYKDLFPKNDAPINITVRTRIAPLLLKMSWDGNPLFWSDKYGWT

FRVLKTDSPAYIAKQMILCEFDDGDPKLRDDRLHAYFKLPHKDGPSARCANPMAKGYLTY

FENGTLSSEYAYAKEALEMNASCSYWISARDRIMSQMVIYEEDLTGAKPKRGRVKGNSNG

FILPQVIPMGTITRRAVENTWLTASNAKKNRVGSELKAMVKAPKGYCFVGADVDSEELWI

ASLIGDATFKLHGGNAVGFMTLEGTKAAGTDLHSRTASILGITRNDAKVFNYGRIYGAGL

RFAATLLRQFNPNLSEKQTMETASKLYANTKGTKTTRKVLHNRPFWRGGTESFVFNKLEE

FAEQERPRTPVLGAGITEALMGRFINKGGYLTSRINWAIQSSGVDYLHLLIVGMDFLIRR

FNIDARLAITVHDEIRYLVKDHDKYRAAMALQVANVWTRAMFAQQVGINDLPQSCAYFSA

IDVDHVLRKEVDMECVTPSHTTPIPPGESLDIIALLEKGKNAKLDPKIVPDARYAPNVDA

IEYQPRVPVMKSIEDAAGNDISFLRAQITGDDQELRDIIRGARKSENAGAVQPKKPRALS

KSSLKKVLPYRSEPALVPMDDLVSVTDAMRPQYRQQNQWQQWTRASGRPSRTTTRL

>SoG_05860.T1

MADIGSYSMVCNTENRLGRLGAQEIKNHAFFRGVDFDSLRRIRAPFEPRLTSNIDTTYFP

TDEIDQTDNATVLKAQAMQQGRSAQTEESLEMSLPFLGYTFKRFDNSYSSR

>SoG_05897.T1

MPSHRSIPTLTASLKSGAVKDREKAVDELSNVFNHRSRPAYIEDLDDKSYHKIFEALFQF

VIQERTGLYGSKKKDTASRNVAARLAKCAAALRSVVARGAAKLGRKTVLALIDHITQILP

GADGDYVPPLIQDYVKTLAEILSHPAHVELLARKGAEGWETCVDFTLTLVTTILPDQSLD

EPLARNSPVPGTRSTLRSSTSTQSQKRPGHLDGGPLRDGLQILHHLVTAVNAPLGRRSHD

LSELVLKVLRTHNFSMGSIQTLSFAIFNEIFSFTQTDDLTHALDMTHAIVPLMSYWWRAD

KVSQDELIRSLRNEISRTLSLVHHYLENVTAHSDAAFRRQVEELADVLWLEYSKRSEPFR

LQLSDVCFGPPSSFPVGSLSLHYFGLRPQNEASEGPWVILKNLAHIESLLAKPKSTDVDD

EERLDEQPRKRRRVNPRPSRLGSKLLSNDLNGRRTAIQLMPFILARKSLHDEVMEYFDRI

ANLAGDKDSVTASWALVVCGSCAATIEASHSRLEMWKQVWHIAARGISHPATSRAACFLM

AKMLETDILPYHEISEDLNTIVTSADVNGPASLSDSSIALMSSIFHIRNMKVPNASRSTC

SQIIRWIFLRWNPAEHSFAAFQSAHIQPLDVVNLLRSCCGSPPIQLAGHDRVIGGPLGET

EKQLSEVSNFTEYLLLLPREPVLESNDQGIMPSELRSLPVADTPTRNASKKLICELLRPK

VEELVELARSWTMKATEGGTLISLDRFSSLLSACLVLALLIPEIGDVPSSPSASIEASLA

EVLQAALTASMGSVEPGSFSSLILRFLRPMLPSLCISQLELVCEQQSTLLRTLSQIHEAL

EKNNERQQHSNDSREILDLDDEFESQGSRHSTNLVEIPPPRHNVSLRLSSLSFYTDTRMR

LSLLFLLQQDTSQSGLLPGAWVDQLLDLSDEELLLCQGLLVELCHSDLTINPLSVTKVIE

RLGHIVSQPRFQCSEVALTLCIDSLEGLHQVWLDDSDHLGDMVGDLYNHFVKTCLTSNIF

SLRAQMSMAQLLLTILKVDADYGRRLGLDSCRSTLLRILTEGTIALKFFIAERLPNVFQY

SILMLHDEIFVEVLDSLPTNSESIAGIALRMLVLHKLACSWPTLLRRCTYHIFEIPGKIP

HAVPYATRCLDDISRSLGLSSPSELFALFARQLLYTWLDEASISTIPYSIFGFDNLGDLL

RSVQADAVGLIVMRGQGLEHDVARLLGASEEDLLLNNFATALAYSMAYVAANSSQKCSGE

NEIKEKIGNTSFVKSVQINFIDIVALLLDSIDQEESVERAFSRHPDLAYAAEIMEKIKRI

AHSTVILPPNQQPMFRPKYVMHELFRLCQWTEFQFEDLWTPTTIVAIARQLINTVHPALG

PLHACSVLRKIRILISLAGSVALESYPLEMLLDSVRGFVVDTECADDALGISQYLLSEGA

HYLAERPSFVAGYGLSTLASLRVFLESSQASTTQESQFKATMTKAQDFHSWLSQYLSSYK

SEAFQNEDQRASFQAITRSASQIRGAGNAEKETPEARLLLSILADGSSKTRLLDDPSRQL

ALRLLCTDFSFPAQNRQDVIQTGHDAQEYATALWKSCAAQRLSDSFLAWAGSVIGKAFSS

NGEIPFDVLRESNLNQYQNHSSGARNSELGVLRLLQELSASSDSKTAGLAEAALRTAVSQ

ALSKDDSLLIDAVEQALSTPLYNASQWGVFHPIPSDARGTAPVRQDTAVWREHISSPSLI

KTIAAHMAHSVGDESIILKAIAPVIAQVPGFAQKAFPFIVHLVLLFQLDRRQNLKLSMSE

ALKEWLSAKQGPALRNVRLLLDTLLYLRAQPLPGEKSIADRFQWLEVDHGLAASAAARCG

LYKTALLFAELVNNDTSRSSRRTSAVNSSNLDETLLSIFENIDDPDAYYGLPEQASLSTV

LARVEHESEGPKSLAFRGAQYDSHLRLRKADSATDGQALAGALSSLGLSGLSYSLLQNQP

DSSAASSSVESTFRTARKLEIWNLPAPIATDNHSVVMYKVYQGLNQAPDINAVKAALHDG

LSQTMKGLVNHSPGAAVVRSRLSAIASLAELNDLLDASNAADSERILKAFEANSQWMKSG

SLYRYHQATQESLNLATTLTHLIKPCEELGLHVDAAVNIEAANSLWDFGEISTSIRLLQA

IDRGSSLQLQTLPVSRSDLLSKLGHRVSVARLEKPQEIQRKYLQEALKELKGNVHGKDAG

QVFHQFAVFCDEQLQDPAGLEDLTRLQNLRKGKSEEVAELKVLISNTRDTQSKHRYNHIL

SKEKQWLALDEKELQRVETARAEFSRLSVENYLLSLAASDDHNNDSLRFTALWLELSDAD

AINKAVSRSLFQVPTRKFAGLMNQLTSRLQNQDNSFQKLLMELVYNICVDHPYHGMYQIW

SGTKAKAVQQDEIASSRVRATEKVAQKLANHRTTADIWISIDKTSRYYHGLAMDRNLTKY

KSGAKLPIKDSQAGQHLVNGLAKYRIPPPTVDISLSATKDYSRVPTISKLDPTMTIASGV

SAPKIITAVGSDGVKYKQLVKGGHDDLRQDAIMEQVFAAVSSLLKGHRAAQQRNLGIRTY

KVLPLTASSGLIEFVPNTIPLHEFLMPAHERFNPKDYKGSQCRKEIFAVQNRSQETRVAT

FRKVTERFHPVMRYFFMEYFLDPDEWFTRRSAYTRSTAAISMLGHILGLGDRHGHNILLD

TKTGEAVHIDLGVAFEAGRILPVPELVPFRLTRDIVDGMGITKTEGVFRRCCEFTLDALR

EEQYSIMTILDVLRYDPLYSWSISPLRLSKLQKARNNEESNIDDAEPNETDLNRSKAAKR

ANEPSEADRALEIVRKKLSKTLSVTATVNDLINQAMDEQNLAVLYSALPSIPATSERHSV

SMPEASTSALSPRRRRRSETLSAAITQDPAKRQRHDQVSPVMATLPLLNGAPAGFQPQLG

AKRLVIQNLRKVTKTQEQLEQYYEKTLKEIGNALETLFESGKPTLPLERLYRGVEDICRA

GKANRLYALLKGSIDRHLQGAVSTRLNRKGRASNQDMLSAVLSEWAVLNKQTILLRSTFS

YFDRTYLLRELHTSVNDIVITHFRRTILPKGHFEDTPGGMLIYGLCDLIDLDRTGDSRKD

PELTKRAVALLYVMSVYTKLFEPIFLTRARRFSKAFAGDRSASSLKDYIKACESLIRSEK

VRFLQYNLESTTQKQMMESVHEILIHEYTDKLLNEGSLTELLREHDVESLKALYDLLRLA

GIENKLRVPWAAYVMSTAKEIISDKDRGDQMTVRLLTLRRSLDIIVRDSFQKSSELREGM

RGAFGSAMNDKAAASCWSTGTSKVAEMIAKHFDLLLRGGLKAIPAELLSDIQDRAAAEKE

GMASSADEDAELDRQLNQTLELCRHVHGKDIFEAFYKKDLARRLLMGRSASSDAERNMIT

KLRDEAGSSLTQNIETMFKDQEIAREEMNAFRDWDRNTSENPSDILNVMVLSSGAWPTYP

DVNLNLPENVARQHERFDQFYQHKHSGRILTWKHCLGHVSLTAQFPRGRKELSVSAFQAS

VLLLFNSVPDDGFLSYEEIKTGTNLQDADLERTLQSLACGKARVLTKHPKGKDVKPTDTF

TYNKSFTDPKMRVKINQIQLKETKEENKATHDRVAQDRRFETQAAIVRIMKSRKQMGHAE

LVAEVIAMTSKRGSVEPAEIKKEIENLIEKDYIEREENTYVYQA

>SoG_05901.T1

MASILGKRVRSISTSSTITIELPAKRFRHDTTKPVLHDEENQDPSDTIHSDEVDDLPELP

SRTPQVKANPVTPSTPRHRDALSGYPTTPRHAVMSAGKLFKRLTPQSPLSPSAVQTVYQQ

ARQLFARGSEPGQLVGRDVERATLTRFLDKSITKTSNGCLYVSGPPGTGKSAMITEMVRQ

RAEDADVRGMYVNCMSVKSSKDLYNTLLKSLDYDSELSEADAIAALQDFFCPKNDDPTVY

LVTLDEIDHILTMGLESLYRLFEWSLQKSSRLVLVGIANALDLTDRFLPRLKSKNLKPEL

LSFLPYTAAQVKGIITTKLKSLMPEGKENFVPFIHPAAIELCSRKVSSQTGDLRKAFEIC

RRALDLIEAETRAKHEEEARERMLQATPSKKPLGENINGASAGSTRSVLQIMAGSLKALT

PETAPRASIGHLNKVTAAALSNGTSQRLKALNLQQRAALCSLVAYENRTRAAMKAGTLAG

TPSKSQTLAPTIKVLFETYCRLCVRDSVLHPLSSSEFREVVGSLETLGLVSAVDGKNGSF

VGPQTPSKRGRKPVAASGDDRRIASCAGDKEMESVADGVGAGILRSILSGDALD

>SoG_05909.T1

MTPTPPSTDNSTGARSPEEQFRVNKKIQSKPAPLSRDLGLTGNRKCDRSQPCSNCTKREG

MDTQSCSYATPVSRKKNQSQGDSSPDDMQNRIDRLEGLVLSLMHGGANLPPQPGGNGASG

SQSVADSGGSSAAKTSNEQGLEMVDDDDEGDSDIDQDLSKSLGVLKVDTDKGKSMYFGQE

SWHTILADISEVKQYFTTHKKELESSYERVKQSKPHAAQEGITLLLGAVPASEIELRAEL

PPKTTVLTLCSRYFNSMDNAVSIIHGPTFQQQLKTHWQDPSKTPIMWLGLLYSILCLAML

SYHKVGDEPPEWKGRSLEMASEFRLRTVQCLIKADYTKPTDYTIETMILYVFGENSTRWD

ADVGLWMVVSLIIRIAFRMGYHRDAKWFPSITPFQAEMRRRIWALIRMADIMFSHQVSLP

NMIYDHDCDTQLPTNIFDDEFHPNIKELPPGRPSTEPTPIAYLIAKARLCNEAGNVLQAT

HSVRKHVTYDEIIRFDAKLRQIKKDLAPHLKLTPLEGSHDPVTLIIARFNLETLYLKILC

LLHRRYLPRARQNPRYAHSRRAAIEASMQALDHLATLHRESQGYGRLRSVGWYIKSTATK

DFTLPAMLIILDLHYDNLARQQSTPDSNSCLYTDEERNDMIKKLEHTRSIWVTLADSSME

AFKAYKVIDIMLEKIKDPAQSLEPMNVATTPLPDLAAGSIDPAMSMPPSMMSPGMLPDFT

GGMDPFAAVNPSSFMGMDFGGMGPSTNSTFSSEGYPQPGAPSPMTMFNIGGMNGQAPDLG

TNFDWNMFENSVQMASWGPDQSFQFYGATGDESSPEKTGSSEHKSSMGGSGLM

>SoG_05913.T1

MLFSKATALPLLAASMPLAEAKLNYWAKKAGLLYFGAATDSPGQRERAGLESSYPQYDAI

FADDDIFGQTTPTNGQKWLFTEPEQGVFNFTEGEIVSQLAEKQDKLLRCHALVWHSQLAP

WVETVKWTPETLTAAIKLHIQKVAGHWKGRCYAWDVVNEALNEDGTWRESVFYKVLGEDY

IKIAFAEAARVDPDAKLYYNDYNLERPSAKSRGAVDIVRMLKEDGIKIDGVGMQAHTIAG

RSPSIDDYIAVINMYAETGVEVALTELDIRIQLPLNETNLEWQKADYRAAVGGCAQSPAC

VGITLWDFYDPFSWVPYVFNGEGAALLWFDDFTVHPAYYGALAALKNATGACDKSCPEER

SYFERRL

>SoG_05917.T1

MAVGTVLITGGTGYIGSFTSLALLEHGYDVVIVDSLYNSSKVALDRIETLCGRRPHFYQV

DITDEKAIDEVLEKHPAIDSVIHFAALKAVGESGKKPLEYYRVNVGGSIALLRAMERHNV

TNIVFSSSATVYGDATRFDNMIPIPEHCPIGPTNTYGRTKSTIEDLITDHINAQRNNLKE

AGKPHEQWNGALLRYFNPCGAHPSGIMGEDPQGIPFNLLPLLGQVATGQREKLMVFGDDY

ASRDGTAIRDYIHVLDLAKGHLVALNYLRENKPGVKAWNLGSGRGSTVFEMINAFSKVVG

RDLPYEVVPRRQGDVLDLTANPALANKELQWKTELTMEKACEDLWRWVSNNPQGYRQEPP

AELLAAAKSSAASK

>SoG_05919.T1

MKKTKSKSESSSSGHIADARFASFETDPRFRLPSKKQGKTTIDKRFSHMLKDEGFVATAR

VDRYGRKLKSDTKKKALQRLYDEDDEEEKEAEDDDDDNISVDDDEIVQRELRAAEEKYDP

ARGGGFESSSEDESDSDDEQEPAHEQQREGDLQRFQDEQGELEAGEVTNRIAVVNLDWDH

VRAADLYALFSSFVGDAGGKVERVSVYPSEFGKERMQQEELEGPPKELFKGKKQEESEED

SDEDSDSDEAIRKQLMQEGDDQDFDSDALRSYQLDRLRYYYAVMVCSDKKTAQKIYEATD

GTEYQASSNFLDLRFVPDDVTFDDEPRDECDKPPEEYQPVEFVTNALQSSKVKLTWDMHP

EEHSRKESIKRAFSGSRNDINENDLKTYLASDSEDESDDAEDGDGVETVVAEGAEPEPKL

SKKELARKKMREALGLTDEASSKSSKADPVGDMQITFTPALSASAAKKDPEAEETTIEKY

KRKERERKERKRQQAKAKRGGAESDESEGEQHVVSGGQDEEDLGFDDPFFTNSEPQMPSK

ASVRKEERLKKRAAREAAEAESSAQKAHLKKIMSADRNDKQAEHLDHFDMNEILRAEKQK

RKKGKGKGKKQGGEGGEGAAGLQEGFQMNVEDDRFKAVFENHEFAIDPSNPKFKGTEGMN

KLLEEGRKKKRGGGGEEDDRGQRKKSKRR

>SoG_05959.T1

MANVPRTSRACDGCRFRKVKCNGANPCSQCAHLGLTCTTSSVPRKRTPGARGRLVEQLRQ

GCTGAAPFPQQSPSANPPQHHHVLLPAPDEVSPNDSSVNSENVTSPKTPVPGRSPFSPEF

FTSLLPDFDEIVYPVNPIVTNQELRAAIDNMHNSFEDAALVYAFGAVTINLAQTSNDVHK

TLPDLMDMSIQAHARAGVGSLHGGGIFGDLPVSVNRVATCIYLEICMMAYKRYDRSFAFL

REAIAMVQIMQTRANSFSRFSPDPRENARRSRMYWEIFIHERVLTISAGYPSVLQPLPEG

LPTLDDSIPVHVAVGFNRIISMFRIMDHRFLDLWISQTSSLPYIAPVTPEWIEAKQSELD

KDQQDTAEAQSKMALDGHRPLNELQLADLFITRLWMRTLIWQIALSCGHLRSDPTQTTHE

SLSLLFPAHRLSSELRRLVTRMDSVMSIGTHGSGMLEKLFEITSTIADVLALPMAQSTTE

RESRSRMDDFEFLVQFLLGFERVNGQQKVYIQEKLEALQQQYQYIGV

>SoG_05970.T1

MDFLKSAVASAIAKGPPFPYNFGDTVDIDESVFTLYNGTRREDGSPCSIFSFDITGANRS

RLPLAKNALKKFRTLRHPGVIKVFDTVETETYIYIATERITPLRWHVKRKSLSPETIKWG

LHSVARTIKFINTEATSIHGALKVGSLYTSESGEWRVGGFEVLSSIKDDESIIYNYGSLV

PDSGRYAPPELAQGGWDVIKKNPHAATDAFGFGTVIFEVFNGDYNGRDQAGLTKNIPPTM

QSSYRRLCNANPKARISVGNFLDQGSRPGAFFDSPLIKMTEGIDNLGVKSEAEREQFLND

LDDLSDDFPEEFFKLKVMPELMKSVEFGGGGPKALGVVLKIASKLPSEDFESKVTPFVVR

SFANPDRAIRVSLLDSLPLMIDRLPQKIVNDKIFPQMTTGFTDMAPVVREQTLKSVLTII

TKLSDRTINGELLKHLAKTANDEQPGIRTNTTICLGKIAKNLGSSTRPKVLIAAFTRSLR

DPFVHARNAALMSLGVTADYFSDEDAACRILPVVTPLLIDKEKMIRDQANRTMDIFMQKI

RKAAASMPDTVLPPPQAADAQPRMGTPQATESTGWTGWAISSFTNKLSAAAGDMQTNGSG

AVSPNAAPSPRSASGRPATASASNLHRQAMKSPTPDTSRATSPNPNTVANAFLAPEMAED

DDTGDSWGDMGDMDDDGFVDAPSSSTTTTKKNQSASATPFDEGEPDFEGWLAAQAQKKSG

SSKPLPKGLAKSGPAAKKPLAKPAPSTKPVVAKKIDLKPKAAEDDDDNWGDGW

>SoG_05987.T1

MKSDVFSLLGLAALALAAPSPVLEDRGTKVDVQLSNGDTIVGSSLLNVESFRGIPFADAP

TGQLRLRPPQRLSRSLGTFDATPIAPACPQMLISRETLGIVGGVLNDLLNLPILQSVAGR

EDCLSITVQRPAGVKKGDNLPVLFWIFGGGFELGATSMYDGGSLLKEASKQGQNFIFVAV

NYRVAGFGFLAGKEILADGASNLGLLDQRMGLQWVADNIAEFGGDPDKVTIWGESAGSIS

VFDQMALYGGDATYKGKQLFRGAIMNSGSIVPADPVDCPKGQAVYDTVVRKAGCSGAADT

LNCLREVDYKTFLDAVNSLPGILGYQSLALAYLPRPDGVVLPDSPDKLVGAGRYHAVPMI

IGDQEDEGTLFALFQPNLTTTADTVDYFSKYLFHNANTEQLNTLITSYLPPIAGGSPFRT

GFFNELYPNFKRLAALFGDLTFTLTRRLFLDIATAVNPDVPSWSYLSSYNYGTPVMGTFH

GSDLLQTFFGLLPNNAARSTRTYYFNFLYNLDPNKGVQKYAHWPQWKENRDLLWFEKPTS

NSILRDDFRQSNYEIIKQLADYFKI

>SoG_05989.T1

MRSNLLSLFGLAAFTLASSSPDLGGPDLEGRRAKVAVQLTSTGDTIIGSTFLGIESFRGI

PFADAPTGQLRLRPPQKLSRKLGSFDATPVAPACPQMWISSNSKTLLGDVLNDLLELPLF

QTISGREDCLSVSVQRPAGVQKGSKLPVLFWIFGGGFQLGSTSTYDARSLLKRASDQDQD

FIFVAVNYRVGGFGFMPGKEVLQDGASNLGLLDQRMGLQWVADNIADFGGDPDKVTIWGE

SAGAISVFDQMALYGGDANYEGKPLFRGAIMNSGSIVPADPVNCPKGQAVYDSVVREAGC

SGASNTLDCLRQVDYQKFMQAANSVPSILSYQSIALSYLPRPDGAVLPDSPEKLAAQGRY

HAVPMIIGNQEDEGTLFALFQRNITTTEKLVDYLSQLFFHNATKDQLYQLVNTYDPKPRA

GSPFRTGMHNQIWPAYPGFKRLAAVLGDLSFTLARRVFLDMTTRVNPDVPSWSYLASYRY

LTPIMGTFHASDLIQTFYGQLRNHAEHSTRTYFFNFLHNLDPNKGNQRFANWPQWKEAND

LMWFRRRTGNDMLKDDFRQSSYKAMLELLDVLRI

>SoG_06007.T1

MADDSALAAAAAIVASLAADPKKSLSAPASKIQLPGEDTPGKLSLEIELQKLALRVSQLE

TRASPAVTFPETPSETNDSLFGTDAGLSPSGSRPSSSKTRMFPGGHPGSLDNPNLVPREL

TKEALEGLREHVDDQSKLLDNQRQELAGVNAQLLEQKQLQERALEVLEHERIATLERELW

KNQKANEAFQKALREIGEIVTAVARGDLTMKVRMNTVEMDPEITNFKRTMNVMMDQLQVF

ASEVSRVAREVGTEGLLGGQARIGGVDGTWKELTDNVNVMAQNLTDQVREIASVTTAVAH

GDLTKKIERPAKGEILQLQQTINTMVDQLRTFASEVTRVARDVGTEGILGGQADVGGVQG

MWNDLTVNVNAMANNLTTQVRDIIAVTTAVAKGDLTQKVQANCRGEIFELKSTINSMVDQ

LQQFAREVTKIAREVGTEGRLGGQATVHDVEGTWRDLTENVNGMAMNLTTQVREIAKVTT

AVANGDLTKKISVEVRGEIAELKNTINQMVDRLGTFAFEVSKVAREVGTDGTLGGQAKVE

NVEGKWKDLTENVNTMASNLTAQASIKLPLIFAPLLTMIQVRSISAVTQAIANGDMSQTI

DVQASGEIQILKETINNMVGRLSSFCYEVQRVAKDVGVDGKMGAQADIAGLDGRWKEITT

DVNTMAMNLTTQVRAFSDITNLATDGDFTKLVDVEASGEMDELKRKINQMISNLRDSIQR

NTQAREAAEQANKTKSEFLANMSHEIRTPMNGIIGMTQLTLDTDLTQYQREMLNIVNDLA

NSLLTIIDDILDLSKIEARRMVIEEIPYTLRGTVFNALKTLAVKANEKYLDLNYKVDSSV

PDYVIGDSFRLRQIILNLVGNAIKFTEHGSVSLTIRESTDKSQQQEIQPGEYAVEFIVQD

TGIGIAQDKIDLIFDTFQQADGSMTRKFGGTGLGLSISKRLVNLMGGDLWVNSTLTDGSE

FHFTCRVRLADNNLDTMEKALKPYRGHQVLFVDKAQSSNSAEIKEMLSALGLVPVVVESE

KSSALTRLKAGGALPYDAILVDSIDTARRLRAVDDLKYLPIVLLAPVVHVNLKSCLDLGI

TSYMTTPCKLIDLSNGIIPALENRATPSLADNTKSFEILLAEDNTVNQRLAVKILEKYHH

VVTVVGNGWEAVESVKEKKFDVILMDVQMPIMGGFEATGKIREYERSMGTHRTPIIALTA

HAMMGDREKCIEAQMDEYLSKPLQQNHLIQTILKCATLGGPLLEKNRERGLKIGADAKAG

HRGSAASSSTSRNSSRHDLLRPQLEARSFTTREPMTSNGMESPALLSADEEDPMARARHE

LSDMRSLTS

>SoG_06008.T1

MEGEEPTQDVLDPRRIGQQNSGFSDEDISDIICILYAHSESARETVLRLAQEDSPHVIGR

TDADGVDPNYDLEDHASRFSLTEAEGNYALILRLSSSVKDPSSGFVFGRNALRCDVVFHN

DPLKRLSNIHFRIYVNKHGSVMIEDQSTNGTVVDENVLTSHPKGPNQEPITRWMLNSGSV

IKLHLHHQIKDLNFRVRIPRRDAEYDVAYMNKVHDYFHRHGLDPEGSPEPAPAVAVPRHL

RNETAMAQAPHTSPVKRKDTAVMRREWTGSGKYNRTGTIGKGAFAVVYKVTSKYDGSPYA

AKELDKQRFIRNGVLDQKAENEMKIMQCIDHSNIVRYIETIDWDDRLLIIIMEYVAGGDL

GRHIVERGPMHEEDVKVMAGQLVDALSYLHVRNITHRDVKPDNILISSLNPLVVKLTDFG

LSKMVDSEQTFLQTFCGTLLYCAPEVYNEYAEYDANGVRNRGKRVRRPLGQRYNHAVDIW

SLGGVLFYCLTGSPPYPVKNGISPNELLHMVMTTQLNTHPLSAAGVSNEGIHFLSRMLDR

SPEKRATTRELVDHAWLNGPISQRVQASQSFDELTDDEGGIESLDNDLEMDMETFRQPTA

EMYEEDRISDSILGYDSEKENGHSEQQHHVPRLFGEVGSSAIGSSGVITSEFLNLHRNGD

SMHETEIMDSYADEAYDSESNPTPRHKSRQIFHPTSPSLAQQQSADQLDSLVHEVASQSL

GGKDHPPANTECRSLSFKRKPPSTETSGEHDDNTPPEKPTMKRLKSDTALESMSDEVLDE

LRLIASVPPIKRLNSGRQIDAPVNKVVFWTQDRKTWHLEYPEMTQLQKDAFVQAAKLRGE

DFQPGGSPLWELAMKHFAPANLNLAGQTRSVPNMRRNEQMLEDDLLEFPPTAAAVDTNVA

TSISQNSLPPTQDSDSQIIVPIHTAASTQQAIALLESTPDSIIQGISLPVTASHLSFGRG

PENTSVFKPPSESRVPKYAFKILLWREGYDPSKDPAKVVPPWLQDQDAAGGNETYYFWIC

TKATVGIQVNGYQLASSDHKNHNGPSQFWARLHDQDELVIWGGSGTENRTKLVFRCFWGG

SSRPRAPEGRGFETAPPHIVQKLNDACQLTERRMRETRDKESLKNEATKDHSWRLRFVDT

ERKKSLAFEEKRKQAIRLLRQVQVARRGSPASVPPWVI

>SoG_06036.T1

MDLDRDGSQTVHDQASAQDSGHEHSEWSLKPKLISFKERDDASGFPERALGVTWQCLTVQ

VVAADAAIHENVLSQFNLPKIVKESRHKPPLKTILDNSHGCVKPGEMLLVLGRPGSGCTT

LLNMIANKRRGYANVSGEVHYGSMSAEEARSHRGRIVMNTEDELFFPSLTVGQTMDFATR

LKVPFRLPQGATSTEEIRAQTKEFLLQSMGIEHTHDTKVGNAFVRGVSGGERKRVSIIEA

LTTRASVFCWDNSTRGLDASTALEYIKAIRALTDVLQLTSIVTLYQAGNGIYTLFDKVLV

LDEGKQTYYGPVSEARPFMENMGFVCNHGANVADYLTGVTVPTERQIHPEHQNRFPRNAD

ELRAEYEKSPIYERMVCEYDFPSKESVSRQTQLFKDAVRLEKDKRLPDSSPMTVGFMTQV

KACVARQYQIVLGDKATFLIKQVSTVVQALIAGSLFYDAPNTSGGLFTKAGACFFAVLFN

ALLAMSEVTDSFTGRPVLLKHKSFALFHPAAFCIAQIAADIPVILFQISIFSVILYFMVG

LTSSAAAFFTFWVVLVAITMCVTALFRAVGAGFSTFDAASKVSGFLVAATVTYSGFMIQK

PQMHDWFVWMFWINPIAYTFDALLSNEFHGKIIPCVGPNLVPSGPEYAMGEHQACAGVGG

AKPGVNFVTGDDYLASLNYSHSHIWRNFGIIWAFWVLFVVLTVISTTKWHSSSEDGPSLV

IPRENTHAHKALMQRDEEGQINEKKSAGNNSGGVTSADVSDSSDDNAILARNTSVFTWKN

LSYTVKTPSGDRTLLDNVQGWVKPGMLGALMGASGAGKTTLLDVLAQRKTDGTIHGSIMV

DGRPLPVSFQRSTGYCEQLDVHEAYATVREALEFSALLRQSRDTPREEKIKYVDVIIDLL

ELHDIADTLIGEVGNGLSVEQRKRVTIGVELVAKPSILIFLDEPTSGLDGQSAFNTVRFL

RKLADVGQAVLVTIHQPSAQLFAQFDTLLLLAKGGKTVYFGDIGEHGKIVIDYFARYGVP

CPEDANPAEHMIDVVSGHLSQGKNWNEIWLSSPEHKAVSEELEQIITKAASKPSGAVDDG

HEFASSFWDQTKIVTQRMNKSLYRNTDYVNNKIILHITSALFNGFTFWKIGPGVGELQLK

LFTIFQFIFVAPGVMAQLQPLFIHRRDIFETREKKSKMYGWVPFVTGLIVSEIPYLMICG

VLYFVCWYYTAGLPSETSRAGANLFVVLMYEFVYTGIGQFIAAYAPNEVFASLANPLLLG

TLISFCGVLVPYSQIQEFWRYWIYWLNPFNYLMGSMLVFSLWGAEVKCSDAEFARFSPPN

GTTCREYLSYYMSEGLGVAANLINPDSTDECKVCSYTKGQDYLRTLNLNEYYYGWRDAGI

VALFAVSSYALVYLLMKLRTKASKKAE

>SoG_06037.T1

MPMLSSAGKRWIASRTGEDVSFHKFESAPNRHTVPLHLPIPRHHQEYQDELYQLPDRSLV

EDLFDMFIHSAFSMVFPVMDKTLFPDILNKAYQSMEPISTADASAKACILAFTTIICLVK

EEDFGFPRQDVENHALKVRYLLSSSSEEASLTTLQATFLLDHEWRQLRNLFWLSYIFDKD

LSLRAGTPPLMSDDYCDLTLPDGYVECYAYLPHLDESVRLRDSETLSPHFVEDPRLSILK

EKVGRRLYSAQTLKKTNAELLRDIRELDEELETWRVSIPALFRPALSISHGTQVDEESMT

LPRSMRHITLHLGYHHLMSAIHRASARCMSGSEASDAEEWLPGVQSSIALALEASRSTLI

YLRAAIRGVADKAFWIMVYYPTTAMMTLFLNILSDPLNPRVEEDVRLLASTADLIHGLAI

QSMTSPVLAHIKLMTDFIAELTYLSHKAIAKARAESEQRQNLKA

>SoG_06053.T1

MSETYSTAEVAKHKSDADGFWIIVDDGVYDVTKFADEHPGGAKILKRYAGKNASKPFWKY

HSEHVLTKYGKNLKIGTVKEAAKL

>SoG_06075.T1

MTRTIPLVGVALLSLVSAQTPDDVPEVHPKISTWQCTKAGGCVEQTNKIVLDSLYHPIYQ

EDAPEYNCGDWGQTPNATACPDIETCQANCRLRGISDYSEFGIQTDGDALLLEQLAEDGT

LLTPRVYLLKEDAQEYEMLKLTGGEFSFDVDVSKLPCGMNGALYLSEMAADGGKSDLNKA

GAYYGTGYCDAQCYTTPFINGEPNLEGYGSCCNEMDIWEANARSNTLVPHPCNVTGLVEC

EGEACEWSGICDKWGCGYNPYQLGNPDYYGQGSSFTVDTSRPFTVVTQFPADDNGKLVEY

RRFYVQDGRKIAQATVKLPQHEDDLNGTNWIDDNFCQIRNSERYNDLGAATAMGDAMTRG

MVLALSVWWDAGGNMTWLDGKTDGNGPCLDGEGNPKNIEAVQSVPAVRFSKIKWGEIDST

YESKCKSRKR

>SoG_06091.T1

MWAAGNARIVRYLIFAVFALCVFYFVSSSRYEGVAIPATASKGGNSPWPPAEAADVAPKS

DSSTEDPQDVLQPNKPPTPPEKQGGKGAGVENDPNAPKKPSVVHIDNKADSGTEIKAPPP

ASAANGGSKVPAAGDASQFPLGMMPGEPGWMTDMNAEAPGPRMNATFVTLARNRDVWEIA

SSIRQVEDRFNRRFHYDWVFLNDEPFDDTFKKVTTALVSGKTHYGLIPKEHWSYPSWIDQ

DKARKVREDMKARKIIYGDSESYRHMCRFESGFFFRQELMMNYEYYWRVEPSIKLFCDIH

YDPFRVMAEKKKKYSFTLSLYEYRETIPTLWESTKNFMKNHPEHLAKNNMMGFLSDDGGE

SYNLCHFWSNFEIGSLNWLRSKPYLDYFNALDIDGGFFYERWGDAPVHSIAASLLLNKEE

IHFFNDIAYFHNPFTHCPTGLQTRLDRKCHCDPKENFDWKGYSCTSRYFEQNGMTKPEGY

ELEK

>SoG_06092.T1

MAGILFNRFLAPATRQSFTPIFRPTTTTIRSPILQRLANTNTPAASSRRAFTTTPPLSTT

LNQVLRGQRKGKRARHAVSPALSSKHCPQLKGVCLRVGVVRPKKPNSGERKTARVKLSSG

AVITAYIPGEGHNIQQHSVVLVRGGRAQDCPGVRIFNQGGVASRTTSRSKFGTKKPKAAQ

VG

>SoG_06105.T1

MLSRPLIDSRGSGNPKLLLAIAVFLLPWLRLVDAESTANGVKRAALSEGKGADDWAATSL

EAIQESRQSHGSHHQGQQPPDHQSSHIKRLQHAKVQNEARALEKEADENRKPKSRKHLKY

KNTDDASALATYAPAAAVRAPHSSKHKRSSNPRVSGVASPQIARSLGDWEVEDFVLLATV

DGNLFASDRNTGKEIWRLQVDQAMVETKHYRANVSALDDDYDAVDHYIWAVEPNSDGSLY

VWIPDSGMGLVRTGFTMKHLVEELSPYAGEQPPVVYTGDKKTTMITLDAATGQVLKWFGS

GGSHSDEAESCLRPNGLHGMSPEECSSTGTITLGRIEYTVAIHRRDHRPIATLRYSEWVP

NNFDNDLFQQYHESRDGRYISSKHDGKVYAFDFGRSKQGAKLYSEKFSSPVARVFDVCRP

WDAPLESNPQLVILPQPPMPSQDQDEEMLRARDNRIFINQTENGGWFALSGRSYPLVVDA

PIAQIAKVDWAELGPIWDTISEAQVSRVLVGTHYLDHLHTPSPGQPPTLPPGMVYDSTED

TERDNESLLPVDALAEDLDATLIKKFKSLPRSAADSVVEFISNPILIILFFGALIYNEKK

LRQTYGHFRRRRSWREIVPYLINGHEPHGKGEASELDHAVEPIAATKETIQSDDHAPPRS

QSGDSIDQKDDEVKDEPIAPASENKNDDAEGSVGNANDEGSDDKTLAANAGPEKKKKAHR

GRRGGVKHKKGKARENSLSRNDEPNSATVEEAVNNAKRLGDRPAFEPDVMTVANDMQAVT

GPIIRMGNIEVNTEEQLGTGSNGTLVFAGKFDGREVAVKRMLIQFYDIASQETRLLRESD

DHPNVIRYYSQQIRDGFLYIALERCAASLADVVERPSHFRALANAGRMDLPGVLYQITNG

ISHLHQLRIVHRDLKPQNILVNMGKDGRPRLLVSDFGLCKKLEGGQSSFGATTGRAAGTS

GWRAPELLLDDDARDAGVMEFSTHSGSGSVLVNDNNLGPNSRRATRAIDIFSLGLVFFYV

LTNGSHPFDCGDKYMREVNIRKGNHNLKALDSLGDFAYEAKDLIASMLDADPKVRPSARD

VMAHPFFWSAKKRLSFLCDVSDHFEKEPRDPPSDALVELERHSSDVTKGDFLRVLSREFV

DSLGKQRKYTGSRLLDLLRALRNKKNHYEDMSDSLKRSVGPLPEGYLSYWTTRFPMLLLV

CWNVVYNVRWEETDRFREYYEPAGL

>SoG_06106.T1

MDGDGEASSTARQRPENQRGRGGRSRGRGGQNPNRGVNGRRRGGPRTAHDSNDRDNSEES

QLGEPGTRDAAAAAAALKSRMLAAAAAAKDDDAQTDDGSVCFICANPVAYQSIAPCNHST

CHICGLRMRALYKTKDCAHCRTPAPYVIFTDDMNKRFEDYSDKDITTTDSNVGIKYTNED

IVGDTVLLLRYNCPEPSCDIAGLGWPDLHRHVRTTHRKRMCDLCTRNKKVFTHEHELFSD

KELEKHMRRGDDKPGAADQTGFKGHPLCGFCGERFYDDDKLYEHCRMKHERCFICDRRDS

RTPHYYLDYNALEDHFQKDHFLCGDRECMEKKFVVFESEMDLQAHKISEHSGRTAGRDAR

LVDISGFDIRQPYQQERRGGRQGDGQSRRGRGRGRDQNADTPIATPNQPLRRDEIAFQRQ

MAIHSAQSVTPRTFGGQLSSTQPASAAPRPNNGQPPSRGNRMSTLEQDASALTLTDTANL

SPEERARLVRHSAVVERAGNLLGNDARKMTQFREHISSYRQGKVTGPQLIDAFFTLFADT

SSNALGTLVREVADLFEDKSKADALRKAWQDWRAINEDYPSLPGLSGMHGATTSSSGWAT

AATANPAIPNAAPSQRHSNRVLRLKNSTRLAGPAPVTNSKPSGWVASSGAQAPSSSAFPS

LSSAASRPSSNATPSWTGSAAQNQAASASRSRGSRGQGGEDAFPALPAAPKPTTTIFGYG

NGRGVIRDYGARETGFQWGGASASSSAPEADASETEAANGGKKGGKKNKKQVLVQWG

>SoG_06115.T1

MEQQQQQQQPQQAQQPTQAPQTQQQQQPSQAPHAGGVQGPAGRRLHIAHRRSPSELTPLM

SMFQNPQMEQLAIQQQIELLQQQQQQIQATQQQYMNMGIMPPQQGVGAGFNPMQPMGNMQ

TAFQFPNQMPQQQQMAPPTQPLSHRRNQSALPNMGMGPPPAPSSGASGSQFGNFEAPPPT

NRENTSGRGGRGGGAAGGGHQRRHSLALADAKKAAELAQQKRTTTGFQFPASPANPEDEN

KTAPSTTDATQAATPTRSRGGHGRSQSMAVNGRGGSSARGGLDGSNDFQRRGSGAGHART

GSRNFEGNWRNQNQAQAQDQTANAAPNQGFQPGHRPRGSMNQSVSSIGAFQYNPNQPQVM

QIPNQMMMPQLYGGQLNPMQISQLQALQAAQMGGQQFQGLQGSQHAGALGGQPQQQQRKT

LFTPYLPQATLPALLGDGQLVSGILRVNKKNRSDAYVTTQDGLLDADIFICGSKDRNRAL

EGDLVAVELLDVDEVWGQKREKEEKKKRKDITDTRSGSTNQGNQSSGNADDNNNGEGGIR

RRGSLRQRPTQKKNDDVEVEGQSLLLVEEEEINDEQKPLYAGHVVAVVERVAGQMFSGTL

GLLRPSSQATKEKQEAERAARDGGNSRHHDSRQQEKPKIVWFKPTDKRVPLIAIPTEQAP

RDFVEKHQDYADRIFVACIKRWPITSLHPFGTLVEQLGRMGDLKVETDALLRDNNFSSDE

FSDAVLRSVGLQDWSLAKEDEAALSARRDFREENVFTIDYNGATELGNAIHVKSRPDGKI

DIAVHVPDVAHFVKANSLVDREAKKRGTSVQLLNRFCALLPPKLAGEVCSLMPNEERLTV

SVVFSVNPHNGAVAEGDSWVGRSIIKSSAKVSLKEIDAALADASSYKNDAVPVKTLQILN

AVAQKFKESRLGAGGELIAPLRLIQQLDDENNPVQENLFDSSPALELVEELMHKTNAYVA

QRLAESLPEKAILRRQAPPNPRRLHTFVERMNALGYDIDASSSGALHNSLFKVDDADLRK

GMETLLVKSMQRAKYFIAGKTAKPLWPNYALNLPLYTHFTSPTRRYPDMIAHRQLEAVLS

EGKIDFSDDLENLVKTVESCNTKKDSAQNAQDQSIHIESCRAMDKKRQETNGDLIAEGIV

LCVYESAFDVLIPEWGFEKRVHCDQLPLKKAEFRKEKRVLELYWEKGVPSSAYVPEDERP

KAAASQRMNNAMAAARQKEEAERVKKERDEAARKQTETGTMSTDDVDALFDDDDDNTSDV

TEAMAGASLAERPTQSVPGSPARTGSNTGDLHRARSDSKVHAAEVPEARLSNKEKYLSFF

KLREEGGDYIQDVTEMTRVPVILKTDLSKSPPCLTIRSLNPYAL

>SoG_06128.T1

MGKTDSSSTNGALHTHVSTASDSAPAAAPDEPSIAEPPDSQFQVVILEKPLSAHTLPAER

VADLLESNLQDGLSNEDAAARLARDGPNTIKGAKGPSLWEIFLQQVANALTVVLIAVTAL

SFAIQDYIEGGVVAAVIVLNIVVGLVQDYRAEQTIQSLYALSTPKCKVIRDGVSETVKAE

TLVKGDLVSLATGDMVPADLRLVSGINISTDEALLTGESVPISKKPDAVFTDEDMPIGDR

HNIAYSGSSVTRGRATGIVVATGMSTEVGQIAELLRKTKKDQPGKTPLGKFLWKAFFTVR

GILGLEGTPLQVTLSKFALLLFAFAILLAVIVFSSAKWNVADEVLLYGICVGVAVIPESL

LAVLTVTMAVATKAMVKGHVIVRQMPSLEAVGGVTNICSDKTGTLTQGRMITRKVWLRGN

LTGSVEGAGDPYDPTSGTVTWSGSLAGSCLRSFLDTLFLCNNSTVTDGKKEPETDSSSVT

TVSSPAEWKAVGEPTEIALRVFALRFGHTRNGHDELIAEHPFDSSCKLMSVVYGNRMEDE

RHVYTKGAVEVLLERLDESQDLKDTIHTKAEELAAQGLRVLCIADKVTSEGAEDVHDRAK

IESKLKFLGLAGLYDPPRVETAGAVAMCRGAGVAVHMVTGDHIKTATAIAYEVGILPRDA

PLRPGTVMAASDFGNMTDEQIDQMEALPLVLARCSPLTKVRMIQALHRRKAFCIMTGDGV

NDSPALKQADVGIAMGDRGSDVAKEAADMVLTDDNFASIVTGIKEGRRLADNIQKFLLHL

LTSNLAQVVLLLIALAFKDTSNHAVFPLSPLEILWANLVTSSPLALGLGLEEASLDILQR

PPRSLKKGVFTKDLVRDQLVYGFSMGSLCLAAFMIVAFGPSGYHELPSGCNEDYASGCDP

VFEARATTFATLSFLLLITSWEVKHFHRSLFAMDERWAGPFSVFRTIYHNKFLFWSVIAG

FVLVFPLIYIPQLNVLVFKHKAITWQWGVVFGCVASYIGLLEVWKAIKRKLGLGIERFAS

GTGAV

>SoG_06137.T1

MSAAVKVKFVASDPNRGGLPVKRKQVQHACDACRKKKVSDLPVPGALAHPMHAITGIFMR

SPAQEATPSVVNGASSTPAMNYGANPGQRRASAYPQQRTPGDASLSSPSESSKQQTRFVG

DLNPEAMFMEAMGPPSKNENSQKYDVGIWLSLNNGNGGSGSGSQFITSRPPQAMDRFLLP

FVKEHCMTTMPPAADFEKLKAVFYQKIHPIFPVIPPSAITGDSDSTTEIVLKQLICLAAS

TDPNSAQYLHLGGTGPGGLLSPQEFSQTISSAVRATLETSLIPDRVLHIRALTILSMYTQ

PTCAEEADLPAQLGGRAIHHVQTLGLQLLGFDAPNCDELESLFCAVWALDRLNAAQYGRP

CVMHERDIGTNLTNCIRKQQPCFRLLLSVIQWLDHVIDLYRPGPTAEASGAQKVAFMELP

VLEAMIVDAEALRVPSFLIATIETLYHAVIILSCRLHRPGTLPAASTLPPPSSNARRSLA

ADRIACAIPRDHLSPMPFVPYAMSLALSVEYRKMRHSRLPMFRARAMNAFRRNCEMLRQF

GDYFWSARVVANLGERVMKEMERAATTLTKEATPPAESATAPQTTNSHAAPNLSAMPLAD

LTAIDTATGLDNLVDFSMVDAVSGQDLFGHIDPNFNLNAVEDALEANLDIGLPLNWGEWG

HFAT

>SoG_06156.T1

MAALPQQPLTFSDVFDDDDVEPKTVQHIRANSSIMHIHKILGEILPCNCLFANRGEIPIR

IFRTAHELSLHTVAVFSYEDRLSMHRQNPPEADEAYVIGKRGQYTPVGAYLAGDEIIKIA

LEHGVQMIHPGYGFLSENAEFARNVEKAGLIFVGPSHQVIDSLGDKVSARKLAIAANVPV

VPGTEDAVATFEEVKAFTDEYGFPIIIKAAYGGGGRGMRVVREPESLKESFERATSEAKS

AFGNGTVFVERFLDRPKHIEVQLLGDNHGNIVHLYERDCSVQRRHQKVVEIAPAKDLPTE

VRDAILADAVRLAKSVSYRNAGTAEFLVDQQNRYYFIEINPRIQVEHTITEEITGIDIVA

AQIQIAAGATLEQLGLTQDRISTRGFAIQCRITTEDPAKQFQPDTGKIEVYRSAGGNGVR

LDGGNGFAGAVITPYYDSLLTKMTCQGSTYEITRRKVLRALIEFRVRGVKTNIPFLASLL

THPTFIDGTCWTTFIDDTPQLFNLVGSQNRAQRLLAYLGDVAVNGSSIKGQIGEPKLKKE

IIPPTLHTADGAKVDVSEPCKKGWRHIILEQGPKAFAKAVREYKGCLLMDTTWRDAHQSL

LATRVRTVDFLNIAKETSHALSNLYSLECWGGATFDVAMRFLYEDPWDRLRKMRKLVPNI

PFQMLLRGANGVAYSSLPDNAIDHFVEQAFKNGVDIFRVFDALNDVDQLEVGIKAVHKAG

GVVEGTVCYSGDMLAPGKKYNLPYYLDLVDKLVALDIHVLGIKDMAGVLKPHAATLLIGS

IREKYPDLPIHVHTHDSAGTGVASMVACAKAGADAVDAATDSLSGMTSQPSINAIIASLE

GSELDPGLVPSQVRALDIYWQQLRLLYSPFEAHLAGPDPEVYEHEIPGGQLTNMMFQAQS

IGLGSQWLETKKAYAEANELLGDIVKVTPTSKICGDLAQFMVSNKLTPEDVKARAKELDF

PGSVLDFFEGLVGQPPGGFPEPLRTDALRGRRKLDKRPGLFLEPVDFAKVKKDLTQKYGG

PISETDVASYVMYPKVFEDYKKWTQEYGDLSVLPTRYFLSRPEIGEEFNVELEKGKVLIL

KLLAVGPLSENTGQREVFFELNGEVRQVAITDKKASVENISRPKADPTDSSQVGAPMSGA

LVELRVKEGSDIKKGDPIAVLSAMKMEMVISAPHSGKVASLQVKEGDSVDGSDLVCRIIK

G

>SoG_06177.T1

MSTSTSTTSAAAAPHKHSRVLACVLCQHPPARKRRRPNQDLQERLARCEDLLKQYAGAGP

PPGTAGSRVGDPLTPNTMETAIDPPNKSNSSPDDDGRSHQWSTPHMRAAGKMIKDDAGVR

FMDSYVWATVIENLQAMKDIIETEEPEECSMVDSENPSPEGNSDLLLGGGTDTTDIHELQ

PDPVHAFRLWQLFLERVNPIIKVVHIPTLQPYVMDGAISIGNVPLNYQALLFAIYTISAV

SVSEAEAPQMLGMARADAIEKFSKGMKIALVQFDFMKNYDTAALQALVLYVATLQGRYDR

HAIWILSGTMLRIAQKMGFHRDGEKLGLPPFETEMRRRIWWQVIMQDLKYAMISGLNHSL

LPVNWDTKRPSNVNDADLYPGLTEPVQERDGPTEMAFILVMTEVCKFMIIAASNPGFDAA

VMGQSLDYAMGNEAQAAANLEKYREILRDLDVNLKEIENRFLDASAGNVHTAALSIRPLI

ASKLSEMLAPIDSTLNTPQDRLMKMLINNLEHNADSFETMSRSGFGWFVQPHFQLEVFTV

LTGLLAEKPNSPMADRGWKTVQIVYRQHPGIHDTSQKPFYRQAQFTLKAWRARENAKARN

GLTVPCLEPDYISRLKASVHAYDSRAPSQLSVTPPNLNQPPQLTELDSFLGGYLDVSQFQ

WDDMWNTMPTASPDQALPTTEFGSLNMGPAPPMMKPEMGPF

>SoG_06178.T1

MATLPRPAKPMVVVLGSTGTGKSDVRKPGYSLEGALLIVTMQLAVELALRFRGEIINADA

MQLYEGLPILTNKIPAEDRKGVPHHLLGHISIEEDPWVVEDFKREAERLIKEIRDRGNLP

ILVGGTQYYVSPLLFSDVTLDEVEGNRSAVFPVLEESAEVMLEELRRLDPEIAQRWHPRD

RRKIRRSLEICLQTGKPASAFYAEQQKRKDEAMQTGSLAPWENLLFWVYAERETLIKRLD

ARVDKMVDGGLLQELHELYSAESRLRQRDTGLNLSKGIWQSIGYKQFESYEAALAAGKDA

EELERLKAAALEDMKAATRRYANYQTKYIRGKQVPLLQDQGEAALNSLYVLDSTDVESFQ

DSVIEPAAQLLSQFLNGEPRPLPADLSDLAREVVSRVSEPQEKKSFTQIKCDLCGTVVMT
[truncated: 254,019 more chars]
