# Supplementary material for: De novo genome assembly and annotation of rice sheath rot fungus Sarocladium oryzae reveals genes involved in Helvolic acid and Cerulenin biosynthesis pathways
Source: BMC Genomics. 2016 Mar 31;17:271. doi: 10.1186/s12864-016-2599-0 (PMC4815069; doi:10.1186/s12864-016-2599-0)
Supplement: Additional file 5: — Secretary protein sequences of S. oryzae. (DOCX 266 kb) [file 12864_2016_2599_MOESM5_ESM.docx]

**Additional file 5:**

**Secretary protein sequences of *S. oryzae***

>SoG_00038.T1

MKFSQLLVASFAMGAIAAPTLENREDQVGLREPASETTEVEMRFTDENDIIVRTKVCKRR

YELVNNECIRRCGRHQIRTDGVCVPGCHKSAKLVNDKCVCPEGERYTKRHGCTE

>SoG_00071.T1

MHFKTLLLTHLAALTVLAAPGSSTMSNTGRDVEELFPVERDIEGRAWSFYAGGGGCLTGW

SGQCNFYCKDNIRTQKEYKSCVLTKSEIGRFGCVWGWSKCKCHCA

>SoG_00087.T1

MAFIRFTAQQLRALLETLAAALGEDSGSGSGPTDYQHTSSDDEVPGLTDTDTRSDSSLGG

NHPGGPGPSSEEELDRELSAAFGPQFNRIEAEIAANDGYLPSSVAGIMAMTGHGSPWSRS

PSPTPSGGSDGHSRNSPGGRPASETFQDWPSDATIWALSDPDDVSVSEWHSDSSNSNSSD

RDESEADAQGEEGEDEGGPARGCKRPHEGNSESEEPPRKRRMR

>SoG_00096.T1

MKNAFILALAAAGLVHATGNTDCTKDSCCEAVNPDFCQQFLQPNYGPPPRIPAQIKQACP

GADKRARVSSACSCNAATQTTQQPSQPTCGDGYGQQPCPPPPGTTTTQSPSQPTCGDGYG

QEPCPPPPGTTTTQSPSQPTCGDGYGQEPCPPPPGTTTTVQPSQPTCGDASLLDSPGDYC

LY

>SoG_00130.T1

MKPGAILLAALAGTSAAADLTSRQDEPEIKAASFGPNQLKASSAPNAVSMKVLREKKVQQ

HQKDREQGVFDLNRYKAQGAASCENGKSGEYSCKNVDLRGFLRHQDMNSRTREGNDVWGW

TAPNGREFGLVGQTDGTAFVEVEKDGSLTYVGRLPTQTVASSWRDIKVVGNHAYIGSEAP

NHGLQIFDLRKLLSVNKQNPVTFRTSTGSDSGLTALFTGFGSSHNIVANEETQTVFAVGT

DTSLPCRGGLWMIDVSNPARPQDNGCVSNDGYVHDAQCVLYKGPQAAFRGKEICFNYNED

SLTIVDISRRAMPRQLSRTTYKGATYTHQGWTATDDHRYLLLDDELDEQEANGPAADGHT

TTYIVDITDLTYPVFRGVYKSPVRSIDHNQYVIDGISYQSNYGYVLSTPLSPPSRRICVN

KVPASSGLRMVNVSSILDDDSGAKFEEIAFFDVRPEDDAVGGEVTFNGAWSVYPYFNSGY

ILVNSIERGIYSVKHNPAPQ

>SoG_00141.T1

MCWVVLRQDTGSHYVAVLALLTLVDADHAHDACGSRLDCDRAREVKLLTVPDHRRARVPE

VGVCFHKMPLSCSWRSHDGLRSARPPIGAHQEGVEVMDRPQAVAAKLQVVRRSANTDFAE

IKGLFAVERGPRVAVGNRRLAETEPVDDWTVIVADVMQDGSLPKCEAESESPLLPHTRPS

ECARKDAPSGWTTLSGSRNISEAMQISWRSFISALRPLIKKEYRPTERALPRHLHRESWL

RRRLHHSVRQESLEQNIDLGLRQLDLPCQHSIVLDQSETSWASSVVGECAVVLGLQERQE

LALEGFKKVNRAKGHRARRPDMTQDEISSYEDLIFAQGL

>SoG_00144.T1

MKPATLLFIAVNYLGLASSMPTPDSEDASPSLEKRKCFSTGETFGKQKQAALDAAEWACN

GDLIGNYNKRETRVKCYDIGGGKSMKLTVGLTGKNAPSTRRIGVNECIDGLHKEIQNCGR

GGDTTYGNWRYRADPNKGGC

>SoG_00149.T1

MFVPALFRFAILLLLSGSGHVWAFEHIKGRHSATEIPADDIGDPFARNHLVEVSWNHPTV

RASLWNGNPLPSPSPSKGRKRLLSKRSPGTGLGPTCLGCFESANGQQWTIGALNTDFLVT

QMLKSEQDIEGKCIFYTGVSEEDKVQQLWGRGENLSRTAAEYACRLGMYSIWTLFPGRNE

NVVPGQQPPDDVTRRNYWEIKTPNSWLSHLANTPSQFTYFENMSRAMARRCGGKVFVMSL

RPEKLTYYGSGGQDNEHSIWNTVELPELRAQGKITELIAIDINDPRKRKRIDINDQSVLG

DYQGTVPREVMEFGDMLQKRATCTENLQYQPPGDDWFGSGRR

>SoG_00183.T1

MFILTNALCAAVFFFCYPETRGKSLEEIDEIFGDVKTIHIEDMKVPMAEKDIGPVEVTEA

RDNRIH

>SoG_00206.T1

MAVVLRTFMVARLAFALMPAGASSPPSEMSVAFKDGLEVLNEGQGVGLNDITPITYELFQ

WPWGTIPQRCYRGAVDDDLCDPYEMEVYDAWFADCTEPWRVCRCKNSPMTAKNVADGMAK

IPVPSRQWTRFWSSYNGTLSAVTGGNDVTMFGDASHVTSLFFHEVAHSIDSWAMGAENQG

SASSGRALWRNIVANDTCVADEYAKTSYSEIYAQIYVMMAYDARVGNIYNYNVQCMANQV

EKGRQLLKSLLHYSPKDECTRRWSERDSPRVCMGHRARMLSRCNGVPDGDGRHRVLREGT

ERATAGASHGDGKSKHHDAALMGMLVDNVVVEKGSIKIEENRAKDPAMQRALEERDAQRW

SDTLLRAGVRQR

>SoG_00233.T1

MLTNSFLQLAVLSAAGLLKSVAAVDDPNVYGTTYIQQPPIVTTTVIQQPPIIPSPVANSS

SVEGETLLSQCADSITACGTTAEVKVIRTHNSTIYKTNYKTVESVVVLGHPTTAPVVLVL

ACTDPAFQELKPKTRTVTQVVVSTTTTGVEGQCRETIVHVEPTPLIVTVEANTTIVREFV

PIVSTTVTTTSWVAHKQISQCIVKPSSTLGVGTGQPLNPPPVAQPPVAQQPIAPPPGSGQ

HNHDRRRAEPAPPSSPGGTGRPSSAPPASSQQPSAGSGSSPGASSSSSTSSSARTRSTP

>SoG_00254.T1

MLKNLLLSIGWSLAGIGPAMAHPRVDKDLRQYGDGDESSTSIATVTVDWLPPHGPESSEY

PFPGDKTSSKASDYDETDARLGENISWKRGPMLTEGNRVVTVTMTVYSPELTSNEVGGGY

TDMYPVPPEPSSDMSSSTPPVVLTTTITHHGPCYPQPPEPSSSTEDISVVIVTVTTTEHR

LVTVTAKVTEVVSTVVRTLSTRGPYAPPLTTW

>SoG_00277.T1

MRLLSFTAALLGAASLSSAAGFSVGEKEMAGWLTPPLLPLIKDAGSSKLFPMADCFGFKL

EEATIDEMQKAMQDGKLTSVQLVMCYMMRAHQTQNYINSIIQFNPDAIAIATAMDLERSQ

GKVRGPLHGIPFTVKDNFATRDNMEATAGSWALLGAIPPRDAHTVKKLRDAGAVLFGKNT

CSEWADMRSNDYSEGYSARGGQLRSSYNFTVNPGGSSGGSAISVSNNVIAFSLGTETDGS

VINPAHRNAIVGFKPTVGLTSRAGVIPESEHQDSVGAFGRTVRDAVYAFDAIYGEDPHDN

YTLAQKCHTPKNGYIPLLADKSALKGAKFGLPWMSFWRYAPAQELEVLTEILQLMKDAGA

EIINGTEITDYERIVSPTGWNWDYGGSRGYPNESEYTVVKVDFYNNINKYLSELENTDIR

TIDDIIQFNLDNVGSEGGRPGVHPAFASGQDGFLASQATKGIYDGTYFQALHFVQSSSRN

GINDALRLNSTKDRRLDGLLVPPDVGQSYQTSAQAGYPVITVPAGESIVSGMPFGLAILQ

TAYGEKELVRYGSAIEDLLKGTKFGRKLPEWRGYLERNIPVPF

>SoG_00339.T1

MRIARLTEQSAWLVFFVSAGLVLTSHHTLSGVNPRPIAAVLAILSVVRLVNEPRSQKDSW

VVALRLFVADYLRNASLVAIALAVGFSAPTRAAVIDQEADWPEEYTSQSSQDPASRILSE

KLVGPMITDWLSDVSSICFKHMGPGSRAPSRSSWSSRSSRASDILLNLDRPDAAREQWIQ

GQGQIIMGRPQNPIPQDILHDHPDLP

>SoG_00446.T1

MRYLQLGLVFTGLQSVVQALQVVDIGSDFQIGQNSFTHRKPGSDFQETFSCAHTGKVLTF

NRARTVAACCTPNQHLDGAYNTEWHCCGGGHSVAGNPAVGYRCCPDGEGYDGRVCHEPGH

GRPGGAVPRPDGHGGYGAMMDNHPSAQVQFFKAGDSQGGNSQGGNSQGGNGQGGNSQGGN

SQGGNSQGGNSQGGNSQGGNSQGGNSQGGNSQGGNSQGGNSQGGNNQGGNNHPHPHPHGN

PNTPGYCHKPAGCQSGITSSKCYLFKMDDGHYFGYNSKKGHYTSEPDSANHHMGKFRFCR

DSNCTAGCAIDPGYGVKIRDLHGDPNKGTGANQWVGGQKDGDHIKKEPNYSAAGEFTITK

WTCGKYCLGGFQSGVGPTCPSDAPAITFTTLDPQSCVPVELVEVPCDIRSTKNNCRWQKE

PGSCGTGDPRHCKCGYDI

>SoG_00541.T1

MGLLRSIALLACSLHIFSLPSYVAAQDAPLFVQGGLEDATVDDATIYNSGGTITVNGFNM

VVPKNVLVQFPAAWVPWKDYVQNKAEYTGFETLVLGNTVNGVHRVAQVVTYEFFEGLASG

FIESINYEDGSLKIRGGPTVRINDPNGVFSVGYKGAPHMTADDQSPSITSFSGFPMCIPR

NSTDPLCPLSNRPFKGPGTFTAPDPMVMAPFQEGDFITFTGFRKGGEVIAFSIVAQNVQI

QTLGDIVYVRMELGLLGIDNPNANAELAESRFIGFTSNNRARIALYAMDVNPCTGEVTDR

ILATMGLRGGRNEQNKFEYRNDILHSYTREYRVIAEINGVAKTRQTKNGIMSGTYVQPVN

VWIQGEQNIPGTPPPPHDFSEMAFLTKGVGRDENGNLWGPLEPFPQTGVFIEDQNCPPVA

AKSRTSRKKRGAMGQWYSRAKAEAEADAENKGTTPGALTIEG

>SoG_00542.T1

MKLISFSAISLFIGVGLAAPTLLQRSSCQVGSVGVGPISNAGDAACSASSLRMQLELVGR

PSLRKLVYGHFDIFDMVS

>SoG_00587.T1

MVLQSGLSLLAAATAASAFVVTPLDVLNQLPLVSHHSSLKTQCELPPVLSPHGDGLPSSK

DLFTTSDALKKQVQRHQAIVRVPSVCFDDLGSFDEDERWAPFYDLHKVLAETYPVVHKRA

KLEKVNTFGLVYTIQGSDKSLKPILLAAHQDVVPVADESTWTHPPFDAVWDGEWLWGRGS

SDDKNSFTAIMSALESLLSSPDWTPRRTVVVASGFDEECSGYRGAGTIGPFLEERYGENG

VALVLDEGGLSIAKVGDALYALPAVMEKGHVDIWFELHVTGGHSSAPFPHTGIGIMSQIV

NVLEAHPYEPSLNKEHPFYTHMQCQARYSPDAIPEVTELVRKDDVEGLAKFIGSRFPLQF

LVQTSQAVDFISGGQKINAMPEVTTLGVNYRVAPQDSIPEIQHNIVKYISGIVEKFDLKV

VAFKGDEEYAAYVAAHDAEALSIPTKIDYNGELVLRATEKSHVTPKAPTEGPVWDVFSGT

IQHTFAGDEKVVPVGEIMTGNTDTRHYLKLSPNIYRFTPGRSTGSENIHTIDERVRMEDH

MEMLSFYYDFIRNCDAADTF

>SoG_00599.T1

MRFHTVLTAAVLPAVGFAWGSQPQTVSPPEYETRQLVELLERHNLALVPRSELTEALQEL

NGLLKEQRLAMPVLIKRQNSTGGGGGGGGGTTSGDSSRASGGNSQAQGLFDIPGLSDLTN

SINGLGDLIKALQGLLTAEFLQGFHDAMVYLAQTLQPPAPEQIRGLLKQAGPLLELVGEL

DLKSLINEIKGIDLAGIVNKVLKLLTDSNLNNINNLVTNGAALLTPQFVNSTGSLINEVG

PLLGDLSPLLKKLTDLDLAGILDALSPILKKDAIQKVVGLVDNAGNLLTPQFVNSTQSLI

NDVGPLLGDISPLLKELTKLDLQGLLKSLEPLLTKESVDDIVNLVKNGGNLLTPQFVNQT

QALIGEASPLIQKLSEIDLKGVLDALSPILTKENIEGIVTLLKNAEALLDQDAVKQIKQL

LKTAGPLIEELGKIDLASVVKALQPLLTDESMKGIVQLLQNAEALLTQDSVKDIQSLLKG

AGPLIEQLSKIDLAGVLKALEPLLTEDSMKKLLELLANAEALLTKDTVDSLKSLLQSAGP

LITSLGKIDLAGVLDALSPLLTKESMQGILTLLGNAENLLSKDFVDQTKSLISVASPLLE

SLSKADLKGLLDQVSPLLAELGKLDLAGLLQSLGPLLSPDSIKGIVGLLDNAEDLLSKDF

VDQTKSLISVAAPLLQSLGKADLKGLLDQVSPLLAELGKLDLAGLLQSLSPLLSPDSIKG

IVGLLGNAEDLLSKDFVDQTKSLIGNASPLLDSLGKADLKGLLDQVSPLISEVSKLDLAG

LLKSLNPLLSPESINGIVGLLGNAENLLTPKFVNQTQALIDDALPLVSALGQIDLKKLIQ

QLMPLIDAVSQLDLGDLINKVKPLLGALMKIDFAKLIEKVMPLLDSLDDIDLAGIIKALE

PFLTPDSIKKLLGLVGNAEDLLTAKFVNETQTLISGAVPLIGVLDSIDLPGLLKTVEPLL

KELDGLDLTGLVDAVKPLLAALKQVDIKGIIDEVLPLLTPANIKGLIGLLANAEALLSGQ

FVGQVSELIGDATPFVNEMGGVIINSEDSSFQKGMWYLRTVRK

>SoG_00600.T1

MHFATSFFGLAAAAAAVNAASVTFKTLDNVTRKIVFTASPGSPAIPSVTVSNAQDTTVQF

PDHYQGNFYAVAEGSQDKPGMLGELLFGGWMGFTYFDVSAIVDPNDKNGVKQMYPAETKQ

PMSGCENFPCNNAYYLPDDVQTKVTHENHIITTLGGSA

>SoG_00613.T1

MHFTLSPLLLLALPVLSDAQLLPFRVGPVLKDPSSTADKRPVHRPLALGQLPIMPPGSSE

DGDMTIQPSVPLNDILGTLRSLTTFSSLTRMQTTTTTLLSDLSTNTTVLAPLNSAIESLS

RKPWESPRDYDEHGVSAYEGDDGQDRANANLRRFVEAHLVTKNPWEKGEKAKTIGGRDVW

WEEKDGKRVVMPDEVEVERVANQVANGEVWLLKGVLNYV

>SoG_00643.T1

MVKLNILALTAAITGLSQAVGQSKITNNCDSSVYLWVVGSNQEGPIEIRAGTQWKRDYFR

DPVTGGIALKVAREADGVPSGDPHTIFSYTLDPDRIWYDLSDVFGDPFEGQNLLLRGEGQ

CPDIEWPNGVPLGGKSRTLRTVDCGPENDTNLYLCGMN

>SoG_00645.T1

MKFLLSFLLASLPLASAQFGGFFEQMFGGQEGHGHGHGHQQQHNNPSDANNYRTQFEQST

NRSRSPKNCATARSPELRKHLVSVHNVERRSIALDIPDVEQEP

>SoG_00653.T1

MVTIIIAIIIAKSFLPKPPTSAVPWVSILGDLVLVPGPRPSFTFADVAQLERPSHLCLLR

AESEVSITREFPSPRPPAASTTSTITTPSYANGHGSNPSAARTPLNVDLSSLLKGGGDPN

ESYFDIQRHQGRPSSVPVLSTSAPIALTPGQPPSYVPNLPAAAAPASSQMPPGNRRGGAV

PPQQGHDQPAVKKQSKWTPDEDALIIELRGSGMKWEDISKRLPGRSAISCRLHYQNYLER

RSEWDEERKNKLARLYERFKAEMWSKVAEELAVPWRAAEAMHWLLGEADMARRAGVVPFS

LAAVNVEGSQGGGARHSPQRTHQMPPPPGQTAHPREVAPAPHHRELHSRAHSMPTAQGAT

PRRDLYAGAPPPSQQPVLAPAPPPPHQQTGHPPQHQPSVSPLAPVKLEFNEQSFAPPVPG

PGLAPIQAQPRQRNADYLPGIAELTTGVGPYGHGGGPQAAPGPPTVTTTPSGPPGPPPAP

SAGYQGYQTMEPMRLKRQASTDAGLNEPTSRRRVG

>SoG_00655.T1

MLFTALLLLGASYEVHACSRLAYVANSDRIVVGRSMDFVADTNTTFWAFPAGLKRTGSSV

ESNPFNWTSKYGSVTGLMYDRVHVEGVNEKGLAGSTLYLDDSDYGKRNDSQPSLWVGHWM

QYFLDTYATVDEVAKAVCPGSGGSPPFQVVATALVPGVKSNGHLAYSDPSGDNIVMEYAN

GTLKCWQSSNYSVMTNEPPFDQQLDLNNYWGPLAAAFLPGTSRPADRFARLAYSNTQIPK

ANDTETAISYCAGMIRAVSVPMVRELVHSPGASDVWPTYWRTYIDLKDMTVFYESATNPT

LFRISMGDLDLSTNGTTKVLEVKQVPWEERVGDVTSKFTNATSAQCAVGDTKC

>SoG_00669.T1

MLRNLLLAALPLARAAEAGRQKFSPRMVEGQMCCPCPSANNGQGSDGTVTVYVTKGNAMP

TVTVTMESMPDANPAQATVTLYKPPGGGPAIGDVDGDGDVDKDDVSLAGMIGDVDGDGDV

DMDDANLAGKGGNGGTAANGLGSDPASKGPGSGNAGPAAGNSGLGNNPSSPPAGTGNGAD

PAAVGSEPLSNSLPGTGNGAPGAGNGMGTNAIGNNPTPAGTASGNQAGNQAGNAAGPAVG

TGRDGAGNNGLGGEGATPGSLGKDPAAASNGGNNAPGSKGMGTDAVGNNPTGTGTSADGL

EEPCDNAAGNSGATTPGGAGVDALGNGAGGANAGTPASGATGTDALGNGGAGAGANSPGS

LNGPAGAGAKQPGSGATGTDPLSSNGVGAGGSSGTNAAGTDALGNGGTGGGPNGLGGNAA

PGAGGDNLGNAANSNASNGAGTPANGAAGTDAQGNAGTGDGANTPGGANGGTGNDALGNN

AAGGGAGGNAGLLTKTVYGNAGPKTVTLQGGSPKVVTVTAPAGQPSTGAAGAAADGLDES

DELSNGGTTPGAPGSDPEDGSAQTVTVQPMPGRPAAAGPGAGDQDPAAADAAADAIAPKP

TDIPCDQADASTSISTVYNTVVVTVGGAPKAKVAVPTTGASNGTETVDKPKHKAKRSWWP

IPGKA

>SoG_00690.T1

MFVSSLVVAALAAVASAKQCRHVHIPLALTARGAEFNLEPLLTEIDTTDFFLGLSKQGTN

YTDILLDDYHTINGIYKIAATYCQPDSGPGHALQILTHGVGFDRSYWDLPFNKGNYSYIA

AAVDGHGYSTLSWDRLGVGASSKGDPINEIQVFLELQALKALTEKAAQGTLPIGTLHKFD

SIAHAGHSFGAAMTYGLARLWPELTNAIILQGFSHAPGFLSYFALGGNFVPVSQVKSLKS

KYPPGYLGVQSSVGVQINFFGPKNFDPKMLEYAYQNQQAFTPGEIISVGAGVGEQNMFTG

PVMVITGDNDVPFCGGNCMGTAVIGNSAPNLIAFSKQFFPKASTFNATVVPGTGHAMNME

YTHPTTYGAMLDFLDSKL

>SoG_00715.T1

MSLSSSMVALALAALVPRSVALNFTIHNGQIFTPGFAILNSPQPETPMGGDNLHISLDVT

ANGRLPLADFSNDDPSQIFNITVFLYSYRTERNFTITNGTAGANNASLGNIMQQEPGSTV

KHINWAWPDCLVGDGRPDSEGSDRGVYNISIRQNFRINNTNHYTIFDVPISVTNRIEEAG

KRPSCDSLNNNILSPEEIGMESANEIGVLFAPGKASAVDIGKMEDKYKDQDPGKNSNSDG

GKEGNRDGNKDGLGGERDRAGPGDGLGDSAVGSHLRWTTLTAKVFPSHESLRPSPPTPKS

FEPFAVSHEEFEPFQASYEVFTLELSHTGLSRSFEDGRLEVTSSNATSARGTASSVSASM

DRPWACWLDDLPAEIFIEVANQMAARSRLTCRLVTRNSYYRLSADDLCLRLLRGLYPRSR

EARIATMLLSRGRMDWVRATDWFAVLFRVARRYEAFRAGDTVKASSVRLPSRYLGSPNEQ

SCDVMPWEMTKGISTDQDIWVEENVEFFKYPDCRYSFAPEDALLVYPAGGPGNIEIVAED

LDTGQVYEIPFNSAGKVIRRLRMAERVLIVEWCEQVPSIALFEETHVASHYVTVFEVTTE

ARLNGRLPLKHGQHHPPWRFIPLAEWELEEVVTIGDRFLSAHNRTHYAVFNWVDPDISPN

SESLVVWQFGKGSSTFEPIVYGVLLDGPIEPIDPTLIRHYDAAELQQQWGITHEDGPWIV

NMYIDKCTWDRHSNSPCGHVYFHTEAHPHLVGQEHPETDEGLIQDAYPVISAGVPINGSG

PQWNDTCVIEWPDPCPRLWPDTSLDPVRAPCWRHQRGDTNMYLMWWLSDDDETIRFFSRR

CEYDNYTPGSLVLGRYMEKLSNRDRLSCVGFEGFDPEGYKFIDAPCFDTNRQPMDDRAGV

AYFEGNARRLVYRMDHGQNPFSEPVILTF

>SoG_00717.T1

MSSSRSIVSLVLVAWLAQYHQPILAWLNGALLGRVSPPKSQLLHWENSTSLNSGDCVVGR

AADACEDVKIHFASGAAFLACGDPKGRAAWYPGAGRHDDRARTDYREKLFRYDLEKKKTV

ELTVQGLEGDFVTHGLDVVDVPGDKTRIYIMAVRHSRGDDAISIFEHRLGSDVVTLVKDV

QHPAIKTPNGVVAVGPLEFYITNDHYYVGGFRRYIEEKFGPWSWATNVVYCDARREEVQC

REVAKDFIAANGIAANDGKLFVGDAQNGTVSVFDMRPDRNLELSHRVTFIQMLTTSGQQT

VFPTLENLPLYLANTETLGRDLLVPAAALRLRKARDYAPELLYMDTGAAVSFMTAMAVDP

HRGLAIGVAAFQYGAFAVCRIGEETMRELK

>SoG_00724.T1

MKLINVLALCGLTLAHMEMIDPAPFRSKHNPFTTNIDYDMTNPLNRDGSNFPCKGYQDLL

DTPQGSSVATWQPGEIKTLTLAGSAVHGGGSCQASISYDRGNTFTVLKSWIGNCPLRNDW

TFTVPGDAPAGEALFAWSWYNMYGNRELYMNCAHVTIGGGNGGALGGRPGMFVANIGEGC

YTAEGYDVVFPNPGPDVENTSGKSAPPVGNCK

>SoG_00748.T1

MVPSCQVRHIAVRFAWLLAALSPVYAGFDAGLKRRPGLTGLRDVVRAEWPRQSRHDGSNQ

PGPEDRIAASQDPLRCFEVHHPVLTPDGPVVDERRMRPLPLQENGHGSSSCDRVLMEHSF

ANSYGSPYVAPYQPPDCAFNRVVLRLDVVSEGRQFDRLAVMYLSDVEVWRTSTAEPKPRP

GIVWTLTKDFTPYLALWREPQTLIFDLGNIVNDKYTGAFNATLTATFFTSDVLDPNGDSA

DRIVPISARRGAEGKGSAFIYPETPAETALTLPRNINRGILTIAATGQADEEFWWSNVPE

KDKDVFNLKAGPLPGLSSYREVQVRLDDKLIALSFPFPVVFTGGIAPPLHRPVVGLQAFD

LREQELDISPFLGLLCDGEEHKLTFDVYGVDDSNGTAKLVPAHMHWVLSGKLFLWLDAEG

AQTTGSAPEVSIAEFNYTAETALVGDAVLIQQAVRRSIQVTALINTQSGSMTRAWSQDFS

MDSKSSLSDKGDTQHVEGLYEHADSSSVDEGISFFSNGSYPINSDSQYRAPDGDYDFTIK

AALLSVLNLNLGGRHVFPTGLEPFVHEAEESWNESAVHTWRRSSAFFHASNGGNSSGGSG

ETEQSYELTGAVPLYMRFVSVANETTVRDDEFGLGRRQSHMTGSGITDPEPTRQSKLDSL

PHWPSLGQHMLRAWPEQWNPELYSPLLDLLRIISDAKVGISVSGHHYFTEYAELDNSVLG

FPTIY

>SoG_00878.T1

MKVSTLAVLASAGPVSALIKDCTGTARNEGGNWFCGAVDHILYSGIKGSGSYQRVTKMTA

GGECLREPRAYSGPLAPLDEDLSLHFRGPLELEEVAVYYPSGSKKRDVEAPSSHVHARRH

GHAHFHSERKAKRADMVVATINGQVVSWENNWFGGAAAPAAPTDKPAPVANAPAPAPAAP

APAVPTEAAPAVNAPTTNSQPSKPASKPPVSNLISKIGSNGKDWNRVAYYNAKNGVADNL

VFLGNYGGQGSGVFDTVWGNSLSYLNAAGSGGASKPQVLQPGLIPSNKEFSIWSAEPCDE

SCGFSRAPDVAYKGFTGANKVFLFSFKMPMDGNRGFNGDMPAIWALNGEISRAAQYSGCS

CWTTGCGEADFIEVLTSGDDKCKSTLHLTNGGGSSDYFKRPVDKFVRYAVVFDESSASMS

VRQLPPGTDFAEGFDAKTVNSWVEGGKNDLFSLFQMG

>SoG_00905.T1

MYFSPSKVLGGVLSLAVVLEASAIPNIEERAASDICKRLDMLVITGVLTATGKATVICDK

IKSLTKRDEQELDPRFFNWPKINIPDIKIPDLQLPDIKIPDGLLSYPKSILTQACNCLAP

QGSGPDFTQIPNYPPSGQFCTYINTYYVAPTTSNSSNNYAAAKADCQAKCKADSKCKFVE

VVKTKGFFGSESVTCTFSSQAYQSNQVFCLSTAGLAQTIAGYNKN

>SoG_00914.T1

MQLKAALIALAGLSAISEAAAFERSSPLDRALARRQFGGGRNRGGNNGGNNGGNRGGNNG

GNNGGNRGGNNGGNNGGNNGGNNGGNGGGNGAQLLAQNRQEASNSDGQANQDAGQSPSAT

DPNNFINFCTGNEVTNGEQKEGGSCNGVPMGRIPGTGNMVSSVITNPQNNANIAADQSFT

IEVRMNNFAPGTFTNPATTYYSAPQNLDGSGNIIGHTHVTVQDLGNNGNPTQPLDARQFV

FFKGINTPANGQGLLTADVDGGLPAGNYRLCTMAAAANHQPVLMPVAQRGAQDDCVRFKV

GGNNGGNNGGNNGGNNGGNNGGNNGGNNGGNNGGNNGGNRGQGGGQGGGNNGGNSENPQN

NNNGGNNADGGNQGGSEGTGAGGGNNGGNGGATNGAIGGLTAPEVTDTGDRNRPFGVNGN

TFTTKQAAVERACDIQNNACSDGVNRGGIQGKTLADCNAQVAKCVSDLS

>SoG_00915.T1

MRASLSIATAALLLAPSAAAWGSLGHITAAYIASRFVENTTQAHFQELLRIDDDDYLAKV

ATWADSIRYTKWGRFTKNYHFIDAHDNPPASCNVDFDRDCKDDGCIITALANYTAQALST

DLPAWRRGQAAKFVLHFVEDLHQPLHNEDVQRGGNGIYVKWDGRDFNLHRVWDSSIAEKW

IGGMRGSPFLIAAKWANQLVVEITDGKYAEEKDEWLNELDFSDPMKTAMAWSREANAIVC

SHVFPDGPEAIVGQELGGEYFEKAGPVIEKQVARAGFRLAVWLDHIAKEFEARQKLDNVG

AEL

>SoG_00921.T1

MRRAMMRQLNLVLLASLRLGAARSDGFSIHSDLLAFPQYEVIFSEDAISEKDAEVLLGSN

APHPTYSAEFTQPTGTGSHDGPDPSTDEAYPRTYELMNMSQQKYLCSIPMIQPPALENET

TNELARAAEAREHSRAAAHGWNLLSELEDTCLYFGSGWWTYSFCNNREIVQYHALPAAPS

ADKPPKRDPRTAAFTLGREPAIPAQYRQRANINGGEDGEAAKPLPAELQMTGDHQRYLVQ

KLERGDVCDLTGRERTIEVQYQCKLGLPHDKIGFIKEVTICAYLMVIYTPKLCNDVAFLP

REEPKANPISCQLVVDDVNAVKPALLDARVRDGRDVRAATGEAAEGEEMDAGSRKQAPIN

IGGIAVGARHVLSTGDEAGKPPTKLSPPSSYFPPSKKGKAKTTEVVARGASKDDGGKVEM

LPKDDIEELGLSFDVIEEMREQLENISGGKGWKLEVVQEEGGQRELRGWIYGMDGDEEGN

EEDWFDKDQYDDEGDSPAGEGGAPGSGNGGRSEEGSEEKFKDEL

>SoG_00961.T1

MNRILFVTAAFGALPCHAEECDSSDLLKPVPRDVKGSELNCPTVTVVGVQPARVVCAGQT

TTTTLVWTYTDCEPTQCVPLPNGHPLPLGDHIDLGAGSKQGPLGLEGAPEAQGAAVNVPG

TRPSGQDSFPPPPPPPSGGRSSGDLPPNIINPTDTGSLPPGIPSSAAPPFPTPGDPAVVF

SADPNESTGIFFVSGSETLVNPTDVPVPSDNPANDAIPAIDLTSDSPAPDVNPTPEDPAP

DIDPTSDSPPLPDADPTSAPTPSDPASDPLLPTEPRISPAATSDQPLDAPSTTSNPGPDT

QSTSETPAASSSTSSPPPPGPACPDIPNGDFSSGQLSPWYISDQVTADSRAVVPAQGSED

HPHAFALIPSQADRAQVHLNNKIPSCQNPPPEVTVQISFDYQWERASSGCSIAVALNRSP

DDILLITDEGAQKGTWLSYRGDPITEQLNYDYLFTVKLRCDADTANTPAILITDIKVYS

>SoG_00964.T1

MVRLAFLGSIWLGCSLISSAISTAVPRANDGNGAVSAVAARDAAPEDAASLVTSLSERQE

SRTQQMGQQLANIITAFVDACRAGQLNTMGEWTKQMSMKLKQENPYKNIMIVHVKHDLNF

AGSRHEHFELKMPCVGWGTRGYEIYVFDYGEFWLHGDRGYENWAFAGADDVFCGRNYEWD

SESNGNYVRFNSISPPPSIPDLPTPAPPGNPVRANPDGNYLVNCVNTGTGVTSSGIAYYR

NMNPLERGNRYQSPDDYVDVSHGWYHTWESGFTGANIEPFPPRPVRFPKSGVTVWLYIVG

GTKPNTRNQNVGFGTNGYVSWRIYTGWHGLLYSVRDGDGWSSWDCYDIYFMY

>SoG_00975.T1

MRVRVALLVFLLSNFADAGLLHWLFGQDDLYVYPEPKWDQHYSDRFAKNPLEEDSKQEAL

IGLVQSYLTTMSSLKIMPWDPDADFQITEADMFFLAAYYNMTTYRHRTEAIPEERAYLLE

INPHFKHRDPDDMRNLIDARWIDMTTGLYIDITAARYALNHSRGEGILFDKNGHEFRVSI

LVKFESLKDMLTAAQDTYLYPLRRTTFENVSALIPYRYEDMLRSEYGKASLTNTDYNGYL

FDKTEKRRTNLDDDDTFGLDIPGLGAAPIAAIEEPAATTGEATQGSAKRRRIERPSSSRS

RSSQDGSSARRTTRNRPGTSSSLADVPEEPSPVPVAAPSTRSLRSSSAVTSTPAHPDSTQ

NNVRNEFETYEEVDASPLDAPGSGRRRVRSSAPASELSTRLQDAIGVSQEPSELSSPSAR

IARRKSDAAALSGRKERSSATRSSLRQAVRPDNEDELSPDRPRDANPEGDVAEEIEAVEA

AKVIGKKRPRRSLSRQSPDIGSQRPQPENETEAAEEAEELPPKKRRGRPSKSPVAQKQPP

KARTAASKAKTTRRTSNEAAKRVTKTKDTNASAKSAPKQRRRKSAGDDDGTEAADDDDAA

IEITVQRFVNLKKRSGADDDDDEDPLHSEIPFANRTGESTVDVFAQVCEEVISNTMGQFH

ELLNNSGADAAKKKEFRIKMRAIEAYKEELNSRLLQHAIHLDHWYTLRRRVRHVQKEKLT

LREEILKLKAEREQVALRMDAIRTKHEADTKESTYRLDASALMHNIDLAVERGRKAPELT

QKEQKEADLANLEFFVAQISGQASSVSAPGGLLKQVTEFNAFLERAAVALESR

>SoG_01035.T1

MMNNVARLALLGLGLGAAVVCATDHTVTVTETVTECASSCYATGPDCTATTSTEYVCTTE

SASSLPAVTTQVTSSSTNPPVETSKTTDSNTLPGTESTGTYGTSPETSETASSATYPLET

TSSYPGSTDSSALPVTTSGSSSYETTTRSTPGTMESSGAPESSSGSNPGETSTCTDYYGC

GSSLSSSESTGTTTLRSTLTSFVTFSEGTSTYGSETSDVPETSTETYSTPSTQTESSGTL

PVTTTYGSETPSTQNYPGTESTPGTETESSKTYPGTTTYITDTESTQTYPGTESTTPGTE

TGTSSKTYPGTATYGTETDSTKTYPGTTETTESTTYEQSTTQTETYDSSTLPITTTTTTT

SGTESTQTYGTTSSSTCTTETSRVPHGPSSGTSWYGGNTTYTQTTKTIRTVTISTEESSS

SSSFSSPSVPLSTWTSYSTPVESSSSYYYTPSTPVEISSSSYYSASTPTADSSTLPLTPS

ESSSTGYYGSSTPIESSSSYYTPSTPSADSSTLPLTPSESPSTGYYGSSTPVESSSTYYT

ASTPPVESSSSSYYSASTPVASSSTLPLTPTESSSTGYYGTSSTPVESSSSYYTPSTPVE

SSSSSYHSKSTPVASSSTLPLTPSDSSSSGYYASSSTPVETSSSYYTPSSSSESSSSSSL

YTASTSVETSSSTYYTPSSPEGSSSTYYVSTETIPVTTPATTTYGSASESSTETVTTPVQ

SSTEDVTSFTSVSESSSTGYYNTSTVPVDSSTSSKYYPPPVTTTILTETETETEIETETA

SETSVYTPSSSTPSGYDFGSTTATTTTSSGYGEKTSTSASNESSAEPVTQSSSAVSPSSS

SSSSSSSSSPSVPTTLQTTSKAYPGITTSTEEYPATTTSSGYGPPPSYGQPPSYGRRFRG

LRW

>SoG_01089.T1

MTALTTFALLALTTPFAAAHSWVERASRIAPNGTMVGPIGFARGYVPRDSKNPPFSDTIP

TNILPAAGSASYSGDEIINKFPPNPNPGFPLLEAAPGDHIAIMHLENGHTTQPQNQPNKP

RNRGTIFFYGTTQPKEKERLFDVHLVWNKDGTGGDKRGHLLGTRNYDDGQCYQPSNAQIS

MSRAAELAPEGAKHEIELACQSDLQLPKDLKPGSIYTIYWYWDWPDLNPKEIDMEKTADG

RYPWAGTFMRGQKDPNGFKPGAIAKNESYSSVIDVKIVAAEEGGFSGKVEAQGLKFIKNQ

NVYTKALEPQMKNNFQVKIEVPENSAPGPVNPPEQPQSSGKPSATAGPPNGGATVTVTKL

VTVPAPTVVKTVTVTQAPGASSTSTAVEIPGGVFVSSIFPGVPKQSASASASSGFSSVTS

RIPNTSTTSMVSPPPAETQTQTNTGGRPNVTPIPGLRARADWGFRWW

>SoG_01130.T1

MYSCYPTLLATMATLALRADALPLNINLGAYSPALVVGDGEISFAGGSDVNSLMNALEGA

AVDTAAGVAAQGGPAPANKAQKKEREEAKDIANILGSKEKVIEPRKVEIIPAKTAPVAKR

GIAGFDRALTYAEAALVKGPKVQLGTGGEGGSGVGIIVDNNPAAAARPGKGTKGEGAAGG

EKAAPPKATKAAVPEPALPSTPAAPAGEAKPTQPAKREIEPQRPQMQRRTKMTTMYIRSG

ISGDAALSGKQVAREVTPEITTKRDNTNSDVSKRDDSENALDSVNLNVPADGVTMTFVEE

VDNEEA

>SoG_01162.T1

MAHLLVSLGLAAGLAVALPQGNTNTIPPSPTGLDCTLHQDHWDCTTPCSTVTSTVLAIEG

TITSYPDWLESMNSKYSTDGVSFSGTRVFVHTTVVTGDFDPFTGYGVYDSNALTRAGYKV

VTGYLTTTKCPPKTTASIGPSPTGSVCSPHGDHWHCDPTSSSSSPPQQGGQCEAHGDHWH

CRDGVPQPTSPPGGSNGGNNGGGNGGSNGGQCEAHGDHWHCPEGVPKPSTPPPSNGGGGG

SSGGSGQCEAHGDHWHCPPGVPEPSSPPPAGGSSAGGTGSNGSGQCEAHGDHWHCPPGVP

EPSTPPPGSSGSSSPGGGSSGQCEAHGDHWHCPPGVKEPSTPPPAVVSPTKKPPAVVVTG

AAAAGFGFCGSGRALGMSVLAVVFALCTQRHSTQSHLVPHSHPLGPGCGSSSLGVKTSLQ

LSSLDRNPLASNGSMDFGVVSGATETRNALTTAARIIRIWNEAMFPPGQRLAPSPNGARQ

RRRSEMMAAACFPPLPPPPPPPPSTSPSPSSEDASGEVVSQDRQASRRHHAAPRQGHGGT

VPQTLGDAGVEELQPLQAVEALDGLQLPVQRLDGGRRPCEVVQDEGQRRRRGLGGRQDLP

GHLRAHLGLAVTVAAVVLDLHVLPEQLVHRGNRPVVVVPGGGGGGGGPLLRLLRRLPPEV

EQAVARPDAEGGAAEGTRLEPGEEPLGEAQAPDDEEVVLAYHTGEVFRRGDARLGPGHDE

EGVEQRDDAQVVHDVLGSPAAGLTEVVDHSRHVPLELGLPVPERPRRRGHHHRPSAWTSD

AGAGARHRPVVPAYPERADGPRRGVQVLPGEEGLHGLNVVDEHHVGGDAHDGPVPREQRV

RLERQPAVPPLPPDPEGGCRGETGAWDRGQGVQRGEVDCPEQLEDHEQA

>SoG_01241.T1

MMPLSLIFLAFTVKALAADGSCIYDAANPSPTDIGTGFCSSYITDHCATVTRLDPEKVIS

YCECLLTSATAPVSPVGNTTHAVETQAPATPTITASSSASLASSASGGTVSLVTSDSQTT

PSNLSSRSTASTVTNGNATIKTSVRGTQTEVTDASTVIGKPGNATSLTTPATQSSSTLSS

QTGQDGNGTTPVTPSISISSEVRPNDPAIPTNGTSAAAGTTASLTQNTSTPGRSTPSTLP

NSTYLANSSSPTPLPSETQTSFSYFTTSAQTTPSSSAVSNSTANAASLKSTTANTSIGGS

SVPANATATLISTPLTTPSDAGVSGTQTPVTAPGNATIATETPSPSSSFVSRSRPVRSSW

TWSGWNVTSTGSQSARPTVSGINATTAETPTTGAALSSSDDLSSTSGFISGTATTLTTRN

DTRTVSGSTSSRTFSWSFMSLTSSITGSATTVQISPATTFPTPATRLSSSSWANWNSTTP

PATPTTPVSAFTAANFTSVTRTAAATKETCYHLARDPQGEAPKRALMRNAKLREKNVSIP

VPYIESVNFESDGIEPLWLTVRDSTEGSHHLDISNKSQIAVVDSDGTSMKLDAHGIYFAT

KDCRYDISITISDLLGQLAELSGVECSARMAKRLFDVPFKKTLVLRDQCGNPVGRSIRSY

PRLSVGSSECTDVSVDEDTGTWKFDCTFPGSESSTLRCQAAIKNDIVDFLTTDPFGGSCP

DLPTVITTLSTTGKDLLSRGSLHRELFTNDLSYGEELEAARALDAYLQMWRVMQDVFAKD

EAGRSALETYLKLYSAYRDFPTDVCEDIHADELSLDLALQAGVSRFVLYTLNFAPEGSAA

QNVTVQDSSKEACCAPGLAGGGSDTCGDPAGGLIGSSGCVCGKTVGGESIAFEYTECENF

VATCETDKDCGDHNHSGFVCVTDTCCGGGVCVDPYACSDNGTQLVTYEPF

>SoG_01280.T1

MRFAAIYIAISLWALEGRAQSWDVPGVTIKCDNKTANPADVVSSRVNAQYLSKITTGEKP

VNGAGPGNCGQVACNWGSAIEWCNENTSPKTLDNFTVIAEAWDLMNSKLTSGAEDDCVIL

QLGALSVAGEIFHPDGFSVRIKGGVKC

>SoG_01281.T1

MKSILALSMSAGIAFGGAIPPVHQAAAAVPQDTGLGPFTFTSTYNLIATPDRVVNAESVA

APGEAGAMGFYNYGINSELDIICYNITLMGVTGPFDSPAVTATHIHEAAEGRAGPPRIAF

PNPEPADSGPEVIKRSFGCLQGPFTTGINGDNGSDTGTGFTLKQIEANPSGFFTDSHTAV

SVPGVVRAQLQAGGSSIGGGIGGAVPGALLPGHGTAPAIAGGCTVQVVAGGAPVTVICT

>SoG_01460.T1

MPSINTLAQVMAVLPAIVSAAVNTFDYNSISLREVGARNTLDWRIWLEKDGEPISFWHDI

PLYPDESNQQIINFYVEIPRWTDAKIETKRNEPLICDVTDQRTPTALDPIFHDDSKGEPR

FVESVWPHKTYPVLYGSVPQTWEDPNFKHALTGFPGDNDPVDLFDVSGIEEGYVGQVKQV

KILGALAMIDDDATDWKVMAIDVRDPVAELVNSVEDLEKHRPGLAKSMHDWYIYYKVARG

KPLNTIVGGAYVNASVAASVVHESHGFWTDMMTGKTQADKISREQTSNKDWRKTYVNKEV

ATKKFGIPKKSKVQEAKPKPERYQHWYYLDSEFKPIELKKSEKKLRRNGNRIEEC

>SoG_01481.T1

MKFNLLSLVAASARLCSAKWTNDSPEPIERVPAPWTLRGDIYGATFVPIGSLPNKAYSPL

ERDSLVASEGKFIGGLGMIQLIRYTESPVGPYDELIVLPGFFKYDGPTKERTNVRITRIY

VSQKYTCWNGRTNWNIPKHLARFDWSENSLGELTVKVYPHDTTDDPTEAKPAEKPWFQAT

WKLDLLGGLPFSTDLYKILGVNTTLAQPPLPQKESHYGELPGTNHWAATIPGQASKRASL

GFLDLRQGDGDVVEGQDTNIVGDEYYPNFWPGLLPLHPALKLENATITFSDPEIWG

>SoG_01536.T1

MRLVFVVTIAGIATTATSNLAPRIPSVAEIESAKSVEDFHDLAMRALEAQQDGLQRRGEQ

RSCTLENASVRRDWIIWTARERMSKGERKDYIRAVKCLWDLPPVDTEFSAAQNYYDEFVA

IHANLTDFVHGTANFFTWHRYLIALWEETLRGRCGYKSALPYWNWYKYQSALHLSPVFDG

SDTSFGSTGEYFPHNGSLVGNRTVHLPPGEGGGCIKDGPFVGYTINIGPIRPAMNGYEPW

VKDQRDYNPRCQRRDLVHAASERYTFDGLHDLLAGDHSHSIELMQDEFQAFGTLSQHGAG

HYAMGGDGSDVFTSLNDPAFYLHHAMVDKLYWMWQALHPAQADTVAGTLTYRNLVPSRNG

TVEDELDVGILGPRLKIKDTFDTLGGTPYCYIYE

>SoG_01541.T1

MGLISISVLVASVLYVLVRPPPWMPLWFVQWRQGHLPALAPRGDASGPQSTKKKDRSIDV

SAESTEKPVNKEKETLANDDTTTIGKPRKDDTSLMPPPPVPFLNTTPMLVEPGEETTPKA

EAAVPSLAVPQLSLSGPVSSPSNSMMPPPPRPSATTSASSSTPNPGRIPSLGQFPAANSA

QRARGPAPLRGPPSTSSGLTPPPTITSKPQKPSRKVLLEPGHSPLDWASISGPNADLRGV

PPETPYLRVTPSMLKEKTGRKGKDAWMAINGKVYNVTPYAKFHPGGVPELMRGAGRDGTK

LFWEIHPWVNYDTMLAACLVGLMVDEPEGSGPSKMDEMD

>SoG_01548.T1

MHSFTTIVAAIAGCSAIVNGAALPPAVQTTTTLHTVTLETRDVAEVSGNSISKREPITVA

ILTAAGTAAATAIVNEAVKAAVKFIGDISNFDSGREAFTKQTTDTMMARNPDPERFQAAA

CYNKDFSVADPANIDGQSSVEFRLGLLHTDYECMYIAAPNQFYTEGDGGFINLSFTHTDR

CTFDQETADLTCI

>SoG_01670.T1

MKVQAVSALLAAGTLVSSAALPPRSIKQCDNPEKRIEWRELKPEDQQGYIDAVHCLKTKP

SRIGLEHALYDDFSHVHFELNTFTK

>SoG_01696.T1

MKAALFLSLVASAISFVAASRLQVDVTVPVECERRTKNGDKVSMHYHGKLASNGQKFDAS

YDRNQPFSFKLGSGQVIKGWDQGLLDMCIGEKRTLTIPPELGYGKRAIGPIPADSTLIFE

TELIAIAGVPQPEKISYRVTESTENVVPETSEAAEKVKENVVEKVASVVGEAAEAAKTFI

ADTDDMQGHEEL

>SoG_01723.T1

MPTALRMTFLGFVGLHGSLATEFHHTQHDQSYRPPRLQETSPLIHINNKNGTTIRLQARA

PLEQALMAREFLGELKLMFHFDIVPRHGGPIAVGLQQYEGHELSHTNPAVANGVLFAAGA

SDVATTPPAPRQGTFQYLGTAESSRLTSTGPSVSQVGNKRKVPGSNALEPTPKRLRSASR

PPSNESCSGVNALLNLDIHLMGNLNAIDGVREMAQELVEDRAFEDWVRDGRISEMALAQE

RNKRAVHLRMIVEKTRHWEGSSIHTPGGQEVPATTVEHTERQPTSQQALAGNRNQINPPS

SQLPPPPLQNQVTHLRHHQLPGRLTAEEEDQVQAQQLLYREQPEANRPILSSTNHPQYQS

NSQMTQNYQLHEGTSTSWSLPSTQLHQPTSQSQQRCQFVQNTFHLHSNPESGPTTVADTP

SPEFLVAPQQYVPGQGSYTVSAIPTDCQTGGVNVQAQSAQPSLPSDNRGSFGISVVVPHV

TNPPATLQNQHTNAGLSSESLSSVIPRTEYPSDTWSWASLAPGMHLAQDMSPIREPEQPA

NARFYQYVSSYAAMKTLKPQAGLERVEFDMEDHVMNALVTNQFRQGLERCQFREDSRRIR

LRLCRYPDNVEDFENIGAPVAATYWPENFYVSLGKCPLQLPRKQHFRTDLPVELTGLVRR

GLNTVVISLPKSPKPCQGSQKYLIMIEIVTVSSAGSLKGQICSYQHFSYEQTKRILQSRV

ACSRKDDDDVVVQDKHLSVSVADPFSSTLCTTPVRSIHCRHVECFDLHNWLESRPTKPSK

RAGEPSRVDEWKCPICGGDARPLQLRVDDFFVGVRERLIANGLVHTKMIQVDEEGDWAAV

VEHEISEDDENSMTEAGDASLRTSRPGSKAMPSVIVLEDGA

>SoG_01740.T1

MMYKSLPLYGAVMALCAAQVSAHAAINPVLGVQGDARRSDVQRPRGNNPCGNVNIAGNID

SSNSVTADASGAFTLNIENFNGGRDGSRQVTMSINADGTGGNFVDGQVTTNGEAAPAGTG

TEQITASLPAGTTCTGGASGNKCLAAFKTLGGFGNCVVIQQGGGNGNGGGNANGGNANGG

GNANGGNANGGNANGGNANGGGNGNNRGGNGGGNGGGNRGGNRGGNRGGNANGAGNANKG

ANANNGGNSNNGGNGNNRGNNRGGNGASNANGGNANGEVATEVATEVAAEVATKEHTENG

WATWWE

>SoG_01747.T1

MKFAIFALLATSVSSVYAVDCFCISTDDDPAAGRQPQRKFNLSGDCEARGGRVDVDARTC

ILLPASGAFTDELCQRESGGRILVANCVQSVF

>SoG_01783.T1

MLLTSELSAALVLAATASATKANAHNYSYNWAGGLIEEPGKVVEVCGQIVVPECSGPYLN

SISGIGGWVGIDGGTNCDTVVLQTGFSCQVVNSDGAQNITYAAWTEWYPAQSVPYDNFDV

QSGDLLSLKVTAKSKTAGSTSVYNHRTGQKKETHYDNQPSLCLDYAEWIVESEFVEEHGI

VGGELTANFTPIHFEQSSYRTSDHKKVLVGADDLLNMVQNGNTTIAVATFHSPGDFFVDF

VQPASWHPWQ

>SoG_01784.T1

MKLLHLLLGASISISTAEAVENPHVRARNLVAAKRPRSAPAPAAPRGLRPSQAIPSFLTN

KTKEFVVDGKNIPQVPFDIGESYAGILPIQKGGSKTDPNQLYFWFFPSDNPAAEKEIVIW

LNGGPGCSSLEGLLQENGPFLWQAGTYAPAPNPYSWTNLTNVIYIDQPLGTGFSPSTPDA

PLEITNETVVATQFMGFWQNFIDTFDMKGYEVYLTGESYAGMYIPYIAYNMLEKNDTHYF

NVKGIQINDPVIGHGHVQTTVPAVMHLNAYSNVFNLNETFMKEMNERADSCGFTSWMEKA

LTFPPTGPLSVPAKAEEPGCSVWGDIINAATLVNPCFNIYHLLDFCPFPWDVMGLTPSAG

PDNFFNNSDVQKAINAPPTNYVECGDPNLNLQDDTSPPSSFGPLPTVIERTSNVIIGHGM

YDYLLLVNGTLATIQNMTWGGKQGFQDRPKDDFFVPYSSILAQFLDQSQQQSFPEPPVGI

VGGAGFMGVTHTERGLTFVTVNNAGHEIPLYNPGAAYRQLEFLLGRIKSLTQQGDFTTET

GNFT

>SoG_01803.T1

MATSKRLVLLIGSGGVGTIAALNLEVGGRAEVHAVLRSNFSKVSESGFRIQSCDHGTIEG

WKPSKGQKDLLNLTMALFVPSSSVRRYDYIVITTKDIPERLPRVADTIAPAVSPGHTAIV

LLQNGLNIEKIYQERFPTNICLSGISMIGSHEIEPGVIKHDFNDDILLGPFRNPALDEKA

EKAAAEDFVSIYAAGKKTQCVLDDDVPLHRWRKLVYNACLNSTCALTNLDTGRLRLAYDS

VESIVRPAMEEIRQAAAAAGINLPADIADFMINIDPLDMYLPPSMLDDVRKVRKDLPVPH

LGLVKFPHSLAMLRWTLGNG

>SoG_01812.T1

MKISLLFTLLLTSRPAFANERGGCHGDNCANHVTGTFGGIGVSYQSRRVLCQSYMRATVK

LAPTTVTHFVTKYTTKMPQTTESARNSLGRSDNATGTTAANETILAEAVPTFAALECPSA

GQFSSACACWSGITASTTTLPRATATATATTYLNVACSPAAMSVSKARQFPCSRQWGMCS

CLRSEEDGDVCVRIGDFGGYGNGAGPCEKSKECNASGGCDDPATMAAPRGCDYQGLPMDG

SQRQ

>SoG_01826.T1

MKFLGATLALCGFSATAIGAAVPVVNNEVQTDYDAVIVGGGPAGLSALSGLARVRRRVLL

IDSGHYRNGQTRHAHDVLGSDGFKGVTPAWYRYSARKQIAHYNTVAMINGTVTKIEPGNN

NTYFTVMAQVPGDKAEVKTMTATKIVLATGLRDDLPSTPGVQDNFGKGIYWCPWCDGHEH

ADQGLGLLGPLKSVPSSVREILTLNKDVVAFTNGTDTPENQAMVEKSNPDYKTYLQLNNV

TIDDRVITSIERLQDGSEHPADPSLPTAPEHDLFRLNFAQGPPVERNAFLISAPSSQASQ

LGPDLGVQLWGGKLGVDAEKGMLTNVFGVYAVGDANSDNSTNIPHALYSGKRAAVYLHVQ

ISRESEAAQLKTYKAEHKIDKRSVDEEARDLWPRMNGEPDELLYAGEFDQ

>SoG_01842.T1

MRSSLWSTAVLLAACVNAKGNVKNIILDTDLFSDVDDVGALLLATTLPTSNLLAVNINVP

STYSALAASALLGYYGQKNTPLGIARPLNNRTFFDSWAYDLGEYASKVAYHFRDSARLEW

QDGWGLGQLEGVVEKGTEDAVELYRKVLAEAEDGSVTICSIGFFNNLSGLLNSTADQHSP

LDGPSLVAAKVTELVIMGGQYPSGREYNFFGDNPYHTAHVVNTWSGRMTFSGAELGGQVF

SGKSLVRNGPKDDPAKRAYFWYNYGKARESWDPLTVVYALEGEGCLFELEGKGGRNHVFA

NGSNEWQWGPDEGPGEQRWLKLNVSNDKAGELLDDMYLRGAWSHTKTTVESCKERDRLEL

>SoG_01857.T1

MIASITALIAASATLVSGACLRRATGVSITPHDQYSSSIGVVGCKIDTNRVAYWPGSIDC

NNLCVKVSHQGRSVHLLKIDTSGGAHDISYDAWNYLGFGKSATEDPQMGGGVDAVYENVP

MDQCKDLLDDGKLPLTAANSMNYLSACLAEPDSWVAKNYALYNIMDPVCSSGHDEKCTLD

LSVSTQPSCPSGLGMPGSLKAKVVNIAYGTGKKVDA

>SoG_01869.T1

MKLSLAALAAVLGVAAADKAIVINSCPETIYVQSWPYDGSSPGPLTTLSPGQTFSENFRS

SGSTVKIAKTRTLDKPLFFGYSFSKNPDYAYCKDPPSPSPGVHTHAADELSNEWGNPFSN

ARNTLSPGAGCQSFNCAPNDANCYSRPGAKKVYGCPQPVDLTATICKK

>SoG_01876.T1

MKAFIASLILSTFATASPLLNGELSRVVRFTSDKSKLDMSSVPFGFTSTYAIKATPDQVV

DNENKFTGGLEGACGWFLYGINSDENVICFNITVTGFRGDFQSPAKTATHIHEAKRGRAG

PPRIAFPNPMGENGHINTIGCLRGPFETGVVNNGKDTGEGFHVRQIEEDPAMFFTDIHSS

EAVPGAVRGQLGPDQC

>SoG_01921.T1

MHSLSTAIATALAFLSAVSALPAANTGPSKASNAPSSCESPAPPTQPGCTPGRVVAPDLI

ALSDLTPDLAVSQPSGNGLSLSLGPEGTNQQQIALFKGIPSTAKDCTFGWKQRKLNTQDT

FDTDGNALVEYRTLPVDGGPYSFAQVTDLAAQNDSPSMIDFTSWDQPGFAADEFHVGGII

SCAERIHMHLRVSKLNGDVGGVRMQGVPAAGSNSELQGVFVEWKC

>SoG_01940.T1

MKVFAILSLAAMAMAGAVEMRDGCHGNNCNRAVTGTGGHLLPVETRKADCSSFQRTVVTP

DAVTTTVTVTVDPDEPPKFKRNLQPLEERDQKAIPTYVKNCKSPEDYAAACSCWGITAVT

STAPVPTSTVTSTVTQDYCEDL

>SoG_02027.T1

MKLTLLLFASAGFAATIPLKRQSPCFVVGNQVLPASTLKAAEALAKTATCSSGAGGRTTL

SGVPDVTVGGVSFSDIDFSKSGQSPLQFALTRFAGARPLADSDLQLFKNELDVYTATEVG

VRSVGGNLAIKVPKFFLEFQVSRIETAQGNPPTEPGRQVDHLLGKVLNNAAKENPALLDQ

VRALAANIA

>SoG_02030.T1

MKASIFIAATSAMLAMASPVGLHKRAMLTEWEVEVVTVTVTAGPAPSGAVFLENVAPKPK

PQPAPKPSAAPPAPAPAPAQPKPEPAPQPAPSQAPPPEVKKPEPQPEPQPEPQPQPQPET

QPQPQPEAKPDTQRQPSNGGGQAEVGGDYKSTCLKQHNIHRMNHSAPALQWDDQLATWAK

QLADTCVFEHDTSMGTMGQNLASWGSTTNIDDEALKSAAGAITNQWYNGEMQSFDGVYGM

ANPPSNLPLTAFGHFTQVVWKDTQKVGCATVKCAAGTVLGMQSWYTVCNYSPPGNFGGRY

GTNVLKPQGQPTVTI

>SoG_02032.T1

MSSQVLYLLALSGLASADGTGILGAAPPMQAAAERYASLRHVFLGPARQQRVLPRRRLVP

EDDGAVHRGVLRREAARRVGIGDRGVVGGTPGDGDFGGLEPGDEAGGHVRRGAEVGGHGS

EGEGGSGRWRVAEGRGGGVVE

>SoG_02035.T1

MKLFATNSLAIAAAVLLLLSDSIGIANATPKAPWAKQRAKGQARRALSAAMEKREVNATC

AEREALLTTAPKTNIWGGLSGEEAAGVVQWLFAQPELNLTKMEDAGSWDNTLLLVELQHP

NKTEALAYIDGSGAEPVRYARAVIDHKASEEPYIQDILVGPVPVVNGTTTWTPLEYTHTR

KTEGKVRSLEADSKAYQAWVTEVSTSVADITMDLWNASALGLDNDTLAVWGIDPYYQDEG

RVKRWDTFWGMPTGVFEDMTLLPMGLFFMSDVTGRDPSKWTVEGWYYNGVFYETTQDFRD

AYWGGKVEKLPGNMEGPWAQTDQQGTIPPLDTTAPPVMVAPGGARYHVDPQSKYVEWMGW

SFYVGFQRDNGMSLFDIRYKGQRIVYELGLMEALAHYAGSDPVQSHVAYLDTYYGFGPFA

FELLKGYDCPAYATYLNSSFYVDETTHTHLNSICLFEGDADYPMARHSSSAYVSATKNIY

FTVRSVSTVGNYDYMFSYTFFMDGSLAIEVRASGYIQSAYYAKNEDYGFHIHDALSGSMH

DHVLLYKVDVDVLGTNNTVQFMDQVATSTTYPWSKGKVFNTMKLERSFLVSEDQGRFNWM

ENNQRQVIVVNRDERNSFGEHRGYRILPYTGASHLAVKNSSVLKNSARWAEHDMMVSVRK

DSEPRGTHAFNNQDTENPPVDFSKFFDGESLNQTDLVLWVNLGMHHVPHTGDLPNTVFTT

AHSGIQLMPSNYFTGAVNAETVNMVRVNYDKGVVSDVVSFGASGEECAVAYEPVSVDLWN

YKGDVVVRKLPYSPSDPYYETDSI

>SoG_02043.T1

MRLLLVASLFAASALAQKACGFPNGPDCKSLGKGASGLEQFADDGCCLLPFRCGNGDGGE

RCERVSGGGGNNNNNNNNPGNGNGNGNGNGGQKPPQNSGGNQGADPNCGFPNGPDCKSLG

RGANGLERFADDGCCLLPFRCGNGDGGERCERVAVGKGKSKRLRRSLRV

>SoG_02132.T1

MRVTALASVFVAALGGAHAVAIPEAVPAAQPDRTLSARQTYTSTDPKWTDHDQFRNSLIN

RHNQYRGEHGVGGLTWNRTLSGFAAQYLNKKGNGLNQCPDFAHSGGPYGENLAIGYGTPT

QAEVAWGEERKQYDFNKPGFSSATGHFTQMVWKDTTQLGCARKFCTSGNPYRGWYLVCEY

YPRGNIIGQFDKQVLRGSYRQKRDEELVPREEDEGEEAIEEVAPPEGVEELSGEPLVFFQ

AA

>SoG_02145.T1

MKLIAIATAVLLAMPTLAEETAKSAAKPAAIPAAKPKGVYRKGKNQNSDAQKFDLIYANS

ESDKGPDTYVKVDEALALETVNVDAQKKPRPHCQHCPQPCQESACKELTCSPTSKPNCAK

PQCSPQRRPHHPHKPYYPTGPVKDLLTDCFLCKKLNVFADELSYVAEIVMHLKCRDGKPV

WKAIYAIEHQLDELDKLIDKAPLDKCFDCRQEAKIACCYKTYAEALIQLLAVIAEKAEYL

PGDVDKYLVTATNSLRSADYAFVYELARRMQCDQYIKEIMKKQGALDGSTPGSVRAAFAK

IVTTPYITGDNFEGWSKDGKKKERKRRDSDLAEAELAALKKLLGGPDKKVSEEELDTFKE

VVEESVEAAMDIMDDLEIEDFEESDKSQELKKFEVPEEHDGSKRRDKVKTSSA

>SoG_02166.T1

MQFLPTMLFLLGPMLAEASPLLTSIGNGDLARGFSLVVPMRSSVKSDFVKDFISARVRWG

GKVSDGLHSTFKLGDSADLEVVDGRVNVEPLGTDDIYIADVKVGDPPQTLKLALDTGSSD

LWVQSSDTIYRTNEEGPWAPQYMPNKSHTSHLIKGSEWDVQYVDGTGANGIVYTDTVQMG

PFKIENATIQSAQVIASRFETEPGLTGVLGLAKSLPNNIYPPRPTFLEMLRKQLDKPVFT

VDVRSNATGRFDFGHINTDLASDNITWLESDPDSVHWDVEFDLTSWTSGKKVWWLHKFMA

TIDTGTTLMFIPDTLASMYWFSVPGMKTDPRLSDAFTFLCSMADDLPDLRFKLQGTEHVI

TIPGKYMNYGPIEDTPGYCWGGLQSAVGLDVSILGDTMLKALFLAFDLEKGRVGFANKDL

>SoG_02206.T1

MIAKSLLVLAVAALAAATPINNSGRIQRRCNGPKPGGPGGPGGPGGPGGPAPQPVLPKNG

DFQELPAPPPGARLKHIALGYGIQNYTCESADASPVATGALAGLYDMTPFYPNPSDPKSL

DQATFDRLTAMVIESADLNIPLNLVKSLPDRAPNSAPGADLASPFLPGDAALPIPGMNGL

ELKHIGRLVHHVRQARRLQRSQELRCRCQG

>SoG_02220.T1

MLRPTALLALLSSLAAAWPSPQEENVEEQSSKYFHEMSGSQELLHYDARYFAGQEVPYTR

HRYVLRGLITSYLDFFERQGYETWIAHGTLLGWWWNGHIMPWDLDLDVQVTANTMEYMAE

NWNGTEHAWNYTIPHEHGKHNENVQTTYLFDINPHYNDAKVDSMNLIDARWIDMSTGMYV

DLTVLREREPISRPGIWSCTNNHRYFSSDLWPLRVTEFEGTRALIPQEFERILAQEYGAS

GLIREEFHELIDICQTADIFFHRHYWNHDIKQWVMNNVEEKETRAADAAAQKQREMNGLH

PDDEEPHQHDHSSGEEGQDHGHDHDHSHKEEVQAHDDGHSHAPSVPSPASDDH

>SoG_02251.T1

MRFTTFAIAAFTAFASVNAQLSPGGVVGAIEEITDISSNTNEILQDLKAGNIFSAIPRAI

AGLNNIVQTATKDVAMLASAGNVKFPEDGQKDVCKAFTGFVKVHQNLLETVIGTKGLLSN

TPFTALIAAALRGLEGIVDKLAFSIIGLVPSCAEQAKMDAKSLTDTLDKAIKAYSFKFPF

GQKV

>SoG_02254.T1

MWFNSFHLAAAVFGVFTSAAADAGPRVPKAKNFIYVVPDGYGIASQVMARDYQSIMNGRG

TVERPNTAKIGVDNLVLGTVRTQASDDLVTDSSASGTAFACGIKTYNGAVSVDDDGMPVG

SILEAAHLEGLKTGLVVTSRITHATPAVYSSHVLSRNSENEIAAQQIGKAHPFGPFVDLL

MGGGRRHYLPKSKGGTREDNVDLIEWATAQGYTYANDAADLKEVAQSGTVPLPFLGLFNM

THISYDMDRDPESEPSLLETTKIALDTLDAATRKSKKGYFIMVEASRIDHAGHANDAPAH

IHETLMFNEVMNYIREYIEGHPDTQMLSAADHECGGLTLVSGFDPTVLARAQSSNEHLTA

AWDRYSGGDRLGFLKTEIVPAYGLGNYTDTDLQGFLDVYTSGGTAKMGLAILRAVAKEAG

VNWSTGGHTAADVVLHGYAKGKKYEEMKRAIGGNQNNIDLPVYVEGALGLSMEEATKALR

KDGVAWVQKRDQLSTIKRRAQAAAMDHAH

>SoG_02271.T1

MALRSLLAVGFASLALAHSGHQNSKAPQVDPNASWMAKHMAVPLEEHHLDNWDEGSFFAL

HDYNSDQEWSRDEILRTYGLMDESNKGVSHERREEIVRQILDLLDKNEDGFISREEWREF

MKTGQTLPDVGTGPGHHGDDEYEYEIHHWEKYHDENTKLEDLTHPEDIEHFRKHEEMERN

QEKLEQMEKMDIIVENIPLKFRRIKPQ

>SoG_02300.T1

MKTFTFIVTLFAAGSAVLAAPTMPQGNDKREMNFGGLTGPLGGAVGANPLGNSGQNAVAN

LFKLGDEILNIPGDVLRKMLAGDPAGAVSGLVQNIGKAASDVPKDAMSIVAPLAPAPAAG

GKNPAPAAGGKN

>SoG_02329.T1

MVKVTTSGMTLLAFVAGAQAMAIPKPGTTAGDMDKEPTPTPSAASHTMTAAERSTVTLGP

YENRPSPTGPPETTVFVAMDKAGNFMTMPSNQWLTKFGSALPEQMDLLMKLQAKKKESES

VQRRETMQSNGAAMSNEGRGDIEPREPAGSLVEKRMISKPQEEEDAKKNKNTIGKIAKIL

SKNQTLADKVMVLAEKPKRSLFDYAEDKDLILEETTEEDASPFAKMSAPSPQSDKAFYHH

FFEESAKDNISPEAREEKLKERNQEVKKEEEEVAAAEEQRVPSQGYNSSPSKGGDDDDGD

QDNRLRVVCCGLENGDEDAPCCRDSDYDGHCGWYLD

>SoG_02357.T1

MKFSNILLACGLTAVASAKGKDGFKFERLDKNDSVSLPPWIEQGYPNAYAPAQMLLVVDH

QVGLYQLVHDFDPVVYRHNLLAHAAIGKVFDIPVVLTTSAEVGPNGPLPKEILEMYPNAP

LIKRQGEVNAWDRQEFRDAVRAANKSQIILSGIVTDVCTTFLALSLIEEGYSVFANVEAS

GTTTPLVRDVSNDRMADAGVQIVSFFSIVMDLMRDWRNTPGAAELIPFLDRWYPSYSYVA

RAHLAAVEGGQIQPGQNETSQCY

>SoG_02395.T1

MRSRYLTIALVVGAVGFPTSGAVEDSGDIIHDSTITKIDGSTGTRSNYAEVTDTSQVEER

STWLKGWEWNIVKEKKRSPDVASDLNWELRWDKEKWVRTRRDMIQVMQGDYLDP

>SoG_02409.T1

MKTPIWLQVLLVLGATTVYALPVDSSTASSNIAAPTSDSEPMVPFDCNSYAYAAVGGMIS

EVDLTTGKTVRIVGNANGIGPDVYHAQSKSATPMPIKGLAYNPKDNLLYGFILGNEGPKG

NSQLIQIDKNGKYKKIGNAHPDIFGPGDFDSQGNLWIASKMGDFWAKYDLVPNSPTWGKQ

VASGQIAPPPYGIFVNDWAYTPNSGEYMWAMGLSSGGLGERRTKLFGFSLHTQDWREMHD

YGVTFPNEGSWSAAFTNGNEVVINDIMSKNTWRYNPTDFKQPLKMMGQAEPLAFPNIKGA

RCMYAPKV

>SoG_02445.T1

MARPAVIIAALTILCSAIAAPTSAPGSHQRTDDLVVLDGDSLRFDPLYNIGDAKKDPEWQ

LEYMESLLTFETDQGLAERSELLEKRVGNGALCFECMVGTLKLITSDIGTRTFAKGVCTP

WCASLALSTIIARASDYAAELGWDWGNGPQTENKYTWQHAARV

>SoG_02446.T1

MKVAMIALSAMALAPCLVGAVATDIFSACQGPNNSGHKEGSGCKWLNGVSDSDGTRSGKC

YAHADIGNGLYCSDHEFSEP

>SoG_02447.T1

MFFFKIAALVATSSLLMQQVGATATNPIAACQVPNNGGKHRGDSCAFYSGPSTTSSIRNA

HCQQGPDGLYYAMSFSEDADANKNTHGDLITIINALPREKSPLQALSQKVEDFMSCGPDA

AFAAFQNLREAITTSSYRVKGRHIEIPVTSDMGLLSRVFSSNLNATTIWRTPTMASTLVL

LFACPSDAQTIHELATDQGRTFGGTPSIDSVRATPSASLLDAYQRAKSAAEMGGQTAVMV

VTLHDVYSLESDEREDVGNYFPFSHFFTICVGPPGVKIWQAGGHNGYEFGQYLREGHARL

RDWDEAERFASDFDALARHEGKWTEHINKLYQRLFLINVDQTCGEGGPEPPVSPEFRAWV

RIRTFENVSYEDVTNQESESVSLTSSL

>SoG_02449.T1

MLPLNYLSLLLAVPSLFASPALARKHVKYADTVFRNGSIYSLDKRSSKHQAMAVKDGVIS

FLGTDKCVKAFIGPKTNVFDLEGRMTMPGLIDAHMHLLAGGASLLKCNLNYQPLSLDEVL

KHIQGCLDAETKPDDSWLEVVNMDWYHLNDVTGGVTKKELDTLKTNRPVLVHSADFHSHW

INSAALKASDITASTPNPPGGVVERLPGGKEPSGVIQDAAIGLLAGPAPPTAAEDIEHGE

AALRLLREEGVTTFQEALSNDRTGAAFVAIREKGRLTARGFFDWGIDAPNSTAQVKPVIQ

EVQAAAKKWNDPSKIGPKPSLKLQAVKIMTDGVILYPATTGALIEPYFVPMDNGTVWEPD

ARRHPAPYWSTEILAATLMELIPNGFDAQLHVDGDMAVRSALNAVEAFRKKFGSKYDYRL

GFAHNEVTDPSDWPRFKELKIDPIMSFQWSQASSVWIPNTLKAMGPVRSNYLEAWGEIAK

FGTRIVYGSDWPIDPLDEWLAVKAGVTRSGDPTNPNSPASLGAPYNGPGLPGLSLTREQA

IRAITIEPARFLRADKHVGSLEVGKLADVIVLKANYFKVPDDEIARQKALLTMVGGEVLY

IADGVDFKNGVKAKFPNDDKLGKKLHKKNVGGIQGRSLSPEGHQAVRRLSVRKVCDHGTR

DLHQNIRRDFHHELR

>SoG_02517.T1

MKASILAPLLALATPSLAQQFGEPTFEFLYTVNATLGERWPIGDMGFGSRVVIPITGGTF

KGPKLCGTVNNLGADWGVTDTKGVFFPDTRYNLRTDDGADIYIQTSGPTQPDGRTLLRGK

FETGDERYDWLNYVIATGVLSRPTGQETGNYVIIDMWQASLVYPLIMNAHNHSWD

>SoG_02530.T1

MRYAFITSALIAGASAHGLVTSIEGANGVTMPGLSVADGTPRDCSSNGCGSQADTAIIRN

REMGSGRASALGRTQGNGPVNAAAVIAAFMGNAKGAAPTNNGTEGATGVEDDLSALQKAR

AQRREEHKRQVGNLFGGLLGGGNRNGAAGNRAAAGNRGGAGNRGGGRAGAVGLGGLLGGG

GNRNNRGPETMIADTTGMGSAKGLPTASDSGEVSMVFRQINQDGAGPLTADIDGTSGGTD

PNAFRRARVTQDVPGLGIQGLSLATNTDFPLKVQMPQGMTCDASVGGANNVCIVRVRNGA

AAGPFGGSAAFTQSKAARKRAIAYRLKKRMELDREELDSEELDLD

>SoG_02559.T1

MSGYIIYFVLALAAVARAQSSSIPPITWVKCPCGTPTGVDCGKIDVPLTYKSGSSIEPAD

DRTVTLRLTRHRAAGNTTLRGPLFVNPGGPGAPARPGVEATGEFRQEVRDMYDFVGLDPR

GVGTSTPVRCDPKIFNRRLRTLVDSEQSYEALKQYGKELGESCAALTGPLINYLDTIHVA

KDHEVVRRALGAKQFDFLGLSYGSMIGTTYLELFPEAAGRMVFDGIVDHSQSEISTLFAE

ATTFEATLNEFFRQCDSQDACPLKIGTNNSKATFEALIRDTRSSPIPAPDCTDTCRSNVT

FQELMTSVQGGLIAAEAWGDLATNLALAAAGNASALSVRLATSETPDPRNESPYASIAIG

CQDWKHSAGSWQDLAQRLAVVEPFTPLTRGVTQSWYYQAACIGWPTRTTFPQQSLDKAMV

ERAPPVLLVNSVYDPSTSIVWANGVRDQLPMAVSVTRNGSGHTSHGLDGETRDAMDFFFA

TGQLPPDGSVYQS

>SoG_02688.T1

MARFSSVTAMLVLAVSASAVSVNADFVNRRNHIYGHCAELDIPISATVPGAIYNIPRVGN

DIEAGAWAIYDATRSTPHDARNIIKNITISDTWSIHAQLCIPSHQGRQDTLHIATHGVHY

DSRYWDSQYKPENHSYVEAVMRAGYSILTYDRLGVGKSDHPDAYEIVQAPLELEILRQLT

LMARDGRLYSYAKATNDQEFKGLGKPDKVIHVGHSFGSVLTSAFIARYGELSDGAIITGY

VFTPTLASSGSVAFAVEYAATGNPRFDRPSGYVVARRSGIQNIFFAGDPKTAFTPELLEY

GISIKQPVPIGEFASAYSILNLTGPSFVAPVQYLLPEQDFYVCRGDCNGITTKEELYRNY

PNATDIDLVLHPNTGHALPLHNNATAGFQLSFDFLKKHGL

>SoG_02835.T1

MIPRLGTFTSTAVMASVSVCLLYDHYEPAFSPTPRNNDITTTPSEKKVHRCPSCGKEFSR

QDHLRRHSLSRKLLNLIIKDLLGTVCMTDSGHTLSVTDTGGKPYSCLFCRQDFARSDRLR

DHYADCPARGDREIPKKTQRGRRRRACAACTASKLRCDGGTPCSCCRRRNLKCDTSRLNQ

PERMDAATGATDLQQADEPMVVHDSPAVSEASDASSIKALIDGGTRRFIEGFNLPRVSDV

EDRENSASMPSQTPEGNLPALPHFEHHVEMDFQIPDMLDFPAHVADLWAGPPGHELPYFA

ENGSGHPGWSGHFAAPELFQASDLESLDLMHDHKPDSLQVALLKDMMLRKAREVHPDPTR

FDEVSGIVQSMFCQAKVDAFVRLFFLNFYPNCPIFHVPTFDADAITPELLLAIFCFGAMY

AHDGTDKLVADRLLHIAEVLIFSTEMFAASHGELPAQQQPKEGDADPDHDADLEWKRFEQ

IQAGYLIVTVQYWAGARSSKAHVMESRFCEVIKAARKTGLTHVRRVPQDRISEQRWIRKE

IRLRTMCMIRSLDCAFLFYSNYPCRLTFGELNVDLPCESSVFNSEHPFAEEDFRFSRSGN

LASVFDELFSEHTPLPGAADKQVEESPHDPGSKSAQPDARKTQTVLDLFLSIHLLYVFAH

THTQLRRFIPRSAQQKSADVKPGQAASVDAYDEITASVQNALKRWHALCVAVRCNSDDAA

VSNSGLYKLSYNFWLIIHLFVDKGKSIDLAKTIDVQGEDKLILFKSLVQ

>SoG_02870.T1

MRPSIASLLLHLLSTALAVTPLTDDDLRNLPSPSDEDFNIKNGRLLAPILIPRVPGTEGN

RRVQKHFVDFFADQLPSWDLIWQNSTSTTPRTGSDQVPFSNLIYRRDPPWAKEGDVSRLT

LVAHFDSKLEPKDFIGATDSAVPCAILMHVARAIDAALTEKWKKMQEDGEADSMLESPEG

VQILFLDGEEAFASWTDTDSTYGSRSLAEQWENQFHPSLSTYSNPLQSISLFVLLDLLGA

ANPVIPSYFLTTHWAYKNMASVETRMRALSLLESKPRRHFLPESDKKASHFGRSGVEDDH

IPFMRRGVDVLHMIPSPFPSQWHNMDDDGEHLDMATVRDWTRIVTGFVVEWMDLREQMPK

RVVRRDQGEESGTEGASSMRTEL

>SoG_02885.T1

MAALMNAIGLASGGLGIVQFLTSLLPSAPGPQGAAIKVKVGNTGQANDPGLGGEIFKAYA

WDSKNQYLGAGDGGYSRAGGVIDIVIDTFIPGVQSAYIGLAAGGDAVCIAWVTVKMEDES

YTGAWTGDIGKACPYDQWFMQDELAGEYTDADGVKHEYRPHCTWLGDDPNAPDVAAMKFR

TLAYGKDVPSTLENDLACSSTIFAMDKGPIADVPTKRSVRAPQKRERLSWMKDTLVMSNH

TTQHAEELCDSETSWGPDFVSYDGKYCDMETKTLYTLCAYENVDGCLDFNDNTNEVTKRS

MAAGLGGRAEKRSFKTFGSVRHWG

>SoG_02900.T1

MRFTSAFIITLASVASAWPIANEKRTGGSAYCIVPNACGACLVACMGVAQPIKEINTSEF

QNMVDIVKAEAVKGLELV

>SoG_02952.T1

MKFSAVATILSATSVFAAPAPENNAGAVSMMAASPQWTIQNMKRVCNGANTQCDWTFGIY

PGSGQATACKYTVKGKDASTANGGPSNCGDYTITSGWSGQFGPGNGFTTLSVVNNKSRQI

VWPAYTDKQLANGQVVKPNQSYAPASLP

>SoG_03017.T1

MPHSGVLCVFAIHAQLALDHPLPQLDHGPDNNEDDADAGRDANHVRGHVATSADVLEVQE

RVDVAPGASVVDERQPQVQQQDDNQPEQVDPGGGVGPGHEDLEQGEARVHGVLADVPPGV

EPQREPGAAVQDAPVDDGDDEGEGHRGGVEQGVQRLQRSREPVEQRPASRRIRAGVDRRD

EEVEGQAPVRQHGEEGEGVAC

>SoG_03045.T1

MRLSPSFLSAFLALSSAVVSAAPSAFDTSRFKPQDIIKRDVAIIGGGSSGTYTAISLKDA

GKSVIVVEKKWRMGGHANTYTDPDTGTAVDLGIVIFHNITEVRDFFQRFNLPLSGSRSFY

PSDLYFDFRTGKRVIPSFNPTFPETLEAFSKYLEQYNKYPGLVNGTILPKPVPEELYSPF

GDFVKKYGIEAAVPPMYFWNPGVGEILTNPVVEQFRYWSSYMAGGVSAGFISPVSRNVSE

LFAKAEAELVADSSVLLSSQVVKSHRPKGKGDVKLIVSTPSGPKLIIAKKLVISIPPKLD

YVAPFDLSGNEKSLFSKYISAGYYVGVIKGTDFLKNASISNGVDGNTEFDFPDLPGAYSF

APSPVEGLQMVTYATKQSSKTEPYTDEEVKTEIINTVYRIQSQNPDLFNVTKPEIVDFHS

HAPYSLQVRAEDIKNGFYEKLYKLQGQRNTHWTGAAWKGEDSSLLWKYTKEFVVPEVLKG

L

>SoG_03054.T1

MGSLAIFGMMIGLMIGRLTIPEPSVVQRIEVSDGVLVAWFNDEPKLHGEIVDGSVALLFE

AEGPAQMGQLKLNGKDVNWRVRLSDKGLLLPLVAARPLRGEWTGSEVDDRWRLEVRLQEQ

>SoG_03056.T1

MKRLTAAAATLSSLILVADISTAQHSFTGVRFFEPQILSNGSHCWEVTMTTATTGQENFG

EKNAKHFDKVAPAEWPQWIINLHIQTLDFLKSPEFASFADLPPAEDSTGRRLLDYACGDG

LISRALKPYFSSALGVDVSGKMLDKFRKAAADAGYSPDEMTAVRADFVTGENDPTDPPLP

EDKLSGFDLIAISMALHHLEDPEAGLKKLASRLKAGGKLLVIDWTPVDGSTPSQRLYQEE

LKVDGLGERVKAAMRSHHASHTVSRPDGFTKEEQEALFGAAGCGDMRWKLADEMSEVPVI

PGAKGQLYWAVATKSASMNE

>SoG_03060.T1

MASPPLLRWGLPISILAIALVLALRQQQPLQPAAITTTSTTTYCYSSIRTLWSLDASPRC

FVVSPDGLFSDVFTPEDGELPDDAVVEEGHVIPGLWDGHGHLRQYGEFLHSVDLFGSSSL

EEIRVRVKKYIAENPGAGSKDEWVRGIGWDQTSFGRMPVADDLVQDPELKDLYIMLDRID

VHCTWVSPAVLGLLPPIPPTVPGGEIIRDPGPGVFCDNAMDLVLPHWPRPSAATQARQVR

SAMAALNEVGLVGMHDAGETRKTLELYRDMAGHDEEWTVRVYGMFECDKRNTFCPDDAAV

VAGESDYLTVRSVKLFADGALGSWGSAMLEPYADHPDTSGTLLINASTLTTVTGQWSTAG

YQVNIHAIGDLANRNAVSAFTTALKSLCTSSADLSTCQRSNHRFRIEHAQIIHPSDQDLI

SSLSIAPSVQPTHATSDMKYALSRLGPDRLAKSAYRMRSFLHHKPAPLLLGSDFPVEPPN

PFRGMYAAVARRDPATGLGGPDEPDQGWHLDEALTLDEALEGFTRGPAWGAFLEDKAGII

KPGAFADWVVLEEPLNITTDVEMLRSLKVKETWVGGRRVYARS

>SoG_03104.T1

MLAQTLLAALTLAPSLAAPSTTTTTTTRPCSNPVIRREWRELSREDKAEYLRAAVCIRNL

PKKVYQYVNAATSRLDDLVYTHRSLNRDIHFVANFLPWHRWYLQHHEEVLRSECGYKGVA

QPYWDWTIDADNNDTQHSPVFDADTGFGGNGKSLPGDVPGFQRCVVDGPFANSNLTIGMG

YPDYDTPGDRLHCFGRDFNNAMGKDEHGRTIIGDMQAGAYNSRVMNTIYSFQTYAEMHNM

LEGLPHAQIHSSLGGDMGPSTSPNEPLFFLHHANVDRVWAKWQGRNATRLADYTGIRVAN

TTIAASVGDTMPVMRLADDEPVVRDYMDTLAGPLCYTYSDM

>SoG_03114.T1

MIAPDTTALSAALLISAVRAAQPGAVKPVAAPMRDLEWGQLNFLHTTDTHGWLGGHLQEP

QYSADWGDYISFSSHMRKKADDEGVDLIVIDTGDRIEGNGLYDASTPKGLFQYDIYAEQD

VDIMCTGNHELYQAYSVTREHNTTVPNFKENYIASNLDYYDPNTGVMVPQAQRFRRFTTR

NQGLDIIAFGFIFDFTGNANNSRVQKVEDTIKEKWFQDVIREKPDVFVVIGHVGLRMEEF

EIIFNAMRKEDWDTPILFFGGHAHVRDARSYDSKSFAMASGRYMETIGWMSVDGIKKKKK

SDELSTETSIKFTRKYIDNNLYGLHYHTGLNESTFPTEHGKRVSKMIARARSALQLDYQF

GCAPQSYYMSRAKYPGNESIYSWIEDEVFPGVIHNEKRADKSRIALMNSGGIRFDIFAGP

FTRDSTYIVSPFVSGFRYIPDVPYGIAKRVLGILNSGARILGENGHMDTRFLGIPEQMFG

GQVIRQHDDDDAERRLELRDVQVPLLDGEPRLIAGYTTKDDIGTDGDDTIHEPLNFYGIP

NCFQSKINFPEHGDPEMVDVVFIDFIQPWIIPALKFSGGDYNDKDVKQYMEGTFTYKMSQ

WISENWRGEC

>SoG_03115.T1

MRRSLQSLLVLGLCSLAAAVFQDEVGHIDFHHKLVGVPQSSTTFFHRPRKEDKASLLYTL

SDVGIVGAVNPSNGAVVWRQQVADEITNGGGHLRAPEGENWVAAAYGSKVQAWNALSGRN

VWQTEFDGTVKDLEILELADTPRKDVLALFDENGTTVLRRLHGGLGTVVWEFREISKDIP

LQVSTDITNIYVIALHGTANSYNLRITSLDIVTGGRVDGWNLGSKGDVQKAEDVMFVGAN

SAAPIVAWTNADAKKLTVNVLGSKSKQDFGMPADTYWVQVHAPHLAQSQPHFLVHMRTQS

GNKAEVYHINLKSSQITKAYELPKRPGYDAFATSSDGANVYFTRITEDEVTVVSSDSHGI

LARWPYEQPQGDSEIVHAAAEVVKKAGGDGFAVRAATTTFGDDWKMIRNGEVDWTRPEGL

TGAVAAIYADIPVAENLAKVLEQEAHSNPFEAYVHRLTRHMNELMNLPDYLASLPSRFIH

GLAGDHKVTQEQALRHDSFGFNKIAIVATRRGRFYGLTTGNGGAVIWSKKMFDPQPGKPI

EIKGLLAEDEKPTAVAIGSRGELVVFEILTGHTVHNRLPNDIPIASTVIVNGKDGRWLLP

LGPDGKVVGALPAEIAPLSTIVTRQGDVLKGMKLVDQAGQVLPTDTWQFQVLPGQKIVDV

AVPATHDPVASIGRVLGDRRVSYKYLNANNLVVAVHDAASSVLSVRLLDSVSGQLLASQS

YPGVDPEKPISCTIAENWYACAFFGQYKLSDGTERSIKGYQIVSSDLYESPEPNDRGPLG

DAETFSSLNPVDTPTGPPLPWVVSQSFVVSQPIQSLSVTQTRQGIANRQLLAYLPESHGV

VGIARQAIDARRPVGRDPTAAEMEAEGVPRYTPALEIDPRSIVSHEFDVVGVQQIIATPA

FVESTSLILAFGIDVFGSRVTPSGTFDILGKGFNKSTLILTVVALFGGGLFLGPMNDEVL

LGVPCRLAVYFPAPPRVLPTDPIALLDPAKIS

>SoG_03126.T1

MQLITLTLGLSAAAAVMASPLTVETRQAAQCPDYGTTINGPIIAPTDAEASLCCVYGQAL

ESCCRNKPFTDDPYWSKALPCDNPWFADVRSFMRFYVCTVDPCRGICEGVPPVYDNNTCK

KN

>SoG_03154.T1

MALGYILVALVIILLNIEKLPEVVLLIVKSAFGLDQAFGGLIGSAIVMGVKRGVFANEAG

LGSAPNVAAVASVEHPVAQGVVQAFSVFLDPFVICT

>SoG_03183.T1

MHPFGGVIALLHSYLLIEVLLPGQVCDRRIDTTAQVLKHHDCCRLAPRRSGGVSIRVAAL

LRFAWAGYGAAGLGAIPRGKIVSLLRLLSTPSARAREAARRSTGAYSSTTEHTTPPPPPP

ISQTPVRQARPGTQAQCLAGQYDNAATEQHLNCLSNGTILKSLNVFNNKFPELAILHLPT

LMSAWQSPSGPSIETKVLVAAVLAVTKAQLCALNLFWANDLLPKEAYASFARSALLTLVL

EAPNIQVAQSLLIFTLYDWGTREFHRAWIYCGIAVRIVQALHSMRVAPCAPEKSWKADNN

PLAEAIETRTYWACSIMDNMIHSGTYNPPMLPLSEMERLNIARPCSAVEFAFGSDGSVPS

ALYDSREPRNPEGPHDITQSFETLVHGFDIWRQVTTFMFNDGRRAPGMCRPENCPWMPTS

PWHACRTRLETWRRNQHRNMHYPATSVAVHMTLCYGETFTCLNLLYYLCTLMLHREYFPW

LPNKDSVPRAPVDPPHLEAEAPPNWWEDSASELFGAAENISLLLNEASECGVHLMTPFAG

FCAVSAGFCNIYVAKFPKMNLGRSLRAKEMTDICTTYLKEFRQVWSIADSWMKTLKYTNL

LYERLAANESRYRGRTRYDYDILHHSVHEFRGVDRSDQQDREINEVEASIDQLPSLGAAN

EDVPSTDMLLSQLMTEISGNLDEQGVWSNWWPQMGDMDDTTPNNEMAC

>SoG_03194.T1

MLSLAQKITIVAALVALAEARFGQEQIPVAAVQALQAGAPGEAATLAGSIPSSLLAAASP

CDKLSLADKIAALGTDAAVLEAAKGLVSAETNFNPFAVDKPFVCADPTLPATAALRGIVP

LVDPAVTGAGTANAASAQSLNAPFNADGLSQAEVMIAQGFSNFQAVDGSGARVQLAGQNG

GAAAGGAAGGAAGGAAGGNAGGNAGW

>SoG_03201.T1

MDAERFDAWQWVMWLLLCLAGLMPGEATVGQAADPSSVRMSAAAFGVEKNVKVTAVGTSE

TVAEGENTPSLVDLAGRSSWPFMQHGKSPPLNLRSTLITPCLFNDRGQYFSQDPSHQKFQ

LTRLRNNAFEYWSDKLEARSQASPASQRHGITCLRLADKGRGIFYWRPCFNTGVAHVRP

>SoG_03230.T1

MKVTLFVFITHVLAARPFLEEPDTGLESVFGDTPVGSLPDLNRIAGLPDFQWAARNYLPL

SNFTYYRNAAGGEWSYRNNLEVFSRFSLRPRFMRDVTKVNRTMETSILGYNFSAPFFISP

TARAGFGHPNAELNLVRAAAEEDILYIPSLSATLSIEEITEARAPEQVSFQQLYLPPSGK

LSDQLLRQIEASGAKAIVLTVDAPANGDRQRAFRERGGIPTRLPIVLKGILSLSDAREAV

AHGVPAIILSNHGGRQLDTAPSALEVALEIHEEAPEIFNEIEVYADGGIRYGTDVIKLLA

LGVKAVGLGRPFAYANVYGLEGVKKAIQIMKREIAVDAANLGVDDLQHVDPDVPFDAFTT

DFFHNTFTY

>SoG_03254.T1

MQITHLLVLAGAAVTLALSSIEEKGCCCCDISRGAIVCNPNQPADDCFCAMVVCPDDAPT

ITESLPEPTPEPIPEPAPPAPPAPEEKREQYTSAPPPPPDMDWCCCCNGHDYVCNARPKG

DMCICTLIACPPDAQTIYRPWTKPGKPTKRDTRDRAGRPGRSVRPARPVRPSA

>SoG_03310.T1

MKQSNFILLGLLNMCTAQTWALGGCDKLDEKAIKASMKDAVTIANTAFNVLNTQYQEEKV

QSMVKFILGSDNALQSKVETAKKYLGNVAKFERDENPLILQGEPFVDNKDVIIWCDNSNY

DSSRRLNKALNIQLPKEEWEVIQDCFDPKPKSGPARVMANTTPSDGVHPDYAVAFTAWSQ

GGSIGPPPDMTPFKVERPEKPNTIDLCRWYLDTLGVKYFTRIDDETIEKVHDQNFPENFP

PDRKPVDAFAETLTGTLLHEVGHPFSVSWVPLQRDVLENTGRELC

>SoG_03311.T1

MRWLLPLTLVSLFLTTVYTEVFDLGDLKQYEPKTDKLQDGLQPDSIYISEVDDGFRVVLG

KDVKAKVDGAVQACGGISDQCFQTIQNILKDSHVQTDPKLGNRQLLLTAGTVGEAFQTIS

KLAAALGFLIMALNALDGKQLYNEKGSHWPELSASNAAKLPEAAPVTISAAGSAIVTVTQ

APGKTTLEGSITPVVTVVESASDGFEKGDLSGTLDAGLAGRIDEYMHRRKDCQAGVDFDK

SHSKKRRDSGSYGQALCAAQAVMQGATVQGPFNDLVLLDPASVHFRFTEAAGAARDAADE

LAEFVKAYAPLIAVPEDVVQEISMYVLALAIDTIVENITLGQKNRIKATMVTTTDSPAAT

STGCPKPASLFCGTGDKDDCGMKVVGDGGDKRAVCEQNPQPKTKADCTDTKTPIPVSVFS

SESMKVYDQLCNDWAKDKERSMTVDSGGKSVKPEPHLRKRMKVDAGTYRDWMFDLSFRPV

KGDDKCTMNCREAFEHMSRACATFPVNILLPQLGPLGSFMYEKGSLDVGCGSFSYNIHKR

ELTELKEFERSCYKREDFESLKGDGVDEGLVSWLLIKPCAGVARPQQVIKKGDKNTFIHH

SVIMRNVPYQYNIWWKDGCTLADDGPTQMQASNPLDIDDSGHEACQKTLWGNWRHCNNGG

VGGTIQIGCLIYEFKADANERTW

>SoG_03367.T1

MKLLVAIFAAGIMAAPTSYLETVEATNAAAAAAAAAPAPAHGVAKRDVNIVSDNEGGNEG

GWDIQSDQDGDLSLSTDAGNDGSWTIKEDANDGSWTIEDNDSDGSWTIDDSDNDGVWTVQ

DNIDGESWNLGDNADGKNFPFWDLFTED

>SoG_03368.T1

MKSFSVVIIAAAGVMAAPMPLASTETVDAPGVWPGPGPGPWPPVPAHNRRAVDAVDEAPV

LRDTGSCRAWLGCNTEEHLYRVHRSEHLDF

>SoG_03414.T1

MKIRNLILTLAAVKASIASPMDPTVRGEYEVNVDAIIAKYNLTDDVALPITDSDFGTLAR

KCYSDDWRWDGEKGYALDRAGRWCSGNGGSGWYNAGQLKGGCYNLNNVKRVNFKIQNLQS

RRIELSSSACFSFLRGIINACDRGGENENGSWWWTADSNIGVC

>SoG_03439.T1

MRFHILSIFPIVGLSLAQLQPNWTSPAGVSIYNPDNFFKTTGPWSLMSSAGDNLYVAGMR

GIDPTTSELVPTGLPRVRQAYLNMKTLVEMQGTDVFSCLRLVVYVTDMATYRPMCNQVQE

EIWGKDPSRYPPRTIIEVRALNQNDIVEVEGTFYAPHLGRGKIHPPRQDAQSEFR

>SoG_03452.T1

MKLPLTTILFAFAPTALAGNVVDDVCKGVQNIWNCKATFDLPTGAKAKMCRNKFFKTDPC

KTKTEYRYPCPTWRKPLRTCPGWTCVPGTYETWIDTPCGIEISTKRVDLCQSVRGALGNP

GKQFIEKSAAMCKCLPKALEMVGSAAYDSVKSGSDLSGAVSNVLGQLVELQKVGFLCMID

NGFNVKDNKAQVLANDLSSTGGWVVVQAKEIDLATYGKLAVALSPCSIGTCNPELIRGFF

TNYLTASNELMAQTLNQVLRGWVDVFKRIEDRIANVADAAGKLVVRIEAVPGNLKKIEDR

VCKEQACLGQAATKYLQKVSQAVAGVKPLAQVQKDVSMAAEVAPKLTQLANSAVGLVGAV

PNVDELIQLIVSGKLRKIQDILESVQVVKKLPELANRIQDSAAPILNLAALFDKDGARAL

ALLQEALTQSIDGVRAELNDDAAKSGILEMQSLLREELEVPLRNVTESVKQLKHVFNGGG

VPIRSGQFKLNAGVSSYRRWSTVSMDLPCTRKKTAHYTVAGGFKGSFDYPEFYACPSGNI

EIPWPNHHIPYFKFNLA

>SoG_03458.T1

MRFAQLCVIGASLAFSTATAATANPLEGDASNNGDRQRVMAPRPDTTSTARRPLETGVGH

FSEWSRETKRLFLLDWQAGRSNEWIIVQGNEGGDLDSMTAALTWAYHLQHSTQNTSNPVK

AIALLQTPTDALDLRPENKIALANSMMSSGHSDLLTIDELPADPEVLSRNIEGIVLVDHA

SPLRKWSEAKILSIFDHHRDVGAGPDAKPRVFEQTASCTTIVARQMLDELERLPEEYHMP

HELLELILSAIAIDAGGLSGATEVDRKTAQRVIKRSNWRKRELEDKMAELDDELSAAKKD

LGHLKVRDLLRRDWKGDLVDTPSPRTPTVSLGFASIPYSLDDQIDRTEFRELFNWFAVEA

AWTAQVGTDISVALTKYKTKDPSTNKKRKIREIILVVRTDVRINETQADSLFATVVKAVE

ADATLNVKPWYRARELGKRQMVWTHETDAGRKVLRPIIEDAVKYWDE

>SoG_03574.T1

MRGQRLLYWLGLATSGFTSDVVVADLDICGNNYGVFYSFSDGLSVCYEGITEMVIMVGQD

ALSQCETCAGREQDDCVGCVTVVGPCASRCAVPVTSTAVQNGTTTVFILTSTSSVTFQAT

QASNTQTVASGSSYTAGVMTSPFSTSVVPPATSQTSSSTRTVSAAPELPSTVNSFDLYGC

LGSTARFPGFSLQTSSSSMTLELCAMSCSDRAFFGTYSQDCYCGDSLDGSRRVGLGECSI

PCPGNLMQSCGGLASLVRRQSVPGNVLLAAYANSVGICFYVNHFPFLGLSLCLHYVHRAE

TVLLMGHYYWKPDIYILPQYFLIIGALNN

>SoG_03579.T1

MKTSSLLAPLTLLLLSLAAAAPQLPPNSCYGCVLESKCIRRDGPLHFSFNTYYLLDRWMD

MFGSRG

>SoG_03614.T1

MALKSIALVILAHAGAVQAGASSGAQTLLDPIRPLLTPDSASAKNPLGHLGANGPWQPGP

DVYGISNEVPEGCVVDQAAYISRHGSRYPDPGAHKEWLELVKAFKSGNYKASGPMAFIHD

WDTPLSDPNIQIAQLSKTGYKELFDLGYTLRIRYPDLYREGENFYVWANKYPRVIQTAKL

FVQGYLGANSSLGTVVSVSGKGFPEHLGDTLAPSDMCPTFVDDSSAQTGAWQSKWLPGFK

KRLLKHVKGDLNLTDSQWSIFPYICGFESQIRGRMSPFCDTFNDKELERYEYQQDLRYYY

GVGPPVKVASKMMVPYLDALMQRFIAGPDAEGTNFNGGKFKVPKLLMSFLNDGQLNQLVV

ASGIFDNQKPLPVDKMPNERDRLWRSSRISPMRGAIAFERLTCGNPSAPPAPGPPGPRPP

QPKESTFVRIRLNDAVYPLPSCQDGPGKSCSVRRYAEYVGKKLEEQGSFAKICNATDPAT

PTKVAGASFFTNLAQSHLEALKL

>SoG_03657.T1

MKVRFGITCAFLCLRPLLVDGQSQSAHANPYAPSYTECPNDLHLRNASEGLSENEKSWRR

ERAREIKASLETYLKNANVTGLDIKKYIDKLDSENVPVVGMSISGGGTQSGIGGFGVWQA

FDARYPRAVKAGTGGLTQVLTYLTGLSGGGALTVSLLATNNFSSVEGIAKSTNFSSPYDN

PSTNDIERMFENTGAKLQAGLPVSVTDVFGQFWATWVPDDWKFASFSAIANKSTAFKSGQ

APMPILTLAEVIPGVSPEVYKIAYPGRNTTNGFKLTSYEVNPFEFGSWLGGRVQAFMPTR

YLGTAMTGGKFQNKSACVEDFDKMSFIQGSTADAFCAWFIDDFYDIPVFTKRGDSPSPSN

STDSNDISIPKSQEQNPLVQLVNQTAANFGMTFNASMWSTYPNPFENYNEQMKGVSELLL

VDGSLGGESNPVRPLIVPYRGVEFIIIYEASSEGKYAWVNGTSLNYTARSASEGNLPFPK

IPTVETMITQNLTSQPTFFGCNAKLDTPLVLYLPNSPWTAYSNYSYMQGSFTDGQLNITL

ENAFQLATYGDGTIDAEWPACLACATVKRSMARVKMDLPDQCNQCFKRHCWNGEESKDRV

TVADLDLQPRLIPNLTFSAWNNTVWEAKQSSPNGSSGASAGNKSNGDKEDSAPVYFAGSA

AALDGLESMGISVISNVFLIPRHNSWGIADLLALQPITAPRFPSSACIEQVSNGKRLRKA

TICNSML

>SoG_03690.T1

MALRNILSLLGALLLAILCASPVDAIDIRNLLSAENDEQPLKARAAGDKYLIGVGKADIT

GPVVELNLAGYADLAQTGTGLRQRLYSRAFIVGDVNKPDDRFIYLVLDVQSGDTAMRFGI

LEGLQALGKEYSAYKAHNVAVTGTHSHAGPAAWYNYLLPQITSLGFDKQSYQAIVDGALL

SIKRAHESLQEGYLDVATSKVKDGSINRSLYAYLNNPQSERDEYGEETDSTLTLLRFQRA

SDQKNVGVLTWYATHPTSLLGNNTHVAGDNKGVAAWMLERDLAGSDSVADGFVAGFSQAN

MGDASPNVLGAYCDDGSGQMCSFENSTCADGKSQSCHGRGPEFRALDLGVRSCHEIGRRQ

FVAAKEAYNAIQGGESTPVVGSSVKSFHFYHDMRFWEFTLPNGQKAKTCPAALGYSFAAG

TTDWPGAFDFTQADSGKPSASPVWKLVSGLLRTPTKEQKECQLPKPILLDVGELEVPYPW

SPNIVDVQTLRVGQLLIIVSPSEVTTMSGRRWRKAVAQEAATFLDEKPIVLVTGPANTYA

HYLATPEEYEIQRYEGASTMFGKHELDAYINLTVSNMNHLQPGATTLPAPGPSPPDNRKK

AISFITGVVQDGTPIGKSFGSVVRQPSPSYSLGATVNVTFQGANPRNNLRLEETYTAVEK

YDSGSSSWKRVRDDEDWFLVYTWRRTNWLLGYSEVDVTWETGGNAEAGKYRIKYYGDAKN

LVGRITKFEGTSSEFELR

>SoG_03735.T1

MLLHTNAASLVVAALLCGSLAAASRRDEARFETRVRQAPEGGPNALPDAFTDKTPPPAIL

EPVVPPETKEADLSILNLDKDLTLAWAGIPGGGAPGGQSKRDAAVLTQGDFVFQYPAVAL

DHSSLITKVSCSDGKLTAVMTQAAYNYAKEKWAAAKTILLVTGVDGCGIKDANEYFKATS

ISFSDQGSSIVAQGVPVHYKDVLVDMKLQWEM

>SoG_03752.T1

MKSAIFKSGLASLLLLARGALASEQCQCRTTVIVIEMPEEYTTHEPIKTVTIEPDAAAAP

PASFMTKVVEASATADNTRQPSQTQQAYTPPKHQKPPVDQAPPRNTTQTYKPCAAPQVDR

KDPKNFVPAKNVKLAYAPADEEEKQQEVKQRPEFVNNRNQTQPKTETKSQQQGSIDMNLA

FKYPAVVLEHIESVAKVSCGEDLLTVSFSSSSGFDEAVKDWDNGGEPFVLVTGALDEGCA

AEFERGYFVVAGITKDAGSLTVQVAATKGELPDLAGEMEMVFTSIPAGELMRRLTVAPSW

SLELAKAVPQHTTLFTDGKHIDITAEEAWFSTKVTFSGKLKYNFWKFKLQELYFDLDAVF

DSSAVLSAQIRAAFQELVQYKPDDLAYSLIDVPGVVSLGPGLAFGLSVNVKASAAVDLYV

GADMALPAGNVHIDFLDGFRTTTSGWEPKYNTFANASQSADVSLDVGADLSVMLQFKLLG

GLVDLSSGFTASPGVANSFKLRGKQQASVRGSLKEISAGIVVPTDAISCTDANALEFVSD

FFFSLSAYATKWWKKELYAVRVPIVDYCLPF

>SoG_03753.T1

MKFSTAFMLAAPLMVDACKITFYQTDNWDELGRRYRVEIKASGVPDMWKALDDTCNIYRS

LTSGRTDIQLPLCYGLADKGIAQADTSVVHGPVGLRIHNGLYDAIVAELQRNSNYGPNCK

YEQHSTW

>SoG_03802.T1

MLFKIIAIPAILAASTSAAAIKHFENFDIPAACGVCDAFGIGCVAACVAGGPLDPICDVC

AGPAIGTCLSCLSQN

>SoG_03829.T1

MRSILGLTALAAALSLVQGQTQGVTTDKGSYSLQNTKGGDVNENTGTNQGANVRVNETTT

GGSGVDIGGHGNVVSGGQTNAQGSYNGYGNLNHDINSSYNGNTIINNNYGNAPKITCIGD

WCVEQPPCADTDASCQHQRVVYQNGVYQTENATDASWNRLVSCEGSRCHYNRCYDEGCSR

KVVCSGGRCAEEPCADDGECLRKLVCRDGGVCQHEACEGNECGNMYNLVDGQYQLCPTCK

GAKCPRPQVPGSEFFSKMQDVVQVQGAAGHGQQTITIEVKISN

>SoG_03835.T1

MSIRKLTFLLSFFLWLHRSFTTFTPLRPTSLSSSNTLAIRPNLTSFAPATAGSPAAAVAA

ADLVPKTAITTHPALSGLQVRCGPRNTMNGATRLIQKRRSGFLARMRSKTGKKILTRRRV

KARKRLSW

>SoG_03867.T1

MRAFNIPGVIATLATIAAVPSYGAPSPGGCTTLRHWPAEAAQSLNAMIRANAHSGAYAVF

DMDNTAYRFDLEESLLPYLEGRGILTRERLDPSLKLIPFKDTQTWNETLFSYYYRLCEIN

DVVCYEWVAQVFSGFTLRTLKGYVDELMAYDGKIESKYYEGDKVVDISVSRPQVYRGQQE

LFNRLMQNGIKVYIMTAALEELVRMVASDPRYGYNVPPENVIGVTMLLANASSPSTPTTA

RKQIAAGEYHEQANLDLTLTSYLWTPATWMAGKHAGIMTYIDQWRKPVLVGGDTPDSDGY

MLFHDVDVSKGGVRLWINRRDKYMNQLNGMIRNNTQRQQELGLEVTADKNWVIVKPEQIL

>SoG_03925.T1

MQLSYLLAALPLALASPVPEAAPQGSLPTIPGANPGATPPSGVQITGVSYAGSGCNAGTV

ASILSSDLSTLTLLYDSFVAQAGEGLSPSEYRKNCQLNVGLKYPQGWQYSVFKVDYRGYA

YLQKGDKGVCKATYYFSGVTQQVSSALTLTGPYDDNYLKTDQFGVETTVWSPCGQAGYLN

VNSEVRLTPIDNKKTALLTVDSTDLKFSQIHYLQWQKC

>SoG_03928.T1

MIAAVAFLGATLAVASAVPAIPGTHSFKKRWEDGVDCTTDLVDRSTPGLLFAVASDGTAV

YCDEIHEVSTEGWGATCYVTVGNDRLGDTVKCPADPAPRKNPDGTDAEVIADGFIGTWCD

ATAGGLAYTFAQQVGCLVYGYYEANGDVVAQNEAGAIAVCNAILFGFCTYLLDVAPAIWE

GRSECVDPPGSSLCSIAGPGK

>SoG_03941.T1

MKINKALALSLLFLPLALATDPNKRPPRPKTGESGRMDETENDNPHKPPKSDGPNPPRPV

GTGEPRLEVYFGYPLIEMCRAKDVTLKLESKYINDLHKYYCCPGRKGGSCRKDLFCSHSA

TTDEPYCCRKTSPGGCTGKRPPLRYYYNEQYRSLQELSPSTLPSRDGPGGSNVPPAPPPA

PGNNTPGDYTTDETGEAKVDPRNTITDGGGSGTDSDDTFGTIDSNAGNGPQATPTPPTPA

NIEPSDADTISETKRGDEKTRPEKPSSTATDKKGNCPGGFELISI

>SoG_04008.T1

MLSLLALISLILRHPDILHAGSVPGDLAPQHRCVLGALDQPALPDHRGQTDDERPEDDPE

REPGPPEDHPAHVVEDGEHGEDERRDGDDTRHVGTGDVEAQLGEQPVEERRDEGGRSADL

EVELGDDGLEGRAGVEEVIVDEGCDLWWEQRAQGRARHRRVHV

>SoG_04048.T1

MKFSTILTGLFAATAIAAPVAEPAPVDTTPNPLDTRAVFDASRLNNLRFNQVDLRYLQGL

NAFDLQLFQNLHVRNNLDIFFFQNVFNAQVFDINALLQLQSLHTLLLVGQTGIFNNFDLR

NLQLRNLNFGVINALAGVDLVQFIDTSFRGQIQTIADGGKFVQHTTL

>SoG_04052.T1

MRLSSFTAVVGLLCTTVLATSEATDKLYGKDPMKILKQLDKKALHNLKEAGSSGTCTLKN

ASRRKDWNRMSKKERKNYIKAVQCLFTLPSKTRVFAEGARTRHDDFVATHINQTLQIHQT

GNFLTWHRYFVWIYEQTLRNECGYRGAQPYWNWFENAEDLTKSPVFDGSDTSLSGDGEFF

LHNGTNVGPPDKQMKIPSGAGGGCIKSGPFKDAIINLGPVAPGMDGYKPVSSDPYAHNPR

CMRRDLTSYATENWMTAEYLLNITVGAASGSIALFQDELQGRTQDGFLGMHGAGHFAAGG

DSSDFFTSTNDPSFFLHHAMVDHVYWLWQSLHVDQADEIAGTITMFNNPPSRATRKDDIL

DLTVMGEGITIEDTLSTMRGIFCYVTAAIIGIVGFDPPERLGRWIANFATDAYTKYASFA

RVGEPVDPCVQLEQLLDTMEREFDELAETESRIERLRSDGKISYIDREHPGPFIDKQIAG

DASKSWNLVFEFYDELLLQGRNADDEGSAAQGCGDSDLSSEQLPFDRKVAETTQSSGR

>SoG_04085.T1

MLSFLFSILRVLVPFLALVSAAPSGLVPHVTLSFGLEPADSSLLDRTLQEVSDPLSSRYG

QHLTREQAHALVAPQGDSAAAVKNWLADAGVSRDQISQDGRWVHAHVPQEHAKRIMPSGE

ISRIKRSLPAGVSEHIAVIHRSPDSEAGRSYTRAFRNGNSPSKTRVLGGRGIEERKPDLD

NCYRVASPTCLREWYDIPMKKQKPAKGNLLGFVGFLEQTAQHAQLEEFQRRFDPFSTGGN

FTTQGINGGKNPQETPDQQYPSGEANMNVQYTVALAYDVPVRYYPVGGKKTDYIPDLDII

KDDDRLGRAVEPFLELAQGLMDLDDRDLPLVVSISWGSNEQHYQREYAREACNLFGQLGT

RGVSVITAAGNSGPGVSCQSNDGKNSTRFIPVFPASCPYVTTVGGTRLESNTSAAGRTGR

SIAAEFSGGGFSDYFDRPKWQEKAVKDYLRKHGKKWEKYYNEKGRAYPDVAAHAGPDNSP

LMNHGIVEYSGGTSLATPVFASMISLINNQRLKDKKPPMGFLNPWLYKVGPEAFTDITKG

KSVGCEGQSYEGLPSPVIPDAGWEAVKGWDPITGWGTPKFDRLKKVACE

>SoG_04127.T1

MKLIRIAAFGVCSLAGSAIAAVDGNTLPRGHQRRANPTGIQEVASYGLVTGACETEFHPD

HLDVAWVELILSNAIQRVNLTSGEQTTYPLNNPVGLPSGMEFMPDGYLWFSEIAGNSMVR

LDPADGSMTEYPFPWTGISLIDNLPVGIRVTIDVSGNRDRGAWFTAVGLNAIGRIDIDTF

EYSIYPLPHPLSAPLIIQPGPGSTMVFSELLGNRVGTIDVHTKEIKEYNIPTPLSLVQGV

ATDPSKDDGIIWWSGTGAGTIGTIDTNTGKVTEISIAKLRLTGKSKVVGGPSLGGSAILP

FPGPMRVGTDGKVYFVMGSIVTAVPGNRIGQYDPKTGRLVEYQTPQSLFGPCDINSQHPG

TMFFSGYLGSATDKLEYM

>SoG_04175.T1

MVMYRSLSCLSLAGLSLLDVASAGHVQFERGRQARQAGAQPVIMVEHQPVMMSSLVCTNT

TLTFGDKTITVNAAPTLLVTNFDATTTRTVTPGINASPIFTGPYTTINGGSWTGTAQTTI

TVPPGAGGSTGTVIVLNPATTTPAFTGPYTTVNGGAWTGSGESTLTLAPSGSGTIGTKVI

FTPSQTTTHFTGPYTTITGGSWTGTGQSTLTLAPGGTDTIGTKIVFTPSGTTAAPFTGPY

TTITGGSWTGTGQSTLTLAPGGTDTVGTKIIFTPPGTTAGPFTGPYTTITGGSWTGTGQS

TLTLAPGGTDTIGTKVIFTQGATTGPFTGPYTTITGGSWTGTGQSTLTLAPGGTDTVGTK

IIFTPPGTTAAPFTGPYTTITGGSWTGTGQSTVTLAPGGTDTVGTKIIFTPPGTTAGPFT

GPYTTITGGSWTGTGETTVTLAPGGTDTVGTKIIFTPPGTTAGPFTGPYTTITGGQWTGT

GQSTLTLAPGGTDTVGTKIIFTPPGTTAGPFTGPYTTVTGGSWTGTGQSTLTLAPGGTDT

IGTKIIFTPPGTTTGPFTGPYTTITGGQWTGTGETTVTLAPGGTDTVGTKIIFTPPGTTA

GPFTGPYTTITGGQWTGTGQSTLTLAPGGTDTIGTKIIFTPPGTTAGPFTGPYTTITGGQ

WTGTGESTLTLAPGGTDTIGTKIIFTPPGTTTAPFTGPYTTITGGQWTGTGQSTLTLAPG

GTDTIGTKIIFTPPGTTAGPFTGPYTTITGGQWTGTGESTLTLAPGGTDTIGTKIIFTPS

GTRTTTGTDAGASSTQTVVSTGGAGASSTAGSAASVQSSGSSAQSSGSSATSGAGATSSS

ASGSASTNGAGGSSTGGSGSSTGGLGASSTQGSQTSATGNAGASSSTGTSGAASSTNGGA

ASSNGGSATSSAGGSGASSTEGTASSTGGSAASSAGGSGASSTGAGASSTGGSAASFTGQ

GGTSSTNGAGASSTEGSTSSTGGSGASSTGAGASSTNGSGASSTGAGTSSTGGSALEPRQ

LEALVLHRLEDLVLHQEEGLVIQKA

>SoG_04180.T1

MLPNFALPIMAVAASASAAVLNIRAPTPASVPRVYTGEQFNQQSQDIQADGSCVRLRDSY

YHNVGSISMPCPAAAPSGMKYICTIYHSTDCGPKDAIFDHVTFEVSHTSLFSRPSDEEEK

KYVGGLASSVKCEVVPASQAVVKGYDHLSTTHPASCLEDSCQSDGDCKADDAGNLFCSNV

SGTNTCTRSFDEGQSCDRDQQCLAYCSSDGKCTSPKDSDLDELDAFP

>SoG_04220.T1

MRILSLNILALATQVVYAATILESIQTVNTATGNLGNIVRSWKGDFFGTLPIIESSGKLL

CDINDGTKAAKASAPLDFNQAIDVAGATITLSGTVNSTLQSIIDRKPDFQKLLLNPVILG

TLKITKSATDDFSAAIEEKVPSEAKPVAQELIAQIQASFQEALDAYKLF

>SoG_04222.T1

MLFTLPLAALAALASGKEIPKDETRAAELYDSGIMHERIMSEKFRQWDEMATMRASSSSS

SSSSQAAADPYVELHFAQCRDGKAVPFRDQPNFFFRCKNMNLHHFLPHSAFGSQTGQGSS

SWGWESDDGREFAIIAQADGAAFAEVIRGGKLRYLGRLPQTAGAAAAIWRELRVFKHYIV

VGSESYNHHIQIFDLKKLLDIDYKKPVVFDPTKDLTGFYGGREICYGYNEDSLTIYDVTD

KKDPQLVSVTSYEGATYTHQGWVLDTENQEWLIMDDEYDEVDGRGPGANGHATTFIWDIR

DLEHPKQTGYYQSPRKTVDHNQYVFGNYTYQSNYGAGLSVLDISSIPAKPNGSGVHEVAW

FDTYRENDNEEGGGTLQFVGTWSSYAGFRSGYILINTIEHGVFLVKVQNPKNGGDWTAE

>SoG_04250.T1

MMLFIALGTFILGSGKALASPMPSGVDWSTVRCPESQSETLPTPTYGYDGGLFSVCTEMI

FNAPAESIYEALFDFQSYPKFSSFVVDIELPPHINKTPDDVYIGLEMTFTTRDIFPVFNT

TSVEIVTVMHGSNDGGGYMMASWRYDDTLHGKFSRSEHPSILVEQGDGTTRYVSFETFYN

DPGTWLLLPVRKQLQDGYAQHGEDLKEYVEGLRRKR

>SoG_04256.T1

MLLSKATQAAVGLSALAGVALAEVAISDFDWSAVAPSTSLNYTTCYTDFKCARLLLPLDY

LNSSNPSQVTLAIIALPAVVPETDASFGGTIFTNPGGPGGSGVGFLLQGGRALQRMTDSD

EKKFEILSFDPRGMGQSLPRSDCWNDEVARTVFNTEELAMGPPDEGLHAIGRNFARSRGF

GELCERAGGGGDGDIRNFSSTSSVARDMVEIVDRIDELRNPSKKKDGGDEDGDEDEEESQ

LELRSSKDLPRLNYLGYSYGTALGNYFASMFPGRVGHVALEAVVDIHDYHNGVSPSLSLS

LSLSQITTNHLKARDATKQTH

>SoG_04258.T1

MLFTSLFVLGTCYQAHACSRVTYNAGNDRYLIGRSVDFVANTNTTIYAFPAGLLRNGGVD

DYPFNWTSKYGSVTALMYDKAYTEGVNSEGLAGSTLYLGDSEFEPRNSSRPGMFVGLWMQ

YFLDTYATVADAARAYCPGNGTEPFQVVVKSIVPTVQTNLHLSFSDPSGDNVIMEFAKGK

LTCYQSVNYTVMTNEPTFDQQLAIDAYWGPIANSSLPGTARPADRFARLSFYNKQIPPAS

DRATAVSYCAGMVRAISNPMLPTSFETAGSADIWPTYWRMYTDLQDMIAFYESGTSPMLF

WYSVKDMDLSSKGKTKLLSLKSVSWQDRMGDMTKEFKDVSTETCQKIWTKC

>SoG_04268.T1

MLSNKLNTALAVLASLSVASAQTFRRLGTCPTLGCVLPPDQSEFLPGQLFDLRVEVHAPI

NGSEAAHGGKPDEKFTVTITKEGGKPEDFAKAFKLDEPKLETWTFKWYEDLFAEDAKTPS

VVNVTSKAYRKLSLTEPGKYTVKLKYYGTEETTAQWVVRDIKPKRKAKNVIFFIGDGMTT

SMITAARLIGHKSINGKYQSLLKLDEFPVLGHQMTHSIDSYITDSANSASALYTGHKSTV

NALGVYADSSKDPFDDPKVETIVEIFRRVHGGAWGAVTTAYLADATPTALTGHCRLRSQY

GPLIDQALNGMTNFTWTKQDGPDVYFGTGAEYFLPSSVKGSASYKGKNYYEEFAKKGYTV

SLNKTSLLSADPTKKALGVFSTENLPVWLDRNVYKDNLKAFKNDPTGSGQAALDLPGLKE

MTTKAVEILHNRGGDKGFFLMSEAASVDKQMHAMDYDRALGDLLELDDTVSATIKKLADL

GILDETLVVVSSDHGHAFDVWGSADTKYIQEKKDDREKRNAIGVYEKSGLSQYTVRKEGV

NYGTPESFPMNWEPRYAIAAGVGATPDHFENYKVHKEGPRKVTRELNGVYYANEKDATGG

FLINGTLPATESVGVHSLTDVAVYAWGPCQELFGGSYSNIDIFYKMAECLGLSGSAGGCK

PKPKPAGP

>SoG_04278.T1

MKTRALIFLGFAALASQAHASSPEAWAAYDKAVLASCIKASGLKTAEPVGTAAQIDDRVG

YTALLLQGQYPQKHMKGATGTELCLYRKKTKTAHVT

>SoG_04281.T1

MKTYFTLSFLIGPALASIFYDLYLGTPNGANITKLSGLIEVPTTFPSGYPDSGGTYYLWP

GLTGSGDGVLQNVMGPNKDGQTWAIYSGFYSPTYSVPWGGGIDTLDGGQQIWFNNYWNGD

GTWTSQTAINGQAPAATNTFPLQGITLDTAYFAIELYSPAAWNFGPLRFTDVDISWVGSD

TSACNPSLGQQNTFTWSATGVSATVSNGYVSCHFDAITLESPEN

>SoG_04353.T1

MKFFAAFALAVLAAAAPPKQAPSPLEVKLEACGNSAVKAIVTNHGKNDLKILKTGTFLDS

APVEKARVRAGEQSIEFDGIRVRLVDGPMADDDFELIPAGSSIEVTFDVAELHDLSVGGA

YSVHSSGALSFAEADSNKLIGSVPYDSNILKMDVDGEAASITRIEFHAKRARIQNSCTGS

RLTASRNALNRCVTLARAAQKAALSGPANKMNEFYKSSSSSTRNQVATVFSRVATECSST

NSGVSAFYCSDPYNVCQGSVLAYTVPTVSLMAYCPSYFKMSAASTYCHGQDQGNTILHEA

THLRQIKGTDDYGGYGYNFVRGLSAAQNLNHADTYTLFAQSVSRGC

>SoG_04388.T1

MQFYFVALLFGASALAVPTGGGGGGGGGGGGGGSGYQPCPSTLFSNPQCCATDVLGVVGL

NCGNPAATPSSKANFVKDCASRGQQALCCVLPAAGQAVLCQPPV

>SoG_04391.T1

MLCELLLRSLALAACLGVIKQTCAEGAYRNTDSCGTDYGVFRRVSDSLSVCYEQISVIKV

VVGREAAISGPDKCEACTGRAAGDCDSCVTVVGPCSSYITAPVTSTIKDGDSTTVLILTP

KNSDAFIGTADQPIAPGGIVPDALSASEIAESGGYGAAASTNAGSYINVGTDLPVSVSVS

VSVSKTTSGTDWISVSFSPDGSFASSSYTTNTRPSGPYSSLETSSHRSMDTVTSGTITSS

GRVPLGTTTTSSQTSPGGIPGFPNQVGTFSLLGCLASTSGFPSFSLIQSSASMNFGLCSA

LCRGRSLFGISGYDCYCGDVLDNTAMVRVPFERCNVRCPGNLAQFCGGRLDIQRRQAVPR

NVLLTVYALPEAMSSSSSLPVTTTSMTSSDEINSRSSSSSLPLISSTSSGFVTASAGTLR

TDATISTTSSSISSATTSSLGPSTITTESPAKSLPNGSSSTPTSTGSLLSSTSLESTSTD

IRSTSTNFAGASFVPTAATSLPGTLPTTIPTSSSLSSTSAVDSTRTSAASSSPTTSSSIF

STATFETTWTRIWNSSTTLDLMGLSSYSIPSTITVVPDSTAAATRLPNPTSPLITADVSS

GSPPTTLIPPTLTLDTTSASSPISSPSLANGTFSISNSASMSTSAAISTSTISTAAITTP

ENTLSSTLSYTPPVTPINTTIHTPIITPSNLSVSTPVSTSVSTPDITPITTPLNTPIETP

ANTPGNSTVNTPSTMPTSNPLSTSNETASAPSTPDTSLAFATLNNTTPANNPAEIETSTL

ATSTFVNATTPASATVTPIDTPTNTPADTPVTTSQNESNNTSSSFTGSSVVSPNATTTPD

NTPSASDSPSSSTPDTTTASSTATPLPMSSFPCDKNYGYTFESANTIYTWDMTTGQNIEQ

VGNSRLGPGGRLFSLGYNALDNYLYALARNSDNTAGLLKIGREGNYNLTANITSVKTSWK

AGDIDSNGKMWVLSADNTIWGAIGLNPVGSFESGSFATAPEASINDWAATSSNPGNLFTV

GNDGNGNTLLINFDTSKKQFSTVKNYGAIVASSASWVSAYTDGDTLLMGRSDVREVWAFN

TRDSTAAPRKFGSGPSSQSDGARCAFSPNI

>SoG_04396.T1

MQISLITAIAALLQLAMAMVPDAAPGTRHMWQKPPGKDIRIKYLNVPYTYDDALVKHADL

AGMLDVYTDGQTWQVMNGKRLFQLPTDGTVHALAVRRSTEVAIMDAEELREVLKSQPSYE

ELHSEAMRIMQEHEASDNLLGRDLLHCTLIWCNRSSDCSRASDSEGICNNCINHDCHWHH

GAMVNFWHNSPDAVHNEESKEATSAVPIEKQASSSTS

>SoG_04407.T1

MKFLHLVTFASVTLAVPNPVARQDSVVGTINSAVKTLEGTAQSNIKTLSMVSRSKKDSPI

PYANPSHPTASGLDAFKGSVDPNAVNQVQDTVKKAFSAIAAELKKATDSIVSVTTKAAGG

VAGAAQSFGQAEVDQLVESIKTASKVVADVKGALERAAKELTPEIKKAVEAEIGAAQKAI

GPFVDPLTVLVDATKRVVGVKDVDTSELDKVTGQLKKNLQGIIDLVGLPGVNVGAGLGAS

IGTM

>SoG_04430.T1

MLLSVFLYFSSFFSLATATAASPYRVHKRAPAEHRGWIRGPRASKDLIVPVQIDFAEPGI

DDAGDRLLAVADPRSASFGKFWSPGEITAAFALPDEEIKAILLWIGDGTDHGTQPTLSPC

RCRLDFNATISQLEKLLKAEYHVWTHGQSGETSLACRDYSVPEHLVSSIDFILPTVAPFY

VPDGAHQISEPSSTGIRTGDDFNLERRQARVDCNRYTTPQCLREWYNMPSRPSDYKIHEN

NTFGVFQPGWSSWLPRDMDRFFQTFEPPMVGSRPVMQIINGGYYDNETENMFFNLEANLD

FQYAMSLTWPQPVTNIQVGDMWRSGNPNHMLAAFDEFYCGSLDPAVDAQYPDPAFPDGYP

RQDCGTHEPPKVISISYAWDEGDFPPDYLRRQCQEYLKLGLQGITVLAGSGDEGTIGRTD

GCPETEDPSRRFHVSFPASCPWVTSVGGTMKIGEPGQTALTKEAVYTHAFDSGANSSSTG

GFSDIFRVPDYQRAGTESYLKIESERFSKLNGTCLFKRRGRGVPDLSALATEYIVGIHGQ

FRPVYGTSASTPVVASMVAMINNERMLRGKSSVGFINPVLYQHPEVFTDVEIGENVGCKY

GFRALPGWDAATGLGSPDYKKLLDLFISL

>SoG_04431.T1

MKASVIITSALATMAMAAPTTELEPRTKCLLRCARGCPKLVPLFLACYAACEVTCGAIAE

PGEPIEIGDIIEGAE

>SoG_04432.T1

MHASTLGLWPGRVHLLLLWAGGPAHKTPNIKLQPLIHSRDMIPSTARSLVLALLAACQVS

AKKEHIEVSDIRIRRVTSAGSESIPEISFRLSGAGAKGQECSAENISWPEPSRSFPCGKT

DYSFLLFPGEDGKEFGLMIYHDVGDRFGWSLFSEFLAPFSQKGFPEYLLTSASYRKADLR

GWVDVDAECKQADKGEEICTLKGPISKVIDGPVRGFLGGLFGDL

>SoG_04436.T1

MYAPAALLALFAASAAAFPAASAGGRTENIDIADLSVRKTQAAGSAEKTIQSVSFKLTGE

DAKDLLCEASNPELPSKVITCGDSKYRFNLDNGKDGYEFALTIYHELATAFGFWGEGVVP

TYCHAGGDGPNDFVCSQTGPTTIVISANGSN

>SoG_04460.T1

MKLWSSLVIFSSCVLPSASLGFPHDESISGNSHQVPLAQGDLSDEALHLPPVKITADTYP

FWAIAHRVLTVDGVRAALEDGANALEIDMTAWNARRKGWWADHGHIPTSAGDTAWEMFNE

IRKQRESGRTVSFVWLDIKNPDNCDFNNPKERNCSIEALQHLAREFLEPVGVGALYGFYG

VDRSSRAYNYIRGSLRPFEALNIDGDWQAADSEFDRSPQIPRKQRVFSKGLASPYGNLGG

MKCEHETGICPELKKAASSRRFGHVFGWTISNLEIPLVPLFLDRTEIDGLIYGFTATYYY

KHKDPKKVLEVIKVLVEASPFRHYAGQNDKPW

>SoG_04471.T1

MQKITVALTLALAAISIAAPAHSIKRSDADEATRYIADKRSDADEATRYIADKRSDADEA

TRYIADKRSDADEATRYIADKRSDADEATRYIADKRSDADEATRYIADKRSDADEATRYI

ADKRSDADEATRYIADKRSDADEATRYIADKRSDADEATRYIADKRSDADEATRYIADKR

SDADEATRYVRASALMKSLCQD

>SoG_04491.T1

MKFTQTPLAALAMIPLVFAAPQPEKPDVLADLFKVDGDHWLEAVKAEAARQEAQGSKGVQ

ARNGKFKVHAMYTDNMINVGDVDYFHALWQRMYDVSNDRGGLSDTTTGAWHKWCQKPNEG

SNIDDRFILDGQWGAVNGVSGWQMRDALIHSMWETAHTIGTTGSNAYTVYSDCYGWAWQE

SKPNNKNAACGPVAKVRCPENNDCPAYGMECEHSKPGAWLPSIIRINVYNPDGSLRADAY

QARISSEMSGGKGCDKLDQIASAVASFLPGAGQYFAAGISVQCTLRG

>SoG_04504.T1

MKFTIAAAAVAALSGVASADDVQSVPFQLQLVSDDSSLNGQVLTACHTGAGMESLCLNQG

TGTNFNFNFTEGQPPVSQGFTATGNVVFDLVYTGGSVSQPMYFQLEPSTNVAMTLFGPST

DQGQPVAFDTTTNAMRVFTYMDDTARPAKAINAQALNRWFLCGTNFMGYQYKTLNWILGS

GLPQNPSCKKVDVIRKF

>SoG_04522.T1

MAALSKTLAIGAALLMPWAAHAQQLIASHFSGTIYALNYSNGQLTITDQNSQAGQRIPAW

VTWDSKAKTAYVSDESWFGSRTGRFASYSLSSAGKLTVSGVATTNGGDLASGVYGGSDGN

SFIAQAAYEAGTLSTWALPIKSSDAPMQSFQYFMSSPGPRPDRQNKPHLHSVFTDLSGKF

LLSNDLGADVTRVWSIDSSSGRLTECPGIKAHTGGDGPRHSVFREANGVTYMYVINELSE

TVSGYVVTYPSSGCLEATHFQTISTFPPNGTKPNNTKAAEVHIKGDFLYASNRNDQTFGQ

ERDSIAQYSIGGDGRLTFLGLANAQGYFPRTFSISSDGRLVAVGGQTDASVAIIERDAAT

GLLGKLLAKISVGKRGTYMGEDGLSAVTWAQ

>SoG_04583.T1

MFVSKLLFATLAVMASAAILPSVHPGDGHYVVTIDENGREVHTRLSDMTDDELDIFRRAH

GAIPGDLEEEYLRHQEEVEEKQKPASLEARAWRLDHVWCGCGYDLHHGETDAATADLKAG

IDRARGIIDTNHYSIKGGTVAFACSPGSRRIFTSRQAAEGFQIITNK

>SoG_04598.T1

MKFTSLFLYALLGQLGQVTSLPVEDDHLPTSPRLIQNSEKRAGPRWDWDVNLPNGDKRDR

VVEAWSGMLDMAEDAYDATAKDYYEEVINRYFDGANEAFGIYGVLQRIVGTDGGGLGGPA

LDNVLVSNRQIDGDQCANPNTLAYAANIELEGKPGCFIKLCDKAYDFPLASEIQCGDLDD

EVSAKMSLLAGIFLHELTHCDEIGKAVVQESIVDHGTEGYGPQHVVKNGKGNVFRSLINA

DNYRWMATEFFWRAQCNKGFKEATDNADFKKCPDAEEGICIIQ

>SoG_04612.T1

MIAFAAAAVVHLRWIPAITGAAIPCHAGLVRFSGLSTAPNPVLARPVSLQLDNSTDYSYS

QQAMVPGTAGMFSFPQTEPQTDFLISSWGTTVEGAQSLQDFQEAGSAHSGEHEDPIFTSG

TSTPRGVRLDPAHNVEKWTNPRIASVSQGGHAMSRMDSNRSSASVLSRSSQLSHAHSTGN

ASAFRDGSQTDGSLPGMNNVLLDGTSGLPTQMYWNDYHPLELNSLGLGDGAYQVTDVNPL

QVVSNTHIPLGSDVIDSPLSWECFSTISRTSSPSTVDEAFAILPLSPHSSPEIACQSPSV

DRKPLVPEDFNILPGSQKDDTILNAVLPARRQSNETDARNHPLYKDVKPHADGLYHCPFE

NDPNEECTHKPEKLKCNYDKFVDSHLKPYTCRVASCNGAKFSSTACLLRHEREAHGWHGH

GEKPFSCTYPGCERAQPGNGFPRQWNLRDHMKRVHNDHGSTGGSPPSASAPQASKGRKRK

TDVQESQGTSARKATVKAMPAPQVSATSTKPLIEEWLEHRQAVESILRSGLNTPEDIGNI

NQISRVKEHLAAMAKMTTDFSTQPRTDIITAPRARAYTTTG

>SoG_04633.T1

MTLLISFLSLMTIIGQVTCLSAKPNSNAHLEPLNGVTFSRAKPITSPSLDASATYSSRLR

SLRGHRSRRSSLTAVSRLSSPQVNAWAWNGSVSPVSVIGGYSTQYAIECTWDGTPVWMLF

DTGSSDTWAAQKGFDCLTSTGMVDDPEICGIGPVKIGDFAHGLVDEVHVFVKYGSGETVS

GPMGYSDISCGGVQVAKQQAGLANSTYWHGNNVTSGILGLAYPSVTSGFYGQVGEEAIWN

TAPYTPWFTKAIKQGVVAPMFSVALERGSTDGVLAWGGLPPNVQWQPGSMVSTDLIIANI

AGVQQTAWEYSFYTIIPDGMRWGEMYDTTKYPFIVDTGSSMIYLPPPLAETIATSFEPPA

TYLYQWGAYYAPCDSVPPIFGVVIEGKTFNIRPDDLIFQDVVDPATGFCAVALTTGGMGP

YILGDVFLQSVVAVFDVGGAEMRFYSRYYSEDFSK

>SoG_04684.T1

MRFIWVTLIIGLVAASPSRSDSLTRRTFVEIADLLGKLGNIIGDSGGGGSLDSLPEYKKA

LLCLGTNDGNNVNETAVETVFPGAKGEKLQKILDFLGKKCENGGPLGSLKNPGNSTDIPP

DAIGFLCQAATTADGFLDEVFIGKISADLGFSNPVQTKLQKVLKTSCVWCPLAPRAN

>SoG_04690.T1

MWSVALLLFGLGSAITLPSGYDTVWDYQSTDSSASMPLGGGDLGVNVWVEDGHLLFYMQQ

SGAFDENNSLLKLGRMNITITPNLLGPNDYFRQHLYINDGYIQITGIEGLEIYIWVDMFN

SNIHINATSGTQVSYNVSFETWRQEGYQMHSPEQQQTSWGVNAVPQLPEPYQYPDIIKFL

DGGLLSYHHNAEVPLFDAQVQQQGLKNPESYFNPMRDNTFGLFVYSPQLKQGDFQNGTYA

NTPYVGYTQWTGGTTSFEMVIGTNQTQTTDIDDWIRELVSVTKKSSSGSQSETINWWNAY

WDRSWIIINPDADSSDSGFQVGKNYQYFRYMMACNAKGKYPMRFNGGLFTFDPDLVSPGV

KFTPDFRKWSGGTFTAQNQRLLYWPLLKTGDFDIMTQEFNYYQNICPNSRKLGQLYFNLD

VSVTSEQIDNTGLPNIYEYDANAYGNNPEQRSPLYPPGIDFNYWLAWLQDTANEFADMVI

MARSFYGEDVSQWISFIEYQLAWFDEFYRQRNGLTNDGELIFYPASGAETYKLALNPAST

VSGLQRTITDMLDSGIEFAKGNTTYYEQYRARVPQTPLQECPGYSGLTCVAPAQNYSYTQ

NDECVAMYPVFPWGEYGLGQPTNLSYALHAYFNDTESASFHGVNGWRQDQIWWARMGLTD

LAKANTIYRLSDSTTYRFSAFKGPNYDWSPDINHYGSSALGLQEMLMQTFALNNSQIRLL

GAWPSDWSGSFKLTAPSQTIVSGKIHSGNSISDLLVTPNERMSDVVFGYDESQNPYDYGQ

YYEATTS

>SoG_04697.T1

MRFSGSALLATCLPLATARFVEKHEADADNVVFYPDTAAEYLIETAPGETQWVTEEDKWE

LRRAGKRFMDITETQALGSLRARSAAKVQFPKKCVRQDEVYPLIGNLTTTEMKTNLEKLT

SFHTRYYKSDYGRESSDWVLSQVNKIIKEAGAEGHVSAKPFPHTWQQSSVIATIPGKSED

TVVIGAHQDSINLWLPSILAAPGADDDGSGTVTIMEVFRALLQSEDVIKGKAKNTIEFHW

YSAEEGGLLGSQAIFLAYEEKGREVKAMLQQDMTGFVQKTLDAGKPEAVGVITDYVDKDL

TSFIKTVIEEYCNIDWVETKCGYACSDHASASKAGYPSAFVIESEFSDSDPHIHSTDDSI

KYLSFEHMLEHARMTLGLVYELGFNDFASEKQLGEL

>SoG_04711.T1

MVRLSVLAVIVTAVTSVAAGVLGTTCQCLNPDGSHCCISYGIEDCQAQCKDAGRNSHKCN

ANGKWSSVSWWTATGRAECTN

>SoG_04712.T1

MVRLSILATILAAACSVQADVWCQCLFPDGSHCCVASGAGSCQSKCQNAAKPLPWSGGAL

QPESEKCNAGGKGFPISFITAQGRTQCR

>SoG_04720.T1

MQFSLATLVLGLAAIASAAPSAPEGFKVVARQNQNRPVPRGQCCVANTSLKQDTCTASNG

QQGRCVPGGNNCGGRLSCVAQASLTCDNNVIERGKSLCRANAGNGRLFDGAQIITNLSQA

KVN

>SoG_04762.T1

MHFATLFKSTALLLAGVGSVLAAPSMEMDKRAPKSPITHLGTKGPILSSAVMTKDLVYTS

GTVPSVNGTIPEGIEAQTTAIINTISGLLEEAGTSWDLALKTTIFLANMSDFKAMNAVYS

KLLPNPKPARTTIQAGKLPGDFLIEIEAVVARPHC

>SoG_04782.T1

MHAIWTYVTLFAALAAQVNAKGRGGGSEPGRRCGFKIAPCPEDQTCVPDSDRCTNMTACP

GTCQFTNQYPRCGSRGPVGVCDEKTETCGDDPRTPNICGLACDTPGICIPNNAPSCAGFV

GQACPQGLFCYDLPNDGCDPANGGADCIGICL

>SoG_04791.T1

MKFIWLTVLAASASMASPAPAPQYNNYPATYDNYPASYDNYPASYDNYPASYDNYPSSYD

NYPADYDNYPSKRQNARRMNAKRMNAKRQYGDYPAPAGGYGSYPPPADGYGSYPPPADGY

DSYPPPADGYDSYPAPADGYDSYKKA

>SoG_04867.T1

MRTSTLLVSALAMVANAAPQYPEFDIYNMKEPAAAVENLSNWFNKVAYKAKAAGVVNQPP

ICDVSSAQMPSSWQQVLMLSVAPDSLAPPSKGLKPHHIALGRGTQNYTCADSTAKSVPQA

VGAVATLFNISCLAAVSPELVTAVANMAIHFNVDDARSRGLGPTPWPISGKHYFSAPGVA

FFNLNEGSTGGKFGEAPCQKNASATAPAAAAVGPKGEKAVAWLKLNALEGATQDIKEVYR

VDTVGGSPPATCQGMSDKFTVEYAAVYWFWSGQIAENKA

>SoG_04888.T1

MRANVTSASLLLGSLTSSVGAVKYGHNHVAVRRDSDIIQSVFEDVDIQLLSPAFLTPEIR

QDGFVNGTQGPTSHSAMDAFLEEIAGRNSYMNYHRPSFTSEEGRTFPYVLLSTGKGSLQG

RSSSGDGEASGKVRVWIQAAVHGNEPAGDEATQALLGKFDGDPGWAASVLEKLDIVLLPR

YNPDGVFYFQRTLASNYDPNRDHIKLARQQTRDIKRLLNRFSPHVVVDMHEYGTSAVFGD

GGRYQHASDGLYSAAKNLNINAAIRNMSESLFAPRIAADMRAAGLRTEPYVTGSSSSSSG

GPGFVASFAEAGTDAKIGRNAMGLTQAIVFLVENRGIGIADQEFQRRTASGLTMLVSIVE

TAAGEAAEVCGTVEGGVEDFIASTEDIVITDYSETEIRPFTVVDNRSGEITRQPVRFSST

TPAFANLTRSRPEAYLIPAAWADLAERMRVYGLEVRTLEDGWRGPVEALTITSTSIASSY

YEGVIRVTATAEVKRSERSLPPGSFLVSTRQKNAALAMMALEPENIDSFVSFNIVPVEKG

DEYPIFRVMS

>SoG_04958.T1

MQTTLALTSILAIAANLAAASPVIAAREVPNVGFGQQIRRARADTITSFAEISSEANYWV

VWKHGETACPNTRTLGPLTDSPCDISFTVPSGTENVKLCQCNGNNEPQALCFESGEVIRE

CNPKNYKITCHGDIHDIVQHGYC

>SoG_04959.T1

MMFKSLLSVAALAAVAAADYRCATNQGSFTITSAVASAARDAGGTDTNTKSRFPHGFGGM

SGNGSPNGHPHEGIQLVFYGSDSRCNAKQPPDADQSNLLEFPVFQNGKIYDKNSKREDGV

LTPARVVYLREDVNVLCGVMTHAVKNGDGSGQGDFRVCDSI

>SoG_04991.T1

MVGLSHIALLAIGAFAVIEAAPLRAAPVSNCTSPKKRQECPTDRKEKKEKKKKKKKKKRK

RKGADSCNPLKNQAIPLRRAKGGLPRGPGLHPPDAPETRPAPGREDALGRARVAAPDPRA

ADTHHGELPALPPVLPPRPRAALGGVRVRGDTTVRKPSKGTPNLFCEGGVVKGGEAKEKD

NTDARGNASPLKKTNRWWDETKDAGKFSSSTIFHPVLGFGGSGSGSHNCLTDGPFVNLTV

NIGPGFTTEPRLMWTRL

>SoG_05000.T1

MMFTNVPIVAASALLRLALAHPGHDPTEEMLQRREFIQALGRADLSHCAEKLEQRGVTAR

NAARRAALVDKAREKRMSSTVLPTVEYVGGEFIRVDVTDGQAGVPLVLDYQVIDVNTCEP

MPNIHLELWHCNATGVYSGVVAQGNGDASDISNIDATFGRGIQETDADGVAHFETIFPGH

YTGRTIHNHLLVHTNAKPFPNGTLGNEVTASHIGQAYFDQDLINEVERGATYVDNKQPLT

LNANDFILAQQANAGGGSGVDPFHEYVLLGDRVSDGLFAWLAFGISPSASRRIQPAVNYY

EEGGVKNPNAGWGPPGGGGGGPGGGPWGPRDAGEE

>SoG_05049.T1

MSPRILFALLLGSLNTCLTEGTECFAEELSATESRPQFLPKAHGSQIIAPNTPYIAASGR

DRLYFIDTCHTPQDAQHIKDQIEWAMVPRDEESYISIDEISVTAEVRTTSNEPLFVTL

>SoG_05120.T1

MVVATSFAAGLVAIAWPALVRAAAETTLNITAFTNEVKSDWTAVYYSSSPDKNPLLIAND

GGAATGGLQVYGFDAKTPLPSVKSLPVGRTKTLAVLNDVGGKDLVVSIAATDSTLRIFEL

PDMKQAKGDFTLLGDWSAMCAWKSKTQNQYLYIFGKRKGVQLLIRPKGKGFQVLEIQTME

VPFEASGCAVSESKALMFLHGDDTTRVHAFALKESTAQPDLKLAGETQHDVTGIAVYNSA

GKEKDLLFTAQADVITIYEASKDFGTSLRGTIKLSGAEGIEASGLSILQAPTQGFKQGAL

AFGVETKKFEGYGIVSLDGVFDDLYIQPNTEYRPGSRPGHADKSTICKACNNSGYCRDDM

GKKGEKKQRCECFAGFAGRTCDTPTCTDNCSNNGYCIGPGTCKCNDGWGGLHCSFLLVEP

AYETEANGGDGDDPAIWIAKDHPEELSRVITTVKSARGAGLGVYDLQGKLVQHMEAPQPN

NVDMIYGFDLGNRKADLAFAACRKDNTLCMFEMLSNGTLATIAGGSQPTPKGYKVYGSCV

YRSRKTGKQYLFVNEKSARYLQYELTAEDGRLKTTLVREFKGGDGGQVEGCVADDENGWL

FLGEEPSALWRYDAEPDSRVEDRVLVAKVGDGKLHGDVEGVTLVNGRTPQEGFVIVSCQG

VSAYNVYRREFPHEYIMSFTITDSKDGKIDRVTNTDGITAVGRRLGSDFEHGLLVVHDDA

NQLPDGTTSDDASFKIVSLGSVLGAEGVKELGLLKEVDPEWDPRK

>SoG_05126.T1

MRLFQVIIFAFISKSFSQTIEEEAPAPTVIWTRDVPRFGKREWDPTVQSEQAHPRHALVT

RQQCTLCDYSPCGPCGVYQGSDGVIACCQHDWCGSLCT

>SoG_05137.T1

MRAVFVSALFVALAVAAPQAPQVPQAPKVVPQVPQAPAAAPQAPPATGRVPSDLGQMTVG

QARSACGKNTQLTCCNDNRSGKDGPQPKGSDTSRVSRGLVADILDGVLGENGLLGADGVL

GKVADGLLDLQLFDQCSKLDITARRIPLTRAFFFGIMLANTLTAVIGVSDLLGNHCSGKV

ACCDGSESKATGGLINLALPCVALGGVIQ

>SoG_05141.T1

MQSYKTWLPVIGALSSVAATPLPLTADSPNHLLVREAADFSCFNASLPNVTVYATGGTIA

GSAGRADQTTGYTAGSIGVQALIDAVPQLCNISNIRGVQIANVASGSIVPEILLNLTHQI

QEDLDSGLTQGVVVTHGTDTLEETAFFLDLTVSSEKPVVVVGAMRPATAISADGPMNLLA

AVTLAASKEGEGRGAMIVLNDRIASARYTTKTDANMLDTFRGENGYLGKFLNIRPVFYYP

PARPLGHHYFNVSGTPSEAGLPKVDILYGHQGLETGLIQAAVDLGAKGLVTAGVGAGGYP

LSGKILQRVFNETRIPIISSSRVPAGFKQGGAPTIGAGFLSPQAARIQLQLALEVGLDYH

EIKDIFEYTKLR

>SoG_05149.T1

MHYLAPAVFLAALSGQVGAQFPPKPEGLTVLKSKFHENVTISYKEPGICETTPGVKSYAG

WVRLPPGFLNDINGEPQDYPVNTFFWFFEARKDPANAPLAIWLNGGPGGSSMMGLLDENG

PCMVAEDSKTTILNPWSWNNEVNMLYIDEPVQVGFSYDEPTNCTVHLSNDPEDSFQIIPT

DFSEGIPELNFTTYVGTMASQEPSRTANSTAQAAHALWHFAQTWFFEFPAYKPENDRISL

WAESYGGHYGPGFLRFFQTQNEKIENGTIEVDNAHYLHLDTLGIVNGALDDVIQGEAYID

FPYSNTYGIQAFNESIYQELKHNWTKEGGCRDQLQECQNRLKSYDVATVNRLGALTADLC

DNIELSCDEGAVSQYFKLDYGWFDIAHPLADPFPPPYMQGYLTEADVLGAIGSPVNFTMA

APAVGRAFASTRDMIHGGFIDAVGYLLDHGVKVHMMYGDRDYACNWVGGEKASLAVPYSG

SKQFADAGYAPLLTPDGVSGLTRQHGNYSFSRVFQAGHMVPKYQPAAAYSIFMRATFNTD

IATGLFAVEDDYSTTGPKDVSNVLNEAPERPEPRCYVIKPDSCVSEVWYKVLAGKAIIKD

WIVVGFVDDEDAENLPEEL

>SoG_05160.T1

MRSIPNFIAAGLLLAGQKAHARVLDDSDDTPLPLVIWHGLGDSFDGEGIKQVGSLAEEIN

PGTFVYTIQLAQDGNGDRSATFFGNVTQQIESVCEALAEHPILSTAPAIDAIGFSQGGQF

LRGYVERCNMPPVRSLVTFGSQHNGIIEFKACGSSDWLCRSAMALLRFNTWSSFVQNRLV

PAQYYRDPSTEEAYSTYLENSNFLADVNNERILKNTQYAKNIAAMKNFVMFLFEDDTVVI

PKKTAWFQEVNGTETIPLRERELYKEDWLGLKKLDEKGGLHFKSITGEHMRIPEDVLNKT

IVDFFGPNDREFGEPQDDSQAMSLEL

>SoG_05165.T1

MIFSQNLASLAFAALLGSSTAIAAATSNGPEFETRYHRTRARRDDAFPNSYTDQPPAPAI

LDPVIPDGIDRASKDILRLDNDPVTLAWAGSTDGLQGGKVKREASVVTQGEFDFRFPTVP

LDHSTYVKKITCKDNLLTAEMTADGYDFVKKDWASAKDVLFITAVDGCGQKDVNEFFHAT

SLTFNDNSKSVLAKGTAGALYQQIANDMKLSWGSPGNAKLKRASDKRDLNLVQIFESENG

LSSRATATGSFKTKWSAYVHEDWALGTDKDAPWDKAALLKRWEKGDGQKDDSYKKGKQPK

KKGGKRALGQTSVVETGLAERDLAKREAEYGLSLYCVECGFSGEATIWGKVEASLWSFKV

SKAEAGFKAEMHAGLNLGMEAFVKYDKSWNRDWRTPLPGGFKIPFLITVGPYISVGIEAT

AGIDATGTLLIGADVNWNDIDVTLDLKNEKNSRARGLSPTINKRAEATGELKVRAGLGLP

IQLGVGINICGGAWKADAAIQEKPKLNAEGSFQGQAAVSNNGAVSTQFADTCHGVSWKTW

VSNDLEAVGVATGLGEIKHKLMDTWNSSPITGCIGYEVQAPPQASDTPAAVEVCKAVQNP

KPPGGSVCKKVVSQSIAFDKFNVGEATEADDLAACALTCLNSGGCISFSFSDQKTCQLYN

AAVNAVALMTKNGEAQLQIYDKACFNGTACT

>SoG_05180.T1

MMSTLTALILLIAMFLGQALSLPSPRPDRIPTRPAADIVKVTLPGYNSQVQPTTNDDSYD

EDPPGNPSWQFNQTCSQLTLGGHGKKGATTLEGTCRDQDGVWWDTSINLNKCFENDGGVL

QYRDSGNFDNECRPCIITEDGSREKGLTLKCNCLNPQGIPQYTFIGMGPNCEPPASDDAV

ALLTHHNTVDHPLTVKAVAGRLICGDQIGNKSPNFAG

>SoG_05188.T1

MHPDIFRVVALVVALAQLSSAAAVGRTRTCQAAVYETTQAYAFFCRGTSTAADATXPRFT

PYGARQLYKLNGTDLATAPKTFPPAPTADLNHVLAQLRCPPVTTESNTHTNPAPTVPSAS

INHQNTVQPQQDASREPSEARISTSQGPPQASPQGSRPVLSQGLAPAGGSPQSMADSVGE

FGSPTDPFQAQGAPSTALELPSRIPGIFLETQLSAQGPISDNLMPTSLISIAQPQVSSVS

AYTARTSAGPPSLSAGPQAISSSNQLLSSIDPPQTSAADPPASSTDSPPIIFINQPASTI

QDPSVATSADPAATIIHPPVVTFTDPPVVSANPSSISSADPPSSFTDFPAIIFINLPASP

IEDPPVGSSAGPATISGDPPVASFVDPTASSTDSPAPTLANPPKVSAGRPGTSSVNPSIV

PIDPITVTSADPPAITSTGLPALAAINTRPSLAKSPAVSTFSSAGPPSSTLADPFPLSGG

PTESAMGASLPTAVGSTTIRSSSSASASTQPHSPHTDSRSGSPAIFAQKGEATLSEASSL

NNGAHPSASSSAFNSVGGNTTFGPVGASSPGAMAMTYNSPSMLAFAVTADPLASVAVSSI

SQLTSGSNPVSPSSPANPAMTSSSSDASFSLPLTSYTGMVSSTAQSVSFASIVDPSSSDA

FSSSAVASTSAVKTAAPNNHKIFTAPYWSDSAVASATGSRKMTSESSSLLAPLSTYFSQT

LVALPSNGTNAQSSLLSLLATSIPVTITLTPPGHTGSLTMTDSVEASSSQTETVTNVFVS

SSTQTQASITGTPQAQILGQTPSVGSDPGSSATSANLRYVSASSTAQSVSISGTISSETT

ATSNFASQQPQSPTNTSSFYAGGSVAQSVESKTPHASASLVNRPAAHVAFASAFTGSVSL

NATNSAAWTINRGSAASTIDLSTLSAAGSVRMSGATSPTTTINSHDAGSTPTIGSSTTTP

GSSSTRTSADTFTASSNVTSIANVTSTDTSASSSIRSGFAPTHAAILNAATTGASTVNSP

AITSATTDSAGGSVSATKSSAINATVAVTATTRPSGADLSFKNTPTTVISTQNTPTTVIN

YSTPDPSTPHASATNSTATDNRTIDFSIMNAYVSYSTTPNTFLSTTWNASATDAAAASPS

TAYPTISNNSIAPNSQTHSSSESLSATRNSSTSSSTMDHYTAQTAATETATTIASVRRAG

AIGIHTTNTSTGDTFTTNNTAIEASTTTSLEITSQEFASAKISATVSPDSSGISFPSNTT

ADVISMGTAAVDTSATNATPPTSRFTAYTGSVLLDTPSVSTPATGESLATVAPATEQSSA

LSSVTRSTTMSAEATVTLSQETSIGMPPHSPAAPGAQKFQVSLSTSEAAMESTSPQSTDP

SSTAGPTPSLSQQPIHISKDSASPSSAASLNSPSTVSDTLMSFLATSSQGSVASLQTHSS

MSGASTPAGSLDITMATSAPGPFGTPVNSLISSNDPPTATSSGSGYLTTASEEPFSIAQY

ADTGGAAKPSYSGSLISESSRVLSNGISSGSSTIMTSVTFQESTTSTHASPEESSSSTSI

SFGSSTSSQVSSEISSVESTSLSSTTSAPQGQDSSSTDSTSTEAQLSSSLSEIEATGTYN

SATMTPVAVYSSLSELPNPSTQLPSDNWMNYAVSTMHTSNQDPINTQSLTTTATTDSLYS

APGSTLSATIASSSQNLLASPSSATDPGTDSSTLPLTTLDALASFGTAGVKVAEMIQSTQ

PLAVPSSQALTMTSSSAFSSASSVMFSDTSTSTSTSTSTSTSASASSSTSTADSTDITAS

ADATTSTDATTSTDATASINLAAFISTDTSTNTAASTFATASTSATKSRSATKSIQIHVQ

PKKAVCIHIQTDDDIHIYIQTNNNVHIQNMDFNYHFESGPAKS

>SoG_05201.T1

MLSIVLLVAILSLAQAHKDVVKVPCCPAKATIAYDKSVPDKKPFYLTQVDLCYTPTTIEL

TFTAYNETSFYFDPSQGTNDDIWAYEVMEAFIHRGTNDPQTYLEFEVNPNNATYQAFIYN

PSKIRAPGAAFDHFFVSDPAADGFSARTTLDKRAETWVSEATIPLGLFNVDEGMARGTHW

RMQFLRTVTSPETYPDQELGAWIPPDVASFHITPKFGHLEFV

>SoG_05211.T1

MLFPKRILLICHALLVTAQEPLQRMKIPGVGFSLTPDHGIAAIYYKNGTWAEVARVEGSS

TYKALMRKAPSTELVPDQLSSWPVMTEVVCPALDIFKLELCKRSPDVESAKDLLRSLKAA

VASRLGTTFCYAQIALPDREWAYQANVVNLALQSTRLRPILDAVNEAPMAFWGSELAATG

RPETGDTRSILIVDYSESGLSASLLAEDEGVVDIMRKNRDESLGAKNRAQPDHQKQVRAF

LEKITERPFGNDYFGQPMYEHLGKIVLHGDATTDDEFLETFRSVVGSEPPDAHGLDPAFA

VAVGAAIVGHDRMDDWQFLLKPALGCTWLSGLHTDKGEL

>SoG_05244.T1

MRTKSLVLATLLAAISLTVQGFPSRPNDIGRVETAEEIVSILNTRQHETRNSYNAAAVAS

ASDLTKRGGPNPPSNCPAGTGGQGCQPCQPGTYNNGQSNVCQQCDAGSVASQDKKSCTKC

AAGTYAERGWAQCQKCSAGQSSLEGACSSSQCFDCKAGSYSQQGGQCTPCPAGQYSSSDR

ATSCQPCPAGQLSTTAGSPQCDKCPAGSFQPDIGKTSCKTCDAGTASKNPGSSQCQQCDP

GSFSKAGATKCDRCPDGQISDAKASECHYCDPGYGAQNDQRVCVKCQPGWASDGTKACSQ

CTAGSYSQGQGSSQCSQCPAGSYSSAAGSQGCTNCQPGYTSDAGAQQCKSCPAGSISTNS

GSPTCTKCNAGYYASSGTKCTKCSANQWAPAGSTSCQDCPVGQTVNAAGDGCTGCPAGQY

ATGDCNNPCAQCPAGTSSPGGTTTCTPCAAGSFSDKGASTCSPCSAGTFSDKAGSGSCTP

CAPGSYAEQGKTTCTPCAPGSQAPDAGSKCCKPCPDGQTSSGGVSGCSACPAGKYSTAGA

PCTDCAKGTFSASPGASSCQACPKGQFQDGTGKNGCSPCPAGKFSSADGAVSCDACPSNK

VSLSGATECKSCDSGTGPKPGQDGCTDCPAGTYGDGSKACATCPDGQFSGPKSTTCKTCD

AGSIPNANGDGCTPCANGSFSKPGDKVCTPCDAGKVPNAQKNGCDTCKAGTFAALGSKVC

TSCAPGTYSLDGAPSCSKCADGSIPQPGQGGCKTCDPGSVAASGSPTCSFCTAGQQPNDK

NSGCETCTGTLVSSGSQKCYACKADQQPNASRSGCDKCPNDQVSDGTKACANCDGGNEPN

SDHSGCNACKENKVSNGIKPCDWCKPGQEPNTQKSSCNPCPDGSFSDGKSACAQCGVGYQ

AKNDGSGCTKCDPNQVSTGGEKCAPCAAGLVPDGNQASCVPCEKGTYALAGAATCTDCAA

GSYAAQTKSDSCQLCPKGTYSSGRASECTQCPPGQFIDVEGSSQCNNCGSGTIPKDDHAS

CQPCGPGQVAISGSPKCTDCGKGSAPNGSKDECAKCPAGKFSAGNSDTCTDCPPGSVSTD

VGAESCTSCDAGNEPSTDKSTCKPCAPGQVSGKGEPSCHKCLAGQQPKEGQAECQACPEG

TYSSVDGQPSCQQCSKGSIPNESSTNCKACGIGQVASPGDPQCKTCPAGQIPNIDKTACV

ECPAGSISAGGVNTCTSCESGSIPNGDKNKCDSCPSGSVAPIGSTECTACTAGFAPSEDK

SVCQVCPTGQIAAVGAPSCTSCEAGTVPNRDKDGCQQCDAGSFSSGSVAICTPCLPGEFS

EKGATQCTKCDAGSVPNQQHSACDKCGEGKVAAKGDPTCESCCAGQAPNADQSACEVCKA

GTYSTPKGCTKCDVGLYSPDSASSCSACPAGEEPKSDQTGCQDCAAGTYNPGTSPKGGSC

LPCADGEYSIAGSKSCTKCPAGSVPNKDQNGCEPCGPGQVSKEGEKTCTICPAGTSPNLD

KTSCDKCPANQVSDGKAQCHFCDAGNAANPLQTDCVPCADGLQSDGKTPCGHCQPGNQVK

GDKTGCEVCPKNQVSDGTKPCAPCKADEQPSANKDTCDSCPEGEVSDGTGCAKCDAGHQP

NKDESGCDKCDAGKISNGIGKCAPCPAGSFANSAQDGCTKCDGNKVSDGDGTPCHFCPAG

SAASVDQKTCDACADGKQTDGKTPCSGCPAGQQAKKDHTGCESCPAGSYSPEGSDTCKPC

PAGQYSSEVGSKECKKCDAGYISSAGSTECATCPGGQFPTVDQSTCQDCAPGKVALPGAP

TCTFCEVGKIPNKDKNGCDACGEGQTAAPGDTKCTTCAAGTSPDKLNGVCRPCPPGQVSD

GKGECKPCDKGSAPSKDKSTCDKCTGGQISDGKTPCAPCSPGTQPSADNSECECCPEGQF

SDGKTKCASCPPGSFIKPNGSGCQSCEAGSYSDGTFACRLCPGGEYSAAGAATCTKCAPN

QIAPPGSSTCKGCQPGQEPNHDSNTCAPCKPGQFGDGTRGCAPCPAGQKPKAGQDGCEKC

PAGQVSNGNGICHACPGCKVPNQEQTDCVSCPPGQYSDGKSPCKQCDKGSYSDGSTPTCP

KCPAGTFSDGKTPCKACPAGSFVKDDQSGCKSCPSGKVSDGTAACRSCLPGFKPNEAKSD

CEPCPDGQVGNGESCRACGKGQVPKSDKSGCNDCPAGQVTDDKGQCSFCPPGKAPKGDRS

GCESCPPSKVSDGRSPCKPCGKGTAPKDDNSDCQPCAALEVSDGDSCKPCPPGQKPKADK

SGCERCKDGTVLDGLGGCKTCGPGSEPKRDGSSCSTCAEGQVSDGTKQCSPCKDGEAPNQ

DKSACTPCTNGQVSTGKTCSACTGGHQPKTDKSGCEPCPRGLVSDGTGACHQCGKRQQPR

PDRSGCDDCEPGKVSDGTGCASCPPGQQSKPDQSGCEKCPAGQVSNGGVCGPCLPGQVAK

LDGSGCDRCPLGMPKDDQSGCDKCQPGCISDGKSTCTPCKKGEAPNMEAGACMPCGPNQV

SDGITCKTCNAGQEPMGDKSGCHDCDPGFVSNGAEKCHRCEAGSSPAPSKKECQSCPVGT

FSDGTASCATCPPGNKPKADKSACESCPNGGVGDGKTCTTCKPGETPKKDNSGCDTCPPG

SVGDGKTCSTCKPGQRPKADKTGCEECQPGEISDGTTCSTCPSGKKPKGDKSGCEDCKPG

QIGDGKTCQNCKPSQKPNKDNNGCDDCPPGQVGDGTKCQKCDPGSKPSQDKTACDKCPPG

QVGDGITCSACTGKQVPKDDKSGCKDCPLGSVPKGDGTCEACGRGQVPNQDGTVCIPDCG

THAKFDSKVNACVCDAPGAHYNGDQTCSCPCDSAALNTKGDACVCSSPEGSTWSKEKGSD

KYTCACPDGQTVKDGKCIVPPACGDNAVDDGHGGCTCKAPGASFNKPNKSCTCPEGSTLS

KDGTECHCNSPKDSELTKDGSGKYSCTCPKDKPKNENGKCVAGPVTCGKDAIPDGKNGCK

CKAPGASYSPEDNSCSCGDGASLSKDGSCCECRTPAGSKWVKNPKGDGYSCACPADKPLK

DGVCGSTPAQCGPSASLNTASGKCECKAPGAKFLDSDKSCTCGPGAKLNKDGTQCECSKP

SGSSWTSDKDGKYSCICSPGQTLKDGKCQGAPSCGENAQANNSGVCVCKAPGATFTDKKD

CICGPGAALSPDGKQCVCSSPTGSSWTKDKNGKYSCGCPDGQSAKNGACAGPNPPAPGCG

KDATPEGPGKCTCKAPGATYNPDKSCSCGPGAKLSPDGKQCVCSDPSGSSWTKDKNGMYS

CGCPDGQFVKSGACTGPTPPAPGCGKDATPDSKGNCVCKAPGATFKPDDKTCGCGSDAAL

SKDGQSCVCSVTGTSWTKGKDNNYGCVCSNGEKPKNGACTGPTPPAPGCGKDATPDSKGN

CVCKAPGATFKPDDKTCGCGSDAALSKDGQSCTCSTTGATWTKGKDNKYTCACPEGQQPK

NGACSGPTPPAPGCGKDATPDSKGNCVCKAAGASYKPDDKTCGCGADAALSKDGQSCVCS

TTGATWTKGKDNKYTCACPEGQQPKNGACIAPSPDCGRFATPDSKGNCVCSAPGATFGSD

KNCSCGPNASLSSDGKSCVCSVKDASWNKGSDGKYSCACPSGQVVKDGCCKVPETDCGRD

ATNQNGQCVCKAAGANFNKDKKTCDCGPDASLSGDGKSCVCTVRDASWSKGSDGKYSCAC

PAGQVIKDGCCKTPELDCGRDATNVNGQCVCKASGASFDKNKKTCGCGPDASLSNDGKSC

VCSVKDASWTKGSNGQYSCACPSGQVVKDGCCKAPEPTCGRGATNVNGQCVCKAPGATLN

KSDQSCSCGPNGTLSQDGSSCQCSCKGSVWSKNKNGEYSCQCPDGQIDNNGVCQPDCGKN

ASPDNKGACQCKAPGATFQSSDKSCACGPDASLSKSGDSCVCKASDAKWTSGSNGKYSCQ

CPNGGSLVNGSCQPDCGSQAKYVSGGYGPGSCVCNNSNLVYNSNSKTCDCGSEATLDPKG

SNCNCNKSGMLWGAVSHTCACPRGMTFTDGKCQKATPGGYGRL

>SoG_05247.T1

MKLQATPLAIAMLASGADALFGAKEPPKPKPHIIDAFKYADPYLHPKIDSFEATCEHTVI

FPAREFTLADLSVAAPKGLQEWGPGLKKLFTGREYPGSWGGLDRHLNDRSILTMEYKDVP

LEVRLWIEEQDRSNGDGKGLFGVFDKPRDGDSKIENTVEVPSADEVDRSLDEQRIAVFAP

GALYHILPIWAAGTSPCKDTMSDLSKYKTEPEDGAVVGWLEHKKPKEKDIEFRIRMRALK

AKAADASGSESTSEAKETEEAKETKAKGEKEEFYRKLKERTVVELDTAIMVGEWDKLSLV

APEPQYRATMLCFMVLMGLVMLDLLRRIGILVWG

>SoG_05274.T1

MYWLETVLFLAPTVWGATLAGFMDMASARPQIEQAGDCQLPAGFMIKNFVGKSNDTGSTL

SSFDFTFRDETTDVTALCQFNSSSESTTPGGLQKRYACNDGQTKFIWEDQGGQLWMIERV

CMGDSGTPRYETAGSIVMHLNCDKQSQQCSSNATEVSARFSSISPIRDPTLRRVRQPLDG

>SoG_05275.T1

MRHPTSLLHLLWLLQMAAAAIAVDISLGQRSFRETLLNPKSVTTHLFAELERLARLVDIS

YCIGTTGVTKPFNCASRCKEFPTLSLEATWNTGILLSDSCGYIAVDHGDRRPDIDSDVPP

SELGHKSIIIAFRGTYSITNTIVDLSTVPQKYLPYPSPDHGGDDPERPEHRCDNCTVHQG

FLESWQQARKLVLPVLEELKDKYPDYPIRLVGHSLGGAVACLAALELKVSLDYDNVIVTT

FGEPRVGNYEFARYVDEVFSLSGDVVPEDSSYRRVTHKDDPVPLFPPTEWGYHPHGGEFY

ISKRDLPPGEKDLQACTGDSDPACSAEGDGSLTDAMRRLLPWAKVNGVPEQEGQVEEKAK

GFPTRFKLWQLFFSHRDYFWRLGLCFPGGDPADWGRDRYDLVNSDQL

>SoG_05281.T1

MRYFYTIVPFAILSLALPARRSPDDDDNWYLVAQFDNYDPGSDAWHLYDSTDLTNTKRDL

EPQPPAAEDYVPYGADPDGDWRWGLEWKMKERRRSIFSLIGDIGKGIGSCVSDLVSGDST

SNCLAGGDKKPPPAAPNGPLFNPIKCVEGLVSGDAEKECDGADQLGGVAGEYPPAWQQYQ

LQPLVSGV

>SoG_05282.T1

MRYSNAIIFFAVASLALPTRRSPDDGKDPSPEILDDPGGHGSCHYVGTGDAGRKRDERKA

PNYDKRDCWSHSGNESPVLHADTTRKRYGSEVRPGYEYLPDFTLHSKDAGHYKRRDNGVD

ADASHCLLDQP

>SoG_05290.T1

MAPRSLLCYALSAAVALIPTGALSSQVHPLWHISESVEEPISSSHPYHKIEIPIDHFHND

SRYEPHSFSTFSNRYWINDKHYKPGGPVILYAAGEVTGDARIPTLDHGIVDILTKEVNGL

GVVLEHRYYGLSYPVPNLKTENMRFLSTEQALADMAFFAQNVVYPGYEDQNLTASDVPYI

LWGGSYAGAFVAIGRKVYPDIFWGAISSSGVTAVIDDWWEYFEAARLYAPGDCAPNHQRL

VHIVDNILLSGDEGKKSTLKKAFGLEQLNDPNFGYAIARGIFGFQSVVWDPAINEEDFSM

YCGALISPAVLYPNTRHQGDLAAKIIEWGGYEATEDVKIQFMNLLGYIQTKFAQEMGKEK

VPNVMSLMGKKENDTSDTDIPQGMLRPWIYQTCTQWGYFVTGSGTPKDQLPMISRLVDYD

YATAACRDFFSITAPPNMTSINSLGGFNFSFPRVALIDGEADPWRQATPHAIGLNEDRES

TADEPFILIKGGALHHWDSYGLDEDDFGPGLPPPEVEKCQKEEIEVVKRWVKDWEKKHES

RVVEEEVQKPLGLEL

>SoG_05311.T1

MRHIGASPVPLALLALAGSVGAFVVPPVQLPLNSDAQRGVVDDGASSAPSWWENLRDLAS

ESSLTPHVPNPDDLAAALEGKVESISAGAKKVAESLKDLHSDIVDFLREDDNDNDNESSF

YASGRGGRHEFPDHTIYDLIKKSNYTKKFAKAIDDFPKVVKILNSTDAGNHTLFVPVDEA

FEHIPHHGDKPSKEFMEAALLYHIGLGDLSAGKLLHANTVPTAYDETWLGGEPQRLRTSI

GLGGLKLNFYSKVIYADIKAKNGIIHGIRELLIPPPMVGRELTWFPGEFSTLLLAYEKTN

FVDFIHNVKMVGSTVFAPSNKAFQKLGPRANAFLFNTETGLRYLKAILKYHIVANATLYT

DAFYDKTDSSAESSIEKREHFDLVTLLHDLHVSVDILEWASLKFVTVNGFAGVQLRDAVA

KNGVIHVLDRVLIPPHKHGKNAEEDLLSNGIDVDELKERLEGYI

>SoG_05312.T1

MRSPQLLQLTFTALSLASTVFAIAEPKNMSLPEDGPLDFQTVALLTEAADGQVPAVLSKK

DASLKGSPRSLCRELRRLVTPFVWPQSENFHSEALVTVVDEMLDALVERQHKDGTFTVGN

RHSPPDTGFLIEDFGIMARLVDADDHPKSAHWESKIRSILIKAGDGLAKGGIHTPNHRWK

VCAALARISRIIGEDDIIERIDEWLAEGIDVDKDGIYSERSANYFSAVSNPSFLTIAHEL

NRTELFDYVRKNLEATIQLSTPEGEVETIQSRRQDQAQEPFFITPFYAQFRELAIRDGNG

RFAAMTRLIEEKFRASLGDYLGAFMERPELFMELPESEDPFGDFKKHFDEVSLVRERRGK

LSSSIFGGTDFYDAEGKRTDLFNVFGSGLSTNPTIFRAWNGQSVLEAVRLVPNFFSMGHF

RSAGLEYSQDSGAAKLHQELDVPYYLPIPPDQRNKNGDYKLSKSVDGRFFSKLDFGNRPT

DMRHLWTNATIEPTEKGYDISFQVDGEDEVEMTLELTFRKGGKLEGVEDAEDEEGNKVFH

LKQGTGRYSIGEDSITFGSGNGEGLIMAAPGEQYGWLKGSLKLNGEKVYITGTTPWMYTL

KLEFS

>SoG_05319.T1

MIITKTIGVIALALGALAAPYGPEHPSLETRQTSNPHWYKYWANDKAVVQAQNLQGGKFI

VEWNEPAGGNFVIGKGYQTGRET

>SoG_05346.T1

MAHNLEGFIWMVALGMTLSSEALPMACEDIDEGSWKAILQLQRDDFERFSTMKRHRHGQI

ARDHDFAMRVWAEELDGLDAQFHAPTRFRDICCLRRMIMVSMDNEAQFPPRCCGQNILRA

SIKKVLTRRDWSKYKLKELELRTPNRLYCHQCSTFIPPNRVESDVGRCSKCWLLTCAFCK

KKSHYGDCPKDEATARFLSLARKKQWQRCPSCRTMVEKTSGCLHIQCNCGFHFCYGCGSQ

YSSCDGLCGGGNQEFEPSSDSDDSSVASTDMGSDITEISIGVPDNSVEDASRPKCPSDAP

WATVEGGITCLSTVLALPVIVREDACIHPCPPTSTCDQSITQQRNGTVASMEHKTETREE

KEPLVLQQTPRHDFNFTAKSLSTDLAAIRAQSSENLPGDVQAKGSTASTSRQAVRGKRHT

KAVSEGNVRRSARLAGKPAELKGLPQKQATRKKQPRPTPASGSPQEGGTIQAPQILDSQH

ILHRFGGHKRTSLSTHIESVPHAAPMFLCSRPPPALALIRIAPRASSGCFVKP

>SoG_05358.T1

MHFQLTSLLALSAASLVAAKKDCGSKTGWETAYTASGTAAVAAAAATAKTSSPTSHVKGK

VFDRVAFIWFENIDYDLAAGDPNFANFAQQGIKLTSYYGVTHPSEPNYMAAIAGDYFGMQ

NDQFNRAPRNVSTIIDILESRGISWSAYQEDMPYSGYEGYEWVNHQNGRNDYVRKHNPPV

LMDSITHSERRLSQIKNLSMIDVERSQFHKDLKDNKLPQWMFITPNMTSDGHDTSVTVAG

EWCKTFLTPLLSDPNFMQNTLVVITWDENETYAKSNNILGVLLGDAVPKNLVGTTDDTFY

NHYSELATVQANWDLPTLGRWDVGANVYRFVAERTGDRLRQWSKAGNFAGHYWNQSYAGP

FNLNGGNGRYAAPNLKLNQGTWAGRPIFGKVMQTWAGSDAPAYYEDTIELPDGRNPPPGY

APKK

>SoG_05392.T1

MAILSWATSLLALGSAVNAASLAGRSSNGRSVKLSANAQSLFDYSMSVSDSRYDSSYNYI

WYQDNGQWSVRFTSWYIPGLLKRGKPADIKAAIAAIENVLANQMNDDFNAPWYGTFKLSP

DEPNPTPNGPLYPPDIYGSYDPNWREFVGSQLIQVVEEFEQLLPRPLVSRIENALEAAAV

GGMRRNGTFPEGDNLILGYSNPGIMRALTTGWAGKRKNNKALVDFARQQGEDLYKLFQRD

QNALAEYNAPNYYGIDIWALAANVAYGPADAPMTKNAQKMLRDLWDDVAAHYNPYLGNMV

GPYDRAYSRDATTHSQILSMIVWGVYGRGVFGQPPLGEGDLLYDIAQGAALALVMDVIAP

TISKEAQSVITAKSEKWKGSRSVKKTIYEDLTSTNGRVVTSWLSSEVMIGGQTVKETKNR

GNQFVPAIVQWAADPNHKPYPYMGPAPASSPSPSPASRLPGH

>SoG_05435.T1

MTQPCRASSFQLCLANLTLLDSNLFSLYKSFSLSRSFSGVAPSIITEQSSSPTVIRTTTE

EFVSIKTFSLDNLLYLPRGTILHPLTSLLLSLGLILSGKEPSPIRWGSGLASTQWLGLSS

SRMRLAAGCVIAAWVLSINRVLSRRAPNPSRKSRTDIKRKVVAITVAAAGVGAALEQKLD

AAGATVIVLDVSALSYTPGRRTPYVKCDVSDPASIREAAAKISSRHGVPTAMVANAGLVR

GHALLDASDDDLRLTLDVNLLGLLWSIRAFLPDMIAAGRGHVLATLSATAFVTVPSMADY

SASKAAAASLIEGLRTELKHHHGNPGVAINAVYPGTISTKMFKDLDSPNLFAMPMLSPDA

AQRIFDILVSGKRWATPEHRLRTGENAYLPSASTAHAWLRVLPHWMFVGMQDAGAHAAEK

LGKKK

>SoG_05558.T1

MKPPVALLSAIALIHLATASPSSPGLPLIANVWSGPFESATNAAFLSLLPNSSTCGSPTP

ALDAVTIGCTVCENEQCDGSVGFGGSPDENCETTLDAMIMDGGTFNAGAVAGLRRVKNAI

GVARAVMEHTKHTMLSGDLATKFATEMGFAEESLSTGRMEERCRAWREEKCQPNYRVDVA

PDAGSSCGPYRPLEKQKSKRQEARQFGHDTLSLIAIDHRGRMAAGTTTNGATHKVPGRVG

DGPIVGSGSYVDSDVGGCGATGDGDIMMRFLPCYQAVESLRRGMTPQEAAEDAVRRMMDK

FPDVSSGLVVVDKDGRHAGAASGWGGTFTYSFRGGNMANAEVVSVPNLQPGKREL

>SoG_05563.T1

MRSSSVLLAFLGGVSLSTAAPKALGIRSEAANLGSLIPRDTCPAAEDVLIEAPKANPFSA

ITPEELASVIGWLTESSALKLNLTNSSSPDLRISDNYIAHIEILKPNKTDVLSYLYGDGK

VPRYARVILNHGAAEVPHVAEYSVGPLPIDCHTKLESLDWMYNGPNGAKVLFNGRFMDRA

FGKAIDKLVNSTMMEINDIVQELTGFAFYGRGDERSTGSYFITNPYSTDGTTGVGWTVWR

KAGLAAYGRPSELYMSWDFAGNDPSLWKLRMIVYDLKVYHSTAEFRSAWQAGGIIKTPSP

SVSDEYLRKDRKGPARELEDRLAPVMLNMGGKRFRIDKKNNYIEYMGFKFYLRFDRDVGV

QFYDIKFMGDRIMYELSLQDAIAQYAGHNPFQAATAYMDRFYGIGLQAGRLVPGYDCPYG

ATYLNTTYSQGEKIFHQPRNICIFEADIGTPITRHAERQYLQSTKGSKLVVRMIATVGNY

DYLWDYGFYVDGTITVDAHASGYVQANDFRPDDNGQWGPRISDQTQGTLHSHVMNFKVDF

DLIDENNSFLKTNIIVENVTQPWFPERGEFEMMRYDFEEVKTEALLPITPNGQTMYTVVN

KNHKNKWGEPRGYRILPGLSNVVLPSKLSPFFIKSAEFAKQPIAVSRQHDTEPASSASLN

QNVPEAPLVEFWKFFDGEDIQQKDLVAWVNLGMQHYTRSEDMPNTLMSEAHSSIMFAPQN

WGDSELTQNLQNAVIYNAVQGEEEAAVVANTNGVQPPSCMVLGPEDSLVGVFEAGAVDKP

AAPHNGYT

>SoG_05596.T1

MKIPVYALALAAAGIIGPDVADAARPANPVYVNISRDTSVSNYNAPLGSQDRLTNVPAGI

TYEATNGPIHGLHGNWNISGLVRTRNQQGNLGMSSNLWRQLVGTGSWNAHMNVSSTGVVH

INDIGSRLPSDLVWYPSTLYNDGLMQWCMSGAVLGWRGTFFPEYDWVNRGVQHWTQDRGP

RHKRLVLANRVANKGQIVNDGTMIIDNLEGFQVASNIVGNGCWKITRNSGLEIRSTHHNN

VPGQVPWTGQSIYMTRSPNGNRIVFARSSWKRSQNFAGRIYGFGSDNGLCFDNNPDVWRY

LPATGILEVTVRRETLSLNIGRGYDNRAIRVVKRNGSHCIVSSQETPDIPDLPETCKAEA

PECRFRGETGLLS

>SoG_05614.T1

MLLSPLPLLLLLTHLPSLTLASEDAVAVADSPELLASTTLVEVHETILTFLQPKLESNDD

DNEVHVQEDDGFGHSKRHEEEKQRLMVRMSKSTGNYNQHHTRHRLLDAMHGFLRYYERQN

EEVERLQGLYKSVSKYQNQVRASPQSPFPHVTNAPPPKKILENHVSYSSRFADIQAHLRK

NQALCDQIVESGLAFYGIPRSELDEHIKSVESGGGRGEKTSVSQGLKHIVRDWTTSGARE

RANTFSCLLATLENLFPNRTEENPTKILLPGAGVGRLGHDVADLGGFEVTTNEWSMYMNI

IYRHLTSSPSVAGPNSTVFHPFLDSYSHHLRRSSQTRQLTFPSTRVRPSAVLLTEGDFTS

VFHREASRYDVVLTYFFIDTARNLMSYFETIRSVLKPGGYWINLGPLLYGTAPFVQLSLE

EVVQVSEALGFEFLETEGELCGVPTFDGTAKVRGMDAVYSFDEMQLTRSAYLAQFWVARL

SK

>SoG_05636.T1

MHSAFALVMPALVAAAHTFTPGPGFDVREATIGSIHAALHTGASTCRDVVSAFLDRIEEY

NPMLHAVLSLNPAALEDADHLDSHGQLWSLDEPLHCIPILLKDNYDLAGVPTTAGCKALA

RLEPISDAPVVAALKSAGAIILGKVNMHEMALEGISVSSLGGQTLNPYDLSRTPGGSSGG

SGAAVAANLAVIATGTDTVNSLRSPASANNLFSFRPTRGLIPRAGVIPVAHTQDALGAMG

RTVRDVATVLEVMASTGADPADNATASRPQEIQTKDYDAALFGGSLKGKRLGILSGGYNY

TSSPEITPVLEAMAGIERLLLEEGVELINITTPAFDMASLLPKVDVQTFEFREQLNEYLS

RDDLKGEPRPRSFEELYGGEDFLVIPQQYDHIKSSHTRSTSDELYLTKQQSIRELTAALH

EEFRAHRLDAIIYPEQRNLVVKVGSGSQVGRNGILAAVTGSPVVVVPAGWSEPDEDAPLG

VPIGMEILGMPWTEDRLLSIAQLISEKTPALRKQPLSAMASVAPKCYAQVPKLRLIEAKP

DMPYPVGVFR

>SoG_05638.T1

MHLNVCALAAALLLAGEASAKCVQECRHNCLHSLVRSDLKQRVYNDCEEYLAVTVRPFAS

TTTTTVVLATTSVATQDSTAVVTLDKTTTASTQTNFLSQTTTLDIATSYTTETVYTIKTV

DAKPKPVRARHLDGLGARTPLPQDRAYPTYASTCGSSDRYSTACSCIGARARTTTVSQAT

VTETSTQYTPVAQTQVVVHNVTSINYTPVTDTVEQTTYIATQTSYSTTATTTEFIYTATV

TSVVRNGDFETGDFEYWVESSDNQIDFSITQPGESGSKYALETGELYDNDLLEFYQDTLA

EAGVRYDCTWDMKFTDYYETKYSNGKTYIPYCHIYLNRDTSYTDWNSPNGNNKGTWLTST

FSFTAIGGDRLWFDCASPQPETGDGSGPNKLYLDKIACIEASN

>SoG_05663.T1

MRLCLFASIAAILTAVGSQQTPFHKPSPSIQTLPSLREQSSLINNWVAKRKKLIPHILRS

NKAEAWLISMLEHGEDPVFWLLKSAEQFSARRRTVYLFLADDSGGRPFEYSWIDNTPQVW

TDLAEVIEERNITKLVTNIDPKIAFSSGLHHGEANEILQGLERVSPGQISWADRLVNVPD

VANRFVALQIDDRLPWYRKLQESAWAIIQDAFSEKVITPGTTTTTDVEWWMMDKLQLHNF

TTWFKPDVSILNSREYVPGDPEVPDRIIKYGDLLHVDFGVTALGMNTDTQHLGYVLHPGQ

TQEDIPAGLADGLRKGNRLQDIVRQNMLPDRTGNEILKATREQMHAEGIEGRIYCHGVGD

FGHSGGTVIGMTNLQDFVPGLGEAYLGTRTWMSVELYAEHFVPELGQTMIFPLEEDVAWD

DEKRTFEWVYGRQERFHFVHTPAKVPVSDDL

>SoG_05669.T1

MKHFLMKAVLPLLAFVVGGLTQCSPESTTIYPILSDGPSQVQFTSCASGADGPRVLPING

TTFDWWYFDAVSDDGVHALTVIFFTSSFVGFSFDLMNPIDPLNVYVFYNNGGDGFSFPIS

STSVTLQLKGNGASGDWNGSGISFKGAPDLSTYNVTFEKTPLNPSIEGTFTLKSHERVLP

HVGWVNVMPAADAAVDVTVSGENIKFTGNGYHDKNWGDAPFLTSLNGWYWGHGTFGDYNI

VFFDMLDTDNVEKVGGYVLRDGKVVGSTCTTGLRVRPVGTPYPPTLDSPNPKQMTLNMTL

DDGTVLDALLTAKQTQIDIGLYTRWIGSIDGTVGNYTASGSALWEQFKVAKSA

>SoG_05675.T1

MKLLAGLVLLFASVEAKKLRVCNAPSEACKSISEEDTQFCLNFIAQNKVCVKPCEAPGPT

VTTTLYGVTVTEVVPNPVVTEVTTTTVTQIGTPVTPSPVTVTTTSTSITKTTQVDTTIQT

SYTVSTTTQTAWTTSTTSRTDTVSVTTTLPYKFPDCRLEDRKRYLKKKRAQVPAQCSCFL

TSTQGYAPATTVTATNGQSVTTKTSYASASTTSTTTQTQTNTRYNGGLTLAAQQTTKVVF

TTVTQTDRTLTTLVELKTNTVTKVVTSTTVTTKISTARATETRRPPNPCDTQEPQFQDLS

LIPNEDVTQLVGNSDNAQQCCERCFSMPNCFYYSSGSLGCRIWGLIQSTDVCPSEQCPRG

LGNLIYNDPDGQFYYTGMCFRNVGA

>SoG_05735.T1

MPVLRLLLHILSSLANKIGIPGHGIHDEEHLGLHHVSTRESDEEDGTRGHSSFIPEATDD

PDATWGHRNGLEDDEDEEELQEGMLARDLPKRTTFYDPVAERQMSQMDAKLFYQRSKLDL

RSGTWTQTTPAESPLLVSGSQPGTEYGADSLVLDEGRLGDTPWSSSRPGSVESMAGLHMP

IRGQDASNLSCQASWKASQAAAETKAAHAASMGLAGSSLFDTEPEITAELSAISKNIQKI

LDIRRKYITLSSQGPDDNPRDHPHWDIYPPPPEPAWRQDVGEESTGATPVQAGLGDILEK

PEKQRQPRSRKPGQAVGEDFNMEDLLPLPGDEDWTFKLDESGVFQVFENEESKEPAIGVP

TIREYYMVLDEILTISSDGPSKSFAFRRLQYLEAKFNLYALLNEYQETADSKKVPHRDFY

NVRKVDTHVHHSACMNQKHLLRFIKSKMKKHPNEVVLFRDGKHLTLAEVFESIKLTAYDL

SIDTLDMHAHTDSFHRFDKFNLKYNPVGESRLRTIFLKTDNFIHGRYLAEITKEVISDLE

SSKYQMVEWRISIYGRTIDEWDKLAAWVVDNKLFSHNVRWLIQVPRLFDVYKSSGLMDTF

EQIVINIFQPLFEVTKDPSSHPKLHIFLQRVIGFDSVDDESKIERRLFKKFPVPKVWNTK

QNPPYSYWIYYLYANMASLNYWRKKRGFNTLVLRPHCGEAGDSEHLAVAALCCHSISHGL

LLRKVPLLQYIFYLDQIGIAMSPLSNNALFLAYERNPFHTYFKRGLNVSLSTDDPLQFAF

TKEPLIEEYAVAAQIYKLSPVDMCELAKNSVKQSGYEKSIKNQWLGPNFDKPGKQGNTMV

KTNVPDRREEFRYHTLLQERDVMRRYVTYDATTDTMTPAATISDYSVAKPSAFASDHTAT

ISTSRRGPSTECLPKTQTWAGGAVVNDPHLSGDDPKMFPGVLTRGRRKNSLRNLGQADDV

TKAGHEGSRIDGK

>SoG_05745.T1

MKLSQGIALWLHASTAAVAMVLEEVKPLPPGWHEFGGTPSPDEAMMLSFAIRQPAIEDIG

RSMASKDARHLSRGEARQLQHPCPNAVSAVMNWLGDNNVPDARVMQDFIRVRTTVQAAEK

LLNATIRNYTFKDKPPVRRTQRYSIPDELADDIKFVHPIANFMSPKKELSSPSPADDEED

MHRLNTRDTPCWRVTTPQCIRQLYGINSTATGMRNTTVRLGIAGFLEQNANYIDILSFLN

RSVPEIAATGYNFTTELINGAKNDQNPAHSGYEAALDLEYSLPLAYPAKITYHLAAGRGV

QLNDSGDPLPDEFVTNEPYLEFLEHFLAKPDDQIPHVLSLSYADDELSVPRRYAERVCSM

FGLLAARGTTVLVGSGDGGARGGRNSSCLVTDGSGKRAAMATFPGTCPWVTAVGAVTSSE

ELTGAEFSGGGFSQYFTQPGWQAKDVQGYVQSLDSRLDGLYNGSMRAIPDLAAIGTSFSV

QFARQVTRLEGTSASTPVIASLISLVNEARFRRGKASLGWMNKILYSDKVRSAFQDVTNG

TSRSCVFEGGEEPGGWPSAKGWDAVTGLGVPKSLQKLVDVLVDV

>SoG_05746.T1

MKIFNQSFLTAGLLWAAAVIATPLPDTTDDNEVSAVNPCSVKPLKVNAQEIRGAHTHERV

EVSSYRQCCEACYKRKGCALFTFVGEDDNYCRLSYNWEPGTGTVTKTCPSKITTAYEATD

GADGRWWNMGAGPCGTAKECDGTCLSSSD

>SoG_05773.T1

MRPVALLAATLAVLPGVFGESRKSAIVWFEDTVSDEVVQKAKHALEQAGGMITHSYEIIK

GFSVLAPEQAFTDIRVQFDNGEQALMHIDNDDEVTYGLQVEREATVSLVVQAAEFDIFHT

KVVQRSLQTVFTDDDVFFLRVVTAAKLGSELDLPRTGIDATRNIGRYCEFTAWHEAKIGQ

AKGRRTPKRFIAYFDDALATVTVVVMPSYNHSAQVALLKAVGIFSNPSEPSLRSTERTAE

NVEDLTNGLLLGRILHQLDPDFDLAALDTQADRPKYLANKHNIQAVYKGLFRFVRRQVPE

LSCQAKNFDLDAISPAPDQQGATAQRNPSLIPSCFHMLAVMVTAAALGPDRGTYVPRMSD

QSFSTDTQAEIMQIICQMEEDNRNSKDDELSEEDIDNFIGERNIDLLVEEQNAALRHELD

IARKTLSDYITRLEHLQMSHDSLKYEKEKNDRELEQLRAEIGQDADKDLSMKALQRQINE

QMDVIAAGEHQIRDYQSRIKLLEADVSKWEARGREAEALRDQVAEWKHAAEEFEKRANAA

DRYKQKLEAQQEMVRELQNVQYERAELQEQLRNLTDERDRVDRNRQSESELQKMIDQSEQ

HLWDERNHKDQLLREIALLGDEVTRLNAQRAHDERYIQELQEQLQHGSDDVAARDPGSGD

EGLGGSLADELTNAATIGEGVPARSLELSRLKAENKMLRGVQSEAAQLRQELQEEKSQRG

MLQSEYNSLFHKHALVQDQAETLVAGLVSEDSQGFINLQKAWRQLAVDNEEIRQAKKSLE

ETFADIQRELTETKAQLSASQKEGMDAISELRNTDQIISASLRRELEQVRKEVNFLTGER

NNLQEQLVKAYINMENIRVNGGGSSDTVIDEEMTELVKKQTNKMDKLKERLIERTQVGEA

ILLELMNTAMATLEEVRKKDATTSRRPSVSSEEHWPCATEMSSPRQHIPVPRPLPLELRE

EPPLERPVPVDTPPTPTKHINAAPRPAVDIDRALFRGRPWAPGMNTQSDRPSYLARFFNL

VETHRKLKAALGGEAAAAQKAASDQVIKNLRRENALMATAWHDLTSRLQSNHVVLQRRPA

TPKSWLNKSRQMVNGQLRTRDSLYLY

>SoG_05780.T1

MARLNAITAAVALSSFLHTNLALAASPGYITTVEVISTAYLDLGLCLNQCQQFAFNNNGG

HAFKTMEAPPPAAAYHTLTLPICEGEAHTSTKQPECAGCHGTVFCFEATDVPFVTVTSVH

HGAEPTIEIILPEDGCNGCTGTVYILDGDSPYPSATVTITLPGANENGPRITLPPSVPGG

PSTVIIGTSGFENNLPTASFPGAGDGSPTNVVFPTDANGNPLPTGTPIVVVGDPAEAARA

VGGAPPTAAIGDRNGPTVTAPAGAGQPPIVISGPGAAGDGSRLGDGADGNDSGGDGDIVL

FSADGAPDDANGNGSPDDTNGNGISDDTNGDGDSSDTNGDGVPDDANGDGVPDNGSGLPA

DGASSSTADGNPTTPNDANTSGGGNSDGTDPGNGDDAATDSGSGGNDAEQPQIFNAQDAA

TNSDEPTDEGEDGSKDGGAADDTADSGDGSSGGADDSPGTGGGDSASNAGDSPPTGEGAQ

GNNDDSSASDNDGQTNDESSGGGNGQTNDGSSGGGGNGQTNDGSSGGGSGTGNSDDESSG

GSGGSGTGNSDDEPSGGSGGSGTGNFA

>SoG_05816.T1

MKFTSALLAVMAVVAQAQPIEEPRAAVQEFPVLASSPQALDARSLEARAANMQFIVWPNI

SKRHPLTCSCIRPAWIPVAGTQAQGTLVCFRADRVHPDCESSPNGVAISAGDAPAKGNFS

PAQKSIKLVALNAGYHVSFYTDSNQQPSGSPNLRLNAGNVGNCWNFSPGWKSWGLFTGA

>SoG_05904.T1

MRQVDSLALFSAIGSFTLAAAGSLPRGVGPEFASHYQDKESFSCITNAAIKLSLSQVNDN

SCDCPDGSDEPGTAACASLDPNSPSQPLPGSISGSTSTKNVLPGFWCENKGHMGMYVPFI

YVNDGVCDYELCCDGSEEFQQVNGVKCENRCAQIGKEYRKLEEEKRSKMERAQQKRKAMV

DRAAELRAAVEARLTELIGEVARLEARRDDLEKAHAKAVLEDKGKVVKGEGEGGKLAVLV

GLAKQRVTELRNTLANLVDQRDGLRREADELREILRKLKEEYNPNFNDAGVKAAVKSYEE

YAARDAAEVKDYMPDSEIGEILQDDSETSGVNWKAFEELEQDDTEVLYNLEAYLPPFLRD

IIRSQVNSLRKWLVSNGILADNTQPGHESSSTKSAREAFESASTDVERKRTDLENQQADL

SKDYGPEDIFRALAGQCISIDSGEYTYELCWLDKTSQKSKKGHGNTQMGTFERVDRLLAD

DDDRLDGRSLGRGERMVLRYEDGQQCWNGPRRRTDVWLACAEKEELWRVSESEKCVYKME

VGTPAACGLLEEARPQPQGKDEL

>SoG_05910.T1

MRTFNFLPAAAGLFGVASAGLLPAISGGVAGGLTASVSAGVNADLAGGVPVLSSTVKDKN

VKPVCDLTHHWEDDVLFKGEVDADVSLDLRLIQAAVAGQVSIDVSSKFCQAPALRLSLAG

VKAFVQLDLSASAKIYESVELFATPELTVDLLEILNIDLKALFALDLVVGVSAAVDVSAG

VYVQFTDDAYIDIDLIKRDVLDVNLANPVIEALPIAIGANVDLAAAVDLSIGLRLRSYIA

VKADVDIVGIDLLDAGASVAIYVNLFDYTTTLIGGHGDCVVSVKNDLCLTLGLAVKVDVN

LLQAIDISLTPSVYVTLASTGEAEVCMPDRGTCPDGSNITPGGPSGSIGGNDGGLRWRLQ

RQHWL

>SoG_05922.T1

MIVFLIFTIMYQITLSRALDPLLYNMPCTIQAEEELLLYPPSDLDDATVMEEGHSQTQEA

KAEDRRPSVPYSARTIPDYKPSFLQKFLMPWKFTDYWTLRRTICRGSFNVIERYPEEVEA

SAYLPPSVRSPPPILWIPRDRAGVSKMEIEDTEDVVGITDEGCVLDGSNRLRWDTAEARP

PIWTEKIVY

>SoG_05936.T1

MVFLNLLTFGLWSKVGRLTHYAFDAVLISTILAGMKRSTGLTPSFKTEKVAGENKDINKW

VDKYLGLGEWVMDQSVAIAGSSGYFERTR

>SoG_05939.T1

MRLNTPLMILAATLAAKVQADRWDILGGCSIFECNYNLGTWYYNDGRSFKFNAYEGCRVP

FIPGVKEYCIDWNRRRGHFYEDGGQKRCMIEVRDDSSFCGTGCLIVDVGWADTPCTW

>SoG_05956.T1

MKLSTNLPMLTITVLIGIAQGAPTGSQAPSDQPPLERANTDDSLEYYVDTAEKRADTDDS

LEYYTDITEKRADTDDSLDYYNANTEKRANTNASRKY

>SoG_05981.T1

MRTSNIVLSLACLAGISEAFPTPGSLAALAERDGLTSRGILEGLAKLAVKKLSFDPVTQP

IQVHGEHEFQPPKKGDQRGPCPGLNALANHGYLPRDGVGSYFQVVEAINTGMSSIDMQCK

YLPGIPSKPTNNQDDDFDHGELLVGPSDEMRHQGTPRGLDGSHNFIEADSSNTRDDLYVT

GDASTMNFDLFMDVYNSINGTMSIEDVGDRAAARLRESIATNPIFYYGPYTGLIVRNAGF

AFGGRLLSNHSAEFPQGGNMDKATFASMWGVSRDEEEQGKLVYRRGWERIPENWYRIHRD

YTLVDLNLDLVSWILKHPEIANVGGNLGRVNSFAGLDLGDVTGGVLNAASLLQGNNLVCF

SLEVVKAFAPNSLSSLFTTLAAPLQLINDALLDPLLDLHCPAMAELSKGGRDILAGLLDK

YPGAKKSGFAF

>SoG_05983.T1

MRTSTLAALVGIFASASAQLKVESIARIDSAKSIARGQCDALDACKTSVPGSYVPDPKTF

FLPNVHNKLSNSWGLCKHDAYCNDDAIYNKVMFTFKHDALVFDFHIPGAKYKDVAIRIQR

ETPPTDVSPSFGLHNCHSNGHGGLSCSLPYKSLNCHGPHDMCPTKDEGGWVFYSQIKLDI

NLNGESFTLYNKAIEKATGTCWFSLSYCCTKCPACPAPPPPSPPCSKPCLETCKSCQKPQ

CPGPDCHKPHQPCPQNCAAKACGKCQAQLPARIQAREPLTPAEAIRRGRAKSKELDVACQ

SAQVVIYGQGAHSHSLNELRKYPGHEECKDRKGHYHTYVKHDLEVTVAGALELNNNAFGS

FIVEFDKQNKNTEIIIKIDVWNALYYVVEAAVFIDCKDGIVENRGGRYGSHICRPETFPY

WYVDEKGIGNYDFHVKDDFKCKGDYVFALWVKVCIAGERKNCHYRSIRL

>SoG_06038.T1

MVSFLSTLLLAGSALALPTKQPTCMQAGAKVANWTVENFDYHASYIFSTPSHQNSWGYVN

FTLSNPALSYKSACSAASSQLSDFFYGNVVYNCVAPADHAGDAATFTFDRPSGALMLNQT

WGCPQEGGRFWAEGGTKLKLDCQEEKWQNDDWKMGEIYSSRTVTCNKVTVKAPVERISAV

L

>SoG_06113.T1

MLSHLIFLFLGLAASTAQEINYPDPTVPCGAETGLCPRPDLYCKPDDNECADLTRCRGTC

AFKNTFVRCGGFRLPETGPPSCAEGESCVDDPRNPESCGMACDAPGVCLSREALECADDS

DCPAGQWCYEDVKNQTGLAKVCM

>SoG_06116.T1

MKLYNLALGFALLPGALSAPPNTKRDDAKFNQGQPINAQGKGGPILGGTNTQLDLQNPSN

LGQQPTDNGVVPNLKWSFSDSKTRILKGGWVREQVIQDLPASRDISGAQQHLRKGAIREL

HWHRVAEWGIAYAGEVIVSAVDENGVYQAEKLGYGDIWYFPKGVAHTVQGLADENEYLLV

FDDGNFDAVGTTFNIDDWLAHTPKSVLAKNFGVDESVFSDLPSPNPYIVNGTSSERNVTG

AETPYATGNSSFVYRLFDHEPEQIGGSGGSFYKIDSTNFPISKTIAATFVTLKPGALREL

HWHPNAEEWLYFHKGKARASVFIGNTNARTFDFSAGDTAVFPDNSGHYIENIGKEDLIWI

EIYKSDRVADIPLTQWLALTPPDIVSQVLKVPIEFVDKLKKKKQVLIE

>SoG_06117.T1

MTASILLTALLSTLATASPAKAGRRGLSLPPLIPSIPGVTEPLNDLVPPLPILQVPTPPL

DSPPFEVENIKPKKIGHFWTGAGDNKHKDFLVTASLDDDTFGKIIHISDVPTSGNSPHHL

GPSADGKTLVGGGLLSLLKTQDTAFYWDVSDPYHPKFTHSNRALLSSITDEIRAKPEGGF

FITYMGSAVGTSPGRLVETDAKGNIIHEWPEDVAGTLNILGEQFSPHGLDVDFKNNVILT

SDFVVPITILKPTLGIQKASTLRLWRLDDRKIISTITLPNGGGIQDVKFIPGSQEGAALA

TAVHSGEIWVIYPHRKDSQGKQGVAELLIDLGEKAKDSVAIYSDISKDGRFAYFTLTLGN

HVAAVDISDLKNPKRLDDPNENQGIIGPHYVKLSPDQRNLLVTGYFVQAGQISVLNTPGD

YKAHWIDVLDDGSLSFNRSIDFESIFTKSRGGARPHSSVIFDLTDPEKPLYY

>SoG_06131.T1

MMRLLIALLALPAALASPVAGNTAREVVADHLACTCKNAAGNSRASGICQARSGSLVPNG

AAPSGEEWVNPRPESPMCKSVTCYSAVEWALPIEELFSSEMCRLNFPKFPEYECKTFRFD

DGLPCDKYSRVC

>SoG_06136.T1

MISRTILSLGLAAVAAGHGQHDSQKSMAGPHEALWFNSLPGDGGTQADSVFSGISTFGRL

PYKQCLGNSDIQYDIAFIGAPFDTGTSYRPGARFGPSGIRQGSRRLNLYGGYNVPLATNP

FNSWATVLDCGDVPVTSYDNKWALRQIEEGHYNLLSRPPKTDAQAVGPAKGGKTLPRIIT

LGGDHTITLPLLRSINRAYGPVTVIHFDSHLDTWRPKVFGGSPSEQAAINHGTYFYHAAM

EGLLANDTNIHAGIRTTLSGPSDYDNDGYCGFEIVEAREIDTIGTGEARLPLPGH

>SoG_06139.T1

MLFAKIASIAAVLSQGAMAEREWRDCNDCLAKCIDREDNRNRANCKDWGDDWCVFRSQEF

HNAVSECSSRECGKVCASIREIYRQIHDENKVKPAREAYTLNWLESTSKEKGRSSHMRRA

APEQKRSKPASPEQERSNLHDCGCGHPKCDCPLPPPTGASDCVQGKPRRHSRIERESKVK

NLSPECAVFHNAHLWPLDKNLGDSICNDPGYIPGCCAKLTGNNIELVEKAHRWVKNFCRE

KRGETIIEVTGGFGGPYTGKVFEEAMDKVQLPSFERGDDRA

>SoG_06149.T1

MWSLEVISAALCLASIASASFDNNLNYQSPSRRHSGLGINVPLVKRRSWKRGSVAYKPEE

LSFTHGVASGDPWAESVILWTRVAPSSKSDASEITVNGTAPLYSHETEKYIKADANPICV

EWRVFDSKKGKPSDKAVKHGKAYTTSDIDYTIKVEAKGLRPLTEYFYQFNICGSDKKSPQ

GRTKTAPRPHDDVSSLSFAVFSCSNFPNGYFNAYGNAARRDEHDFVIHLGDYIYEYEAKG

ERASDPPNEIFSLYDYRTRHGQYRTDKDLQLLSQNHAWITTWDDHEFANNGYRDGFSGLN

NTEDSFLNDSPRVSVDTRKVHATRAYFEWMPIRQTDLDDGLRVWRSFQMGKLLDLVILDT

RNYDRSITSLGWNDKYIDELRDDPSRTLMGSRQENWFYQAMSESQKRGATWKVVGNQIIF

SRLFENDAGEMSGDNWNASGYVANRNRTLEHLYSNKITNSIFLAGDSHQNWVSDLAWLGT

KDYNKETGDGAIGVEFAGTAVSSSGQTGPIEPKAGNYARAMIRRNDEMQWQEGYYRGYFV

LNLSPKEATARFYGSPSVATRNSWEIPLANFTVKAGENHLQRPIAGGRVESGALRGGEVK

HTNLTLNTDTGKWEVIGFDKMFIPKK

>SoG_06211.T1

MLLNTVASAAALASIALTIDIVEKDVVIVGGGASGAYAAFRFREDYGKSIALIEKESILG

GHVDSWTDESGRTYDFGVVTMIDSGNATAFMERLGLGLGSFPRAEVTQEFFDFTTGEAVN

FQPQPFPSQLAALEAFRSQAEKFEPIIQAPGYFNFPEPDDIAEELLMPFGEWIEKYNLEA

AMPFIHSSTGLGVGNITKELTLFALQAFGASMARSTLGSQGSFVPASLRNQDIYDAIANE

IGEDIYYQSMVVESYRSRKGIKVTVKNSVTGKLTMIRAKRLLISIPPTRKNTAVFDLDKN

ERDVLGKLYYNNEYTGLINNKNLAANWSYYNMPVASEPENYLVLPEAPFTARIDAMGNGT

IFRVTFIGDTNTSASEAKAIIQKDLDRLVESGAIGTPDDGDKALSWVDFSVHDSMHARVS

RQDVEAGFFQKFNALQGQRSTWWTGGAWAVNFQTHLWEYDDLIIPMMLQGLD

>SoG_06221.T1

MHTSTWLSGALSLLGAVAAQACNGCDYNALFPAAVCIPSTNGICQEQVQVIEVVVGKASN

RVCDVCVERHHNDCAHCVTVVGPCPTPVNAAVTTTIINNGVTTVIIQTPTNAPMQSSAAA

SSSVSASVSASASASSASATPTGSAVPFSCNNNGYVMDGSDLYEVDLVSGQAVKSIAKNI

AFDGAILAMGYNVLDNLIYALAPSSPSTVRVIQIDANGGQKFLGDTINTMYSVGDFDARG

QLWVATADNTKWARIDLRPGSPNFGQVADSGTMTPLTEKTEDWAFLPSDGNFYSFVTSST

GNTLAVMWTRSNLQFQVLTDFGQIAMNGTQPPAWGPAFTDGTQIAVADTKSGQIWKFTPG

GRAIDLKQMGTSAQAATQYSYHGGARCMDAANV

>SoG_06232.T1

MRSASLLLLLATLWDAASCVNLRKIEFDILKSSRPSLFLFTANGCKECDRVQEVLDEVSA

TLSTTPIATVNCRDDPAACDESRIFTVPTLKFTTGDGNLVSYKEVMDAPSITRYLERQSG

SPVTELTRDNYLDFAASSRVAVIAFLQPSVSQADRQTFESVAERWRTSFSFGSVDAVESG

NGTKASIAVYSQDSDDVVHYQGKFNVNEIEAFLQNATTPLIYEFDPLIHEKLTKLSQDGK

PLAQAFVKKRSERAELAKSLFPIAKKFKDQMNFVTILADDYHQQRDNMHIVKDVERGFAI

ADARGRAYPMNATEVNADEISKHIDAYLAGTIAPTIKSEPVPEPSEDLPFVTTLVGSNFD

DLTLDETRDILVQFAVPWCEYCNGLLEVMNKLGAEYSESQLSGKVALATINVEANDVPIQ

IDSYPSLRLYRAATNEIVSFTDGFHQMLTLEQLDSFISSKGKHGVSVVGESKAERHEELG

PHTKTTWRLRHKPATIARTHVHYAPVAVPERTTAARQYTSRPVAFLSLRVARHDPRPADH

LLRPLGLLCTILSRCPSTAKDHVR

>SoG_06248.T1

MKFTILAVACLQLSAGLAVPVVSRDSQDVQIFKEGGRGAGRDGAGRGAGAAGRERAGGPA

RGGAARGGAAGGGAAGGAAGGEGEGEKKENEVELQAKFGEAVTLQGGDVKQDALFPPGTN

GVFETEFQNAAGNTLTVTENKTPAAPPAGFSALEPVSYVVKLADNAAGATLQKIDYILNA

GNALDISKGKVGKLDAATNTFVIDNTVGELEFEKEENELTLTVADLNGEWGIFVPAAAGG

ANGGAGGGAGGGAGGGAGGAAPTTGNSILDSVLKLIGEGAAAADAAAAAGKAAA

>SoG_06267.T1

MQVIAVALVALAGLASAAPGAPIEDRQTYVPCTGLYGSAQCCATDVLGLANLDCGQPPET

PTDADNFSAVCSAIGQRARCCVLPILDQGILCNTPAGVDE

>SoG_06308.T1

MKFLNVFTVLSAAVLASAAPLTSIDKRAIFSQSTFNELSISGGTAGGAAKAALAKLGPLP

ADLATVEQSDVDFLDSVNKICNEAEKQAFNPAIDAASGAAADALQRGKIQNKVLKLTATV

LKLQIQQAQGKTGLEAKLAQEQKKLTNNIKQDEAAAGQPATSLAFNAST

>SoG_06319.T1

MKTGTVAILASCLLGQALAAPVAGKAQDVAVKVNGETKDIKLDDLKAAIMDLAKESRSVQ

YGSPSKRGDDALKKVLGTLGLSPKDLDKDTLKKAQDSISSISNASASPISRFSDLGGLLS

ALQPYLDKAGKTTGTAPVKRSNKDVDDMLKKLGIDPKKLDKDDLKKIESKVKEVTDKTAA

PNVRAGGLGDILGILKPLLAGSNEEKGAAAAPAKGTSKGLADGATKDLPLGGSAPGAAGS

IAGNLPAKRLVDGSVLDGLLTTLGVETGDEEDSKKPDLADTTGGVADGALDQATGAVGDA

TKNLPTGALPTKREISVEQVSDLLKTLGLDDTLKSVTKSGKGLTSGVASPSLDAAKGAAP

ALDRRQIDALSDLLGGLTGGGKGGKGGSPVDGLLGGLTGGGKGGSKGGSKGGKPVDGALS

GVTDTVKGATGSLGSATKGAAPGLSKRQLESLTGLLGGLLGGGSGALGSASSTFGGLTGG

ATDGLTDAAKGAAGSVPFMNKRQLDALTDILGGLLGGGEGGSGSGSPAVGALDTVGSTLG

GLTGGATDGLTDTAKGVAGSAGALMEKRQLDGLTDILSGLLGGALGGGKGASGASPSKRT

VDLDSLLQGLGPVLGQLQGGADASKSGASKPKASSPTPVSLPTPPAIPTPKMKRDGMDVS

LPAPAGPPAPPAAPAAPVPPAPGAEAGPVPPPADVTPVPLPSPVSTEPLTVIYDDLTEVT

SLLGDVQGEVPADQADNLRQTIDSFLSSISDLMPPGTAPDADSLPVDGPAGPAGPAGPAG

PAGPAPVDPAGRPIP

>SoG_06343.T1

MQSFLIFSLGLASLAEAICHHGTTLFSRAEAGKPKFGYTNLDGPLMWQALSAENKLCAQG

TKQSPISVNEKTAKPAAGSSLQFDIKDFPNGAELLNLGTTLEVEANGTMALKNKTYNLAQ

FHFHTPSEHRMNDEHFPMEVHFVFQAADESLAVVGTMVEVAAMEVPAPFLSSVFANLDKA

KDAGAAAATTPINFSALKDHIAASTIWQYEGSLTTPTCAEGVAWNVVERPLFVSSTTYRR

IKSVVKFNSRYTQGNPGEANLVSNACNVLAAAEAE

>SoG_06351.T1

MQFSVLSLLSVAVTVLSVPIPIPDPPGIPSSSTASSLLNGLTVRAWTNTNTYDRDLFPHW

STISGTCNAREYVLKRDGSNVVVNSACTAQSGTWRSPYDGETTNTASDLDIDHMVPLKNA

WVSGAASWTTAKREDFANDISGPQLWAVTAGVNRSKSDKSPDSWVPPLSSFHCTYARSWI

QVKSSWNLSVTSAEKAALKDLLAKC

>SoG_06354.T1

MLHNPHLLICWLPRAVAAAPAAVASAALFAALVESAMQHHDDQDHYHHRAAGSRSKPRRP

PNINTSLAAPKATDYPPLVTPPSRLLDAQDDHRCHRSPQRDSGHPQDLCLADASFLENPT

PVDDADMAEQNQSRVAQRDSHDLSYSNRNVTRDSLVTNMLLSLDQFSSMGGPPGTNGFAP

DDPYEDAPQLFDTSRSWANSVVPSSRHRQQSYSSDVEVNDDSSRLSSQTSRGRRSNSSSN

FHNTYSRLNSVRESTHRSQPTTPRVGKHSRGGKESKSSSTNSVDVAYNQLQQQQRHARAA

GRSASFDHGTSNQLDSLAVSPFQIEFSNSFFHDDYNAAPTPTIPSGPRRMPSTPAMIPPP

PPPEPRQQQPFLERKRSLTRSLKSSSGKRGLSSRDAAAAAPAPALVSPDLDSAPAPHVGY

EKSKDGTRNGTRNGTQNGVAAAPKEKPGFFRRVFGGGGRGKDEAQAARVASPPDRSLESS

GNNNTSAPPSRDTSSSHSQRPTLQKKPSSFFRRRKKSTADQAPPIPTDTPPVPLVAPAAP

AASVAPVAPVVPVEPMMEKGKPVDNRESVSSLREVMNPYLDKHVQHHTPLRDITNTTPNR

HVDPRSEYKRDFSPDYEPSPNARIRAVQPDSDHERSTHDTPSKPPPSRPGEPQTRTDSFL

NLDGGSDGDDDCARSPKKAEGEGIAGSAAEETIRQKKGTFTLYQTDSEEEAGRSRLALPI

EGTRAASPASASSHVESNPGTSAPPSVFIEAPAEPSPRRASPKVLGTLETMKSKPLDEPN

FVIGDPTQDDRQKAQKIFDGNEDFISKEKAASWMGEEGPVRQRTLQAYMELYDFANQSVV

NALRQVCGRLVFRAETQQVDRILVAFSKRWCDCNPKHGFKATDVIHMICYSIMLLNTDLH

IADIEQKMTRSQFIKNTMTTISQAVKEVAPDAFSRPTILPEKGSGLGIDTGMEAETRSFR

HSFRPPPRTDTQNSASSDAANDCGPLVRAPFEGSFKAWEEQVENVLKLIYASIRDERLPL

YGAEPDVQAAGNSQSGLSVIGMLKRTPSVLSKAPSESAMSTARGRVPEYGRAANSRWASK

SRSRPGMGRNGFSSSRTSFEEANSMWSPALSSATWSKYSLGRTHTSVSQDSFAVSLHRGD

YQQSIGFANALSQAIIRNDDSIGPDGTASIMSEELPEGLLDDESLELAGPPWVKEGMVIH

KHHLDGVDKKARDRNWTEVFAVIQKGQMSLFSFSNKSLRQKNRTRAAPRMNAPVGGGNWQ

DNATSMGTFSLRQTLASALPSPGYSRSRPHVWALSLPTGAVHLFQVGTPEIIKEFVTTVN

YWSARLSTHPLVGGISNIEYGWGENIVNNSLVMAINESNVAANASGKESRPGSSTAVGRR

TSIQSGRASIRSASFDLGARAFTSGGGGGKLAGDRIHIAAWAPPTQSMRPSNAREEEQLA

TLTAYVKNIEEDLQAHNQLRSPMLLAFTPRGSNANKAMSNWERKSAYLLREIVKYRTYVD

CLQQAAQRRNEVYSERKLARKAARGDLSDGDMEVSGDEQKGGTVRAR

>SoG_06386.T1

MKTSVNAVFAGLVAVATAQIQLEVRFSDRMIALTRALGRSDVGNVNLQEITRDVIYAEAG

NQRSVLTDKTHASNNKNCKSELEGDDLAVQVKMNGAWGRTPGLGENDMRDGLIAAIFEVL

TNVAAENAVDVYTECRGLVFQESVSHVPEAACGRLAAQPCTEVCKNDSASPLLTQCMKVS

KSHRVPASMRVTAFIENALQPDDLIFEFSSQANDLGGGCGIVGDIAAKLAGFAIPVAGGF

FADGITLACSS

>SoG_06389.T1

MLLSEDPDKVRPAKSLTTFFCFLFLLLQSGLPLVADTFPSPEQLISHTVDNFNIEPDKLA

VARITESLSTLQQARDLRVNEAHATLKKLSRQLHTLTSQHDDLVSSSSQGGADHASRIAR

LDTEKFRTAKAASDAEVEAERLALRHADLVARLQELEAQGVEGDEASRRRDPVDDEVLLR

LKVYRSLGIDLERDERDGEWTKAVVRNDRKGDVHVVNLDKKFSRYFYANYFWQTL

>SoG_06399.T1

MAAKLISHLKLWLNSLIVADSLQHQKKGYDEEKLEQSRETRAFPPEHECELWHKEIGSQL

AKMLEMAEYPTSSQALHAEFFLNSVTPFLGYHPRGKAPLPHFWKSFMTDDHTPIELSWAW

SSGNKRTPAVRYSVEPISRSAYRGLDAENVDANVRMLGESLRLAPDLDLCLHRHLGKALA

VTRRKDTGGDDKDAQDTGADPGVPQSQCFIAFDLQESNLMVKQYYIPHRRSRELGCDNWS

LVRETILKIPVAWVQTIEPALSCLTNFIGSCDAEVMPSVEIMAIDCVDPVQSRLKIYCRS

RATSFNSVVNMLSLGGRKPLQPEEEENLWELWLAVFGLTPEPYSSRDEPLSGSEHRTSGI

LYYYELRGNKPCPNSKVYLPVRHYAQNDDQIARGLSDYLEKRGKGLASGSYHEALQKLWY

VKSHLAESPIKFTPLEPALEEKLADGFHTYVSWAGDKGKWNITAYFNPQIYSR

>SoG_06433.T1

MRCNIQLTTAAYLLAAHFVQAGPLRWKRAFWERDEASTPGELPTLPPTTLPTATADPTSI

PSTTTIIDSTPLTTTIEPETSTTTRSAADDESGTGTSEVSFPSDETPRPIFSTVTGGVTD

GLQSELTSLLMTGEKSTSTTNPFTMPAPNPTSVEVQPLPDGLTLLPTTGTTPTATSETSS

TSSNPPAGTPSSSLETSSLISITRSESSGETPKSSLETTLSRSTTRSESSVGTPESSLAT

TLSRSTVETPRSSLATTSSASTTRSKAPIETPSSSLGTPSSALATQLTSLSSQLPLIPSP

TKIITTTIIPQEPPQTPVPDPNGTMPGSGPSSSAQSSGLPAESSFPESSSSETTASSTTS

ASPPADTPRFRTQPNPQQSSTSPGETTSSTSATQNPTLPSQAASSTQTTQEYSTTKSELA

QPAPTSNVLTQTIGDPDAPKETTSVMPELPSTTSSSSTTKAAEPATTTTTTIKQSQTQTI

TQSPNPAIPTAEIQTTTISVSPSLWEANLARARGYNKVYDTLSPDMACSAGQVACISGKV

GQCAFGTFDLNSCQQGSRCFALPSETADGVVVSCFDVEHAEAVLRGEKGPASHSTSTSTT

STSAAAALPTRVARPAPSDEQASQPAVVQPTTTTSRSTSSSSVTVAKPTTTTAPPAAPLI

NTYFPGEDPFTKIFPITGDDDLPPAAPRPTDVDSGGRADSISDAGDDGSSNRVTQTVNGT

PTVSVTVTVTVASTTVTQAGVTKTVTQKETETVTVGGSGDRVDTVSIGN

>SoG_06434.T1

MRPSLLRELALASCLGVVQQTYAATPQSSPESGTCGTEYGVFHRVHDSLSVCTEHVKVMT

IIVGRDAALAHCQACTGRAADDCSKCVTVVGPCASHCTSPVTSTITHGDLPTVLILTPTA

AADAPSHPLGLASFSKASLAPVAGTNTASSESISSSAHLFPSSMNTVFSGNIASSATSGT

MAYPGTSEPITSSSRPVASGIPGFPGIIGNFALLGCLASPGGFPSFSLRQSSASMALGLC

AKLCDGSNLFGINGYDCYCGQTFDVSAIIRVPIEKCSTRCPGNPDEFCGSSRLRRRQSVP

EGTLMTTYVLPAAMPSFIATDISATKTTSSDNNSASSFLSTLSLNSLSSGSSSLHVSVSV

TSGPTSTLASSSADPSLRSISISSSSSSAQFSSSLAGSLVQTVSSNDQISSSLSSFSTGT

LLSSTPASPSSRSSLLSSSSSSSQVGTGSLNGTTSSSSSIISTITLSSSILSSSSNTASI

STSPSSAQIISESSSGPAISSLRTQISTTPGFSSLTIAPPDSITVDTPTFTSTITYLSTQ

LMINGTQTAPEANTPTNTPVISAVNATASLIESPASTPVGSPSTPVTYSQDNTTAPANTP

TPTPFTTPNPTSNSTSSTSPLPTFACDKNAYAVEQNSLWEWNTTTGQNMKRIVASMGPDG

VLAVALSYNMLDNYIYALGQNGSNPALVLKTDSNGNYTIISHMLTGDLWNTGDIDGNGQM

WVLSRDNTVWGHIDLNPSSGTYGQVAQGSVSTTPESPMRDAAFINGGSDLWGVSRDSNNN

SLLIKFSTTTKALTTVANYGAITSRNWFSAYTSGDQLWMTFNSPPPSQLWSFSVLNPSAA

PKQLLSNTPGYMQGGANCATGQNS

>SoG_06481.T1

MKVQSLVSLALLSAAAQACMQFGARITSDGYLRGAIVDNDRATCQLNAKMPQDQYWLQCV

DGFAAFVTGDLGTLAYANGPNNYRIAIQRNGDYISASAFC

>SoG_06483.T1

MLFTKGALVLAAGTSVTSGQFCEHQWDGPFAEERDGGTILQARQGGQPPPLDIQPLIING

PSENRVDLIFFGDGYTEAEKDKFFNDSMSLAKEMTSGQTFADHLPLMNYWAGFSASKQSG

VGTNGKPLDTVYGLYRPGTELRGVYYSKAEVAFAACQSTDACDYPMLLGNDPFYGGLGGT

FTVVTASPHNGAAVLRHELGHSIIGVGEEYDGGQVYRGVNSASRPSSVPWKEWYTSPSSE

PKIQRSNMPIQAYPWTMLNVSTAWTDTFTSAGVYDNMELQFSISGVLASSDLKVELDGED

LGWEVNEVVGLDRWIYNMKFDKALTPGAHKLTFRLLNKEREGTAQLCNLQALEYGPPSQL

PRPLPDVLGLEHHDVPPDERQMSDANDALGRLLRRVHRGPLVLTPEASVPDRQRHADRIR

RRRRPHRGHADAAPPGAVQGDPEADEGELRDPVARRGRRDGAGRVDEQHDGEVEQVRAAG

GGGGG

>SoG_06504.T1

MRLQSVVAVLVGLRLSARVEAQRAYADFQAPLQKDNDIVSSKFPDIEDVELRSPSFLNPD

KVAAGFANGTSSPLSQVDQARNEWLTYVTPDFLSEEGRPMPYLYLSNERKPTQNSAGKVR

LFIQGHMHGNEPAGEEAVLALLGKFDANSTWTESVLEKMNIMILPRYNPDGVAYFQRQFA

TGYDPNRDFMVLQRDQTRRIKELHADFAPHIFVDAHEYTASTLYGTGETKHLLKAEDGQI

GGPKNLNVRREIRDLQNGLYLDSMTAMMERRGLRTSPYYVVNSGEGLILEEPDSNSQYSD

HSFVLSQAVAILTETRGIRLGDQHFQRRTATGLLLLEEIVQITADNAERVYRTVEDARQE

FANSEEDIIVTDHAREHNVTWDFMDVNNGSIVSVPATFLNHTPPVVNITRSRPEAYVFSR

AWADVAERLRIMGVEVKKLEADFSGSVQVLTVETAELASSKFEGIAHTTVTTSTSTKNVT

IPAGGFWVPTKQVNAAYALVFLEPENVASAVSYNIVPVEAGDQVPIFRVPKS

>SoG_06568.T1

MRVSLSLRVVGLAALAVAAPTRAPSNLFDYAKRSVSDTYRFYTGNGSPSQGWPDFGSWGS

YDELWDTNAPVMAQSCEWNGWGANNSPEEISAIKSAIPQVSGDTGVDKRFILAIIMQESA

GCVRAPTTNNGVRNPGLMQSHNGVGTCAGVNPCPASEILQMIRDGSGGTPYGDGLQQTIA

KAANAIGDSGVRSFYAGARLYNSGSADYDNLNDGITSTACYVTHVANRLTGWALAPANCH

L

>SoG_06582.T1

MRFSAAFACAAACLSALIQSHPINGDSSIDIVERTVSGWLAHPAHDLALRSTTELEPSEG

FIGKRASSRWSKGTVSCPGPFKHAPSKNFREAVKELRKRKGRPSMAARGQYPTCETVCSK

GVQISWCNKDNKRKTLESWRSVAEGIDVLLGPSGCTAKKNGVEMTGGAIYHPTDWYVFAG

TC

>SoG_06793.T1

MNKFLRLFASTALAAQLASAQDNLGIGNGYLNLSTAFLDIELVKDAQVLSSLRPRASSFD

FLPRDKLSLRASNQQYHWGDLTYRYRVPAGSGDWADGDTAASRAAVQAGAGNKADEVLAS

AVLTKTLPQSPLEITREWLDVDGDLGLRFRIKNTGDEPLELGSLGFPAEFNSIFSDRTAE

EMRSHCSLSDPYIGLDAGQIRVTPVTGTGEALLVTPLTNTTVSRFEAYRNLWEPASDPLW

YGSQTFEGFYEWQVLSKAWAEKEWASVEPWNDATSLTLAPGEEISTGVRFTLSDQVRKFD

DTARSIGAPVAKSVPGFIIPRDQPATLWLSSSSSVVGFTVKPEGAVTVLPHDNGKSFIVQ

PSPGAWGRARVSVEYKDGRVQTLHYLVTKDTTETLGDMGRFLTNEAYFEGDDFFGRTPSV

MTYDYEAGSILRQDGRVWITGLSDEGGTGAYVAAVLKQVLQPRADEIKKLDDFVQKVVWG

KIQKEDYGVVKSVFFYEPDKAPGYQYSNDIWWGSWTSWNKDQADSFDRAYNYVHVTVAYW

TMYRVARAYPDTVSKTWDWYLDQAQKTIMRMMDPSVGYNLVGLMGETVFGEVLKDLKRES

KTDMATALEAEMRKRAEHWHSLEVPFGSEMAWDSTGQEGVYYWTRHFGFEDSELKTINSV

LGYTPNVPHWGWDGNARRYWDFVYGGKLQRIERQIHHYGSGLNGQVLLGAFRDDPTDNYL

IQTGYAAISGPLSNINQDGFPSAAFHSFPDTLKWDGISGDYGGGFLGMALNGGTYVADDK

DLGIVVYGGVIDRNERGTITVTPKGAVRHRVYIGPLKVLIETDAAVVEQFRFDAQRRTIW

LSLGQDRDAPKANETAVWVESLASGVSWTAKAGGGVKEGRGGWLVSLPASGSVAVNLSAI

PHHSIAWPRRHTGNISQLFVSGWKCDKCRCVVEVVEVVEAPISSRPTVLRPLAEGQPQPA

LQRPLSPGQPPPRELRRPRAISRSYHPAHRETEPLRQPRFNEHANENERLDTSLSPRDVL

AARQRETPEFDPADLSTWETRAPSFLHGGAIFPAARRSLMPIEESFQDKVEAPQRPSSLG

TAAADPESKEFLDRVNECLLLPKPRTLEVNEAVRRVRERTASSEYPSEGQCSPPERVVAS

PAQGSSMNLKELRKYGSATYNLSDIRSRTSSSHSRASIHHNDRDVIAEAEAVITEWLGES

KHGQSRASSHRRYELADSCEVLPLDSLSQQGGSTESLAIVTPAPKAPTTTNSRRPSEVTF

GKTLRTPKEHQHFRDSPQVESPDPSKEPPRLSARLGNGKRTASHFSLRSLTESLSKRPRL

GIKKLAGSVYKGGKRALAHMRQNLKQHNNQAKWDFEAWKAKRRRERPADSPKGKPGKSFG

GFASENTRRRDAEWWSEGVRKYQVPDWMKFR

>SoG_06800.T1

MKTQVPVFGLLLPAMASATQFAERCASLEQTLVEQTENTTIIATTFVSAGTVLLEPARHP

SCQALNQTSKFDFCRVQLTAHTGPESEVVMETWLPVGWSGRWMTTGNGGLGGCIGYSDMS

YGAGRGFAVVGTNNGHDGNYGEPFFHKPGVLEDFAYRAVRVGTQLGKQISRLFYGKEHTK

SYYLGCSTGGRQGFMEAQRFPEDFDGIVAGAPAFDFTSLQVFSGSMYGITGSNTSATFLT

PELWGLVVNDTLAQCDHLDGHVDGVIEDPDLCQYRPERLQCGPGQTSGCLTGTQVETVRR

IFSPFYDTRGRLVYPRMQPGVDSSAVLWSGKPFPYAADWWKYVVYNNASWDGNLTVDDIE

PARRINNYGIDAFSGDLSGVRDRGSKIIHYHGLQDNLISSDNSARYYDLVSRTMGLRSRD

LDSFYRYFRISGMAHCGGGAGAYFIGNRASTHAADSPDQDVVSAIVAWVEQGAAPESILG

TAYADDARTNVKFQRRHCKYPLRNVYVRGDPNLAQSWQCQ

>SoG_06807.T1

MRWADLCWALSLVAGTVEGKHVDIQYTSVGGFFLQDDPSTNPKNFDYAQVNLGLMNRSYP

TDGSFDPHGQQPQWAKFANYVKQLNRDSCHQNKGKGTRTVYKVLVMGRHGQGWHNAAESY

YGTPAWNCYWAELNGNGTAVWADAHLTTAGESQALKANAFFKSHFGKEGLPYFQSYYSSP

LTRCTQTAELTFRGIDMPPTRSFRPVIKELLRESISIHTCDHRSTKSYIRSIVPGFRFEE

GFTENDELWRRTMGETDEHQLERSKTVLDDIFTHDTATWISITSHSGEISSLLKALGHRS

FSLSTGQIIPVLVKAEMKRVDSQPATTTAAGFTSEATCTSPPITSVDPQGCGPGDANPNP

SHFLGPHYRQQSHIHPLSHELSLTRLNVAFSIIKRVNSSLRSCLEIALPGISGPVILSLH

RSSRVPNNMDPAIEREGLQGPPAPELSEGRLWVDGCFDFFHHGHAGAIVQARQLGDELYA

GVHSDEDILANKGPTVMTLDERLAATNACRWVTRSIGHAPYVTSLPYITHYGCKYVVHGD

DITSDSEGNDCYRFVKEAGRFKVVKRSPGISTTYLVGRMLLCTKGHFIKSLEAVLEGTEG

NGTEEERKALGASMLERMKLYATDETAKAPGARVWFWSASEPAKAEDTREEKGTFRKLID

GKGPQPGQRVVYVDGGFDLFSSGHIEFLRKVVEAENELARQDGWFSEQAVNERLGKGAEY

PPAYVVVGVHDDQVINRWKGVNYPIMNIFERGLCVLQCKYIQEVIFGAPFTPTESYLTSL

PLGRPDAIYHGPTSFMPLTYDPYTAPKSMGIYREIGEHAFAGVNAGEIVQRIMKSRDLYE

ARQKAKGVKAEVEQAAREREILEEEQRKKEEAMTK

>SoG_06810.T1

MFTKSLFIAGLASLASAHIKMSSPVPYGAATLDNSPLEADGSNFPCKQRPGVYDAAGASN

VFPQGSTQQLQFVGTAVHGGGSCQVSITTDLKPDAKSVWKVIKSIEGGCPAKGAEGNIGN

DAAAKVPFSYDYTIPAELAAGNYTLAWTWQNKIGNREMYMNCAPLQVTGAKSDKGFLNTL

PDMFVANIGNGCETTHGKDVKYPNPGKDVDQFNGATNVFEMPTAPGCQKPTGGSAQPTTA

PPSQPSAPAPNPTVPGGIFITVPSGGGSAPAPTQPPSAPEPTPEKPIEKPIDQPKPEQPV

EKPVEKPGNTAPAPAPAPAPAPVPANPGSGSGSSPGGGFAAGSACTQEGVWNCVGGSSFQ

RCASGAWSTVMNLAQGVKCTGGQSPTLNMAAAKGKRTLRHAARII

>SoG_06835.T1

MRVLAVLAPVALLATQVAGQLSVIQGVVSNIRSDVVAYGAAFTANDAAQIGAGSDKLLAT

AAAGTATVQAANSVSLIEALSLRSSIQALQADVQTTYDNAVARKAAVTALGIAAEVHSDF

VTQRSTVAVLGAALASKVPSSVRPLAQTYINNIDAILVNAIAAYA

>SoG_06876.T1

MKLTHGLTAALAGLTSAAGTQQSAQVYLVPSSDSASSQSPASVSASLARLIFLQRLSPLG

EGPSVLDVTGGTKLDDVVDAMNRFGKAPPARWPSSGSRSRALTRA

>SoG_06878.T1

MQFKLLTLLTALVAPVAVTCTCPPRNPDGFEAGTELKKWFTDTVEDFFPFRDGWEKGYDE

HFSRDVTATFNGTWYDFDGLKGFYKNRANPILQHAFKGTFQYGFSGVVAVPFPGTVRGGW

VYATVWEFGYRWGNPDLPWNVTLGAFGLVEEESDCKLRFSEWREAGNLGHI

>SoG_06905.T1

MRFTVSVLALATVASAFPFPPKALHKGRHGLDNKPVAKPGDKQTYEEAVNKLMGFAYFDV

ERAGPLAHGNDDNAIRVAESVLRHYEER

>SoG_06977.T1

MRSLIPFLTLLSVANALFFYIDGSSTKCFFEELPKGTLVVGHYSGEEWDDRSGSWQQHQG

INIFISVDEIFDNDHRVFTQKGSSSGKFHFSAAESGDHKLCFTPSSTSGRSGWLSTSNPN

GGIRLTLDLVIGETNEIESTDKDKLETISNRVKDLNARLNDIKREQVFQREREAEFRDQS

EATNSRVIRWMLIQLAALGITCAWQLSHLRSFFIKQKLT

>SoG_07050.T1

MVQFTSHAAAWAIAFSLIPTSMAQISHPSQLPALQPGWDEGGKIIPGLLAGLPPMGVRWV

EDWASIHILDWCKGQTQGFGLNPNDVETFNVHYDDCAEPWIMCRHKDAGASKQQMVEYWG

RIPLPMREVVRHIGLWPSHSDAAYSSGDNIAFVGPGIFRLPVIIHETAHSVDGYLLRHID

SNIPFSHGSTWRNIVSRDTAAISEYGRTGWADNFAESMSIAVTDHNAPGGVGPMHPNPFP

AYWQFVGIRELIPEVIKSRAKGQCNWRHRNGNAVLKNDGLRTSAEEAPLPDTGFKTDMKV

IEWSGDNTTYVDEF

>SoG_07103.T1

MQPYGMNTLALLGALPLLSGLAEASSTLFWGGTIVGFNNDTNSLEVFRNGSILVENDRIA

EIHEEAKPKDEAGDDIEIVDVTGQILTPGFIDTHRHGWQTGLKTLGSNTTLGEYFGRYGS

PASGQDFRAEDVYIGQLAGIYEALNAGVTTMVDHAHHTWTNDTAWAGLNASVESTARVFW

CYAFDNGTGIDGPRTVDEQISNFRQMVDGIDWENTTTEIGIAYDSWGPRPDVNERDRVMN

LAAEFNVSVITTHSLGGPWGITNMPTDLAGLDIITGDIPIIFSHASFLSANDRELLRKNN

HYVSITPESEMHYGHTHPHSYLIQDQAALGVDTHFTFSTDILTQARIWLQSTRYRLFNQV

LENWKIPRNTPETVNQAFLLATRNGGLSLRRHDLGVLSKGAKADIVVWNARESPALLGWN

DPVAAVMLHASVGDVHDVMVNGKFVKRNGKLAIDGYDEVRERFLASAKRLQDLWANKEYP

VLEGGWSVGRSEFADTMKADVVRGEGDGYGELFVE

>SoG_07132.T1

MLFSIFITAIMAIVVSATPIHHKRELGGVSLPYLQSTRPNPPSGITSIVSSGALLVTLDQ

ILLCTGANSTGTCTHMVAPLSSVGDVKCQQLDKPYFQNTSTFAPDGEDFECFPRLYSCGE

ICRSPTGCTFGPVDFAYENKYDLGRIEWNNLIQSFDCALKKKNKKTTTTEGEEETPKY

>SoG_07196.T1

MKHLILACFALQQAFATTSFEQDKGTRHIWQKPPGKDLRIKFLDVPTAYDDALVKHADLV

GMVDVFTDGQTWLMMDGKRLFQAPTDGTVDPEAVRLSTDVEVMSPDELREALKTKPSYDE

AYAKIRKVLDDYHAAGGALVERDSLHCTLIWCHNNNDCYEATDTEGACNGCRNHDCKWNR

GIYQNAIHMSPYETGDTQGKDSD

>SoG_07211.T1

MSTIASVFIALAALLSSAVAAPGRQNGKADGQLDGPLHFNVDGSFHISVMQDTHMGDFAY

EVYGPQQDAATLEVMRSVLDEEKPDLVVLNGDIMQGDRLVLENATQYVDLVVSPMLERGL

PWASTYGNHDHQYNITGKTILEAEQKYRNSRTRSMVHHRDAGASNYWLPVYAADCLDCKK

CAPELLLWFFDSRGGVRFQELDSKGQKVPNPSWVHESVISWFTKTREHLERRYRKVVPSL

GFVHMPTNASRALQASTWDPKHHPGMNQDVPIHGQASNYCSDGKLGNCTYGGQDEPFIRA

IASARGMLGLFSGHDHGQTWCVKWDTLIQGTDLKGNGANLCFGQHTGFGGYSNWIRGSRQ

IRLSREGLKSAELDVWNRLQDGRVVGAVTLNSTYGTDVYPEVAQTEKSFCDCDYPEDKQL

>SoG_07225.T1

MKSILAASVASLLAGNALAESMSVLKELKVKQWAKQEAEGAFDLDRYEALQAATACTNGR

AGEYRCSNVDLVSFLRHQDMGSATRTGNDVWGWTSSTGREFGAVGQTDGTAFVEVLSDGS

LVYLGRLPTQTVNSSWRDMKVIGNYLYVGAESSNHGLQIFDMRKLLTVNPASPRVFSITS

DLTAHFRGFGNSHNIVAHPETNMIYAVGTGSAAGCRGGLFMVDVSNPASPRSPGCLSAGG

YVHDAQCVIYTGPDARYRGREICFNYNEDTLDITDVTSKANPVTISSTGYNGASYTHQGW

LADPEMRFLLLDDELDEQRRNGPASNQRTTTYIVNISDLRRPVFTGTYQSPAVSIDHNQY

VHNGLSYQANYGSGLRIIDVRSLSTDSTGRGIREVGFFDCYPEDDSVGGRAEFTGTWSVY

PYFKSGFILLNSIERGIFSLKYTG

>SoG_07234.T1

MHSQSILLITLGVSQGLALSATGGVISNRYALPVALMAKPALGVPVLDFLYARVPGQKAN

QNADAANGGAANADEENANAGEAGAVNAGAGNAGAGNADAGKAGAGKAGAGNAGAGKAGA

GNADAGNAGAGNGGAGKKKAGNANAGNGAGQACNANGNVNNGNLNAGRNGTANANDGNAG

AGRKAGAGRKKAGNANAGDAANAGAGNNAGAGNKAGAGNRAGAGNRAGAGNRAGAGKNKA

GNADAGNANAGNANAGEAANAGAGNKANGGNDNKGQRANQRANRNGNQNEEQEDAEDEE

>SoG_07235.T1

MLYSWMKPFLSVACLSSAAFATAITSNVPLLKQQAVQNYRGFPVPYPTEAYWQDPPHRIA

NLRTTEELPTEEVFDYVIIGSGISGAAVAYKLLTRDPDLSVLMLEGRTAASGASGRNGGH

CKAGSYKSVKTWIDKYGEDEGLRIAKMEQDCVDDLRDFVLSNNVSSGWQDVETADIYWTT

EAFKSAMETVRYQKELEARRPGDAPQNNKRTIYAGDAAREKWQWPEILGAVVYRGHTQNP

YHTVCAMLEMSLRRGLNLQTNTMALGLGLNKVPHGTEWQVKTERGTVRGKQIVLATNAYT

NALHPGFARTNFITPDRSQASAVRPSRDTSGSPVFRRSSSYPDLHSGNNYIAAVRAPGAA

SDVVIGGSTQFSPTREKNITDDSEINEDIANNLRKVGRVVYGHRNWGADTEVLKDWTGIT

CSTPDGLPLVGGVPREEGLWAIVCMNGHGMAWSFRSAEALVDIMLEGDAKDWFPKAFRSE

RAWS

>SoG_07245.T1

MQFSIIYVATFVAAAMAATTGASGTKPCPQTLKPKTATTSGREQRSVMIMRREEVTNPCV

SSIDVTKRQNRANVNQPVMADAQGNVLPFNAANVYLPSKGQRRNKRFADEQ

>SoG_07253.T1

MVPLRLLTPLALATLAFAGDPKSCPSDIPLSCQNTTVVEDTCCFITSGQLLLTQFWDTNP

ATGPSNSWTIHGLWPDHCDGTYPANCDKSRAYTNITNIVASGASNGTLAYMNKYWKDYQG

DDESFWEHEWSKHGTCISTLEPECYQNYKPTEEAVAYFERTVALFKTLPTYQWLAEGGIV

PSNTKTYALKDIKAILKAKHGAEVTVSCKDQKLNEVWYHYNVKGSLQEGDFIAAEPDGTK

GSCPSKVKYEVKSGQQSRMRRVYDEQSERTNAEMHIY

>SoG_07299.T1

MVGFASPWARLSVSGLAMAALLAFANSAEASHGRFLTVCSKLPVEYTTASTARVVNAPNL

QGNRACVDVEFAPKPRGHGTYYGKVIWTNDSPQYERYPMEIDETLTISADGKCSASYQVS

YNGPNGHWQWIDSEGCQFENGSEVVFGSN

>SoG_07304.T1

MKTLLLFFLPACLGAVIHEEQREENTYPIPSSHTYSYVRVSYPTPSVTYPRSEATTSSTY

LSHDDSSALTLPVTQSWEPLHTYSTFPMPVPSVTYPRDDEDDGGEGGWETETETVVLTRT

STKIVTVYATESMQFEENPAVLDSDGGVETEKPGGCQ

>SoG_07330.T1

MSRLFKSLSLAGVALLDLVSAIKDYPVQRQPVVLVQNTPIILSTLVCSDTALSFGSTTIN

VTSAPTLLVTSFNVAETSTISAGSGITSFNGPFTTINGGQWSGPGFSTLTLPPSRPDGSA

TKIIFTPAPTATSTIDTVHSSAINSGGIPSSASASDSASGSFTRGSGPQSTGSAAQSAGA

SGFVSSTRRSGPQSTGSSGPGSSTGGSGAQSTGSAASGSSTGSSGSQSTGGSGFGSSTGG

GGGQSTGSAGPGSSTEGSAAQSTGGTGHSTGISGSDSPTGGSEAQSTGSTGSSSFTGSSG

AQPVNSAVPGSSTGGPGAQTTGGQSAGGSRSGSSTGDPGAQSTGGAGSGSFTGSSGGQST

SGAGQSTGGSGPQPTGGSGVQSTAGSTSRSPTGGSGAQRFWLSFHSRSRSILN

>SoG_07333.T1

MKHFYLSAFALLAPAASCARSTQGTDSCPISSKAIVGDACASYAALERLNAKIKPAVDDL

TRTTDYFSHYRLNLFNKKCPFWDEENGFCANIGCAVETLDNEEDVPEVWRAKELSKLKGP

RAKHPGKKARSQHPDRPLRGELGDDVGESCVVEEDDECDDRDYCAPEDENDAAKGDYVSL

AQNPERFTGYSGEGSWQVWNAIYSENCFAKSSFPHSAGLGQSSSNWAAGPAQQDFKQILN

AAGRHAQLQQWGKEEKKGPFVANTPFEGEDECLEKRVFYRVISGMHASISAHLCWDFLNQ

STGEWQPNVACYKSRLHEHPDRISNLYFNYALITRAVAKLGPYLQKPEYTFCTGDPSEDA

ATRAKVREVTDRAASVPQTLDESLMFVNGEGPSLKEDFRNRFRNVSRIMDCVGCDKCRLW

GKVQTNGYGTALKVLFEFDNSKDDVKLPPLKRTEIVALFNTYARISNSLSAIGKFRTMVE

GLEPEEIIAGEDEEDGWPEDTPKESVNEKSNPELEEFIRLRNKRMENPQMKDVFEHEFAQ

VILAFKVVLRSWMNVPRALWNMFTSEAKRLYQFWVGIPVTPRNWKIELPNLNREDL

>SoG_07346.T1

MQLPNAALWSLLLAGAEAELRFVRRETNLCSKEIVETIVKTLTITATEEVVLPCQTAGHG

SGPAPGRPGPPGPPGPPPADGRSASITTTASYGSPSSQPGKGNGGKKENLDSNLPFDSLI

PSDYFPEITLALPTMTLSAPSSPDTTPFEPGSPDDEGPTSLPTSVASSAKPIQSSGSEKP

GYSNPGGDTQPSGPSTPAGRETTTSSKPEASSHVSSSFSSVETGMPVSSVSSSPESSETG

NYGAPPDEETTEPSQPAETTTSSDGLTSASTGGASVSSQSASGTPGLSATTGSSATTGSS

SSSGSVSQATTTTTTTSAAQTTTTRASTTTSAAHSTTSAAQTTSTLSTTTSAAQSTTSAA

QTTTTRASTTTSAAQSTTSTSSSSSSTRSSSTPAPAPTYVRRYAADMVDKLQDASMDKLK

EYLKSQGGSDCTIENAAVRREWGDLSLAERKEYTTAVQCLKERPPRTPSSVARGAKSRYD

DFIVTHIQQTNLIHNTASFLSWHRYFTWAYEKALRDECGYTGYQPYWNWARYASDPANSP

IFDGSEGSLGGSSQGSDGCAPPGPFKELTVSMGPGNILDYNPRCIKRDASQYWAQQCTAD

KTMIVLNEPDIGTFQDRMQYGNSIHAGGHFTIGGDPGGDVYASPGDPIFYLHHAMVDRVW

WIWQLQDLDTRLSAVDGRVGGGSNRRGTLQDSISLGYADEGAVLGELLNTTGGKGGAFCY

IYY

>SoG_07375.T1

MRTSQICGATAVLVGGALAARPFLNEPDTGIDEVLKDLPVGQLPDLKDMIAIPDFDWAAR

HYLPITNYTYYRNGAAGEWSYTANLEVFQQYHFRPRVMVDVANIENTLPTSMLGHNFSAP

FYISPCARAERGHPEAEVNFVKGAAAGDILYVPALYSAKTMEEIYDARKEGQILWQQVYL

TGNDTSNAAILARAEKNGAKAIVWTVDSAAGSIRQRAARFSVGSANTEYQSFTWDLYRKL

TEMTDLPIVLKGISNYEDAVLAVEHGVPAIILSNHGGRQLDTTQTSLETALEIHNEAPDV

FKKIEVYADGGVRYGTDALKLLALGVRAVGLGRPFMYANIFGVDGVKKVVDIMKKEIAVN

AGNLGLNDLKNITAKSVKWTSRGYSG

>SoG_07429.T1

MNPSALLLWSLPTLFSTGLAVKTIGEIFTVDFSASANGGCDEPGAMVLNQEVNDCYTLAQ

AGIDLLQSFNSGNDPAAKRLVDVYFRTGRTPSSEAREKAMILATYTSVSNLLRNGPSGTP

ARLFCNSDWLTRTRKRQVLTSQSPALDISGNPIKTINGQTATLQEVYQTKWRKWINGGLV

PYWAPVYNAYIMDANYGGPNVGYCDEGGNKGATQDQTSPPSITICPRAFSVSLKTLSPPP

KIKTTTVQDIQKNPENPSATGLQALLERLAPLTLYHELFHLVLTPKGSPDTTYEVSEMIN

MNKYPQYNSALLATNPETYVAASYAWWLFKQTGAEFFTGYATQS

>SoG_07432.T1

MRSRILLLAIGLCIADTALASPCKPRHSSSLASTTVSVASTTTSTSAAEATSTTLSLTES

YTSTTVESSNSIASTTQSTSSTGQASTSVISGTPTETTTSSTSVALSTTTTPVTTPSSTS

SSAASSTTPAPVATPTFTIVGGSGAVNGASLQGVDQGGSVLLFNPQTGNLATRTFILDPN

TGRLRDQKTGISVCAYYGNAASPSTPANVALCQDGNTGKNAFYDYLTCQVISGKLACAAP

RASCPEDDNGFPTCATDPGSDVNNQFYYRLTQGNGYYLYISSGSPNSYTSVDLVAQT

>SoG_07499.T1

MRGLRALSWLAITASGLTYVVAESDTCGTDYGVFQSVSDDLIVCFESARVISIVVGNEAK

SQCEVCTGRAFNDCANCVTVVGPCACQITVPTTTTVLATMAVTRPYQRLPSWDLDLSSPR

LPAPPCPLEHQRPLPLLLSLRLASLPGFNSFALWGCAGSTNNFPGFTLRTSSDLMTLELC

ASSCSGSAFFGVYNRDCHCGANLDSIVRVSNINQCSTSCPGNSCEICGGLASLERRQTAS

RNILLTVYANLVPPVLSSPASGFTSTTEIASFSSAQSSSSSSGSITGRQNSTTSGPPPVL

STTTLTDSSTSSTTVSSSASSQTPIPSFLVNLNLNLNSSFNLLNSNFNFVHFNSNFVHAN

SNSDSDSDSNPNSNAGTFDIYLQRAMVGRGPIRPSGEDLGALGYNPLDNYLYSTSGSHEL

VQIVQIRKDGSYSAVQSFSSPHTWTVGDFDSNGNFWIATQDGSAFKVIDLDPNSASYGQV

TNSGTVTAPGYRVADWAYVPAVSGGMHSVVTVSGGQVAVMVFDFTTLQWRTVQSYGTTLE

PSPNFLAMYSDGDSIVAESTSAFWKLGVTALASSRIWTVSSGISVASGARCQLAPNL

>SoG_07500.T1

MKTSIPTAVTALLLVTVAASPVSPAMSNLLAARDSIDDNFISPFDSNTAASPEIDAVRHA

LQNAETYTERQNALIPNPPDATNFTFTFINNTVHRNTGGTVALGTRQNFPALIGTEVAQA

VGWINPCGLNVPHSHPRANEWLTVIRGKLVGGFVLEANNESNGDVVGQPDSVSGPEPLVN

VTMNTFTGMLFPKGQAHWQFNPTCEPAVFTAAFDSSDPGRLQVARNFFSNYPDLIIQEAL

GDVEILSGEDIERIRHSIPNAYATVMHECAKRCNLLGNAE

>SoG_07507.T1

MRFATTIALCFGALHHVSAFQNWIDKTIAHTGEPIGKEIDDNGLTIYVSKPTCKPKKTAV

IYITDVFGINLTENKLLADSFSRAGYLTVAPDMFDGQPAPEDLNAPGFNATEFLLAHGPD

VADPKIERSIAYARDLCGADCGIALTGYCYGGRFALRFVADGKGGDVAFAAHPSGLEDGE

VSSIKGPTSVAAAEVDTITPKQRTRQIQDILETTDQPYQVTTYSGTMHGFGVRANVSNPQ

EKFGKETAFLQAVHWFDSWA

>SoG_07526.T1

MMNTIVALSLLASSVAAQQITDAPKALFKQASSLNLAAANGNGQVCLDISNLLLQCSNKW

GGVDKIESQPQDELVGCACCDGGRGVYSDYSSCSAYLRTAVPSLTSEADVYGQLATQCSS

LASCSASQRGSASASASSATQRASEASGGTGTITSPRGSITSAAVLEATECNLMSSMFVS

CQSATPRFADLGYKSQAQCYW

>SoG_07544.T1

MKTVSFLSVAFLAASALAAPLVPVDTNNQGGVDSVVKEVTNVAGGAVGGAVGTVNQVLRE

RDIADAAGFLSALKGTQSDLSGPLGSLQEITEGVRAGDITKQEATRQAAPQLKQVEKVLS

GLTSELSGVTGRLNVASIDVGGVTSLVNVLVAEVLTTVSDLTTVLGIQAPLASILTSVVG

LVARLLNLVVGLVGELVPGIIAGLTPLLAGVGSGVLAPVLTPVASLLAGLQAPQRQIATG

CHVIAQEHPVSFKLCISNEKIVNERAKFFKITGSLNLVPNQQLGEKHHPEVRMGGHVNMM

DDVLISNPLQSVPPDLLRSTLRLLVSLSRDNRRAFLSHARGKLATLETKDVVEPTRLFPG

NGDASAESWDYLAETRCLFSAKMPLQAVARLSRFCDAVVTAEASWTPGSELEKMLETFGG

DTIQSMQALKEEEATAAGIPGTDELQRGLRVLARSLVDRRKYCRGASLTYPLARAERQVV

DAYCFFFPSAAAAGPLEVGVVETEEERAIEIDMATSGGSIEMVEIGKMRFPRLLNGLWQL

SSPAWGSASAESQDEALEKLIQHGLVAADMADHYSPRPSGRCRAGLRELPQQAVSRHAGR

RPRRDEVVRLQEDRGAHDGGMGAAERAGTVQEAEGACRSPPVPLVQLRRPCLALHPGVSR

SLDEESAGLGQGGWALQLRLAAHGGGL

>SoG_07557.T1

MKFTAVTVAFLATLAAAAPAPECKPGTYDCLPNKTGWRVCNTSSKWVFAGTCPPDTVCKF

YQPSLSPYCVPPNFQFP

>SoG_07612.T1

MTPFIAACLCAAAVGLASADSGRHAERFRSVAIEYTTVYVDLAGAMDMCARGELTCAPTN

VPNFPSTTEHGVTRLDVTRFRDCQGVQELTLACCRSVQDCSHGHSCGTNGVCESLSGPKC

QYCHECQGGHMCNSNGHCQKIPGSSCEADDDCTSAAICSDAGVCELTQGDPCFGNSECAH

GQICNSRGVCQSVALPECSSAGECPEGSICDARGVCAVTDNEDRCDETSDCNGNEICNSQ

NICQRSERPLCASQSDCLQDESCTDGFCSRGPEDKEYLCNE

>SoG_07708.T1

MRAVTLTILAGLAAPVLAWNTDVHHQIGYSAEKFLSPEAKEIIGSILEPEADGSLGRIAA

WADAYRKEPEGRHTTTWHWINGADDPPRFCNLYLNRDCTSDGCIVAALGSQTQILKGCIK

QAKFGRLNTTETKCANAVKFITHFTQDIAQPLHVTGQARGGNDHAVVFGGEKTNLHAIWD

GRIVYTLANVTKFSTGGIDPYFENLVERLRADTLFVPRAEMTACSDPGTPTACALAWARD

ANEWNCDYAFSQDFNATDLLTSGYAAGAWPIAEIQIAKAIVRIATWFNKLVENCYHERDV

VLDLVPSWVGGPNSGA

>SoG_07790.T1

MRLTATALLPALAGANLPVASDIGHALQVTLLADTNRDGFVDENDLSDRDVWTRDKGALF

LANIGDTDQRCSTKLIDPEYHWNVTDEEVLRMERYLDECNDASDEVQRNAKYLAPLPPQT

RIFVRSPDVSDGDDEWIYVSRNRTFKMNEFVNGLELGVDARDVRRPGPEGWDGRVTIEFA

IFSRGKRIATDSVAMRVAPVLTHHHLHNTQRTFVTSGPAPDGDEPVDPELSQFASEFLET

SERAGIKEPTFKFQHWDRWTQDFFEAGFMSIPGPKGPKVLRVMIRSSQTQRTSGRQVFYK

LRSGSVGAVQHVGLGNTIDATGNLETVPPFSHDGVSYPSGRIIMGRTEGQEPLIMEFLRA

QEEQSTIIVDSDWLLVKHVDEFLQFLPAANERRWVLMVQDHIAGHQLLKTAAASNGSQVA

ISRPAHSNDQLNCQYNDTLDDVLAYPYLYESQIFADQKVNASFEILKRETGLRGDEIFHV

PGLATNWITRDFKCWSLQVPPPYDDEWHQKCVRKENASTSFLKLQALSGPWGRAKSILDA

SMPPSYKRTQSQRRQQVGIDFPSECPDNFEGFPAWMKQTLALFPSSLNSVVMTDSLILAA

KPWGPVIDGIDVFEEAIAAGYRKVGFNVTFQDNWFTHHLLQGEVHCGTNVWRETDVAWWL

R

>SoG_07799.T1

MCCELFPTLALASWLSAIQQADAAPSPPSSSDVVGTEKISIIKIVVGREAAISQCEVCAD

RAPGDCDNCVIVLGPCPSSVTSPVTSTLTAGGQTTVFTLTAKEFGAAATNNANNPASASN

VQGPAPEAGEAAQPPSYNCSAVAGTELPGVVGSFDAAPRASAKTASMELGSAFTKLLPTA

TGTELPKCRGSLLLFGCLASPEGFPSFFLTQSSVYMDMAVCSGVCNSKSFFGISGYDCYC

GRTFDVSAMVSLICRRQAAPISTLLAVYALPAAMPSSVATTFSSTDKSSPTENSASLASR

SSVISSSESSSTPVITLSNNPAGMLSSTSAGSLAYSRFTPAGTFIKTTISMPMNNTANKP

TSTRNDTSIAAAKGTPPNTAANMAETVGSTTLIRTPVHTLVKTPSIDPTTLANATTSPTA

TATPINTLDTMMNTTSSLGAAVITATLLSNATVTPVHTTIVHTPLTPIFANNTSPHTLVP

TSTETTITPVSDSMITLVQSPVATPFTSSSLSNATITPANTLVGSSSNSTTTSINTTVYS

QADTLITSSQNATRLLADSPASTWITNSLTPTIAPADSSTTHTLINTPVNSLVNSSMSSG

SNYMISPVKTPVNTLITYTPSSNPTSTTTNTPVSTAQNGTTYPVESPSSTLSLVSSNTIS

LTTLDPHTPNDAIVSTRSTITLLSVTKTRGDTPIRTSAISGTATIAMPPETPKSDSSTAT

PFSSETSRSSITPGLKRRHLDYIVSGILIISDGIGTVSIGAAGKMCALGYNSIDDYFTER

GLSNLNRNIGNCGRFYTGSITTHAPATVHDWVSLPGSQDLWTVIVDSTGDSLLMNLATEL

DIVYDIPRWEPVIDDKQSPARSVGFQRVDPFYVMSLIKITSTTPSNLSSGARCAAALNV

>SoG_07813.T1

MRMSNRWRDTLILGLLSHHTIAADLPTVSHSDSRGPHHAIETCRCEAQIFDYATHILQPV

CLPSVRTEPITTTLKPSGGSDSPLAKATTTGLVGDEGDGGSIADGAGAHFMSFEDWKEEM

LKRTGQDPQELRTRKANDPRKPSDGRIPEDGGSAWGDEEEIELDFGEYTHPQTHNHEKGE

DDTARGVEEYGHDSAIAYDHGKGFLHRSKDAGKTCKERFSYSSFDAGATILKTSSGTKNA

KAILVENKDSYMLLECGRENKFVIVELSDDILLDTVVIANFEFFSSMIRRFRISVSDRYP

VKVDKWKELGTFEARNSRDIQAFLVENPQIWAKYIRIEFLTHYGNEYYCPVSLIRIHGTR

MLDSWKDTDGAPGEDSAEDDDREARQCEDLAVEISTTDASAVSVQESQTVEQNLIPTHET

MTSMDLFAIFEATCPALPTQLAATGRFVSNMTRTGTDVSSPTHHQASKDVEPTSSGDIAS

TPDRLPASSGFQSPQLSAQQTSDQILSPTNIPASTEKAAVDQIPPAESPSVKGTPTSQPP

KQPQSGNSGPRSRGNPTSNAASASPTVQEGFFNSITKRLVQVESNFTLSMKYVEDQSRHI

QEAMQRMEQKQSQRITLALDALNSTLTSELHTMREQHDQLWQFTLRALENQRERNEQDAV

ALSARLNVLADEVVFQKRMAIAQAVLLLSCLVLVIFSRGVPLPYLAHPQQVGEHNSGLAS

SQSPPSAAHCHGPASSDVSLSSNLPSVRLMTPESTITDSLMDGNTFEADNIGSHPRADSS

YHGTYTRFYRPSPPLTPRTHAEDELKYGELGTEGGMSRLPASDQHNSRKPLPALPEHP

>SoG_07851.T1

MKFSTVLAGIVAISGVTASSWFPGTKVAYNKWHETELERWLSDHSIPYPTPADRRDLESL

VEKNWNNFVVAPYSSWDTAELSSFLQAKGKQAEVEAEATKDSLLNEVRSNWYETEENAHA

AWGSVRDWILDTWTESQLKAFCDKRGIPVPQPRHRDTILQKARTSYDSVAKKLGETAAYP

GNWLYETWTESELKEWLDTHGFPAPQPSSRDKLIASVRRNSRLAYLQAQSQAASASASAQ

AAYATLTDMIIDAWSESQLKEFADKNGIPVPQGTKANELRALVRKNRANILSSASSAYGA

ATSNAQNQFASASDSASLAVQDAFNSIVNTWSDSRLKAYLDARGVPVPQGSKQDDLRALV

RKHSHKAATGYNAWTFDDFSVEKIRDYLSKHGDAAAQQASKKKDATRNELVSVAQSAYSS

ASKTGGAGYAAATNYLALATGSAKGNAFDSWSETDLKAYLDSYGIPVPQGSKLEELKAQA

RKQATYFRYGTSSPGGTFLAKVEETLHNGWNWVAAQLQLGGEVASAKASEASAEAKAKVN

KEL

>SoG_07865.T1

MRGVLVRIVVALAMSQTGSAAMNVKIFADTNRDGKVDVNGKTDSTGKCSIGPEEALFLPA

ITDTDRRCSKNAHNLENEELEKCHDGTDDVLHHPERLAHIRTVPLKNIAKTVYGSIAISC

DGSFPSCTDKVRLFHRSGTGVWNYLSENHVFQDTALVDGLVLGIDGRDIRRPNEWDGRVT

INFNVWDNVTSETFTDCVKMRVSPILTHHHSQTAKRVFTTGSHLLDTTQTQFVKELREHV

EANGIELQVFEDTLDIWTQDFFEPGYTSIPGPDGPIGIRVMFRSPQISRSSGRLVFEQLR

SADVGAVQYFDRDWGRGHTLDSTGNLETIPPYRNGDANFPAGRVYQGSWGNQKPTGLSFF

AAQEAQAPLELDTSFLLVGHVDEFMQFLPAENERGFVVVVDDPRAGMELLLNASAAGHGG

VMALSRVIQEYDGPWACLPSDSIDEVLGFDGFTSDNDYASNIIEKNLATLKHEIGLGDGD

IFRLPALFYSGWWECPWWQEDPCGDSSDCTATPAPEPLPTPAGGDETMEIQRLKRPGTAR

KNSGKKNILEAVNGETPKRMTKAKMTRRQENDGPPKTTAFFPGIVNGLVLPNNVVLGPKP

WGPIIDDVDIIAQAAVEVYDKAGAKLIFMDDYFSHHVGAGEVHCGSNSWRNADAAWWE

>SoG_07904.T1

MSLKLAALAAALLASSAHGHAVPPQARGSRFSIDAVTPSEPEPFDPAAEIRRLHAKYAVT

TDRRFRETRDNAQQGSIPVTPSTSGLSFYVPTTVGNQTFSLIFDTGSADLWVYSNESSPW

QSLDHPVYIPTSSAELMPNYTWSIKYSGGESVSGVVFTDTVQAGSLVVHKQAVEAATVIP

YEFSSDGILGLAFSTINQVKPVAQKTFFENLMPTLERKLFAANLRADGKSSTWDFGFIDD

SKFTGDITYTPVVSKKHWTINVGPYAVGSGAFTGSDQQIGPVVTDSGTSLVYLPDAVVDA

YYGQIEGYELTEGGTHTFPCNGTVPDLHFKIEDATMTIPGSLVNYGVYDKSKNTCAGAIS

TQGRMKNSVLGNLFMKRYYVIHSMEDETPKMGFAPFA

>SoG_07919.T1

MPQISKVLALLTAVTSTLGAPAIHPRQLAGEGSACNSLLSSSDNGVGYGTENAENNLASL

LGGTPSSGGGTGNGTPPPPPPPGGKVKRQGDKIANGAANVLNALGQVQLAGLVQTDGDNV

DGQLTGDAATVGEQVGSDEVDVLERLGNVVPGQAPTAV

>SoG_07945.T1

MLSSPRGSSLTGLALLLGLANASPALRERADQVRPVVIVEKTPIYLSTLVCSPTALTFGA

TTISVTSAPTWIVSTFDVVATSTATTIIDGDSGDRTISISTQNTLSQGQGASGAATTSQQ

EGAATSTASTDAGKASDPVLTASSFGTSAGAGAGSQSQPSGEASTGGAGGSGTGLGEAAT

ASSAASAAASGTAGSGTGPDVSQTAGSDVTGAASTSLGSDGVAGTATTQVPGSSGGSDAA

GASSTGAVDAGATESGAASQTQDASGAFTGTTSSEADVGSAAASATDDGSAASTSLSGAV

GPSGISGNEGTASGAAESTDLQSAGTGAATETAGAAATTSAGSGSEASSADSAVGISAQT

TGAGASGTAQESGAPASGTEGAQGTGAGSSAAEQTGASESASSQQTSGSDGSGAAQTSGS

DGAGAGQSSASGASGSQQTSGSGSEGGQETSGSDGAQQTSGSDGSGAQQTSDGAGATQTS

GSDGTGAGQTSDSGAGAGQTSASDGSGTQQTSDGAGAAQTSASGSEGGQQASGSDGAQQT

SGSDGAGQSSGSGSEGGQQTSTSDASGAQQTSDGAGAAQTSASGSEDGQQTSGSDGAQQT

SGSDGAQQTSDSGVGAGQTSASDGSGAQQTSDGAGATQTSGSGSEGDFWI

>SoG_07969.T1

MKASTILVTLFGASAMAASVPVVGRQNVLPQNQVALDEARQDSATAIERGLGRGRILKDA

ASCSEACSRCRDSAVFNAVAEVLACGTAAVAIDVLTAGIATFLEAAGFAACEAAVVANLN

FKEEDCLSKK

>SoG_08050.T1

MQVTLALNLLIAAVMAVPLDSGERTGNVHMRLSEVLTPHWLRAYGGDHYGDGKNEDDCDD

DAGNGPGGGDHDGDGDGSPGFSPCVAGIPQCCSADLVGLINIDCSPG

>SoG_08079.T1

MVSLKVVPVLALAMQVAALPSSNGVPSTNSKLLVKLACLPPSNCSPIGNCAYCCASGVKP

NSASCHTHGGSGCPSGQTNYHCDEDH

>SoG_08083.T1

MVPKWMLALGLATIASASCPFADPGHLAARAEGREGGSRSHLSQYEVDDSDAYLTSDVGG

PMEDQQSLKAGERGPTLLEDFIFRQKITHFDHERVPERAVHARGAGAFGTFTSYGNYSNI

TAASFLSGEGKQTPIFIRFSTVAGSRGSADTARDVHGFATRFYTDEGNFDIVGNNIPVFF

IQDAIQFPDLIHSVKPAQDSEIPQGATAHDSAWDFFGQQTSTLHTLFWAMAGYGIPRSYR

HMDGHGVHTFRLVTDSGESRFVKWHFKTKQGKASLVWDEAQHIAGKNADFHRQDLWNAIQ

SGNFPEWELAVQIVDEKDALAFGFDVLDPTKIIPEELAPLFPLGVLRLDDNPVNYFAETE

QIMFQPGHIVRGVDFTEDPLLQGRIFSYLDTQLNRHGGPNFEQLPINRPVVPIHNNNRDG

AGQNFIHRNIAAYTPNTLNKGNPKQANQTQGRGFFTAPGRKVAGTLIRKRSSTFDDHWSQ

PRLFYNSLTKVEQQFLINAMRFEMSHLSPRVQENALFQLNRVSHDIAQRIAKALGLQAPA

ADATYYHDNKTADISIFGTKLPTIAMMQVGVLVSTSSEASLRDAAAIKGAFKADNVTVAV

VAETLANGVDMTYSQAEAVGFDALVVTQGALPLFNPRQRSSLYPPGRPAQIAADSYNWGK

PIAFLGNADEALKNAAVAKGDGIYIVDSTDAAVKNLRDGLAVFKFVDRFALDDEGKSNST

KKA

>SoG_08089.T1

MRRLLALSWLALAISDFVLALSELDVCGNEYGVFHRLSADLSVCFERITVASILIGQDAK

RQCEVCANRASDDCVDCVTVVGPCTTPCTAPVTETVIRSGITTVLILTPAITNAAVPTEM

PSNPTTGRYSSLSSVTAVAPSISSSSASATSTNPATVPGLPSTVNTFDLVGCLASAAGFP

GFSLQASSSVMTLEQCATLCSGRAFFGVYQQDCYCGADLDTCVPVEAQKCNIPCPGNFYE

SCGGLRSLNRRQAIASDVLLAVYALSVAPSRLRTIVLHRDEQHSFTNYYVYLEPHVFKYN

YDFITTDFRYIIIQLGKRVVFGNFTIVFIVNRIVNHAVNHIVNHIVNHIVDYAVNYTISF

NIDSFSCSRYGYSMQNDKLYEVDLITSQIIRQIGSGTLQPRGFSLRGLAYNPVDNFLYGF

IMPTSSSIQVVQVASDGSYILLQEHPTSTQTAWFSADFDPSGSPTYDQVISTGHTGANLF

SLDWAYLPGSGAGASLWTMARTGGVMTLASFSLATKTFSIVQTFAGAYSWGGAYSDGNDL

LMCAYQTGDLYRISVTSPQLTLVTSGSMTSVMYGARXKAFTT

>SoG_08186.T1

MRFSTLTGTMAISAVSVLAKELAVDEERASKLYDSGLMHDKIMKAKISHWEAEEKAGLFN

SSRWPRLDYTKCVNGIAQAIPGSDAHKFRCKNVDLYDFINHATLGSPNTDYRGKSGSSSW

GWTDPDSGREFVVSGMYDGCAMIEILPEGRMLHLGYLPKFAPTADRAYWTEIRSYKHYMV

IGSELEGNGVQIFDMKKLLDIKPEDAPVLFDNSKDLTGHFNSSLPLGRSHNVVINEEAEY

GVAVGVQPRNQGCMGGLHFFSLDDPTNPVDLGCDGQDGYVHDAQCVIYRGPDKKYHGTDI

CYGYNEDTLTIYDVSDKKNSKIISRTTYEGATFTHQGWVNNLEWQEWLFMDDEYDEDELA

GPAADGYPVTYIWDIRDLENPKQTGLFKATNRGIDHNLYVVGDHIYQSNYGAGLRVYDIS

SVPEDPTGDSVCEIAFFDIYPEDDHLEGGGIIAFSGSWSSYAMFPSGFIFINTIERGGYL

VKMTKTEKCKPKSCNADNCLRAMRASHIKGRLEESQEFCGGFLDGWEANVEVVPKYAQET

CGENIISRVSSACSCLPTATP

>SoG_08189.T1

MVYSTHLLMALAASGALATPVAPRAKTLTVPVKHVSTVKDVKTLVEKGQARLRKVNGDVE

FRKESSSGTVTNEDVSYVAAVTIGGSTYSLIVDTGSSNTWCGAQNSCEPSSTGRDTGNSV

SVSYGSGSFSGEEYIDSVSFGGLTVQSQSVGAASSSSGFNGVDGILGVGPTDLTQGTVQN

TDSVPTFMDNLYNQGSISTEVLGVSFRPESGSDNDDANGELTLGGTDSSRYSGSISYFPK

LTSGSAAPYWGISVSKFSYGSRNLGSTNNAIVDTGTTLIYIPTSAYNSFLSATGGTTDSS

SGLSSFKTKPTANFGITFGSKTLTLTPSQYLVPQAQYGAFGLSNGKYYSWINDGGNSGVN

SIIGQKFLENYYSVYDTTNSRIGFATAV

>SoG_08202.T1

MASLTKLLFLATAFVSVAASCQGPNIDCAGLREHIDFASYHATCLNATYHQPGTFHLDGP

TGPVTNNISFHQVHASIAYAEGGAELVFSVWLPERDAYRGRFLAVGNGGYAGTIDRVSML

HHLNLGLGFAIAGGDAGHDAWAETNGTDAGKPGLYIPFLNHEERTRAWLHNAISIFTPLA

RAITKAAYGRDPSHAYFNGCSAGGGQGFALAQFHPDLYDGIVAGSPANHQSHMWLGILWT

FQSQQGDGALSADVLLFVMSSVLKLCDGLDGVADGVLENPLACPFKVESLLCKGGEEPAG

EDGAIKCLTQAQVTAATAVYNGARTSDTKEPIYPGFPFGAESNWIIPVLVGLANGFSVPI

LQNLIYKDLGWDPATFNYTKAEVAHIDQKGGPLVNAISADLSAFRGRGGRILSTMGWADP

AVTPLSAVEHRERLRTGLGAGESLEDFYRLFMVPGGGHCSAAHLPQTPGNWHVMEALVEW

VERGVAPKSVLATDPQDGSGRTRKLCPWPETAVLVGDDENDWESFECRSVHA

>SoG_08243.T1

MLPTTVLLVIATALGFGNTAAVPESVDSSLEKRRCFKTGMKYGQQSRAAQDRAEEACKNG

VLNGNWKKGQSASKCYNIGGNKHVKFTISLTGRNAPKCMHLGSSECFGGLSSEITSCERG

GETTYGRWYLRADPNNGPC

>SoG_08266.T1

MVLIQVSAAALALFAQFGAASPLVNRAEASSTILSVPSSTSTSTAGYDWSAGWETKYQIH

ASCNSTYRHQLVAALDETEQLAKHARDHLLRFGNNSEIVRKYFGAASTAEPIGWYDRVVG

ADKSKVLFRCDDPDQNCKTQDKWAGHWRGKNATSETVICDRSFEIRRWLSGMCGLGYTVA

GSPLNTFWATDLLHRVFHVPEISEGHVDHWAEDYEDVLKLAKEDPSKAVRDSNTLQMFAV

DVWAYDLAAPGVGCTGQMTEEQKKKKKEKHDSKPSVTSSAAPSATSAASAVSQALILIPN

VMRENIDADRDNDNKQSCHTHDDGFVHC

>SoG_08291.T1

MLQFSPLIAGLCASNWVMLALTLTRLSQPGQARGWMLAACASFAVRPLLRSFQSYPHGVI

AQANGVWGSNSYVVALAEQHDTFSLPCTSVPQLSSMAREPNLTQASSFSLYRRAPPKWYQ

QRTTPFFTSRPSPRMEKQLAEEAERQWQGQNLAPGLQRHCAKGSLMPWLSYARTSTRLKL

ALKGHDSINLPDDGACNPDCAMFLCCFPXNEIIFREAKHAQGQNLSTSKGIFEPSKDRYS

TQPPMEILAEGPPQPTGPPRARSASASSLKGKDIEGSSNSLSTIPEVSREVSGAGDTGED

GPVPGPLAAKRIARPHALNADSTVSVPVAKATFPLINYLDKQWFLIPANDLKENTKPEDT

VVEIAEAAPIEVERTDRGVWNFFRRRKAAKDAEPVIWHVLADDTRSPHASTALKHTLVED

TPVVKGQGSRFAEHILEHDRLSPSMVPTINVSHELADDPVFSLPTDPSEGKHVLSEDKSA

LVKVAASTHALDRDKRAMPSLELRTGSQDDTEASKQDKGFVATFLSGIAQPLRSASASPA

APETSDRREGSESGGEAKEDSGSESATHHVGNQVLNGQ

>SoG_08301.T1

MRLTLVFCLAFGLVASLPQDPPPIPPVDPAPAPPEQPPPQQPPAPVPAPPTDQPAPVPDP

PATPVPDTPAPTVTGGPPGPICECGYTYCSSVLQAMKEPWSIEQLSDAYCNTPNTTCPDG

QPRTDVAEALYVCLCDDPEEDLGNHLNLLCGCDECLVVGPDYRGRCAMPCYGSCNS

>SoG_08357.T1

MKISLTTAIVVQLAARASCAPGAEVREQKTTFSLQQVKNNNYHGVDAPTALVDAFAKFGK

PIPKAMLKAIEMNPELGQKFKNRLATGGKVTASVPNYPSPFYDSEYVVPVQIGTPPQTMY

LNLDTGSADLWVFSTDTYPPSANGHTLYKPKKSTTSQRLNGQSWSVLYGDGASAGGIVYT

DKVQLGATSFNKQAIQSAVQVSAAISGDTFASGIIGMGRSKANTVRPTLQKTYIDNIQSS

LYQGLFTVDLQPQRPGTYNFGYIDHSKYDGGIQWVPADPNSPYWEFTVDGYKIGRDSKQF

RPYRFQGIADTGTTLLLLPTGIVNDFYSKVNGAGFDPYTGMMVFPCSTTPPDFVIGLSKY

RGVVPGSYINYGRSNATHCFGGIQSSDGIGFAVMGDVFLKAQFTIFDVAKSKLGFANKKI

PAK

>SoG_08387.T1

MVSLQILLALAVLNLSHPASAQGDQIPLSTSPQIPDDYTCEHPASKVHLLSKSPLVIYIE

NFIPHHERLHLQSLADGHFTHSGVVGARGSSVRHVVRTSQSTSVPWDKLVNCVGSRALEI

QGFDLDQSHLEPIQLVKYAPTERYHFHTDWFTDPDRAVASLGGNRLSSIFAYVKADNVTG

GGTNFPILRVPSGEQWCEFVDCDEEYENGVTFKPIEGNAIYWENMVAPGKGDDRVLHAGL

PVVSGEKIGMNIWTREKPLPADTRG

>SoG_08408.T1

MQIHFFFFFILFFLHRESLQKSEQLEIQVKSSMPTAPAVEHSANWYDNDVLDVAPGELLA

IGYLCCFCQSSPNTDDEPAHPLYTQLILAPLNYDDSLPDDLVILYEYSAERMHQMISEPS

EEMVNCNKQLKFGGPEPQATTAPMHY

>SoG_08416.T1

MLPAIRDHLPLLLAAPGLVSLVAAESPSQQHVIAAPPAVRQQADEATVLAALDAHKDPVD

AYLAIHPEAADELAEPRFLRVAGEEATWMTEGDKMRLRRDGLFFMDITDHDEYYAQHAAD

AAVAGKPHLPELRYQGSVRRLFPKVSTHRMEKVLKHLTSYFQRSYWTTLGETSSVWIHDY

LAEIIKDAPFHTYISLEYFTHRFPQSTIIARFEPKIRNFSAPVTIIGGHQDSANYIFPLL

AAPGADDDGSGTVSILEAFHVLAESGFVPKDGPVEFHWYAAEEGGLLGSQAVARWMKESG

RTIGAMMEFDMTAFIARNATETIGLIKTEADAALTEWVQNLANEYLKIKAHVYELGAGAG

SDYMSFTNLGFPSSFASEGNPMKGGFPGEFDPYVHSTKDTMDVDDEYGYFSFDHMARFSE

LAIAFAIEQAGWDNQWR

>SoG_08429.T1

MVRTTTTLLAALLADLGSTKVLEFPITFRNSYACLDVSVGTPPQDHVLVFDTGSATTWMP

DKKCADGGCNNFSGYPWKGYDANSSSTSKDLGIYDSIVYLGGATGGEAYSDKFSKDGFSW

TQTFLAANKTSVGFLPGGGFFGLAFSSIAEEGTKTVMETLMQDCEVEKPRFGLYYGTEFN

DTHGHPGKGVLTLGDSKEDKYVEGDMTWVPGKKDKGVYELWRSNLKTFYGERKDDDGKPQ

TSGSLQYKLGDANGVFDSGAGSMYVPDGVISSVYDSIGWNYTALLHGHYIPSCSDMNSTW

SVTFTFENDEGSDFRNVTLTGDQLRDPGFANREDACNPPFSTSGNSHLFLLGQLFLRNFY

TVFDFGATQVDDYNVSIGFGNLKKEWKA

>SoG_08430.T1

MRFQSIFIASAVATVLATPVPQGRGSGADSCPAEALDPATWTKLDLDGFLKGWVQANYTA

PAAGASTIQALAATFGAPNFFWYATTAARRETFTDHVTTVFSGLDAFCNAGQPCLPVTIP

GWYVMVAIQNWNNYMNSINTAVNFASSILSLKLPGIVSDFLPDTVDDVTPLKTVIRMVTT

VLGFVPLIGPVATGRNAVNQGLGFLLGSLTPPEETDKFVQWSNVAGSLSAVVSDYQATVS

STIDKIIRADPLDPNLGIASLLSGGNFLGVTQNFTSNDLQSGVDAALTKAAIAAAITASG

TYVLHFQNAAPCRDDDVSICQQNGGSSVNVILKQRDFGEARSTAKTLTEKYGITKDEFLV

SVVNCWTSHGKTNKFDAFKESLPLDANTPCLFYLPVCDLVPGKLLPGADKFSSHCAQAVK

L

>SoG_08514.T1

MRLSSVVALAATAVSGGAATGSYVKAMAPNAQQLFTESMEWMDTFYDEKAGYLYDFSASA

ALRHETRSSVWYAFGLLARNEGSDAAEAEKIIKNTIDAQYKVPAEEWYGDYQQEPEEPYV

GSPAYPPKIYGSWDPNWRGFVGTTLVMCIEEFPHLLSKGTQDLILQSLHNATKGDEYRFG

NLDKTKDNLYPAYSNPSIMRAFVSGWTGRRLNDRNMTTGGEKYAQDIIDLFNKHDTLSEF

NSGTYTGVSLFGLILWSKYLPKDSVMSKNGPRMVEKTWDAVAQLWHPTMKNMAGPWDRSY

GYDMNRYLSLMALWFWTLTGKESSSLIAHPQTMSHMADYAWAPLFAALDKTHQKLIPKKT

LRKLSRFQGEHTFKASAYYPPFDTVSRNITSWLSEELTIGAESYNEIVIGGPSQYQGGFN

PAVVQWNTGDEISFISLYPTEMSLQTDVKPGKLSLSYPNGTASSVFTFIVGTFERKRTVG

SWADVQGLEVKVSGNVNATYSVSFGGGYGGADTPIRDFEFWNFTYTMPSGFQGVPAVELD

LKLI

>SoG_08539.T1

MSTSSRAFLLFVLLACVSCAYEYDQDIRPCEEEKCSVDIHSDVPRCWIQGGNQVIDYMSP

MDGGTHSIPNACMRSNVFGGDGYLAAYPSKGGIMLLETPSAVDLRHLGLPNTYDTARVAD

EDDDDDIAGRMAQLGAQWWPDWDTYLRHSARVDGGVFYDYHFPSRVEVAFPSAGGVWVAN

FTRDAPRYQYENKACESWLPHAPALWPLKMLYALTMDDKAETIKSLGGTFYSSAGEVPGL

ASSVDEAVSLFEPFKQRLRNMEDDDYRRRFCAHDETGDESIHPGIEAGNKQHKKPRWGTA

WLFPELR

>SoG_08594.T1

MQFKYLFTAALAATSVSAGVITERQANIGDLSNALKDIADSITDGITTIKDIGPDNVLSI

IPKLGKDFNDIANTIGQFIKEVGKIKPSDIRDIPNSDICDTYSDFFDVYNNLVDVVIGSP

DGLLARIPASVITLPVAGILQVANNAINSFVYSFIQIVPNCRPRAQEELEEANKKFTDAI

QDFAANVYRPIGGDFSGLGEGKSN

>SoG_08663.T1

MLFHALFITLRSVVPVSQWHLLGVGPGAGAAKCWLLPAHPPPTPPESNPQYLVTRTVLPS

GGEPQSKQHPNPVLVIHIPDYDEYVGMEARVRQFRSCADRLFDTSMDGSA

>SoG_08709.T1

MRFTTALSAIAVLAFTDIILAAPTATTSTPVSTPNPNECEVDAPKTSDNDGTTGGGGGLS

LTAKLQLADTVADRFKLLPNDKDFVFDFNQKQKNPGKGGELIAANRKTFPALVGTGAGMA

FGRVNACGMNTLHVHPRSAELQIVTAGKLITEMIPENGILDNEGKRRVIRTELTKGMMTP

FYQGSVHTQYNPDCEPAEFVASFAAEDFGTGQIADETFALSDNIVLATFGQSFNGEELDK

IRKAIPASIALGVEECLKKCGIQKK

>SoG_08770.T1

MKFSMILVPAVAAFAAAQPIVETREPAEADPMAMARALLTANKRDNVIYPPGSCENCVKF

CTEGRAPVVSALCLAVACGIDCIIV

>SoG_08783.T1

MLVKSLLLAAAQLVTLGSSLHIPYQVNPRQQQQSMNQPARRQPCTLGFDETCADEYICAS

FGPMDEPHCVHQVLSL

>SoG_08791.T1

MAPSLVIAQLWILILRSTPAALSFLSQPHSLLQPQHHHQAAAIARIANMATADSTAKQAR

TSVRYSTYSIAAPSVSPTVQSSDSTDGELRTIAVGLDRMENKALSSQRVTLSEEKTDNMR

KLALGAKLERALDRRMSSQDAVMRPRGKTVSAEKEAEKSG

>SoG_08807.T1

MSLATLITLLALTTPIAAQAKAKLSSKGCADASGLEKCQADASSKTSSCIAQANKDSSQK

ELLACTCQDYVNNYNCFAAHCWNRVWECEYQDYMVGYFQTCLTAAQPVPYFPIPGGVQDG

CSCNLGRTFFALNGAIKQSTECSNKANTIGDPGQSLQVMEGCSCCAISGSLSA

>SoG_08812.T1

MRFPHQSAPVWALLLPQTFASPSKPIACSNDINVDVAIIGGGSSGIHAAINLKDAGAKVV

VIEKKDQIGGHAETYINPDTHIPANVGVVIFENTAIVQSYFNRLKVPFKTITTTEFLGNS

FKSYDFALGIPIPAQTPEEQAASQQAITAAITAYAATVLPKYPWIDLGYYIPQPVPEELT

LPFGQFAQMYGFEALLPLISQFNWYAGNITMLPTLYGIKSFGPGLLQSFINSFIVAESGD

TRSLYRAAAAELGDNVLLNSDIVHVDRHDDGVTLVVKQKDKQPKKIHARKLIVAIPPMIK

NVGNFDLSDEERRLFSKFSALTYIAGVANIPGLNETLYNVGAYTPSHAPLVPGSNGYLNP

GSPGQSLLGVAFDGADHTTEEGIAVIRRELHTLGKLGAVPSNAAEEVTFPYLSNHAPFNV

RVSGRDIADGFYQRLLSLEGSRNTYWTGAAFAGHNSGVIWTWNNGTVLPLIMKDLASGV

>SoG_08818.T1

MRATLPLLLPFFASLPVASALPYHAMRQGRDTASSPQYSVVPLEPGNGDDEPQGPPGGGD

AGGSPSNGGNQNTAGSGGSAPSNGGGNSNAQAPTASGRSEIITIIETVTRSHEPVTITHT

QQPDTVTYTKVVPTTIVSVVDMVEEKTTTVVIPTPQIIVVPPVVVPTEKPAPEQSAAPQP

APQPAPPQPAPEQATTTTSSTIYSTPMVAMPSSTISSTTLVTPIIPDTSTTTLPAPTKSP

ILPAGQNQPHLDVPSSPPLPGTDTYRWQPEPPPTSVIAVPTWTTLQTITTAMQAKSSSSS

SASYDNGQWHTSYPAWNETAWRANPF

>SoG_08823.T1

MVRAFSIVAVLFAAVMGVQADGYCRCLYKDGSHCCVIPLQGCVVLYLLFHPPVSVPFANS

PRQTLFAEWLYAPSSLAEIHLSSSFQTPLFTTTMASEQKIAIVSVYDKTGLLDLAKGLVQ

QNVRILASGGTAKMIRESGFPVEDVSAITKAPEMLAGRVKTLHPAVHAGILARDLASDEK

DLADQNINKVDYVVCNLYPFKDTVAKINVSIPEAVEEIDIGGVTLIRAAAKNHKRVTILS

DPQDYAGFLSELEKGEITEQSRNRYALKAFEHTADYDSAIAGYFRKEYSSGEQHLALRYG

ANPHQKPAAAFTNDTPLPFKVLCGSPGYINLLDCLNAWPLVKELKQALGLPAAASFKHVS

PAGAAVGLPLTEDERKVYFVDDIEGIETSALAQAYARARGADRMSSFGDVIALSDIVDVP

TAKIIGKEVSDGVIAPGYEDAALEILKKKKGGKYLVLQMDPEYNPPQTETRTVYGINLQQ

HRNDFEVTPRTFSKIITPKNSAPLSESAARDLAVATITLKYTQSNSVCYALNGQVIGLGA

GQQSRIHCTRLAGDKADNWWMRFHERILSIKWKKGTKRPDKSNAIDLLASGQLPTDGPER

EAFEGVFEEVPAAFTPEERQAWMSKLDQVCVSSDAFFPFIDNVFRAARSGVKYIAAPGGS

QNDVPVFETAEKLGITFVEQNIRLFHH

>SoG_08824.T1

MVRFLSIAAILFAGFAAVEGCGWCQCLNSDGSHCCVQSAPSGDVDCKKACAEAQPQGLNQ

FEPGPKCTGGGKYVCVSAWNAHFRQKCQGYPT

>SoG_08825.T1

MVRILSLATVLLASAFVAEAAQFCQCLFQDGSHCCVYSDIEIGNINCQNACRNAHRADGA

TNPKDPLEVGTPCNANGKYEPVSGWNAQFRTSCYKQ

>SoG_08866.T1

MARYLNPAKIGLLLLLELYTEEAIPSDAILPVLGFISSHILDHYPENPSDSRWVRAEKTV

SLVVSIRDFEKLLGSYPFLMGLPGRRLWDKFLDKIWAINSLHELHNFFARLQDLIAKTKE

ERKRETGMSSPEPEEGVKLARNSPFGAFIRRSQVEFQRLRFHDSTELWKNFVLYRQPTSQ

QRKRKTPRMAFDKVLLEGEQLDEWDPASTSALASVVYGDMLTGGQSSLPVSTDDIDSLLE

FQIQQMQSSSVWQNPEGNGGLTGGLAARQFCGSKHNTLLIVRIMPTHPTGSIMPMHLTGS

IMPMHPNGQHRADILDQADFGCYTEAVDAMLETVSTARENRDMSCLNFALNWLFHFGKCH

PSMINDLETDSVLGSAKESLAFLRVKAKETGMFSLWSSVLLTEAKTGLENGESLATAFEH

ITRSSQLVVEKDLKTMFGSQMSLLTALWDRLGVAELGTTTTEVFLRCHVRHSMFEDELRL

TCRLALQKADRGRYEDALQTLESIDENSLRSRKAGEYWFKYRGIVKLLRDLRHGYLDGAK

ELLDQLLQTKDDELEPGLSFLIDTLYIDYLTRRGDLQGAFDKVESLLLNLRDNATDVDYR

IRLLLLKARLFDKCGRPRRGFSIAVQAASLAMRARLMTALWASMSALCSILVSLNEFGPA

VELLTAVIPRAMECDSYELVARLYASLADANMGLAGARDVKAARKTEYLTRALGAVGKAF

DYYSAAEDAEKQCEMMAKRAIMMKVSGDTVLAADCAAAYSSLKATAVARSLGGR

>SoG_08876.T1

MGPRRRRGNKSPCASTRDVTTGLMLSFLALSPTVAADSYDQRFHEDIVFQPGPLLPTSPH

PPKPAQHAFSLRHIYHHGTYKHPRLHRKRDIRNTETRVFLVAEDGHDEQTVPLLSAKSRP

GEIERLRDRRPSVVDPMVVESRRQGFSAFLDASAWTTDEIPVPDVTDKNTVLSMAYMAAD

AYVENEDGADWEDVGEGFDKREDFGWETDGLRGHIWADQNNSTVVIGLKGTSPAVFDGDG

TTTNDKINDNLFFSCCCAQQGQWTYHEVCDCATGTYSCNNTCVKEALNEEHRYYTAAKEL

YANVTEIYPESTIWLSGHSLGGAVSSLLALTYGLPAVTFEAVPEALAASRLGLPVPPGSD

PDAPQTRDKTGTYHFGHTADPVYIGTCNGATASCSYGGYALETACHTGRECVYDVVADKG

WRVGIGTHKIRAVIKDVILQYDKVPECKFTPECRDCAQWKMYESNSTETTTKPGESTSTR

TRTRTSTCETPGWWGCLDKTTTTTGETTPTTSTSTTSTSTCKTPGWFGCKDKITSTEEAT

TTAPTSPSPTSTCETPGRFWGCLDEVVSTTRETSGAMLVPITTAPTATATAPSPSDSTEK

GDCRRRNWLGLCKEWDMEDDEKWKNEEM

>SoG_08886.T1

MQKVFLCVQLLRISLASIWAEMPLEPMSWLPMQSRPPAYDGKCLQVASDRQPVALASSPP

WSMLGDDSMPPYGAGGTRANCDTQPKGGAAGSDSDLFNKQQPSAKLEVITPDKCTNDKHP

EINHQTRDGIEWMMELGEGQGTGKCWSLLGSVERLVSERRLYRRRRVCERLSGTPSDKKG

AWRKGCLVNETQGMPVTDPATVVPAIWADSGVPKWEQQ

>SoG_08888.T1

MHIFNLLATATLASAAALPHQPPLQARDKDVLAGHCCFALKDSSGRTVQQASTGEVRIAA

SQPDGWYCLDLSSSSSKVLRDDANSACIVDSRGQFMCVDPIPGDVAWTLGTGGELKYGGS

TTFSNCNGQVYGGSRSGCTTTTLKASGKQGSC

>SoG_08910.T1

MKLNQATLLALAAASSTYAAPPKKEPRCRFALDWTTEEIMRNPDKFAWDMLYWEGQFHQD

GIAYNAENGMTYDGRYIDYVTGEPITEKPFSASSKESMQVMLYAQAVAGNPEAARFLSPK

KPKDAAKIATDIMEKKLKTYKKFNETFPGFGGFLPWMTTSTKEITPTQDWHNRVPGLDNG

QLVWAVYGFIHALEQSKKPGDRKLADGWKEWLDYTKTTADKVFYDSDGKVCAVTAIKSEK

LSPTDPQQGYSCETKTYLNDPYEGELMTYFLQLFTGLSDEDKKSMWQYKKWQLIRDEYNQ

GGVGPVTVERGFWFSAHEPWKVLELPYYDSDIMRSPASRVYHNAERVRTCNAIVTKAPGL

YASINNFTDPATNEVGPYISPAGIPSIANNTEQYHVVATPYGSWPTMLFDRAVGLAWWHN

MVSGKKMQNKYGSTEGFKIDGTGVSSMVTWDSKILTVVSLLGGVKDIVRDAMKKDGIYDD

FLAQVNRDYSRTFPELKGEDVELCLPQFTVPDKGLEDYTSCKA

>SoG_09089.T1

MNFFSFLLFAVHLATAISVAGLNQSPSTSLLSNSIKEVRAYLRQELSAEERAAYRGITLA

GMTGIKFERLIAVDTPEELIISEWFGKQFIPADEPQSVYLVSVGRGLTAQFHPDWVARMF

QDKGPALASNKTKSEMDAEYRLSPFLSDAEEGKVEELLNGVASSYLAWVDDERSGQSGLR

ERQQGYYCKSQRQVCGHTSQCVRVKTIQRKMCYCGIYDTCTTDDLPLPPYPCIVSFPHPV

KHKPMAPNTIRPTTIGEVRAHLRQELSAEERAAYRGITLAGMTGIKFERLIGINTPEELI

ISEWYGKQFIPADEPQSVYLVSIGKGLAAQFHPDWVARMFQDKGMNRPSNKTKSEMDAEY

RNAARPSPPEEEDAELEELLDSVATSYLAWAETQFSSLKSGPSQTKGEELRLDSSASDTT

SSYELGADKDRNE

>SoG_09095.T1

MKVQASAAVALLAATSQACMQFGARITGDGRLVGAIVDNGQTTCQMYATSDPSTGAFVCS

NCQAGFSCYVRWDLELLMYTNGQTTHPVSVQRNGDYLAASAFC

>SoG_09103.T1

MKFSTTTIVLAAALGASAHPSGHAHRHAHRSLEARGDFVMNMKPVPPPAPSTTQKAAPAP

APAASSQAPAPPKANPQPPTNGAKQPSKGNGVAKPFCGGQKYNKRATLADIASVGNIGAP

GNYGCNLMLVDEDVAEQYDYLTTFTNNADKAQQCACFNKIGPVNNEINGFFNGKEALKFN

IAAGEKKYLAADVNTQGGCSCGPGTVALTNWGEFADTWLEFDFASQRNKGWSGADASCLV

AAASGMPIQGMNVCGHDTCSTIFPGGTGKNAYLGGMEAADGVGLNIPPGQVRLQVTIGYT

GN

>SoG_09116.T1

MKFETSCSFLLAVSALLGNAAAGPGAKVRACVNMEVEPAEPFTVYWNIGAAGDQCMDDQG

TSGEMKVSKQGLTCRYLGWVSLKNSWGCYAKQSWWTLAYTTSKAYSGSTQSRWQYMFSSR

TVTLHNYSPGTQLCGSEALCPTTKYEWSNNNGALYHSAVYIPTHQVDVFGEQHGGSQTRT

VRRAGNDLQQGRLVAWRQILEILD

>SoG_09198.T1

MIARTASLFASAALFARGVLSDLVINNYCDKDVEIWTAKDGECELGNGGCLVDGFLPWKL

EGGKGVTVWQQGWRGESVSVKIAKAGKEGILQFEYSLNERGLWWNLSDLDGAGPGLVGTP

FRHDDVQATPSGNGSGVGTCVTILCKADEVCLDSYQHSDDPNTRWCPIDTGDMRFDLCLP

KELFWAEGSA

>SoG_09210.T1

MNLTFLLVLASSTFSVAVAQSAGETYDMATRSLEHIKALTQNLTKGIASWDGKSLQAALS

NIHEPSTGLITYLGNATEPLQRSQAIFTLDQAFRIATPTQMLAYAVNESVATLTRRLKDF

QAAQIATIVVGDLTNLLNATQKFSDSLIAHVPSELLPIGENLSIQNTNSLKQGIACFNGT

SSLCYTAIVDPLRTYNLAIEYNAMNPDGSPIA

>SoG_09258.T1

MFLQIAILSLTVSIAVANLWLGRRRYPDVPILRLSARPGRLGRLDDAQTYITDCMKIYQA

GYDRYSKHGKHYLYHIPQEMEMIVAPAFYEEIYRAPDTHLSSALANSETMQFKWTLQEDL

MFKSFHVGAPLTKLTRSLAARLTDIVEEGRMAIDEYIGCPTEWTAHPLTEQAFRTVTRTA

NRLLLGIDLARNEEFLQLSIEYTGILFGGANTIKKYPDYLKYFMLRWKTGLSSANKLAMK

HLVPIFKARVAERQRYADQNELDIWEKKVKKDDCVQWILDAAPPDELRNFKSLTHRMLHI

NIAAVHTSSNNFMSMMQVLSLMPELQQELREEILEVFERDGGWTKQTMTHLKKLDSFMVE

SGRLCGDSAVKLKRKVVKDFRLSDGTLLPKGLVIFVNSIPFLTDPAVVESPEKFDALRMY

RKRLEPGQENQNQWVMYSETNQTFGAGKHACPGRFFAANEIKSLLVLFLMRYEFRMSSGH

TLDDIYKGVWHNEARTARTDTVLEFRALRMDIPEALKFSF

>SoG_09259.T1

MRFSSLAISACLGLLVPAGAFSNRRLERSDLSKRAEGACFGEQAPSAPPHKNFWGGLTKE

ETRDVLALLHSDAVGFNLTNATAAGSRDNKIMSVELMMPNKTDVLPYLSNSCGTPDRYAL

AAVMFGAAPEAYLQEFKEGPQDPSHQPRRGGLWRVQSQEHEGGRRCDEEALEPRE

>SoG_09270.T1

MGWLLLLFYPPPPTPTYPPPPNRTRDATLLWRDAASCKYQEAQTFAWILNTRNPPNPDVG

SARSRKKQSNEFRHCLTAPSPAILPSIDTDQPCTVRIFRPQCASTCDPLSRSPAE

>SoG_09332.T1

MMKSLALLSLFSLAWGCELEAKHEHVCPAAGTVTIDQILLVQKTPVYVSATYCQTTTLTV

GPSTIAVTNVPTFLESRFEVDNTVIASSTRLPGDNFNECVCDGSGGLNGNGNGNGSGNGN

GDGSGNGSGNGSGNGSGNGGANGANGANSGDSNGNGAGFGDGNGASNGPVILIPGGTEGG

PVTRVILQPAPGGLPDGTGPLSTTTWPYDGSTTSTITIDGPGGTPIVVVLVPPGGGGSDG

GNNPPGSNSNPPDGNGNPPDGNGNPPDGNGNPPDGNGNPPGGGTSLQSDPGSGASSTQNT

GTDGSPTGTATDPNGGQSSQPPDPGASSTGIPATSGSPTGPATGSATDPATGQQSQSSGA

SDPGGSGTSTSSNGDPISQEGSTGGPGSTTSPREDEITRTVTVTQTQSAGSSGDPSSTVT

STTSTSTTQCIVTATSVPECCMNELPDGCKAMWTTNGTALKPVISDCEKAIGKYLTEDMA

DCWYGEVTQDSKGEYIADCLMYELEDCCIKELPQECKDLSGKTGSEVVDGSGRCRDALGL

FVHGIASPCLDAKNIDQETKGQSIVDCLEVAYGFRSGPVTIFDAELCPRTAAP

>SoG_09333.T1

MKFSAFLVSGLAVIVAASPLANPVQIRAEIEGRQCAGCEPTGKGASGATSGSGSNSNSNP

SQNTNSSANAVNNNSNTNSINLTFPSNGGSGGGGSGGNGGNGSNGNNGGSNGGNNTGGGN

NNGGGSNGGNNNGGSNSINDPDGSGGNGQGAGGFPPGSCQACVAFCSVSRSPLEQALCFA

VACGFDCIVF

>SoG_09336.T1

MRVAGPSTVALVFATMAVAQLQAAPIVEQIAPGSKSCDAANKECRTAEQAAPFLAQGMTR

YGIYNVNEMAAVLSLMAFESVDFKYKTNQVPGRPGQGTANMQMAAYNLKYAKSIDRIKEK

VADINSVEGLSDEELNNIRSLVLPDEYNFASGQWFLTTQCDQSIRKQLQEDVDKGFAAYM

GCVGVQVNSERTAYLDRAKKAFGIN

>SoG_09347.T1

MFFSAKTTSLAVATLLLSSPAFAGDSLATPQSSHARRDAANDVHSPVTLEPVIPHGIDQN

DLKILDLDKDVALAWAGTPSGMSKRDNSVLSQAQFKFAYPVVPLDHAEYVTEVKCAEGKL

SARLGQTAHDYAKKQWSNAKDIVFVTSVDSCGLKNANEYFRASGVAFDDNAKTFSASGSP

ASYSDVALAVKLNWGKAGSQPLKRAADKRDFFESDAVQPRAYEKFRTSWNAFLQDDWALG

TDKSSPWDKAALLAKWSKSDGIFDEAWKYGRAIRQQAGRIGNRLLGRGLSKSDLVVRDTA

EWGMALYCVDCGYRGQAEVWGEIDADVFRFKVNRVRVGIKGDMHAGMTLGLDSFIKIERS

FNQNFRQALPGGFRIPFLVAVGSFLSFGVEARAGLEASGQALIGGDVHWEGMDMMVDVLE

SDYSYSRGPSPRGVSRAEAAGQLKLKARLAMPMRLGVGINILDGKWTADAGMEEVPALNS

GGFFKGQVSRAPDGRITGGIEGSCYGIDWSVFMTNERNAFLRATGIGEKLFPIMDPKRDA

PIGRGCIGYQRRDKA

>SoG_09367.T1

MKSSLLSHLAGGMVAALIPLSFAAPPRHNYGNPGVPAAVTTEWLSQNLHKPDLLVIDLRE

TAAYNQGHISGSLSVPFSYASVWARTSDEEILVMPPADELMNDLTAIGFRNNTSAVLVGD

VANMPVQMARVTRTAATLKFAQFPMGNVGILSGGFAAWTAAGLEVSTSPTAPNPGAYSAE

TDASFIVDRQYVHGSLNKASEGTVLLDVRPPSVYKAGHIESALSIPLALIWTQGDAKFRS

EAELQSSLNAVIGDLPVTKTSGEIIVYCYEGLLASGWFYTLTSVLGYQNVKLYDGSTSDW

VKEYPLVPS

>SoG_09374.T1

MAYDHLKLIIKALRLFLPLLGRLALEKIAAIRHNLIWHDTPDAKHVVVIGGSFAGIELVK

RLAESLPTGYKVVWIEKNSHLNYSFTFPRFSVMTGHEHEAFIPYTGVDHSGPAGILRRVQ

DSVVGITNTQVALKSGEKIDYDYLAIATGSSQPLPVQVAATDRREACLELQSVQQMINES

PKIAIVGGGAVGVELASDIKDFYPDKDVTLIHSRDRLLNNFGERLGEYALNTLQDELKMR

VLLNERPKMPDSGNMSRCASLTFADGHTEKFDLVIGCTGQRPNSSILASLLPGSISKENS

RILVQPTLQVQADAMGPSEPSTKMRIFALGDVAEHGGPRMARAGWSQSAIVQANILALIQ

GKEPPRTYKPQPFLEGAIKLTLGKTHNVVYSAEADGSDVMVPARNNRLDLGIQRAWSQFS

VSGDFSNPVNRPVQKTV

>SoG_09421.T1

MVGLASMSLFGSAMIGLTQAAGAGSKAEYASGAVHHRIMEIKMKQWEAELAAGTMNSAQY

PELGYAPCVNGFAAAIPGDKNNTFRCSNTDLYHFLPHSALGSKEGRGSSSWGWTSEDGRE

IVALGQYDGTAFAEISSEGKLVYLGRLPQFDAIGSQWREVRVVGDLLVVGSEAVRHGIQI

FDMKKVLDLDPASPKVFSQDDLESHWGLKYNSTDGSEVPFWDYLPVGRTHNVVVNHELKY

AMAVGSVGGNETIRVRDNLPCKGGLIFLDLSDPKNVHATGCASADGYVHDAECLVYRGPD

KRYTGRDICYAYNEDTMTVFDVTDKTGNNSKIISITDYPGAEYVHQGVVNNEQWQEYIFL

DDEFDERDAREGPESLMQGRPTTHILDIRDLENPVWAGSFAGKFRSIDHNQYVKGNLLYQ

SNYGNGLDVWDISTVTKDPSGDSICQAGYFDIYPEDDDNEGGGSVAFLGSWSSYAMFKSG

FIMVHTIERGTFIVKMTSTSCPKPPVCNADNCLRALRASHIPDRLQESTNFCGNFTNRLN

DDEGLLPEYASKGCPENRGREPIERVSSACACIPTNPVPDLPRTTTRPPVPTVLP

>SoG_09444.T1

MKAILALALGFQAVTAKVSYDGYKVYHIETTDYDATESALANLEYVSLNCESNHKTLEVA

IAPESQAAFEKLGLHAELTVGDLGVEIASEGELKPYKGKTKKRDTCTGLPDFSYFNSYHV

FEEHLDFLDDLQTSFIRNSETFVAGESLEGRPIKGIHLWGKDGPGVKPAIIWHGTVHARE

WIVAPVRKSLSFFAFAFAFADELLTLGGI

>SoG_09462.T1

MKITLQTLTLLGSIGLHHALGLPGRPVMDLALRDKPSGTAPEEQYQLSKDPDFHYEILRA

MSYTSSEGADLGEVLVAAQKIKPRDFESFYSAFNTLAERVHEQAMAVDHVKYPISARNAY

FRASSYFRYADFFLHGNWSDPRIYSLWEKQLKAFDSAMALLPIAGQRVVINATDGTFTIP

AIFFTSGRPGPRPTIILGNGFDGSQEEMYHVVGEAALKRGINVITYEGPGMPSVRRYQNK

GFIPDWEKVVTPVVDFALTRPEVDPKSIGLWGYSFGGYLAPRAAAYEPRLAAVFAVDGVY

DFGESAMHGIQGPLKKLYDAGDQKDFDRHVEGALANTSTPTSVRWSLEQGMWSFNIDSPY

RFLKAAQMYNLTDVAERIKVPVFVGDAQGDDFFPGQPEKLNKSIESATYHLFKTEDGAGE

HCSVGASALLQQVTLDWFEEVLQKRTLG

>SoG_09528.T1

MPRALLALLMMVALPLWAAEPLDLAWAEMIPPDAPPEAPNMTPLHDLSQMGNALSAESAP

AAKQDRPNAPVVKALAGQQIRLPGYIVPLEVSEEGRTTDVLLGPSCM

>SoG_09547.T1

MLLSLVLGSVFALTAAAAMPSSNCPPFAASMIEFSPDFKQPEPPVIKHDFTASFVQHKWN

TNLSHITQGYITNSGSRLLVQVSQGNDDGPSSSVFDYKNVTEDGLVDNTVTTYPMDAGRP

QVFWRGYVNSNFPIFREDLLVVSGAVFTGLVDRDFVGRVASVRAH

>SoG_09548.T1

MKLSFTVALALAGTGLAMPTFFGDGSPNFASDIFDTAACIVSSFIGGGNPKCRGNGSGSD

SSSSSGDKGGAGSGSKGGDGDGAAVPDKDKDSGSGGTKSDTGYSYDFTNNPDGSATYTVK

PNRNGKSCKVTLKKEERDNLNAVLGKLAKECN

>SoG_09550.T1

MQFRGIITASFVAVVAVSGVRGGCVAKNPCKNNSALSVIEQIKATSFCADFIKVPTSTVT

DKATVTQTDVRTVTAQVTATDGQTARATTLAVLQETRTESVDGPTVTVTDFQTHRDQCGD

RDGDPDQPSNIDDVPSKWPCEATPRPGDPACPLHDDLLVPNMVFLLSSLRVELKGVAETA

ISKACSCFVTGVTVTSTSTVFATAEATGTVSQGVTVSPTVATQVETKTTVETVTVTETRP

PVTNTATVPDTVTQTTQQVATATVTVTGSPKFVNGRSYNFVQSFSSRCVPYQYRQLNADV

PGIPLTYDALFQYCAATCAADSGCTQIFVALNNPPTRNGFWCLTGGGLSSQTWTASTFQC

NYPPIGSNGYWYDSA

>SoG_09620.T1

MKLSSLLSGFLLVTSAVALPSLNPRDDAVDQESVAKLTKSIRTNIIQKLDEHEAKLHKRG

RTATCNARTVVFRREYGSLSKAERLDYVNAVKCLQKLPPRTPSSVAPGAKSRVRPYGKFD

DFVVTHIQQTLTIHYTGIFPPWHRWFVYQYEKALREECGYKGYQPYWDWAKWATAPQDSP

IFNGDPYSLGGNGQYVPHEGPVIVPPPGVGGGNISLPAGVGGGFVKTGPFANMIVNLGPV

GGVEGTKPGVDGGLGYNPRGLKRDVGPAMNMRYANYSTVLNLLLKPDLEQYRLLSEGVPY

TPEIGPHGGIHYTIGGDPGGDLFTSPGDPAFFVHHGMMDRMWTFWQSLDRKHRLCAVSDG

DYGHITWANQPPSRKARLSDVIDMGYAGDSTTIGEVMDTLSGPFCYFYI

>SoG_09737.T1

MKFTAVLALAMASFAVAAPADFSVEKRTGGGSHPPSGGSSPCSDNRVQVNVQETQGVLGL

VSIVGQILNNNGGGSFCCDAGAPQVHTNFGGGGGFKLTKE

>SoG_09779.T1

MRLNIFILVSSLAQLTCGEQQAEDAPVEVDVAIIGGGASGAYAAVRLREDYGKKIVVIEK

AGKLGGHVHSYFPPDGGEPVTYGVQAYINRPRTADFFKRFNVSLVDPKLSDGINLLLLTK

DVDFQTGKSVDVDYGPLDIVGVPADLLGYSALASKYQPWFENGYFKQGDVPEDLSNPFGD

FLSKYDLGGVWGFIRNILWVSEPLKTPTWHVMAVTGQPQIAAFGLGLAGPSFKYPGTHSS

ETLYNRVLDLLADDVLLMSTVVASKRSADGVSLNVHTPSDNIGAWDLDDNESALFGKFIW

ETLYVGVINGTGLPKDVTGIRNSVPNKEGYFVPHGNFVHSYVRSGDRDLWASRVIGDANL

TFDEARAMLLPPLHTMKDSGLYDISQPSVVAFASHGFTSSSLPPEELKNGFYEKLYKLQG

QRSTFWTGLTFAPDYTPILWDFNEKLLSQIIEGL

>SoG_09825.T1

MKFGSVAIIASSMLGLALGKSTPTTGTAVAGPVTPTPGVFPSSLDDFAKQLDSLKLLDPL

TTLTKRISDRAVHDPRMAKRQVPGLSSIPGGSSVPNPFANLDVNNIASGMDKYMQAAKPL

LDLFGLGGIAQQYEQGKKSLQDMLNKWKDEFDQKASPSSSATPSGTTDSASTPTA

>SoG_09873.T1

MRAPKPFGALVAGSLFALTRAQANTYGDPNGNGFPNQNGNPDGWQPPSGNGAPLPSGNPG

DWQSPGGSGNAPSQGSGTWSDSSGISPSANSPGNWPSGNGAPADSIPGTSEPGDGNGIPP

VAGNSAGSGSPGDWQNPDGSNFPSSTGVSGNWPQNPNCVMQGTTTIFVTILPSSPAQSYP

SDTTSPGSLPPNGSSADGTSGDGSSPDGPYPAGTTPDGSSPSAAPPYDSPDGINPDGSVP

EPNAPGGSDTSPSNPESNGPTESGTNPPADAGQVLPGSVKPFTTLTIDIWGTGNGPATLA

SDSSPQATSEDSGVVTDAPAPMPSAPSDSGPQGPEGDPGTAPPSSPDGSTGTIPEQPGDS

MPVPMPAGSSVPAREDPNAQSDAGSPSNPNDSSYGQPDQPPIFVTDGQPGPSGVSFNLPP

LPQTTSLSNFGAIPSGSTGSPADGNQNGDAPYSVTIIGPDGLPTIVSHPWGHDPQAGDEP

TPGDAYTSDTDAQTVQDPVPAGGTMVPSVPTTANGPGGAGNTNADDSGAATCVTIIGADG

KPTVVDYTVGVPSTGANDGPATKTALTDGSALAQSMTIIGSDGKPTVVIAPWGNPNSAGE

TEASPSMTIIGADGKPTVVHSPWEATSPPITSIPFTTSAPVRGTGENGNEGGDGGVATSD

AQLPDGVTTCVTVLGSDGKPTVVDWPWHFPDAPGGLSSIATAVASSGGLGNDGSGNGDIG

VVTEGSALPQTAPMTHASFTILGSDGLPTIAETTWVPSPTGSGSGPGQDPVFSQASLSGL

PSGVSVQLPNPSNLATGGVSGPGDGIATCFTVIGADGKPTIVETTLPVPDATGGPVIGPG

ATLLPPSSSGILPGPLPPFPDPASQIMSNGGVIGNPNHKPYTTCTSYTVLGPDGLPTVID

TTWVVPPSFETGSALATDSSGLLSAPAGQITVGPVAPVSNGAGGITTCSTYTMIGLDGLP

SVVETTFVVPAQNSGALPTDVHLTSGIQAAPWSAASGGPAPPSAGGFVPSEGAGPSDDGL

LTTCITVMTTGTDGALTPVEQTIILPPSSAIDLPPGLSSALPLTTAGLPGASPSGPVLGN

GGTGPGDDSGSNISGYNGDPSNGDAGTVLPPSGPILSGASVDPLTAGEPTLTVTGTRTLT

KIVIADPSGGPMVSGPSGLPLSDYVVGDGAGDPSPVTAQPPGGGPGAQPSEATLFPAGSV

AYGNANPAGATVTSTWSNKVPDGTTTHLLKFPLTTLATLPVKRALRRQFSSPIWGNSTAT

ASSPSHSHTESRPSPTMCASGGNIGNRTVNFDSEKAGPLFNPSNDLWFSEGFLIAPPSSQ

EKQSYFASSGGQLIEFVPAALTPNLGEEGSDVAEIGVGPNAPKGCYRFDLFGASLGCAAE

GTEEWCEFEISAYRWNPESAREEPIDWSETKRVPACANFPAGACALTPVEFDGYTDITGI

LITLRVGLDLRAWWGDDFRYGWTDNSCEAATCRSSAAAHRVKRETVETALRRGVWHWTPE

GVERLDDDFIWDAVQ

>SoG_09877.T1

MRLISTLLAAWAVGHAVATAEASSDLRIITYNIRQATWWPGWNELYWRDRRPLMTNQLRQ

ESNENTLICLQEAFYYVIQDINSDLGNNWSWIGRGRDDGYKKGEYAPIFYRNDIWDVEAS

HVYWLSETPDKPSRSWDATQNRVVTVAELKNKASGDKLTFMCTHFDWDGKEAQAKSADII

VDLVDGYSDRPVFFAGDLNLEPNEKPYGVLTSSLTDFRTFSKGRSSGVMPNNSEKPLGND

ENIMTYTGFTPEEENLLIDYVFVKDPATVQASQYSVRSNVKDGDYISDHRPVIVDVQLA

>SoG_09881.T1

MKLTFALLPLLAALVSAAPVDDELFRRDAANCGGQYYSANAVTAASNAACDYYTNGETAG

SSNYPHQYNNYEGFSFNGVSGPYQEFPIMASGNIYNGGRPGPDRVIINTNCEQAGVITHT

GAQGNNFVACSGTD

>SoG_09914.T1

MKFSASVLIASIASGAFALIPSKDTRDFYYLEGRDLAAFQTILSNVNTDILNVDSAAQAY

TGGSGSNIISLSDTLINTLNNGVTTANAQPMLSLQDSLALQGPVQTVTNSTTKLVDDLTA

KRPLIISNGLCSTTRGRIDSINTSANNLINAIVAKVPSAAQGIAEMLVANLRTQLQRAKT

NFNTTNCP

>SoG_09932.T1

MAPTLSILSAALGLLTTAALAKEKPVDEFKAARLYDTGIIHNEIMEAKMSQWKMERETGV

RNSDLYPELGYAECINGWVEAIAGDRNNTFRCSNMDLYHFISHGAMGSTGAGSSSWGWVS

DDGREFVAIGQEDGTTFAEITSEGKLLVLARLPQVSSPSDWREIRSYKNYMIIGSEAVGH

GIQIFDMRKLLDIDVTKTGVVIYDALDDLTGYTNDLPVGRSHNVVVNEEKEYIVAVGSQP

RTDECLSGLIFFDVSDPSDPVRLGCAAGDGYVHDAQCIVYRGPDKRYDGRDICYGYNEDS

LTIYDVTDKANVTNIISRTSYFGGWVDDPMNQQFLFMNDERDERFQAGPSFDSFPVVYVW

DIRDLENPKQTGTFKYPTRSIDHNLYIKDGIMFQSNYGNGYSLLDVSGVGPNDVEGKTIC

QAAFFDCYPEDDNLPGGGVVAFVGSWSSYGWFPSGYHFINTIERGAFIVKPTSLECPPPP

KCNADNCLRAMRASHIDGRLEESQEFCTGYTANLVTEVQALPAYATSACQGDAISRVSSA

CDCLPTIENPQTPCIPSRGIGCEGVSGPW

>SoG_09942.T1

MKNLPLLAASAALTASSSAGALPEGAILVPMIRDGNQTAYFAELDVGTPPQKTWLKVDTG

SPTYSFIDPRNQVCARPGEPCSTYGTFDNRTSSTCAYAGPGFANALSNYGNGDYLNDTVA

IGGVSTEHMYFGYVNRFGFPERAIDPIYSILGLSLNCGFAGPQCTWEGPYLLVELKNASK

IDRMASSIYLGPDERDSAHSEMILGGYYDEAKVDGDLFTLEMVDPFSFAANQQTNSVNVT

AMEVIVGGNQTKAGTYGDADVGVPVLMDTGVASWPVPEDIASAIFAGLGGLLNGAFDPRS

QWQDVDCKYRDPAIAKGHVTVRFGSAGQVDIPLHNLVTRFSDDHCATFVSSRGDEPFLFG

DPFLRGAYMIFDQENWTLTLSKAKYTQEQKIVPIPEGGFKAKRK

>SoG_09945.T1

MKFTIALPALLAVTASAISVPNEKRNEALPEIDLTNIPNKFYSLAAAPEGQAHEARSLTE

RQSCPAGYPFLCNGICCQYNICCSKQCCLPSTQFCGADGLCYVWN

>SoG_09949.T1

MAATLTYLLLIATTLTSLLLSRSPLLMSSSPPTKVWADSPMRLVTTPQFESKKRHKTDLF

TTGATHMALLHNSIIRSFNSIYNQADHVSEADKSDFVNYSLTWHRFVVSHHDDEEANLFP

KVEEVLDDKNIWEETHKEHGAETFLGGLEEFRTYLTNLKSPTDFSGPDLVRIMDSFREPF

EHHFHHEIAIIAALSAHPNVPAPSTPAHESASAIFKTWGKKTVSKAGMADVLPFFLMNLD

RTAEDGIWANWPPMPGPIRWMMVNVVGGWYGSWWKFASCDSTGQPQELYALKGVEEVKA

>SoG_09967.T1

MLRHIITSLSVFTATASASYAFYVGKNLTKDGSVIVGGTGEEVSSHWLQLFPAKDHPPNE

TISVGVTEDAELPGEIIQIPQVNHTFRYLSMEYSDFEGFPAPLTNGGLNEKGVAVRDVWS

SNRDDLVGMTPNPQRGVQYSDLARLVMERASTAREGVELIGRLVEKYSEATYGGNSHLIA

DQDEGWVVLEMAGGKGLWAAERLGENDVRVLYPGYIEDFPVNFTGNPDYMGSPNIVSFAV

EQGWWDPDGQKPFNIFEVYGYTGEGRSARDGGFKYMSQKELEDETRAMAPVTEEHMMERV

RDPRIADDQAGYGQVVSLRDGVLPDLLRLWVAPTGSLAAPFVPWWLGVQKVIPEFGIHRY

LTDGASSTFLNTDYQLQEASVFAGRVFKRVLYYMCSAPEALHPVVTKMLEGFERESAEDI

TWVETAAEALLAKGEGAAARSLLTYYSNTRAEKALALGQTAAAALDGYVKLSGSWKDHKG

SEINSPSGKETVNCLVGHNPDKPKLRKQPRTEL

>SoG_09972.T1

MKFTVATTALMATSVMAAPKPDPWCYRPGQPCWKRDALPEPVAAPEAKAEAEPWCYRPGQ

PCWKAKREAEAEADPWCYRPGQPCWKRAAAADAFAEAIATSGGIKARTPEADYSNAPGGA

AFVAKRQINELAALIALAEADPAGFYSGLGLQNEFGPDTEPEHTEPENTETAESTDDTKK

EKRDASPWCYRPGQPCWKRDALPEPVAAPEAEAEAEPEAWCDRPGQPCWKVKRAEEHDKR

WCYRPGQPCWKAKRAAEAVLEAVAAPDAEAFETKPFDPAYFAKREAEPWCYRPGQPCWKR

DAKPEAEADPEPWCMRPGASCWAAKRDIQAMATVARSIIAAAE

>SoG_10038.T1

MQLSIFSVLSSMLALGSAVTVSYDTGYDDAARSLTAVACSDGQNGLIQRGWHTQGQIPRF

PNIGGAQAIAGWNSPECGGCWRLDYNGRSINILAIDHAGSGFNIAKRAMDELTGGQAEQL

GRIEATATKVAGGNCGV

>SoG_10046.T1

MVSLPFLSLLLLSLSSRILAQESDGEDGYFGYKLERRGDEESAGYETANTEDPIGNLNPV

PDVYLNASVSVGEINIEVDNLTAKVNLDAKVLNLLHFTAGVSASVDKVRLNIQNVSAKVE

LEARLENVVGMIGDVLDVIDANPIIATLGQGLGDIVGNVTDELGGGNGNGSGGGGGGGED

DDDDDDESGAAAALARRNLQFKLDNNILYSVNDFSGSTHRNRILAQNGSLVDSFLDNNGD

VSRERVVGFYSRDMTFTGHNRTIYDPLESPLGGVGSGGGGGGEEQGEGREEPGRVIKEFE

LQYHYAPFPGLDAYSHIFVDTEGNVVRTQVIAEAEAGGTATIAKAKDLR

>SoG_10047.T1

MRASVLTLLYTGLALVAAQTSGSEFPTSTEPSTVQSEAPTPTESDSGGGGGGGGDGGGGG

GGNGGSPPSSSSTPPADVLLRIPELSVDKIELNVDNLQAEINLAAQVASLVELNAGVQLG

VEKVNITIADVRAELDLVIRLGHLAKIVNRTLSSLDLNPLLINVLDTVTDVVGDVIGAVD

GLLGSIVQGDSTINFLIDNLGNIVQEVAGGAAGAVSTIVGNFRNNMTYTGSQKQLSGGLI

ERTYEYSELGALVNVVTNAADQVVRAVVVKGGSGGGGGGEGGSSMETQSGTTEPAASSTA

F

>SoG_10115.T1

MKAAFATLLLIATATAQPVNVRDSQVSASTDLEARAGSQLLNLQVGGKKDPNEKPALIDI

SALTEFLGFSRN

>SoG_10157.T1

MLFSKSSVGLLAAAWTAFAAPSSNIVKKDVLVIGGGASGAHAAVRLRDDYGLSVALIEKQ

DILGGHVNSYTDPETGEVYNYGVQTFVETGNATAFVNRFGIETTSPPRVPLTTRYIDFKT

AQRLNYTGPTSAEQRAGLTKYLELCEKYESMTYPGYWNFPDPADIPEDLLLPFGEFAQKH

GIEAAVPIIWRVGAMGVGDATKVMTLYVIQTFPAALTRNFLGLATSWIPASGRSQDLYDA

IDEHLGEDVYRSSTVINAHRKKCSVHATVENHKTGEKTEIVARALVIAIEPTANNMKPFN

LDEEEAAVFEKFQYTNIWAGILANEALPVGGSLTNMAPGVQQPKPRNWLAYPDLPFSVRF

DYMGGEKLFRIMIQGDGGMTDAGARQLIQSDFERIIDAGILPEPKSREIEYVAWEPHGPM

HARASREEIEQGFIQKQYALLGRHSTWYTGGAFSMNFQTSLWEYNDVMLPKLVKSLKGRR

>SoG_10277.T1

MVVLANVVFWIFGFLLGKAACGTCIGTEEHYYSPVLNFTKLPIRTQQIEAQLFPHDSDPI

SFAAAPPSEAVDEAWAQISRNMIFGLTGSDVLRLGKDPNITVKFNPDWNITSDEDLYLGV

LDVFHQIHCLNMLRQNLIINYDYYWGKAYGFRPEAFRERHLSHCTSILLQNVLCHADTEV

VTHVWREGNPVPWPDFGVKKQCRDFNALLEFRDKFDLDESWEKFRLYTKRPADAAVLPQE

EGVQELKASADEFRNGDYYKDSRVKGCNA

>SoG_10307.T1

MAKLVWLITGCSSGLGQALAQEALSRGDEVIATARKLESLKLLEAKGATPLQLDVTSDQE

TLNKIMTDACAIHGRIDVLVNNAGYVLAGALEETPVEKMREQFETNVFGPLKVTTALLPH

FRERRQGTNVFISSLVGLVGHALTGPYTSSKHALEGMVESLRHETKSFGIQTMLIEPGRF

RTLLLEESNRRTNTSRIPDYEDAVKNHYAALDGESGRQPGDVQKGVSVIVDVVRREGVAE

GKTIPFRLPLGTDCYEDVKQKLDGVLRDMEEWKGVILSTDY

>SoG_10323.T1

MVIGLLAIAAIPTVTGVGQAISAQKKQNAASREQEKCYLAGMLPTSNGFEDSGSCVLIGG

QVGVQRLLSNEACYKVHLHTQQLFVDLPEYPVPGHKFCGYYFKYPSEEGHRGLVSTISED

PPMLNWIFVDKDTHALRYGGRKDTVGHVIGPWGWSDDADGRFLTLQGSHASFVLRRAEIG

GGRVLWGVYWDPDQEMLDALEPDQCRPLRLHRKPVLGMESKYVRD

>SoG_10330.T1

MLTFQLMSLTIAYSTIAAGQGSKCPEIPDTGIGIARGTSEPNFEAGGGKIGAIVGDSIQK

CDADGECAFFHLNYIRPEFIDPAVRFIADAFAA

>SoG_10371.T1

MHSLWILSLSFSLAGAICHHGTSLYKRHGDIKKPTFGYTNAGGPLEWHGLSENNTICAEG

QNQSPISITSESSTPVNGSTLQFNITGFPQGATLLNLGTTLEVEANGTMSLDNTAYRLRQ

FHFHTPSEHRFEDEFYPMEIHFVFQAADESLAVVGTFVEIAGARETPSAFISSVFSNIHT

AREFGSAVPTAALDFTALEEHISNSTVWQYDGSLTTPTCNEGVAWNVVQRPLFVSPLVFR

HIKSVMKFNSRYTQNVPGGVNLLSDACAGIIKEEIKQLIQ

>SoG_10389.T1

MRPPQGRSIAAFAAILTLWNFPSQSRADMAPFLHMESFEKVVDESGGFPNQTYRSSGIRG

PVFQVNSQDLTRADDSRHIFIGSVYGEGKAGPMILDSRDLSLVYTDQQYANTYHSDVQII

NGQPYYMFWEGQRSRGHANGCCLFFDEKYNLTYTVTAKNRPGVLADMHDLRVTHDGHVIF

TTYFNIPFDTSAVGGEADSMIMDCGFQEVDIVSNEVLFDWAASDHFNITSSKAPYSAGYG

VGPDSGYDFAHINSVEKTREGDYLISMRHLSVIALLSGEDGHPIWILGGKLNQFVDLSAG

RATDFSWQHDARFHLGNTSQITMFDNHGEQTQPCPPSQCKTRGLHIEIDSTAMTARLLKE

YYHPEGLNSGAMGGMTTLENGNVMLAWGYNPSYTEFAPDGSVVLSFDRGRRRHLPDMFAY

RVTKGNWVGRPTWPPSIDVDRPTESSENATLYLSWNGATEVATWVVRVSDSSHDRFAVFK

DVASSDRRGFETVVALGTSTHVPRRFIVAVALDARHQALGATAVIDMSDGSVARYGKVFP

VAPSLKLKKHAWLERAPILMLAIITFCLASFKWRRRAVVSDVEKGWHKPLYRIE

>SoG_10392.T1

MWTLPLLSLASLAIASPIENYSRALQGRAVGVTEQNLNDFQHYVQHAAAAYCNVKPSPGA

RITCGNKICPDLEGNAVTVVDGFTGLLTGIAGYVATDNARKEIVLSVRGSNNVRNFITDV

IFTFTPCPFVDDCKVHKGFNAAWAEISTAAQNAINKALKANPDYRVVTAGHSLGGAVATL

ASAYLGQKGIKADVYSYGSPRVGNDKFADFISNSGAVEYRVTHTDDPVPRLPPIIFGYRH

TTPEYWLSTDATSNEYPLTDIRVCEGTANIKCNGGTFGLNVPSHLQYFIAIASCSSLSSS

ATSRSLDATTSATGKTSKLDISDEDLEQRLNIWSQLDQEYVNHI

>SoG_10397.T1

MLFLCIVPFIAVVAFACQRDFNLPKRHTHRQPILKRNVNWPPVLESGEETIIVNSFDNVT

TDQWSYYYGHQNKLAGLGREAAEWTRDRWSENGLDARLEEYHVYLSYPVSQSIKITFADG

RTEDINIQEDVIPEDDVTGREDSLPTFHGYSASGHVTGQYVYVG

>SoG_10410.T1

MRVSVLITAILGVAAAEGVAMPSVPSPKDDHASLAEAPCTPGDFSCVYMAKDWAGPDQLW

TCNTARVWVLSAVCGGRNCCEHTFSGAKCVC
