# Supplementary material for: De novo genome assembly and annotation of rice sheath rot fungus Sarocladium oryzae reveals genes involved in Helvolic acid and Cerulenin biosynthesis pathways
Source: BMC Genomics. 2016 Mar 31;17:271. doi: 10.1186/s12864-016-2599-0 (PMC4815069; doi:10.1186/s12864-016-2599-0)
Supplement: Additional file 7: — Protein sequences of CAZYmes identified in S. oryzae genome. (DOCX 988 kb) [file 12864_2016_2599_MOESM7_ESM.docx]

**Additional file 7:**

Protein sequences of CAZYmes identified in *S. oryzae* genome

>SoG_00010.T1

MSPSRSLSRSSPEQWWNPYRRIAWSKRNQTWDAADLESNQQTSRGRDPKRSHTTYGNEST

REDVTLHDHGVLPPTGRPTDLSTADTMSAEPAEMNGGAGPELRQRKVDEKTNGSAAAPGD

NREDTSSKTSTPPRTGNGKRREKTEKQGLIRHVQPKTPFTVGNQIQRTILNSWINLLLIA

APVGIALNYVSSINRVVVFVVNFIAIIPLAAMLSFATEEIALRTGETLGGLLNATFGNAV

ELIVAIIALIDNKIRIVQTSLIGSILSNLLLVMGCCFFFGGLRREEQFFNETVAQTSASL

LALAVASVIVPTVYDSAIDTPTSDVAKLSRGTSVILLLVYAAYLLFQLKTHSAVFSEESQ

KVPAKPWRRNSLGPGAISSGLAMPGGLMGPAMPNQDEHERLSKLLVNPPGNGDDEDDEEE

PQLHFFVAIATLAISTAIIALCAEYMVDSIDAVTKQGGLSEEFVGLILLPIVGNAAEHAT

AVTVAIKDKMDLAIGVAVGSSMQVALFLIPLLVVIGWGMGKDEMNLSFDMFQIAVLFVAV

LLVNYLIGDGKSHWLEGFLLICLYSIIAVCAFSQVCKT

>SoG_00020.T1

MSSRCYIVPPHLLRAIADSTANPEAIRQAAQASIAARDRVTSVRQERLAALTRPRGYSRS

ASVNFTPHHIVPEALLRHVAESDQVDEATRTRAQRDLGHLEQVLERVRTSQQGLSPQQQT

LFAAEEGKKDTPKDKPYRAVYTMNNSNNEADLPGKMVRAEGEPKVKDKAVNEAFENVGTV

LDFYKDHFKWRSIDNENADVISTVHFGKAYENAFWDPEKLQMVFGDGDEFLNNFTGCIDV

IGHELTHAITEHTSPLDYYGQPGALNEHISDVFGIMIKQKVQNEDAEHADWLIGEDCILP

GVKGTALRSMKAPGTAYNDPRFGKDPQVGNMSQFRTTFEDNGGVHIFSGIPNRAFYLAAK

AFGGFSWEKAGQIWWKAMTSGRVPPKCTFKQFSDVTIDIAEEEFSKEAAQKVRKAWAEVG

ISRGSG

>SoG_00021.T1

MFNKIFSTAAVALAASSIVSAQTFTECNPMKKDCKPDPAFGNEKVDCDLTKGPCKAFHEL

DGTKLEYGDQGAVFKIEKESNAPTIATKNYMFFGRLEVVLNPAPGKGVVTSVVLQSDVLD

EIDWEWVGGDVNQVQTNYFSKGDTSTYDRGAYHPVQGAVGTPHTYTIDWTAEKIDWIVNG

AVIRTLKASDAKGGKAFPQTPMEVKLGTWVAGRKDAPQGTVEWAGGYTDFKDAPFNAYYK

SVKIVDYAGGSGPASGPVRQYVYSDKSGDWQSIKVEKGDGPADGENDKPTTTAKSAQPTG

KTIEPEVKSTSERLYTKPKTTIEPSTPVPTTLVTPSKTASDEAQQTTAGSGQSQTSGTTS

ASARPTSTGGAAGSVQVALGSVVFAGVFALAQLL

>SoG_00022.T1

MSPKFDWDNFMASHFLDGKYGRRFGQAKFRGSLSRRKGLEGRRPVACGRNKGDRMLLGET

SPQPNEVIRGFSGHLHHNGGVGLVFRTGEVARRNDLVNWDPSLPWHKARDMASAEYKFEG

WIGHDKDSVHGNMVWGEFQPKPWEENDVDIEITHSGVCGTDIHCLRNGWGNVGYPICVGH

EIVGKIVRVGSKVEGGLKIGDRVGVGAQSDACLSRFGPCADCSDGEEQYCSKFVATYAAQ

HFNGAKTMGGYGKYHRCPSHFAIKIPDGVRSQDAAPMLCGGVTLYSPLRYWGCGPGKAVG

IIGVGGLGHFGVLFAKAMGADCVVGISRRENKRAEVMQMGADDYIATSDSDDWAARYDGR

FDLLISTVSSVQVPLGDYLKLLKKDGTLVQVGNPDDGAMTFHPMPLISRRIRFTGSTIGS

PEEIRSMLALAAEREMRFWVEMRPMAEANEVITDMEKGRARYRYVLVNTEV

>SoG_00029.T1

MKLHRPPFRALSRRNHSPSAALTTRTNTPRQLPIFATQRRRSPLQRATSCLAIRRYASIS

AADLQFGQPVYETHPHILRAGELTPGITAQEYADRRAALAHSLTDGSVAILHAAPLVYKS

GAVFHHYRQESNFLYLTGWNEPEAVAVIRKTGPNLGDYVFTMFVKPKNPREEQWYGYRNG

VQAAEDIFNADEAYSIDTVESMMPRILESARLVYTDTSKGTPARGLIGFLSGNTTRPNKT

PLFPIMNKLRVVKSEAEVTNMRMAGFISGRAITEAMRRSWDREKDLHAFLDYEFTRNGCD

GPAYIPVIAGGERANCIHYTVNNQSLIKDELVLIDAGGEYGTYITDISRTWPLSGHFTAA

QKDLYNAVLAVQRTSVSLCRASSNMSLEEIHNVTVRGLVDQLKSIGFDSISMSNIGELFP

HHVGHYIGLDVHDCPGFSRREKLVKGHCVTIEPGIYVPDDAKWPAHFRGMGVRIEDSVCV

DQDSPYILSSEAVKEVDDIEALR

>SoG_00037.T1

MNTYWSSDDSSASPSPHSPVSRASSPITTPGSGSDQGDAIFPNGSAMAHGATFTLVIGGL

GFIGSHTSLELLKAGHNVIIVDDLSNSFEDVFSKIQKLAAKHCAEAKLAMPLLHFHKLDY

RSQSMRFLLESYSTLVSMPSSNGSPRLTRQSQITGVIHFAAFKSVPDSIAKPIPYYRNNV

CGLVDLMDLLGRNNIRNFVFSSSATVYGNKADLGRPLREEDLVHHVEEFVDENGERSTAE

PISIGLQSPYARSKHFCEAILADIAVSDPSWRIVALRYFNPIGCDPSGLLGEDPKGVPTN

LFPVITQVLTGSRPELDVFGSDWETRDGTAIRDFVHVSDVAVGHVKALAAPAAVPFRTFN

LGSGTGTTVTEAVQSLERAAEKPIPVRHTDRRAGDVGFCVASNDRAYKELGWKAERSIPQ

CARDLWHYVTKTRSVA

>SoG_00045.T1

MIAVPLLLICGLSGLAALGSASSGSGIARQESKEMILDDAYFYGESPPVYPSPNMTGVGD

WAIGFNKARDAVANLTLEEKVSPAIEAICCSGMIPAIESIGFPGLCLSDAGQGVRGTDFV

SSFPSGIHVAASWNKNLTYHRGNAMGGEFRSKGVNVLLGPVVGPMFRVARGGRNWEGFSP

DPYLSGTLVALTVAGVQDRGVMTSTKHYIGNEQETNRNPSTADNVTIEAVSSNIDDRTMH

EVYLWPFQDAVKAGTANIMCSYQRVNNSYGCSNSKTLNGLLKTELGFQGFVVSDWDAQHA

GVATALAGLDMAMPNDRGFWGDKLVEAVKNGSVPGQRIDDMAIRILAPWFLFGQDKGFPK

PGIGMPQTMTDPHEIVDARDPAAKPIILSGAVEGHVLVKNTKDTLPLRKPRMLSLFGYSA

KSPDLFAPADGLLFTSWIIGAEPASPAAIFTGSSEGDYEAIAINGTMLNGGGSGATTPAN

FLSPFEAIKTRTFQDNTALFYDFRSAKPAVVPVSDACIVFGNVWAAEGYDRPSLRDDYTD

SLILSVADQCNKTIVVFHNAGVRLVDKFIDHPNVTAVIFAHLPGQDTGPALVSLLYGESN

AWGKLPYTVPRNESDYGSAGLRDPSRPEGKFGRFPQSNFEEGVYVDYLHFDRANITTRYE

FGFGLSYTTFEFSSMSAELVDGAKTAELPTGAVISGGQADLWDQLVRVSVLVRNTGNVAG

AEVAQLYVGLPSGEGKADAPRKQLRGFEKVNLEAGQSEELAFTLRRRDLSVWDVTAQRWR

LQPGEYTFWVGSSSRSLPLNGTVQIGA

>SoG_00056.T1

MRSSMPLAGAILACISPALCYPGMAAQFDTLKKMDARQASTEMIGDLKTLSDSQLTPTGK

AIKSILAGDGAPEDLTSTYTLIPSLGTSACNEDPCCVWKHISNEMRLSMVGSAGRCTNTA

RAAVRLGFHDAGTWSRNTNRGGADGSIILAGECESRTGDNAGLEEICDQMRVWHNKYKQY

GISMADLIQWGANVGTVLCPLGPRVRSFVGRKDSSTPAPTGLLPSVNDSVDKLISLFSDK

TFTPGQLVALVGAHSTSQQRFVDPSRAGDPQDSSPGVWDVLFYGETTNPNSPKRVFKFKS

DVALSKDSRTSGAWQAFTGASGQAPWNGAYARAYVRLSLLGVNNINQLTECTKALPPFLV

GFLNPDDAILDSFLNGPYHPENAQRLLDGDLLWS

>SoG_00063.T1

MASPPNTGSPAAMSPPYPSPAQIPNKKRSSTLEVNGPPGKRRKASNVSNTTRLRQTSFPP

ETVGSPFTARSPSVDATSHVSGSAVSGTAPGKKKRGRKAKNAKGGAGDESNEQTPSLVGG

RAPTTVSGHGGDKDLEDDEDDDKAEMALEDVTARTQEQKQEEVRLRAMLVEAFDSEQYDR

YELWRAAKLSESVVKRVVNATVSQSVPGMVSTAVRAVAKLFAGEIIEQARIVQAEWIEAG

EQQSEVPPPPPPPKKDADEEGENPDQQKMRRGPLRPDHLREAWKRYRFSGESCGVGLSQA

DIMHCVRISTLILAAVGWAAVSALPAGEGLARIVSRQEVKDVYDYVIIGGGTSGLTVGDR

LTENGKYSVLVIEYGIFHEQTGMNPTRMFNITSQPNPELNGRSFSVGIGCCVGGSSCVNG

QVMLRGTKEEYDAWAELGGGSDAGWDWGGLLPFFKKGLTLSPPAPDVAEEYDVRYNEEFW

GNETPIYGSFGDRAPAPLTKVLHSAMAAMPGMTLAQDSGAGENGLYWYSLSQDPVTYQRS

YARTGHWDNVHQQRDNYEMVISSKVNKITLDDDMNATGVQFVSLRDPDSPAVMVKARREV

VLAAGAIHSPQILMLSGIGPAPLLKQAGIDVKVDLPGVGSNFQDHSYIPSISYRWGTPPE

TGGGGGGGGGFGRWPNLVAMIGLPVLAPDAYEDIASRYEEQDPTSHLPLSYTKEQVEGYR

QQQAVFSRLMRSPNTTFNEMMMMGPGGSVQNLHPVSRGTVLIDPTNPEAEPLVNYRGGSN

EIDLEIMAQNVHFMRRYMMQGELAQYNATETSPGLSYETTEQLVEWARRVTIPSVYHPIG

TCAKLPRKWGGVVDESLMVHGAGRLSVADASIFPTNVGATTQQSVYAVAEKLADMIKART

YDEEDRK

>SoG_00067.T1

MAESAGGIDRKADEKMEFSTSKEVTVHPTFESMSLKENLLRGIYAYGYESPSAVQSRAIV

QVCKGRDTIAQAQSGTGKTATFSISMLQVIDTAVRETQALVLSPTRELATQIQSVVMALG

DYMNVQCHACIGGTNVGEDIRKLDYGQHIVSGTPGRVADMIRRRHLRTRNIKMLVLDEAD

ELLNKGFREQIYDVYRYLPPATQVVVVSATLPYDVLDMTTKFMTDPVRILVKRDELTLEG

LKQYFIAVEKEDWKFDTLCDLYDTLTITQAVIFCNTRRKVDWLTDKMREANFTVSSMHGD

MPQKERDSIMQDFRQGNSRVLISTDVWARGIDVQQVSLVINYDLPSNRENYIHRIGRSGR

FGRKGVAINFVTTEDVRILRDIELYYSTQIDEMPMNVADLIA

>SoG_00076.T1

MHSKLIFAGLGTASLAYGAALQNRDSSCSTGMTVSGSINFPVGQLSSGQARCGASIPKGT

FTLSNGGMRDSQGRGCWWTPPTMTLQCDVGQEPTTGWAVGCDGTVSYQGQTTFYQCFGGV

VGEHNLYLGPNAFECGPVTIKADGCHQGCPASTAAPPPPPPPPTSVMPPPAKSSMPAPPP

PPPPSNTGSSTGSCPTTLTGAWEFPHLIIPIDSSKPSSAPSSSYFGEVSSTISSIFNFDI

PYSDSGKTCSLVFLFPKQADLETSSFTFSGDGGVEFSQMASPAVEGQTSYASCPAKSKSF

GSMTIAPGNSYVVSTFPCPAGQRVGYEMSATGNTCFRYFQDYNPSPIGLYITVC

>SoG_00078.T1

MVLPGGGSSWKSSKGGIPQLRAIKSQLTRTRVVQFLAAIGILTLLWRSLSNSYDPRFYCW

GPAKSPMDMTKNEHARWNAHLQTPVIFNHHEPINVNSKSISHVDLNPIVSTRDALVREQR

ILILTPLKDAAPYISRYFDLLIQLTYPHRLIDLAFLVSDSRDDTLAVLAAELDRIQRRTD

GTAFRSAMIIEKDFNFFLSQGVEERHAAAAQGPRRKFLGKARNYLLATALKPEHSWVYWR

DVDIVENPPTLLEDFIAHDKDILVPNDYNSWIESDQGRRLAASLDKDVVIAEGYKEFKTG

REYMCLLGDRKANKDEELELDGIGGVSILVKADVHRSGINFPAYAFENQAETEGFAKMAK

RAGYQVIGLPNYVVWHIDTDEKHRNN

>SoG_00082.T1

MADLASRITNPNEAAPVDGAPAEGTTATAGDNGVDGAQETGLTEVEFDVEVSLSDLQANE

ATPFHSATQWSDLGLSEPILKGLLAVNFLKPSKVQGKSLPLMLADPPRNMLAQSQSGTGK

TGAFVTAILSRVDFTKPDVPQAMVLAPSRELARQIQGVIGVIGRFIENLNVAAAIPGALP

RGEPVRSAIIVGTPGTVMDIIRRRQLDPSGLRVLVLDEADNMLDQQGLGDQCMKVKTLLP

EDIQILLFSATFPDKVNNYSSKFAPNAHTLKLQRSELTVKGISQMYIDCPADNTKYDVLC

KLYGLMTIGQSVIFVKTRESASEIARRMTEDGHKVSALHAAFDGAERDDLLTKFRSGENK

VLITTNVLARGIDVSSVSMVINYDIPMKGRGDTEPDAETYLHRIGRTGRFGRVGVSISFV

YDKQSFMALSSIAETYGIDLVRLDVEDWDTAEETVKNVIKTNRAKASYAPSAFDKTQAAA

AEGAEPAPSPAVA

>SoG_00091.T1

MKRGESLALIRTWHQEYGKTFKVQLGRPRVITIEPKNVQTVLALKFKDFELGERNKALSP

LLGQGIFASDGQVWEHSRALLRPNFVRTQIADMHVYERHVSNLIKRIPKDGSTVDLQTLF

FQMTIDSATEFLFGESIDSLGAGDSQPKFARDFNLSQEGLAIRTRLGPLMFLHKDRAFSE

ATVEARRFVDQFVQRAVEYRASHSKEAGASDASEEGYVFLYELSKRTADRKMLTDQLLNV

LLAGRDTTASLLSITCFTLARRRDVWDKLREEVLALGDKTPSFEDLKSMKYLNWVLNETL

RLYPVVPLNSRTAVRDTFLPTGGGPDGKHPTFVPKGVDVVYSVYSMHRLPEVYGPDADEY

RPERWATVKPGWAYLPFNGGPRICPGQQFALTEACYTMVQIVRHFKSMESRDDRPFVEGL

TLTLASENGTKVALTPA

>SoG_00097.T1

MTLLSPCSGRHGTPLFDVGSQTMRPAIRACKVQLYIHPSMIDAHAMTTQLSLHVHPAISA

AQGFAVVSGWVGCFEPPPRNRGSRLVDAGGGGGGGGGGGLPSSIKTTVDRASYSEILALL

EQVRVSDSNYPFRSPLISYCRTILIDESSRLAWNHLQAKAALDPSLDMEKILRLRLEHLV

LLGAQKLPSLDSLMAVFLALERIVEDALFDGDREPLNTLVNALQAAYDPWQSWSRCDYVD

VNADLFALIFFTVMKRAAFEEVYTEATDRCPFFLPQPDQAAVFSELWVLGSQCEIYFSLL

PRDLGEIVYRRYQKFLEVDPPTAADRKGTEIMTMYSGSEASSSGGGDAETDKDGSSAARM

TRHQRMESWKKRMTEMGAMSIFCLPAVIDVVLLTFIGRGMFMTAFMDPRHLQAASYALLI

SLLLTAGVTGWVGSTGNFYLAHYAYDNMIYFHVQKLSGGFVLTLLVAVGGIVGFSLVHDV

GVGFVFAAYVVVITTYLNLLGVMATMHQPNSPITSGRTVLWRTMPIVLVSPVVSTFVNGY

DLTIYLPVTYLFLLLLLWQYRRLCHEWTGWMDKIPSFNEKDVLEWYGKISKSGKSVVGSG

SDTDGEKSDNQSKDSQSALRLAVLGYQARSRDAVRNGHMKDPFVSKVAEGMPYVDWLFKK

SAPGGKGPEAFTTSWFTSLGEAVKQQRQLSRGLKEHNVLMLFRQAKYDLAQNLGLFLVAL

MDRWVSIVMAGREPTASMYTDKRSRYGICFALLYFCLSVMLLDTTLQKYWSVRFKLSTEK

LRDYDHAQLVAEKSERKRLRKLGQALSELVTKIMLVFGLTALLIWIFVENPETTILYYCY

VLGYTGVIVMQFNRCFTTSVRAHVTIIMLSALAGFLVGITLHAVPSTAGWLYNDVVAQNV

AAVLAATGTLLWSWKDWSSPAKPAVITEAEEKEDDTGSNRSVFVQRHLYTEETESTISAD

RIADLSGGAYINNGTKDSKLARMILELLHSTFTHPNETAEKAPWSEQVTQRALDLWRSEK

LTVITATRESFQSRGLSHVASFSRVRNGSLSVTVGFLGLEELQSDAWRDLLVTLTAEAIL

YHVARAHFNFSHSKAVHAEHCLHGTTTISKRFDFELALESPSTIDCMRRRTNRLLLSHLC

LDANVDAEWLGMPKVVRESIVDRISGRNVRITREWEAWFSNRGGLDLGTLDFHLQLTNLI

FQKGCERQYPLSEFPSKPWHTPEPRAELYPISLSTASTRSLLRRWFKNLLSIPVSFVKWV

AIISGAGSDIERELWYCLRGVFMRSFLLRAILLVWQACRFLKNAWLYTVLIRHRSNLLRT

MRLAGKGTRRKIVRNSINVELPRRSITGFATDEEDGTMVLAVYDGTLKEVPSGKSPIFKA

IYDGHRLKGRVDDGSVGATYHYEPGSRSRWPISKEVMGSRFRTVGFYDKYGRVSQGIIIL

GGSADVLAADFKLVASGSDDILSVYWGIPLGHDASDYNWVPSNKICRIVKVINGKRYVTE

AEYPHRRDPVITTFVEEEDGSRTAITSCPRVFDEEALLLARPNNLSFDMDDLLVYHSPLQ

VRQMKRHAGKTPTFISNLNPLTWLTWGGKRVYQSSGTLDAEPLLRRYWQARDRGQLLKAK

QALDDKIDQIVSAIEISTEVSEVCLLPIRTADLYAMGLAKDANQVTNRPEDCFSDTKDRI

SVIFNDIGCWPEAPGGVSNCRRDLVNGHSTIRNHVLAECANDFGIPRFQVEKNVQSIKLL

PLWGLDGKTASHGLIDNLLQSQVDEKIHDTDVQRDIVDVFIPLLRDFVKGARTKRYSRQD

LIKYSNVMLSMSKYYEHKDYSQTWASKEVESAWAEAWLVPYDDENIADPAKCFDLERPSM

SDFREALGIYLAYFFIFSVKIPERSLFGMVLKHRRGVTFGIWDHAILWRECCLNISPAQC

ELPISVQSMLLSGIGMATRLAYFHADVIMPCTSLFNPMWEAEIGTDQGKLGSRSAFSRRI

DPIVNGISNMDSFTPVDKVRTEKPTVVMLSNVQFIKGVKIAILAADLIVNRYGFKDYQLV

VYGAKDRQPSYALEMAKLIVDNKLSENVHLAGFGNPKEVLKDAWLFMNSSISEGLPLAIG

EAALAGVPIVATEVGATALVLTDPKDQDQRYGEVVPPNDPVALARAQISMLSMVGPWVKF

TEGHSIDEENMALPDDITAEDVAWLTERMHSKSEDRRKLGLLSREVVLHSFHGNRYLREH

EQMYWIQWHQAHMRANPTLDALARQTFKFGAHPPLRYRDAEESEDFEEEEQPNLSDSSVS

SEGKEGTAAAAAAAVKGGGGRGRGGKGAKRHSPPPQSSYKWQDFGRGSEEWKPQRRRLSK

QSGMSAATGTSVQYENRQRQLDCV

>SoG_00098.T1

MHNGTSAATTTTTTTFVDQQNHHGNEDVKLASSAHGGDQDSDDQSDDDNGEDGGYRGRKR

KRPISVSCELCKQRKVRVHLPPPFYRLSTDILDKVKCDRGHPSCGWCTRNGAKCEYKERK

KPGLRAGYGRELEGRLDRLEEVLKSHADILAVLAPNHPQQMLQGSAPSMSSQRPESRDGA

NMMGNPAHALSPRAGAAMFLQRSAGFTPTNQHMGFDQQPPALPGSFQPHMPMPDVSPAAS

MPTGPSEATPQDYYTGGQSGSLSMPASTPVTGDQDMPPYDLLYALVDLYFRHINTWCPIL

HRKTTLDALFGPSTLSETDRILLHAIVATTMRYSTDARLTEERRRHYHQISKQRVLLYGM

ENSSVQALQALVILALDLCGSSNGPPGWNIMALITRSVVQLGLAVETTSALVSPNFTSIY

TLRAMVLPEPRDFIEEESRRRLFWMVYLLDRYATIATAFEFALDDKEIDRLLPCRDDLWM

KNQKVETRWFRSDTSAEDPDEQHQINNPENMGAFGWYVEILGILSNIHKFLKQPVDISAL

SDVEQWQLRYKELDQCLGGWQFSLPNELRMEKLYQTGNKNLNCGWVMLQATYHTAVMRLH

SSAAYPTTRSPIFTPSYSASQRCHAAVESIIAIGEFVVKQNMLSKLGPPFAFTLWVAARL

LLVHGSTVDNKLSPHVGFFVDTLREMGRWWRVAGRYCELLERVLDERGEGERSGEGVMVG

SVRILADMRRTAFDLDFLISRQPRRGTAMGQKMGMEGTKTPRLDDLEYLDVFDFFNVPRL

NGPGMVQGILQQEDMEQAGNGRNGGGGGGGEYNITNFIVDANSDWLFKQDGMA

>SoG_00124.T1

MAPESLDIEVRNNTSSPQLYVSVTGTHPTLGLFMLASDGRTAHHPASPTQTLTPPSVDVG

IPVGGPGSSRTLTVPRLSGARIWFSKGSPLKFFINPGPALVEPSVMNGSDENYESDWAFA

EFTLNQDQLYVNISYVDFVSLPVSLGVISVGQDQAGMRTVPGLPSDGLAKVAEGLEKQGG

PWARLVIRKKQNGGGDVLRVLSPNSAAVVFQGIWDGYYDGYVDKVWKKYANQDLTVNTQY

TWGDVTGRVDTSRDLLAFKGKESTYTFARPSAGDIFSCSTGPFAHLPSTTQEQLNVGARL

AAALNRSTLLANDRQPEGEVIARYYTEQDATNHYARVCHEVSVGGRGYAFPYDDVGPSGR

GEDEDQSGFLNDGNPARLVISVGKPL

>SoG_00125.T1

MHQLVTLLSLASCAMAKCECGYSVSNPGYGSPLLFTDAIESNFGTIDKLGEESGWIPQQF

NVTAKDGRGRYGKMFLPDNVAVEPRGGDTKSTGDEGLRLNVGGQLAEDAVSGAEVDSKRL

DIHWGSFRAGMKYFNDTQEIDMEFLSREYDRNEEYYPINLVVQTEQSKEAGFDASKTGHF

KRVNLGFDPAAQVHEYRFDYTPGKVVFFADGKMLAEMGGDGMPSSGGHLILQHWSNGNPQ

WSGGPPEQDAVLVVSYVRAYFNSSDTEGSSEENDGERCRDEEVVCEVKDVTDGSTGDVGN

GAGDGNTGDEDDANSRGERSMMMVSGMGWTATLAAMMGLLD

>SoG_00128.T1

MEEFMRNKPEIFEGRPGYEGYLNFRVASAAEILKDSGYFTALSGKWYLPLFLPPSLSLSL

SHIHSSLTSLCWETRHLGLTKETSPSARGFEKDFSYLAGCGNHFNNEPQLEEGSFTVPPL

NSDGLWMEDGAWLDRKRDIPEDFYSTRTFTDKFIDMLGNRDAEAREKPFFGYLAYTAPHW

PLQAPPEVIAQYRGMYDDGPEALRDRRLASMADKGIIGRDVEPAPMAGSSNDDDREWADK

SPEDKALSARRMETYAAMVQCIDEQLSRVVEYLARTGELDDTFVLFMSENGAEGKALEAL

PILNGSPLSMVIQRHYDNSLANIGNADSFVWYGARWASAATAPSRAFKTYTFEGGIRCPC

VVRYPPLLRAAGRVSHEFTTCMDVLPTMLELAGVAHPAPTFRGREVAPPRGKSWVAHLAA

GGSGSEPVHPADQGAMGWELFGRRAIRRGRYKAVFTPAPQGSDEWELYDIDDDPGEVRDL

AADEPDILHGLLEDWGNYVQDTGMYDVMIRVSNAPNAAPPEVAK

>SoG_00146.T1

MRFAFIAFILTSFALAASDHENDAATDTVPGVSLRSVNPLVPRQDDKVIRCSRTEPCSNG

DCCNGDSGYCGRRPEFCDKSVCTSNCDAKPECGIRADKFDCPLNVCCSYWGNCGFTEDFC

NERCQSNCARPPSPGHTQGDVRDMVIGYYESWTLAEDGCRVMKISDVPVSSLTHLNVAFA

YIKPKTYVVHPMEPATDQIFSDISELKQKAPGLKIWVSLGGWTFSDNGTDTQPVFGDLSS

TPEKRAKFIDELIKFMRAWGFDGVDLDWEYPGAPDRGGNEWRDTENYVALLAAMRSRFES

EPEDWGISFAAPTSYWYLRWFDIERMMPYVNWVNLMSYDLHGSWDTEDSYVGNYVNSHTN

LTEIQTALELLWRNNVPASQVNLGIGFYGRSFELEDSKCNKPGCKQKGPADAGPCTGQAG

VLSYDEVQTLINGFNLNTVHDKEAGVKYLSWNSNQWIAFDDAETLQEKVKFANDRGLGGL

MIWAIDLDDKKNSALDALLQPDGLGKFAKQNGVSLDLGDWKRQGAQCELGPCSEKPTCPK

AGYLAHGHDLECPKKGERRRVCCDSKNFPKETECSWNDGTSSNSLLSLHGFSCGQAACPS

ENQLMVENDRYWIDGGDSRCILGGKAKYCCKLVGSPMFPIPGAFFTPVPDRNCDFRYGAS

DSDKAKLCGSDKYFFGYMGGDCEYWNEEYQPVCCPNDTPKDACYFPGSQDHNDVETRCKD

YTSCDKGAFATDGSPFTLPNGRGCAYASGYSEWATSLPKKPLCCWIDKLKVETKNVPVRL

DWLFPKLGDESNEETFNLKVKDDKGASTDADNNAFGFVVMSGPKKDLVNLDKRDGSHWEL

FDCEKHDKRQTIRAICTDTGPDSNCGDIYLGQVAETVVELPPGCGAGRYAVAVSLQQSAN

QTIPDHVKPRIRKRRTIAPRMYDFTFDYDFSPIHARDKSDVLLRIDYASEPGYWGKIVNK

EGEKRKRSLQEVHQIVKRDYGGSYPAYMEERFRIEKRETPEHKLHELHERWFSSDLGRWL

DEIRDVDLEYEVVRHRVHEHYKWDIFDESIQCVNYRATMKSWIDLDIDIQVTATLSLIGD

LANPKTWDQKALLFRTKGGVTTKLTVEAYAELFFTTGPFELFGAQNFGLTFAVPGIVTIG

PNLRIIGSISGLATLSLDASVQHKLIEWDYTQRYPIEGGKTTVKDATLVDSEGKPAQAGA

SNSISTGDPKFKADVSARGEITVSVTPKVEFGIVFNYEAIPDTTISMGLENEMTLYGDSG

IGLNTAFYICYGIDARSNLYAAVDAPDIFGVSLRNRWDIFDKPTERRYNNGGTQAFSSPE

RGPAPVLYADRKTTLPKACMLSITSIGNSIQTYKTLHFTRRVYNGLFVRNPKLQKASDKG

ANPEDRTDKLVPVAQNDPKGTDQLTPLAARLFGTWTTITCLIRLYAAYNLHIGPVYDMAI

WTYVVAFGHFASELFIYKSMTFGLPQFFPFLFASTALIWMPSVRSHYVQIQ

>SoG_00147.T1

MMMDSNRMMRILLAVISLCHVASAQLRLVDIKQSDFLTIPSTCFSVLTAEVNCPPVIIPA

RGLGGGYTRYYRDNDLSKLCGSTCVSALTNWHRRVIGACANVRIPSSVGKLTLVAQFAQE

YLETQQTVCLNDKSGKRCNLVMGSAMGVNPSNQVVTRAPGASVLCEDCFLSMLSTQLALP

LAATDSRIKSNYEFFTKSCSKTGWTIPPTPSTTSWDIPVSTGAGPEPTCSGTSVAVSGKS

CQEISMANKVSTRHLLEVNNLAGNCYQFPKSGNVCIPSDGICTPYTVKNDDTCSSLARLA

KATQAQVIGWNSELDSEFGSLSGISEVFLLTTSIIVGCSNIKNLVGLVICLSQPGGAWQN

PDPPPSPTSSVFESIPPLASMADIINLPEPTRVGWNNSNEVAPFANGTRLDCDLYVKAPI

LKDWVTGEETSSCADVAKGYNIDVDQLTEWNPSLKDNCVLQDDVQYCVQLEGQTVPKATD

KCAQFLMARPGNDCGKFVSLYGLDMKEFIAWNPDVGSSCENFKTGVSYCIDVWKYRQTGI

VSTCNQFTVANNTRWVDEPCRIIEDKFGVGHARFVAWNPAVLSNCTGLYKNYEYCVSIPG

YKPTFTTTATAAART

>SoG_00150.T1

MRTARALAILGLAGLVASQFTVEPPTTAPEDTIQDCTNWYVGKAGDKCADIADQSGITLD

QLYRYNPSLKASCNIKADTSYCVEENWGVPPPVTTTAATSTTSSSTGIFTPQPTQPPAIV

DGCTKFHYVEKGSSCEDILKKYSLTIAQLYAWNSGVGSDCRGLWSEVYVCVAGPGGSVTS

TTTSQAVTSTSTGGNGIATPTPTQPPAIVSNCNKFYFVQSGTSCSKVLSDNGITLSQLFT

WNSGVNADCSGLWANVYVCVGVIGGGPSTTMKTTTSTTSSGNGITTPTPVQDGMVGDCDK

FHYVAKGQTCSDVLKQYSLSLPQFYAWNKAVNADCSGMWAEVYVCVHAKSGIIATSTTTQ

RSTTTSGNGIATPTPTQPVADGDTCDTITRKTGVSKSNLLAWNTQLGSTCNVWLGYYICI

GVR

>SoG_00167.T1

MIGPKHEVLLVVRLHEGRGLSLPPPLQTAGGPKASEHLPQGTDHQLLPYAVLNFSESKGF

VCAISGTIESPVWGYEKMRIACERVGLESSEFTVHFYIRDPRSSQGTRDISLGFVTTNPF

LLHKSQTRSEWLQVQGGTGQIRLDIRYTGGTRAQMDGEHPQPNIWKFGHGFGDDSRRRRP

PPPPTTTTREARESNEENYDCQRIWKRNLSPRLDLVRPLLSQPISPFIAPLKFVAESHDE

IHLFSPFFQGGPLFYYLQRDRRFDLARSRFYAAEIVCALGCLHEFDPSYQRLKPKHIHLD

ALGHIAICDFPIFNLEEEDTNRFARPVLECPSPETLSDEGLPPDAVKWWTLGVFLYDMLT

GLPPFYHEDAKEMHQRILSETPHIPESLPSSAKDIIMKLLSLQPETRLGAKGASEVQAHP

FFDGIDWEKVTRREYEPPFKPPEFSQFFKSPVWFVPRPQPAPEELFSGFSYVQPVVNLGI

VLPSHSVMELASPTVATSSPASVTVTPHSPVVVLSTPSIAVSPPPLVATKSSPPPEVSQP

ANDETTNWQLVWEQADRLFYFYNHITKSKQPILDNHVWELKKQSTIEKSQVPAIPGTSAP

LVDGKPSEAHKYAVLMEMLKRNYVHLIPQFLKEYNLTLNPPFHTGGRPLQSAIERGDIAL

VRMFLKNGADVNPDISEASREWPLRIAVANKNQELVRILAPKTKRAPGTRALSDAVRHGD

TAIVEILLANSVKCDFGESDQEKPPTEPLKTLNGGCTFTGLEPDANETVSPLIAAVLSRN

VELAKLLLAHGANANVAYHDLGSLHSSYGLRLNVNCGRAVELAMDLECHDLVQLLLDAGA

DIDLPQPVWVHQCDMVPRTEHLKITARLRAVVAERELARGPSPQEYFKWRRGTGFEFYQF

TPSYIFIFLSVFITIIVVIHIIVFMIIFVVIPAIINKWPEQHPVRRPLLEHGVISPSPCV

QAGPQQTFMDCHGWFVGGCEAPEVFACTGPPNARRDEMVEVIVAPETRAALAANRTLRFL

LKTRYDRDSRHSKVAIAWAQSRAEMLAKAVLEQTGQQTAGFSVQMNIVELLSVHQSFLCF

DLFLDECHKDVRSEVELSFERPVHIAARRGKVHYVQRCDKKTERMVAERYSSIGEVDNGP

LPYFVDVINPPIYDEGFRLEEEPEEGGEEAENTEANPPASEVEKNGPEETRADEPAPEEG

TTEEDDPAIQAHVDSRATREANNAVGEGKAGERKGKRKRIEEDTNAADGEDGSAA

>SoG_00168.T1

MPATTLKTVTTTTGDGISTPSPIQTGMVATCNKFHLVASGDTCGAISTAAGIALSDFYAW

NPAVGSTCTLLISGDYVCVGILGSATSTKTTSTASITSTAPKTTTAITMTGNGISTPSPI

QTGMASNCNKFHLVVSGDTCSQIASAAGISLGKFYAWNPAVGGTCSLLLLDDYVCIGVIG

SAQATTTSKPTGNGISTPSPIQTGMTSNCDKFHLVVSRDTCSAISTAAGISLANFYSWNL

AVGSTCASLLLGDYVCIGVTGSVAPTKTRSGNGVTTPTPIQTGMVTNCDKFHLVVSGDGC

YDIATAAGISLSDFYSWNPAVGSDCATLFLGYYVCIGIL

>SoG_00170.T1

MRLDSLVTVSLLAVACYPVAYAAADLQAARRAAESYLAGITTRPAPATLASLDRQTARSH

VLASNYSAPVDNTTRKRCPASCSSAGNNSNQWFAYSNPSRLDVCNETMLIDFSLLDQLDN

AHKTSKIAACIADLDSASGGQSIDKNITCQVRGLNKAKMTGSLNMITSGPSTSAHLSNIV

DSLQQLQRFFESFGNGCDEMIQFSYSGRSAVGVYVGSRLASQGTIVSVLEKLSTTLQSDG

GVSESVAVQLCNHLSARYSLGVYISTNADFSEIQKAVQTWKNGSCLADSTTSAWHDIDFL

HPTVNKENSLSRNSTAKSSTGNTLATSGTCTTVQVVSGDTCTSLAAECGITPAQFTQYNP

ASNECSSLAVGEHVCCSPGSLPDLSPKPDANGNCYAYTIKSGDTCDALAAAYTIKSTDIT

TWNENTWGWTGCGDLLIGDLICLSSGYPPMPSPITNAVCGPQVPNTPTAPHGTNLSTINE

CPLNACCDIWGQCGTTTEFCTISESATGAPGTAAPGQNGCISNCGTDIITSPAPSSHYTI

GYFEGFDWSRPCLNGGIQGLNTSSYTHVHFAFITFNSDWSLNTTDVDPQLPMFFALRGVK

KIISIGGWAFSTDPIPYALFRDVVATPASRAALINNIVNYLNENNLDGVDLDWEYPDEPD

IPGIPAGTEADVTGFFLLLDELKESMASKAPGKTISITAPASYWYLKSLPVVALSTVVDY

MVFMTYDLHGQWDYGNAFSDPACPGGNCLRSHVNLTETINSLSMITKAGVPSNMIAVGVA

SYARSFEMTTPGCWTEMCTYVGPTSGARPGPCTNTAGYISNYELDLVMAHDPTAQQYWDA

SSWSNIIVYDQVQWGAYMNDTNKAARQMIYEALGFLGSSDWAVDLQDDFGDSSQLAGPDT

IYVNPDIWNSATPVVTASPGVTLIWPPMPLGTPTTITFPPWTTTMSYSTLTTLTTTLSGG

STSVYPWYYYVPIPTVISIPPVTTTEIPVWGFYLNSSATEGTILLTSSVQPSPVVVTVTP

VLNGTTSIIGATATSTSSGGIFVWQGKTEILPPGTETLGGSTTIIGGITLPPHITTLTPN

PHPTTTQTTPDPEINPKTIPWTSGRPPSPTSPPGCPGCGLPCILFCDPDCPFCPPGVFGN

PNGNGDGEDDDSTSSTTTTSEAGSATVLAAEDIGDAFPGLASDDALFSSLFTVELSMWDS

LYPDTIAFPSTTATTSTSTTSTATSIPSPTAECYIWDLIAYEFEIFNIEGWATDGGSELK

HQESGCGALTFWTWHDAAADKAAYVYFDLPTLIKAGCVERAIVSAGGPKLSCKGQGVTFM

VDNGKSDAAPEVPGLTSEKLEQLVKVYGNHSVEAKAHPYVPMNWGANPKFVYRRWGLETV

AMENWNGGRSI

>SoG_00180.T1

MRLVKRTGLVAAGLVIAILLLVFTTSSTSGGGYKLNDHRNGAKLPQGTKSPKLPVCVHDN

STHAQNWAQLKAKHAHLMGDKFTIVVSTFHRPKELKRTLDTLSSLPIPSLHEIVVIWNNF

DEETPATYNTQHGVRVRYRKPTRDSLNEKLWPDPTYETQGILLSDDDVYYRPHDLEFVFQ

SWRKFGQERMTGALSRCTKLNSDGSWHYDFCALTGEEPYSLVLTNLAFTHIGFLDHYFSE

DAAIKKIRESVDEKFNCEDIALNFVSSILTGAGPLLVRGQHQYVNVNPSVGISQQKGHLQ

QRSGCINEFAEVFGCMPLMDEEARIERGHKHNVWYKTVWDRILN

>SoG_00181.T1

MRTRSVKYLVLAAILILLFSLYEHIRQVGEVVRTYGTFHRMIRTNPDLLYRYPQAVDSER

VVPKKMHLIALGNANLTKYQDAIQSCRDLHGDWEHNMWTDDNATEFLSAHYPDIVPHYTG

YHQNIQRANILRYALLHHFGGVYLDLDVSCHVVLDDTPLVKLPFVSPGAHPAGVNNAFIA

TKPGHPFLGFALALVPQYDRYWGLPMRIPYVENMLSTGCMYFSNRWMDYVRDLLAGSGLD

RVYILANEEGDMAPHMLRGKVTTPIFTHGGASSWHGWDAALLLLIGNHYMMFLFGAAALV

SALSAFIFYRYVHSRKRAGYRKIDV

>SoG_00185.T1

MKFLVAAACLASAAEAHTLFTTLHIDGKNQGDGTCVRQPEDPGSSTAPIYPLNGDVMACG

RQGEKSVPFVCPAKGGSIVTFEFREWPAADQPGSIDGGHKGPCSVYMKKMNNVLSDPAAG

DGWFKIWEDGYNTKTKKWCVDTLIENKGLLSVNLPSGLPAGEYLVRPELLALHNASKGDP

QFYTSCAQIFVENGPDAELEIPKEFGVSIPGYVSSNDPGLTYDIYRDTLPEYPIPGPPVF

VPKAISAASTVTKKQTQGTIPKDCLVKNANWCGKPIQSFSDEPGCWKGVDACWKQAEGCW

GSVPPSGYANCDVWQAYCKSMEKACKDQNFNGPVEFTGKEVFAGLPGDIPKPYGKFDETV

VGGGNGGSKSDEKGSTSTVPATTSAAAAAPTATASPQKPVDEGAGKTPEPPKDEEEEYDG

GQPDQSPAEESPALKISTDGRCGGTTGQTCKGSTFGECCSRKGRCGRKTRHCTCGCQKGF

GECKE

>SoG_00187.T1

MPPQTDVRGETEAPPTVITTGPEPETNPNASGTSTPAVRFSSAVDVAPDPPAVTTTNDHD

DDAQDDGHQSFSDVAADQLRALTKSIHGRPLQSKRLTTSYHFEAFSLPASRVQSREDDSH

DSTRLPTPSSTHRQSPHSSPRMSAISSPPLTPSGSGSHGSTEQKQENKEPSERFPGTEIP

TITPEPSSSSHERHGSGDQRVLSHRPRSEGSDIRRASSAEHAPHNEQSHRRGMFKVGPGS

VPGSRESSPSRSSASHFYSKPATPYGDANDPYAKGKRPAQQGFNRQSIDPRFVFSRKKKH

GSPGSSKTNLSEKRNSGIFSGNDSTVSDLGHHPQGSMADLKRFFRKSGHHKKRESSPAPS

GTRTPPTSRSTHQLPFDEHHDLQSKYGKLGKVLGSGAGGSVRLMKRKEDGTVFAVKEFRA

RHPYETEKEYTKKVTAEFCVGSTLHHGNIIETLDILQEKGRWFEVMEFAPFDMFAIVMTG

KMTREEISCCFLQILNGVTYLHSVGLAHRDLKLDNVVVSDKGIMKIIDFGSAHVFKYPFE

TDVVPAKGIVGSDPYLAPEVYDSREYDAAAVDIWSLAIIFCCMTLRRFPWKIPKMTDNSF

KLFAAEPTPGHDPNKLIARAKSTNDLSNTRAREFLTGDEGKDRLPHSQHHQHHSHQHESS

HPKDEKSEDGSAAPANKSSGEKKETIRGPWRILRLLPRESRHIIHRMLDLNPKTRATMEE

ILRESWVADTVICQQLDDGTVIPAEDHTHVLEPHSSQGQQK

>SoG_00188.T1

MITFMPSSRLSTLIVSSLVFLLLLVSFHAWSPQLRPVSPEDGRPIWINDQQSGAFEPSQS

TRAKQIGGIIRKAQEVAASPIKAPFKYKFDELGRLTISARKWVRFLDEAPSSENTHELQK

SIESAIATLYPFLSKSPKHPDSRTPFQDLRDGIVPGSRGIVIPTGKTTMRYAAHLIRSLQ

EVLNTKLPIMIVYAGDQDLPPADRDKMQSRFKNVEFLNILTVVDDATLKLGDGGWAIKAF

AALYAPYEQVILADADCVFVQPPENLYDDPLYTETGALLFHDRLIWQHAYQERHEWWRSQ

IHHPSAALNKSLVWTEDYAEEGDSGIVVLDKSRLDVLMGMLHIAWQNTKAVRDETTYTIT

YGDKESWWFGLELTGAGYAFEKHYGSMVGWPRPGEGKGDRVCSFYIGHVDAQDALLWYNG

GLLVNKRVDSKAFGTPTHLMVDGTWHKGGNRKEDSCMDETKALELPEETVRLLNRSVVLA

KELDHELGFLEHD

>SoG_00191.T1

MSHQDTLQETKPLVVSGPRTPALWTKTLSTLIDEQAALYQDADAIVVPWQSARLSYRQLS

YRSKLVARCLLARRLRHGDTVGVMAGNRSEYLEVFTAAGRIGCIAVVLNNTYSPDELISA

VTQAECKIVFMGSHVGNRSLQDHIGRLLKGKALTPKVVCFGDSSRPEVERYADFVSRYLP

QSVASEAKLKEAERTVNATDVLNLQFTSGTTAKPKAACLTHSNILNNARFVGAAMKLTQA

DVVCCPAPLFHCFGLVMGFLATICHGSSIVFPSDSFNAEATVKYAAQERATALLGVPTMF

IAEIEALEEKKYDISSLRTGLAAGAMVTPALMKHIHDKMGIKGMLIAYGMTETSPVTFIT

SLDDRIEKRYNTMGRVLPHTAAKVVDSEGKALPVGSRGELCTSGFALQKGYWKDDERTRQ

VMRYDSEGVLWMHTGDEGYLDGDGYGHVTGRIKDLIIRGGENISPSDIEDRLMEHESIGE

CCVVGLEDHKYGEVVAAFLGQAKSNPTRPSDQAIRDWVSQRLGRIKQPKYIFWVGEPAVG

PIIPKTGSGKYQKHLVRALGNKLVREGAVAKL

>SoG_00202.T1

MKFAILLLSAALQASAKIYYAGVAESGGEFGLWVNSNGEKMGLPGRFNADYAFASPKTVD

VFVDEQGFNLFRVAFVLERMCPMDTGLGATFNETYFDQYKEAIDYITVTKNAYAILDPHN

YMRYNDPYQQPMSGSVIGNTTDPKAATTEQFAAFWNELASRFKDNDRVAFGIMNEPHDMP

TKLVFDNNQAAVTAIRAAGAKNLILVPGNNWSGGHSWTQNWGGELLPNSEFMGKIQDPAN

NWVLDIHEYLDKDYSGTLAECVNPYETAMAEVTAWLRANGLKAMVTEFGGSDSDGCKAMV

GDALAYMESNPEYIGWTAWAAGPLWGSNSPCCSSSQQLGSLEPGSTAAGGGPGLYDKVWK

PVFVPNKPATLEWDGAIVSHNGNGTTCRRRRA

>SoG_00214.T1

MDVHLLVYDLSRGLARQMSMGILGFQLDAIYHTSIQLSGREYVYDGGIISIVPGSSHLGQ

PMEKLYLGRTHLPMDVIEEYLDSVRSIFTVEAYDLFRHNCNNFTDSFANFLLGKGIPSHI

RDMPQAVMESPMGRMLVPQLTQGINGSRQNGSILGLQDTSRPAQPIAHRAPTGAVRVPKD

STEAQKLLDDASASCAAVFFTSATCGPCRQFYPIFDQLAEELGQKAMLIKIDILQPQMMA

IAQKHSVQATPTFLTFCKGKQEDRWSGGTPGSLRGKLQLLVHMAFPPHPHKKLRLPTFSS

SDTKPVLYSKVPPMDKLMVKMGTDTSGKPEIQSLRKYLEARAKDGLSEAVLPNMTQLGSL

VQDSLKSLPQEALFPVIDLYRCALVDQRISAYFVEEADHQTINAALDLVNKSTSCPYALR

LVTLQMACNLFTTPLFPDQLFRSSTLRRAITMLISSSFLDEAHNNVRVAASSLLFNVSVA

ARHSNSKSTASASEPALTEEDQVELAAAVVEAIGQEDKSSEALHGMLLALGHLVYGMSME

SELADLLRVYDVQSTVLGKKGAFPEEILIAEIGGELLGKGLRRP

>SoG_00225.T1

MVFRTLFPLLAGLATTSNLAAGASASSGPCDIYAAGGTPCVAAFGTTRALYKAYSGPLYQ

IQRGSDGKTTDVTPSSAGGVANVSIQDNFCANTTCAIPIIYDQSGNGNHLSRAPPGGGSK

GPAPGQYDDIAAAYGAPVLVNGQKAYGVFVQSTVGYRNDQTKSVAVKNEPEGLYAIFDGT

HYNNRCCFDFGNAETTDRADDNGSMEAIYFGDSTEWGSGAGSGPWVMADMENGLFSGQGS

GYNPGDPTISSRFVTAFLKGDSSNLWALRGANETSSSLSTYYSGPRPNGYYPMDKQGAIV

LGIGGDNGNLDEGTWYEGVLTSGYPSDETEDAVQANIAQAKFTATSLANGPPLTIGSTIT

FRATTPGFTNRYLAHDGDKVNTQVINSSNDTAAKQSGSWIVRKGLVDSALGCVSLESADT

PGSFIRHSSFELYVNSNDGSKQFTEDATWCPQQSFDSTGTNALRSWSYPTRYFRHYANVG

YAAMDGGWNFFDSANEFPHDASWFVSGTFQD

>SoG_00231.T1

MSLPQHLTDFLAGRPGLKVIRRDDDTFHVTKQVFITTPDVPLVIVRPQTAEDVAALVAHL

SANATPLVVRVGGHDLSARAVAQDAVQIDLRDLASVRVAEDGKTAVVGGGVLSQGVLDAL

AERGLVTAVGNSGAVGWSSWAMNGGYGIMAGKYGLGCDQIVGARVVLADGSIVDADERLL

KAVRGAGPAFGVIVELTIKVYPVTQIVAGVILCDNTPDISDALTTYFDNCRALMDDVKTL

PEELYFQPMILTSPGATSPGWVVQFVWAGPESGKSKEWLEKVLSVCPGGKAMVGAAMPAD

YIRQFTAGGPQHLEGGESRPVSIRSMVPSKEAASVIAKHSSLVKPGYGALFIHMLHGPSV

APGSDHPPSVFRSREPHLLAEILGVAFADAKDAPAATRWCEEAYEALQGVEGVMHDTYYA

LVQASALSLERTFGDDLEFLRGLKKELDPKNVFFNPMPLAG

>SoG_00261.T1

METATFLRLAVGIAVALGKLHRQGLVHKDIKPSHILVNCTDGQTRLTGFGLAPRLPRERQ

EPETLASTLAYIAPEQTGRMNRSIDSRSDLYSFGVTLYQMLTGSLPFTASDPIDGSPPFP

RCFP

>SoG_00274.T1

MRVLDLAWLLGASCVLAAPQPAAAACSTVTLTSTVYVTGALPKTTTSAKTTSQTTSSAST

APTNIKPASGYRNILYFTNWGIYGAQTFPKDIPADKLTHVLYAFGDINYNGEVISSDSYA

DVEKQMPGQTIDWNSPVKKAYGCVGQLYDLKKQNRNLKILLSIGGWTYSQAGKFKSPAST

DANRQKFAASAVKLMASWGFDGIDIDWEYPDNKQEGNNFVLLLKALREALDNYAKRNNQN

YRYELTVAASAGPSKYDLQDLKGMDNYLDAWHLMTYDYAGAFSSLTGHQSNLYKDPNNPG

STPFNSDSAIRDYISRGISPSKIVFGFPLYGRSFGNSQGLGKPFSGPGKGPLEAGMYLYK

NLPAPGARATFSDVTGATWSWDSSTKELVSFDGPKSTNFKADYIIKKGLGGSFFWEASGD

QKGEKSLVGVMAQRFQGKMQQKANMLSYPESPYDNIKNGQKS

>SoG_00291.T1

MASDGRKQPPWIPPQARPNAELPRLKIYNSLTRRKDDFVPVDPLGERVTWYACGPTVYED

AHLGHAKNYVSTDIIRRIMKDYFGFEVKFVMNTTDIDDKIILRGRQRYLFMRFKQEHAVE

DESVSDTVLGSVKAAFRHYIGKHFPCLPPETSVETFEEAVEHAYHQEAKPPSTTDPSAAN

LNQALTVADLLLKAHIGTAQAAIVALKNPGKLSEFFAQTEDILLPYLDALHGEEVDSRNY

KIYLELSQKFERRFFEDMSALNVLSPDLLTRVTQYVPQIVRFVEKIVDNGFGYPASDGSV

YFDIDSFEKAGHSYSRLEPWNKNDSVLQADGEGTLSKGKSTKRSENHFALWKASKPGEPA

WPSPWGHGRPGWHIECSAMASEAIGNTIDIHSGGVDLRFPHHDNELAQSEAYWSTPGCQI

QWNNYFIHMGQLRIRGLKMSKSLKNYTTIRSVLAQNEWTARSLRICFLLMPWQDGIEVTD

ELMKAVVGWEGKLNNFFRKSLDLLKHSSAGSTVTGPADKQISRSLGEAKADVEAALCDSF

NTPVVMRILSDLVTEVNSAEALQDQTVILVAQWITRIVTIFGLDPEGDLRDLERIGWSGL

DIATQAKPYIYPASQLRDGVRALACSGLLDHAAISSLADEITAAVSAPEVNSSSCSEPYS

LVLLKFHTDVKALAAQQAPAKDFLALCDQLRDVHLWNLGIYLEDRVNSQHALVRPLDKLL

VASRAEQESARIERAKAKMEQEARDAEREKELHERAKVSPFLMFKSSTEYLKWDVSGIPT

VDASGTAVSKNKRKKLMKEWENQKKRHEKWLATQ

>SoG_00300.T1

MQQRQPKEDDLQALPHAAPSTPQEATANLDLPVTREGTSSVSGNSPRDPARHRQTVGKTA

WPPSTASASSDQQHQERQRSLARGAGTSFPPPFPSGATASALGSSYSHASPARTKSAKRS

SSRQAKGDGQSKASTSTMASAVPAKKARTGSSPTRQLNIPPAASSPLTAAAATNTQQRDD

LPTSPLFFSHKSTTRHTHRPHAFPPTETTASMLSRIREDSSGGVTTLKLPRASVSTTALS

GSTPGSWGSSMEVSVPSLSPDCRTWPTELQFINSVGVLEILEQDDRPTFLVDLNNTANAT

RTGFHIHYMNPALRRSRTLLNDILVLDETDMDAAEAYAHFKAWLFSPLKTQDGVDLPQTP

YAYANATWSSSTLRRRFRFVKADASYLEFRTDSPLPITDEVILPTSNSRAVSPSLPVTPD

ADAVEAADYFATAARKSSQDSDTVPPLDLVPMDTDGDRRHPDDFTNEVLRSQPARSSFDW

TRIELNDSLPEHIKFARSVDWGATSLGPIEEWPHDLRAMANLVMGSPHPAAMYWGPDHIA

IYNSAYIELAGKKHPSMMGSSYSVAWAEIWDEIKPIFQSAWEGGQATMKSENQLVINRHG

FLEEAFFSWSIIPLVGHDGEVVGLFNPAFEDTRRLVTERRMLTLREVGERTAAATCISDF

WPQVQKGMEYNDLDIPFGLIYAVKDDLESDMSSLHSGSLGHAPQLILEGAIGVPEGHPIA

VQSMDLRSSEEALAPYMRQSMTMGGLPVVLSEEDGTLPQHLLEGIQWRGFGDPCKVIVLF

PVIPTTGSEAVVGFIVMGANPRRPYDDDYKLFVNLLGRQLATSMASVVLFEEEIKRGQKA

ARLAALDRQELSTQLQLRTEEAVQSEYRFTRMAEFAPVGMFIADSKGHINYCNDMFWQIS

RHTRAEGAEDLWMDSVQPDDRERLNSAWQKLIDQKTSISVEFRFKCSQQDGDHTIETWVL

MSAFPERHPDGALKSIFGTMTNISSQKWAEKVQSERREEAVEMKRQQENFIDITSHEMRN

PLSAILQCVDQITNSISSFSHYTVKDEVEQLLESCLDAANTINLCASHQKRIVDDILTLS

KLDSNLLAVTPVDDQPVRVVQRALKMFEPELIARDIEFEFNVDSSFAKNGWKWAKLDPSR

LRQVLINLITNAIKFTQGREKRLIVVDLSASRNLTNLKQNGVNYFERNDNQRNAAMDINN

EEWGTGEVFYIHCSVADTGPGLNAEELKLLFQRFQQASPRTHAQYGGSGLGLFISRILTE

MQGGQIGVSSRRGFGSKFNFYIRCRRCTDPPSEYEHISPFRIARKMQSPENLGGSQPPGV

SLSPAQSTQEAETGPLYDVLIVEDNIVNQKVLQRQLRNCGNNTFVANHGKEALQTLEKSR

FWSGKEAEGVDISVILMDLEMPVMDGMTCARRIRELEREGTINAHIPIIAVTAYARPEQI

ENAKAAGIDDVISKPFRIPELLPKIEELVGKYKNLSVSS

>SoG_00302.T1

MANKIIAPYGDWPSPISINTITGKSRGISDPRVSPSGASYYLETRDNGQNTIKKVTETGV

VEVLPEEYYAGNGAYEYGGSSYEVLNDERIIFTHGKDNTVNVLDPETKHVTYLTGRDPVL

RYSDFSPNLTNPWVLAYQEDHTDSKPGDVKAYVVAINTETGEERRVVSGADFYFTPKFNP

EGTKLVWIQWNRPDMLFNDSELYAADFEAASCTVSNARQLLSKGNGLAEPHWGPDGTLWY

CAEKHNYRSLFRILPGEEEASEVSVKGQHEDHIELGELGLGQGSRTYAPLSARYVVAGGY

CNGITKLTVIDADAGSSRTLLEDEIQHLSGRSMARLDDRRVLVVSEGGGAQRSLRRVDVE

SGASTVLRAASDEQMAGWVVLSKPETVRIEARGPPKRTIYGFLWMPRNDGCEAPAGTKPP

LIVDMHGGPTGAVGCGLDLRVQYFTSRGYAYLKVNYTGSTKHGREYRQALFGNWGIVDAA

DAAECAEHLRAAGRVGKVGITGISAGGYGTLQCRAQGLRRAHAQARDGLHGCAGDQGGHI

R

>SoG_00325.T1

MSSSTIILLNRAITPRLLATCSRQQLRKASNSTSTPCSSLPLLRRIPKKPLTARSFVSSA

TSLKHDNNMSPSIPTEQWAQVLEATGKPLQYKKIPVQKPGPDEVLVNVKYSGVCHTDLHA

IKGDWPLPTKLPLVGGHEGAGVVVARGELVKDIEIGDYAGIKWINGACLACGYCLSGDEQ

LCQKATMSGYTVDGSFQQYAIAKAAIVARIPKECDLESVSPVLCAGITVYRGLKESGCKP

GDTIAIVGAGGGLGSLALQYAKAMGLHTIAIDGGEEKRAHTKNLGATAYVDFTTTQDIVA

DVKAATADGQGPAAALLVAVQEKPFQQAAAYVRSKGVVVCIGLPADAKLSNPVFDTVIRM

VSFKGSYVGNRADTAEALDFFRRGLIHVPFKTVGLSELQKVFDLMEQGKIAGRYVLDTSK

>SoG_00331.T1

MGGLRSRIISTLLLCVSSLGLSQANEYVLSAPRGSRGAVASEAVECSSIGRDLLAKGGNA

VDALVGTVFCVGVVGSYHSGIGGGGFVLVRDKDGNYEAVDFREAAPAAAHEDMYKDNVLS

SMIGGKAAGVPGEVRGLGYIHAKYGVLPWKDVMEGAIYIARNGFRVSADMERIMHDAMNA

YNYKFLSEVPDWAEDYAPNGTLVKTGDIMTRKRYADTLEKIASEGPDSFYRGEIAEAIIN

ALDRSDGIMTLQDLANYDIISRNVTTTTYRNLTLHGIGSPAGGAVAFHLLKTMEHFPPDT

FSEDEPLTNHRLVEAMRFAYGARLQLGDPDFVENVGSMEKEMLSDERAKETRKRILDDAT

QPVENYLQKKVFLRDSHGTSHIATADASGMATSLTTTINLLFGNLIVEPKTGIILNNEMN

DFSIPGEPNEFGFPPSVANFIRPFKRPLSSCTPMIVTSNEHSNPPSFLATLGAGGGSRII

SATAQVLWHVVEHRLTMADAIAKPRLHDQLIPNVTALEHAMRESESVVDGLVDRGHEIVW

VPPTKSAVQGILRGADGVFEAAGEYRQKNSGGLTL

>SoG_00333.T1

MPGGAWSTFFTRAAATVFYKSVQPKISADDGKVTADLLTTQAVAAIETLNDEWYNVKTGI

WDDAWWNSGNAFTTLADFASLKPAEANEINIGGYLRNTYIQAQTVQVQTTKEIDAVGKVK

SFSSIGAASTEHDVQNDTESGSGRWGKNTWDKRQFAQFRNEFYDDMGWWALGLIRAFDVT

RDRGYLDSAIVIFNDFATGLGGPCNGGVYWNRERKYVNAITNELYLSIAASLANRVPSDP

SYLKVARDQWQWFKKSGMINDKDLINDGLNGECKNNGMQTWSYNQGVILGGLVELHRANR

NAGAPKDDVSLLLEAKRIALAAIKHLSNEDGILVETDKCELRAGNCGRDGQQFKGIFVRN

LRYLHEAAPEKEFRKFILDQAVSVWKNNRDEAQGAKDKGRSRLGVAWAGPFVKATGASQS

CALDALVAAIAVV

>SoG_00335.T1

MHRVLVPALQKPAAAPTANSALPRNDWHLTQYPSEKFQTSTTPGEPVSTTCFLAYRLSTC

NKPAFVASRACGALPSDKPAVAEKAARDILYPNLSFTHASPFSIGNTSFGLTDTSSHLSH

GPRRIVIPGAVLWKASNLPAHPEVGLKFLPSYTALHVSPLLQPAKSSRRVADVSMADRSP

SLFDSFGASSLLNMPPARPARRLVSNENDENSNSTRMTRAKAAALNVDELAMPAKGALQT

KRSAVNANTAAGRPRAALGDVSNVGKLGAADGKKAVGGKAVSKGVGLVSKAAQPTGVQKN

TTRAPRPALASKETNKVEVKKTGSGALTQGQKRRAPTAASSREASVTEPEPARKKQQAEQ

ARAAPQPKVEPVVEPAPVELDDPRNLDDDDLDDPLMVAEYANEIFEYLRDLECTSIPNPN

YMEHQDDLEWKTRGILIDWLIEVHTRFHLLPETLFLAINIIDRFLSEKVVELGRLQLVGI

TAMFIASKYEEVLSPHVENFKRIADDGFTEAEILSAERFVLSTLNYDLSYPNPMNFLRRV

SKADNYDIQSRTIGKYLMEISLLDHRFMEFRPSHVAAGAMYLARLMLDRGEWDATLAYYA

GYTEEELDPVVQLMVDYLARPVVHEAFFKKYASKKFLKASMLARQWAKKNAPMFDVVNPH

LSLDQIS

>SoG_00366.T1

MQLHDLRGFPLSHRRSKHHPVSEKGSYGCVGAPGHKISGAMPPVPPSATVPRIVVYYQTL

HDPSGRSISILPLLTQPGIAVTHVILAALHINGEPSELTLNDHSPAHPRHETVWAELRVL

QAAGIKVMCLLGGAAQGSYQRLDGSEAEFERFYVPLRDLIRQRSLDGVDLDVEEEMSLGG

VIRLIDRLRADFGEGFTISMAPVAMAMLDSTKNMSGFDYEALEVMRGRDIAWYNCQFYCG

WGDCSSPVMYDVMVRKGWPPEKLVVGTLTSPDNGPGFVPSSALSPVLNVLKGRYAGRFGG

VAGWEYFNSLPGARERPWEWARHMTGLLGGHVTDQRGAGVAKKAEEEDGGVGGGGGGEEA

DPDDDSSAKAAPVPSQFEYYSDGVDED

>SoG_00368.T1

MTRLVNLLLVPAAALALLAGVAGADGSCTGRSDACSQNPIPLRKFNPLNFHAETCNLKWT

LGCTEPDKHGGVQQKPPPYSLCDGSRRAAGLIPVDAYTLALQNNDFAPKAAGITLHGLWP

GSAGGSGSKNQPYGCLNGDEFDQTILDTFRGLLEYYWPTDPKYKNTVPCFILSEWMKHGT

CAVITGADGTAYRLPQDAYFRTAFVLAAEVNANEALRENLQYAEDDEPLITTSCVECAYK

ATVGWTGTDVDSVPRMSPDCIDQCFACGDHWDRCPPAQLDSTVKPDPSNAPSAVKQGDGG

DEEKRDSRNATVPMPDYGMPAFVVMSSGESSLSPWQGTWRSFGAEQLGSGKFEFAQTFSD

DVLKVTTDSPYPQTCRYQQQGPGGMVLRCGTPDKHHLEECLVQRLPDVGGLEAAFLACNH

SGTPAPVSFYAAMDTPQCGNFLMFRCKPSSEGCSFSPDVVSSKASESTNTESEPDSKPSP

VSHSQPGIGGVPPSLLGSWRSLQVSSDYEEGLARWNFTSDGQASLQWPNYLGRGLQRYSV

SHSGEDPSVVRLASAGGAVTTCRFQFQYQPVYSYAVLDCEAGDGDDDDLSNTKWAMGRCN

PCNAHCRYSCGPENDFCGAGSCDPAKSVVQAAAAASLPSPPPQSDDALAAEESLVGSSSD

AQGHDWCTPEDPSCWPSETAIRKLELELDPAVPRTGLRWSSYPQPQPAPVPSGSDKNQSF

YGLGADSLKALYCYQDVSEMKRPCFNPADNPSVWNTASDVCKAAVHQNEYRNWNPFIVVF

PLNERHVAAALTFAARHRLCISTAGTGHEYNSRNSCPTGGILIRTILLKDKTFLPSWSED

TALAPAGAFRFGAGSIFAEMHAFSAEYKRVVASGWCSTVGMVGFHLGGGHGPFAPSMGLG

VDNLLEIEVLQVGRDARGQPVVHKKVASRRRNPQLFWAMRGGGGGVWGVILSMTIRAHDV

PQGGLSRVFIAQNGTFCQDDRQFGYEWLRGMWTRFAAWQLTLNSKVSTQPGFFINTTKYT

TTTTTTGAGDLCDVKWSFTFEYFYAGGQSEPDYVMFRDRLRDVVLEAKPEDVQENNFNSA

YEYLLTMPPNKFLLSATNPLPEPHPPSDDATGSQNSVFVSRQAMESGFPSTMMKVLDICV

ATLEKPDYTSPDPSGHRCGFHYLYTSITGNLGSSRPGDTAISPGFRSGLMLWNARTLTTK

QSDDTIYRLGPNSYFSESSYVMHNWTDRYWGPKTYPQLLAVKKAHDPGNHFWCHHCVGDD

PNDAYGDPLAAVGKGE

>SoG_00371.T1

MKRRPSLSTDDDAESSLESDLRTGHAAPPKSSSTRRPSSTRSTRSNRVNWEHTSGLKGYA

SSDSIGDSKTLPQPSLTTVHSAVSMVKERLSEGSARPTRSLWTVSLLTFVVAVLGITILG

GIVGSFVTRQSDPKGCRMSFMRPSYIPFAEFDTEHTRFATKYSLYLYREQGVDDERAVRG

VPVLFIPGNAGSYKQVRPIAADAANYFQQQYRQGIFHDKSGVRNLDFFTVDFNEDITAFH

GQTLLDQAEYLNEAVRFILSLYSDPQRSSRDSNLPDPTSVIIMGHSMGGVVARAMLVQPN

YLANSINTIITMSAPHARPPVTFDGQLVQIYDEINDYWRQAYAQKWANNNPLWHVTLVSI

AGGSLDSIVPSDYASLESLVPATHGFTVFSTGIPTVWTAMDHQAILWCDQFRKVVSRALL

DVVDVNRASQTKPRAERMRVFKKAFLSGLESVAEKTGPVDGATTLLTLSAASETLVRSGG

RLVLNGLGGSSTPKAHLLPVPQETSPGSKRFTLLTDAQIDGNGRNNQVDVLICSVSPSQH

PTVPSSHFSATIDLSGSKKPTAILTCRNAAADRILLPASTVASKTTFCLERDCDSPTFSY

LQYDVDDLYDHRFIAVVDKTSKPSSNFVIAEFTELSDFRRVDNSSLAQLLTIGTTLEMPE

KRPMTVEVTVPSLRSALLAYDLDVSRTQCSNGRMALFQPLMRQYLEKPYESRYFVNVQHA

SVTLHGVAPYMPGPLEPNLEDGLTLQLWSDPSCGDAIQLRLRVNVLASMGKLYMRYRTVF

AAFPLLIVALVLRQQFRVYDNTGVFVSFSQALDLALRQSIPLLLLSLTLLSMSISGATIA

ATGLLRYTYSTKSNSDNFYANELLVGTKDPFFWFLVPLIGVVCVGVCVLVHYVALAVTHV

GALLYWLTSTVRRSSTGTNRTRALSPAFVPSTPRRRMITTVVLLFLVATFIPYQFAYLVA

CLVQLFTVTRALRIQKSISSVGNTNYYNYAHSILLLMLWVLPINLPILAVWVRNLAVHWL

TPFSSHHNVLSIMPFILLVENLTTGKMVPQVTSGLRFVTSVLLFGTAVCAAVYGVSHAYL

LHYLVNFVAAWLVVIHSTAESWSLARLSAMFEVNPDDARNRGKRS

>SoG_00385.T1

MTGHPTQQPAAASHVLDTSSPPETLSSSPCAVNDAQRQRTILRTPEGEVEYPGYVPAEVE

SPLATTKSHTAALQRPASISAREPDLLPDATAEARPRRSFSHDYSSIQPIQSSPLGNRPK

ATRQWQTELPDARACGRTRPAAAQDRSYLGKAMREGNEEHMATHDEFPCSSSSSESETEH

AKTKPERNSNPKIRKHRPRRRNRTAADRMAAHKTGRFNVGNENYKTKGEVKRDGRLAITV

QETANTGYIAKALGSAMHKFVSSDEGNYAKRRRESRLSTASTETPDISPKPCLNIVVMVI

GSRGDIQPFVRISQVLRDEGHRVRIATHPAFREFVEKDSELEFFSVGGDPSELMAFMVKN

PGMIPKLETVKAGDIGRRRAAMAEMFDGFWRACINATDDEKDSHNLKMMGSKHPFVADAI

IANPPSFAHIHCAEALGKSSTPFMPSVLPLGLRYADVSIATGITVTLVNPFPYTPTQSFP

HPLASIKKSNVDPGYTNFISYPLVEMMVWQGLGDLVNDFRVKTLCLDPVSTLWAPGATYR

LKVPTIYLWSPGLIPKPPDWGSEIDVAGFVFLDLASSFNPPTELEDFLKAGEQPIYIGFG

SIVVDDADAFTQMIFEAVKLAGVRALVSKGWGGLGGQDIPENIFMLDNTPHDWLFPRVSA

CVIHGGAGTVAIALKCGKPTMVCPFFGDQHFWGQMIGSAGAGPEAVPYKSLSAEKLAEGI

KFCLTEEAQRAVNEIAESIEAEGDAAENAVKAFHRHLSLNPPNSVRCSVFKDRVAAWKPK

DSHIKLSPLAADMLVQSGHVTWKRLRLHRHMEWNDFEGPGEPVTGVAGSIAKSLGNVFGG

IGGVPQRMHRTTKKRKELEEEAARGNDKQGETGDDGRMQNGEAASGSPTAIPHSDTQPMT

TEEVAEEYVGDVTSGFGQTASAIAKAPVDLSVALAQGFHNAPRLYGDDTVRRPVRITGIS

SGLKAARKEFGYGVYDGFTGVVRLPVRGARQGGAVGFVKGTGMGLMGLVLKNMAAVVGPV

GYTLKGVVKQVERRREPLKYVRRGRIVQGQREASLIDEKARDSAMSQAIENWTLLKELET

EIASGEGQRGIVGQFDKVLLDTKVLFEDVDTAKHALESLKAGVSLELVIRDALGLEDERG

STDGRQSRSLRRSLNFGSKSRKSLSGAGSKSLKSLVSPTRANDTSRPASSTSRKSLKSPT

GAGPKRKLSSTVEGEEVEGSH

>SoG_00402.T1

MGLSTAFTAGLLALQLFISPIAGVPAENSVSISAPRQVNAAASDWWFATIERKGTVPFGS

VGEGYKVFRNVKDYGATGDGSTDDTKAINAAITDGGNRCGQGCDSSTTTPALVYFPPGTY

VVSSPIIPYYYTHMVGDLKSLPVLKAAPSFQGMAVIDANPYDNQGNNWFTNQNNFFRQIR

NFRIDLKSLPKTTGTGIHWQVAQATSLQNIIFDMVEDSSEDNKQQGIFMENGSGGYLIDL

VFNGGNYGAFFGSQQFTAKNLVFNNCKTGIYQIWNWVWAYHGLTFNGCGIGIDLTAGGNS

QTVGSLTLQDSTFTNTAVGIASHYNPQQGPWTNGTLVLDNVDMSGAGVAIKNPGSGGTIL

AGGSLVTSFVQGRDYTGSQGQAVQGTRDGIQKPAVLLDDKGRMVSKEKPQYPDVPASKFI

SVKASGAKGDGVTDDTAAIQAVFDKVAADEIVYFDHGAYILTDTVNIPCNVRIVGEIWPL

IMAGGNSNFQDQNNPKPMLRVGKPGSTGNVEIQDLMIETKGPQPGAILMEWNVAGETKGS

AGLFDVHFRVGGSAGTDLQSDKCSKTPQQTTPAKPECIGAFMLMHVTKDASIIMENDWLW

VSDHELDRPDFNQINIFNGRGILIESTKGAWLWGTASEHNTLYNYQIDKAENVFMSCIQT

ETAYFQGNPNALVPFTPQQSWNDPTFADCKSENCARTWGMRVLDSKNVFLYGGGLYSFFN

NYNQDCLNTEDCQQNMVEVNNSQTTILALATKASVSMLNVNGKVVAVHADNDNNFCATLA

IFKSQS

>SoG_00403.T1

MVGSTAPYDWAAQNAMFDGGYDSNMSRAHNQDSGKAGFARSQQQKLRSLSSSFLWSRPKS

SFGLTMALDHGTSCTNEVIVEDEWDTPEPAGPSTYEQPITGFKSMVRRASKSLKGMVSRR

PSTAADEALGSSANLRPTTSHSLWTGRLRPRNGSAHHARSFYGLEFTDEPKPIRDSQPRD

IPAWSPGTFLGPPIIPEHTGAAAKASAALQNEYLARSLQTKWLSTSPSDDGNDRESGIGI

AVSVSGADVEALSRSESRQTISRVDFISELPVELAIHVLACLDALALCRASLVSRQWSKV

CRNQHVWRESCLREMTTTYATSGPVKPNTGFGIPRNLPTSDWRQIYRAKQELNQRWKSGK

AHPIYLNGHKDSIYCVQFDEYKLITGSRDKTIRVWDLHTLKCSLIIGPEETLRRPDMLES

ADGMITHNATGSDNRVVSESVPEMVSFPMHHDASILCLQYDDEILVTGSSDATCIVYDIK

AGYRPIRRLQHHTAAVLDLAFDDKHIVTCSKDISICVWDRATGALLRQLKGHTGPVNAVQ

MRGNTIVSCSGDYRVKLWNIDTGKNIREFSGHTKGLACSQFSEDGRFIASAGNDQTIRIW

NANTGELHREIQAHEGLVRSLHVDSVSGRLVSASYDTDIKVWDMETGRQLLDFPHWHASW

VLSAKCDYRRIVSTGQDPKILIMDFGAGVEGIELLESDQPDAQKRASYI

>SoG_00404.T1

MVNYPEKFEGFQSPGLQNWTDFQKNAFTPRPFNEEDVDVEIECCGVCASDMHKINGDWGD

CPYPLAVGHEVVGKVVRVGSKVTLAKVGQRVGVGAQIYSCLKCKQCQNDNETYCAHQIDT

WGQEYLDTGHITQGGYCSHIRVHEFCLRHRVYPIPEKLKSAQAAPMLCAGITVYSPLKRF

GAGPGKKVGIVGIGGLGHYGIIFAKAMGAEVWAISRSRSKEEDARKMGADGFIATSEKDW

NIPHKMTFDLIVNTANATDGFDLNQYLSLLDVHGRWNSVGMPGGEGFQIKNQDFTENGCF

IGSTHLGSRKEMLEMLELAAEKGLQSWVEELPIGQPGLKEAMDKLKNSKARYRSCMVGYD

KVFA

>SoG_00409.T1

MAPRDIFAEPIDTKAPPSNIVRQKDHPVPRTGIATKGPLQTNKFFANFHLGDRHGPTFTF

PYSIAWAGGKGATGSWGMACSHVEARQRVFGKEKLNGASSYYLNPVGIQALVLGAADLGK

DTALSMDSITAFSTRIHLSRDVNSPPAVSFPVVQGMAFITGLYSGSIPRIQTGIYFKTVT

RVTKFAKSHVTKFNFNLEDGSTWRLYAYRTKGDDLELEVVNNMLAQSKKPFFGSIQIAKD

PGTTGSEQALDDGAGVYPVTVTLSGTASGETGTYSFNFQREGHVMGNLYMYALPHHVESF

DGETTGGLTNVQLQTTTKGIATLVQGSRWTMLEHLPTHMHVAPWHPEKGNMTKLSDEAKG

IVHAAATKELSQNMVAQSNLDSMYFSGKALAKFATICYVAHDLVGDISLAQHGLGQLKAA

FGTFAANQQKFPLMYESAWGGVVSSATYVTGNAGADFGNTYYNDHHFHYGYHILAAATIG

HLDPDWMKQNKDYVNTLVRDVANPSSKDKYFPQWRNFDWYHGHSWAHGLYAAMDGKDQES

SSEDMMHAYALKLWGKVSGNADLEARGNLQLAIIARSLQQYYLYRNDNQVQPKQFIGNKV

AGILFENKVDHTTYFSPDLEAIQGIHMIPILPPTPYVRVAEFVQEEWDAFFSKGRVDNIG

NAWKGIIYASYATVQPKLAWQFFSGPEFDPQWLDGGASLTWSLAYAAGEFEKFKHKSKRL

C

>SoG_00430.T1

MICVSFLRWLTYLLLVSLYHKSVSAEVWDGPNPCKGGLAPDVWLNQQGGLFNAGQIKFDE

GWNNGYAGPGGGGGGSSFSWTKRDVFYSPIDRGEVMNFANLGGLGRADLIQVYPLTNEAY

TFFKPYRLYTSNLEPAHQKLPPKLLQSSIPGDKHKSATQSERQISAPVAMDSPILSQLFR

QLFRHRPAGCRNRVRVPLNLPYSHAATMHQTRLYTGKRMTTTKDRGMKTNESRWQQRTNI

LPEDRLQEFEKYPFITGEELRHRTSRPKRVKMYLRDFIEDSLYNPSYGYFSKQAVIFSPG

EPFDFGAIRDEIEFQAELGRRYTTFEDKLDDEEGRENPTRQLWHTPTELFRPYYGEAIAR

YLVDNYRLTTYPYHDLIIYEMGAGRGTLMLNILDYIREVDPQVYDRTKYNIIEISPALAK

MQKHHLLSTAESRGHADKVEIVNRSIFAWDQYVPSPCFFLAMEVFDNFAHDGVRYDVATE

EPLQGHVLIDADGDFYEFYSQELDPVVARYFRVRHAATGGKYPKPYPSNPALRYLSTKMP

FAANLSDPEFVPTRLMQFFDVLERYFPGHRLVTSDFHALPEAIKGLNAPVVQTRYQRRMV

PVTTPLVHQGYFDILFPTDFDMAEAMYRAITGKLTRVMSHGDFMRRWAYVEDTETQSGEN

PLLSHYTNASVLVTA

>SoG_00444.T1

MAPKAASSGKDKYSVILPTYNERKNLPIITWLLNRTFTESLRRKCANEFGGEKNSKLDWE

LIIVDDGSPDGTQEVANQLVKAYSPHVVLKPRAGKLGLGTAYVHGLKFVTGNFVIIMDAD

FSHHPKFIPQMVAVQKTANYDIVTGTRYAGDGGVYGWDLKRKFVSRGANLFADTVLRPGV

SDLTGSFRLYKKSVLQKVIESTESKGYTFQMEMMVRAKAMGCTVAEVPISFVDRVYGESK

LGGDEIVEYAKGVLSLWLKV

>SoG_00451.T1

MRIVHFTTVVFIASQVADATPFIFPFTGRGWGGRSRSDRDRSQTEARLPLSHQLLRSSTG

FVAFGDSYSAGIGTGVNGSSDSCLRGLGAHAQLIHRDFTELVGEEQTSFQHLSCTGATLD

DLLSGGKYSQIDEFNTTETADFALLSIGGNDLGFFDIMNSCIFRFYSFYSGTCEAALENS

ERQLQDPAFEERLRLAITEILERVRWEKRLWFTVTVTGYARFFNDETEECDECSFGVWWG

GPKLKRELRQRMNEMVRKVNQKIKASVDAINARFAVPRVLFVDYDAAFEGHRFCEPNVTE

PDYTRNETWFFLVGGQDNGQSLGNHTLPEEPPTKHSLSAESPLVDPDHCLEPAQRSGDWG

QLALCMMAMSVRDDPTIKTTNGELAAQKSMWHAPLYYGKTFHPRSDGHHAIRNEIYRLWK

DQLRMQDEYAEL

>SoG_00462.T1

MLEGLVAGLLNRFLGMYVKNFDPTQLKVGIWSGDVKLNNLELRREALDQLKLPINVVEGH

LGALTLVIPWSNLRGAPVKVFIEDVFLLASPKEDAEYDQDEEDRRKQRLKMEKLDSAELL

KERNIEGLSQEEQKKSQSFTQSLVTKIIDNLQVTVKNIHVRYEDSISAPGHPFALGLTLE

EFSAVSTNGEWKPTFIQESTMVTHKLATLGALAVYWNTDSELLGTGREALDHDREPMAHD

DLVEKFRDLIKQNRDASGSHQFILKPVSGQAKIELDKSGDIRTPKFKANLLFEEIGLVLD

DDQYRDSLMMVDLFHYFIRHQEYKRLKPQGVSVKEDPRAWFLFAGNAVLGKIHDKNHKWS

WDYFRERRDDRKRYIVLFKKKKQQQQLTVEEAEDLNRLEWKLGYEDLRFWRSLARNELKK

ENAEALKRQPQQQEQQGWLSWMWGAKPQEKIEQNEENTQMTEEQRKELYEAIDWDEKTAL

ADDIDTPREAVKLALEASLSTGSFTLRRNANNNPTDLLSLHFDVFKAKALQRKDSMLANV

SLGGLRVNDGTTPDSLYPEIVRVKDAPENRKQKRLSLVELENEAEVEPFFEFEVEKNPIE

REGDIAVLGKLKPLEIIWNPNFVVGIADFFRPPERHMESITALMESAGATVEGLREQTRA

GLEFALEEHKTINAELDLQAPLIIVPVSITSRGSTCLIVDAGHIHLNSELVDQSTLKEIQ

SKQRQAYSDDDFKKLESAMYDRFIVKLTSTQVLIGPSIEEAKLQLTERDSENMYHIVEQI

NVDFVVETSILPKAPNLTKVKVSGHLPLLHAAVSDSKYKSLMRIIDVAIPKFGNDLPLSD

GKKKTDDETPGRPPLPRQSTNRSRRKSQRDRRLSSAFPFMQPQTAVVLDDIDDDDDDFED

ADDGADKEQLKMQQRGFEFKFKVDTLKGSLYRSDPDKKKPDTLLVELVAERFDLEFYTRP

YDMAAEVSLGSLTMDDFVDNPPAEFKSIVSSGDSEDLQAGRSLVSVKFTKVNRLSPEFMP

VYEGIETNINAQVSTINLVVTRKTLLTLLDFILVTFAGGNNDAQPAGSPASEDKDFDDLV

LDEAQQGTPGSIRVKVDLKSIRLILNNDGIRLATLSFDRADAGVFLRGKTMRVSARLEDL

SLIDDVNQGVSRDSSLRQLVTIQGQELADFRYETFDSTNQKAYPGYDSSIFLRAGSVKVN

FVEEPFRKIIDFLVKFGKMQALYNAARQAAVSQANQIQQSPSRIKFDVVVKTPIVVFPRT

VVPGQPKRDLVTAYLGEIYLQNKFAPLDDSEDADVAMKVSAGLRNVRLTSDFHYTDDRSE

ELEMIDHVDIGFNITHAEHKPGSKRPVTEIEGSMSDFNLCITQYQLKALMEISRSVPAAF

VSESADGDEEAARAVDQETLQKARTWNAEERSKDDQPLVSLGPELSSVETTWTTLDFVFN

VNTIGLELFMAPEDEPVGDLKSRSLSRFSLDETKVKTRMLSDGSLEAELLIRSFTISDSR

PRETNKFRRIMTSSSKEVQQLMASVTMSGGQERSLIAMATIDSPRVIFALDYLFAIQKFV

SEGLATDETSIVDDESIMETPDESDAESMQVTFTGAGSRASRSQSVQHSDTPRSTAAPEK

EKQSSMSIAFRVNLVDAQIILIANPLTSSSEAIVLSVRQMLLSQQHAMTFQVSEIGMFLC

RMDKFETSRLRIIDDFSIQLSMDSSQPTITSIHLEVEPLILRLSLRDVLLALQIISKAGE

LSDSGAKELEKSPAKQKADELRNAGLKSRTASGKGRSTYAGKGTGAKSTAASGRPRTAQS

VAPVATAAKKHEELSATVEGIRVVVIGDLHELPILDLGIKDFTASAENWSSNLKAETSID

MYTNIYNFAKSSWEPLVEPWQVGFGVAKDPTSGLMSIDVVSKKTFDVTITTASIALASKS

FDFLTTEQDILGKPRGVEAPYRVRNYTGFEVVVHSKSPTSDEPITLKLDDGSEAPWSFEH

WEKMRENLTTESNQNSVSVQLEGSGFDPVKNIRINREGEHIYNLRPKTDNVLHRLCVEVV

LGADNVKYVTLRSPFVVENATQIPVELGIYDFEDGHLLKIEKIAPGQSRPAPVGAVFEKR

LLVRPDAGFGYQWSGEHLWWRDLLKRPTKQLMCKGENGDPFYFQAHARFEKSNPLTKSYP

FMKVKLSAPVTLENLLPYDFKYRIYDKNTKKDWSNFLRKGGVSPVHVVELSHLLLLSVDM

QDTIFKASEFCIINSGSNEDYRKEARIVVKDEQGLPLQLHLQYFKIPDSGGAFKITVYSP

YVILNKTGLDLRVRAKTFLQGAKAAAGQHPLMDTSDEERPKALPFMFSFGNDDHRNRALL

KVAESEWSKPQSFDALGSASEVILNSTSKNTEIHLGVTVKSGDGKYKLTKVVTLAPRFVL

SNKLGEEILIREPSSSGYLTLKTGALQPLHFMQKSPVKQLCLCLPGVNNHWTSPFNIADI

GTSHIKIARAGQRQRLVRAEILMENSTIFIHLSIESKNWPYSMRNESDSEFTFWQANPNI

DEDGVEDRSGWKPIRYRLPPRSIMPYAWDFPAAKFREIIISSATGRERHVKLAEIGNQIP

MKFVNQDGQQKIIDINVAADGPTQTLILSNFRASKSMYKPKALSRTNTGPEAFEVKDLDT

GATFRAQLKLSGVGISLVNAQLRELAYITFRDVQLRYSDSPLIQTVSMAIKWIQIDNQLY

GGLFPMILYPSVVPKKAQEVDAHPSLHAMVSRVKDDSYGVIYVKYASILLQQMTVDLDED

FVFALLDFSNVPGASWTMVDQEGKLCDENLDIPEPSQQQSGQDIYFEVLNIQPMQIDLSF

MRTERVNAEDKTSSRNPIMFFLNVMTMAIGNVNDAPLRLNALMLDNVRVSTQVLSQNITS

HYSQEVMRQIHKILGSADFLGNPVGLFNNISSGVTDIFYEPYQGLIMSDKPEEFGLGIAK

GAASFAKKTVFGFSDSFSKFTGSLSKGLAAASLDKQFQDRRRITRARNRPKHALYGVTAG

ANSLFTSVASGVGGLARKPLEGAEQEGALGFFKGVGKGFIGLATKPAVGVLDMASNVSEG

IRNTTTVFDGSELDRTRYPRFIPNDGIVRPYNQREALGQYWLKQVDDGKYFDEQYIGHLE

LPKEDMVVMVTFARILLIRSRRLTSEWDVPLKDVQTIAKERTGVSLTLRGGANGPFIPVG

GGSERGFLYKMIGVAVEEFNRRFRGGE

>SoG_00473.T1

MAHPRVLSLPSALPPHEAEQSRRAWISRSLVCLGLCLVFIATVCLAQAGALGRHFSDEFH

TVLERRQATGAEPRQGFEDQVGISPENIASTLTQSVTKVFLRQTWDDSQQRQSSLSDSAF

VQPSSEVLDSSLGLFQTEKVNGETTMDTLFMTTGFLKPGTASSPEATDYPSRRPNIGAQA

VNEEYEEDEFMAGLGKEARTGEVEGRRLDAFSVSATLASPARPPAASFTEPIRRLPDVAE

KALVASGTSPWLKYHLSTARQPQVRNLGNGQAVTKPASLVSSASSRVSEREHLGPGSLGT

VQSEPAQLLLALLTPAIIPGEPWLPVPAKPVPGASPPDVQGPRLPSNIPSSSAGVPPQLP

ANPSPTGTITALQDNSLGSSYDQPWLLTDKSSPPAPTAGQIIPPNPGSGMPGEEPPSLPE

ASHGFQGSIVTLFPPLSSQGASDDWQSETAQHGDVQAQNPYPPDEQGGTLWNYVGCFQDH

VLRALVGAQPLDYLRGNMSPVLCVAHCEARGFPMAGVENGQECWCGKAIRDETIRLPESC

CEMPCQGNQGATCGGDWAVGVFLKARPDDAAPALMM

>SoG_00475.T1

MSYTLSQLAWAASFAVMPFAAAAYDANASDNIAIYWGQNSYNQGTGQFAQQRLAYYCANN

DIDIIPVAFMNGITPAITNFANAGDNCTTFPSNSQLLDCPQIEDDIKTCQKTYGKTILIS

LGGATYSQGGWSSAGAAQDAAQMVWDMFGPVNSASKVDRPFGSAVVDGFDFDFESGVNNL

VPFGQKLRSLMDSSAGKSFYLTAAPQCVYPDAANGPALAGAVAFDFIMVQFYNNWCGTIN

FNEGSSTQNAFNFDVWDNWAHTVSANPRVKILLGIPANAGAGGGYTSGSKLAAAIQWSKQ

YSSFGGVMMWDMSQLYANSGFLKEVKDDLEKSPTVPPTASSTTSGGGAQPTGPLVPQWGQ

CGGEGYTGPTQCQPPYKCVSGGQYWSSCQ

>SoG_00478.T1

MAHKPCPIDVKGTSFALHGENMSYHIHVDDQTQDLFLTHFGGPAPESEVFPSDAGDHFQG

WTTRANWRRREFPDLGRGDFRLPAYQVRCASGHTVSELRYESHEVLEGHKPKNKEGLPGL

FGEKGDVDTLVVRLRDKISGLRAELLYSVFPKHDAIARSARIINDGKDHVTVERLSSLSV

DFTEEELDIIELRGDWARECQRTKRKVDYGTQGFGTTTGFSSHHHNPFLCLASRQTTEAH

GDAWGFSLIWTGSFNAEVEKNSQGLTRATLGLHPAQFSWKLAPGESVVSPECVAVYSADG

VGGMSRRFHSLYRKHLVKSRFSEQPRPALLNCWEGVYFNFDAEKIYQMAEATSALGIQLM

VMDDGWFGGKKYPRVNDTAGLGDWEPIPSRFPDGLPALVDRIVQLDVKDKKMKLRFGVWI

EPEMLNPKSVLYETHPDWVLHAGDYPRTETRNQLVLNLGLREVQDFVIDTLTKLLKSADI

SYIKWDHNRAMHETPSPNASYEYMLGLYRVLETVTTKFPDVLWEGCASGGGRFDPGILQY

FPQSWTSDDTDALERLDIQFGASLVYPAASMGAHISAVGNHITGRVTSLEFRAAVAMMGG

AFGFELDPTQLSSEEKSKVPGLIALAEKVNPIVVTGDMYRLRLPEDGNWPAMMFVAPDGS

KAVLFTFQKRANVNRGFPYVRLQGLDSKARYKVGEDGPVVSGATLMNVGVQHSYRGDCDS

KVVIIERQ

>SoG_00482.T1

MSYPESFSGFQAPSAEKWQDFQKQTWKPRDFGDHDVDIKIECCGVCASDVHTLRGDWGPM

PYPLAVGHEVVGKVLRVGSKVTLARVGQRVGVGAQVYSCLECRHCKNNNETYCLQQVNAY

GAPYPGTDYVTQGGYASHARIHEYW

>SoG_00484.T1

MGDLQGRKVFKVFNQDFIVDERYTVTKELGQGAYGIVCAAVNNATNEGVAIKKITNVFSK

KILAKRALREIKLLQHFRGHRNITCLYDMDIPRPDTFNETYLYEELMECDLAAIIRSGQP

LTDAHFQSFIYQILCGLKYIHSANVLHRDLKPGNLLVNADCELKIADFGLARGFSIDPDE

NAGYMTEYVATRWYRAPEIMLSFQSYTKAIDVWSVGCILAELLGGRPFFKGRDYVDQLNQ

ILHILGTPNEETLARIGSPRAQEYVRNLPHMPKKTFPSLFPNANPDALDLLDKMLAFDPS

SRISVEEALEHPYLQIWHDASDEPVCPTTFDFAFEVLDDVGDMRRTILDEVMRFRQTVRT

VPGGGVGGAQGQQAQAGQVPMPQGGGQWTAEDPRPQEYIGQHGGLEQDLAGGLDAARR

>SoG_00495.T1

MTKSDFDHRASSHTNASTDSGHSSVTVKPSSTRRPAHPPARINTSRKRRSNSRSEESDSH

VSPTTSIAATSGVQTDPNLLRDCNMSELGNHRRQLSVLDTDRVPRIKQQPPTGGPSPQIA

PWMSSSNSSSNQPMQTSFYNDSTESLPSTLAGQSLPSPGHLRTGSRSGFVLVDKDANGTP

SFTDDRRPSVASIATTASSTGSKTSGTRGGLRKLQGFFGEEFPGRDGSDGSLPTSLASKD

HRGRSYSHSRSHRGRQFSNATDPTREASPSPARPRTPAPEVVPFLYQDNTDILRYGEAPV

REELTGPDKERYLVDNPPQVPPKTSSSSRSGHSGVHLPGHHHRHNKSNDDPRALRPSVSR

EDSAHAQYAKDKSGTSPGMFYTRSRGQSPTPSASSAPSHTLKHAQTDGAAAAHGKRGLLG

RLRRHHKDRDGPDSASKLRDLPPSSRSLQPKKSKTDYGPELSPSAFPSSTADLSDTLIQD

GRPGAQQRGATFNNKFPFSKKGRGPRPLDFGDDAIGPTDRYDKHMYHLDTNLNDMEGILT

KPPPLTPMDTSFVNSIVPDRVPEPVAATTTATVATAAATAAAAAPDPAPKSRWDAPDSWA

VRRNTEENADIAPDIDDIGSPPRPEEKKTPYCIRIFRSDGTFSTHSMPLDSSVTDVISQV

IKKTYVVDGLENYHIILKKHDLIRVLSPPERPLLMQKRLLQQVGYEEKDRIEDVGREDNS

YLCRFMFLSARESDFHSKTADLGVKPGQKLSHVDLTGRNLITIPISLYKPAPGLVGLNLS

RNLSLDVPRDFMQSCKSLREVKFRNNEARRLPASLSRATKLEILDVANNRLEQLEHAELH

NLTHLRQLVLANNRLTHLPPHFGAYRSLRSLNLSSNFLELFPPFLCGLTSLVDLDLSFNT

IANLPAEIGNLKNLEKFLITNNRLAQYVPSTFRNLTNLRELDIKFNGITTIDTIAELPKL

EVLYADHNSISSFVGKFESLRQLKLNSNPLNKFEITEPVPTLKILNLSNAQLASIDTAFA

NMTSLEHLNLNKNYFVSLPQQIGTMSQLEHFSIAHNSVGELPPQIGCLQELRFLDVRGNN

ISKLPMEIWWASKLETFNASSNVLENFPRPASRPPRVPGEDLPGPPPAQNGRSMTLGTLS

STPSSEELTDERRPSQASSTLLSVGPSPISEADRKGSVVSVYGKGGRKTSVVSRTTTQNS

VASSKNAPPPPATRKDSSVAAKYSNTFAASLRNLYLADNRLDDEVFEQLTLCTELRVLNV

SYNDEIGDVPQRSMKSWPQLVELYLSGNSLTNIPADDLDESSLLQTLYINGNKFTNLPAD

ISRARQLAVLDCGSNQLKYNISNVPYDWNWNLNPRLRYLNLSGNKRLEIKQQGWQGPGPG

SQQKEQYTDFSRLLNLRILGLMDLTLTQPSIPDQSEDRRVRTSGSLAGHLPYGMADTLGK

NEHLSTIDLVVPKFNSSETEMLLGLFDGQALSSGGSKIAKYLHENFGFHFANELRSLKSR

QNETPGDALRRAFLALNKDLVTIAIQHQEERPKTVHKGSAQPVILNKEDLNSGGVATVVY

LQGTELYVANVGDVQAMVIQNDGEHKILTRRHDPAEPMERSRIREAGGWVSRNGKLNDLL

DVSRAFGYVDLMPVVQAAPHVSNMTIREQDDIILMATKELWEYLSPGLVADIAKAERQDL

MVAAQKLRDYAMAYGATNKIMVMMISVADLKRRVERSRMHRGTSMTLYPSGVPDEAQVLP

IRKRGKGRAEILDSSLNRLDPEIPAPIGNVSIVFTDIKSSTNLWEMHESAMRSAIKLHNE

VMRRQLRRIGGYEVKTEGDAFMVSFPTATSALLWCFAVQLSLLEVSWPSEVLNSINGQPV

FDKDNNVIFKGLSVRMGIHYGDCVSETDPVTRRMDYFGPMVNKASRISAVADGGQIAVSS

DFISEIQRCLESYQDTERNGSQGSEDAFEEDSYASAIRKDLRSLTSQGFEVKEMGEKKLK

GLENPEIVYSLYPHALAGRIEHHQQQERRDDTVDRPAVLGGNSELSFDPNLIWSLWKVSL

RLEMLCSTLEEVRGPGLQPPETELLERMKTRAGEVTEGFLVNFMEHQVSRIETCISTLAM

RHMAMGSGILRELNDLRAPMSSVLDHFVKEQKKLAKYRARYGDISSDESSDSEAEDRDQG

RITEIPNNSDEGSDTAGDVFYILGLTFSHAPHDPDLWHHPGPKPPPTCNPTCEVPHSVNQ

PRQAKDGSIIRFGCCRRLLARPQQFQTGEAVDDANDLPLAESLGRIFHERVVEFAPDPGP

GETDLVFCPVEEEDRLGTAGSGLGASDVGGVEEGQGEAVRVLPEEGEPGHAGEDVGVDAD

DAGFSGGDVVELGEGVAAGVVERGEEEHGGARCRGEEDGHCERALQEGGEERLGGADGDV

VRAEGRNGGEGRDDVEDEASLFVRLEGVDDEEEDGRREEQACLGGCSGVDEGVDDEGPER

ALPEVGVEGDGEEEGGEEAAGEGREEDPEGERRRPGRVDELDGEDGGEEKDR

>SoG_00501.T1

MKLSTRSLFASLVSWSVLLLTSGAVVIRDAGNTNGMVTFDKHSIIVNGKPLLLFSGEVHP

FRIPVPGLWLDIFQKIRSMGFTAVSFYTNWALHEGEPGVFQQDGVFSIKGFIDAAKEAGI

YLIARPGPYINGEVSGGGLPGWIQRVNGPPRTRNGTDAGFLGASMNYWQFVCEVIAAAQV

TNGGPVIMVQIENEYVFDFSTGYTFDPGYMQAYIDTARQAGLVVPFSYNDVTPPQSNGIF

AYGRGEGSVDVIGQDSYPLGFACWSPEYWPPGGRGLPFPTDYARVHQKLSPKTPMSLPEY

QGGSVDGWGGYGYDNCARMVNGAAFRLFWGAAASFVTTVHNVYMIFGGTNWGNIGQPDGY

TSYDYGAAIAEDRTIFREKYRNGKLVATFLASSPEYLQADPQDNTHQYGAFTQDKALATS

QAWNKLAGTRFFIVRHSDFGSYDRTTYKMSIPTKLGNITIPQSIDGIVLNGRDSKIHVVD

YPIGNGVSIWYSSADIFTYNADGPKSVLVLYGGNDEPQELAFATKLPKPTILEGNGIKFS

TKQGRQVLSWMDAAERRVVDLGRVRVYILDRDSAQDYWRLQISSESNPPFIDTAKPSVIV

SGGYLMRSASVNGNTLSLTGDITNTTELEVIAAPAIISHLKFNGQDLAFKATPYGSLTAT

VNYQAPTLDIPDLQSLPWKYIDSLPELSAGYDDGKWTLANIKRSHNDEFQQSTPTSLSSS

DYGFNSGSVVFRGHFNATGDEKWFSIGTAGGLAFSSQLWINGQHVGSWPGDEQTRDHMDN

YTLPAFAKGHHVITVLVDHMGMDMQSHIA

>SoG_00518.T1

MGSLSKLASVSLLVLGSTAATGPCDIYSSGGTPCVAAHSTTRALYGGYSGPLYQVKRSDG

STTNISPMSAGGVANSAAQDSFCSGRNCVISIIYDQSGKGNHLTQAPPGGAGKGPAAGGY

DNLASATAAPVYVNGHKAYGVFVAPGTGYRNNAARGTAVGNEPEGLYAVLDGTHYNGGCC

FDYGNAEVSSDDTGAGHMEAIYFGNCNVWGTGSGSGPWIMADLENGLFSGYNAKNNPANP

TISHRFTTAVLKGTANLWSLRGGNANSGSLQTLYSGQRPAGYNPIKKEGAIILGIGGDNS

HGAQGTFYEGVMTSGYPSDSTENQVQANIVAARYSTSG

>SoG_00519.T1

MSAPVFRLKHPQLGTVEGTDGDVVTFRGLQYATLEHRLAELVLRLKKLEAKEEAISPISP

NPPGAFQLEMKLMQQTQPDPEYLTSSDTQALNLNIWVPKTQDGQLPQNLPVYVFIHGGGF

VSGSGNSPHYDLTRLVRLSEEKGTPIIGVTINYRLGLLGLLTSQELRRAGLMPNNQLRDQ

RAAFQWLKRHISGFGGDPENITVSGESSGAVDAGLHLLSREALFARAYLTGGSPLLMPAA

GPELHEYIYKQVVEALGLASLSPEERKSILINMPYDELAAKMPANLPYRPMLDGDVIPFA

LTHANVQDRESKVPGRQWLKGLVIGDCQFDASILAILAGFKKANIATKLPEILKAQLGDN

AATDMLLAAYDLDPQKDDEKVFEGFLKFSTDIGYYASTDSFARGWAPITYTFAFNEPNPW

PGMFQGQASHVFDVAMLFQNFQSDLPPAQAEAGKQFAADLCVFAHGKAPWVTASEGTLVY

GPSLKEPVRKVVSGGLSGEKGRRSAIIEAGEQIGKDLIAAALEAFMAAP

>SoG_00528.T1

MGFDLGHLQMYLTLLAGGGLIYTAGFVIYQVFFHPLAKYPGPFLAKITDAHQLWHGYKGD

RHLEFWRLHQKYGKVVRFGPNSLSFNSNTALKEIYGFKANVGKSEFYNAFVHPAANTHNM

RDKEAHARKRRVLSNAFSDSATKEMQRYILNNIRTFCEQIGSMAGSSSEKKGWTAPKAMS

DWTSYLAMDILGDLSFGRAFQMLEKPDNRFALGLVEAATKRHLICGTMPIVDKLKVDKVL

FPEIAAGRARYMKYSKGQLEQRTALGEETDRRDFFYYLLKARDPETGLGFTTPELWGESN

LLIIAGSDTTSTAMAATLFYLTRNEHALRKVTEEVRSRFSNVEEIVHGAALSSCTYLRAC

IDEAMRLSPSVGGILPREVLAGGINIDGERIPAGTVVGVPHYAIHHNANYYPQPFQYVPE

RWTAGAENPLSASFERRTTADEVSLASSAFCPFSIGPRGCIGKNLAYVEMTITLARTIFL

YDMRRAVGVEDVAEGRKDLEWGRQREDEMQLVDTFTSAKQGVMVEFRAAQRD

>SoG_00540.T1

MKFFSWVLSLDILGRLAAAQEWTYDNSAEEKAALQAVVEDQAEEVFKTMKKDESPEYPLI

YGRPLPIPPVKEPLEVIKNPVTGKDIWYYEIELKPFTKSVYPNLRPANLVGYDGMAPGPT

FIIPKGTESIVRFINNAQHENSVHLHGSYSRAPFDGWAEDITKPGEYKDYYYPNQQSARM

QWYHDHAMHLTAENAYMGQAGAYILTDAAEDVLGLPSGYGKYDIPLVLNSKQYNADGTLF

STVGEDQSLWGDVIEVNGQPWPFFNVEPRKYRLRFLNAAVSRSFALYFVNSNALNARLPF

QVIASDTGLLTEPVQVSDMFISMAERYEVVFDFSKYAGQTLELRNLEKVGGIGTDDDYEH

TNKVMRFTVSSTPVEDPSTVPAKLRDIALPQTTSNQVDHQFRFHRTNSEWRINGIGFADV

ANRVLAKVPRGTVEIWELENHSGGWTHPIHVHLVDFLVLDRDGRRGVMPYESAGLKDVVW

LGRGETVRVAAVYAPWDGLYMFHCHNLIHEDQDMMAAFNVTSLEDFGYNGTDFLDPMQEE

WRAKPYVLADVKARTGPFSKESLVSRVEAMAKANPYGNIEEVEKALEEYWASKKVVARDE

PQQAGPIPRYRRFVI

>SoG_00543.T1

MLLILALSSIVGLASGSIQPGFAHQPTTQPFEYCPVFYTDPPKNCRPLPDTFGKDLKNDF

EPPRAGHEVIQDAFGALEDLQDEFFDPDFGTWPSSIDWTGAVVETIVSGMLTTLTKTFDA

LPDGLENDRWTAKENLLSSYYAEIINSYYGQDILSLRGEAFDDMLWVVLGWIETIKFVRE

HANRHYFRGDVDAHRRRSRLRKALDTKPWHGKKWVPAFAHRARIFWNLASRGWDTSLCHG

GMVWDRRLSPYKNAITNELWISASISMYEFYPGDNITSPWNFDGGYGKKNPAHLAAAIEG

YKWLMSVNMRNDIGLFVDGFHISSNQGNTECDLRDEMVYTYNQGVLLTGHRGLWTVTGSA

SYLEDGHNLIQAVIKATGWSLEKTGPVESPESDARGRLPSWHGLGRAGIMEEQCDASGTC

SQDGQTFKGIFFHHLTGFCAPLDPITVDPGVTLDVEGFQKVKTAHLDACGHYLAWVRHNA

LAAMATRDSRGIFGMWWGAARYGDALVSRAKDGIDRSEPNTTDYRNEGTPRDETWGRRFR

YLPGDKGRAETGNSQASMNQQQFMLPSSRRRPQTSAPYISDEAGPRQDPNNRGRGRSVET

QMSGLALMRAYWELSQGETSSSQRGPYRHH

>SoG_00568.T1

MQSTGRTNQHHFSVQDLNGDERDEEKPWNFQELPRWKRSKRCPSLPRVPCADNETEEVER

GRTKALPALSLPKNRSTGNLALARSPDPSTATAADKSRYRMSFDFSQAANIDPESAIHRS

PLMADHEHGLGLSGLRRIRQHHPPSRAPTLPNSTASSRSPSVVALSRSTSMSAMLAANSS

FPLSTGPSSPSFTEDLSRFPSESLHSFSFAHQSEDFLHNRENVLKRSIEYMKDRKGWSVS

SMQAGLASAQARATGDIEAQHMLELLARAQLIEAGNLPNAESSLANPGPLTGPAEVSGEN

IFEKHFIPRTSSPEPLPATSNSTATQPPSKSQRSQLEGSTPRAKAEQIPKHKEVLASEDS

SRTTTGESTATADTSPPASRPTSLKRTMTDTHGITMQTKLMDTLAQPYVLGQQPIPESIA

SPTFQSFTPTAPTFPAALGPVAHGHTNRWVPAAQAIFTTEAKPPWTIIAANDLACLVFGV

TKAEVRRMGILEVVQEERRAWLERQLLRNEEDDVSEGGDAKKGTPAASVATALLGGRGGI

TAQLLSKPNSRAQPPKTPPRRAQTVHSGDPSPPKTRGSANHRSNLSRGVLLCGDVVPIQK

RNGATGSASLWVKEKRIGLIWVLEEIHEDVANVALDEEGIVQSISGAAHAIWGIDDRQKG

FDIAKLIPRIPRQGINPKEGDVDFAEIARRRYFTATNSKKTNLPCTVEQVRGKIELKVST

FPHMAGIVVVDPENLQIRSSNTAFCSALFGFEKPDGLHITTLIPDFQKILQTLTQEEGLQ

LMDGMVIPEHRFRRASAFLALKEHRPDAAAAFLQPDGLQAKHRDGSDLKIDVQMRIVKSE

KQPSIPEETVHEEVSESEEDQEGHDLFPIQRSEIVFALWVTYSRHIHSSQGHLGTASPAA

SGTSTPLHQPSPGQTVVNSPMELSSDGEESKNKKKPEPKTTPLSKQLKDAALSAAAKLTG

HSKLAPKTEEAPVAETPSDPHHKKTIDDYTILEDMGQGAYGQVKLARNKRTGKKAVLKYV

TKRRILVDTWTRDRKLGTVPLEIHVLDYLRRPEFRHPNIVEMEGFFEDDVNYYIEMVPHG

LPGMDLFDYIELRANMDESECRSIFVQVAQAIHFLHTKAMVVHRDIKDENVILDGEGNIK

LIDFGSAAYIKNGPFDVFVGTIDYAAPEVLAGKPYGGKEQDVWALGILLYTIIYKENPFY

SIDEIMDRDLRIPFTMSDESIDLIRGMLNRDVKERADINQVIEHPWCKVQV

>SoG_00584.T1

MKTTAVLAAGLCAVTGAFASDPLTPEKAIADIKTQELQRNLWNFNKIARDNGGNRAFGLP

GYEASGDYIMERVQKRFATTIDAHKQYFNHTFAQIRDISVTGPKGESVEVIALTYNPATP

PEGITAPLINTPVDDVRGSGCFEDQWTGIDATGKLALVKRGACAISDKLKLAAAHGAEGV

ILYHNAPGTPSAATLGFENLGLLPPVGTIPLSAGQAWAAQLAAGEEVVVKLYVDSVFEER

ETFNIIAETKEGDPNNVIVLGAHLDSVQAGAGVNDDGSGSSAILEIIGSFKKYKGLKNKV

RFIWWGAEESGLIGSLYYTRNLPEAEADKIRFYFNYDMIGSINPKFDVYVGDYEEADSFG

AKLLEEYLLNEGFPAKRAPFGTGSDYVGFIEIGVPSSGLHTGTSAGGYDPCYHLKCDDLE

NINWEALTGNTKAAARAAAIMANSDLKDLPRRRKTTPAKRDAKSIRAEFQRWAALSEEVE

HTHSCSHAKQEVV

>SoG_00618.T1

MRKNSRFAGYCPFCQISTVPSALPQGLKRPPAYASVTSSRTTTLLQSSVAPPPYAAAPSS

QAPVDDEKAALAQAEAEKPADDTLHFLNHDHDSIASLSLRYGVPAAALRRANNINSDHLL

LGRKTILIPGEFYKEGVSLSPQPIDGEEEELRKGKIRRFMTSCKIHDYDVAVLYLEQHGY

DLDAATEAYVSDEEWEKAHPGGQSKTRSGNGKRKSFGLFGPR

>SoG_00641.T1

MRTKYTLEKPRFLMFPSGSTLSERCIQIVRSRVFQRIIGSIVAFVVFCVCLGTVVYVNPG

GHLPDPPEQIENIIGQTHRSLNPKCKEVNNNGNRDLVQQALKKYDNLRDDKFTIAIQTYR

RPKQLEETLKKLLDGDDKIPSLLEVVIVWNDLEEKPPKDFKSRHGVNVRYRQSEHNSLNS

RLIPDPAFQTQAILLSDDDVYYQPLDLEFAFQMWRKYGRNRLTGAMARCSRVNELGHLEY

VFCSDGRDDHYNMMITNLAFSHIRFLDYYSSEDPVMQKIRAEVDRNMNCEDIAMNFVTSL

ITGEPPLLVFGDIPYVNMNPPGGISMQKGHFERRSKCLDHFSDLFGCLPLLSSQEHLGLG

SGRHRWDGYKGPA

>SoG_00658.T1

MTLLEVLSQNAPLVFVTLVLKDLAGAAIFKTFFSRSCKAFPLPRHPRYGPKDASVVLAVI

DPPESFTRTLADWVLQGPLEIILVTTRRWKALVEKHAQEAVKGVASHPPIKVLVSEIPSH

REQQVIGYRAARGKIILRSDDDVFWHHPKAISYLLAPFGPMSFADAAEEEKRLQDRNEIG

GVIGMHMPAIPPISASRLPVTPWQAISLKTLEDIIATQKRTFAADRGVICLIGRTVALRA

DVIQTEEFYRYFLNEPWIFTGKVHAGDDTAITRWVQARSRTVYQCAPEAALTTLTRTEFG

DWLGQVKRWWRSRAMHFIKLLWGPPRLPIRMAAYPYTTFQLLSTLGGVPMAALYSVSWAV

TVSNLVLAINLGKDGGLDKQSWAGRWSDIALGILALCLGLAWVYSTALWSIRLGRRFHLS

WDGTVKIMMWRLVGTFLLETWTWMTIWVDKWGGRAEGIEMAKAE

>SoG_00662.T1

MASEIDLDDILHSIKQLEESFNSRYLYHWVFFSTKPLSEDFRRLTSNATNATCIYEHTSK

AASALEPPLMGYDSPRMVFGPRNKPRSVRMSNGVKWHQRSKPWDWSSIAKADRLKDYDWF

WKIEPGAEFPQNLGFDVFRFMRDHDIAYGFTEMHIKQSKIQSPSRQVQFMMDQHPDLLHE

ELNVSWLLNHSDAWQANYLSRDLDWPLQKLSQADVDNDEGNDEDAVSAANDFTSWLAGIY

DASLTTTLEIGSLAFFRSSADQTMMKDIVKQCDETEVDIEVLDFSPIEDLPVRTLSASMF

LPKQSVVNFRKKIRRRASTPRPTTEPRSRSERASPKMEANENAYWVPSNLQQDGLAQAMK

HQFALWDIISQDFSRQGMGPALMSGNTVIDERNFAMM

>SoG_00672.T1

MVSKLALAATFGMAAAAPAAVPAPVPMPVPQQNQPLIVGGVEANSGDFPYIVSLQENNSH

FCGGSLISSTVVVTAAHCSVGFKPSEVKVRAGTTLTKRFSQTWSSGGKLVGVSSITVHPS

YNQATIDNDIAIWKLSQPVAAGNGIGFVSLPSQGSDPADGEMTTTAGWGYLREGSGSVSR

RLMKVDVPIVDRDTCAAQYKDVGPVTSSMVCAGYPEGEKDACSGDSGGPLVDESGKLVGI

VSWGSGCARANYPGVYTRVGSFIEWIAQNI

>SoG_00685.T1

MTSSLPPTAPPGAYIEEPTNPFTPEPEPEPTNPLTPEPEPQPTQAANPRDERSDEGQERS

ITETPIALSLLSLEELPSEDTRAVVSPVEPNTNPNTAATSFYLDTDDSPRFLAIVQSSKT

VHFSLNFGRRHCRFYYDAASDDVVLLNTSILSLWVAELDSQMPTDTAPQRFNEVRYSEAM

VLPPGLWRLAREEKSGALADLLLLRRRYTTELKSKVSSATGMKRGLEPGESSSRKRTPNR

NTEDTSFVFLSRPGNEASDEEQETRTLQITTGNPLDRMTAGSRLTIATGGKSPATTEAYS

ILHLRQIVGSWALSSIFSAKHGHVGQVVVKVIQNRHGSSELRVPRLAREWIKEQDVLKQL

RHPFIVEFRGSDARFFSIYMEYLPFPDLGTMRNESHLFTGTDNHAYVVLHGMASALSYLE

DKGIIHNDIKPANILFDRSRGPILIDFGLATYHDERPSYGGTPWYIGRESIAEKIRSAVS

DVYALGVTLLYLLKKLPLPDQTGPRWNILRAVEDWERGGPNVEAAVGWLSNIQNIASGLS

RSGVEGHVRTMVESDIRVRRSAKSLWQALESESGGILGAEKLESDGGAHTDS

>SoG_00691.T1

MPTLGFLKKKRTKDDPAEAQGPKSPNTPTKTGFFHRHRLSSNANHTKQQPSSSQSVESQS

PAQSQQQVNNAASLSADKEKQPSLLPQSGHLEPDIPPSAQSLSPGTIPAPGVSPFVPASS

PPQEQPATSGAQEQQQQPQSPQPDPMVLDGALSNSDSNNAAATTNADTQNHHAAAAQSSS

NVTANDLAASSTQNDSAAAGSDQNNSHAPPYLGQQAVPPPIASPGTNPNQQNSTMQSQVQ

QQQQSDAVQKVSHQQQPNQGGSLPRVTKGKYSLADFDILRTLGTGSFGRVHLVQSKHNQR

FYAVKVLKKAQVVKMKQVEHTNDERRMLSDVKHPFLITLWGTFADTKNLYMVMDFVEGGE

LFSLLRKSGRFPNPVAKFYAAEVTLALEYLHSKNIIYRDLKPENLLLDRHGHLKITDFGF

AKRVPDKTWTLCGTPDYLAPEVVSNKGYNKSVDWWSLGILIYEMLCGYTPFWDSGSPMKI

YENILKGKIKYPAYINPDAQNLLERLITADLTKRLGNLYAGSQDVKNHPWFAEVTWDRLA

RKDIDAPYTPPVKAGAGDASQFDRYPEETEQYGVTKGNDEYGYMFTEF

>SoG_00692.T1

MLTRNESLSFLKLCKVKYYNFSPIHSVQKNFSFQTGDPLGPLSKDSDGGSSIWGHVSGDP

AKRTFPAFFHPKLKHLERGTVSMATAPSASDPDTRLAGSQFIVTLGEETDFLDGKAAIFG

KVVEGFDVLEKVNDAMVDEKGYPFIDIRIKHTVILDDPYPDPPGLREPSSSPPPTDAQLK

TVRIADEAELHEDDGVDEEELERRRKERDAHASSLVLELLGDLPFAEVKPPENVLFVCKL

NPVTGDEDLELIFGRFGKILSCEVIRDQKTGDSLQYAFIEYEDKKACEAAYFKMQGVLID

DRRIHVDFSQSVSKLSDVWRDATNNKRKRNAASRGGWGGVRELEKRRQYRNVEERVDGKY

SMVHGEEDMRGRHERDPGPKDRAEGKRRSRSPRRSRDRQDRRRDDDRRRDDRRDRDRDRD

RRDGRRDYDRRDDRRDYDRQRDRERDGRRHR

>SoG_00696.T1

MDAIKNTISENFGGDAAAKLGTHQFSLEDTPELTGKVAVVTGGSEGIGYGITYTLLKHNI

SKLFILSVSKDVVDGAKGSIARELGQEAADRTIWLQCDLSDWKQTKDAAEEIKNQTDRLD

ILVNNAGRGVMTYELTDYGVDRHMALNHMGHVVLTSHLLPLMKETADSGNTVRIVNQASN

AHQAAPSDTMFEDLGELNQDLGPNAQYGRSKLANILYARYFARRVTSNGHPNVLMNATHP

GFVSTKMSKEDIHEAFPLGGYAMSVGMEPFKKNQFEGAVSSVYAATAIRGSGKYICPPAV

PEAGSNLAQDDELADRLMKLTRRVIMEKTKSESTQQGCPMDDLVLH

>SoG_00710.T1

MEANEEARAQKRSHAEMSQQDDSDSSSDDDMGPQLPSEAPKKKRRVLPHEKLYVAALPKS

ARYSRSLMHKEQLLFVTWTPITEFLITSSIDGVVKFWKKIAQGIEFVKEFRAHNGEIKSV

SASQDGRSFATAGVDDTIKIFDVVTFDLLSMLSLSFTPKCVQWVHKKGASLPVLAVSEES

QPLIHIFDGRGEREEPIHTIRGLHRKPVHLMAYNESYDCVVSADESGMVEYWRPGDDYKK

PDNVFEYKSSTNLFDFKKAKSVPASLAISPNGQKLAVMSMPDRKIRIFEFGPAKLYRTYD

ESLQAMEEMQQAGTSSYQMETVEFGRRMAQEREVESQTMRNKFNIIFDESSNFIIYGSMV

GIKVLNTFTNQVVKTYGKEENFRAVNLGIYQGQPQKKGITTVEMGASNNPLLQESETRDP

ILVATGVGKVRFYMFTNEEDFSKSTRDIQNEKPTMLGGKKDAQAKKTETGTGAVIHTTYG

DITVRLFPDAAPKAVENFVTHSKRGYYNNTIFHRVIRKFMIQGGDPLGDGTGGESIWGRE

FEDEFSSLKHDKPYTLSMANAGPNTNGSQFFITTEKTVSATPPTHGKHTIFGRAIAGLDV

VHKIENARAHKEKPEEDIKIVNIDVT

>SoG_00726.T1

MAPKRSRATEESRPKKVRRVAEEAAPDSSSEENEVDQSGSESEEEKNEEEASVTIDGTAA

EAEEEVKTFAELGIVESLCEACEALNYKRPTPIQAKSIPEALKGRDIIGLAETGSGKTAA

FALPMLQALLDKPQPLFGLVLAPTRELAAQIGQQFEALGSMISLRCAVIVGGLDMVPQAI

ALGKKPHVIVATPGRLVDHLEKTKGFSLRTLKFLVMDEADRLLDMDFGPSIDKILKFVPR

ERRTFLFSATMSSKIEALQRASLKDPIRVSISSNKYQTVSTLIQHYNFIPHGKKDTYLIY

LINEFAGKSTIIFTRTVWEAQRVAILLRSLGFGAIPLHGQLSQSARLGALNKFRAGTRDI

LVATDVAARGLDIPKVDVVLNHDLPQDSKTYIHRVGRTARAGKSGVAISLVTQYDLELYL

RIEAALGKKLTEYPAEKAEVMAFQHRVEEAQRHARVEMKAIMDRKERHGKTARKGRAGKS

SRDNMDAEEG

>SoG_00739.T1

MRSPALFIGLCALAQTALGDPTWPSEIDELEEIMYQLSSFKARKFADTVSPCNNQASGPG

RLNAAEWLRTAFHDMSTANTFFGTGGLDASLQYELDNGENTGPGHRTTLEFMAPFVSSRS

SLSDLLAMGVYHSVRSCGGPVVPIRAGRKDATSRGNTGVPQPQNSVLTFQQQFERMGFTN

EEMIQVTACGHTIGGVHAAEFPDVVPVGSTADGMSRLDSSEAVFDNKVVTEYLDGTTKNP

LVVGPAVRIDKHADFKVFNSDGNATIGALTDAEKFKDVCKVVLQKMIDVVPPSVTLTEPI

VPYKVKPVDLQLTLASGGAALSFTGYIRVKTTGLAEGSLQNIKLTYKNRSGGSDCGSSSC

TITSTVQGVGQGFDDTFSFFPIQATIPTATGISSFTVTLNFADGTEERHDNNGKEYPMQD

DVLLQAPQSCVRGSTGALTVVAAVRNDVTTGAEAAISYKMSQARSPIPSLQNATVELSKG

VCVGRYTLYSADYTIEGGLPYEATLDITSGGKSDSFKRAANLGGTCREFADPAPCNADGE

PTASESTSLAPESTTTMDEATSTSQEEAVISTVAEPAPSPYHRDTLGEYNLVSCWTEGAG

TRALSGPAMANDTMTLEMCMEFCAAFVYWGTEYGRECYCGNSLAASSGEAPFAECNMLCG

GDASQYCGAGNRLELYSTTAAQPSPTSSPTPIPDPVHRETIAGYSLVGCWTEGSGVRALA

QRSSASANMTNELCAEECAAYRYFGTQYSTECYCGSFVHHSTVSAPLVDCFMPCSGNASE

FCGGPNRLELYMNTNIVGGEPEQPAAAGDFVFLGCRTEVVNGTRALSGATTSGDQMTNEV

CAAFCTGYEFMGTQFGRECYCGQELSDKALEAEVGECGMLCSGTGLEFCGGPNRLSVYQK

KEVVEVPEGA

>SoG_00743.T1

MISAADLPPDIFDRTTLVSLAATVVLVLTAYTASLILLRRSVGPALRFLFVWHLSDALCH

FVLEGSFLYHCFFSYVPVSEVVAKGGVRALAGDWFPTPFNWLGTEGRLYGPQAGGDGFFA

QLWMVYARADKRWAGVDLGVVSLELLTVFFDGTLALYICYCIRNKDPKASIWMIVLATCE

IYGGFMTFCPEWLVGNVNLDGSNFMYLWVYLFFFNMLWVFIPLYAFWYSVRDISSAFSLR

EKQDAKKKSK

>SoG_00760.T1

MSYVDSPVSGPGFSSRPHKPVTEATRERETFKYDPFLFTDFRFNNTGNAIPSSELVSCGD

SSLTGLAQLAAYQTGTERAFISLFDTGHQYIVAEAVPGMPLAPSLPSDKCPSPLALCGVA

IPRAHGTCEHVLYQTNADPTDETTLPISLVQNLSLDDRFNTRPYCQFGKDGQFYAAVPIR

SHRGINIGSFCVMSVKQPQTWGDHCVEHLRDVSRAVMYHLESNRSRNINNTNERLNKGVR

AFIAGEDSLVNWIKDGDAAAFLTPAYGPLARPRLRAQESTNSEPVVAKAQAPGDAAETMQ

SPNSLTAANAEPWAGADRRRRRRPGDAFSGPHAGGRMPTRSHEQPTRVFSRAANIMREAF

EVDGCLFFDVSHAALRRQSMPKPAHVEETSSQPSATSSSDDPSHYATTDEPDTMCEVLGF

STAQKTSVNLGVVHDGEMGLVPKRMLATLIRRYPSGKIFNFDNVGELQSSDSSDESEFET

AARRKATADLPIATQERRKRRTLKEGILINKAFPNARSVAFIPVWDLKRERWFAAGFVYT

CTPTRVFTVDEELSFLKAFTSLAATEVLNQEVALADQAKSDALGSLSHELRSPLHGVLLG

TELLNDTDLDVFQANLTHTIETCCCTLLDTIDHLLDFSKVNSFATKRKRLLRDHRGVQKY

DNTEQFGKKQLSVDVSLDALVEEVADSMFAGYSFQNLSTKKLATLDGLMNSGGQSRARER

LDIAQIDSSMNGETPPGARVEPVSVYLSIDPSCSWDFHLQPGAIRRVVMNLLGNALKYTE

RGIICLYLTQEKASAKRARSERLVKLMVQDTGRGMGNEFLEHKIFVPFSQEDELAPGTGL

GLSLVKTIVNGMGGHITARSELGVGSTFSITLPLEPVVADAPNFDISSPKSKAFAEQAQA

LKGLRVRLFGFENKGDAQHGDGRAIIESICRDWLKVELHAAGDKSELTPDLVLWSHEALP

ESTEELSHLTKMPHVIICQDSQTARRLLKEYESAGYQGVLEFVSQPIGPRKLASSMQHAY

KRWTGLTKASPPRNTPFASLQNGDGPKANLVIGMPSPLEELTSLGLDSRLTPRNEQPPEL

PNLNASLVPAEAEKKTSLPRTYSNKDTFLLVDDNHVNLKVLAAYMAKLKLNYVTAVNGKE

ALDTYIDNPSRFTAILMDVSMPVMDGLEATRQIRAYERDNRLNRVSVLALTGLASDRTHK

EAFESGVDVFLTKPVRLQTLREELGLAT

>SoG_00762.T1

MKLDFVIASTNLKANTSLQDLRVGNKYRIGRKIGSGSFGDIYLGTNIISGEEIAIKLESV

KAKHPQLEYEARVYKSLAGGVGIPFVRWFGTECDYNAMVLDLLGPSLEDLFNFCNRKFSL

KTVLLLADQLISRIEYIHAKSFIHRDIKPDNFLMGIGKRGNQVNVIDFGLAKKYRDPKTH

FHIPYRENKNLTGTARYASINTHLGVEQSRRDDMESLGYVMLYFCRGSLPWQGLKAATKK

QKYDRIMEKKMTTPTEVLCRGFPNEFAIYLNYTRSLRFDDKPDYSYLRKIFRDLFVREGF

QYDYVFDWTVYKYQKNAQAIAQAAGQNNGEDDKDGRNASKNATGGQSGSAKPNAIPSSRR

KMLERGAGAGPDTPDTNRAIGGSDRM

>SoG_00764.T1

MELIKALSLLAAVLPVVYGAPTQAASSLHPEILAAMKRDLGLDAEQATARITREISASEV

IDQLRATAGDSFAGAWVADNGETISVAVTDKSLAEEVTAAGAKPVITANSLSKLEQAKAA

LDQLAQQPTTLSTGGGSGVAAWYIDVVSNKIVLEALKDNKAEAQELAAKVGLKESEFEVK

TVGQMPTTFATVQGGDAYYIGGGRCSVGFSVTTGFVTAGHCGTSGTRATTSGGTLLGTFA

GSVFPGSADMAFVRGSSGNTFRPYINGYGSGSLPVSGSTASPVGSSICRSGSTTGVHCGT

VRNLGVTVNYSEGSVTGLTGTSVCAEPGDSGGSFYTGAQAQGVTSGGSGNCRSGGVTYFQ

PVNEILQTYGLTLSRA

>SoG_00765.T1

MFAAVEAAPVEERAAGSARRPWVPAVGESWQIVLNAPLKIDPKKPAVTPDVNVYTIDLFD

NPAQTVAALHKLNKKVIAYFSAGTYEEWRADAKRFSKSDLGKPMDDWAGERWLNLKSKNV

RDIMAQRIKLAAEKGFDAIDPDNVDGYSNKNGLGLKKADSIDFIKFLSNEARKYGLGIGL

KNAGEIIPDVLNVVDFQVNEECVKYNECHTFSAFIKAGKAVFHIEYPKGAPRSMATSSKA

TIFSAKGAEGFTTILKTMDLDGWVEYRNGKQFTTKTGN

>SoG_00766.T1

MSLSKLITLAALAGARLAIAQASGSADAHPGLTTYKCTTSGGCVSQKTSVVIDYQYHWIH

SPSGSQASCTTSSGVDKTLCPDQATCDKNCVIDGTTNYTSSGVTTSGDTLTMYQYVQGAN

GLQNASPRLYLLAADGTNYEQLKLLGQELTFDVDLSTLPCGENGALYLSEMAANGGTKGQ

AAFGAGYCDAQCPVPTWRNGTVNYSNQGFCCNEMDILEGNSRANAFTPHPCNSAGTDCNK

GGCGLNPYSQGQKNYWGPGGTVDTSKKMTVITQFITDNGKTTGTLSQITRKYIQNGREVA

SAIQGGDTITTAQCNQWDPNAATFGSLPTMGQALGRGMTLVFSIWNDASQFMNWLDSGNN

GPCSATEGDPSLIMKNFPNTHVTFSNIRWGDIGSTTGNTGGNNGGSGTTTKKPATSTVPS

TMSTATKPATTTTAKTSSMQAATTTKPSGAQQTHWGQCGGNGYTGPSVCESPYTCKAQNE

WYSQCL

>SoG_00768.T1

MLSLSTLALALTAAAGALSQSAGCNSGGGLQSGRASINLNGQNRDYILRVPDGYDGSTPL

RLVFGFHWLGGSMNEVANGWYGLEGLSQGGAVFVAPNGLDAGWANSGGRDIAFVDALVEH

LTGNLCIDEEQIFATGFSYGGAMSHSVACSRPDVFRGVAVIAGATLSGCDGGRQPVAYLG

IHGVVDSVLPIDAGRQLRDQWLSTNGCQQQNAPEPGAGSGEHVKTTYQCSNAPVTWIAHG

GDHVGDPNNNGNYWAPGETWEFWNNA

>SoG_00770.T1

MQIYEDSSKWPELGKIDPQLAQIIQGNPSLATQSLADFPDIPTAREAVAGGPAADLSGVR

KSEIQIPVRDGSSIRAVVYEPQAKPVDGSSFAVTFHGGGWCLGTAEIEEDTSLPLVRKCG

AVVVSVDYRLAPEHPFPTPIHDSWDALRWCAENASTLGADPSKGFLVGGSSAGANISAVL

SHLARDENLSPPLTAVYLNIPAICAQELLVEKFGSEYMSYEQNANAPILDVRALKWFRSH

YKPDPKSELFSPLLWPSGHEGLPKTYIQVSGLDPLRDDGLLYARELSSAGVATKVDVYPG

VPHIFEVAIPGLDMILKAVQDREAGLRWLLEQ

>SoG_00773.T1

MSRQNPPNNAQASRKISFNVSEQYDIQDVVGEGAYGVVCSAIHKPSGQKVAIKKITPFDH

SMFCLRTLREMKLLRYFNHENIISILDIQKPRGYDSFNEVYLIQELMETDMHRVIRTQDL

SDDHCQYFIYQTLRALKAMHSANVLHRDLKPSNLLLNANCDLKVCDFGLARSAASQEDNS

GFMTEYVATRWYRAPEIMLTFKEYTKAIDVWSVGCILAEMLSGKPLFPGKDYHHQLTLIL

DVLGTPTMEDYYGIKSRRAREYIRSLPFKKKVPFRTLFPKTSDLALDLLEKLLAFNPVKR

ITVEEALKHPYLEPYHDPDDEPTAPPIPEEFFDFDKHKDNLSKEQLKQLIFQEIMR

>SoG_00775.T1

MTSYTAIKSVTQTRSPKDIRGSVSLPNGLLTELSRKQNTGLDSLGLSLFQNIQKEYSALE

KRSGEKKGPSIAADIRAFLESNDNGNGSVMDPCIRWIMLYHSVKAENDDKAAKMIIEVIL

QSWPALAFVCLGSGQSSHKDKRFYSADCGNKVDHKSTEKEIPFILAVRDGNHGVVRIMVS

ELNKLSSPVEDEEVDHPFGDASPLERFQGKKVDASDHPLSIAQKGSATALETVMELLKIQ

DLKIPYETVEWAVKRGCDKVLEAYLQFGIDASSLSKGILLALEQLKYDVEDDMSHSSLRS

STGCRLNIVESFIKRVTTLRDSTVQTIINQNLTQVWKVWRDKSDKGRAARQEQKWLLHMA

VLHQRVEFVSLFVEDYPDALSREAIIPGHPEQDGHTNKFPLWYNNWELQKPEKIGGGENP

QRYVQKPAISDPKGDRAKIRNILVTKMIHVLDMDKLPDILHRCHEPFNDLCFDISRFNSA

MFRVSDIVDSLTLQENPESRGMHKYEPTLKYVAFPPLDTMVADREVYAENKSLKSDHDEV

FKVLRWLRDRGVTKIIKLIVPDRLINPHNDVKMAKYVKQFKVEDLDWKVLDLCISIFKPV

ESSESGSTEKGEATPNLQSKRAAQSKVVEKGYLKTLHLYSSGKRAVIDHWFSKDNGVQSL

TELKELHISVIRLDNVIEYIGLQIQDLGDRKGFTAFRASELTWYPTPSVANLKKISYRLS

PPLADWLSGLVTFVKRREAESGGKYQHKPTKVAILDNGVLSIAPVSKKANSTEPSKKSGA

RLVPEEADATNGVNGSNAKSSGDKARTDDDEDNLKSRIRAGRSFVDGNLNFSPWQFPSDP

HGTQMANLICAVDPLCEIYVARVAEDTSGIKAANVARAIQWAIDEKVDIISMSFVLDRDS

DDDVMKKEIINACKAGIVLTCSTHDEGTRIPTAYPAAYKGDECKPLLVLAACDNYGRLLR

EVSPKAYDFKLRGQDIPAGVVPFVKSEENSVATATAAGLCSLILTCARVADPELQFDSQV

NPKLAPQNQPETTRINMIIKHLDRMVSETEGEDGYVLLETFGKIARPTVGDGVKHDGFDN

GRHNEPPTARKVLEEQFRMGRKAKR

>SoG_00783.T1

MKLFTFSLLCGVAAAIATDVVPGSYIVQLKPGTVQTAHHRDVRSILRKRDSTTVQSFSIG

DEFEAYVATSLTTEDAAALAAREDVLTVEPDVWYYIEDLDGTEHGVVQERALTAQSPAGT

WSLADVSHRAAGAGDYVYDDSAGAGQTVYIVDSGIRITHQEFEGRARFGFSGITGSTAYE

EDDDGHGTHVAGTVAGKTYGVAKKAQVVAVKTFRAGAGQSSWLLNSFNWAVNDIVSRNAK

ATSVINLSLGGPAGSASNAFENAMRAAWNQGIVSVSAAGNADQPASTWSPARLAETITVG

MTNVDRRRTQVIAGVFGSNYGPELDVFAPGQDIVSASWQSDTGARSTTGTSMATPLVSGL

ICYLRRLEGGLASPDAVKARLVELGSKGVITDVKGSPNVLVYNGSGR

>SoG_00785.T1

MPPARSANTCMQCRSRKVKCKSFGGPGTSCSDCERLAFGCSFAERTSSSPPQPEKRRQSS

ACLACRSRRSKCSGDVPSCKSCAERASHCVYPESRRRSGATPRKDTTSLDGSARSREEQQ

QESQGQAEQSSPLRDHATATATEDAADLRTSAASRIRSYNHFSANDAAASRAASAGQRFL

PLPSRAAGVVVSERGVRHAALPRWNAGRGAFAGDMLPHSDHAQVPQVLPWVLIVWMRRVE

DIVFRHLEEPTIVRTQALALLIQCYTRAGEFKRAYMFMAIAARFASALRLHYERVDCTPL

AQEIRRRLVWSLMIVDMSFSIGLRESETCSPDCIYLQLPCSEEQFHEDLDMGVEATTSLD

GIPEGGLLRLYMRQAIVRHDVMRLMRQVNIQTRPMPELQRLVEDLVQAHNLTEPAPYSPA

ELQRYSRSRWLVRFIFARLMWHQGMCDIYRMFLAGYREGAPSVVISGFSKAYVSHAAANC

LSHAQHILSIIDDIDKYNVELLVPPSEMGVCGYHASRLVMFLSKSPLIPLPQNISPDAAA

HAALRTLSILQRLFTQSVLLEQTLKDLADIISAHRGGAAHGPDGSSEEDQDDDGAHAAQP

VFSMAIRKHKRLSKHSALWNARFRDDADDGLGSLARLTTTNLSPPSLASQSELQQPLSFA

QATLSPTHSRSSMAKRTDQPLGGGLPNSEAEDVAAVTDDFSFEPMDFGMWDTFGSWGWGT

TAFAGSGSSEQYKRRSWCRLPTSDPTSTRRSMMVVTELVRSTDKTSWFADDARLPDQLES

VIGTSLRETYTGPMITESTSWSIATMASSTAPNILFIMADDHAAKSISCYGAGINSTPNL

DRIAQEGMRFDHCYVTNSICTPSRAAILCGTHNHVNNVLTLDSKISTRLPNVAKQLRSSP

QRYQTAMVGKWHLGEGREHEPSGFDYWSVVPGQGEYWDPQFIEPQGTHNVQGYATDIITD

KCLDWMEKRDKSRPFFAMCHHKAPHRSWEYDEKHKDLYKDPVRLPDTFTDDYKNRANAAK

VAKMRVADDLTYNDLGLVQPEGPSSRIGVKMANLWWWNDRKLPSPSEAEEVQKLRFICKN

TGDVFTFRTKEELVEFKFQRYMQRYLRTIQSIDDNVGRMLDWLDREGIADNTIVVYTSDQ

GFFLGEHGWFDKRFMYEESFQMPFMIRYPREIKPGSTCSDIICNVDFAPTFLDCAGVTIP

TYMQGVSFLPLLRGERREDWQQIAYHRYWMHNDVIHGAYSHYGVRDRRYKLIYWYNEDFG

LEGARPAAAEDKEWELFDCEEDPLELFNCYHEERYRDVVKDMTAKLERKMLEIGDQPVHT

GLKLERMK

>SoG_00792.T1

MLSYDSTVPMPGQPAPSSDLVSNGLSPQVSSSSNFSSAMSSSTAPRKYNHHHIWLVTGPA

GCGKTTVAEHLASVLSMPYIEGDSFHTAANVEKMRTGTPLTDADRWDWLTALREESMHRI

HGGSDGVVLTCSALKRKYRDVIRVAAYYDPHLLIHFIYLDATEETLLQRVAARKGHYMGA

NMVHSQFADLERPSGDEKDVINVDVSQTLDAVKVEAARRVLDLMNQDAKSE

>SoG_00803.T1

MSSSSSRRRIAHSAAKVMYMNAVYFPNTRIYNGDTPGQLNYSYINHVYYAYASVSQDGGV

FLSDEYADARAPVDGVQGGLGSLMHLKQKHPHLQVVLSVGGPAATTVFPIIASNAVLRDN

FARSALGLIEASGLDGIDIAWEYPCDAQQGYNFLALLAAVRIHLPEDRYFLTAALPANKA

VLQLIDFSIAASYLDFVNLMAYDFFGSDWSHRSGHHSQLYALSKDEPSASSGISYLVSHG

LPAKKILLGIATYGRSFLGASGPGQKFKGGGGDEGTFDYNQLPRKHCKEVVDKKHIAAQC

VGGDGGFVTYDNPDTVKAKASYCKQKGLGGLFYWNAPADSKERSRSLIAAGFGTLHSS

>SoG_00810.T1

MAITTNGASHDDKCLGTPIHANRHMRVICVGAGASGLHLIYKLKREFANFSLDVYEKNPD

IGGTWYENRYPGXFTDVSQGACDVMAHNYVYSFEPNPHWSANYASSVEIFDYFSRFADKY

GLRKYIKTEHEVVGATWVEDRACWTVRVRRPDGTVLEQECDWLINGAGILNAWRWPAIPG

IDQYEGKLLHSAAWDQGVDVTGKHVGLIGNGSSGIQILPKMQDTGTQMLTWRGWGKHRAA

AHVTTFIREPSWISPTRGMEYRQYTVEEREDFVIHPEKLRSLRKASERAIGEVFPVILNG

TPAQKQSEAFIKQQMIQKINDKELERLLIPEFPFGCRRITPGTDYLECLTKPNVTTVYGE

ITKITKNGCVMADGKEYPTDVLICATGFDTTFRPRFPLIGREGRDLAEEWESEPRSYLGL

AASGYPNYFMYLGPNCPIANGPIIFAIELQTEYMLRFMNRWQKENIRSFDARREAVDDFM

HQKDLFMERTVWTGDCLSWYKSPRDGRITAVWPGSTLHYMETLATPRFDDYAVDYNGRRF

AYLGTGLSQTELNPDLDRTYYIRNHDDGSVLSRNLMSTFNVNTAIDIGTLGQSEKTLMEM

N

>SoG_00818.T1

MAASVLSLRRDPRMLKVPPYLSLFCIVIGTAWLLLLPLDDYSRRTYISENALLPGQVHTY

FGGTDQSFAIRRNDVLEKLLKGAGLKVGRQNYTYVSAGDEYAGENIYGILQAPRGDATEA

IVLVAAWKSIDGRHNSNGVALALALARYFKRWSLWSKDIILVIPPDSTTGTQAWVDAYHD

AHDAEQVAPLPIKSGALQGAIAIDYPMTGRFESVHIVYDGTNGQLPNLDLINSIVNIARN

QMGMQTFLQNMSKHSQTYQDRLQVMLRGMLNQGIGAAAGPHSSFIPYHVDAVTLQPFGQG

WHEEMALGRLVEGSFRSLNNLLEHLHQSFFFYLLMDKDRFVSIGTYLPSAMLLAANFSIM

AIFLWVKSGQTTNISSTPATTQNTLKEKLNDAPKADPSSATSERDLIMPLGVVATCFALS

ALPLSMFNHIPSNMLDSAFKAFSIASACLPFVISNFLSTFHKPTKQHFQLTKSFSLLFLG

MALSTLATLNFSLAFIVSLLASPLTFVQPTQSVTLRFVVASLLNVVAPTTIIFTAALYAG

TSVAEVLKQAAFGWNVWGMYTPIVLWCVWWPAWLVGMINVLSVTTV

>SoG_00826.T1

MAHRCLDSDDDVNDVTSFLKESSGNIMRTPKALGKTPGRMLIPKSTVKPSTRKIRRLRDV

SSHMGANPLFQKWDGDLEESPRKSPTKARQPVRKMQSSFEESDLENVLAPSTVLRARSKR

PKAERPKDESDDSDLFGGNGTPQIKIGGVTLDVATDDHTTDDISNAEQTDASKAALAESV

ADGQTQYEQSPQPASSLQQPLSVSPSLYSRLQPMKEEAGSEVTEESATSTAGDETSEYTN

NTYSDFDSFESGGSVGNRTSAKLLFQKRPDLGADKANSLHPDNTMLSEARNMTVVEEWGE

DSETADLIQNMLDLQLRDSMLAAPASTSLQEVGRPTTPVIPRGKEAAAAQEAPSPSKQIR

IPKTPHRQSSDSFWSQDLTNDWNDKHSPTKLILPAPKKSPSKCSSSKEARKKFDTIKEQV

ARDFLAQLDERITEGQIAALAEGSGGVKLNWTKSLNTTAGRANWKRETVRTKEANGMEAD

VQYKHYASIDLADKVIDNEARLLNVIAHEFCHLANFMVSGVTNNPHGKEFKSWATKCSRA

FADRGITVTTKHTYEIDFKYIWECIGCGHEHKRHSRSIDPARHRCGKCKDELKQTRPVPR

ATTGKTSYQLFVKEQMKLVREDNPGKKQKEVMKLIGEKWKARNATVKKDEGEIKPTSVST

EDVAEKLVDLAMAD

>SoG_00827.T1

MSGHPGSGGTGGRNDYHDQYDDGYGQQGGGQAGYYQDDQHYDNGYDARGGYDNRGQGGAY

DNRGGNDGYYDESGYYNADPNNPYHQDGGYYDGHDQYQDDYYNNNGQDGYYDQYDQQGYD

RQRGRHGSEEDSETFSDFTMRSDMARAAEMDYYGRGDERYNSYGDQMGGRGYRPPSSQIS

YNGNRSSGASTPNYGMDYGNVLPAGQRSREPYPAWTSDAQIPLSKEEIEDIFLDLTAKFG

FQRDSMRNMYDHMMVLLDSRASRMSPNQALLSLHADYIGGDNANYRKWYFAAHLDLDDAI

GFANANGKAFGRRKKKGKKAKKGQDEAEALAEIERDDSLEAAEYRWKTRMNRMSQHDRVR

QIALYLLCWGEANQVRFMPECLCFIFKCADDYLNSPACQALVEPVDEFTFLNNVITPLYQ

YVRDQGYEILDGVYVRRERDHKHIVGYDDCNQLFWYPEGLDRIVLQDKSKLVDLPPADRY

LKLKEVNWKKCFFKTYKESRSWFHLLVNFNRIWVIHLTMFWFYTSHNAPSILVGPSYQQE

LNQKPEAAKQWSVVGFGGAIASLIQIIATIAEWAYVPRRWAGAQHLTKRLLFLILVFIIN

VAPGVKVFMFPTPGTVDLIIGIVHFVIAVLTFLFFSIMPLGGLFGSYLTKNSRRYVASQT

FTASWPKLTGNDMAMSYGLWLVIFGAKFGVAYPYLTLSFRDSIRYLNTMKVRCVGDALLP

GKDILCKNQPTILLIIMAITDVVFFFLDTYLWYVLINTAFSVARSFYIGSSILTPWRNIF

SRLPKRIYSKVLATTDMEIKYKPKVLISQIWNAIVISMYREHLLAIDHVQKLLYHQVPSE

QEGKRTLRAPTFFVSQEDHSFKTEFFPSYSEAERRISFFAQSLSTPIPEPLPVDNMPTFT

VMIPHYSEKILLSLREIIREDEPYSRVTLLEYLKQLHPHEWDCFVKDTKILADETAQMNG

DEKDEKDAAKTKIDDLPFYCIGFKSSAPEYTLRTRIWASLRSQTLYRTVSGFMNYSRAIK

LLYRVENPEVVQMFGGNSDKLERELERMARRKFKLMVSMQRFSKFKKEEMENAEFLLRAY

PDLQIAYLDEEPPLNEGEEPRLYSALVDGHSEIMENGMRRPKFRIQLSGNPVLGDGKSDN

QNHSLIFYRGEYIQLIDANQDNYLEECLKIRSVLAEFEEMKTDNVSPYTPGVKSEQRFPV

AILGAREYIFSENIGILGDVAAGKEQTFGTLFARTMAQIGGKLHYGHPDFLNGIFMTTRG

GVSKAQKGLHLNEDIFAGMNALLRGGRIKHCEYYQCGKGRDLGFGSILNFTTKIGTGMGE

QLLSREYYYLGTQLPLDRFLSFYYAHAGFHVNNMFIMLSLQLFMIVLLNFGALKHETIAC

DYDRKVPITDPLFPTGCANTDAIMDWIYRSVMAIFFVFFLSYVPLIVQEIMERGVWRAVL

RFLKQFFSFSPFFEVFVCRIYAISVQQDLSFGGARYIGTGRGFATARIPFGVLYSRFAGP

CIYFGIRLLMMLLFATVTCWQAALTYFWVTLLGLTISPFVYNPHQFAWNDFFIDYRDFLR

WLSRGNSRSHSSSWIAFCRLSRTRITGYKRKALGDPSAKMSADVPRAAIANIFFSEILAP

LFLVVVTVIPYLFINAQTGVHAYNNDDKKPQPTDSIVRLLIVTFAPIGINAGVLAVMFAM

ACFMGPVLSMCCKKFGSVLAAIAHGLAVVFLLVFFEAMFLLEGFNFARTMAGMIAVMALQ

RFFLKLIISVALTREFKTDQSNIAFWTGKWYSMGWHSVSQPAREFLCKITELSMFAADFV

LGHWLLFMMLPMISIPKIDMLHSMMLFWLRPRYVLFAEILLANLDIDLRSSRQIRPPIYS

MKQSKLRRRRVIRFAILYYVMLVLFLALVVGPAVAGDKIMPSMPSMLKPTGSLAGFKLVQ

PWNLNNTDTNSTMLTGTGRPGYKTESGNAKATAKVKLF

>SoG_00828.T1

MELDALQSQLGSLAQQASLNFQKVPGSAVLMRYIQSSYQNDPVRSAIELVLVIFFIRYLL

SPSYPTDKGNYVKLREDEIDELVNDWTPEPLVAEPLPIEVAEAERLPVIIGLTMEIDDRP

TGPKSKLANGRTVTNLATYNFYNFNANEQIKEKAVQTLRTYGVGPCGPPQFYGTQDVHVK

TEADIAAYLGTERCIVYAQAFSTISSVIPSFCKRGDVIVADRAANYSIRKGLEMSRSNIK

WFNHGDMDDLERVMKTVAKEQAKAKKLTRRFVVVEGLSEMSGDTTDLPHLVELKERYKFR

VMLDETWSFGVLGRTGRGLTEAQNVDPQQIDMIIGSLAGPLCAGGGFCAGAKDVVEHQRI

LSSAYTYSAALPAMLAVTASETINLLQSNPDILTQCRDNIRAMRAQLDPRSDWVVCSSAA

ENPVMLLNLKPEVVQARKLDVEDQERILVDCVDEALVNGVLITRLKIRPLEHAMKAKDGG

WEPVPALKVCVTSALSKKDIEKAGTTIRHAITKTSIRTAGSQAGMAKVERVTSRANRTRV

VDVPKEAATPTAWGDATVG

>SoG_00836.T1

MSAAVPVTNLEAEVPSVQEKLRALGLTIPPNAIISLKCQEGPNVIESPDANHAESISDTS

TVQELAKPPYVGKELQIEDDVAAARKWVDEVVMQLPRGPSAQCYRRPLAQFKIENAMVTY

DNDQCFRIRIYTPVRERGSEASIKRRPALVMYHGGGWIHGYPEVDEDLSKFFSSELDAVV

LSVDYRLAPENKFPKPFDDSYRAIKWTIDNADVYGIDKDRVAVWGCSAGGNLAAAVTLRD

TMEHEKPRICHVNLVVPFTCHPALYPDVLHAASSSVNRFGQDALGILAAQLLLGKFTLLL

SLHSPLFHRSRIFLADKYAGDMGCHDFVSVLNAKIPKNHPPAYTVVGGCDFLRDEAILYS

LRLRDADVDSQLEIVPGMPHGLNFPPTTHAARQFFINQVRMLEAAYRRVKAA

>SoG_00841.T1

MNAIFPKYLPIWLLLAMSAFSQGRPLLAEDPEDVEKRAPPALPNTDDFYIVPQNISNYAN

GAIIRSRLPPNKIAMRSKYPVNLANAYQLLYRTTDSLDQATATVLTVLIPHFADTSKILS

YQVAEDASNINCAPSYALQMESDPSLGIIGTQSELLLMEAALEKKWVVIVPDHQGPNAAM

LSNKLGGQAVLDGIRAALNSTSITGINSNEDKKPMISMWGYSGGSLVSMWAAELQPTYAP

ELSIAGVAAGGAIADILSVVSMVNGKASAGLIPAGILGLVHQYSELDTMLKDELLPAARA

NMTKANSQCAAANLADFAGVDVMSWFKTPDIVNVNKAFRQVASSNNLGQHAPKAPMLVYK

GVDDEVSHINETDTLIASYCARGSIIQYERDRTANHSTTALVGAPMALWWLDQRMQGIPN

PNTACNIYTVESSLLSIQALTALPGLILNALLDLIGKPVGLF

>SoG_00845.T1

MEDFESLIEKDGFVEHAKFGGNRYGTSKATIEEQTAKGRVVLLDIEMEGVKQIKQCGMDA

RYVFIAPPDMKALEERLRGRGTENEESIRKRLDQAQKELEYSKTPGVHHRIIVNADLEKA

YAEFEDFVYQKPQ

>SoG_00868.T1

MRFQAALLLAGAASAVADRVSYDGFKVFRIHSSDGEEDLREKVADLNTVEMTCGHTDHLD

VAVSPDDLEAFEKLGLDFETVVEDLGVDLAEEGVVESFYADSGFEGEEANKLDARQSSLP

SQSWFSAYRPWAEHRTFFNQIQAALPSNSKIVSVGNSYEGREIYALKLFGTTEGSKPVIY

FHGTVHAREWISAAVVEYLTLQLASGWLNNDALVRGFLNKYEIIIVPFVNPDGFVYSQNS

DRLWRKTRQPRSGSSCIGTDGNRNWNYQWSLPGGASTSPCSETYKGAAAGDTPEIRALTA

FTNTFRSQGIKLFIDWHSFGQYILLPYGYNCQARAPNHAQQMAVAGTAASRIGAVSNTRW

TYGPSCSTLYATTGSSPDYMSGAMGAEYSWTIELRPGPDNGSSGFVLPASQIVASGVEQW

EGIKYVLSTI

>SoG_00874.T1

MSYALQTSFMGESLLNGFNWYDGKDLSNGFVSYKNYDDAKALGLWSLDPSNDVVRLGVDS

THTYGLDQGRPSVRLESKESWDHGLFIADFLHMPPSQCGLWPAFWMYGSDWPYGGELDII

EGANTAHRNIISAHTADGCYQDETSLFTGEQRNTDCAIGSQNIGCGFNPPASDSSSYGDS

FNAAGGGVYAVQWNSDRIKVWHFPRGSIPADIEEKKPEPSAWGLPQAVFGGQKCDVDSYF

KNMSIVININFCGDYGDATWASSDTCSSYAPTCDGYVANNPQAFTNAHWDIKYIDAYQLS

GNSSSTTFAPITTPAAKIVKPEAFPPAPVNPDSINTFSYLGCFTSANGFSTFKTGENTQE

MNLGRCVESCKSAGHMFSGVYGSQCYCADTLDAATTWAVGDQTRCSTPCPGNSTQLCGGG

DPNARLFTVYAGVRDEEKPLALPMAGVPSTVTVVVCPTATGTDNIQTQVIVPTPAPSNNV

TFVRPVARPAVTPPMVAGAVAKLPDWRLGALSSLVVISALVLF

>SoG_00888.T1

MARPSSSRGKSPQPPRNRGKSPQPSPMEDQPAPVAPAASLAPATAATPSLKKKKSKSKLN

AASSYKSDGVEDNDVFLLPSSDYWIMLGMTVLAAAVRVFRIYQPSSVVFDEVHFGGFATK

YIKGKFFMDVHPPLAKMLIALTGWLAGFDGSFDFKEIGKDYIEPGVPYVAMRMFPAICGI

LLAPFMFLTLKVLGCRTMTAMMGSGLIIFENGLLTQARLILLDSPLVAATAFTALSFNCF

TNQHELGPSKAFSPVWWFWLVMTGLGLGITASIKWVGLFTIAWVGALTVLQLWVLLGDTR

NVTLRIWSKHLMARAFALIIIPVTFYLAMFAIHFVCLVNPGDGDGFMSSEFQATLNTKAM

KDVPADVILGSRVSIRHVNTQGGYLHSHPLMYPTGSKQQQITLYPHKDDNNVWLLENQTQ

PLGPDGEPINGTLAWDDREPTYIKDGMILRLYHIPTDRRLHSHDVRPPITEAEWQNEVSA

YGFKDFPGDANDYFRVEIVKKKSHGAVAKERIRTIETKFRLVHTMTGCVLFSHKVKLPDW

ASEQQEVTCAKGGTLPNSLWYIEYNEHPKLTGDVEKVNYRKLGFFGKFIELHKVMWKTNA

GLTDSHAWDSRPESWPILRRGINFWGRNHTQIYLIGNPIIWWASTLTVAIWVGFKAIAVL

RWQRNCNDYANTTLKRFDYELGTSVLGWALHYFPFYLMKRQLFLHHYFPALYFAIIALCQ

LFDYATARVPVLSQAQKRVANRVATVTFLALSVSAFFLFSPLAYGNQWTKGECKRLKVLG

TWDFDCNTFFDSAWLRRHGHMSTESSRATSKQTTRGIYDQYAEMSNAPTISSSVTPDAAK

PVQPNNQAKEAAAQSPKGELPADISGAPAVPQGARVLRKEEKLEYRDQDGNLLNEEQVKE

LEGKVEFQTRYETRTRVVDEAGNELEMPEGGWPADYSPVAPPHPDVQGVDKETVRAEGEQ

EVPKEVDASKDGEKEAEKSQAKPASEGKKATGHEEL

>SoG_00892.T1

MPPPPVPSKSIPQDPKVTSGDNGAEVNGTRLFKELHLGLFEIGKPLGKGKFGRVYLARHR

SSGFICALKVLNKDEIRREGAEMHVRREIEVHSNLRHPGIVGFHGWFHDSRRIFLILDYL

PGGELYRVLRREGRFSERRAAKCAAQGAQSLEYLHSKNIMHRDIKPENILIGLHDELKVA

DFGYSVHAPSNRRDTLCGTLDYLPPEMLTSSKAQYTKAVDQWTLGVLTYEFLTGEAPFED

SPAMTHRRIAKGDMTPLPASLSKEAKDFVHSLLVMDATKRMPLSSVMLHPWIVRNCKNK

>SoG_00897.T1

MKATQVLIALAAVAYAAPVAEQTKVLAVDEDFPLAELEAYFAHHLSGDDSEVSALQARQF

GSNTYNQLTDGTPCRPVTVIYARGTTQAGNVGDPAAVGPVFFNNLASRIGLSNMAVQGVA

YAANVVGYLQGGDPNGSRTMADLITRAASQCPNTKIVISGYSQGAQLVHNAAGMLSAAVT

NRVTAVLTKAVTFGDPKQRQAFGTIPSSRTRVFCRSGDNICDGGIIVTPAHSQYQQDAPA

AADWVAARV

>SoG_00899.T1

MTTLNAPVAVQAPPTSIFEGGHHVSSFSSQQTSLSSGQPFATTISSRETPSHSAPAASKT

RSSTTHSRWRPRYHIMPSTGWLNDPCAPGYDRIHDVYHVGFQWNPNAPEWGDIAWGSATS

KDMLFWETESTPSIDPSSKLEGEAGVFTGCMSPATAKPGTSTAFYTSARILPIHHTLPYN

RGAEAICMANSADAGRTWQRASKTTVVQGPPAHLQVTGWRDPFVAAWPSLGRLLGDKADT

DLFGIVSGGIRGEGPAVFLYALHPSNLATWSFLSVLTPALPSSDLCRSPWTPDYGVNWEV

VNFLSIPDPDDPDSSYEVLVVGVEGRGKSPATRADRLTHTEFRRDHRQMWICGKLAKTPG

GVQMQFRLGGALDHGALYAANSFHDPKTAQQVVFGWIVEEDLPAELKELQGWAGSLSIPR

VIKLQRIHRVVRALKSDLKTISSIEVQRESGRPDDEALPLNQSSETYSITTLSAVPDPRL

QNLRYAKKTLGPGAYRTPSGTFDSLDLPVDCASWEADMSFSLGSDEVDAIGFVITHSHDH

HPDSELYTTVSFNPKQETLRVERVNSTHVEGICVADEEAPHTLFSTLPEKSNPASENAIT

QEPLRLHVFFDVSSLELFANERTAITARVYPDTTDAMSIQPFVTLKEGHQWSGKLLECVA

WELRTSRA

>SoG_00901.T1

MAVSDYLLSHAELGDITGITRGDDIVQFRGIPFASIPGRFRQSVLKQGRLSSQTFDARQP

GSICPQLQLPFPTYWTSPPPEDYPTLNVPSQDEFNCLNLSITAPRKALESKQQVPVLVFI

HGGAFMGGSQSIQLVGREIYDATTLVRASIARGQAIVVVTINYRVGLLGFLTSDRLSERS

RKLGEAVGNYGLHDQRQALEWVSRFIDGFGGAADKVTIHGTSAGGASCHYQVNFPNRKFQ

GAILSSGTSIGLAARPMPWHQERLSYVVSQLPGLDTEAPGLLELLDALPLDKLLPATPPS

LYSPLVDGEWVVGAGLKPVLERMADAGESLPALMIGATEYERDLTLLLLSDMSKPLPHPP

RPETEVTEVARDVLCGSAMVPQLAAPDSPFNMSEICQAYSLSSKKSPLESFEDLAGLMAD

TAFRAPPIYSASIIKQHQQKNSNSATSPVLVYEIQATNPYVAWPLSYGRANHGVNDVMLF

NPAEDQVPADRLDNWRSAVAQVQGAWLDFCHGKMPWAPFEGLEGVGGVEGPVYVIPDGGE

GRLCRNLEEVDGLKTATRWRALMTAASQTQQ

>SoG_00919.T1

MESFGVHHPADNPNSTCYGHFKSDGGTYEIWDKYNGNIHQLWSVRTTRRVGGTITTRNHF

RAFEAAGLKLGRQGEMIIGIEGQWGSGQATITAGVRPTTTVRESATPTTRTQVTTRSGTC

TASLDIS

>SoG_00930.T1

MLRSTTAAVAALLLAPLAVAQDIGKDSCVKGLFMVVARGTTERPGTGITGAVADRIKERI

PDSGIVGLDYPATYTDPLYTQSVAEGTTELAAVLTNYTQNCPGKVAVFGYSQGAHVVGNV

FCGTNLPLFGNEAPLPKKLVDDHIIAITLFGDPTHNVSAPYDLGTSKKDGIFNRANITAC

ENYPIKSYCDTGDEVCDSGTDDNVHGQYVVRYGTQVVEDVLKAWNKATNGDVNTDGGAQF

TRPPRNSTQTAGATPSATLSTTMTSSQTRAPAASGTSGSGSGSGSGSGSGSGSGGNGGGD

KPGAAAGLSAFGSMVMGAPLVLIALWNVL

>SoG_00936.T1

MESFEAELAKAASERDLLGAVAVVIDEKEVKADRFRSGSFVYRHAAGHQLLDGNSPSLDP

DCTISLASAGHFLTIIAALQLVERGILTLDEPISKHLPEIDKCLVIEKEGAEMRQRPPRR

SITLRNLITHTSGMGTVDTYERVGCKDEVQPLDFPDDAHPLVKDGFTHLFFEPGEGFEYG

WSIYAVQLLVQRLGGKETARQYIDENIFAPLGMTASTYLPAQTPHVWERRLQMVRRQVGA

DGKASLVASDKDTSGLTCSISDLARLFGDLISPGCKLLRQQEHRDLLFAPQLTPGSQAHQ

SMLAETTNHSFLFPLEHGVSHEVSWARTSPLAVNWTAAGLLVEGDGLPGTGIPKGTVSFE

GQPNVIWTMNRDRKRAMLFGHQLLPGYDVKAHNLAVRFLSEAWKTFG

>SoG_00950.T1

MVSAKLLSFLVLSVAANALRYVGVDWSSLLVEERAGISYTTQSGAKQPLERILVDSGVNT

VRQRVWVNPSNGDYNLDYNIKLAKRAQAAGLQVYIDLHLSDTWADPSHQTIPSGWPTDIE

NLSWRLYNYTLSVSNRLAAEGIRPNIISVGNEIRGGLLWPTGKYDKLHNIARLLHSGAWG

IKDSNLNPKPKIMLHLDNGWDWSVQEWFYSSVLAQGPLSASDFDMMGVSFYPFYGDQATI

SNLQAALRNMANRWGKELVVAETNWPTQCPSPQYPFPSDVRNIPFSADGQATYIQRVAAA

VSSVNGGAGLFYWEPAWMNNQALGSSCSSNTMFTWPGRALSSMSVFSRIR

>SoG_00955.T1

MKFPFATAVVAATALLPTQACLLDSEVEAERQHQLHGTPIRRQQFDKRQSSKTFPIGTGD

RFNSGSITPLGLGVSDRNLESVLNPGEVQSALQGLAKAYPRVQLFTPPQHTYQNASLHGA

FIGERPRVFIMSGIHARERGGPDNVIYLIADLLRAETQGTGLRYGNKTYTHADVEKALSV

GIVVLPLVNPDGVAHDQAMSNCWRKNRNPKSAAENSEVSIGIDLNRNFDFLWDYEKAFSP

LAETMNAASNDPHSEIFHGVAPESEPETQAVLWTAKQFNELSWFLDLHSYGGDILYSWGD

DNVQHTKPYENFANASYDGTRGFLGDDPAEASFKEYMEEKDFRAELNVARKMADAMDDAG

LIKYTPLESVLLYPTSGASTDYMRGMYYRGTCGAKKLKSLTVEFGRPTAATAGGCPFYPT

KEEYHDSMRSVDTGLMEFLLNAAGPDGEPQWRIC

>SoG_00957.T1

MRVSTLIAVLPMAMAAPQVKRTSPAPLIIPRGVDLIEGKYIVKMNDDTVSASIQGAIDKI

NADADFTFSQQFNGFVASLSGDELTNLRYDPNVLYIEQDAKVTMWATQEDAPWGLARISS

KSPGGSTYTFDESAGEGTCAYVVDTGIDVSHPDFNGRATWVANFVDNSDRDGQGHGTHVA

GTIGSTTYGVAKKTKLFAVKVLDDNGEGSNSGIIAGLEFVVKDAAQQNCPKGVVVNMSLG

GGYSSAVNAAAASISRAGLFLAVAAGNSHADSKNFSPASEASACTVGATEKDDSLASYSN

FGSPVDVLAPGTDIESTWTGGGVKTISGTSMASPHVAGIAAYFLGQGQSASGLCEYIASN

SLKGVVSGVPNDTKNLLINNGGRNNTTTLPDRPARPARPGRPNRPARPF

>SoG_00962.T1

MAKLSSWLLMSLYGWSAVFAQKATVTVAPDGSGDYTAIGSAISYAQANSIPTVTVLAGTY

TETLSVATAAVTIIGQTSSGASSKRDGGDFSIANKVVITNGGGAGRPLTIPANAKGITWR

NINFINSNSGSSLGVVSLQGSANAFYGCSLVAAGPYGLTGSIASGIIANSYIEAFDKPFS

AYSSLYVFGSTVTLLDTNGVLVYTKGYTINGILYNSTTVLDSCQVAQKDGTSPKNVYLGA

PNSAGSVALYRSTSLPAFIAAGGAHPTTLTTASTQYLEYGTTGPGSFANNAAQRAGLVTL

VTDSSALAPYDISSFYSTVYPWVAVPNTNWIDSSVKSAIVASNAANGQTGSSSASSSSSS

SSTSSSATSSSYSTILSTPSSAASSDLSSSVSTANSISSVSTVLTPSNVATTTPSPVVAS

NCLPSSMPSTALIVGPSGNSCAQYNSITAAIAALPADSTTQYIYILAGTYTEQPSITRVG

PVIMRGETSNPGSSSANRVTIQNSSGRLSSTGGSSSTASFLANKYETQLMAFYNINFINT

FSAQNNYIALAVYAKGQKVAFYGCSMISSQGTLYLDYGNVYFSGGRIEGSTDFVWGQGAG

YIHNSIIVTDGTITGQTIAAQKYQNQYGGSQLVFNLCAIVPQSTSLPQQGTYLGRDYSAN

AQVAFVNSYLDAHIAPAGWLINSASTFTGSFAEANNTGPGAATASRAASVKILADSSPYT

AKNVLGDDSWLDAAAIAPFSGWPASVYFVATTPSALTTPTTLATVTTTPSSSTTSSSSAV

GATLTVAPTPASGQYGTVASAVAALPADGKPYTIYILAGTYTEQFSITRRGMVTLRGETS

FANDFTGNQVLIQFSNGVSTSAGQNEQTPVINWKNTNGDGLSLYNLNFTNTFPQTWNYAA

LAADFFGTNMAAYGCAFKGFQDSLLVNQGVQVFSNSYIEGSVDFIWGYSRAYFHQCYVAS

NTAGACITASNRPNSAWAGGFVFDKSLITYTSSYGSNMGTTYLGRPWSQYAVAVYMNSYL

DQHIASAGWNTWATNDPRTSNIMFGEFNNTGPGNWTNSRANFATSLSESQASAYSLSNFI

GSTSWLDMTAYNLAPSYSLSKDSSSSTPPSGGAAWTHPASGTEPPAGAVLVSVDGAIASS

FKSLTSALASLPADGSTQIIFMYPGTYNEQVPAINRNGPVMIIGYTESSPGKTYTANKVT

ITQARGLSVSPPPAGHSNAETATVSTASTKIAMYNINMINSDNLDGATPSYVTLAASIYG

NKIAFYGCSFIGWQDTLLTGATAGYQYYESSYIEGAIDFIWGYSKAYFKGCTVGAKRRSS

AITAQSRASTSAVGGYIFDQCLFTAAPTATVDLTGAVYLGRPYSQFALVVIKNSYLDKTI

QPAGWKAWSTSDPRTGGVTFAEYNNDGPGNWGNNTAARQAVGFATLLTSDSYPLTTVMDS

TDWIDMTYWDQIQTPQPSSAPPPVANPTAYDGTTPPAGAFIVSKDPIEGKTTYSTVQAAL

DALPASSHITAIVFIYPGTYDERLVISKSGTTIFLGYSTAPQDYTKNQVIITHNLGVDTQ

TAASNSDSATVYATGNYFQAVNINFANTYGTAQNFATLGFGVKSSKYAGLYGCQVYGNQD

ALLVSGYLFASNSYIEGNIDMIWGSGAGYFLNSTIATNRDGIDITASKRGTSTTAAGFVF

DQCTITAANGASFSSVSLGRPWNQFARVAYIKSYLGPMIKSSGWDQWTKTDPRTDGVLFG

EAGNTGPGASAANRASFSKQLCPPDAAQFQLGTFFSSTTWINGTFVYATPFVASSLPDAC

ATPITTPSTTTPTTTLTPLSLSTVTVYVTKVSTLKETLFTTTTAAPQTLTVKSTSTVDIG

TTITPDQVTVTTTQKSTTTEVQTIKQPDVLVTKTTAQTVDVGKTITPPPVTETSWQTIPS

TTVLVNTVFGKDTTITTTVSETSYVTKTPKPSTSTTTVVNVVSNTRTLTPKAVTISSTTT

QTKGTGGVTTVTAKATTSFDTVFVTSTKVSKKTTTITCIPTNKKRGLEAAPEPTAMDYNE

FARRQASLADKTVTVTVSSVFTTVVKTSIATIAGTTETDNLIITKTTGKTITLKAVTTTS

TTVSMATKFQTTTLPGATQTTVETEMKTIGKTTTLKPSTTVITSQTTSVKTAKSTITQLG

NTARTTATDVRTITSVLPPVVAIVTKSRVITEVKTITLPANTVTSWATAKTTLKPSSTVT

QQATVFKTTTVKVPQTSTSWATKTSKGAKTCTAS

>SoG_00987.T1

MELFKFLSLAAAVLPVVYGAPTSTMSELHPEILAAMKRDLGLDAEQATARVQREINAADV

IAQMKDTVGEHFAGGWVADDGKVINVAVTDKALAGKVEAAGATPVIVSNSLSKLEEAKKA

LDELSRAPATLAARSTGGVASWYIDVESNKVVIEALADSTEQAKALASKVGLNEGEFEVH

TVSDMPTTYATIQGGDAYLIVGSGRCSIGFSVEGGFVTAGHCGSEGDRTTTQSGESLGSF

AGSVFPGSADMAWVRLSGSHTTTGYINGYGSARLPVSGSTVAPVGSSICRSGSTTGVHCG

QVRRFGATVNYADGAVTGLTGTSVCAERGDSGGAFYAGSQAQGVTSGGNGDCTSGGTTYF

QPVNEILSTYGLTLVTA

>SoG_00994.T1

MAEPQIPKTHKAVVFSNPGTTATQVVDVETPSPGEGEVLIRLSHSGVCHSDYGFIANAWN

HMPEATPVGQIGGHEGVGEVVMLGPGVRTHKIGDRVGVKWITSCCLTCESCLDGNEGRCA

LRKVSGYRNAGTFQQYLTSDASYVTPIPDEITSAAAAPLLCGGVTVYVALQKADVKPGNW

VVLSGAGGGLGHLAVQYAKAMGLQVLGIDHGSKKDFCSRLGADAFLDFTQFSDEGLALEV

KNVTGGGAHAVLVNNASTKAYDQSIDFLRYGGTMVCVGVPEGVAQPITKAVPHHLITNKL

TIKGTVVGNRKDALDCIKFAAQKSVEPQIQLQPMSKLTENLPLLAIVDVVSEAPIHSGRK

VRIIGIGAGASGLLLAYKVKYNFYDSDVEIQLYEKNADLGGTWLENRYPGCACDCPAHTY

TWSFEPKTDWSQTFATSGEIHQYFIDFAAKHSLNQCIQFNSAVSAASWVAEDGKWEVEIT

TENGKVQDSCDILINASGILNAWRWPAIPGLETFQGPKLHTANWDQNLDLSGKKVGLIGN

GSSGIQILPAILPQVHSVVNFIREPTWISPIMMPGFEAKRFTEAEKLEFRDNPTKHVDFR

KTIERNGNSVFPLFLSGSEAQAQAAQQFRQGMKDAIPDVFLKDKLVPDWDVGCRRLTPGV

NYLSSLTSPKSSVVYGEIAKVGSKGPITEDGREHPVEVLICATGFDTSFRPRFNLRGVNG

VSLTEKWQHEPAAYLGLAADGFPNYFMFLGPNSPVGNGPVLIGIEAQADYMMKYVKKFRE

DNVNCITDISCIGRSFMPQEAAVKEFQQHKDTWMKRSVWDKDCRSWYKNPEGKVTAVWTG

SVPHYVETLESPRFEDFEWTYLDNRNRWSFLGNGFSQRESVGADLGWYIRQEDDAIPLGK

RERFVFTPEDNGVKPEKAVPSSAANGATPLEAIARL

>SoG_01000.T1

MTSTTNERYSTFSPEFEAFLKSQATSEGNALASLPLPEVRKAVEGVETAVWTQASDNITW

DDCTISGSDGSSIPLRVYQLKASKGSPKRVALMIHGGGWSLGGVYSDSWAIRALITHLDM

VVVTVGYRLAPEDPFPKPFNDCLDTLRWILNGPEKLPSSGEKIVMAGFSAGGNLAAAITQ

WACTNGLADRIAAQVLISPATCHYKQLVNASKGGTVELGSIVNASSPFLSGEMMKSYWDN

YDPDGGEDVRVSPLLAEDFSKLPPTYVQVSGADPLRDEGIAYAAQVQAAGIKVGLEIYPG

MPHAYQSDIVAMPGRERTMVDMVRWLGGTLGHKVSL

>SoG_01002.T1

MGEFIPQPQYKIEEQWHSQPAPIRVICVGAGAAGLLVAYKMQKRFTNYDLVCYDKNPAIG

GTWFENRYPGVACDVPAHVYTYPFDQNPDWSSFYACGPEIRQYFEDFADKHGLRKYVKLN

TRVISATWDEKRGLYEVELEANGQKATDFCHMLINGTGFLNDWKWPNIEGLKDFSGTLLH

SANWDTSVDCTGKKVGLIGTGSSAIQLLPQVQKVAQHVIAFMRSVTWISPPVAGEMLNAS

KNASNDKKEATRPDAQYFYTEEEKKRFRDDPQVLLEYRKNIEVSFNAMAEIYLKGSDVSK

QTEQALRAEMERRIGPGHEELKKKLIPSWAPGCRRLTPGDGYLEALIEPNVTPVHKEIQK

VVPEGVVDSSGELHKLDILICATGFNLAFAPPFTVKGVHGVDMKTEFGDEPKVYLGLAVP

KFPNYFVVNGVRGNWAAGTVLVTHEVQVDYILNCIERAQKEGIKALEVKSQPVEELYQHI

GKSAASIIEALPQSSQVTNLSFSWYKNNIVGGKVWVWGGSGLHYNKTVKVVRWDHYDLRY

KTNNMWSFLGNGLTEAQVKKDISRLSPYIRTEDTPFEI

>SoG_01013.T1

MASEAVASPSGATRPQLSGMRSSSYLSDHQQYRPPHRSPEGFHGIDTVVEDRRVGSHASS

SPKPILPFKGIPSDDNPPTIVDSGVNHSYSHPNCAPAAGEQTDKLVATLFYKTKARAAAS

NGSAAPTGDSNEHLPSNMAPTDDMAAFPLEPPTHENDSLDHLYGNYISPLCITSFLHLMS

TFPMPHEGDEPHSSHRCLDNPENPRVVELTLSPAPRADYLSLQDLRKHEMIYRFEQEWNV

DVVLQKETVLRRHPRLVVFDMDSTLITQEVIDLLAEHIKDPPDLAARVAEITERAMRGEL

DFEASFRSRLALLKGLPDTTFNALRPVLDVTKGVPELLRALKRLGVKTAVLSGGFLPLTS

WLAGELGIDYAHANEVVVEDGKLTGEAKGIIVGKERKQELLIEIAKKENVDLKQSVAVGD

GANDLLMLGTAGLGVAWNAKPRVQMEASARLNGESLLDLLYLFGLTAEEIDVLTAA

>SoG_01017.T1

MDSSIPSSTLVTRGELDAASAISAAEPSGTIAPYTTALNGVNQEMNMYLKRVLWWTLGAL

ALFILVIRIAELLFSKLRLINAMSQPRTKQTYWKIAQWSWMPWLKKYIIYAPLWKKRHNR

EIRLSSVLHFGTLPSRLHFIILFVYLGCNFAYMFLLNWHNENRYAFCAELRGRSGTLALV

NMVPLIILAGRNNPLIGLLQISFDTYNLLHRWMGRIVVLEVVIHTIAWAIPMVAAAGWEG

IREKIATDLFIGSGTVGTVVMVIIAIWSISPIRHAFYETFLNAHIFLAAVAIVCTYVHCV

ASFHPGGLPQASWTIAIACLWFADRLARVIRMMYANWGSKGSTTAYCEAMPGDATRVTLT

LPKYLKIQPGTHAYLRFWGANAWESHPFSIAWVQHDFDDTLPITEKDPLAAYERAGKTTV

SFIIGAQTGMTRKLFNKASAQGTITMRAAFEGPYAGHHSLDSYGHVVLFAGSTGITHQLS

YIRHLLDGYNNGTVATRRITLVWIIRSHDSLEWVRPYMDTILRIPYRKDVLRIQVYITRP

RNPRDVSSASTTVTMFPGRPNIPLLLAKEVQEQVGAMCVTVCGAGALADDVRGAVRAVQG

ETSVSFIEESFSW

>SoG_01018.T1

MNDPVPDPASEAPPSDVDVPSCQECRRRKLKCSQAKKEAKRRPVKLLTFLSAVESPCVYD

LKKNRPGIKAGAVGNLSRRVDALETTVEKLVHDQTLDQSSSTSSRAPTIPALNPSAAQDL

VTILATLASEVKALKSNTQQQQQQQQPTPHPLPVETHRDYTVVSPATSQATASQASSRVH

YSSRYDGVSSSYWVGGQPRKRRRVDSCGNASLDLAVHLEEDELDALSSLPPADLLEDIID

TYFDLIQPWIPMLHETQFRRQVADRSQRWQLGIVLHAMVVAATRFTTSSRSRLSAREIDT

YTARSRRFVVLNSLDNLSVEGLQALIIIAYDDVGNGEASRAWSIIGSMTRTVDYLQLSLE

PEHREREPLLKPLPMLDDPRNWTEEEQRRRVFWNIFCLDRFCSVTTGWNTSLTSSDVQRR

LPADGGLWHKEEGVTTPYLGVWDRSTAKIGNSIAFLPGNYLSPEQTKDPSSLAQASSSSG

GSGGAEQRKGDPDMTTVGAFAYRIEATESLSRITTYFLQQKVDFTDRQQVSDWLTRFKEL

DLRLVQQPTLVRMDPNLTLAHVTHNTSMILLHQRIAYPDRSWSNSFKIPSFCSAEACQPR

HDTVRLLRLHQRESAPG

>SoG_01022.T1

MLRSVITIAVTAAASLVSAVTIAEINGDAYISPLNGTKVTDVRGLVTAKNGQGIFLRSTE

PDGDERTSESVFVFNSAVRGQVNVGDIITLDATVEEFRSNALHGYLTELTKPANVKVVSS

GNEVRPLVLGSDTLPPPTELFSSLDEGGIFGVPNDVARVSARNPRLEPRRYGMDFWESLV

GELVTVRDVVQVSRPNQFGDVWVRAGSLRATGVNSHGGLTMLDGDSNPEAVTIGTPCDGT

KNPSDTKMGDSLGDVTGIVYNAFGLYRILPLTAVKPVRSAPTDHPAVSFNSSGDCRGITV

GSYNAENLNPRSAHLPGVVRQIIEKMRTPDVVFLQEVQDGSGPANDGVVSGNATLAALSA

GIEEGSGVVYEWVEVEPEDNKDGGQPGGNIRQAFLYRPDIVELYRPNQGGPNDRTEVVTD

ASRPNWPMLSYNPGRIEPAAEAWASSRKPLVAQWKPVKGTGKTFFTVNVHFGSKGGSSTV

HGDPRPPINKGVEKREVQAEITADFIASILKIDPAARVIAAGDFNEFTQVRPLQTFMSRG

KMLDLDEAAGIAPEERYTYLFDMNSQQLDHVFVTEGVVRHDRGPGGPKGPPRGPPKGPRG

PAGSPKGDGRLPLMEHLHLNTWQNNDGQVSDHDPSVAKLNLCGCAA

>SoG_01024.T1

MDTLGALGGEWEPSERSFLNLLKGALEYSVSTEARATKIANDVLHFYMDNTPEGEVGGML

FELWGVWIEVAYRIPHGHEWHRCMALAVDLIRQDQRASDRGSKASREHDELPELFMSVRE

RWEYKPFEGAAHDHGEWKNLNSFMSLVVGSGFVQQIHLPLWEISLALEEPSSKELALMDY

RVWVAAEWIINCREVLKGEMRKPDEELTVESREILGIGTLCDDSIKPRSWQRWEFWGRQL

AEILEESDDIGLEEETKKHVEKALEVVRSTXPLAATLTEHTERHNFQAFCCPAAGPPAAP

QTWNRPRSRFVELIATSAQALASLLVPASSSIPSPPCFFSLLYFGAIYARLTHAGTSLIS

NTIIDSPCDRAQPGASEGAILTTSLRTPSDINVCRGLSTSNMPELSPRGVRFSAGEEDMR

VERLKDPRLSTVALESEDSSPESNGDPNANDQDDDLGGMDRYVDASRHMGSISSLTPSAS

LNNVNKSQRSNGGDDGTSFNGAWPQRPLGPTRTPSSTYNPGSSRKPAPPQSAPSFVDSMR

SSSKTRPRQSESSRFRAQERAYIQKLRQDYSGEYFASYETTNGNDSDSEGETPSSEGPFD

DRYDEETIMFYGNETLQPTEEDLQNKENRERLEWHGMLAAVLMGNVVTQEKQRLHGSSDK

EVGKTAHKTELWVGVRAKVCGRRLPVQKRMVEDARREVDRMLDDIINFSVKGAREVGKPP

YEQVKDIVGKIEKCESLYPSWKSLKAEHKAADSAPFNEAYDSILSWYNTNEMINTELAIL

KKWVGNDEMDFSRTKQRSPAVHGISSDETSFLDRLMKEDGLRSLYNDDEKSTQKGMLRPI

SAIISKAKQTLIKNSGPFEKRHLPPYLEELLTLISFPSRLIEEITRTRLAYARRVKESAQ

QNPLMQDQMISQFQLLLRFAIRIKAEYLAIAAPEPGWELPPLIDESFDQVILDALKYFFK

MLNWKLSGNRNTFKEAELLFEQWGFANEIGGHLLRGEIEVAEQFSSLTFKALNRLSQAFE

RELQAKPKETAADMSKRYKACLDSVRVRHRMLQRFSRNLSEQYEHCCDFSINFPLDEMQR

IYDQLLTTGHFHVTTGVFEQDGVYILASPGLRERLDDIQALMAVTSKETFDPELSDQYLL

ILRPEEPLTWCGPSISLPLREQNIDLKRGQVRLCATGSTALPISRRIFLDAVDTHIDLIQ

EHRSNIHKVNTRLTETRRVAYKLSNTFMDSVEIVRKQAEGKDCQELVQTCFVFATEFGQR

SLQSMDTNRRQMNNIKLTKLALDWISFIVDDCVASDRKTFRWAVQALEFAMRMTMGRHIL

ALGEDDYALMRDKVAGCMGLLISHFDIMGARSTLAAQAEKEKLEAMVGHFKRMDHNGMLL

DDAEASKSITEQRMDSIELVDGFRREKEAERQALGRVLEANTEADRSLAYLSSSATNITM

RWQQGHYVGGGTFGSVYAAMNLDSGHLMAVKEIRLQDPKLIPTVAEQIREEMGVLEVLDH

PNVVSYYGIEVHRDRVYIFMEFCSGGSLASLLEHGRIEDEQVIMVYALQLLEGLAYLHES

GIAHRDIKPENVLLDHNGIIKYVDFGAAKVIARQGRTLVSDLTSTKPNKSMTGTPMYMSP

EVIKGENPGHPGSVDIWSLGCVILEMATGRRPWASLDNEWAIMYNIAQGNPPQLPTHDQL

SASGIDFLKKCFARDPRKRSSAVELLQHDWIKAIRSQVVEPATPSDSSSSVQSTPMGSWP

SSRGSAGPDGFY

>SoG_01027.T1

MLFLPLAAVAGLVVPAVDAAPRGVGSKQFDTRDSLARHLQDHESGHYRERRTPHHNLPAA

PGGPPAASLNDLDDCPTAQVIVSTTTVDVTVYATAPAATGEPCDEDAAAPSANGAGGNSA

ANPATGTGADPAGGNNAPVDTNGDGKPDAVPEDVDGDGVDELVPEDEAGQDATAPGANDG

DGTPDEEDPCDEDPNVPGANGAANQDAANTTTGGNGANGAAGDEAEVDTNGDGKTDAVPE

DVDGDGVDELVPADEAADATNPAGSSTTPGDKDGDGVPAAEDPCDEDPDVPTPGGAGNNA

EVDTNGDGKPDAVPEDVDGDGVGELVPEDEATNATTPGKGGKGGKKPPVDTNGDGKPDAV

PEDVNGDGIDEIVPTNGAAGGNDVTTPTKGGKGGKKPPVDTNGDGAPDAVPEDVDGDGTD

ELVPENGATGGKTPGAGTNGNGSATPGAATPAAPSTAPGTPKPGDKDGDGTADAEDPCPE

DPNVPTPNGGAGGGDPATPGSGTTGNNGNGSTAGGAGSGSSKPPVDTNGDGTPDAVPEDV

DGDGTDELVPGTGAAGGKAPGAGTNGSGSTAPGSTTPGSGTAPGSGTAPGSGTPGSGTAP

GSTTPGSTTPGSGTAPSNGNGSTTPGSGTPKPGDKDGDNVPDAEDPCPEDATVPGTDGNP

GNGSNGGKKPPVDTNGDGVPDAVPEDVNGDGVDELVPENGATGGKTPGATTPGATTSGAN

TPGTGKTPGASTPGATTPGSGKTPGATTPGAGSTGDKDGDKVPDTEDVDPNGALTPKPGD

KDGDNVPDAEDPCPEDPTVPGTDGTKTPDGTPGIDTNNDGKADVVPGDVDGDGREEIVPA

NGATTPGSGTGATPEEDCETAVGAGADPAAAATDSFVTKTRAPVAAAPTETDCEEEVVPA

AMPVETTKPMKADKEEDDSSSGGLLGGNGLLGGLLGENGLLGGLLGKRDEPIYATTIPII

DNTKYHPTTTSDPVIIDTSMTPAVYPTTECDIDSPPVPTATMCVHEVKDGKTKEDTAYCG

VNGKPVGSYFLAEFFEQRSGEPITEEGCYQFCDSVMEATAGCQSYRFYHNDAGAPRCALY

GSSVTKAMYDLDADREDTWWDVSCGSPSAENWHKSMPGHY

>SoG_01031.T1

MSPVSGTTRGDVPTTKRRRTDSGDESPTRRGSDGGQGRRRDGDGTRDTSTTPRRSPSRGQ

SRHRSRSGASSRSSSRRRPRSYSDSGSRSQSSDRTRTDTSPSPPPNARQPTPPRPSLKPD

YRPRLALHGHTKPVSQVRISPDGRFIASSSADATIKIWDAATGQHMDTLVGHMAGVSCVA

WGPDSNTLASGSDDKAIRLWDRVTGRPKTTARRTVHGQEMPPLRGHHNYIHCLAFSPKGN

ILASGSYDEAVFLWDVRAGRLMRSLPAHSDPVSGIDFCRDGTLVASCSTDGLIRIWDTST

GQCLRTLVHEDNPAVANVCFSPNGRYVLAFNLDNCIRLWDYVAGSVKKTYQGHQNEKFAV

GGCFAILDGQPFIAAASEDGGVVLWDVVSKAVLQRVDGHKEGVCFWVDVHGDTMVTAGQD

CTIRVYRHNRVVEEITEKNGAVNGESKRLELPIRQEDVKMEDV

>SoG_01042.T1

MKKKPHHFLAQPSPPTLILSSPVRFNQLPRLSLQLLAVFFNLIPPLYCSVQLCAAVRIGL

NGFSLPLVRATQLALSSSPPDLNSPRQIFLPAMASDAQPTRDIKNHLLFEIATEVAHRVG

GIYSVIKSKAPVTTAEYGDRYTLIGPLNHQSAAVEVEEMEPTNPAMAATMQAMKDRGVGM

MYGRWLIEGAPRVLLFDTKTAYGYMNEWKADLWNVASIPSPPNDDETNEAVVFGYLVAWF

LGEFVCHEKSKAVIAHFHEWLAGVALPLTKKRRIDVTTIFTTHATLLGRYLCAGSVDFYN

NLQWFDVDAEAGKRGIYHRYCIERAAAHACDVFTTVSHITAFESEHLLKRKPDGVLPNGL

NVTKFSAVHEFQNLHQVSKEKIHDFVRGHFYGHYDFDPDNTLYLFTAGRYEFRNKGVDMF

IESLARLNHRLKAAGSKTTVVAFVIMPAQTTSLTVEALKGQAVIKSLRDTTNIIEQSIGR

RIFERSIKWHEGDPMPDEKELISAQDRVLLRRRLFAMKRHGLPPIVTHNMVNDSEDPVLN

QIRRVQLFNHPSDRVKVVFHPEFLNSANPVLPLDYDDFVRGCHMGVFPSYYEPWGYTPAE

CTVMGVPSITTNLSGFGCYMEELIENSSDYGIYIVDRRTKGVDDSVNQLTSFMYDFCAKS

RRQRINQRNRTERLSDLLDWKRMGMEYVKARQLALRRAYPDSFGGDEEEDYIPGVDQKIS

RPFSVPGSPRDRTGMMTPGDFASLQESHEGLSTEDYIAWKLPEEEDPDEYPFPLTLRTKQ

PGGNSPLNTVPLNGNGTGN

>SoG_01044.T1

MAEQLILKGTLEGHNGWVTSLATSMENPNMLLSASRDKTLIIWNLTRDESQYGYPKRSLH

GHSHIVSDCVISSDGAYALSASWDKTLRLWELASGTTTRRFVGHNSDVLSVSFSADNRQI

VSGSRDRTIKLWNTLGDCKYTISDKGHTEWVSCVRFSPNPQNPVIVSSGWDKLVKVWELN

TCKLQTDHIGHTGYINTVTISPDGSLCASGGKDGTTMLWDLNESKHLYSLNANDEIHALV

FSPNRYWLCAATASSIIIFDLEKKSKVDELKPEFPAAGKKSREPECVSLAWSADGQTLFA

GYTDNIIRAWGVMSRAKSREEENCARLEDGGKDEERSGLW

>SoG_01045.T1

MRISSATLIAFAAGAIGAPSSTRVKSRQAPGACSSPVTLDASTNVWESYKLHANSFYRGE

VEEAAAQMSGAAAEAALRVADIGTFLWLDNIQNIDRLEPALEGVPCDEILGVVIYNLPGR

DCAALASNGELKVGEVNRYKTEYIDVIVDIIKSYPNQAFALVIEPDSLPNLVTNIDEKNC

QDSADGYHEGTAYAIEKLNLPNVVMYLDAGHGGWLGWNDNLKPGAEELAMAYKNAGSPSQ

LRGFATNIAGWNQWDMVPGEFASDPDAQYNAAQNEKLYVELFGAALASAGMPNNAIVDTG

RSGNPGGRLEWGDWCNVVDAGFGPRPAASPDSSAPLLDAFVWVKPGGESDGTSDTSAVRY

DAFCGKADAFQPSPEAGQWNQAYFEMLIENANPAI

>SoG_01050.T1

MDGKRHPSSFQQLEKLGEGTYATVFKGRNRQTGELVALKEIHLDSEEGTPSTAIREISLM

KELKHENIVALHDVIHTENKLMLVFEYMDGDLKRYMDTNGERGALKPATIKSFMYQLLKG

IDFCHQNRVLHRDLKPQNLLINSKGLLKLGDFGLARAFGIPVNTFSNEVVTLWYRAPDVL

LGSRTYNTSIDIWSAGCIMAEMYTGRPLFPGTTNEDQIIRIFRIMGTPTERTWPGITQFP

EYKPTFQMYATQDLRSILHAIDHTGIDLLQRMLQLRPELRISAADALQHPWFSDIHMAQQ

AQQQQAQAMQARAYQQQGVPAQNFDGY

>SoG_01053.T1

MRFQACIMFLSGAAAILIPPPTGRFPVSVAVKSLTDESRVDPYSPPNQREKRQVLVSLYL

PIDKDSTSSEKRIIPYMTPVVAQDYGLQAAAAGLPNNTFAAFELETSVVISKSPCGPKYS

KGKHSAVPLVLFSPGGGQSRLLYATMARSLASEGYAVVTIDHPYDASVVEFPDGKVVRAA

NISDEDIPALTKMTQVRAEDVSFVLDQLQSPKIFSKELASHNVAIDFNRVLMYGHSQGGA

TAALAMLTDSRIKGGVNLDGRFFGAVMDRGLSRPFALLGRPNHGAQDKTWPEVFPKLRQS

RFEMEVANTTHGSFTDFPILIDSLGLPEAARGAVAALIGAVSAKHMDKTVKSVMVAFSEL

VFKQNKAPEVLKKGQTVVSGLTVLNSDFRN

>SoG_01062.T1

MGSGNHTDFDAYTAAKDYNESLSTYLFIILGAVSIAVIVWKVTDVITKYTRTVACLDNDR

QRYFSIPSPNLAFIKRHVLYAPVLSKRHNREIQLSSAVNVGTLPTRFQLLFLTAYLATNV

VFCVITIPFASDYEAVTTAVRNRAGALATANMIPLFILAGRNNPLITLLGISFDTYNLIH

RWLGRIVVFEAIVHTLAHFAKGRWSMGSLQAAVTVPGLMWGFIATCAFVAISFQAWSPIR

HAFYESFKLLHIALAALAIAGLWLHLHLFELRQIKYLYAVVAVWAIDRAWRLFRILRQSI

GQFQKTGIVEALPGNACRVTLTLARPWNSQPGQHAYLYMPKLSFWQSHPFSVAWVDGADD

IDRDKLASHRQDLAAMQKMRISFIIRARTGFTNKLYQKALAAPGGKLETMCFVEGPYGAR

HPLNSYGSLVLFAGGVGITHHVPYVRDLVAGFAEGTVATRKILLVWTIQSPEHLEWIRPW

MTEILGMERRRDVLRIMLFVSQPRSTKEIHSPSSTVQMFPGRPNIPTILSMEQEQQVGAM

AVSVCGPGTLSDEVRLAVRARQSRSHIDFIEEAFSW

>SoG_01072.T1

MEPKDDDHMQSERFSGSSDDADSDLEELQGDIAKFDESVRQFLASHHGATGEVGFPGRGG

PRGRGARGPRKAAKPRGDITARLAKVNQAFLSGDYDRALDLAFEVIRINAETHQAWTALS

STFAEIGDTSKALSAMVYAAHLRPKDVAGWVRCASYALDTIVEEGAENLQTARLCYSAAI

RADPQNLEARIGKASVCHRQGHLGTAVAEYDKVLKRRPGDIDLVRRLAEACMDVRHAETA

VPMAICAYRRYFTLKMGGGSEGKPAAGLWEAVHELKRLARWLVGRSDETVWDKWQDDDRE

WDFDNARQFDLTQDAGLPTPNDRLDYSAMLPPDLRARLAIYRFKLDQPDEALHHLAILQS

DEQVMASFTDEFPYLSFDLACSLAQFGHNEHAKGFLESLRALPGEADPAVLLQLGRCNLG

LGDSSAAEECFLAAIDADEMAIEPRIELANMYESAKEGEEAFILAAEAMAIREAQGQSSD

LSDIGPFQGLSHGEEAGAHRRVGSSSAQQGLMFGLHLRQHPMIPRRYRPKRLADPTKRRQ

EEQAHAVKLAHQYQVVRDLKRQIAHGREELIPTWMAASQDLIDDFRSLKRFYTWDRYLHF

LGSKSNDGRSAEAGADNDLSRLYQRLARNVAPVEEKSNQLDAAFVFEAHQSISSDQWLEL

FLDYAIALAVDYRQDEAYRVCESARDSTVFQSAAHDFSIHVAWTVCAILTSDEGKAITLA

RHLMRDMTSSDGSRLYALLANLCQSPISWYTSGPAQKFILRQIKMIDATPVSRRQLTGDD

YSPTNIPEPELDLCLLMLYGHILFTSTSYTFALGYFLRARSIDPNNALVNLSLGLAYVHY

GLKRQSANRQYILLQGQSFLTKYAKIEGRGAGQLGTAESNYNIGRLFQLLGISYFAIEYY

SKALRLFHGSSKNSDVAKMAMVNYINMLIVVKNKALALVLTKQNVVL

>SoG_01076.T1

MAALALGFTTSWGFATTAPAVEPEPGTGSTAPLRPFRLDQVRLGEGILQEKRDRMKSFLK

EYDEKRFLILFNQQAGRPNPEGLAVPGGWEDGGLLSGHWAGHFMTALSQAYADQGEKIYK

DKLEWMVGELAACQKAITDRMNNGENGGNPGNGSETEKVGRVPGRFGNALQLTAEGGGAE

YITLPQEAINQVNDFTMATWIRLNQEQAWSRVFDFGHDTTTYMFLTSRSGSGSGAPRFSI

TTSGSGGEQMIDGDAPLPTGEWVHLAVTMASGSGKLYVNGKLVGTNNAMTLSPRSLDNPG

NRWIGRSQYGDPLLNGTIDEFHIFNRALDDDEISSLQESAAGSTGGGNIAWYRFDEENGT

LVKDSSPNKRDAGIVGGKDGDDDASWVPTHPGYLGALPEDTVLRLGPPRWAVYGGDLKTN

TWAPWYTQHKIMRGLLDTYYNTNNKQALEVVTKMADWAHLALTVGDKNQPDYKGNLTRAE

LNLMWDLYIAGENGGVNEVFPEIYALTGDERHLETAKFHDNRQSLFGASVADKDILVVRP

QDNPGPRRPDRLHANSHVPQFVGYLRIFEHSSQDEEYFSAAKNFYGWVVPDRHFASGGVG

GNYPGSNDNTELFQNRGNIANAIGDSGAETCTTYNLIKLGRNLFLHEQNATYMDDYERGL

YNMIAGSRADTNTTSDPQVTYFQPLTPGSRRDYGNTGTCCGGTGLESHTKYQETVYFRSA

DDSTLWVNLYVPSTLDWQERGFTVKQETKFPREGVAKMTISGGGKLKLNLRVPGWIRRGF

EVTINGRPFGGEEKKKRKIHGGSYYLTIDRDWKDGDAVEIKMPLSIRVERALDRPDTQAL

MWGPILLQTLGTPSGGSGSFHELSLYRHLKRDGDYAAAAVNQTGTTAKGDPLFTIADPGV

GGGGAGLLTARPYYISDGEAVSSYFRRVESEVVFGGLGSGVANRKRNDGLPSYNVPVQGI

TSPGADGPTFLDLVWDGAPFQTHEDFLRAVRTVADGFVSAGVYSVEERDKIIETARKAEA

ELEPVKKKGGRSRDL

>SoG_01082.T1

MPETNNGNISGDKTVPKISDEDFRILVQDYNDTDDISTLGLCLPGLFEAVVKKYPHNIAV

ISGTTKLDYEEVNTYANRLAYALVHDWHVAVGYAIGVALDRSADMVVAILAVLKSGAAYI

PIDPSFPEERTMQTITDATPKFIMGSDETLSRLRLGKDMCLSFDELSHSFHDMAGSERGQ

NLLTNRPKSSDLAYVIYTSGSTGRPKGVEANHGALCNLLLSMQRMPGCSPEDCLLAVATI

SVDMSILDLLLPLSSGATVVVPQTWQLRDTSALLDLMEQHKVTMIQATPSFFQMLLNGGW

DNNPRLSKVLTAGEPVSRRQLDSLLHNADNVFNGYGPTEATVYSSVGRVLPGDKDIVIGQ

PLANFKLYVLQSDDAGNLSPAPMGGLGEIYIGGVGVNRGYRNNPELTRRNYLECNPFHPG

RLYRSGDLARFLGPGKLSIVGRTDSQVKVRGYRIELGDVSAAITEHPLVAEAHVLLRDGQ

LVAYYTRRSFSCSRSESLDSDLRAWMADRRPKYMMPAYFVAMEKFPMTLNGKIDKKALPN

PAASKPSSRQSTITDTFAGIEDEVRQAWASVLGHENFGVCDNFFEVGGDSVRVPRLKKEL

ERRLNQPMPAAKLFEHYTIRSLSAYLEIGKAQNGVLPAPNPVPPNALKQGGRDDIAIVSM

ACRLPGGITTPEEYWKLLSDGVDGITDVPKGRWDAEAIYSADPDSTGKSYCQRGGFISDT

EEGVHSFDAGLFGIAPKEAQTLSPEQHIMLETCWEVFERAGYHLPQLRGSQTGVFIGQTN

ISAYNTESRPLDGLDGYAVTGSIGASTSGRVSYFFGLEGPSMTIDTACSSSLVSTHLACA

ALRQGDCEMAVAGGVALLLTPALHVEFSRLKGISSDGLCRAFDDQTDGTGFSEGSTAVLL

KRLSDAQRDGDTIHAVIKGSAVNHGGRAAASLTTPSGPAQVRLIRRALAESGLKPEDIDY

VDAHGTATKLGDPIEATALAEVFQGRSPSLPPLHVGSVKSNIGHTQAAAGLASVIKVILA

LKHECIPRTLHVSKPTSLVDWDASRMALVLQNTPWAPRDDASRCAGVSSFGISGTNAHLI

LAEAPQPARPVDVSAVTDSQAIVIPIITSAQSAKALKEQIQRLYEYISSMEGFSTDRQLF

INVASSLATCRTQLQKRLILLPKDGKHLLELLESCIMNGIEKPSPSWNIRSNIGIGAHGG

SGQAGQGLAFMFTGQGSQQLDMGKSLYQQQPVFRNKLDETVALFKTLGGPLLHVIWAELL

KRTDYAQAAIFALQVSLFRLWQSWGIEPTAVVGHSIGEIAAAHVAGVFGLEDACKLVTAR

GHGMQQLTCLSTSLPGKMASLECDGDQATSIISALGLGGCVDVAGLNTPMQTVVSGDAEA

VKKLVTYVSEDLHRKAKVLDVSQAFHSSHMDEMLPSFRETAASLRYHEPRLRFISTVTGR

AIEPGELATPDYWVNQVRQPVRFADAFKVLYEEEGIRTFVELGPRPVLSGMAAASVNIAK

TLLDSQAIFLPSLNTGKQDDSVAIGAALAELHVRNYDADWAGYFSSLSCQRINLPTYAFQ

RQCYYRPGRVVAGQNIGVQNRRSNGVDASGSNKSAVQLNDMRNNSNKMEFHINWERLAEG

NLPTLKRGSHWGLWSPTNGTVSWETKVWKALLQAGLKLSHVDSFERAESLDGLLCLWDSE

SESDVPGDALRLTSDALLQLQGAAEQSFRKPIIWITRQAVGADLFDDHVHESRYAQSPNG

TANGKSNGCANGHTNGHDAVECNLQSNGHKEVQGPNSFNGYTKINGSSAHGQQSLAAAPL

WGLMRTARSEHPEVRLRLIDLEVVNEDFDALASAIGISSQEPECAVRQGSLLVPRMQKAD

MVTKGSHESRNSGPVSAARDGAVLITGGLGGIGKQVARWLLENYDIRNFILVSRRGLKTP

GAKAAVAELENLGASVIVSSCDVSDYQSLQHVFSMCEEGKISVRGIVHAAGLVDNGVLSA

MTRQRCATVFAPKVAGAWNLHRLTEQMDMDFFVMFSSIAGTLGSMGLGNYAAANTFLDAL

ASHRLAHNLPATSVAYGVWEGDGMVEGLTGRTTLTHLNKFGLGQLKPAEGLELLGKAISS

GHALTVAANLDLQQLERYLTNDDGNRSGIPPFYQRLFERTGKGAGTGEENGHGPTHDKQT

WSIQKELRGADPSRHADIVLNLVRDAVVQALGFNSSEEVDVDVPLKDLGFDSLTSVLIRN

HLASLTSLTTLSAADITWKYPNLRALSQFLLSQLRAHDDVADGKSIHGEGAVNTDTLWPG

DVDLNPSDAVSMLGMRAITSGCVDASFTFGNASRMQGPPQSVFITGATGFVGAYVLRDVL

ERGVEAYCLLRARDADHARDRLISALVTYNLWKPSFAGLLHPIVGDASRAFFGLERETFE

YLANSIDAICHCSALVDWMRPLPEYIGPNVVSAHEVLRLASSGRGKAVHVVSTLATLPMH

LGYHVPEDDREYGYSTSKFIAERMVAAARWRGAKASVYRIPFVTASTQGQFRQDRGDFLH

NMIVGALEMGCFPALDADLSVVHPVDYLSQTITSVMFDDRDRIGRDFDFVNDKDPVAIND

FFKLMDAVNGGDENKASLTPFEEWRERALGYAMKHPRSSLARISTIIEGLKDERAAVGMW

QGLPTGGNVFGGEVYPVPKVDAELVRNYFYKIQESRV

>SoG_01087.T1

MDVINFISKEPGLPDVEITGADPNCPLQNGIELYFNGPWLHEHLHGLRALLDKYGAFSVG

EMPGAETDDQVAKIAAAGRKELHTNSQFDVRVDIDNSPEGKYAREEPWKLPELKEIINRW

QTFAHRVGGWNSVFIENHDQARSVSRFTKHRPEHRELAAKMLATLLCTLGGTLFIYQGQE

IGTANLPKEWGIEEYKDIESSNYYREALEAAEGDENALASVWRELRLKARGHARAPVEVS

TCSSDFLGEELPQQEDEAKISSSQWDVTGNAGFSTTTPCMRVNEDTVRCNVKAQHIQKDS

VLNYWRQALKVRKEHKRTLVYGHFDM

>SoG_01088.T1

MDIASTNAAQEPPANPQEWWKEAAVYQIYPASFKDSNGDGLGDIRGIIEKPDYIKELGVD

AAWLCPRCGRAHPRALHDRDMRCILDLVVKNSSDQDKWFQESRSSLDNPYRDWYIWKKPR

FDAAGNRKPTNNWAASFGGEGFHDCPPLKPISTEIGFRPGPAWTYDEATGEYYLYLFAPE

QPDLN

>SoG_01091.T1

MLNNKITLVAAAATLAQQVSGLSAHHHLHNLEKRALAAEARVAELEARDVVVETVWEYVT

KTVHWSPGQPPAAPTDNAVVVVQDVPPAPAPQPAPTTLAKVVVAQQPAASAPPSNPAPAP

APAAAASAAAPGQNDKGTSSPPSGAPFSGKRGIAYNDANLANTFGGSCKSCSWGYNWASR

RDDFDSKYNYVPMLWGNSPQWTTNWVQNANDMIAQGAKALFSFNEPDIASQANMSPAQAA

QEFQTHMNGFAGKALIGAPAVSNSGQPNQGLDWLKQFIQACNGQCKFDFCNVHWYSEPQY

ADTLFTHLQQAHEVCGGKPIWLTEFAPLGTGPAVDAFITSVVPKLDKIDYLDAYSYFMVA

PDHLMSGAGLSSSGQAYATV

>SoG_01096.T1

MGIVELVLQNAGKAGLVVVAFGFLYFIYSCIYNLYFHPLAKYPGPFMAKISPIYSIWGLF

RGRWPFDVHQLHLKYGPILRTMPNELTFVDPEAWKDIYGHRQGHPQFHKDPIHVGSVQDI

PGSTTLTMADDANHSRQRRTLAHAFSQKALLEQESIIRGYVDLFVKKLQPFAENGTPANM

CDWFNFTTFDIIGDMAFGEPFGCLREGKFHAWVSLITETIKAGAFEQATRRMFTTGSFAQ

TFLCKFIPESLRRKRFQHLELSKEKCLK

>SoG_01103.T1

MLSKPSLPSTFGILAAASLAAAGPCDIYASDGAPCVAAHSTTRALYSSFTGSLYQLRRNS

DGATTDVAPLSAGGVANAATHDSFCSGATCVISTIYDQSGNGNHLTAAPPGGAASGPNPG

GYDDVAAPLGAPVTLNGKKAYGVFVSPGTGYRNNNAKGTATGDEPEGMYAVLDGTHYNGE

CCFDYGNAETNSHDTGNGHMEAIYFGDNSYWGSGSGSGPWVMADLENGLFSGENVHNNAN

DPTVTYRFLTAVVKGEPNHWAIRGGNAVSGDLSTYYDGVRPNADGYNPMSKEGAIILGIG

GDNSNGAQGTFYEGVMTSGYPSDSADDQVQADVVAAGYAVGALSTGDKPSVGSSVTLRVT

TPGYTTRYLAHSDATVNTQVINSSSSSAAQKSGTWVVRAGLANDQCFSFESADTPGSFIR

HYGFKLQLNANDGSKQFAEDATFCTQPGLSGQGTTSIRAWEYPARYFRHYYNVGYAASQG

GPQDFDNTYSFANDVSWIIGSGF

>SoG_01104.T1

MTMFKLTTTACILTLATLTHGSPVETRQALPFNVQPIAEFNEPWSMAFLPDDRLLVTEKS

GVLILIDPSSKAKTKVTGVPTVKYAGQGGLGDVALHPHYETNNLVYLSYVEAAGTGDESG

AAVARARLDMDENGGAALQDTEVVWRQSPKVNSGGHFSHRILFSKDGDDNNATLWLTSGD

RQMFDPAQDMSANLGKILRLNDDGTIPEGNPFADQGGVAAQIWALGVRNPLGFDFDADGN

LWEIEMGPKGGDEFNLIKKGGNYGWPVVSEGDHYDGKPIPNHDTRPEFLPPKVFWNPVIS

PASMIVYKGDMFPQWKGSALISGLGAQGLVRVEMSGDTAREAERISMGKRMRCVREDADG

GLWALEDGAGGRLLKLTPS

>SoG_01108.T1

MSFSHGSEGGTLALPSPTHAHHMDVSSAVRTLRRSMSRSPSTKYLSRSNSNSSDGTTSQQ

ISPQSPCRRFGLTPHRRASPSAHAQTAPPAAIYPPATNASQSPSFTPLRPSGRLSLRSVK

SAKTSTPQRPLSRLRASPKSPLRRALNTNSDSGNSSLSTVFSAIAPTTGQENVASFARSP

PRRRNLEKPSRHSLHLDVSGVSQHAFLKALDANKSPSMTSTGALKRSDATMNLDQPNQGS

PVAKRRSLHGFSTLVHGDDFIAASPGTVPAAPSFEIHEDANTEYELTTAAEAPRSDFAQS

ASAGITVSQRSSSLRKTTLQQRSLSRRSGERHLAQLAAEQASPARSRPRLSSDHFLPPTV

PRDSPFNSKIPLPSTSNHSIEASKPHPLSKTLTTSSSGGSLEEEQPPFYASFKSFEAPKP

HPFSKSLPVGATRPSAPKPLHERVVATPNGSGQLWLGAFNSTGLVSKVNRNLEEEAAKKI

IPPDTPCKKHSNPFATYPPKFSSVLKKRENNRKSFGGIPSTPFSSTPGPAPDTFGKPGKG

LSIFQRGNALRNSRRGSILGLDDEDRKLLGETSDSGCTGDSDVPPTPTRNTLTPSGSNLS

QQSLESPSANRTQAAPMSAVRPPISRESTSKSTPGMFEVPADADGVPTGENVPASNGLGQ

ATVPSMLPPTSFGNSRAQQGCRVPAPILSVTTFSHSVASDAKTTLANPASPVDGRRTPQT

PQESYLPWDTSRLSISQAANGVPAESMPPPMTPTGGRDLRSSTSIFVTPANARTQNLDID

SSLTGRFDKVEYIGKGEFSSVFRVSVTNHRQNALDALSGTEAPPSSAQGSIFAVKKSRYP

FQGPKDREAKLREVRALQALTHAEHVVQYVDSWEHNYHLYIQTEFCEEGTLDKFLGNVGR

GGRLDDFRIFKMLQDLCLGLKEIHDAGFMHLDMKPANILITFEGALKIGDFGLAQPVTSS

EGVDVEGDREYMAPEMLKGNVSGSADIFSLGLITLEAAANVVLPDNGPTWIALRSGDLSE

VPSLTWTPSVESRRTGHPTDSGLSDELGAVRTHDGGNLFGSLKRSELQQPPDFMVDPTHP

SSLDSIVRWMTAQEPSQRPLADQILELEGLRWIAEHRSAPATVYEGSWGPAETMPVSIML

DSDSEMIDV

>SoG_01119.T1

MKFFGGSKKSSSSKKHHHASEPDGPDDFEDSHPTRSEPSSPSKSSSRSPKKPSRPSSYID

SAPKQSRPSRASRHSTDPGYTSSSSRRKKHDSSKIDPNTHPLNLPPEERKRFSALSQSAM

SGRNSMDIDQEPPANNGPSTPPQAASAQTNFAVPIPNGTTHQEDAAPAPPPHKSNPSSPQ

PTPADEAESYKAAGNRFFKEKNYAKAIEQYSKAVDLFPDSPTFLSNRAAAYMSNGQYEAA

LDDCSRSADLDPQNAKVLLRLARIYTGLGRPEEAMLTFGRIKPDPSAKDVAPAKEMMQHV

NSAKGALESGSPSMVLYALDQAERGLGVGVSKPRKWQLMRGEAYIKMGKENALGEAQNIA

MSLLRGNSQDPEALVLRGRVLYGQGENEKAIQSFRMACGCDPDFRDAIKWLRIVQKLDRM

KDEGNTEFKAGRHQAAIQKYTEALEVDPSNRNMNAKLLQNRAQCKIKLKQWDEAIADAER

AFSLDPNYTKARKTKANALGQAGRWEDAVKEWKAIAELEPEDRTVQKEIRNAELEVKKAQ

RKDYYKIMGLEKDCNPDQVKRAYRKLAVKLHPDKNPGDDEAEAKFKDLSEAYECLSDPQK

KAAYDNGDDLVDPADMFGGGMGGMGGGMGGIDPEILFNMMGGGGGGGFRQAGGGFPGGAR

GFPGGAGGFPGGATFSFGGEGAESSEGDIGDGNEHEWTDASEDDEKAAYAGGAKARDYMA

QRDEARTLLVGKVRQYGVWLMAAIVLLPPALVAVGAVVGFLVYTCMKWWRRRRSVRTSPV

G

>SoG_01121.T1

MRIVKPTWLSHSGEQKDFEVYSCHVSPDGKRLATAGGDGHVRIWSTEAIYNADDPSYNEP

RQLCHMSHHLGTIHSVRWSPNGRYLASGADDKLICVYHLDKGPPTATFGNTEPPPVENWK

TYKRLIGHDNDVQDLAWSYDSSILVSVGLDSKVVVWSGYTFEKLKALPAHQSHVKGICFD

PANKFFATASDDRTIKIFRFTSPTPNATQHDMASNFVLETTISAPFKSSPLTTYFRRCSW

SPDGNHISAANAVNGPVSSVAIIERTVWDSEINLIGHEGPTEVCMFSPRLFHTNNPKETD

GEDGGSLVTVLASAGQDKTLGIWNTNTSRPVVILQDLAGKSISDLAWTPDGQTIFASSLD

GSIVVCKFDEGELGWVAPAEENFKALQKYGVSRKGMGVAEDVEGLVLESHSKAGESRAVE

SRMGALMGDLQSDSPKEPTPVTNGTRPTTTTATPAATNGDATPEKAPEEVDKSAERVKEL

KSRVTIGKDGKKRVAPLLVSSSGTGQSSLPQTQLVGSSSTLSKAPRDDTPTTVLDLSKPF

DGLPKGGIASMLLGNKRKMNGAEVDDEDEPTAKRAHSGPIPIVTNGTDGSIEPAPLTPVQ

HGIVPTPEFLRPAVLNPAISYAQVRLSVPRVRSQILRPLERGILQPETTLEDASKIPENI

ILEAKNDVNPREPSHVTVTKRGALIWHEFLPRAVILVAASKHFWAVACEDGSIHTWTPAG

RRLLNPMILESQPVILECREYWLLCISAVGIAYVWDIRTQTAAHPPASLGPILDVATTAL

NQHTATPGPGVTSAHLNSTGHIVVTLTNGDGFYYARDMYTWQRLSEAWWAVGSQYWNSTD

SSISALQSTAVGPNSRNGKDCEGSSTAAAAVSSGIIPFLERHTTNEFLLKGRAYALQRII

KTVIQRSEGEGLESSVSIAHLENRIAGAMQLGAKEEFRLYLFMYAKRLGAEGARPKVEEL

LNGLMGGVLKEKGDRGTGRGWFSKEGDICGWERIELLKGVVMILGKYRELQRLTVQYAKV

LDLDLEDGTADVEAMDVEV

>SoG_01137.T1

MAHSPAADYDWWTEWASFVKSNNSIPDQYVWHEENGSGSDFSDAYAGLQQVISRYGLPQR

QININEYATYDEQVPAGSAFWISQLERHDAIGLRGNWLSGTQLHDLAASLLSKPKPSDYA

SGGYFPNGDWWLYNYYSHNMTGLRVPTSVSSDGRLDAYATVDPETRTARIILGCHPPTTG

TYDVTISGLTSLGLPSDGSLQIRTWKFAVASDVHYGEMGPPQDLGYYGHTISNGQISLPW

FQTDRVTTYAWEFQY

>SoG_01142.T1

MAPRGFDDEELTISLSSSHLRRQQQQQQQQEEARRAREEGSRQPPMPTIADGVAMDAPPA

TERVKAKTEQRIDAYKVIRTLGEGSFGKVKLAVHTSTGQQVALKIIARKKLISRDMAGRV

EREIEYLQLLRHPHIIKLYTVIKTNTDIIMVLEYAGGELFDYIVANGRMKEGEARRFFQQ

MICAVEYCHRHKIVHRDLKPENLLLDDNLNVKIADFGLSNIMTDGNFLKTSCGSPNYAAP

EVIGGKLYAGPEVDVWSCGVILYVLLVGRLPFDDEHIPSLFAKIARGTYSIPSWMPAGAV

NLIKKMLVVNPVQRATIEDIRQDPWFMTDLPPYLQPPVEEFFNTGMDPAKAIEKSDIAPH

ASEVVQERLHNEVTEKISKTMGYGKGDVEEALKAEEPSAIKDAYLIVRENKLMQVSQNPD

ALIVDEPEGAGSSPLMSLSSARSGMSQIPGQVRPYVSKVGILPSSIPTYHKDYVERLKSG

ILESHSGTDNPDEEPAPARTEAEKEEVSRRLKPHTRSQIRLDDAAKPQSMTPVTPKKNKP

VRWQFGIRSRNAPWEALLTIHKALHKLGATYLPDEDFELAHRKEPEGSRSGDSSFVREHD

GGPVGGGSTASLDSTQGYKLPADPWHIKVRWDSSTIRKHSQSPSQGGEKDKYGTPDSFHV

YSPEEASKKSPFVAMHLDIQIYEMEHGVYLVDFKCSGYETSQGLLLEEKDVTSPFPFLDL

AAKLIMQLAEAD

>SoG_01146.T1

MSDAFAPRSMKRKNVKGLALTPAAPKPPPTAETSFVDPGESNRDEQLEIGIEYKLDLRPE

DLEIVKELGSGNGGTVSKVKHLTTGTIMARKVIHVEAKKEMRKRIVRELQIMHGCHSDYI

VTFYGAFLNYNNDVIMCMEYMDVGSLDRVSRVFGPVRVDVLGKIAEATLGGLTYLYTKHH

IMHRDIKPSNVLVNSRGSIKLCDFGVSGELINSIADTFVGTSTYMAPERIQGEKYTVKSD

VWSFGLTIMELAIGKFPFGASEALSDGDGAPAGILDLLQQIVHEPAPKLPKSDAFPSILD

DMIQKCLYKEPEARPTPQELFDRDPFVQAAKRTPVDLREWAVGLMERDNRKSHLAPQLSP

STQELLRSGDSPIFPPQHEERSLQTPTSGEIPIAGTGFMSPSEALGHGSRSPTRNGSGRT

PITPHPGLGQRSTTASSIPKASIYPTSAGPTPNSASAATFSLPMRPGPPGGPLPPPPPRK

ETPDESRREYRR

>SoG_01159.T1

MAAFQAANKDLDYDVLVIGGGLSGIYSLLRMRELGLRAKVIEAGEAEGGTWFWNRYPGAR

FDSESYSYIFSFSQEVLDEWNWTEHFAPQAETLRYIQFLVKKFDLKKDMQFNTRVKSAHY

KQETNSWLLTDEAGKQYTSRYIITGIGILNQPTLPDIPGVDDFKGKAWHTARWPADHSEL

NGKRVGIIGTGATAIQTIQEIYKTAGTLTVFQRTPNWTAPLRNSKITPEEMATIRKRYPE

IFKACLESYACFIHVGDSRKTTEIPKEKLFAEWEELYQKPGFAKVLSVSADIATDHEANR

LYSEFHAEKIRARVNDPAVADKLIPKNHGFGTRRVPLESGYYEAFNQPHVKLVDLTETPI

ERITEKGIRTTGGSNGVEHEFDIIIYATGFDAVTGSFNAIDFQGVDGVKLRDTWSGGIQT

YLGLTVKNFPNMFMIMGPHQMFGNIPRSIEYAVDWVANFIRYARDNNVLRVEPSQERMDQ

WYQHVGDCAKGLLANEVDSWMTGVNTNLKSKQKRSLTRYNGPAPGYRKKCDEVKARNYSD

FEILCG

>SoG_01160.T1

MHGGAHSGVAARSNDDTAYAHRDQLFLFQFYDSIYDFSNTAPYPQSGFELMQGFVATIAD

TLEEGQVGKYINYADSQLSRKDAQKLYWRGNLRKLQKIKAKYDPYDVFGNPYSVEPVA

>SoG_01161.T1

MFAASQGFALAGCNESTRYLISFGDSYSQTGFNVDGERPSAQNPIGNPPLPGWTASGGLD

WPGYMVTGFNRSLTLAYNFAYGGATVDASLIAPYQPSVRSLVDQVAQFSGSIAARPAYAP

WDAGNAVFGVWMGVNDVGNSFWLQNVEDVLGKTVRRYFDQLQIMYDAGARQFAILTVPPT

DKTPTFLMADAWSREHLQSAIALFNKLVAEGLSAFEASNQGVTAKIIDTAVPFNKAIANP

TAYGAPDATCFNSNGVSCLWFDNYHPGTAIQRLVAAAVAEAWSGFFKS

>SoG_01166.T1

MRQIIITTVLALCASAGTHASLSGPAESQPVQGPRHCRYIAGDDGWPDLERWTALNKTVA

GRLIKTRPIGSICHYPNYNRAECSALARDWGLVKTHEPLPAEFLSLYFQNNTCTPFTSPQ

TRCQLGNYVPYSIDVRSINDVRAGISFAKKHNIRLVIHNSGHDFYGQSTGNGALALWMHN

YNKVEVVQHYSSTYYNGPALRVQTGAEGAVAVAHAASHGYTVVSGACPTVKMAGGYLAGG

GHGYLNGVYGFGADNVLEWEIVTANGDRLVATPEAYVDMYWALSGGGGGTFGVVLSATVR

AFPNSVTSNAAFSFSAEQAGGPEQFWDAVDTFHEQITPLLDEGFVVEYGFTKDILAVVGI

MASGRNSVELQSRLQPLIKALTSRPSSQLTTESLPIKLTQADSYYDLYKTEIPPVLEPLV

LGPAIAGRFVPRAAVDSGSAALNQAYRAIAERGYSFSVIALNALNAVRNRTAPPIAPNAV

QPNFINAYSSLMINASWSHSSPWSEVQSVQDTLMDEIIPIFDAAVPGAGAYKNEANWAEK

DVKQSFFGGTYHRLEEIKLKTDPQGLFYGLVSVGFDKYETDSDGRLCRRLVA

>SoG_01168.T1

MLPIVILNETYAHPQYLSLAAGVLCYFAYFHHGEHHLHGTTYIKIHTAVIVGSLIFLYLR

FNLQLADALFLALRYDTLFLSGLFGSVLVCRALLNPLNAFPGPWTARITSFDMTLRIRKG

QMYKTLQDLHGKYGYFVRIGTGELSITHPRAVQDVFGADSKCHKSPWYDISRPQDSLLLR

RTAKGHAELRRVWSAAFSTKAVRGYEQRIQPYRNKLIAGLDECVGEMIDMNMWLALYTWD

VMGDLTFGHSFGMLDTREQHWAIAALNSGMSVIGQHLPMWLFRILVSIPGAQKDFKIMLD

YATEQMLDRRKNEPEIPDVMSHIFVPYKNGKPWDATAINLMAGESHLLLNAGSDTTRTTL

AVALFELAKKPEYLQKLYEALAKHISESSSEEILHDQIANIDLLTGVINEALRLYPPNPS

HPTRVTPPEGAMIAGKFIPGGTQVFTPQYVIGRDERIYPQANEFIPERWYSSPELVTDLK

ATAPFSIGPYNCIGKPLAMMNLRVTLARIVMRYNFRFASECSNPVEEFVGRMHDHFTLQG

GPLYLKLERRNQGRE

>SoG_01173.T1

MESQDKQEPIAIIGAACRFAGEASSLGSLWDMMSNVRTGHCKVPEDRWNSENWYHPDPDR

KGGIASQHGYFLQQDISVFDAPFFSMTAKEASIMDPMKRLLLETCYESIENAGVLVEDLM

NSKTGCYVGCMTNDYEMTSLHDIYDVGGTAASGLSEAMIANRVSWFFGLKGPSLTLDTAC

SSSLYALHLACQSLRLRETNMSLVTGVNLMFNPNTYHQLTLMHMLSPEGISHTFDDRANG

YGRGDGIGSLIVKRLSDALRDGDTIRAVIRGTGANADGKTPSITQPSSEAQAELIIQTYE

DAGLDPALTGFFECHGTGTPVGDPLELTAIANTMGAARRNAGKEPLLIGSIKPTVGHTEG

CAGLAGVFKAILLLEKGLLVPTYGVERVNPKLKFEEWHLALPEKVMEWPTDGLRRVSVNS

FGFGGANAHVIMDDAYHYLQERGLTGRHNTTVLPPANAQVSTKKEISNGTNGTNGTHITN

GTSSAAHDAQPKLLVFSGKEATAASKRAHSLSSWIDSTSDEDVLDALAHTLAAKRSYLDH

RTYAIANSLSGLQEKISKGLPAPIRSLRNGNNIVMVFTGQGAQWPAMGQELLRNEHFHRS

ITASQSYLESFGCKWDAIEELSETADSRINLPEYSQPLCTVVQVALVDLLRHWQVSPVAT

IGHSSGEIAAAYGASLITHKDAIKLAYVRGLSSAAVTKQGAMMAAGVSEAEAEGYLKDVT

KGSAVVACINSPSSVTLSGDVEAIDELEKLISADGKFARKLKVNTAYHSPHMRDVSESYY

NMIGKLENANGDSSSSRTVMYSSLTGAMISSAEELDARYWVENMQRPVRFLQGLLALLKH

KRSASESARPTATQWGAFVEVGPHAALQGPLRQIIDASGNKHAKEATYVSMLVRGKDAIE

SSLIAAGALWATGAKVDLSAVNQQNQSEPGSEPRMLCDLPTYPWNHTKGFWHESYASKAY

RFPPHVRSDYLGVPEDSQNAHEPQWRNYLRIAENPWIEDHVITGTVLYPGAGMLVMAIEA

ALCTADATKVVQGFRLTHVRFARGLVVSLDDMAPVATRISLHPHNTNSDAWEFTVYSMNK

GTSWTKHCSGTIAIEYKRDTSAIELGTTDAIWDRQVKLRKALTGDASSFVIDTDAFYENL

DKVGMQYGPVFRNVKTLSAVPGQNASFGEVAVPDTQSTMPKHYESPCVVHPATMDSIFHL

ALATLNGGQPVKQAAVPYGIDEIYVAREQPKDPGAFYSGYGRLLSHDGHEINTELVVSDQ

DWLEPKLTIKTFALRQVSAAETSDAAGNATEEYKKCAGITWIPDLAYLRSDDALAHLLSA

SDAASRFDKFFDSLFLRTSQATALVVMLDTSESSMRVLRHLKQRTHKPSHVIALTTSEEN

QNSMQAALGAEDPNMAVTYQWLPESELIASLAAEANDVVIGLGVVADDDSTVKLAALAPT

ACLVGTKGQAFKVPEGRFSCTFSEDDVSYQLSAAATSPLSGDLPAAAVLLMPPTPSDAVT

TLAAALEERLLSIGVTASREAFDANGVATLGGKYVISLLELENPLVYGWDESQFSAFKAL

ISTVSHLFWISRGGVLQSWSSSVEFATTQGLLRVMRNEYPMVTAPLLDLSTLADTKDSRT

ADIVLSTWRSSLADGAEMEYAEQDGMVFVPRATCLSGLDYELQLENKAAKPAIAEIGSLK

GPSKAVSSSDGAGAVWILDEASLQPLGENEVEIKVEHMGLKVGQPLISAQNAVGLVVRVG

GQVSSVNLGQRVVAYSPCAASTHIRQTVDLVSALPQGIGAAQAISLIEPLLAAHYVLHDA

ARLQPGQVLLLDNAASPIGQAIIHVAEAHGASIFALVGSEGERDTIITCAGLEASRIFDA

GLQNFVPLIGDATQNRGVDVFISQKPGAHTIGALSSLRDFGTFVDISGNAGALPLQGFKS

NMSYARVDVSAALQADQTLVSKLFQQAFNGDTLPAFPPPAELPVTHFSSEKTFAGADDVV

ISILDSSRVNMPAPPPKELSLNGEVAYVLAGGLGALGLDVANWMIDCGAKSLVFMSRSGG

KKNGADLERFRARGVDAEAFACDVNDAERVARTFAAVRASGKKIGGVIQMAMVLEDSIFD

NMSFTQWRRAIEPKTKGSRNLLANIWPGDDSFFILLSSITGVIGNTAQANYASGNTFEDG

LAQHARTHLGINATSIDVGLVVNSSHFTTTGEFGDITGYLNKYQHGWKGLQTSLGELGVV

MRTIMRGSTTDGHTLPPQVVLGLGTGIEYDEEAGGYTNDKKFELRVVKRGQQDDDGSTKK

DIGTLLSNAKTMAEAVAAVEGSIKELVAKAMDVELEQVDGQKPLYDYGVDSLQAADIRSR

AQKNMKSDISVFDILSAMPLAEVAAKIASKSQLVKVAASEE

>SoG_01175.T1

MADWATCLSYLSINLQLFPWRIRMGFLVSYISEPTSALARSQDRPSGDLYLDVANKYPGQ

DLVNLNFKRDLLLATSARALADILVHRPYDFAKTKNARDWLRLILGDGLVVVEGDRHKFL

RKNTLPAFGFRHIRDLYPMMWSKSETLIRALRQEAGRPDPVAQRGPGVIQLSDWASKVTL

DIIGVAGLGRQLNAVEKKQDPLTSIYERLLNPTREMIVYSAACIAFGYDRVKLLPWKMNK

SFADLISSLDDVCLDLVRDKREAILKKGDDHVDILSLLIKSNNFSDADLKDQLLTFLAAG

HETTASAMTWACYLLAKHPEVQDALRREVNDALPADPSADPSLDLGGVLEQLPLCNGVIN

ETLRLYPTVPQTVRQAQKDTLVAEQPIPEGTAIVLSMWQINRSPEIWGDTAAIFNPSRWI

SEDGKPNQNGGRASSYEFLTFLHGPRSCIGQNFARAEMRCLLAALVRSFSWELAMDDKDV

LPKGVVTIRPLNGMYLRLRCLSD

>SoG_01176.T1

MVLTIAKRNALSHAARQGNEAATRVLLSEPIDANTVDAVHGRLPLSWAAEMGHDGVIRTL

LEFHADVNATDERSGRTPLSFAAETGNTSIVHLLLQENADFDAVDSTGKTPLLHALTRRQ

TEVVSMLLDRGARAETPDRYGRTALSYAAELGQPEIVHRLLEVGAAPDFADSDGKSPLWY

AAGSGKTDIVRMLLANPAVDVESVDSTSTSALSNAAFHGHVEVARILLQRGASVESLDDM

GRSPLSFAAENGFVEVVRVLLESGADPTALDEDCRSPLSYAVECENEELVGLLVGLTPDP

DLGGSLRHTSDLAVIRLLLDNGANPYSAEYGTGDYCPVALAAERGKDELLRMLLWSGTAG

DEGVKKKQCEAALRCAASEGVISTVRILLEEGLFEDRGECVKYALWFARTHEHDDVVDLL

SSLDQD

>SoG_01179.T1

MPASHQLPHDDSPRPPVDQGYDAVTPAIPSGRTDYNFETTTDLPSYFSRADPLISRNDPD

DLPLLRQDGQNPYSTSLPGPMARRESLSDIRASNPDLALTGNIISATFTIPHSFVYRKGG

SWDLSLRRGQSALFDSLSHLSSDATPWNHTVVAWTGEIESPSDVPLSPPTTPGNTSSSAS

SHNPLSAPVPVDAESKLPTPPPSDGLWLPKADMENLEYQLSHSKTIRTVPVWLADEDEIF

DDGMKLKDQARWRRYAEHDLYTLFHYKQHEPTDGRAERLEWADYYRMNQKFANRIMEIYK

PGDIVIVHDYYLMLLPSMLRQRIPNIYISFFLHCPFPSSEFLRCLSRRKEVLEGMLGANL

IGFQSYSYSRHFLSCCTRILGFPSDSLGIDAYGSRIQVAVFPIGIDAAKVLSLASADKVT

EKYNALKAMYKDKKIIVGRDRLDSVRGVAQKLQAFERFLEMYPEWRDKVVLIQVTSPTSV

EAEKGDAETKIASRVNELVMKINGQYGSLGFSPVQHYPQYLDQNEYFALLRAADIGLITS

VRDGMNTTSLEYIVCQKDGHGPLILSEFSGTAGSLSDAIHINPWDLSGVAEKLNSALTMS

DERRMAMQSSLYRHVTTHTVQSWINKFIQKTYTVLSESKSTNATPLLDRSQLLQRYRAAR

KRLFMFDYDGTLTPIVREPSAAVPSERLVHVLQILASDPKNAVWIISGRDQDFLHQHLGH

IKELGFSAEHGSFMRHPTEEEWENLAEKLDMGWQEEVIAVFQKYTDKVPGAFIERKRCAV

TWHYRLADPEQGIHMSRECHKELETTIGLKWDVDVMPGKANLEVRPTFINKGEMAKRLVH

FYNDQADETPIEFAMCLGDDFTDEDMFRSLNGLSGSVLKDEHVFTVTVGASTKVTLAKWH

LLEPADVIECMALLAGAGGEGQKEWLGEVNLAAVSAVEGHIPEEEKLQME

>SoG_01187.T1

MPLLNGTTSSTATNGDDPHSPTTHYDVVIIGGGFGGCYSLHKFREAGFSCHVFEAGSGLG

GVWHWNSYPGARVDSEIPYYQFSLPEVYKDWNWTQRFPGHAELKEYFNHVDKKLSLSKDI

SYNSVVVGADFDDDTHTWTVETQNGHLVTCQWLVPATGSSHKPYQPVFSNMESFQGRIIH

SASWPAADPELEGKNVAVVGAGASGIQCVSEISKKARKLTNYIRNPNIALPMQQRSLSEL

EQRINKANYGNWFRNARESVAGIAVDPCIATSGDHSPEEREAMWEELFARGGFNFQSANY

VDYLVDEKTNKLLYDFWCRKTRERVKDPAKADIVAPLEQPYPFATKRSSLEHDYYECISR

ENVELVDLRKAAISEFTASGIRTDDGDEREFDVIILATGYDNMTGSLTSMGLRGCNGVDL

RKKWAEGVSTYLGIFARDCPNMFMIFGPQAPTAFTNAPVFIEMQVEFVVDLVKKLRDQGI

QRINVKKEAEEVWYNAVQDISNATLFTKAVSWYMGSNIPGKKREQLNYIGGMKGYMDACA

QGTKDWSNFDVNPLPRGEMPVVR

>SoG_01190.T1

MASLPTRERRPSSGAPIVDIQGAIGPAGISRPKHKRTVTGLAPGEIKNVEASIPEPQREA

WKRSQIKAFETPEDFQKEVVRHVETSLARSMFNCDEYAAYAATSLSFRDKLVLDWNQTQQ

RQTFRDPKRVYYLSLEFLMGRTLDNAMLNVGLKDTAKAGLNELGFRIEDVITQEHDAALG

NGGLGRLAACFLDSLASLDFPAWGYGLRYRYGIFKQEIIDGYQVEVPDYWLDFNPWEFPR

HDVTVDIQFFGSVRKDVNAEGKTVSLWEGGEVVRAVAYDVPIPGYDTPTTNNLRLWSSKA

SHGEFDFQKFNSGDYESSVADQQRAETISAVLYPNDNLERGKELRLKQQYFWVAASLYDI

VRRFKKAKRPWSEFPDQVAIQLNDTHPTLAIVELQRILIDIEGLEWNAAWDIVTSTFGYT

NHTVLPEALEKWPVGLVQHLLPRHLQIIYDINLYFLQSVEKAFPNDRDLLRRVSIIEESQ

PKMVRMAFLAIVGSHKVNGVAELHSDLIKTTIFKDFVEIYGPDKFTNVTNGITPRRWLHQ

ANPRLSELIASKCGGNGFLKDLTKLNQLEKYINDTEFRKEWAEIKYANKVRLAQHIKKSA

GVTVNPAALFDVQVKRIHEYKRQQLNIFGVIHRYLSLKAMSPEERKKQQPRVSIFGGKAA

PGYWMAKQIIHLINAVGSVVNNDEEIGDLLKVIFLEDYNVSKAEIICPASDISEHISTAG

TEASGTSNMKFVLNGGLIIGTCDGANIEITREIGENNIFLFGNLAEDVEDLRHAHTYGSH

TIDPDLAKVFEEIQKGTFGDSHDFQAMINAVKDHGDFYLVSDDFHSYIETHAMVDEAYRN

QDEWVEKCITAVARMGFFSSDRCINEYAEGIWNIEPVVARD

>SoG_01191.T1

MAEFVRAQIFGTTFEITSSSARDQLTNQNVAIKKIMKPFSTPVLAKRTYRELKLLKHLKH

ENVITLSDIFISPLEDIYFVSELLGTDLHRLLTSRPLEKQFIQYFLYQIMRGLKYVHSAG

VVHRDLKPSNILVNENCDLKICDFGLARIQDPQMTGYVSTRYYRAPEIMLTWQKYDVEVD

IWSAGCIFAEMLEGKPLFPGKDHVNQFSIITELLGTPPDDVINTIASENTLRFVKSLPKR

ERQPLKNKFKNADPPAIDLLESMLVFDPKKRITATEALAHEYLAPYHDPTDEPVAEEKFD

WSFNDADLPTDTWKIMMYSEILDYHNVDANEATMADGFNGQ

>SoG_01199.T1

MSVTSSSQLKELVLETAPDLHPSWVAAEQVLRQASDPSKPPMEPLLRQPIYAQECRDLYA

SVTAPGSRDHHLSKGISKKEFTVPSSVDGFPIPVMQLELEAGENQDGGEEEEPEVIVIYY

HGGGLHVGEADSEEYSCRRIIKGSPARVRLYSVGYRLRPKYPARICASDARDAFHALCSK

GSKIIVVGSSSGGHLAASVAQKAPKGSIHGVLLRCPATCDPTDDRQHIPEWLRPFHTSRH

QSFATFLLDKSKGTVVPRGELDDVPLEATREELRGYPKTWIQVCSNDNLYSDGICLAMAL

VDVGVETKLDVWKGWPHTFWLKTPHLQEALEADESMLEGLQWLLE

>SoG_01200.T1

MKSISCLCVLTFGQNALANIVSGSLKTITNGADNTNQAAWWSPMDEAGGYDWLSYVRNPA

TGSKLNNNIMVARVDMSTGDIVQDCVRATSADDCSLFIDDSGHNDPSLAVDGDGFVHVFT

SMHNNPWRYFRSQEPYSSTTLVNRSIEMPDANVLITYPVVKRDASGNLWLMVRGEASDAT

GRGGYLYQYVTKAKTWSRIARFAYRKGFAVYPDDIQFSSDGDVHLQWEWSKYPSSVGRHE

GSYLRYKPSTGKFLTITGDEATLPVTQGTPNLVFQPLTGGEIYQSVNSDPPILQSAKMAL

YEDFAGAVHVQHAYRFQNETSGPFQIRRARSTGSASSPWAREIVQASPDTTAAVGITHDG

TTVRIYYCRKGGSAWVLEKAGNAAWTNNELTAVQGKKVERLQALMRSDGTDVLYLGAPTN

VDSTTGSVYLLDVAGR

>SoG_01207.T1

MAYSQHDHTFFVESDEHHHTKLSARDAARMIARSRQDVIAGELSRLAGEEYLEDIMEHMK

HMEIETLPDANLIDMQREIQWFMRPYLIDFLIEAHAAFSLLPETLFLTVNLLDRYCSKRV

VYKQHYQLVGCAALLIAAKYGDKKDRVPQIHELNNMCCGLYDAGMFTQMEMHVLNTLEWT

IGHPTVDFFSQLMVMEERDDREVESMAAYLCEIAMYHRDFVSTKPSTMARSSLALARAIL

GRSEVTEGEWDQADDLTVLTLSQHLHQPSPTLARKYSSSNYCRVAQKLADFMAQQAAIAR

RNAQPPTPPVIEATKQASIYSTPQKGHGAVAGFDGYLTPPITPDGASFTGGQQQSYPLPS

QCPTTPTPQGHSAYAQHVQQYTGFVQHHHGR

>SoG_01211.T1

MLQSQLDPLPRDLPFRIISKTVGRGAYASIKKAIPLDSSEPVFAVKLIHKGYAVKHGRIS

AKQLAMEVSLHSHIGQHPNIIEWFASGEDAVWRWIAMEFAEGGDLFDKIEADVGVREDIA

QLYFTQLVSGVSFMHSKGVAHRDLKPENILLSQDGQLKLADFGMATMFEYKGQRKTSSTL

CGSPPYIAPEILACGRVDRRAGTGPKYSPDLVDLWSCGVILFVLLVGNTPWDEPSQGSWE

FQEYIRTSGRSTDALWERIPSQALSLLRGMMSVDPRKRFTFAQVRQHPWYTRHNSLITAD

GQVSDPIHLATQMLENLHIDFSQQVSSSQRPVSDDMDVDTGLNAGRFASTQPETPIADTD

WDWERPALRSMAAPSSSLPTAQADDARRMLLNHLADEPSMSQFAKKAGPTMTLTQQARRF

RDICPPESLTRFFSHVPPAHIVQMLSDALHQLNVPLGGVPVPNPYGNPMATLKVKALDGR

QQELRGEIVVDRHQLPDGNEVLDVRFVKVKGDPLEWRRFFKKVVILCKDGVYTPDS

>SoG_01220.T1

MAPDLNSMPPSALHVPARSPETADTKPNILYIMADQLAAPQLKMYNPDSQIKTPNLDRLA

QSSVQFDSAYCPSPLCAPSRMSMISGLLPMKIGAYDNASQINSEVPTYAHYLRSKGYHTA

LAGKMHFVGDQLHGYEQRLTSDIYPGDFGWAVSWDEPDKRLEWYHNASSILQAGPCGRSN

QLDYDEEVMFKSTQYLWDHVREGPNKRPFALTVSLTHPHDPYTITKQYWNRYENVDIALP

KVRMAKEDLDAHSQRLLKVCDLWEQDFTDEQIRRAKRAYYGSVSYVDDCIGKLLETLEDA

GLADNTIVIFSGDHGDMLGERGLWYKMSYFESSVRVPLLVNYPKWFEPHRVSQNVSTLDL

LPTICDLIGTKPAPYLPMDGVSMMPHLKGEEGNDTVFAEYTGEGTVRPMMMIRRGPWKYI

TCPADAPQLYNLAQDPLELDNLARFHKIEPRTEEQREAKTIFGEFEAEAAAKWDFDTITA

QVFQSQRSRRVVWDALKVGQFTSWDFDPVDDGRQKYIRSTIPLDDLERRARFPAVDTYGH

EVSKSVKNSTRDG

>SoG_01237.T1

MRSAFIIGALAGTVLAQAGPYEQCGGANHKGATTCANGATCTVINEFYSQCIPANGGGGQ

QPPPGNGGGVQPPPGNGGGGGQQPPPGNGGGVTPPGGGGGITPSPVPTPPPGNGGGVTPP

PDNGGGGGGQLPPGGGGQQPPPDNGGGGGGQLPPGGGGGGGQFPPPGNGGGGGGQFPPPG

NGGGGGQFPPPGNGGGGGGQFPPPGNGSGGGGGQFPPPGNGGGGGGGGGLPPTIPTSDPT

LPSNPLPTVIPPSDNPIGTPTPLPTIPTADPNPIPTSITPIPVTPRQDAGAGPFEQCHGT

DFQGPTTCSDGFVCEVMDATYSICLPSGLPPFPSGGFPGSHSGGFPPPGGELPTGGLPPP

GGEPPAPTGGPVPRALPPLSPVLPPLPPVLPPLPPVLPSLPPVLPPVPTGTPVLPPLPPP

PSTFKTVVTPVP

>SoG_01238.T1

MAQPDMTVQLDGLQTETRNANSVDIDHLTTLELCSVLSNEDSTVHTAVASCLPVIAQVID

RVSERMRNGGRLFYIGAGTSGRLGVLDASEIPPTYSAAPGQFVALIAGGDHALRHAMEGA

EDDRQAAAVDLEALKINSKVDSLIGIAASGRTPYVLGGLAYARALGCTTIGLVCVSPSEV

WAEGNADHLIAAVTGPEVVTGSTRMKAGTATKMVLNMISTGIMIRLGKTYGNLMVDLRAT

NLKLRQRARNVIRAVAGKSCQASDAELDHLLEACGGSVKVACASLTLGLEPDAARERLDK

HNGVLARAVEDAEHGREPSPQDEMLVLCVDAGGSRCKAVIMSADGKTGTGSAGPCNASTI

GLGPVIASITSAVTAALAAHPSAQGRTLRATTFASAWVGIAGYDRPLIRPQINEALSLLL

KLPIGDGLTVSADIDLLPASISSRPDIDSVVVLVAGTGSVAMRYQRQGKDFVRTGRAGGW

GYLLGDDGSGYALGREATRVALLASDMCQPAKSELESPPSFSPLAMAVLKHVQHLNPACT

PDDILSGVLDLSRAAEGTHRAGASPASVVAQFAEVLFSVADSDSEAESILDAGASSLCKL

VATVAQGGELDVSKTALVLGGGLMQNDLYRVKVLENVRQSLGDFAHVEPVSQPVLAGAMQ

LLMRSKAAMNSKHWSSSSTTTGSHAR

>SoG_01243.T1

MQIFVKTLTGKTITLEVESSDTIDNVKSKIQDKEGIPPDQQRLIFAGKQLEDGRTLSDYN

IQKESTLHLVLRLRGGAKKRKKKVYTTPKKIKHKRKKTKLAVLKYYKVDSNGKIERLRRE

CPADTCGAGVFMASMLDRQYCGRCHLTYVVDKQ

>SoG_01244.T1

MRAYVFPGQASRLRKVPVAVLQQNPQIRHLFEVAHEVFGIDFTAIDLQNELGIRSKDTAL

AQPLVFLASMADYLQLQLEDGPKMMAGHSLGEIVALAASGSLSFKAALELVRLRGILMQQ

SCDENSSGMLAVLGLNQEEVQLICDSWNAEHIRHDQLTIANFNSPRQFVISGSESSLQYA

TSTLVSADRLVRLASPGAFHSQYMSKANEKFKDHILAMPITKPTCPIYLNTSARCTTEPC

EIREELLRHMVSPVQWTATVRNMVADGATEFLQMSTGSALKKIIQQIDSCVTVSSINARP

GNQLDVGSLSASEKLMRDIWIKCLPALAHTPLSKESNFFECGADSVDAMRLSSMARRKGF

RLSLETINNYPVFSSMALSLEDEEYRADSGEQDAARAYSLLPSGLDLDTTKSQVAFICGI

PESDIEDIMACSPLQEGLISLTSKREGMYVTQKAVRIASGVNLERLQSAWSSAIQKTPIL

RTRIVHLPSGDFVQAICRRDGIWTERHKEQTPMGAGTWLMRLQLEDEPGQGPVRVIMTIH

HAVFDAWSLRAVLEEVSYTYLGQTPAAQLPFSRFIAYLKDNQAEAATSYWNRHFEDLEAP

QFPKLPTSLHQPHASGSSIASFQGISWNRKLATPSNVVRAAFGLLISKLTGSSDVIFGST

VLGRQLEDLRDAESIAGPMIATVPTRVLVDMDMTTATFLGTVQQQTQNCSPFVHIGLSKL

SKISSNTRAACAFQSLLVVQHLPRQRSSQVFAEDVQNLPATKAAMGNFSNYALIVEISIA

DNGFSVRIQYDQAVSDEVAVGRITSQLIHSIHQLNNPAHLHQPLQEISWTCQQDLEHIAT

LNKPDLSSVDSCVHELFVQRAAERPQSPAICAWNGSLTFYELDYLSSRLAQRLISHGIGS

AYKGFSAVLPLYFEKGVWTVVAVLAVLKAGGAGCFLDGSLPEDRLNSIANQLDPGLILSS

RERFGEAQKYWQGKAEVLLVDQGLLEALSPLPDGFEFPRVDSSDAFCALFTSGSTGKPKG

VILSHSQMCSAIKYHADILEWSQDTRYYDLSAQAFDASWLGITQVLCTGGCLCIPSEADR

RDNLAGSFARLACNTTMTTPSVARTLVPESMPGLKSLLIGGEAVTGNDSAQWKHLPNFVS

VYGPAECVILSTALKLNSKFLKPEDHANFPTGILGSACGANIWLVDQAGKQLALLGGIGE

IWIEGPKVGAGYFGNEEATRQAFHDDPAWLPENMRGILGRKLRRFYKTGDFARYDSYGRL

IFMGRRDLQVKINGQRIELEEIQTRAEQALKAPDLTIFAARQMQLHINVITPRSHCKNVL

VAFIASRADHEQELKQSDCNKPLTEAAFLITRSLARQFPDFMVPRIFIPVTTIPMSATGK

TDQRRLREFGSSLTLEELIRHNPLRNDDRAVPESGAEIALSKLWAEVLQLDSEMINTLDT

FYQLGGDSIAAMRLAGLARSRGMAITTLEIMQNPVLCDQALLLKMHLSRRLDVPTAAPFS

LLDPALVQDTVALGAAMCGVDKAAVQDMYPCTPLQEAFMAATVREPTKFVAKLVYEVDDA

VDGRPLIRALQSAQREFDVLRTRIIDAGKHGFMQVILTAVCPIYEWPSLCEYRKAQQPAF

GLGTSLARFDLIHSPEESKRYFILSAHHTIYDGWSLPLILDRVASHYREPELPLRPAVSY

STFVRYVHEIDSVAARHFWLEHLNTVEATIFPQAPRQAYEPLSQHTFSQPAADIIWPRVG

TGITTNTLLTTAWAAVASRYADSEHVVFGTISNGRLAPIHNIESIAGLTIATTPLCVHIS

RAEQLSAVLQQVQDSLSAKIPFEQVGIRTIKGYSEQAARLCQFQTLLAVQPNNEVDESSR

QGHLFVRSNEQDLVVKEFGHDTGNFGSHILSIQVFLETNGFTVKVTHDPDVLGEAPARRL

ARQFDAVVRQMVSCINSGQDICIEELSLHNEEDITQIWKWNDRVPPLVEGRVHDLVHQAA

LMYPERPALCSWEGEMTHGQLDELSTKLAMHLVHIGVHPGVIVPLCFEKSLWTVVAILGV

MKAGGASVTMDVGQPVERLRQIVSKASPIIILCSANRHDLAMDLTTQLASKPVIQPVSAD

ELKRLYAYGKEQLCLPIIAPSSPLYIVFTSGSTGEPKGAVVSHANFCSAIEYQRGPMRYH

PGSRVFDFVSYAFDVTWPNILQTLTVGGCVCVPSEDERKNDITASMRRLKANAIHVPTSV

ARLVDPASLPDIETVVVGGEPVALSDVQRWGPGVEVIQVYGPAECTPPIMASYNVHSEAS

MRNIGHGYGIIPWIVDAEDYQKLSPIGVEGELVVEGPLIGLGYLNEPQKTADAFIESPRW

LVEGTLGHVGRLGRLYRTGDLAKYEHDGSIIFVGRKDAQVKLRGQRVELGEVEFHISRLL

KHHDRQAEIVVDVVRPKDSAIDVLVAFVPIGLAASGTEEEVLATLRDIVAPLHSLATTLP

IYMVPMAYFPVGKLPVGGTGKIDRRCLRGIGASLTMEQLTSYNPDRENKRQPSTEWERRF

RELSAEVLNVDSNSIGADDSFLRLGGDSITAIRLVGLARKKYGLVVKVSDIFTNPRLSDL

ARLASGWIKERDDTQYQIDVKPFTLLGEVVNIDKVKANASEVLGLPESLVEDVYPCTALQ

EGLLASTSIASHTYVASGRYSLGSSIGVSQLRKAWDFVVSQTPILRTRIIELPDNGLVQV

VLRDDSQQWVEDIAYPQEFGLGLPLLRCGIVIAPSGETQFWWQMHHAMYDGVSLRLLTQS

LCQVYEQGTLPTITPFNRFISYINGTCGTDGAGLTAIEFWKSQLGGPTVRPFPSLPSRSY

RPQSDEMYEQCVDGIAWPRSDLTAPNILRAVWALLSARSVATEDTIFGAVVSGRQAPMTG

IEKVLGPTIATVPVLVHIDEEASLLSFLEQVQSQAVDMIPFEQTGIPRIREISAQLERQC

QFNTLIICQHEDEDDDARLDCSLWRDCSSEGSATTNEQVSQSAAFNSYAMFLQCVIVRGG

VRLKFDFDSAVISQHAVQWIAEQFESGLRQVLAADSSSTLVKDVSFICERDLDTMWAMNS

SLPMDIDRCIHDMVDDVSRTWPDRLAIDAHDGQLTYEELERATNFLAKLLVSKGVRSGDI

VPILSEKSLWTSVAALGTMKAGATAVLLDTFLPTERLHAVVEQVQPTLMFSSVDASDLAS

QLRVAEKLFLSQDLFRNGDPGADVTLPQVSPADIAYIVFTSGSTGTPKGVTISHSNVSSS

IHHQSKALGFADASRVFDFASYSFDVCWSNMLQTLASGACLCVPSESERRNDLQGSLVKY

GIDTVELTPSVARTLDVDALSISLPGLKLLHLGGEAVTSDDVTTWLKVPNLKVINGYGPS

EATPTTVAHALTERDIHEPLIGAGLGVCCWVVDISNTARLAPVGSIGELWLEGPLVGAGY

LKDPEKTGSSFIVDPPWLTSGHSSSRCVVSGRSGTMYRTGDLVKLSADGQLVYVGRMDTQ

VKINGQRTELDEIETIVRNHLPRGSPLTVVVDLLQPPETSESTSKIVVAFLALASTSEEQ

GPGPTSALTEAALELDRQLSLVLPRYMIPSVFLHIDKIPLGVTGKTDRHQLHHIAQSLSK

EAWDAADLLHSRHLSNSNGSTLISAPQTRTEVLIRQFWAELLKMSADKISRHDSFFRLGG

DSILAMRLVGLARFHKIKLDVSQIFKHAELHALGAAADGAKGTGTSNGEICHDVRGLHED

GSLSLPAPFSLLDLDESGLDRVLREASSSLGVPNGQIQDMYPCTALQEGFLALTEQQSDK

YIAQYHFELPVNVDVDKFKAAWYRVSAQMPILRTRFASLDGLGITQVVLKSGVELVEYKD

WDTRSKLMSQLPRFGVGATAALYELVYQSDSSNRGPIFIWTLHHCLYDGWSLSMYLDAVQ

RAYHDVEFVSGSTPFNVFIQHISLEDKQASADFWRKELESSHAVAFPRLPSVTYQVKPTS

RHIHSVRGLSLSGRDFTAATYLQAAWSLLAARHSNSEDVIFGSVSFGRQASLAGITQVAG

PTVATVPVRTSVNGAQAVSQFLQEVLEHNATLVQFEQTGLQAIRRLGGGLDTKSTFNTLL

LMQLDMDEAREVSTDTKDSLFSNSLNVYPSNQAREANHGGQVAVDEFAFSSYAMTILFTT

SKEGVDFDFTFDERVIEPDLVHRMSRQLETLLRQLCNEELGGAALATISAASACDLEDVW

SWNSTVPPDVNKRIHDMVKDNMIAKNLHGSMAVQAHDGHYTYGELDDMSDRLAAILMASD

GYTVGPNIVVPLFFEKSKWTVVSILAVWKAGSAVLTLDDNLQDEQIRGMIDQISPSFVLA

SSTCAERCQRILEVKPIIKASITVVDAAFISSLPDPSDCDAAFQSPEAQKVQPSDLLYVN

FTSGSTGKPKGVMVRHRNFAAAATYQAEAFGFSEKSRVYDFGAYAFDITWLNLIFTLTVG

GCLCIPTDDQRRDDLVRSFRSLGANLINLTPSVSRTIDPASMSGLETLGFCGEPVLGTDA

SQWLNKGPQSRLINTYGPSECTTATTAHILTEEDVTSPATRFGVGLGLNTWLVEPDGSGL

AAVGSVGELWLEGPLVSAGYINDPLKTAASFVENPRWLARGAPGHAGRGGILYRTGDLVR

YNTDHTLSYVSRVDTQVKIGGHRIGLAEIEFVAQQVLDGHTKPGSNGGRASLGMQMQVTV

DLVRPIGSETKLLVAFISAQAEGNVQSMMSASSEQLVAEWLRASEFREGMAKQLPRALVP

RVCIPVERMPVNNSGKVDRRKLSAFLQDMAVDEVLRLDCASAPSAAAVKRAPETSNEAKL

RKIWSRVLSIAEDKIGIDDSFIQLGGDSILAMKVVGIARQQGMVLRASEVIATPVLHEQA

KRLRNWQHEEHTRTDEAVMLAESRLTSSIVAASASRRVTMFQTLCIDAARHMPPAWYGFC

IMDIPASSPTLGDGPEAAGRLCTWLWCTFDILRAVFLPGSPSKDSMGYTMTFASPDEVPL

AALRTLKISESLETATRAIRDGEGYENLTLGQPFVKFWVLHQPGAGTTSPRTRVMMRLPH

AQYDGISLNRMLHQVSSYARDGTFQRPLHFDHYLDAVSRSKDLSMQYWRRMLQGANMTRP

GAEPELPSGSRTRDGSLAVPSIRTRLPNWPPKWSNNVTPATLFTAICARTISRVAGVDNV

VFGRLVAGRAMAVTHSAETTASNGSCGGMDTVAGPCINFVPVRVEDAAHRSDLVQAIHEQ

LIDGMEHEGVGLHEMGFASQTGFGFTVQYQNHQNMHRDGDEEEEEEEEREAVNGSSSGSR

EQPGVRFGWRHGQSEDRDEALFQDCVAIRAEPVENEDGGDTDMIGGSSGEHGWDVIVSGP

ERRMDVMKRVAEEITKTVQEGRDLRGRW

>SoG_01250.T1

MRSSSTSTAISAGKEKLSATINPFATPGRESPENPFATPIASFTRLPQSGSTSSHSTGLH

EARRRFKSSRLVGEYEKPWLQTTKKGIDWDRIIFYTCIAIGLGLGGFLCWRQTTTVPKNE

YCLIMDDDFKSLDNWNHEVQLNGFGTGSFDWTTDDKANSYIDDQGLHIVPTMTLESTDIT

YDQLVNGFTVNLTTQGGMDRWGKCTSDADLAPTTNDNSLYVRRPCAAVSNATNMQIINPV

RSARLNTKGKKTIRYGRVEVTARMPQGDWLWPAIWMMPQDSVYGTWPKSGEIDIAESRGN

DARSYPLGDNIVSSALHWGTATQNDRWRMSYGEWGGQRTRYTQNFHTYGLEWSEKYLLTW

LDGRLRQVFFFDFSKNKNLWTYGQFAGEPVNGSVPVDPWSSTGQVNTPFDQEFFLILNVA

VGGTNGWFPSYSLDVRPRTRQPQRGTRRKDATNLCFFILPVFSQRLMDGRADSGNSDAVG

GKPWADNSQTPMHDFWKANSTWLPTWGPPEERGMVIKSVKMWQQGKCSGSST

>SoG_01253.T1

MDQQKNLKDDLPGKENYSVVVIGAGFGGIGMGCQLRTKLGLQSGQMLIVERQSAVGGTWW

INKYPGVQCDVPANFYSLSFCQNPDWSSFYPSGKEIFNYANWVVDKYDLRRSLSLNTEAV

QCRWEPESNNWRVTLRELQPQCGDLSTAERLHKIEKDGYSSVYKPSREIWCKILISAVGG

LVEPAPFPPNVGGLESFQGEVVHSARWHDDVEVRDKDIIVVGTGCSAAQIVPQLLKNGYG

ARSVTQLMREPPWVVPQPEPPVGHSVWERWAPFLNRNVPGFMRLMRLLVAYEVDQGWKVF

QVGSAGELRRKHLEKTLKDYIMTAAPKKVSDSKCQTTLRRDHAFTDRITNLFEKYQELLT

PLYPVGCKRRIYDTEWFQCMHDKRFNLSTLELAAIEEDQVILKTGKKTHSSEKRSGEVRI

HADLIILANGFAVHNWFHPLTIIGRGGKTMHEVFEERGGPQLYRGTALDGFPNLFILFGP

NSYTGHSSVILGLENHIGHAIQLMRPLLNGDALTVEVRKEPTTEYNESIQRDLSQMVWSS

GGCSSWYKDNGGRNSASWPYDMMHHTRLFKKPVWDDWDITPTSRAISRLRLRRVFTATTI

FASLAMCYIVGTGRYGEALKTGTSELQKKVMAAITTRI

>SoG_01257.T1

MGARKFSPDEDIPSLEGKVILITGGNSGIGLETARQLLKHEPARIFLACRSQTRFDQAIN

ELKQQGSNTDAISLLTLDLASFSSIRKAVKEFQAESNRLDILINNAGIMMTPEGLTEEGY

EIQFGTNHMGHAFLTHLLLSTLQETTKINSDVRVIFVSSAAEMMVPKDPYRIERFKTTMP

DSYTEARYGISKLANIHYAASLAERHPNLRIISLHPGVVHTNLANPVKQSSPIKGMFIRL

VNSLVAVDSAKGALNQLWAVAAPEAESGVFYHPVGVAGKGSKLSQDKSARDKLWGWTQEE

IQRHMA

>SoG_01262.T1

MADEVYDGAVGIDLGTTYSCVATYEGTNVEIIANEQGSFTTPSFVSFTEKERLIGEAAKN

NAAMNPANTVFDAKRLIGRRFDDPTVKKDIESWPFKIVDDNGQPKIEVDYLGERKQFSAQ

EISSMVLLKMKEIAETKLGKKVEKAVITVPAYFNDNQRQATKDAGAIAGLNVLRIINEPT

AAAIAYGLGAGKSEKERNVLIYDLGGGTFDVSLLNIQGGVFTVKATAGDTHLGGSDFDAA

MLDHAKKDFARKTKKGRTPSWRRRTFQFYLSSVKSSKSKVKAKSFCVFFFSFFSNVPKMS

EEQSEVLQVVSQLCWEVLEQPGWKNPRFEFILQSQEHLYSLLSSAGVQFPLGLIACLQNP

RPPPVSWFTSQTFPIDTKHWGVYVLLLQKPESRDLLYTGSGTSLRDGVRARYQEYFSLKC

DILPRGVKEALNNGYKISSMHLLASCSTPTTGDWLAIRQEAHYAFGNVCPWEQEVFEYHG

LCSHNPLYDKVAGDVSLSPEQMLELAQQTRARKRQQTMESRARVGRRKPTLAENQASYAR

RKDIIRATEKQALEEKKFFCSTCNISCPRAVDLRRHEATIGHKNKVLGIAPKNAGQFQYM

SGDARALRRLRTACERAKRTLSSGAQATIEIDSLFDGEDFTMTITRARFEDLNAKAFSGT

LEPVAQVLKDAAIEKSAVDEIVLVGGSTRIPRIQKLLTEFFDGKKLEKSINPDEAVAYGA

AVQAGILSGKATSAETADLLLLDVCPLSLGVAMEGNIFAPVVPRGNTVPCLKKRTFTTVV

DNQQTVQFPVYQGERVNCEDNTSLGEFTLAPIPPMRAGEAVLECVFEVDVNGILKVTATE

KTSGRSANITITNSVGKLSTAEIEKMVNDAETFKSNDEAFSKKFEAKQQLESYIGRVEEI

VSDPTLSLKLKRGQKEKIESTISDAMAALELGDSTAEDLKKQELALKRLVTKAMSSR

>SoG_01275.T1

MTHQIPENKFVMPDDKVEEIYERMRAEASQALNLNPEDIECVQPCTPVQHDAIEFIASTK

QSIIGHIVFEVSSEVDLERLNVAWENAVQHMPCLRARIFTSKSGEPLQTVLVKSFTWRNA

TFAKDLKEIVEEETAASDHDHHSTRFCVFHDRSRDQRLLVWSFSHALVDDADQRQALARA

LAAYDGVDLNGSTRARAKESPGLAEAKEAIAFWQQHLHGLDVSAFPTLPSHSRLPRPSAQ

AEHRIRYPVIESRGWSDSLICRAALAVLLSRYMNAPEALFGILVDYDDPQMFNDGGRPIG

SVTRTIVPTRVICNPLDPVSHLLQTVATHEETIRHLDYIGMDGIRRSGDYGHAACGFQTI

LSVTTGNALEQPPSNRLHRAVAGSEPFVSWRDRALLLNCHIADNSALLTARYDPAIIDVP

QVARLLRQFGCLIEQLRGCGIDKPLRALDAITPEDREEIIGWNSTAIRISNKCIHEVVAQ

RVISTPDAPAVFAWDGEWTYAELDHVSSLLARVIYNDIGSGHIVPLCFEKSKWVVAATLA

VLKAGCAFTLVDPTNPTARIAKICQQTSATLMLTSRTHHGRMTSIVDHCLVVDSELVQRL

PQGDDPFISSAKPEDVAYVIFTSGSTGEPKGSMIEHRGFVTCCIEFGPRLAFDSSTRALQ

FASYAFGASLVEIVTNLMHGGCVCILSEDDRMNNVPGFIRHARVNWALFTPSFIGALRPE

ELRGVKTLVLGGEAISTEMRDNWASRVHFLYAYGQSECSTINSVADVLPNTTQMNNIGRA

IGNRFWIVDPDVPDRLAPVGSIGELVMENPGVARGYLISPSKDKSPFLTTTPAWYPSQRM

AGTASFFRTGDLVRYSSNGTIAFLGRRDLQVKIRGQRVELGDVESHLRPQFPSHIMPVVE

AIKRTGSSNTFLVVFLIGPFRDREDFYEAIPAADAFIIDNIASERIKSKLGEVMPRYFIP

SYYIRMNHRPTTTTGKTDRRTLRGISLKLLDEQDEKMKSGLEENLPSSMSTWEGKLRHLW

FASLNLALDNTSAKATFFELGGDSIAAIKMVNMAKSMGIELKVTEIFQNPSLSTLANIAR

PCSKEHNDIAVTAYDGPVEQSFAQARLWFLDQLNVGASWYIVPMAARLRGPIQVNALSAA

ILALEDRHETLRTTFVEKDGVGLQVVHDENTQGLRIIDVTSTNEVEIEDLLRREQTAPFH

LSSEPGWRVLLLRLGEEDHILSIVFHHIISDGWSLDVLRIELGQLYAAATQGKDLRSSLR

PLPIQYRHFAAWQKQEEQVAEQERQLEYWATELTDSSPAELLCDYARPKALSGKAGTVPV

VIDGPIYEQLWSYCRLRQTTPFNVLLAAFRAAHYRLTGQEDGTIGTPIANRNRPELENLI

GFFVNSQCIRLVIQENETFEGLVEQARAKTTAAFQRQDVPFERLVSRLLPKSRDTSRNPL

AQLMFAVHGQRDLGKIELQGLQSSVITTAALTRFDVEFHLFQEETRLSGYVAYAKELFDP

VTVQNLAGIFREVLQRGLEDPAQQLSILTLNHGLETLRSHGLLTIHKSKYPRDLSFVDIF

RQQVALSPNAIAVTDSESGLTYSELDRQSDRIANWLRRRHLPVESCVALLAPRSCQSVTA

FLGILKTALAYLPLDVNMPKARLRTILSALGGRQLVLLGTGVAEPDVQLPHLEVVPVDEI

VLHDCENIEPFQPPSANSLMYVVFTSGSTGKPKGVMIEHRAITRLMTGNNAIAKFPHPLR

IAHLSNTGFDASVAEICAALLSGGTIVCIDYLTTLDSASLEATFLREKVNTAVLPPALLK

QCLANAPDMLSALHTLLVAGDRFDSRDALKARALVQGSVFNAYGPTENGIQSTLYAVTEH

EHSLNGVPIGQAISNSGAFIMDPRQQLVGPGVVGELVVTGDGLARGYTETSLNQERFIDV

VIEGGITRAYRTGDRARFRPTDLQIEFFGRSDQQVKIRGHRIEPAEVEHVILSHSAVRDA

AVVVRRSQGKEPELVGFLAPHDTVLTEEDASGQVELWKNFFESTAYADIDSITDSIGKDF

IGWNSMYDGSPIDNTEMREWLEETIASALEGQDAGDVLEIGSGSGMILFNLGDGLRNYVG

LEPVPSTVEFINKTIQKIPALADKAVVQVGTAEDTTACVSKGLRAELIILNSVVQYFPTA

DYLVNVVNGLVRVPGAKRIFFGDIRSLPLNGQFAAGRAVRELGVEATKEGVTQRMTQIEE

GEEELLVHPAFFTRLKRRLPEVVRHVQILPKRMQATNELSAYRYAAVIDIGDDETASETT

INPDSWIDFEASNLDEEGLLSLLQENTGCEVIALSNIPHAKSIVERHMLDQLHDEGYDDY

IDGLTWISAVRSKARTSPSFSAVQLIEMGERADFRVEISWARQGSQNGGLDAVFYRQAPM

KEGARVRVNFPTDDLVQPEAALTNRPLQRVANYKIEAQVLDHLRSLLPAYMVPSQIKVLD

RLPVNSNGKVDRKELQRLSRIRKKATRISERKLPRNEIESILCDEFSSILGTQVGITDNF

FDFGGHSLLATKVAARLSRSLETRVSVRDIFDQPVIMDLASVIQKGSSQHRPILQTTYTG

PVEQSYAQSRLYFLDQFNHGAAWYLLPIAKRHETLRTTFEEQEGLNVQVVHEAGYCSLNI

IDVADDQGDYTNVLLKEQTTALDLATEPGWRVSLLRIGENDNILSIVLHHIMYDAWSLEI

LQRELSDFYSSAVRGVDPLSRTDPLPIQYRDFTRWQKEEAQRAQQDIHLEYWKQQLDGSQ

PATLLCDKARPATLSGKAGSVEFIIDGSLHKRLLAFSKMQQVTPFTFLLSVFRAVHYRLT

GAEDATVGIPAANRDRSETEGLIGFFVNTQCIRMQVHDDDSFNTLVPQVRSTTAAALDNQ

DVPFERIVSAILPASRDTSRNPLVQLMFALHSQEGLGDIRLGGLDCETLPVPAQTRFDLE

FHVFQKADRLSGTVLFSTDLFEPETIRNLVDVFQEVLLRGLEDPDEAIAVLPLRDNVTIV

REMGLLNVHKPHYPRDSSIVEVFLDQVLMYPHRTAVVDAYSRLSYAQLHDRSSKVAAWLR

EQYLEKETCIGMLAPRSCEAVVAFLGILKANLTYMPLDVRAPPARIEAILSEIPGRKVVL

LGNDVPQPKFRGETQGLQLVRIGDTLITTQQGERANGQSNGHIDIDTQTKGKFSGHGQSA

AMGPSAKDLAFTIFTSGSTGRPKGVMLEHRAIVRLVKQSDFVSMLPESPCVALIANLAFD

VSIQEMLMALLNGGSVVCIDYNTLLDTKALREMLRKEQVTVATFPPALLKQCLADDPGAI

SCLDVMFTTGDRLDRRDALEARSLVKSLYNAYGPTETHICTMYKVTEEDNLENGVPIGRA

VSQCGAYVVDAHMQLVPLGVMGELVIVGDGLARGYTDKGQGSQRFVQISTDGEFIKAYRT

GDRARWRATDGQIEFFGRMDQQLKIRGHRVEPAEVELAMLRVNSVRNAVVVVRKNDGEEA

CMVGFIAVAHDSTSQNSNNGIDAMNRGAKVEREVGDELQTKLPSYMIPKRIIMLDQMPVN

ANGKIDRHRLGHIAATVSLVPQDQARVRVAPRNDIETLLCEEFSNVLGVDVGIADSFFDL

GGHSLLAMKLVARLSRRLDTRVAVRDVFDHPTIADLAGTIRQGSLPHHAIPTGSYTGPVE

QSFAQGRLWFLEQLNPGAAWYTMPLAARLRGPLDTKALNAALHALEQRHETLRTTYEDQD

GIGVQVIHEFQAKDLRVIDAAKAGSCFAQLLHDEQTTPLNLEMEPGWRLSLIRIGEQDHV

LSIVMHHIISDGWSLDVLRHELNHFYTLALQGAEPLSQADALPIQYRDFALWQKQDAQVA

EQERQLTYWVEKLKDSIPAEFFSDLPRPAVLSGHAGLIDFFVDGDVHRNLQAFCQANHVT

TFVALLAAFRAAHYRLTGAEDATIGTPVANRNRPELEALIGFFVNTQCMRTTIQASDSFG

TLVQQVRTATSEALENQDVPFERIVSAVLPGSRNSSKNPLAQLLFAVHTQKDLGKVHLEG

LQSEPLIPAATTRFDMEIHIFQEDGQLDGHIVYSSDLFEINTIRNLVETFQVILREGLEN

PQMHIGVLPFSHATEELRKMKLLEIDRAEYPRESSIVDAFMEQLVASRDAIAVVDAKSQL

TYAQLDQESSRIAAWLRCRQLDSETVIPILSPRSCETIVAFLGILKANLAYLPLDVNAPS

GRIETILSEVSGRKLILLGEDVSEPQFQDPRLDCEFVRIGAALAAETETDAGAQPSATSL

AYLIFTSGSTGKPKGAMIEHRSVLRAVKQGSLTSALPPAPRVAHLTTLAFDNSVWEIYTA

LLNGGTVVCIDYFTTLDSKALETVFITERIQTAMLTPSLLVQYLANSPAAISTLDVLFTI

GDRLHSRVALQAQALVQDSVYNLYGPTENTIASSMYRIEGDEDFTNGVPIGRAIRNSGLY

IVDVAHKLVGLGVMGEIVLTGDGLARGYISPALNVDRFIQLTIDGKPVKAYRTGDRARFR

PSDGLLEFFGRMDQQIKIRGNRVEPAEVEHVLKDHDILLDGCITVRREDGKDREMIAFVI

VRAEMKRNGVNGVNGVNGVNGLPPDRDLNDAQDDQGQGAETHIEMEVRKRLQTLLPTYMV

PSHIVELEQMPTNANGKIDRKALSEQAEAMSLGPRLQLASQHVPPRNELEASLCDEFRDV

LGVELGVTDSFFELGGHSLMATKLAARISRRLDVRVSVRDIFDYPAIADLAAIIHRGSNP

HSPIPSTAYTGPVEQSFAQGRLWFIDQLEIGANAWYLVPLAVRLRGTIDLAALNAALLAV

ERRHETLRTTFEDRDGVGVQVVHPASSKELVVSVLPGNKNGSYSEWLRREQSTPFNLSKE

TGWRVSLLKLGEKDHVLSIVMHHIVSDGWSVDILCQELGNFYSIARQGKDLLIHVPQLPI

QYRDFATWQRQEEQGAQHEQQLQYWVQHLKDSAPAELLADKPRPSVPSGSAGTVKLSIQG

SLYTKLEDFSRATKVTLFTVLLAAFRAAHYRLTGAEDATIGTPIANRNRPELEHMIGFFV

NTQCMRLAVGEEDTFESLVEQARTIVSAAFENQDVPFERVVSALLPGTRDTSRNPLVQLM

FAFHAQEDLGHIQLGDLLCESLETESAIRSDFEFHLRQGKGRLDGGVIFAIDLFEPKTIQ

NFIDIFLEVLNQSLTHPQIRIAVLPLSRRVEDLRHHGLLEIEKPGIPRECSVVDVFLHKV

TVHPDEVAVIDAHSQLTYAELDRFSDKIAKWLRRRHLAPETLVGILAPRSCETVTAVIGI

LKANHAYLPLDVNVPSARIESILSSVAGHKLVLVGTGVSQPEINVLDVELLPVSGILNEN

IVHEDGNEPIVLPSSRSLMYVIFTSGSTGKPKGVMIEHRAVVRMMRPSNTTTKFPRVPRI

ANLSNAAFDASVWETFAALLNGGTVVCIEYMSSLDSKGLSDAFIQGGVNVAFFTTALFKH

SLVHNRSILGTLDVLLVGGERLDCRDALDARDIMPRTALYNVYGPTENGIYSTIYSITDN

GSPASGPPIGRAISNSGAYIIDTDCQLVPIGVIGELIVTGDGLARGYVDSALNRDRFINL

KIEGRNVRAYKTGDRARYRPADSQIEFLGRIDQQVKIRGHRVELAEVEYAMATHSYVRDA

AAITLQDQQTGQLDIVGVFTVRNEDTMAEDEQTIQAGMRKHLKTVLPAYMFPNQLILLSH

MPINTSGKVDRRELERLAKNTPKSQQVGDRVAPRNETEAALCEEYTDILHVDVGVMDNFF

DIGGHSLLATKLAARSSQRFGKKVSVKDVFDHPVLADLAASIQSQTGAKYRPIPSTTYSG

PVEQSFAQGRLWFLDQLNLGKTWYTMPLATRFRGKLDVGALDAAFMALEARHETMRTTFE

GHDGVGLQIIQPVGTSRLTIIHLSDDEPGTYLEHLRQQQSIHFNLASEPGWKATLLCLSE

EDHILSLVMHHIMYDAWSLEIFQRELACFYTAAKGGNDPLSSVTPLPIQYRDFALWQRQE

DQVSEQERQLEYWIQQLNGSQPATLLADKPRPATLSGSAGTVGLRLGGPVFARVQDFCTN

DTTIGTPVANREHPELQELIGFFVNTQCMRMTIQDDDTFSDIVDQVKEVTAKAFKNQDVP

FEKIVSEMLPGSRDISRNPLVQLLFAVHYQRKLGDLQLEGLTSEYMSADMVTRFDLEFHL

FQDDDCFEGFVLYSTDLYESETIENMVAVFQEILIRGLEQPAIPLSVLPLVSNGQLAHAH

MENSTDYSRDLSLVDVFCEQAALNPDATAVTDSNSLRLTYSQLDQESEKLAGWLRERRLA

PETFVGVLAPRSCETVVAFLAILKANLTYMPLDVNAPDGRTETILSEVAGHKLVLLGRQV

AVPKFEVLSYETVKFVKIAETSDNMEIAGVANQKPASASLPSPTSLACVFFTSGSTGRPK

GVKLEHRAILRLVKQSTLSSFLPPAAPMAHLANLAFDASVWEMTITLLNGGRLVCIDYHT

ILETKDLEATFLRENVSIAIFPPALLKQCLTHNPAIISSLHTLFTTGDRLDSYDAIEAQA

LVKSGVYNAYGPTETHVCTVFGIKEGGNLVNGVPIGKAVTNCGVHIMDQHQQIVPVGVMG

EIVVSGDCLARGYTDETLETGRFVQVKINNQSTRAYRTGDRARQRPVDQQIEYFGRIDQQ

IKVRGHRVELGEVEHAMNGHPAVRDAVVVVRGSSEHEDFGLIGFVTRDNNTTKIHSAASG

TEASERSQGIPCSEQLDAELERDILARLQALLPSYMIPVRIVTLGRMPVNANGKVDRKAL

ARVGQTVSLRVGNQATSSRVGPRNEVEACLCSEYSDVLGVDVGVFDSFFELGGHSLQATK

LAARISRQLDVHVSVRDVFNHPVVADLATTVRLGSSPHAPIPSTAFSGPVEQSFAQGRLW

FLDQLNIGSTWYIMPMAMRIRGLLSIEALSATLHALEMRHEPLRTTFEEQDGIGMQIVHP

FSPSRLKIIDIGSDDYHEHLVAEQSRPFDLSREPGWRPSLLRLGSDEDHILSIVMHHIIS

DGWSLDILRNELGSFYTAALNNEEFLHSVQPLPVHYRDFAAWQKTQITEHERQLKYWVDQ

LQDSVPAELLADKPRPPMLTGKACSLDLMIDGPLYTRLEEFCLSNRMTLFAALLTVFRAT

HYRLTGSEDSTIGTPIANRNRAELEDMIGFFVNTQCMRIIIRDSDSFNDIAKQVRAITIA

AHENQDVPFERIVSTLLPGSRDTSRNPLVQLIFAFHSQANLDRVELEGLQTTFLSPTITT

RSDLEFHLFQESGRLSGGIIFAEDLFEKHSVQCFMDIFLEILSQSLQQPDTPLALLPLTA

GADRLSSMGLLNMSKTDYPRDSSIADVFRNQVSISPQAIAIIDSSIRLSYTELDDMSERL

AGWLRQRRFPAESPIGVLAPRSWLTVVAFLGILKANLSYMPLDANAPVGRIDSILSTVVG

HKLVLLGTDVPVSKLDLENVEMVRISDVLTSKIQTENLDTECHSNTHPSAASLAYIIFTS

GSTGQPKGVMVEHRNILRLVKESDALSVLPSAVRMAFMINVAFDVSIWEILSTILNGGTL

VCVDYMTTLDSKALVTLFANERVNTAMLTPALLKHWLATTPDILAGLDVVFVGGDRVHHA

EAVAAAKLVQMGIYNAYGPTENGVQSTMYKVHEREAYVNGVPIGRAVSNSGAVIMDAQQQ

LVPLGVMGELVLTGDGIARGYTIPALDKDRFIEVTIGGRLIRAYRTGDRARCRPDGQIEF

FGRIDDQVKVRGHRIEPGEIEHAILSSGNIRDATVVIRKPEGDEEPELVGFLTVVDDGDG

KQGEASATVEDWTTHFEMVAYADVEGIQNDNIGKDFMGWTSMYDRKAIDKEQMQEWLDDT

MCTLLDGAAAGHVLEIGTGTGMILFNLGSGLQSYVGIEPSSAAVRFVNDMIASNPSFSGK

AKVHVGTAMDIDKVGELRPELVVLNSVVQYFPAIEYLSEVVEALTHIRSVKRIFFGDVRS

YPLNKDFLTSRALYELGDIATKDQVRRRISELEALDQELLVDPAFFTDLSTRLGSRVTHV

EILPKVMKATNELSAYRYAAVVHLHSQDCQGDPIRPIGPEAWMDFQKEGLSRLGLESLLR

DSPDALAIAVGNIPHEKTLVERLLNASLEKDSQDDGQVHVDGQAWVAAARTEASKYASLS

ATHLISLAKELGFRIELSWARQRSQNGGLDVVFHRYMPDQDGRRVPFHFPKDSQDHRLQS

LSNRPLQQLQSRKVESDVREHLSAMLPAYMVPSRIVVLDQMPLNANGKVDRRELTRRAQA

VPRGRETRSNHEAPRNEMELALCEEFAELLGIDVGVYDSFFDLGGHSLLATRLAARLSRR

LNAHVSVREVFNYPILADLANKIQQESTQHDPIQPTKYEGPVVQSFAQGRLWFLAQLNVQ

TSLYHMPLALRMRGSLNIEALNTALLALQDRHETMRTVFTDQDGVGVQVVQPNGSILVDV

VDVPCSDDGVHNYMHLLQKEQTKPFDLSSGPSWRVMLFRLGEGEHVLSIVMHHIMYDAWS

IEVFQRELAVAYASAKRGQDPLSQLSPLLIQYRDFAIWQREQKQIAEHDRQLEYWKKKLD

GSNPATFLCDKIRPPMLSGVAGAAELVIAGPLYERLTAFCKLYRVTLFTALLTAFRAAHY

RLTGMEDAAIGTLIANRERPELENLIGFFVNTQCIRINIEREESFSSLIDQVRTTTAEAH

DNQDIPFERIVSALVPGSRDASRNPLVQLLFSFHPHKDLGQIQLEGLQSELLGADPSTRF

DLEFHISQQDGGLGGVVFFSSDLFEAKTVENMIAIFHEILTRALDQPDKPISSLEMTNVS

QSLRDMELFEAPITDYPKESTVLDIFRQQAAAVPDLIAVTDDTMQLTYAELDQLSNWLAV

WLHNERRAVPETLVGVLAPRSCETIVAFMGILKAGRAYLPLDINVPTARVEAILNEVPGK

KLVLLGSDVPAPEFAQSNIELVRIDKLLVQVTQESIAELDAIRPSATNLAYVVFTSGSTG

KPKGVMVEHRSIVRLVKQTSMMEELPRIPRVAHVTSIAFDNSVFEVYTPLLNGGVLVCIN

YFTTLDSQSLESIFKREQVNTAMLTPALLKQCLTRAPEIIATLHVLFVQGDRFDDRDAAQ

ARSLVQKTLYNAYGPTENTVTSTVHRISADEVFANGVPIGRAISNSGAYIADTQQKLVPV

GVVGELIVTGDGLARGYTNRSFDENRFVNVTISGRSVRAYRTGDRARFRPTDGQIEFFGR

MDQQVKIRGHRIEPAEIEKAILGHDIVRDAAVVVRKDGDQQPDLVAFVLVNGADGPLNEE

YALQTGTEIRQRLRAILPPYMVPAEVIVMERLPTNVSGKVDRKQLAQLAKAVKATKAVSP

RVAPRSQLQLILCEEFSDVLGMEVGATDNFFDLGGHSLLVTKLVLRLNQRLSIRVSIMDV

FNHPVPTDLEKKIVLRQNEEKNTAPGVGPTAVDSRPFQLLQCQDPDAFLQNVILPHLQQH

EGKVLDVYYTTQVQKYYLWDPNTGRPRTLAPFYIDFPPDSDVARLRQACLDLIQHYDIFR

TIFVPHGNEFYQAVFDYLQIPIEVMDAQDDLTKVTQSLWTVDKANPPHMSDPLLRLAFIR

GSSAIRVMLRMSHALYDGLSLEHILHTLHALYNGARLQASPKFVRYIQHMVDSRPGGRGF

WRSHLHGCSMTVLRDPTLSAREDLEDRTWSLSRAIQLPSQARTDGITQATIFTTACAMML

AKESKLRDVVFGRVVSGRQCLPPADQNLVGPCTNVVPTRVTLRDSPDLGKLLRDVQDQYI

KSLPFETLGFDEIKEHCTSWPDSIAAFSCCTTFHNFSMQPENHSHNQSIQLQSLSFEADD

GSRNVAMLHDVELSGVMEANGQLLKVTIASKLNCCGKEMVVRMLDELCESAEALMTTF

>SoG_01277.T1

MFAFSWRSTGVVALAFTCARALSQGKSTSAIQLVVLFAGFWSLALAAWAVWLVWIYPLMV

SPARHLPSPSGNHWAMGQGLAIMKEIPGGAELRWMKEIPHNGMIWVRGFFNQWQLMLCSP

EAIAEVTVTNCYAFQKPPFISGSIGRIIGFGVVLLEGDEHKLHRRKLLPAFAFRQVKGLY

PVFWDKACEVVRAMTVEAASKDDGLLELQDWASRCTLDILGVAGLGRDFGAIEDAENPLV

KSYGYIMRPTTTALVLNLLRQFLPGKLVSVLPVRQNDDLNKVSEQIRTLCLDLIAAKKGK

LETGEQSDRDILATAIESDAFTDEQVVDHMMTFLVAGHETSAASLNWAIYSLCKHPDMQV

RLRQEVRDRLPAIDSGNSITHSDIDAMPYLNAVCNEVLRLYPVVPSTVREPIQDTMIQGV

LVKKGTRLILPAWGMNRYAKYWGPDAEEFRPERWLSGGSSDPSQPNECNDITEKTKAPTK

PATSTRGGATTPYANMTFLHGPRSCMGQSFARGELACLLAAWVGRFEFTLRDQGDIGWKT

AFTVKPGKDGLWVKAKALGEF

>SoG_01278.T1

MRYRIPLVAALAASASASASTPDKRDSKLFTLEIGPGETIEVTEDEKFDMIDNHVHFFDI

TEWKDHEPAVRLMSARAASYPTSVSHRSAVEAIIDKVDITKVKKQLQCFSSFHNRYFNSR

YGVEAAEWLHSEVKKTIQRSGHPFASVRLIQHQAWSQPSIIVSIPGQVRLKTIVTGAHLD

SVISGDRGAGRAPGADDNGSGSIMLLNILGAFLEDPRISKGDHKNTIEFHWYSAEETGLL

GSQDIFNTYSRLGIQVEAMLNQDMVGYAGRDGVERFGLVTDFTDPAQNEFVKVLVNEYAD

IPYEESTCGYACSDHASATRNGFPSTFLFETPFGNHSPFIHTPMDTVENVNFDHVLQHVK

VTTGFLYELAHNDFTA

>SoG_01279.T1

MLYITALCLNYLCDSSRSLTLLFKYLSQTARDLAMHHRSVLSPLIVLASLARAAAYEAAD

FNVIDALEHSGINTTLLHSLYGPSSAARAETFGCSIACSVLSVSFGTEAVTSQGEPDYER

FIGSYWSLHQEEVSPYCIFMPPSPSAVSVAVLLSRLTRCRFAVKSGGHAAFANSSSAEGG

ITISLEKLTAVDLPSDRQEVIIQPGNRWVDVYKKLDSQGLTVTGGRVSSVGTGGLTLGGG

ISFFSNIYGWACDNVVGYQVVTATGSIIDATTESYPDLFWALRGGGNNFGIVTSFTFETI

NFPEGRMWGGSRTFLEDSFDGVTNAFAELIANSPQDPKAGTWVAWLKLNGTKLAVNELWY

TEPGGADAPIFDGFNSLAPVADSTQDRVLHEYTLSLDESNPSGLREMYSAMTVKATPEIA

KMAREIFFEELPAVDGVAGANPVLIFQGITLGQIKNMGKNGGNPLGISPGDGPLYLIHIS

SWWEQESDDKLMYSFASRVLTRINSEATALGVANDYVYMNYASAFQNVIASYGSENVAKL

KRIAALYDPTGVFQELQPGYFKLDRAAVPDSGYFHLS

>SoG_01282.T1

MKPIPSPAGHILIGNVLDIDPNHPQESLARLANIYGPIFKLQLSSPRVFVANHAIANELC

DERRFCKAVTGALEEVRNAAKDGLFTAYPGEHNWEIAHRILMPAFGPLSIRGMFDEMQDI

VSQMVAKWARFGPDVPIDASEDFTKLTLDSIALCAMGERFNSFYHDDLHPFVHAMGGMLV

ESGARTRRPEIIRPLYRSSNEKYHKDIQLLEDTARTLLKRRRENPTTKKDLLNSMINGKD

PKTGEQLSDDSIIRNMITFLIAGHETTSGLLSFLFYELLDHPKALSTLVEEVDRVVGRDP

VTASHLTQLPYMEACLRECLRLHSPAPAFTLQAIGDQIVGGEYEIKDGETASILLECVHR

DPEVWGEDADLFKPERMLEESFNKLPRNAWKPFGNGARGCIGKPFAWQEALIAVVTILQT

FHMTKDSPSYKLQIRTSLTIKPKDFFIRARLRDPEFVHNLNVISAPTSQGVADKRAGQNK

SAANADEKKSLPPLDIVFGSNTGTCESLAQALASDAQQNGFSPTIKTLDSALRSLSKSTP

TVIITASYEGQPPENGMHFCEWLSAAEKDEFKGVSYAVFGLGNKEWHTTYQRIPTLVDAT

LSEKGATRIADRASIDVGHDNIFDLFEQWQAKSLWPALRKLTGGSDKPQAQQSVRELSIT

LGTSSRSRFLRHDVLPALVTATRLLTKEGASRKRCISIKLPTEATYRAGDYLAVLPLNHP

SVIRRVMSRFQLPWDATISIAEDANTPLPTGQELSVHDILSGMLELSQPITNRLAQHVAE

ANPVREEAEELEKRIADGEFSKTSVTLLDLLEEYSSSAITFGQFLAAMPPMHLRQYSISS

TALKDPSQCSLTYSVLDEPAKGSRKGRQFHGVASTYLERLAIGDYLQVAIRPSRNGFHLP

KDSSKPIIMACAGTGLAPFHGFVEERALLKEGGQQLGSMLLFYGCRAPDTDDLYREEFDK

WESEGIVSVRRAYSATPDASEGCKHVQDRIWLDRSDVVELYKNDAQVYICGAGAVGKGIE

DAFARIKQEGSDEDLQAAVDWVHERKGTRYWSDVFS

>SoG_01287.T1

MNLLLKLFALTQVAGVVVTGQAVQPVTEGNVPVKARAFDISQVVIDDSRMKENENRTLNY

LKFVDVDRLLYVFRSTHGLDTQGAQPNGGWDAPDFPFRSHMQGHLLSAWSQCWASLRDPI

CREKAETMVAELKKCQDNNEAAGFAEGYLSGFPESDFDLLESGQRRDVIYYSMHKTLAGL

LDVWRHIGDATARDVLLGMAAWVEKRNENIGYDHMQRILEVEYGGMNDVFAELYFYTNDE

RWLKVAQKFDQESTNSWLSRNEDRLNGMHANTQIPKWIGAIREYKATSDPRYLDVARNAW

DMVVHTHTYAIGGNSQNEHFRPPNAIANYLKHDTAEHCNTYNMLKLTKELWTLDPSVEYM

NFYEQAVQNHILGAQDPESSHGHITYFSPLDPGGRKGKGPVWGGGTWSTDYDSFWCCQGS

GVEQNTRLMDVIYGYDANSLYVNMYAPSTLEWKEKGVEVKQTGNIPQEDTFNLAISGSAS

FDMKIRIAPWASSAEISVNGETQSLEASTGQYATISRDWSSGDVVSVKLGMQPHLIAAND

DQGLAVIAYGPVVLIGNYGDSQISQAPTVDLGSFTRTSDSDLKFRVTADGTPVDLVPYQD

GGAHFNYVTYWRTSGSLP

>SoG_01292.T1

MFNRQLLFILALTLFVGGLLLTYTRLDAKLVGVCDGEDGCWSFSGIREKISGSAPSAPPT

NTTSDQDWGRPVSTDGQHPHGAPKSAEDPCLGFPDTSDVLLVMKTGASESYSKIPTQAVT

SLLCLHDYLIFSDMEQIVAGEYIADALNSVLPEAQKDNKDFDLYRRQKDCPVDQETCNKD

TNAASAGWALDKYKNIHMAEKAWAAKPGFSWYIFIDADSYVFWSTMMPWLKTLDPNRPQY

LGSIAYLGGFPFAHGGSGYMMSNALMKQMFEGKEGVANDWDQEAPKTCCGDYLLARAVDN

TTSVKPQHMVSCNACRNLSRGSGIDFPSERIQNADQLRDAQWPTINGEKPYTLQYDDRQW

CQPIATMHHLTAEETAAVWSYDKQRGFDSEPMRIKNLFHQFMEPRLQDRRSDWDNLSSDM

YYLNATAAEEVLAKEEKKEKLGPDELKFDKGDIDRSKGKNWHLSIDEEVAFVNVASCERA

CLTKPDCMQWKYKPGLCMLDKRASIGRPKIEREDKYKMTSGWMVDRIKEWAEKKECNGKV

TWPKP

>SoG_01306.T1

MLCGTPSVTTPIGAEAMHGELRWHGAISQRAEAIADAAVQLYSDPTRWNLAQDAGLTLLA

ERYRREIHGPALIERIQA

>SoG_01310.T1

MKLFIALLLSGFAAAVDVAPGRYIVQLKAGITTAAHHRDVRSILRKRDVEVKSFNIGEDF

SGYVATSLTKEDAVALADRDDVLSVEPDIIVSIDDIVNTQQEIRERDLTTQLAIENWGLG

DISHRQANVGNYVYDDSAGEGQTVYVIDSGIRTSHDDFEGRARFGFDAINNSPNYYPDEN

GHGTHVAGIAVGKEHGVAKKAEVVAVKVLNANGRGLNSHIVSGINWAVNDIVSRNAKSTS

VINLSLSAPAQSGNYSVVENAVKAAWNMGIVSATAAGNNDTAASRVSPARIPETITVGMT

KANRWRSQEIPGESGSNYGPELNLFAPGEDIFSASAFSDTGSYSMTGTSMATPHVAGLIC

YLRKLEGGLTSPASVKARLVQLGQKGVVNDPRGSPNVLAYNGNGI

>SoG_01338.T1

MASQTNGNGAKPQDKFDPNFTQHVIDLMSPETPPRAREILTSLIKHMHDFCREVELTQEE

WVYGVNYINSLGQAYKKNRNETWRVCDILGIESLVDEVNHTIQTEDGRKPTSSAILGPFW

SPETPFRDLGASVVQDMPKDGELTYFHGKIIDVDTGKGIPNAVFDMWQASTNGKYDVFDP

ENQTRHNLRGKFRTDADGKFYFYCLKPTEYAIDTTGPSAELLRVMGRHPYRPAHIHMMVT

HDDYVGVTAQLYPNDDPYLETDTVSAVKDDLLLDFKPIKDDPKGAVLEVEYNVRLLSKKY

KPDNTMLMSNANQDNF

>SoG_01339.T1

MKFTIPISIVAFAFGVLGDPTCPGGLTIKTSTGTFTGTVDDKFPNTRQWRSIPFSQAPVG

SRRWLPPGKLPPSDEHRYSSRFPASCAQFVTADAGMHFWASDYTRGNLIYNGAQNDSSGL

VAEASSEDCLHLAVWAPASDAPVPEGGFPVVFFMTGGGFILGGVDIPWQMPTGWVERSQS

HIVVAINYRLGIFGFPNARGLAGEDFSQNLAILDQRMALEWVRDNIQAFGGNPGRITQWG

RSAGALSADIHAYAYHDDPIAQGYWVQSGSAAGSAYIDDKTYSNFTFVARQLGCGAPCDN

SNDEAAAKFELDCMRQVPFAQIVNFIGQYTDRHEGAPIAFTVTLDEKIIFSDYYARGEAG

LVARRPVIASTTANEFGSLFPWPSRNWTEGPPQDEVDKWNVMLSCKAYNLTRMRNLVDVP

VFRLQYAGVFPNLNPYGWLGAYHASDIPASFGTYHLMDFIAPSTRLQADTSLAMQDYFLA

FVKDPHRGPQREFGWEPMDASRPNGGTLLRLGAGGKAVQLIDGIEVDGVCEGKREYDSFP

E

>SoG_01348.T1

MDILKVLSRGTKKTSRPGAATVAQLPSAGAQANPQLFHDPVRGLKRKRSDEQAETKTTTE

DLPEVDFFAPKKESVSKKDKIENASKGKDVVPDPAAQLQLSEDECRQLLRSHRLKVTMLS

KREDDSKVTKKKKNKKKDRQEEKSGKKDPKKQLTPQPLSSFAELRSVYGISRRIAENLVT

EGYRVPTEIQLGCLPLLLQPDLALKNEGGLDSGVDLLAIAPTGSGKTISFLLPIFNNIMR

RRAQAKLQGIHELEAVVVAPTRELVHQITNEGRKLLDGTGLKIMAMKRRMNIACEKQEVI

ADSSDEEDEDEEEEEDEDEDSKKLQKPKSVAKTDVLVTTPALLHKFLQEGPDGTARVLPT

VRDLALDEADVLLDPLFREQTLGLWAACTDPTMRLTFWSATMGSNIESLVTEQLEKRAIE

HAMPQKPLIRVVVGLKDTAVPNITHKLTYTASEQGKLLALRQLLHPRSKIDDGPPLRPPF

LVFTQTIERASALLEELKYDIPLEAGGSTRIAALHSGLADSARASIMRKFRAGEIWVLIT

TDVLARGVDFAGVNGVVNYDVPGSAAAYVHRAGRTGRAGRTGGVAITLYTKEDIPFVKTV

ANVIAASEKQAGKKGDEVEVKKWLLDALPDVAKKDRKILREKGVEARRTGRKGAEISTKS

GWERKKENNRKGAAEGSKRRKVGNKDGGSEPKKSASREEWGGFDD

>SoG_01351.T1

MKPSGRVANLAFTQCQYGAHQLQRLGVWLAAPSGGVQGGDHHHQSGYWIIFIHGGAWRDP

RNTFDDFKPSIEAVLNSDKISETSIRGFASIDYRLSPHPSFPQNPGETPAADLRVAKHPD

HILDVRAALAYLDKHYGIGQDYIVIGHSAGATLAYQLLMGEAALAGQTMVPAPLPRVAIG

ISGIYDLPGLVSRFEGLHGDVYRLFVTGAFGRDEQLWKTVSPANYSGKFSWPGGKIAMLA

WSPEDTLIDEPEIDRMTNKMKTDGSSLVVVKDLTGDHEVVWQQGDQPVAATLDAAIVRDE

HSMTPPPQSCAPRYSPAAWSMIAPPVGGPANVPHAQIVTTIPNLVPIFRGSFVRLASAPR

NMPWLAPVMIPKNALKTYMSVLERTPVQPKRMAAKSA

>SoG_01379.T1

MVRLTSAAILTCEYSKASLKANITDVEDLGVAIVCAAPSPQLKPDPAGARNVGNGQGRQF

ITGACLSNADCASGCCAGLNGGGICSGPAVGNAQGKTGCGFGGSGGGRQAAAPASSPGAG

KGGKANKGKKANKGGNKKTNNSGNKKANNGGNASAAGIDQSAPGAGNVGTGKGKQFITGQ

CFSDADCASGCCAGRQGSKKGACSAVLVAKADGKTGCGFVQA

>SoG_01384.T1

MPSLPGFSDNPLRTRSDLVRATLALLRPLLPRFSNGKGRIRIPISTAAHFDETAAQLEGF

ARPLWAVGALLLGQESTTAAVDGDDGLDAEINEVVQPWIEGFANGTDKSHADYWGTMEGT

DQRMVEAEILAFALLAAPRRFLGHLNDRQRCNIADWFGTLHGKPMPDTNWRWFRVFANIA

LVKALGRPLEEVQEEIQSDLDLLDTFYRLDGWSADGPWLSPEKQDAENRMYEETGRRDAV

GVGRQADYYSGSFAIQFSQLLFVRFASDIDPERASKYAQRARDFGSGFWRYFDSAGSAIP

FGRSLTYRFACGGFFAALAVSGVSTAAGTPLESPGAVKGFLLRHLRWWAAHSEDIFYPDG

TMNIGWLYPNMYMSEDYNSPQSPYWCLKTLIAVALPEGDPFWTSQEEAYPDRSSGSVQLL

RAPEQIHCNHPQGNHHFMLTPGQFVGWPMKASQAKYCKFAYSSAFAFSVPTGPLIQQLAP

DSALFLSRDGRETWATKWKCEEVRFTDLNISSGRGPGTAVTAARVKWYPWGDRSVSVDTT

LVPPTDLWPDWHVRTHRIRVHKKIETLHAVEGGFSVLGRRESDGSALPLIQELMPTAQLG

HEGVLLETGDSVLILSSAGASGIVSSSTSSPGSVESPVPQLSAMKPDSNTNLACQRTLIP

VVEHSAAAVGGLDEGTELLLAGSYFAISTAANGGWKGDGEPLLKRWNMRPRVRWEGSESV

GIQVE

>SoG_01401.T1

MGGSTKPADMTLRSKAGLYAAAAVLRLLLFIFFPALPDLLTGRVEISTPVTSFKRLQEGL

FLYNHNVSPYDGGVYHQAPLLLPLFSLLPSVRAWPIFTAILYTIVDILSADALSKIAESG

EAGQSRLYSSPRRAKKASALVVAAAFLFNPLTIATCIGRSTAVFTTCATLHAIARAVHGS

PLNAMVAISFASYLSMYPLLLLPPLILLAFDRQPAGRPRMDSLVAFALKNVFVTVICISL

LLAMSLLVTDGSWEFLARTYGIQLTLSDLTPNVGLWWYFFIEMFDSFRAFFLAVFWLHLT

AYVGGLSIRIRTQPLAVLSILLGIFSIFKPYPSVADTSLFLGLLPLFRHIFPLMRYTFVA

GAAILYATFLGPAFYHLWIYAGSGNANFFYAITLVWSLGQSLLVSDLTFALLRDEWEVER

PEMKGKEVKQI

>SoG_01415.T1

MTWSTTATKLSSTILRIAALAFFRLFPAHLPSIAFAFFAIYVPSFVSSYYADTQLEVIGG

EADVLVEEIKEATEPQLTEDGFVQPEPAVVVEEITVNETLVLAEKDPKPWQILLSGVPSR

RHPIATAATFLINVLMVALTADAIYRIRLFHPAEDLSFVRVGYVSHTDAKFLIREPDQSK

MPVTLEIHIKDPQAPFDNPLWQTAGGVRWTSNDTDFTASIVVPLLHSKQRRYEWRTSNNH

SGEFLAAPKPGQMPELTGGKFTFLSTSCILPRFPYNPFDHPLAIPGARHLAAKLPELQAQ

FMLFLGDFIYIDVPRQFRESVDEYRMQYRQVYASPDWPGVGQNLSWIHVLDDHEILNDWS

SNSTGVYRNAVQPWHTYNADLNPPKAVAAGSRKARPTATWYEFVQGPASFFMMDTRSYRS

SNNLPFDAENKTMLGAEQLADLLAWLAKPAGRGIRWKVVASSVPFTKNWPVNVKDTWGGF

LAERKKILEAMWDAGAHGTSVVILSGDRHEFAATKFPPPADSRWPESAAAHEFSTSPLSQ

FSSPLGSYKQTDDEDVMMNYIPSGNSKFGAFTMEKVGGQDTLQYRLFIDGKQRWETVLSA

APETDTTRGSFFGKIKFI

>SoG_01419.T1

MSVLSKPLAAALALAAGVAAHGHVDHVIINGVRYDGYDSPKFHYTPNPPNVFGWTIEQTD

DGFVEPNAAGHPNIICHRDAVPAKSHVELAAGDTVFLQWNTWPESHQGPVVDYLANCNGP

CETVNKNDLEFFKIGNAGLIEPGGAQSYNGYWAADQLIDTGDAWSVRIPPNIAPGNYVLR

HEIIALHSGGNPNGAQFYPQCFNLKITGSGTAKPAGVKGTSLYKANDPGVLFNIFTNVQS

YPIPGPTLAPGGVSWVPQTRTSATATSSATPHGGSGGGGGGGGSPPTSSAPQPTTTSGGG

GGGGGGNVSSPPPGSEAFVVVRVGPGQLAAQAAHAAEVTTTTRSASDTPSIRFEPWTEAH

GPSVEADG

>SoG_01421.T1

MAGNSELPTVEKPVEKGKGLHPSFHIVNWIFFSNVTILFNKWLLDNAGFRYPIILTCWHL

VFATAATQLLARTTSLLDSRHSIPLTRRFYLRTILPIGLLYSGSLVLSNLVYVYLSVAFI

QMLKSVSPVAVLAISWMWGVADPSRTQLINILFIVFGVALSSFGEIEFSWIGFIFQIGGT

LFEAMRLVMIQVMLSGEGLRMDPLVGLYYYAPVCAVMNIFVAAVSEFPRFQWADVQQTGI

GMLFLNAGVAFFLNIASVFLIGKTSGLVMTLTGIFKNILLVVASILIWNTQITPLQFLGY

GVALSGLVYYSLGYEKLAQGYTLVRNYVVSPGEATESTEGSRKSKRWMLVAVPVVLLSTV

LTVLVVYHGHDTIKSQMEKIKLPEWFGSS

>SoG_01439.T1

MAIARPIRGLLFVAIIVWCVFLWQVFLSSAPVISGPEGKNVNFDRDPNLDATGEPQGKLT

RTSEGYAVDAENPERINATILALARNDEVDGMVQSMIDLERTWNHKFNYPWTFFNDEPFS

EDFKKKTSAATKAQCRYEQIPNEHWATPNWIDDQIYEESAKVLKESGVQFGNMTSYHHMC

RWNSGMFYKHPALKDMQYYWRVEPNVHFFCHVDYDVFRYMQDNDKTYGFTINLYDDPKSL

PTLWPETVKFLAEHPDYLHDESAIKWLTDDTARPQHNQKAQGYSTCHFWSNFEIAKLDFW

RSQQYEDYFDHLDRAGGFFYERWGDAPVHSIALGLFEDRRKIHWFRDIGYQHTPFFQCPN

SPKCSGCTPGRLTDGEKWLHEDDCRPNWFKYVGMD

>SoG_01447.T1

MAPSEDQQIINQLQSNWIWAPDWVDSSSQNTAARVVRFCRELNLRSRPTRALLRFSADTR

YKLLVNGTRIAVGPARGSPLIWYYDSLDIAQHLKEGHNEVCFVVIRYFAASRGGMPFERT

ALPGLTVIGDVEADGQSMSLDSREGWLAEVDSTTTYPMGRPDDVFLHINEIVVPGLPSGV

VKPVPYNITTRNGELSPWRLRTRQIPVPEQTCVGVSAISSTTGSLAVDEWTAYLAGKQPL

ILAAESSHTIEVLAEVHSTAFIRWKFKAAQKSQIHLRVTYSEGYEHEPRSYPFFRTKSDR

LDATNGHLIGPYDEITLDLPPSEAVVHEPFWFRTFRIMRWEITVGPEPVELCSFDATQVN

YPLAVKGSWEEPGDKDAQRIWDVSIRTMRNCMFDGYSDCPFYEQLQYSGDSRSVGLFHYH

LSGDDRLMRQAMTNFAASVTAEGLPQSRFPSHVPQIIAGFSLYWVLQVCDHHLYFGDAVY

ARSFLPRIDGVFEFFETHVDSLGLVSGFPEDVWQYVDWVTAWGATDEHPDKGVPTSGRRS

NRHTYFSLLYAYVLKEAARLVRDVGRPGYAEEYEARAAAVVKAVRTHCFDGKFFTDSTTD

VAGEGAYSQHCQVFAVLCGAARAEERSRLLKEAFANTKFSKCSYVMMFYALRAFAAAGDE

VYESAWGRVWDPWRQMLANNLSTWEEDDVRQRSDCHAWGSVPIYEYCTELAGVHVVAAGS

SKIVFSPRLHLSQEVRAKMALGSSNVATVSWSSADNGRKKVELRLERPIHVISRMPGSAE

EDHGIVDRVNLVV

>SoG_01461.T1

MWANDFRPKAFEGLSAPPVDMDIRNQTVRPELAWNPASSKKEVFGRIATVSMDNLQSYLY

NKPETANTTMLFATASSVTVGVYAGANLVDRNIARLFVPLYSILNKAGLTASKSALIQVC

SGRVADEMFGLVVTADQDFKAVHHAVGQWANGSCVDTSTYAERKNVNETTILVYEQPELK

PANHSTPNITRSVLQARADCRTVTIVQGDLCGDLIKRCGVKLTVAEFEKLNPTKNFCNNL

QVGQRACCTTGTLPDIRPKKKADGSCFSHQIKSGEYCALIAARYGLTVKEIEGFNKNTWG

WNGCDYGFWPDNWMCLSDGTPPFPSPISNAICGPQKPGTKMPSDSQSKDWAKLNPCPLKA

CCNIWGQCGTTADFCVDTNTGPPGTAKKDTFGCISNCGMSVIKGSPPQNFIKLGYFEGFN

LGRSCLNMDASQIDTSYTHIHFAFAMLDNDFNVYQQDAMAEYQFQQFKKIKGPKRIISFG

GWVFSAEAPNYQILRHAVKPENREKFTDNLVKYVVDNGLDGLDIDWEYPSAPDLPGIPAG

APSEALDYLGIMSILRRKLPKDKSLSIAAPASYWYLKQFYIEAMSRSLDYIVFMTYDLHV

QWDAGNKWANPGCPSGNCLRSHINMTETVNSLAMITKAGVPSSKILVGISSYGRSFQMAD

PSCTGPMCLFTGDRQTSFARKGRCTKTAGYISNAEINEIKGRTFIDGSQSMIMVDGDLWV

SYMDDALKQSRINTYKSLNMGGTSDWAVDLAKLYPPPQDAAKLGIGWSAFKENVLGGRGG

TCPDAKNPKGAFTRKTCSDEAEYWEPAKEQWDALECKSLWYWLMQVWKGCHEGKGSMRFD

YAVNSILGLRGSPQCWNMLSTGSCVNVQSCKGVPVTGGHLIWNSIVGVHDIFKSYWKLLE

IVGNNLTKKQEEFVTTFAPKGANGGEVVELLFSFLQIPYGIGLSRVLGSGVSPDLVKRFA

PAEGARASLLESLAGMGVDTARDALASAADAREEIAFNEIFEATIASWQNQTNFILEQIF

SNEGKGYKLISTLVEDGKMLAQPNGEDRLSPGSYMSTKVLDLQRAFYGAGIPMLWKADRR

GPVVADFGPGCEIDARKYFDVGPSTYNVAWECINGHSYILAGVREGRQESCGIIFPDGPE

CPPVLQWTFDLLPGIDLLKEAGGDWQTSVREIIMGAVKTYESTGKKNRLEEQVPMSTSPS

DLQEFMADQNVDIGKVPGLVRIPVCSPQTAEANLKAGIDWYTEEHGSAANWPC

>SoG_01463.T1

MEGDTCEQISTDWTITIDDFIALNPDTECPNLEAGRMYCVDGDRPVPTSTAKSPSSTTAP

GNGIKTPEPIIPGMTSNCNKFRLVKTTTTCQGIVDYNKITRADLVKWNPILATNCDSALT

LGSYACAGVIGGSTEPPPTTTLPSTIPGNGVSTPTPIQSRMTKDCNKFHLVKSTTTCQGV

LDYNKIDLANFIKWNPAVKSDCTNLQLGTYACVGVIGGSPAPASTTPSNGVKTPTPTQSG

MVQNCNKFHEIKSTTTCQGVLDYNKISLANFIKWNPAVGSNCQNLWKGTHACVGVIGGTP

TEPSNPTPTPTQAGMIKGCTKWHFIKDTTTCQGVLDYNKISLSNFIKWNPAVGSNCQNLW

KGTHACVKGP

>SoG_01470.T1

MKLNTKVVEGRWDEAKGIWNLTLEDQTTKQTWQDWAHCLVNGTGILNSWKWPDIPGLHDF

KGEKMHSAKWNLDTDFKGKTVAVIGTGSTSVQIVPALQPVVGKMKVFMRSSTWISPPFGG

GVLESDLRKGEVDDQPGQRQYTFTDADKEKFKNDPEYFLFFRKRIEAEINSLFGMYQQGS

EMSETFRKVITDEMNRRIGPGHEELKRFIIPTWAPGCRRISPGDGYLEALVKPNVEPVYG

GIERITEDGLVTPDGREHKVDIIVCATGFNVAFRPAFNLVNGSGQSLNEDWGDRTNLYLG

VSAPRFPNYYTIVGPGATWSSGTLLPSIETTIEYSIQMMKKIQHENIRSIDVKQDALDDI

YAHFDEFHKTTVFQENCRSWFKDGKIKNRIYLWPGCVGLPSLSLSLSLSLSNTLQWLLSL

Q

>SoG_01475.T1

MAGYNRPMGFSERRGSLLTEDLMLNTAGSPSGDQRLDKAASHHHGQQRQIRGPPTPSMST

PAAEFERDLEPQLTGSPPPPPTPAASPGPSHYQPDWSDAADDEDFFLSTVRKHFKNCSGP

QRTRVLADLLNLCTSQQLSFVHQFVSPLLKKDPFTSLPDELCLRILSFIDDPKVLARASQ

VSKRWRDLLSDDMTWKNLCVKHDYGRRLSEVCTSTAAFAARHPLVLPTTTESTDTEMTCS

NGHPLTTSGASYPSGSKSFDASTPGRPRLRSYKSHFKQRYLVEAAWRSGGASITRHITQE

GGVVTSLHLTPKYIIVALDNAKIHVFDPSGDQQRTLSGHVMGVWAMVPWGDTLVSGGCDR

DVRVWDLQTGACLHTLRGHTSTVRCLKMADENTAISGSRDTTLRIWDIKTGLCKNVLVGH

QSSVRCLEIKGDIVVSGSYDTFAKVWSISEGRCLQTLQGHYSQIYAIAFDGKRVVTGSLD

TNVRIWDHTTGECLGVLQGHTSLVGQLQMRGDTLVTGGSDGSVRIWSLESKRAIHRLAAH

DNSVTSLQFDETRVVSGGSDGRVKIWDLKTGQLVRELIAQGEAVWRVAFEEEKCVALALR

SGRTIMEVWSFSPPEEMFNDRPMSLAQQRVLEEDRSRPMSAMVLDFKTAESEHEGSSREP

DVDMMDAEPGPSQGQSSEQRNDEVSGNKTFFDAD

>SoG_01492.T1

MAPPPLENVRPMFDLKGRNYVITGGAQGIGFAATRAICEMGGNVAVMDIQAKPIADFDEL

ASKFNVKTVYIQTDVTKEESLEASFAKAIQELGSIDGLVPSAGIAIDKPFVDQTWDEFTR

IQEINVRGTFFAAQLAVKQMLKQGNGGSMVLLASQSAHISLPGYRMAAYNASKGGVFMLA

KALAVELAPHRIRVNTISPGFVDSDMTRTVRETKSKREGEQMWLAPPMQRLSNPNDLTGA

IVYLLSDASPFTTGTDIQITGGLHCGTQDGLISYDN

>SoG_01508.T1

MQLLPAEITSIIQDETSTRLLDAVAEAALSPPLTDYIFIHFEDVFADICARWITKAVDES

RRIRVASALARVLPFAPYLSVFIAQPAALSRRPQHASSHAQPLCLHLPDFNCISTIISEA

ELTQLLITGWRLIHFDLKTFRDTIPAGSMQRLFRHASKAIRYLAIRIYCELLRASDLKLQ

QLLKEHVGESEAVPADFDGRSIDFCFLTLHERTRAKRILLAREERQYRDQESAPWAPAQT

LSRYVVSYEDTVLPRPLGPIGDPSHLIMTSTTRRNLRNLAKILQTDDPVLLHGLPGVGKT

SLIHEIARDLGMYSDMVTLHLNEQTDAKMLVGLYSTDSKPGSFQWRPGVLTTAVKEGRWV

LVEDLDRAPTEVLSTLLPLIERKELLIPSRGEKIKAANSFRLFATVRTLKGMNGRESLPS

LIGMRFWQTLHVEPITATEVEQVIVGAYPFLRKFVTGVLAVFRRLTTVHNGQSTRSSGRP

ITMRDLLKWCRRLQDCLQTAGSKTGEEPISETTRDWMFLEALDCFIGSTPDVKTAHALGH

AIAEEMHLDKDRADHYMAANIPPLEEDETRFSVGRTCLRKNRFANRMRKSKRPFASTAHA

KRLLEQIAVAVKLEEPVLLVGETGIGKTTVVQQLAESLGHKLIAVNLSQQSEVGDLLGGF

KPVNARNLAVPLKEEFEDLFSATGISTTKNQKYLDQIGKCFAKGQWARVSKLWKEAPKMF

LQIVAALERKQDEHVQARENDHQPSKRRKTGSKLEALLDLRPRWDAFDRSLEQFDIQIAG

GSGNFAFSFVEGNLVKALRNGDWVLLDEINLASPDTLESIVDLLTGPQERPSLLLSETGE

IEKIMAHPNFRIFGAMNPATDVGKRDLPVGIRSRFTELYVRSPDADLKDLLNIIKTYIGS

NAKNDQAADDIARLYLNTKRMAEEKRLVDGANEVPHFSLRTLTRVLTYVNTIAPSYGLRR

ALYEGFSMGFLTLLSRESEAMLVPLIHHHLFDRHGSSHSLLSQAPKHPNDGREYVKFRNK

DRDRQYWLFQGNQEPVQRADYIITPYVERNLLNLVRATSTRKFPILIQGPTSAGKTSMIE

YLANFTGNKFVRINNHEHTDLQEYLGTYVSGADGKLKFQEGLLVQAMRQGHWIVLDELNL

APTDVLEALNRLLDDNRELLIPETQEVVKPHENFILFATQNPPGLYGGRKALSRAFRNRF

LELHFDDIPEDELEYILQQRSINTSPPDCRRIVTVYKDLSRLRQTSRLFEQKDSFATLRD

LFRWALRGADTREEIAAQGYMLLAERVRNEDERLAVKEVIEKVFKVKIDLQALYSIDALP

ELKMIAGQTNSQGVVWTHAMRRLYVLVARALRNKEPVLLVGETGCGKTTVCQLLAEALSK

QLHIVNAHQNTETGDLIGSQRPLRNRGAIIDALDQDIRTALAVAGLEPHALHAELLEQYK

SLSDEASEAIPDELRRRIATNEMRSKALFEWSDGALVEAMRDGQFFLLDEISLADDSVLE

RLNSVLEPSRTLLLAEKGIDDSLVVAHSDFQFFATMNPGGDFGKKELSPALRNRFTEIWV

PALSDSEDIYQIVDAKLDEKVKHFTGVIIQFASWFGAKFRPNSATTFSVREILVWVQFIN

QFESENPLVALVHGAFTVFIDSIGANPSALLAVEGKTVNQQRLLCLEKLSELAKEDLASY

YQTQPDLAMSSEFLEIGSFSIPRLPSGAADPDFAFHAPTTRLNAMRVLRALQMKKPILLE

GNPGVGKTTLVTALAQACGQPLTRINLSDQTDLMDLFGTDVPVEGAEAGNFAWRDAPFLQ

AMQKGEWVLLDEMNLASQSVLEGLNACLDHRGEVYISELDQVFKRHPDFCLFAAQNPHHQ

GGGRKGLPASFVNRFIVVYADTFTEDDLALIASHNFPDLPAEVITNLIRFVSQMDHELIV

TKSFGSQGGPWEFNLRDVLRWLKLLTTPDPVLRTRNTDDYLDIVIRQRFRTQRDREQVDR

IYQDVMLKAPEPHSLFHDITEDFGQVGLAVLKRGHNTQLEPFPRIDVVHRLSELESLMIC

VKQNIPCILSGPSGVGKSALLEHVAAITGNALVVFPINADIDTMDLIGGFEQADPLRELN

AALRDLHQFLQESVLSLVPAHAPSVALDLLHLLQGAAVDTESIAPMREAVNSLLNQVPSD

SEVGKSLKNVSILLDRPMVLSDPRFEWLDGVIVKALEKGQWLVLDNANMCNASVLDRLNS

LLEPNGFLSINEHCGPAGEPRIVRPHPDFRIFLTTDPRYGELSRAMRNRSVEIFLGAMPP

TLDPSTKLVPDIESRLARYLTMAEFTDALGPRSKAQETALEHLHCSDIAALPRYQSEALS

FSLRSSEELSVVAEYVAYLQSPAGNAMVSSIAQFYSQSTDVPQAVLHQLPINPLNDPPIA

NLLQPKEQARWLSTCLDFGRELFIMKNKLDSLHRQAQSSKISSLSRLQRSLVSDRVVAVS

KDSTVGLGQCISLMMSAMEALLASIPNESAESLNRTLGLRIVASYLNRTIDMANGATFDE

AQFQAHLALGSKLMHGQILNLTDSIGQQFATTIVRLLDQSFAQEFTLKTGLSMERLWVTF

RPILVPNQESMSRILQIIDLGKRFDQLRWKTASSLPSLSKALTTLEQSFQIARSDKPQLD

ELVDSLQSAIESFEAHTASDAPAHEPYLAQQFDSIRQLVILQSLRLSRDSHQALEIPQES

LDQTVVTLSNVPSRSSLLLSGMEGPASRLQAMDFLTVTGGQVWDGKLAASTWLKLRELDS

VTLGSLALLEAELPVLGRQLTKFSELVIDDPLLCLNEVLQQLIIDLFASYDPTIRDTYAS

MRSGNREMQHFIDALTDVATTISAPDMREVSQQHLLPALQAIDSARHREADRAHFSALAW

VHFSTGFVTLFVPDRMFDPEIRPRMERDFHKILLEGLESKIGALREFEEFFTGRTSNLRI

HGLQKECEAIGPLPAEVLPVYRPETSDLSRLQAEFSNILKIAMGPSIVSLLKQDASSKPD

MDELQLVKGNLERLLDRLGARFEAYQDITQPTSNLLRCMLIGLSMFEAKAELRKPLDTPV

YFSKVPFLGDSRKSNETVASHSFGSLQMMRTASCIEGLRDLGLEDRQTISECFHVFFLEW

QQRLESDRKAEEAKASLYRFRGSADDEDEMDEREFHELFPSYDDEDEKKPSARHEDVRDT

SRRVAEIHAQIFGSQSNLPAEAIHDLIEDTTERVCQSVKHRVFMDHGWNRSLLSGVVTLL

QEKQESLTSVIANRQYNFYTDANLEEARQLVALANKIKSRFRELQLVDEIGHMQPLADVI

AACDKLLDLVHTEPLAKILTRVEQLHALVYEWQFGGWASKVYGSPVLYDALTETIVRWRR

LELSTWAKLLDTELGKCQDDARSWYFIAYEVVIAVPLSMVESISELRQYAISLAQNLQLY

FSTSILGQFKARLELLDQLQNHVELLAQDHPSLTIIADAVANFSSFYHRYETTVQEAIAK

GRAPIEKKMKDVLLMASWKDTNINALRESARKSHQKLFRLVRKFRTVLGQEMKAFIEQGI

PDREQPASVRPVKPSEHLALPAGSVQELKSHLPGWLDTHKRINNMPQTVSIMRRVDDKVA

ETCAVSHLLNDFVDNLHSSAAELRKETPSTLTEENKSLVKHLKTRKRKLFADALRNMRQM

GLQHNLAQDRLTDQDSTAKIFSTVAPVKSFEDPSVPVADYYFDKVIDLLPKIRDAARDHS

EDLTPAEVGRSIGFAEGILHLLLSQRKQIALAIRDFEDLHRSTREFASIGALQEEKDLLM

LQHNNSNCVRRTTWFVSLLSYAITLIKTHERLSAGCGNVAELLQENVHDLQTQLTKLKNC

QQLPVSLTSKHRSDLESTLQEKCSQSRQALVQYGREWPSLCYILDPLLKWTEADSQAAEL

RTEPFALQEYADTLSTLCDKVLVAVERAKRNCGLEPVVETDSEWLTKSNNKLFSMLTGLR

MSVVQAEVDSSLPILLGSLSSDASSSVGAVSAARLVAPILEQYVACCRNVIQQLCDNHRA

AAYMGFKVGSLFLQITSQGFCTPQEKSDEESGADGKVESGTGLGDGEGAEDISKDIQQDE

DLSELAQEANKEPGDDVEDQKDAVDMADEDLEGEMGSVAGDDDEEDDKKEGDDNEEGEDD

IDEEAGDVDDLDPTAVDEKMWDGKDEEEAEKDQQGDKAKGPKKDDEQMASDEKPQKEDKG

PQELDTADTEDTQSEAEEADEEHEEVKEAAEMNRQEQTAEENDALALPEEMDLDLDEEAS

EIEEDDEMDQLSDVEDDKMEDTQKPGSEEEEEGATAEDVRQEQEQDGDDQVSEKGDEEDD

LAGKDDGQPTDELKEEEEPQVDEEMEGQNEQAPQSDDQGPTDLDNAAPSDVRGSGEDQNA

DSKDIEQQFQSNAANQEEGKSGDGAADHAATAGKDGQMSRSDEPMERGEPETDESQASTN

DPFKKLGDALERWHRQRSDIQQANSGEDAQPKNDNEQSQEQAHQEFQHLQHDDDAVDTQA

MGTAKADEVQPVDETMGIDDDMEDPASRLLDDTKAEEQTEKDADLMKQDEKEEPDADVKI

QKDADDGRSGVQTRQGNYHRENSPSQEEAVIGEGQDSDETVDEASTQLSTTHITDESRPL

RDYDEAAKQWSDFQNRTQGLSLSLTSQLRLILTPSQTTKLSGSFRTGKRLNIKRIIPYIA

SSYKRDKIWMRRSIPTKRTYQILLCVDDSKSMGESSSGKLAMESLVMVSRSLTMLEAGQI

GVLGFGGDVFPAHSLTEPFASDAGAKVLRNFTFSQDKTDVALLIRRTIDTFKEARQQTSG

TGSDLWQLAMILSDGLTPSSAHDSIRRLLREAIEERIMIVFIIMDDTEKKGDSVLELKEA

KFVTEGGESRVVIERYLDTFPFQYYLIVHNLDDLPGALAGLLRTWFAEVST

>SoG_01511.T1

MRPLQPLILASFALLGAEARTGHGFVGIGIYNFDPKCAFACRDAISGATLNCSVVHSAHD

MHGMNMAMSGMGGGGSGVMVMTDPDCYATDEAFLTTLAWCISTRCNDTSIADLERYWQTF

AVGSDDVQRSPEHTYSESLAMVNGTPNVAYESGSLNQTSVVPYDTWWPSFYTSRVFWDQE

SQQVRYGFVLMLSGVVIPIAFSLLRFIPFPSAWRSKFNAYIVDPPAFGSRTHDYSSSAIL

RLGHMPTRGQTLFICYLIVVNTVLSAVNYELADPNNWFPDDGRRWMEMLVSNRLGLLSFA

NMPLVFLYAGRNNLLLWITDWSHSTFLLLHRWIAAISTLQAILHSLIYLVVYARAGTHNA

EAQKPYWYWGIIATLGMTLMFPLSARPIRRKVYELFLAWHVAISILVVAGCYWHIVFRYS

HKWGYETWIVMCMAIWAFDRVFRVARLLRHGVRRAVITPIDGDYVRVTVPDLSVSGHAYL

YFPTVTWRVWENHPFSAAASFVPAGSSSSSSSFSNISSDKQPQSADKAEVAGIHTSSAPV

EGSDSEAQCSPPGRHAVVAEPGKAHEAGTVFYIRCQAGLTSSLRNHRSVPVLVEAGYSSH

SFISSEPAGRKSPRLLAIAGGVGISAVLPALRSHAGTSRLYWGCRSHALVDDVRRAGLLS

HVDADVTVGRRMSIREILETELVHGPRVEVCVLACGPNEMADEVRNVLAEIVRAHQVKAN

LVVESFSW

>SoG_01517.T1

MFSKIAKAILFLRLVAYSIGLILDNYKRNRATQRLSSSSSSSSSSSSSSSSSSSSPSLKD

PEAASQPRTIVIIGASFSGYHAARLIASSIPVDGSWRIVIVEPNRHYQFTWTLPRFCVVE

GHEEKTFIPYGAYLPERARAAGIVSWVHQRAASIGRTTVTLQGTGEEIPYDYLVVATGSG

VGLQLPSRVGSVDKQEGSRLLRQMQRDIKEAQRLVVIGGGAAGVELAADAKDKYPDKSVT

LIHSREAVMHRFGPELQVEALKGLRALGVDVILEERAVRDEEEPGMLKLKSGKKLAFDFC

VNCSGQKPSSELLRDIAPGAITETGHIRVKSTLQVDHPSLPNVYACGDVAKTGVRNANAR

AAMKQAQYVADNVVLAVRGEEPMKLYAPAWADEVIKLTLGLSKAVTHFSDGKTELLMRSK

EKDETLMVGQVWTHMGATPYEDSTDAGENL

>SoG_01520.T1

MLSHASILNNGRLIAERMGLSPEDRIVVPPPLFHCFGSVLGYMATATTGAAIGFPSPAFD

PHATVRMCSEWDATGLYGVSTMLVAVLEVLDSSTSPAPRNLRKGIVAGSSVPAALMRTVQ

SRLGLEDLVICYGMTETSPVSCMTTPHDPFSKRTTTVGTPMPHTTVKIVDPADPSSILPL

NTRGELAASGYLAMKGYFNDPEKTAEVRRVDADGRVWVHSGDEAEMDEDGFVQITGRIKD

LIIRGGENIHPLEIENCLFQLRGVKEVSVVGVPDQKLGEAVAAFIVPVKGWETAEGHGGS

PGGNVLGKMDVRKWVAEKLSGHLVPRDIFWVDDYPKTASGKIQKFKLREMAVELLKAEAS

A

>SoG_01533.T1

MSDQFEKGHSVPVTKQSFPGKDVDMPNPQPTHHEIPDDIGGGKTTYKAAGKLKGKKALIT

GADSGIGAASAILFAREGADSTIVYLPEEEEDAQGTKKEVEELGANCYLIARDLTKKEHC

KEIVDFALDKMGSIDILFNNAAYQMMVEDILDLPEEQWEHTFNINIHSFFYTSKYALKHM

KRGSTIINNASINAYVGRPDLLDYTSTKGAIVSFTRGLSNQYVGKGIRVNAVAPGPVWTP

LIPATMNDEAQKNFTSPMGRPAQPSEIATCVVFLASSDSSCISGQTIHCNGGTIVNG

>SoG_01543.T1

MPQPIPTASRLLDLFNMKGKVVVVTGASGPRGMGIEAARGCAEMGADVVITYASRKEGAV

KNVEELIRDYGVKAEAYKCNVSDYEDVQRFVDEVVAKYGKIDAFVANAGATADAGVIDGT

VEQWNKVINIDLNGTAYCAKAVGTQFKKQGHGSFVITASMSGHIVNYPQEQTSYNVAKAG

CIHMAKSLANEWRDFARVNSISPGYIDTGLSDFIDQETQALWRSFIPMGRNGDAKELKGA

YVYLCSDASTYTTGADIVIDGGYTCR

>SoG_01549.T1

MDTNASNNRLYLNFNGNDRLGAAPNDRTYPTTPSTFPQPVFPTSSQPGGMQSQQGYNAGY

APNAYFQQGQQYQQQQQQQPYGGQQMNDYGNAQANAYQPRSNTPGTNDPNTGLAHQFSHQ

NLGGAARNAQYNSRGPSPSQRPRTAGATGQPGAPSYANYPAVPSQSAAPAQSFQPAPERN

PDRYGSNANSNQKKCSQLAADFFKDSVKRARERNQRQSELEQKLQDPNQNPARREQLWST

AGRKEGQYLRFLRTKEKPENYTTVKIIGKGAFGEVKLVQKRGDGKVYAMKSLIKTEMFKK

DQLAHVRSERDILADSDSPWVVKLYTTFQDAYFLYMLMEFLPGGDLMTMLIKYEIFSEDI

TRFYIAEIVLAIDAVHQLGYIHRDIKPDNILLDRGGHVKLTDFGLSTGFHRLHDNNYYQQ

LMQGRSNRPRDRSSVAIDQINLTVSNRSQINDWRRSRRLMAYSTVGTPDYIAPEIFTGHG

YSFDCDWWSLGTIMFECLVGWPPFCAEDSHDTYRKIVNWRQTLYFPDDITLGVEAENLIR

RYEESSVLH

>SoG_01554.T1

MTLRGSRSFTPLLPFLYPSLIPRSSARLPHLACHQARASSSTTAHDDDSTSKVNDLPESR

LNPRPEDYGAANFADKATLTLYAGRGGNGCVSFHREAFLPDGPPNGGDGGAGGNVYIQAA

HGETSLHKLSRRRFIRAGRGKHGQGSAKSGSRGEDVIITVPVGTIVREIERTDPAAEEEE

IFQLYRAAQRQRKKEERKAEEEKRIQLKKERQARDAARAAAVEAGEIVEDAASASDIDHA

EEVDTEEWEEGRPLEEDEEDPDADDPQREKWLMYPGMSKNDIKSAEFPRLPRRDRILKQP

PAPIYLDLSRPTERPYLLATGGIGGLGNPHFTSRSITRPVFATKGDDAVTMKIELELKLL

ADVGLVGLPNAGKSTLLRSITNSRTRVGNWAFTTLQPNIGTVVLDKYSGRPTVNLKPSLS

PTDDAESGPRTRFTIADIPGLIEGAHLDKGLGIAFLRHVERAGVLAFVVDLGAGNAVNAL

NALWREVGLYAEMRENEERVREVDSLVEWDLSSEASGTINLMSHSGPAPPRNTTPVQEIA

GKPWFVVATKADLPGTQENYKELQKYLESVTKGEVPHPSGALGESWTRDCRAIPVSAING

QGVERIVHWVAGLLSE

>SoG_01575.T1

MPSKSSRDWGQQDRGKPQRHTSSRREHRPPDQHHHRTPPKPLIRTDTTHSHYINMLLSQD

EIPKWHTITAAICVWLLLAGFLVFPGTFTSLQETIDDRGDDNVSKAGEAILGTVKNIPLL

VVGAVACGISAVGMLALLIRHRINFVWLLNRLLIPGMANSLAGLISTLIGVYTQQHGSWS

ITAKVTAIIEGVYLATCAALFGFFTYKLTKLKKRHQTFYEDSGRRRWKSPEERRTEQQKR

EEEIAALEPGGSIV

>SoG_01580.T1

MKIVVIFLAFITAVLQVAAADPPADDFTCPCDPGWGAQWSQSENCPLNVCCSKFGFCGTT

ADFCGAKKVTKPSCGGTSSSKRSIGYYEGWSIGRACNGMTPEQIPVGAYTHLNFAFAFVD

PKSFKVAPMSQADTELYPRFTALKDFNPGLKTWISIGGWSMNDADQPTATTFSDLAASSS

AQSAFFSSLLSFMQTYGFDGVDIDWEYPVAPERSGKPADYKNYVSFLKNLKAALGGSGHN

YGLTLTLPSSYWYMQNFDIVSMEKIVDWFNVMTYDLHGTWDSTDRFIGPVVNAHTNLTEI

DLTMDLFWRNNIDPDKIVMGLGFYGRSFTLTNPSCTSPGCPFTGGGKPGRCSASAGTLMY

SEIQEILAGGATPTLDKKAAVKQLVWDTNQWISYDDKETIQMKIDYANGKCLGGTMIWAV

STDTTDGIAANAYIQTNGLSQRSLFGGGGNPKQQDALSTCIWGECGKDCPAGSLPAQRSD

GKNRGNAGIYTGCETTGNSRNYCCPKDQEIPTCQWRGTAPFCNGKCKSGEVQVSSDTSGT

GSECWTGHKVLCCTSTKSDAGIGQCKWEGAAPFCGKHGRVGEHYGCDEGDRYEETYDSLG

AGGESYCFSGYKSFCCTKPPPFQNCEWTSKEHSFLHPFTCPNGCPAGKQIIAQDPSKLSF

CSTGSAFYCCDAPVEELPDNDSSLNFCASSDGDYVLSISDTSTNNYDDDGNPADIIELYW

YEDDVFVVPNDASPDNMKRGVDAIDELVSLSVDRRWSRETGLSNHTFSQVCLSRDECFNI

LEPELPEVQRLISEIRLTWEYTGTFRRGHHDDSSLVSRARTTVVKLKNNRRTRWTASTYY

NVAQLAANGRQFFANGGLKKAAICLQNIGAFGARQLARKYVAEHVTELQTPAMFAQSMID

NKYPDGTVPSGVSSGYNWEDVFGDNGYVHMTWKDLGIAQPAGLVGDTPLEALYHALGSKG

SNADYTNLLICDAQTNSLKTSAWALHTNIISNDRWNKATPATRLAYLNNLDQSLIPYLNS

AEAQASLKHGYEVQQTIWKALDSSSKVTGPGSSNFASLHKTWYQRYFEEFDNNLQVFLKA

KLDKEVTAWTGGSGAYGLYKSVQRNKIAQIPNDVA

>SoG_01611.T1

MRTSIALALIGRFLTGVAAQISLPAFEDKDFNVTQALLDQGIDPVQLPPTASDTTLSVNA

HCAAACASLSLMFGCNTILTSRNPAYAAFTSTYWSAQQADVEPACIFLPSVNTQVSILVL

LSRLTRCPFAVRSGGHAAMRGASSSPNGITVSLQKLNHVKLADDKRSVVIQPGNNWGQVY

AALDKQDVIVTGGRVSLVGTGGLTLGGKNSLPVH

>SoG_01613.T1

MSSTNLPTIRWGIIATGLISSWFVADLILDRPDAKAKHIIHAVASSSIEKGKGFAAKHLP

GHSPRIYGSYQEAYADPEVDIIYIGTPHGFHKQNCLDAIAHGKHILCEKSFTLNAAEANE

VLAAAKEKGIFLMEAMWTRFYPLVQSLQDLVHRQKVIGDVLRVSCDFSLDQGIAQLGPSS

RLKDPALGAGSLLDIGIYSLTWALLGLEPPQKDGNPAEEMPKVLAAQTLHDGVDVGTSII

LKYADGRQGIATCNSNFRTTPPFCLIEGTEGKIAVGDDAAAAPSYFTVFKTGGEEQKYEF

ERPGKGFYWEADAVALDVTAGRTQNAIMPWSETMRVMMIMDEVRRQGGAKFPQDEK

>SoG_01621.T1

MAQTSTSTPRSPRSSGSSFTWILLAAAPLLLLASLHYPGARDVICGNGSSDVHPAVTIKQ

GTIVGKLLDDGKYPAPIEGFLGIPYALPPVNNLRFRPAVPVPEGNGTLEAFFMGPRCPGR

QLVPFLEDPLLGPDAESEDCLTINIYRPLGHDSTRNKLPVSVLIPGGAFNRGAARMHNTH

TMLAHSPEPFVAISMQYRIGVFGGLNTELTEDEGLLNLGLRDIYAALEWVQENVAAFGGD

PEDVTIMGLSAAAHAPKKLFHKAIMDSGAHTARAVHPPSADLNTRHFRELLDMTPCSHYT

DLKDPAILTCLRALSSETVDSAGKEVFARSDASVRWAWQPVIDGKIISRRPLDAWKSGRW

NKVPMLTGSTHNEGSYYVPRKADSPADFSAFFRELLPHMSAAELAELERLYPDPATDPES

RYADPTLASHGAGSQFRRLEAAYGHYAYTCPVRQTAIWGSGTAPPDPPVFLYHWAVNKTA

VFGANHGDQMRYQTYNPEVRGFSPAQDEISGKFHSYCVSFIVSGDPSATRSGRFGAREEW

TAWTPTGANTMLLGEGNDEMAGGTSVGVAAQMVDYEWGKKEYRTPDLISEERHSLTPPPP

PPPPPPRC

>SoG_01625.T1

MTAASQSQERNCLVTVGATVGFPALTSAVLEPAFWANLCSKGFTSLRVQCGPDIQKASEQ

VALRSGDIPSGFKVEVFERRRNLLKEEMVLCKKGEGRSQGLIISHAGTGSILDAWKIGVP

LIVVPNDALLDNHQAEMAAHLAKEGYATACKADLADLHGAIEESETLAKSNKDRWPAHSL

HGTGNGTSLWSLAPEDVAKEQNMQMIHD

>SoG_01634.T1

MEASAKTINRLSQRLLPDQPHHLSIHPDWRYKPHEDRRPDRPEEWGNVPLQYMTLLSDAD

RGVLLTRSYYDMREETQRPVPKEVSSLARGGEKKKLSLSDYKNKKIPGAASTSPPEPSIA

KKREADAGAGASAISNGPSPKQPIEPRNSKPINGSDKPMHASSVSSAESRLPPKPSKHPL

PPRPPTPEARKRIADPDDDLRHTKRPRPESDRLAYEKTRTARDDVLRRRDPAHDRTPARD

GRVSSASSSLPNGRSMLKDATGPGRNSPAANSHSRNSSLNGIRHNASTSGGMRNTPTKVD

VSSKASVPPLLSPLHFDLDDMEADVVERREKKLRERDRERERERERERERERERDRERDR

EREREREREKERERERAREREKEREKERERERERERDRERFRESGRDRDDRPTKLKKPVD

QLIKQERSPVKLPPLLSPTLPPEIEDAIEKLRKKQPSSSSDDGEPLKKVPPLQLPGKKPL

PGNFSEDEVDTKPVPRKKMLVILRIPKRLRRDVSRILKMGDRDSLRQETQRSPEVPGARK

RPPAGEDGAGGNESLAAKRPRPSDANPILPNPATPSKKTSIAMSRVSSANSAANTPGEPA

SHQRPSSADMPMANGTARPPPPISPADQARAERLRTRETGYLSLGKQLKKEGESLIALGR

NRSDLSRGKIDVKLGCVKYLEGLFAFALAFQTQYTQRQLVRQYVSASAWSSILPAVDALR

ADIRRLGSAGSSLAALVVLLQARAFEEILKCYGTYPSPESRVRSEELLRIVNQRAKALGQ

AREVLADGPTGRGGVLKGVEIGAWLTVEDMCEAAVRVMKRWCAEEEVNWTPEMSASNRGS

>SoG_01661.T1

MVSHTPFLAKEFKSSYGPKYSFQPNYRGITGQQATRVALKAGLFGGAIGVAALFFVSGIP

RIQKDILMKIPGMRGWYEKEVHPADNVSEKLSSQDTRDYVKQLLTPGK

>SoG_01662.T1

MDPSSSPPRPPITGASLFWLIGLIPPLLGLHIGLSPRGNTQRWRWLWYLGPVALLSLIWV

HVASHHVPEPYLDEVFHIPQAQRYGQGKYWEWDDKLTTPPGLYAFSLILLLISGAKSRFE

TLTSFDLRATNVVALLILGYLALLCRHEVTHLLSPTSSTGPPKPVSSYTLHTAMNICLFP

LLFFFSGLYYTDVASTAAVLASFLNHLRRVRRGTAGEEMGFISDLITLILGIMTLFMRQT

NVFWTVVFMGGLEAVHAVKSLNPEPAIKGAKSDGTIIGEVKYFLRRSCIGDVHDVSLSSA

WVEDWVLTAVSLLAGGICNPFRVLKATWPYIAILASFAGFILWNGGVVLGDKTNHVATVH

LPQMLYIWPFFAFFSLPLLLPYALTILDRAWSFLPLARKPPREQTPKSAKSVPFRSLILA

AHYVLGTIVVSLAIVHLNTIIHPFTLADNRHYMFYVFRYTIRRAEWVKYGLVAAHTVSRW

LIWGVLSGSADTASATTSTGKGVSRLDGGGSNTLVPTSTGIIFLLATSLSLITAPLVEPR

YFIIPWVIWRILLPAWYIRGGVDGGMIQAAQGCNRGVTKRILAYSQKHDVRLVLETAWFM

LVNLATMAIFILKPYQWRAEDGKLLDEGRLQRFMW

>SoG_01678.T1

MPPSFRLEIENGQFRDGHGRQVMLRGINVAADAKLPSEPNLPSHEPNNFFDGDNVKFHQR

PFSKDDAHVHFSRIKRYGFNTIRYVFTWEAIEAAGPGKYDEEWIQHTIEILRIAKSYGFY

VFMDPHQDVWSRFTGGSGAPMWTVYACGLNPESFSATQAAIVQNTYPDPDDFPKMIWSTN

YYRLAAATIFTLFFAGRDFAPKCVIDGMNIQDYLQSHFVNACAHLAKRIHEAGDLEDQVV

MGWESMNEPNRGLTGYLDLTVIPKDHPLKKGTCPTMWQTMLTGMGRACEVDVWEMGGLGP

YKTGTTLIDPHGQIAWLPKDWDDTKYGWKRDAGWKLGECIWAQHGVWDPSTDTLLQKNYF

SKDPTGVDIDYPRFTNTYWLDFWRRFKKACRAQHDNCIMLMQYPTLELPPEIKDTEDDDP

RLVFTPHYYDGITLMTKHWNSTWNVDVLGVLRGRYLHPAFAIKIGETAIRNCLRDQLKSL

RQEGIDRSGNHPCILTEFGIPYDMDDKKAYKTGDYSSQSAALDANYFAVEGAKLEGHCLW

VYAACNDHARGDQWNGEDLSIYSVDDKQPPLSSLPRTLTNYGSTSDLGKPVTASHRDVAD

DNLVTPENLQRTLTNQSLSSMRSTKDPELTNTPGLRAAEAFIRPTPVVVAGDLVSYGFDM

RKCTFALTVRAEKHAADDAPTILFLPDYHFPNGHCEVAVSAGKWEIRSDESEGVLLQRLR

WWHPEGEQTIQVNGMVRKHNEPVGTEEDAGYLEQCQSNCSVM

>SoG_01680.T1

MYQGGHRHGERPYQVPPPPPMPNPGAGGHQMNGMTQFPPPPPRYPSGGAPAPAGMVAPPP

PGPPPGSAMGQQAPWHSNFGRVYDARGALVPPPPPSSGQHQPYNPQHHAQLAGQPMSIPP

PPPPNEQMSATYIPHGDTYGEVAGFPAFGPDDWAGASGATMPAPNFYTTASNPQSATDTY

QTTSLDSAQTRGLSNASSATNTTSTSTSNIPPEVAAQWPLETVLLWLAKNQFSKDWQETF

RGLNLYGAQFLELGTKTTGRGNLSLMHKQVYPRLIHETTSSGTGWDPAREREEGKRMRRL

LRNVNAGTTVEGYGLGNHHTHNRRESGTSAPPASLPSAGTDQAESPNPLQAPGPGFSAKR

YSQSRSTTMPTLTNNTMSSDSGYRTALKGFDGDRKNSPNHGEGNDAGPFRGAAMRTDSPE

GSPALSSGTFAPAMSASPNTTKFPHRAARLSMDSQSSNAAIYGSGVPAEAASMLSRSLNL

NEALASARSPVERPRQSPVDSGSAGTEPPSSAKGQGFLSFLSRKKRQKEDGSHPSPDDLE

SPTLEAFAKPPFGNRSATASETNLDRSSHSSIPRGRSDSQGKRGPRLFVLATMDYWTYRM

CDVTDAETAADIRQVVCMNLGVGNFDGSSIYLTELGKFDHSEPLDDQKLLLQRKTRADAA

GTLKFFVAPAWNQTNTDGSTKAGSGVPLALSPGYLPPGASMDAEAYDQLNGRQRSSSSPP

SSRSNTLLGEQVDGQALAQEASEYRAVMERKQREYLAKRKQAGKGQSPTAPEVASGYGIV

GRNVDFDQPRNSPFEDKKPDQLFPQRKPPAPPSDPSATLIKANSLSRKPGHNGRASSGSM

EGVPTPRHPTTWSPENEPDRARKARYQGASNAAVSGIGAALVGMGRNLGAVGQSSANGPR

GVSPGRAPAATEDAPQSGKRPSPTTSHSKLDSGTGVRKPKSGSPGTLTWSPGSVAFMVPD

YVRGQPARLEEWSPPNSPTSAHAELPPAIVPPTSQSSPSMQQRSTFPLESNRRRSGQEVE

KGDRDVQFNSPRPTARQAVPPQVNDSDDDSDDGLFAIPLASRNKGKAADKPGHAQRPSLT

VKTQRSKKNLSVSFNSPQSSVLEGTDETPGTRTTGSSRRTPMTPASDTWESEQSEGKLNR

RKSFIEKDVWANRPPTDALLNNLDDFFPNLDLDQPVLEDGVDGEPGPSPIPESDENGEGA

APNSATVQDPRASASAIPPSRQPSLYTNDGDTLGSDESTLKALERPSSYQGVAQRSMRRS

GGLGRMKSIREVARGAHEANKRYTNASQDMGKASGSGPGNMSSNILRRKSTKMFNANIVQ

IRPDQRGSMVLPPVPQDTLPKRQTTFRWFKGQLIGKGTYGRVYLGMNATTGEFLAVKEVE

VNPKAANGDKAKMRELVAALDQEIDTMQHLDHINIVQYLGCERKETSISIFLEYISGGSI

GSCLRKHGKFEEPVVSSLTRQALSGLAYLHREGILHRDLKADNILLDVDGTCKISDFGIS

KKTDNIYGNDKTNSMQGSVFWMAPEVIRSQGEGYSAKVDIWSLGCVVLEMFAGRRPWSKE

EAVGAIYKIANGETPPIPEDIQETIGPLAVAFMMDCFQV

>SoG_01683.T1

MAPQDALIVRQQAEPDHAVVKYSAEDLSRPKVGPANPFKDENNGLKRKNVLTGNASETFI

SEHTFRSKHRAIEREGGPEREHQSGAQLKAEAARTRAGREGKGDATIAEGDGAYLGPWAK

YKRPEFEVVNNEAELGSDEEYEIVEEEEEEDIVESGTVLQAPAQAIARRKEVEEQGEETT

KFHGSEQYDYQGRTYMHVPQDLDVDLRKEPGSITNYIPKKQMHSWKDHSKAVTALRFFPD

SGHLLLSASADTTVKLWDVYHDRELLRTYSGHTKALSDACFNASGTQFLSASYDRMMKLW

DTETGKCISKFTTGKTPHVIKFNPDPDHAHEFLAGMSDKKIVQFDTRAPPKENLVQEYDH

HLAAINTIVFVDQNRRFMTTSDDKSLRAWDYNIPVPIKYIAEPDMYPMTRAVAHPNGKYV

AYQSSDNQILVYGANDKFRQNRKKSFRGHNNAGLAIELDVSADGQFLASGDSAGFVCFWD

WKTCKMYHKLKAGQQAVTCVKWHPQETSKVVTAGMEGEIKLWD

>SoG_01685.T1

MSTTAQTSVMDPQPKPQAKIRKRAPKACLSCRARKRDLCCHQQGLQICSHLIPSRRSQHE

NGDNVQASHPPYPPESAAEQHSAEAVVKSRLNGLPAPMPSNRDQDSPCHPSAEPHANEIS

SQGSHCQPLDGQDDARQRKRSLPSAIPLASASGEPATLVVKHGDWHQADRFSVASTPFVG

QAATQGIPTFNASYPAGASWTAESQIGLSSDVVYSYYPFLTIGNLPNLPPQDVNFLEMQG

CLRVPTRAILDEFLQQYFLHVHPLLPLVNEGDIWDLYCQNPNSTSPTERISLLLFQSILF

ASCNFVSKTGIKSLGFSSIRAARAAFYRRAKLLYDLESESSPLAISQSALLLSYWSPTST

RKPNTAWLSLAIQHAKSAEAHHYAAMPVFSASTHPLQRRKQNILKRLWWCCIIRDRTLGL

LMRRPINITRAHFDFDSNPMLGYNDLANEFERSKVYTVGTKKCLGEVLSQLIELYVVLTD

ILMMVFPLDDTPGWGREMKPEDADKVRECKTALRRWYKDATLRFPMFGGGPMPRIVTESN

KEFQHDSVILYTNLMYMYYHSTRVVLCHHEVLHLAILQAAPNADMSRDLSRIYENRHELQ

DAASGVTECLKELVHLKLARWLPISAVACTALPLVLNILDVKLSASASSANQTADAAAAS

KQHRLNVLIEAMKTYQPQYDGVDWISEIVRHIVNLSQLDNTRPQDGSKPKINDWTDILAS

QPSAYLRLALALDLSLSKGRLPEDGDFPVSLRGLFTGGFSPLKALIEANRPNQQHSRVAA

PASVMSNDTGGMHQSECDDHSHGSPDSHMSNADDLANEALRDQVRDHIRQDRPASTNAPP

SEGIQGLGANDNGGVFGEAALGGEQDESPSASSEFGGMYIDGPVPDVSVEWIENLWDEMG

DVEDKTDRDTAKFLLEALKDGDMGELA

>SoG_01689.T1

MAPKIAIVYYSMYGHIKALAEAEKKGIEAAGGTADLYQVPETLSEEVLTKMHAPPKAADV

PVIDAKKLAEYDAFLVGIPTRYGNFPAQWKTFWDTTGGLWASGGLYGKMAGLFISTGTLG

GGQESTAIAAMSTFAHHGVIYVPFGYAKAFAQMSDLTEIHGGSPWGAGTFAAGDGSRQPS

AKELEIATIQGTAFYETVAKYSG

>SoG_01699.T1

MGISRRPKNKGATGGGASTTAKPKKATFDVAKKKEIGVSDLTLLSKVSNEAINDNLKKRF

EGAEIYTYIGHVLVSVNPFRDLGIYTDQVLDSYKGKNRLEMPPHVFAIAESSYYNMKAYN

ENQCVIISGESGAGKTEAAKRIMQYIASVSGGEDGEIKQIKDMVLATNPLLESFGNAKTL

RNNNSSRFGKYLQIHFNKAGEPVGADIVNYLLEKTRVTGQITNERNFHIFYQLTKGASSQ

YQQQYGIQKPETYLYTSRSKCFDVDGIDDVAEFQDTLNAMKIIGLRQDEQDEIFRMLAAV

LWIGNIQFRENQEGYAEVVDQSVVDFVAYLLETTSEQLIHGITIRILTPRNGEVIESPAN

PAQAIATRDALAMAIYNSLFDWIVERINRSLKAKQSTSNSIGILDIYGFEIFEKNSFEQL

CINYVNEKLQQIFIQLTLKTEQEEYAREQIQWTPIKYFDNKVVCDLIEQIRPPGIFSAMK

DATKTAHADPAACDRTFMQSINGMSHAHLTPRQGNFIIKHYAGDVSYTVEGITDKNKDQL

LKGLLNLLQTSGNKFLHTLFPHQVDQDNRKQPPSAGDRIRASANALVDTLMKCQPSYIRT

IKPNENKSPTEYNSPNVLHQIKYLGLQENVRIRRAGFAYRQAFDKFVDRFFLLSPATSYA

GEYTWQGSYEDAVRQILKDTSIPKEEWQLGVTKAFIKAPETLFALEHMRDRYWHNMATRI

QRMWRAYLAYRAESATRIQRMWRKKRTGAEYLQLRDHGHKVLQGRKERRRMSLLGSRRFL

GDYLGLNASRGPGAQIRQAANLGTNERAVFSCRAEILEAKFGRSSKPSPRTIIVTNSKFY

IVAQMLVNNQPQISVEKAIPLGSIRYISISTSQDDWFALGIGSQQEADPLMNCIFKTEML

TQMQRVMPGGFNLKITDHIEYAKKPGKMQQVKVLQDSQQRTDFYKSGAVHTTQGEPPNSV

SRPTPRAKPVPPRPITKGKLIKPGGPGGRPSRVAGNRAAQPRPGVSSTRSVPPRPGIGGG

AASSTPAPTPRPVAQPLAAAIPSHTRSQPSTTTIASTTPATTRVPPPPPPAAAPAKPKPM

AKVLYDFAGHEENELSIVKGEVVEIVQKESNGWWLAKKAGQQAWIPAAYVEEQAAPPPPP

AKAKPAPPAPPAKRPAAGRKPAELHPRDSGMSLNGANGSDGSRSSTPTPSLGGSLADALL

ARKNAMQNSRKDDEDDW

>SoG_01701.T1

MSGIASLIFSLGTYLAIGQVTKSTYFCFSATDSRPRTISLQFAGLALDALIVILFWRMLA

WSRTTKLKLEALGSTLVLSSVSITLLWMGHRMFHGARSLEVAFDSLYGFDLVVDSIAFAT

LAMSSTLWVCDSAIVVPSTVLTVLVGICATLSNVQQLGTWEKLSRSQVLLPPTLIFCGFT

WFSYTSDIKTFVFLRRIHVSILLASFLATITIYSMARHAPSFERHPLKDLIIKGNIEESR

WLRWVKTSHDLPIALQVYRDRHEGRNPPPYFHEWYRLAKDTAVVDEFRQIDRDLSPFRKV

SPKHLRQRVNIIARAPGVATITIKSSGQVSHSDAGDKGRNIELDRLVEIISKFAQYLPDM

VLPMNLGPSPRVLPSWHEAHSTAGFDLSTVVGLLQPRVSAKNSNAGGGSNEMQEATPWPL

VSARDLQDMHLAACPPASRSRTSPHWRISDFCFACARRHSKGPIMTEWDKSLEICYQPDI

YHLHSFFMTNPEAPILNELLPLFSPSKMDGFSDIVFPLAGSIDSQSDIGMPLSDRKNELF

WRGKVGGQRASPQVLRGSQKLRLMHTVTQPESDDEVTMVLPFSEGEAQDNAGETQEEKEA

FGYARVSAKKANEAAPLNLQWQQASTCRDPVCTMAEKAFPLTASPASTEALEHRYILLTD

EDNGPPLGVIGVLKSKSIPFLSTTFRTWYTERLLPWLHFVPIDTRYQSLHTTVAYFTGTE

GQAVVSGTTRDMKARLDDAQWISSQGQKWAEKALGQKDLEVYLFRLLLEWGRLIDDGRDK

IGFWQDSQGEMQSSGWTPAQEEGTTS

>SoG_01703.T1

MAYEPRGDRGGGDGQDGGFPKIRGRRPVTDYSATILHWQHNRVPNYKGGYIGEAERPSAS

YIVDMIPPAGRPNRPADSIPSKHLHSSLNKIKHPVNVVRWTPEGRRLLTASTSGEFTLWN

GTGFNFETIMQAHDSAIRAMEWTHSDDWLVSADHDGVVKYWQPNFNNVQSIDAHSDPIRD

IAFSPNDSKFITASDDSTLKVWDFAKGEMESKIEGHGWDVKSVDWHPTKGLVVSGSKDHL

VKLWDPRATPRCLTTLHGHKSTITKVLFEKVRGACFATSARDQTARVFDLRMMRDICLLK

GHEKDITTLAFHPIHPNLLTTGGMDGSIYHYLLDTPNPPAGQALTVSPYDSPDPDSVPAQ

SIWPAHKVTYAHDYAVWSLDWHPLGHVLASGSNDRITRFWSRARPGDTDVFQDRYHIGEA

AAEAQGTWDRRGNRRQRQEEEQLEFEDEMDALPDQNASRPAVPGLPGIPGLPLGGGLPGL

GAIPPPPVIPGMASGAAPPPPLPFPLPGLNGAPPPMIPGFDPENPPDPQMLLEIMKKAGM

QIPPPGALPPGFIPPPGSIPPGGLPPPGNFPFPPPPPPPPPGGTDDARRRGPLPSQEDSL

RQEQRAGRYTRAR

>SoG_01708.T1

MLANFVRNGMMNPMRVMLIVSFVLFVFGLTFFGTVMRQYRYEPLQPVNVVRPLNKTGAND

PTELLANLWKPFVQGITEQNYKAADGKEYRLPDGDQIWTEPLGGNVLILDVDSRFTLDAG

NLLNDSRMHMDVVEKKTAGRLAHLIYAMIHGYDYRFVRAPNFPDRHGTWAKVPIIREAAK

QYDTVVFLDADAGFVNMDLPLEWLMNYWKIKPETSISLAKDPDSPHNQDSKGLVLLNTGF

IITRKNDRTEEILRALEDCVDDTTYPGCSKWKKDWAHEQRAFGEYIRYDYNKTTDILALS

MPEANGPAGVFIRHDWPKQRDPLEDVRNSFMDMLVGRAHLQFHEGLEHYYRDIGTLEHPL

NDVQI

>SoG_01712.T1

MEKIADSNAVPDIYSWHQIGDANGVPDTTVPDFNAMRESYGLPEKPIDINEYAWPEAQNP

AHTTWYLAQLERHNLRGLRANWGSGSLLHDLMADLVFHEEHEGGGTTTYRPNGEWYLYEY

YARMKGTRVRTAASEDGLFDAFAVVSGDGAKVIAGSRNADGSYEVALTGLSTALGLPTEG

TVQVRTTRFDWEGKSTDTGPPKDLGLSEHRYSGDKVCCKSTHNGRVFFFPLCLWLDVDYA

NAPTQLVIMVNPPTNSTSFAYELGPGLR

>SoG_01718.T1

MATTTGRFSGKVVAITGAAGGIGLAITKMLYSQGATVAVSDFDREALARLQSTISEVPAQ

PGQGAKFSAVDVTKSSEVDAWIGDIVSTFGKLDHAANVAGGGERPCLLGEKTDEDFDRSM

ELNARGVFNCMRAQIAHLSAGSSVVNISSATSLTYVPGIGVYAAAKGAVNMLTAVAATDY

GPKGIRVNALCPGMIWTERMQTDGAQWAKPTLDATPLKRAGTPDEVASCVAFLLSDEASF

VTGAARNLLIISSLPLQLSWVDLLVTANHGPWQSLVSLLFLLELARYISPGLGSPSSCIT

AQLTSQILILLQNPYCQFSKMKSKTLVLGCLAFASRVSAALLDADLPPLTTTAAGYDARG

TTSNPTAAPKVHELRPRDLVERQTWFFAPNNTCGYIDGDYNKPYGCGTSFRCAILTSSAG

GRGGIVCCNENAEECTSRIVRCFDRLQTVGMTGIDNFARTCSLRESPFCATFSISPGILG

YACSATKTSAEVSLSFRGQDPVKFTALWLRDGTRISQVVTDIFKPIPQISTSSDSSTTST

TSWVQTSSAVPKPDGGGGNDLPVGAIVGGAIGGVAILVLLGLGIFFYTRRRRTQATRNSH

QPTFAMQDQQQQQQPQTCTEHPRDSAHHDVQHSSPQPGTVSTPTLADTRVSYMSAPCPPI

YVAEMAGSSPAPVLCELPTKNSERREYDTQELP

>SoG_01719.T1

MHDWGAGSTRRPGCHDEQAVSRMGSQDEDSYPSAWIISLEKCPPPSQLTFVFVHSLGLRP

ETTWHDDSTGKNWVEEPEFVLGLGWRARVMSFRYNSDIASNMSAASILIHAVDLLFHLEK

AMASSDNIGLFFFAHGLGGVIVKKALGLRDLLANCPRVRAATTGVVYLGTPHTDTNSEAL

LQTVKNTVQLLRREHHAIEDDDIRHFASAVASINATYISKKPAALETISLWETEPSTWEG

PDGREVSDLVVPESCRKPDLWNNLDIAGDCKYLGLPHFPSLFDARFTKFTVDFSTLVSRV

LEHKTVIINAVRISATEVPMSGRPRSPPRMRGRRTRTGEAILARGGSDTETTDSEDEWFA

SWQKRRQYQEEQARLDTFLRSLRGWLPQNTGHEVFVTAGTCEWISSNQALSNWLKYTMYG

TFFCLAEPGSGKTHIAKALASHIEKTYPSNVVMAFFCTGNEPTPPIWDYFTWSLLQDRPS

WFSSVPKRYLGRDEGSDRLSLGDFVGIWTSLRQYEEDGAKETIWLVIDGLEACGDEAFLR

FWHSLEQLRKRQADSKKPLCFLKVLFTSTLTPAVAAVSGSVSRYIVPPAVVKDEIATYVD

ARLISFTTAGSQPSIKSMAERLIRDVKAAAEDYWLFARDAADEAELALGSNSAHVASFMS

RLPAKLEAHVQQTLLCLRQSVGPARYLYPLLGIILASGKRLSVSQLKDALSSLYPTEEVD

RLDLAELAMRHCGGILVRGKGDCLTFRHSGVARWLGRDFTQSRRRATLAYLCMNYLLQDR

FRSLPPPDEVGPDPFNWLDRNHPFYYYAARKSLAHLSLAEGDDKLLPLFVRLPLESPPQW

EISQSWLRASLDYRATREEFRTLPLAVVLRDERLLPILERVCPPPQEMTSLLCLQSAASR

WRSWHLRRNDRTPRFALPEGWLSTRNGDGYTPLLAAAKQGNIDMIRYILKWHPNVNERGG

PSMQPVLTMLYCSDACRAQPRGPSRLPAITLDLLGMGVNVNLADCRGSSALHVACDQGSL

PLVRLLLDHGAVSNISDQDGCTAWQVACERGGVDLLRELLARGVEADSMFDTGENALTYL

ARQGWVDKLRAVLPLADVNGFDLKGEAPLHVAVRLDEDRYEVLNLLISWPGVNLDLVRRL

HGEADPEQATGLLTAIKADDGHGVELLLRAGATAGHLPGLKVLPLQLAVRLGYRDIAELL

LRHRAPVNEFLFTPAKQTALSIAARAGNIDMLKLLLEYGADPTIEDGLGLPNAALLVLEC

KTADVAVLALLLECPKPPCIVQDEDAGTQRATSHSEVFMRACSLRDVEFAEALLGCGVQL

SVWLARPDLCSPLHAAARAGNLKVCRFLTEKEPRFLNFLCRSGSVTHSPLHEACLHGRSE

IVRLLLDKGADTKLLSAHREQSCLLAACIGNDTDTIKMILAADPCMADVADYNGESPLQV

ASRNGQVLVVGGLIEAGASMTRCYSNRHSCITQSLFSALGDSAIPLLRTLIKHGLDIDRP

SDSLGQTPFFCALAAAPPSVVSWLQNKGGNSLRTCRLSASPEIWEFGLRGIRRELSPDVL

EAVISPCREHLADVDWSGQNALGNFSNGWRPYLTRLFWLCEEIREETSRDLFAEMMVQPD

IFGISPLDRSNGTTSCRTRSRPDVARVVIKFIDSYLQTPSHQVLGQIIHLPAAKLMHHAP

EDIGPLLTAQYSARGIYTRPEDGSEAQSHASSTQRCRVCRKGVFEEAFWCAKCVRHLCHK

CVGKDEAGICGREEKHLWIWYALDAEVTAHSDKVQAALRRLRAAMLSIVSEQSADRLSQS

STVISAEGESGGEEARVQASLQLATLHAFGMLAIRRRLGAMYLPLSATALHIISPFEGNI

APMRKAWERRSLDEESQVERRREEMRYLTEGMMRTAYTDEKLFRQDMVLEELARLFAEG

>SoG_01727.T1

MKAALVLSLSAAVAGTRLPQYEWDPATISSCVEWYNNGMDETCEYVRVLFGVTPEEFTSW

NPSVGLDCKPWRFQSYCVVTQEKLDASSKTKTTASPTATTITSTTTSASLGPSPSSWEAL

GCYAEVAAPNSVLERRMSSETGDAVLTIPRCLDLCYRAAFPFAGVENGNQCWCGSYVGGE

FAQDSSDCDLACSGDAQAICGGKDRLNIFRAKGIDEHTTVSATASASSSIQASLASPLAS

QSTSAGKKGLRLF

>SoG_01730.T1

MRISLYQWLVPFALSLVYSLDVSRANDLPGLPTAVPAAKALKAIDTEGNADLAEWLQANM

RASPEVLRACPSQCSQAINSTDAAGWFLIPEAARLSQCNETMLLDFVIKSDNATAIRACK

ADYSSKAKIAYRPTSDKAALCPTPNHKIVETPVRVAAGLPDGNDHGIAFSTEHVLSAGHQ

ISRYLSTQVPSCTQNALVFGYSQSAVVGVFAGAELHQHGLTADILNRFLRLVEQRTISET

TFVQLCPNDGLGADYTLGIIATGAANLQRAQEVVGLWAGGKCIATHDERDWIQATIRVPP

PVRTSNSTASNVTAITKRFGSKAASILSGRAECKTAIVKADDGCWALADRCGISQDDLKR

YNTRANFCNTLVVGETVCCSAGALPSSIPPAKSDGTCETKDVKAGDSCGSMASKCGLSAN

DFMKVNTKENLCSNLIEGQQVCCSQGKLPDRRPKMGADGTCASYKTNNGDSCASIAASRD

LTVKDIENFNKKTWGWNGCDILSLGFRLCVSEGNSPMPEAVSNAVCGPTVPRPGESGPTA

APKGVDLASLNPCPLKACCNVWGQCGLSDDFCKEAPSKTGAPGTSGLRNGCVSNCGMDII

KSSPPTSKINIAYFEAWNSERTCLNMNVDQIDTNKYTHIHFAFAEVTRDFKVNISRVQDQ

FDAFKAMSGVKKIISLGGWDFSALPGTFNILREAVKPANRDAFKNSVVDFVNQHGLDGVD

LDWEYPGAPDIPDIPADDPQNGINYYKFLVTVKSALGASKSVSFAAPSSYWYLKAYPVSL

LARSLDYIVFMTYDLHGQWDYNNKWTSPGCPTGNCLRSHVNDTETKDALAMITKAGVASN

KVVVGVASYGRSFKMDTAGCDGPMCKFTGSPRVSNAAKGRCTNTAGYISNAEIAEIIASG

KVNKQWKEVGSNIMVYDDTEWVAYMDDNMKALRSKFYEMYNFAGTTDWAVDLQAFVDGSE

GGGDDDYEQEIDDDYLSECGGSYASLDSIEKSRDSMAPHCAEKYVADVEVAIMSDALKKY

SDLVKGGYDKKFQIYRNYVIGLVPLQVDAFMASDEVQKYFTCTETGTFQCCGSCRYFCGN

EGACQKFEGCKDGKGTVNIQCPQMEHELDMLDGTHIPNATFSLKDKSGFWKAIGEKYGIE

ESWVTWGRRLMRVNNGCQYAGEKIKECMDKQNNYFYSYPTANLDKVTVYNPRDIISKSYD

DTEGMLENFKIIRDFAEFDDTQPWSDAVDAMSLPSLSLQMAVENMQEIAEKADEIKKKER

EQFILNMIMGILFFIPFAGEAAGAAGLTATRSLIRLIEEVGTAGLAVYGVVNDPKNAFNT

VFGYMLGKAIDHGSLGKAANAKRGIKDIAASGLPVLAFAGINQVPVASTLQARDAKPGFP

YDDNTTSYCSFWYDNDGSMSFQDVISVFGVPLDKFLRWNPSVTTSGGNFLASRSYCVEAS

GEPTSAITGTPTSTSTHLITTHSTTESVSTIVTTTTPSNGIKTPLPTQPEIVGNCNKFYF

VNQGENCDTIASKNGVPLNDFLTWNPKAGQKCTGLWADTYACVSVIGYKPPATSTMPSNG

VQTPVPTQPGMVSNCNKFVFVKTGDSCASIASKAAISTSDFLKWNPQAGGQCKGLWANAY

ACVGILSAFRLKTRYHADCTGDVYNDVSVSGAGICINTDCKVASLEVATEGYCPDGQVQI

SYWEQPGCTGKWFGYGYSNRGQCRGLWTEGWKFKAIHLNCARKETDCVTLGTCTYDAEPS

RNIC

>SoG_01734.T1

MKRKLDQNDRPAGSEGDSATKTDESKTEPSFADFGLDARLVQAIAKLNYQKPTLVQQKAI

PLALTGQDVLCKGKTGSGKTAAYVLPVLSTILKKKQVSPGTWISSQYRRNEKLTNPLKTQ

TPQSTGALVLVPTRELADQVHKVAEQFTAFCAKDVQIVKLTDKLSEAVQRSMLSSHPDVV

ISTPARAWQNVNSGALSLESLSCLVLDEADLLLSYGYDQDLENLARSVPKGVQTILMSAT

LSPEVDTLKGILHRNPAVLDVAEPDSEGEGVTHYVVKCGEDEKFLLAYVIFKLKLIKGKC

IIFVSDIDRCYRLKLFFEQFGIRSCILNSELPVNSRIHVVEEFNRNVYDIIIASDEKSEM

FGDEKSPEDEEEEAQEQEQTETRANGAKSKKGKKEAEAEAETEDSQPKKKRKVAKKDPEY

GVSRGIDFKNVGTVINFDLPTSASSYTHRVGRTARAGRRGMALSFVVPEKLYRKHVPTSV

STAENDEKVLKKIIKRQAKQGKEVKDYHFNKDQVDAFRYRMNDALRAVTKVSVREARTRE

LRQELLKSEKLKRYFEENPTELSHVRHDGELRTARQQAHLRHIPDYLLPADGKKAVADTV

GFVPLRKQSRIANGRRGARGSGRKVRGGSRRGDPLKTFKARRKTK

>SoG_01737.T1

MNTPFVLSVLAGALFVFFVRYGWTFLTFVFKALANARPTRYRHAGKPEDEHVQVLVLGDI

GRSPRMQYHAISLTKLGLNVDLIGYKETARHPELIGKPNVALHALTPTPEWITWGNLPFF

LTIPCKVIIQFWTLWHTLAYKTEPAKWIILQNPPSIPTFHVTLVVAWLRGSKVLVDWHNY

GHTLLATGSVFKKPLVPIYKWYEMFFGRHIGNANLAVTDAMAEQLREQPFRISQPIYTLH

DRPAEIFQPILAKKDRLAFLTKLSETRSDAKNIVDGSVRLLVSSTSWTPDEDFQILLDAL

VEYANPSAENGTMDREPPSPILAIITGKGPEKARYAEKIEALQQEGKLPGIRIATAWLSN

RDYASLLACADLGISLHKSSSGVDLPMKVVDMFGAGLPVAAYNAFESFGELIKEGQNGRG

FETAGELAAILRTLLSMAGEKSLATLKEGAVREGSLRWDEEWNRVVKPIVVDT

>SoG_01742.T1

MGATQSTDKPIVHKTSKGKLKGIQRIETQTQKPVYDRFTAVPYALPPVGQRRWRRPEKLP

ADFTYNAANGEAGDYTKFPYVCPQPVYGHGAAELPNKDAAPQPETVFDEDCLYVNIWVPS

QSPPHGGWPVQFYIHGGWLQVGNANHSNNFDPFHLIQEFPRIIVAPAYRLNLFGFMSFSG

LHEEDSTHAPGNYGFWDQRAALEWTHASIAEFGGNPDNISLGGLSAGAYSSIFQLHYDIL

RPKKADRLIKRVYLYSNAVGVQPNSVNSSANEAQVDELLQECGISKSLPSLEKLEALRNV

PSAKLIEAAHRMERHTFRAGTDGEFVNPNFLADIVSGKFGRLLADQGIHILLGEVAEEAA

LYRMVNPASSYDSLVVQLNNYYPESVTKALLDHYTLPKEGASKDDWADIGSIIIADCQVH

ATIRGFTSSLLKAMPADHVMRYRISWRAKALDNWLLPSAGICHASDVPIWWACGRRAGYT

QADLDVVSEWLKPFHQFLCGKTVNWGTSGLGRIKELKAEGEIEFVDDANLQEGLRVWEVM

IKSQLS

>SoG_01750.T1

MKSFTSLFLLLLPLVSTLPLSSKEGKVVPEKYIITLKPGVKVPEISGHLNWVDGVHKRNL

DARQWIDMTARRNGKKEDRKGRGKGKGKKKKHRGVEKVWTGNFKGYSGEFDDETIAEIAQ

NHDVLFVEPVQVVDLYATVTQNSVPWGLGSISSRAPGSTKYRYDESAGKNTWAYVVDTGL

YTAHREFEGRAFLGYNAYKGSEFKDVQGHGTHCAGTIGSRAYGVAKKANLVSVKVFDAGS

VGLPLPTYSAVARLLTRSTKSTTDVVIDGYEWAVSNITNTPGRASKSVISMSIGGSFSRA

FNSSIENAYRAGVLTIVAAGNNNADASLFSPAAAPNALTVSAVDENNNRASFSNYGPLVD

IFAAGTNVRSTWIGNEGASAWMSGTSMACPHVAGLALYLMGLKTENLRTPRQVMDKIKGL

GTANVVLNGGPGSPNSLAYNGIGG

>SoG_01756.T1

MSIVNKFITRTAATDDDNIDHIGTETPGSGVATPQPDLQDKRLPGIMSYFGQVRQDSALC

HGFPTACSPPGESSCLTKELAQNAMPISVKSQHLDVGQVLKADTRTLPDLKHLVISHAQP

NAELNTVHGGAGHLVPYSIPVISQSPSSRWPNEANAQEPISTCDEALEGLAGNGGSVSRK

MEGSNSSSILRKPTAVLSQAVKSPVAAHSADNSIPPLLTRPRPPGKWFSLDGFMELTRGV

MFKSGQSTPTRALSTAQPSQSEGAQSSGRNSHDDGTASGTQTPRGSGGAQVPASKGKLTI

KIKEARGLRKCRDPYVVAVFQRSELISGGPRPGENEEPLNAATSAMAGIPIQRQGSDSGR

PMAIPMRSRQSSNTSISDYNTFRNRPRRQSFTNPTWDAEAVFDVVESDMLVDVSVYDHTT

SGEEFLGHVDFQAKKDAEGVVRGWFQLRGHADTMAENAPTGELYVEALYQRSEKRHFGPS

DFEILKLIGKGTFGQVYQVRKKDTQRIYAMKVLQKKVIVQKKEVAHTVGERNILVRTATS

DSPFIVGLKFSFQTPSELYLVTDYMSGGELFWHLQKEGRFDERRAKFYIAELILAIQHLH

QNDIVYRDLKPENILLDANGHIALCDFGLSKANLTKNDTTNTFCGTTEYLAPEVLLDESG

YTKMVDFWSLGVLVFEMCCGWSPFYAEDTQQMYKNIAFGKVRFPRETLSQEGRNFVKGLL

NRNPRHRLGATDDAEELKRHPFFADIDWDLLAKKLITPPFKPKLKSETDVSYFDPEFTTA

LEQNGSLNERAAALARGYAASTPLSPSVQANFQGFTFVDESALDENMGHRGRHDDEDMDD

AQDDDWDNMNDIDPRRANRMSGILKTGQDDQMVGGQHFDV

>SoG_01776.T1

MIDHVLGRPSSKSRRLQVLAVLSFWGFYLYKGNRHGPPPAKPLSRLLSKRLTAWQTVLIT

MMYLYAARNFSALVGLASPEPMANMYDATFFRATWVLTALDAGFWTAMKIRSKWLRDLAS

IVFSVFYLFAAERADEKVRKVRGMLTVEHLRVSWNKGTTPYLRFFQGLMRPRFTRWPPRQ

IRIPRPSSSDYKEHVDAWLYFDGPLTALSNQNKLILDIPGGGFVAMDPRCNDDKLFAWAA

RSGLPVLSLDYKKAPEYPYPFALNECFDVYSTIHQTRGRCVGMSGKQTPRIIITGDSAGG

NLAVATTLMVLEASSVTRRRLSSRGDLPPPDGMVCFYPALDMNIGNWMTDEQMSLIRDRK

MRKTNQSIVKRKSMQYNQLAGTPHHSDEEDDAPVAKAEPTTMENLEVSYEVVRPQESEDT

ESQNGKPKSPKQGKTSPGGKRELTGSHHSEPLSTRLATSSIISYFNDRVLTPELMRAMII

LYIGPHDRPDFMRDYLLSPVLAPDELLARFPKTFFMTGERDPLVDDTVIFAGKVRRAKEA

AARRGRGSSSSRFAKSLSEEGYDTPEVLLIPGTSHGFMQFPSVYPPAWKHFERCAAWFEL

LFANADAQRRRERAEQVRAKAAAAKGAESSEEDRPLEISMTRMRSSTAESSGEKDGQVDK

KINGNGKKGKMLSKSKSLVKLSSEDDLLHRRMKGLTSGLTGTIDPE

>SoG_01778.T1

MALRLSLRRQLVASLSPSAAASSRVALRAYATTSNLAEDVKQAIDREASLPNPDPTADSA

SGALVSDWAPYMVSTYARPSPVFVQGKGSYLYDVENRKYLDFTAGIAVNALGHCDPEFAR

IVADQSKTLVHCSNLYFNVWTGALSKLLVEKTREAGCMHDAESVFVCNSGSEANEAAIKF

ARKTGKIVDPSGGKTEIVSFNNSFHGRTMGALSATPNPKYQKPFGPMVPGFKVGTYNDVA

GIDELVTDKTCGVIVEPIQGEGGVHAASEEFLVKLAKRCREVGAVLIYDEIQCGLGRTGT

LWAHGNLPKEAHPDILTSAKALGNGFPVGAVIVNKKVGDNMKVGDHGTTFGGNPMASRIA

HYMVTRLSDNQLQADVATKGEIFRQRFAKLQERFPELVQETRGRGLILGLQLSEDPTPIV

KAARERGLLIITAGMNTLRFVPSLLVTEEEISQGLDILEDAIAVTRP

>SoG_01795.T1

MLFFDAIVSFATLMQSTQAHSDTAAASKTFRDTCLAFKPEKHVRGSTRTRLEFISAGTTL

NLDDNVPSCGRRSQVVRSDLCRVALQIPTSPRSSISFEIWLPIDWDENKRFLATGNGGVD

GCIKYEDLAYGAANGFASVGTNNGHNGTTAVDMLNNPDVVEDFSYRALHMGTKYGKELVE

KFYNEQHHHSYYIGCSLGGRMGISAADNYPDDYDGIVAGAPAVDFLNMNGYRANFFTITG

DPDSEDYIPISTWTGLIHNEVLQQCDEIDGVKDGIIEVANECHFDPSKLRCEPGQRAPAD

CLTDAQIKQVERVYAPYQYPDGRLIFPRMNPGNELNAVERLLAGKPFAYSVEWFRNVVHS

DLDWDPVSYGFDDVALADRQNPFNIRTWPAALPEYKKRGGKIITYHGGQDNQITAFNTER

FWERLSSADPRIDDYFRFFRISGMNHCSGGPGAWVFGQGGNAPAAGIPFDAEHNVLAALV

AWAEKGQAPETITATKFVDNEVSKGVDFRRSHCM

>SoG_01798.T1

MAFQPAAVHQEPSTPTQKLSVLHGPSDPPLLDLTLGELLSLQTYQHGPKECLVFPWTGAR

WTYNDLHQQSSLLAGALLDMGIGTGDRVGIMAGNCEQYASVFFAVAKIGAVLVILNNTYT

ATEAMYGLEFSDCRVFFTTQRIGRVDNTKLLGELESRRVNGEYSPKVVILRGETGKHTTT

YDQLIQSSRRQDHERLYKTMSRVLPHQVVNLQFTSGTTGLPKAAMLTHHNLVNNSRFIGD

RMRLSSNDVLCCPPPLFHCFGLVLGLLAIVTHGGKIVYPAEVFDIDSTLRAISDERCTAV

HGVPAMFDSLFQAELPKDFNCERLRTGIIAGAPVPRYLMELLVNKFGMTEFTSSYGLTEA

SPTCFNAFTDDALDRRLTTVGTLMPHAQAKIIDREGNVVPVGQRGELCIAGYQLQAGYWN

NSEKTNEAMVRDSAGVLWLHTGDEAVFDEQGYCSITGRFKDIIIRGGENIYPLEIEERLA

AHPSIIRAIVVGLKNKHYGEVVGAFIELSPDCPRDRRPSDLELKEWCRRKLGGHKSPAHV

FWLGEDGVPAAVPLTGSGKARKFEMAKLGDELLARRGSAKL

>SoG_01799.T1

MRNVDGSSAGGLLTTLETQTNQDTTSKVEEASSGSQTPLPSSTADLDTIWSWNSVVPETI

RRCMHDLFRDQAAQRPDHLAVQSWDGSLTYSQLDELSTKLAMHLLDQGVKTGTRIPLCFE

KSMWAVVALLGAMKSGATVSLTDPSQPEARLKTIVEQTEANIILTSAAQSVLGRRIAGNA

KVVPVSQEFLDKTSPVAASDSLPIVDPASPMYIIFTSGSTGKPKGVVLSHESYTSGAVPR

AKAVGYTASSRVFDFPSYAFDVTYDCMLCTLSVGGTICVPSEESRMDDLSGAIRSSKANM

VHMTPSVARVLEEDIIPSLDVLGLGGEAVGARDAAEWGKHTSLVIAYGPSECTVGCTINN

TVYDSTGIGRGVGGVTWIVDPDNHNRLMPLGGVGELLIEGPVVGVGYLGEPEKTAEVFIE

SPTWLTQGHGSVAGRQGRLYKTGDLVRYEDNTTNGSIEFVGRKDQQVKLRGQRVELAEVE

HHVRSCLPPGVKVAAEVVKPERGSPTLVAFLSESGTAQPGADMEAAKPSSELTEALAAVE

SSMAEKVPRYMVPASFITLNTMPSLVSGKTDRKRLREFGALMIQGSPGRAPLSEEENEEP

QTELEKKLQRAWHKVLGSNAVIFRGSSFFSLGGDSLRAMRLMATARSEGITLTVADVFGN

PVLRDMAKKADICMQEVSADVAPYSLLESSWHVSSAQKEVALMCGIHADQVEDIYPCTPL

QEALMALSAKVKEAYVAQRVVELDSIETAGRLINAFATASKESAILRTRIVQVPGRGLFQ

VIVRDDFNFHQGDDLSLYLASDRDKAMTLGQPLVRYAIISDTLKGKVSFVITMHHAIYDG

WSMPLVVERVNKAYHSKTLTRPAEFRSFIRYLSDLDRSASASYWRENLKGASSNQFPRMP

KAGYQTKADSLLEEYIKVPGTLPVNTTVATLIRAGWALVAAQYVGRPDVVFGETLTGRNA

PVVGVEEIEGPMITTIPFRVRIGSDATIAEYLQEVQSQTVAQIPHEHFGLQHIRRLSPDA

LGACDLATGLVLHPSGEDKEETIESSPADLLVPAGDAEAAQEALKFNTYALMLVCSIDPK

GFLAMASFDSNTVEVPLMQRILGQLARVVEMLCDPNVSSVSEIEALMCSDLSSSRSMRDE

GALLTLPDLPPFEGAYIVDEQISATVLPLGAPGKLIIRTSSTVDLPEVQTPPFFKRADLP

LPSGHFYDTGKLAVISLSGTISVLEAPIAAPPSAHRPNIRTKRLESSFTRREQVLRTLWS

HVLQVPEADISRDDSFFALGGDSIFAMKLVSEARAKGITLTVMHIFDNKLLKDMSKVMVE

DSSAGKRDELVVPPFGLLDLDNKDAFINSVIKPQLSDSSWAIQDVLPVRPLQGIAVKGTV

NIPRYSARYELIKFNTDMPTAGIRKACQELVARNEILRTVFVEHQNKMYGVVLDSLEVPF

VEYNVDGDVNAFCHQVCRLDVMTRMPLGSSFVKWFFVRGDKGSSLIFRISHSQYDENCLP

IMLQQLSAIYENKPIPKSSSFSRFVAHIIRENIPASIPYWEDLLSGASMTMLEPDTPITQ

RSHFAIEESVDLTGWSSDTTLASLPTAAWALTLARQCETDDVLFGEVVSGRKTDFPDAHS

VTGPCWQYLPFRLVLSRDWTGHDLLRAVQSQHVSSAAHEGMSLSEMAELCNIPGLKRTDW

FGSVVHQAVKPVKSLEIKDAGGETETVYVHEEPLREWSVQAFFDERELTIEVITLESWKD

HAADLLKDITATARHLINNPSMKLF

>SoG_01811.T1

MMTPHLELKPSTHKMLVAPPRLNLRRAASYNAQDRGPLSSTSSRFNFNHLLFSPPPSPSL

PALVPRPKKSPTQKIFATRPSRVIRRAFYLLILMSNFYCLAFFLRNQPVVRAIWPYFAHE

EFEMVGQDELPEFATPIVVADPQGHLRWTVSIPQHQDFPLSIKEYAEMSSQCREVSARAR

DMHHRKPMTEQAMLEYDAPDDYFVDIDAAEAMGILPPHYHQSKPKVGHLLGVDAHSVRGK

PVCKSSMTFILESSDAGLGNTVMMLWTFYGLAKEQGRAFFIDDSRWAYGDYTSIFTAPPL

PNCRPPPRHHMLPCPFQAEHLLVTGATAKEVFPALLAKHHRMSGTADGLRGLFELARKGF

EDLSPLYREDREYVQRRIKQLKERATQDGTQHPVIGFHVRRGDRHPFNFQYHASYIPAEV

YQETSDRLVAEYYKRHKGTRDQRRALKILASDDPTVQDEPTLSDASLAQERIRLAVEEQQ

EGRDTSKSDRLVLHDFEAEPFGWEGGFYASMFWNLGEKTKNNAPTGDMAPSALTLNLRNF

VGRAYMMDLAVLAGASDHVVCGVSAIGCRLLAVMMGWERAMEMGGWVNVDGDFGWTGIQW

>SoG_01828.T1

MPPRTDFPPVRACIFDMDGLLLNTEDLYTDAINTVLHRYSRPTMPWTLKARLQGRPGPAA

NALMSEWAKFPVPDSQWQSENAEEQKKLFPNAQPLPGVKELLETLGAAKELEIALATSSH

GGNFKLKTAHIEELFEVFPHHRRVLGDDSRIPPGRGKPLPDIFLLALKTINDSLPTGTTP

IAPEECLVFEDSVPGVEAGRRAGMRVVWCPHPKLREVYEGREDEILAGRMGEADVQEEGE

ILGEIGDGWAELRESLEGFDYGMYGIRVSA

>SoG_01843.T1

MPESGFTFLPLGAIIQTFTVNGVNIVQGFSTPEQYKSHNAPFFGETIGRVANRLSNARLD

SFNGGKTYSLEANDRTNSLHGGKVGWGKKEWKGPTPVGLREIPGVEGLEGGESVEFTLVS

EDGDEGFPGTVEARIVYTTGKVAVEGGGGKEAVVLAMDYSARLVDGADETPINMTNHSYF

NPAGVDAETYADTEITLSTTSHLPVDDVAIPLGTVTPFPGLTANQPFTLHAAEPFIDHCF

TVAADPSSVPIDTRSLPLDRHVQAYSPHTKIHLEVLSTEPAFQFYTGDGIDVPEVDGLKA

RGRRSGFCCEPSRWVNAANVEAWKPQVALKKGEEYGCRIVYKAWQD

>SoG_01849.T1

MKFTLSLMALGAVANAHYTIPRISTGGGGGSDWQYIRTTANFQSNAPVTDLSSEQMTCYE

RNPGTPASGTLDIRAGGSVTFGVNPNMYHPGPVNVYMAKAPSTAASFNGKGNVWFKIFED

KPTATSSAITWPNYTNGHAPPTDQASQTVTIPQCISDGEYLIRFEQIGLHSTGAPQLYIS

CAQVRVSGGSGSASPRLMSFPGAYSMSDSGLSANIWWPIPTNYKSPGGDPLKC

>SoG_01858.T1

MSSSEDKYETLEKIGHGSFGVIRKVRRKADGFIMCRKEISYLRMSQKEREQLHAEFQILS

TLRHPNIVAYYHREHLKISQDLHLYMEYCGNGDLGRVIKDLALKGQRAQESFVWSIFSQL

VLALFRCHYGVDPPEVGSNVLGLTQGFVAGTPKVPAGTMTILHRDLKPENVFLGEDNSVK

LGDFGLSKMIKSHDFASTYVGTPFYMSPEICAAEKYTLKSDIWSLGCIIYELCAREPPFN

AKTHYQLVQKIKEGKVAPLPEVYSAELNQVIKDCLKVNPDRRPDTTQLLNLPVVRLMRKE

KEVVDLNKSIRSREEALRKKEKELNEALANVNREKEAMRHEIDSQLRREWEVKARLEIDR

LTNAEIEHLQKRFEEEVQARVQAELQKKTVTFKIEGERSSTPQDDFSSSSKSDYPQSSIG

ASGDEFPSTTDITEYSSLDSPDTSRELKKPNTRTPFGRAQTMYAGHAGTPMDIEMMSPSP

AAIASLSLSPRRGGATKAPSANTGNIFAAANARDPDTRWDIPRDLGLIDSDDDDIMPSPT

RNIKSTKNPFTSKTRPVLTSQKSCPLNRLKTKSSSSGLISKQTGQVATPAGTASASTSPT

RRISKIPSAATLQSDGSALSNSPVLGRKPPAKNGASGSDSDSSSALGKVTAKNNLKGRTL

VELQQARAGGRPLSAVMGAPAAGAGENNGSPKRAFRDRIERRLSVESVAVWDPERDEMPS

PFLVRGRRAIIKSDS

>SoG_01872.T1

MARIATKLAALLSVLSVILAIEPPRGTHQPTGNGERLLTFNETVPSARLRPSGMSVRWAA

SGDDGLFITKNSNGDLVLENIATGKTTTFVTADQLPKDTHEYWISSDAQKMLIATNYTKE

YRHSYYADYWILDVESGETIPVVEDQVGDIQYAVMAPDSETIAFVRGNNLYLRASDGSVS

QITADGGPDKFNGVPDWVYEEEIFGDRYTLWFSPDSKFVAFLSFNETGVGTFTIPYYMDN

KKIAPPYPAELELRYPKVGSKNPTVQLNVLDVASAEFTEVTIDAFAEDDRIIGEVAWVTD

AHSSFIYRVFNRVQDLERLVVVSPESGSSKTVRERDGTDGWLENTRAISYVGSIDAKSDE

TYYVDMSDESGWMHIYLHSVQGGEPVQLTKGEWEVSSILKVDPKRNLVYYAASTRHSTER

HVYSVSYTSKQVKALVDDDVPAVWSASFSSGGGYYILSYSGPDVPYQELYASNSTKPIRT

LTTNEAFYKRIDEYNLPNITYFELEHPDGYSMNVMQILPPNFDPSKKYPVLFTPYGGPNS

QRVLKSFQLYSWPAYITSEPELQFICYTIDNRGTGFRGRKYRSAVTRQLGKLEPKDQVWA

AQQLISKNNFINADHIGMWGHSYGGYLTAKTLEENSGVFSFGMISAPVSDWRFYDSMYTE

RFMKTLNDNAEGYNSTAVRNVEGFKNVAGTFSILHGTGDDNVHYQNAAALIDLLVGGGVS

PGKMKMVPFTDSDHGFHWNGASLYRYKFQTKQLWDEVQRNPKEKELVHQWTKKSFTA

>SoG_01883.T1

MHLKISTLAFAAGLLATAGVATAQDIPSDLPVSELLSTAQSHLARGETIEALTFYDAAIA

RDPKNYLTFFKRATTYLSLGRTNQATDDFNKVLALKPGFEGAHVQLAKIKAKTGDWDAAR

AQYVAANKAADSLEVTELEAAKIAAKLAEEAAQAHQWEECVNQAGVAIVIASRQPKLREL

RGKCRFERGELEEGMADMQHLLQMKPGDTTPHVVISATAFYGLADMALGLSQMKKCLHSD

PDSKVCKKLHKQEKAIQKTFKKAEGQLEKGQTTSAGRTLMGTGEEPGLVASIKAQSETLR

ESGRLPSQAAFGLYNRVIDMTCQAYTESNHRDVEKYCTEALQQNPDSFWGLLHKGKAQLR

AENYEASINTLEQAKEADPSKRDKVDPIIGKAQIALKRSKTKDYYKVLGVAPDADEKQIK

SAYRKASKQFHPDKAAKQGVSKEEAEKKMASINEAYEVLSDPELRARFDRGDDPNSHEGQ

QGGGGNPFHGSPFGGFGGGNPFMFQQGGGGSGGGFKFHFG

>SoG_01900.T1

MLEKDTTAGGVETLKAKIDRLRPEVCEDEKVKSQPSINLWASSTLMIHTAEALAGSVETQ

VPRSAVKYTREDVGLTDGYVGSAQANDEDSMTFTAKRPPRSLDPPLPIDFETLEFLAHGT

SGAVYGIDENWVLKEYWELDRGAIERRALTRLGSHTNIIGYFGEIDAKHIVLERGEPLLS

LIQQGHISAEKKLAWIRELAEGLQHIHQSGIIHADVGCENMVIVDDRLKIIDFEGCGIDG

GEANAGYKWYNRRDLSVDLQSDIFAYGCTIYQILTGRPTFHELAASNDRSDLVRRLYAER

KFPNVQGLPLSDVMLRCWSGQFKSMGEITSTLDSSTPWRFSITRLFGSICGWIVDRQNMG

HRTQARSAIGDGKSTLS

>SoG_01901.T1

MKFSYTGFLLFASSALGASRTKAPSGCLTVSPNGTYKTVQSAVNALSNSSTSDQCIFIDR

GTYNEQVLVSSRSAPRFTIYGYTTDDRSPRANGATIVFGLSQKDGLNNDGTATLRVKANG

FRLYNVNVKNSYGQGSQAVAVSAYADSGYYGCSFVGYQDTLLSNVGRQIYVDTQITGATD

FIFGQQATSWFERADIRVVSASLGYVTANGRDSSSNPSYYVFDHSTISAADGNSVPNGAY

YLGRPWRSYSRVVFQRTSMTSVINPAGWRIWNTDQPNTSNVFYGEYQNTGAGSQGTRANF

SRKLSSPIAIGTILGSDYTQQKWFDASYFAGKGADVGST

>SoG_01942.T1

MTSITSLLRPLPLFTIASLLRLVLLLYGLHQDASSAIKYTDIDYLVFTDASRFTSRGLSP

YDRDTYRYTPLLAWLLLPTALLRPQALWFSFGKVIFALADLLAGWLILSVLRRDRGMARE

RAGAFAALWLWNPMVATISTRGSSEGLLGVLTIALLWAVERRRLTLAAVLLGLGVHFKIY

PFIYAVAIVWWMDDEHLGNKKNQKKKNKSSPGKPTHSSILDKLIGFVSVERVQLAVISLS

VFMALNILMYLIRFLFSLLTRSIPLGPSPPFDSYGMPFLEHTYFHHVTRLDHRHNFSPYN

TLLYLNSALPPSSAVHIESLAFLPQLLLSCVLIPLALAKKDLATSMMAQTFAFVTFNKVC

TSQKRRASRRGRV

>SoG_01944.T1

MGKAIRGNRRPNQRSGQQGPPHHQVQVRDEPKAAAPAPPLTMAVPEDTPRFSDLTGSSIV

HPTIVETLTQDLGFDHMMPVQAATLEELLPPNRSDCLVQAKTGTGKTMAFLLPAIQNMLS

QNRPKRGRGVSLLVVSPTRELAMQIEREAARLLQRFPEYKVCIAIGGTNKNTETSRFARG

CDVLIATPGRLLDHMNDEGTESLLSHVDTLVLDEADRLLDMGFMPDLKKIVAHLPGKRES

NRQGMLFSATIPPRVQEVAGIVLSPGYKFISTIPAGEVNTHEKVPQVLITCPSFSLTAAA

MLGAIRDEMEIVEALEGSPAFKAIVFTPTAAIADFYGRILSTLSDVPAVSVLHSRISQSK

RTKVTDEFRVAKAAILVTTDVIARGMDFPDVTSVFQVGLPADKETYIHRLGRTARAGAGG

RGIFIVAEAESWFPKWTLKDIKLQPQEPNFNAAAEVTTIVASLNDEELIAKTYKAWLGYY

KNFLKPMKWTAEELVKEGNIYASEGLQAPETPPIEKNIVGKMGLKGVKGLRVVPGVQKVG

RGGGGRPPQSKDGEGQGQGRGEGGGGRGRRRPNNRN

>SoG_01955.T1

MKTAASFAASALLVAAVTAQPHIHNPHNAHHQMHKGRQEALQDLHKRLKVVTEIEWVTEI

ETVTERIEAYTTLYLTGGQEPPAPTTSVVPPPQPEPKKEEPKQQAPPAQQSSNGDKDGVF

FEHPTTTQAPAPPPSEKPQTQPQPPAKAPEPQPAPQPAPQPAPQPAPQPQAQPEQPKPKP

EPTTSSAAAPAPAPSTGSGSGSGGGAGGATHSGDITYYTLGLGACGEDDTGADNTKNIVA

LSHLLMGTQSNGNPMCGKTITIHANGKTATAIVADKCMGCDPDNVDVSEKVYKELFGDLG

SGRMPCTWSFN

>SoG_01958.T1

MGQTLSEPVVEKASDKGEDDRLLYGVSAMQGWRISMEDAHTTVLDLLPTAKRGDADKSHP

ENLAFFGVYDGHGGDKVAIFSGEHIPSILLKQDTFKSQDYAQSLKDTFLATDRAILNGTP

FYQLLLPNTTLSTHLKRSIAPPNMRPVANARFVQTPDMRRRYQAALLVCDDARCAGYQGP

RQASIPGPQASARESVPSVLSAERVFARVPITNMSRATPLTLPDEKNRIIAAGGFVDFGR

VNGNLALSRAIGDFEFKKSAELPPEKQIVTAFPDVEEHELTDEDEFLVLACDGIWDCQSS

QAVVEFVRRGIAAKQDLDKICENMMDNCLASNSETGGVGCDNMTMIVVAFLNGRTKEEWY

EEIAKRVANGDGPCAPPEYAEFRGPGVHHNYEDSDSGFEMDGENKGKTFGVGGYRGRIIL

LGDGTEVLTDSDDTEMFDNADEDKDLESQVSKTTESPTTAPNNDDSPKTETTEASTTEAS

TTEEKGTESKSEEKKD

>SoG_01963.T1

MTGRRDFLSQPAPENYVAGLGRGATGFTTRSDLGPAKEGPSEDQMKEMLAKRAAQLGLDG

KDKDKGKEDDGADDDRYQDPDNEVGLFAGGVYDKEDEEADKIWEWVDERMDRRKRQREAR

EKAEREEYERNNPKIQQQFTDLKRALATVSDDDWANLPEVGDLTGKNRRSKQALRQRFYA

VPDSVLAAARDAGEMSTTVTDDGGASTGAGENADGTMTNFAKIGAARDKVLKSRLEAASQ

SAGGESTIGGSSTSIDPQGYITSLNKMQMTEGQAQVGDINRVRELLQSVVKTNPNNALGW

IAAARLEELAGKIVSARKTIDQGCMRCPKSEDAWLENIRLNHESPNAKVIARRAIEANGR

SVRLWVEAMRLENIPSNKKRVIRQALDHIPESEALWKEAVNLEEDPADARLMLAKATELI

PLSVDLWLALARLETPENAQKVLNKARKAVPTSHEIWIAAARLQEQLGQAQKVNVMKRAV

QVLVKESAMPKREEWIAEAEKCEEDGAVQTCENIIRETLGWGLDEDDDRKDTWQEDARGS

INRGRYETARAIYAYALRVFVNSKTLWTAAADLERNHGTRESLWQVLEKAVEACPRSEEL

WMTLAKEKWQAKDVDGARLVLKRAFNHNPNNEDIWLSAVKLESENDNEQQARQLLEVARD

KAPTDRVWMKSVVFERVLGNTETALDLCLQALQLFPSSPKLWMLKGQIYEDLGKIPQARD

AYATGVKAVPKSVPLWLLYSRLEEQAGLTVKARSVLDRARLAVPKSAEIWCESVRLERRA

GNLAQAKSLMAKAQQEVPKSGLLWSEQIWHLEPRTQRKPRSLEAIKKVDSDAELFVAVAR

IFWGDRKLDKAQSWFEKALVLDSDRGDSWAWYFKFLQQHGTEEKRGEVVSKCVASEPRHG

EMWQRVAKRPANARKSVEEVLKLVAAELE

>SoG_01974.T1

MYAGQRDSEATISINSTRQRATHSGKCLEHDHGRPHSRMTQWQLGLPDLPSLTGNNFAPP

FVPSMSRRTDSPDTPPSLLSDSHTPPRQSADDPIPSSPPLASPSSTTSSSYIGSMFTGLI

RRFSTEDSQLGSQIDTFFMGPPTEQQRAVPASTSQPQKSDGINGVFQPPVKRTISPFRPP

PLDPLVLHGHGEGSGRLLTQGIAEEIRTMIPERLRITEDWRIVYSLEQDGASLSTLYQKC

RHFQGKRVGFVLVVKDQDGGTFGAYLSEYPHPAPSYFGNGECFLWRASQLASLPPPPSSD

TTFLTRNTQLAPPPRSPKPNTLSADSAPPSRSPTPSESIRFKAFPYSGLNDFCINCETGF

LSVGSGGGHYGLWLDDSLDVGHSSRCETFGNEPLSDEGDKFGVIGVELISSGRRCVDLRL

QQRCLLRGLRGAIAQQSLPPASAILESPELAGPFEQLDEQQRIPKQGLVKGSLDQDTKNR

ICLVAAGRGQLETLRILFKSVTMNEAGGQCPSNRAEAIDARDHASVQIGNNDYSRTFYGG

IHNHASREVTRDSVLADLASHSYKERKDINRDRVPNTCEWFLSHERYKQWRRHPTSSMLR

VSAYPGCGKSVLAKYLVDEELRTTKTRMTRYFFFKEGHGDQSSATTALKCILHQILSQRA

DLFSDRILRSFQQRPYLMASVVELWDLVVELSSEPEAGEIILVLDAFDECNPEGQIALER

VICHFYGKESSHVNLKLLLTSRPHDRVVRGFRGMELSDLPVIHLAGEDEDQIKSICKEID

LVIAARVTELSLRLDLDAHEAEYLRTRLSSFNNRTYLWVHLTMDVIANASRTARADISSI

TRELPESVYDAYEKILDRSSDHLKARKLLDIMLVATRPLQLFEVAAALDLEETHRTHREL

EVFPEHKVVKYLQNLCGLFVMVINAEVFFLHQTAREFLLKEKDKASSGVSTKQKTSFLSE

KSSMDAKSWNWAHTISLEEAHHTLGKLCVAYLLLDELLDDFLDGPLKRIEQGESHHIDAP

RLCQKGPVLPFLEYASRNWHLHCRKAGFEQGLTRSMMRLCGHGESGPTSKVWIVIEGGGG

KDAGSTSSLSPLMIASRFGVTPVVQSILRINPEAAKAIDPQTRRSALSFAAEKGFVDILK

LLLKCNYSKMRSIIGPARNYVDNRALDGRTPLSFAVLNGHTEAAHLLLGFGARPRKKDYF

NGTPLSYVLCSQDEDMVALFLNRDKAMGPRDLVDSLLTILCRAITQDETRIITRIIETNC

INATTPLRNEFLSDKSIQLEGFHPGPKINSRQSRFSHAETKPETVAELAACAGSRAFFKA

FEAIGKPVNAQNGLIGELISKGAFIDAMEETKGHTPLHWALWACNTGTAELLLSLGADPH

KKTPGGYSAFKMGYWPHTAATMLRANVCIPGLERIQSADRDKLKAFAGFEYSLAEEWLDV

WQKTFHEQPRFDLSQLITAVAGSESGDIAVLEILVHAGVDLRFKDSHSRTLLHRDQELSE

ALLLLDHGADPNAQDEEGQTPLHTAARSGNSDNVDLLLRCGADPNIQDNKGETPLHMIAS

KKELMGIRFWLSYGQSNLGVLKLLLSHKADPNLQDRRGQAVLLKIIRCWPKPGTLFETLP

GQRGLHTEYFGYLHGAVSLLIDRGAEVDKQDNDGQAALHVAASHGFFSIVEELLKAHADV

GLSDGKGNTPLHYALSNGHRDIGDLLMESGAFPGPLNNAGEPPMYDGIGFGNFRLRMTVN

GPRGS

>SoG_01978.T1

MARRTYNIAMVSDFFFPQPGGVESHIYQLSSKLIDRGHKVIIITHAHDDRKGIRYLTNGL

KVYHIPFFVIYRQSTFPTVFSFFPVLRNIWIREGIEIVHGHGSLSSLCHEGILHARTMGL

RTVFTDHSLFGFADAASILTNKLLKFTLSDVDHSICVSHTCKENTVLRASLDPLMVSVIP

NAVVAENFRPRDYPASSHHPGIGSEPVRQRLGPRDPIVIVVISRLFYNKGTDLLTAAIPR

ILEDQPNTRFIIAGSGPKAIDLEQMIETNVLQDRVEMLGPIRHEDVRDVMTRGHIYLHPS

LTEAFGTVIVEAASCGLYVVCTQVGGIPEVLPSHMTTFAKPEEDDLVAATGKAIAAMRAG

KIRTEKFHDQVKKMYSWDNVAMRTERVYRGITGDIPESEFYGIDTSGYNGSRVRNFALID

RLKRYYGCGIWAGKLFCLCVVIDYIFFLCLEWWFPRENIDICPDWPRKILPERKSRLERD

GSGSRASPPEKMEPPILE

>SoG_01979.T1

MYARYIPPPAGSVPDRRGSPTSPEPVDDTTTTEQPARVGGEGFTYSRYIPGSRPAAPPTP

IAITTTDELPDRSSPLVAKKKRKAAEEIREQSPIASKKLKEDAAPIEDDSQLEEGEDSRE

GRKSRRDKGKKKSRYDNEDEKISSKKSSKKSSSKNEANRTNGEPERDDGEGNPSNTGANV

EAMEVDQPTKLEDDGAENLRREERKKRKERRALERKAAQQDDGLDEELEEKLAEAERHRS

IMERKTKSLKLAEKLSHTAEADEMEIDPADIHGLEPLPQPEQVVFDDTQPGYETLPPWLA

QPIRVSPDHKTPFGDLGVLPKAARFLEEKGFENAFAVQTAAIPLLMPTSKQQPGDVLISA

ATGSGKTLAYALPIVRDLSSVVVTRLRALVVLPTRELVGQAQDVFELCAKAYQGGGRKRV

RIGVSFGGQLLRNEQDALVERETRFDPDEYVRLQKELKSRCAAELGLEEGDEDAVDADVE

HTDPRLGTWEGDVVDFTSKVDVLICTPGRLVEHLEQTAGFSLDYIRWLVVDEADKLLSQT

FQGWLDTVLEKFKTQIYGARQFPAMEYEGVRKVVLSATLTRDLGLLNQLALRRPRLVVLE

AGEDPRVVEHSLPAQLKEYAVRVHEPNLKPLYLLDLINDGHMTGNSTPAVQGEKVDEEDD

ETSSDGSSTSSSDTESGSGSDSDTSSDSGSDSDTSSDSSSAAETKAAATPSKAPPAKSLG

SHIPTSLIFTKSNEAALRLSRLLTILSPSLIPHIATITSTTPTSQRRKLLRNFASSTSPL

RLIVASDLVARGIDIRNLEHVINYDLPASVAGYVHRVGRTARAGRSGAAWTLVADEESGW

FWGKIAKAKGIERAQQVERVRIEEMSEEMVADYEKALAKLGKEAIVRKK

>SoG_01982.T1

MSQILAFNARSVGWCILNRLSPAAPHISTSLSLSPYSPSPAFGNGKKNPSPTKSLNDFRP

QILCQLVCLSRALNMSSTIPLQSTGKPRLQVWYHDLSQSGTGPPKMEDPESAAEPSFPSD

ASSKKTQKELDKERKKAEKQAKLERKAARAQISASSSNNTEQKKAKQKKVEEALPPFIED

TPAGEKKRLKPFDDPHYSSYHPIAVESAWYSWWEKEGFFKPEFAPDGNVKVEGSFVMAVP

PPNVTGALHMGHALANSIQDTLIRYNRQKGKTTLWLPGCDHGGIATQSVVEKMLWKTKKQ

TRHDLGRSKFVELVQDWKGEYHQKINNVFRKMGCSLDWSREAFTMDENFSAAVSDVFVRF

HDEGIIYRANRLVNWDSTLMTALSNLEVDNQELTGRTLLDVPGYDRKVELGVIVHFKYPI

EGFDETIEVATTRPETMLGDSGIAVHPEDPRYAHLVGKHAIHPFIKDRRIPIVADQVADR

EYGSGAVKITPAHDPKDFNVGAAHNLEFINIFTDDGRINENGGPYEGQRRFDVRYAIQED

LKRLGLFVDKKDNPMTVPLSEKTKDIVEPIVKPQWWVKMSALADEAIKVVETGEIKIKPD

TAERSYLKWMANINDWCISRQLWWGHQCPVYYVRFPGEDGEEVTENRWFAGRSREEALSK

ARAKWPGVEFDLVCQASITAREEALCIIIFQVSVTPWLCLRADSEEIRDEDVLDTWFSSG

LWPFATLGWPDETADFSRLFPTNLLETGWDIM

>SoG_02001.T1

MPLLSTFSCYRVSELLFSHFLFLSLPLCFSLTFSSPLHSSIFRRHFQTSPFRTPWTLPLS

IPDTLIISHILELRQTQTTATMPQITTLLFDCDNTLVLSEELAFEACADLINEICVARNL

EVRFTGETLIKEFVGQNFRGMLTSLQKMHGIEISPEDMESYVTREEDAVIAKLKASLRPC

DGVDAQLEQLAASKKYLMAVVSSSALRRVQASVDKVGQDKYFKTADGKNLIYSAATSLPK

PTSKPDPAIYLHALKDLGKTAEESVAIEDSKSGTLSGTRAGIKVIGYVGPYADDKQAEME

KVLRDAGAVVIMKNWAEFPDALKKVEAGEV

>SoG_02002.T1

MASNGAGAWKTPIVTSPRNKKNDTSFPGLRPQGQDPGSKIEYEELQQNEVMALEAIYADD

FVNHTSVQQSAWKVGRHRRNGGLEIKASTNEDFAVTIGFVLTATYPRTPPVLSIKNNELP

AAITFKVQKFLETEPRLFAREPQEMVDKIVEGVRDILEDAALAKAQGALLPSLEQERERH

EAALAKQAEEQKQEDERKRLEETREEERVIAEMLQQQLDRQRQKHRESKHGKRSNGVGDH

APTMSADNPPDKVDFDQTCNAVDKAGNAMSFRSVVGKTDPRHGPVSVVYSVRPLLPSGQS

SSAMALKEAVLRPKEKDSKDFKKQLQSLESHLQDLKAGKKIQHRHIVDVFDFKVQSGLVH

DLSASNVWTISILGPLAEKGSLEELLELAGRLDIGKVRSWTRDLLDALNFLHNRNLAHQD

IHPGNILLFRETTGEIIPKISDALYQRELHTISARKQAPPGFGSAKSAYWLPPEIAATSK

PQYTHKTDIWDFGVVFVQMIFGLDILQKYSSPRNLMESLFLSHSLQELVGRFFKEDKQKR

PRAFELGASEFLATDAPVLQEEVAGVMSSSASHTSMPAMPGKFRRDSTTRGIASSRFTED

FVEEGRLGKGGFGEVVKARKKLDGQIYAIKKITQRSQASLTEILKEVRLLSQLSHPAVVR

YYNTWVEEIPNDTDTEGETSTEAATETQDTASAGVDIQFGTSTGGLDFMSSNAGVEFDYS

EDSESDADYDESTDEEDSTDVEAGDQALSPEKDRNSFLQKRARFQRQYRTVLYISMEYCE

KRTLRDLISRGLHKNATEIWRLFRQILEGLAHIHSLSIVHRDLKPENIFISISSDGVDNV

KIGDFGLATSGQFSIDKSVTNLGESDDMTRSIGTVYYSAPEIKSSANGMYSTKVDMYSLG

IIFFEMCYLPMIGMQKADVIGHLRRPKPVLPADFKPGDKQTDIVLSLVNHNPKERPTSAE

LLKSGKLPVQMESETIRRTLAGLADPSSPYYGKMLSTLFARPVEATKDYAWDMFATTISP

AELLNQGVVKQALTAIFRRHGALECTRSSIYPRSSHYGDNVFHVLDQNGTVLQLPFDLTL

GHARMLAKQSSGPVMSRTFTFGSVFRDRQDTGQPQIFGEVDFDIVTTDTLDLALKEAEVI

KVLDEVIETFPSLSSTQMCFQLGHSDLLQLIFEHCAVEPANRRAAADVLSKLNIHSHTWQ

KIRSELRSVAGVSATSLDELQRFDFRDTPNRAFSKLKTLFEGSDTYQRASPTIAHLKEVA

EYCKRLGVSTKIYVNPLNSLKENFYVGGILFSCLYDKKGRDVFAAGGRYDNLIKEQRPKM

GGQLQERHAVGFSLAWERLARVPKSGGKAFLKKAEEDTTGLFNGKRCDVLVASFDAALLR

SSGVELLQTLWAHNLSAELAKDARSPEDLMAKHRDDSYSWIIIIKQDAIIKIKSVGKKDV

ADVDIPATQLISWLRGEVRDRDSRALTKVRGGSSDNTGAGIDKEHEQSVKVLVSQTKSKK

FNRRTVVEQAQASAATLVESFLNGPILAVETTDQVMDLVQDTSLGEPESWRKVEHAVTTM

EKKYVREIHDQLEAWRFSYEQESGPRHAFVYNFRSGHTVYYDLGS

>SoG_02005.T1

MVKETKLYDALGVKSTATQDEIKKGYRKSALKYHPDKNKDDPSAAEKFKECSQAYEILSD

PEKRKIYDQYGLEFLLRGGAPPPEGGAGGNPFAGAGGMPGGFDFGGMPGGGGARSFHFST

GGAPGGGGGFSFSNPEDLFQEFFRQQGMGGMGGMGGGQEDMFSQFASAGGGGARPGRSSR

GRSSFGDSRGRDATPEVTTVERPLPLSLEELYNGVTKKMKIKRKQFDEKGNRVTTDQILD

VPIKPGLKKGSKIKFTGVGDQSMEGGRQDLHFIVEEKEHPLFKREDNDLYHTVVLDLKEA

LTGWKRTVVTIDGRQLNLDKAGPTQPGSEERYPGLGMPISKKPGTRGDFVIKYKVNFPSS

LTPAQKAQLKEIL

>SoG_02007.T1

MAMNVLMIGTGEYTTGFVDGAASISDKKVGVVGLTLFDLRRRGKVGNLSMVGVNGTKFPA

IREHLNKNITQVYNGLDTSFESFPANDKKDPDAYKTAIDQLKPGDAITVFTPDPTHYPIA

LYAIERGIHVLLTKPAVKLLEHHQDLMTKAAEKGVYVYVEHHKRFDPAYADARFRAQKLG

DFNYWYSYMSQPKYQLDTFKSWAGKESDISYYLNSHHIDICDSMVADRGYVPIKVSATAS

KGVATDRGCHEDTEDTISLLVTWERKDQPGKRGIGVYTSSWTAPTRAGVHTNQYFHYMGS

EGEVRVDQAHRGYEYAEDNAGQLQWLNPFYMRYAPDEDGNFNGQTGYGYISIEKFIDGCR

AVNDGKLKTTDLDAKPLPTLKNTIATTAILEAGRRSIDEGREIGITIEGDVWKLV

>SoG_02008.T1

MTTTNGNGANGHSHEQRPLPCGIYAPTMTFFNPETEDLDIPTIKKHAERLARAGLAGLVI

MGSNGEAVHCTREEKVAVVKASREALDDAGFQSMPIIFGATEGSVRGTIELTKLAAAAGA

DYTLLLPPSYYRPQMDEEAIFKYFTNVADQSALPIIIYNYPGAVSGIDLDSDVLIKLAQH

PKIVGTKFTCGNTGKLTRVALATDAKTPGKEGSGYMAFGGMCDFTVQTLVSGGSGIIAGG

ANVMPKLCARVWNLYAEGKREEAAELQKVLSRGDWPLTKAAIAGTKHAIQAYYGYGGYPR

QPLKRLEQARAEAIEEGTKEAMKIEMSL

>SoG_02022.T1

MDAPRDDLPSTPTQQEQPDGQTPEQQSMETPGPPTSLRIETVAQSDTPSIPSTSSQPPQR

VASPEPFSSWSASSERQLGHHSVASDTSDRVFPIRSVVSVDPANKSEDYFPPLSDSEGRG

IPVHRPGLPRGETSAELKRSGTVPPSPRSAFQSEQVQHRRRKSTLAGPMSSIQADAARHG

SQPLDLNISTSDKDTEEEAQQDEPPAVAAAVEGESLSGVSRVSMDTPHVTTRFTHVMTDE

GHAVITGRDDVLQRCEDEPIHTPGAVQGFGVLIALREENDGRFVARYVSENVEKMLGYTP

HHLFRLQNFLDILTEEQQDNLLDHIDFIRDEDADPAVNGPEVFSISVRPPKRKSTKLWCA

IHINPAHPDLVICEFELDDDQEFPLRPPDEMTPDSPADTLHANPTFEEIETSTEVLSKPL

RILRSARKRRGEQGAMQVFDIMSQVQEQLASAANLESFLKILVGIVKELTGFHRVMIYQF

DSSFNGKVVTELVDPAQTRDLYYGLNFPASDIPRQARDLYKLNKVRLLYDRDLDTSRIVC

RTKEDLETPLDLSHSYLRAMSPIHLKYLANMAVRSSMSISINAFNELWGLIACHSYGDQG

MRVSFPIRKMCRLVGDTASRNIERLSYASRLQARKLINTAPTDKNPSGYIIASSDDLLKL

FDADFGLLSIKGETKILGIIEQSQEALAMLEYLRMRKLTAVVASQDVREDFPDLRYPPGF

LVLAGLLYVPLSVGGNDFIVFFRKGQIKEVKWAGNPYEKLIRDGTAGYLEPRSSFRTWHE

TVIGKCREWNEEQVETAAVLCLVYGKFIEVWRQKEAALQNSKLTRLLLANSAHEVRTPLN

AIINYLEIALEGSLDQETRENLAKSHSASKSLIYVINDLLDLTKTEEGQNLIKDEVFDLP

TCIQEATEPFEVDARRKGIDYKVAQHEGLPRFVHGDGRRVRQALSNVTANAVAHTHSGSV

SVEVYVKEIRDHHAIIEFVVADSGSGMSSQKLDNLFRDLEQVSTEEPDDEAQDPSDTRTL

GLGLAVVARIVRNMDGQLRLKSEENQGSRFVMQLPFQLPEETPRTGEEGGGDTRQSVPET

PPIAAPNDLTASPAGEITLIQRASHLNLRQPDLTMPDDLSGGSRRSFGSQQSQESQKSDA

ERLINAIQTPLSLHDNEQEYFLSRHPSRGSSTKARSTGGFSEGSRSLSPESRKSQQQQPQ

IGSPSVRSKESGSMEVRDTKTPIRAVKIPDDYQEMPGRPQVSESSRVLFEVASDKSQKTR

SESVTSGGTGDGYSLQVLIAEDDPINLRILKKRLERAGHDVVHAVNGEDCATVYREKPQA

FDVILMDMQMPIVDGLTSTKMIRAYEKSSENKGHSKLAAGNGRIPIFAVSASLVESNRQT

YVDAGFDGWILKPVDFKRLNTLLMGIFEDDARNSCLYVSGEWERGGWLCARDQEPGSQET

TPRAEDEKKELVTEAEATAQEATRIAEESTAEQEAT

>SoG_02028.T1

MHPQLHDQIRMIPTVAETVLLAGAASSAAAAAAAAVAPPDIHRSVSLRADWMFLTRDLAD

PAQWSHAGKRLHYRKTVEGGFAFIEVFADSLGRRDPFNASKLAGALGQATGTTYEALNLP

FDTFDYEDDDNDGTIVVSVDDSSWTCDIGENGDAGAYSCISTPSQPAGRPRAFGVVRDLK

VPADNSPRISPDGAWEAFVQNDNIMLRARRSNATAYPLSSDGAADNFYDPETIVCSPDSS

KLVVYRVRPGCAREVVRVEAAPRSQKQPLVHTQLYPKPGDDIDQEQPVLVHVKERESIDV

DTELFPTPWQLSSPLWRDDGSEFVFDYQRRGHSMARVIAVSSETGEARAILTEETDSFIY

ADRRYRHDVHDGSREMIWISERDGWRHLYLVSTPDGRIQQITKGDWVVRDVLRVDDEKRL

IWFSASGVDADQGKDPYFKHAFRIGFDGADLTRLTTVDASHEVSFSPDMSLYVDTYSRVD

LPTVTELHHANGSLVQTLEKGDISRLSAAGFRPPEVFVAKGRDNLTDIWGVIVRPRDYDP

GKKYPVIENIYAGPHDSFVPKTFWPFGYHSGGDKVVGMQAQADLGFIVVQMDGMGTANRN

KAFHDVAWKNLADSGFPDRILWHRAVAARDPSYDLSGGAGIYGASAGGQSSLNALLFHGD

FYRFAVAYAGCYDNRMDKISWNEQWLGWPVDGSYGAASSVDHAGRLEGQVLLVTGEQDSN

VDPASTMQVVDALVREGRDFELLVVPGGEHTVGRSTGPIDYVQRRQFEFFVRKMLGW

>SoG_02029.T1

MEATSRQVTINLSGGPITALTDGTLIRARGIRYATATRFHKPEAFESWEAPQDCTRPAPI

CPQNPSRLNAVNGNLERGKEQSEDCLRVNVVAPEGARDAPVMVFIHGGAWVSGGGDLDAY

SPHKLVRKGGVVAVNVTYRLGVFGYLPIPDVAPANLGLMDQIEALRWVQRNISSFGGDPG

NVTVYGQSAGGDSVHCLCMAEGTDGLFHRAIAQSAPTSLREDKWLEPTWKAMSKHAATVV

TPENAASLPTERLVALTGQLLGVARTVAPGMLGYSPLYGHYPLPPADEAQQRLIARAAEK

QLVFGWTANEDSAFTTIDPRPEAKGLILDMFQAATEKLVDAIAEGTGRRPAVYVVNWHPE

TSDVLRAAHCIDLPLILGDWEAWEEAPMLQGPGVREELEAVGDSVQNLWISFARGADLSG

KRFVIDKGFRYHL

>SoG_02031.T1

MGSKVRRGFQTPWRSSRRGAAASCAYADLGDAVGEFWHGYNSLIIAGTSIGLPILISILR

FLPGHSRLSSRVQAILSQPLVGQRHRSPLPFNMGSMPTRGQSLFILYILVVNVVLMCVPV

SSFFPNARFKSKTVHRLQVMGDRAGVMAFALYVSLFLFSARNNVLLWVTDWSHATYLLLH

RWIAYACVLQTVAHSVLLLAYFVIRGDHNSEARLPYWYWGILATLATVIMWPLAVLPVRR

RMYEVFLAVHQILAAFVLVAAFLHIWYLYKYDWGYEIWIYAAGGIWFADRAVRVMRIAAV

GRKTARVTTVDEGSDLLRMEIDDVVIRGHVYLFFPSLNWRVWESHPFSVLSSFTGSTQAS

TLTVQGGEKRAEIVGKHTASSSDDERTNMAAAVAPTSIRPRAVLLIRPQSGTTRQLLERT

RATDGGSLSLPVLVEASYRSSPATRSLSNCSTLVAIAGGVGITAVVPLVRSFEGPRARVF

WNVRHEDIVRAVEPEIRQLEEAKGSGIVRTTIGSRTDVREVVREEVMRIEEEGPLGVVVC

GPPGMADDVRRAVGEVVGGGKAAREVVFVDETFSWTMKWNEVGAVMTSFPMAKISAFQDQ

PVHRGNEATQHMDGIPPQAHTNRAVQDGSKQPVSRYRQASITYIGYVSSMSMWQNGIAER

LT

>SoG_02034.T1

MESRIASVEEFCLSDFDFLVIGGGTAGLVVASRLSQSGTFSVGVLEAGILAQDDDRINIP

AFYGRSLNGERDWKFVTKAQPGLGGRRLPWPRGKVLGGTSALKFMTWNRGCREDYDAWEK

LGNPGWGWDDLLPFFKRSETFHPPSEELQATHDVSHDPDAFGTSGPIHISYSNEYSASHG

LWHSTLNKLVIPTNPAHTSGLNVGAWTNINAVDPRTSRRSYSVDYVRMLPSDARLSVLCG

ATVAKILLEKEEDRCIARGVRFQVGGKEYEVLARKEVVLSGGSVASPKILELSGVGNPKV

LREAGIDAVVDSPMVGERLQDHIMLASIVEVDPTLANPDDLVNDEAVAKEAMERYRADHS

GRLTILPCSICYVPFSRILPESRLEELYHAAERLQTYDRESRSILRERLNGEATLGQMEY

IFDLGNWSPRFKGEPGKKYGTLLQILQYPFSVGSIHVTSSSPHESPGIHPAYYEGPHGEL

DLRVMQEAMSFGRRITETETLSRIIREPAWPPAETLNDRDRAKQWIVDSTITDWHPVGTC

SMGGRGGIKAGVVDQRLRVYGVERLRVIDASVMPLQISGHLQATVYAIAEKGASMMLDDH

GCPS

>SoG_02040.T1

MPFSKVTKFALAALSILAGHVRADACDDVESLTTVSVSRPLTLSYASEQNEYWSKACSLL

KPVCIVFPTTAAEVAEVVKILGRHDHDFAVKSGGHSPNKYFASVDQGLLISMSKIEHAHL

DQQTGVLKAGPGNRLDGIAKKLDGTGWTFVGGRIGNTGIELPGGLILGGGLSYMSAQYGW

SASSVYEFEVVLADGSIVTASEDQHTALFHALKGGGNNFAIVTSYTLQTYRQGDVWGGNL

AFLRTSKTDSKLLQAVRDFTEYNDDDKAAVIVTAERGNIDLVDSWILFLFYDGPSPPAHI

FKNFTDVGPVLNTCKTQSYSKLIGSSNWVIVPASHVLMGTETMPIPSAHDSARVFEELHS

FWRSVSTTTLLVPGIIASIAYQPFPRRIAQAARARGYDLIDAEPDADKLIIEINYSFLPL

TDYDKMSDVLARTYTGYRERIQSWQQDGTLPDNVYLPLFMNYANQRQDYWGRLKPASRSR

AASAAAQYDPSGLFRGRTGGWKP

>SoG_02048.T1

MASTDRFSELRNALPKGKVLLPGDEGYDERLQRWSATCVKPAAAVVMPATVEEVSATVKF

AVANKVAFNVRGGGHSTSQVSSAPSPEGMVLDLGLFRNVSVNVENQTVSFGGGCLWGDVD

DALLPHGLATPGGTVSHTGVGGLILHGGFGVLTGLHGLTIDCLVSCEVVLADGSIVTASE

KENADLFWALRGAGSSFGVVTGFTSKVFPQGDIYLGVMAFAPDKLPEIIAFVNYWDATTD

GSTAIILFITHAPVPPGTEPPPGGPPRVVSVMFGHFGPDAATAGPAYFENLTKIDAMFQD

VGMKPYPNANRLNDQSHFRPEERYQFGGSNFTLPTTAEKFQAIAEKWWTTTAPEKGLNGS

VMVLEGIPGNFTRKVPVEAMAFNSRGAYYNIGMVWKWEDPKMDADIRVLNRQLQDETRQM

GYNDAENKDGVGRYLNYVADGLAAEDAFGGHAKKLRDLKEKYDPTNVFDKLWKLLKKGEE

QHPV

>SoG_02052.T1

MSTSIRNGLVTALALGGVNALQIPLQLPVQLPDLPSWLSPSGQQSSSLPLIDTEKLQASI

SRKSLKKRAEDFYELAKKGEDEYGHPTRVIGGKGHLATLQYIRNALAEAGSYYQLSSQSF

PAVTGEVFESRLVIGNEVPSSATPMSLTPPTKNKQPEYGNLVLVDNNGCDASDYPAAVKG

NIALVKRGACAFGAKSEGAGKAGAIAAIIYNNEKGDVHGTLGTPSPDHVATFGISQSFGE

KTVEKLKAGEKVDAIVYIDGEVRTIMTKNIIAQTSAGDLNNCVMLGGHSDSVAEGPGIND

DGSGSISVLEVALRLTNFRVNNCVRFAWWAAEEEGLLGSDFYVASLSEEENKKIRLFMDY

DMMASPNYAYQIYDANNEENPAGSGDLKQLYIDWYEKQGLNYTLIPFDGRSDYDGFIRGG

IPAGGIATGAEGVKTEEEQAMFGGKAGDWYDPCYHQLCDDTGNVDLDAWEVNTKLIAHSV

ATYALSFDGFPKRTDEKRVESYAQKSKYRGSKMVI

>SoG_02057.T1

MGRRKIEIKAIKDDRNRSVTFLKRKGGLFKKAHELSVLCSVDVAVFIFGNNKKLYEYSSS

DMGDLITRYQYHGGVNEHKGPSDFNGGNDEDEDEDNDGSPPHGHDGMDHSQMPPPQFHGQ

QPPFPQIRHHTASASPPIGNGVAFGHPGQPQRAHTPNPPNMGSRPSSRNDVRRMQAPVMV

PQQPHHAMAYMPAPAIYAAPPPSVNQPGVMPQQGPQYPYQPPQPQHQHQHQQPPPQQHSY

VDERRAPAPPTYSQAPVQSLKPEPSPPLPLHHQNIPRISVSPQPEKRFSSPQPPPPKIES

KSEFHERPQVPLLKTDAAIKKLPQRKGHSIFTPVEENRSILSQHLASFQSADLKSESGAS

IAAAAAANRASEAAKARNGSSSSPPQPQRSSSQSSLDKGRSAGNTDFPPPGRSNSLRVGG

PGGGGRPRGPPLTLQIPADEASEAGTSAAGDSSPHPPNSATHVPQRHNSHSSVVLPPPSP

SGASAILSAGATGPPNPFARPPPQQGVNGETPVSALPSRFLNHEFLPSPSSFYPEWNFRG

SDSNTLPSPLNFATPIGGSGPSFLRDDSSASNTATTSATAGSSSGTTATASTTTTLGPVK

RKSPDYEGAEHEASHDASEAKRVKV

>SoG_02065.T1

MASLPKKMRAVVFSSVSGPLQLTEKQLPTPEPGELLVKIKAVSICASDFVPWKGYLGDVE

GLVPGHEGVGVVVGTGQGQFCSTGGKNQGFDIDGYFAEYAVVSAKFSVRIPDGLPLERLA

PLFCAGVTAFRAVKLVDAPARGKLGVFGLGGVGQFVVRFAMSMGFDVIGFDVSAESREQA

MRAGVSRALDSSNMTAVTEEVHKLTGGRLLDGAIVAAGVAAAYDAAVRLTGVFKTIVAVG

VPRDPILVDPLSLIKKGLVLRGTVTGAATDLNEMMRAVEQHQIIPDIELGALEDIPELFA

RSTSGKSTGKLGAVIS

>SoG_02066.T1

MPPSIRRDFAPILQAMASHPVPIGDVASRRNMMDPVMATMSAAAKKAQTDLEAKIDLRTH

LVLRTEEGYEVPVLHYALKTRQTTGITAAVVNMHPGGLITGSAKFMDASIRQHVLETGIQ

FLDIEYRLAPEYPYPTPQEDCWAALVWIRDHASELGIDPKRIALMGESAGGCLATSLAAH

AASTNSMSIAKLILTCPMLDDRTGKETPEEGVLYSWTPGDNITAWDAVLGADRSLVKGEI

PPVPASITDVSGFPPTLLQVASGDLFLDEDLALGQLMARQGVDVEMIAYAGVPHGFEAIL

PDSEPSKRARRDRNRFLEDLYFAAPSARLGLALSPAPWAIASNCVTLEVIIPVTGCLLDA

HMRLSGENAEASPGTNDDEDQNPNPSLESLLKLENSDMTTFILTRRHTEMPEIADHMASL

ILMYRLLRLTLPHPVTIDFLAFPALRDAMIKGDPAYPDDSSEAQMVFERDFTLNLTVQWP

PLEPIVVRTSADRYSSGGPRFQLNPVFEAHCLVFENWAMRDEWIDKYPQFAGYVHKCRPS

FASRPKQWAQPP

>SoG_02067.T1

MPSAPVDSKPASAASSKPSSLKSQDAPARSPHSTPQNGTSSPVNGATSPPLTPDGSLKPL

EPKASVKERFTRMFSNKGDIPKVTPPASHNAAPSSQQGESSGRSRGSSLNSASGPVPAPA

TPATATAAAHAPAAAPAAVAASQRDKTSQRKESYSDQGSERSAVPAKPQRFVLLPESQGS

GHEHHLKSSRRQEKLSDMWRSLIGKKHEPPPENDLSLVSSWVDTLRQEKEAASEEKKTGV

AAPTTLVEKYGKCQEVVGKGAFGIVRISHKKLTTGNGEKLFAVKEFRRRPEETEKKYSKR

LTAEFCISSSLRHPNVIHTLDLLKDAKGDYCEVMEFCAGGDLYTLVLSSGKLEVQEADCF

FKQIMRGVEYLHEMGVAHRDLKPENLLLTTRGAIKITDFGNGECFRMAWETDAHMVSGLC

GSAPYISPEEYTDKEFDARAVDVWACGVIYMAMRTGRHLWRVAKKDDDEFYARYLAGRRD

EEGYGPIESLHRARCRNVIYSVLDPHPSRRLTAAQVLRSEWVREIKLCKAGEEGL

>SoG_02083.T1

MWRRTYLLLVLIRLWFALSPSYLHPDENFQGPEVIAGEIFSYPVRRTWEFTSEHPIRSVF

PLWPIYGLPMLLLRWLWIGNGQDGEIPPIAVFWTLRVLMFLMSFVLEDWALHELIQSPRH

RRIAVLLVASSYVTWTYQTHTFSNSVETLVVAWSLVLIQRIVDSQQRSSILSSTVLGVFA

VFGIFNRITFPAFLLIPGIRLIPHFMKQPMSFVVMLTAALATIFVAITLDTAFYIPETIT

WADLIRRPVITPLNNFMYNSSVENLAQHGLHPWYQHLLANLPQLLGPGVILCFRPHLSLR

LYSAISGIFVLSLFQHQEARFLMPTIPLILSSVRLPNNPTSLRIWAAAWIIFNLFFGILM

GTYHQGGIVPGQVFISKQPDATQAIWWKTYTPPIWLLNGKNEVLRTHDVMGLKGDELLEM

LNGLATCDTPADRRNQEYLKESNGTYLIAPASATWLDPYLGNKGLDGLRFREVWRYRQHL

NLDDLDFGEDGVWNTLSRVIGRRAFILGQAKRSDQISTLKSKKVRRDLGVSI

>SoG_02087.T1

MLGLKALMTAIAAFCATGLALTPPQYNGYAGKWSDHFDAPADAPANPAKWNTIVTSAVWN

NEWETYTANVANVRHNGAGRFQLIPRKDSRAVHGWTSGRIESKYTFTPVPGKITRLESQL

RIGGVSPSQKQGIWPAFWLLGQSYRQGTIWPATGEVDIFENVNGNNIASGAIHCDKTPGG

ICNEPNGLTGSTTIDTFSWVLWRVEFDLRNSDWRQQYIIWSKNGQEFFRIAGSRVNNQGV

WNTLVAQPLYIIFNVAVGGNWPGPPNSQTADGNGNILELGYVAHYISR

>SoG_02089.T1

MSSDAACRPLQDPGISSPSSASTTPQLSAREAESPEPEDDFEATRRPSDFELDELLDNGG

TKPLLPQDNSDVPEQQSAAGPSLKSSSSAFKSVFWTLVNVVATVLIVFTNKSILSGKSFK

HAQLSFAAFHFTITGLTLFILSRKRFNFFAPKHVPVQQMIPLATVMALNVIFPNLSLAHS

SVPFYQISRVLITPCVAAMNFVLYKATLPFHAFLALIPACIGVGMVSYFDTKPTSKTANA

TSGLGVIFAFLGIFFSSLYTVWLESYRRRLSMTSMQLLLNQAPLSAFLLLYFIPFVDSPP

AWEDTSLSLWFLILMSGFFAALVNISQFFIIAEMGPVTSTVVAHGKTCIIVAIGWYMSGR

PVADQCVIGLVMALLGIILYSAAILKQSAKGTGQRRY

>SoG_02092.T1

MPGVHTNGCANGSTNRYLIWGGNGWIAGLLKALLKHQGKAVFSTTVRMEDRVNVVRELET

IQPTHVLNAAGCTGRPNVDWCEDNRPQTVRSNVIGTLTLADECSRRGIHCTIFATGCIYQ

YDDEHPFGGPGFKETDAPNFDGSFYSMTKAHVEPILASFDKVLILRLRMPVSDDLNPRNF

VTKISSYEHVVDIPNSNTILHDLLPASIILAEKGDTGVYNFTNPGAISHNQVLSLFKEIV

RPNFTWKNFTLEQQSHVIKAGRSNCELDTTKLVEKLRGYGYEIPEVHEAYRQCFERMKAA

GIQ

>SoG_02093.T1

MAYSFLPERDIWQHAPILTGSTKFEPLQDVKNIMITGGEGFIASWLVRHLVLTYPGVYNI

ISFDKLDYCSSLNNTRALNDRSNFTFYQGDITSPSEVVDCLERYEIDTIFHFAAQSHVDL

SFGNSYTFTETNVYGTHVLLESAKKVGIKRFIHVSTDEVYGEIAHGSGDVPEASILSPTN

PYAASKAAAEMLVNSYQKSFKLPIIIVRSNNVYGPHQYPESKRSLLGCWHVVSLLTLWKE

IIPKFISLLHRKQPLILHGDGSPTRRYLFAGDAADAFDTILHKGAVGQIYNIGSSDEISN

LELCDILLAEMGIDVQDRLKYERWVKHTHNRPFNDQRYAVDATKLKSLGWTQRTTFAEGL

RTTIDWYQRYGSKWWGDISSVLTPFPQGLIKKPQAEEVVTPESL

>SoG_02096.T1

MLSNAILALGALAATVNAAPASHSSHGSKSRATNCEFTTLSALENGKSNCDTIILNGISV

PAGQTLDLTGLKTGAKVIFEGNTSFGYSEWVGPLIAASGNQINISGASGHVIDCQGQRWW

DGKGGNGGKTKPKLFSTKGLNFSTIQGLNIKNQPVQGFSINSVNDLNIIDVTLDSSLGDT

LGGHNTDGFDVGNAQNIYISGATVYNQDDCLAINSGNNITFTGGTCSGGHGLSIGSVGGR

SNNNVVGVRILKSSVSNSENGVRIKTISGATGKVNNILYQDITLKNIANYGVVIEQDYLN

GGPTGTPTGGVPITQVDLEGVTGSVKSSGTNVYILCAKGACSNWQWNNVKVTGGQQHKTC

ENVPSPAQCS

>SoG_02102.T1

MPIIVKEKSEYTPLDPNYTPGKNAHYAELDPIFAPLKPVIDAQLDQIWNPESTIDEFRKG

WSANSPSPKGCPVEGEDVVTEKRTVKMRDGAEREICIYRSKEDDGKGGKALVLRCHGGGA

PEYKFPYAVNDCFDALKWCHENAESLGCDPNKIIVNGGSAGANLATVSALAARDASIPVA

GQVLTWPSVCHPKFQPKDKYELKSYQQNHFATILTAPRMEWFLDQYMPEPTDDWRMSPLL

AESLKGLPKTRKANQTLSSPRSLPPALLNEHLPFMALLCEGVVTIAGYDILRDEGIAYVE

RMKSEGVDVDYKIFKGLPHCPYGMADHPEVIDYYDRIVEFVKGISG

>SoG_02104.T1

MKAGQWDGKLKKVVINDIPIPEPVENQFLVKIHSASLCHSDLMNELRPEGSVTLGHEGVG

FIEKIHPSAEGKGFKIGDAIGFNYFLGVCFECDGCMVHNLRCERSPAQLQGFVADGYFQE

YCVVDYHNAVILPKELDMKRAAPLFCAGITAFHAVDSCELKPGEWLAVVGCGGLGQYATQ

YAKAMGYKVVGVDVNDDVLESVTRLGADATVNSRTNPNAVEEVRKITGKGAHAAAVFAAA

PRAYSSAIEYLRINGLLMVIGISTEDLKVSTFDLTTGRYRIKADSTSIPQRMAKAVEFTA

KHKIQPDVEFRKLEDLPQMVADMHAGKAKCRQVVVF

>SoG_02119.T1

MADDDAPCVSGDTTRSQPPEPRTSPGPEKHHPAHDSLPNPDSIAQPGQALPQNTTTAAAT

TTTKPSAAPDSGMPLIHEDATIAQDQAITVVGSKAEHVDERHLKTAHDTDAAQGATSTRK

ETPQELAKEISIEPSNPIVTPATPAPPGVSGLTAKQSVGGKTKTQAETRPMPDPAVAIHN

RPPEPATDAALPPASNHPTSDPPVPHFVAPSSYLRPKNSQRSGRPPTAAMASQEQRPPPG

PYDKEQLQGLVSSWCPSVFWQRSAFAHPTPRNDAACCWFLSATCDKKAIRDFLKVRTSYD

VLPLSFRLIVLDTNLLIKKSLNILIQNSIVSAPLWDSQASKFAGILTSTDYLNVIRYYCQ

FPDEMSELEQFRLSSLRAIEKAIGAIPIETVSVHPSQPLYEACRRMLKTRARRIPLVDVD

DETGQEMVVSVITQYRILKFIAVNNEHNTVLLKKSVRDIGLGTYEGLMTANMMTPVLEAV

GLMVDHNISCIPIVDKSNKVLNVFEAVDIIPCIKGGVYEELDGSVGDALCKRSDDSPGIY

TCSQDDRLESIFDTIRKSRVHRLIVVDDDNRLKDCGVERPLSRPCLEHGWMGHLVIRADE

ERPVKSPFRHLLHSLRTLVELSLLPVCQLLHMPSPLGSARMAARIHQLSAPAQRRSPSQR

ENGSHRTARRAIIPSQSSASLGFPHSVTVQNLGAAHGPQGRSSRTWTASSGDFGLLSDTD

ELEDRAVFVMEYNRLAKKHGVRLLILEDFHRRKEPAVTSLGRRGWLQKLLRSSSNSTPSS

TKGHTAANPRHKRSVSDVAHHLALSRRNAATTVDLQAMVRISGKSVLYLPADHAPGAIVL

PTCIRAIAQHLAHNAMTRGIFRIPGSVKIVNALFDYYCYIEGGAAGLSGTVRCVNLPMHI

QSSVHDVASTFKKMLSVLPGGILGSLAVFDAMIAIHSQLHGSPEFPRTKQTRVRARLIAL

AIGTVTSKFRRELICAVFGLLSLIGRIAEVTPREDEEGRALPTADLMGYNALGIVFGPLL

VGELLDQYTMRIANPGAGLMLFPVTPQKIRRDRRKSKQVEEGVPPAHTEVDKVLVANSIA

EMLITNWRDVVRQMKALGTNSTKDLSMASIRSESLRPSASETFIIKKPQDWDNKGERATA

AREGNESPQPSTPTMGAKPQRPKRQKSATSGHLTARPVTALSPTFEENTPVESLKSREAS

EPTMIQGPETPSFAQQRYRQELQRLEAEAMATTLEGSVELQPVTGEAPTSSGRHDMRMRR

GNSTYSSPHVSLESIPPRTSSRPRHLAGTIRRSEDVQQSSLEGNSECGYRESIDGIRGRQ

RRKGNNRGNGDRSTDDFPSSISRLTRPTPEPTPHSSRSSMAMKQFGTEIRGKESSPRLTN

QHSEIIRGKQGSQCQSAKEETDHRLHTSDIDGIQDLSQRKDDENIDNGSKPHGAYGYFYL

SRNDESGNHRRPHSSEGDQEGETPQQFYAPMQTPSRLMRQAKSGRNLPSTRTEA

>SoG_02128.T1

MHSVKSQSSLLLAALAAAPALVAGHGHVTQITVNGKVIPGFQPGNPSTDSVGWATTVQDN

GFVLADSLQSPDIVCHRGAASAAKSATVAAGDSITITWDTWPKSHVGPIIDYLANCNGDC

SSVQKTSLEFFKINEGGLIDGSQAPGRWVTDELIEDGLKWTTTIPPNIAPGNYVLRHEII

ALHEGNRQNAAQMYPQCINLEITGSGTAKPAGVVATSLYKSSDAGIFFNPYTNNLKYTIP

GPANSFGSGNGGGNGGGNGGSAPAPSPSAPANGGGGNGGSSAAPVPSASSPPANGGGNGG

GNGGSAPPPARTTLATSTRAGSAPTQAPSDSCGGTVRGQALYQQCGGQGFNGATTCAQGV

CTAMNPYYSQCVPKN

>SoG_02141.T1

MAPTPMTAQYWRTVPEAAATEVQGTVTQASRRSIFLREACAPRLATARVTFTLSPPTLLP

LFASPLRAASSAAFYRLSSPFRTSTFLSITTIRCYAKSRSKMAPKKQVEEKKIPLGRPGN

NLKSGIVGLANVGKSTLFQAITKCNLGNPANFPYATIDPEESRVIVPDDRFDWLVDKYKP

KSVVPAHLTIYDIAGLTRGSSTGAGLGNAFLSHIRAVDAIFQVVRCFDDAEIIHIEGDVN

PTRDLDIIQEELRLKDIEFVEKALENQKKKTRMGGQSLELKKARLEQEMIEKILAWLQDG

KDIRKGNWTPKEVEVINPLFLLTAKPVVYLVNLSEKDYVRKKNKHLPKIAEWVKEHAEGD

PIIPLSISYEERLTRFETEEEAKEEQKNVGAESALPKIIAQMRKALQLGSFFTTGPDEVR

QWTIRNGTKAPQAAGVIHGDFEKTFIQAIVYNFSVLKELGDEAEVKSKGKVMTKGKDYVV

EDGDIMLIKAGAAKG

>SoG_02144.T1

MALSPVVTQTLDRSKLAGSPLKIVGTSAQTSANGTPLPAPTGETISSLPPTLDLAEQMND

EEKRKYVKGKKLGEGTYALVFLGHVRRDPSQLVAIKKIKVQKEYTEGMPPDAVRELKFLR

ELQHPNIISLLSVFSSRDQNLNLVLEYLPLGDLEELIRDVDSVRYGAPDIKAWMGMLTRA

IWFCHENFVLHRDIKPNNLLIAADGEIKLADFGLARSFADPHINMTPNVITRWYRPPELF

FGCKHYSGAVDIWSVGTVFAELVLRRPYLPGNTEIDQVRLICENIGTPTESNWPGVSKLS

EYTVVEGHALKTRQEFEMRFGIVGRDGVDLLMKTLSLDPKKRITARGMLDHPWWHAEPQP

TKKRDLPRKGGGEEKMGADLKRRPGKVEDDRGSKVARKLDFGAMK

>SoG_02158.T1

MSEQLSFDVIIIGAGISGINAAYRIQTDGPSDTTYAILEGRDSIGGTWDLFRYPGIRSDS

DIFTFGFPWAPWKHKESLAAGDKIKNYMIESAQSVGIDHHICYQHKVSQANWDGRKNRWE

LQVTRPGDEKPVTFHARFLLLGTGYYNYETPLETVIPGLENFKGKVIHPQFWPEDYDYTN

KDVVVIGSGATAVTIVPSMSDKAKRVTMLQRSPGYIFPLPSHSLFTTILFTLFPSRIAYF

FNRLLWLFKSYMTTLFCKSYPGLAKSVIKRVTIKQLPPDVKWDPHFKPRYNPWEQRFCAS

LNGDFFAAIRSGKADVVTDRIKTVTENSIELESGNSLHPDTIVTATGLKLRFGGGIKFLV

DGKEVNVSDRYAWRTTMLQDVPNLFFIFGYENASWTLGADVGAQLFVRIYNRMRNRSQTV

VVPRLTPPANPVVKPMMSLSSTYLKNAGSILPKGGEGQWSPKTNYFADMVGAKWGDITSE

LRYT

>SoG_02159.T1

MSAFGPVKKLFLWLYVILYTPFLILHVTFDITRYILPWARPSRQWSLRQAIRMRVVRFLL

YHWSLTKAGDRLHLGQGREKNRFEVVAPRSPKLYRGVLLDNSVQPAQVGMTWTPARPPPP

YLISADMVVALHFHGGAYVIGNGRDEDTGLLAKSLVRHAGCTHVCTPQYRLSSSKGGHFP

APLQDALTVYLTLIKTKGIPADQIVLSGDSAGANLALALLRYIKEHGKQDEIPLPKALML

WSPWVDVSAALMQDMTQSPNYGTDYLNKEFGRWGAQTISNNLALDPAGPYLSPLHHPFEL

DTNMATFVHAGSSEVLYDDIKEFCKRYEKVGWRVHLTVSDNAPHDIILLGERVGFGAEAD

RAASQARMFLSGATGLKLRGCPS

>SoG_02163.T1

MVFAGYLPPQAEQGGKLPVLYWLSGLTCTDENFMQKAGAQRMAAELGLFIVAPDTSPRGA

DVPGAPDTAWDFGLGAGIYLNATQEPWARHYRMYDYVVQE

>SoG_02167.T1

MASFKLSPLTMLLGAVFFFSAQVFAASAVLGVDLGTEYIKAALVKPGIPLEIVLTKDTRR

KETSAVAFKPTKGTPQDARFPERLYGADAMAIAPRFPADVYPNLKAVLGLSVDDSVVQEY

AARHPAMQIFPHPSRNTVTFKSKTFNADEEAWMVEELLAMELQSIQKNAEVTAGDGSIVR

SVVLTVPPFYTTGEKRAIQMAADLAGLKVLSLVSDGLAVGLNYATSRQFPNFSEGEKPEY

HMVFDMGAGSTKATVMKFQSRNVKDIGKFNKTVQEVQVLGSGWDRTLGGDALSNLIVDDM

VAQFIESKGAQKISATSEGVKAHGRALAKLSKEAARVRHVLSANQNTGASFEGLYEDVDF

KYKITRAEFEAMAEAHAERVGATVNDALKMANLDIGDLTSVILHGGASRTPFVQKALEKI

VGAGDKIRSNVNSDEAAVFGAGFRAAELSPSFRVKEIRISEGAMYASGMEWKGNKDKMQK

QRLWSPISPLGGAPKEVTFSEHKDFAIAFYQQVAAEDKIVKTLTTKNLTATVTAFKEKYP

SCDDTEIFFRVGLKLNGDNGEVEVSKAAVECEAEVKEGFVDGMKNLFGFGKKDQEPLTGD

DAEADSVKAEGSEEEVKDGAAKSSATDATGSSSSATDASASGSTEEAKTEIKKKLVGIPV

EVELLQAGAAGLTKEEVAKSKDRLKAFTIADKARVAREEALNQLEAFTYKIRDLLENEDF

IAHSSEAERSKLSEQGSTISDWLYDEGSDASKDDFKAKLKQLQAIASPIQKRMEEAEQRP

GLISSLKEALDQTKDFTGKMLKQMEEYEAWHASSSASKSASSTIASSESTEAPAETPTGE

FDGLGDEASATPKSRTMEDVTEEIGPIPPLYKREDLEEVVAVETNIRQWLAEQEPKQEAL

PATADPVLLIKDLKSKREKLDKAGVDLAMKGVRNFDKKSKKANKSASERKKKTKTASADN

AEKTLKFEDFADSEDGPKVYSGDDIEELMKKVQREKEAWIKAEEAAAKAKGESSGKEHDE

L

>SoG_02192.T1

MLNANAFSGRIIALTFFLIFVLICIFYVQSTEITSISSISSITAAPSALWHSATSFGKAE

LPAFHTSKGGEGISYQLDIPPSIGCEDKVNELQQRLIQDYTRLFKGIRYANIWGYLETEN

KGDAAIWSAQQILLSMLGIDTMEACRFMDRDCDIEKFRDKLKEHEPHSGIIMAGGGNFND

YYWEDQPSRMKMISNFTDFPVRAFPQSVYMTKPDRIEKTKEDFTKHKNLQLAARDQPSYD

WLESTFKNEEGIESVLVPDIAFMWGNRSDFRVNTKKTHDILILARKDMEISAGDSSEIEF

GEGTLDIGSAVNVTYQKVDWKFTDTPGINPEKESAEGYKENGKNQRAWAKAIAGFELLGS

ARFVITDRLHGHILSTVIGVPHVLMDSKLGKNLNFHNTWTRDCDCTRITTNISQALDVAK

LYFESVDSDDVMQNRTD

>SoG_02194.T1

MRSSVVLFLAALWDTGACIDLRKIELDVLKSSKQALILFTADGCKECERAEEVLSKASSV

LPTIPFASVNCNKEPVACDESSVFSVPTLKFTPGNGDLITYRETVDVASVVRYLERQSGS

PVKSLSRDKVLDFAATSRVAVVLRFGPSTSEEHRKVFDAVAEKWRAHYNFASIDGTDDET

EESSITVYSQDRDDVVNYHGMFNVEKIEAFLQDAIKPLIHEFDPLVREELAKSGKPQAQI

FFDRQDERRELVKSLFPLATKYGDHMTFVTVHSWDYNQQCDRMHIEKTVRRGFAIADPHG

RAYPMSNTTAFTANRITKHIEAYLAGILKPTIKSEPVPEPSTEQPYLTTLVGSNFDEFVL

DKSRDVLVEFYSAWCQYCTELHEVINNLGSKHVDARLSEKVALAKINVEENDVPIGIDGY

PTIVLYRAGTNEVVSFEGDFTKMLTLEQLDKFISSRGGHGVSALGQSQLDSRDEL

>SoG_02202.T1

MHVHSQYLLLGLTWAGQALARPSCVEPKVTVANGTYEGIHNREYKQDLFLGMPYAKPPKR

FTVAESLDEKWTGAHDAKNYSPHCIGYGEDMIGYDMSEDCLYLNVVRPAGIDRTADLPVA

VWIHGGGLIQGGAADKRYNLSFIVERSVEQGTPMIGVSMNYRLSAFGFLNSKEVVDAGIA

NLGFRDQRLALQWVRENIGAFGGSPDKVTIWGESAGAESVTAQVLAYNGRDDGLFRAAIG

QSGFGGLLPRLPGGFNATYVVQAIYDGFVSSTSCAHLVGSPESIDCLRQAPLDEINGVLA

AGFAPAWSPALDGDFFQDFPSNQLEAGRFVQVPLLTGANTDEGVSFRQSGSGGINTDDEL

ANLLSIYLVSDNVEETQQELVAEVMELYPNDQSKGIPSLETWPHIIEPGDSFAKQLGAQY

RRESAIMGDVVIHYPRRRANQAWTAEGVPNYAYRFNIMPHGTTGPQFGSNHFKEASDVAF

VFLNTEGHGYAINPFGGDDEEYRAHAKEVSKTMGTAWINFITNLDPNGPAELPGSTVWPE

YTSSVGKAGRSLVWDLGATRVEDDDWREEGIAWLIEHALLVFGN

>SoG_02203.T1

MTSLLPSQEPSSLPGKDAGSQKHEALQPIHPSMKGKLDPVFEKLYNDNVANTPLKPIDLS

VLRAKYSVLYSYGTGPAPDVAKEYDTRITTHEGIDLDVRVYEPETPGPWPVHVDYHGGGW

GLGDLDTESHICKHICKEAGVAVIDVAYRLVPENAYPCGVTDSFAALKYIYENGATRFNI

DPKRISIGGVSAGGFISLALGHMARDAGIHLSLIAVGTPVVDDISQYSSAAESPFPSMQE

NEHAPTLNWSRLAWFDKLKWQSLGSSSSEEEAARKKEAVPAIYRNLFKADRYDGLPRTVI

YTAGADPLRDEGEAYGRLLVENGNEVTMKRFPGVPHPFMHMDKDLWQASAFIKETAAAIR

VALHGAAKDL

>SoG_02207.T1

MDTRKLQPNDSRVSYKTATVNGKTYSYILGVPPNGQEPIETVFLCHGFPDMAFGWRYQIP

VLMSLGFRVICPNMVGYAGTDAPKELAAYSGKSVADDVKELSRQIIGEGKQIILGGHDWG

GQVVFRVCLWHPELVKCVFSVCTPYGPPMANWISLEDIVRTRLPNFAYQIQFAGPDVEQN

LQGPEKIRQFLVAITGGKNGNGERMSEMTTDGVNFKKLEGMDKSPVLTDEELDHYVSEYM

KQEAPQLRGPLNWYRVRKINFDDEKRFLEERGPAAVKLEMPVLYIGATNDIALPPIMSKG

MDAVVADGKLTRNEVKASHWALWQTANEVNEIVGDWLAKVLQKKLKPSL

>SoG_02210.T1

MAEPWASKQAARRRGGLLSTAAESRMLVLERPFHFAFVTFLVHAGLLRALRSWIAREARQ

AVTRDPEFGLPLTEKDGDRKMVVQPSPTLVDSVRDPRRAPALWLFSIGVALRCVAAWWKS

ATYLKFALSWSLASMHSLSSARISTSAVCPNAWWYDPFVPVLQVIICAIDAHLLVGFSNM

RHKYLQQGKSHAQILTQVSLLGAALIVVFSVTSWVEDRNFFWAMTFHYADIRDLLIDGFL

AASSMICGFHLLAVLSPSTVAFAFTSMAMYGVHIPYYGLSDMIAPLDMEAINLKMAFAAF

SAAPMWLLLRPPLVNTRPAVTAVVHRWLVLCYVAIVALCLSFWMLARPVPIARWSLAEAV

DELSRMGRSYSTAWQADASASTSLERAVEKYRERYGIPPPPNFDKWYNFAIGHGSPIIDN

FDQIHSDLLPFWGMQPADIRERTYEVQRYSTVEMVGLRIRNGAVEHSPYIHTSHEWMMSS

IERMVKPFAQWLPDMDIAINVADEPRVALSYQEKQRLESEAAKARTRALGHAEDGHSDKA

PRTQWPSEFPKPVDKEGQMILPRGFTNYIRKPIMNDLVSDSCPPGSLLPSSRWWDPSAAC

TECQLPHSLITTSGPLVLNDTRSSDLCHQPDMAYLAGSVLSPSAMFATRQLSPVFSQGRI

TGFSDILMPSPWNFDERSSYDESQDMDWSAKTNSIFWRGGSTDGYAAEGSWTGFTRARFG

REAYEKAKRLNQYGPAPGNLGINVSFSGEMTRCHRSDCKAELRTFNLWAAEASGSAPSTD

SPRSADQLPPVTPFSEHWRFRHLIDMDGAGFSGRFLPFLRSQSLVYRAALYRTWMDERVH

AWRHYVPVDVRLGQAFWDLVEYLAGGGEKASGDAVARTIAEQGRQWASQALRKEDMQIYM

FRLLLEWGRIVDDDREYLFYKV

>SoG_02215.T1

MPTFTVFKGSKEGAAKEATTTRPDELQGDDVLVKITVSGLCGTDLHYKDQDMVLGHEGVG

VVERIGPDVKTLKQGDRVGWGYNTNSCGLCSRCLEGEDVFCPKRGLYGSVNRDQGSLGAQ

AIWREAFLHPIPDSLSDEDAAPLQCAGATVFTTLTDVTPNETVAIMGVGGLGHLAIQFAA

KLGCRVVVLSGSDSKKAEAMRLGANEFISMKNLSKDTKDVPLKGAIDRLLVTTSAQPDWS

VVLPMMAAKSSIYPLSVSFDNFEIPYMPVLLSGISIKGSLVATRARHRQMLDFAAQHNIK

PVTETFPMTEKGIAEAIDKLESGNLHFRAVLKSQL

>SoG_02229.T1

MAPKRYVIVGMGVRSAFYYQSIIQDHNDVAKVVAICDTNQTRMNAANDRMVELGGEKVPT

YKAEDFDKMVTENKADVVVVTTIDLYHHVYCIRAMELGCDAITEKPMTIDEDKLQAMIDA

EKRTGKQIRVLFNYRYAPHHTKVRELIDSGVIGEISTVHMDWILDCAHGSDFMRRWHRDK

ATSGGIQMHKSIHHYDLVHYWMNTEPVMVFCVGDLRFYGKENAQRRGEKNLGDRYLDNED

AKSDPFAIDIQNNAQLKKLYLEAEHEDGYIRDRNCFADGITIEDNLSMIIKYKNKAVMTY

NTYAYAPWEGYRCVFNGSKGRLEINVVEGGYSAGGEAVTKEGLGDLEKNYIQGGVEKTQI

VVYPLWDKPYEVEVVKGHGGHGGGDPVLLEDVLVGGKVDEFKRAAGIRDGGNAVLVGVGA

NHSMKSGLPIMVQDLVKW

>SoG_02232.T1

MKLSISALVLLSTALSGVLASPGDPKGHHRGYRPKCLCCSALAKELDGQVWTPDQQRYTT

RLGQYYSANAAQAPWCMVFPESAEDVSKVVKILQKNECPFGMRSGGHSAFKGSNGIKDGV

TVDFGYMNATTYDESTKIASIGPGSDWGKAYKALAPYNVVAVGGRADVVGVGGFTTGGGY

SFHTGARGFACDNVVNFEIVTADGEIVNANKEENSDLWRALKGGSGNFGFVTRVDAEVFP

SSRIYASLNIVPFDQKAAVRKVYLDFVNKQDEDPASQIIVASNYAKGAYSCGVILSNIDA

KENSSSFNDAKKLPISQSIPATGPANEVVPIFTGPTPLGLFANWQTGMLDHKLDIMEAID

KIMVEHIEKMRAVVADDAFEMIFQWQPVSPGNVRVMNERGGNILGLEAVVADGPALMYNI

VFTVNTASNQDIVLPIAFEMNAAIQAKADEMGRNKHWQFLNYAHGTQDPISHYGAENIAF

MKDVSRKYDANSMFQRLRQTGFKLPGSAGPRGPAN

>SoG_02250.T1

MDAHHFDDFGSFPFDGLPDDDHILSLADQHSLRIDPPPVHAFCQAAGIGSASTPSFSKGP

ASISSANVAFDIAHSHRTSSIDRQDDDGLTVITNEKQTPASGTAEESNAGDDMSVSHRGR

ADGTDLGGKPKDEKSDNTPTWSDLKTKAGKERKRLPLACIACRRKKIRCSGEKPACKHCL

RSRVPCVYKVTARKAAPRTDYMAMLDKRLKRMEERIIKVIPKAEQAATATVPRAVLKPSI

PGTNPTSKPSSRKRNAGEAFGGLDAFLKSSSSNEQVETEKANAQRIKEAEENHLFQEGAD

ALPSKEVQEHLAEIFFDNVYGQAYNLLHKPSYMRKLKNNQLPPVLVLSVCAIAARFAGNP

KFHPDDRKFLEGEEWASHARAICVRRYEWPNITILTCLLILGLHEFGTCHGGRSWALGGQ

AIRMAFALQLHKELDHDPSMPGSKTPLSFIDREIRRRIMWACFLMDRFNSSGTDRPMFIR

EETIQICLPVSERCFQLDMPVPTETLDGRVLSLDASKDGQQSDLHENMGVAAYLIRSVAL

WARIITYLNQGGRDADPYPLWDEKSGYAALCHAAEGFQGKMPEVLRFTTENLALHDTENT

AKQYLLMHITIQQNILFLNRAASMTKAHNGKTPPEAFISETVAKTFDAARRISFLLEEAE

KTSQFVSAPFAGYCAFTATIVHVMGIRLGNPLAATNTSPNVEVNIRYLNKMVKHWGMFYW

MVEHVRSEYRAALDAARAGKTGKDGGVSLLQYGDWFNEYPHGLADSDIMDPVNNNKKEKG

ADGVMEPKSELQSVEEFFTTLSPQNQEKSDGQKGQGPSKRKQAARKQSAATIRTGQGKHA

PAETSQQVASQISAQLQLQQQQEQQQQRRFSGQHHGQATGPPAYNSMSPASAQAGAFGVS

PISPVAIQNQFAQQTHGHGPDRAGFFASGMMIPQQTNPLLQSMEPQMMLDGFTLEANGVL

GAQGMIDGNPDWNTMQMAAMHNQRGMKRDQGAAMGQPHGGNGRDMMGGFDPQDTSWFLPF

NMEPPAPNTDVSMDASNLDAFNGLFNSNGNGMTTPNPLGGLQQGQ

>SoG_02261.T1

MPSILNDDDKDTVKRFVPKQTNKIQAVAVARLFVAYPDRSRWTYTGLQGAIVLANDLVGN

TYWLKMVDISPSARGVIWDQEIFDTWNYNQDRTFFHTFELEECLAALSFVDEKEAKQFKK

KMDDREKNASRATRSNPFGGSQPMHKSSRLGSLFGHRHSSAPTPPESPRSNLPPPPVSHN

TSLSSMSLNGSHHSEFALLDAFDPLWREHFGQDLRDKGLTDDFIKENQEFIVDFLREEQQ

NAQAATSAASHAPPPPPPVSNGHDAGRSRPPPPPPPGGRGHEESGGKTGAPPPPPAPRRA

TKAENESTPPPPEPPAPAGPKFRAPPPLADAGKFARKEVPPPAARPVPAAPGPGPPPPPR

PAKTPLDDDHSGHKFGVPPPFSGSRAVPPPPPSRNTGPPPPPSRNSGPPPPPSRNAVPPT

PPRQVHAAPPANAVPPPLPPKVPTGNAPPPLPPTSSRPVPPPPGSSAAPPAPPPLPPTNA

PVPPPLPPTNAPVPPPLPPTSSGGPPPPSPLPPTAAGGPPAPPPPPPPPNRDSGYSSGVP

AAALPKADGGRSAMLGDIQKAGGIAALKKVDRSQIRDRSAAQVGDSAGSGGPPAGAASAA

AAGGGGGMADALAAALQKRKEKVSRSDDESDNDDW

>SoG_02281.T1

MSELHNFENIFSLEGKTCLVTGGSRGLGLHMATAYLRSGCSHVIITARKREGPQGIDQAV

EKLNRLPGIKGRAIGIAANVGNTEAIARLVEEVKAIVGSKGLNILVCNAGAAWGSRFEDA

PPEASVKILDLNVRGIFELVQKALPLLEKAASKEDPARVITISSTAGTNVPHVGEHGTIM

YSTSKAAANHLARNLAVELGRRNITSNVIAPGFFPSKLASGLISNLGGEEELSRDNPMGR

LGVPEDIAGVAVFLCSRAAGYVNGVDIAIDGAARLSAGRMSRL

>SoG_02282.T1

MPYVSPFPNLDIPQTNILSYLFDSGGPVSDEPLWIDSKDTSKNLSPKQLLQWVKRLGFGL

QRLGLKKGDVAMICTPNQIFVPVAYLGIVGAGCIFSGANPAYTVPELVHQISNTTAKAIL

AHPSMLSPILEAASKVGVSKDRIFQFSDERNQPLHGVLDWQDMIGTPAEGDKWNWPQMSP

REAATTVATINYSSGTTGLPKGVCVSHYNLISNVEQTLVSRYAHKPYSLEDRPPERWVGF

LPLYHAYGQLYTILMNVKLQVPVYIMAQFGYEDFLEVIGKYKITSLQVAPPILVMLSKRP

ETSRYDISSVRDILCGAAPLSRELQNECQRRFDVQINQGWGMTEVTCGAMHVPGGIKDDS

GSVGQLLPNSECKLVDDDGNEVLETGKPGEMYVRGPQVCLRYWRNEAATKEAIDEQGWLK

TGDIAVCNKEGYFWIVDRKKELIKVNALQVAPAELEAALLENEDIADAAVVGITLQGNEW

PRAYVVIQEASKGKVRPQDVAEWLAKRVSKHKRLVGGVVFVDEVPKLASGKIQRKVMREW

AKRDAAEIEKQGGGAKAKAKAKL

>SoG_02288.T1

MYRDRRTLLLIVPLVAFFLVATSLYLNREYVSRVVLPKLHIPSVGQGKEAIAPEEATSIV

LLSSIATVLSATPTPTPTPTPTPTPLPEIVVGNGSILVGSTIHHYIDAILDPNVTTFERL

PCPAINETRYAPLKSLGDSDAGDAPIDFFFALNLRNNLPLLPRLIGSILEVIEFLGKQRC

AISIVEGISPDGTGDVLKALEPFLDRLGVRYYYSTSEIDSKKGNRIEKLALLRNLALEPL

LDLFNSTASANTTVIFLNDVAACPEDILELTLQRQTLGADMTCGMDWFSGGDEPTFYDVW

ISRSINGDSFFQIPLSGSWEFALDLFWSDPETKARFEAHQPFQVYSCWNGAAVFSAAPLV

EGVRFRDSAEGHCKHGEPQNFCKDLWSTGYGNIAVIPTVNLEYSNDKGLLIKELKGYVSD

AIVNGEGDAEIEWTGPPAEVRCIEDWAHQFWKPWELSRRDLPDRQS

>SoG_02289.T1

MVSVQTPGLLLLLAWFNGLSCLAHSASASGHAKFGLIGYGIHMWQPPCAYACQRLIALSP

LECSVEKHHEGRSLEGRHGGDKDDFSTSPDCFNHDKAFLLSLAWCMHQHCSDTHGAEPWR

LERFWATDAVPTSHHAPNYTYAEALDLCNGGLEPVLSRGHHLKEPSSVNEDDYAAMHTYI

KDYSRVEVQSTRYGVFLVFTMVALPLVLALGQHLLAAASPALERKLLAHVVQPALLGRRH

LVSLPYEIGMAPLRGQAILIALVVFLNTILSVVHYKGSMPSFKYNTESDAFWTYLSNRAA

YLSFANLVVVFLYSGRNNPLIYLTGWHYGTFILLHRWVAYICVAQGSAHSIVKLVRHIPV

FSEVFAKLYWNIGFGAFLAFGVMFATSILPFRRRWYQLFLDIHNLLAVVALVLCFFHVYV

KFGYNWGYENWVVLAGGLWVVERLVRLTKIASYGVHTAFVTPLDEEYFEINIGGATATGY

AHLYFPKGWWRLWENHPFSIASAVNPLAAGTHSYTVPRDIPEAMFELGADDGSDAASTRS

DEIDHGDADVMLKRQSGDIPLRSLASNTHDCSPDSPTTPLNFVENVGQEKLDDDEGDVPL

LPSGHQAAPSLSFLVRRQRGVTNQLFHATQRRKVLVEASYPSVSTLPSLSMATAQVVCIV

GGSGIAAVLPLLRARASGTAGRTVLYWGCRSKALVKASGVKSLGRGIETHVRVGQRLDIE

QIVRLEAHGCDGEVALVSCGPPGMADDVRHAVVEANRRTEGKGLLRLYEECYGW

>SoG_02290.T1

MPESIVNAIYYPSWRVYRGKPPSSLELQSVTHVLYAFLRVNIDGTLRLLDEFGDLRKPVD

GETGCLAAFAKMKRRNPHVRTLVSVGGGSGSSEFPALAADPEARATFARQTREFCDQYEF

DGVDIDWEHPTTTEQGTNFLQLLHAVRQTMPMPQYLLTTALPTGEYCLKTVDLERAAEYL

DFLNLMGYDFTGSWTEVAGHHAQLHAPNDSVLHHTTPDNKKSCARGVEYVMNAGFPSSKI

LLGIPAYARNFPLATRAGQSSHKIAGEVDYCDMPEHWIQDASVDTELGAAAIVDNEKGFV

SFDVPRTVGIKAKYAKAMGLAGLFYWTGTADRNGADSLVDAGYRELTARNGSY

>SoG_02292.T1

MSPSACNSDTAAMDGTGPREPRVLVPGIYVPTLAFFDPETDELDLKSITKHAVRLAKSGV

TGLAVQGSNGEAVHLTFEERARVTSNTRAALDGAGFQQMPLIVGCGAQSTFEAISLCKQA

AGAGGDYALVLPPSYYSALFDKATVASFFSDVADQSPIPIIIYNYPGATSGLDLNSDTLI

SLSKHTNIVGCKFTCGNTGKLGRVASAVKAKGENFLCFSGSADFTLPSIAAGGAGVIGGL

GNVAPRACVKVWELGQDARQAEQARALQAVVGQGDWMMIQTGVVGVKAALRDGFGYGGWA

RKPLPRLGDEKIEWIREGMKGLMEAEKKLENDS

>SoG_02305.T1

MASAVPVANGHASVARKSDDRDDDDDDLSSGGPSLLNENALRGPGRESNIASGGGGSDVV

EQHVWTDAPASTPISGFFERLGFSRRRSSASAAYTTLNEDDATSDDPSSPAPPIPRTRTG

QFFYLLRESFFSSRLNLLIVFVPIGAVLYFAEANPAVVFIANAIAIVPLSALLTDSTERI

ASDSGDTVGALLNISFGNIVELILFVALVNNHIRIVQASILGSILVNLLLILGSALLASS

MADLEAVYSTQGTQLLGCLLFVAVFAFLMPTAFDYTFKHLKHSKGAILKMSRICSFMILA

IYIFYFVHELKTRKKPVVVDEENTTPPAQPEMTMRPSSSHGSRHIRFADESLTSPKPLET

FEMQIRENGCSSKNQEESVDGNERHETTRRSLDSAARPSSSGSRGGPSGRIRSGSGASSR

MRSGSGSSRFGHGRNNSRESFTGSEGRRTYTRVPEDLGPEGRRVLMRSALPTRHILEAEM

LHPRHHVGVTRFVSVLVLMVTSVLMSMSAEFLVSTIDDVTHRGQLSESVIGLIILPIVGN

IAEYITVVTVAVKEKLDLAIAVSVGSSIQIALCVTPLTVLAGWVLDRDLMLTFNFFEMST

LVGTVLLVNLIILSEGTAGPRSSALKGGLICGCYAIIGIGAYLSPELE

>SoG_02308.T1

MKFSTLASALLSAGSAAAQCDLPSSYRWSSSGALAQPKNGWVSLKDFTIAPYNGQHLVYA

TKYDSAYGSMNFGLFSDFSQMGSVSQNGMSSGTVAPTLFYFAPKSVWVLAYQWGQWPFLY

RTSTNPTNPNGWGAAQPLFTGSIANSDTGPIDQTLIADSSTMYLFFCGDNGRIYRSSMPL

GNFPGSFGSSSQIIMQDSKNNLFEAVQVYKLKGLQKYLMIVEAIGSQGRYFRSFTATSLG

GSWTPQAATESNPFAGKANSGATWTNDISHGELVRSTADQTFEVDACKLQLLYQGRNPND

NPSDYNRLPYRPGLLTLQNPSGSNGGGGGNNPPASTSSSSPPSQTGGNGGGGGGGGGGNC

AALYGQCGGQGFTGPTCCSSGTCKKSNDWYSQCL

>SoG_02315.T1

MSESYPTLGQCALVAVAFKILLFPAYKSTDFEVHRNWLAITNSLPLSEWYFEKTSEWTLD

YPPFFAYFEWALSQMARLVDPAMVRVYNLEYDSWQTVYFQRATVIVSELMLVYALQTVIE

STPLGSKRAAQVAALSILLSPGLLIIDHIHFQYNGAMYGILVWSFSLARCKSTLLASGLV

FAALLCFKHIYLYLAPAYFVFLLRTYCLSAKSIFRIRLMNCIKLGAGLVAIAGAAFGPFV

ALGQIPQMLSRLFPFSRGLCHAYWAPNVWALYSFADRVLIHLAPRLGLPVKVEALASVTR

GLVGDTAFAVLPEITPRICFALTLLFQALPLVKLFGQPTWENFIGAVTLCGYASFLFGWH

VHEKAILLVIIPFSLIALRDRRHLGAFRPLAVAGHVSLFPLLFTPGEFPIKTTYTIFWLV

CFLMAFDRLAPASNKPRFFLLDRFSTVYIAISIPLIVYTSVVHQMVFGKSYEFLPLMFTS

AYTAIGVVGSWIGYMVVYFTA

>SoG_02316.T1

MVLYKRKPVQFLPPETVEDEDAEVWYIPQTGEIFASYENYLSRMDFYKQRRFNDQISGHS

GMTFFEALKNELAGGEEVEAAFPEALKGPVLRKAQFQTVSRLDHLVDMIFDTFKNDYFPG

ESVTVFYPDDDKRLAGLVRDKVTIGARPQADGSMSQPVTRYLIFVVGRDEESSFEETQVS

RERGVFTKSMIRSFMRKTVSREAWNGAPWLVKPDYAAQYYIDTRIPAHLRYDTKLMERKQ

LQAQKRASLPNDHSAGGPVRLPELKPAAKLQPKKATGGAKGKWPPDMAVHGANPAIPEMP

KIPREPTPPPPPKYPIEDLQLELRENVVRPALEFYCHDPPIKAANGTTNGTSRYSQVDME

SVGPLLETWDTLNVYCEIFKLDSFTFDDFVEAMCVASEQVVVQLFEEIHCSVLKILVDSE

ADGGKVRITLPQLEEEDDSDEDGEEEDEEETPEPQPKPTGRATRSSLAKAEAERLAAAAE

EESLRAELETKHRAEELLQDYDWIDELRKRNFPNGGWERIVVGLFHQLSKNERYEQRCEE

LLLQLVPPVEEPSQEAVSLKYANLDLNHRVKALQMMCMLTMETKTVRGYMDECSETMTKY

RKDKIEWQRQKRQALEDLRQLNEQRRDLQPPETAPNSPKPESVKEEEGESRIDADASQVD

KEQEEGDQTDQDVQSRRKRRKPLTEKQRKQEEERESKAKLKEKEKKEAAVPPPSKQLLKI

LKEIQKQEAIIKNCEDEVATIENDLREADCPRTRVMGKDRFWNRYYWFERNGMPYAGLPD

SSTASAGYANGCIWIQGPDDLEREGYIDLPEEQQKEYKEKFGMTIPERKAKEEGGTSVFT

ARQWGFISDPAKVEELINWLDPRGFNELKLKKELVLFKDRIVEHMGNRRKYLAGNDNADG

GEEEEAMTTVSKRTSSRIREKTPEPPNHLCLRWENTMVLEELGHLHSEAPPKPRQRKQSK

KREAAVAEATTVPAKKTRRK

>SoG_02317.T1

MIPRRKYRTFVLCAVVVLFLLYRISQSSREDQPGFRGTQAPPFPLDHETTAGRKVERPQA

LPEGVEPPRVPVEGFAKVNVPDLKEPAPVDRLAIEKEQAAKAPISEAGIDTSTDTTIPKT

PAPPADSDGDGEVAEEEKVHWVKQPEKFPVKDDDMKKLPSGTPKRLPKIQFDFSTESTEA

KAKREHRLQNITAEIARSWAGYRKYAWMHDELSPVTGKHRDPFCGWAATLVDALDTLWIA

GLKKEFDEAVKAVANIDFTWSPREDIPVFETTIRYLGGLLAAYDVSGGSKGEHGVLLKKA

TELAEILMGAFDTPNRMPVLYYEWAPEYASQPHRAVQVTVAELGTLSMEFTRLAQLTGKD

KYYDAINRITDALIEMQESRHTSIPGLFPQGIDASGCKKMEEPDPESLSDAAQSQLNAAD

AVGEPMGWNEAGDIITPAGHAPIDEESNQDPRLHKREAGGADDADDVLVVDAVQARKQPP

FTVKGDGQKWDCEPQGLVAGGYGDFHYSMGGSQDSAYEYFQKEYLLLGGLEPKYKKLHED

TVEAIDKWLMYRPMIKDADKWDILFPAKVMSWDNPADRIFQYEITHLTCFIGGMYAMGAK

VFGREKDLETAKRLTDGCVWGYQMTPTGLMPEAAEILACPTLNKCEFNQTLWEEKLDPSA

EWRNEAAASWDAKQEALAQMQIASANEAAVDKAPEVLAKEEADLLTEKAAAAEAIAEAQK

KSEGKLASGGLAKRAAMPPAAVVDSESQLPDSLKEKLHLESQPKPAKNKFPVPLAEHRES

QPGGKGEGSAPVVNSIPQLNVPQKAMDPETTEQTRPQTHEEYVKDRIESERLEPGLVNIM

ARSYILRPEAIESVWYMYRITGDPSWQEKGWNMYEATMRATRTEIANSALDNVMDVDSAL

NDEMESFWLAETLKYYYLLFAEPSVISLDEWVLNTEAHPFKRPS

>SoG_02324.T1

MASGHRTRTSEDYGVFDDAKTYYATDERHTNRSGVRTRTFSQNTLLKQFDNGSSKPPFRR

GSHDEVGSTSHRRFLIQVDPTLENLRAQEDTDDNMQITIEDSGPKVLSLRTAASAGHNKF

DVRGTYMLSNLLQELTLAKEYGRKQIILDEGRLNENPVDRLSRLIRDHFWEGLTRRIDAS

SIEVAARDPKDWTDDPRPRIYVPVGAPEQYQYYKKVAEERPEMRLDVQLLPAKITPDLVR

DMNDRPGLLAVATEERTDPTTGKKTLEGLPFVVPGGRFNELYGWDSYMESLGLLVNDRVD

LAKSMVLNFCFCIEHYGKILNATRSYYLCRSQPPFLTDMALRVYEKIKHEPDSKEFLRRS

ILAAIKEYHSVWMAEPRLDPITGLSRYRPEGRGVPPETEASHFVHILKPYIEKHGMEFSQ

FVRAYNYGEILEPELDEYFMHDRAVRESGHDTSYRLEGVCANLATIDLNSLLFKYETDIA

RTIRSVFGDRLEMPEEFCRGTPYTPGELLSSAAWDRRAKRRKLMMDKLMWNEEEGMFFDY

DTVKQERCTYESCTTFWALWAGIATPKQAAEMVRKGLPRFEVSGGLVAGTEKSRGEVGLD

RPNRQWDYPYGWAPQQMLAWTGLLRYSFTDDAERLAYKWLFMITKAFVDFNGVVVEKYDV

TRPVDPHRVDAEYGNQGLDFKGVAKEGFGWVNASYVYGLQIVNAHMRRALGTLTPYPTFV

KAIELNDEKALADLQLK

>SoG_02349.T1

MQVMNALISLVAVLATGAVAASCESVCPVIFDGRVPNNASVKDFDTANGGGWNPFNPGFV

KGNNLLWSQIIKLPEVAERSPFDAARDTRPLEVTLSDESIFQKQNGFRRAGLLFNKDSNT

GSPGSRGVVTLHLSLRVDEGRRANLSHEYLLAWHEAADFSGNQFNLQMGALIGQESVAEP

DTYKLLDRKNKLLWQTKILDGQWQNFGITLNFDKNTLAAAYSTGDKPLELVLEPTPNDNS

GEGQYQLGMLKKPTGTSDVVNSGFQESGLDEGLIYGGVFIEDSSGGCISRGEKAREVRMR

RRRRTWTRV

>SoG_02353.T1

MAAGNPSIRPKGREEQQQQQHQHRPQQHMTPPSSQRWDDETRWGSNTDGPGMEHVRSDQG

PEQQPAPSAGSRESSASSEDGPPQQNSAATPSQRKKRKMQQYQKISAEPACGWCASHNRN

CVYLERQKPGSRSGFNVELEAKVNRIDALLQVLGRRIEEHIANDHPAPSVQAATPGAPAA

YRSPPQAIHDASSSEFAHGPGPSSLGRATPMESGRTASTPGGGGGGLVDHYSRMDASSEQ

LGGGVDIQPPAGFTSLPYSPTNTATPRSLSTTPDLPPQDMIYTLVDLYFKHCNTWCPILE

RQTIFGAFFGSTSLSEPDRVLLHAIIATTLRFFKDPRLTPQMKAHYHATSRRIVQTYVLD

HVSIPAMRALLIICLDELGTANGPKGWNLLSLLCQNVKQAGLCEEISVYLLADADDIPRI

GSVRRVVAGRPESWIEDEGRRRLAWMVYLLDRYCATATTTFEFMLDDRRMKRFLPCSYDL

FCRNVPRETRLPSKVLEPLSDNPSNTNCNNAAQSDSLGSFSYHCEVLRIMSEVHNFLKTP

VDVTSPAEVAGWRNKHQLLDAALDRWLQSLPSEYSRISALCHSDPASRVANWFMLHSAYV

TAVIRLHSSAAYPTVRTEIFVPSHYAMQRCLSAVQSLRDLSRDVHEANGLDLLGPPFAFS

LWVAARLLLVHAATVGAPVDDKFDFFVETLVYVGQYWEVANNYARILKRVVQRGQEGDLS

LSDMRWRARDLVTLTGSPRPSGLDPTSTQASSLSELDSIDVFDFFHSPKVTPEVMAKTNF

GQTNFLHPSSSAMGGGGRQGPGAVPDPEADWLRMSQVYQ

>SoG_02359.T1

MTVTKPPYEPEMAAFLDVYPAPKDITRDFIPLMRQSPLSVLTADEVIAGEPYTHEERQVK

GHDGPVTISIFRPLQAGQASVGPTAAVKLPALFYTHGGGFLCGNRFTGAKDVLQWAKAAG

AILVTVEYRLTPEHPYPAAIDDCWAGLKYVGAHAAELGIDADRLMLTGQSAGGNLAAALA

LVARDNNGPKIVGQLLDCPMLDDKNTTPSSKQFVEEGTWSRGSNVLAWSLYLGPNAGRSD

VSAYAAPARSTDLSNLPPAFITVGSTEVFRDECVAYASGLWKAGVQCELHVWPGGFHVFD

ALVPTNPMSIASVKAKTDWVTTMLARKIQSKL

>SoG_02370.T1

MAPSQKRKAIEDDFIHTISDNDEDLVPAEEEELAPAQPPKKKTKTAKKGKKSKKGKQQDV

DPEESEQDEQQDEQQGIWGQNEEDDGAMNPDFEFQTEHVGDFAEEEFEGWGFEGAKKDMS

KQHATVDLDEIIRRRREKKNGKTESENGPKDSEEAGEDVEIDMDDDDDEVLADDAFGMNA

ASEDEESQAEGDEPSGDEDEDDEAASDNDSVATPVAHPDDDASDQSDSEVDEEEAAKRDA

FFAPEEPAKPGKKVDVSSFQAMSLSRPILRGLAAVGFSKPTPIQAKTIPIALMGKDLVGG

AVTGSGKTGAFVVPILERLLYRPKKIATTRVVILTPTRELAIQCHSVATKLAAHTDIKFT

LAVGGLSLKAQEAELRLRPDVIIATPGRFIDHMRNSASFAVDTVEILVLDEADRMLEDGF

ADELNEILTTLPKSRQTMLFSATMTSTVDRLVRVGMNRPARLMVDSERKTVGTLVQEFVR

LRPGREEKRMGYLAHICKTLYTERVIIFFRQKKEAHRARIIFGLLGMSCAELHGDMNQTQ

RIASVESFRDGKVSYLLATDLASRGLDIKGIDTVINYEAPQKLEIYVHRVGRTARAGRSG

VALTLAAEPDRKIVKAAVKAGKAQGSKIVSRVIEPSEADRMQAQIDEMEDEIEEIMKEEK

EEKQLAHVEMQLKKGENIMEHESEIKARPKRTWFASQDDKKKAAKAGQAELNGVKDSLKK

KGNGKLSNKDKKKLDARTQRTEGREWKKGKSSEADMKAAKKATKVAKKKGKS

>SoG_02391.T1

MASDASQAFSLQRRQGYQSQNLSDGGIVDYWGYAARVLPCTNDPGTCEYFESVYGGHERG

ILYVGIMWAVVGGLLLLWAVGRHFWTPRAPEVTRSNDAASRSGIQRLGNAIASTSRRYLL

PDCLRFVFGRVSRLQVLVLGILFTYVCVFSFVGIMWKTWTTPIGKAYRADHPGLMQTRSW

LGSWSDRIGTFAYALTPFSVLLSSRESILSLLTGIPYQSFNFLHRWLGWIILIQSIGHTI

GWTVIEVHFYQPQPVVADTWIKQLYMIWGCVAIILICLMALLATPWSIRATGYEAFRKMH

YVLAMVYIGACWGHWQPLKSFMIPGLCIWFVDRGARLVRSFLIHYSYLPDGSMGFRTADA

EMSLFRDEDNGDVVRLDFTHPHNAWAPGQHFYLCFTKSSVWQSHPFTPLNLPAEKDGLMH

HAYIFRAKKGETGKIAKMAAAVTEGNTHPSTPVVLQGPYGEDHLAYLSPDANVLCAAGGT

GITYVLPVLLWLVNQPVSPDRRISLVWSVRRRQDVEWVRKELDVLALAAKHGIKITIHVT

RELGSETSSSKTAAAAAEKSADCCAPSSSASSSSGPDSVLDVAPREHPDLSAVVPGFVSE

NVRGKTVVFASGPGGMISDLRRSVSAANSGAKVWKGDESADVQLVCDNRLEW

>SoG_02408.T1

MQTLPGLKALLLAILASTSCVNSQQCPEQPGAVNDWTWLGCYTEATASRALSGRTFASDD

MTLEKCAVFCAGFTYFGTEYSRECYCGNSFNAGSVLRGDSECNMKCAGSGCNYCGAGNRL

SVYSKGGTGPGSTSIQPGSSTTSALPAPTGFPDGWESYGCWVDGVNGRILNYQAPDDPNL

TLQSCVQLCAERGSAVAGAEYSKQCFCGNSIVNGGRRADSNTECNTPCSGNNNQFCGGGS

RMSIFALAEPQVVGPPGPIQTVGDWTYKGCALDNVNDRKTFIWQNFFPRTMTPEICLDRC

AEFGYMAAGLEYGEECYCGDPENMKTAGSVFVAESECNVPCPGDASAICGGGSRLSTYFW

EGDPFYSWTFPAAGSAAAGSYDFLIGGVVIPLITSQAITGKVTFVEKWGTGPPNSTGAYE

LDLSVIDDFSAAWRTMHVKTDVFCAAGVTLPDKAGRQLVVGGWSGDSTFGVRLYAPDGSP

GVKGTNDWEENVQELSLQNGRWYPSAMIMANGSVLVIGGQVGSNGAPVPTLEILPYTGTR

PLYMDWLERTDPNNLYPFISVLPSGGIFVAYWNEARILDEKTFATIKELPKIPGAVNDPM

GGRTYPLEGTAVLLPQRAPYSDPLGVLICGGSTEGPANALDNCVSIYPDAANPEWKLERM

PSKRVISCMAPLPDGTYVILGGAQQGVAGFGLATRPNLNALLYDPSKPLGSRMTVMANTT

VARMYHSEAITLLDGRVLVSGSDPQDGVNPQEYRVETFTPPYLLGGKLRPSFTLTNKDWG

YGQSVTFRLGAAARNGAITASLLGSVSSTHGNSMGARTIFPSVTCGGTSCTVTAPPNAHI

CPPGWYQFFVLDGGIPAIGVFVRIGGDPAGVGEWPAGADFTRPGS

>SoG_02411.T1

MSNAAASSSGSGNYMDQMASALNTAASYMKLPALASAGVAAVLTSLLYFKQKALIYPSNI

PANSRTELPRPSQFGITDFEELVIPTDDGEKLSAFYIRAPRNSRSRNVTMLMFHGNAGNI

GHRLPIARMIISYVGCNVLMLEYRGYGTSTGEPDEAGLMIDAQTGLDYLRGRAETRDHRL

IVYGQSLGGAVSIKLVSKNQAAGDIAGLVLENTFLSIRKLIPSLIPPAKYLTYLCHQIWG

SESVLPTITKVPILFLSGLQDEIVPPDHMRQLYELSAAPSKVWKPLPGGDHNSSVLEQGY

FEAMQEFVASVTGTEESEKENKHPVA

>SoG_02416.T1

MKWYTSILTLASTALAACNGHDALCSRRYSNITYMGAHNSAFDGFTPFHNQFVSVKEQLD

LGVRFLQAQTQDEKGHPQMCHTHCWALDEGPLEDYLGKISSWLDENPNEVVTLLLTNIDA

IPIEKFDDAFRTTGLRDYAFEPEKRLAKGDWPTLQEIIHLGTRLVVFMDYNMDEKKVPYI

MDQFKHWWETPYGITDDSFPTCTVDRPDRGDPTQLMGIMNHMLNYKVLGIVFPSMDAAIK

TNSARSIDAQVRRCVDEHKTQPNVVLLDWINVGQAFRVAKAMNGL

>SoG_02419.T1

MLALSEFLVLFELCTALDKSALDIDLDTSFISNGGDSLRAVALAAACKAHGLNLPRERIL

RSQTLRGIVSTTSSLDGTDPTQVKSYLMSKAVSVDATPLSRVSSTSSSDSSPSGMNDTGS

INHWLPQSANTSLAGSVAKDGLHVTPSATLPGVIGPPLTEMQLHFIHGSLRRPGTNAIVH

SETYDTIHIPVLREAWRHVIESEPIFNQDFPRYSAADAGPTGFNWVETTAEESGQKQEPE

KTLIGSFFQVAPVDGSPELSTIRWTVHHSLIDGYSASILFEKVLRIANGEQALLAGPSFL

QFTRELDSFREASRELGIAYWEDRQTELRKAQHELLLPPPSIADQGYVGSQKYLLDIKDI

ARDVQARAKSVGVTPASVFNAAWALTLSLFADADVVSFGAVLFGRSIDISGALDVVGPLL

NTLPLTISVVRGMTIEELLRNTFDELVMLEEYQWTTASDSGFDRTFETALSVQVSEPELP

DSWAIRPRERFTRQEHEVPLGITINPHREVSFDYHVNRFSADHIERLAQTFRRAMELLLA

PTGGLIDDVARNLLPTSSVEVLHRFGNCSLSTLNSSVKEDLVMLFERHARGIPDHVAIEK

GCDQMTYGEMDLAASKIASRLSNHIEQGEVVCVYSDRSMLWLCAIFGILKAGGVYCSMDP

MVPQEVRDRNFRLSGAKTFITAKPCQLPIVPKDCPFSFTVQSTLDSWEFEPAEHRRVASP

QAPAYVCFTSGSTGTPKGVVCAHAGLVAFQSSLDVRLFAAPGRKIAHVMSVAFDGSIHEL

FSALTHGATLVLPSGSDPFGHLHSVDSAILTPSLARLLDPEEFERLKWVYFVGEPVPQAV

CDRWASVKQVYNMYGPTEGTCGATIKRLLPRQPVTIGVPNPTTRIYILDSNKALSPPGAV

GELYLAGVQVAEGYLGLPQQTQERFLPDTIWPSGAGERMYKTGDRGYWTEDGEIALLGRR

DREIKLRGYRLDMGDLEIRIARAYPSLEAVAVTRNDDLLIAMVQPQDVSVDILREELRKA

LPQYAMPHTIVATDKLPITAAGKVDYKAVAKAASHPRDAQAHADKLASTAEVAVAEAYRL

ALQLPDDAEITASSNFVELGGHSLRQLELLRHLSAAFDVQLSLKMILVCPSVRELAKAIT

GCINSSQSVVRLDQRFAVSEELATPIETEWMRKYETSSGASSFNVCFSSVFDRSEVRKES

LIEAWNTVLARHQLLACRYTYRGDTKLIRINSGQVPRVQTPCSFNLWAEANRPFSLGLEQ

PVRVFVTDDRLTVVLSHIIADYTALGLLLREASDAYNGTALDEDPRSYSLANVWYGPPSQ

AILDFWTGYLQHCPEPPHPFGGQVRRSNYSGTSAISIIDTGIFEQALRFSSSANATMQQI

SIACVALCLDQDQAGTDILLGVPHINRDTADDLDTFGLFLQPLPVRIRHEADQAGSFMDS

VKTSSQMALAHAMSWHQLLQHLNIQTEYPNHPLFDVMVTMHDFRHTNDLKMEVPGLEPSY

VWSEGAKFKLLCEFTALPNGKLLLRLEYDADVVPGIEIRRLQKAIPLAMHMLSTGSQHGE

VKFAVREGMELPRDLAGHVLQDTKCLFGTCLRDI

>SoG_02440.T1

MVLVQSFVLAALAATATAKSAVLDLIPGNFDKVVFESGKPTFVEFFAPWCGHCKNLAPVW

EELATSLESQKDKIQIAKVDADAERSLGKRFGVGGFPTLKFFDGKSKDPIEYNSGRDLES

LTAFIAEKTGIKPKKKLEMPSSVQMLTDKTFSETIGGDKNVLVAFTAPWCGHCKNLAPTW

ELVATDFANDENVIIAKVDVEAGNSKATAKEQGVTGYPTILWFPAGSKEAVKYKGGRTEA

NFLEFVNQNAGTHRLPGGELDTTAGTIEALDTVVSKLTGSNIADLSAEAKKQAEALKDSA

QYKYAEYYVRVFDKLSKSDSYAAKELARLDGILTKGGLAPAKRDEIQAKTNVLRKFILDA

AKKVEEKAEELKDEL

>SoG_02452.T1

MADFPIPDATNPGAMDASRRLAGPIHAERHIRIVVIGAGASGLLMAYKLQKHFRNYSLQV

YEKNPEVAGTWYENKYPGCACDVPSHNYTWSFEPKLDWSAVYPPAAEIFDYFDSFANKYG

LKRYIKTQHQVIGAFWNNQKNGYDVKVRDNVSGNTISDFADVLINAGGILNNWKWPAIPG

IEKYKGTLLHTANWDTNVSLKGKHVGLIGNGSSGIQVLPAIREECGKVTTFIREPAWVSP

VQGLEQHKFSEQELGEFRNKPGALLDYRKQIESGLNGQFGIFLKDNQVNKDTRDYMLEQM

KLKLNDKYLEEKLIPEWSVGCRRLTPGVNYLESLTKPNVEVVYGEITSITERGCLCDDGK

EYPVDVLICATGFDTSFKPRFPVVNHEGQNLQDKWAVDPESYLGLAAANFPNYLIFLGPN

CPIGNGPVLSAIEAQADYMLKLIDRFQVNNIARFAPSEAAVRDFVEYKDYFMQRTVWADQ

CRSWYKGRPDGPILALWPGSTLHYIEALMNPRFDDFEFKHSGNRFAYLGNGYSQCEIDNT

ADWAWYIRERDDDAPLTTNGRNRLISKSGTVTTRELVSWSGDGKQVQAKI

>SoG_02459.T1

MAADKATATGADLGDGLRKRINVPAAPAPGVLQPQDNKKLAKKEPTFFESFDAWEPIFAP

ILFTLLAIGTRLWKIGISNIVTWDEAHFGKFGSYYIKHEYYFDVHPPLGKMLVGLSGVLA

GYNGTFEFKSGEKYPEELNYTFMRAFNAFFGIVCIPMAYYTAKELRLKKPAVWLVTLMVL

CENSYTTISRFILLDSMLLCGTVATVLCWAKFHNQRHRSFEPEWFFWLFMTGLSIGCVCS

VKLVGLFVTALVGLYTIEDLWNKFGDTKMPLTTLAAHFVSRVVGLILVPFLIYLLSFALH

FAILNKSGPGDAQMSSLFQANLKGTEVGKNSPLEIAIGSKATIKNMGYGGGLLHSHVQTY

PEGSGQQQVTCYHHKDANNDWFFYPNRNEPDYDSTSDEIRYLGDNSVVRLIHAQTGRNLH

SHDIAAPMTKSDKEVSAYGNLTVGDDKDHWKIEIIRDAASRDRSRLRTLTSAFRLKHTAL

GCYLRAGNKNLPQWGFKQIEVTCTKENNPKDTYTHWNVEAHTNEKLPAADAGAYKSPFFH

DFIHLNVAMMTSNNALVPDPDKQDDLASHWWQWPILHVGLRMCSWNDDVVKYFLLGNPFV

YWGSTVSLGFVAAVVAWYLVRWQRGFKDLNAQEIDQIHYAGIYPVAGWFLHYLPFVIMAR

VTYVHHYYPALYFAILTFGFLTDWLLRNRIKEVQIVMYGLFYTVIVGLYIYFIPICWGMT

GSNKEYSRLRWFDSWRVSDPQ

>SoG_02465.T1

MKATLALALGASLVSGQRLASSKCDDVHVFIAKGNNEPYPGRQGRLVSAICNGIKNCDYE

DIQFYNPLPAPYCESVAQGASNGVAQITDYNRRCPNSKLVVSGYSQGGQVAGDILGGGGG

VLFEDCQQPSNAGIPANSAAGRKKDEADRVLVVAALIFGDVRHTSNQPYNYLDGSGRNGV

FPRPASQLANLAEYTGRLRSYCDATDPICAGGTVVANHLNYFDVYSDSAAAWVQSLINAA

PEGTDGDKPATMSTKVYAPAPTTLIPPKHQNGTVITTTICPEVTQAIEAVATSTYQQPPL

TFAPGPSGTYVPPKPTDVSPPNGNAPTPVPTAGASTLERSALAVVAVAVSLFMMLRGLGD

ISQASQFAPTRSRDGWGAEHIIMARGGCDETRPACGQCRRGGRECPGYSRAMIFVDEGLK

LARKRKSKAEPSGSRKIITRNLSRAVNLQPVPDPAQLERFQLLSACVSTMFPKAPTRTSY

MGSWLWHLLPRLGHSKALDRAFMSLALSFLSSKNPLLRQYAQQAYGAALCSLQAALGDPR

DALSAETLAATLLLTYYESVASVDHAWVCHAGGAGRLLQLRGARRCCDSPFEYLMFLACR

GSLISEALLSGTPSFLDSPEWQAVPADVMKYPMLPSPVELSHQFFVLASSLPRVLHGKLP

SPCDRLLSVTEASSLRSQFSLWFQEYTSFENGARQPVIILAPNLFPKGPFHEVFLYYDTA

SATMIVTYYAYLILLNQHIAQHDMGDDTEESHDSTAKENIELARWICMSVYYCAWLAGVC

GTSTLKMVLPIARAALPSEYHSWVDQW

>SoG_02472.T1

MLHQRLAFAILGALAGFAAADGESDVHQLTQATFGDFVKANDLVLAESAAMRQARLTLVL

SPPPVFAPWCGHCKALAPEYEEAATSLKEKNIKLAKVDCTEEADLCKEFGVEGYPTLKVF

RGPENISPYTGQRKAAAITSYMVKQSLPAVSILTKDTLKDFKTADNVVLVAYVASDDKAS

NETFTAIAEELRDTYLFGGVNDAEVAKAEGVEAPAIVLYKSFDEGKNIYKEKFDADAIKA

FAKTAATPLVGEVGPETYSGYMSAMIPLAYIFAETEEERTKLGNEIRPLAEKFKGKINFA

TIDAKNFGAHAGNLNLKTDKFPSFAIQEIEKNQKFPFDQEAEITLDAISKFVEDFAAGKL

EPSIKSEPIPEKQEGPVTVVVAKSYEDIVLDDSKDVLIEFYAPWCGHCKALAPKYDELAT

KYAGSEFKDKVVIAKVDATANDVPDEIQGFPTIKLYPAGDKKSPVTYSGSRTVEDFIEFI

AENGKHKAAISPKEEMEEAAPAATEEGKEEKKEEKKKDEEKDEL

>SoG_02489.T1

MVSLKSLLIAASAVTTALGRPFDFLDERDDNSTSVIEARQVTGNSEGYHEGYFYSWWSDG

GGYAQYRMGSGSHYQVDWRNTGNFVGGKGWNPGTGRTINYGGSFSPQGNGYLCVYGWTRN

PLVEYYVIENYGSYNPGSNAQHKGTVYTDGDTYDLYITTRYQQPSIDGTQTFNQYWSIRR

NKRSSGSVNMQNHFNAWSQAGMRLGNHYYQILATEGYQSSGSSSIYVQTH

>SoG_02490.T1

MKPLALVSKVFLNRLLLLGGSIVLLSFLLISTTEYAPKGLPLPKFTNRHHYSQTSEDFWH

LFAPVRHPVDALNYTDNAGKLFKIKPDGPFWHKPLKNKVLIVEIDTRAPNGTNQILNETR

MDWEKVDMGKGKSKSAASLSFLNHFLYAQIHGYDYRFFNAAERPGIHNTWIKPAALARFL

QDYQFVVFIDADASISHLEVPIEWLFNRWGITQETSIAMPIDTAQVFGEDTHVSEDRFGK

IVLNSGFIVVQNLPITFKMLKAWGECPLVGLSKYPGCEKWLHRWSHEQRGFGEYVRYDFF

PNGKGMAEIPCDDAMGYPGLSDHPTLTVDCQGQFLRHHTMDKGMTKKSAEWATMQLMTDL

LHGQLMKGKEDYWIREDPPESVRFGPGGMLDQEVAEHIKAEKEEAERMEKAEKLKMEKEK

ASKESYQKAKAAEEKAEKEKIEAEKLAKEKAEADEAERKKAELDKAIQAEVDKKKAQLEK

EMLEEVERKKQELEAAHRQQIENQAAQPPNPPPAEVVNTH

>SoG_02495.T1

MDHWQQQHHHQQPPPTTQQHYHQQQQQPYVDAAGNPSRRQNGTTQQLPRDYVPQQHHLQQ

QQPPPPQQQHHQYPPASSSAAHASYKYDQYRGGAPVSAAHPASAGASAAASPLSVGPPQL

RDGNGDVPMHDAHDPHAGIKYPMRPHHQSHPSAGRVPTLQHPNQEPSAAAQRYSPMEALS

PASPYGSKPVQYGAPTSQRQSPTKPGDYPTSPYFAGRSQGQQLPPISPYTSAPDGYASSA

VANFDGQFNDPKSPRRHMAPQMPPQKGPVPEFKNVRALSDLRPKNSQQPPFRRANPEGGF

ISPLQALTCHLPATYRICNPNFKYETSRNPRRVLTKPSKGTKNDGYDNEDSDYILYVNDI

LGSEEAGHKNRYLILDVLGQGTFGQVVKCQNLKTQEVVAVKVIKNRTAYFNQSMMEVSVL

DLLNTKLDKNDDHHLLRLKDTFIHRQHLCLVFELLSVNLYELIKQNQFRGLSTTLVRVFA

QQLLNGLALLNKARLIHCDLKPENILLKNLESPIIKIIDFGSACDERQTVYTYIQSRFYR

SPEVLLGLPYSSAIDMWSLGCIVVELFLGLPLFPGSSEYNQVSRIVEMLGNPPNWMIEMG

KQAGEFFEKKQDEFGRRTYHLKPMEQYAREHGTKEQPSKKYFQANTLPEIIKMYPMPRKN

MKQSEIDREMNNRIAFIDFVRGLLNINPLERWSPQQAKLHPFITQQKFTGPFVPPMNLKA

SSLNRSPAPGTQQQQQAEALSKQRAQAAQASANSAAQGAYGALNQYGPPVHGQPPPMYGA

NNAVYSSGGSHSNVPPSYGTQGSQYGPMVMPQQPQQMPQASYGGPNPQQNMYQHQPQQPP

QGMRNNRQRASTMDQQQSGIPAAIQRVASHLDPSQPIRLQPSPAYYPPPGEGMPGVDQGA

NRQGRRGSRAQQGGRGNRDFIRNLEERTLEEGFMGNQPNPWH

>SoG_02506.T1

MIPSAVREPATPILTLSLVTLPIFLYHHHWASPSSAFWDLLCPQRGAIGNFDACFCPGFP

AEGIAHPLLLLNPANLFAMASSVRSVRAVAAAARPITFAGTNAAVKSFSTTSRVLAPWTA

ARPSIAPKINASARTAFRRAYADQAPKPKPGAFRRTFRWMWRFTYLSVGGLLGYTAWVIY

EDRHPQPQFEADPTKKTLVILGTGWGSVALLKKLDTENYNVVVVSPRNYFLFTPLLPSCT

TGTIEHRSIMEPVRAILRHKKAAVKFYEAEATSIDHERKVIKVTDNSEIKGATSETEVPY

DMLVIGVGAENATFGIPGVREHSCFLKEIGDAQRIRKKIMDCVETAAFKDQSKEEVDRLM

HMVVVGGGPTGVEFAGELQDFFEEDIKKLVPDISPQFKVTLIEALPNVLPSFSKQLIDYT

EDTLREEKIDIMTKTMVKNVTDSTVEAEISRPDGTKELVKIPYGLLVWATGNAVRPIVKD

LMSRIPAQKDSRRGLAVNEYLVVQGTRDIWAIGDCAVAGYAPTAQVASQEGNFLARLFNN

MAKTESTEARIHELSSGLNLQQGNAADAASEIEALERQLRRIKDVKPFRYSHQGSLAYIG

SEKAVADVSWWNGNLATGGSMTYLFWRSAYLSMCFSTRNRVLVLVDWVKSKVFGRDVSRE

>SoG_02507.T1

MSGYGGGGYGGGGGRGGYGRDRGDRGDRNGYGGGGGGYGGGRSNGYGNGNGYGGGGGGGG

YGGGGGGDRMSALGAGLQKQEWDLATLPKFEKSFYKEDPEVSNRSAAEVDEFRRKHQMTV

HGNGVPKPVETFDEAGFPRYVMDEVKAQGFPAPTAIQSQGWPMALSGRDVVGIAETGSGK

TLTYCLPAIVHINAQPLLAPGDGPIVLVLAPTRELAVQIQQEITKFGRSSRIRNTCVYGG

VPKGPQIRDLSRGVEVCIATPGRLIDMLEAGKTNLRRVTYLVLDEADRMLDMGFEPQIRK

IIGQIRPDRQTLMWSATWPKEVRAMAADFLNDFIQVNIGSLDLSANHRITQIVEVVSEME

KRDRMIKHMEKVMEDKENKILIFVGTKRVADEITRFLRQDGWPALSIHGDKQQNERDWVL

DQFKTGKSPIMVATDVASRGIAGRCYTISFMSANQLHKDVRNITHVLNYDYPNNSEDYIH

RIGRTGRAGAKGTAITFFTTDNQKQARDLVSVLQEAKQNIDPRLAEMVRYGGGGGRGWGG

YRGRGGGRANANQAPMGNRRW

>SoG_02513.T1

MADEKPSVLIVGGLGYIGRFLALHIHKNELASEMRIVDKVLPQLAWLAPEFSEACSQERF

MQADASRSDALARVFDRADGKQWDYVFNCGGETRYSQEDEVYKRRSLDLSLALGQEAAKR

GIKSFVELSTGMVYKSDSSPSKEGDKLKPWSKIAVFKLQAEEELAKIEGLNLVIARLPHV

YGPYASQWVATALCMARVYQATEEEMKWLWTKDLRTNTVHIHDAVRALWAMAAWNASGKA

KWDEKTMGKVPTFNIVDKGSTSQGALADIIADIFKIETSFQGQLISTFARLNLDSVVDDV

NDDTLPPWADLLNDAGITRPGPLSPFMEKELLKDTDLSMDGSRLESVLDFQYEKPKMTKE

LVEEVIESYKSMNWWP

>SoG_02519.T1

MYPGQGYNGSGNHHGGGYGRPPPGPPPPQQYYGHPPSGPPPPGPGYGQYPPQQGWGAPPP

GPPPGQYNYGAPPPGPPPLGPRPPVQYESAHGRPPPGAPPQHLDAYGYPVQRAGYASHAR

SGPPPPQGAQQFGHGAPGGYTFQYSNCTGKRKALLIGINYFGQDGELRGCINDVHNVSSF

LVERYGYKREDMVILTDDQPNPVQQPTRANIIRAMGWLVANAQPNDALFLHYSGHGGQTE

DLDGDEDDGYDEVIYPVDHKQAGHIVDDEIHFRVVKPLQPGVRLTAIFDSCHSATVMDLP

YVYSTKGVLKEPNLAKEAGQGLLGALSAYASGDIGGVAKSVFGFAKQAYMGDDAYKKTIE

TKTSPADVIMWSGSKDDQTSADATIAQKATGAMSWAFISALKENPDQSYVELLNSIRELL

ESKYSQKPQLSCSHPLDTNLKFVM

>SoG_02526.T1

MNEDEAIQNIYKKIEREKVLINGANAMRAQTNNEGVRSRLDSQMRDARRNLQFFEEKLRE

LQMRRVNQGMGDVSLGAAGEDGAPPAPPPKDSDGNWSSGDVGNYGNAQYSQIGGHGDLMP

PRHPYAPPGPGSSMPKSRPNFTKLDLIKYDTPHLGPRIQLMLSQIQFKLNVEEQYLKGIE

KMIQLYGMEGDRKSKADAAARRVESKQKIVLLKQALKRYEELHIDFDSADAQDDDSINTP

NLRKPLSGQLAIRVLAVKDVDHAMTGRFTRGPETFVTVKVEDTVVARTRASRNDRWEAEY

HSIDVDKANEIELTVYDKPSEHPMPIGMLWVRISDIVEEMRRKRIEAEMNSSGWVSADRM

GSTGGAPAQFPMSPSQGGFNNPASPSGAGQEGAFGAPGPQPQVITGPIDGWFNLEPTGQI

QLEMTFNKTNVDRRPVDLGLGRKGAVRQRKEEVHEMYGHKFVQHQFYNIMRCALCGDFLK

YSAGMQCEDCKYTCHTKCYSSVVTKCISKSNADIDPDEEKINHRIPHRFAPFSNVTANWC

CHCGYILPFGKKNCRKCSECGLTSHAHCVHLVPDFCGMSMAVANQILEGIRTQKQRQAKG

TSLTEKTLRAGRMSPTSTHSPSPSLSGSIAASFPSGSQEAADAARAMYGTQSSQQRPPAP

DRTSSSSTAAAAASAAMSSQAQRPSDYGRYGGHEQPQQPQAQEDPYGQGQYQPQQRRYNP

ADYANVNPQYQSQPPQQRPAQQIPPQQPPAHQQMPPPHQQAQQQVPYQQPTPAPKPPSQE

PVQAPVASAGQVTAQRKALPLATDPGTGQRIGLDHFNFLAVLGKGNFGKVMLAETKRSRK

LYAIKVLKKEFIIENDEVESMRSEKRVFLIANRERHPFLTNLHACFQTETRVYFVMEYVS

GGDLMLHIQRGQFGTKRAQFYAAEVCLALKYFHENGVIYRDLKLDNILLTLDGHIKIADY

GLCKEDMWFGSTTSTFCGTPEFMAPEILLDKKYGRAVDWWAFGVLIYQMLLQQSPFRGED

EDEIYDAILADEPLYPIHMPRDSVSILQKLLTREPDQRLGSGPTDAQEVMSQPFFRNIVW

EDIYHKRVQPPFLPTIKNATDTSNFDSEFTSVTPVLTPVQSGKYQALLTLGFEGAIANTI

CSIRNITRYWERSYDQGRTKCLVKEAAIEVGHGSEECDMARSWSHQDDRTVESQMNQHVV

TSNLIASRNGIDDDEGYSESSKGSGFA

>SoG_02535.T1

MRVVVGSSRLARSLVQTQQRRGFAVTARRLDNYGFIGLGQMGYQMAKNLQSKLKPTDKIT

IFDINAESMRGLEAEMKAASNGASVELATSALDASKDADTVVTVLPEPQHVQGVYKSILT

SDLPKKSRVFIDCSTIDPSTSRQVAKSVADAGQGTFVDAPMSGGVVGATAGTLTFMLGAA

ESLVPRLEPVLLLMGRKVLHCGDQGAGLSAKLANNYLLALNNIATAEAMNLGMKWGLDAT

KLAGVINVSTGRCWPSEVNNPVKGVIETAPANRDYRGGFGISLMKKDLRLAMMAAKEAGA

KMALADTAFKVYDDAEKLDKCAGRDFSVVYRYLDGKE

>SoG_02550.T1

MSSPIKALLFTATSGYRHTCISSLARALAGLPSLHLTHTESLSTLVDLLPSQDVLILGHN

TGEYLDDEGKDAVRRFVEGCGGGGGGDGGGQAKKGVVGVHAATSGMKKWEWYGEMLGGAF

SEHPDPQWGVVRLHHYRHAAGQAGDGEEDGEEHFILRDLPEPSSSSVPSSAPPRSQSATA

PTDFPWFDEWYNFNPEPRIPPRDGTVLLSVDKTTYEGWNGPEDGRTQHPLAWCRETRAGT

RVFYTALGHFEEAYEDPWFMAMLERGILWAARRDV

>SoG_02552.T1

MFRARTDFLFIHPPNFPIVLTETPLVRDPAVSMLQSARSLARPALRTACRRSQCPAARRM

FLSRPSASDGASNDLDINRIRAQRRDYENNRTAFLAAGAVAGIISFIYTAYKLKQALDER

AEKEKKGDGFKGANLDSPVNVDTFKTEAGEKRKVVLHDEDGSEIVPTGNSVIPAFPRLLD

VKLPTVPATEPSSPIAASINSSDGTEFTLVGLGMRSVTFIGINVYLVGFYVATQDVEKLQ

HYLVKKVNPLATTLIPTEKDTLRKSLKDPNEGEQTWDALLRDAGCRSIFRITPVRDTDFH

HLRDGFIRAIQDRSKGNSQFSDEAFGAAVRTFKEMFNRGSVPKKKEMLLCRDAAGKLSVM

YGNGKKGSKLETLGTVDDERVSRLLWLNYLAGSKVASPQARENIIEGVMEFVERPVGTVA

TQVL

>SoG_02553.T1

MPVITERDLTDLVLPESASDLKDGSLLDSFSLVSGPETRFQGFEWKIEFPFPNAYRIIVT

GPDRPRPPHDNLNAPSDFSSFKLATLDREKCLAVFDFPQATDASTGLSCLREERLQLRLH

WKHQVCSQVVSVEGHHQNSGSGSADLSKKESLSSKLIISDLEARALALTEHGMIRHWPID

RTRLHLGLGEKAAPIDLTGRSFAMHASDSAYYDTYRTDPLYKHTPFLISTPRPTEDFEQG

ITYAIAHGTNSTATWDVGCEIDFPSGGLSKRYVQDWGGLEEWVMFGRGVEGVVRTFAELA

GRPMLLGRDWLGYLGSTMLLSDKENAQELLEEWPEMCAEHDIPCSAMHLSSGFTVEEESN

DRWTFHLNEKRYPDFKGMMKIYHKAGIKVIPNVKPYLLRNHPASDRLIKAEGLYYDPITK

GPSKQNLWSSGEGVSGAGSWADFTAQETRKWWAEGVRKFIDLGVDGMWDDNSEFYTRDDD

LLFKNDMPEGRREVATPAGPVRTGLMGRLLGNELMNKISHDTLLAASPTRRPFVLTRSAN

IAAFKYACSTWSGDNLTSWHNLRGSQHIQLSSAMSLMNSTGSDIGGFCGDAPGPELFVRW

VQLGVTHSRFCIHSGSLDTQGREKLSTPWMYPEVLPIVREHIKWRYMMLPFLNNLMWRAH

LSASPTNAPLFYGPFSKDANLYRQETLEGFNSWLGVGQLLVAPQLHEGGATQNVYFPKAS

REDKSLYFNLHAPFERYTAGETKAVPTPIEHGALFAREGAVIPIGKPKATVTALSGDPRT

HTDGVDVILESEGGQVGLDDWRGVLLFPGREGRSYTDAWIEDDGISVNPGTSRVRVTYTG

REDIVDVDVKVEEKGFTPLWKGELQVVLPMGDLRAVSGANRSTWNGRDAWTLKIE

>SoG_02556.T1

MLTARVVFPALAAFFGLGSAQVTTKCNPMERSDCPADPAFGTSKNFAFNETQNGALWETV

VRGVTYDPKNGAAFTISKQGDSPTLRTNFYFFGGRTDVIMKAAPGKGIVSSMMWLSDNLD

EVDWEFIGVNKTHASTNWFGKGVEDFHHAGWHSMAPPQDDYHNYSTVWTKDKLQWYIDGN

LVRTLLPKDANNTQAYPQTPMRLSVGIWAGGDPSLPEGTRQWAGGDADYANGPYTMYIKQ

VYVEDYSTGSKEYVFGDRSGSWDSIQVVKGNSSAYEAMHTVPEKTMSEKWAQDVPQTAKI

AIYAGASAVGALLLGGLVFYCIKQRRRGAAEAQAAEARAQADAAEEARLKREGIDPDGFR

EQGHEYDAHELRNIGNATANSYNVSPTNEKSWAAMGAGAGAGAVAAAGGMRPTVPLLRDG

AQSPRIQSPGPMSPYHDAPRSPGSPGPGTMGGQYRTQSPGMQRMQSPVIMSQSPVMPPQG

ALPPIPYQSRGSPGPQRMQSPGPMGPVRSFSDNQPAYGANRMQTASPMYPNRGFSQGQAS

YRGGPPQSRDHGNNGGGNY

>SoG_02558.T1

MNIQPPAATTVVRGFQATPVTSADEDSDYNSGIDTPRLKSAQRGRTHRIDDVISANCSPT

LRPAASPSISGIAKLRMQMEPLSLEEASRSSSMSRNGSYQGRNVRYYKGMANSRSHSSDG

ARSDAGSDGSESYEVNLEHDFVSESVRDRNGFMDQFEGGLNPKRKMTTEDFETLRCLGKG

TYGTVHLVKQRLTGRLYAQKQFKKASLVVHKKLVEQTKTERQILESVNRHPFVVKLFYAF

QDQEKLYLILEYGQGGELFTHLNTEKMFPEPVAAFYMAEMLLAISHLHNDLGVVYRDLKP

ENCLLDADGHLLLTDFGLSKVSAEEDDDSCKSILGTVEYMAPEVILGKKYGKAVDWWSFG

ALGYDLMTGNPPFRGQNHAKIQDNIVKQKLALPYFLGPDAKDLLTRLLRKDPNKRLGASM

PKDLEAIKKHRFFRKIDWKKLAARELEPPIQPMITDPELAENFSPEFTELSISPVVTRDP

GFFNLAKDDPFGGFSFVAPSSLLEGNAFPMAAAP

>SoG_02560.T1

MAPSAAITDRDALVNRAVPFMALSDGSESSMTAPPRVPVAADPAQGARERFQVRGNAIVT

GGAGVLGLRACDALLEHGASGLAIVDVNPDQARKDIEAMRSKFPDAKITTSKVDVTDEVA

VEAAVSEAATALGSIDALVCFVGVVGCVESLLMPISQWRRILDINTTGAFICAQAAAKRM

VEQKSGGSISFVSSISAHRVNYPQPQAAYNVSKAALVTLKNSLAAEWACYGIRTNTLSPG

YMDTILNEGDGIAEHRRIWAERNPFGRMGQPSELTGALVLLASNAGSYINGADIVVDGGG

IVF

>SoG_02562.T1

MSPPAANGTRPRAASSRPLSSRACLECQKRKTRCNLAGDSLSCAYCLRANRRCIFEAAPA

RTPLTRKNLDALEKRCNHLEDLLRSMNLGGLAAAADQTRPGYTEDNGHQELAENQSSSPD

ERFNWNESPALGGSKSPYEEDNGGLASMAVESSGYLGSSSASNIVQSILPQEKPTSPPKD

TRHRGPSASNPLRPRNFYLSASLASAENTRHLVDAYFLYFNRAYPILHEHAFREQSRHWP

RNGPVSCADAIYCLVLAIGQWLLDEEAQDSVYFNAARSWLSATALESGTHNAVQAFLLMG

NYVQKRGKPNTGYNMIGIAYRMALGLGMHREVDKASQNLVLAERRRQLFWTLFCFDSGLS

ITTGRPTTIIDSFMDVRMPRNVELEQLTYSKYLDAESAVPEEASHPTTASAIIAQSQLAM

IANEIQTRFWSLKSAQLDSTHDAALGMEVKLLNWRNSLPRYFTSHNVPDWFLGPRAVVTW

KEQSLRMMMWRTHQRHNLADTTRAVADLKCYAAAVETVRDISDFCNENAGVLHTGLAWYA

IYYVFQAVLVLEISNMKQSSSGPRPGSETPSSQYEIAAWSKAIEQGRACLQLLDKPTTAA

SRCLKTLDRVRASVQRNAGSPPPSPTSRVGQPHPANHQSYHPTATAQGDAALPPAGQQVP

AENYGMQPESAAGGPSDDMVWMASADSTLHMLLNDSQMDTFFRGMDGFPGTLDNDLYGYF

TEWPPTS

>SoG_02569.T1

MSPPGIDGFTCFWWSEFLTRDKQLPDDSKWEMVKRDPNYGNNEEQFFIADISVAAVWQGE

LCIMPQYDGKRWTSARLHCRENFHADDGRIMVIQAELKTGVLGKEFWSGIWPSFWTLGKS

YRGVGDAAWPACGEIDIFENASGESFTIPAAHTTGPMIGEVGYHVPFNRDEWNVWAVRID

RKNSNGDWQKETIEWLLNGRVIYTWKGEWVGNFEQWKSLAHQPHFPILQVAVGTNWGGGS

KPNDKTATGAGVGLRCRSVAVFFDGHWSKLKREMKPSAAFVSYDDLVFRFHSLEDAKSFA

STNKSAALGTVDKHNVHVHAPSSLRHVRAYPGKGVIGLVFYKKADRDAFLHGMGPIGDLD

TTVDQSSWRVFVPHTPGKHDH

>SoG_02575.T1

MAGYRPIQQQDPGPPHATPKSTPRFPTWPSLLVRHSNSDVHWHHEEGDHRSSAASCRAFS

GRHLRRSILRHSRAFRWALIFGVFALIVTLLAILQSTKEAYLDMRWVPDPDTLLFEQQPL

PPPLERTRIANYSLPLRTRGRYIVDAQDRRFKLASLNWYGASDQHFVPGGLDVLHRKDIA

ESIKQLGFNSVRLPYSDEMVMSNPVPGHEHLLANPDLMGLRALDVFEAVTATLTDAGLAV

IINNHITSATWCCGADPCDAAWTNSYLGPLCRISMSEEQWILNWEDVMSRFVRNPLVIGA

DLRNEVRGLWGTMPWSSWAAAAERCGNQVLALNPDWLIIVEGTSSANDLSGVRSRPVKLS

QEDKLVYSAHVYGWSGWGSWAGRFKQQAYSTFREAMRYNWGYLLDGDIAPVWVGEIGAPR

QATEGDVIYWKNLWHYLKSVDADFGYWAINTEKPSGERESYSIVELDWKTAVKDYRLKEM

VEHMRE

>SoG_02586.T1

MSPAMTALNDPVATPPRPPTPTQGFGTLAVHAGSPHDPVTGAVIEPISLSTTFAQSAVGK

PVGEYEYSRSANPNRTNFETAVAALEHARHALAFSSGSATTAVILQSLAAGDHVISVSDV

YGGTHRYFTQVAKAHGVRVTFTPEIEVDIAEHINDQTRLIWIESPSNPTLRLVDIRAVVS

AAHRHGILVVVDNTFLSPYVQNPLDLGADIVVHSVTKYINGHSDVVMGVAAFNSDEIHKR

LAFLQNAIGAVPSAFDSWLAHRGLKTLHLRAREATHNATAVATALEASPNVIAVNYPGLD

SHPHRHIARKQHRDGMGGGMLSFRIKGGHAAAERFCQLTKIFTLAESLGGVESLVELPSS

MTHAGIPRDQREAVGVFDDLVRVSCGVEDAEDLKKDVLQALEAAVAGSKVAANGN

>SoG_02614.T1

MKLSSIGLIAGFVGTSLGHAIFQKVSVNGTDQGQLKGVRAPDRDYPIQNVNDGEFACSKN

IQHKDGTTINAPAGARVGAWWGHVIGGAQYANDPDNPIAASHKGLQWFKVAEQGLNNGVW

AVNDMIRNGGWHYFNMLSSVAAGDYLMSVELLALYSAGTQGEAQFYMECAQIRITGSGSN

AGSNFVSFPGAYFATQSGILINIYDNTGKPNGGGKPYSISGPHVLSC

>SoG_02629.T1

MAILPALPPRRLLPLLKSLISPAVEPHQPSCGTEHGQPPCSTPPVITTTPCQGEDCPCDE

YGCQTRSTTTKASTTEETTSKPTTLETSTKPESSADSSTKPATVDTTSVTAKTEESTTKY

TTSTVYTTTCYTVTTCDHDCPHGPTTTTITETIPITTTVCPVTETHPTPPPPPPATSSEH

PPPPATRTEETTSKPTTLETVTKPGSSADSSTKPATADTTPVTAKTEESTTKYTTSTIYT

TTCLTITTCDHECPNGPTTTTITNTIPITTTVCPVTETHPAPPPPPATASEHPPPPPPPQ

ETKPPVGTPPTEKPPTEQPPAEQPPASQPPTEKPPTSQPPAQQPPASQPPAQQPPASQPP

APQPSAPQPPVQKPPTEKPPAQQPPAGTGPVQVPPPPTVPGYPSPTSPQIVPTAAAGRIS

GSYEALALVAAAAAFLL

>SoG_02643.T1

MTVQAAHFTPEVLLSAPRRSPGIPNPTGDLILYTVSAYSFETHSKSLQIRVHSLKDSGSH

VVSDDATAHEPVWISDAEILFLVPGKNGTTSMMRLDVTKQPLEPELVYNSPGALSNPKVK

ILSDTEVVFCCSAPTTPSGKLFCEASESKPLSTAKIYDSLMVRHWDTWLTKNRSSLWYGL

LEKKDGSWSLGDSGLKNLLAGTCLSSPVPPFGGTGDFDVSSKGIAFVAKDPELNEARYTK

TDLYYVPIRDFRSRPTAPQVVKTGRLRGYSMAPAFSKDGRKLAFVRMRSDQYEADKTRLL

LIPDVEDLSNVQEFYETEDGEGGWELRPDWIVWSHDGRELFVAAEQYGRSVLWKIPASPR

HATQLPPEAIHEDGCVVDAKPLGSYNSLFITRRSRVESSSFAILDPNTKTVTEVSSSLKH

GKSVGLRQSQCQDVWFPGGAGYDIHALVMRPSNFDTSKKYPLALLIHGGPQSAWTDDWST

RWNPAIFAEQGYVVVCPNVTGSTGYGQAHIDAIAKNWGGTPYEDLVKCFDYVEKKMPYVD

VDRAVALGASYGGFMINWIQGHDLGRKFKALVCHDGVFSTRSQYSSEELFFPEHDFGGTP

WDSIEFYDKWDPAKFLGNWKTPQLVIHNELDYRLPISEGLAMFNVLQARDVPSKLLMFPD

ENHWVLKPENSLVWHKTVLDWINKYSGVGDAPHQ

>SoG_02658.T1

MPRLASTKTPDGGEYEYVTPHGYSYYANATSISPEFYQVLLDRNWRRSGELMYRPNQRKS

CCPHYTIRLDSHEFKPTRSQRQVVNRLNNFVLGENYIKEAARLYPRSRDHAKKRETEFSL

VERIHEAEDIRVKTPPQPAHKLEVTLESNEFTEEKYLVYDNYQKVVHRDAPSERTQRGFK

RFLCSSPLRAKTEVGDDGKERRLGSFHQCYRLDGKLVAVGILDLLPQCVSSVYFMYDESI

AQFSPGKLSALGEIALALEGGYRWWYPGFYIHSCPKMRYKMDYAPQMVLDPETLTWDPLD

QTILQLLDEKSYVSISRERNANAEDEMEVAEEADKDDSSLFRSGFPGIPTIAEMEGVNLD

EIPVKFWKDIDVLLQTPDLGGWKRSSLEGVRGVKLMVAEMVAAMGIDCVSKVCLDLSEMM

EE

>SoG_02673.T1

MADINVDEVLKKLTISDKVDLLSGVDFWHTKALPEHGVPSVRLTDGPNGVRGTKFFNSIP

AACFPCGTAMGATFNKELLEECGKTMGEEAKAKSAHAILGPTINMQRSPLGGRGFESIGE

DPFLAGQGAAALVRGIQSTGVQATIKHFLCNDQEDKRMGVQSILTERALREIYALPFQLA

VRDACPGAFMTAYNGINGTMCSENPKYLDGMLRKEWGWDGLVMSDWFGIYSTSQSVEAGL

DLEMPGPPKFRGEALKFIASTGKPILHKIDDRAREVLKFVKKCAAAGVKENGPEETRDTP

ETAALLRKVGNESIVLLKNENNVLPLKKNKKTLVLGPNAKTAFYHGGGSASLTAYYAVTP

FDGIKSKLESEPGYTVAAYTHRFLPLLGAQVKNATGDTGMSFKVYNDKPDTEGRQPLDEV

HVKKTEMHLVDYYPKDLNSLWYGDLEGSMVAEEDCTYELGVVVCGTANAYVNGKLIVDNS

TKQRQGEAFFGSATLEERGRFEMKKGETYNFKIQFASAPSFTLKTGAFTPGHGSLRVGGC

KVIDDQEEIRKSVALAKEYDQVVICAGLNYEWETEGSDRESMKLPGVLDQLISEVVDANP

NTAVVMQTGTPEEMPWLDKAAAVIQAWYGGNETGNCIADVLFGDYNPSGKLSLSFPKRLQ

DNPAFLNFRSEAGRTLYGEDVYIGYRYYDFADLAVNFPFGHGLSYTTFAFSGISVSSKGG

KIVTTLTVKNTGSIKGAEVAQLYVQPKQKAKINRPVKELKGFAKVELEAGESKSVTIEEL

EKYAASYWDEERDQWCVEAGEYEVVVANSSAITKENSVRGSFTVSESYWWSGL

>SoG_02674.T1

MNRDASLTAGRMAAPRLQEVSGSHTLTKHLAIEDAKNTQKLVEDSCAIVGHDSPPYILSE

LIGKGSFGRVYKASRKTAPTQVVAIKIIGIEDADSFNPGTSDTLRDVLHEINTLKLLAEG

GAKNVNSIIDTLLVGSTIWLITEHCAGGSVSTLMKPTSGLPEKWIIPILRETAVAIQWIH

KHSIFHRDIKCANILITEGGHVQLCDFGVAGIISSRFDKRSTVTGTTAVDGPGVLRLQSV

LWDRG

>SoG_02680.T1

MAPVNSLHVLCFGDSLTSGFHHFGMSSHPYSHRLAARLKEAFPDLEKIHVVPNGVPGDMV

MNAGFLDRLEKAGTQNTHFPCSNRFEYTLADIARLLPVDRGIYDWVLILGGTNDLGSRAL

AEDTASALYEVWNTAMAQDCKVLAMTVPECAAKLTWLDSNRDMLNHMILTHEDENYHTFD

LKSKIPYHSLTDEERETYWDDGLHLTEEGYDWMGDHIADALIPLVKQDRRISLKKSIITP

VIPPRVWGDDTPLEEEIGDPRDIHSGYVVVRKKDLD

>SoG_02692.T1

MAVSSTASNLEREDHARDANFNKALHGKSAQARGGIAAMLSKGADAQKAAVDEYFKHWDN

KAAKDETPEERAARTAEYATLTRHYYNLATDLYEYGWGQSFHFCRYSHGESFYQAIARHE

HYLAAQIGIKEGMKVLDVGCGVGGPAREIAKFTGAHITGLNNNDYQIERATHYAKKQELS

HQLEFVKGDFMQMSFPDNSFDAVYAIEATCHAPTLEGIYSEIFRVLKPGGVFGVYEWLMT

EEYDNDNLRHREIRLGIEQGDGISNMCKVSEGLAAMKAAGFEMLHNEDLADRPDPFPWYW

PISGELRYIQSIYDVFTIVRMTKWGRWAAHNFAGAMEALKLAPAGTKKTADSLALAADCL

VAGGRDKLFTPMYLMVGRKPE

>SoG_02710.T1

MSSHRPNAFNSLRMGEVIREKVQDGVTGETRDLQYTQCKIVGNGSFGVVFQTKLSPSGED

AAIKRVLQDKRFKNRELQIMRIVRHPNIVQLKAFYYSNGEREFVPETVYRASRFFNKMKT

TMPNLEVKLYIYQLFRALAYIHSQGICHRDIKPQNLLLDPNSGILKLCDFGSAKILVENE

PNVSYICSRYYRAPELIFGATNYTTKIDVWSTGCVMAELMLGQPLFPGESGIDQLVEIIK

VLGTPTREQIRTMNPNYMEHKFPQIKPHPFNKVFRKADANAIDLIARLLEYTPTERQSAI

DAMVHPFFDELRDPKTKLPDSRHGSSQLRELPDLFDFSRHELSIRPDLNQQLVPPHMRSV

LASRGLDIDNFTPLTKQEMMAKLD

>SoG_02727.T1

MIFSRKAVAAGLGSLNLLAQAQQDWTGTPFAAPFREPLHIPPLKQPFTQVTNPVTNQPID

YYEIEIKSFHKEVFPGLGSTSMVGYDGMSPGPMFLIEKGREAVVRFTNKAEQNSTIHLHG

SYSRAPWDGWAEDHIEPNKYKDYYWPNSQSARLSWYHDHVHHQTAQHAYFGQAGGYIIRD

PAEASLGLPSGYGEYEIPIILESKSYTPQGVLIAPGAPNGGSQFLNIIHVNGKAWPYFKV

EPRKYRFRLLDAAINRAFELFLSETEQGEKIPFQVISSDSGLLPEPVTAPTLSIAMAERY

DIVVDFAPLAGRNVTLRNVAAANTVFAELNSIMRFVVQEEEVEDPSSVPSTLRQLPVTPS

SDGTVTHQFRFARTGTPARWTINEIGFGDDSRILANVPRGTVEIWELQHGGGPVHPIHVH

LVDFRIISRTGGTGIVRPYEQGLKDVVWLGPGETIRIEAWYAPWDGLYMFHCHNMQHEDD

EMMAAFNVTQLEELGYPETSFSDPMEARWRAVAVNAQDYDPDAVKNKIEEMALLQPYSNI

VEVEEALNEFHGYSEEASRVNEPTRIKSRRINLMK

>SoG_02729.T1

MDRHKAAVSKIASAVKTYFERGEPFRITHGSTNSTRPRPTGRLVDVSPLSNVLSVDRDSR

TALVEPNVPMDKLVESTLAHGLVPPVVMEFPGITAGGGYAGTAGESSSFRHGFFDSTIRS

VEMVLGDGQVASATPKNQYADLFRASAGAVGSLGVTTLIELNLLEAKKYVQTTYHRAGSV

REAIEKIRQETANPDNDYVDGILFSKDHGVVVTGRMTDQKPSDAPVRTFSGAWDPWFYMH

VQDSTRSPARSSVTEYLPLAEYLFRYDRGGFWVAAAAFTYFWFVPFTRFFRWFLDDFLHT

RMMYRALHASGQSARFVVQDLNLPYENAEKFIDYTAEEFAIWPLWLCPLKQAPPPTFHPH

TGETRIATDPVTGEETRVPEESLNIGLWGWGPKSYPAFLQKNLSLESKLRELNGMKWLYA

HTYYSLPQFWDVYGGTKTQAWYQSLRKKYNAEHLPSVYDKVRIDVDARQRELAQRKGSFK

NMWPVAGVYGIYKAIKSRDYRLHRDAKWKYKGE

>SoG_02745.T1

MSFLGGGAECSTSGNPLSQFQKHVQDDKTLQRDRLVSRGPGGQLNGFRSQNVGGSQDEMM

NGFLNGAPSMQQEMPMQGGPAQLPQHHPAQLRASSTSPTWAHDFNSQPGMEATHKPAGVP

FLNADEFARFQNMHSQSPMSHPEAMQNNVSPQMQQSRPMMGMGMGMGMNYGQPMFQPMYQ

NQQYHQQQKEPEGKGKGRLVELDDNKWEEQFAQLEVQDKAEAKAKEDEEANAAERELEDM

DKGIQSETNEFGDFESIWRGIQAETAAARSMVNEEQFFDQFDAQWNKDSLTDLSHLDSWG

RYGEPIPEQYRFEEENIFQDSKNAFEEGVKIMKEGGNLSLAALAFEAAVQQQPDHVEAWV

YLGSAQAQNEKETAAIRALEQAIKLDPNNLDALMGLAVSYTNEGYDSTAYRTLERWLSVK

YPQILDPSDLHPPAEMGFTDRQQLHDKVTKHFIKAAQLSPDGEHMDPDVQVGLGVLFYGA

EEYEKAVDCFQSALHSSELGTSNQQEQVHLLWNRLGATLANSGRSEEAIAAYEEALNRSP

NFVRARYNLGVSCININCHHEAACHFLAALEMHKSIEKSGRAQAYEILGEGSGARVDETI

ERMSAQNRSSTLYDTLRRVFSQMGRRDLAEKTVAGVDPAIFRPEFDF

>SoG_02746.T1

MKSFFGVQAATLASVLLARSHAAVVEHDFNVTWVTANPDGMLDRPVIGINGQWPIPRIDA

NVGDNVVIHLHNQLGNQTTSLHFHGLFMNGTTQMDGPSQVSQCPLQPGDSMTYNFTIQQP

GTYWYHSHTISQYPDGLRGPLVIHDPEFPFRKDVNEEIVLTLSDWYHDQMQDLIPPFMSK

GNPTGAEPVPQAALMNDTQNLTISIQPEKTYLFRVVNMGAFAGQYLWIEGHNLTIVEVDG

VYTKPTVAEMIYLSAAQRCSFMVTTKKGETENFPIMASMDTSLFDSLPDDLNYNVTGWLV

YDDKKPLPDPQPVDSFDDLVFDDMTLVPYDDMERLGAPDRTVELTVRMKNLDDGANYAFF

NNITYKSPKVPTLYTALTSGDLATNPAVYGDYTHPFVLEKGEVVQITLNNQDNGRHPFHL

HGHHFQALYRAPKAGGDFNATENPDDSFPKTPMRRDTVVVWPRGNIVLRFRADNPGVWLF

HCHIEWHVTSGLIATFVEAPLELQKNITIPQGHIDVCKKGNYAVSGNAAANTVNLLDLSG

QPTPPAPLPAGFTPRGIVAIVFSGVAGVLGMIVVAWYGLSGAAIEAGKPAGGGVGLVSQH

EPDAAEEAASHEGSASDAAGAVKPVETSRATAQ

>SoG_02750.T1

MINHQDSTNGTFDSGHSASSGYSFDPEIPHGPNTDGVDLPYHTNAAGYSVPDTTYKDPQN

RRIRVATIGAGFSGILLAYRIQNELENVEHVIYEKNGEIGGAWLENRYPNCACDVPSHSY

VYPFAPNPEWPEFYSKSESIWAYLDRICRVFDLRKYMKFNHRVSEARWDEDAGLWHLKID

KVYKDGSIDQFDTTCDVLLQASGLLNNPILPKIDGLSDFKGRVIHTAQWPSDYGEKQWKG

QKVAVIGAGASAVQTVPGMQPHVDEAHVFIRSKTWLAAAGPEPLIETWSDEQREEFRLNP

EKLIAEARIHEVPVNLMWASMFKTSPLQKAAAQDAADKMRHYLERDDLTGGLIPSFPYGC

RRVSPGVKFMKAVTKPNIHCHFTPATRITSDSVIGADGTEVRCDAIVFATGFDTTFRPHY

KLVGQNGVSLAEKWKDVPEGYLGIGCPSFPNMVLFFAPAWPVFAGSVTASLTAVATFALK

LIRKIQTDDIRSIAPRQDVTDAFNLV

>SoG_02752.T1

MGFFETFTLALAASSVGLVAADGLNARAKVLGKYMGTEYNVGELSESTFMNIANNLEEFG

SAVPGNEQKWDATEPNRGQFSFSQGDRIANNVIQAGQLLRCHTLVRNGGFNNQTLISIMQ

NHIQNVVTHYKGKCSHWDVVNEALEENGSYRSSVFYNTIGEAFIPIAFRAAAAADPNVKL

FYNDYNIELAGAKSSGAQRIVRLIKSYGARIDAVGLQAHLVVGQVPSYTQYQSNLKAFTD

LGVDVAYTELDIRMDLPSSSQKLQQQATDYGNVVKACALTEKCLGVTMWGLSDAHSWVPG

TFPGTGDALPWSSSYQKKPAYSAILNAWGSGGGSGSSSSSTAQGTITSGPATTANTLTTR

AATTTSAGNGGQCAAMWGQCGGSGWQGPKCCAQGTCKYSNDWYSQCL

>SoG_02763.T1

MLRIYLRPTANLVAFSSLPATTQSRSKRHDAFGSHLDQDALTEARTWYRQFDPSQLPKGN

TAFARSSGPGGQHVNKTETKAITTFACRDILAALPRHLHPAVRQSKYYTAGSDSWTFQAQ

EYRSRTANADENRRKLTDEVMRIYHGLTPNETSSEKRQKHREIPLRRGTNAKQPDSVSQL

HSGHQPTQAMAQQQPQVQPCRYKVGKTLGAGSYSVVKECVHIDTGRYYAAKVINKRLMSG

REHMVRNEIAVLKKVSMGHQNILTLVDYFETMNNLYLVTDLALGGELFDRICRKGSYYES

DAADLIRATLSAVAYLHDHGIVHRDLKPENLLFRTPEDNADLLIADFGLSRIMEEEQYHV

LTTTCGTPGYMAPEIFKKTGHGKPVDLWATGVITYFLLCGYTPFDRDSDFEEMQAILSAD

YSFTPIEYWRGVSSHAKDFIRRCLTIDADTRITAHEALQHPFVTGVRESDGAGENLLPTI

KKNFNARRTLHAAIDTVRAINKLREAQNLMMDGAKSKEPEKGNPTKGPPSARKDDSAISM

NNGGGPGAQNWDSGYGTGSTDGGRDVTMGGTAGAPRQGAAPETSKGLWSPGTRR

>SoG_02765.T1

MRPSHFLSGLCLVAGATSRVGRDKLTEDWSVNIIESTMKRFTPQTIGAWEYFVSLYLMGQ

YVVYKRTGKESYMQYIQDWADRWFDEDGLFNVSISTLDSMQAGNIMLIVYEETGEEKYKD

AAEVIRGRFDEYPRTSDGGFWHGPQLHGQLWADGTFMANPFLARWGHIFGEADYADDETL

KQIIVYGDYLQAGNGLLIHAYDETRKAPWADQVTGLSEEQWCRAIGWYGFAIADLLLLVP

ENHPARGEVITKLQRFLAGVKGTQDQATGRWWEVADRPDDEENWTETSCSAMFTYAADVA

LKFGWVEDNDHEYGDMVKAGLAGVLDQVRKNDEGLTDVFEIVVGTNVGDLQHYFDRPRKT

NDLHGLGAVLLMNEQVVHNGHLTIGL

>SoG_02767.T1

MSGPVSSVSSSGILESCVSPHLRRASRRATHGTLTHCLLSVEDSARLANIKLGGARLLPS

TNSPITQYQNENSRLANIVPSADLGLIGLAVMGQNLIMNMADHGFTICAFNRTVSKVDRF

LENEAKGMSIVGAHSVEEFVSKLKSPRRVMLLVQAGQAVDDWIEKLIPLLSAGDIIIDGG

NSHFPDSNRRTQYLNSKGLRFVGSGVSGGEEGARYGPSLMPGGNEEAWPHIKDIFQSIAA

KSDNEPCCEWVGDEGAGHYVKMVHNGIEYGDMQLICEAYDIMKRALGLSNKEMGDVFAKW

NKGVLDSFLIEITRDIMYYNDEDGKPLVEKILDKAGQKGTGKWTAVNALDLGMPVTLIAE

SVLARCLSSIKDERSEASTKLEFVGRNTTFEGDKEQFLEDLEQALYASKIISYAQGFMLM

QEAAKEYKWKLNKPSIALMWRGGCIIRSVFLKDITNAYRKQPDLKNLLFDDFFNKAIHKA

QPGWRDVVAKAAMLGIPTPAFSTALSWFDGYRTKDLPANLLQAQRDYFGAHTFRIKPEHA

NAKYPEGQDIHVNWTGRGGNVSASTYSA

>SoG_02776.T1

MPSACPAPILAPSLIVVTGANGFIAQHCIAVLLSRNYKVIGTVRSQPKVEQVLACHSHNP

NLSVVVVHDITSTDAYLSVLRPLQPVAVLHLAAPFHYNTADYEQDLMIPAIKGSTAILDA

SKELGTVRRVVHTNSFAGIYDAAKGAQPEKTYTAADWSPLTYEDGVSAPSAAVAYRASKT

VAERAAWDWMKEHPLAGFDLVGLNPAMVFGPFLPGAIPSEPSEINTSNQIVYGVVSAGED

KELPPTRGPVWVSVQDVALAHLRALEVPEAGGGRYLLAAGVYCTQEIADMSRKVAPKHQS

SIPRGVPGRREADTHFAVDPRRTEEVLSIKWQSMEEMLSGLLPQLFDIQQKA

>SoG_02781.T1

MARSLPLVAAALLGLASAQTPDNTPEVHPSLTTWKCTKDGGCKAQDTKIVLDSLAHPVFQ

KNAPSFNCGDWGNAPNSTACPDAKTCQENCVMQGIPDLSQHGVVTTGDELYLDMLRDDGS

VISPRVYLLSPDEQTYEMLELTGNELSFDVDVSKLPCGMNGALYLSEMLPDGGKSDLNKA

GAYYGTGYCDAQCFTTPFINGEPNLEGYGSCCNELDIWEANARATHLAPHTCNQTAVYKC

AGAECKFDGVCDKNGCGYNPYAQGNPGFYGYEKIVDTTRPFTVVTQFPANAQGELVEYRR

FYIQDGKKLDNPPVRDANGTSTGKNWMDDPHCVATGAKRYMDLGATKGMGEAMARGMVLA

FSVWWSEGDEMKWLDQDKAGPCKPGDGAPSNIRKIQPDTAVTFSNIKWGEIDSTYKLRDK

CKRTHIQHRI

>SoG_02789.T1

MKSILVNLEHHSPSSPLFIYLPPLPPHPHETVPLPNVLQPWPIAIVRYRWSNRTSPDDSV

SSESEAGSLRWPTPVHDTGFAYSWLQDALAPSGVNRRDIYVLGTHLGASLAMSLGLTEAH

SHSKFGVRGVVAYNGIYNWTMFLPDHRINKAATGKRKKESVQPPPRSPEGSHLWNLQQRL

PDLFNTPAQMFDTFASPSLLFHGPGILIPKSFTLSTSEAFALDSLLDPESAPLPAKPPRR

SHLIFPPRRSTLKIPPTLLLHSTANLFTPKLSRRSTMKGNSFETQAEELAEMMRKSVDKV

ELKMRSRWDDEMDALEEEADRRVRVVDVGEEREGIEMGDEAERVLEEWLDQKMC

>SoG_02802.T1

MIAHDGKVTLLLTTDLDQRSGYRHDSIALSTIAPSETSRHWSNASTQPPPLFAGTSRTAQ

FLSSLEEPDAAAQLPAFVRPLSSKIAADDVAYLFAKGALTLPSVPLQNALLQAYVEYVHP

YMPLMDLHSFLSVINSRDGLNGQCSLFLYHAVMFAATAFVDMKHLREAGYSTRKAARKAF

FQKTRLLYDFDYELDRLVLVQALLLMTYWYETPDDQKDTWHWMGVAISLAHTIGLHRNPG

TTSMAPPRQKLWKRIWWSCFMRDRLIALGMRRPTRIKDEDFDVPQLEEDDFEIMALPDNI

TVIPPECALMRDVAMQRELAVMCIYKAKLCVTISHMLKAQYSVLIRDHMKPENTTNSTMM

LFPNKEMDNLDKINEVDMELLAWAESLPACCQYRTLTPMDVQNGKSTMAVQRTLLHMVYY

TTISALHRPQFLPSSPLRIPTASRSVQEMSRVRVRGAAMHITRMATELHHLRLERYLPTT

GVTVILPAMIIHLLEMKNPSVEARERATRGFRQCMRVMEKLREVYAAADYATGFLDAALK

KASINVNPNIAPSSLAILKSATNYSAQTPPPDNLPYMTASETLFNEKPSAGEARMPMMPP

ETINGAALDLSSGTAGNLNGTAGTNSPPHTDLGSAADLTPSASGGSDVGNGGVEQLDAMD

LDFMQGHDEFDWNAVAGTDFDVDQWLQFPPEGVNGGGEDGSAVAGAMRRDQDVVMATGQT

VGWDGSMATGGRRDAAPALA

>SoG_02806.T1

MMKWLIFLAGATLASAAERKNVTSPFPDFSGDPFSKYNISARGINASFVPYGARLTNMWV

PDRNGTQRDIVVGYDDPKDYTTQRNFFGAVVGRYANRIKNGTFEVDGHTYHTPLNEKGHD

TLHGGDVGYDMRNWTIINHTSDSITFFFYDAAYEGFPGDVVNMVTYTVTDEPAFYSRITS

IPTGKRTPIMIANHVYWNLGAFVSEEGLTILNDTLHMPAAKRYINIDGWEVPTGNISLTK

NTPLDFVSPKKIGKDIHNTTNGCGTGCLGYDNAFILDLPRSASPGDPSSEALVWSSPATG

IKMHLYTNQQSLQLYTCDTQDGSIKAKQDQQHINGNTTYDQFGCMVIETQDWIDGINQPQ

WGRDDYQLYDTKTVPFLNMQKYAFSTTT

>SoG_02809.T1

MKAFRFETVEKGLRLEDVPIPEPEADQVQIRVMATGICHSDCHVVDGHGAKFVTHTPITL

GHEVSGVITKLGSSVTDGLEIGARVAVVIPCYPVEIIDLSLGIGLGYDGGHAEYATAPAS

RVIKIPDDVSFAAAAVATDALAGSYHAVVTEAEAAPGKKIAIIGIGGLGMPGLRFGALKG

AEVYGIDIAESKFAEAMSLGATACFKSLAEAKHIRFDAVVDFYGSSETTAAAVKAVKRGG

KVVGVGLQSPVVSIPSVAAGYIAKTVTLVGSLGSSMDEILAVIELVRSGAYRPNLIEVPF

LEIPATLALLSKNAGAGRYWANPSKGRWPTTEKL

>SoG_02812.T1

MGFKTFGFAGGRADTWEADESTYWGGETTWLGNDVRYADGNPGSAASGVVDADEKHAHKD

IHNRKLEEPLGAAHMGLIYVNPEGPDGNPDPVAAAKDIRTTFGRMAMNDEETVALIAGGH

TFGKTHGAASSDNVGPEPEGAPIENQGFGWVNSHGSGAGPDAITSGLEVTWTKTPTKWSN

QFFEYLFKYEWELTKSPAGANQWVAKNAEPIIPHAFDSSKKQLPTMLTTDLSLRFDPEYE

KISRRFLENPDQFADAFARAWFKLLHRDLGPKARYLGPEIPKEDLIWQDPLPAVNHPLVN

ESDVAALKQAVLQAVPDVSKLVATAWASASTFRGSDKRGGANGARIRLAPQKDWKANNPQ

QLAQVLGALENVQKQFNAQGNGKQISLADLIVLAGNAAVEKAAQDAGHNVSVPFTPGRTD

ATQEQTDVESIGHLEPFADGFRNYGKSTSRVKAEQYLVDKANLLTLTAPEMTVLVGGLRA

LNANYDGSSHGVFTTRPGVLTNDFFVNLLDMSTQWKSAGSDDVFEGVDRKTGAKRWTATR

ADLVFGSQAELRAIAEVYGSADGAEKFANDFAAAWVKVMNLDRFDL

>SoG_02816.T1

MSYGKKDEDADLGLVKVDRTQVFQEANRADAARLFNSSPIQPRRCRILLTKIALLLYTGE

SFPTNEATTLFFGISKLFQNKDASLRQMVHLVIKELANSAEDIIMVTSTIMKDTGGGTDA

IFRPNAIRALCRIIDATTVQSIERVMKTAIVDKNPSVSSAALVSSYHLLPIARDVVRRWQ

SETQEAAASTKSSGGFSLGFSSSGGQLPMNNSTMTQYHAVGLLYQMRSHDRMALVKMVQQ

FGAAGAVKSPAALVMLVRLAAQLAEEDASLRKPMMQLLDGWLRHKSEMVNFEAAKAICDM

RDVTDAEVTNAVHVLQLFLTSPRAVTKFAALRILHNFATFKPNAVNVCNPDIELLISNSN

RSIATFAITTLLKTGNEASVDRLMKQISGFMSEITDEFKITIVEAIRTLCLKFPSKQAGM

LTFLSGILRDEGGYEFKRAVVESMFDLIKFVPDSKEEALSHLCEFIEDCEFTKLAVRILH

LIGLEGPKTAQPTKYIRYIYNRVVLENAIVRAAAVTALAKFGVGQKDPDVKSSVKVLLTR

CLDDVDDEVRDRAALNLKLMGEEDDEMAQNFVKNENMFSLPYFEHQLVMYVTSDDKSTWA

SPFDISKIPVVTREQADAEDRTKKLTATTPTLKAPKVGPTKAAPSSGAEAAASATAQAQR

YAQELREIPEMAEFGSVLKSSPVVELTEAETEYVVTLVKHIFKEHIVLQYEVKNTLPDTV

LENVSIMATPADEEDLEEVFIIQAEKLATDEPGKVYVAFKKINGEGSLPITTFSNVLKFT

SKEIDPSTGEPEESGYDDEYEVSEFDLSGSDYIIPTFASNFSDLWEKIGAAGEQAEETLQ

LGSMKSIAGEKDADAPPQQPQVVSYDRGDMLTQSHRGDGAAGTGAITAAAGRHGRAGQHK

HAHTQAAGQERVRGQGGCNGADGVLVQVWRDDEDHGAERRGERGGLGGCVSSIKEEVKTA

GSIAGVDGYKVPCECCVWKPGGLCGSLGENKDYDKFLIR

>SoG_02828.T1

MFNLRRLFVSAALLLGLSTLLFAQVAEAAKGPKITHKVYFDIQHGDQQLGRIVMGLYGKT

VPETAENFRALATGEKGFGYEGSTFHRVIKNFMIQGGDFTKGDGTGGKSIYGNKFKDENF

KLKHSKKGMLSMANAGPDTNGSQFFITTVITSWLDGRHVVFGEVLEGYDIVEKIENIKTA

PGDKPVETVKIVKSGELEVPPEDLYADAEWVKVDGEWVRVHIADEVGGSPLDGQVGHADM

GGMSTMQNFMIIGLVCAVVAIYVRSRMRADAEAAMLEKSRV

>SoG_02836.T1

MRPLRVVVIGAGISGILAAIRFPQRIPNLSLAIYEKNADVSGTWLENRYPGCACDIPAHT

YQATFAPNHEWSQFYASAPEIHAYWKKIAHRYGCMKYIKLQQQVVSAIWDEDQAKWLLKV

RDLTNNTEYSDECNVLISATGALNSWKWPEIPGLHDFQGKLLHSAAWDESYDYSGKRVAV

IGNGSSGIQIVPAVLPKVAHLDHYIRGKAWLSPTFARKLVDERGGGVDNFTFTEEEKAEF

KADKNKYQAFRKAIELELQSVHGTTVRGTPEQLAAPGFFAESMRKRLHKNPALQEELIPS

FPPGCRRLTPGPGYLEALTDDRVSTIREGIARIDATGITTDDGVHHPVDAIMCATGFDTS

FLPRFPVTGRNGRSLAEKWQSFPSTYLSMSTDDFPNYFISLGPNSALGSGNLLFVIEKAM

DYFTDCVLKMQRDNISSMAPRKQAVDGFYEYCKGYFDRTVFTYKCRSWYKGGSFDGPISA

LWPGNGSTDKEVPRFAGSSLHAKEVFAYPRWEDFEYRYVDNRPNGWIGDGWTQNEKDNLI

NVDYLDDEEIDFPPILQANGHA

>SoG_02837.T1

MPPRWLGKVESRAKRLLLAAPQSRSTAPERMPSSTALQPANEPATEAPSRLTSSCSTSVD

QEPTLVLSLQERLWNDAYDNLKKSEPKLVDAYEKILSIQLRRKDPSSVACESSQNEIGDT

RETRNHQMQQLVRSGLDSTQNAASIKKGIDEGLQAVQAVRGLVDKAMQAVPQGAVAWVGV

CVGLEIISNPVTEARYNRTGVAYVLSRMEWYWNLASLLLDENKAEQSSAGLRIQLEKHMV

QLYEKLLSYQIKSVCLYHRNWAAVVLRDVFRRDDWNGQLDEIKAAEVNVRTDSNDYNNEQ

SKSHLQKLGEMANSLQMNLQDIHSAIKDQTLHQEKRYQDDKYEMCMRDLYVTDPRKDKKR

IQDTKGGLLRDSYCWILDHVEFRQFCDNPKSRLLWIKGDPGKGKTMLLCGIIDELETSLH

SPLSYFFCQATEAHLSNATSVLRGLIYLLILQKPSLISHVRSKYDVVGEKLFQGINVWVY

LVEILNDILNDPTMRDAVLLIDALDECLIGRDQILDLIVQSSSTSSSRVKWIVSSRNWPE

IEEKLDGTRDKVRLHLELNQDAISSAVDKYIEYKVARLACDKKYDTDTKEAVVKYLASNA

KGTFLWVALVCQRLALPRARARNALATVKEFPPGLEALYKRMMEYIHDLERRDFDHCMKI

LATASVVYRPVNFDELRVLVEPANGLSDDVLEETIALCGSFLTVRQRIVYLVHQSAKDFL

LKHAPEQIFPSGIQGQHHDLFIRSIEALTETLRRDIYNLCAPGTRIDQVSSPDPDPLVSI

KYSCVFWIDHLHGSDPWVRISDHDSQDAELLCYFFQRKYLYWLEALSLLRKIPEGVMAVQ

KLDASVKPTTARQLRELIHDARRFIRHHKHVVELAPLQVYASALVFSPVRSLIRELFKDE

EPDWIKLKPKVESDWNACIQTLEDHDIAVTSVAFSADEQRLASSSHDKTIKIWDVMTGTC

IQTLEGHTDSVTSVGFYMTSRYLVSGSIDTTVKLWDTAVGCTKTLKGHSDCVASVAISTS

GQHLASSSYDTTVKIWDPALGTCVQTLEGHENAVVSVAFSINSHVASGSGDGTIKIWDWK

AGACLRTLRDHATSVISVAFSPDGECLASDSDDATIMIWDVATGNCAKTFPHGKSRAASV

VFSTNGRYLASGLYDGTVETWDATTSRRVQTLKGHGSRVSAAVFSGDGQHLASSSYDGTI

KIWELSAGAGAESLEVHSDVISSLVLSADGQCLASGSYDTTVKLWDPETGMNTRTLRGHQ

LFVSSVAFSSDGLRLLSSSKDGTAKLWNIATGTCIKTLDVDSGGLTSSVFSSDDRYIASS

AHSGVIKILDAATGTRLKTLEGHNVAAIKVLFSPDNQYLASSPYDDNVKIWDVATGKCKY

TMKTRMLNSWWGSAAFSADGQRLASSWEDGTVEVWDTSTGICIQILDVGRLLLDLHFDAT

ARSFFSTEIGYLNLDHLDPPPDASDVQVSQRSRSNAPHARWYLSDDGSWIVKDGKEMLWL

PSDYRTRERAVSVKGSTVAIGCTNSRQVLVMKFS

>SoG_02839.T1

MANLKRVHFFPVFFASAFIRWSFGKPTFGSVLEPIGNAILTIGSGEGLVEGTLGALKGLV

GGQQSFDYVVVGGGTGGNAIGVRLAEAGFSVAIIEAGTFYELSKPVLATTPGGAFFGAGT

DPRVNIPTVDWGFITEPQAGLDGRRLHFSQGKCLAGSSALNFMAHHRASTGTYDKLENWL

PFFKKSVTFTGPNMEIRRQNATPTWDPDAFAPEGEGGPVQVTYSNWAWSFSTWLEKGLAA

VGLKKTDGFDNGQLLGYHWAQATIRASDQTRSSSVSYVYSAMSSNEAKKNLKVFTQTLGK

QILFDGKKATGVKVSLLGAFPDYTIKANKEVIVSSGAFRSPQLLMVSGIGPKDTLDAHGI

PVISHLEGVGQNMWDHVMFGPSYEVTVPTLDSTLLNPVNLASSLSDYTLKAQGPLASNVI

GFLGWEKLPEKYRSTWSESTREAESWFPDDWPELEHLSSSAWVGDFGFPLLQQPRDGKDY

ATDLGALVAPLSRGNVTIKSADTAQLPSINPNWLTHPADKELAVSIYRRLREVWDTPEMR

SIRADGDNEAYPGRQYDSDEQILDVVKKSMLTVWHPSCTCKMGKPDDDMAVVDSRARVFG

VEGLRVVDASAFALLPEGHPQATIYALAEKIAHDIISGQSESGCPIKSLFVFAVHSFSIT

RDFGGGGVDALMWFPFLKSRNTKAPHGKPDRRIHQIPLQPPPGSWLLRTLXSPQSIMSSS

ASATVGVGADDDPIVLPDSPPIGRSTITFMLNDPLAPDQRVSRTERLRQRRVMPPQVNTQ

PPAPAVIDLTEEPDSPVEQRPPAILASSSSSSSRTLPWPDAGFASNRHRRPDGSRHPRRT

NSTRISPPMLTRSDSTLMGGRHSIIDLTQDAPEESHPLQLPRISRQTSGGPPRFPLFNRH

AHHHHHHHHPNLHSSRPPHPTTTDEMVEVVTTYGSVGEFVQNSLRHYHGFITGGFSGLAR

AFNQPQLDVTRNAFAPRQDTPQPAMEPVPPARAGFTRDTHVENDKGDEEIIAVCPACDEE

LAYDPDDVSAQKSSNAATTAAGKKRKRAAGEHHFWALKKCGHVYCADCFENRKPSKANPR

GVGFLLPSNKTGNQAFQEMRCAVEGCETKVAQKGEWIGIFL

>SoG_02856.T1

MAILTLRDGTEIFYKDWGNKSGPVVTLSHGWPLNCDDWDKQMIFLGEHGYRVIAHDRRGH

GRSSQPWDGNDFDTYADDLEQLFEHLGVKDITMVGHSTGGGEITRYFGKYPKRVSKIVLV

SAVTPYLVQNEGNPDGVPVSVFDGFRAGLKKDKAGFLWTIPDGPFFGFNRPNANRSTQIV

QSWWNAGMMCGIKNIYDCLKTFSETDLSEDLKKMNIPVLILHGDDDQIVPIHVAAQRAIK

LVPNGTLKVYPGASHALPVMNADQVNKDILEFLKQ

>SoG_02860.T1

MNGLERLPSSSRSPGKEASQAENAAGLPTTSVDSKSGPRPGPLWLVTLGVVIVSISKTEL

PLIELYLLTPDHFNDLANIDATALNHSTCLRARLLSGRMPWLRLPRKSPEHDSGNDSHRF

VLDSVFARLPPFETCPLTVPNNWAVTRLQRLGPQREETQRIVAVHNSGGFNPLPLPCPQG

IGLSQLASWRVATSIGTFSLLSTVNPLSYSSSFRALSYIVASLLAIAQIIGMLPKTTATC

KTFLWAFALIPTTSYLATEFSVSRAQQLAQRFENSSQPHPVETLTADAKEAFDGLTSRQS

RTFDAASAEYRHRYGMDPPPGFRGWYDFAVSNQSPAIDDFDTIYDSVSPFWKISGLQVSE

MMREAQRAPNSELWLCSFSAGSAQTSCEHPHRTFDRKIGLEFSRLMRNASLDLPDFSFLV

NHLDEPAVVIPPTDSSLSMNEALGRLNLTHMSLRPVGKEITKLCPARQLPDESAEQNGVH

TFGLPFVVNQSSAMDLCQHGGYSNLHGFFMSPNSFRLIEGLVPVLSTGAPLGMKDILFPS

PAYSESEFEYDESHDVEWDQKRNNLYWAGSTTGGFANSHSWRNFHRQRFVDLVQNPGRKQ

QYHYLDPGAGAVASKVSSFLNLRQYDVAFTKVVGCDRRHCRDQRTYFNIKSWADKDEALR

SRLVFDVDGNGISGRFYKLLASRSTPLKMTIFREWHDDRLVPWYHYIPISLGMEELPELV

MHLTSTESGERTARNIAEQGRQWHSEALRDVDKAIYLYRLLLELARLQDPERKAT

>SoG_02861.T1

MVAFTNIALGLGAAAALSLAAPAPAPAPAKSPLEERGPMNFVMGYDHPLSKRFGNFSVRA

IGPRSDTNYKQDYRTGGSVNFSPGTNSFSLNWNTQQDFVVGVGWNPGGHSPITHSGTFSV

NSGLGSLGVYGWTTNPLVEYYIMDTNVGINTGGSQRGTVTSDGATYAIWEHQQVNQPSIQ

GTSTFNQYISIRQSPRSSGTITVDNHFKAWAALGMNLGTMNYQVIAVESWSGSGSAQQSV

SNTGSGDSGGGGGNTGGGNTGGGNGGGNNGGGGGGNNGGGGGNNGGGGGSGGGSCSAIWG

QCGGNGWTGPKCCSSGSCHFQNDWYSQCM

>SoG_02886.T1

MAPPPRHPRRLDFLVILPLALLATYLCYTLLALRTEVCLHGGPNCHRGFRSAYSFDAVAS

HRPDWMKSIPDETNLTSLSIPGTHDTMTYEIENQRLECQNWNLSVQMEAGIRYFDIRARL

KNDELRIYHTNGDTGFSYEDVLLSMCEFLEENPSETIVMRLKEEGVPLGDNTLTFEDALN

TYRESRTSTKTCVSNHLYLNDSTSSTKEPPKIPTLGELRSKIFLLQNFKAHSGKSYGLSW

DGPQMKLEDFWIMPDASFLKQKWESISKALKLANETPLNNEVIFLAHISASVGVLPIEAA

AGTADGKMTGMNDMTGDWLDDHVKEAIRAGIVIFDFPGKKAIDAVLSWNPK

>SoG_02888.T1

MLNNGLATPLFLAIGFAAALADAGSVSKRDIRDEYPSRPYYPAPFGGWLDEWTESYAKAK

ALVDSMTLAEKTNITSGTGIFMANISSRRCNGNTGSALRVGFPQLCLNDAANGVRQADNV

TVFPDGITVGATFDKKLMYERAVAIGKEARGKGVNIWLAPAVGPMGRKPKGGRNWEGFGA

DPVLQAVGARETIKGIQEQGVIATIKHLVGNEQEMYRMYNPFQYGYSANIDDRTMHELYL

WPFAEGVRAGVGAVMSAYNAVNGTACSQNPYLVNGLLKDELGFQGLVMTDWLAKMSGVAS

ALAGTDLDMPGDTQIPLFGNSYWMYELSRSALNGSVPMERINDMATRVVAAWYQMGQDSE

SYPRPNFDTNSYDREGPLYAGAWPASPRGIINEFVQVQADHDVIARQVAQEAITMLKNND

GLLPLSTSRSIKVFGTAAQVNPDGPNACADRNCNKGTLGQGWGSGTVDYEYLDDPIGALR

SRASDVTFYNTDKYPSNAVVGDDDVAIVFITSDSGENSYTVEGNHGDRDASRLFAWHNGD

KLVKDVANKYKNVIVVAQTVGPLIFEEWHDIPSVKTILIAHLPGQEAGDSLANVLYGDAS

PCGHLPYSITYKEDDMPESVTKLIDFALFNQPQDTYSEGLYIDYRWLNKAGIKPRYAFGH

GLSYTNFSYSDAKIEKVTQLDTVPPARKPKGDVLNYSQDVPDFKEAVQPDGFSKVWRYIY

SWLSERDAKNAAADRESKKYPYPQGYNEDQKPGPRAGGGQGGNPALWDVAYKLSVKVTNT

GKKHSGKASVQAYLQFPKGISYDTPVIQLRDFEKTSTLAPGESETVELTLTRKDVSVWDV

VIQDWVVPDVDGAYKIWIGAASDNLGTVCNVDGLKCESGVSGPI

>SoG_02890.T1

MRLDLSAALAASSALLFGRGSAQTFTECNPLHTQCPPDAALGMTINVDFRKGAVNSFAPF

GSPTYSNQNGASFTVRQGGDAPQLQSVFYIMFGRVEITMKAAPGAGIVSSLVLESDCLDE

IDIEWLGSNPDEMQSNYFGKGQTTSYNRGQFHKVTDTQGKWITYTVDWTQDRIVWMADGT

VLRELKASEAETNQYPQTPMKVKFGAWAGGDPNYNPPGTVAWARGPTDYSKGPFSMLVQK

IIVTDYSTGKEYKYGDQSGKWQSIQAIDGKINGNVGNSNAVTVTASAAGTDSTGVAGVPQ

GGLGDDGSDATKTQTGWPWQPTRGSQSGSIPDGWIMTNEGKLIPKGSSSTLRPTLTNAVL

TPLALILLRRLV

>SoG_02895.T1

MLNRLHGQPESYDKKSKYKFGRTLGAGTYGTVREADSPFGKVAVKIILKRNVKGNEKMVY

DELEMLQRLKHEHIVKFIDWFESRDKFYIVTQLATGGELFDRICEQGRFTEKDASQCIRQ

VLSAVDYLHRNGVVHRDLKPENLLYVTTEPNSDLVLADFGIAKTLDSKEDSLQTMAGSFG

YAAPEVMDRQGHGKPVDMWSLGVITYTLLCGYSPFRSENLKDLLIECTQNTVVFHERYWR

DVSEDAKDFILRLIVPDQHKRWTSEQAMHHRWLSGETATDHDLLPDLRSYRARQKLRRAI

EVVKLRNRIKALKEKDEDPENSDMADLDDEKEGGDGARLHALGLFALKDAKAKQESLQVE

ENLAKEAKRRSGQFSG

>SoG_02910.T1

MFRQSLRRCASRVATASMVPRATPILRRTVAPSVARPVFSSRLLQLASRNYSTESEAPVS

TPQEAVEPSETFDSLKELGVHPNILRAITDDMGYDVMTPVQSKTIRPSLDGTDIVAQAKT

GTGKTLAFLLPLLQRMINEDPRLASRSAIGTARSTDIRGIILSPTRELAEQIAQEARRVT

RHTGLVVQCAVGGTRKADMLRQTQRQGCHLLVATPGRLNDILSDPRSGVSAPNLAALVLD

EADRMLDVGFEAEIASITSQLPPTSEKVRQTMLVSATIPDNVIRLARNMVRPDDFQFVQT

IAQNESLTHDRVPQNVVAMNAFGNMFPTLFEIIDRGVRESQQPGARPFKAVVYFNTTALV

QLAGVLGQERRRNKTLEIPTISINSKLTQAQREKSASLFKRVQTGVLFSSDITARGMDFP

DVTHVIQMDLPGDRETYIHRLGRTARQGKEGTGYLVLPPHNVRWARKMLEGLPLTPDNTL

ECATARTDDETNAIITEVREMGKIVYEDILETAYKSIFGGFKGSDKHALVDDLNDWMVNG

FGLAQPPVVGRTWAQKMGLSRSNLNIGGEAYNGVGSANFGGSSARFDRRGGGGSSSSGDP

FASQFSDRIGDDRRGGARFGDQRRSGSSRGDYRGGSRGSYGGGSRGGSRGGFRGGYGDGE

GSSW

>SoG_02914.T1

MQGPQPKTGFDVDHVLSQLKQNEKIALLSGIDFWHSSPIPEHNVPSIRVTDGPNGIRGTK

FFAGVPAACLPCGTALAATFDKKLMRRAGELLGDECLAKGAHVWLGPTMNMQRSPLGGRG

FESFSEDPYLSGAIGTAMVQGCESTGVIATPKHFVGNDQEHERRAVDCIVTPRALREIYL

RPFQLVARDAKPGALMTSYNKINGKHVAENPALLQDLIRGEWKWDPLIISDWLGTYSVVG

AMEAGMDLEMPGPTRYRGKYIDSAVQARLLKQSTIDARARRVLAFAKRCSEVKVSEEEVG

RDYPEDRALNREICANSIVLLKNDEQVLPLPKTVKKIALIGSHVRVPAISGGGSAALLPY

YAVSLYDAVKEVLPDATIVHETGCYAHSMMPAVSTQLQGGALVHFYNEPPTDPRRKILGE

ENIKSASFQLMDYNNAPGLNKALFWGTLYADFVPTATGTWEFGLTVFGTANLYINDELII

NNTTDQRKGTAFFGKGTVEEVGTVEMEEGKTYKLRIEFGSANTTTMETIGMVNFGGGAVH

LGAILKMDPEQMVLQAIKAAKEADCTIICAGLSGEWESEGFDRPHMDLPDGVDDMISRVL

EAAPNAVVVNQSGTPVTMPWADKAKAIVHAWYGGNETGHGMTDVIFGDVSPSAKLSLSWP

FDVKHNPAYLNYESVNGRVLYGEDIYVGYKFYDKTERELRFPFGHGLSYTTFDLGNNAQV

DVEPKIFTPENPSVAIVRVKNTGEVAGAQVLQAYIAAPHSPTGRPLKELFGFEKVFLQPG

EEKEVRIPIDQYATSFWCEIESMWKSEAGSYDILIASSSKEILGKGEMIVPKTRYWIGL

>SoG_02918.T1

MLLGTASALPSRAQTMARRQVSELRDEYDFVIVGGGTTGLTVAYRLSAALPKSEPLFSFP

FLSSPPPSSRCTEDQHLRPETVLVIEYGQVEDTVGYFDPPEDGRGAGRLVINSPPVASVN

NRTATVILGMTVGGGSAVNGQFLDRGSRHDYDEWARLWSPEFDLDLDDVDDDRESSWDWG

NFGPAFEKSLFLTEPSQELVDEYGYTWDSSHYKGDSIEASFPPFQWPAQRIGWQSLKEFG

LSTPRSCDGGDKHGVCWIPTAQNSQTVERSHAGVGHYTRVAGSLPNLDLLVGHKVVRLVV

SRDSDDDDGDDKKQLSVPAVEFRPVSGGKVQTIHPRHEVILSAGAIHTPQILQRSGVASA

AYLESEGIAVVEDLPGVGQNFQDHCAVPMVFSYDSPSPEEADMARNETFAAESVAQFRER

PARGPYTLGMGNSAAYVALREVTPRWEGIVAGIRRQAEDLSGLRHLPPTAGKTVQQGYLA

QLEILAQALEHPEQAILEMPFTAAPGVAFLLKPLSRGSVVLNSSDHDGTPTVRYGTGSNP

IDLDIMAGYVEYVRKLFARTEAWRSRGAVEVSPGAEVTEHEALVEFVKDNVIQSLMHPCC

TAAMLPRDKGGVVDASLSVYGVPGLRVADCSVIPTIPGSHTTTTAYAIGEKEVEEEQ

>SoG_02924.T1

MSSIRDVDGVVNGINGINGINGTNGMNITHDAHIRNGINGNHNAHLSNGCREANSTNGAN

GHSHTNSTTYSSGMRDAATASHRTIPIAVCGIGLRLPGGSSTPEQFWEFLANKGDARVRV

PESRYNISAYHETSKRPTTIASEYGYFLDDSVKLGAMDTSRFSMGRVELEYADPQQRRLL

EVVTEAFEDAGEVNFRGRKIGCYVGNMNEDWGEMMNRDPLWHGPNKIDGYQDWILPNRIS

YEFGLKGPRYVYDHGLRESVEDLLTPYVSKSMIIRTACSSALVGVNEACSAMQRGLCESA

IVAGANLMLAPGMTQQMTEKGILSPEGSCKTFSADADGYARGEAFTAVFLKPLDAAVRDG

NPVRAVIRAAVANSDGKTQGITQPNAAAHEEMIRLAYKQADISDFSKTAYFECHGTGTPV

GDPIETGAVANIFGDHGIYITSVKPNVGHTEGASGLVSLIKAVLSLQHREIPPNIKFNVP

NPKIPFKERKLAVPVELTPFPEDRLERVSVNSFGLGGSNAHVIVDSARSFAIPEETVTDE

ESLEGPHLLLFSAASTPSLKSMIQSYEEWLTSRPGTGSQVHDLAYTLANRREHLTYRSFK

VVGTGDLPASQGRRIPNQPVNLVMVFTGQGAQWPRMGRDLLLREDLPFGDTIRALDRYIH

EISNAPEWTIEAELSRSAKTSNLHRAELSQPLCTAVQIGILDMLAKIGVKPAAVVGHSSG

ELAAAYAAGALTAQEAIIGAYQRGQAAKLQSRKGAMAAVGLGREEVEPYLSPPQVVIACE

NSPKSVTLSGDESAVQSAVARIKDDMPDITARLLKVEKAYHSYHMREVGQDYNAMIQGHL

AGRRPVVPFFSSVTGTGKPEQRVLDAKYWQQNLESPVLFSQAMAGVLEQVANPAFMEIGP

HGALAGPARQIFTKASVSPPYLSAMNRNEDCVESYLTAVGKLFELNVPLYYSALAPSGKV

LPDLPRYPWNYEGEFWAESRMSAEWRNPRFPRHPLLGRRALESTSLEPGWRNMLNVEDAP

WLRDHVIQNAIIFPAAAYLAMAGEAVRQTTGIEGSYSIRHMVLSQAMMLQEGVETEVVTN

LRPHRPHDNQDGSDQWWEFSIASYNGNMWTGHCSGQVSAVSPTKPAQPEPTEHLPRRLDS

VKVFSTLAKAGMQYGPAFQRLEEIGSATLDRRAVSKLVGAANGDEKSYHLHPTIIDAALQ

LGLIASVYGKLDEHNCAAMPTLIEDATIYRAEPGTTLTASAVTEILSGGEAIGGRFQVLE

GREAVLSIRSASFKSLVVERNDTVNHLPTTGRVWWSPHIDFIPTNTLIIPQLDGEKCMPL

LNELGELCYIFFQRRIGELTDLSEKPPVQKLIRWVGRQYQALAQDHPAHAWDLEAIVDRA

HEIGELLSGTQLAACADSLLEVGGSIDELLTGQVELVGLLQKDDLLTRLFASANTFTYSP

FIKALAHSKPSLRVLDIGASSSQMITPIMKDLVLPHGACLYSKYTFADSDPHAIVSAKKR

YEHLSHMSYRTLDIGKDLNEQGFNDDDKYDLIIARNTLHATESLSVALSNVRKLLAPGGR

LLLQELDTQAKWINCILGFLDDWWLGENDGRIDEPYVGPERWEKELTSAGFRTSGFVLDS

AAPHQLCALTIATIEEPASPFRGNSVTVLAHEHDGNIVGVVSQELERRGYNVSTRRLGDD

PPDESQDVISLLDEPGPFFEGLNESRFKNLQRHVANSGSSGIFWVTRMSQLQVRDPRYAA

IIGASRSIRHENTMDFATCEVEDLSASLPAVIDSFVHFHSRQEDEYFRPDYEYAIVDDTI

NVPRVYPFTFEDEWNSNHPAQERFVLASQKNSAALRWSSPDSNSLVGNQVEIEVFATTLS

RRKDVASTFGAATHSSEGFTSGCSGRIVGIGPEVKSLAVGTRVMCLIQGPFSSHVVAREE

LVEEIPSYLSFVEAATLPAIFVPALIALREVNTLEHGQPVELTEKPQSLLILNAANDVGL

AALQLARESAVDIHATVKSEEESELLVNIFNISKNCIFISANGSFQHEITTLTEGRGVDL

VLDARSGEPFSAIWNSASEFGKVIDLGEPEGSHFVNLDLRSLSGARSYTRIDIGAWISRR

PSTIKRLLKSTVNLAGGGAIAPRVVFELSQLDEAIKLVQEDRNYTTTVIQLRNDEASLLN

RDVPIKAADKSLVVDSSASYLLAGGLGGIGTVIARLLAENGAKRIVCLSRDPGSRPEERN

AIKELESLGAEVVLVKGNMINREDVFNAVRHAPNLKGILHAPMFLQDEAFNNMTVDQWIN

VMGPKAKGAWYLHEATEEAGIQLDFFVFLSSMSGLTGQPGQANYSGANTFLDAFVLWRNG

MGLPASSINIGPVADMGYAVRNPQMLQRLLKTGYSGVTESGMLDAFRTAASYEVPELEFD

TKIAPYTSRNTFAIGFSSDKSHRHPEARAHWRKDIRTGIWHNISDAGSNDASAGGNTFKA

FMATAKSDPEVLSNPETANYIAVEIGKQLMSLLLRSEEDLDITLPVQQLGLDSLVGIELR

NWWRQTFGFDISVLQLLGFGTLEELGKHAVKVLAEG

>SoG_02943.T1

MPAQLAEEWLQFEANIGARPLPPGKTIQEIRANIEKMSSGPPPVPKSSSTLDVREDIVGD

RSVPIRVYAPKAGTESLPVGILVADQEAFLTHQAQRKYIKSPADVSRGIAIIGVSAGAQL

AISTTAQLVSEGLPVRSVAALAPFAVPPASVSQGLKDSGKYASYTENSDGPLVSPEALNM

FLDANGADPGDSSFSILFNPYLSKFPPTYIVTCGADILRDDGRLIVETLKRNSIEVKHDE

YPGYPHVFWAIPGLKILEKFQANLTAGLRHVL

>SoG_02945.T1

MASFKGKVIAITGGASGIGFAIAELLAKRGAMLSLADAVEGKLTQVCDKLEALGAQATVH

CVDVQDPDQIEAWMKATIAAFGRLDGVANMAGVSGRKEYPQGVIDQDQRDWEFVLGVNLT

GTMNCLRSQLKYISNGGSIVNASSIMGLQGAASTSAYTSSKHGVIGLTKCVAQEAGPRSI

RINAIAPGYINTPMFTSALGEKADDLKRTVPLGRIGEPEDVAFLIAFLLSDESKYITATV

ISVDGGLHG

>SoG_02951.T1

MKSFLTLLVLAKLASAAILWDGRFNDMSSSSDLEKWSWGNQVGPYQWYIHGSGPVTEYVN

LSPGYKNPADGGSSKGAKISLTSSAYWNGQNMRRTELIPQTSAAIASGKVFYHFSIKRES

ENAPSTNREHQIAFFESHFTELKSGWISGAPGTSDPELKWMVGGQTKWAVNWDAGVWHNV

AYEIDFSARTVGFWHSTGGDPLVQTVAPVSASTSSNGADWHVGVLELPRSGYPDSNEDFY

FSGVYIESGSITTSVSGPGGSSGGGGGSSSSSTSRLSSSSAATTLSTSITSRGSTTVPRT

TTTSAPQPPSTTSTKPTTTTTTQPPAAGGAEHWAQCGGSNWVGPTACVAGYTCSSINPYY

HQCL

>SoG_02954.T1

MAAGCGSPWLAIPTVMALVYIVSRLARRNYSSQLGVSGVGDDFEGSRSFDWRSEEPRKLR

PFKDIYHITMAIQADQVSNLITIDRDYHCRIEHRKQILIHKGRGVHGCLPNGEEAVRELY

GFLMKDFLPKRFPTIFSLASGGKSTHNAVTAMDYPSDWTDSDMNGALRVLAENVEEDLFL

LRETEDGHFLDAFVCCFPSGFDPSHKLGKLLKDIHGPVPSYEKIGSSMERFFKRLEVGKN

VKRMNWSVQTHEALTAITGNHITEEDQSLFQKQDFKPEDHAKISTGKMIAVVILPLVALV

AQVHGHGYVATFTVNGVDFPGFRRLDAPPIPNSVGWSFSTPDEGAVLDATSPDMICRHDG

KNAPGSAPIAAGQEVDFSWTSADLERNPDGWAKGHRGPVITYLAACNGPCDKADKTTLRW

NKIAEAGLISGPANREGVWATDLLRQGNNSAMIPTSIAPGNYVIRNEIIALHIPQKPEFY

MACASIEVTKGGNDDLSDKGVLATELYKPVNKQLYGFSIYESTDSSWPIPGPPLYKAANR

GGSTKPDSVDSAPSSTGAAASPTATDHLRYAPCGAA

>SoG_02959.T1

MQSSRAKGDAHGRKAKLANSYQELLEMFRADLVLPTMSSEFASKDLKSVGNYTLGRLIGK

GSFGKVYLASHKLTNGSKVCLNITLRSTYLQYGGTDWAAQVVLKSANKDDSNLAREIHHH

RQFVHPHIARLYEVIVTETLVWLVLEYCPGDELYNHLLEHGPLPVHKVQKIFTQLVGAVS

YVHMQSCVHRDLKLENILLDRHENVKLVDFGFTREYEGRANHLQTFCGTICYSAPEMLKG

EKYAGEKVDVWSLGIILYALLCGELPFDDDDDNVTRTRIISEEPTYPDHLAPDAVSLIKS

LLSKRPFPRPSLPDILSHPFLAEHAVAQQAILNKGVTPPFGTALETDCLQRMRSAGVDID

AVIESVLAQKVDALAGWWTLLLEKERRKMQRRERKRRERDSESKSLRRLSAASSRLERMG

PMLQDVDEDGGLTSQFIRLGEMQTSRTRGRSERRSAHYYGDLGIPDLPQVSEVGSGQNTP

DDIPPTPVDKDSIRSVSTSRHRRPIPPPKEGVIRSARSRGSTLHLVTTSEALGGTSSNSS

QANSQPKARKKPSQAIIAHWKNWTHWIFENTTRRKKGHERRTSRSVPDLHNKDGNGIDKD

GKVSPRPQTSKYPTTGSPGAPPTAALPKGVVANGHVTRGQATPSMQGARGTRIPSGQPPG

PPRISTAYKRQSLSPTPLTPRSTMRRSSAGLRGRKSTSSSLSSVRSIHHHHHSHSKASST

SSTGSVSTTKTPLHRGHSPHHSSVKVLPATPSTSSPFPSNIRLVRASPAPLALWNEGMPG

SDGAPPGSPNPFASGVQFAKRKKNLFKGPTLNFGTSGGARAANSSSHSRNTSASGLGRRS

GEITIQEEDEDYVGEDAEEIEEVEAFNPIVRRPGEIVEEEIIEDGEATPTRLRAVDPVPQ

DHDKRRDDGRIEEQEQPKEEQLKSGQAISDDNALPRDQPASVEARA

>SoG_02968.T1

MPPVDNPSGDIDDQPTVVFFHPDLGIGGAERLVVDAAVGLKSRGYRVVIFTNHCDPSHCF

DECRDGTLDVRVRGSWLVPPSILSRLTILCAILRHLHLLLSIYFSSELSNLRPAAFFVDQ

LSAGLPLLQYLNPNIPILFYCHFPDLLLARGRETSALKRAYRIPFDWLEEWSMGFSQAIA

VNSEFTKRVVEKTWPGLTGRVSTKVIYPCVDTDDAQTSDMPLPDELKNQKIILSINRFER

KKDVGLAIKAFAALPEGKRKDARLVIAGGYDSRIAENVEYHAELQDLATSLSLTHHTTSS

LQTPPPPTPILFLLSVPSQLKQSLLSSARLLIYTPSNEHFGIVPLEAMLSSTPVLAANTG

GPVETVLDGETGWLRSPDDTPAWTAVVSRALQLSDAQIGDMGRKGRDRVKRMFGREKMAE

RLEATVDEIVELKRPPPVLNAVLNFLGITLVFVLGLGTANVMSRLHGKA

>SoG_02969.T1

MNPSSNNVPDQGLLQNRGDSRSPAPLQTQLPPAPSIPSKTAQSTPMSSPGLFSPTGIRHN

AAAAHISLSESTTPSGFGSLHPLQNHRVRETHKANIDSDNVTGRKSINQYEVIEEIGRGM

HGKVKLARNQQTGENVAIKIIPRFSKKRRLGKLSGLAPQDKTKKEIAILKKIRHPNVVAL

LEVIDDPELKKIYMVLEHVELGEIVWRKKGLPHICIFERRRIEREMRGEPPSADEERYMQ

LLERRQRIKQMKRERMAQHYPDASNYWSVEHGAADEASSYGQHSRIASQDDFAIEESMPG

SRSSSRAPTRSLSNRSFNEAPFELEEDVDWDDDMETPGPLRSNHTSSAGLEAGSFDIPGE

GEYRGRSPSMADSIISHMSSIDFPHQSHDAFVDDFSYVPCFTFQQARSTFRDTVLGLEYL

HYQGVVHRDIKPANLLWTKEHRVKISDFGVSYFGRPIRDGEADETVSESEAQDFDDDLEL

AKTVGTPAFFAPELCYTDVNQEQPKISEQIDVWSLGVTLYCLIYARIPFLADDEYQMFKK

IATEEVYIPTRRLKPVDPSTSPVVVSLYKRQNSDPYRDDNDLEYEDVDHDLIDLIQQMLI

KNPEKRIRLRDIKRHPWVTSDISNIIGWLDDSDPARPTEGRKIQVDEKDMSYAVVPIAIL

ERARTMTRKVLNKVMHPLGDRGDSRSRHRASSSAASSAGDSMANNVPTTPGQRNERRRSI

LPDDYFATAIQDSLMQAETSTAPSQSDGATTPNQLYDPLATVLRASEIPREHARSGSAAA

AFDFSAIPSRTMSSGHRHGYSVGRLPRHGQHLWMPSGRHTTPTTPFGEVRAQSPAIPANP

AQAVPQMRDVESSQEELSRSKSVDRGLFASTDKRAQAQVSLNKAAAPGSIMTPIQSSNMA

GKSSQARATVLASPPHLVQQRSDNAAAYAYHEPPSDSSIHANFRDTVEHRPQTAQKSSHE

HTLHGLGSFAYPEHFAQVQADQHRRHQIELAHAQKNSSPLRDGSSTMTTSALWASQQRLP

AQAEISSTTVQSSSTESMGAVGTPLTSPSEITSPISVRPDGKNSNESMAAFQSDPSLPAL

LSGASSVSADIEGELLGRPGIVEGQPSMLLETTDSLTPPAMMKEPSTFPIQEVFANPPAM

DSGSLAVHLANAHRPHSPSSRSHTPPAECHDGDDEDDSGSDDGLLIMAKSKKKPISPYSP

AGSPSPSPFEPRRRDTNISIMSTETAKKVSID

>SoG_02971.T1

MTTDTANEAETRANVGKQAETLPARKQIRFVNNQGQPPSKRRRINAACLTCRRRKTRCAG

ERPLCSTCTKNGHECLGYPEERRDGPDGSEPKSAQDDSNETDGDEQELAEKAHHTRPSQS

EPVGRVNPVTAKTKEEAVPSTTDMSAADATAATAQPFIPAHQRAVNSISDSDPLSPTLHR

NSHSHRVPYFRYFGPTAIVPGFKQMVVSVRDRHHSGSHSAISPLSTPSGVQRASTAGSDV

FMEELPVYDVNDPTPVHPIIISLVNTFFTHLGCNYPFLKQRKFLQMIKEKTVEPILVDSV

CAIAARFSDLPALTGGNEKMPRTDRGAVFAQRAKAATVDTFPAPSVGAVQACLLMAYEGF

GANQDSALWMYLGLAIRMAIDLGLQKRVGIRYQGDQDPWFTRQRSRQNGEDSSPDLKKGE

ADALTLEEQKEIEQERIDTFWAVFFLDRVISSGTGRPVTLREDDFELSYPQSHMETLTKW

PAPFSALVEIIHLYGRVSDVLNNIRDIKDLTQEKWDRLRKLEHQLTRVYKNWDPRLQFNV

NNFKAYLGQGQGTTFILLHFWFHALFIILHQPTLMTPFGDLRSESQLLSDSRELSMSSAK

TICDILAFADLIDPNSFVGNPFTSQPIYIAACAFLMESSANASAAPSREGSQPRTEVPAT

KSDGAGKSSTASRHSRHSLLASAANQNYQRCYSSLQHLHTYWGGIKYILTALDQKSKGIW

DVETFTTEEYESTDIRRTRELGSQFPFEGHATSPKMTGPPIAWSLAGTANSPNSSLTLMY

QNVSGINAASQSQGLAGPMSMPSPGNMVYDPIRQNSSEGSMLPPSVPQPNIFNACAERLT

TQPQVLGFDGSGNRNGTRPDDANNGGLGKIYMPPNFTPGGQPSASFDAFSVSPASGSLPD

GGGGHHLGGGIYGQAAFPVSAWGMTGMDAITFDSQDIDIGALGLQGQELMGGWLDYIPGD

MRGLYGEDHMGHQGQ

>SoG_02974.T1

MAIYVTEITEADIDGAVKAVQPTCPKSSATKAQMRIMKYSTFNSQRNHASLALRMRWGMR

NGIFHVAKEEGSDKVLGVAMWLRPRPADQPVTWTDWIEGWRLWAGQVAMNTYYGRGGLNV

KRYYIWKDAQAKVQQELWTDPRGYYFLNIMVVLPEAQGKGVGAKMMRAVSDQADAEGMKC

YLESSRDVPNMAIYGRLGFRFQKEMICDDDGDAIKLFTMIREPNAKPGDGR

>SoG_02983.T1

MEWKTFNETTDGQLVKTEPLARSCYGEDKSLRECAFVNKMWSDQEFQTSNPIGRAYPYNI

TCAPVDYAQGGEPTTSCILGSLPFFAVNVTEKEHIRNSLRFAREHNLRLAISGTGHDLNG

RGDGYGSLEIWLRHYRNSIDFHERYESVNGCQKSDWDGSAIKIDGAWQWRDVYPVAKKNN

VIAVGGGSIGPGAIGGWASGAGHGPATRNYGLGADQILEAEVMLADGRIVTANHCENVDL

FRAIRGGGPGYGVTLSSTIKAHPNVDIVTVHKLAIAPLKETPENGDLLDAVSLMLQSLPQ

LSDGGYAGYGYWFREFPGPFVGEAHSGYTHGFWTIGKDKETAEDTWAPVQRKLEEFEDRL

LISSTFSTYPDYWSFYDAESGLYDPAGDTAILTSRLINPAALSDLPKLRSAVKTISGFPG

QIVSNVILLVSGGQVFEDASDRTSGLHPAWRTSHFVIVSGSGVSKTPTLEERRAANDAAT

YVRGEALKSLAPRTGGYMNEGDRNDPGWKETFYGCMYKNHLRTKRKYDPDGVFYCATCVG

SEDFVERPDGPLCRV

>SoG_03000.T1

MAQRVPPFHLIYLLRLFNALTLATFFQPDEFFQSLEPAWNLAFGSGSGAWLTWEWHHQLR

SSLHPILFSAAYKAVSSIFSILPPSANDVLLTPALLAAPRVLQAFIAATGDYFTYRLAAQ

IYGPSSTASLAAIYLQLLSPWQWYVSTRTFSNSLETTITIIALAYFPWPLLSSSTHPTKE

NPKGLRPSIPLCSLRISLCLAALAVVLRPTNLFIWAPIALSTFWRLSTSNILRFMREVTL

CGSLVLGLSVFADRWYFGFWTFPPWRWLTFNVTKSLAVFYGRNPWHYYLLQGIPLLCTTS

LPFALQALWKPKGPEADANGHKTATLSTLSWAVFTTVSILSLISHKEVRFIYPLLPALNV

LAAPYAASFFTSPSESQPSKHRTLSLRNKPYLFAALGLNIILAGFLSFLHQPAPLSVLHF

LRSDYARIYKPSTILTPAVHLSDANTHDDDDLFALFLMPCHSTPWRSHIVSPSLHARALT

CEPPLHTQPNTPERDLYRDEADRFYDAPLSFLTSELFSPSLSTSLKTLPRYIVGFEGVEP

WLQEFAAETEVGKSLGLKLERVWEGFNGFFHEDWRRAGKIVVWETGAYTPGAPGRTSLGR

>SoG_03001.T1

MAPHADESVAPSSPPTNPRFHATSTSAAIASENEHAAHNYHPLPVVFASAQGVNVWDPEG

NHYIDFLSAYSAVNQGHCHPELVKALTEQAGRLTLSSRAFHNDVFPKWAEKVREMFGYDM

VLPMNTGAEAVETAIKIARKWAYKVKGVEKDQALVFCAQDNFHGRTMTAITLSTDPESKD

NYGPYVPNISASCPITGKAIRYNNVEDLQTVLEAHGRNTAAFIVEPIQGEAGVVVPDEDY

LTRVQELCKKHNVLLICDEIQTGIGRTGRMLCSEWAGIKPDMVTLGKAISGGMYPVSAVL

SSKEVMLVVEPGTHGSTYGGNPLGCAVSIRALEIMQEEDLTAKAEKLGNIFRDGLKALNN

PIIKTIRGKGLLNAVVIDESAANGRTAWDLCMLLKSKGLLAKPTHGDIIRFAPPLVITEA

EIRKGLSLIAEAVKELPNVEKSKGH

>SoG_03005.T1

MPSSEDIGLDMFMATRLAEVAHVAKDIGYARILGTVTAAWLLWKVTHALVLSPLRNVPGP

MLARLTSKRGDLDNFSGRVCQTADHDIARYGEVYVYKPNAVCISNPDDVRRVLGSQEFRK

ASFFDIFDDGSTKNIVSQRDPALAARRRRQMGPFLNYGYLTKMEPVIQRHGYLAIRAKWE

GLLAESEGPAVEVNYRIDTQLVTFDIMSALAFGRDPSSISKGKSSITEWSGIIMKLLENP

VVLALLSLIPFPMLMRPWKKMYRDLAVYSKESGRMRKEFLATGAPAPADMLQAFIEAEDP

ESKVKMSEQEVQAECIMMMLAGSETTSSAIMWVFHLLLLHPEKLRLAVDEVRSAFGPDHL

ISNKDVLTKLPFVEACVFETLRMSPTTAGLTPRVSHDRGIQLQDHFIPPGTEIYVNLRSV

NMHESIWEEPARFRPERFVGNEEAKKTLFTFSYGPRNCIGRNLAWVEMLTIVANVLKDYD

IALSPDCEWRPENVDENGNPKLLPAKCFIASFPSNPERDCRMMVSRRSDLSV

>SoG_03006.T1

MDRPSTPLGARPPDYSLPSYDDEHDTPNGTNSAAVRLLTSVDDYDSRPYVPPRSPCLPNE

ASSIPSSSFSLVPPQQRSTAVPRGPSTTARKPVPQKLPVPLAPLLPSENSEEKRRETKSS

ASFTTAGQHLRTLKQKEDLVSQRASSPTQRGSLLHEDQSNPRRSSCLTRPASVKLQKRNK

PKQVVISEFVESLPFIPEGPSDRLAQRSSSAAPHPHHSLSSSSPPPPSPPRPVHGNPPKK

NSVTMNPPKYTRRSPRKLPSPPRQTRQSYQPSVNSSHSRPGSIMGDVPTMPPPDSTYVPF

AGRDTAGSPQRPWTPSSRMSDYSRPPPSNVSYEPADINGSPRPGTPSSRYGGSPRRPLPP

APLFSNPGRNSQAFAEDATIDIPLDEPNGRHGRDNDDVFGPESDLSEARPLPVDRNSYMS

ESQVTLNQDPEDDDAEYYDEKTAHYGPAPDGAQERRGVRAPQTSTREVQLINGELILECK

IPTILYSFLPRRDEVEFTHMRYTAVTCDPDDFVPKGYKLRQQIGRTTRETELFICVTMYN

EDEIGFTRTMHAVMKNIAHFCSRSRSRTWGEAGWQKIVVCIISDGREKIHPRTLDALAAM

GVYQHGIAKNYVNNRAVQAHVYEYTTQVSLDSDLKFKGAEKGIVPCQMIFCLKEKNQRKL

NSHRWFFNAFGKALNPNVCILLDVGTRPGTNSLYHLWKAFDTDSNVAGACGEIIAMKGKF

GTNLLNPLVASQNFEYKMSNILDKPLESVFGYITVLPGALSAYRYHALQNDETGHGPLSQ

YFKGETLHGQHADVFTANMYLAEDRILCWELVAKRNERWVLKYVKGCKGETDVPDAVPEF

ISQRRRWLNGAFFAAVYSLVNCRQIMTTDHTLARKILLSIEFVYQFVQVLFTYFSLANFY

LTFYFIAGGLADKRVDPFGHGIANVIFVILRFTCILLISSQFILSLGNRPQGAKKLYFAS

MIIYAIIMLYTTFACVYIIVSQLTAKDGEKVEIGDNVFTNLIVSTVSTYGLYLFMSLLYL

EPWHMITSFVQYFLLLPSYICTLQVYAFCNTHDVTWGTKGDNVMKTDLGNAVGKGQTVEL

EMPSEQLDIDSGYDEALRNLRDRVEVPSSPPSESQMQEDYYKSVRTYMVLIWMITNGILA

MGVSEAYGSSGIGDNFYLRFILWSVAGLALFRAIGSTTFAVMTAVSFVVDGRMKMSLKAP

KWMGGVGGKISDKVSSVGSSMRS

>SoG_03011.T1

MNATVTLRQAPRPAARLARKVIIWPCASSQPVASSTAPFTLQARAVHGLPQLGNGSSRPR

LSSRLPTTFFLVRSLSGKPLPQRKSKILNFAYRAAAWVGVTLTVFGVSVIGFFLYDASTY

KEHATHSDINVDQLALQPRKGGPKGLPIADVFIDDDDCEERRRQKQKPRLVILGGGWGGV

ALLKELNPDDWHVTVISPTNYFLFTPMLPSATVGTLELRSLVEPIRRILASVKGHFIRAK

AEDVEFSHKLIEVSQLDSSGKEVRFYVPYDKLVIAVGSTTNPHGVKGLENAFFLKDIDDA

RQIRNQVIQNFELACLPTTPDEERKRLLSFVISGGGPTGVEFAAELFDLLNEDLTRHFPR

LLRNEISVHLIQSRSHILNTYDEAVSKYAEERFARDQVDVQTNSRVQEVFKDKIVFTQKQ

EDGSVITKELPVGFVLWSTGVSQTAFCQTLAKKLGNSQTNRHALETDTHLRLNGTPLGDV

YAVGDCSTVQNNVADHIITFLRTLAWKRGEDPETLQLHFSDWRQVAQDVKRRFPQAVNHL

KRLDKLFAEFDKDQSGTLDFGELRELLGQIDSKLTSLPATAQRAHQQGRYLAHKFNKMAK

LKEGLTANDIRDGDLDAAVYKAFEYKHLGSLAYIGNSAVFDLGEGWNMAGGLWAVYAWRS

VYFAQSVSFRTRSLMAMDWAKRGVFGRDLVSF

>SoG_03019.T1

MMKRDARPVQLHPEPSVPIKPQRGAIVNVSSTTGVTGMGFAAYCPTKHAVIGLTRNGAHF

YESHGIRCNSIAPGGTETPMSIAASPEDQRGQYGADTMALVQPVPLKTFARPEEQASVIS

FLLSSESSHVNGVNIMVDRGFANTRTS

>SoG_03026.T1

MSDKRPASTGLTRPKRSPCSTALVSPAQQAQTRDDSGPVGLTPSVPGRPYEMSSPSNSVN

RPEATDPGPLGPNQSIAWRLDALENHLAEAQGSSSFTTRYTNHAVTPTCGTDAAHEVLAL

LAKELPKLFPSSTTDRTQPYQPGSNTNKRRRLDHKDSSDAVPQPLGKPPLPSPELIKLIV

DVYFTHAHPWIPMLNQDRFRQRVDDPAGLPMLETIIHAIHITVAKYLDEGETMTNQWSPD

RIRRWVTVTAMETLSIENLQALIIIAFYEIGNGNASRAWSIVASLSRTVEYLQITIDPEE

GESQPVNRPFTYLPPAKDWTDQEERRRVFWNVFLLDRFGSITMGWNTSLTSIDVHQRLPC

DGILWRKQEAVTTPYLGIWDKSTGGLGNLSPLSSRLHDADPTAEEMDRRVSWSGSFPISR

STETDVSRLGAFAYCVEATESMSRVVTYFLQRRFDPANSEEVTAWLARFKELDLRLIHWK

MFLPQKWKSKMPQQASRMDPNLTLAHVTHNASTILLHQLIAYPPLSWPFRTRLPSVWSSE

TCCLAGMEISTITRKYLENTPQSLPLASPFAFCVYIAARVMLIHWRHDGQNRQLDEFWSL

THSLEEMSRRWNGPSVSPEAERRDICAKYAWTLRDLHRRCIRDKHYRINVMDYTQEIDHG

AFIVLSERPYPGGLGMTLSAAETQEPIDPQQPPWQPAPSDAPACFPDGAAASNDTSHIPV

EQPAMRLPHYNVGLTPIVTETQTPHRPTIPNDTEMTRQMATNEYLVDMDRVIEFNDGSLF

SADMGSGVWLPSDPVLDTDLASVSHGGMCFLTCYEVTQEVFSNNHGSLSTAIGELVAAYA

SEGLIRNKETLRRDSTGRRVP

>SoG_03029.T1

MGSDPYGQLSSAEETLNHPAFPTTVWNLEPDRNGIASVAQKRGGPFRISWEVHGVGPSKI

LLVMGVGGTRRTWQQQTKYFGHDRREDFSVLVLDNRGQGESDKPLTRYTTSELALDIIDV

LDHVEWTAPRSVHLCGISLGGMIAQEIAHADPGRFRSLTLVCTTSAMQSSKPFLEELSQR

TSVLVPKSEYRAAVDTARQIFNLDWLLGPDETIPPVPGETPKCAPPRGGGTEYLKFDNNW

QRFLAGELTKRRDPGFYSKMGVICQLLAAGGHRKSPEQLRAIADVIGRGNIMIMHGTGDN

MIGIENGRALMTIMEPGTALVVEGMSHTPVLDRPHWFNQLLEEKVRGWDKDEQLNR

>SoG_03032.T1

MEHEEHKSWLIVDSNPVPGGLASTDVTPEGFLFDVGGHVIFSHYRYFDDVLREALPQLDD

WFEHQRVSYVRFGGQWVPYPFQNNIAVLPAEEKARCMESLIDAALEARVRSPTDKPSNFD

EWNVRNVGKRLTEIFMRPYNFKVWAMPTTKMNASWLGERVAAPDVRLLMKNVILNKVAGN

WGPNATFLFPARDGTGGIWKAVAKTLPSTKTRFGAGSAVVKVEPEAKTVHLQNGSTVRYK

TLITTMAIDSLAQVMNDVALQAMCKPLFYSSTNVVGVGIRGDRPERIGDKCWLYFVEDNC

PFYRATIFSNYSPYNQPGADVKLPTIQLASGQKAESVDPQPGPYWSIMLEVSESSYKPVN

QDTLLEECIAGLVATDLLTPTDEIVSTYVRRFDHGYPTPSLERDSVLEKALPYLQQKNIL

SRGRFGAWKYEVSNQDHRRRDRNDLRMLISGHCSFMQGVEAVDHVLNGAVELTLSYPDFV

NRRNNNERRLRSKE

>SoG_03034.T1

MTVASDLTQRASSRASSHAPVALEDRFEVIKEIGDGSFGSVVLARVRTAGASVARRGTVI

AIKSMKKTFESFQPCLELREVVFLRTIPNHAHLVPALDIFLDPYTKKLHIAMEYMEGNLY

QLMKARDHKCLDNASVKSILFQIMQGLEHIHSHHFFHRDIKPENILVSTSSHQDSSNSFR

RYSALVTPPSTPPTYSVKLADFGLARETHSKLPYTTYVSTRWYRAPEVLLRAGEYSAPVD

IWAVGAMAVEVATLKPLFPGGNEVDQVWRVCEIMGSPGNWYNKAGIRVGGGDWREGTRLA

GKLGFSFPKMAPHAMETILQTPQWPPALANFVTWCLMWDPKNRPTSTQALAHDYFFDAVD

PLRPKSSASKILGRKQSDLGRNSKDSSAVTPPTSKQSWFRKSLIGRVEVAEPIIPAVAPQ

LKDPLARPTPIQPTTITEITAQIQPQKQRAVNAKRSTWTNGASNMAPMPILPSIRPISPL

SDAVTARAHEAQANLQPEKSTKIGRQLSVASSTNNYTEMHRQQAERALNGGLASPPNGQK

EGFFSHLRKRARRLSGRHQTPVSPAYDDLEAGAGCGPWNSNRSSMIVDQQTQVPIPKSEV

YESLDRALRDVQNSLDSRQPMASNSGSNTTLKRHHSLPGQQARSVDNLIGAVRTGPISSR

TRRAQAAHGVQQYEAPDEEDELLDEVLSSTHRAMKRMDQNSKPLRQSASNLGLSNPYPTP

SPSASGNILFSDANEALAPRPLDLSKKTEGQPKWPTPPYEESEWAASASASIWAAGQRF

>SoG_03048.T1

MAEYLSMIPEDACSRAALAVVGGGTAYLLSLAIYRLYISPLSKFPGPKLAALSGWYESYY

DLVHQGKYLFEIEKMHDKYGPIVRVNPFELSLRDSDYYDTLYVMGSVRPTNRHEAFVDGT

VDFKGSHIATIDHHLHRQRRKPLDPYFSRQGISKLEPMLAELTEKLITKRFESLKGTGKI

VRLDHALTAYSGDVIQRLCIDEPPNVFIEDPEFAPWWYYMFHHGISMLPVFMAMPWLISL

IRLVPLSVQARLSPSAQTFNDFKKFCDNQLQTVKKEQAAAGSKGSLLLNGRPTIFRQLLS

SDLPPSELTDDRLSKEAQILIGAGTITTAGTMCFISYYIMTNPNIKKRLQEELAPIMADY

PRRQPSWAELEKATYFQAVIKEGFRLSYGTMHRRTRVSPDQPLQFKDWVIPAGVPVGMSA

YFCHRDPKVFPNPEEFLPERWLGEVTAAMKRNFVPFSKGSRHCLGQNLAYAEINYVIAAL

FRPNGPDFDLYETDERDVKPTHDLIVPLPSLESKGFRVIFH

>SoG_03051.T1

MTSRRVVAIDQNWKFKQAGKDDSDYRAVAQFPTMVHLDLMHHGLIPDPNIGKNELYVQWV

GETDWVYKTTFVSPSTANGDKAILAFDGLDTLAEIFLNGQNVGSSDNMFVAKRIDVTHSL

KPEGEENELFINFSCAYTAGEELQKKHPEHKWKCWNGVPSRMAIRKAQYHWGWDWGPMLM

TAGPWRPVNLEIYHTRITDLFMEIKFDGSLRSAEVIAHAPIEGDGTNVRFDVSLNGEKVA

SETVKSNGHAAATFVIQNPRLWYPRQHGDGGGNQPLYTIKATLVEDDGKALDTMTKRIGL

RKVELVQRELDGAKGTSFFFKVNNEPVFAGGSNWIPADNFIPRISREKYYDWVRLLAEGE

QNILRVWGGGIYEEQALYDACDEMGIMVWQDFMFGCGNYPCFDEILENIEKEARYNVARL

RHHPSIVIWAGNNEDYSVAESEGLTYNFEDKNPQNWLRTDFPARYIYEHILAEACKDLCP

RTFYHPGSPWGAGKNTADLTVGDVHQWNVWHGNQEKYQDFAKLMGRFVSEFGMEAFPSIK

TIDGFLPKGKDDPDRYPQSSTIDFHNKAAGHERRLALYLVENMRYKFDPIEDYIYCTQLM

QAECLSAAYRLWRREWRGEKREYCGGAVVWQVNDCWPVTSWSICDYHLRPKHAYYAIRRE

MWPIVARLERKEVESSQPPTVRVWASNLTYAEVKVDCVVKFWDVETGEELASKSVAHSQV

LKANQTTDFENLTMADLRLKYHNDKVVIGVFLHHNGQQTSRYIDWPQPLKYLHLAKPKKL

VVELAEEGGEQAVLLSAEVPVKGVAVECEDEGVVFRDNLVDLVPGEKVKIPVEGARKDTV

FTTRSLNGLF

>SoG_03057.T1

MRFSALFAALLAPSVVLSESPPEPEAPKKIPILKLPYGWWEASGESKGVWTFANIPYAAP

PVGDLRWAKPQPPLKRDRLQPGNRNGNACLQAGLYQRNLMGPEDGTIFGQAINAASGSIT

AIPKGKPLDESEDCLFLDVQVPEKAVTNSSLKLPVVVWVHGGSFSAGSKDFLQPSFPLYD

GSPLIRESGGEVIFVRFNYRLGAMGFLAGTTMEKGGLPNAGLWDQRAAFQWVRDHIHLLG

GDPNRVTAFGHDAGAASILFHLVAKGGTLDPLFERAILLSPAYQPMWDRAGAIENTFQQF

ATLAGCKGRGLKCLRAAEVDTLRHANGGLLGQQIPGTFAVGAAPDGSFIRQSPTAELWLG

AAWPVKGLIMSYARHEARVFVNGYKNVTTGVDRFVDAILPESEFTEGFRERVIKDTYPPP

GTEGGKYSDARVRLDDMIRDSSMVCNTRWLTEHYGQDSVWLSQYNVEPGLMYTNLFAVFW

NEVGYGLWQIADFIRWAVDRGLYNGLFMRGLSRAYQTYLHSFIRTGDPNASRLSRWDNRP

YPGTEYWRHPWVVKDEDDKDGIIKHVLDVRWDLFKFFDPFDSNKLPKDKCDFWRRFTLSA

TVAGMYVPDGEWLNNTWTDTLDPSARYRGGNKAPA

>SoG_03072.T1

MSAFTALNGGSPKGSEPPATFADSNNAPEDRPRSSAAQSQAGTENSAPRDGWTSQGQEGS

ALQTSPKIDADSSLKRKRSHSTEARREHLIQERTPDTATVPSHGDSRDPFDTPQRDRDQW

YGHQGREERSHHEASQSSRTSPGHAEEHTGDALRRATQNERSEYEQTSPEGEERSAGAYG

SPHTTAQRQGPILQHDPKKRKRNFSNRTKTGCMTCRGRKKKCDETKPECINCVKGGFVCA

GYPAQKGAQWQKPSEKTPTVPLESKDPSYVPPGAYGMPQQGPYASQPGKREPLPPYRGQA

LRIDPPQGRPLTTEDDRPTASTIPSASVASPDNKLSAISSYTTGNVFPTPVSANTQPPPF

SERMGKEYQRVPPLHDLARNEPETPHPGSHLPQINILHASRKNSPTQGGQQPSSNPQVAA

QLALSHPGFPARRTQKEDMLSGRSYYPFDKELCLERERCASACWRFNNLTIPSHGVSHEE

RGRLFMDILQPREAVRVSPTELSPVTNVGRVGREVAVETPFTCDYGYNITIGSHVAIGRN

CHINDVAEVRIGDNCVIGPNVNIYTATLSTDPKRRMGGQGPQTGKPVIIDQDCWIGGGAI

ILPGKTIGKGATVGAGSVVTKDVPPFTVVAGNPARVLRGIAS

>SoG_03077.T1

MADEGVAEHYQVLEELGRGSFGVVYKGIERATGEVVAIKHIDLESNDDDIQDIQAEIAVL

STCASPFVTQYKCSFLRGHKLWIVMEYLGGGSCLDLLKPANFGEVHIAIVCRELLLGIQY

LHNEGKIHRDIKAANVLLSETGKVKLADFGVAAQLTNIKSQRNTFVGTPFWMAPEVIQQD

GYNFKADIWSLGITAMELANGEPPLCHIHPMKVLFQIPKNAPPRLEGNFSKDFKDFVAQC

LTKDCDRRPSAKELLRHRFIRSAGKVEALQELIARKQMYDANQNRQKHPIYYQETLQTIS

PKDDEQEWVFDTVKSVAPPKRPTVRHRKPSSIFAADEAMRKLDLKDAPLGTSSPAPAHGT

VRRSTVRRAPSLAQVSSMHRNGSPRGSIAPKKPLQPDMSFGNSGSTMRLFRRVPSDSSTN

GQLGRPTSPDDVFQDENLPPSIATPVEPYGKEAILGRRLYNKALEPTLAELHAQTSAMQK

REALAKLSDAFAALDAVDPEGAHHLMTNLVATMSQDKKLNACFLQQAVQKTPDDGTPQGT

VLIKPSTPALSPSKLVLAPNNPHLKSHRRRQNESPALNEKDYERSMLEHKYPGREAKAGM

EHCKQLSEVLYSRWSDGLRLRWPAT

>SoG_03086.T1

MAIPEQEPIAIIGMACRFPGGSDSPSKLWDLLKSPRDLSKRIPADRFDATGFFHHNGSQH

GASNAPNAYFIEEDVTRFDNTFFNIQPAEAEAIDPQQRLVMETVYDSLCAGGQAIENLRG

SNTAVYVGMMCDDWAQLVNRDWDLAPTYAATGDSRAIISNRVSYFFDWHGPSMTIDTACS

SSLVAVHQGVTALRNGECPIAIAAGANLIVAPGEFPPPERNERGSGGLRPADTDMTEPRP

PIGMFIKESSLRMLSPTGSSKMWDAGADGYARGEGIAAIVMKPLSAALRDGDNIDCVIRG

TAVNQDGRTAGLTMPSNIAQAQLIRDTYARAGLDINEPKDRPQFFHAHGTGTPAGDPQEA

EAISRSFFEQGRPVADDKLYVGSIKTVIGHTEGAAGLASLIGSVMAMQHGIIPPNLHFKR

LSDRVAPFYDHLEVPTVATPWPQTPPGQPRRVSVNSFGFGGTNSHAILEYYEPEEANKKS

VADASSVVPAFTPLTVSAASSSSLRAALDDLRSYLQVNPDTKMRDLAYTLQTHRSTLAFR

KPIFATDVKDTVQKINSLLADTHADDNGLNTRFFDVSKPGILGVFTGQGAQWPRMGARLV

EESPFAAQRIAELQRALATLPDDDRPDWTLMGQLITQPKSSRLSEAVIAQPVCTAVQIVL

VDLLKAAGIQLRAVVGHSSGEIGAAYAAGFLSATTAIRVAHYRGLYAKLAHAGGPGAMMA

AGTSFEDAQELCELVGGIQVAARNSLTSITLSGDEDAIDEAVEILKDEGKFARRLLVDTA

YHSLHMRPCAKPYLAGLARAGYAVQEGNGTIWFSSVIEGGHIMTKKDLQSPQYWADNMAN

AVLFEPAISQAVAVAGPFDMAVEFGPHPALKGPALDTIEHAAGHRISYTGLMARKKDDVE

ALASALGYIWTNLGASSVNFDEFENLVSGGESLPQKKIVANLPTYPFDHSRSFYSLTRFS

GAHRHLHSLPHPLLGRRLVETETADEISWRNLLRPSENSWLQGHALQGQSVFPAMGYVSL

AVEAVAATADDARRKLGLVTINNVVIGRALSFDGDNSGMETKVTLSLVRSSDDEISGRIT

CHSGLPFDSGSPLTLNFSALITAAFHEAEPDTLPAIRHDGEDEEINLVDAEAERLYSQFT

KLGYTYGSPFTGVRTIRRKLGYAVGEIEDESGDGWEDELLLHPGWLDSAIQTGFAAYSHP

HDNRLFTLCVPTSIRSVVVNPYFCEKRSAVRQRKLQYQTSTRATPEGHMMVDIDVFPGGQ

GAQGHPFVQFEAVELQPFAAPTPRDDAVIYTRYDYRVAAPDVRLAIKGDEESNMTSSEND

KALLAAERVAFFYLRRLHEAITDWEKADAAEHHRHLLDFAARAVEAVSAGRHPLLPCEAL

RDSPAFINSLIAKHHGHEYIRLLDLVGNHLAKEILSVGGSIFDNNHPEVEALLNSFYQQL

TAQSGQDNANAWYARVVSQIAHRFPRMHVLEIGAGTRGGSMASILPALGDAFSSYTYTDV

SAEAIEGVQNRFQDQIRTLGDRLVLATYDIDQPPAEQGLDEGAYDVVLASMALHTAVDLD

ETLANARKLLRPGGYLVVLEINSNHSLSLSTIFCSRPRWWAGLDGPALSLDQWDSLARRH

GFSGVDTATAFSTSNGSDEDKLIWFSVFASQAVDDRVNSLRAPLATPAVAQGPDQLTIVG

GHTGAVSKLAKDVGALISSRYMSITILESIEELFERGLTPGSSVLCLTELEQQLLETRTE

PKIEGLKTLWRNGHSILWVTREARYERPYSSIILGVSRVLRHEYPTITLQLLDFDAATEA

TPEILASTLIRLELGAYLKKTEQLTDLLWTIEPEYHYVNSQLLIPRLLPYTEANNRYNTY

RRAVYDKFDPYETAMMLEPAANGSSFELATVSPLRVLPSPRTAGRVVAIKVEQSLLQVLK

ICEIGYFTLITGIEVETGQRLLALSDSAVESRAHVPIEWTARLTGSKTEVFSISLGNLAA

HLLAQTILARVSRSSGTAVVHEAGQLLKDALNKEADREGVQVVFTTATKNYPAYAGRSSP

YIFIHENLPTRLVCGLLPRRISLFVDLSSEPASACSDLLARSMPSHTVRVKAADFLRTHP

GLSSNSGLDAGDTVDQIGRILKETWKRVAPRGQHESFSSLHSRVPVLALQEVTRSPALHT

KFKVVNWITSSPVSALVRPIDHGTIFRADGTYLLVGLTGELGQSLCSWMVAHGARNLVLS

SRRPKVSQRFIDSCAVEGATVRPMSIDVTNRESLREALTTIRASMPPIIGVANGAMMIDD

AMFDDMKFDSLQRTAPPKIEGSVMLDEFFYDTPLDFFILFTSLANVVGNTGQSAYIMANQ

FMAALASQRRNVRGVAGSDIAISSIQGLGYFEHANHLDKDHFTRIGYRNVSEQDFHGLFA

EGILAGRPGQKGGSEVCTGVSPFREGATLLANPCFSHLLLHDAAAVQGGRGGGSSGKAER

PRARLAAAKSAAEAKAILRESFVERLKRILMIPQGESVNEKVTLVEQGVDSIMAVEVRTW

FLQEFSLDLPVLKILGAGSTLESILGDAMKSIPTEILDLDNIVSDKTSSHHGSASQASAP

APAVQAPPTASLSSTKPSTETSYSSSESGSHPAISTPRSPSDTPLERSLDLSDEIERKKL

TAKALERQLEDSRRGKLTAALNLPEHTEPMTFGQRRFWFLSHYVEDDRTFNIAYQFKLSG

RIRIDDLAWAVESVAQRHEALRTRFFWSDDDSRTPMQGILSKAFVRLETAMIESEVQAAK

ELDAMRDHKWHLGDWGQLRLKLLSLSDTQHFLLMGTHHISMDGHSMNMLMLDINQVYNNR

GKPLPPLSDAVQARSFGQQQLLAHKSGKLQPAIDFFRHNLQDIELSRPIELLSFARSQVR

RPLQAYRSHIARIRIDSLTVSKFKQLTRSNRATSFHGYLTILQALLFRLLPAETTQKLII

GVADANRLDSRFMGSIGNFLNVLPLVFDRSVTGGDQAMFGPAVEETRKKVHSALEHSALP

FDLLLEELAAPRSNAWPPVFQVLMDYKLFTREQSEMPWAGCKVGEHQWHAARGPYDIALE

IVDDGEGAMMALHMQEALYSEDATNLFLRSYVNILREVVKPGGDKLKLGRLDKWDEGDVK

KALALGKGTDMPLEWPPTIAHRLDQVMNEHPDSLAVKDGFGTVYTYAELDERVNIIARSL

LEQLPDQRHQQAVVGVFQSPSADWIASMIAIFRVGAVYLPLDMKVSIPRLKSYVKTAKPA

VLLTDNERMVLVPDLTVAAEDTLDIINISNLPVKAHVSIQRVATTAKQDSTAYIIFTSGS

TGEPKGIAVKHAGIRAMVEGFVREWDIATLGRVVLQQFSLTSDGSLKQIFSAIATGGCLI

VAPADARGDPSELTRLMVEHGVTMTVATPSEYGMWFRFAPDGLRRCTTWTSAWFGGERSP

QSLLDSFQSLSSVLPNLRFYTTYGPTEATVSTMKGVADVRDPNLAVPVPGRILPNYAIYI

MDEESRPTPIGVPGEIVIAGAGVGQNEYLNRPEATAKQFHLDPFAPHDKKSTGWGRMYYT

GDYGRLDSRGYIAIEGRVAGDAQVKVRGFRIELAEIEGTIMKEAAGTLAHAVVTLRTGDG

DDDGFLVAHVVLENKAMDETQVAKTVNELRKRLSNSLPNYMVPAVVIPVDEVPLTAHGKI

DRKAVQALPFPEFKTSSLEQYGQQTKSFTPAELRVADLWAEVLPSLSLVAEPLSPQSDFF

RVGGNSLLLVKLQSVIRRTFGDAPRLGMLMSAPELSAMAALLESQGSAPDWDKEIALDML

DHSLGAQKQSRKSSTIGGSPGLRILVTGATGSLGKNVVPRLASDQRVAQILVLARAAEGR

DLANLFPSLSDKVRVVKADLPSLPTDNTAELEQADVILHMAADRNFWDGYGALKAVNVNS

AKALAKLALRTGATLHVLSSGALVDYEADGDAGGLPRPDPAHGYVSSKWVAERYLAGAAR

RAGLKVTAHRPTPAPTATHVSMDELTTAEDALVHSYLVNSLHLGVRPDFAQLGGIFHVAP

VDAVAGAVASLVVQEPSAEKTSLQIVNHPGTAALRTGVTSTHAEALFRMPENESVLSLPT

VPALHWVGMAKRAGLFEWFITSQELVVADAEGQKIVSKR

>SoG_03096.T1

MASVARTFTRAATRTVARNTLANTSRTTFRRAYSSQQSPKSSSSPLLILSGAAVAGGLGY

YFLSGSGAAAAAKPFVPTKEDYQRIYNEIADRLEEKDDYDDGSFGPVLVRLAWHASGTYD

KDTKTGGSNGATMRFAPEGDHGANAGLIAARDFLEPVKAKYPWISYSDLWILAGVCAIQE

MQGPIVPYRPGRSDRDASACTPDGRLPDATQGSNHLRNIFGRMGFNDQEIVALSGAHALG

RCHTDRSGFTGPWTFSPTVLTNDYYRLLLEEKWQWKKWNGPAQYEDKTTKSLMMLPTDMA

LIQDKKMLPYVQKYAKDNDAFFKDFSAVVLKLFELGVPFPEGSESSRWVMKPTWDESK

>SoG_03101.T1

MIRSEESNVEEMESLFCLFGLSIKQPRIDLENMASAAEVDAVLGRLTLEEKISLLAGSGF

SELTAIEAKGVPPVKAVDGPNGVRSAATDETIKSACFPAGCSLAATFDRSLAHRAGIALA

REARGKNAQCLLGPTVCIHRHPLGGRNFESFSEDPLLAGKISAQYIRGLQSGGVSATIKH

FVANEQETQRTTVDEAIGERALREIYLRPFEIAVREARPWAVMTAYNLVNGVHCDGHDWL

IQKVLRGQWGWDGLLMSDWGGTNSVADALNAGLDLEMPGPPRIRKLPAVLEALEKGQVTE

ATIDRRARSVVEFALKLDHHRKQPSTAPLKPGETTDTPELRALLRDSAAKGMVLLKNEDH

ILPLSAEKLSGKKVALIGFSRDAMAHGGGSAAVNSYYKVSPWDGLRAALGEDVEFTFAAG

AHRERLLPPINKDGSVGAIVGLDGQPGFTQILYTHGTTEKYSVKHGHARSAYSPLGSQES

LWRSLEIVGDFTPLETGSHYIACSGIGPTWVYVDDDVVFEQAGNSSDPMGSLFLAAPEPE

VQHRFDAGRTYRVRIRSECPVGIGLEILEGRSGVRVGFSLESVHDADLKGEAARVAADAD

LAIVFSGHDPQWETEGRDQDSFDLPRGQNDMIAAVAAANENVVVVNSTGVAVSMPWLPQV

RGLVQAWFPGQEAGNAIADILTGRANPEGHLPVTFPRAIEDAPAHGNFPGEYVDGKLKVK

YEEGVFIGYRHFDRLERSKVNFPFGFGLSYTSFETTSLKVDRQGETLRVAVGVKNTGSRS

GGTAVQIYAGSLAGSAEHPVKSLVGFDKVRLEAGKSKDVELEIPLRDAAYYDEAAQKWTI

DAGRLGVFAGFSAADVSRKDELVVEKMTWEP

>SoG_03121.T1

MGVCVLVQQGMLTLGNDGATLAGVIEYASDLFDRTTVERFAQHFQTLLEAMVADVEQPVL

GLPLLSPAQRQASPAALPPKAAFSTEALIHQSCEQLAAAQPHSVALVFGETQLSYQALNR

RAHRIAPTLLAQGARPDDRVAILAQRGGEMVCAVLAVRKAGAAYVPLDPAYPAERLGYLL

DDSTPVVLLAQPT

>SoG_03122.T1

MRLHPPPPPRVFDAVANELNAQHIYINLDNHISRGEWCCGGSDGNTWWGDRYFDLNNWVR

GLGYMAKHGTKWKNLMSMSLRNELREPQDNASLRATYNWQNWYPNMQKGADAVHRNNPGP

LVVLSGLNYDTTMQPIVRGQALSPGTQKFVPSQFAKGYANKLVIELHNYQTTIQSCPDLQ

GSIYNAGAQAMNPKDPSTVNAFPVFITEFGFDQSQYQSVYSTCLASWLPKNTAGWMSWVL

VGSYYMRSGTPDYDESWGLLNHDWSAWRNQSYVDDLLKPSVAETTSGNPKRH

>SoG_03123.T1

MVTMLKSIEDLRVMLLSFILLISCAQVSAVPGKPPSPGGRERSSINSGWTFKRWDQAPDG

LSWSQLEPWVLPSANDFIKDSSKHTKRPSGSSPNVEYAQTGFDDSSWEKLNLPHDWAIHG

PFFEGDDSPVGGSVGRLPIFGVGWYRRTLDVGPADKDKSIYLEVDGAMSYSSVWVNGVIV

GGWPYGYASFRLDLTPYLKPGPNKLAIRLENEPDSSRWYPGAGLYRNIWLTKVNPTHVGQ

WGVHVLPKDVSSSQATVDVQVNVENAGSSDKAKDIRISTEIFEVDSATGNPGPKVAKLPD

SKVKVSGGQTVSSNASVKVGNPKLWGPVPSQQPHLYVAVTRLYDGPREIDTYQTTFGIRT

MDYRNDGLYVNGERIYLQGTCRHSDLGSLGAAVNDAALERQLDLLLEMGLNSIRTSHNPP

EPELLDLADRMGILIFDEIFDTWNEHKVDNDFATIFPTWSEPDTRAFIRRDRNHASIWGW

GFGNEIPEQGNSKGVATAERLHTIIVEEDNSRKTALALNNAGPNQAIIPIPGLIGLNYQG

EGKGYGAPTFQNFRNAHPDMFIFSSESSSVVSSRDTYLFPVTNLSNEIVNADGVGADPKT

RQVSSYDLYAVPWGATPDKVFAAHDKYPYVAGEFTWTGWDYIGEPTPYDDSSRSSYFGSI

DLAGFPKDRFYLYQSRWAPNVKMAHILPHWNWPDRVGQVTPVHVYSSADEAELFVNGKSQ

GRKKRGQYEYRFRWDEVFYEPGKVDVVAYKKGEEWAKASVSTTGKAAKLRMESYKDRKSV

TADGKDLLFVSVAVVDSDGRVVPDADHSIEFSVEGSGEILTTDNGDPTDMTAFQSKTRAA

FHGRALAIVRGQDGGSGSITITAKADGLEAGTFTASLQ

>SoG_03135.T1

MASFRLLYAAIAALCVAPVLGNPTTRDQDHESQGLRKKVFDWTITWEDYAPDGFTRKMFL

INGKSPGPVLEVNQGDTVVVNLHNKSPQDTSLHFHGIEMYGTPWSDGVPGVTQRPIHAGQ

SFKYEWTATQHGSHWYHAHTRGQIEDGLYGAILIHPKKAIANPFELISKDKKTIKDLERA

EQHAHPLLISDFTHLTSDDKWQKTLESKIEDTCYDSIVFNGKGRVECLDPGLVAESITDL

QKTYLALANATMTDKSCVPAEALNLIIGGGAGNPDVWPEGVFYGCVPSKGSLETISVNRK

PSKDGSQWVAIDLIGAINFITAAVSIDDHDMWVYAMDGSYIEPQKVQALVLTNGDRYSVL

VQTKKPGDYKIRVNANSAPQVITGHALLSVDGPREGSQQEDGTAHIDLVGNPTSPNVRFF

NHLAAAPFPPSPIPQKASALYVLNMLADGASYLWAMNSTRLMPDEIETLEPPVLFNPRPD

VANNVTITTRNGSWVDLVFYASNFPMPAHPIHKHGNKMYEIGRGKGDWKWQSVEDAVEEM

PGSFNLRNPPRRDAFTSPEAVEGRAWTVVRYHVTNPGAWLLHCHVSNHVVGGMMLVIQDG

VDAWPEIPEEYQLS

>SoG_03147.T1

MFGKVLAVSSSFFFFSWALASPATLQAETRSPPPHAQQSSDVPYEGYAFLYFKNGDEQLY

LALSNGNNVLSFTEVNGGRPILVSTKGDKGLRDPYIVRWKEGNQFSILATDLCIGCGTDW

GTAGRYGSRSMEIWHTPDLITFSEQQHTLMSPESYGMTWAPEAYYDAELGTYVAHWSSAI

YNDTANPGRENGEYIRVVYATTDDFRTFSEPTVWEDYPPDGRIDASVFRDDDGTYYRFTK

GTVDGCADFIQESSPDLTAAQADWTPIASCIGRSRGTNDLEGPVILKTNPGDVGGHRYIL

LGDMIYDEATNRGYVPLQTDDLSTGNWTLLDTNTYPFHPRHGVVTPLTAAELEALWKAYG

GK

>SoG_03148.T1

MRTGLLLAAGLATGGVVSATRFNMDAHRFARRQEGGSGSSCSSNPFTGRKLTANPSWGAK

LEPTYDKYLAAGDVENAGKVRTIQEIGTFFWVSNIASLPNIDDAIAAARAEKAATGKDQI

VGLVVYNLPDRDCSAGESAGELDSTQGGLERYKNEYIKPYAAKLAEATDVSFAVVLEPDS

LANLVTNMGVEFCQRAAPVYREGIAYAINALQMDHVNLYVDVAHGGWLGWDDNLPLSE

>SoG_03149.T1

MAAAVRASDCDCYLTGGSSPTYFRDYGFWDFRSLPQHAGVPPLIDTLDGNTNAGFTSDFF

SRSSGFQEFWAPQRWRSQGGMARTNSFNNLYILANEDGSSSSSSSNETMLRMRTARGSGF

QSTAAFQSKSMIDHASVRVYSRTHGAAGACTAIFTYLGAENVRDVQEADIEVLTRDPADK

IRYTNQPAHLGNGTLVHGAGSQVTIPGGGRWTDWMVHRMDWTPGLTTWHADGREVRSQSF

QAPVDPSRIVLNAWSDGGRWTGEMGEGGQAFQDIQWIEMAYNLADEGACSRVCSVDGGDV

GSPAPVQGKVPKSNLVRR

>SoG_03158.T1

MRFATLLAAYASVASATVTWTLSKASNPTADQIDAYQRIEAAMTAAAARYNRLGDAQKSI

RVRYDPGIPTAEANYNGDLGFGSNRSYMSERTALHEISHTLGVGQTGGFFNRCSSGDWPT

ALPLLRSFDGPNAVINCGGGHFWPYGLNYDNEWSETNADRHCLMVNAMIDDGM

>SoG_03159.T1

MFPCSALLALAAQGALAWLPESRDLAAFNQTARFAELGKRFEPSLPSGVTKIRGVNLGGW

LISEPWMMGDVWKVTMGCGSAGTELDCMLQNYAGGNRENGNQAFAKHWQDWMGPDTVQSI

HDVGLNTIRIPIGYWSYSDVVNYDTEPFADPGPMLEALDAVVGKAADLGLYVIIDLHGAP

GAQQEDSFTGQTQRPAQFFNEGNFDRAERWLSWMTKRIHTNNAYRTVGMIEVLNEPVSNH

DRNSDGSSRYPAPGQVPGLLESYYPGALAAVRDAEASLSPPVSDDKKLHVQFMSEKWGSG

DPRTVSQIMNDAAVAFDDHNYIGFAVDDNGDQYKLMHSACTDSRVVDGESLIIAGEWSMT

SSVGWEGDDGKAFFKRFFTAQQQLYEKPGMAGWIFWTWKTELNDPRWTYSFATYLNLVPT

DAAGLEGNVYQDVCAGFT

>SoG_03187.T1

MPRLGAWLQLQLLALPLLSASATVTHDATQQHILQSAHTQAHAQRRKLQGRFLHITDFHP

DELYKTHSSTEEGVACHRKKGLAGAFGAEKTDCDAPVSLVNATFDWVRDNIKDDVDFIIW

TGDTARHDSDEKNPRDTESVFRTNRLVADKFVEVMSSDSGKLDIPIIPTFGNNDFLPHNI

FYPGPNKWLHAYSEIWHRFIPEEQRHSFEYGGWFQVEVIPGHLAVLSLNTMYFFDRNAIV

DGCRDPSEPGFHHMDWLRIQLQMLRDRGMKAIIMGHVPPARTEGKQAWDETCWQRYTLWL

KQYRDVVVGSVYGHMNIDHFMLQDAKEIDILSSASVDAEEEEEEEEEEAGFDKGFRDDAV

DIKSKGDYLTELRDVWSGLPSAAVEDLIKAAGHEDNEVSSTGKGGKKKRKRRKFRKIGGK

YAERYSLTLVSPSVVPNYFPTLRVIEYNITGLEDAKTWEDPFSASSVPGARNRDLTQIAE

ELRQELKRDMETGVSVAGKKGRKHRKKKGRKGKTKPHDPNLIVPEDPPKKSLPGPAYKMQ

PFTLTGYTQYFANLTHINNDVQEAVGGSGWRGGDHENKTPGHKPRPRKFGYEVEYSTYDD

KLYKLDDLTVRSYLKLAYRIAKKEPSGKKMSTFGDNDDDEFADDFYDDHDDFDEDDEEDE

EHRSGVEEVGKGKEKKNKVWLHFLRHAFVKTATKDELKKL

>SoG_03192.T1

MAYPQTTFTQTTFTGPSLLSSVRNTVSKTTLRTQLDQIKSTGRYDCFKLEWHPIYADKSM

WPVPFHLFWDSDIAKWIEGACYFLQGGYDAELDATVKDLVEMIRGAQQDDGYLNVHYTVV

EPDKKWSNLRDMHELYNAGHLIEAAIAHAQYYKNDSLVEPMKRYIALAHKKFGPSEGQLR

GYPGHPEIELALLRFYALTGHRESYDLAKFFLTERGNPKGQDGKHYYDYEEDLRGDSPWK

RPDAYPEHRAHWYNQAHAFIQEQPTVEGHSVRAMYLLTAVADLVALSGEASTELDKPEQE

AWIETLRRLWANMVDKKMYITGGIGAIKQWEGFGIDYFLPQGTDEGGCYAETCASIGVMM

LAERMLHIDLNARYADIMEICLYNNIMTSMSTDGKKFTYDNQLASSEQTKSVRNDWFWCA

CCPPNVTRLYGSIGGYLWDQDINEGHISINVHMYTCAELEVKSEDGASLKLRQDSKWPWE

GRVSFELATTGTQLPVTLRLRLPAWCKGNYSLEPAAPANHTKIENGYLVLDPAYVSQKAR

FSLEIKGFEPRYLSPHPYTNQHTLALARGPIVYCAEDVDNAWETNHFKDVVLKMDSPVTE

VRRRDETTGDEYVELRTKCWTRSATTGEVDEAKEARELVLVPYYYRCNRGGNGHMRVGMV

SGSGKPGNIFPEK

>SoG_03195.T1

MTPFSALLLFVAILACEAAKFRMARADFPQLVVQRQNGTSNLAYSPPVYPSPWMDPQASG

WEEAYVKARDFVSQLTLLEKVNLTTGVGWMGERCVGNVGSIPRMGLRGLCMQDGPLGIRF

KDYATAFPVGMTAAASWSRNLWRDRGKRLGRTHYQSGVDVTLGPAAGPLGRNPTGGRNWE

GFSPDPYLSGIAFADVVTGIQSEGVVATAKHWLANEQERFRQAGEARGYGFNISESMSSN

MDDKTLHEVYAWPFQNAVHAGVGAVMCSYQQTNNSYGCQNSKLMNGILKDEFGFQGFVMS

DWQAQHAGVSTAAAGLDMTMPGDVTFNSGTSFWGGNLTLAVINGTVPAWRIDDMAMRIMA

AYFKVGRTVENQPEINFSSWTQDTIGPVHMAAGENIEQINFHVDVREDHAHHVRESAAKG

TVILKNKGALPLAKPKFIAVIGEDAGPNSRGPNGCPDRGCADGTFAMGWGSGSVEFPYLI

TPDSALQAQAVKDGTRYESILDNYDWEKISQLVRRPNATAVVFANAGGGEGYINVDGNEG

DRKNMTLWRSGNELIKNVSAINPNTIVVLHTVGPVEIDEWYDNPNVTAIVWAGAPGQESG

NSLVDILYGKRSPGRSVFTWGRALQDYGVDVLHEANNGGGAPQQYFEEGAFIDYRHFDRD

FPEGSAKAPIYEFGHGLSWSTFEFSNLQVEKRDVRPYKPTTGMTIAAPKFGNFSTNLADY

TFPANVRYIYQFIYPWLNTSSSGAEASTDPHYGQTADQFLPPGALDGSPQPRHPASGSSG

GNRQLWDIVYTVTATITNTGSVMDDAIPQLYLSHGGEGEPVRVLRGFERVERINPGESRT

VRMELTRRDISNWDTASQNWVVTPHAKTIWVGSSSRNLPLSATLP

>SoG_03218.T1

MELFADVVPKTAENFRQFCTGESKNAAGRPQGYKGSKFHRIIPKFMCQGGDFLNGDGTGS

TTIWGHKSFADENFNLKHDRPGLLSMANAGPNSNGSQFFITTAATPFLDGKHVVFGQVVD

GMEEVVRKMENTKTGYRGKDMPNLDVVIAQCGEM

>SoG_03221.T1

MSEPGGGEASRHDDSSTPASVLPAGTRSRSRVACQTCNRRKVRCDVSQTSHPCSNCQRDG

GPCIVLPRKKHRHDAVDSPRRKRVDLSPSGSDLASSRGPTASDIGKGIPPRSGQTGAALE

SNDFSCSEDDDDHFISQEPEGDTILPDDSSFTYLGDKRGPRSAVYDICDAIPPQESRPLL

PPKEVIHTLHKPHQVEYMRAEGVFATLAADVCDAMIRSYFKHVHFFLPVVDAEDFLNEYH

RQGSHKMHDLLFWSMMLAAANFADADLLRRAGFASRKAMKTAMYERAKVIRTESSIRHQA

SVLLLSFWYTDPQDHTGAWYWIGIAISLAQGIGLHRYPRSNSRSQQLSPREQALRRRIWS

ACVVRDRWVSLAKGRPMRIHNEDCDTPIPSSQDILGELDFIANEARNYFLPADPAALTSL

WLRFVRISDVLGDILRLHYRVRGPDATITEIDTFAQRLQLLSTASSGISDDWDEDLSVHA

YQIDLFYQFVTLAASWLGPVTDKLRRATVAILYRPYALNSSTSLPSGSPPDWQKLALRRA

RDAATSTNCLMQSYHFLERLSREATALTSSIRRITAIIPAMQIHLSDCKSGKSLIQGLAR

HKLQLCMLVLSSLRTTYWSADVMYRLFDRAQNILSKSRTLESSSSDLTEPQLNTATYSRQ

AIRMSESEGRVPASLEHEHGSTSMPTPDVPSMADGSKQLWFTVSPQFSNVDQLLSPGFSL

SEDVFQNFFPNYDGRVYGQHASSMGDKATDEILYSNMGF

>SoG_03222.T1

MRLLSLALLSALAEAAALPACLKARLLDGCNQDLPADQSPGQVSNVRITSGDTERSFLVF

IPPGYRKDQATSAIFSYHGATRDAEDQKELDQLTSPEFNTGSIVVYPQGLDETWQGVPGV

TTDDVAFTGHILDYVQSHYCVDSTRIYATGKSAGAGFTNVLACDADMSLRFAAFAPVSGA

FYIDTLPCHPDTVKFPCNAKRDDIPFIEFHGGNDTTIRYGGGERRDACLPAVPHFVREWA

ARDNLGSSNVSTSIAPNVVKFSFGSGQKQGLVEHFFQSDIGHDWPSTAPNNDNQRAGHHV

ASYNATPIIVDFFSRHSL

>SoG_03223.T1

MSSELPPSPDGLEPRFSLPHDTPRSSKLSLVVGGVQIYIYGLDDLEEPAGDDIAVLYLAH

NRTRTYLVTEGIAHEVLYRYRTDGRKKRMQMIAVTMNMRNHGDREVDKKANRTWSEGNEN

HGIDLMALISGSAQDFKLILDYLPAYLPRFRRFHNIMLGVSLGGHTAWRVASLAPDKLEA

FAIVVGCPNLSSLLLSRLGVDARAFGVGEDELDTVPYDKLEKTMTEEQRRRWPRALAELA

EEGDKKAREEFPRTTPLLMCNGKYDSLVPAHFSESWLERRRIAMNGGFATKSQAKLFVQD

NTGHSCTKEMVALIADWLGVLYQS

>SoG_03232.T1

MRGRRPGYSDPDASNTLIIVGTIGHSKVIDDLIKDGKLDISEIKGKWESFVTKVVEKPFD

GCNEALVIAGSMPRGTIYGIYDISEQIGVSPWYFWADVPVKAHKDIFVSREQKTQGPPSV

NYRGFFLNDEQPGLTSWVAENFGENEWNGSVGYNHKFYAMVCELILRLRANYLWPTVWGS

MVYTDDPNNQPLIYAYEVVLGSSHTEPMMRAQNEFGKYYKSAWAYNLNNKTIDDYFRVGV

QRAKPYARNSLWTMAMRGTGDTAIEGLGIDVIVKMLETLVHNQQDILKKGLGEEDLTKVP

QLWCLYKEVQGYKEEGLEVPDDITLLWADDNWGNIRRLPLKNETDRVGGAGVYYHFDYVG

DPRDYKWINTIQLEKTAEQMHMAYSRKADRIWIVNVGDLKPLEIPISHFLDLAYDAETWD

VDSTEDWIKAWIGREFGGDNVDEIASIMIRYGMYAARRKYELIEARIYSVLNYNEAEAVL

AQWNTLEEDAQTIYDGLDEAYQPAFFQMILHPVIAGRIVHEIYVTGAMNNLYAWQKRNAA

NDKISHARALLYDDANLTRRWDKVLDGKWKHMLDQTHLGYDGYWQQPMANTLPQMVFVQD

TITSLAGHVRVGIESSNASIPGDDQFHPNTGATLQLPPLDSFGVASRWFDVFSTGTDNCN

WTAATDAEWVKLSRSSGTVGPEGNDTRVWVSIDWDKAPAAPNSTIIPINITTPCRKFEKY

AYKEPQILLPVNIRSLPKNFTKGFVESDRTVSIEGPHYQNIAKPGGGGNSSSNITYHTFK

NYGRTFAGVGLWPMDLDKIELGEAPALQYDMYLYSNHSVANVTLYLSPTHNYLSDLNPLE

YAIALYPANDSTPQDPKRVRFVGETQGAGMPAQWGHSVSDAVWGVRSNTSTTGFNVTYEG

AYTLKIWCLFPSIIVQKVVVDLGGVRPSYLGPPESFLMGRDEVGKYNMSSFLDSPDTLGS

VILGKKEQMGALGSTDNDDAAVLLSPGSVWSVTLAAILAMYLLL

>SoG_03236.T1

MVAFSTVLAACAVIAGASASPAADLGKRQVTPNREGYHEGYFYSWWSDGASPVTYNNGPG

GSYSVQWQRGGNLVGGKGWNPGTSRSISYSANWQPQNNGNSTSFKYLCIYGWTRNPLVEY

YVIESHGEYNPGGQAQQRGQVQHEGSTYMLYESTRHQQPSIDGTQTFQQYWAIRQNHRQR

GTVDMAVIFRAWENAGMRLGNHYYQVVATEAYNSAGQASVTVESPP

>SoG_03270.T1

MLASPTPAPAGPVRRTSQRQALRRPASRSMLTRSESQQALVSGGAGPGPAIETRANQSKQ

YRDDSSEDEIPVPMKLSALTKALLNDPDEPVARGPSPPRTRRQASVLNSSTNSAQSVEER

RHLRSRSNQPQDSSRMEREASPGKSREHSPVRKRVVRLSRDSSNLNHMGPISTKRRSTSV

SRGMQKDSNRPPSRPSSRTESHGEEKSELQRDVNTPSQAPLRVVRISSSSSVKRRLAARR

SSGLNPDTSGADGSALDLGQDYHTRKEQVDSAARIGSSASKSSGSRYPSSSLRHRSEENP

ALHSSMRIKRVGKTPGGFLSGPARRGKRRQSDEDGGEEMGEAEPLFSSHERGVPAGEDGG

VMSHVRNFNSGSPVSSSAAARASHRRQASQADIQLGSRQPSPRGLELSRKDGILESLDVG

FPSPKGIEMVVRTHGKDQSDARRSIRPELPSNHDQENEVPGSWRRSKPSVDLIMEKIPSR

PQQAEVPAPKPISSPERKPLAAIAKNTPSHAVPPPPPKMSVLEAATSNAGAAATTQAKQR

RNVLRVNGKVYTRLDCIGRGGSAKVYRVSAENGHMFALKRVSLENADEVVLRGFRGEIDL

LTKLNGVERVINLFDHELNSEKKVLYLLMEMGELDLGSVLKARHALDESKFDPVFVRFYW

KEMLECLQSVHHYAIVHSDLKPANFVLVRGRLKLIDFGIANAIADYTKNVHRDTQIGTPN

YMSPESLLDSNSPANNSGARMNGLPKLMKLGRPSDVWSLGCILYQMVYGAPPFGHIANAM

ARCQAIINNNHTIEFPLHAPCGTRVPNSVIKTMRGCLIRDPSYRPTVEQLLKETDAFLYP

RELPNDSLPVDREMLGRIVQDAVRKYIDGQDHPDPRKRPNREDLRKEFSDDYWHSLRKAA

VAKGVMW

>SoG_03293.T1

MMKWFWDNYTTAAGQRNEISASPLRASTTQLKGLPPALVQTAEADVLRDEGEAYARKLDA

AGVPVTAVRYNGMIHDYGLLNVGSQVPAVRSAMLQASEELKQHLK

>SoG_03297.T1

MTLKAFILLWLSLLSLEASAALVRRAEWTVSLDPIQAVIAQDSEGAFSIQCQTSGKTVVT

ASRIGLSTTQGDVGPALQSCDALPQTEDLVEYTIPTGRTTSRSAKYRTQSFTCKTTDGKD

TQVDVTIGNDGCGFRTKVPDGRYNVTAESSTWTFGQNGASFLIDDPGNTSYEAEWIQSDV

STGLSGSRRVMMPYLADAGASANGQWVLLVEGDVDGRWQGSQFAHSSGSLDYTFELAENG

VVQVEQSVFTPWRIAGRSSSSWVGALILMLTVVGDLQTLYKNHFDQHHSPPSSIEDPSWI

RPGNVAWHWLTEPWAAGDLNRLKQYADLAALEQWPFVLVDEGWKSDDIPQLVQYAAAKGV

SVLIWHISDMKNWGVKGVKIDFFLSDLQATHKQMDYILEQTALAQLMVNFHGCPPQRGRQ

RQWPHLLTVEAVRGDEAPDSSALRQTVIPLTRNLIGSMDFTPSLYTAVAVHWSEQRQAEQ

QAECSVGCGLAKAVVFESGWQHPGDKPGVIESYPLAVRVYKGLPAAWQDSDLLAGSYPGK

AVILGRRSDALDRYYFAGVFNGGEQTIELKPTNLPGARTFILDVVHDSPGDDTNRTAIER

TVHTGVSAGSSVSIPVKSNGGFTAIACEIAGSDGGCLPEA

>SoG_03302.T1

MPDATRADMVQRFPRKPRVFILSDISNEPDDAESLVRYLLYSNQFRTEGLVATTSTWMRT

KVCPQDMRKIVDAYAQVVDNLNSHAHPEDPYPTAEYLHSIIRSGLPVYGFEAVGRDVPLS

EGAELLLERIRDASREPLWVLVWGGTNVLASVVEKIRAEHTPQEAAALRAKLRVYAISDQ

DDTGAWIRNTFPDIFYIASVHAWNQYGLAAWTGISGDRFYGFDQGGPDFTKVSKEWIKEN

IQIGPLGTAYPDYMFIPEGDTPTFLYLIQNGLGVPEQPGCGSWGGRYQLTDASSSGLCSR

HYSDCSDVVVGADGKTYKSNHATIWRWRDAFQNDFAARMQWTLSPSLPSANHHPVITLNG

VQGSEPLYITAEASSTVTLNATGTYDPDGDALTYKWWHYREPSATQWQAFFEVEEVKIQK

VGESDEVVAVTLPEPEKCCVELLSRDPLRLGQGLHVVLEVTDSGTPSLTSYRRVIIQTTN

PELRGGNGGGGGQAIGDVMAALTA

>SoG_03304.T1

MDPSIDNPDSWVEYAEPDTELQQLLDSGVQPRFTGPVGGLAGLAKHREEFDDIFLRDAAE

ARDAAGDAVREEELFIPTRDGATVRALVYTPAAADSDAAAAGRPLAILIHGGGFIVGKAE

METPPCIAVATSYDCICISLEYRLSPEVKFPVAYEDCWDAIIWISENAASLWGADLRKGF

ILGGLSAGAHISIPLSHRARDEALTPPLTGIHLGVAPSLMPQALTPRYAPLYRSREQLAN

GMTLTAESTKLYDEAVQPDMASPYWSPLLWPTGHAGLPPTFFQVCGADLLRDEALIYERE

LRVENGVRTRAVVYQGLPHVFWYNFPDHSQSKKYLDDTRRGYGWLLGKEDF

>SoG_03307.T1

MPGITAMTSRIGALLSVITVAIAAKDIPAAELVSSKVHIVPGPLKVTELTFTVPLDYDDP

DGKTITLFGRSVVNYEVPIVVSDEEEEEPSSLPWMVYLEGGPGFGNREPQNHALTSTALE

RGYQVLYLDHRGTGYSTPVSTRMLNQLEGGEDAQVKYLRLMRQDNTVRDCEAVRKLMMKG

KSGNAAKWSIFGQSYGGFVSLSYLSLHPEGLREVFLTGGLAPVGRTADDVYRSLYPRVMK

RNKAYYKKFPEDIENVHQIARHIESEDGAIDLPAGGSLTVGRLMHLGIDFGGAGGFDSVH

TTIKNLKTALDQFGFLTRASLTPIESFTTFDNNIIYAILHEAIYCNGPNDTSNWASDRVA

RELNLFSWLSSGNSSASANTSEPLMFAGENIFPFFFDTYPELIPLKGVANKLAEVDDWTY

IYDKEQLAKNEVPVYAATYVDDLYVDYEYSKETAALVQGVKTWETNAYYHSGLRTNTAEV

LAALWNLRDNVID

>SoG_03320.T1

MELVTPARRHVSPGPASSGSEGRSTTGGNGQPLVKRRRMHTACKICQDRKTRCDGMRPTC

STCQRRGVAADCVYDSAAVPPASRASSSFAAFEERLGRLEQARVPDILNHDQRGNAVYVS

PRDDARSTIAPEVRVSAIGLPTPQTVQESGDVDGLATVSSMDDGDCLYGASSTIALTRFF

RGKAAESHGTPVDASAVEATHRTTSEKTSHGPLPELVYESDVGAALFPSRPMADDFIACF

WEFVHPLFPVLHRTSFMYRYQQFWTEQAENSIPKDHASHNDYAIFTSTLNIMFSLGCQFS

TMVPAHRCMSLAQDFYQRARRLYNYEVLDSMSIPLVQLLLLSGVYLQSAQNANRCWNIIG

LAIRVAQGMGLHVERPRSRQTQLDREMRRRIWHCCLVLDRLLSMTFGRPVMISERSGVPI

PSLIDDEFLRRQGEGVQPAGTPSGLGLLAYSSRLYVILDDILSDFYSSNPGCNVMEMTPD

DINVQKVLSEIMSRNRRLDEFLGEIPVYMRYGSVPISSRSTPQEKSVEIQRQILYCRFLY

TRLLLFRPVVYLSTKYPSLFLSQYSTKASATMDDQLVKLVCDSSVHSAVELISAIHRNLE

HSHRSSAWHTIYFTFGATVLLLCTQICPAVGNDVLQQTFEEAWERAQAILDYYKLQRPVA

RQSIQTLKALRESIQRHNSGPVEFDSPAQRQLAAQMDLPEFDLLGTGPGGFDEWFNLDMG

DFGGFH

>SoG_03329.T1

MSSQQLSFSLRVSSSVKTVHLLGSWDNYVGQLPLSKDKSSSKSGSWKGTFKFQNSTLEPG

QRYWYYYIIDGYHVAHNPSEKSTTEPTTGRELNILDVPNGSSSKGSSSSSSRHHSSSHKS

SSSSKHSSSSSSRHHSSSSSRDKERERDHHRSSRSSRKSLTVDIPKGRPLSTSQIKAPKP

VSPTATKKILESDYRYYDDDEIADLTARFGYAVIDEDDVVTNFSNSPMSSAGSSLSSYES

DSSSGSPSSSLSGYSTPGSDLSSCTCERYGITRKGDRIKIDCRGQYCGYEDSSDCSSDSG

YEDRYDSPVSHGSSRRNGIIVS

>SoG_03330.T1

MAQVVGRSNNRYPLTENANRVNTAGYGHGGQSSKPRHDYIVVTSNNTPAPRNNYNIPVNH

PAAQNPAQNPAQNPAQNPAQTPAQTPAPTHDAAMDSRAMSELKPPSDSEESKRHSQASYS

SNASRSSRYKSHIGPWQLGKTLGKGSSARVRLCRHNVTNQLAAVKIVNRRMAYLVQDSSL

AALSKWDKSLPELDGEMRVPMAIEREVAILKLIEHPHIMKLYDIWENRSEIYLILEYIDQ

GDLFTYINSHGRLSEEVSLYFFRQMISAITYCHSFNICHRDLKPENILITADMQIKIADF

GMAALHQTDNHRLATACGSPHYAAPELLKNKHYKGDKADIWSMGVILYAMLSATLPFDDP

DLRVMMGKTKRGVYEMPKGLSHEAEDLIYRMLQTNPENRITLREIWRHPLVQKYSYLDDL

GENSGQPPDTRKGFQYAPVPRQSLDAQLIRQLRSMWHMFSEQQLAQKLTSNEPNDQKAFY

WLLYNYRDQQLEDFKPELAHSMSDYHHLQPGVWKKRVSTCEFSQPRLDGHGRSVSRFTVI

SNAAETEVGTVQSYDPYRGSRVLHPSQSQVSQTKVTVHRDAQSPGSYSAQASRMRSGSNA

RHRRLGSVRSSINGRPASSRGSVTSMRSARHNTPSARGPNLRHKRGVDFSHVRDRANSVG

RAQKASTRMASLALDEVPSLAPVEPLPEVPRVRESASPDLPTLTTGQTHPKAVGGPAPLA

VAPDESLALNEELRHFSYNIAKDLDEAFGSSLIEATSFGGSLTDSDGRTRETSPLSLTFD

SMSISTPPSEVSVKPWDTRPLPPLPNQRALSSHPVVSEAPVVAKAEVAVRVVPSSLQPPQ

ADRRIVSAPAYPQNSAVKPTSLPSINENAGAVFNSQDKARIVSAPPHTPPKRRAERLASA

EYLSKVEHSIRVVHSPTAYSPVKVPAPLNVRKASGTEDFGRSLHQKLAYHADSDEPAHES

SSQSSQDGGKQKKKTSWFKRVSKAESETRTREAQTTQVEQPLSPVDFDDILRPDHGQLVA

TKKKSITFSFWKGGKNKEPKMKIDETPEQSRNSRGQPAVLKKAEPSNWRDSRSSSGMRVI

EVKQNWLARLFKVKPAVSHICMTLSRKRARQEVAILLREWRKYGIRGVSIDKQRNIVFAR

VAAKNHLNMKEVEFAAEIIAVIEHGKKQPLSIVRFTQERGAASSFQRVVDTMKAVFDSRN

LLVADKNRRKMMIKTLNSHE

>SoG_03331.T1

MFASAMIEAAMAVATLVLRATSFLFVRHHPIGKLYLNRIYLSFLVYVSSLAGAIAVSRRR

RQPWWAILTLGLIDKRRVRFSMATALVNCLSMAAVLDFVYRGEVLHRARDLSFSRVGYVD

DTSSRIVLRAPSESPAFVEMVVSLDGSRPVKKQVVEVSEAGDFTATFHFRDLRPDTEYLY

ATNSSHYGSFRTAETHPKTWSLAASSCIKPFYPYNPLSHGLRIGGLEHLSNHCAGEPFDM

MLFLGDFIYIDLPVPHGWSREHYRTAYRQVYASPNWSPALRNIPWVHVYDDHEIINDYHA

QDGERLYEPAMAPFHEYQGSANPPSAFGPGKTHFTFQKGDVAFFVLDTRRYRSDQALADD

ERKTMLGEAQRADLENWLRTEKKWKVVVSSVPFTQNWQGPDKNDSWAGYLWERNYLLGKM

KATEGVVILSGDRHEHATTVFPARRPSEKPVIEFSTSPLSQFFEPFSRHHREVDPSTDQS

VYNWPWGNSKFGVVTFDTRDPRTLKLRYKLVVDGELNWEYDWSVSR

>SoG_03333.T1

MKVDLKLSLGLAATQAIGAVAQSCPSIHIFGARETTVSPGYGSAGALVQQVQSAYPGATA

EAITYPACGGQSSCGGVQYGDSARQGTNAVASAVNSFNQRCPNTKIVLIGYSQGGQIMDN

ALCGGGDPNSGISSTAVPISSSAVNMVKAAIFMGDPRWQYGLAYQVGTCRAGGFAARPSS

FVCPNASKIKSYCDSQDPYCCNGNDGNHHQQYVSIYGSQALTFIKSQLDASGGGNNGGGG

NNGGGNNGGGNNGGGNNGGGNGGGGTTCSALWGQCGGTGWSGPTCCQSGTCKSQNQWYSQ

CIN

>SoG_03338.T1

MGQVSSQAAEFGIAMLAVRYLDETDEVAALYNVEATEVFLDSPAPTVADVETPPERCVGL

AAQTRPGRSETFSSGERLGHLRSLTLTHLPANSRLCMYNEDDVAARDILFPILDSHGEIL

MRYYLEYMCNWFDLTDSSRHFALEVPRRAMTCPTLLNAIYALCSRHMSKRRPFDEFASDR

YHQECLRELSNIPRDSDALMNDDLLAATILLRTLEEMDVPLLGEDFEGHLLGIQVFMNHH

ESSLREASSLRRASFWIGLRQEVTMAVASQRSINISLTRTFIDTSFSPADADTWANRVIL

HTARVVEFCFGDEPQSVAVYRALQEYEDGWMRSRPSSFLPVAYSPRDPSRGEVFPRIMYL

NHAVVIGVAHASLARALLICYDPTIPKVGPARLAAQEQQEEEVREQIRELCGVALANPAT

IPAMITASLGIASCGDRFKGEEERQALLEILVKTQSPPDLEAASKKMASRKLKIGVAGLG

RMGKRHALNFFQSVPRAELVAVSSPDATEREWAQQNLAPAGVLVFEDFADMVRLEGLEAV

CIASATAVHADQAICAIAAGKHVLCEKPLATTAEVKVVDAAAKRRDLKVMCGFSRRFDES

YRDAYTKIKDGLIGRPAVIRSQTCDTLDPSGFFVAYARFSGGIFVDCSIHDIDLALWFFG

EDGDGKVRSVSAVGINAVEPGLFKHNDRDNAVGVIEFTDGRIAYLYCSRMMAAGQEDTTE

IVGTKGKLTVNAVPTANLVRIHEPGGIRHELPQNYWGRFKNAFTREAVEFADCCLDDLPV

PVKLETAVSAVKIAAALQESMITGKKIWFDGAGNVESRAHL

>SoG_03341.T1

MVSLTDGQKAALSAEAVEFLTKLRHPPLADLISGLLTMPSRIKGLRETFASKLADGERKL

IEDHGLLISSVEIAGVPVVVIRPPRVDPSKKNKIMLNIFGGGFTLGSPRDRAALTTAAEL

GVTVYSPGYTRSPEARYPVARDQCLAVYRELISQGPPGGDGPIHPFNIYAQGSSSGGQVL

LSMLAKAHQDGTPLPTAGMYLCTPAADLSGAGDTLVSNGHDRDVLPASFLRSMASQNYPP

EEGIDGKDPLYSPLYYKGYDKTFPRTIITVGTRDLMLSSGVRLYWVFRDAGVEVELLVFE

GMWHGFNWDPNIPEAKQARAAVLAFLESSDHDAKVDLVL

>SoG_03346.T1

MSLIPFHPQEGREIVLRHRNALVVRDPTSHRLEIRGLSNCPTCHRPWQRSSSPDRHFDRS

AQLSRDPYVDPNYFRMLRPAGDDFIPDRPPSSPVRRLAEPALPTNDNLQSSYVEIEDEER

RGWRSSSSQAPQTSSKIKRESFSQNYFNDFFIEEGILGKGGKGVVLLVQHQLDGCPLGLF

ACKRVPVGDDHAWLERVLVEVRLLAQLAHPNLVSYRHVWLEDAQLTRFGPPVVCAFILQQ

YCNSGDLHKYVLGEQNRETSTEELKAQMRRRSKGQAELPRMDNGKPPLPPEEIYSLFKDI

TSGVAHLHAAGYVHRDLKPSNCLLHREGGFITCLISDFGEVQPSDAIRKSTGSTGTISYC

APEVLQLDASGKYGNFTFKSDIFSMGMILYFMCFGSLPYQSANSIQEELEDVDELRAEIS

DWQGFEDEKRLRPDLPSKLYALLKQLLSVNPAQRPSANDVLKALTSEGRFDGWTKSGRAA

TPSFGLQRVQNLDSPAAPSTPVPEPRKQSRRSSETQDLNPAWPQPALSEMGNEHDNMHGL

RRRNNAMTLTRSHADFSPVLPANISEDPSIDGHDGPLISPLLLPSPPSTSLLARLSRTAR

LGAHRLSQITGIDSTTLEYALRLSLFLIKISSVAQPCWPFMPTLQVAIPLVLMAALDLRT

SPSQAPHTRRSGVRRRAVAMSAVLLALHVSLLWLARSRDFLCIAVQHDGWPDW

>SoG_03349.T1

MHTIPPPPEAPSPRVGSPSTTLHGNLWIFSGRGGLEMQPIEECGALWCYDVEASKWTLIQ

PVDPLAPHPAGRSYHCIASDKRSKIFVHSGCPESGRLSDLWVFDVNTRSWSSLPSAPDPA

RGGASVAYAHEKVYRMNGFDGKTEQGGSLDVFDIFSQSWSTTTFNPDGVHGPEARSVGTL

LPIEVSGRTYLITMFGERDPSSLGHAGAGKMLSDVWAWDIEQSKWQNVVASGAAPEPRGW

FDADVLKDGEEQDTVVVHGGLNEHNKRLGDVWKLSFH

>SoG_03355.T1

MQHHHAPYGYPSPPASPSSYDNAKFESQGYAARHYQPRCVPLAPEERLGKLLQGKLHLTD

ILGTGAYGVVYSAYDIDTGSRYAVKCLSKFNPDGTLLERRQALYQQREIRLHYLASGHEN

VVSMLKIVDDVDCIYVILEYCPEGDLFLNITERGRYVGKDDLAKKIFLQILDAVEYCHNL

GIYHRDLKPENILVTDHGDTVKLADFGLATSDDRSEDYGCGSTFYMSPECLQPNSRKPFY

RCAPNDVWGLGVILVNLTCGRNPWKQASYQDSTYRAFARDSTFLKSILPLTDELNDILGR

IFNPSPEHRITLPELRNRILNCSQFTVPAMCVSPPTPPVSPDHITAYVAPEEAIVDDCDY

DSPLSPASTNSDEGSLTSSAPTIDDLDDEVFIDCQPPQADLGQDMNTVAFESDMSEGSPT

FHAQEFMPQQHYTGPVPAAPIVVHSQPTNSQPSMQAQPHVSVQAPCQPKSYFHFWDVVKY

VQQTPMLPNHGPFHQQVSFFPVQGY

>SoG_03359.T1

MTSGDIAIVGMACRFAGDAKSPEEFHQMLQKGKDAWSKIPNDRFKAESFKHPSNDLSGTM

VCEGGYFLDQDVSQWDAPFFTCSASEARSYDPQQRLLLEVAYESLESAGIPIENIANTDA

ACFVGAFSEDYKIISSRDIHAAPKYAKTGTALSILSNRISWFYNLRGPSVTCDTACSSSM

AALHLACETIRSDANPTRCALVGGANLILDPDDQCGLNALGFFSPDSRCFSLDARANGYA

RGEGLGMIVLKHIDDAIRDRDPIRAVIRGTGLNSDGKTAGITLPSAEAQARLIVNTYKMA

NLDPADTQYVELHGTGTKAGDNAEVRAISSTIAAENRKQPLYCGSVKSRVGHTEAAAGMA

AIIKCVLCLEKGVITPNLNFATANPRLRLDASGIIVPTSNIPWPECEVRRCSINNFGFGG

TNAHAVIDDAFNYLRLRGEKLQQPADEAGSVPQQPRVFVLSAPEQAATTRQRRAHADYIE

SRGPESRLRGLAKTLGENRSVFQWRHAVVASSAQELLASWRDDTIKPAKAGPSPSLGFVF

TGQGAQWYKMGRELLCFPEFVRSVRLSAEQLASLGCAWDAWTELTQPTCADDSKVNQAEY

SQPLCLVLQIALVDLLAHWGIRPTSVVGHSSGEIAAAYAAGALSRSDCLKIAYHRGIVSK

LAQVRKPGGCMMAVGKSAETVGALLASSGGSKDVVVACINSPESVTLSGDKPALEKLKVH

FDAEKIFCRILQVDNAYHSPQMQSVSADYLEAIKSISPLVAATSVSFYSTVRGQKISLSE

LTAEYWVENLCSPVRFVEALDDMIYATGAADKQTKAKADAPGMLVEIGPHSALAGPIKQF

KANRHALEHVAYISMLVRDKDASFTAVSAAANLWSSGVAVKLNKVNDVDATVETLTDLPN

YPWNHSASYWHETRQSRNHRQPEFARHDLIGARIDAYNPMEPVWKNYLKVSNLPWLRDHQ

IQGDIVFPAAGMICSAVEAARQIAATENSGEAVSGFELRELSVARALVIPDDDMGIEVHV

NLKRRKTGMGSGVGAWFEFSYYSCQDNDTFVEHAAGLLQIQYRKKESEVDQGKEAREETL

AQNRRWESKRAMCLDKVARSSHYNFCKEQGLLFGKTFQGLRGAHQNGLTVAFDVGIVDTR

ACMPAYHESDYLLHPATLDAVFQAMMIAVPRMDGVEKQVWVPTGAASMVISSDITRSHGA

ILQGLAESSMTGVREMVASVLVKDTQDQQAAPALVIESFRFTGLGPTQKSTQSSDVLASK

LYSAPVWKPDVDLIDTQTLRAMSGVMGDADGMSRYCSMANSLLKEMCKSALSHLSPIISA

SLPPHLLKLADWMHRQYSHQQTGLVIPVDSPDICQRAKLPALPDLSGIQSFKEHYPIDGQ

LALHVYRALGAIYAEETTPIATLRQDDLLNKAYQEVYGLQIHVELMKTWFELKAHKQPAL

RVLEIGAGTASTTLPVLQHLSQTGSDTPMFSSWTFTDISAGWFESAKALLADWKGRVEYK

VLDIDVDPLEQGFEAGSYDVVLAVNVLHATKNIGTTLQHCNTLLKPGGKLVLGEYTNPND

LANFVFGIMPGWWAAEDDRKAGPLLPQSQWHASLLSAGFSGADMSIADSDDLAAHRMSTI

ISTKPCQLPSRSMAVVIPQVCSDSTKKLAAEICSQLKQQHGHEGVVMTLAVAALKAKDKS

VVSLLDYEAPFFADTSKADFEAVQSLLLQSDELLWVTRSELHETPSHPMKRMVSGLMRCI

KTEDSSRSLYELHLSRPSAEDVESTSAAVCKRLQTIWEAAGDDGVQEMETEEKSGCFYIP

RHMPCKAMNDSLARATQVVAPRAELGALMQENRPLKLNIAHPGMLDTLRFVDDERALEPL

QDFEVEIEVRACAMNFLDIMIAMGQIQRQVLGHEACGTVSRVGSKVSEVSRGDRVVFVGP

GSMRTHIRAHESVVQKLPDGLSFSEAVSIPIAYATAYRSLIEVAQLEKGESVLIHAAAGG

LGQALIQIAKMLEAEIYCTVGSNEKKETVVSLGVKPERVFSSRDLSFAKGVKRMTDGRGV

DVVVNSLAGEALRQSWNCLAPYGRFIEVGKKDILGNSGLEMQQFVNNTVFAGVNLEAMMI

DQPVRCRKVVAKVFKLFEDRLIDFIKPIVVRDFTEVESVFRDMQRGAHIGKLVLEVTPSS

RVPILPRAEVPLTLDADATYLLVGGLGGLGRAQAVYLADNGARHIAFISRSGDAKPEAKK

LLATLNESGVEAKSYAGDVANLDDMRLIIDDMSRTMPPVRGVIQGAMVLADSLFHRMSHE

QWEAATRPKVQGTWNLHELLPKDLDFFIVLSSLAGIIGSVSQGNYAAGNTFQDALVHYRR

SKGLAAQSLDIGVMTGIGYVEEHDDARARTSQLRVTSLGEKQFMHTLRIAMAGTVDGNEA

PMPQLLVGAGSGGIQQLIKEEDPSSDFYWLRLLSAFSYLQQMDIQGQDLESTEGQDEASK

LVAELKLCKSMDEANDVAQRLLVSRIAKHISIDASDISTSKPIHAYGVDSLVAVELRNWL

SMELKSELTIFDLTGSDPISEVSKKIANRSKLVTEVKTSD

>SoG_03371.T1

MPRRAASPAASEPELDILGSLYPGEDEGSNQGFAPDQGFEVDGILDGADGGDDGDEAFIA

LQQAASNRKASNLKGRTVKKGGGFQAMGLNANLLRAITKKGFTVPTPIQRKTIPLVLDRK

DVVGMARTGSGKTAAFVIPMIERLRAHSARFGARALIMSPSRELAIQTLKVVKELGRGTD

LKCVLLVGGDGLEEQFGAMSANPDIVIATPGRFLHLKVEMNLDLSSIKYVVFDEADRLFE

MGFAAQLTEILHALPPSRQTLLFSATLPTSLVEFARAGLQEPSLIRLDAETKVSPDLQSA

FFSVKGAEKEGALLHILHDVIKMPMGTPQNNQQHNGDKSSKKRKRGPDVGPGKPTEHSTI

VFAATKHHVEYLATLLREAGFAVSHVYGSLDQTARRIQVEDFRFGKTNILVVTDVAARGI

DIPVLANVINYDFPPQPKVFVHRVGRTARAGQKGWSYSLVRDTDSPYLLDLQLFLGRKLV

LGQSESNPSFAEDVVIGAPKRDLVELQVEWLNKILEENVDISSLRKIAGKAEKLYLKTRN

SASSQSAKRARELVASTGWSQLHRVFGEDANNEQAARADMLARISGFKPQETIFEVGHGD

KGKSRAAEVMKELRKRVVPKGRNQSTEFDKNNDDNSEDEDSEDVDGMDVDTGVDLDDQEA

AEWEDDDDDGLEVTVSNVNGKAKGRTDWKDSEVFMSYTPRTVNAAEERGYGVHSGGAGSS

FIEAARDATMDFTNDETAKGFGAPTRAKMRWDSKSKKYVSRDNDEDGSKGARLITGESGV

KIAASFQSGRFERWKRANRVGKMPNVGETERPGAGQIPSGVRYKHKQEKAPKEADKYRDD

FEVRKKRVGEAREKRIGRFRDGMGNKKELKGADFIRKARAEKQKKIAQAQRQRRK

>SoG_03379.T1

MTDPFNTKAMTQALPATVNPHPDDKPWVAAEAFDYAAMTVAPDDQKWDGHARVYEWKDEF

GDVGPEMPELELELFGSPEERNDKAGLDFSTITSIEVQQEGPVKISPIKSFDSAGLHPAV

RNNVRLSGYCVPTPIQKFTFPAILSGHDVIGIAQTGSGKTAAYLVPIISKLMGKAKKLAA

PRPDPENFREGVDEVVAEPLVLIVLPTRELAVQVFNEARKQCYRTMLRPCCVYGGTPLRQ

QIALLNKGCDILVATPGRLTDLIYRPNVLTLRRLRYTVIDEADELLKDDWKENMQTILQG

GEQDVGNIRFAMFSATFPKALRDLAKEYLSASHVRFRVGRAGSTHKNIKQIIIQVEPREK

RARLLELLADMPGVRTIVFTNSRASVDNLDDYCFNVGLPCTSIHSERTQIEREAALRAFR

SGKAPILIATGVTARGIDVKGVQHVINFDMPSMDHGGIEEYTHRIGRTARIGWRGVATSF

MTERDEPIADVLTRFLLETEQDIPDFLEGYAPVGEARNHLKFETESDYDEQEAAGSGDAP

GGAWGADSNVAVPASGGGWGTEAPAKQEPSSGGWGDAAASTRAAADGGWGAPSASAYGGW

>SoG_03383.T1

MVDIASRIRAKLSRRRHSSTAPSLASSRSAIDEASSVTSQSQGHSLGLSCEGSSLGDAAT

TKQGNPIVAASAASAQDGVSKDDKGHSSERTWDADGYDGDQAIANAQSTMAPKASSDAAS

DPGAAYAGGRSDNGTSPRPTVASLSSATVPPTNSGNSPPNGDTPATSPHQSRAPRAMSTA

TSAETMAAIGDRRRLASSIVQGQAQPTSNLSSIHESSNPASPSNREAAIHEASEDAGGDG

DDENEDDSDYPHRQGATLRGLANAACAQGSSTPNSVPTPTPGFLIRSPTGSSSRPQTISR

QQSLVSNRQSALVPSFVLPTLTTMGDGVAPSDAPMVTRKIWVKRPGGSATLISIREDDLV

DDVRDLILRKYANSLGRTFDSPDLTIRIHPREQQHRDRTLGPEEHMCRTLDACFPGGQTV

DEALLIDIPRRTPKASPRAPLHPYYADDGRPSEAGEGYFPPVNSVPSPHLPLAVPVTASS

HNIPHSISVLGTGQIPPIPSPGATSSRRGYRDRPERPRLGRTHTSSPTVLGVGAPNPAPA

ALPAVNHGTQQFLTRPSRSRTHSDSSDQPGHPPTAPPLPTSPGPEPIARTATPPLRTQSP

RPGVRARRTKKAAPEPPTVQPGAFSGGVPPINVLIVEDNPINLRLLEAFVKRLKVRWQTA

MNGRDAVKKWRSGGFHLVLMDIMLPVMNGLEATREIRRLERVNSIGVFSSSPGSLPDDPN

GELAEQDRLENLALFKSPVIIVALTASSLQSDRHEALAAGCNDFLTKKVMEWGCMQALID

FDGWRKWKDFSQSGEDEASKKTTSAKPKTKKNRSSLTSAA

>SoG_03385.T1

MAPLITSTYTADPSAHVFNGKVYIYPSHDRETDIKFNDNGDQYDMADYHVFSADSLSPPS

EVTDHGVVLKTEDIPWVSKQLWAPDCATKNGKYYLYFPARDKQGIFRIGVAVGEKPEGPF

TPQPEPIKNSFSIDPACFVDDDGQAYLYFGGLWGGQLQCYQKGNNVFDPEWQGPKEVSGE

GVAAQGAKVGKLTEDMLEFSEEVQDVIILDPEAQKPILGDDHDRRFFEAAWLHKKGDTYY

FSYSTGDTHFLCYGTSKSPMGPFTYGGRILEPVLGWTTHHSIVEFNGKTYLFYHDCELSG

GIDHLRSVKCKEIFYDDDGKIKTTTAD

>SoG_03388.T1

MMASRFSRALPRATTITARSAGLRAPLASRFARYESTAADGEGKVQGAVIGIDLGTTNSA

VAIMEGKTPRIIENSEGARTTPSVVGFAEDGERLVGVAAKRQAVVNPENTLFATKRLIGR

KFSDAEVQRDIKEVPYKIVQHTNGDAWVSARGQNYSPSQIGGFVLNKMKETAEAYLSKPI

KNAVVTVPAYFNDAQRQSTKDAGQIAGLNVLRVVNEPTAAALAYGLEKEADRVVAVYDLG

GGTFDISVLEIQNGVFEVKSTNGDTHLGGEDFDIHLVRHMVADFKKTSGIDISNDRMAIQ

RIREAAEKAKIELSSSLQTDINLPFITADSSGPKHINMKLTRAQLEKMVDPLIQRTIEPV

RKALKDAGLSAKEIQEVILVGGMTRMPKVGESVKSIFGRDPAKSVNPDEAVAMGAAIQGA

VLSGEVKDLLLLDVTPLSLGIETLGGVFTRLINRNTTIPTKKSQVFSTAADFQTAVEIKV

YQGERELVKDNKMLGNFQLVGIPPAHRGVPQVEVTFDIDADSIVHVHAKDKSTNKDQSIT

IASGSGLSDAEIQQMVEDSEKYAEADKDRKAAIESANRADSVLNDTERALNEYADKLDKT

EADGIREKLASLREFIAKSQSGEGTATAEEIKEKTDELQMASLNLFDKMHKARNEQSSGE

QQPNEGEQKKDEPKA

>SoG_03397.T1

MSANANSNSKPRRSGDERTPLLNGSSSPRDDDNGGENHYPTQSSDTMVFLFNSEHTPGKD

HHNIAIRAFAHSWHVTKVTLLSNYVNFLLVMVPIGIVAGASGWNPTAVFTINFFAIIPLA

AVLSYATEEISMKLGETMGGLLNATFGNAVELIVSIVALKDGQIEVVQSSMLGSILSNLL

LVMGMCFFFGGLRHRGSSGNGTEQTFSSVMAQTTCSLMTLSSASLVLPAALYAILDQSDS

NEDEKNKSILILSRGTAIILLFLYALYLIFQLRTHSNLFDAENQNPEEHNEETPNLGPVA

AVMVLVVTTLLVAVCAEYLVGSIDDLVETAGISKNFIGLILIPIVGNAAEHVTAVVVAIR

NKMDLAMGVAIGSSIQIALGVTPFLVIVGWIIGRDMTLHFETCKSLYPHPPEEDNC

>SoG_03400.T1

MLFCVAILPAFSLLFTCALALLPTSYDVVWDKPGGKGSADSMPLGGGDVGLNTWYENGEP

RGLGWSRSLTDAGTGTILMYIAKSGTFDENNSLLKLGRVRLSFDPNPFDDESFEQRLVLN

DGYVRFTGRENTTAKIWVDVFNPVVHVEVDSPSKLSVKASYENWRYQDRAIVNEERNQGS

WGIYTSKVPDGTTYADKIAYHESGVLMSHRNEKLDLWDFQMEQQGLREHSEKMYNPMRNN

EFGIFVHSEQLKPGNLTSGHYINTDYKSWNLVTKTPGKSFRIDLTIHQAQTASHDEWYKG

LKGVIKSAVKNTQDASISWWHDYWSRSYIIINEDRGEDDAGFQVGKNYQIWRYLMGCNSR

GEWPTKFNGGLWTFDPVFVNKYRPYTPDYRRWGGGTFTAQNQRLLYWPLLRTGDFDTMRQ

QFDFYKRITPNAVLRGQVYQGLDAAYFLEQIDNTGLSNVFEYNAQYYDDDANTPRPSFFP

DGELWNVWLNHVQDTANEFADMILQANLYSGFDVKPYLEFIEYQLAWFDKFYTKQMQMRN

PWPMTGINGNEHLVIYPGSGAETYKESYNPVSTLAGLRQVIKDLLLVDEYAIGNRTYYTR

YLSQIPANTLREQQGRTCIAPAEAYTRVQNSEIPQLYTVFPWPEYGLGLPNLTHAVNTYL

YDTETVSFHGNVGWKQDVIWLARMGLTANATQMTEQRYAPSTVCRFPTFKGPNFDWTPDL

NHYGAAAIGLQEQLVQTFVGSDIRLLPAWPETWDARFKIWAPRNTTVEGSVKSGRLGEVI

VLPKDREGDVIVGQNSQESRSIVDSSSPRSLPVRKSVCRRKGVLVIINQPPINAQRNADT

HTLGLSNQYKTSDDPLDGVSVCLRFPIRSSQRSHNKKVTESSSRVGD

>SoG_03401.T1

MKLSIRLLAFLLPTVLSQSLYIDTIPGYTKLPPCAEEPLSTIVRDMSDGCGDNNALTSYS

CFCTRSSSKMSQTISSKVAKNCPASATAVAASALEVFASYCRLGDSYTSTAAVSMPYGVG

NATALGPIQTEDSAAQTVTSPAKLAFIIWSAVLHSLLGIHEQVSIVVRDGWLEESGGKRG

AIIMPWRRAWRPQQPDLHGRHEQEHLVFKVHDIQFGHSMLLLGHTVLLGLTATAAAVTCH

RNETGTSPSWTGTNLYYLQALSDADQDAYIQNLIDYDAKAIRLWVNSHQPGCEKGSQRVR

TIPPLEETLGVYNDGCLDELDRVMAKLEDKGIKAIISPHDSNSLLGDYRADIYYETFGRD

AFYVSEAAFDAYDARLSHILNYQGAHSGRVWKDWPEAIMSFNLQNEPMTPDPSVCQGGDA

QGWLCGRATHLRDELGSNSPIIVSTGGVGGDFSHSCTFVSAVTDCPAVDAISVHRYASVP

GNWDFVLDGWLDEANGKLVYLEEWGIDSSRYDRATAFPQEVGTMNSVGFPSMYWQILPPA

VDGCPYDPVQDSGDHFGIFTDSGVGLAGPMKGAMQVTAAQDWTGIVY

>SoG_03404.T1

MFRQAFSSFPLFLVFLAVALLWWSPREALVDHSLVLGRNNTVLFLTSDYPGLTNVHIATA

FALVENHPTVDIHYASFPRLEKKIRQVSDAAQAKNPSSNPIQWHTLPGPDMKTTFRRFFG

GPNDMVELPGARGMGKKVKDLRVLLTSWTAEEHWTIYKVLIDLIDDVDPSLIVIDHVFKP

ATDLPGNVNRRFITISPNSLLDLIPDRQPWGGMFWKFPAMASGYRFPVPWHLVPANIFIN

LRIILGTLTAPGMGAIRAYLEERGIKDPLAFGLYPKRTQVLASSVPEVNLPMDFIPPEIS

FYGPIVLETRPAMQQDPELMRWLANSGGKMVLANLGTLYEYDETRARQLAEALAQVLDQV

EGSRILWKFLKDGDFDEKSALEPLEKFIKTDRARVVPWLKVDPSSLLASGHIDLFVNHGG

GNSYHEGIFAGVPQVVLPIWLDCYNFASTAEYLGIGIWPGRDTAPMWDPDTIAKGIVRGL

VGEESMSLKRKSQALAAKARSYGGRNSAAREVARLAAH

>SoG_03409.T1

MHRSVNALVAVFGVAFASLHAPENSLGARSARGYAPTLEIQSPTLTSKWVEGSDFTQIIE

MVIYNSNANNYLTAADTLNVTLQSDSLELVQSATLTRLAPSQAAVVQIGVKNKPGVARGS

QCSATVVATYGQNYGVRSTTKQFSGTCGIPDYQADTNSLGFHWTPEWYNNVKFGIFIHWG

IYSVPAYGSVGSNEDYAEWYWCRMHDPNYKTKTYQYHAQTYGENFNYDQFMPNFTDAGYD

PKEWVDLFAEAGARYMVPVTKHHEGFALFNTSDSISKRSSIHYGPKRDLTGDLLDAAATY

QPQIRRGTYFSMPEWYNPAYSRYKNSNPSWGGGCFGADDVNPYTNAGVEYTGYVPVDDFV

TGIQLPQMRELAYNYNTELMWCDIGGANNSTIFASEWLNWAREQGRQVTFNNRCGIPGDF

STPEYYTNGGTVVSKWESNRGMDPFSFGYNYQTPDNEYLTGNDIVQSLVDIVSKNGNFLL

DIGPTHNGSIPQIMQNGLRDAGSWIHPHGEAIFDTRFWSTTPGQDNLRYTTTKDAFYIFY

LTQPPSTLTISDPVPWLPGDTVTALGGSADGTVISTQRTSDGLVLQIPDAAINGDKYVWC

FKVAYTSDW

>SoG_03411.T1

MFNRLTHRLRGGKKEISNPSPLRSTDPSSAEASTLHSEIPDSSDGSSSGTRNRNVALKAS

DGLITAESESRQHSCSNQMGLFSVAEDNRQHHHDAWPVDIIAIHGLNGKAFSTWTHPVSG

NMWLRDNLPSYVPGCRVYTFGYASKVRDNPSVASIPDFARGLLDAVRNLRQGSEETLSGS

SVNYLRLSQSWRHRLQADRRHYGELLDSIIGVVFLGTPHGGSTVADRALPIGGALNFLSS

AASIGMKSGIIKEDLLEVLKYDSQALLELNLSVRQLLGGMAVITFYETLPLPPLKTPNAR

LFAKRSGGFRFQLGGIARPLPGPEADAPPHQVRQSSHRTCLSEIGLLISLLSAGFNDLER

SCMNLFSNFDLRDYIARLPSPVHGTCTWIRSHPSFVSWAEDPESAVLWLTGNPGCGKTVL

SLSIARRLESESQRVLIYFCDTKINSQQDGKAILTGLIFQLVYSDRRLVRHIRREYEICG

ASMVSSFSTLWSVLKHMIKDLRDKLIYVIIDALDECEVSSCNHLLGAIRELTDSDSAKIT

DKTIKFLLTSRPTVQVSVQTNRTEGLVAIDEGESGYLDDIQRFIHSRVEEVALKHGCPEQ

TKDHLLKAMLSKSENTFLWTHMVLELLEESFLASTADFDSIMASLPPNLESMYASFLTKI

PKSNLLTAHRLLSLILASSRNLRLDEINFALTITGRHQESAEVIRDWQPAIARTIQGILG

PLVRISDDQVSLVHQSLKEFLLSSDESEPRSCSRDASQDMPAITSKSAALELATVCIFFL

LLEDFQTPVASPESSPIDAVHTNVCDEAEQPVDDGELWGQDAMGLSLFQEADDLDADLSH

ALSLHHPFYSYAATNWHYHYSMCEDVALPELADAVMTLLEVNSTAGKTWRSFARSRGFDT

TTSFPAETGSLVFASYLNLKGAAGVLLEHGHCSQADKDEALFWASSRGHPQIVSLLLAAN

ADPEYYGMDRRTALLTSAANGHLESVRLLLISGRCDVNIRGKGGRTALSLAAGNGHYDIV

EYLLGMESTRADLGDQSGATPFIWASGGGHFAIMSALAKDHRVDVNSQDNQGRTALSWGA

GDGMDDVVTFLLRRIRGIDPNLQDKTGRTPFSWAAGSGHSSVICVLKKNAKVNRHQSDHA

RRSPLSWACGHGHEDVVRILLANKPQGLEDKDIDGWTPMAWAIQRDAPGVISALFDAGAK

NLDEGPRTVLSWAMEYGHLSVVRMLLAKGADPATARDRIPFAESMGRMDLAKELSDALSR

GSSLIKHRPDEGNIKEESAEGSLAV

>SoG_03413.T1

MPSFSAWCFRFYFEWIMRAKAINLSPEKTRARVDEGYIRPKPFHPPTNLGSDLTIERVDV

TGWPLYRVSAAPAGTPTKSRPAMVYIHGGSFFVEILEPHWRFIAQVARETKLDVLVPIYP

LLPHPKATTAQHIEGFLELYANSPQPVVAIAGDSAGGHLSLLSMQQLLERDPKLAKTVRC

LVLISPCLDISLTHPDLEAQEARDPWLATPGLRPLTKELAGGLPLKDPRCSPLYGNIEGL

PPVLMMCGTDDMICADTRRLSGKYQGKGTEVGMPGSFKNDKLTYIEMPNMMHVYPLLPHW

EGGQARLIIMDYLRECTK

>SoG_03422.T1

MTKTTPYNLIVGLTVAMGSFTYGFGFASFATSIGQPGFYEYFGLSLQGPQADFTNHILGA

VNALFFFGATVGALAGGPFADTFGRRYALMLASVLSIIGGALAAGSVHIAMLIIVRVLQG

TGLGALATLTPIYLAEASTPSKRGMLTGLHGFFLVLGYNVSAWVGFGCFFSSNLTFGWRG

PIAFTCVPAIILLIGCVWIPESPRWLLMVGRNDEAWAGISRLHTDASDPDQLATHEEFYQ

MRKQIEFERSNPSGYWAILTTPSYRKRAFLSCYVQFAANSTGGLVINYYSVVIYTNLGLK

GYMPLLLYALYTMVGAIGNLGSLLTLDWTGRRFALITGFSGCLVALIIETAMIAEFVVSD

HPSEAGQKVAIFAIFFFVFFYGFFIDAASFVYSSEVYPTNIRSRGVALATATYFISCITY

VTPGATAIAAISWRYFVVFACLTAVTVLVLYFVYPETKGKSLEELNAIFGDAVVVRLTDA

TEEELREIDRDIKGQLGVEQSTTPFSPSMSYKLQPGCTVKDADGIAKNNVSAFWANTWWR

ILFSSSEEALLERIARRTPSNLLSQRDVKRHQVVIDASTGDVVGYARWILPATHDAEWIE

AQVPAVSDLDIARYMAISEETPLPFGENQNEMDALDRPLYVKQRELEPKVACLSTFNPKR

KEKRHIKEDSHTDMICTELDYLATHPLHQRRGVSSMLVKSGLDQADKLGIDVMVTAMGRA

AKSLYKKHGFELHWDLEQGLEPWGHNDVYYTAILIRRTAGEKRGQFNFPTSL

>SoG_03433.T1

MSCFIKLSSRVFSVTGGASGMGGATARLLAQHGAAAVWIADWNDGNFVNIKEDIKRTNPL

TEVHTAKIDISDAKQVDDWVGSIIATSGALHGAANVAGLSQSVLSDVQPAILSETNEDWA

RVLSVNLNGIMYCTRAQVAAMAKMPRESNPALVNVSSMASLLRGPRAFAYSTSKAACAHF

TSCVAKDIYPLGIRANTVSPGNTFAPMTKQFFGESSSKEDLEEKFSHWGLGNKMLDPEDI

ARLIVWLLSEASMGINGVNLPVGEGAP

>SoG_03445.T1

MENTPWSDGVPGVTQREIPPGGSFQYKWTATQYGEYWYHAHHRGQLDDGDFGPLIIHPKK

DRQTPFSLISQDKTTLSAIEQAVANIQPVMLSDHRNIPAPEAWDLEVAANMELPCYDSML

VNGKGKIDCWSAEKIASLTTPQQKALLKIVNATSMTPKGCLPKEADAAILAAGRTVNLSA

IPAEIYDECTPTEGSRAVIEVKSNGCGSQGTWAALEIVGAYSLFSTVFSIDELPMWIYAV

DGEYIQPQRVTAIRVTNGDRYSVLVNIVETGDYTFRHASTLPVQLLSGQATLSYRDGKKP

ANKSGDSKPYISDAGLPLSSDVVFFNQTAQKSLVPFPVAQKADQTFVLGMGNTETAYMWA

LNGTSQPMALDAEDPILFKAQPNLMNNLTITTLNDTWIDLVFVTTQVPEPAHPIHKHGNK

MWLLGSGQGAFKWSSVDEAIKAVPQSFNLVDPPRRDGFSTMDAPHAPTWTVVRYHVTNPG

AWFLHCHIQTHLLGGMSMVIQDGVDHWPKVPSNYLEYGS

>SoG_03447.T1

MSDQGHLYLFGDQTSDFVPGLRQLLRVRDSPLLAFFLERTHNALRLEISQQPGEVQSLLP

RFSRIVDLLSSYSIDADSNAALASTLTVIYQLGSFIRYYEDGSKSKPYPSGQNHVVLGMC

TGQLAACAVASATSLIELVPLAVEAVVIALRVGLHVTKIRELLEDGPAKHQPWSYIIPKL

SAEEASPRIEQFSQSANIPFGSRPFVGTVTPSSVTVFGHPDALKAFTASPYMSDAKSLPA

SVFAPYHAPHLFSQEHISEILSGLPSPVSHVSAKLRMISSTTGEPMKATTLYELLHTSLE

EILRQSLRLDNIAKSLSTSCNAVLHGKWLILTVGANAPQTLANALCENQAAAVTVEPISS

TQNVGRTSMPNDANVGRQDQSKIAIIGYSGRFPEAVDPESLWDLLHEGRDVHREIPPDRF

NVETHYDPTGKKKNASQVRNGCFINEPGLFDCRFFNLSPKEADQSDPGQRLALMTAYEAL

EMGGIVPDATASTQRDRVGIFYGMTSDDWREVNSGQDVGTYFIPGGNRAFTPGRINYHFK

FSGPSVSVDTACSSSAAAIHMACNSLWRNDCDTAIAGGTNVMTNPDNFTGLDKGHFLSRT

GNCKTFDDGADGYCRADAVGTVVLKRLEDALADKDPIQAVICGAYTNHSADAESITRPFA

GAQAAIFRRILNDAGHEPLDVSYIEMHGTGTQAGDAVEMRSVLDVFTGKSSGRSANDPLY

LGSVKSNVGHAESGSGVTSLIKVLLMMKNSEIPPHCGIKTKLNTGFPKDLTERGIRIALE

ATPWQRPADGRRRMAFLNNFSAAGGNSALLLEDAPSCSEDASLSAEDPRTCHIVTVSART

PKSLLANAERLAKHLSQNKQNMSLGSISYTTTARRMHHNYRLALTGRSIEDITAALEDSA

VSLANSSKPIPRKAPTVAFAFTGQGSVYAGMGKQLFDSFRVFREELRRLSEISMSFGFDP

FLGLIHPSNAEKDPDFQATPQATQLALVCFEISLARLWKSFGVTPSVVVGHSLGEYAALH

IAGVLSARDVVFLVGSRARLMQEKCSAGSHAMLAAKAPLSDISAISQEYGLDVACLNGPT

QTVMAGLVHDVDRAEKAMQQQLGIKSTRLDTPFAFHSSQLDSVLEEYQAITRGVVFRKPR

CPILSPLLGEVVTEKADFDSDYLVRHCREPVNLLSAVQDSISQKLISEDTICLEIGPDAI

VSRMIKSIVGTSTRTISGVKKNQDPWETLSDAVASMYRAGVDVQWSEYHRDFDEYCHVVS

LPSYQWDYKNHWIQYVHDWCLTKGDAPNMEVVAIAPAAASDVQPTYESLSSTCQKVLESL

HGTERSEVLVESDMSHPDLRAVFEKHKVNGAVLCPSSVYADIAMTLGDYLAKRNPRQANT

GIEVAEMATTKPLLMRNPGKPELFRISAEADWNTQAAKVVFYSVTTSGERSTEHATCTLR

FGDPEAWKSDWKRIAHLVQGRMRCLRDASGQADSSCHLIKRGMVYKLFENCVEYGDSFQG

IEGVYLDSKSHEATAWVNFQDKSTCFFANPYYIDSLGHISGFVMNATEAFDYRSQVFLNH

GWESIRCAAKLSPEETYQTYVKMNSSDGKKYVGDVYIFHGEEIIGVNQGVAFQSVPRKVL

NMLLPSPARGGDTVQTTRTKPSPTVASPEVKSQPPPRIPQSIPAASRVLPSDPRERPAPA

APELVLIMQAINIIAEEIGIAPAEMNNELEFADAGVDSLLSLTVCGRLREELNMDISSSL

FMDCPTVRDLKRYLGATMGPTPVSSYDSSSDGTQGSGRSDQGEESDATSIDDNFDTKHGR

EMVIMESSSGETPLISALCSILADEVGVQVEEIWSAPSLSDLGVDSLMSLQVLGRLREEV

DLDLPTDIFFHDNMVTIREKLVGTSTQSPMQDTSISKTALDSTLRTSSSLVDIPAATSVV

LQGSLKTAQKVLFLFPDGSGSATSYASLPRVAQGVAVVGMNCPYMKRPQDLKCSLADLTT

PYLAEIRRRQPHGPYHLGGWSAGGICAYDAAKILIAGGEVVESLILLDSPNPIHLEKLPT

RLYHFLSGVGVFGSGDPSKALPEWLLPHFLAFVDALALYEPDPFPPGRAPSTHAIWAADG

VYRSTGGKRLEKQADDTRELRWLLEDRVDFGPNGWDKLVGVNKLKMQVLQGANHFTMFNG

QQGQQLSRFIATSLGI

>SoG_03449.T1

MGSIDRDVLIIGGGLGGLCLAQGLRKAGIPFRVFERDVNASWRPQGYRLRINGEGANALR

ETLSPELWERFEKTCCSAELGETDINAVSGMLVASRAGGGPVMKGMKPYTCDRRVLRDIL

LVGLEDRISYGKELERYEINADHREVVACFKDGTVASGSFLVGADGKGSATRRQYLADQL

PLDTEGMCIYGKTPITPELIERFPSKAMRWMTLVVDQTPITQTLDVDDTAVTLLLEPIRF

TKKGDEFDQYMPEDYMYWVLIARKQIFNLPSNERLANMTSDDVVAMSLRITECWDPSIRS

ILHLQDKEQTSLLRVLSAHPDMKTWTPSDMVTLIGDAIHVMSPCGGVGAATALADGANLA

KTIRERGVSAESIGEFEETMRRLAGMNIRRSYIGGRKLFGQRPFEECKEYRASVFNTFSH

TTLLCPTRVSLEKTHNTVDRHKMGSTSTQSHETIDQILRQQVDGENPVLHNACYYVMGKD

GLLYSAAYGTSDLASSTPVTTDSVYWIASLTKLSTAVATLIAVEKGLVTLDDDVRKIVPE

LADIEVLDGFDEDGKPLLHKCTSPITLRHLLSHSSGFCYDIQDEGLKRWAAYVGKKENTF

TGSYKGYLQPLVFEPGRGWAYGSGMDWAGRTVEVVANQDFEAFMQENIWAPLKMSSTTFR

PWLRPDLEDKMVELAWRKPGGGPLIKGKVPYGYPAADCCGGVGLYSSPSDQAKLLSAILN

GGGGIISPESIDKLLKPQTEDPSHFVSVACGSKRAHLGQMWPEGSTGDFGLSSSINFQDF

PGRRAAHSASWQGMPGVHAWLDRKSGLAGLFVTQVLPPGDAHVTRCFCDLEAAVYELYGY

DKET

>SoG_03464.T1

MKTSSTSLAIVASLINRISAATYEPTWESTDKHNAAPEWFRDAKFGIYWHWGAFTTPQYG

SEWYGRYVYQPTGYVREEHTRRYGAPEVWGYENFIVGANDLKGNHVQFSPKLVSEGGQFD

PESWIALVKASGARFAGPVAEHHDGYSMWDSKVNEWNSVAYGPKMDLVKLWEGLVRQNDM

KFLIAMHQAFNTNGFFQYAPKQNDTSHQKLYGQLPKEESDQLWLDKQVEALDHVQPDIIW

HDFSLDSPGYCQDSGFPCNIGEQQRLQYLAHYFNRGVEWNKEVLTTYKHFDKGFRDTSAV

ADFERGGPADLVRPYWLTDDAISASSWSYSVGMPLYSSIQMIHSMLDRISKNGNMLLNIS

PTAVGTIPSEQEQVLRDFGTFLSRYGEAIYDTRAWDIYGEGPNKAGGGSFTAPLTGNSSD

TRFTRNKDKSVLYASILGWPADSTVFIAALGSNAGVDLAGLMSVEVLGDTAGSYIPAVGW

EQSAESLMITLPSQPAESQAYVLKFVFESNIPVPQIKGGASAFTSDSVKGRGISLPQGDF

TSAFFKDEGTAVGDVVLLRVDEGASATVYFNGDFTGDSAVYASGEHTLQKGSFGSVKVAS

A

>SoG_03477.T1

MPGTTETGAAASDAPATNAPGRLLLLSNRLPITIKRSEDGNYTFSMSSGGLVTGLSGLAK

TTSFQWYGWPGLEVPESEVDGMKQRLKEEYGAHPVFIDDDLADRHYNGFSNSILWPLFHY

HPGEITFDESAWAAYQEVNRLFAKTVVNDIQDGDMIWVHDYHLMLLPQMLREEIANTRKN

VRIGFFLHTPFPSSEIYRILPVREALLAGLLDCDLIGFHTYDYARHFLSSCSRILGTPTT

PNGVDWNGRFVTVGAFPIGIDPEKFVEGLNKPAIKERIAALKRKFEGVKLIVGVDRLDYI

KGVPQKLHALEVFLTEHPEWIGRIVLVQVAVPSRQDVEEYQNLRAVVNELVGRINGRFGT

IEFMPIHFLHQSVSFDELTALYAVSDVCLVSSTRDGMNLVSYEYIATQRDNHGVMILSEF

TGAAQSLNGSLVVNPWNTEELAQVIHDAVTMSPEQREANYRKLERYVFKYTSAWWGASFV

SEMTRLSAEGSQPKTLRNISGSVVGDIGQKVKQAIEGVEKLALGDSKDGGVIEEEKTA

>SoG_03480.T1

MNQTVKLWDAGSGREVQTLKGHSGPVQAIAFSLDGKTLASASINNTVKLWDAGSGREVKT

LKGHSGPVQAIAFSPNGKTLASASIDSTVKLWDAGSGREVQTLKGYSGPVQAIAFSPDGK

TLASASEDWIVKLWDAGSGREVQTLTVNSVITSLSFSDTEAGLHTDRGYLLTTTPSDFRA

LSPSASSPPSIFIEEAWISLNGKRLIWLPSEYRPHVTSVRGSSVGFGYKSGQVLLLRFAL

>SoG_03482.T1

MSLPDPTEDLDWGGYVGGIHEIFHKNAVAHPDRACVTETKTSKAPTRTYTYRQIDEASNN

IANYLRDSGIQNGDVVMIFAHRSVELVCAYMGTLAAGAIVTVLDPQYPPQRQQIYLQVSQ

PKALISIRKATEESGPLAPLVEKYIDEDLGINIKIPDLRFTDDGVLTGGAEGSADIFANV

KERASTPPDVLIGPDSNCTLSFTSGTEGLPKGVLGRHYSLAKYFPWMAERFGLSSESRFA

CLSGIAHDPIQRDIMTPLTLGAQILIPAKEDIQHVKLSEWMRDWSPTTTHLTPAMGQILV

GGATAQFPSLRQVFFVGDVLTTRDCRSLRQLGPACTIINMYGTTETSRAVSYFEVRSAQD

DPAALEQLGDSVPAGWGMKNVQVLVVDREDQTKICPVGVVGMIYIRAAGLAEGYLNDPEK

TKEKFIDNWFVDNNKWVEADKASDKGEPWRKYYKGPRDRLYVTGDLGEYRPDGSVRVLGR

MDSQVKIRGFRIELNEIDANLGGSPLIRDSKTLVRRDRHEEPTLVSYIVPEIAEWKRWLE

TQGLQDVEEEGVEMGPCLVYLKRFRRIQAEVRDHLKSRLPAHSVPSIYIVLQKLPLNPNG

KVDSPNLPFPDASLMTEDASEEDLKSWEGLSETEKAIATQWSTLIPGLNAKMVRPGSSFF

DCGGHSLLAQQLLLDIRKQFRVDVTIGILYSDPTIRGLASTVDRLRSGQSVVVDNSNETV

YSDSLDELTKTLDAKYQSADPEARTPSSGAVFFLTGATGFLGAYLTKDILDRENTKLIAC

IRGAKDLKFAKERLIRSLKGYGLWQESWVDRISCVIGDLSKPRLGLDDASWKHVAETADA

FIHNAAYVHWIARYEQMMGPNVLSTIDAMKLCNEGKPKLFSFVSSTSTLDTDHYISLSDA

QTATGRGAVLESDDMMGSRTGLGTGYGQTKWVSEQLVREAGRRGLRGAIVRPGYILGSRN

SGVSNTDDFLIRILKGCIQLGARPRIINSVNAVPVDHVAHVVVASTLNPLPGLQVVHVTA

HPRLRMNEFLSALSYYGYDVPEVDYDDWKSQLEEFVSAGAVEKDQEQSALMPLFHMATSN

LPSTTRAPELDDRNAVAVLRTDADRWTGVDDSAGEGITRDDIGRYLRYLVEIKFISPPAG

RGRKLPDIDGSIAEALSQWGVGGRGGSS

>SoG_03486.T1

MVDRPNKPSALATTPTMPPQAQVSISHGNSRVTAVLPTGESVEILLYGATVISWKNAAGD

EKLWLSDVAKLDGTKPVRGGIPLVFPVFGTAPNHEATSKLPQHGFARNSRWEFLGKSTSE

GSSSSVKLDFGLSSETLDASTRSMWPYSFGLLYSVTLDRDSLNTTLVVTNDGDTPFECQT

LMHTYLKIKDITQINITGLEASSYIDKVDGMATKTQSATVKITGETDRVYTPVKGPKHPI

IVADDAGTTLYRLVRDNLDDVVVWNPWVDKSAGMADFEPKHGWKNMICVEAGSVKGWQRL

DKGDALEGAQTIYLE

>SoG_03493.T1

MSTSKWSTTKTACSNCHKRKVRRSFTSSYGEKRRVERSEDIKAQNGDLMCNPHCRKRKTQ

QVTNPAPKRHLQHQEQQSEASFLSRRAPVPDCPPELPTTYLLHTPEHSEPSEAPSDRHET

VSEARLGDESPYVSRTEILGPGPFTERLLPRKAILSTEDNDLRCREVLKIFDADATVSEP

VAARLISLYRTFCWPWAPIVEKSWLLPKSRSSMPLLLLQSVLLTGSRVSSTGPLSQSMQL

YLKAKARFFYGDDRMPLASVISAILLQWWSPTGPEHFSLGNSGFWLHITIGLAHQLGLHR

EPPQGPYRGLRRRIWWTIVTRDILISVGVGRPRMISLEDSDVSPPCIHDFREDSTETRVF

ISYVAICRILGDVAEMRRKSRLTRERRREAEKSLMTWLRDLLSELRLFSSDAAEVPSQFD

LQSRQLHIIFFVSLIVLANAEDANAASVTSIVSASFVASIFAELQEHGELQRLGPIFAFH

GLAAALPLLSAQKFRTIASDAMLDFEKIYSALQELAEKWGSARGLLEPLAAARETARSSS

MEEGSSSRQPPAAKDSLLRILSNFGPGLCLLWWLIPEDSGVVDCISSSLRVDKAESEKRA

DVSQDVPPERQDALTPGSFSWAQLADFDNVNLTHQPWEGLPMQTFLPDDSWMDDLADP

>SoG_03498.T1

MHFSLSSVAVAALLSASLALGQATGTVAKGPFPVDLNGSNFTYPFPVQVFKFKSQNENVE

MAFMDVKPKCEPNGKTAVLLHGKNFCGATWETTIRALGSKGYRVIAPDQIGFCKSSKPIG

YQFSLRQLAWNTRGLLNTIGVDNVTVIGHSFGGTLSTIFSLQYPDTVEDLVLVNPIGLED

YSGKGVPYLSIDNHRANEAASTYQSIRGYEQAVYYLGKWAPEYDTWVRMLVNIYYGSKRD

AFIHCQAKIVDLVLTQPIAQYFKQLKPRTALMIGLKDTTAIGAQWSPPAVAATLGHFDVL

GPQVADEIPNGQLISFPDEGHAPQISLPQEFHDKLLAWLSS

>SoG_03504.T1

MTGLMLLVRHVFDLSVDLAAWTASCCIVCILLAASYRLLLHPLAGIPGPKAAALSNAWLA

YHVRNGNSVHIGRYLHEKYGPMVRVGPNEVWFNSKTSFKTIYCPGSRFDKSDFYLATALP

RPELDWRLDPISADTLDLLSERDMKRYRRQRRAIGPIYHASNLSRYEDAIDGVLTRAIQR

LKGLQNEPVDLKEWMHMIAVECLGAVVLSWSPGMIKRGTDAGTSYHSYLSWRRKSVFGLF

PTITKLAYLSKTLDRVFSVIWGITYKPPRDFRTFFPDVGKRVSKRINVALHRQIKPGTHH

DLITDLIELHVRKPDFTETYLKRMAVTNFGAGHETLAATLTSTFTLLASDADSYQRVATE

VRGHPSPTRFAVAFDLPLLQAAVKEAKRLRPVIGMSLSRRVPHGGVVLHDRFFPAGTTVG

CSPTALHCNADICGSNPRAYNIDRWSDPETSRDMDTFSLSWGGGSRTCPGRHLAELIVFK

TTAALVLHFELEVTVPREEDMPSYFMSMPTGVRARFLNAN

>SoG_03511.T1

MASFAPAFNRTRVPLRRAFNVTACASRAFATVPPGPNEPSQPAPTRRKTYFKDTTVAPFS

EFVGGAEQPLSTSEAYSLRTAEVGPEGKKRTITRLPEWLKTPIPAGNDNFKSIKKDLRGL

GLHTVCEEARCPNISECWGGSDKSAATATIMLMGDTCTRGCRFCSVKTSRTPPPLDPHEP

ENTAEALARWGLGYVVMTVVDRDDLPDSGARHIAETVRRAKQKRPDLLVEVLTGDFWGKL

HDAKIVAESGLDVFAHNIETVEGLTPYVRDRRATFRQSLSILKHAKDVMGDKIITKTSMM

LGLGEQEHEVMDALRELRKINVDVVTFGQYMRPTKRHLKVEKYVTPAEFDMWHDRAMEMG

FLYCASGPLVRSSYKAGHHNLLRPVAFPAAVQPDESLYRLIIFLQARLVMARLWPVFALI

ATVVGQFWMEDIAHQGKSPYHADSAYQVFRNVKDFGAVGDGVADDTAAINYAISYGGRCE

PGVCNSSTISPATVYFPSGSTYLISNSIIDLFYTQLIGNPIDPPVIKASPGFANQSFGLI

DANPYQSYGFLGWRSVNTFFRQIRNFVFDMTDLPSDFNAVGVHWPSAQATAITNCVFHMS

TVPGNSHTGLFIEEGSGGLLNDLYFYGGGKAAILGNQQYTARNLWFFGADVGIWMTWDWG

WTYKSVHFQDCRIGIAMDVDLLAVGSITLIDSWFERVGTAVLTTRVADEIYGTNGTLTME

NVLFKDVERIVEGPNGTIVDQTNVYETINDIFVMGHVANNIGLYSQTGYSETSSRSPYLI

VQGKYYERSKPQYEDVPVEGFYSAREFGAAGDGIADDTTVLNDFFKYVTARGLIGYLDAG

VYIVSDTVLIPPGARIVGEALAAIIMGTGSNFQDMSNPRPVVQIGLPGQEGNFEWSDTIV

STRGPCAGAVAIQYNLYSPSGVPSGMWDVHIRIGGFAGTNLQLNECAAGAGPETNDPDPN

CIAAFMSMHFTPDSGTLYTENCWIWVADHDLEDPWVRRLSIFAGRGLLVESRRGGIWLSA

TSSEHHVLYQYQFHQTKNAYLGHAQTESPYYQPQPLARYPFPVIPELNDPDFAKDCAADP

APGCESAWAIRIIDSSDIVVYGAGLYSFFHSYSDSCSRENSTTDCQGRLLSVQGETNGIR

FLGLSTVATRVMIHRDQQDFVQAQGNKGTFADTLALYLP

>SoG_03528.T1

MASMAPAAVPAISRRDSDNLIRKIMGSILNRHLSAICQLNGLKSTGVKADMQHRIVNQIQ

EAVKANDPARFYHIRSSIEQTVGNCNTATQPISPSHPFGLHTPSTSLSHYSSQYERMTKN

SPNLQAPHDWPVAGTNGHRYGGPSHATPYLPLTFSPSPFYQIETTIGEPRICETMSQHRN

SVNISLRLSDNSQLQKCMDDKTYRVMVFCAGDCVGTQEITFPHQSELKVNGGDMKANLRG

LKGKPGSTRPVDITNALRLKSKQKFYLVVAVCKTTPVEDLVQKVKTRSRIPKESVIAELN

RKAQDPDVVATSQVLSLKCPLSYMRLTVPCRGKACKHMQCFDATSYLQLQEQGPQWLCPI

CNRSTPFDQLAVDEYVKDILQNTSSSLEQVTIEPTGRWRLSNGDSKLRQSNNASYQAAED

DDDDDLEISEVNIIGSGRRLETPKTTATGLATPGSGTGSSSSAAGPRALGSTSQKRPAPT

VIDLTLSSDDDDDEPIQRPAKRQNINSNGFHLPSSTAFYQ

>SoG_03538.T1

MKFGRNLPRNQVPEWASSYINYKGLKKLIKSAAQKAKLGEPVDPAEFFFALDRNLEDVDS

FYNKKFGDACRRLNLLHNRYGRIPDVVATLDRDEVEEAMGALLELRSQLRNLQWFGEINR

RGFVKITKKLDKKVPGIATQHRYISTKVDPKAFAKDGNTARLLAEINRWLSILGEAQTFD

DSMSDRSTRSLGRVSNKAMLNLPQAQLDVLDNAVRTDSVDSLKEGLRDVNIQGGSESTQT

LLLNLLQRSISGRSRKCITYLLENIQDLEESEDINGRNCIHRLVIHIGRVKSAPEKHDAN

SYPVPVGTQFDHRHPQGTAPNAANRKPDTQVLLGKDDEAIQILMYLLDNLTSRQRVALKA

RDSFGRLPLHYAAQYGFVVVCQIIMAKMQEWDQFDSKDGIDAPEWHDNDGYAPLHLSVMG

GHALATETLLKGETWQGSSESKDHIRKTTSKSGAVLTMATKSNYKIIVDLLVTAGVDVNW

TDEQGESALHVAARFGHDECAAVILKGGGSQKADLEIAEKSFAWTPLHVAAVDGQLKIVE

MLIEAGSDLSKPDSSGWTAKEHAALRGHMAIARLLAAHTKDEEAETKSSPTLAPTNPPGA

QSIGERRSNGTSNNGNLRTSAEPVKTFGHRYLTDESLVLVSLGSMDMRKTVEAVSLDQVP

LTEAHNTQLDTALSVVVSANGAQGEPTIIDLPVSENISTEPIVFTTKEFSKVKLLFDIVP

TYSGSEKNKIGRAVALLSSIKPTIGSQRMNLQGDVCIPIMSSTLDVIGVVNFNFLVITPF

HHPNMEITSRQTYWKKLESTMLIGHRGLGKNITSNRSLQLGENTLPSFIAAANLGAQYVE

FDVQLTKDHVPVIYHDFLVSETGIDAPVHTLTLEQFLHINPDSTRNGYENNGHKNGSRVD

SVKRSRSSSFTPRRTNSVGFPGEQNAALEERMKHTRDFKEKGFKANSRGNFIQAPFATLE

DLFRKLPENIGFNIEMKYPMLHESEEHEMDTYAVELNSFCDTVLSKVYDLAGERHIIFSS

FNPDICLCLSFKQPSIPILFLSDSGCSPVGDIRASSLQEAIRFASRWNLLGIVSAAEPLI

NSPRLVKVVKENGLVCVSYGTLNNDNIMVQVSQLVRGWRSLKIGLLTMEQRQVKEGIDAV

IVDSVLAIRKGLTTSSAEAAEKTNGVKEEVEDAISGATGAVI

>SoG_03546.T1

MVKLALLCVAVLLRGIAVAIPVDPPNVKAVDTTAKAVNLNHTDAEAKAAVFSMLAAEPLG

SRMDRTGWTVTCSTANGADPCSNVLDGNADTMWHTAYQGTTPQPPHVITVDMGSSKVVAG

LAMRPRSSGVNGFISRHEVHLSPDGTNWGSPVAFGTWYGDGTEKFANFEPQAARYVRLRA

LTEAGGNDQFASISELNLYSAEARTQNPSTGTWGATLDFPVVPVAGFVDPPTGKVVVWSA

WRYDRFVGNNGKGFTLTATWDPATNVISQRAIQNTAHDMFCPGISMGAQGQTVVTGGNDA

SKTSIWNPATQDWLPAPDMKTPRGYQSSVTLSDGRIFTIGGSWTGGRRDKPGEVYDPDTQ

QWTALPNAKVIPMLTQDHQGVYRADNHAWLFGWRDGTVFQAGPSTAMNWVNTSGNGSTTS

AGNRTNAPDQMCGSAVMFDAVAGKILTLGGAPSYQDRPATTAAHLITIGDPEDEASVESV

PKGLAYSRTFQNSVVLPDGSVFVSGGQAYGVPFSDDQARFTPELYVPSLNSFTAQTPNSI

PRTYHSMSLLLPDATVFVGGGGLCGGCTTNHFNAQIFTPAYLYNASGQLATRPSIAGVSD

TTISIGETLSFQTSGQIASASLVRYGSATHSVNTDQRRVPLAINGSGTSYSAGIPDEAGI

MTPGYWMLFVMDAAGVPSVARTILVTL

>SoG_03551.T1

MRNQTSSQRLAGIMDAESLPLVLRGLASRYEKHLQALSFAIPSWLHRLGISGTLAAVVIA

YATFTVLYALYLSPLRRIPGPLAARLTAKRGVWSILTGSAASDAQADYENYGDVYVAKPN

AVFLCDPDDARAVLAMAEFRKTDMYRIFEYEGVPNVSTLTEPAQANRRRRQLHPFFAYSY

LAKMEELILRHGTRALNARWGAKIEADAAKGERTIVNYRLDTQLAMFDITGALVFGRDFN

ALKTDNLDYTKWVNNTLTFMLLAHYFPLLKRWPFSHLVASLRRSYDDLVAFSKESIAIRR

ALLDAGGEKPADLLQALLDSEEPDSKVRMTPGEVQAESIAMLVGGSESTSSVISWVIHFL

LLYPEDLRKVVEEVRGKFGAEHVVTHAECRAELPYLEACIYETLRCIPTASTSFPRISDR

KGITIKGFYIPPSTEIVTNKCAAHIHKESWESPFDFKPSRFLDNNEAKRNMLSFAYGTRF

CIGKNLAWVVMIVTLANLFKDYDVALPETSLFHPGNVDENGRPRIMPTKLGVATMPAHPE

RDCLMVLKRRVE

>SoG_03558.T1

MLFGASSVGLLALGLQVALAEDGQIPLDDDYSAACPDYTQYSTHPHPPLTEGRYGLPFQR

PDPRCRTFRSDEIERVIEEVTSRMKDPDMARLFENTFPSTTDTTVKFHTAGEKDTGIFNI

GSFRSSWDSEAWQGPQSFIITGDIIAEWLRDSTNQLRPYQALANKDPKIKTLLLGAINTQ

AEYIIQSPYCNAFQPPPIAKLQLSNNHQEDNVHPAYEQSVVFECKYELDSLAHFLALGND

FHDHTKSTDFVNKRWLLAVETVLEVLEQQSAPTFDPETGAFKRNIYTFQRRTNTGTETLS

LGGVGNPLNSGTGLVRSAFRPSDDATILGFFIPANAMMSAELIRASAMLEAAGKPKLASE

FSKWGRQLREGVLEHGVVEHKKYGKVFAYEVDGYGSSIMMDDANYPSLLALPVMGFCDVD

DEIYQNTRKMILERSGNPYYLKGSGFQGIGGPHIGFRNAWPMSLLMQAQTTDDDKEIREC

LELVLRSSELGLVHESIDVDRVSAYTRSWFAWANGVFATTVLDLAKRKPHLIFKEGEGSY

EI

>SoG_03564.T1

MTAGLFHQYADGLLPVFKQLRYLIVGGDVLDPAVIGRVLKDGAPQHLLNGYGPTEATTFT

TTYEIQSVGEGGIPIGHPIGNTRVYVLDANQQPVPVGVAGELYIGGDGVAKGYLNRPELT

AEKFVADPFSADPAALLYRTGDLARWRADGAVDYLGRNDDQVKIRGFRIELGEIEARLGQ

CAGVNDAVVLARQDDNGPIRLVGYVIPEEGVTLSVQTLRSQLASTLAEYMVPSAFVSLVA

LPLTPNRKLDRQALPAPDADACASNWGWS

>SoG_03569.T1

MSPKEPVEPTSPPPVGPKKTRPRGARACVVCRRRKVRCNVALVGMPCSNCAIDHETCSIP

EKCQRKWSRKGRASNTQVTAPKPDEQAQHAESVKINVSDAVHPPETIEATMTITRPASPL

AGQHCSGQQTPTFEHSWDQGGRGPWHEHRPVERPFAATVPMSAYPFLTSSNLHRLPAEDI

NFLELKECLRVPRRALLDEFLQQYFRYVHPFFPLINEAFFWDVYNGVDLVADNPLPRFPL

LVLQAMLFTACSYVSPDTLRQMGYTSVRAARRVMYERAKMLYNLNAESSRLHLAQAALLL

SYWTPAFEEAAVKPNTGWLRAAVENARSVRAHRWHTTGKTGKAPMEDYEHISLKRLWACC

IFRGGILAISSRKCCQGLGLDSDINVGFPLTFEDLEDEIDESIVYDSETKRRLITACLGL

AEICVHVVATSMLLFPFDSGQHDAATRLQKAQGEEVIRIMACKMTLDESHKRALRPFSHL

ARREEVSALGGNVKDHASVTLFENLRDIYYYSAKIALANRQMLSCHDTYPTRTEEMEALY

EEIQDATSAIGKRLKTLTDLRLARFLPVSVKKMAPHLETPIFTEVLRTYSHLYDGVEGLA

KAVRTIVSQVIHNDSADTPQQGVDSAFGSSLQPSDGGINITQQPLRYYMRLALSIDLSIS

MGNLPDETDFPARVEEHLSASPESVDRADGSPEKADVPPVTAVQNMEMQTPMVDMQYNLP

MEQWVFPNGQAVTTQVTALNQGTWNLEGMSPHTMERSILENLIWSSPEGELA

>SoG_03580.T1

MNASSANDAEHPADLTPKTGGIEEGKDAYQPGGFHPVYIGDVYAGRYEVMSKIGYGRYST

VWLVKDTSKSTDHEHRFQALKVLSAESYGEECPVFEREILIHLRNANRSLLGYKHVCHLI

DDFELTGPNGTHVCLLLELMGENLRTFGVWFADDMVPTTLMRKFSFQLVCALDFAHESNV

IHTDIKPDNVFVKLRDMHRIESEYLVQEPISSELDIALGDWGVSSWVTRHLTESIQPVAL

RAPEVLIRAPWNTKVDWWNLGAVLIEALLEKGDVAAQEIFDEEGKIKGAEPMNRPPLESE

AFLPGLDQPSREVFGSFLRTMMKINPAERPTPEELVEHAWLDPKSRW

>SoG_03593.T1

METPKNGTQQREMPRHSLQGRKTKGEKNKWPFSSSFAAAALSGLAIAVAMYYLKPYLLLA

FAASQAASKPAHGAPKVKVANGTYAGVHSKEFKQDFFLGMPYAKPAERFTVAKGLDTAWH

GVKDAKAYPPHCFGYGDDNIGFEQSEDCLYLNVVRPAGIKHTADLPVAVWIHGGGLYMGG

SADKRYNLSYIVDQSVQMGKPMIGVSLNYRVSAFGFLNGKEALDAGITNIGFRDQRLALH

WVQENIKAFGGSPKKVTIWGESSGAESVAAQVLAYNGRNDGLFRAAIGQSGFGGILPRLP

GGFNETKAAQAIFDAFVSSTSCAPLVGTPESIDCLRKAPFEELNTVLTSGKVGDLPSTWP

PVLDHDFFQDYATNQLVAGNFAKVPLLIGTNSDEGTGFGIGRSGGPVNTDEGFAKLLKDI

LKSENTDKTPDELAAELMQLYPNDQSEGIPSLETWPHIIQPGDSFAKQLGEQFRRVSAVI

GDYFFQYSRRRSNLVWSAHGIPSYAYRFDVVPNGLTDFIGATHFQEVAFVFHNLNGDGYA

VNPFGGNDQEYVSQAKQLSNVMSTAWVNFITGLDPNGAKGLPNGIAWPEYSASAGKVGQD

LVWDLGKSFAEDDNWREEGMAWLMDHSLSVFGN

>SoG_03617.T1

MAENGEVRAAVEPSERVVIGIAFGNSNGSIAYTVDDKAEVIANEDGDRQIPSVLSYVDGD

EYYGTQAKAFLVRNPSNTIANFRDFLGQDYKSIDPTHCHAAAHPQDVNGAVAFTVNDKDD

AESPSTITASEAATRYLRRLTSSASDYLGKKVTSAVITVPTNFTEKQREALIKAARDAGL

EILQLVSDPVAAVLAYDARPEAEVKDKIIVVADLGGTRSDVAIVASRGGMYTILATAHDY

EYAGVHLDNVLMDHFAKEFMKKHNNVDPRSNPKSLAKLRAEAESTKKALSLGTNAQFSVE

SLAEGLDFSATINRIRYEMVGRKVFEGFNRLVEGVIKKAELDVLDIDEVIMCGGTSHTPR

IANNFRGIFPETTVIQAPATSATALYPSEILVRGAALQASLIQEYEASDIEQSTHPAVTT

VKHISNAIGVVTVGADGSELFTPVMPSETAVPARRTVQIPGPADGGDVLIRIVEGGTHIK

VTKPEPKEPKSNNATVEDADEDDSDFDDSDEEEEERREKVWKIGGQLAETAVRDVKKGGK

VEVTINVQADLGVTVTAREVGGKGGVRGNIKGQ

>SoG_03625.T1

MAAVPDTVRPAISDQPQPSYHTPARSYSTRSSRPSPPDDYYYPSNPAVTHDQPAVPRSQS

QYASASASARAQQYQHRRSASSSAVPSPAVVASHSRHLDIHHHQQQQSQTHHVSNPSDPA

QPQRYHRRTSSSVRPLQEALPENDYEASNLATYPKRSPSRDRPLPPSSSRNPPRPAADVE

SSRISHNRTASTRKSNHHRTSSQAQSHGQNSRSMAPSASNGASAPSGGPADGTHSSSGKQ

TRSRTTIPTQSGKWILGKTIGAGSMGKVKLARKEDGSEQVACKIIPRGSTDDGHQSRADK

ERADQSKEIRTAREAAIVTLLNHPHICGLRDVVRTNYHWYMLFEFVNGGQMLDYIISHGK

LKEKQARKFSRQIASALDYCHRNSIVHRDLKIENILISKTGDIKIIDFGLSNLFAPRGHL

KTFCGSLYFAAPELLQARAYTGPEVDVWSFGIVLYVLVCGKVPFDDQSMPALHAKIKKGV

VDYPSWLSTECKHLLSRMLVTDPKLRATMQEVMNHPWMTKGFNGPPDNYLPAREPLSPPL

EPEVIHAMQGFNFGSPESIRAQLTKIIDSEEYQRAVKLFQKEKELPQPNKDEQKRRGFGF

DFYKRRNSGTSRDTLTGPSSEALQLGNDPLNAFSPLLSIYYLVKEKQDRERTDGPAPTPS

TPREKEKERERDRDRDYRDHREPRDYRERVREEREKPVDALPEIAPPQAAVTNVTTYEMP

GEKPTGGRTRPRARTHGEDDVPDVVKQSPQPDHRPEHQPPKKESAASGLLRRFSTRKRRD

PERLDKDRSHPPVVQVHSPAEGASLMPRKSFSIRRGRRERDGSIEPRLRSGSSQPQHSEL

LSPPATAGGSRDSRRGGLGRSTSVNSAEMRRQKARGTAKEPPPTSGSDQSMAEEPSAGPS

LVHGHSKSVAYRAKSLGHARRESIQQRRMRREAAQEANVPEETDMEQEASGVSTERLDSS

DLAKPVFLKGLFSVSTTSGKSVPAIRADIRRVLRQLNVDFIEIKGGFTCRHMPSIDLNKV

QDPPGSPGPVSSGGGHRRRFSFGGLMRGDDREDVRDPSDRPPATPRTPGRSDRDRSDRDR

SYSNSETSVDSIPRRTNNGASRRAPGETSTQVQSDLGGSMVLEFEIFIVKVPLLSLHGIQ

FKRLDGNTWQYKNMADQILRELRL

>SoG_03637.T1

MSDYAVYGGIHEEWVAAEKANPMVTSFLPVEEKKKSQNEVREKLAAESLKPLRAEVTIKD

HTIPCRAGHTVEGRTYRPSAVGETEKLPVYMHFHGGGFMTGTLASEDAICAGIAISAGVM

VLNVNYRHTPEHAYPAAWDDSEDAFEWLHDNIDALHGDAQSVVVGGISAGAWLTASLLMR

KNLGQSTTSSRPAIAGQVLMIPCLVHVDCFDAQRKKLSDPTRTSYEENADAPILPLSIAT

EYLKHLRVTSPDPNDVRLSPGNATPEQVAGLPPTVFGIAGRDILRDEGLLYAKMLAEAG

>SoG_03638.T1

MASNDPRGEPSSWEPAMEPISWNTENIQRHDSTGRRSVNTEYYHHGSSHASPYAGPVRSV

SVGSSRRPLRPISQDGCHSVDEAESLPGPRHPGSISPGVKRYGSQSRPPRAYDTRYSAPP

WNGGSSQRDSVQDIRDDAFQEARPEPRRHYDIENRPSLSHKNHLPRDEEMGRVRDDGDFE

HDGIYHAPASLYGRGGLGWENMTKDEKREIMRLPWLQWMNSNVKNHFVAAVGEFVGTIMF

LFFAFSGTEVANTQSRAAAGSKTTTGSATGFDVGVLVYIALSFGFSLMVNAWVFFRISGG

LFNPAVTLGMMLVRAVSPARAAWLLVGQLGGAISASAIVMALFPAGFNVRTTLSPGASIV

QGIFIEALLTAELVFTIFMLAKEKHRATFMAPIGIGLALFIAELVGVQYTGGSLNPARSF

GPCVVTTTFDAEHWIYWAGPTIGSLVAVIFYKFIKILEYEMANPGADGDPANDPTKNPEK

RATTMV

>SoG_03643.T1

MASQLALVDGPKEPRLWQMTIGDLVKERAAELKTKTAVIIPWQKFRCTYGQLAERSRLVS

LALLHYGLRHGDCVGIMAGNRFEYIDVFLGASRIGCPVVLLNNTYSPSELLNAAKGSCKL

ENHLGWGQQVDRVIACKLVFMATKIGSVHLGKHMDMLTTPQGNSGQHFKIVCFDQMSGPS

RIPYAEFLGAARYSDSQRELERAESKVSPEDVINLQFTSGTTGAPKASMLTSLNLINNAR

FVGDGIRIRPDDVLCCPPPLFHCFGLVLGVLVAFAHGISILLPADSFDPVRTMDAIHAEK

ATILNGVPTMFLAELEVVAKTGRKPTTLRVSFGAGAAVTANLVHHLNTEMGIKDVLIGYG

MTETSPISFMTVPEDSLEKKLHSIGRLLPHCRAKVIDTKGQILPIGQKGEICTTGFGLQK

GYLNNPVKTAEVMKRDAEGTVWMHTGDEVFIDGDGYGHITGRIKDMIIRGKSISEDNIYP

AEIEERILENESIIEASVVGIHDDKYGEVVGAFLRLDPSASRQPSDEEIRKWVLSAMARQ

KAPQYVFWVGEGPGCHFAAYPKTGSGKYQKVHLRDMGNKWVAANKPAKARLMIPQTQFGP

AGVKKRSRQACTNCRSRKIKCSAERPTCTNCRQNCRSCTYEPYSNRPNRDGEESSNIQAM

GANVSLLLRRHKPSVEARFNFGVQAELLKRISTIETMLGRLNPEALEQVQRRSSLTAAPV

STSPNGQNTNSSLQRSNSISTSESSRFRVDSFESLPPPRVMQSLIDTYFLRVHGQPYLFF

HEGEFRRRYDTNNTPKHLLLAVVHEASAAYAKQACHTDNQHLSGGRLYXVSTGWLKVGLA

ARLAQGLDLMDEPPAYIPIAEQEERRRTFWSIYLLDRLISCARARPPAISDDDCNIQLPS

EPGSATTRDENNPSRTLRPLLSWATKLSTPPRGFSLSILMAMVLGRCTKFAHGRADSEPV

PPYDPKSDFMAINSSLMLLESYLDIDKNPIAEVIREVRQNDTVDDTKELGHLIFARALFH

ISHCLLNHPFLLRYRLSSFTNKVPRSFSLRSLQLAEDHAKKLTYLLSSASEGGMLVESSF

YTYCVAVSGAIHTMADRVPQNEDNLGQYDAAQYFQRCIDILSRLGHLWPMAKNIETRLRR

FHDVFPEQLGLFDPSLLATPIDPSFDELLWSVVDYNTLAGELPKWDVFPSMSALPSPSLW

DMDIGLPSVTQGFDLQVPNLFAETGDMFSQNDVQ

>SoG_03648.T1

MSNLTSRQAEELHKSIIAYLAANNLPNAATALRQELALGEDAFDTATAKKYETLLEKKWT

SVVRLQKKIMDLESRNTALQYELDHLTPSSLSKRNKDPASWLPKSPARHTLESHRDAINC

VAFHPVFSSVASGSDDYSIKIWDWEGGELERTIKGHTRAVLDLDYGGPRGGVLLASCSSD

LTVKLWDPSDDYKNIRTLPGHDHSVSAVRFIPNSGNLLVTASRDRSLKIWDTTTGYCVRT

LTGHAGWVRDVDPSPDGRFLISTGDDMSARIWDISTPSNDFKSVLLGHEHFNECCAFAPA

SSYQHLGPMAGLKKPPPATSAAEFMATGSRDKTIRLWDSRGNCLKTLVGHDNWVRDLVFH

PGGKYLLSVSDDKTIRCWDLSQEGRCVKVLADIHEGFVTSLRWAPGMSKSTASEGAEAPN

GDANGTPSKAGSAGAAPLETQIRCMIATGSVDSKLRIFAN

>SoG_03649.T1

MHYKQALIAISVAITGAQAQCSGPAINAAALDLIKSFESWQPDIYTDPTGNPTVGYGHLC

ADSSCSDVPYPIPLSESDGEQLLRDDLTNFQNCITLDTTSNVVLNANQYGALVSWAFNVG

CGNAKSSSLIRRLNNGEDPNTVIGEELPKWNRSGGQVLPGLTRRRAAEVDLAQTDTSDPA

LPACS

>SoG_03662.T1

MSLTSLPAISSLSSSPEAAQIQVLDALFEPSPAIHQTLLPVLRGRIFASYPILIDACYEQ

LVTLAGPSPSSPSSPPPSSSSSSSSSPSPALLSVLGSHPRLGAKKVDSAQSAAEQANLQG

QGEELAKLNEEYEAKYPGLRFVVFVNGRGRPEIMDIMRTRISRGKYDLEVEEALKAMRDI

AKDRAKKLGAVDS

>SoG_03682.T1

MKALSLRAVGAFLLVLNHPAQGAGVRPHQPFQSPGRHPTAHQASGNRDPVLIPILTKLRD

GAIELLFGRHPSKADSEPPSFPDVRALYNNEVVLRFNVTTFDEEAALAEAAARLFLDIWA

FTDEFVDIRLHVDAVGPLLKLLPASLQSSHSTLIADLPAAVYESLSSSHGSELAASNNEP

WDVSANVQSGDNLFFQDYQPLPVIGRWLRLLEAMFPSYVEYISIGESFEGREIPALRVGL

SSMSDPAEPRKTLVVTGGLHAREWISTTTVNYLAWSFITSYGKEQVITKLLEEFDIVFIP

AVNPDGIEYTWKVDRLWRKSRQDTNFRFCRGMDLDHAFGYEWDASHIQSDPCSESYGGEQ

PFQAIEAQQLADWARNQTQHNVKIVGLIDLHSYSQQVLYPYSYSCAATPPNLENLEELGA

GLAKAIRLSHGEPYSVTSACEGAVAAEEAERGGLGLRLESGGGSAIDWFYHELGAHYSYQ

IKLRDTGSYGFLLPKEQIIPTGEEMLNALKYFGDFLLGNNGIEKHFGATTKGSDGSGKLE

KVPVFETLDETTGQELRRRRLRR

>SoG_03685.T1

MAWLTSLGQRLDLPPAVVVPLAVLVVFSAVHFTRAVFSRHLQKKKKKKKKTQASSSSPST

PADYKFPRPEPYNGWSIETTKPLPYRAFRYGPKYAVTMGLRTIQPEDWIELDNHYPKYHA

DKAARLLERQDKCVHTTPEAYPAAIELLDELAEYLPARYPTLFRRTAVGIDNLWSGESFN

ITERPLKDDPMAIACRLLQDDLAIMMEGPDGRYYLKAGAILLAGFWRLSDKLGMPLEEIH

TSGDVPHYNEKLHRGMASFFSRLKCDQLYGRNNYFIQVDDSLAWSWSIGDEDSPDVSWST

AEKDRAVAHHWFRSERQSLRRLPKTRAVIFTIRTYFHPVTDIATEDYVPGRLASAIRSWD

DKVGAYKGREKYERVLLEYLDAEHQKQLDRGLDMEKEDDVRKYPW

>SoG_03709.T1

MASSSATEEHDIVIIGAGLSGINAAHLLRLQMPHRTFTVLETRDRVGGTWTFFKFPGFRS

DSSMSSFGFQWHTWPYDTKVGSAEQIATYLESAVDAEPDLREKIRFGHKVVSMEWETGRA

RWRLDVETGGEKLVMDAKFVIACTGFYDYDKPMEAPITGLDNFAGEVVHTQFWPEGLDTT

GKRIAIIGSGATTITVLPELVKTAARVVQVQRSPSYVASIPTVSRMEQFLKLFLPLSWVH

RTGWFTNTLYELVMSEFVLAFPNLARTILRKGMKNMLPKDMDVDVHFNPSYAPMRQRLCM

TPNGEYFDALKQDNCDVITDEIETVTKDGIKFKSGQELEADMIVLATGLRLQLLSGLTPK

VDGKPIRSGEKFAWRGCMLTDLPNMAFIIGYVRTTWTPGSDLMSKIIIRLLQHMERRGAD

VAVPRIDEEVARTAEKKLAVDLSSNFLLKAADRVPKVTGNGVWYGRENLAKDMMALLFGD

MDEGMVFSGPGYDSTKRAKRE

>SoG_03713.T1

MRSIAHLSGLLGLISAAQAHMEMAWPPPFRSRNNPFTTSIDYNLIDPLRADGSNFPCKGY

HSLMDTAQGRPVVTWEPGQNYNFTIVGTATHSGGSCQASLSYDRGNTWTVIQSFHGGCPL

RPNWDFTLPVDTPAGDALFAWSWFNRVGNREMYMNCARITIKEPASRRSESEAAGVARRG

NTGTAFSSRPDMFVANVNNGCGTREMFDVVFPDPGPNLSGVSDRPAAPIGNCGRQVGNTL

STSTTNAPTATVSSALSQAVRTSTRTSSSAVSNPTGAAKLWEQCGGANWSGPMRCDAGLE

CNRQDEWYSQCIPPTKKSSSVPVVSSSHTTTSAAQATVPTIRTTTDVVAQTTSTSKPATT

SSVNPPSLAAVWGQCDGTNWNGPKACQPGLVCNRQTEWYSQCIPA

>SoG_03717.T1

MHFTSFLSLAALALPLASASGNKTEPKPDKDGKYWLKTDHMKLGFVPYGAGISDLRLNDK

HGIERDVVAGFDNATYYTVDRQKPNFGIVPGRYANRIKNSTFEIDGKWYNVSANDNGGLD

TLHGGKNGWGLRNWTVVSHSDNSITFSILDADGENGFPGDVLSYATYTVDGWDWDIKISA

LSLTKRTPIMLTSHTYWNLDGFANNETQSAMNHTLHMPYSGQRVGVDNILIPTGDILANA

KGSVNDFWSKPRQIGASFGEKELEGNCGFNCTGYGEYSSFPPVRR

>SoG_03718.T1

MEKLTRSTGGCDAIIADDRDSKDTVGRTDSLLCPDISYPSRERVVILGSGWAGYAVARSL

DKAKSSRVLISPRSHFVFTPLLASTAVGTLEFRAAIESVRRLGLDEFHQAWASHIDFNRK

TIRLEANLNPDAGARTDVPLQKGPEYEIEFDKLVLAVGCYSQTFGIEGVKEHACFLRDVG

DARNIRLNVLQAFEKASLPTTTDEQRKKLLHFAVVGGGPTGIEFAAELHDLVHEDIEKLY

PGLGQFISITVYDIAPKVLPMFDQNLASYATDMFSRQGIKVRTAHHIQRIRREGDVLLLN

IKEEKEEVGAGIVVWSTGLMQNPLVSHLTEKPIPGIGQVVKDHKSGSLIVDGHMRVQVQN

PSGKNEVLPDVFAIGDCAKVEGSSLPATAQVASQQATYLAKRMNKGDIGEGPQFKFRNLG

SMAYLGSWRAIHQGSSDELKGWPAWVLWRTAYLTKSMSIKNKLLIPVYWFTTWLFGRDIS

RF

>SoG_03738.T1

MKTLLATSFLIAATAAQQPLWAQCGGQGWTGSKTCVAGAYCRFSNEWYSQCVPGTASSTT

LVTSASSQAPATSSRAQATTTTTTRAPATSTQGPGSGSTAIPSANGTKFTIDGVTKYFAG

TNCYWCSFLTNNADVDLVFDHLQASGLKILRIWGFNDVSSDPGSGTVYFQRLASSGSTIN

TGANGLQRLDYVVSAAEKRGIKLIVNFVNNWDDYGGMKAYINAFGGDHNGWYTNSAAQAQ

YRRYIQAVVSRYASSPGIFAWELANEPRCQGCSTDVIYNWAKSTSEYIKSLDARHMVTLG

DEGFGLPGDTSYPYQYSEGVDFVKNLKISTLDFGTYHFYPDHWNVPASSGGKWATDHSSA

CAAAGKPCLFEEYGAMGNHCSLQRPWQLASAAAVGSGGDLFWQFGDTTSSGRSHDDGFTI

YYGDSDWQCLVTDHVKAIG

>SoG_03746.T1

MKFTSSTLALAALAQLGSAHYFFDVSVSNGSPSRQWQFIRQFTRATAYNPIKFSSNPAGD

IRDNSHADGDDIVCNQGAFSNAGKTGVLEVAAGSSVTVKLGVGAKMEHPGPGLVYMAQAT

GNNVKAFSGIGADWFKIFEEGVCRSGGDFTKDAWCTWGRDTMTAKIPADTPAGEYLMRFE

HIGIHRSHVNQPEHYVSCVQVKVTGSGTGKPGPTFKLPGGYKASDPYANFSIYGGAKAFP

MPGPAVWTGGNGGGSSGGNTGGNTGGNTGGNTGGNTGGNTGGGSTAPPSDGGNDGGNEGG

NEGGNTGGGSSNPPVNPTNCAGKWEQCGGAGFTGPKCCSSGSSCKAANQWYSQCI

>SoG_03749.T1

MMASHPLFAVLALFSISCLAITEAFYPSSKRGLIFIPNANFPSDDRVWVQRGSDLTWYYN

YKMYPSRIYENNTSLQFVPMLWGAPSSFEDTTFLQNVTAQIIGGRNITYVMGFNEPDNSF

ANGGSNVPPEAAAKYWIKQLEPLRKLGVSLGAPAVTGAPSGFTWLANFTAACNGSCTFDF

IPIHWYGSFDGMASHIAGVLDVYPGKKIWVTEFALDFSSLAATQDFFQTSVKYLDSNK

>SoG_03750.T1

MSTEELTKSDRRPPRTPNESSSRRRLQERPYFHSRRIKKGTVERPELTIKDPRRVWVTAI

PLLGVAVGLTAIGLLSWSGYESVHNYKYCQVFTDDFSRGFNSSIWSKEVGVGGFGNGEFE

VTTNDDENVFIRDGYLVIKPTLQTDKFLSETTKINLTADGTCSSSASQDCVQVANLTAGE

IVSPVKSARISTKKSSVIRYGRVEIVAKLAAGDWLLSKLMMFPAEDYYGSWPASGEIDIG

MVRGNNYTYNNGQGNQKVQSELHWGLDTSTDRWVSTSGSRNALLTTFHQDFHTFGLEWTR

NYLFTWLDHRIAQVNYVKFDNPFYKLGGFHETFANGSRTSNPWAGPGTSDTTPFDRPFYL

IIALAVGGTSGWFPDGVSGKPWSDDSVSPRKDFWNARNQWYPTWDRNSGGEMAIKRVSMW

QQCDRAATDLSQFAQN

>SoG_03760.T1

MKYSASFFAGVSALLFGGAQAQYTLSKTYDASNFFDSFNFFTGSDPTHGFVEYVDGPTAN

ANGLVGTADGAIFMGVDKTTVNPPNGRRSVRLESRDTFTKGLFIVDASHMPASECGAWPA

FWMFGDGSPGWPQQGEIDIIEGVNNQVSNINTLHTGPGCTITNTGTTPSTHLEGADCNAG

NANIGCGQLTSNNQNYGDGFNDIQGGVYATEWTSDHIAVWFFPRGAIPADITSGNPNPAS

WGLPQARFNGGPGCNIDQHFYGHKIIFNTTFCGDWAGSPNVWNNNPECSAKAPTCKEHVA

MNPGAFEQAFWKVNYLKVYSANQTKRSSVPTAFRA

>SoG_03766.T1

MTELSDSDLRTLVSQLTFDEKMSLLAGETMWETAPVNRLGIPSLKMSDGPNGARGSSFVD

GTTAACFPACVSLASTFDINLARRVGVALGQETKTKGASVLLGPTICIHRSPLGGRNFEA

FSEDPLLTGNMASQYVQGLQSERIGATAKHFVANEQETRRFTVNETVSERALREIYLKPF

ELVIREAMPWCVMSSYPKVNGFYIDSQPNFLQHVLREDWKYDGMVMSDWGAVTNAEKSIK

YGLNLEMPGPPTKRKPDCVKKALDEGRINTDDIDECVFRSFQLLSRVGKFADKSDTSLER

AINDPEHGKLIRQVGADGIVVLRNERNVLPIKRTLEKIALIGPLAKVAAAHGGGSASLNS

HYKVSPYDAFIARLPDHCITYSKGCHIFRALPDFKENLFNKNGSPGFLAEFYKNTDLVGE

PFHSQELRRGSFNTAMDEGPKGACGARITGTYTPIITGRHCLSLSGVGPSRLYVNGDLVI

HQEHEISDAMLFMLGAQDEKRLAYQFEAGQEYEIVIETLVSPCKNSDLPLLENLVAVHLG

FILQEEYEADLLSEAIAAARNADVAICFIGHTAQWETEGQDITSMTLPVDGSQNRLVSEV

AKVNKNTIVVLTTGAPVELPWLSEVSGLIQAWYAGQETGNAIVDILTGEVNPSGKLPVSW

PKKEQDTPCYGHFGMDSFESREVQYSEGIYVGYRHYDRTYGSENEVQFPFGFGLSYTSFA

LLKTTVTGQLDEGDSTCEITVTVTVKNTGAVRGAETIQLYLAPPEDSTSDRPPQALVAFS

KIFLDPGEQGEIDMSFKRDGAAFWDDSTKSWAVEKGAHELRVATSSHPRDVAAREVIHIS

AGFHYVA

>SoG_03773.T1

MDEKTWRATFGSGHRLRELDEKMHAAGKRAIAHGTCPSVGIGGHATIGGQGPMSRMWGLT

LDHVVEVEVVTADGKIQRASEKENPDLFFALRGAGASFGIITEFVVNTHPEPGSVVQYSY

HFSFGRQKEMADVYRAWQALHADPDLDKRFSSLFLAEPLGALVTGTFYGTEEEYKNSGIR

EKLPTAGRIEANITDWLGSLAHEAEVAGLSLGELPSSFYSKSLAFRKEDFMDDASIDALF

DYMDDSNAGTLLWFIIWNSVGGAMNDVKQDATSYPHRDKLIMYQSYAVGIPSLSRSTKNF

LEGVHEHIQKGAPNATTTYAGYVDPDCSREQGQKLYWGDMLPELRKLKKKWDAGNVFSNP

QSVTAGE

>SoG_03775.T1

MKFQSSLLYAGLAALGVLASDKLTPDAVEADIRQDKLQNVLWNFNKIARDNGGNRAFGLP

GYNASMDFVLERAVTRFGKHMDTYVQSFNHLFEQTREISLTGPDGEKVRVATLLYNDPTP

LPDGVTAELVDTPVDDTRGSACFEDQWAGVEASGKIVLIKRGACAISDKLKLAKNHGAVG

VVLYHNVPGDSLTSATLSAENVGLKSPVGLVTLETGNAWKARLAAGETLKVNLLVDAIFE

ERPSWNIISETKEGDPNNVIMLGAHLDSVQAGAGINDDGSGSAALLELMGSFKKYKGFKN

KVRFAWWGAEEVGLVGSLYYGSQLSEAEADAIRIYYNYDMIGSKEPQYAVYADNEVHEKV

SQPLVDYLKENGADAFYGGFGSSSDYVAFLELGIPSSGIFTGAGAPYDSCYHQKCDDIDN

INWEALTLNAKAAGRVAAHFANDLSGVPAREKTSTNPRSKRGVARSLQKWDRASRVAQKH

ATCGEARNRVV

>SoG_03778.T1

MTPRGSTPAAPPQPSTKAAPAAPKLNNELEMDSMPTEGGAQKPKQDDVMQLARLGDVAAM

EKLFETGGFDATYTDDEGITPLHWAAINNQYAMCKFLIEHGAEINKKGGESVATPLQWAA

QRSHYYVVNLLLQHGGDPLITDAQGYNTLHISTFNGNVLLIVLLLHQGIPVDVVDSYGHT

ALMWAGYKGFPLCVDVFLRWGANVQLTDEQGFTALHWALVKGSPACIMKLIEYGADRFAK

THSGKTPSVTASELNTEGAWHKALRECGYDDDGHSLTPPWPGASYFLQDKKGFINRFMFF

CPFVLVWAVISVLAHAPIYLGIPFAFIVGYGILWIAQQVLEYAPSGMGALHKTPWMAGIF

SGSLTLVAVNWLTTILPATTFWAANSNETHPLLNFIFAIFLGLTGFFYVASMRYDPGYVP

KMNGIAEQKAVIDELLKEWKYDESNFCTTCTIRTPLRSKHCKACARCVAKHDHHCPWVNN

CVGVNNHRHFFLYLICLTIGILTYDWVLYYYFTARSASASDSCNVLSPKFCSLLNADSYT

LVLAGWATLQLTWVTMLMFTQLVQVSRAMTTYENMYGIRSVSSTTAFTSTGTPLDPNHPS

LSANTDAHGGHGHKHGGGMFKRWSRLLGVDPFIETISGRGAATGKNKKKKNPYSRGCISN

CKDFWCEPTPIFGQKENGSAVLGGERVDYTMMYESPTLMNLASGRRARDGYEVVGTEEV

>SoG_03795.T1

MAKYLLGLLCLAAIAPAVLAQSPAYGQSSSGGGQGWSGATTCVSGYCCQFSNDWYSQCLP

GSCNGGGGGGGGGGASSSAPGVSTTMATRTSAAGGSNPTQSFTNPVLWEDLADNDVFRVG

SDYYYTSSTMHYSPGAPVLHSKDLVNWEYVGHAVPSLDWGNKYNMNGGRAYVNGIWASTM

RYRKSNGLYYWLGCIDFGTTYVYTSPSPSGPWKMGGKINTCYYDAGMLIDDDDQIYVAYG

NSQISVAKLSSDGFSQVSTQRVFTTPSSIGTLEGSRLYKRNGQYYIFMTKPANGQYVIKS

SSPQGGYGSAFQVLLNMRSPVSGSGIPHQGSLVDTPNGDWYYMAFIDSYPGGRIPVLAPI

TWTGDGWPTIQTVNGGWGSNYRYPVTPVNLGSTTGKDSFTGSSLGPQWEWNHNPDTSRFS

VSGGLTLSAATVTNDLYSARNTLTKRIHGPQGTGTLELDFSNMADGDRAGLALFRDKSAW

IGIARDGSTNKLQYVTGLNLGTGNGWPTQGTGSVSATQNISGTKVWLRIRADIAPGGSKT

ATFYWSTDGSSFSQLGGSFTMNSDWQFFMGYRYGIFEYATKSLGGSVKVVSFTSA

>SoG_03797.T1

MSAEEDLIDYSDEEIATNETAAASNGKKGELAAGGNVDKKGSYVGIHSTGFRDFLLKPEL

LRAIGDCGFEHPSEVQQTCIPQALLGGDIICQAKSGLGKTAVFVLATLQQVEPVNGEVSV

VVMCHTRELAYQIRDEYNRFSKYMPDIKTGVFYGGTPIKQDMETLKNKDTCPHIIVGTPG

RLKALVRDKALRLGSVRIFVLDECDKMLDQPDMRTDVQDVFRATPTQKQVMMFSATLSEA

IKPICRKFMQNPTEHYVDEDTKLTLHGLQQYYIKLEEKEKNRKLNELLDDLQFNQVIIFV

KSTVRATELDKLLRECNFPSIAVHSGVSQEERIRRYKEFKEFKKRICVATDVFGRGIDIE

RINLAINYDLSSDASSYLHRVGRAGRFGTKGLAISFVSSDADQEVLKEIEKRFEVALPEF

PKEGVDASTYMAS

>SoG_03806.T1

MTPPEPIDENQPLRAIRPVHHGDQNSPESIPPLQSPKDAHAHSSGGGRFLREPLSQSFSG

RRSASQSPFRLEMPALSPAQLAFSAMQYLPVPVMILNNLKTVVQANEAMARMLDLLPEGA

EEDSTIVLEKLRGQTLSQVGIDMLQDGRPVWLTWEAFFDAMVDEMGIRGVSGDVRRESPN

NGEATPTATVKASSPSTGDPGSPTTSHAQNAVVEVVISRKSIAKPNMTSKLNDPSANHHI

YAKMIINIWEVEDRQPFFTLTFTSSQSRPSTLIQSRKSIARPSLLEAADRPSITNSNSSS

VASSRDSNSPSFHSPGVVTMSSSPFPPMGPPAVASQSSTPSLLQKMMLIKDALLDNTEMP

ILAMWKDGSAMFPNKAARKVFGSQIDGSLSSDGFAMIERWQMWNEDFTRQLEPDELPISI

LIKTETPFSNMRIGTHHPDGHKVVFDVLGEAIRDDTTGEFLAGVVTCRDITTMSEEIVQI

KERDEEREEHFRLICDTMPQMVWTATPDGMHDYFNSGWYEYTGLTEEQSLGLGWQIPFHP

DDLDEARPRWLRALKTGEAYEVEYRCKSKEGEWRWFLGRAAPARNKETGEVEKWFGTCTD

VHDNVQTKIAAAQTREQLLSVLKHSRVTLFTVDTDRHVTLLEGSLIWDKAEELSKSNRWF

IGQNMYTVFNRLTDQLPEGERPEFLQPIEDILDGKISEGVREHRIDEQWYRTRFIPYHSQ

GKAISQDTTAGVIGVIMDVTEIRRREEALEKQSHEKRKAMAAEAAAKEATRLKSQFLANM

SHEIRTPITGVLGMAELLGGTQLDESQRDFVDNIQSSATSLLTVINDILDFSKVESGRLD

IEEVQFSLSLIVKEVGKMLQFAVKRKNLDFRSDIGGDIKNDMVVIGDPGRVRQIITNLLT

NSIKFTNQGFVRFSVAKERETADSVEVRFTVEDTGIGIQEEVRKRLFQPFSQGDASTARR

FGGTGLGLTICKNLLDLMRGRITLRSTLGKGTTATFWIPFHKPSSPQNSKTVQAGPLPDR

LQSELSLSCNSSEHEHQGRHSPASSDVVPSPTTSRARQLKSDVPEPAPESSKVDRSKILV

LVVEDNAVNQKFAIYAITKMGFQVKALWNGKEALDYLMGVIDGKNVKPDIILMDVQMPNI

DGYKVGNACADLNPSLVSDMHQCTHILRHHMPYKALVHDVAIVAMTASAIYGDREKAFKA

GMDDYVSKPVTRGIIEHMITRWTTMRPRKTISSAATDISTSDCSELSEQCENADIPGTGI

EDNAFVYGEDSPAAFDPNSPITPRPLTTNGEPEPSPFDSLGSELEVHQQQQVRRQEGEKE

WTSKLQENKMLEAAGGPMAVRASQRVDDEGQGEKLTEENMSKLEEENEHTR

>SoG_03821.T1

MATSRSRNPSQQASREDYPMMDRSPRASQSHNHGSGLPPPTTFTQYKRRRGTETKEARVP

GRSMIQPAGESGRRGFHPLKFLRISFRSASRASLVCNVLWPVVPAALAVRYALPEKHVLI

FTLAYIAMVPCANLVGFAGQELARKLPHVAGVLAEITFGSIVEIVLFMVLLSKDMFEVIK

AAILGSILATMLLCLGLCFFAGGLWHDQQTFNEAISEAGSGLLLTAGVVLAVPTAFERGL

SSSSISPGAEQLEIKTLNISRVISIMLIIAYCVYVFFQARTHHGIYTTSYEEDEARDRDG

HKDRAKDKLTFIEAAIGLAIGITLVTLIAITLVLQIEFIIEESAVSDAFMGLILVPLVEK

FAEHLTAIDEAWDNQMNFALSHVLGATLQTALFNGPLAVIVSWGIGSTLDLNFDLFNLVM

LLLAILTVGRFLQDQKSNYLEGILLVVLYIAIAVAAFHYPDPPHGGGASAEGGH

>SoG_03831.T1

MLFQGYLFALLCAVTSWYGMADAAPSELVKRASLTQVQNFGTNPSGVKMFIYVPANLQAK

PPIVLVLHACQWTAGAFFGTTKYGQLADQHGFIAIYGQTPTDGACWDVSSTQSLSHDGGS

DSTGLANMVRYALQKYNGDASRVYVTGESSGAMMTVSTHGVPNRRGMLTDSRKQQVMAAV

YPDMFEAASEFSGEPAGCFYTGSVRGWNSQCANGQVKKSPSEWAAQVRAMYPGYSGKYPR

MQIYHGDVDNILNINSYNESIKEWSGIFGYSGQATSALNNNPGNRLTKYIYGDRLQGIWG

HGFGHVVPTNETEALQWFGIIGSRSNSPTTTAAGPPRTTTTTSASRPTSTGGSGGGGGGN

GNCAKKWAQCGGQGWSGATCCESGSTCQVSNQWYSQCL

>SoG_03846.T1

MEHLELAVIGAGFYGLAAAKQYLSQHPGSSLAVYEASASVGGVWASERLYPGLKSNNLLG

TYEYPDFPMTTERFGVQPRQHVPGEVLHSYLEAYAQEFGVAEFVRLNTKVLSAEHLPEGG

WILQIRDSRDDKAQPVKVTAQRLIVATGLTSEPFMPRINGQEEYKRPLFHIRDFQKHEQT

LQTGKRVTVFGGTKSAWDAVYAYGTRGVQVDWIIRPTGHGPAWMSPPFVTPLKKWLEKLV

NTRLLHWFSPCIWAQDSGYRGIQNFYHRTAVGRAITNGFWNTIANDVVTLMGFDQHPECA

KLKPTAEALFTGASFSILNYDTDFFEPIRNGTVKIHEADLSHLSEGKVHLDDADGTVLDS

DAFLCVTGWKHRPPIKFLPEGIERKIGLPYVVTAADQGCPPDESLAAQSGLLEKADAEIQ

ALFPRLTVPMKFNPNYVPLIQTKAFSSTPATEAEPAASSPLSNPMLYHFMVPGTSEFLRT

KDLAFAGGVSNFSNVICAHIQGLWISAFFDGKLARDPSSAVMPMDEDAGKAERQPAMTLD

EVHWQTVLHNRFGKWRYPNDAGAKHPDFVFEAVQYLDMMMADLGLQVHRKKSWLKEMTEP

YGPEDYRNINQELDARFTKEE

>SoG_03848.T1

MEIPTTAINPSSAQHTHTVVFLHGRGDNVPNFINSLSYSRDSQGRTLADAFPSFRWVFPQ

APTRQCASSSNKWPQWFDVWNVADFTDREELQLEGLKQVVPEIRNILKAEAERLGSGDAP

AWDKVILAGISMGAATSVHVLFNLDAPRLGAFIGFSCRCPFAGRRLEEMRKLLSLEEVPA

HDQVVRNTPILLEHCIDDPLVKVEFGRLMRDTLKSFGANIFTREYPQGGHWFNSPSGMDD

AIAFLSQVLTSS

>SoG_03872.T1

MIPKSVLLNAALVATALAAPLEERQGACASQWGQCGGINWSGATCCSSGNTCTKLNDYYF

QCLPGSAPASSTTARSSSAAASSTSRAATSSRATSSAPQVSSTSAATKTTSAGSSVTTNP

PSGGSGSATWSGNPFSGVNLWANEYYSSEVHTLAIPQLSGAMATAAAKVAEVPSFMWMDT

LSKTPLMDKTLGDIRAANKAGGNYAGQFVVYDLPDRDCAAAASNGEYAIADGGVAKYKNY

IDTIRSIVLKYSDIRILLVIEPDSLANMVTNMNVAKCAGAKSAYLECTNYAIKQLNLPNV

AMYLDAGHAGWLGWPANQAPAAELYAQVYKDAGKPASLRGLATNVANYNAWSLSSAPSYT

TPNPVYDEQKYVHALAPLLSQNGWDAKFIVDQGRSGRQPSLQNAWGDWCNVIGAGFGKRP

SANTGDDLVDAFVWVKPGGECDGTSDTSAVRYDYHCGLADALKPAPEAGAWFQAYFEQLI

QNANPSFLIAPAVATIQREQHPPAYNTSKRLDSRRNLRTWFCHQLDSGSAPAASACPVLL

WYFFSTCHGLQRAKQMLSLLSVITALGLHSPGVMAADTGGDTSGCGKSHAGNETQAWSLM

SSGMNRTYYVHVPQNYSSNDQHPAIVGFHGRGGTGLYFAADTRLSEAQFTGDSIMVYPDG

VDRTWAGASYSAVSVEQDLQFVWDVLAKVRSDYCVNSARLYATGHSNGGGLVNLIACNAT

VGAEFAAFAPVSGAFYENQYDGSGCKPARNVMPMLEIHGVEDQVISYKGGKGRGGELPSI

TDWYVMFPTDEIPSLLCTQREENLIMPALTKVKSCAHQVVRMGEKGWL

>SoG_03874.T1

MRLHPFLHALGSIAAFAWVASSQAANPQERPVPPSQDPFYLVPKDIARAKPGEILQHRRP

LSTFEAYGFKPPHIQEAHQILYRTADNVGNATATVLTVFIPQNANMSNVVSFHVAEDAAS

VDCAPSFGFQSAVLDYPSLSAAVMQLQLLIIESALARGWVVVVPDFQGPMGAYAANRLAG

QAILDGIRAALQSGSTTGIPPNADVVLWGYSGGAAVTESAASMQPIYAPELKLKGAALGG

IGPNATSLDQILKLNKDPRTGLLATALVGLSNQYPAFEKALDAHLDSQYREKFYSPRKEC

LDKTLETFFNVDLLGWFSDFAQVFADPVVLQIGDDSVKDLTTPETPMLWYQTTHDELVDI

KEIDARVEKFCAEGVVIDYIKDTAPNLLHKNYGVVGASDALKWIEARFEARNESTECTEK

TISTPYIDPDFLKLFPDNIVQALEQLVGAPNRNNTRTV

>SoG_03875.T1

MLMLTSQVYIVTGANTGVGKELARILYSKNATVYVAARSEEKALKAIEDIKAAAPDSTGK

LVFLRLDLADLTTIKASAEDFLSKESALHVLFNNAGVMKPAQGSKTVQGYELQLGVNNVG

TFMFTQLLTPTLIKTAKTATENSVRVVWVSSSAADAPICPTDGVEMDNLDYHKDKSSFHK

YMVSKAGNFLHSAEYARRHASDGIVSVALNPGNLSSDLWRTQGWAASAVLKSFVLHPPVY

GAYTELFAGLSPEVTLKLPGDWIVPWGRFAGIRPGLQRASKQVSDGGNGTAQKFWDWTEA

QIKPYL

>SoG_03882.T1

MRWQIRAAFLACLSSFLSATGASSLPGLRIQPLGDSITKGSLSSHNNGYRGYLRDMLEEI

STRGVDMIGTLRDGDMRDNSHEGHSGKFLADIEEYYKLSIRARPNIVLIHAGTNNMDKEV

DLERSPDVMESIIDGVHEEGPDVVILVMPIIWANDTRMQNNTDRFNLALAGIIESKMENG

FHILNVSTNLTLADLADFKHPNDAGYEKMARAWLGSIMEANSRGWINDPVPVNGDELEGM

GLGPEALSIGGEESPGSKNEGSQHPSDKHAPAKDIGKPKEEGTANSPDKVSSASNVHRSL

GAWFMYGWLATVAFLDAVA

>SoG_03902.T1

MIFSLFLTAQAALGAVLNDRQTNPSCDAKCMEAFKTGLQIDSSQWATLNFSDDPFYTTPA

NASNAKPGDLLKWEDLSAEQLLKNWTGVPGGMSVSRFLYMTEDVDRKPIPASAFVLLPLT

ASIPGCLSRTFKTIAWAHGTAGRSRQCAPSNHKTLYYGWEAPLFYAATGYAVVAPDYAGQ

GTDIPGGFRYEAGYLHAADVAYSLVAARQRIGHLLSDEWVVAGHSEGGMTAWRTNERLAM

KDQEELLKAGKFLGAVAAAPALRPIDLIPKSIKIANGGGMGAPVHVYMVQSLVEIYPELK

LEEIFTQRAIDLLPLLDQGCLIAGDMLFHNMSVTDIYRSTAWLESKEFQDWQTRINGAGP

HKLAAPMMVVQGLGDTLTYPEECEEDFDRTCDKFPDSPAELYLVPELDHGPSFEAAKQYY

MPWIQSLFEGRQIKKGCKKITASPINERYARGQPLG

>SoG_03906.T1

MRIAIREQLALLVLFAVLIALTIVSVPTWIYVNDHIATNLKDGLALTASLKAARISADLG

LIQTSCFTISSRVLIQDALSRFYQYNSTDWSAATNDIQSALSVGATTGLLQARIYSRNST

GGDPTGLLNVTAPNVAEVLLPYKDQNGQRIYLNDTAMGYPPMLYPNITYENLGRQNKVRH

NTSAFSANAFDGIPILANSPLLLGPLIINETAALLSVTIPIRDNMDTFVLGYMTIISLAN

SLIEVRDSREGLSQSGMVLLVGPVNPWNRFNDSNPPSNDTFQADRAAFAGVDVKFILPPK

TPDDTPSRHSERQYIGGSTALTFPLSDYPAALDSFVDRNSAVNNAAALLDTTNEQGVRVA

VGYARTQTPLVNWTILVEKSRSEAYEPIATLRNILLGTVFGTAGFIMLIVWPCAHLSVMP

IRKLKAATEKSVHPPGYDDSILESDYDDEPPTSGGTSQRSKKGFVANMMRIMKRKGRKPD

SHRDVARRMFKIPGKVDDRKHFITDELTELTRTFNDMSDELVKQYMSLDEKVAERTRELE

ESKKAAEAANESKTLFIANISHELKTPLNGIMGMCAVLMEDNDVTRIKQSLKVVYKSGDL

LLHLLEDLLSFSKNQIGQPLNLELKEFRLGDVRSQILSIFDKQVREGRINFSVNFLGNDD

GDFRSSWNGSADLSRLPALGPTGAGRLKDMCLWGDQHRILQVIINLVSNSLKFTPPEGKV

EVRIRCLGEHETPPEEESRASSMSKHSRTGRTRHRVGPGSTHSSSSGGRGSGAAFNGTGT

ALAINPMDPKATPHVQVRERSPTPPPITAKSYIFEFEVQDTGPGIPEHMQQKVFEPFVQV

VSGLSKQFGGTGLGLSICQQLAGLMGGAITLRSTMGVGTTFTMRIPLKYVQDRASSTASS

SIKSRPPSVDTLDPENQRHNAATPSKVPSAIATQPANIQPRLVGLSQPYFAADHPTQPKS

TEEKMAEIGRAMAKKEGQGKLRVLVADDNSTNIEVVSRMLKLEEVYDVTIAKDGQEAFDL

VKANMEMNQSFDVIFMDVQMPNVDGLQSTRLIREMGYTSPIVALTAFSEESNVRECINSG

MDEFLAKPIRRPALKQVLKKFATIPEEPETASLATRKTSPERSTGSTTSNSQQDSDFEKK

EAAVDVYTPMSDLGQTPPAKEG

>SoG_03915.T1

MLRRLVSSSSCLQILKVPTVARRAARRVNEVTSAHPASQLTAQEHRLAMGGRVTRQSPNG

ITIEQTTKLTEWILSRSTRPSLYETRSWAREQFNKWLDDEFVEKFIDSVIQPTVMAHYKA

VEEQAGGTELQLPGFGLPVKSMPPKCERKDGPPAHFPVLGSDEDGWKATTLLIREICMLS

AVEELTNKPEWWLKCRNPDIANKWKEEALAMDWAARRQYADFTPAMADAVLEELRHKADI

YEQTGLIPVLDYCIAAIKSDNLLADDLVAELKAAVAPLESVPEEQKDWHPGSNNQVLDLV

HPSLWPLVYGKTRILPRGRVGIDDALDLCGSGVVVPEPLPIEFEEGEYFSRRFQWLPSDV

RISPEGKAEIESYVNNLHPVEHAALYPVLNKLIEKALPAWDVLYRWPQEFEMQRLTTTKC

GVACPDPEVCSANYMCMPANRPIGDDEPPRVEDEQWEDDYEESERGKLDMAWFRATHPID

LPDVDPSRLPVRFQSKDVRKSGFFDSASRIQVIVKLANIHLTPENPSYEGGSWHIEGLQN

EHICATALYYYDSDNVTESRLDFRTRADREELSSTVEYEQGDEYTIQRTFALDTKPIESN

SEATSVQLIGSVVTRPGRALFFPNLFQHHVSPFRLADPSRPGHRKIVALFLVDPKIPVIS

TANVPPQRQDWWKGATHPEGPMARLPAELRNMVNDQVDFPYPKAEADRLREELMKERSAS

VKEFDEHLTNLAYSFCEH

>SoG_03920.T1

MEWPIRDIVYTAIVGVVMLMACLEWFLWLAAFLYCLIKVFQKSEHWSINVLCILVGVAFT

LLRCIFLPIMIVTLPLPSQVVRYWPEPMVAFLQWFAFWSFAILLTVPWLFCIYQLVTNQL

GRTKRIKQVLDDVTAPKVVIVMPCYREEPDVLVTAINSVVDCDYPPACIHVFLSFDGDQE

DELYLNTIDKLGVPLTLDSYPNSIDVVYRAARVTVSRFAHGGKRHCQKSTFKLIDKVYQE

YLKRNDNLFLLFIDSDCILDKVCLQNFVYDMELSPGNSRDMLAMTGVITSTTRKHSIITL

LQDMEYIHGQLFERTVESGCGSVTCLPGALTMLRFSAFRRMAKYYFADKAEQCEDLFDFA

KCHLGEDRWLTHLFMIGAKKRYQIQMCTSAFCKTEAVQTYRSLIKQRRRWFLGFITNEVC

MLTDWRLWKRYPILILVRFMQNTIRTTALLFFIMVLALITSSKKVDDLPVGFIAISLGLN

WLLMLYFGGKLRRFKIWLYPLMFIMNPFCNWYYMIYGIFTAGRRTWGGPRADAAVADSHT

TAREAAEQAEEKGDELNIVPETFRPALEARRAGIRTEPQGTAGVVRKRSVVRPPDTIDGK

FSARRRTAAGVYAYSDETGRTGTDGAMGSSRKHSEVTEPPRAAWEGDARYSLESLVGEPN

WTRMEDYMGEEDRRKYAMAQRAQQSRGHHRTQAVHLVGPPSTPRRPEASGQHGKRKNVDD

QYGSSMV

>SoG_03933.T1

MVVPRAVRLLSLLGAFACIQPAVIASPLLDSILGPIGDVVDTIGSGAGLIQGTLGGLQGI

LGQSQSYDYVVVGGGTAGNAMGVRLAEAGFSVAIIEAGIFYELGKPVLGTTPTGAFFGVG

TNPLDTIPTTDWGFITEPQVGAGGRRIHYAQGKCLGGTSALNFMVHHRAPTGAYDMWADA

VGDDSYKLDNWMPFFKKSVTFTGPNNDLRWANVSTTWDQSAFAADGQGGPVQVTYTNYVS

AFATWMEKALGSLGFKKTDEFNNGGLLGYHYAQATIRNKDQTRSSSAAYIYSAMSSSSTS

PAKKNLKVYTQTLAKEIVFDGKKATGVKVSLLGALPTYTIKAKKEVILSAGAFKSPQLLM

VSGVGPKATLEEHGIPVVAELEGVGQNMWDHIMFGPSYAVSLPTLDKTVGDPVTLAQALL

DYTLFAKGPLTSNVADFIGWEKMPGKYRSTWSDATREALSRFPDDWPEVEHLSGNGWIGD

FGWPAIDRPHDLKNYATDLGAMVAPLSRGNVTISGRSTLLPPRINPNWLTHPADQELAIT

WYRRIREVWDTPAMRSIRTDGDREAFPGRENETDEQILEVIRRSLITVWHPACTNKMGKP

DDKMAVVDSKARVYGLEGLRVVDASAFPILPPGHPQSTIYALAEKIAADIIKG

>SoG_03935.T1

MAPSSDFAAGVSRRVSELVKRINIPLTPTSPPGLVQANTVDPFIKSFKYGLGWTYFALGL

MALVMIMRIWHFWQDKIRQAIYKQKLEEHYRDVYNVDASLYLAAMRTGTAQHFFPEGEGM

GDKQFKPKAHFSSVTIINDILALFRWVFYRPIPDLIIGKHRITFSSLAVLTSVFVATAFV

TLYCFLQQPLYWQSIQYGSPPLAIRSGMMAVALTPWIIATSMKANVLTWVIGIGPERLNV

FHRWMGYLCLFLSLVHTVPFYIQPVWDDNGMEVFQRLFAGGSGYIYGTGIACLVPLVWLC

VASFPFIRRIAYEMFIILHVPVAMAYVGLLFWHSKNYLMSWGYLYATVAIWVFCYIVRFF

KLNWTRPGRLSFMVGDEAAITLMAENAIKITVPTQMRWKPGQYVYLRMPGISLFENHPFT

IASLCSEDFPSEYGEKYRDLQVVFKPYGGFTRKVLETAIEKGPFHTYRAFLDGPYGGMRR

DLAAFDTCILIAGGSGVTALMSQLLNLIKRMRDGKAITRKVVVVWALKRLEAMDWFREEL

RICREAAPPESVTCKFFVTSAVRNRGQQMTGPLHNPATTRALSHIFHDKLDGFVAGIASK

RNSALIQAEAGGDAAREQELRAEDEDRITALPRQKYLQPHQMPPQQHEINTQHHQYTPAE

ESLRKLEGLDFPEDKKPHLTIPTGGDGYHFPAMKKENSPHFNYAPSSPNKLPGTTPTQDD

FPVRAPEFAHLRNSSNLSAADRQRPTSTFGPPSGFDFGFPETPTEFQKNLMRFAFPVPHQ

IDGGWSVEYGRPDLGYMLKEWATGGADGRGILGRRTAVFVCGPPAMRVGVANTVARLQAE

IWGDPELEEIFLHTENYAL

>SoG_03944.T1

MAMIHWIKGRSVIRTLCAVGIIWLVLHIYSHSHVSTPRLPYIPGTLPHTGPDPVKGSGHP

IDDLIRDARARFATALEKQSETLEEAADKYRDRRGRHPPPGFDTWFKTAKKKNAIVVEEF

FDRIYHDTNPLWALNPLQMRKAVRNQPFMVRVRNGKAFPEVESHDVPYRVEQWLKLVKEM

MPHIPDLDMFVNVMDEPRTLVPFDTMKKYVAEEQKKRKLIPAAEAVNKYSGLGEAKDWKE

WHDPHWVTDEIAKYWDHYEVTCPPGTPGHGKKSLEKFNGPVDFPTEPIAAYTYKGYIQNW

TAAHDPCLQPHLRGMHGTFIDSISMQSTKELLPMFGECKLPGNSEMLIPGAVYLDNWKKY

SGGGVQGGGWASKKDVFVWRGSSSGARNKPDNWWHLHRNRFVQMLNASTVQAVEDGKVDV

GRSFNLDASKKYDTPALKQGKLGEWLARVADVGFTDMLCHPFEYWHHWWYGKQQKLTCSW

DQPFLSLAEPIPMGKFYNYKYIPDMDGMSFSGRWRAFLFSTSMPLKSTIYAEWHDDRLFP

WVHFVPFDNTYMDIYGIMDYFVGDGLGRGGRDEVAERIALEGKMWAEMVLRREDMLLYVW

RLLLEYARVTDDNRDKLAFVGDLKR

>SoG_03958.T1

MILGPVSLIDCLVFCIFLAPQLLLQVGVCGTISTTLRCLPFLLLRLPYEFAKERYLTPRP

SQSRFVQDATAFEDLVIRCVRYAFANVPASVGRVFFSKRVALPFVRWRMLRHGYWTYPVH

VEETTIGEDSPKTRGVWIKQDPEKTPDVVIFYVHGGGFAMGSSYFYLEFLMALHHLLLDA

GYTNPAVFALEYILVPDDVYPRQVIQTLQGYRHILNIAGGQSRVCVGGDSAGGTLVLSLL

LELGAQSRSGKKRGGLAIPQSIPKLRLPDKVILISPWVTLLSSRHHESTVDFLDRSTLWR

YAHAYAGDGMLQQAPASPGSCEDNELWQAAQPMEGWLVAFGDQEVFAGDALDLIKRLRKL

GQDVRVHKVAGGVHAWPVASLFLSSTVDKRLLGLRTIVDEVRRRRYNDSMNVEKM

>SoG_03962.T1

MAPTPAAISSLLRQTIYYHLDNSAYENALFFSERLAAQDPRSSEAVHLYALSHLRLRDFR

SAYDVSKTTAYRGTHIGCAWVFAQACLQLERFKDGITALEKSRNLWSQENSLAKHSAHSR

AANPDAAAALCLLGKLQRAYGDKKRAISCFEEALKMSPFMWDAFTALCDMGINVRVPNIF

RAGEHLLASLDQNLSHLPTKETGLAEAPNKKATTRSAIADLTADPFSAMGSDPSVPTIDM

FVEPAETDFMSKIQAAKLRLASSTNSQPGLDNMETPTSNYSADTIPARHNSSHEPPQAPI

RRIRPATVDSGAEAPPRMNYRLGAKKTGRTLERGNDDAETHMSEAMNPPPLAQPARSAIP

AVERKRTVSGHPVARPTAKIDEHITRRSARLNMFKSSVKANSGATTIGASAGRELKKARP

QISRLVRPGSSGPSVGRVVSGNRKPLEDQNGADVDHGETARVREAPLQTVQAAPTVQAKI

AELDSVKLEEALRWTLDLLKKLGSGYYHLSRFQCPEALQAFSSLSTAHQNTPWVLAQIGR

AHYEQAAYADAEKYFRKMRVQAPSRLQDMEVYSTVLWHLKREADLSFLAHELVDSAWHSP

QAWCAVGNAWSLARDPDQALKCFKRATQLNPKFAYAFTLQGHEHVASEEYDKALTAYRAA

IAADRRHYNAYYGIGRVQERLGSYDKAHTHFQAAQTINPNNPVLICCIGTVLEKLQQVDL

ALQAYTRAAELAPRAPQTRFKRARGLLAVGRVKEAQNELMVLKDLAPDDGMVHFLLGTLY

RSMNEKQLAVRHFTIALALDPKVGFFSSAPSPQHVVHALDHVAYKILLLGGSED

>SoG_03976.T1

MAAKAALPLQEKEGTPDHQNPAVIRRNTLPPRSYFIPETSLLLNGKWDFCYTATPEESPS

PDDSQPEDEWGTIDVPGHWQLQGHGHPHYTNTQFPIPVCPPHVPTENPTGCYKRRFFVPS

DWHNGMELRLRFDGVDCAYHVWINGALIGYAEGSRNASEFDVTSFVNKDGPNEVFVKVYQ

WSTATYIEDQDQWYLSGIFRDVHLIALPSTNRISDWFARTDFDADYKNAKLLVSVDVLAS

DSGDITLTLSELPRNGGGVVQEAKATFTAEDCKVNIEVDVAEPKKWTAETPYLYHLEIAF

GSHKVHQKIGFRKVEIKEGLITVNGVAPRFYGVNRHEHHPRFGRAVPLEFIKKDLLLMKT

HNINSLRCSHYPPHPALFDLTDELGLWVMDEADLETHGFYDAIARPLDIPEEWDYEIRKA

KTNGPAAAYTTDNPDWQHAYVDRMVQLVNRDKNHSSVIMWSLGNEAFYGQNHKAMYDWGK

KFDPSRPIHYEGDAKAISADMFSYMYPPLDRLLNLCKTEGVKEDGSYEKPIVLCEFAHAM

GNGPGGLDDYVEAFENNRRLQGGWVWEWANHGLWKEDADGKSYYAYGGDFGEWPHDGAFV

MDGLLHSTHEPTPGLIELKKAYQPLQLSIKEGKLHIKNKYDFLGAQHLVASYKVEDLGAK

SSVVAAGALDLPDIPAGGSVSVDLPQSKFPAGSQNDLHITVSFRLRDSHDWAEPGHEIAW

VQGQMSDPASAEAPAIAPRSALTAPLTVSKARAQATVSGSDFSFVFDTARGLLASWTAGG

RAILEKSPITSGAIFPGIYRPPTDNDQPKDHPYWLHYGVHKMTSLLRAMDIKAANDKVVV

TTTTFLSPPILDWGFDVKSIYTIVPSGTLTVELTMSPNGKKPDYVPRIGLDLRLPKDLSY

VKYKGLGPGEAYPDKKSAQRLGVYSATVSELHQHYEVPQEGGNRMEARYVQITDEHAGGL

QATPSSADGWKGAVSRNFNWQVSHYTSEMVRDAKHPCDLVEENATLLRLDANVMGVGTAA

CGPGVREDLLVKTEEVSFGFVLQAQ

>SoG_04010.T1

MVRTALAFAAVAAVLSGHAAATRFSLENRHAEIAARFDARAAADDEGCEDDEPTPVVSPT

GSATATYVAPTNSVPATTTIGASGAYTTSTVYSTNVYTITKCPVTVTNCPVGQVTTEYVA

IYTTVCPVGEVQPQPTGGSSKPPAGGSSPQPPAGGNSQQPPAGGSSPQPPAGGSSQPSQP

AVVPPKVTPVTTAGAATLSVSFLAVLGGVAAMFL

>SoG_04018.T1

MRAARYYGKEDIRVEEINEPVVRAGQVKVAPSFVGICGTDLHEYLGGPNFCPMTPHPITK

ETTPITLGHEFSGIITDIGPDVTGFKVGQRCAVQPTIFCGSCVACNSQCENVCHNGGFVG

LSGYGGGLSEAVCVEATHVFPLPDNVPLDIGALVEPLAVAWHAISAASEITQDAVVVILG

GGPIGLATVLCLRAKGVKHIIVSEVASSRQNFAVQFGAAKVLDPFKEDLRQVVLESSSNR

GADVVLDCAGVPASIKSAFDVVKTKGTVINVAIWENEIPFNPNWVTFKESIFKSVLGYQK

KDFQAVIDNLQSGAIKPHQMITKRIKLEDIVDGGIKALINDKDNQVKILVNVDTNEAIQS

VELE

>SoG_04019.T1

MAPHADNQAEVNTQVAPTKASLVGASDAKWLRDQVAALYEANIVAKILRTAIEGLQNNNP

PVAYPEYVPQIGEDNGRYILREADFWTCGFFPGSIYSLVQRLVQFPQAVPAANKQQLLDA

LLALGKDWSEPIKPMAHRTDTHDMSFIVQPSMRVRWEVLHDREALDTITTAASSLFTRYD

ENVGAIRSWDELTQESVHITSMTEDFLVIIDSMLNLDLLYYAAAQTGNKAMHDAATRHAK

TLIKTHMRHEKPGPGNRNLYSTTHVVNFDPATGQVKERRTAQGYQATSTWARGQAWGILG

YAQTYLWTGDLDFLETAMALSEYLIMRMETSPACVELNVPGEDRRRGRYVPLWDFDAPIL

DETNPLRDSSAGVIAANGMLVMSQALSGLGRGEESQRYLGMALAIVKDTLDTSLSREKAT

IELDPNGVISVKDETTGRRFDAILKNATANYNARDYKRYWDHGLVYADYYLIEFGNRLLK

MGLL

>SoG_04031.T1

MTVFICTLFLPKTIEFTLPGTPPQPSESAARRSQKVLSPPKPERQALDRQPTSLFTAQPD

LTPPTTPEPESASPNPFANEDGFRIQIPSHVGSPADKDSPAWGGRANQPRSRANSPPPAA

ISEHARTLEKARELGRMGIRQPRSLLRSDSHDRVFASANWKVVNADQGNGGLRNAIEAAS

RDGHCSDYTWIGTLGMPTDALDGTQQKQDIEDTLATEHDMLTVFCSDKDFSGHYTHFCKQ

ILWPVFHYQIPDNPKSKAYEDHSWKYYVNVNRAFADKIVKNWKRGDVVWIHDYHLLLVPG

MVREKLPDAKIGFFLHVAFPSSEVFRCLAVREQLLRGMLGANLVGFQIQEYTRHFLQTCS

RILNVEATPEGLQLEDRFVDVINSAIGIDPVILAQHRAGQDVVRWLNIMQERYKGKKLIV

ARDKLDHVRGVRQKLLAYELFLNKNPQWRGNTVLIQVALSGSENSDLDTAVSDIVTRVNS

SWANLAYQPVVYLKQDIDYAQYLALLTIADALMITSQREGMNLTSHEYLYCQDGKIHGQS

KHGSLILSEFTGTSSLFNKNELSVNPWDYRQCAEAIREALEMGEQERTTRWNKLMDCIAD

HTGSHWVSHYLSHLDKVYEEQQKRHQISVPRLSIPSVVSKYQKASRRLFILDYEGTLVSW

GPVNQIIPISPQRTLDVLNDLLLDERNTVYVMSGRRPEELDRVFRRVPNLGLIAENGCYL

KDCGSDCGTEGWAGMADAKHVAAWKKSVKAIIMYYLERTPGAELEERRCSLIFHFENAED

KEAAARLASDCASHVNDVCESQRVHAVVVDNSIIVEPVDWTKGTAAQRVFEGLQKKRPES

PLDFMMVIGDGREDEKVFKWANKLGKEELVRDVMTVSLGSRSTEAASTLTQGVSVHESI

>SoG_04033.T1

MISKEPEDIRAMERAAYDPHIMLLSDWSNFNSSQVLEATAKSKLQIFCVDSILINGKGSL

HCPGKQWLTDMQIPFMQISWPNDTITDKGCFPFVPSMEGPWLPEGDTSQIPYGMQEGCRE

STGQKETIDVDADDRWVSINWIGASTFKTLQPSIDEHEMWIYEVDGQ

>SoG_04034.T1

MLVWAGERYCAMVRLDKRKMDYEIRVIDGGYSQMIGGFATLRYRGGEQDLAEPDRFGVTS

ISRPWYGYNAWPVGTPSMLNKNDLPPFPPNPPARMIDETYILMLGKANSTWEFTLGGKKK

YPEDHSAYKPLLEHPDSAEANDEDLMIRTRNGTWQDIVLQVGHDKLWPVKFPHAIHKHAN

KYWRIGSGMGRFNYTTVNEAIEAQPDLFDLENPPCRDTFLNDFTGTMWVALRYRVTHPGA

WLLHCHFETHLSNGMAMAILDGVDVWPSVPEEYANNGKGFRVEGGKSEEALSNEGSQLYC

AKRPSVINDKVPITYLGTVTEDGKVEQFMSIRYGQDTGGEQRFRPPIPFVPEADSVVDAT

EPGAACPQPEQPMNQDPWTRIKNISEDCLTLRISRPAGIYGEANKQLPVMVWIHGGGHMV

GTI

>SoG_04037.T1

MATVVPPPSKRQKREQLERTQIQQDVTAASGPAGSFKARFLDGDGQQMADVIEVPLADAS

EKNLSLLLNTLMMREKEDWLPYRFRIHIPETDIIVDQYPTDLLALLRSHGIENPYETTIT

LSAEPQAVFKVQAVTRMAHRVPGHGEAILAVQFSPANSARLATGSGDKTARILDADTGTP

KYTLSGHSHWVLCVSWSPDGERLATGSMDKSVRLWDPNTGKAVGSPLTGHAKWVTNIAWE

PYHLWKDGTPRLASASKDCTVRTWVVNSGRTEHVLSGHRSSVSCVKWGGTGLIYSASHDK

TVRVWDAVKGTLVHTLSAHAHWVNHLALSTDFALRTSFYDHTPVPQGEDARRAKAKERFE

KAAKVQGKLAERLVSASDDFTMYLWDPSQGTKPVARMLGHQKQVNHVTFSPDGSLIASTG

WDNHTKLWSARDGKFINTLRGHVGPVYQCAFSADSRLLVTASKDTTLKVWSMASHKLAAD

LPGHQDEVFAVDWSPDGKRVGSGGKDKAVRLWCN

>SoG_04041.T1

MQLSNLLPVALMAYTAAAVAIPDGPNDHSHHDAGASPATSGSAAAAAAGFAPAVNCRNKI

CYSAVVPAATASSGSGPIWFQIYAPTTFSWVALGTGTEMADANMFIIYQDGKGNVTLSHR

QASGHTMPQVPSNSGVTTTLLPGTGVSNGFMVANFRCDNCTTWKSNQKVDFSSQGTPMIG

AWREGSSLDSADVKERITQHTGSPRIFSLNLANATKSTAGNPFVGDFAVPAEAAGAANGT

DGGKKGDSAGSGRFSMLSGSVAVTAIMTGFSFLL

>SoG_04044.T1

MLFDAGPAALVGLAFALWYIVSTVRQYLRLRHIKGPAVAGFTQLWLIRCVGGGRTHLQLW

EVCKKYGDIARVGPNDLITSDPDLMKRMLNVRTKYKRSAWYDAMRLDPTKDNVLSQRNDD

LHASTRSKMAAGYSGKEVDHLESTIDENVQRLIDLLDAKYISQGKAFEFGHKASYFTLDV

ISAVAFGEAFGDVETDSDVHGYIGAMEESMPTIIVSTVMPWVMKLLQIPLFKGMLPSEKD

AVGVGRTMAIAKRVTAERFGPHAKVQRDMLGSFIARGLKQEEVESEILMQMQVLSLLLSS

IRAIVLHIISNPRVVEAMRREIDQASPSLPIITDDEARAMPYLQAIIKEGLRVHPPVVGL

MSKEVPPGGDTFKGVFLPEGTQVGYCAWGIYRRRDIFGQDADEFRPERWLEASPDQLRLM

EGTLDLVFGHGRWACLGKNIALMELNKVFVELVRRFDLVVVNPVQPWTSINVGVFLQSNY

WIRGYKRAVA

>SoG_04045.T1

MPSVPSLASLVGAALVALPAVMAGYNQNANDNVAVYWGQNSYGQGSGSFVQQRLAYYCAN

ADVDIIPIAFMNGISPPMTNFANAGDSCAVFAGNSWLLSCPQIEEDIKTCQSQYGKTILI

SLGGATYTQGGWSSTAAAQTAAQQVFDMFGPVNPSSSVQRPFGSAVVNGFDFDFEATTNN

IAAFGQKLRSLIDSRSVSTPMYLTAAPQCVYPDAANGPALQGAVGFDFIMVQFYNNWCGT

ANFQEGSASQYAFNFDVWDNWAKTVSANRNVKILLGIPANTGAGGGYTSGSKLKAAIAWS

RQYSSFGGVMMWDMSQLYANTGFLAEVVGDLGGTASPSQGGGNTASSFRTSTTVAPPSAS

TTANASGGGSQGACSAATVTVTVTVTAGSGGGQPAPSSTSASQPPAASLVNQWGQCGGEG

YTGSTQCKPPYTCVSSGRWWADCSYGTVSTVNGQVKTVQQSIEAFVALPTAGSMYTRTLR

VRRRP

>SoG_04051.T1

MASRGRLARLLRVPARPVARASVCCASAQRQGSKAATTRWIATSPASLLSQSTARAAKLT

SETYPELKRDERFSQVTPEHVDYFKTLLGESAVLDGVNTDAEADLEVFNEDWMHKYRGSS

RLVLKPGSTEEVSKILKYCNENLLAVVPQGGNTGLVGGSVPVFDEIVINMSRMNQIHSFD

DVSGTLVVDAGVVLEVADQYLAERGYIFPLDLGAKGSCHIGGNVATNAGGLRLLRYGSLH

GTVLGVEAVLPDGTVMNDLCTLRKNNTGYDLKQLFIGGEGTIGIITKVSVQCPQRSSAVN

VAFFGLESFEKVQHAFREAKAQLSEILSAFELMDARSQKLVQDVKGDKHPLDEEYPFYCL

IETSGSNGEHDYAKLEAFLEDVMTKEVVQDGVLAQDATQAKALWGWREGIPECLGHWGGV

YKYDVSIPIKEMYDLVEDTRVRMDESGLAGDTPEHPAIAVVGYGHMGDSNLHLNIPVRRY

DPAVEKVLEPFVYEWIAERQGSISAEHGLGLAKKNYIGYSRDETMVNLMRQIKQLYDPNG

IMNPYKYLSEGKSSARYRDEEEDRGQLGRSDPATEGRNVQPYTHTPPVVVVTLARTVRVE

ADALRHRALPPSHAPCPMPHAPCLQGRILRGFDSSASDSHASPTPVYLYQEGHVGWADAT

CKPRNLHGSATKMPACRCESSTPGHAVKERVAPDSTTQHTHAHTHTGLL

>SoG_04054.T1

MRFIVALTSIAAVVSAARVGTPTRKDSPSRREPVASLCTKYAYYANNDYEVLNNLWGEEA

ATSGSQCTYYYGRAGDGVSISSNWTWQGAPNNVKSYIYANRLFKRPLVKDIKGLPTMATW

SYNTSDIRANVAYDIFTHKDADHPNYNGDFELMIWLNRYGGIWPITDSPTGKPVETVRIA

GYSWDLYTGYNGDMRVYSFLAADGPLHTFSADVLDFFHFLSKHYEYPASTQYMLSESTDL

FLPFPLLAFSTPCLSLFLFLSRLYIWRVDVAFYNFGTEAFTGGPANFDVPQFQADVHV

>SoG_04069.T1

MVRDALDAFARFDADLALSVAQYDKIIDREYKTALRELATYMMEDPRSISRVLSIIWVLR

SLERIGDQARNITGLVI

>SoG_04086.T1

MHNLVQAYERFKPSFEGIADVRTHINSTLLRATALQFRDLSSAREKAEQLAKLPEVKSFH

RFHSFSRPEPLNRTSVAEGSSSHLWQRDGGIAYNNRPHIMTQVDRLHTKGFSGDKIRIAI

VDSGIDYKHPALGGCFGKGCIVSYGMSFVDAKDSSRLAKPGNDPMDCLGHGTHVAGVIAA

QEKLNPYGLKGVAPRVELGSFKILGCRGTPDEATIIEALLSAHAAKSDIINVSFGGEAGW

STSLLSITLTRISREGTVCVAAAGNEGHYGMFYSLDPAASTDTIAVGAFQNVIRPAQKVD

FKATKNPKVPWVDNTDDPGLPSEFTSWGPNWDLNVKPELGAPGDNILVLDTIKNGGYGIV

HGSSFATPLVAGVVALIKEKRKGLKPAEIRNLLISTAKPASFYDNKKVVNMLAPVPQQGG

GMLQAYDAAMATVMLSVGSISFGHTGRPKLEQKFSVKNVGQKELSFEPRYRPAATAFTFA

AGETRPQLFPPDVSNVHSSVKLQPNKFKLKPGAQIDLTITALPDKSMDPKRVPVWSGFLE

LVASDKQVYSLPYMGVAADMSKLSVLSKNGNKILFGDADTAMYLNEQPMADEERLKKLGI

TAPPGNLKFTLPRQGDHSKFNMLPIFCVDLDMGSPLIWMDLVPVGKFDNKDFPFKKHLGL

RSIGILHERTKNKLSRGKYMFAWNGQLRGESGKYVPAGHYMVVTRVLKLFGHDANPADYE

TWESPFFYISYRQ

>SoG_04088.T1

MLKTLRDRGLKAVIFEAGSDLGGTWRWNCYPGAAVDSEVPEYEFSWPEVYTTWNWPSNYP

TFKDLREYFDHVDKVIGVKKDCSFHTVVVGGQFDTEEGRWHIKTADGRLTKAKYLVLGTG

FAAKRYIPEWPGMEKFKGIIHHSSFWPDEEIDVSDKRCGIVGTGASGVQITQAWGPKARD

LKVFQRTPNLAVPMRRRKLTAEDQERLKVLYPEMFRFREYCFGGFTYDWIERNTFDDTPE

EREAVYEKVWNDGGFRFWVAIYKDNLINPEANKESYKFWAKKTRARIGCPETRELLAPTE

MPHYFGIKRPCLEETYYEQFNRETVHLVDVKNNPIKEFTETGITLEDGTHHELDVIAVAT

GFDVSTGVMTQLGLKSIHGTELQKEWIPGAKTYLGTTVSGYPNMFHIYGPQAPTLLSNGP

TTVEIQSRWIADAITKMEAGGVKYINPKKEAADQWKQRILDLNNATLFPTTTSTYMGGNV

PGKAYEPVCYTGGVPTYKLEIRMALDNWTQGFDVVKA

>SoG_04092.T1

MVRLVDLTATAAAALTFAVAAQAATWLPKRAVVSVNDCLAAAGVEYHEEDSKGWAVDGTP

FNLRLKYKPIAIAVPRTTAQIQAAVKCGLDADVKISPKGGGHSYASLGFGGENGHLMIEL

DRMFSVKVGRNNVATIQGGARLGHVALELLDQGKRALSHGTCPGVGIGGHSLHGGYGFAT

HKHGLTLDWMVGATVVLADASVVHCSKTENSDLFWAIRGAGGGFGIVSEFEFDTFKAPEN

VTVFQVTTTWNRTQHVKGLKALQDWAEKDMPADLSMRLAINANSLNWEGNYLGTPAELRS

VLEPIMNKTGGAKISSAKQTDWLGQVKAWTYGAEVNASTPYDGVRPE

>SoG_04096.T1

MSEPLVELAPGFPCYVGGEEEARYIYKEIFEDHTYDVAKLPHNAFIIDVGANIGLFSAYM

KQEYPSSQILAFEPAPDTFATLRKNVELHRLQGVDTHQCALGSRDAVQMLTFYPNFPGNS

TLVPEEKAEIQRVSNEKGIGALVEKIFSGAVEIEVPVKRLSQVLQDQADLPSIDLLKIDV

EGAELDVLAGLDDAHWLLVQNIVVEICDLRGELRQLEDLLESKGFVLKKELASFAPEEVQ

IYTVTARRGGRNCDKALKQRGQRFGWWRLEATSIARPILWDYVVGKERMGDQFDDLSFTF

SKRASYQPYVPWGNCASHTNPEAQCSVSMVIEDDKRKQQWMVNMLAASHRYDEKKVDT

>SoG_04097.T1

MASEERPLLLCTAFPSAGHFNPIMAIASHMARTSCFDVVFVMPAEYKEPIERAGIEHVET

PSVMAEMIAGFQQTAHMPATVDRMSMQMGLIFFGTLAGRSKRFNEILEQKINSRKGSRKH

VVVLEDALGLASAPYRHWKAISPSQTPKFASQANGHHGNGDSKCRKEDETSLLAIGIGVS

PLLLESEDVGPLLMGLPPDDSPSGKLRNRALHQLVHQGPGMRLFQEKWESMLQETNCSAG

DDGAPEFKIWNTGYEAYDIVYQLSVPGSEYPMSDLPGNVQFSGTLPREDLKSYAYPPWWD

EVTGAERLGGSRRRVVFVTQGTINSAYEELILPTMRAFESRPDVIVVATLGAPGASLPSD

IDVPPNARVVDYIPYDVILPHTDVFVTNGGYGTFTHAIRNGVPMVAAGETEEKKEVALRI

AYSGAGVNLSTQKPSSDQVRNGVDKVLGDSSYKQRMEELMDESERLGCLDIIERKIRSFV

S

>SoG_04104.T1

MKVIFTSLLLLLLLLVGSSNAYKTGLMGYGQSWYDPACAYACRAVFGSAPLDCPGHDNMD

MDMGMNMHGGSPMAACISDNFDFLSTLAYCMNQRCSPEGVSASKLESYWADQATGDKTIL

PKWTYGAVLANVTQPPTRVFKKGDKLNYTALVSDADWTYQYEFDIFFDWEEAVQSTYVIV

LISVGVASPVVLSLIGYFPFMTTAIDKIKPYLIYPFTVAGYNIRPLPFLLGNAPTMGQSL

WIAMFVILNIILGSVSYENFSGVHPWGFTKTAEILAYVGYRTGHISFALLPLTVLFSSRN

NVLLWITDWPFSTFLVLHRWVARLCAVHAIVHSITLLAAYVSLGTYYTDVHKPYWIWGIV

ATLCLVILLFQSMIWFRRAAYETFLSLHIILAVFVVVGCWYHVYYWKPMSGVYELWLYMV

SAVWFFDRLVRVLRVAKNGIGRATVVELSPDIVRLDIQGVRWSPEPGHHAYVYFPTLHRL

RPWENHPFSVTHTAMLRSQKHDVASAHASSHSRDLDVEMSKGPVVVPSQSETMTGTDTIS

MYVKKHQGATKYLVETVDLPVLLEGPYRGNLSSEVLKCDRVLLIGGGIGITGLLTWAFSH

VNVKLAWSLKQSSAPLLQDLQNALDKLEDKVVVTGQRLDVEALLASEAEAGWKKVGVVVC

GPPGLCDATREAIVKFGKSRKTVFELEVDAFGW

>SoG_04109.T1

MALKYDPEWFATAGPQLASQGEVLPVGDVETRRSRYERLFVSYKYDIPANLELKILEAPS

SEGHDVEIYHLAKKEAGDGNGGTVESTAAVLHIHGGGYTAVSARHVLPSLVGFVTESSVP

MFSVEYRLAPESPYPGPLEDCWAALQYLHANSSALNIDPARIAVMGESAGGGLAAGLTIL

ARDRCLSPPLAKQILIYPMLDDRTTADHTNGLAIFSSNDIVTGWSAYLGDLYLASKVPAT

ASPGRITDVTGMPALYMDVGQLDVFLHEDMAYAQQHIRAGIETELHVYPGVIHAFQRWAP

RSDVVKKALANRVRAIATL

>SoG_04112.T1

MLEGPIDGIAAHSPSSHMEATPSTSNDIASVQYKYNEERDKRVRQDGTAQFATLFSQEKF

KHFQADLWADTETKASLEHLPAPKEGDRSEILIVGAGYGGLLYAVRLLQAGFKLEDIRMV

DSAAGFGGTWYWNRYPGLMCDVESYIYMPLLEETGYIPTMKYTSSQELREHAERIAKHFG

LEKLTWFRQRASGFEWNEDAKEWMTTLVPQLGQGKEGSPITVRSRFTVLATGVTMVPQVP

LIPGIDKFKGPMFHTARWDYTITGGTHEKPEMTKLQDKRVAVIGTGASAIQVVPAVTPWA

RELIVFQRTPSAVDVRGNKPTDMETAKKLFSKPGWQEERSVNWHKFVLDYPDKPDVNMVD

DGWTRFASFSALGGGPKSEFSSPQDMQKLVERLHGVDYPRQEAIRKRIDGVVKDPEVAKK

LKPWYPGWCKRACFHDHYLQSFNEPNVKLVDTNGQGVSRISENSIFVGDKEYPVDILILS

TGYRSPVLYSPPGRVGIDVKGRHGISLDKKWSSGTTTLHGMMSHDFPNFFWPGLNQAGSS

PNFIFTIDGSAKHTAKVMAHAAESASSLKEGTQSGGVYRLNFTVEPSAEAEEAWSQVIVS

GAGAFAATAGCTPSYFNAEGEFMREAPIEVQLTRARGSIWPKGALDFFEHIAEWREKENY

KGLEISTV

>SoG_04115.T1

MTKTEPGDWDDEPIRPKDADQIGWEGVPLSETCAYYGHIKRAALPTNDALGFDPDYKSID

PKAIVNEFMKAFSKALEAQDVAGLLDLIGPDGYWKDVELLTWDIRVLRGHEEIGTMLRER

LGKTGIKNVRLDARVEPQVETLGEDLSFILFHMEFDFEHGTGVAVSRLSPLKAVNGSSQE

LSQAKAWQIYTIGTALDTVDGWDAEHGDKMRYERGKYHDPAGRGRSYAELRRDELNGTDG

LDPVAVIVGAGHTGLTMAARFKVLGIPHLVIEKGDRAGYSWANRYSSLSLHGPTFTNHLP

CLPFPSWFPIFLPAQQLADFLQNYARIMDLNVWTNSEIEGKSAVYDEEQGKWTLTVSRGD

GTKHVVHPRHLFITTGISGTLPSVPEVPGMGEFERNGGIITHSSRHKIRPEMHGKRAIVV

GAATSGHDISYELSEHGCEVTMIQRSATHIMSVEKSVRALFKAREDINRRGGKPLDVVDE

ANFLKHPYPVEYELLRRGQRMARGIDKDLLDSLRKVGYRLHDGYHGGGAYSMFPFDQGGF

YWDTGCCKLIADGKIKLVHSEIERFTETGVIYKDGTTQDADIVVFATGYMNSKSAIQALL

GDEMAKKCNERWEKGNAFFVGPEGESTINYCPLPQKGLYSMFHQFAFSRFHSKRLALRIK

AEELGIDVTPYGNRPRGPPSTAAGRLPRPEGVPR

>SoG_04117.T1

MVFTSRYSDIDYPEDLTVWQWAFEDARYSPIHRYPEDEVGGFVDAVTRERLSFTQVKEHA

RYLSTALVRRYGLQPSDTVSMFSPNTIWYPVAMFAVLRVGGRVNGASPAYSLEEMCHALQ

TAEAKFILTIPGSIEVALAAAEQSRIPKDRIILLNGQLDGFASVKQLVNWAKEQGEEAQV

PFFRIPAGQTNDICGYLNFSSGTTGLPKALQDVAGPGKKRFLASLPLFHISGLVRFLHWP

IASNDECIMLPHFTMENFLQAIVDFKITDLTLVPSIVIRLVNDPIVDRYDLSSVKVIACG

AAPVGPAVLTQLQQKMPWTGFRQSYGMTESCCCLTTHPPEFYGYEYADSAGMLLGSTVVK

VVDVDTGKELGPNGVGELLAKGPQIAMGYLSNPTETAETFGADGYLRTGDIGCIDSQGFI

RIVDRIKEMIKVKGQQVAPADLESLLLAHPDVDDCAVLGIPDDYSAERPKAFVVLKASVE

PSESTGLALMDFVKGKRVRYKWVKEVEFVREIPKSPSGKILRRLLRNRSRSSEIGIVVKD

VVSRSRL

>SoG_04118.T1

MTEVAPLPGRLASVEHTNAHSPCGQLVDEAFIRKAIDVSNLNALRMALLQVTGNPELAAM

RTERYAIRGGAMFAHVLSKEDTPRLKEIAFEYLSKRREDDPIPPPPSKAESKKLADMFGD

EPVDERQFNYDYEELAFEEFPRAAAWSEKKPTAEKINNFKIVIIGAGISGIAAAVQFKRL

GLNFEVFERQSGIGGTWLLNTYPEARVDTSSYLFQFKFVKNYNWSEFYATAAETRAYLEH

VADEYDVKDRFFFNREVVSAVWKEDTSLWEITMKHSDGTSEVVTCNAVVSGSGLFATPNL

PNIKGIKDFRGSVFHTARWDHSVQYQGKRVALLGTGSTGTQLAATVAAASKHFTVYQRTP

NWIMNLEGYRAPRTEHQRYIFDNMPYYWNWFCYAGHVAAQQLQYLQEVDQEWIAKGGRVN

KRNDLAREALTDYIMSKCKDIPGMVEKVLPKYPPLVRRLVVDNGFYDMLAKDNVELVADD

IECFTEKGIKSKDGEEREFDVVILGTGFKTAQYFWPCTYVGREGKTLEELWKKDGPRSYL

GLTMPGFPNFFSFYGPNHQPRSGGFYSWGEIWSRYTAQAIIHLIENDKRSIDVRRDVFDE

YNAKLDEKTATLIWEYEGRGYYVNEHGRQSVNAPWHTADYHKMVLEVNFDDYEVS

>SoG_04119.T1

MPRQYEPEFALVFQALQAHMPKREKLQLETIPAIREVREAGYKAAFDQIPESPDVKEGWH

YATSRDGASVAIHSFMKNEAAAEATSAIVHFHGGGLVLGSSKAFARSLAAMVSQTSVPIF

SVNYRLAPEHKGKTLVEDCYAGVQWLYDHAEGLGIDKSRIGVFGESAGGGLAAGVALLAR

DRKLEPPLAKQILVYPMLDDRTVTPNPTVEPLAFWKTEDNVTGWTAVLGTNHEEVQPEDL

SYVVPARARDLSGLPPAYIDVGTLDIFLNEDIEYAKRLLDSDVEVELHVYPGVPHAFELV

QNISATKRATDNRVRAMQSI

>SoG_04120.T1

MAFEGKVIAITGGGQGIGLATAKLLASRGASVSVADSNPTTLAEVEKYFAENSWHIHTSA

IDIRQSEKVNAWIEETVKKFGHLDGALNGAGTIGKFHGRSPITEIDDDDWNLVMGVNVTG

MMNCLRAELRHLKDGGSIVNISSNQGSKGAPGCAPYSTSKHAVIGLTRCAAHDYGSRGIR

VNVVSPGGTYGPLMSSVVGDNPPPTTAVLGKYGQPEEVAAMICWLLGPESTHCSGEIFRV

DGGESV

>SoG_04123.T1

MGPNQHGKRPLSRQGCHQDPPVKRRASQACLSCRNRKVRCDVVNGGMPCTNCRLDNVRCE

VKETQRGRRPCSGRPSKKDTVTRPAKDLERNEIPQQDAQDVIQAQMTLEDEDEQEEGDAE

GDSEADEGSLIYCQPSRTPTTQQHSHSENPELSNCDEEQLPEEANGHAARLQSAPISPFL

VMSTPPRPGSQQHQIPQQPQRNSRGHQSRTPLPHGYHLLDRSTQLFPEAIRKPGLPLYIQ

PIPKHLDHQDVEYMESKHCLEIPDDELRDELLRVYVSIVYPLLPAVEIDEFIEAVMANDG

RNPISLLLFQSIMFVSVAFVDTEYLLARGYSSHKAARKDFFNRVRILYSLNYEKDRVALT

QSLLAMTYWYDAPDDDKDTWYWMGVALTTAQVAGFHRDPSSAVVAPRLREARLQRRIWWC

CFMRDLFLALGLRRPPRIRDTDYTVTMLCEADFNFGTHSATFGSLFRSSKFPCPDQTMSR

ELAAMSVELARLCVTAGHMLQTQYTSAGAYYGGSEYLGSHKPKRTSEQIATLAQCAAELS

DWIQHQNARAPYVPGDEQTSSDKGDERGKIIRFHRIQLHMHYLAALGALRRPSVFCQGPT

QRSHSEQTSLSAEVVLDPAIEMVKLAFDLQHNDQLRYLTTLAVPAYLAVSLVHLLDSRQG

EEETRNLSLGRLYQSVYVLQKLQSIYSSADYAICFLRSILANTKLRIPLLWMDDAATSRR

QDAPATKISMEMAPDEVAAACMYPSPSASAQLNKPVGDSPTGSSPPPVGVDQWELPWEQD

LIGGIAPFHELVAGSPFLGNWCDSDGMMPTAVDMGGTAPGFSGDISSRHC

>SoG_04125.T1

MSTSVLARFFREATVTSYFTVVASATAVYLLGSAFYNLYLSPLRKYPGPKLWAISQIPFS

IAWTTGQCHKKILELHLKYGEVVRLSPNQISIGYPEAWDDVMGHRKRGQEENGKDPDFWR

GDDKLTLVGSSRERHSRLRKILSHGFSAQAMMDQQPIFQQYATLLVKKLHEACTSGKSVD

ITSYYNWTLFDMAGDLIFGEPFGCLEETRYHPWVKLIFMHIKGIAISTAVIRFPFSDALI

KLMTPKSVAKDIQAHHDFTTSQVAKRMAYDNPRPDFMESMIRAYEENRVTKGELHANAHN

LIVGGSETTATTLAGVTYLLATNRDKLQKMYEELQATFKSENEINLISVNKLEYMFAVLH

EGLRVYPAVPTTIPRKTPPEGIQIGEDFVPGNTVLGVWQWPMFHNPKYWTDPEAFVPERW

TGDAKYENDKKNACQPFAVGPRNCIGKNLAYAEMRLVMAKLVWNFDFELDPRSKDWIDQN

KVYLLWEKPQLWLKLIPRKREDLALCLSPWFLT

>SoG_04132.T1

MGSPTTRRDTNPRKRPASWETHAQTAVAEVSGNSNFNRPAKKRAPRACISCRDRKVRCDV

ISGGHPCTNCRLDNVSCILKESNRGKHNSANHYARARTASLASVARPSPPSTSDIDADTT

VSVPTARSAPAAINVPPTGSGTGTSTQLPHCGGNRNQAQGDEEEAICDDKEGEVEEEQHV

GGQAATEQMNAPSYPAEEFPKRQPLRGKLSQTRREQHQRHTRRGSTPCSASSAPPANPST

SDYLVALAFEGQDKAATAASRRHSFTAEYSTGTTQNQPMADQDRQDTQHSMPWFIRPHPP

HLDADDMQYLERKDCFAIPDEKLRSELIRIYVFVVYPFMPAVDLGKFLGAITGEEQEEKI

SLLLFQAIMFACVTFIDVAQLKRRGFESKRAARRVFFNRVKLLYSLDYESDRLTLVQSLL

LMTYWYDSDSDDKHTWYWMGIALTTAQVEGLHRDVDEPQRMTRVGRLRRRIWWSCLIRDR

LLGLGIRRPSRIREDECSIRPLTVEDFDLTTPTAAVSRLLATTEGQGPDSSSRRAMAMIC

VDLSKLCVVIGHILHSQYTIGSTPQQEGSNYLRKAIVRPKSSEEQKQSFLRCDTALDEWL

QNCAPESKYEPGVDRNAAQGDKSMLRLHQALLYMNYLTALGALHRPQVFYSGSDRADLGR

KADSRRKVTEAAVAITKLAFDLQRTNQMCYSPTSSIPAFLSAALIHLLNTRSSNEETRNI

SIGRFCQCLDALHQLQTMYAAADQAVHIVNNVLENAGLMVPILGVGKPAPRLERATVGGR

PGGGPSGGPRNTRLFPDMWPSRVASAYPSPAAAGSHDENSHDGNSVPATAPLGVVDGPIT

EDLNHNMVRVAPAVAPAVPSYDPFTMPLFMNTWTVGGIGTVDRNNPLPTDGMPGSQFDLD

AWYDVGDILDPALMNFEVEADFVPAEAMAM

>SoG_04137.T1

MMLSRALMALGSLATASLAQQLTNPVIWEDLADNEVIRVGNDFYLTASTMHYSPGAPVLH

SRDLVNWEYIGHSVPTLDWGSKYDMKGSRAYVNGIWASSLAYRESDGLFYWLGCIDFSTT

YIYTAPAPEGPWERRSTIGNCYYDAGILFDDDDTMYVAYGNGQISVAQISRDLKQVSTRR

VYSVPSDFSGTLEGARFYKRQGQYYIFLTRPANGQYIIKAGSPMAQYSAAHKILLDMPGP

IPRGGIPHQGSMVTVPNGDWYYMGFVDAYPGGRVPVLAPITWDSEGWPSVTTVNGGGWGV

NYPYPLPPSPVKSPLGKDEFPGTSLGPAWEWNHNPDTSAFSVNNGLTLRAATVTNDLYSA

RNTLTKRIHGPVGTGTLELDVSGMASGDRAGLALLRDQSAYVGVFRSGDGFTVNYVDGLT

MGDKWVTTGTGTVRESRAAAPPSGRIWLRVVADVAPGGSRQATFHYSTDGSSFSRIGGSG

AFVMNTNWTFFMGYRYGIFEHATEKLGGSVKVLSFTSEAS

>SoG_04140.T1

MQYLRLRHIKGPPVAGISILWYLRVLLRGKMHLETLAINEKYGETESSWGESKGEPRIST

VFAGLVSAGRSYTPKGPLARIGPNDVLVSDPDMVKQIQAVGSKYVRSYWYDATRFDPFRD

HVLSMRNEAEHNARRPQLAPGYSGRGITGLDQFMDEIISQLMALLDDYTARGQRLDLVRK

LQYFTIDLITRAAFGAPFGFMAKDEDINGYIQTTESFAAVLGLATVFPFINNIRRSRLFA

ALLPMKKMAAGHVEIMNKARDTAADRFIPGRMVQQDMAGSFIAHGLNQEQLEGELALTLL

AGSETTAHVLRNTLLHVISNPVVLAKLRAEIDSTAAALPPSESGSSVPFASAQKLPYLQA

VIREGIRILPPVSGGFAKESPPGGARIEDTFLPGGTRLHINFWGLTRQKRIWGDDVEEFR

PERWLLDETDPASKERLKEMTVVGDMSFSYGKWQCLGKGLAQKVLAKSIFEPMKSSNHFT

FNQSDFWVRAKRRR

>SoG_04158.T1

MNTESLPDYLTIPGKTIPSFHCRVGKNHNDRQPQPANSYGYPVPGVQEPSSTSVMALDYF

ARYGVMLRALFRLIPELTLDPAAFSDQTWANILAHGDFDYIVVGSGFTALAFIQKALELD

PNVKILCLERGGFWLPSHFQNLPIPFKMVLGGPSETFPWTLSRKTFETKELKFCHGSCPF

FGGRSTFWSAWSPRPTLDLMRDFPESMKKTAQQESFWKDAAGLLNVTHADQIGDGVFGTL

QTAIDRILKDSVSKIPNADYVESAPLAVGRRSPTSTLRFNKFSVPGPLLGILEQQRHLAM

EEKGAPLEIMLNCAVKSMIKGDDDDFVRVIETSKGTLSWTGNNTKIILCSGAIPNATMLL

NSFDSCRDTVGKRLTGHYVTHISGRCPVKNVKGWSRGKTLNMAAAYLAGKDPKTGLQYHV

SVTALNSPNPKDDAEDAARECPDYAAAATLDQLTGSEDYVVFALGEFSEKNAQNYVALNK

GTDPTCNVTLQYTLCKDDHSAWDVMDAAVYDTITAMAGGDEHKSSVEWWDETTHGWSRKR

PSVDTIRIPGVVHESSTCFMGPKEQGGSVDEHYRPHGIENVYVTGAALFPTAGSWNPTMT

MCGYAQDLASKLHGMKAE

>SoG_04179.T1

MARPMGSVRLKKGNPITLILGALLCIFILAFLIFPSETKSRARISVNTAVHHLSPPTSPY

RKPKSRAGVELGPPPVSHYNLNNITITSNPIANQENILILTPMARFYDQYWDNLLKLTYP

HELITLGFILPKTKEGNAATSALQKRIQRTQHGPEKNRFKSIIILRQDFEVPLQSQEESE

RHKLSNQKARRAAMAKARNSLLFTTLGPSTSWVLWMDSDIIETPHSLIQDLTAHDKPVIV

PNCFQRFYDEEKKAWSERAYDFNSWQESETALQMASKMGPDDILVEGYAEVATYRALMAY

METEKPDDKNELVPLDGVGGTALMVRADVHRDGAMFPPFAFYHLIETEGFAKMAKRLGWQ

PWGLPNYRVYHYNE

>SoG_04183.T1

MATNSGRGGTIPGTEGIEQRTNPPPAVPAEAKQDIASSSQTGNHAAGQDSGAAPAAEGPA

KPKTEKELEKERKKAEKAARVEAKKLKTAAQAAPKVAKEKKPKKPEEAPVPEYVEDTPVG

EKKRIRSFEDPNFKAYDPIAVESAWYSWWEKEGFFKPEFTAEGKVKDAGSFVIVHPPPNV

TGALHMGHALGDSLQDVMIRWNRMQGKTTLWLPGCDHAGISTQSVVENMLWRKEQKTRHD

LGREKFIDTVWEWKGEYHKRINQALTRLGGSFDWSREAFTMDENLSAAVSETFVQLHEEG

IIYRANRLVNWCTKLNTALSNLEVSNKELTGRTLLDVPGYDKKVEFGVIVHFKYPIEGSD

ETVEVATTRIETMLGDTGIAVHPKDERYKHLIGKNAVHPFIEGRKLPIIADEYVDMEFGT

GAVKLTPAHDPNDFTLGQKHKLEFINILTDDGLLNENAGSFKGQKRFDVRYTIQDALKEK

GLYVDKKDNPMKVPLCEKSKDVIEPLMKPQWWVRTKELAEPAMEAVRDGRIKIRPETAER

NYFRWLEDINDWCISRQLWWGHRCPVYYARIEGGPGDIPEDKLWFSGRSREEAEKKATAA

LPGKTFTLEQDEDVLDTWFSSGLWPFSTLGWPKKTHDLETLYPTSVLETGWDILFFWIAR

MIMLGLKMTGKIPFSEVFCHSLVRDSEGRKMSKSLGNVIDPLDVISGIELDKLHAKLQLG

NLHPSEVQKATKYQKTAFPDGIPQCGADALRFTMINATTGGGDINLDIKVIHGFRKFCNK

IFQATKYVLGSLPSDFVPAKDASAVGVTLAERWILHKMNSAARDINIALADREFAKSSNI

VYRYWYNELCDVYIENSKAIIRDGTEEERKSAIQTLYTTLETALTMIHPFMPFLTEEMWQ

RMPRRPEDKTKSIMIAKYPEYNPALEDPKSEAAYELVLGCVKAARSLMAEYSLKDEADVI

IQAFTPTAEETCTEQSTSIKSLSGKAVRNVQILGPDAPRPAGCVAYPVSTEASVFLYVKG

RVDMDAEIAKAQKKLDKATTNIQKQEKNLNDPGYQARASPEVREGDQKKLADLKQEARSF

EETIKQFEQLKLE

>SoG_04184.T1

MPIDPEELERRAKAARAAVAKPRFIPRKERERLAAEKAAKEEAEKKRKLEDQERAHRESK

KQWLEEAEKDERRKRDLERRGANDHKSRNGGRDRDTQREEPGDWKDVREARNGNASTSNG

RKRTAQDIENENLLTRYLGPEVNKHSKFSAAKKRQRTAANKFNFDWDPSEDTTRDDDYGT

AVKPKIPKPSGSAVSVGNRFDEAAEQRALIKASAIRERDRETGEERAKGIMDDFYRSREK

EEQRLQKSSMGRKWATKSLADMTERDWRIFKEDFAISTKGGSLPNPMRSWQESGLSRTLL

EIVDKVGYSEPSAIQRAAIPIALGARDVIGVAHTGSGKTAAFILPMLDYISTLPLLTEVN

RDDGPYALILAPTRELAQQIESEALRFAQPLGFNCVSIVGGHSLEEQSHAMRNGAEIVVA

TPGRLVDCLERRLLVLSQCCYLVMDEADRMIDLGFEESVNKILDALPVSNEKPDTDDAEN

AQLMKSFSAGMNRYRQTMMYTATMPPSLEKIAKKYLRRPAIVTIGNVGEAVDTVEQRVEF

AGGEDRRKKRLQEILSSGEFSPPMIVFVNIKRNCDAIAHEVRRMGWSTVALHGSKTQEQR

EAALQSVRDGHTQVLVATDLAGRGIDVADISLVVNFNMATSIENYTHRIGRTGRAGKSGV

AITFLDGTEDPGILYDLKQMLSKSSLSKVPEELKKRAAAQPKFPKAGR

>SoG_04189.T1

MISSDSTRVVDSPKIWACLITNTNYLPGLLTLHYTLSHTLSSAYPLVALTSPALPSSALA

ALDARSIPHQPVPYLQPSSPTTSEAGEGGGEEPKLAYSDSRFRDTWTKLAVFGLTGYERI

VLLDADMLPLKPMDELFDDSFLPLDDPPPSPSDPLGHRVLAAAHACTCNPFKKPHYPPTW

IPSNCGLTPLHDDTALAQTSGGSPGALGVLNSGLLVLRPSGVYYALIHDHMQAHGHSYIF

PDQDVIANTFPRRWVPLPYVYNALKPLRFKGVHDAIWRDESVKNVHYILAPKPWDRDEAD

EKTEQNEMDWWWIDANKKRKLSENIRGIRDGC

>SoG_04191.T1

MKASPLIINWHDQNAPVYSAHFEPGGKGRLATAGGDNNVRLWKVECDGLERKVDYLSTLS

KHNQAVNVVRWAPKGELLASAGDDGNVILWVPSDAPPAAFGSDGLEDKESWRAKHMCRSS

GAEIYDLAWSPDGSHFIIGSMDNIARIYSAHTGTLVRQIAEHSHYVQGVTWDPLNEYIAT

QSSDRSVHIYSLKTKDGQYTLTQDEKPSKLASHAKTDLPPRRISSSSPAPPDFGHRAQLS

VLEPSASVGSPAPSAPGTPTSVALPMNPPSVVSHSRRSSFSSSRRSVSPAPSMPLPAVMP

MEASPKPHSVTSSIGLGMKNASLYANETLTSFFRRLTFTPDGSLLLTPSGQYQNQHQSDK

DAKPTYEVINTVYIYTRGGINKPPVTHLPGHKKPSVVVKCSPIIYTLRASPPSTEHITID

TSSAEEPLSSLPDPLSKPSPAPTEMEPPPPPPPPPPTTSTPADTTSSTGKTTSAEPAPTS

TPAPKSAFSLNYRMMYAVATQDSVLLYDTQQKTPISIVSNLHCATFTDLAWSSDGLTLMI

SSSDGFCSSLSFANGELGEIYKGEIGPPKLTATGPVSSQNTPTPTPTTTFAPPSPFPNGS

HHQHRNSASSFTAPSPPAITQRPSSPTRSNSTSSIATQATQSSTVPNTSVVSNPTLISGS

IPSIAAASSGKVTGVPLTTPPETPRSTAGSVAGTKRDASESEKEDALDSMPSDILTQWPP

APAISRLHAQVPITALAIHDGHLLAAQDTSLCVYDIDSGALIQQRSPAAFASLNAGQPIH

GIHVNPISGAILLWGASAVAYLPGGWEDETEVSGKANDWVYAGALSTCRDGRGNGFGVVV

TAHNDLVPVTLDDASGTLVFGKPVSPSRPILYSATLSFLSATELLIVGGTVFGEIIVWRC

HFDSSTRTGTGAEVLYTLTGHEGSIFGVAISPPLGPGRRRLLASCSDDRTIRLWDITEGK

ACESAAHERDSRAARETGFITAPPTESKPLATAMGHSSRIWGVRIVAAEEEDSPTILGVY

SFGEDATMQRWGVDIDAGRLTHEKTYSLNDGKHLWAHALATRADGILLFATGGADSRISL

IREPSLAASPGPPDLTSIDVRDIVRNAPALPSGSKRAKEIITRYDFLSADELVAMTSSGR

ILRGAVGAPASEWRIVPIEDGKHAEDLGLCYALRSVAPGLVALGTTSGNIYLYGKDSGLS

LVASLDGRIMDLIPVEGSSLPTGAVDVYVQLQGNKPPYYLTIDAFAGTLTSRSPLTDLDD

RFVAVSALRARDYIFIGSRHGYLSVLRAQAHESGTSYVAIYTLPPRSPDAISCILEIPSV

NALSAPLYVLTTSRDGHYRIYRLQAPQAPSTSIALDLVHEVPAPFGPVLTGAYFTTTTTA

SSPDLILHGFKGKDFLIYNATTRTDIASIPCGGAHRMHSLYADPSSPERLRFAFTRTSLV

SVYTQDAVSHRTLAPGTHGREIRALSSNGTHLATGSEDTTIRIWSTGAEEPSSSRPEHLM

TMKAHNTGIQQLRWLGSSHLLSSGGFEEFFVWRVRHLEGSLYTGVAVVREARLEDTTGDT

DGDLRIMDFDACWASGNDDSDDSSSSRGDQDRSMIITMVLSDSTLKTYTYSRGGGFVPRA

RGRYTGACVTQVRRLTDSRGGSKALVTGSTDGHICIWTEDLGTETCQLSASMRVHQNAIK

AMDMVPHAAGFLVLTGGDDNAVHLSRVASAPAGSPPGERIHALSKLASFPRAHTASINGV

AIVRRSCGGGLLGVSCSNDQRVKLWDLGHSGQEVALIDDRYSGVADPGDLEVLDEGQGTL

AIGGVGMELWHVTGRIPA

>SoG_04195.T1

MDLSRRAHKSGGAREQAAELPLHNEEKGRRRRMPNRLGFLSRPLAIWGDSGISMPLGLAI

LFPIMIIILIVFMFTRQPNSTGRFLMPGGAPPAIRKISEKHDKVFVEGCLEPDTSKPRAN

AAFVILARNSDLDGVVQSLKSVERHFNRWYHYPYVFLNDAEFNETFKTTVQKYTSGKVEF

GKVGPDMWGYPDWMDVKAAKEGIAKQGDSAVMYGGLESYHTMCRFYSGFFYKHPLLLKYE

WYWRVEPDIKYFCDITYDPFLKMIEHNKTYGFTIAIQEFRETVPNIFRYASAYKRINKIK

STDLWEMFVEPVELKSDKAKDMDPKPPKEMREQSKHFSPDGDPEAMEGESYNTCHFWSNF

EIAKLSWFRSKEYEDFFQMMDRSGGFWMERWGDAPIHSLAAGALLQVKDVHYFRDIGYRH

TTIQHCPANAPGKQLPRQPFLEDTTLDKKARQREDAFWDDWDEERQNGVGCRCRCDKDVV

DVEGKEGSCIAEWVDVAGGWAN

>SoG_04206.T1

MKTSAILSLLGLGATLASAHMEMTEPAPFRSKSNKFATQKDYDMTSPLAPDGSNFPCKGY

LSDFGTPAGASVATYSPGQSYKFTINGGASHNGGSCQASLSYDKGKTWTVIHSYIGNCPL

QGSSSFDFTIPSDTPAGEAIFSWSWFNKVGNREMYQNCAAVTIGGGKRKRAPSDSFKSRP

AMFVANVGNGCGTLEGKDVMFPNPGPDVTNNSQGTAPPTGNCAGGSGGGSGGGSNGGSNG

GSGGGAGSAPAPPAKTTKAPVQKPSATAPGGVFITSAPAGGGQGAPSAPTTLITLTNPVA

PPPAATQTPGTPKAGKPPAVNPPSTPAPTNPPSGGGSGGMPAGQACTNEGDWNCVGGTRF

QRCASGRWSVLMPMAAGTTCKEGMGSSFAFGAKGGKKRAVRYARSLRF

>SoG_04211.T1

MVKELKSLGVELMVSIWPTVDYRSENFQEMRERGLLVRTERGLRTNFDFEGQTVYYDATK

PEARRYLWGKARENYYSHGIKLFWLDEAEPEYSVYDFDNYRYHQGPVLKVGNVYPREYAR

TFYEGLRQEEKTDSIVNLLRCAWVGSQKYGALVWSGDIASSWPSFRNQLAAGLNMGIAGL

PWWTTDIGGFHGGDPSSEEFRELFVRWFQWGTFCPVMRLHGDRDPKRGPEDSSSGADNEI

WSYGPDVYEICKKYLAIRETLRPYTRRLMEEASSRGSPVMRTLFYEFPADPQSWEVHEQF

MYGDTYLCCPVLGPGVERMSVYLPPLVSGERWFDFYDDKVSHEGGQRIEVACPREQMPVF

ARRKER

>SoG_04218.T1

MSKTFSASDVAAHNKPDSLYISIDGDVFDVTKFADEHPGGKKILQRVAGKDASKQFWKYH

SEGVLKKYKPKLQVGSLDTKPKPKEEPKPTPAAAKPAPAPAAQKPATKTSHDDDSEALEA

FGAQIPFSDPSWYQNYHSPYYNESHAALRAEVREWVETEIEPHVTEWDEAKKVPGSIYKA

MGERGYLAGLLGLHYPTEYGNNTNKAVSPEQWDLFHELIVTDELSRTGSGGFVWNLIGGF

GIGAPPVAKFGTKALKDRILPGILAGDKRICLAITEPDAGSDVANLTCEAKLSEDGKHFI

VNGEKKWITNGVWADYFTTAVRTGGEGMNGVSLLLIERGEGVTTRRMDCQGVWSSGTTYV

TFEDVKVPVENLLGKKNQGFRVIMTNFNHERMGIIIQSLRFSRVCFEESVKYANKRRTFG

KKLIEHPVIRMKLAHMARQIEASYNWLENLIYQCQKMGETEAMLRLGGPIAGLKAQATVT

FEFCAREASQIFGGLSYSRGGQGGKVERLYRDVRAYAIPGGSEEIMLDLSMRQSMRVAKA

MEPHLNSSPHTKVHQRTFWTSPTDLGSCSIPIKEQPVQSPFHCSHLLRPLLASRHLFRPD

FCVLVSLRFEAPAPHGPGRPDSKSLLITTVQSQPGSSYTHAFFPPPPEILLLGKPPVWSS

HAKGLLQNSGLLTCPASDGGATGGDDWKKNLKLPAKDNRQQTEDVTNTKGLEFEDFALKR

DLLMGIFEAGFERPSPIQEEAIPVALTGRDILARAKNGTGKTAAFVIPTLERINPKINKI

QCLILVPTRELAMQTSQVCKTLGKHLGINVMVTTGGTGLRDDIVRLQDPVHIVVGTPGRI

LDLAGKNVADLSECPMFVMDEADKLLSIEFTPVIEQLLQFHPKDRQVMLFSATFPLSVKD

FSDRNMASPYEINLMDELTLRGITQYYAFVEEKQKVHCLNTLFSKLQINQSIIFCNSTNR

VELLAKKITELGYSCFYSHAKMQQHARNRVFHDFRNGVCRNLVCSDLLTRGIDIQAVNVV

INFDFPKNAETYLHRIGRSGRYGHLGLAINLINWEDRFNLYNIERDLGTEIQPIPASIDK

SLYVYENPESIPRPISNLPKSAQAPQAQGQQPQQPQSQGNWQTQQGHQNGSYGGRGRGRG

RGGYRGRGGGGGRGRGAPRDGQAPSNPQ

>SoG_04225.T1

MDLETRSVLNPSPQRMTGPSLLHELVRSSSDDTALEHLEEGTLSSYTYEDLHTASDNIAR

DIQAARKQDADKFIVPVLHPQSPCLYAALLGILKAGGAFCPLNLDVPPERLSFILSDVAA

TVVVISSDLLDKFDFPAHVQVVPIEPFETYTVPGPGRVPPNISPADLAYVMYTSGSTGVP

KGVAISHDAATQALLAHDCHVPELSRFFQFAAPTFDVSVFEIFFPLSRGNTLISATRSET

LEDLPSVLNKANVDACELTPTVAAGLLRSRQAVPGLKTLLTIGEMLNKPVVEEFGGKADG

SSILWAMYGPTEATIHCTLMPSFESAMPVGIIGVPLKTVSCFVIDPENPTTEGNDLNVLP

KGEVGELAVGGFQLAEGYLNRPEQTAKAFIETSSGRVYRTGDKARLNDDGLLECFGRLSD

GQVKLRGQRIELGEIEQAMLKTPNCHVATALVVNSMLVGFCVADASVSEDNILSGCRTWL

PPYMVPSEIIILDDLPKLPSGKVDRPALKLILSQREEAGNVDQSTSMAEHDHQDRVIRAV

AQCLETEVAPRMTLRSLGVDSLRAIKMASVLQSLDLNIGVADILRARTVSDLIPRIQMSK

SLSTDSEAASYSLLPHLDQIILGAEPLVKVRDKIEEIFPCTPLQTAMLTASYHDPRAYHN

EMEFEVDDDISADELARGLESICDSNEILRTCFVAWERQVVAAVFSTCMDDTVQIVSPED

EAQQSNATLTWLKPLQIRIFTGYQDRRSRILISAHHAIYDGWSIDAILTDLWRFTKGDDV

PSRPQFRQIVNHQATLVPETQERDKRFWADHLSTWKRKPFPVLIEKPATSSAPSVVTQQC

SLDGASINQATQRIGCSTSSLFLAALAQTWKGILGSEDFTIGTTTSGRALPISHIEDIIG

PCIATLPLRVQFQNLETSDQLIKSIDNAAKRLIEYSALSLSEIRKLAGLQTFEGLYDVLF

TFQESLFSKSVKEGPFRQVRHLDRLETKLLIEVEPMSSGYSLQATFHTDVIDQSMATQML

QQMANYLYNIASQEAASPSIVLSSESLPLSVHNERVPEPSHVLDLAAGFEVVAEKHPDLD

AIDFMMSGQDSTVRETLTYHNLNSQANQVARLLQAHGAEPGMVIGIIMEKSVSLYVGILA

ILKTGCAYLPLLPSTPPLRVKEILSQASVRLCLVDTDDGPALNETTTTQWLIPKRQYFEH

LSDANICIPPDGDRLAYVIFTSGTTGKPKGVSVTQRNITCNVLHLSTVYPVKETQQGRFL

QACSQAFDVSVFEIFFCWLSSMCLCSGTNDTLFADIEQSARTLRVSHLSLTPTVAAMIDP

ENVPTVEFLVTAGEPLTRTVLNKWGSLLWQGYGPSETTNICTVKRMDKKYNIEHLGHAFP

NTSTVVLQAHGQSILPLGWVGEFCFGGDQVAQGYMGDAALTAAKFINHATYGRLYRSGDL

GRMLPDGTLVILGRIDTQVKLRGQRIETAEIDHVLSMHQGLHSAATLLLNTPKSSSSQCL

ASFLSFSQSRKNDDIALLPFDPTTQLSLLATLKARLPAYMIPTYLIPISSIPQTSSGKCD

SQRLRSIFSELSTEQLNQYAHAHEGAEDDGEWLPLEKTIRNTIAGSLGIPEIEIGRWSSL

SGMGLDSISAIRVAQDLSTALKTRVPISALLLYPCIALLAKQLAEAKQEHTVGEPLLSDE

LTTSVISRMLDQTYAVAKVLPCTPLQEGMLSQGRGSYYNRVLLRLRSTPDRIRGCWEEVC

RRHEIFRTCFMTTSDRNTPVVQVVLDGWTIPWRSYSSNDLSIVGAVQTHMASLLEPVDSM

VPPLSLALIANRKSYFLSFLCHHALYDGTAIGALWREIEALARGEPLLPPIPYEPFLRAM

RELPVDYLSFWKEKLRSRQNRLVFAGQRHTTAAQQAIHTATFELRLDETQRALQSVQVGL

QSLCQAAWAIVLCSLTGSEDVVFGNVISGRNVDVAGIERLVAPCFNSIPVRKTLDKSVQN

VQLLQELQSLNTEMLPYHFSPLREIAKLAGSRRLFDTLLLVQQPLEEMDAAIWTLEEDVG

DMDVPLVCEVTPCPNLNSVAVKLYYDMNTLTEEEASTISSEFTQSVSRLIRSPYASVLLE

PLNGLQTRPQVADSYPHVDDGAGDPEAGTSSWDALESQIRTCKTTSMFRLGIDSISAIQL

VSMMRERGYSITTADVMENSTCERLAQKLLLRSAPAKRHTKSFDFDAFRESISEQVSRAV

PTEMPLEAILPCTPLQCAMANSSRSDNSQYMNLISFQLESGLRAADIAAAWETVRSQHSM

LRTALIPIDNRNTPFAMVRYPPRALELPLEVIDSRDEAPKFENSMRIEVATSLLMQPHLP

PWKVVLTSTKEDRIHMHLVIHHALYDAVSLQTILSDLASALGGNKIRGTANPEPGLSEMM

MKDATLNANAEAFWKSLGPQVVINRFPVLTPLRESEGQRKTAIYSSRMKFGTILKKCKDS

DVTVQALIEAAWTRILAEYTGEDSIVFGVTLSTRRTEATKNTPLPCITTLPVIARNQPSN

SDLLKSILGHDAKLASFRNSSLGDVQRWVGHPASPIFDTLVTYQGHLEQTSADFPWTILD

DVGLVEYPVALEIKPMPDDQVILSLGYSTDVVPPKHAEHILRQFDATMYGLLHCSTSRDD

ELFQSDPSLFSVTPAELEEIATEIKCLHEFVERQSIRTPHAPALEFHQTATSIARTWNYR

QLNDMGNRVAFTLRRLGLEGSLVAVHFHKCPEAYFSILGALKAGFAFVALDPSMPRARKE

FIMRDSGACCLLAADSSIPDFDVDVPTRIISESGLTALEGFDPSVASENISPFHTCYCLY

TSGTTGTPKGCEITHENAVQAMMAFQHLFRGRWNEDSRWLQFAALHFDVSILEQYWTWSV

GMTLVVTPRDVILDDLAGTLARLKITHLDLTPSLARLLDPQDVPDLWNGVFITGGESLRQ

EILDAWGPKGVIYNAYGPTEATIGVTTHSQVPQNGRPSNIGKPFLNVGTYVLHPGTDLPV

LRGAVGELCIAGKLVGKGYINREDTTRERFPWLPRFSERVYRTGDLVRLLSDGSFDFLGR

ADDQVKLRGQRLELGEIDHVIRSAMPEIEDLATIVVTQKSPDRSVLVCFLTNGKGKDHEL

RPMKSDRGLDRQAKAACRSQLPSYMIPTYFLEMPYIPLSINNKAEHKTLRSVFEALSPDE

LMDLTSSSALSLSMSAQKALDVILQAFSDFTKSDDISLTPTSSIFEAGIDSINVLLFVAA

LRRRGVQCSAAVVLQNPILADLADALSAQKPAGEANKNVREAQMIIKASFHRNLLAVRNV

LGVGPEKIEYIAPCTPLQEGMVTTALSRMSGNAYFVSLDLRLSPAADVERLRDAWLALIQ

RHAIMRTVFLQTPEGCIQVASREADLDWVNQATESERDARKLLWRQANEHNILKPLQFTV

VRSSDQTRLAVDIFHGIYDGISLDCMLQQVKDIYNNTVPKEAPAFIEVMAHGPLWQFDHC

KEFWIDHFRGWRASNLWTAQPDVHDGSQAIVATKNLPQGSIGALLKSLRVTHQTLFLALW

AAALSQVFKSLPTIGLIIGGRSLDLSGVEETVGPLFNTLPFYSGSVHSQNWSDLVTRCHE

FSSAILNFQHVPLRKIQKWCCSGQNVLETLLSVQPESNNGKDQELWTVEEPTAPLDYPLA

LEVTIKQDGNIHLHLVADRTFVTEAVVERLLDQLETLCASVVQDSETQWTEELLDAASFS

QPSVNAHAAENKMNDKIIWSATALTVRQTLAEIANCKAESIEAHTSILELGLDSIDVLRL

GSKLREEGIDVAPSTLMNFPTIATLMPQIEDGVILEPRSDNGVKMDELRHKLRSALLSAG

RDLSTVETILPTTPSQEGMIKAMMDSDFQQYFNHEVLQLDPAVDLDKLHKAWSSVVESNP

ILRTGFSEVSDETVELAFCQVVHGFEGLDISKHELGSLPESSGHFETAKSLARQIGGWSD

MFQVRFASCGEDKYMVVSVAHALYDGWSLGLVYQDLQRAYKGKLLNRPSPEGYLSQSINR

DNNESRHFWESFLYAAEPTILCPDEQMTISSSEICFRDSVSVVEAADVTQFCRDLGISLQ

VLCYACWSVILAHQARQLDVSFGSVLSGRDFEGAEELMFPTMNTVAQRCILSGTCASFLS

YLEQGMRSIRPHQSYPLRRALASATEGRALFDTLFMVQKSLGSGERAEHRLFKSVMGGSE

TEYPICAESEVLGDQLVWRVASKSSKHGASVVAGILRDLDAVLQYFIHSPDRNVVNLLGE

EVSICGLDPVSLRPVLADGPSPSKVALNYEGHQWSPPASAIAEVLSSVSGVPVGDIKIWN

SIYHLGLDSITAIKLSSLLRAKGIALRSRDLLQAADIAEMARIAEERSGTEAKQESETAV

YIQTWAPPASVSTQDLLSRHGLSETDVEAILPALPMQLYMIRSWYSNGGSLFFPEFRIRL

PRGTSSTQIEHAKTTLREETPLLRTYFCPTGKDECPLLQVVMKSNGMRGHDAWWGCWAET

DSDGEVLLTLKIHHALYDAFSLSCLLQRLTSLCSGTNPKPEPVPLSPWVPCTTRSSAETR

QAQRQRFWTDYLGDAISDHHNAALEPTGFSKTRTSHVDSDTNCSSPKLRQAASAAGTSVQ

ALFLAAYATSTATETHGKDFDGSVVFGMYFANRDEAETLPSSYPTLCITPIKVHVSPTRT

LLSSAQAIQRDLNTISSQGAADVGLWEIKRWTGLTIDRVVNFIADRGAAAAAELQWLPPR

ETEGGVSGVVTPETLGDWDSPLMSSAYPATIEIEASMDENTLTVGVFGPQTLLTEGQAAD

MVRRITTTLLEGAFFLRK

>SoG_04242.T1

MHESSTDEHSLGELAVCYSYYTHSPTERDPALQLLHSQPLPDVRHDPGYSSVSVQAHNVP

FWVVENTPPNPARSRHPVIGLQTVAKPVARCFCNQRAHLAINCFFARRHTRSIKGHKVAR

LLQLDLRRANHGARFANTRSPPGANLGFAAPVASGGGGTWAVARPCAGFFLSLHPFNAES

QSQSNMAESKCPASRLMNVGGGGTRNRDWWPDQLRLNILRQHNPASDPLGKDFDYAEAFK

SLDYAALKKDLTDLMTDSQDWWPADFGHYGGLFIRMAWHSAGTYRVHDGRGGGGQVCHTP

TALLLPRSSSAN

>SoG_04257.T1

MQLSLLPLLGLTLPVARGLVSPDGTGRLPAMGWNSWNEYACDIKDEVFITIADLMVKLGL

KDAGYQYVNIDDCWSDKVNKRDNTTRRILVDPVKFPKGISHVAEEVHARGLKLGIYGDAG

TATCGGYPGSLGYEEIDAATWAEWGVDYIKYDNCNVPEKWVDVPYMYPDDGGLIEGYVPP

GYDWSTSSSSKRYNQMRDALLIQNRTIQFSLCIWGHANVVQWGNNTGHSWRMFSDIWPDW

TGSHEYSQALMPILNQAAFYWNVSGFWGRNDWDMLEVGNGNLTHEENRSHFALWAALKSP

LIIGTKLDAISDDVLSILKNEELIAFNQDPVYGKGAMPYLWDGVSNGTHPANYWAGNSLK

GVHVFVLNTMEHEMDMEVVFADLPGLRRKKSQSFLVHDMWTGRDMGVFKDVFTVRVDRHD

TAALRITKVDGRHPNPEWMLKG

>SoG_04267.T1

MHIFHSPNQERLSLFWLVASVVCATATQRLSSLDAELPSEVLCWIILPLISWTSRRTGLK

SYSVGSSDLGDGTSKIVAPTKPSSSALAAIALGIGVAALAASEFKSQALLPTLPPLILLI

ERWLHDDSRSLRTRGLSAHLIESPLVAAAIALFSIITLSKGDLLAALTSVPALMGFLAVY

ATLTLRAMSGSTAHPGFDFEDAILALSVRAVVVAAGCLLFRCLFLGSGWPGLFSVAALGM

AKACLWYSLILTSKHSSWMVAPVVKLFSITSTIDFMSLTTERQAISCVLSSLLSLGQLAG

LMPQHRKSRRVMGLFLLLAVFPYVVNIWSLSDSYSHAPDWGTKELHPIDKLIEDAQQQFQ

QIRKRQSITHSIAQEEYRRRYGIDPPPGFEGWFEYAVSHQSPIIDEFDAVFESISPYLQL

SGKQVKEVMRAIHERPLNELWTCLFQGEDAKASCHHRKRSVNKDRDITLLFNRLLGDVGI

QLKNIKALVNHLDEPRVVYPPRSTPDGVFSMRNLKREPTWADVTAHCPSDLEDWNAKHPI

VQQYGLPFVANRTSVMDLCQHPEYEHMHGLLISPKNLRIVEGHVPILSTGSFSTMGDILI

PSPAYIQAPFQYDDHGDVPWEKKRNNLYWDGSNTGGYAKEDDPWLQFHRQRFVSFVQRLE

GRSYYYLQSFGGKVLKTASNLLDGRHYDVAFTRILQCQESVCREQRNFFRRRTYTPATAA

LHSRLVFDLDGNGISGRFYRLLASRSLPLKQTLFREWHDERLVPWVHYVPISQSMEELPE

VVRWLTTTERGQMRAREMAEAGRRWYYEAIRDVDKGIYLYRVMLELGRIQDEKREAGGS

>SoG_04269.T1

MHISKSRLILHIIPRATTHLNFLPDSACGAAHESTVALIPGEHTQYTSDTGIENHDKMNG

DAADMATSRAAFHIAKSSARGPATPRLGTLSLKGRTPISTPNFVAMTSRGVVPHLTPDMV

IKATDFGAAHMALEDFIERKDPPIYETAGQDRPRLHDFTCFPTDRVTILGPRRSPPVLAP

AGNISDSIQVYTAFGFHSLTLPSYASAVQRLGPDIAIGPADLPFLKKAPHSKKLIRMVER

TEEWMDDFLSKVGGSGTGADGTAVFAPVLPVELPIQWDYLRHLSEDVLESLAGLAVYDTN

MLPELTSYSSLERLPKLSMDTADSPRELLRQVALGMDLCTVPFINNVSDAGIALSFTFPA

PEASEVLPLGINMWDTEHRMSLQPLVEGCTCYACSKHHRAFMQHLLNAKEMLGWNLLQAH

NHHVVSQFFAGIRKTLSEGGEAFEEACTKFERVYEPELPVGTGQRPRARGHHFKSEAAQK

PFNAPAWSNLGGDDAVGGLADGQEVETPVVPSVSAAALAGTGFATAQS

>SoG_04282.T1

MIFPKVLLTIVTLGFHPVLAQQPVWGQXSCNASGGSPTTSGTAPGTTNPPSAGASRSQGC

GKAPISSGTRSVNVNGKNRQYIVRVPTGYNSNNAYKLIFGFHWRGGSMNDVAGGGTDGAA

WAYYGQQRVGQETSILVAPNGLNGGWGNSGGEDIAFVDAMIADIESNLCVNQSQRFAIGF

SWGGAMSYSIACSRAKVFRGVAVISGGVLSGCSGGSDPIAYLGIHGVSDNVLGISGGRSL

RDKFVTNNGCNRANPAEPAPGSGGHVKTEYSGCRSGYPVTWLAFDGGHYPGPVDGSGESG

ARSYVPGEIHSFFNKLS

>SoG_04284.T1

MPSHVSRIALFVLAGLSLANPANATTCSPRVRLQQGTYIGLENKEYSQEFFLGVPYAEPP

VGPLRFKAPQPLTERFNGHRNATQYGWMCIGYGSDTSNLGSPVSEDCLTLNVVRPSGTKP

GDNLPVGVWVHGGSYVQGGSRDPRYNLSDIVEQSVREGKPIMAVSINYRLSLWGFLFSQE

MQDENAGNIAFKDQRMALQWLQSNVAAFGGNPRKVTIWGESAGARSLGMQLIAYQGRSDD

LFHGAALESGSPIAKFHNAARWQPYFNALVAKTGCDSRSDRLNCLRGLSWQTLSDIFSGK

NALSVPVPTLSAVIDGDFMTAQGSVLLRAGQFAHVPLLTGNNFDEGTAYAKTGIDTDDQF

AAWLASLELSQDQISKISELYPNDPAVGIPEFYKQTPPASFGSQFKRVAAVAGDYQQHSG

RRLLAEAYAAAGLPVYSYLWNVIVNGLPNPIYGATHFQEVAFVFNNVHGRGYSANPFAGK

PETYVELADLMSKHWAAFMHDGMPTANGVAWPRYEPGSRTNIVFDANVDGLHYTAKDDYR

SEQISYLLDKVFV

>SoG_04285.T1

MSSLRDTSRLPASNSQGSLAQMWQSPDGNNAEKLVQSQKATRKKKFLWLGLAIALVLVAA

GVTVGVLASKGVISTSYSKSADSAADAKQSNPPVAEDDALRSISSQHTKTASTASTPSTP

SSTPSSTPTGLVCPTKDDIPKNARGTDLDTTTWLDMNGFNCTFTSETVGDLPLVGLNSTW

DNSAKANPNVPALNKPWGDYASRPARGVNVGGWLSLEPFITPSMFQYPDSAGVVDEWTLC

KHLGGKAPEVLEKHYSTFITEADFKAIADAGLDHIRIPFSYWAVQTYDDDPYVARISWRY

LLRGIEWARKYGLRIKLDLHGVPGSQNGWNHSGRSGKPNWIAGKDGASNAKRALEIHDRL

SKFFAQERYKNVIAFYGLVNEPARSLDTDKVKDWTEKAVDIVVKNKVNGYPVFSEGLLGL

KAWSGAFPDLQKDHLVLDAHQYTIFDPYLLSLKPKDRIEYGCKDFSKESSDSMKAYGPTM

VAEWSQAHTDCTEHLNGFDMGNRWTGTFPDVPGPTCPTEDKQCTCSVSNDETKYTDAYKL

FLKTWTMAQMDAFEKGWGWFYWTWKTESAPLWSYKHALDGGFMPKKAYARDWSCSQPIPD

LSNVKPYE

>SoG_04287.T1

MNGVHVTDNANGTNGTNVTNGNHVTKRPVRAWWKESSVYQVYPASFQDTTGSGFGDLKGV

ISRVDYLKNLGVDVVWLSPIFASPQVDMGYDCSDYKVIDPCYGDISDVDVLTNKLHERGM

KLVLDLVVNHTSDQHPWFKESRKSKDNPFRDWYIWKPPRYDANGNRQPPTNWMSHFQGST

WEYDEATDEYYLHLFCKEQPDLNWENPAVRKTVHEIMRFWLDRGIDGFRLDVINFISKDQ

SFPDSDLPILGGHEHYASGPRLHEYLRGIGAILKEYDAFSVGEMPCVHDEKELVRAVASD

RGELSMIFHFELMDIDHGEKGKFTPRRWELSELKSKVSRWQNFMIKNDGWNALYLENHDQ

PRSISRFAFDEPENRVASAKLIAIFLGFQSGTPFIYQGQEIGMTNVPRDWAMNEYLDVDC

LNHWNLFKDHADEQTKQRLRVEYQKKSRDNARTPMQWDASPQAGFTTSRPWMRVNDNYKK

INAAAQVNNPDSVYHCWRQVLEKRKAHKEIFVYGDYQLVDPDHDKVFAYKRVAENGDTAL

TVCNFSKEPVVWEFQVSAREVLISPFGKLVADVNGGSISLSPSEALTVLL

>SoG_04297.T1

MVNWAKSTYGADPSRIFVTGTSSGCMMTNVMMATYPDVFQAATCYSGVAAGCLAGSPGSS

PSSADPTCANGNNIKTGDQWAQIVRNMYPGYTGKYPRLLTYHGLADTFVHWRNLQEQLKE

WSTIMGVSFTRDVANTPKQGYTQHIYGDGTKLIGVEAQGVGHVVPAIEDVDLQWFGITPG

GPGGGGSTTTTTTSSSSRPAVTGTRTSSAPPPASSTAAPPTGNCASLYGQCGGNGFSGPK

CCSQGTCKFSNDWYSQCLN

>SoG_04303.T1

MLELPVSETTGRRGSIINFASLLTFQGGLFVPAYAASKGAVAQMTKTFANEWTSKGITVN

SIAPGYIATDMNEALLADTEREKSISARIPAGRWGEGDDFKGTAVYLSSKASGYVSGHTL

LVDGGWMGR

>SoG_04315.T1
[truncated: 562,968 more chars]
