# Supplementary material for: De novo genome assembly and annotation of rice sheath rot fungus Sarocladium oryzae reveals genes involved in Helvolic acid and Cerulenin biosynthesis pathways
Source: BMC Genomics. 2016 Mar 31;17:271. doi: 10.1186/s12864-016-2599-0 (PMC4815069; doi:10.1186/s12864-016-2599-0)
Supplement: Additional file 9: — Protein sequences of Cytochrome P450 genes identified in S. oryzae genome. (DOCX 184 kb) [file 12864_2016_2599_MOESM9_ESM.docx]

**Additional file 9:**

Protein sequences of Cytochrome P450 genes identified in *S. oryzae* genome

>SoG_00091.T1

MKRGESLALIRTWHQEYGKTFKVQLGRPRVITIEPKNVQTVLALKFKDFELGERNKALSP

LLGQGIFASDGQVWEHSRALLRPNFVRTQIADMHVYERHVSNLIKRIPKDGSTVDLQTLF

FQMTIDSATEFLFGESIDSLGAGDSQPKFARDFNLSQEGLAIRTRLGPLMFLHKDRAFSE

ATVEARRFVDQFVQRAVEYRASHSKEAGASDASEEGYVFLYELSKRTADRKMLTDQLLNV

LLAGRDTTASLLSITCFTLARRRDVWDKLREEVLALGDKTPSFEDLKSMKYLNWVLNETL

RLYPVVPLNSRTAVRDTFLPTGGGPDGKHPTFVPKGVDVVYSVYSMHRLPEVYGPDADEY

RPERWATVKPGWAYLPFNGGPRICPGQQFALTEACYTMVQIVRHFKSMESRDDRPFVEGL

TLTLASENGTKVALTPA

>SoG_00504.T1

MELTILAVQWQLLVPFITAFSLALRWKAARQAIQAILNDSLSWLLNVTLNIWYPVRHVHH

KSALPTVPYRFPNGQGDGAKFLHGRENSLIWQRQYGSLYRIWSGMAPEVVLTKPEHIQEV

FKDSDRHLKAKNNNSGYFLGELLGKCVGLVSQNEWKQMRSVCEVPFVRSKSVTYIQQMER

RTDRHFRQLWKTSNLSESVIDPAQDLKMLPFWIVAEILYGNLTADMEAQLAELAPLRESL

FQYFLKGGVTRYEWSKYLPTAANRHLSEFKSRWAAFNRQARTGAHPNTPIASYYSAIDEG

TLTEESILHTLDEVLFANLDVTLGGISWNLVFLAVYTEAQARLRAEIAQERAKARDGGAF

ESYILRSDTFVAACISESSRLRPLAAFSVPQAGPTGRVVGGYYFPPGTNFIVDSYALNQR

NEYWGEDSQTYRPERFLERSAVKDRYNFWRFGFGPRQCMGKFVADMMIRVLLVHLVENYD

LAMVDEDKEWERNPENWINHPMMKLRCELRQSKGDNM

>SoG_00521.T1

MLDLADSDEVPNKVRKWHQDHGDIFYTKIGGSDFVWLSSPKVVKELMDKRSSIYSSRPAL

PMAQDVASGGQRQLFMSYGPRWRQLRKHTHALLNLNASIKYMPIQDFESKQVLRDLLDNP

KEFYTINRRYSTSVIMLVTYGHRVPNFDDPLIKKIYTVLDNVTYITAFGAHVVDAFPSLA

SWPQWLFGNWRKKAIKIFEHDSKIYLDLWNRLRREVDNGTARQCFCKDFYLNDPSKNGID

DLQAAYACAGMIEAGSETTATALNNWVLCMLLFPDTFKKVQEEVDRVVGKDRLPQWEDEP

QLPYLRAMIKELMRYRPGNKFGMHHATSEDDWYDGMFIPKGSWAILNWWAIHHDPTIHPN

PDVFDPSRYLDKPQTAAEYMNASDPYARDHFIFGAGRRACPGVHVAERSLFITIVRFVWG

FNLTKAKGPDGRPIEPSTAMLPGWLSVPVPFDCDITPRSAGHEKIMREEFAKAEAAGI

>SoG_00528.T1

MGFDLGHLQMYLTLLAGGGLIYTAGFVIYQVFFHPLAKYPGPFLAKITDAHQLWHGYKGD

RHLEFWRLHQKYGKVVRFGPNSLSFNSNTALKEIYGFKANVGKSEFYNAFVHPAANTHNM

RDKEAHARKRRVLSNAFSDSATKEMQRYILNNIRTFCEQIGSMAGSSSEKKGWTAPKAMS

DWTSYLAMDILGDLSFGRAFQMLEKPDNRFALGLVEAATKRHLICGTMPIVDKLKVDKVL

FPEIAAGRARYMKYSKGQLEQRTALGEETDRRDFFYYLLKARDPETGLGFTTPELWGESN

LLIIAGSDTTSTAMAATLFYLTRNEHALRKVTEEVRSRFSNVEEIVHGAALSSCTYLRAC

IDEAMRLSPSVGGILPREVLAGGINIDGERIPAGTVVGVPHYAIHHNANYYPQPFQYVPE

RWTAGAENPLSASFERRTTADEVSLASSAFCPFSIGPRGCIGKNLAYVEMTITLARTIFL

YDMRRAVGVEDVAEGRKDLEWGRQREDEMQLVDTFTSAKQGVMVEFRAAQRD

>SoG_00604.T1

MAVLSTLPSIRTLAVASIAFFVIYRITSRLSLAARRRALKRRTGCQPPVDLDALPSRAAV

ARRYWPFDWLLGLNIGNFVYVGELARQHRFYPARMEIWAAAAPSKTCRTKLANLEWTLTQ

DVENVKYVMATDFDNWAFVPGREHGLGQFLGKGIFTTDGADWAHSRNLLKPNFTRFQVSN

MALFERHLQELLAVIPLDGSTVNLGPRFFCMTMDIATEFLFGASSDTQRRGENKEFSDAF

GYCQDHGLALLRLGKLGSWLPHPRKFRRARKLVYDFVDGYVDAALEEKRSLGDLKQDASE

KGRYVVLHELVQQTSDRVQLRSESLAILLAGRDTTASTLTTLWFILALYPEVWRKLQAEV

ATLDGRHPTFEQLKELKYLQAVLNEVLRLWAPVSLNSRICLRDSVLPRGGGPDGSAPLFM

EKGAQLSWTLYSYHRQRDVWGEDAEEFRPERWLGEGAMRFGWHYTPFNAGPRICLGQQFA

LSQVGYVTVRLCQEFDGLELRGELREWQENHSLIISHSEGATVSLRKRE

>SoG_01004.T1

MEAVANKQSFAKSHNQLLWSAGVLVLGYLFFSLRSKEGIADDIPVVGVVPWDLFYTKTKQ

RFAQDAVAILKSGLRSDAPFQVVSDKGAKIILPPKYINEVRSHPDLDIDEANNMQFFVNW

PAFNSFKPFTRFHIMQDMIKKKLTGSLTLITEPINEECTIAIEKLWPQTKEWRPTYFLQE

ALLLVSQVSSRVFLGAPLCRDQRLIEIMRDYTTVLTPAAQKLREWPAILRPLVFPFIPEI

RDLLKIVDTAHKILDPEVEKRRALRKSAKDKGEHGGKNLDSIEWFDEVAGGRDYDAVGSQ

LCLSFAAIHTTSMSLARCLYELIPYPELVEELRQEVIQAVEEDGGWQKTTLTKLHLMDAV

MKEAQRLSPVGAAPFRRTAKRQIRLSDGTIIPKGAEINVSTLHMQDDSWYPNAEEFDGKR

FLELRMQEGKENRYHFVSTSPDHMGFGYGPHACPGRFFASNEIKLCLAHLLMKFDWKFAH

GKKPEDFMFGTDKAGDLSAQLLYNSSTIYLSRILASDLALPAILLIGALTTSAIGLAMPN

LHNGASLLKRFDNPGTSVEEDFTIPEDTPEGRHSIYIDAAGVTDP

>SoG_01096.T1

MGIVELVLQNAGKAGLVVVAFGFLYFIYSCIYNLYFHPLAKYPGPFMAKISPIYSIWGLF

RGRWPFDVHQLHLKYGPILRTMPNELTFVDPEAWKDIYGHRQGHPQFHKDPIHVGSVQDI

PGSTTLTMADDANHSRQRRTLAHAFSQKALLEQESIIRGYVDLFVKKLQPFAENGTPANM

CDWFNFTTFDIIGDMAFGEPFGCLREGKFHAWVSLITETIKAGAFEQATRRMFTTGSFAQ

TFLCKFIPESLRRKRFQHLELSKEKCLK

>SoG_01168.T1

MLPIVILNETYAHPQYLSLAAGVLCYFAYFHHGEHHLHGTTYIKIHTAVIVGSLIFLYLR

FNLQLADALFLALRYDTLFLSGLFGSVLVCRALLNPLNAFPGPWTARITSFDMTLRIRKG

QMYKTLQDLHGKYGYFVRIGTGELSITHPRAVQDVFGADSKCHKSPWYDISRPQDSLLLR

RTAKGHAELRRVWSAAFSTKAVRGYEQRIQPYRNKLIAGLDECVGEMIDMNMWLALYTWD

VMGDLTFGHSFGMLDTREQHWAIAALNSGMSVIGQHLPMWLFRILVSIPGAQKDFKIMLD

YATEQMLDRRKNEPEIPDVMSHIFVPYKNGKPWDATAINLMAGESHLLLNAGSDTTRTTL

AVALFELAKKPEYLQKLYEALAKHISESSSEEILHDQIANIDLLTGVINEALRLYPPNPS

HPTRVTPPEGAMIAGKFIPGGTQVFTPQYVIGRDERIYPQANEFIPERWYSSPELVTDLK

ATAPFSIGPYNCIGKPLAMMNLRVTLARIVMRYNFRFASECSNPVEEFVGRMHDHFTLQG

GPLYLKLERRNQGRE

>SoG_01175.T1

MADWATCLSYLSINLQLFPWRIRMGFLVSYISEPTSALARSQDRPSGDLYLDVANKYPGQ

DLVNLNFKRDLLLATSARALADILVHRPYDFAKTKNARDWLRLILGDGLVVVEGDRHKFL

RKNTLPAFGFRHIRDLYPMMWSKSETLIRALRQEAGRPDPVAQRGPGVIQLSDWASKVTL

DIIGVAGLGRQLNAVEKKQDPLTSIYERLLNPTREMIVYSAACIAFGYDRVKLLPWKMNK

SFADLISSLDDVCLDLVRDKREAILKKGDDHVDILSLLIKSNNFSDADLKDQLLTFLAAG

HETTASAMTWACYLLAKHPEVQDALRREVNDALPADPSADPSLDLGGVLEQLPLCNGVIN

ETLRLYPTVPQTVRQAQKDTLVAEQPIPEGTAIVLSMWQINRSPEIWGDTAAIFNPSRWI

SEDGKPNQNGGRASSYEFLTFLHGPRSCIGQNFARAEMRCLLAALVRSFSWELAMDDKDV

LPKGVVTIRPLNGMYLRLRCLSD

>SoG_01277.T1

MFAFSWRSTGVVALAFTCARALSQGKSTSAIQLVVLFAGFWSLALAAWAVWLVWIYPLMV

SPARHLPSPSGNHWAMGQGLAIMKEIPGGAELRWMKEIPHNGMIWVRGFFNQWQLMLCSP

EAIAEVTVTNCYAFQKPPFISGSIGRIIGFGVVLLEGDEHKLHRRKLLPAFAFRQVKGLY

PVFWDKACEVVRAMTVEAASKDDGLLELQDWASRCTLDILGVAGLGRDFGAIEDAENPLV

KSYGYIMRPTTTALVLNLLRQFLPGKLVSVLPVRQNDDLNKVSEQIRTLCLDLIAAKKGK

LETGEQSDRDILATAIESDAFTDEQVVDHMMTFLVAGHETSAASLNWAIYSLCKHPDMQV

RLRQEVRDRLPAIDSGNSITHSDIDAMPYLNAVCNEVLRLYPVVPSTVREPIQDTMIQGV

LVKKGTRLILPAWGMNRYAKYWGPDAEEFRPERWLSGGSSDPSQPNECNDITEKTKAPTK

PATSTRGGATTPYANMTFLHGPRSCMGQSFARGELACLLAAWVGRFEFTLRDQGDIGWKT

AFTVKPGKDGLWVKAKALGEF

>SoG_01665.T1

MPGSFASRAPIPGLSDFLVATHVPLILLLLLLLLGPPFSLLLHLPRVFLHTLIKLTPPCL

CTPAQKPQIFIRPPLLLGLAIHRPSSCNQGNRVLLHSIPSTFNTTNQPAVLSYLLTIVSR

RTLFSIHRREKGSFGEARQGKDDDRAGATSLNPHQYIPPDAFDAGILYIVAPVGRNFDWR

AKFLHLEKQSNLSYARGIPDVELSTRSPADTQRRPREGVSATKDLSTPERPPGIRTLKEE

ENLGLNRAFESAVRPFQAWREIFIGRRPATVERDRLAAAAFVTAASNETYRHPITRPLCA

HLCYFLLESGVGDXRTLVLSFSFFSLRVPGKAERARRTQPQHFFFSGNHQRLHLILEILF

IHDDSIINRPRRSYRTQHACPAITLNHDPRLHPQSASNAHHGVLAFRLSFLRLTNETYSR

TKDTISLPSAAYKALHSRPQTKADEFLQDNTWFMGQTLKFLEVPWIPNLFRQWQATWPEA

PFVRYLGLFNSETLVANNITAYREMLSTKNSSFVKPDGARRVANVIIGDGLPFAEGHEHR

QRRGVVSKPFSTSRVKSFMPTLQSKARQLVDVLSRQRDASHISEVETPIWKTVLDVGGIE

ALGVDLNHLESNESSLHELFTQTMQQPVWGHIIHYLSSYLPLRQLPFDINKDFVRNTSEI

RRILWEHVSRRRTMWENGAWPAGKEPDALQCMIEQGTATWSDAEIVEYIMNLMVLGHDTT

ACALVMTVYSLAHNPSCQERLRSEISTLLADSHTPTFEELDRLPFLDNFLKEVLRVYCPL

QYIPREACQDVQVAGTFIPKGTIVQLSPAMINLNPEIWGPDAESFRPDRWEKLEGPAANS

AYAFQTFHNGTRMCFGRQLAMTEMKSVLVELLSRFRVEALDLGKPLELASPSFTLRPKEK

LRVRLSELSASCPVVDSEKA

>SoG_01788.T1

METGAWSSSLPQPLADLGTSWYLPSGFDKYQAAGLIFCAAAAITVLSRFNSKDSDKWAVI

NPASAFDLFGKARRDDYQRNSMSYVAQGVKQFGEKPFKVLATHGDLTILPGKWADHIRND

PNVHFMKSIHEDFHADLEAFGSFAASTSDDALLQSVARKQLTKYLNKVTVPLSLETTFSL

NVNYGNLPEWKEFPLYPTMLDVVARISSIVFLGDELCRDERWLEITKNYTVSAFRCAEEL

RVYPKWYRPIASLYHPGYQRLLQAIVDSRDFVGGVIKRRRELAARGEYTPSSTDAIDWFE

EEAAGRPYDPALCQIALSTAAIHTTSELANGTLLILAQRPELVEEIRTEVVQVLRQDGLK

KISLYNMKLLDSVMKETQRINRPALRLNRKVLKDTILPDGSVLRKGERFAVSTSLMGSPE

VYENPDEFDPYRFLRYRDQEDKKSHAHLVSTGPAHLGFGHGKHGCPGRFFAANELKVLMC

HMLLKYDLKLPEGYVPKLRFSGSSASSDPKSRILLKSREPELDLDALEG

>SoG_02377.T1

MGILEEVAAHPLAQSFQSSSLSFQISIVLAALIGLSVVYHVASQLLFKNPNEPPMVFSWF

PIIGSTITYGMDPPAFFKENREKVHSLSRTVSCGHFGDVFTFILLGKKTTVAVGPAGNDF

ILNGKLKDVNAEEIYTNLTTPVFGRDVVYDCPNAKLMEQKKFSSTQFMKIALTTQAFQSY

VPIISNEVQSYFKKNAHFKKTTDVCDIPKYIAEITIFTASHALQGSSIRNKFDESLASLY

HDLDMGFSPINFMLHWAPLPWNRRRDIAQQTVAKIYMDTIKERRAAGDHDGEYDMMKHLM

NSTYKNGTKVPDHEIAHMMIALLMAGQHSSSSTGSWIMLRLAQNPHVIEELYQEQVKALG

ADLPPLTYEDLAKLPLNQSIIKETLRLHAPIHSILRAVKQPMPVPGTKYVIPTSHSLLAA

PGVSATDPNYFPEPDKWDPHRWESDSPLAPKLPTGRDEDEEKIDYGYGLVSKGSSSPYLP

FGAGRHRCIGEHFANVQLQVIVAEIVRLFKWENADGSNSIIGTDYQSLFSRPLQPANIRW

TRREA

>SoG_02481.T1

MVLLMQLNLQVTLISFAALVLLALAKSWHSPRITRNGKPLRHPPNTLPIVGNGILFLQAR

QKLFFWFTRCIHLTGPETLSISVPSLPPGVIISSPANLDYVFRNEGIFQKGHFFTHRSRD

LFGAGIINGEFEGADGGGTAGMRQAEREGLGRCGRTTKGGGSAGGLARDHDSGHGKDGLR

REDARQRRTKAFEHASGATAERFQNPLWQLTELVTPGGFKLRRSLSIIRSQGKQIVSRAV

AARHKVPPSQRQPRTTGAEGEEAIGHVSGSLIQSLLDSIGDESLVADSALNDLSAGRDTR

DTVAQVLIWTFYLIMRHPEVLQKLRGEIQGPEGSIVPPDDLFAPQRLTPTNLPYLLAVFY

ENLRLYPPIPFEIKQAQHDTTLPDGAFLPATSVVVWCPWAMGRSFDIWGPDACEYRPERW

LGEDGQVQHRSAAEFSVFNSGARLCLGKRMAECMAVRVIAVFVASFDFEPAYEGERESVS

SLTLPMRDGLPVRLSQSSKE

>SoG_02632.T1

MGLVDQVWDNASPQNVAIVTAAGITLYYAWFRVDEHVRIRRLGKYGPTIPHKLPLGLDFI

VRSVQHTLSHTNLEFWQDFIFRSAKTRTPTVTGRILGQRIVFTHEPENIKAMLATQFSDF

GKGEPFRAEWHEFLGDSIFTTDGAQWHNSRQLLRPQFTKDRLSDLHCFEEHFQTLVKAIA

NGGPLDGEGQYIDLEKANGRVLDVSDLFFRFTLDVTTDFLLGADTKSLCTIKNEFANAFN

AVQQFQNVLARTGNLKHFVPRYKMRRSLAVIENFINFYIDRALRMTPDELAAHTKTDEGY

TFLHELVNATRDRKALRDQIMAVLLAGRDTTASTLSWAIYELGRNPAAMRKLRREIVDMV

GDEDKLPTYEHLKNMPYLKAVLNETLRLYPVVPFNVRLALRDTTLPRGGGPDGSEPLPVL

KNTPCGYSTLCLQRDPSRYPPVSEDFADPAIFSPERWAKWHPKPHDYIPFNAGPRICIGQ

QFALTEMSFVLVRLFQKFSRVESHMQEVDGGNPLLKADIVLSPGQGVKVSFHQDDVKA

>SoG_02715.T1

MSDPPFSALFSNLTWRPTRNWALAVITYHLLTKPELLAKLRKELNETIDDPLQLPAWTTL

EKLPYLGAVLQEGLRLSYGVSARTARVPTQESLLYRGEWNKKPVEHVIPKGYAIGMSAAL

THHDESVFPDSYAFIPERWLDENNERRKDVERGMMAFSKGSRACIGMNLALCELYLVLSG

LALRVLPEMQLYETTEDDVLYDHDMFVPMTKKGSAGVRVTMA

>SoG_03005.T1

MPSSEDIGLDMFMATRLAEVAHVAKDIGYARILGTVTAAWLLWKVTHALVLSPLRNVPGP

MLARLTSKRGDLDNFSGRVCQTADHDIARYGEVYVYKPNAVCISNPDDVRRVLGSQEFRK

ASFFDIFDDGSTKNIVSQRDPALAARRRRQMGPFLNYGYLTKMEPVIQRHGYLAIRAKWE

GLLAESEGPAVEVNYRIDTQLVTFDIMSALAFGRDPSSISKGKSSITEWSGIIMKLLENP

VVLALLSLIPFPMLMRPWKKMYRDLAVYSKESGRMRKEFLATGAPAPADMLQAFIEAEDP

ESKVKMSEQEVQAECIMMMLAGSETTSSAIMWVFHLLLLHPEKLRLAVDEVRSAFGPDHL

ISNKDVLTKLPFVEACVFETLRMSPTTAGLTPRVSHDRGIQLQDHFIPPGTEIYVNLRSV

NMHESIWEEPARFRPERFVGNEEAKKTLFTFSYGPRNCIGRNLAWVEMLTIVANVLKDYD

IALSPDCEWRPENVDENGNPKLLPAKCFIASFPSNPERDCRMMVSRRSDLSV

>SoG_03048.T1

MAEYLSMIPEDACSRAALAVVGGGTAYLLSLAIYRLYISPLSKFPGPKLAALSGWYESYY

DLVHQGKYLFEIEKMHDKYGPIVRVNPFELSLRDSDYYDTLYVMGSVRPTNRHEAFVDGT

VDFKGSHIATIDHHLHRQRRKPLDPYFSRQGISKLEPMLAELTEKLITKRFESLKGTGKI

VRLDHALTAYSGDVIQRLCIDEPPNVFIEDPEFAPWWYYMFHHGISMLPVFMAMPWLISL

IRLVPLSVQARLSPSAQTFNDFKKFCDNQLQTVKKEQAAAGSKGSLLLNGRPTIFRQLLS

SDLPPSELTDDRLSKEAQILIGAGTITTAGTMCFISYYIMTNPNIKKRLQEELAPIMADY

PRRQPSWAELEKATYFQAVIKEGFRLSYGTMHRRTRVSPDQPLQFKDWVIPAGVPVGMSA

YFCHRDPKVFPNPEEFLPERWLGEVTAAMKRNFVPFSKGSRHCLGQNLAYAEINYVIAAL

FRPNGPDFDLYETDERDVKPTHDLIVPLPSLESKGFRVIFH

>SoG_03059.T1

MFFNLLAAAGGLIGLVYLIAVFPRLQQEWKLYSHRSELPPGPKTIKTGIRKPWLWFQELT

NEHGDVVYLQLGPTPTIILGSAQAAWDLLEKRGSAFSSRPRFIMGGELLSGGMRGLMAPY

DAFWRRWRKLLHSGFMQRASEGYRPVQSLESKVLMRDLLVAPGMFRTHLERYAASVIVTI

TYGRRVDDVRNDIVVRRNAEAMERLTLVK

>SoG_03504.T1

MTGLMLLVRHVFDLSVDLAAWTASCCIVCILLAASYRLLLHPLAGIPGPKAAALSNAWLA

YHVRNGNSVHIGRYLHEKYGPMVRVGPNEVWFNSKTSFKTIYCPGSRFDKSDFYLATALP

RPELDWRLDPISADTLDLLSERDMKRYRRQRRAIGPIYHASNLSRYEDAIDGVLTRAIQR

LKGLQNEPVDLKEWMHMIAVECLGAVVLSWSPGMIKRGTDAGTSYHSYLSWRRKSVFGLF

PTITKLAYLSKTLDRVFSVIWGITYKPPRDFRTFFPDVGKRVSKRINVALHRQIKPGTHH

DLITDLIELHVRKPDFTETYLKRMAVTNFGAGHETLAATLTSTFTLLASDADSYQRVATE

VRGHPSPTRFAVAFDLPLLQAAVKEAKRLRPVIGMSLSRRVPHGGVVLHDRFFPAGTTVG

CSPTALHCNADICGSNPRAYNIDRWSDPETSRDMDTFSLSWGGGSRTCPGRHLAELIVFK

TTAALVLHFELEVTVPREEDMPSYFMSMPTGVRARFLNAN

>SoG_03547.T1

MQSAPDQSADIDRNGNLSVWCVLLLLLLLLGATFNNTNEPVDEGMWMAWGLLLLSMAVCY

IKTAAARPDLNYLIAYCKAKDPLQARFTLTPIRTTAKRRRGPPGNMAAAIITYALWVVPA

AWALYKILTFGSREKGLPPGPPTIPVLGNAHMIPLKDIHILTATTLRFKEWAQKYGSVYS

LKVGRGTLVVLCDREAVFELLSRQGAHYNSRPPDQQVNAALGDENIAFMYEGPRWRAQRK

IIATYYSPKNLDSTLQPVLVAEYVTPPSPRSGSSPSRGTHLADMQTRISHLMNDILANPE

NFRDSVKRTTASISTDTVFGHHAPDCDSYWAKAAYLSIDQAIDVIAPGTYLPVDQLPILK

LLPDRLNGPFREAKKRFPIICDIWAKAREQVEERRRHGDRRESLIDNILDENLDLGAPHT

YSELNNFFGAIQMASADTTSGHTLASILFLSQHPDVQAKAQEELDRVCGTERMPAWSDFE

KLPYVNCIIKEGLRMRAALPTGIAHLARKDTWYKGMFIPQGSSIIVPPTAINNDRYASGD

PAEYNPDRFLPVAGKLAPELSSSPRWQERDHYSYGVGRRICPGMHLAERIQWRIMAQLLW

AFKIEPEGELSTGYDMFEEAFLHYVKEFKVRFVPRSEKHAEMVRAQFKDNEEFLKRFG

>SoG_03551.T1

MRNQTSSQRLAGIMDAESLPLVLRGLASRYEKHLQALSFAIPSWLHRLGISGTLAAVVIA

YATFTVLYALYLSPLRRIPGPLAARLTAKRGVWSILTGSAASDAQADYENYGDVYVAKPN

AVFLCDPDDARAVLAMAEFRKTDMYRIFEYEGVPNVSTLTEPAQANRRRRQLHPFFAYSY

LAKMEELILRHGTRALNARWGAKIEADAAKGERTIVNYRLDTQLAMFDITGALVFGRDFN

ALKTDNLDYTKWVNNTLTFMLLAHYFPLLKRWPFSHLVASLRRSYDDLVAFSKESIAIRR

ALLDAGGEKPADLLQALLDSEEPDSKVRMTPGEVQAESIAMLVGGSESTSSVISWVIHFL

LLYPEDLRKVVEEVRGKFGAEHVVTHAECRAELPYLEACIYETLRCIPTASTSFPRISDR

KGITIKGFYIPPSTEIVTNKCAAHIHKESWESPFDFKPSRFLDNNEAKRNMLSFAYGTRF

CIGKNLAWVVMIVTLANLFKDYDVALPETSLFHPGNVDENGRPRIMPTKLGVATMPAHPE

RDCLMVLKRRVE

>SoG_04009.T1

MPSPTVSVQPWQWDDHCKQAAVSTGLSVVYGTVPGGSREEQEETVEAVQDFVSRLSNAAR

PGTFVVDIVPWLQYLPSTSFEKDSAMFSKWLQVPREEVKRGGERESIGRSLVENPSLSDS

EATWLAAAFVTGSMTTMGVLGWFVLAMVLYPEVQHRAQVELDRVVGRDRLPDFEDYKSMP

YMQAMVREILRWRPVDPIGLPHSNGKDIEYKGYVIPKDSVLLFNVWGMNRDPEVWGPDAH

DFKPERHLNPETGELKIVTGGVRESHASFGFGRRICPGRHVAVDLVYVFSSMVLWSFNLS

REKDPEGNPVPIDVDGSIDDGLIMYVASSFRSHITVVLLCSPSTRNL

>SoG_04044.T1

MLFDAGPAALVGLAFALWYIVSTVRQYLRLRHIKGPAVAGFTQLWLIRCVGGGRTHLQLW

EVCKKYGDIARVGPNDLITSDPDLMKRMLNVRTKYKRSAWYDAMRLDPTKDNVLSQRNDD

LHASTRSKMAAGYSGKEVDHLESTIDENVQRLIDLLDAKYISQGKAFEFGHKASYFTLDV

ISAVAFGEAFGDVETDSDVHGYIGAMEESMPTIIVSTVMPWVMKLLQIPLFKGMLPSEKD

AVGVGRTMAIAKRVTAERFGPHAKVQRDMLGSFIARGLKQEEVESEILMQMQVLSLLLSS

IRAIVLHIISNPRVVEAMRREIDQASPSLPIITDDEARAMPYLQAIIKEGLRVHPPVVGL

MSKEVPPGGDTFKGVFLPEGTQVGYCAWGIYRRRDIFGQDADEFRPERWLEASPDQLRLM

EGTLDLVFGHGRWACLGKNIALMELNKVFVELVRRFDLVVVNPVQPWTSINVGVFLQSNY

WIRGYKRAVA

>SoG_04125.T1

MSTSVLARFFREATVTSYFTVVASATAVYLLGSAFYNLYLSPLRKYPGPKLWAISQIPFS

IAWTTGQCHKKILELHLKYGEVVRLSPNQISIGYPEAWDDVMGHRKRGQEENGKDPDFWR

GDDKLTLVGSSRERHSRLRKILSHGFSAQAMMDQQPIFQQYATLLVKKLHEACTSGKSVD

ITSYYNWTLFDMAGDLIFGEPFGCLEETRYHPWVKLIFMHIKGIAISTAVIRFPFSDALI

KLMTPKSVAKDIQAHHDFTTSQVAKRMAYDNPRPDFMESMIRAYEENRVTKGELHANAHN

LIVGGSETTATTLAGVTYLLATNRDKLQKMYEELQATFKSENEINLISVNKLEYMFAVLH

EGLRVYPAVPTTIPRKTPPEGIQIGEDFVPGNTVLGVWQWPMFHNPKYWTDPEAFVPERW

TGDAKYENDKKNACQPFAVGPRNCIGKNLAYAEMRLVMAKLVWNFDFELDPRSKDWIDQN

KVYLLWEKPQLWLKLIPRKREDLALCLSPWFLT

>SoG_04140.T1

MQYLRLRHIKGPPVAGISILWYLRVLLRGKMHLETLAINEKYGETESSWGESKGEPRIST

VFAGLVSAGRSYTPKGPLARIGPNDVLVSDPDMVKQIQAVGSKYVRSYWYDATRFDPFRD

HVLSMRNEAEHNARRPQLAPGYSGRGITGLDQFMDEIISQLMALLDDYTARGQRLDLVRK

LQYFTIDLITRAAFGAPFGFMAKDEDINGYIQTTESFAAVLGLATVFPFINNIRRSRLFA

ALLPMKKMAAGHVEIMNKARDTAADRFIPGRMVQQDMAGSFIAHGLNQEQLEGELALTLL

AGSETTAHVLRNTLLHVISNPVVLAKLRAEIDSTAAALPPSESGSSVPFASAQKLPYLQA

VIREGIRILPPVSGGFAKESPPGGARIEDTFLPGGTRLHINFWGLTRQKRIWGDDVEEFR

PERWLLDETDPASKERLKEMTVVGDMSFSYGKWQCLGKGLAQKVLAKSIFEPMKSSNHFT

FNQSDFWVRAKRRR

>SoG_04186.T1

MSFTSDILLSNFVIWAVGLWLVILWITLKPGKPIKGLKATFPDGPKPWPIIGNLFFFIKL

FKDPDNVLMKLAKDYGGTCMLWFSSAPVMIISKAEDAKLLLDKNSFREKAWPWRLVTTGV

GPQFRLLRRVYHNLLGPQQSAGFRKYQDYESKIMINDLISMPEQFLAHTERFAISVIFSA

VYGVRLVQLDHPTMNEFYSVWEDMLNYFQPGTLLLDYFPVLQRLPERFQPWLKLAASLRT

RESALHRAFLRTLKQQVAANKAPDCFGTELVKIQDHEDINDEKAVNILAMLIGAGADTTS

SVLQSFFKVMAMNLDAQRAAQEEIDRVVGPDRLPSWDDEPSLPYVRSLIKEVHRWAPIGS

LGVPHATSEDDTHLGRLVPKGTIIFPNLPALSRDPAVYANPDTFQPERFLEDKLHANASA

NDPDFRKRDHFHYGFGRRLCQGIFVAEASLYIVIARLLWAFNITEEAGAPPLDLGDKIGR

SSLP

>SoG_04293.T1

MLNELLGGAVPWTAIVISAGLYYWYGGQPSPFPIINSHPNDFLGRKAQNALQQRAGEIIR

EGLATFGGPVSIRIPYGLKIVLPANLAGWVKSNKDLDHRELVKEDFAAGYHGFEAQTTLH

SQDEVLKTIIRSKMSSNASIIPVINKSLSKGLPLLWGNPESWHTLNWYPDSTGIIARAAC

SVFVGPEKAEDPEWLELVQSYVGSFFPGVSDLNSYPRWSRPIMQWFIPNAATCRKLVARA

RVIMNEVVAQRQEEELSAERDGRPRPQYNDALFWSQEAGASAEPGDVQLALTMASTLTTA

EVFRHVLLELARDASLAQAVREEVIEQTKAHGVTLSALSNMVLLDSVLKEAQRLASGQVV

LERMALKDTKLPNGQVIPRGSHIMVDGTNLWDPAVYPEPNTFDGYRFFRRRQDGDSSVQF

VQSGSDFHVFGGGRHICPGRFLANNILKLALSHVVLKYDLRLEDEGVQSVVPYGFYTIIN

PMAQVEVRRKTTDDGFLDL

>SoG_04315.T1

MTSFTVVAAGGAASILWLLYLVLEPLVLSPLAKVPGPKLFALTKWRLAYEDWKGTRTQKI

DDLHRQYGTVVRVGPNEVSFNSQSALRTIYGPGSRYGRTQFYRMFDVYGRQNLFTFHSSV

EHGRRKKLLSFAYSKTNILNDAVACMVEGKVDKFMKLIDSEPEHISDVFSTLHYYSLDNI

TAFLYGKYGSTSAMEGTQAHRSLIGDILDPARRTLSWFTVHLPSITRWLYTRTDMAERLV

RPILPMQKPATYTGIRAFALEAYNRFRQDVESGKPESSYEADDESILSRLWKHSQNSKSD

RLDHLDIASECADHFLAGIDTTSDTIMFLIWALSQPGSAGFQEKLRQELLELPEEALTAT

GTTRAAASDKCAYLNAVIKETLRLYAPLPSYEPRSLDVGSEIDGFLIPAHTVVGCSPFSL

HRNPEVFHDPLEFNPDRWMGADAKELNRWFWAFSSGGRMCIGMHLAMAEMTTLAATLYRR

YQTTIAPGFEGATPGITARFELFYDPRFPRMMEHYCPIKFQEL

>SoG_04319.T1

MKKILFLLGRFHFAAHFHGTNCIFTSNIPSPKPSIIQLCAPSTRMPSLARAATGENIAVL

SVAVVGLFIGWKLVYAYYLSPLRKIPGSSWARLTTLRAIYNRLPKRVTRAALAEHRRYGD

IYVSRPDTVTISHPDDVRAVLGAADSWKIDVYHGLDDPIMSNLVTFSHPQLASRRRRQIS

PFLNSQGYLAKMEAVIMEHGILALMSKWEGALGDAGEEGESSLQINYRNETQLATFNIMS

ALAFGRDHSMASDGSLIVDWISATAVYVGVSINFKSLMTFPFSMLIRSWLKKYHDFVEYG

KQSVAQRKQALADGRLGEKPADMLQAFIDAEDPDSKAKMTSVEVQAESVGMQLAGSETTS

ASLTWALHLLTLYPEVLSRAVKEIRDQFPSDHIITYSDCRARLPYLESFIYETFRYAPIT

SGFMPRYSKRTMELQGHAIPAGTRIAFNLIALNNRPDVWDRPDEFVADRFVGDDEAKKNI

FAFSYGPRSCIGRNLAWVEIMTILANLLNNYDMSLPGDSVFGPGNVDESGMPRLMPSQCH

IVFAPTHPDRDCRIVLRRRAS

>SoG_04368.T1

MEITDTTAFVCAWLLLVGIVVLLLQKCRMFSKAAVRDPYLGLDFVYDNLFKSSVQSRLDT

RNKTFMQLGQTYCVQRWSHHTVYTCDSRNIKHILSLRFSEFALPELRSRAITSFLGSGIF

SLNGDAWSRVRSVLRPGFSKSRVDSVPELVERHIHSLWEQMPCGRETVDLEPVFFKFAMD

VATEFLLGHSTSEQRGSALRSKTDQFVSDYFLCSEAIVKKLRLGPLQFLTGSAEAFKAKA

RVLHYVDSFIAEAKMRQSHIEPKPQYDMLKELRKVAPDHSTLRDQVLHILLASRDTVACL

LSNLVFVLSKHPVVYSRLRKEVVGCCGSGRPSIEQLHSMKYLKWCVNEFIPTNGREALVD

TTLPFGGGPDGNSPLFIKKGMLVLYNIYAMHRDPEMYGADADSYVPERWASIRPGWNFLP

FNGGPRVCIGQQFALTETYYVIARMTQRFAAIESRDPREWQEGEALAITTKHGVSVGLIR

EDTSLGHENS

>SoG_04488.T1

MTQPIPQPKGIPLLGNIFDVNPNNTWLSLKTLIEKHGEICKITVLGHTLVFVGSVELAEE

LCDEKRFRKFVGGPIVEIRAAVHDALFTAYDHEPSWGIAHRIIAPTLTPDAVAKTFDEVR

STTQELLDKWATLGSSNKICLIDQLNRLNLEATTLTLYGNKLNCLDGPEHPMLQAMEDST

SEAMKRPTRPKLLNWLVHGRKFKKSIATMRKYAADMVEHRKANPTDRQDLLAALMSGTDP

ESKIGLTESQVLDNIVSMPIGSSTAPCLVTTAIYFLLKNPACIDKARMELDSVVGNHPLT

HQHLGRLPYIQGIMRESLRLSFAAPGFNIEPIPRESEAPVKLAGGKYEIAHDQAVIVVLA

GVNRDPAVFPDPLAFNPERMMGAAFEELPLGARRWFGNGKRECVGRHYAVQWNLGVLAML

IQNVDFAMADPSYDLEQDGWFNLRPVGFNVTVRLRGH

>SoG_04568.T1

MTDTTSRGLGLAAQALEQAPTTILYILGASFGYRVLRALYNISPFHPLYHFPGPRIAAVS

YLYEAYYDWILKGRYGKVIADMHKTYGPIVRINPDELHCSDPYFTDEIYAGPGRVRDKWQ

HQLNTGGAGPVSVTGFSTVPHELHRVRKGALSRFFSRQNVLKLEGEVREFSQLVADKMLQ

SGGGAGGGEAFDVKEAFNCYTADIISQYAFGEPMGFIAQDGWEPNFATWVKSFFNSAYMM

RHNALARRMAQVMPMLADYMGEDVRNVMRQMTVVIPGYIQAALRDPDNGRVFAELQESKV

LPPEEKTMYRLSGEGFNFLLAGTETTAATLTVITYHLLAQPRIYERLMRDLEGLDPADLR

WTELEQRPYLWAVIQESLRMMPGVSHRSARIARDEDLVYRSRDGRTEYLIKRGTPIGMTS

MINHWDEGLFPDPDEYIPERWLVDGQPNHKLARFLIAFGRGSRSCIGEQLAYAEVYHMAA

LMALRVLPRATLHETTVEDISYDHDLIVAQTKKGSISVRIKID

>SoG_04594.T1

MGELHTGLEFLFSRNYTGREISLGAALIAVLIGARLWSTRQRLPEAPIISLSNLPGAAGT

AADIAAYVKNGSVVMQAGWDKYSKKGLHYLIKTPTETLFVVAPQFTEEIRLLPDSKVSAM

KANSEIMQFRHTLHPRIEVDQYHNLVVQRQLTQSLEHTLKAYNKQDEGKKAFAEELGKFK

VPSDIVVWDVAFRIVTRTANRLLFGKELCRNPEFLRLSIDFSFVMFGGADIIRTYPEILK

PLVMWWKTSYGKTMSRLQALLVPPITNRLAQMEKHEKEGTVEEWNKIKPNDAIQWVLDIT

PLKDRDPSMLLYRMLHINIAAVHTSSVTFLDCVYELAARPEIHQELRDEIRSVFAEEGYQ

WRKQGLTKLVKMDSFMRETVRFNPLFSSNLDRIALQDFKLSDGTLVPKGTYLTVPSLSMY

MDNAYYEKAHEFDAFRFSRLRQEPGQERHHSFVQTQATYLHFGHGKHACPGRFFAANEIK

ILLVLALANYDISLPDPHAKPEPIWFTKGRTPNPKGVIRWKLRTDAPDYAAI

>SoG_04596.T1

MGLHELLPSALTQGEPVQQLVYIGAAAASTYCLYSIILGLYYLYFSPLSHVPGPKLWIVF

PFFRYLTFLQGQLERRVAAFHQQYGHVVRIGPNELSFNDTQAWKDIYGHGHKQLPKAVFF

HEPDGKPDHLIHASIADHSRYRKIMSGSFSEKAIRKQEPLIKVYVDLLMAKLRQMGSRGE

KVDLVKWFQLAIFDLIGDLTFGEPFGGVSSSEQHDYVRTMFLFIKAGAWLSLFHHYPLLG

KLASLLTPKALAEAHARQWKFAEDVATRRMNNKEQQGRGDFMDSMVETRNTPGGMTDQEI

VVNANILISAGAETSATTLCGAMFLLLQNPEKLAKAREEVRSSFKSEEEIDFITSTSKLP

YMVACLTEAMRVYPPLPSTLPRFTPDGGSTCISGIQVPAGTIVGVSQLAAYWDESNFHAP

RDYVPERWLSEVHKDPNSPFHNDHRDVLQPFSTGPRNCIGRTLAYAETRSILARLLFNFD

FELCTESTNWMDQRIIFWWDKKPMWCKVYPVERIA

>SoG_04763.T1

MISAAIQSSALVVVFLGLVTSGIYKGYQQRKLFRNTVARSGGPSLPGHSWIFGDLITVGK

IMGGKLPSSVHGHMVFYFLARRYPEIQRAGCVYVDLWPFSSPTLATFHPDLASQYTQDVS

QPKHSLLRIEFEDFTGMKDLVCLEGEAWRRWRAALNPGFSSQNVMALVPDFVQEAEVWKR

YLVEAADSGDVIKLEDRLMLATCDIISVPVLGASAKIQQGNSRLWWALKGAIALLYSDWS

PRSWFKVFNPLRPLILRYFNHVLREELVPLIKAQMQEEETNSSSSSPEGPKTVSKLALRA

YMKEREAAPPAERSRLEREFLDATVEQMKIFLFAGHDTTATTLCFAYAELSRHPRKLAKL

RAEHDEVFGKDASKASQLISENPVLLNHLPYTASVVKEALRMYPPVGTVRAGSPTLNLVD

PNDPSRRLPTDGFIVHDSTVALHRNPEFWPRPDDFVPERWLPDSEEKHDVRKHAWRAFEL

GPRNCIGQELAVTEMKMMLAMTARELDVEMAYDESDPLVMGSQFFQAHMPKELIAHPSKG

LPVRIKMRRS

>SoG_04897.T1

MLNLKDWLSAAFKCAAALPLLYCVLVPVYRRTLHPLRNVPGPRLASLTGLYAGYFILRKD

THRQVLRNHIKYGPVVRHGPDRLLFNTISAVKSIYQNPRALKSFAYNLLTELDGYQSTID

AIEKHDYRRRRLLIGRGLNDSTLRSIQPVIARQLDILLKGIEEGAKYEVSMNMTTLIRHL

MFDTMCLATFGYPLELQTNHRDRWMTESFTQLNQVTSTYLNVPDISLLRLHSVLSLLDPV

TEKRFADLCRRILSGSQSECPDIFTILREAWHGNEDMGADANEIFQETLLLLIAGTDTVT

TAICATLFYLSRERACYDRLAAEIRSAFSSAGDVDSGIRASSFPYLRACIDESLRMSPPG

AGVPWREQDSASKKAGEPLTVDGHVIPPGTQVGVGIYAVHHNEEYFASPFSYEPERWLDS

GSSPERLRLMQEAFMPFSRGSRACPGKSLAYAQVSLVLARIVRYFDFEKSPGALGSTGSR

LVRTAGGEVKEEFDTYDIFVSEHDGPQLMFKAREDMGHIEEPPACII

>SoG_05073.T1

MAIIDQAATFVSQRSLSEILGLMLLISVARFLWDPLDPREPPALRSRIPIIGHLVGRLYA

VWDAAIVQQLQRNKDLSFFPFLRHFIKKETDYDPRHEEIMQRDPGVIEENRKVMQSSMTL

KHVRGMTLTALNHMAGELDQITKKEPVEIDNMWIWFRDTMTMATCEALFGSENPINKTEK

YPTIMDDAWVFEAGVDRLVMAPWPSLTCRKIVGARQRLQIALSEYYINKSDEREDVSELI

RGRAAMLRKFGLSDADIGGYEVMVIHLALTNTIGAGFWFFVHVYTTPGLVEVLREEAQAA

IERKSNGEVSVDIAHINEKCPLLVACFRESLRLTDQFIGNRQLHADTKVTDSTNKTYLLK

KGICIQWPARFLHTSKKVWGEDAMEFRPERFLEMHKNPDSPETKAKLESFVPFGGGKHHC

PGRGFALAESVGVMLTMMLGYDVQPHNGTWESVGIPGKDAPGFASAIYKPVNNGEGWGVR

IQRRQGWQNAIWNGQRRYPGNWNQDTGGQASRRAMSTNNAERKRGDVDGLVNPPERSEAD

EKIWKAAIKGAKVEHNNHTSGEKPKQQQAKEQQRNVSMIHT

>SoG_05129.T1

MVFQGQYSKALPAAILSLIAWFLLRPKQRYPDAPWLRVSTKPGRLGVLEDQATFQTDSMA

ILSVGWERYSKHGINYMLDTAEGKRYIVAQKYLDEVVRAPDTHISALPASNDLMQLRHTL

HPLAAVDQYHFDLPIRKSLTQALGPRLQDIVEEGKLAMDEFIGYCKDYKPLNGMQTSFNV

VARTANRLLFGIPLTRNKEFHQLSIDYTFIMFGGADIVRKYPVFMKKLVMWWSTKLYPAQ

ALARKLLIPLLEERVREEKQARASGTAKQRKKPDDMIQWILDYSVDEELQPDRLVYRMLH

INIAAVHTSSQSFGDVLFTLGLFTEYQDELREEIIRIFRQEGGWSKETLTLLIKMDSLMT

ECGRIQPNATLKNARVAIRDWQFKDGTKVPKGTHISTNQLAYHLDDEVFKDANTFDPWRM

YKARQKPGEATKHQYVMTSDKNLHFGHGKHACPGRFFAANEIKVLLVLTLMRFDIRVTNM

TWEEVKRGVFYSVGRSPCDKAMVEFKSRDEEIPDDLRQMFL

>SoG_05347.T1

MMYSSFWLMPVSAVLFMFGNIVRQLLPRRKEQPPLVFHWLPFIGNSISYGIDPPKFFSQC

REKHGDVFTFILLGKEITVYLGVDGNDFILNGKHKDLSAEDIYGPICTPVFGRGVVYDCP

NSKFMEQKKFVKFGLTTRALESYVPLISQEVKDYVSGNPIFDGQSGVVDVTHAVSQITIL

TAARSLQGEEVRKRLTGEFANLYHDLDQGFSPINFIMPRAPLPRNRRRDVAHAKMNAIYT

DIINTRRMSGSELGPDMIWNLMGCTYKGGCPLPDSEIANMMITLLMAGQHTSASSVSWII

FHLSSRPDITEELYQEQLNALAHGSNGLPPLEHSDLAKLPLLKGVIMETLRCHNSIHSVM

RKVKNSLPVPGTDFVMSPGQILLASPAVTMLDERHFREAQSWDPHRWDNQLSMEEEEADL

QGLEQQEISRGARSPYLPFGAGRHRCIGEKFAYVNLGTIVASLVREFKFSTVDGKKSVPA

TDYSSMFSCPVRPGVVRWERRKKQRLQTYFEVGEKAKA

>SoG_05369.T1

MIFRSLAWCVGLAAAYKCCSLALNMRRNLAAAKRSGFPYVISPTTPVFMPWQLFHKVLVP

LIKLLPKPWWQTWLPLVLPDMSHHSGHDQFAIHGDMILLVCPDILFLMVANAPAMRTIAA

KREAFPKWTDSYEVLRIFGENVVTSEGATWRLHRKTTAASFNEKNAELVFHESIRQAKGM

LGLWTAEERKGKIIDSLESDCMRLTLNIISYVGFGMKMLWPGQVLTEEADSAASKYGSLA

PPEGHNLSFVDTMQYMLEHIVLLLVFPTALLHRLPFKAAKKAATASDDYLKYMDELLEVK

ENEARKGTRKADEEGMDLMGQMVRSKHEAEKRGKKGQGLSLSRDDIIGNTFIMLVAGHET

TANTMNFTLIEMATHPATQRRLQRDLDEIVGETDPDTWNYEALVGPLLGSMVGAAMNETL

RLVPPVTDIPKIARHKDQPLVIDGKEYTVPKGTVISLSVAAAQRNPNHWPTKPSKFRPDR

DDLWDYEPERWFIKDDAKGGSAADPENAPADEDFGGYMGPDTSAQLFRPERGSYMPFSDG

ARSCLGRRIAQVEVIAALAVLFREYSVELAVNEEDGDDDAARYKAAQERSRRTVEAATSQ

ITLKLHGDLKVPIRLVKRGEERFVSWVDR

>SoG_05747.T1

MVSAGLDTVPGNLIMGLAYLSTKHGHEIQERALAEINEAYPDGDAWEKCLVEEKVPYVTA

LVKETLRFWTVIPICLPRTSIKDIPYKDAVIPAGTTFFMVRTGFQVLFSEGAYFRLDTDC

RQNAYAADYDEDRFKDPESFIPERFLNDNEGGTPHYGYGAGSRMCAGSHLANRELFTAYV

RLITAFHIFPGKDPKQAPIIDPIECTINPTSLTTDPKPFKIGLKARDEGLLRKWIADAEA

RTQELR

>SoG_05762.T1

MEFFGQEVTLRKVAAALIFTLFVQGFFRLYHARSKFWALQSNHGLVTLPHSFLFGHLLLV

GKALSASAPDAHQQLSMWLLLENYHPELWAQGFFYLDLWPVQYPLLTCFDPGLIAQFTQI

ESQPKSRILKDQLSGLTGGHDLVSSDGGEWKASRAVFNPGFSGKNILELVDSFVEEIEVF

RRMLQDAADDGRVVKMSEQASKLAFDVIGNAVLGTRFHSLTKEKSFYTTFLKRLSWNFPD

WSLHYVSKVMNPLRPFITRRLDRAINRELCLYINEALRPGGPRQGPKTILHLSTEAYVKQ

LPEDTALPRSVLDLRHGYLQNMLAHLRAFILAGFDTTAYALCAVFWQLHRNEGCLRMVRE

EHDRVFGKDVKLAAQMIRDNPVILNRLPYTSAVIKETMRVQPVTGTVLVQEAGILAAALV

GAGRPRAASSEERVQTIRAWPQKLHRAGGGSDGDEGGHGSDSEGV

>SoG_05850.T1

MAVLSHFLVSRSESGFLELLAAVGLSCSVTALYYYLNRQRYPDAPLLRVSEKSGMLGVME

DTADSVTDMVKLLEVGWHRYSKHGVNYLMNLPREKIYIVAPKYFEELRQAPDSHLSNLAS

NNELMQLGHTLHKKLTSNQYHFELPIRKSLTQCLGPKLPDIVEEAKLSLVQYIGDCDEWK

PFVMSRLSFDIVTRTANRLLFGTHLARDEEFQELSIEYTEIMFGGANMIRNYPEILKPLV

MWWKTGIYKAQATARKHLMPIIEERMAEERRYMAEGRGEEWQQIKPDDAIQWVLDVSPPE

DRRADHLVYRMLHINIAAVHTSATTFLSAFLCLAMMPDVQRELRQEIEDVFRKEGGWSKQ

ALTYLVKLDSFVTEALRLCVMSSLKMGRITVKDWEFSDGTKVPKGIKVFCNHLPLVLDDH

YFANPREFDPWRMYKKRQEEGQANQHQFVMTSETNLTFGHGKHACPGRFFAANEMKTLMA

LMLMLYDFRAVDVKGGLEEVIKGHWYNMERVTSQTAKVEFRNRSKEIPEDLEPYFSLY

>SoG_06046.T1

MDEVREKVADGTSPNCFAKHLLEEQASLGMTDLEIAYTAGSPFGAGVETSAGSLASFFLA

CVKFGPQFVPRAQEELDRIVGRARLPEFEDLPRLEYVRAIASETLRWRPVAVLGGTPHAS

TADSVYKGMFIPKGSTVIAPLWSIHLNEADFPQPHEFRPERFMEDRQYPGPWGHSAFGWG

RRVCPGMHLGASSVTLNIARILWGFNVTPAKDEKGHNVDVDIFAYSDGFNSSPLPFPCTI

SPRSEEHVAVINSEFTAAMEDLQAYTAVHSKLS

>SoG_06058.T1

MCVHADASCVPSRLVHGAYLMDEGYFTLYVFMAVSASLLVLLCLRSRGSYQQLAAGVPVV

GGNSKGAILANRKRFRQEAQRMLIEGYKETKGGLFYVPSPLGERLMIPIKYLDELKTAPI

DHVDFVATFIEGPEMFEGKYTTMGSRSTLHPRVVKAQLNHYLADVIPEIEEEIVDAFKED

FPACDDWTPVPVVETLTLIVARVSSRMFGGRTLSSNREWVRSSINFAIDGFIGAQKLKSY

PHFMKPMVARFLREIRNIRKHYKAAEAAAIPLLEARARTKETAMDLLYWMADQAKGDEAD

PAFLASILLKISFAAIHTSAAAPSQLIFDLCEHPEIVDPLREEIEAVCGSEGRIGKNGYL

KMNRMDSFMKESQRFNPLLLITFERVVHQPWTLSTGFTIPAHTTIGVPTHAIAMDPELYP

EPESFDAFRFSKLREEYPEMDGRFQYVASNTSSMAFGYGRHACPGRQFAAQEIKAIMAHI

LREYDLRSPTNRGRPASMASETQYLPHPTATVEFKRRSRFG

>SoG_06071.T1

MAAITTSDIFLVIALYFCGRVLYQIVKYRFYHPLKQFPGPWLASVTRLWIAYHHVRANES

YTFRELHKKHGPVIRITPTLLHIADATKLPEIYCRGADKSQHYISGSFGTIESVFNMQDH

KVHAYFRKIAASPYSFSNIKKMEPLLDATIGYWISRLDDLFAETGKKFDFAPWAVYMAYD

VISEVGFGAPFGFIKEGKDVGGLIEGFHVGMVRFGILARLWPFTSWMKTTFLAKYLVATP

EQESGIGAVMRFRDNLLAQRLADNERTATGSKRIDLLQNFLETKDEDGKPLDLEYVKAEI

LLVLLAGADTTGTTFQAIVRHVSQHPSALERILAEIESANLSEIPQYDEVLNRCPYYIAC

VREAMRLNPAAPSIFPRLALSGGLDLYGMHAPEGTELTCNPWIVHRDPQVVGDDPDEFRP

ERWLEDETRAKNMLKYNMVFGYGPRECLGKHLAMMELFKAPILFFRTFTPEMVNKKNPGR

YMFKGGISYFQDMWMTIKRRSQSV

>SoG_06094.T1

MIQLQDLDLSDPTIYGALFGSGVLIHLAAFRVGEWDLWTPRLISGTAILHAAATYGLSHL

AGDASLPILEAAKTAGLLVGSLVGGVYSSILMYRAFFHRLNRFPGPFMARLSNFYVTGLS

VKNFHLFEETQKLHAKYGDIVRLASKGPSELSIIDPRAHDLIHSNRSPCEKGPWYNVLYP

HVSLQMIRDRKVHAQRRKAWDKGLGSQALRDYEPRIEKYTKQLLREIEKNEGKPLNVTRW

FHFYSFDVMGDMAFSKSFNMLKEGIVHWYMKLVHKNMLAVGIFSHLIWIFPVVKAIPIVN

GEHLKTQAWIKDQVDTRWANKPKTPDLFSWIIADYESRKNPTKQDLINVQSDANLIIVAG

SDTTAATLTSLFFALTHAPDALQKLRSEIDQYWSENEEASAASMAKLNFLNACINETLRL

YPPVPGGVQRMTPPEGLQLGETFIPGNTIVQIPTWTVQRDERNFVQPNDFIPDRWTTKPE

LVKDASNFHPFGVGRYGCAGKQLGLMEVRSVTTQILRDYNIRLANSMTPKQYMDGSQDTF

TMTMAEVEMVFEKRTKA

>SoG_06212.T1

MVTDKTQYAGIETTALPCSYNAYLSILLAIRSERHEPPHCNIYLALSALSTPGKYGPTSN

TSVLPVSSKKPPRISIPFDERAGQERSAPHSVPLKAKDERAIGPEKEAYTDTGLPILGNL

LEIPEKHSWVKFGEYVEEYGPIVQLNILGQSHILLGSEQVVDDLVRARGAIYSSRPQAPA

ASMLTQDLHLLLMPQSKELRERRGIIASLLTRSAASQYEPYQWLESFRMLRDIVRDPTEY

EEIFDHWSTVMGSRLLYGRAMPDRNGPQTEIMDITHKIEQTVQPGAYLVDVLPWMKYLPE

AIAPWKRYIRKLSERDEAFYWKMWNFTREDLKNGRDVPSWARLCMEDLAHEEHGKHHMTE

REAVHLVGAVFTAFGTTSHNLKSFILAVTRHPEWWARLTGEMDSVVGGDRLPALDDVPNL

PIMRAFVTEMTRVWPVTPGGVPHVLMKDDVYEGYHLPAGSVVHIVAWACSRNPETYPEPN

EFKPERWLEPAFPTYKEPLTEFPTLNNTVLFGAGRRQCPGMLVGTRNVYIQAMMLIWACD

IKRARDEDGNEIIPPFYDFVHGFNVGPNRFDFDLKPRSQERLAMVEKAYKEALAGDPMRD

>SoG_06398.T1

MISVITSPEDVKRFHRDSSLHAKSASSNGGWLFHQQLGDCMGLINGEPWKRLRATFHLHF

TRRSVAGTYPQIVSSAANHLKQVISSHCSTGTIHIDAANLAPFPFMVTAEFIYGPLTHAE

KEQLWALGQRSLQLMATVLSGGLYRFRAYEWLRPGTHRLVKEFERDWTAFNGHIYASRKI

AAASGSKMDQPPILGIWEGFTETGLPKRAFVQTMSEVLFANLDVATHVLIWLLIYVASKP

DIQEQLRQEIKDAIGMDEYVANKDTLLHMCFIESARLRPFTAFSIPESSPRGVNLGGFKI

PANTSVVVDTVAINHNPAFWGPDSGDFNPFRMRNMSATELRYNLFAFGFGTRKCLGQYLG

EAMIKIFLITALEDYELRLSETEKQKGLEEASNGDTWVPLSNLRISLRERKKRSV

>SoG_06439.T1

MYYTGPVVRVAPNELSFSTANSWKDIYDKKKGQKPLIKSDFYDGGNFADEAHSIVSERDP

EEHARMRRFLRDAFSERSLREQEEIISMHIDEFIERIGEAGGHPDGIDIVMWFNLATFDI

IGSLAFGQSFGGVASDARRHEAYTMELVKLRVNQKSDRKDFLTNILSRRESERISDIQIA

AHSSDFVIAGSETTATALSSITYYLQKNPAILQALREEIRGRFTSYEQITHSSTMNLRYV

NAIIQEGMRMYPPLPFPLPRVVPEGGKNIDGNFVPAGVVVSTNPFAACMSRRNFQDPWNF

DPERWLKPESADILDASQPFSLGTRGCLGKRNRP

>SoG_06454.T1

MARKVFNSPTFVKPTVVDVAPKLLGHDNWVFLDGKAHVDFRKGLNGLFTRRALEMYLPGQ

HEVYDRYFKRFVQITKEANNKPVPFMPEFRELMCAVSLRTFTGHYMSDDAVLKIANDYYL

ITAALELVNFPIIIPFTKAWYGKKAADVVLSEFSKAAAKSKVKMAAGGEPNCIMDAWIIQ

MINSDRWREAQEKGNAEEIEKPQPLLRMFNDYEIAQTVFTFLFASQDATSSAATWLFQIM

AQRPDVLDRVREENLKVRNGNVKAELNMDQLESMTYTRAVVRELLRYRPPVLMVPYAVKK

PFPITPEYTVPKGSMLIPSTYLALHDPDVYENPDYFDPERYYSGDAEEKGSKNYLVFGTG

PHYCLGQQYAQLNLALMIGKASLELDWVHHATPVSEQIKVFATIFPMATGERSSEMQELE

ENE

>SoG_06475.T1

MTSSLTSSILALLTQHYLLILPILLALYLVRNHFHNGLNKYPGPFLASLTDWWRFWDVYG

QRPERTHLALHKKHGDVVRLGPNTLSFADPKALKAIYGLNKGYVKSDFYIVQQSVVQGHR

LASLFSTTSEPFHASFRRSVNSAFSMSALVQYEPFVDNTTRLFLSQTEKLFTSAHNSINA

KVCDFTTWLQFYAFDVIGEITYSKRHGFLERNEDVEGIVSYLGNLFLYVAPVGQIPFLDL

LFLKNPIYLKLSQWGLFDSTFPVAKFARARMAERLIPELNNTEKKSADDSVLPLAAASTP

KMQQGKSPDLLSKFIAASEARPDFMTADLVQTMAVSMAFAGSETTAISLSSTFYHLLRNP

ACLARLRAELDDFARKGGFSDYETGLVTFAEAQKLPYLHACIQEAFRIHPAAGLPLERIV

PPQGAEIAGHFVKGGTIVGCSAWVIHRRPEIFGEDVDVYRPERWLPRQGADPESEARRIK

EMTGHMFQFGMGSRTCIGKNISLLEIYKVVPSLLRRFDVSHDSLEGRKQVLLADNISPPQ

IEFDDPTKDWKLINAWFVKQVNFMTRFSRRDIVQPEVPLSEKPVAAVAAH

>SoG_06480.T1

MASSTIAAILERMTTGTALLTVLSASLFTVFTKCLYNITLHPLAAYPGPWLWGASRWPWL

YHELKGDLHHVLSRMHRQYGQTLRIAPDELSCTQPGAWKTIYGHRAVEMEKNLKGAGLLP

PLKQARSIVTADRHNHARMRKTVSHAFSEKALREQEDFLQSYVNELIQRLRQHSRSGPQN

LVNWFNYTTFDVIGDMAFGEPFGCLKDSEYHPWVKNVFDGVKMIPWVHAFVYMNVSWLIF

KLVPPSLRKAKEQSDADAYAKVDRRIARQHEYAQRRDFLSYMLPKLGESGMSKPELQETA

VILIIAGSETTATFLSGEIYLILRNPKVYQRLVAEIRSAFKSDEEITITAVQSLPYMNAV

INETFRCYPPAPSTFPRVVPGGGEMIEGNWVPAGMTVGVNPWSAFHDSSNFYKPDDFLPE

RYLKDSDRSEEDLPSELFASDKFETVQPFSYGPRNCIGKNLAYAELRLITAKLLFNFDLN

LAPESDNDGWIRDQKTSLIWEKLPLMVKLAAREM

>SoG_06758.T1

MENFQQHITQLLPAAAGVLTAYFVLDAVYAAYFHPLAKIPGPKSWAISQLPYIYHFSRGH

IHEVLRDAHEKYGHVVRYGRDEVSFATSEAWKTIYGHKVKGEEFAKDKRYYRQTPDPSIV

IADREAHKRHRRLLAHPFSEKALRSQEDIIKHYVDLFINGLAERSGRNEVVDIAAWFNFA

TFDLIGDLAFGESFKCLETGEYHPWVSWILQNMQLFGPEHIIRRLSLTALMPWLIPPSLA

RAFAEHMKLTEETGRRRIAKKDMGRDDFMSNILKQNDTEKGMTEGEVLANVPVLVIAGSE

TTATILAGAMFYLLMNKNSYDRLVQEIRSTFKSEDEITITSVAHLQYLVAMFSEALRMYP

PVPNQPGRIVPAGGAVVEGYTIPEKTKVVVPQWAVYNYSGNFRDSEVFDPERWLGNPYYA

DDNRDVVQPFSTGPRNCIGKNLAYAEMRLIMARLLWNFDISMEPGFETWFKDQKSVLTWV

RGELNVRLTKVVHE

>SoG_06973.T1

MTALTSVLGLLAAALVVALLTFASLGSRLLRLPQAIRVVGSKGNTVSDRLCAYLRAWRSC

YHAVGQGYDESQYSRRGEPFITAHGTGAPEVVVPQEWVKWLVSQPATMLNARISHSIMKV

LLYLYPAPHGEANLRAEFMPPFLRRHIPVSGVDKVQVDLAAGLRYWCSVHLGSEEGWRRS

SLGTTVDDILCKTGYRIFFGPELSQNVQFVKAARRLAQWIDLAEFTVGHVTPPLLQKPLA

LLFRIPVNYHMRTYLRIWEPYFERCCTAQAVAAKGNETETKFDSATMAFARWFVESGHRR

ESLESVVYPVFTVFNIATRALTYTGMAAIHDLITLDAAKKFTPLMLEELRSVFEGREDEA

WADPDSFTPSTTPLLEAFVRESIRYSPTLATIIPRVVVPEDGVMLPNGVTLPKGTLLSAP

IAFIAKDERHHVDPSLFDPFRFLEELAVSGRGAGPKPRRYTLRKDTDSMSGTNDLFLEFG

HGRNACPGRFLAMRLMKMLLACVLLYYEIEPVRECPRMTIGLTSPPPVCMPIRARRILRD

>SoG_07000.T1

MDNQSTVASSSVHNEQDFAFLTILATRSRPTYYLTVATILFVAWLLRANKQGKIDAPFYK

ASLAKWIFDAENLIKDSYTKFQDRVYQIKATEGIQTLIPVKLIGEIKGLPDNVLSQPDAI

AEAMQTKYTKFSPGHNSDMLALLVRTRLTQNLARLIPRLKGELEYVVAEEFPPCEDWTAV

KFQPFSLRAIARLSGHAFVGSEISRTEEWMKTSIEYAITVFMACVKLQLFPEWARPLAQY

FVSDLYKLRREVKKVKGMLKPVLEERLRLRKLGAPNDPKPDDMTEWLLDALPDDEKEDLQ

VHAELQLILAAASIHTTNNLVCECVLDLAAHPEAQEELRKEAYQILEEQGGWARKESMAK

LKKLDSFMREVQRLRGNITSFIRKVNKPITLSDGTHLPPGTKVLAAQAGISRDERFFANP

DEFDALRFYHMRKESEEASNRWQFTSLCDTNINFGAGRHACPGRFFASNEIKTVLAYFLI

NYDLRLKPGTERPMPMAMVMTKAPSPKTEVEFRRRSPAI

>SoG_07113.T1

MGIASSTIAAGLTLIAYLVCRRNARKSKLKLPPGPKPVPLLGNAKDFPPEDTPEHLHWLK

HKDRYGPISSVTVLGMTLVIIHDRNMARDLLEQTASKTSGRPSMIMANKLCGYEDTIISQ

GYTPVFRRYRRFLHQELGTRTSAAQYQKLQEDEVKHQLVRALKDPKNLLKHYKKTSAASA

LKMAYDYNIEPHKPDVLVNMVDQMMKEFALAASPMAWAVDIIPALQYLPESFPGASFKRT

ARRWRKSIQATAYLPYRFVQRQMAAGVHQPSYASRLVEKMGAEEGFKLSEEDEHAVVWTA

GSLYGAAADTTVITLTSFTLAMIMFPDVQKRAQDEIDSVIGIERLPTLQDRENLPYVDAV

VKEALRWWPVTPMGFPHMATEDIEYGGYRIPSGALILPAVWWFLHDPEVYFEPESFDPSR

YLAPRNEPDPAAEAFGFGRRRCPGRFFADNGVFLNVAMTLATMNIARDVGSDGKEIEVEV

KPKPGVLTYHTDFDFKVSARSEAHKQLIENLERECPFEASDAELMESIDDFEVRY

>SoG_07127.T1

MSVISKIFGFPTLTLAAAVEALVAIKLFPEYYGSRSHLAAVGTLLCANWAVGLVFWVVVN

PKFITPLRKLPGPRTFSSAVNKALVVRGRPSGDLFIDLAKEYPGEDFVVLNALGTQVALM

NPKLMADLLVHKCYDFSKPKRIASFLRYVIGDGLIIVEEDHHKFLRKNTMPAFHYRHIKE

LYPMMWSKTEVLMKAINEDINESGSSVTEINGWASKVTLDIIGIAGLGRKLDTVEKRRDP

LAEIYDRILDTSRERLIFSMLCIIFGRTAVGLIPWKMNDEFNYLTGSLDAICRTMIDEKK

VEIAEKGDDHFDVLSLIMKDGTFTDEALKDQLLTFLAAGHETTASAFTWACYLLTKHPEV

QEKLRAEIKENLPEDVRNMPAAELASLLEQLPLLNGVMQETFRVYPTVPLTMRQALRDTQ

LGGQFIPKGTDVAVPIWAINRSTDIWGPDATEFKPERWISEDGKPNQNGGASSNYDFMTF

LHGPRSCIGQNFAKAEMRCLLAGIVRSFSWELEMDESLVMPRGVVTIRPENGMYLRLTPL

DVKAQ

>SoG_07427.T1

MKLCDKILAEGRKPFNVALACSCFTTDVITEYILGESKGCLDRPGWAATFKSSVDIYKRY

LLTFRYFPMVADIMEKIPARVLGLFHGEAGVIANDIQVVIPSKIKRAMNMPGVEVGEGCP

TILSTLLKSQLPESELSVDRLSGEIMAMLIGGTESSSVVLERLTYFVLSNPTIHKQLHDE

ISASTKDGSTVPSFQELEQLPFLGAIILESLRLMYGIAGRAPRVAPDEDLIYTHHEAADQ

KAPSASANSYIIPRGSAISMSCYLMHTNESIWPDPDKFIPGRWLDSEGNLDRSLERYLLT

FSKGSRNCVGMQLAYCEL

>SoG_07442.T1

MAALSLSQTLLTYASEHWKLLILSGAAAALLCYRSFIYKPFYPTLPLVGEPPGRRWFSLR

NRLRYYNDCSSLFEEAYHSFVKKGRAVVVPSIGPHSEMVMPENAMDWAMSQPDTSLSVVQ

AFLDLNQTVYSLGHRRYWGDPWQFQLVKTHLNSILQTLIPQLNDELGDAFDRHLGVNTGE

WKEIHLEHTMRQVIAQASSRFIVGLPLCRNDDYLDLTYRVIDGMMTTIWSTIPLPAFLRP

ITGPIAGWRTQRVIAKIKTHLEPLYKERLAILSNEGNSGDTKKKENEPQDLLMMMLRFAQ

KKRPRELHNLDIITRRVCASSFVAMHQSTIAATNTILNIIGSDDQFDTISVLRKEAAEIL

GEGDSGSCGGGWTKDKFAQMIKCDSVTRESMRLNFPFGTRGSMRKVMRDGIETPEGLRLQ

KGTTISWLASCAQTDPERFENPRLFDPFRFSRAVAQEHPPPASAAAAAAAAASGSTPARP

DAFTTTSPQYLPFGHGKHACPGRFMVDFELKMIISHLLRNYDLAWPAEYRGKQPLSVWQG

ELSIPPPGARIMVKRRSA

>SoG_07443.T1

MLIPNNLLLTPVVFPLLLLLSSGLVAVFASRRLLRNHGKDKHRAQGCLPEPRARQWDPIF

GLDIALSQGRALQQNRYLDWLRDLHASMPHTKTFSVNFVGYRWIYSTEPEILKAVYATNF

RDFGVEPIRQHPPGFKPFAHKGVSTSDGEDWEFSRFLIKPFFDRSVYINTDRIKPFADRF

MSLIPEDGETFDAQPLLQRWFLDLTSEFIFGKSEDAMTYPERAEVTWAMADVLRGGRQRA

QTYKVLWAFKWDWWYEAVDKVHAFLNPHIRSTLRQLAERRDRIEKGLPVDGHERTDLLWT

MATNLGDEEELRSQLCLIFVPNNDTTSIFIGHCLYYLARQPIAWQKLRDEAVAIGDDPIT

FELLRNLKYLNGVMNETHRLIPNNVTQIRAALRDVILPLGGGPDGKAPLHVGKGDIVSVT

KTVMYRDKDQWGPDADEYRPDRWDGMRGGWHFLPYGGGPRRCPAQMMVQTEAGYMLTRLA

QKYSRLEARDPEPYRARMRIGHSSLHGVKIAFYK

>SoG_07445.T1

MSIREILIYGDSARTSLLLGVATCVLLLVARLFLVSQSKQRPLPPGPPGELILGHLRVIP

TFSPEYAFIRWSKEYNSDVLSFKILGQAVIVLNSVQAAVDLLDKKGGNFCDRPRFVLFEV

MGWGKTLTFLRWGPTFRKHRRVLQKGFQKSNIVQYRSLQERETAIMLGGMVNEPEDWENV

LRRFATAVVLGIGFGLNIRGADDPFIKVAQDASYALAHGGAPAGTPVDFFPFLRWLPSFF

HDRSLKFAKEWRWAIENIHNKPYDAVVLSSNRENSLISTLLDQRSAQITEGETPEMDVDD

IKGAAGAVYAAGQDTTWATLMVFILNMVLHPDIQLKAQECLDRVVGRARLPTFLDRPKLR

YVDYIVQETLRWCPVSPVGVPHRSLKDDTYKGYFIPGGSYVFANARAMTHDENTYSEPDI

FNPDRYVPASEGGRGEPFPIGQFGFGRRICVGQHLAEASVWIVLASMLSTMRILKAVDAQ

GKEETPNVKLTDGLTCHPQSFRCRILPRGRESLTTVRRAADEAQSTIS

>SoG_07787.T1

MEKTAPIPEPPGWPISGHAIGFIDNEHPLKSFEELADKYGQIYRLRLFGTPVTFVSGYKY

IQELCNEKKFRKVPSGVMKELREAANDGLITAHLEEDNWGIAHRVLMPAFGPAAIRGMFD

EMHDVATQLAMKWARFGVNEPFNPTEDFTRLAMDTLALCSMGYRFNSFYTPEIHPFLDAM

GRVLLTSRYRARRSKVPLADLYYRQDNLRFYKDIAYLRGLADEIVSVRIKDPGERKDLVA

AMLNGIDSRTGKHMTDKSVTDNALSFLVAGHETTAGLMSFTLYYLIKDPRVIQKARQEVD

EVLGDGPLTAAHLMKLPYITAILREVLRLEPPLPVFGLQPYEDEIVGNQYVIGKDESCVV

LLGQAMRDPELYGEDANEFRPERMLDEHFNALPPGAWKPFGNGQRACIGRNFAFQEATLT

LAMLLQNFEFFLDDPSYTLKYRQTLTMKPRDFKIRANLREGLTPLLLQRRLTGGTTAPLT

NGIAQHPTTNGNADNGKPLTILFGSNSGTCEYLARRLAEHAPAHGFKAVKVASLDSAAGH

LPKDQPVAIVTTTYEGEPTDDARLFFSWLEGCATGHTLKGVRYSLYGLGHHDWVTSFQKI

PKLIDANLEKAGAQRLLPLALDDVASSEVISEFDAWEDESFWPTLMKEYGCEGDAKPVTG

GPGIAVDLLSPRAKILHHEVYEAVVVDNKTLTAPGEPVKMHIEIQLPNNMTYRPGDYLVV

LPINPRETVARVMRLFRIPWDAHIKITAGQRTSFPSGDSVALTDVLEAYVELTQPASKKN

ILALVEATEDQKLKEELRKLSSDLYVSEIVTKHMAVIDLLERYPAVNLSLAAFLDLLPPM

RLRRYSISSSPLWKSGNATLTYSVFRVPSPSGDGFHVGAASTYLESLQPGDRLHVAVRPS

HAAFHLPASTQSTPVVMVGAGSGIAPFRGFIQQRASEIQSGRKLPPAMFFYGCREPGRDD

LYGNELANWEKIGAVKVHRAYSRTPEKASGHKYVQDALSAERDAVNELWENGARFYICGS

RELGQGVGQTMVRMHLDKTRKLGKEMTEEDGKEWWESLRNERYAIDVFD

>SoG_07873.T1

MSGAQFSTLAVAVSIVSLTIICSIYTKYRNKPEHAEAFLKLPIIGDLHKSPITKPLRNWD

EWVQDKGAVVSTKLMGIVPIVILNTCEAATELFSRRSQWYNNRPPSVTMDMITGAEPGQC

RFTLMHDYDAHLRLYHRLLSPSLGPSSAGRFMPLMELESKQLLQDMVELLGKNTQDEGII

SIDAIYPLLERTQSSIILALHYGIRISQKTDTIMREVISVQAKVTYLAANPRLPDMIPYL

RHLPFFLSPWKRSADKLFAEQAKLYRYLVEYGKDSHGWNATKQALKAAEKYSGVSELDLS

FMLATSIQGGMETSPRQLLWLFVAALQNPSSVRKAHDVLDVVVGRERLPQFSDRKQLGYI

DAIVHELLRWRPVSPGGIPRRADTDDDFDGIQVCKGAVVLANAWSIGRDSAVFDPKLGNL

HDFCPERWLSHEPDGAEVKKAEIQTGLPLPLFGHGRRMCLGKRVALDGLFIQVANLLWAF

DMEPTREGVEPWSMRVTGFMTAPSDFGLKLVPRGPWVANVIKKSWQAADKDLTHVMGVPM

>SoG_07890.T1

MDVLSSYKERLDGTMPPMDDWFLPVTVFTALGFGVAVYKSIFAMGDYAGSDLPLVAEPEG

RKSFALKTRLRYYYDCAALYTEAYLKVSEDLMCEFGKKGKPVLVPGYGGNADLVMPESSV

DWAMSLPDSTLSVSQAFLQANLSTYSLGHSKYWGDPWQFGLVKTQLNSMMQSLIPAVDDE

LQYVVPKVLGTDTENWRELNVDEAVRTIVCAASSRFTVGVPLCRNEDYLKRTLAIIDGVM

VTAIIGNLTPSILRPITARLASIPTWRNLARIKAHIEPYYQERLTALSKDAEEQPQDQFQ

VMLRFAAKKRPQELHDTGVMATRLTATNFVSMHQTAATAVQMLLNIVDSDSEYGTVEKLR

EEAAQAQETGPWTKEKLMYLHGHDSVARESMRVTFPFGNRGLLRMVMKDGIVSDNGVPLK

KGTIMSWFASQAQMDPEKFPDPEKFNPWRFSSSIWEKAEAESSSQQQQHDEGDATAGKKK

LAYHENSFVTTSPDYLAFGHGRHACPGRFLVDYELKMVTAYILTHYDLEFPPEYNGRRPP

NGHVAELNTPPTTARIRVKRRPDKAMNGKS

>SoG_07892.T1

MALINLDFSLFVLLFSGFTALLVSRLSRGGKHHGIDGDANGCQPALRLWQWDPFLGLDSV

ISQVRALKNDYYLDWLRNLHEDRPKTFWLRFFGTRWFYSSEPEVLKAVYATNFKDFGVEP

MRRNSKITMPFAEKGANTTDGEDWIHSRALIQPFFERSVYHDTDRIAPFADGFLNLLPAK

DGETVDVQPLMQRWFLDITTDFIFGEPVGALQDPGRARLAWTMMDCLRGARLRTQAFRFM

WAFNWNWWLAAVAEVHEFVNGHIRATYAEMDERERRLSNGMEVGPERTDLLWSMASQLRD

DEAALRSQVCLIIVPTNDTTSIFISNCIWFLARHPEAWEKLRQEVADLGEDAPLTFAALR

NMTYLNGVMSESARLPAGHGAARRRRGGRSRPAAREEGGHRVGDQDGHVPRPRVLGPGRG

RVSAGAAQGDEELLGVPAVRGRAEAVPGADDGPDRGCVHAREAGEGVQPDRGAGPRAVPG

RHAHRALQQDGGEDCPLQVIGAILIDARDRDVVRL

>SoG_08028.T1

MASVQQFVGALLRDIVSTWRSLSGTAIVAGIASTATVLFLADTARSWYRLSHVPGPFWAS

VSKYWMVKQSLKGRQPYAIQEVNEQYGSLVRIGPNELATADPNVLRRMMAIRSTYTRGPW

YNALRFQPGKDNLFSMRDEETHTILRKKMTAGYSGKENESMEGTVDEHVARFIHLLETKY

LSTNDQYRQVDIAQKIQFFSLDVISDLAFGQPFGYIEQDKDVYDFIKITREFFPVALVMA

NLPVVVSILHSRLFSGLLPKETDKLGFGAFIGVISIANKKVAERFGPHATSQSDMLGSFI

RHGLDQDEASRESLLNVVAGSDTSATTLRVMLVHLLSNPSAYLKLQKEIDDNVRAGKISS

PVTDAEARQLPYLQAAVKEGLRIKAPAAGPLFKTVPPEGDMIDGKFIPGGTQIGTSPFAI

YHSKETFGEDASLFLPERWLIDDNERITHMSSVVDLVFSSGKYQCLGKPVALMEINKIIV

ELLRRFDFTMSRPERPADIINAGIWLIEDFPVRITRREL

>SoG_08153.T1

MDVNMTLVSLLPSQNFWIYVLLVIISPLLATYIISLWNSSATIRSFIDPRRPPTAPYYLP

YIGHAIDILFRPFDLFEENWERFGRDGLWKLILPGQASYQVSTADQVAKVFRGQGMEVSE

SISRSLEYSFGMPREAASLYERDDSGYGPNIATDSKVKPNERILVTQHDFMARQLSGGSL

ESIALHYQKHLVETLRGVYHDLGDDWVQLPNLAEFMHEHITKAALISMYGPHLIRLNPTI

VEDLWAFIPDVMTLFLNPPRFFMRKAYQLRDRLLDNITRYYDHAKNNYDLNGPDEEWEEF

WGSQFIRKKHSELWPRFELMNTKARAAEDLSFLWASNANSVDGAVWFLVQTLLDPDLLRR

VTKEVVQARSISTNEAGSRIIDTQKICSMPVSQAIYAEVLRLRVSVFVLRKAKHDLDFAG

WSIKKGERVCASSVARARDDRIWNTGTAEDPHPLNEFWADRFLVYPDDPASGPVRRTADL

AKEKEVAEDEDSENEPRFSMAGLTNSWIPYSGGARLCPGRHFAKQEIIVTAAVMLSLFEI

ELQTPKGWVAETAAGIFGGGTLPIKGKIPCRIRRRRSGRIENYIAAIRAEFGIKLDNGAN

AIPDSHQVLLTIALPMAFVRVV

>SoG_08286.T1

MALFSLTPSALLGTLSAVISARIIWEYLFSPLRAFPGPFVSKLTDAWRAYIVTLGDADKR

HLAWHRRWGSAVRVGPNTISLNDPDLIKVVYGGRSSWRKSNMYRPNDAMMDGVRIDNLFN

TQDEERHAKYKKPIASMWTLPKILELEPLIDETLRELVQKLNQEFVEPGAVCMMDDWASY

YTWDATANVSFGKEYGLIQAGRDVEGIIAESTSGLKYFAPVRDTPSRRHSLLGDEYEQVV

IDDHDWLDKNPWYRIGPRPLVNGVLYTMRIVSEYQQEVAAGAASSARRREQDLFLDKYNA

LKDRYDFIDDQQVTKYLMANVLAGGDSTSGTVRAVFYHLSKNPERRGRLRDELDEARLSF

PARWEDAVRLPYLDACMKESLRLCPGIGLMLEREVPESGLTLPDGRFVPGGTVVGLNPAV

VTRDEGRFGGRVDEFLPERWLPRDGEDGEEFARRNRRMQEVVDFTFGAGGRICMGRHLAK

VEMYKLVASLYSIYDIQLKDPEHEWKYCNSWFMYHSDIPLVLKPRVDI

>SoG_08311.T1

MEGYKIIIQSAGLAGAALSVLVATLILKLSDKKRPLPDGPQGCFPFLTRYPEKTLHRWAQ

KFGDLYSLWIGDQLFVIISDPAVAKDLMITNGRVFSSRKEMFVKSQIVFAGRGITATPYN

DRWRKHRRLSMVWLSQKAVTGFTDTLDFEATDMIRCLYVASANGKNLVNPQPFAGRCSLN

NMLTITFGIRTKSVDDPLVAKSLRLSREFMNCTGPVSNLVDFVPFLQCIPTTIRSRGRAL

HRELVDTYGGMINDIDHRLKRGETLPDSLSKTLLSVRDEEDLDHLDMAILASAFMIGGVE

TTASIMQWFSALIPAYPEIQKRAQAELDEVVGRDRLPTIEDEINLPYCHAIIKETERCHN

PFWLGTPHVATEDFTYQGKLIPKDTVCVLNTYTMHHDEKRYPNPTNFDDPYERDHWMFGA

GRRICPGMLVAEREIWLVISRLIWAFDMEQVPSHPIDLNEYDGLSGRSPVSFKIRLIPRH

DKVKTVLEAATGSSM

>SoG_08779.T1

MASNSWLLGDLSTPVAITLTLTAILLASVGANVIQQLYFPNPHRPPVVFHIFPLIGSTIE

YGIDPHKFFFRCRAQYGDCFTFVLLGKKTTVFLGAKGNNFILNAKHADLNAEEVYRSLTT

PVFGEGVAYDCPHERLMEHKRIIRDGFATESLRKYIPGFVKEVKDYVQTSPNFQGTSGIC

NITEALAEITIYTAARSLQGMEVRSMFDSTFAILYRHLDDAFQPINFVAPWLPLPRNRRR

NHARAHMEELFKEIIQRRRERGNVKGETDMLWSLMEGEYKDGSKVEDIHIARLMIAILMG

GQHNTAATGAWIMLYLAHDPEYQKRLFEEQKEVLGWPLPPLTLENMQNLKLNQHVIKETL

RLHSPIHSILRQVTRPMPVPDTDWVIPTSHTLLASPSFIARTEEVFERPLQWDPQRWEDI

SRRESAEHDGEEEAGGSKDALVISKALTSPYLPFGGGRHRCAGELYAYAQLGAIIATMVR

LMEWTQVDPKAPVPPPDYSSMFARPTMPAVIKWRLRE

>SoG_08804.T1

MALSSPINYLTTVGIVPLAVLAIAILYLVHSVSQYAKLRAFKGPRWAALTKLWLLNCVQS

GRMHHIFHEISKKHANAEGRHKKGKFARIGPTTLLVADVEFLRKVNAPKSKYHRSDWYKG

SRFTPNEDTLISMTDEVEHKSLRSRMTAGYNLKKENERCESAINSQLGVMLDLIESKYLS

DPGSGKVRPLDWGYLASYFSIDSITDISFSQPLGDLKEDKDKWNFLHNTEDNLKPMSFFT

IYPEILNIIPTTLMVKFLAPHPDDNSPFGMVMGFARRRARERYGPNKVEKNDMLGVFVRQ

GMTQHEAERTGLLQLVAGSDTVATSLRAAILYIASDPSVVRRIRAELSAAGVSPSRPTSE

IIGYSDARNIKYLIAVVREVLRIHPPAIATMEKQLGNEDDVLPDGRVIPARTIIALSLTT

ILRDPEVFGSFASEGRLR

>SoG_08843.T1

MLSVTELGTLDWRTIIASLTARDVLLYGASAWAVLKAVQALYNISPLHPLSRVPGPKLAA

ATYWPEFYHDVILFGRYTREIERMHQKYGPIVRISPNETHCNDANFADEIYASGGRKRDK

PLHQINGSAVGTKNNLGTVDHDLHRLRRAPIGRFFSRAMITRLEEHIHSLVQKLCDKLLE

ERETPEKPFDVGMAYSCFTADAISEYCFGEPFGHLAREGTWTPNFREATLAILKPVFLFR

FFPLLIRTAPLGKYFVDHLPADAALLVRTIQIDIPARVWKTKADLDAGVLHERPTVFADL

LQSELDTSEKDPARLADEAVAILGGGTETTTWALAVMTFHLLSKPRMLARLDTELRTVVT

SPENLPAWTALEKLPYLTGVIQEGLRLSYGISSRTARIPTQEDLVYHGEFDKKPVEVKLP

RGYAIGMSAALAHHDERFFSNSHEFQPERWIDEKGETDRSAEKGMLPFSKGSRSCPGKNL

ALCELYLALTALTLRVLPHMKLHETTQRDLEYDHDMFVPVPAVGTKGVRATMHY

>SoG_08956.T1

MEEHLPTLAVPRSTIWMLLAGIFVAYDVMRRILAWRRLRHIPGPPGAAWTKLWLIRKTWQ

GKVFEDLGRVSKEFGPVARIAPSYVLCGDPVEVRRMWAPRSQFRRAPWYKGFQLDPPNDC

TLSMRDDYVHTVIRSKIAPGYGGRDVEDLEKSVDTGISRFIRLIEDKYLSTQTDYLPVDF

ARKVQFMTLDIISKIAFGEAFGFMDADDDLYGYIKTTEDTLPMMQMLALIPWLLGILQSP

LCKAMMPSERDTVGLGPIMATAKRVVDQRFSSDSLDRKDMLGSFVRHGLTQREAQSESLV

QIIAGSDTTATVLRTVTANVIAHSGVYVRLQAEIDDAAAAGKISSPVTDLEARRLPYLQA

CLKEGLRIWPPITGIMPRISEQDAVVCGISIPAGTNIAWSAYAVMRNQDVFGLDADVYRP

SRWLEANADQLRAMDNTVDLCFGQGKWGCLGRPIALLELNKMVVEASFMIQSQGSVLANT

S

>SoG_08983.T1

MGSLLQVKLPWFGAAGDVLQSNSTGLQGLGGQLVELAKGITGELDRAAVIRTALTALAFW

VVRIEPNFVSIMSPSSVQAIYGTGSQWGKGYYYSRGPVEKQVLVNLASAVDKKIHARKRR

IISHAFSEAAIRSYEATLLDKIRLFCKQISDPTTFHGDYKNMSRWFSYLTYDIMGNLTFG

YEYNMLTKDNDHFIQPLIDTFQHIQIILGCVPWVERFGLGPLMFISMASAVKRFRDYVAN

QVTQRIDLEKAGKGPDDIFKLLLNAEDKKTGEKMGFKELSDEAVVLIIAASDTTGTALTG

MSFYLARYPECYSKLKEEIRSTFSSLEEIVSGPKLLGCRYLRACVEEALRMSPGVPSYLV

RESPKDGVIDGHLIPAYANIGVPGWAMHRREDVFPEPQVYKPERWLTESKDEIEKLRSAH

LPFSYGPRACIGKNLALSTIYLTMARIAFLFEIESREELPLEFHVKDHFAAGDKNGPFLK

FIPRDPRYLA

>SoG_09024.T1

MLPLLSIVPDFPTITFPLTVAAIVSYLVYNRYGYGLGRVSGPLLASVSSAWRIGLVIRGN

AHLEYRKLHDKYGRVARTAPNVVDISDPAAIPIIYGINSKFIKSNFYPTLDMTFEGKTMP

SLFTTISPEEHKALKRPVAQKFSMTSIRSLEYLVDPCSEMFINSMKDLEGKAVDLGAWLQ

WYAFDVIGSITFEKRFGFMEKRKDVEGIIEAIEHALIYASVVGQVPWFHPFLAGSHTVRR

FIQWLGVSDPMKISCDMAQAALDEYDSSGGSDDRHDFLAYLRQQQKSTGTTMPRWEMMNH

LLNNLLAGSDTTAISLRAVFYYVIQDNRVYKKLQKEIDEFQAAGRLSPIITYAESLEMSY

LQICIKEAMRMHPGVSFPLERVVPAGGVQVSGAFLPEGTIVGMTAAVVHRDKEVFGEDAD

TYRPERWLGEEEGVKVMERTLLTFGAGARTCIGKNISIMEMGKFVPQVLREFDVEWASSD

PEWDVKSYWFARQEGFIARFRRRNV

>SoG_09219.T1

MEGGLLTQVLGICAAGSLLYAVVVAVYRLYLSPLSRLPGPKLAALSLWNEFYWDVVKRGT

FIWQIEDMHRKYGPCVRINPYEVHIIDPEFYDELYVSHKKLDKYRWWTNLAGADGSSFST

VPHELHRQRRGVLNPFFSARSVARLQPLIKSKVEKLCSRFQSIAQTGEVVRLDAAFMALT

MDVICDYSFANDRKYLDEPDFKLLWKQTIIGAFEGGAMGRQFPWMLPLMKKLPRSMVSAM

DASVGHLLNWQDGVKVQVQPIIDGTDELSRKGDTSRTIFHTLRDSDLPAHEKSLQRLCDE

GEILTGAGSETTAQTLTRIMFYVSQVPEVLTKLRAELDQIMSEQDSIPDIAELQALPYLS

AVIKEGLRLSYGVTTRLPRVCHEDIVYRDLVIPAGTPVSQTPYFILMHPDIFPEPKRFRP

ERWLEAEKSGSRLDKYLVSFGKGTRQCLGINLAYAEMYLALAAVVHRFDWDMFETGLDDI

ICYHDFFVAVASLESKGVRATLTQRKLSC

>SoG_09258.T1

MFLQIAILSLTVSIAVANLWLGRRRYPDVPILRLSARPGRLGRLDDAQTYITDCMKIYQA

GYDRYSKHGKHYLYHIPQEMEMIVAPAFYEEIYRAPDTHLSSALANSETMQFKWTLQEDL

MFKSFHVGAPLTKLTRSLAARLTDIVEEGRMAIDEYIGCPTEWTAHPLTEQAFRTVTRTA

NRLLLGIDLARNEEFLQLSIEYTGILFGGANTIKKYPDYLKYFMLRWKTGLSSANKLAMK

HLVPIFKARVAERQRYADQNELDIWEKKVKKDDCVQWILDAAPPDELRNFKSLTHRMLHI

NIAAVHTSSNNFMSMMQVLSLMPELQQELREEILEVFERDGGWTKQTMTHLKKLDSFMVE

SGRLCGDSAVKLKRKVVKDFRLSDGTLLPKGLVIFVNSIPFLTDPAVVESPEKFDALRMY

RKRLEPGQENQNQWVMYSETNQTFGAGKHACPGRFFAANEIKSLLVLFLMRYEFRMSSGH

TLDDIYKGVWHNEARTARTDTVLEFRALRMDIPEALKFSF

>SoG_09368.T1

MVFKKLNLNLPRLPGGIDNLALRWSHLVYIVLALILVQAVTRQVSRAIVEWRKGYKPVPR

YPQWIPIMGFDIAMSMAKSLRDHTFLLWLRKLHETGPGTSKTFTIDFLGRHVIHTIEPEN

MKALSATVWKDFGVEPLRRNTGASMPFADKGVNTTDGHDWAFSRFLIKPYFLRDAFSNTD

RLKTHTDNLLSLIPLDGSTFDMQTLMQRWFLDTSSHFLFGESISCLLYPERAEIAWAMTD

VLRGLRLRLQMSKWLWMFRWKPWFDAIEVVHEFIDRQVDRAYEERAKSIATGSTKSEESS

NFGVGLKPERTDLLWSMVGNVPEDRARLRSEMLLLFVPNNDTTSIFISNVFWNLARFPAV

YAKVREEVLAVGEDTQLTYECLRGMKYLDAVLNESEHISSFRLPLFCSWRKRNQANQRFS

AHRLYPNGIMQVRYCIKDTTLPLGGGSDGKSPMLVRKGDVVQVNKSVMQRDKDIWGEDAD

EFRPERWFGLRPYWNFVPFGGGPRRCPAQLLVTTEASYVVARFCRRFSRIENRDLSGYIP

VMRAGPVNTRGVKIAVTPA

>SoG_09369.T1

MNSTQSPTFASLLPYRGLEGFTLTSRSSVLAYGLGLVIIAIFLSQNGNKLKCLDMDGVPM

KELRVSPKAAALFTPQVSRQGEKLAHDEPYILRDGNYREVVLHTAEHVREFLRNDSKGKL

CTPSGRTNVGDFETLTAGFNSDHFRPENLGFGDYFYRVLGQCVGAISGDQWRVIRSYFDP

AYTHSAGLSMLPAFQSEVSKWLNTMKNDSLRSGVGRIVIHAPTSCKALTLRVIPQSFYDE

AYDDEAYEKLDRISKLQAQVLKNATTGRWQKYSWFNVLPTPSKRQLDEYHHDWHAFNIEI

LENSRKVTGTEHAFKGVQPNNAISIEQYLQTIDEMIFTNIEVTGSILAFMFGKLAEHQEY

QQRLHEEIEAQKSSEAFHIRNYLTLQTSLLHYLVLESIRLRPATWFSAPECITIDKVIGR

YKVPAKTPVVIDVRRLNTSALTWGPDSQDFRPERFSEISPNEYRYAYMKFGVISGKCLGK

HMAEILMKLAVVTVLEQYRIEHVEMNIGVKEGDLAFIKRN

>SoG_09385.T1

MVAHQVLYAAYALIFYAVFKYLTATDVPKIKNLPEIPGVPVFGNLIQLGTDHARVAQKWA

KKYGPVFQTRLGNTVSISSFSCSTFFTSCHTWLTPFHFATQRVIFANSYDSVRHFWITHQ

SALISRPTFHTFHSVVSSSQGFTIGTSPWDESCKRRRKAAGTALNRPAVQSYMPILDLES

VVSIKELLEDCKGGTNDIDPTPYFARFALNTSLTLNYGFRIDGDVNNQLLQEITHVEREI

SNFRSTSNNWQDYVPMLRIWNKQNAEAETYPA

>SoG_09412.T1

MPYTYEHFWETCFAAKSKCALYQSTDSGPKDVSTRFSAWISELEASPGTYLTSTGLHAVT

PYHVAVAMFAPMYNPMRLFPRTASVLAEAMRGNYTGLFDMLPVTGFGAECPLKTPTSYTW

DRDAMSSVACGDALPQTGVTIPEFQDYIGELKSQDPAIGPYWSSIRGSCTGWRYRPKYAF

SGPWTTPEHDPSGDPGRPLSPILFISSKYDPVTPLRNAREMMKYHPGSGLLIQDNVGHST

GSTPGKCRDDVVRRFFAKGEVPNGEVYCKADCRPFEECSGGGSLSTYTVGNSPLYHRSPL

QIVLSTPVEPPKHLQIGTAHRPPHEGFLAGGNLSRVWPILTTATGITGAKMTLLEIINDN

LPLAISGIIAVASLVTWSLTRHDPREPPLATTPIPIIGHIIGLSRETFNYYVNLSKKLPS

PIFTVRLPGQKMYIITSADLVLKVQKQHQTIAFPPLATAFSVRTSGTTRESVKVIEKDDY

RLMHLSNEILVDALKPGENLDSMNRIMLGEISHSIEQLEPARGETVRINFNQWLRATLTR

ATTRSVYGPKNPYEKEGVVDGFXNARDFEGGLIALAIGILPSIVAKKAIAGRDKVTAAIR

NFYAEGGLETSSDIPRNRYRIARERDIPASEIAKFETGIALAVLLNTGPATFWTVLMTYL

TPDLRDELRAEIDACMETTIDDAGITTKSLDILNLKESCPLLVSTYQEALRYFHVGTLVR

LVTQDTYLDGYLLKKGAMVHMPSRAIHHDPSVYGPTATSFVPRRFLPSEKKNRPKDYFFR

GFGGGKNLCPGRHFATNEILATAAVFIARFDMEPAGGKWEMPTGWNSGSAGQIMEPDNDV

EVILRTRQGFEDVKWDLRLKTSDKIFAIVTEDVNDV

>SoG_09429.T1

MNTIPNDGIIRFRGFFNQENILVCSPAAVCEVTVTHNDQFEKPSLIRGIAGRIVGNGVLL

AEGEDHKMQRRKLMPAFSFRRIKEVYPIFWDKAVEDVQALTSHVAGCGEIELEAHGWFSR

CALDIIGLVGLGRDFGAVADENNSLAKTYSLVMKPNRLAVLFALLRLVFPHWLVNLIPTQ

RNNVINSASQHIRGVCCDLVRDKKSRLEKGEQPDPDILSTALQGGGFTIEQTVDQAMTFL

AAGHETVASTMAWAIYALCRNPEAQAKLRDEVRWRLPSVDSGKGITSADIEAMPYLNAVC

NEVLRLYPPVPLTIRQPSRDTTIQGLPVRKGTRIVIAPWATNRCHLYWGPTGDEFRPERW

LEDGRQVNKGGGDGNDDEGVEGSAPGSRLGGSTSLYANVSFLSPARGPALDRPSPGPSSP

AYWRRSSAGSRSVSRMGARERGPSSRARRGWVYRRARSPQSRARTGWS

>SoG_09512.T1

MIFLHPAFLAGIGLYVLGIFIVRRMSTPVTWLPGPAISRITGLYLKWQELRANRTLYVHA

LHEKYGPVVRIAPNEVSFTSWAAVKEIYCSSGSGYDKSEFYNLFKVYGRRTMFSTLDKTD

VSKSARRDLLNAAELPQTVLPTYLWVRSASNCAWKFLGADNMHQALHAYACDCITHHLFH

PYGTDCLRKTADEDMMHQVAGDDSLQNRLISYYVPTLHSLFSSVLHAFWPPRQTPLADNY

VLSTAQKPDPAQFTLLSRLEEKKGAVEGSELDHTSVAAECLDHMVAGIDTTGDALCFLMW

ELSQPRSLEMQRKLQVELRENPQAPLDQLPYLDAVVNEGLRVFPAIPMSLPRVVPLGGRN

IDDVWVPQGTVVSCQAHSVHRINSHVFPNPDKFDPERWLQPEGDAERRRLMFAFSNGGRG

CVGKHLALAEMKTLLRDTYSKFSTTPDPSMRAESMVMSDQLISSRPLGQRCLLRFHPIGE

AETV

>SoG_09546.T1

MLASSGGISHLLEVLADFGYARLGALWLLAILIYRLGYQLFFSPLRNIPGSLLSRLFASH

SVLKRVLAEGSRSVQKDYETYGDIYVNKPNGVSISNPRDIKTVLTSYEFRKTDIYQMLDI

KGRPSIFTNRDPAQASQRRRQLGPFLNLGFLGRMEPLILKYSIVAIKNKWDALIDKAGGN

PITVNFRDDTQYATFDTIGALAFGREFNALANNDPTIIRWIEATGLYLGTTKNFPLLNWW

PFSKLVSRPKAMFESFINYSEESVRQRREMLDNPSTTEKPVDLLQGFIDAEDPDSEKKVP

MTPHEVSTESIAMQLAGSESTSFVTSWVIHLLTLYPQHLERAMNEVRSQFALDHVVGFDE

CRKNLPFLEACIYETLRYSPITSGFMPRVNPTNGITIQGHYLPPGTEVAINLIGAHANPD

VWDRPSFFDPTRFLDDDVAKRNVFAFSYGHRNCIGRNLAWVEMMVIIANILKDYDISLPE

DSFCGPHNVDELGRPKIMPTRSALFTTPKYPERDCRIVVAKRRT

>SoG_09659.T1

MTISSVVLDVIRRCQDSPIRSLAAIVAVAPVIYVLINEFVRYSARLPGLKSPPGLPLIGH

LWYLRGDAAQKYKEWAKTYGDVFQIQLGNIPVVVINSAAAAKAIVAQNSQALASRPEFYT

FHKIVSDTSGTTIGTSPYSDSLRRRRRGVASALNRPAVASYSPLLDNESREFIRALYIDG

RAGSKAVDALPAFLRLALSLGLTLNWGVRVEGAQAHLFEEITHVEDQLSRMRSTSSLSNL

QDYIPLLRLNPFSGQSAKAKDLRARRDAYLKELNEGLNSRIADGTYKPCIQANVLTDPDS

KKLTKTELDSISLTMLGGGLDTVGAAVAWFVNCMSQRPHEQEKALREIRNVYGDDQPLCD

VNDDQQRCEYVVILIKELLRYYCVLRLNLPRASVKDIEYDGVVLPKGTVFFLNAWACNYD

PAVWSDPDVFRPERWLEQPDAPHFFFGIGYRMCPGYMLANRELYLLFTRLLSSFEIRKLD

DVDSDPLTGNADPTNLVAPPPRARCYFVPRNEKALSAALGIGKTG

>SoG_09761.T1

MAILSLLLSPWTLLAATVLAFVYPELVTYRSLRGIPGPLLARFSDLWLFSTARRGKRFLL

VHEAHERYGPVMRIGPNHISINDDAAIQAVYGHGNGFLKSDFYDAFVSIRRGLFNTRSRA

EHSRKRKIVSNTFSAKSIGQFEPYIHHNLELFVKKWDEMAKHQATPDGTVHVDCLNWFNF

IAFDVIGDLAFGAPFGMLSTGADMAEVRSSPDSPPIYAPAVEILNRRGEVSGTLGCMPGL

IPIAKYLPDPFFSKGLAAVKDLAGIAIAQVRRRLDNPPPADRKDLLARLIEGRDENGQPL

GREELTAEALTQLIAGSDTTSNTSCALLYHAVRTPGVVDKLRAELDAAIPRDVTVPTFEQ

VKELPYLQNVINETMRHHSTSGIGLPRQVPPESPGVEILGRFYPGGTVLSVPTYSIHHST

DIWGPDADDFRPERWEKATERQKNAFIPFSYGPRACIGRNIAEMELKLIVSAWVRRYDVQ

LRQGEMETREGFLRKPVALEIGFKRRP

>SoG_10141.T1

MGLASLSIADLTHLLLGSLPPGRVVLGCLLPAGCLLWAVHSWRRLSHIPGPFWWSLSPIP

LLRANLVGESHQILNDLSLKYGPVVRIGPNAVLTTDYKHCQKMEAHKSPYRKGPWYGTFR

FQKGVDHLFSMRDEAKHSATRTKVGPGYAGTFLVEEAIDRQLARMLRIIDEQYVVEPGEH

KPMDLAVITQFLALDIVGDMTFGKPFGFLDQGRDIYGWIEWNEGFFPVASTCATLPFLAS

MVQTWPFSEALPKATDKKGLGCFIKVAQDTLEERFKPDAEPKRDMIATFIKHGATKEEAT

SEALVQVDSEPPPAPRVSLTSVFPFPSVAGTDSVAVALRMALLYILSTPRVYNKLVGELR

DAERAGLASTPIRDAEAKRLPYLQAVIREGMRIFASQTPLLNKTVPDGGDVIAGVWVPAG

TQVGMDGAPCSKIHQWGILRSKETFGPDAHVFRPERWLDVEPEKQADMAAALEALFGYGR

YKCLGRNVAFMQMGKALPELLRRYDFTVNDPILPIKKVHSAAFWMMTGFWVTVRHRDAGV

AA

>SoG_10205.T1

MSVIQQLRSPLRTVQGPFLARLTSLWQYGNVVRYGPNRYSVSDAAASKLIYGLGNPFPKS

SWYSSWSSPGQWSIFGDQSVERHAHNRRQYQSTYAMSALVNYEAFVDECADIFADRLSEL

SAAHATVDMRHWFQCYAFDVIGMITYAKRLGFLDSGEDVGNVISALEDHLGYATIVGIFP

GLHKYLFPLKNMLAGSRGSGRAYIIKFTNDKIREAQVKPKVEPTTEDELGTQDFLSKFLA

KRTQDPENFTSFHVLSGCVSNMVAGSDTTAISLSAVLYYLLKNPICMQRLKDEVDKKTRD

GELSTNPTFKESQQMPYLQAVIKEALRLHPATGLPLERVVPEGGATISGQFFPKGTIVGL

NTWVTHRDPEVWGIDAEKFRPERWLDPDINKLGIMNRQWTPFGLGSRTCIGRHISMLEIC

KLIPRLIRDFTFQLDGPLQTGNWRTSDYWFVKPLNFKVKVHQTLMRLIDTEMTHSGALGE

>SoG_10242.T1

MGALQQLLHQVENLQAASPVTLGAVAAVATLGLAYLWRSSRPKPHGLPVVKVTGNNVVET

LEDAHKKYPREPFMLSLPGMEMAVLPDSEIETIRGLPETDVSIKKHHYDVFLGEYSYMGT

KADEFDSTMRNVLTRNTPAVLASFTAEVEYAVNKMIGPCKDWTAIKPRYAMSRIASIMSG

RAFVGLPLSRQEEWVEATVDYTAGVSRAWLVLRLIPWPIRFFVAPFLSQVKALKQQRAIN

EQKLAPVIAEKRSGAKKDKGIPGGDLLDWFMSQYSQPPNAQQLGRDQLLATFASIYNLSN

ALSYIIFDLAALDPEDVELMREEVLQHVPADGRIDKMNLPKLKRLDSFAKESQRLSPPSL

VNIPRIVTNPNGLQCSTGQVLPQGTRMTIHAHAINQNPELYPNPSDFQPFRFSKLRETAG

NEHKFQHATTGVDNINFGHGIWACPGRFFASAEIKVVLAYILRHYDVTLKPGEPKPKQQH

FGLAILPDPTAEVLFKARQ

>SoG_10275.T1

MVGAIETSRAAVADHTTTIAVVVSLLVLFATTRLLTGRTRSSTGGNDAPSMPPYWVPFLG

HVPQVVLDFDGFLSGLMKRYPEGIFSVKLVGKVHNFISRPSLATLLMNQPESVASSNWLH

LRLVYTVFGYRESDGEDLIRKTYDVYHVLSSTHSLNSMTETTVKTLNQNIADWVTFNSQP

ADQMEWEQVAEADVVSDGNGGTAVEVDLMELTRAFVARVATPSVFGTDFCDNFPDFAQHV

WQLDQNFVLLASGLPGWVPYRPLQLARAAKRRLLAYLEEFHTQLDKVADGEDPDPRWENL

DNVSAFLKARLEVYRRERAPIRARAAIELGLLWAMNANSAPLVFWMLYEINRDPVLLEQI

REEMAPYMHAVQPRNDFGLAVWMAPELRNVDVDGLMEKCPLLKASYIETLRLYTGSYTIK

WMNEDAVLGKQSDAAGGGYLLRKGTYAHAAHDLHQLDPESYPDPHEWQPARHLKTVVGKG

GQDVMTADLGTIRPYGGAHMCKGRAFAARELMVFTAAILSLYDIQPPTGHKWGNQKPVKA

TSTKRPSRPVKAWVRRREVREEV

>SoG_10310.T1

MAVIDYLDPEVVRGIVANWWPALVLSGGVVYMTTKVIYRRFFHPLANVPGPFLPAVTRLW

VWYYNVPVEGQLYKQIDRLHDVYGPIVRITPDEVHLSDPSHYDYVYGVKSGFYKDPRFYS

ALGIDVATFGSIHNEAHRRRRAAINPFFSRKKVLELEGIVHDKADKLCRILNSDRAANKP

TNIDAAFRAISVDVITDYAFGESWDQLGSPDLGVWFSDMVRGSSAMFWTLQAFPALKFLH

SLPPSVAKRMSPAVADFIGCQVRTLKHVSDVQKEVDQGIKPARETIFHQLMDPYAAEDHV

VPRTADLADEAFAICTAAADTTGNAMGMAAYRTITDPEIYAKVKKELHDAFPDPSAEMKF

TDLEKLPYLTGIIKEGQRLSYGVISRLPRVVPEGGATLEGFFLPKGTTVSMSSWMMHHNT

EAFPDPEVFDPDRWTDPTTFHDRDRCLVPFSRGRRMCVGMNLAWCELYVTLGKLFHRFQH

LEAFDVSDEDMVYQEYFSAFHPPEKRKFRVVVRDD
