# Supplementary material for: De novo genome assembly and annotation of rice sheath rot fungus Sarocladium oryzae reveals genes involved in Helvolic acid and Cerulenin biosynthesis pathways
Source: BMC Genomics. 2016 Mar 31;17:271. doi: 10.1186/s12864-016-2599-0 (PMC4815069; doi:10.1186/s12864-016-2599-0)
Supplement: Additional file 10: — Protein sequences of putative gene clusters involved in Helvolic acid biosynthesis. (DOCX 134 kb) [file 12864_2016_2599_MOESM10_ESM.docx]

**Additional file 10.**

Protein sequences of putative gene clusters involved in Helvolic acid biosynthesis.

>SoG_03551.T1

MRNQTSSQRLAGIMDAESLPLVLRGLASRYEKHLQALSFAIPSWLHRLGISGTLAAVVIAYATFTVLYALYLSPLRRIPGPLAARLTAKRGVWSILTGSAASDAQADYENYGDVYVAKPNAVFLCDPDDARAVLAMAEFRKTDMYRIFEYEGVPNVSTLTEPAQANRRRRQLHPFFAYSYLAKMEELILRHGTRALNARWGAKIEADAAKGERTIVNYRLDTQLAMFDITGALVFGRDFNALKTDNLDYTKWVNNTLTFMLLAHYFPLLKRWPFSHLVASLRRSYDDLVAFSKESIAIRRALLDAGGEKPADLLQALLDSEEPDSKVRMTPGEVQAESIAMLVGGSESTSSVISWVIHFLLLYPEDLRKVVEEVRGKFGAEHVVTHAECRAELPYLEACIYETLRCIPTASTSFPRISDRKGITIKGFYIPPSTEIVTNKCAAHIHKESWESPFDFKPSRFLDNNEAKRNMLSFAYGTRFCIGKNLAWVVMIVTLANLFKDYDVALPETSLFHPGNVDENGRPRIMPTKLGVATMPAHPERDCLMVLKRRVE

>SoG_03552.T1

MEGFLRPATAVSYPLSAFDMQGSFSNIPYAFFFENHDGSRDDFMPTDLVKKSLARALEDFPILIGHIRAQATGKLVVEVSPDQLNIPDFLETACTDTSFGDIKAAKFAWSSWPEGVATVGPFATPSVADGEIRLLTIHLVRLKDESGVILFVNIPHYAVDGAGYFAFIKHWASTMASMVQKRTADEGASRLGQHSKEPLVIDRGCIPKYLPTNDRKPLDEISKAMYTASNFFCDTLAWFSPTTLGNLLARLGRLSPPGEAHLFHLSQKTLNSLKDAAQQHIHSDSSRITTNDILVALIGKTYVQSQPPAPAPKAGWFRAAPPAETHFTFRIPCDARPRLGVLEKFTGNLLIPMLVREPLEQLAQPTNPLTLASAAQTVRGVIGSVNSGLIAEYHDTIAAHPTSHMRPLSFAASHQTTSMVTTSQVRFGLYDADFGYGRPRFVCLSDRFAGSYTMAAFLPTPPPEEGEEGGGVNVLLTSNTLAMKGILANEFWRQAAELVW

>SoG_03553.T1

MHLGWFKSSSKPKEPAFDHEVDFLVVGAGGAGLTAALRAQHLGHKALVIEKNAQIGGSSAYSGAGLWIPQNSYVREAGVLDSKESALEYMETVIGDVGPASSKARKLAFLQHGPEMVDFLRGQGFLWRCSKGVPDYYPKVVGAMSSWGRTIEPGVFDLNRLGAWRGHLRARPRQPPPIFTDEASSITRAGSSLRDFFKAASVMARGLWLSTVHGQAPATMGQSLIAQLLCMHKDAGTPIWRSASLVELISDKDGSVVGAKVRRAGGNDEGEKSDGNGEGEGVVQSIRARRGVLLAAGGFAHNQHLRDKWGPAPASTKWTSTPEGDTGDAIQAGIKVGAATALLDEAWWGPSILDPNVGKFYFSLQERARPFSIIVDSKGKRFMNEAESYIDAGHRQYARHKEVDAIPAWLILDQNHRKRYALGSLMPRQKATKGLEAGYLKQADSIDELASKIGVDAAGLRDTVERFNEMAATGVDTDFGRGDNVYDNYFGDPKVRPNPNLGPIAKGPFYAVQVVPGDLGTKGGLLTDEHARVLREDGSVIRGLYAAGNTSASVMGRTYPGAGSTLGPALTFAYIAANHMASFKSDRSS

>SoG_03554.T1

MEDFLRGLKSRTYELSPLDIIPSHLYISNVFFFENASTTFGDGSEFMPDGTLKSAFYDALQRFPILAGRLTRNGLTNTMHIAVDRDDPNLPAWEHSLVKIHFSQVKRRSMHRDAWPEGINITDPLVQNAPKGSESPKLARVHVHRFAGNSGVAIVVRLSHSVFDAKGCTQFINHWASCCRARQGGRSESPDELPPVLDRAVMYGHLPASVRPKPVSWFLWPVSLLLMLLVSAISWFSGKQTTTGSSESHLFRIPRDTLDGLRRTDARDDGQVSDHDIVTALFTMAYAQAKHSGKESSAENIFGMKKRPPAKVSAIVPCDLRHRLGIPESYSGSCAIGVYVTAATSQLLAPISPSSLAAVASISRRAVAELDLAAVEKLTRRAMVAITLLGERARVLYSLMVCQAFSNQSRLPFYETDFGAGKPVLVAPMAYSKSVAVVFPPETASVSSGKGDLCVFLTLEEDDMASLLKNEGFTSIAKLIY

>SoG_04319.T1

MKKILFLLGRFHFAAHFHGTNCIFTSNIPSPKPSIIQLCAPSTRMPSLARAATGENIAVLSVAVVGLFIGWKLVYAYYLSPLRKIPGSSWARLTTLRAIYNRLPKRVTRAALAEHRRYGDIYVSRPDTVTISHPDDVRAVLGAADSWKIDVYHGLDDPIMSNLVTFSHPQLASRRRRQISPFLNSQGYLAKMEAVIMEHGILALMSKWEGALGDAGEEGESSLQINYRNETQLATFNIMSALAFGRDHSMASDGSLIVDWISATAVYVGVSINFKSLMTFPFSMLIRSWLKKYHDFVEYGKQSVAQRKQALADGRLGEKPADMLQAFIDAEDPDSKAKMTSVEVQAESVGMQLAGSETTSASLTWALHLLTLYPEVLSRAVKEIRDQFPSDHIITYSDCRARLPYLESFIYETFRYAPITSGFMPRYSKRTMELQGHAIPAGTRIAFNLIALNNRPDVWDRPDEFVADRFVGDDEAKKNIFAFSYGPRSCIGRNLAWVEIMTILANLLNNYDMSLPGDSVFGPGNVDESGMPRLMPSQCHIVFAPTHPDRDCRIVLRRRAS

>SoG_04320.T1

MSSAANVAIVTGAAQGVGLSAAQLLAARGMRVVVSDVNVEGGEQAVADINKEHGEGAAIFVACDLSKTEDIDELIKKTVDRFSGFSILVNNAGFLRAPFLAITAEHIKDTIAINLTATVYSTHQAIKFWDEHPEIKGQVVNVTSSSSFKTYASIAAYGAAKAGAAQFTFACRDFGPRIRVNAVAPTAIATAFDKSTLMRVPTDKKGPGYTPEEEMRNMGLARLQPEEVARVVLECIDDETRFGKVVHLDATDGQTIHSGFLS

>SoG_05635.T1

MSPASVHDVVVGKAAHSDTKAAYEYKTDLTRWRLNVDNGRHMWEYVTDNDELRRRPQSFLEKYWLGLEFELPRMPTPTSPQQALDNGWSFFSRLQTQDGHWGCNDDGPLFVTSGIVIATYIVGIELSQHMKDEMIRYLMNFANDEGGWGLWIDSPSTVFGTAINYVMLRILGVPASHPVLVDARIALHRMGSARALPTWGKFWMCVLGVYEWDGIMPLVPEPLLAPAFLPLNPGRWWVHVRNVFISMSYLYGHRFVMPPNELTRELRRELYDVDYEGIDWFAERGNVSAHDRLAPATVVQRGVSAVLSTYEYVKLPFLRRRALREALFQVEAEVLNTNYLCIAPVSFASNMLVLFHAHGRDSHWITGMRERVIDPMWVCREGMAGSGTNGTSLWDTVFTLQAAIDSGLAGLPENREIMVRALHFIDASQIREDPLGAAHVYRQPTKGAWPFSTRDQSYAVSDTTAETVRCVLQLQAMPGIPKLVSDERIREAVDLILGMENAGGGYSAFEPIRAPKFLELLNITELYENVMTDNLYPECTSSVLLCLDTFSRAFPDYRAVEIASCVRNCVSYLVRSQFPSGGWIATWGVCFTYATMFALQGLESAGYTSVNSETCRRACDFLLRHQNADGGWGEDLESIKEKRYVQDPAGSQVTCTAYAVTGLMAARCADRAAVRRGVAWLVSRQQKTGDWLPGSLEGVFASPGGMRYPNYKFHFTLAALGKYVALYGDEQLF

>SoG_09546.T1

MLASSGGISHLLEVLADFGYARLGALWLLAILIYRLGYQLFFSPLRNIPGSLLSRLFASHSVLKRVLAEGSRSVQKDYETYGDIYVNKPNGVSISNPRDIKTVLTSYEFRKTDIYQMLDIKGRPSIFTNRDPAQASQRRRQLGPFLNLGFLGRMEPLILKYSIVAIKNKWDALIDKAGGNPITVNFRDDTQYATFDTIGALAFGREFNALANNDPTIIRWIEATGLYLGTTKNFPLLNWWPFSKLVSRPKAMFESFINYSEESVRQRREMLDNPSTTEKPVDLLQGFIDAEDPDSEKKVPMTPHEVSTESIAMQLAGSESTSFVTSWVIHLLTLYPQHLERAMNEVRSQFALDHVVGFDECRKNLPFLEACIYETLRYSPITSGFMPRVNPTNGITIQGHYLPPGTEVAINLIGAHANPDVWDRPSFFDPTRFLDDDVAKRNVFAFSYGHRNCIGRNLAWVEMMVIIANILKDYDISLPEDSFCGPHNVDELGRPKIMPTRSALFTTPKYPERDCRIVVAKRRT

>SoG_03005.T1

MPSSEDIGLDMFMATRLAEVAHVAKDIGYARILGTVTAAWLLWKVTHALVLSPLRNVPGPMLARLTSKRGDLDNFSGRVCQTADHDIARYGEVYVYKPNAVCISNPDDVRRVLGSQEFRKASFFDIFDDGSTKNIVSQRDPALAARRRRQMGPFLNYGYLTKMEPVIQRHGYLAIRAKWEGLLAESEGPAVEVNYRIDTQLVTFDIMSALAFGRDPSSISKGKSSITEWSGIIMKLLENPVVLALLSLIPFPMLMRPWKKMYRDLAVYSKESGRMRKEFLATGAPAPADMLQAFIEAEDPESKVKMSEQEVQAECIMMMLAGSETTSSAIMWVFHLLLLHPEKLRLAVDEVRSAFGPDHLISNKDVLTKLPFVEACVFETLRMSPTTAGLTPRVSHDRGIQLQDHFIPPGTEIYVNLRSVNMHESIWEEPARFRPERFVGNEEAKKTLFTFSYGPRNCIGRNLAWVEMLTIVANVLKDYDIALSPDCEWRPENVDENGNPKLLPAKCFIASFPSNPERDCRMMVSRRSDLSV
