# Supplementary figures and images for: De novo genome assembly and annotation of rice sheath rot fungus Sarocladium oryzae reveals genes involved in Helvolic acid and Cerulenin biosynthesis pathways
Source: BMC Genomics. 2016 Mar 31;17:271. doi: 10.1186/s12864-016-2599-0 (PMC4815069; doi:10.1186/s12864-016-2599-0)

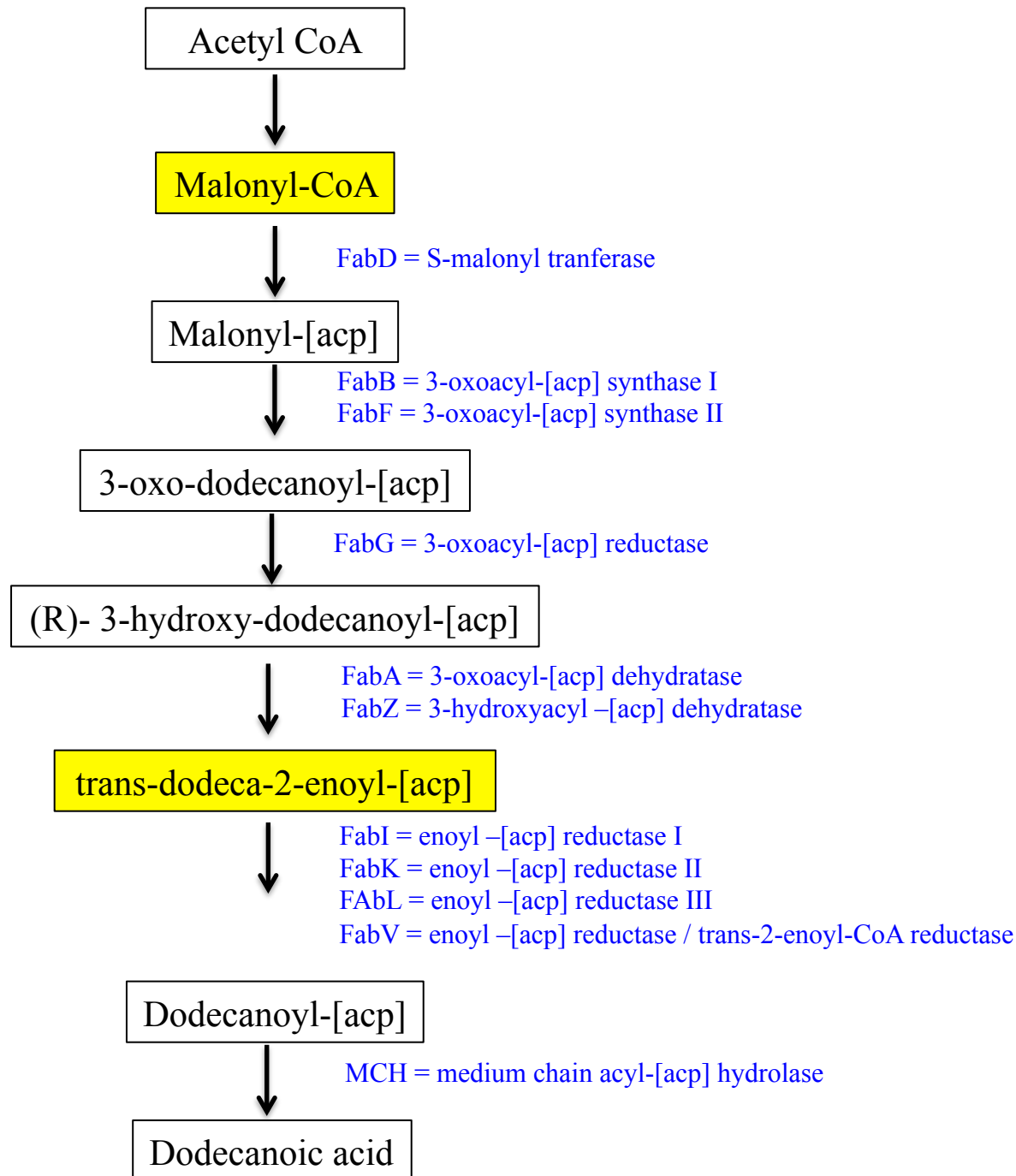

Supplement: Additional file 11: — Biosynthesis of Dodecanoic acid pathway. (PDF 47 kb) [file 12864_2016_2599_MOESM11_ESM.pdf]
